# AI driven 3D subcellular RPE map discovers cell state transitions in establishment of apical-basal polarity

**DOI:** 10.1038/s44387-026-00074-6

**Published:** 2026-02-06

**Authors:** Davide Ortolan, Pushkar Sathe, Andrei Volkov, Dominik Reichert, Sheldon Sebastian, Arvydas Maminishkis, Nicholas J. Schaub, Bengt Ljungquist, Devika Bose, Jorge Ferrari, Nyusha Lin, Gianluca Pegoraro, Carl G. Simon, Ruchi Sharma, Peter Bajcsy, Kapil Bharti

**Affiliations:** 1 Ocular and Stem Cell Translational Research Section, National Eye Institute, NIH, Bethesda, MD USA; 2 Information Technology Laboratory, Information Systems Group, National Institute of Standards and Technology, Gaithersburg, MD USA; 3 Information Resources Technology Branch, National Center for Advancing Translational Sciences, NIH, Bethesda, MD USA; 4 High Throughput Imaging Facility (HiTIF), National Cancer Institute, NIH, Bethesda, MD USA; 5 Materials Measurement Laboratory, Biosystems and Biomaterials Division, National Institute of Standards and Technology, Gaithersburg, MD USA

**Keywords:** Biophysics, Cell biology, Computational biology and bioinformatics, Neuroscience

## Abstract

The retinal pigment epithelium (RPE) is a specialized cell monolayer that forms the barrier between the subretinal and choroidal spaces. During development, RPE cells polarize perpendicular to the monolayer plane such that organelles attain specific intracellular locations. This allows the RPE to differentially interact with overlying photoreceptors and underlying choriocapillaris. When RPE polarity is disrupted, tissue homeostasis is disturbed, leading to retinal degeneration. The subcellular organizational principles of RPE polarity are unknown. We developed an artificial intelligence (AI), specifically a mask region-based convolutional neural network-assisted high-content image analysis platform combined with mathematical modeling to develop a quantitative three-dimensional digital twin of RPE subcellular structures during the establishment of apical/basal polarity, polarity organization with learning-based analysis for RPE image segmentation (POLARIS). We discovered, during apical/basal polarization, cells constrict along the lateral axis and elongate apically, nuclear volume decreases, nuclear envelope develops invaginations, junctional complexes consolidate to the lateral membrane, the endoplasmic reticulum and mitochondria increase in volume and translocate towards the nucleus, and lysosomes move towards the central-apical side. AI algorithm and mathematical analysis reveal non-stochastic cell state transitions and organelle interactions in 3D during RPE polarization. These integrated AI-based quantitative data provide a reference digital twin to discover intracellular defects in diseased RPE.

## Introduction

The retinal pigment epithelium (RPE) is a polarized cell monolayer of neuroectodermal origin that forms the selective barrier between retinal photoreceptors and the choroidal blood supply^1^. As the RPE interfaces with two very different environments, it organizes its organelles in a specific apical-basolateral polarized fashion so it can accomplish different functions on each side^2^. During polarization, cells use extrinsic cues and interactions with neighboring cells to orient along the apical-basal axis; this process requires a drastic rearrangement of cytoskeletal structures and intracellular organelles. This polarization creates different intracellular compartments, aids in selective barrier and transport function, facilitates polarized vesicular trafficking, differential protein sorting on the two membranes, and selective cytokine secretion^3,4^. The establishment and maintenance of this polarized phenotype are crucial to the health of the retina-outer-blood barrier, the photoreceptors, and the choroidal blood supply^5^. For instance, non-polarized secretion of pro-angiogenic cytokines like VEGF is linked to neovascular stage of age-related macular degeneration (AMD) and late-onset retinal degeneration (L-ORD)^6,7^; loss of RPE barrier resistance is associated with loss of immune-privilege of the eye^8^; subcellular lysosomal position is linked to RPE’s ability to participate in the process of autophagy—a process that is critical for AMD initiation and progression^9,10^. Overall, significant evidence supports the notion that disruption of RPE polarity leads to degenerative retinopathies, such as AMD, L-ORD, proliferative retinopathy and diabetic retinopathy^5^.

RPE polarity is established during early development. A strong evidence in support of this comes from the analysis of diseases called ciliopathies, where RPE cells fail to establish complete apical-basal polarity leading to cells that are “flatter”, and express lower levels of maturation and polarity markers^11,12^. Ciliopathies are genetic diseases characterized by structural and functional defects related to the primary cilium, the sensory organ of the cell that integrates signals from different signaling pathways. Both global and RPE-specific disruption of cilia function have been shown to lead to defective RPE polarization^11,12^. Furthermore, chemically enhancing ciliogenesis with prostaglandin E2 (PGE2) was shown to promote the expression of RPE maturation and polarity markers, while using the small molecule Hedgehog pathway inhibitor 4 (HPI4), that is known to disrupt cilia function, caused a reduced expression of the same markers and disrupted ciliary protein trafficking^11,13,14^. Although the molecular mechanisms responsible for achieving RPE maturation and polarity remain incompletely understood, it is thought that a precise regulation of protein sorting, the acquisition of specialized morphological structures, organelles segregation, and a reorganization of cytoskeletal structures are all events that need to be achieved^4^. Discovering the exact morphological changes that occur during RPE polarization at the subcellular level is the first step towards understanding of how RPE polarization is achieved. Furthermore, a precise 3D subcellular map of RPE cells will help ‘diagnose’ disease cellular phenotypes where RPE polarization is impaired.

To address this gap, we developed a mask R-CNN-based algorithm—POLARIS (polarity organization with learning-based analysis for RPE image segmentation) to segment and analyze RPE cell images in 3D and develop a digital map of RPE cells. We used induced pluripotent stem cell-derived RPE (iRPE) to obtain high-content subcellular images of an entire cell. RPE derived from 16 iPSC lines expressing green fluorescent protein (GFP) tagged to organelle-specific proteins were imaged using high-content imaging platforms. Cellular structures were geometrically aligned, and cellular borders in 3D were segmented using our mask R-CNN code (POLARIS) that was trained on manually annotated images. Images segmented using POLARIS were statistically characterized to generate an integrated RPE digital twin—a complete, data-driven computer model of an average RPE cell. A condition (HPI4 treatment) where the acquisition of RPE apical-basal polarity was prevented was included as control^11^. The results of these experiments revealed that during polarization, iRPE cells contract along the lateral axis and elongate apically. The nuclear volume decreases progressively, and the nuclear envelope forms invaginations when cells polarize. At the subcellular level, junctional complexes consolidate to the lateral membrane—distributed from apical to mid-basal sides, the endoplasmic reticulum and mitochondria increase in volume and translocate towards the nucleus, and lysosomes migrate towards the central and apical side when cells polarize. Mathematical modeling was employed to predict changes in cell, nucleus, and organelle morphometry with time and treatment. This analysis revealed that the transitions between cell states during iRPE polarization are non-stochastic. Furthermore, organelles and subcellular structures sort spatially to form specific interaction partners. These holistic morphometric digital twins provide statistically accurate representations of cell state transitions that provide a reference to discover structural abnormalities in conditions where RPE polarization is disrupted.

## Results

### Study overview

**Fig. 1:**
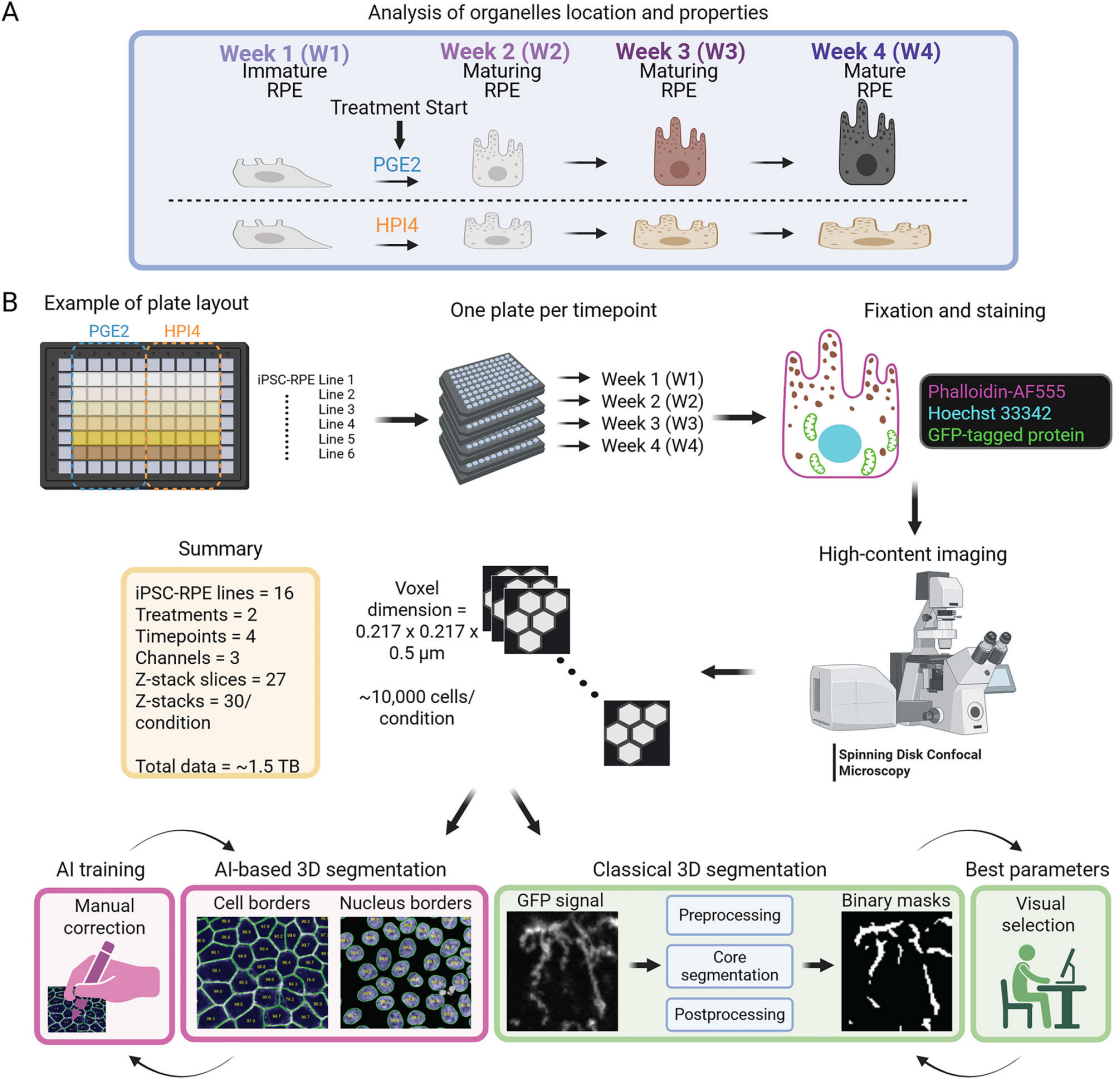
Assay workflow. **A** Immature iRPE cells from 16 lines, each expressing a different organelle protein marker tagged with GFP, were grown for four weeks in the presence of PGE2, a primary cilium enhancer, or HPI4, an inhibitor of primary cilium trafficking, to promote or inhibit iRPE cell apical/basal polarity. **B** Immature iRPE cells were seeded in glass-bottom 96-well plates. Seven days after cell seeding, PGE2 or HPI4 treatment was started to respectively promote or prevent cell polarization. Once a week, a plate was fixed in 4% PFA to preserve RPE cell states at different timepoints of RPE polarization. The first timepoint (week 1) represents a baseline since treatment with PGE2 and HPI4 started after the first collection. Cells were stained with phalloidin-AF555 and Hoechst 33342 to visualize cells and nuclei borders. Automated high-content imaging was performed with a spinning-disk microscope to collect 3D image stacks. An artificial intelligence algorithm was trained to segment RPE cells and nuclei borders that were used as a reference to report organelles and structures’ location. Each tagged organelle was segmented with a unique combination of classical and traditional image processing segmentation algorithms. The quality of segmentation was visually inspected by two experts to select the best parameters for the algorithms. Organelle location and morphometry were calculated for each condition to generate reference maps representative of cell states at different timepoints of polarization.

Sixteen previously published iPSC lines^15^ (Table 1), each with a different genetically engineered GFP-marker localized to a different intracellular compartment, were utilized to develop the 3D intracellular map of human RPE cells. Using our published protocol^16–18^, all 16 transgenic iPSC lines differentiated into RPE with similar efficiency and produced resultant RPE cells with a similar degree of maturation (Fig. S1). To generate 3D maps of RPE cell state transition towards complete polarization, immature RPE cells were cultured under PGE2, a primary cilium inducer, which has been previously shown to enhance RPE polarization^11^ (Fig. 1A). As a control, immature RPE cells were cultured under HPI4, a known inhibitor of cilium function and RPE polarization^11^ (Fig. 1A). Cells were imaged weekly for 4 weeks. Volumetric images (voxel dimensions: 0.217 × 0.217 × 0.500 µm) of 30 fields of view (FOV) and an average of 10,000 cells per condition, per timepoint, per iPSC line were acquired with an automated spinning-disk confocal microscope, generating data with ~3840 FOV and ~1.3 million cells. A supervised mask R-CNN segmentation algorithm (POLARIS) was trained to recognize and segment cells (using the phalloidin- stained cell borders images) and nuclei borders (using Hoechst 33342-stained nuclei images). All AI-generated segmentation data were validated by trained experts, and inaccuracies or mistakes in the model-predicted cell and nuclei border segmentation masks were manually corrected. The corrected set of cell and nucleus segmentation masks was then used to re-train the segmentation model before final analysis. 3D segmentation followed by traditional image processing operations to improve the segmentation results were then used to segment the intracellular GFP signal from fluorescently labeled organelles and other cellular structures. In the case of organelle segmentation, optimal segmentation parameters for the traditional image processing operations were confirmed by visual comparison of microscopy images and the segmented image (Fig. 1B).

**Table 1:** Abbreviated list of the 16 cell lines used in this study

**Line abbreviation**	**Protein abbreviation**	**Structure**
ACTB	ACTB	Actin filaments
MYH10	MYH10	Actomyosin bundles
FBL	FBRL	Nucleolus (dense fibrillar component)
LMNB1	LMNB1	Nuclear envelope
DSP	DESP	Desmosomes
GJA1	CXA1	Gap junctions
TJP1	ZO1	Tight junctions
CTNNB1	CTNB1	Adherens junctions
CETN2	CETN2	Centrioles
SEC61B	SC61B	Endoplasmic reticulum
ST6GAL1	SIAT1	Golgi apparatus
TOM20	TOM20	Mitochondria
LAMP1	LAMP1	Lysosome
LC3B	MLP3B	Autophagosomes
RAB5	RAB5A	Endosomes
SLC25A17	PM34	Peroxisomes

### AI-driven discovery of non-stochastic cell and nuclei compactness during RPE polarization

**Fig. 2:**
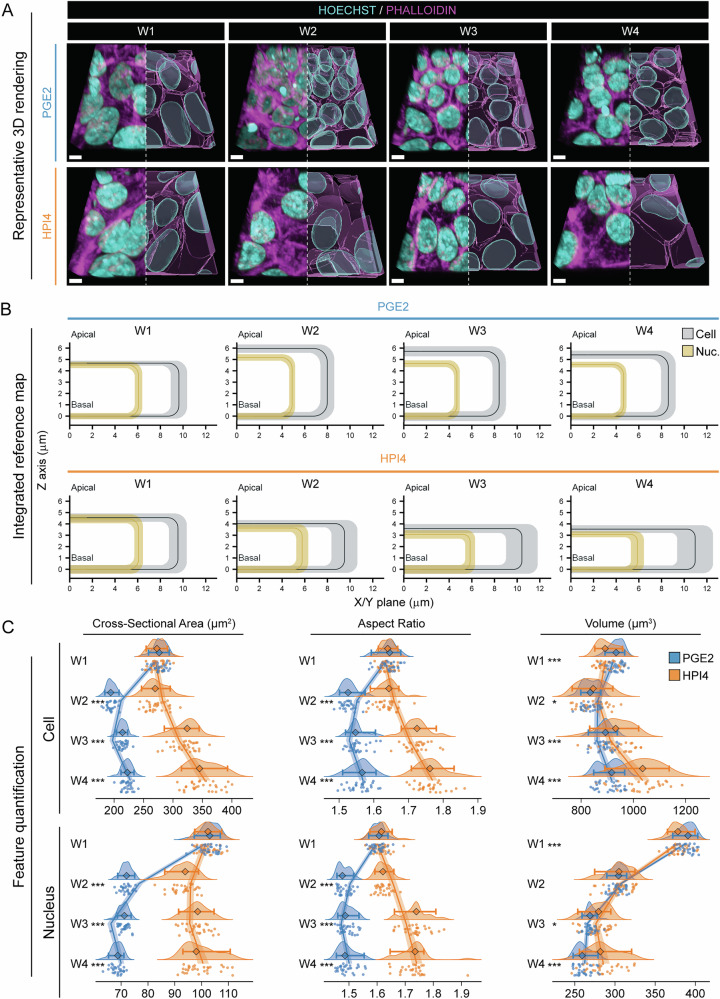
Mapping of cell borders and nuclei. **A** Representative 3D reconstruction of iRPE cells treated with PGE2 or HPI4 at each week of maturation (magenta = phalloidin, cyan = Hoechst; scale bar = 5 μm). **B** Integrated reference maps showing cells and nuclei dimensions and their relative location for each condition. The average cell and nucleus dimensions (height along the Z axis, and half of the average between minimum and maximum Feret’s diameters from images projected along the Z axis) were calculated from the entire dataset (16 cell lines, 30 FOVs per condition). Half-cell and half-nucleus are displayed as organelles location calculated in the subsequent figures, which was measured using absolute distances from the cell membrane or centroid. The location of the nucleus was calculated as the average distance between the cell and nucleus centroids along the Z axis and the X/Y plane. The black rectangles represent the average cell dimensions, while the surrounding gray bands represent the confidence interval estimated using two standard deviations from the average cell dimensions (95% of the data). The average nucleus and its confidence interval are shown in yellow. **C** Raincloud plots depicting the changes over time and between treatments for cells (top) and nuclei (bottom) cross-sectional area, aspect ratio, and volume (the points indicate the average values for each FOV; the diamonds represent the median, and the error bars indicate the 5th and 95th percentiles. Two-sided Welch’s *t*-test was performed to compare PGE2- and HPI4-treated cells at each timepoint. *N* = 30; ***P < 0.005, *P < 0.05). The solid lines indicate the generalized linear model maximum likelihood fit for the two treatments with week and the square of week as dependent variables.

3D reconstructions of phalloidin-stained images over the four-week transition towards polarization confirmed previously observed changes in cell morphology^11,19^. RPE cells and their nuclei became smaller and more regular with PGE2 treatment; in comparison, they stayed large and irregular upon HPI4 treatment. Furthermore, unlike PGE2-treated samples, in HPI4-treated cells, the actin signal visually did not concentrate on cell borders. Since actin concentration at cell borders is required for a cell to attain polarization^20^, these results suggested weaker polarization in HPI4-treated cells as compared to PGE2-treated cells (Fig. 2A).

With the goal of determining if these cell state transitions are stochastic or not, we first set out to quantify morphometric characteristics of cells and nuclei during polarization. At each stage of RPE polarization, we computed the average cell and nucleus height along the z axis from the entire dataset (~10,000 cells per condition, four timepoints, two treatments, 16 iRPE samples) and cell and nucleus width by averaging all Feret’s diameters of cell projection on the X/Y plane. Segmented data were presented in Fig. 2B. Our data displayed that in the first week of iRPE cell polarization, cells acquired an average height of 4.6 ± 0.2 µm (mean ± standard deviation) and an average width of 19.1 ± 0.4 µm (Tables S9 and S10). As cells continued to polarize in the presence of PGE2, their height increased to 5.9 ± 0.2 µm in week 2 and decreased slightly in the following weeks (5.3 ± 0.2 µm at week 4). Cell width reduced to 15.9 ± 0.3 µm in week 2, and slightly increased to 17.1 ± 0.3 µm by week 4. In contrast, cells cultured in the presence of HPI4 decreased in height to 4.0 ± 0.2 µm in week 2 and continued to decrease, reaching 3.5 ± 0.2 µm by week 4; their width increased to 18.9 ± 0.7 µm in week 2 and continued to increase to 21.8 ± 0.8 µm by week 4. Nuclei attained an average height of 4.6 ± 0.2 µm and an average width of 11.6 ± 0.2 µm in week 1 (Tables S9 and S10). Interestingly, as the cells polarized, their nuclei initially increased in height in week 2 to 5.3 ± 0.2 µm and then decreased to 4.8 ± 0.2 µm in week 3 and maintained a similar height in week 4. But they contracted laterally to 9.5 µm ± 0.1 width in week 2 and maintained that width until week 4. With HPI4 treatment, nuclei shortened to 3.5 ± 0.2 µm in week 2, reached a minimum of 3.2 ± 0.1 µm by week 3 and maintained the same dimension at week 4. The nucleus width shrank to 10.9 ± 0.3 µm at week 2, while it slightly increased to 11.3 µm ± 0.3 at weeks 3 and 4. It is worth noting that the standard deviation in cell and nucleus morphology parameters kept increasing in HPI4-treated samples, whereas it kept decreasing in PGE2-treated samples, suggesting a convergence of cell and nuclei populations to a steady state as cells became polarized.

Computation of the height and width of cells and nuclei allowed us to quantify cross-sectional area and elongation (aspect ratio) and changes in cell and nuclear volume (Fig. 2C). In the presence of PGE2, the cell cross-sectional area decreased drastically from week 1 (248.7 ± 11.0 µm^2^) to week 2 (172.9 ± 7.2 µm^2^), before stabilizing at 198.3 ± 7.7 µm^2^ at week 4. Instead, HPI4 induced an initial slight decrease in cross-sectional area at week 2 (244.4 ± 16.3 µm^2^), and subsequently a progressive increase to 319.6 ± 25.2 µm^2^ at week 4. The nucleus cross-sectional area changed in a similar manner. Treatment with PGE2 induced a large decrease between week 1 (102.0 ± 3.3 µm^2^) and week 2 (71.3 ± 2.0 µm^2^), and a smaller progressive decrease to 68.0 ± 2.0 µm^2^ at week 4. Treatment with HPI4 also induced a reduction of the nuclear cross-sectional area, although relatively smaller compared to PGE2. On average, the nuclei shrank to 92.2 ± 4.2 µm^2^ at week 2 and stayed at 97.0 ± 4.1 µm^2^ at week 3 with no significant change compared to week 4 (Fig. 2C, Figs. S6, 7, and Tables S9, S10). Aspect ratio is a measure of elongation, as it is the ratio between the major and the minor axes (perfect circles have an aspect ratio of 1.0). PGE2 induced a decrease of cell and nucleus elongation. Cell aspect ratio decreased from 1.68 ± 0.02 at week 1 to 1.57 ± 0.02 at week 2, and 1.60 ± 0.03 at week 3, with no significant change at week 4. Nuclear aspect ratio showed a significant decrease between week 1 (1.62 ± 0.03) and week 2 (1.50 ± 0.02), while it remained constant in the subsequent weeks. In contrast, cell aspect ratio increased progressively with HPI4 starting from week 3 (1.77 ± 0.03) and reaching 1.81 ± 0.04 by week 4, while nuclear aspect ratio showed a substantial increase at week 3 (1.78 ± 0.05) that remained stable at week 4 (Fig. 2C, Figs. S6, 7, and Tables S9, S10). Interestingly, both treatments induced a reduction of cell volume from week 1 (813.5 ± 29.9 µm^3^) to week 2, followed by a slight increase. Cell volume with PGE2 increased to 776.7 ± 29.1 µm^3^ at week 3 and remained stable at week 4, while with HPI4 the volume grew to 925.0 ± 77.0 µm^3^ by week 4. The nuclear volume decreased steadily from 377.9 ± 10.6 µm^3^ at week 1 to 257.2 ± 9.8 µm^3^ for PGE2 and 279.2 ± 20.4 µm^3^ for HPI4 at week 4, with the largest change between week 1 and week 2 for both conditions (Fig. 2C, Figs. S6, 7, and Tables S9, S10). When calculating the ratio between cell and nucleus volume, we found that the ratio increased with time in both conditions, with HPI4 having a slightly larger ratio [week 1 to week 4: 2.2, 2.4, 2.9, 3.1 (PGE2); 2.1, 2.5, 3.1, 3.3 (HPI4)]. Overall, POLARIS-based analysis further supported the notion that both cells and nuclei became more compact with continued polarization of RPE cells.

To determine if transitions between states of polarization for cellular and nuclear phenotypes for the different treatments are non-stochastic, we analyzed cell and nuclei shape metrics (area, aspect ratio, and volume) using generalized linear models (GLMs) that incorporated both linear and quadratic time interactions with treatments PGE2 and HPI4. Our models demonstrated high significance for all variables (Tables S13 and S14). These findings suggest that polarization state transitions for RPE cell and nuclear metrics are non-stochastic. Overall, these results indicate that cells and nuclei grow taller and compact laterally upon polarization; in contrast, they shorten and spread laterally in the presence of HPI4 when they don’t polarize. Because we used the entire dataset (16 cell lines, 30 FOVs per condition) for this analysis of cells and nuclei, it also provided a reference (average) map to analyze images of intracellular organelles and structures’ location developed in the rest of the study. The corresponding representative fluorescence images and their respective segmentations are shown in Fig. S8.

### RPE polarization leads to a unified actomyosin cytoskeleton

**Fig. 3:**
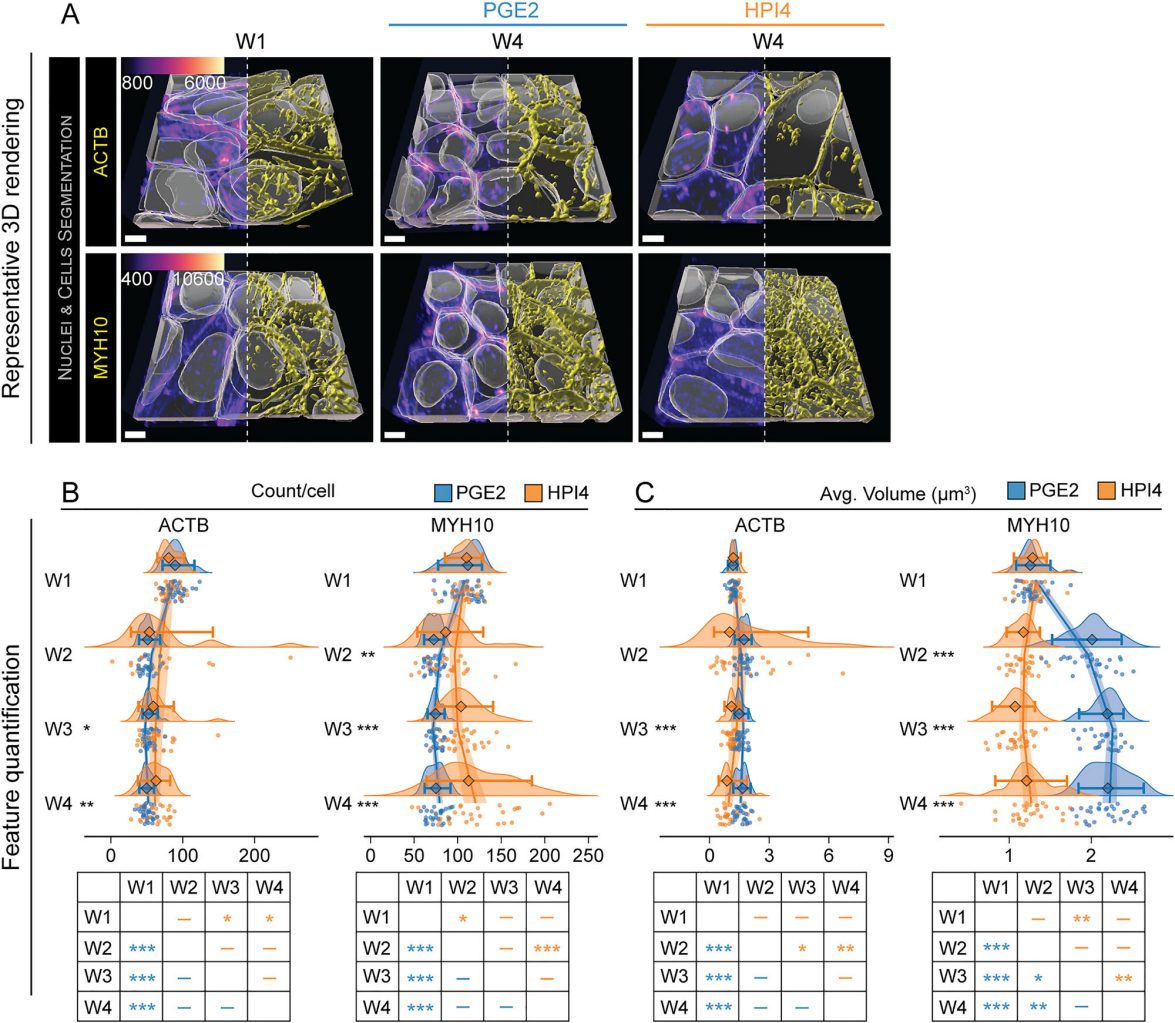
Polarization-associated cytoskeleton unification. **A** Representative 3D reconstructions of iRPE cells expressing GFP-tagged ACTB (actin filaments) and MYH10 (actomyosin bundles). Images from W1 constitute the baseline, while images at W4 show the endpoint for cells that were grown in the presence of PGE2 or HPI4 (magenta = phalloidin, cyan = Hoechst, yellow = GFP-tagged protein; scale bar = 5 μm). Note that, in these images, the phalloidin signal from the top of the cells was stripped to better view the cytoplasmic reconstruction of GFP-tagged protein signal. **B**, **C** Raincloud plots demonstrating the changes over time and between treatments for **B** object count per cell and **C** volume (the points indicate the average values for each FOV; the diamonds represent the median, and the error bars indicate the 5th and 95th percentiles). Two-sided Welch’s *t*-test was performed to compare PGE2- and HPI4-treated cells at each timepoint. The solid lines indicate the generalized linear model maximum likelihood fit for the two treatments with week and the square of week as dependent variables. The tables at the bottom display the results of Tukey’s HSD pairwise comparison (*N* = 30; ***P < 0.005, **P < 0.01, *P < 0.05).

The actomyosin cytoskeleton has been shown to regulate cell morphology, planar orientation, and apical-basal polarity in the lung epithelium, MDCK, and epidermis^21–23^. To determine the role of actomyosin cytoskeleton in RPE polarization, we examined the morphological changes occurring in the actin cytoskeleton by imaging RPE cells expressing fluorescently-tagged beta actin (ACTB, line: ACTB) and non-muscle myosin heavy chain IIB (MYH10, line: MYH10, actomyosin bundles). RPE cells presented several visually apparent actin and actomyosin stress fibers at week 1 (Fig. 3A and Figs. S9 and 10), a sign of an immature monolayer, as contact inhibition suppresses stress fiber formation in confluent epithelia^24^. At week 4, both actin and myosin were organized around the basolateral and apical cell borders. RPE cells treated with PGE2 displayed a more regular organization, as compared to cells treated with HPI4 (Fig. 3A and Figs. S9 and 10). To determine actin and myosin density in cells, we counted the average number of objects, i.e., connected group of voxels, and their volume from the segmented images. We hypothesized that a reduction in the average number of segmented objects with time, in combination with an increase in the average volume of the segmented objects, would indicate that actomyosin fibers consolidate during polarization. We found that the average number of actin and myosin objects decreased with PGE2 by half for actin and by 30% for myosin already at week 2 (ACTB: 83.7 ± 13.2 objects per cell at week 1 vs 49.3 ± 9.2 objects per cell at week 2; MYH10: 108.0 ± 15.8 objects per cell at week 1 vs 72.3 ± 7.8 objects per cell at week 2) and remained constant until week 4 of polarization. In contrast, HPI4 reduced the number of segmented actin objects by only 25% at week 3 and week 4 (59.1 ± 16.6 objects) and, after an initial 19% reduction of myosin objects at week 2, it induced a slight increase by week 4 (118.9 ± 40.3 objects) (Fig. 3B). Of note, HPI4 increased sample variability, as seen by the overall differences in standard deviation values, suggesting a larger heterogeneity of cell states. GLMs fitting for ACTB and MYH10 number of objects showed positive squared coefficients for both treatments, thus predicting an accelerating rate change for reduction in object count for PGE2-treated samples and an increase in object count for HPI4-treated MYH10 samples. For both conditions, the inflection point was around week 3 (Fig. 3B and Tables S13, S14). Overall, this mathematical analysis confirmed our visual analysis that with RPE polarization ACTB and MYH10 fibers tend to merge providing the necessary cytoskeletal strength for cellular polarization.

In line with the object count observation, we discovered that the average volume of segmented actin and myosin objects increased in the presence of PGE2. The volume of actin objects increased by 45% at week 2 (1.2 ± 0.2 μm^3^ week 1 vs 1.6 ± 0.3 μm^3^ week 2), after which it remained constant, while the volume of myosin objects increased by 54% at week 2 and slightly increased until week 3 (1.3 ± 0.1 μm^3^ week 1 vs 2.2 ± 0.2 μm^3^ week 3). HPI4 induced a transient 25% increase in actin volume at week 2, which reverted to the initial levels by week 3. Instead, HPI4 induced a transient 15% decrease of myosin volume at week 3, that reverted back at week 4 (Fig. 3C). Mathematical modeling of volume changes with GLMs indicated a decelerating curve for both ACTB and MYH10 and treatments, except for MYH10 in the presence of HPI4 which showed acceleration. The inflection points seemed to occur earlier in HPI4-treated cells (ACTB: week 2.1; MYH10: week 2.7) compared to PGE2 (ACTB: week 3.0; MYH10: week 3.4). (Fig. 3C and Tables S13, S14), suggesting a different trajectory for polarization vs non-polarization cell state transitions. Taken together, these observations suggest that the actomyosin filaments become less fragmented and increase in volume in polarized RPE cells, supporting the idea that a continuous actomyosin cytoskeleton is needed to exert contractility and develop apical-basal polarity in epithelial cells^25,26^.

### RPE polarization induces features of terminal differentiation in the nuclear envelope and nucleoli

**Fig. 4:**
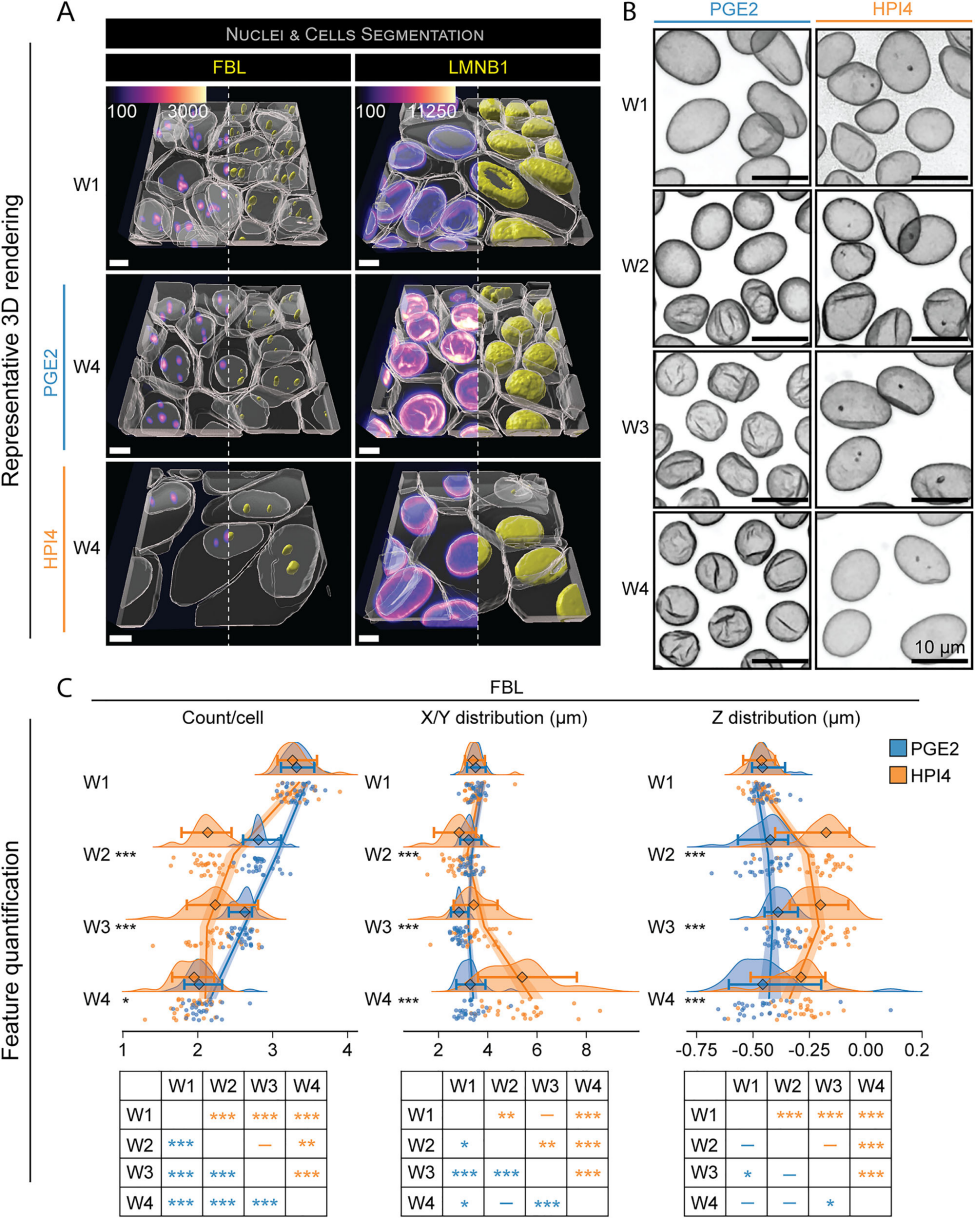
RPE polarization is associated with nuclear changes. **A** Representative 3D reconstructions of iRPE cell lines expressing fluorescently-tagged FBL (nucleolus) and LMNB1 (nuclear envelope). Images from W1 constitute the baseline, while images at W4 show the endpoint for cells that were grown in the presence of PGE2 or HPI4 (magenta = phalloidin, cyan = Hoechst, yellow = GFP-tagged protein; scale bar = 5 μm). Note that the signal from phalloidin was stripped from the top of the cells in these images to better show the reconstruction of the GFP-tagged protein. **B** Panel of maximum intensity projected fluorescence images displaying wrinkling of the nuclear envelope occurring with polarization (scale bar = 10 μm). **C** Raincloud plots indicating the changes over time and between treatments of FBL for count per cell, X/Y distribution, and Z distribution (the points indicate the average values for each FOV; the diamonds represent the median, and the error bars indicate the 5th and 95th percentiles). Two-sided Welch’s *t*-test was performed to compare PGE2- and HPI4-treated cells at each timepoint. The solid lines indicate the generalized linear model maximum likelihood fit for the two treatments with week and the square of week as dependent variables. The tables at the bottom display the results of Tukey’s HSD pairwise comparison (*N* = 30; ***P < 0.005, **P < 0.01, *P < 0.05).

The morphological properties of nucleoli and nuclear envelope can provide insight into cellular transcriptional activity^27,28^. For instance, as cells differentiate, the nucleolar count per cell increases, but in terminally differentiated cells, the nucleolar count per cell decreases^29^. In line with the literature, we speculate that the number of nucleoli in iRPE would decrease according to the differentiation status. To determine how the nuclear envelope and nucleoli in iRPE cells change as a function of polarization, we differentiated and imaged RPE from iPSC lines expressing GFP-tagged fibrillarin (FBRL, Line: FBL, nucleoli) and lamin B1 (LMNB1, line: LMNB1, nuclear envelope). 3D rendered images revealed 1 to 5 nucleoli per cell at the start of polarization, and this number decreased in both PGE2 and HPI4-treated cells from week 1 to week 4 of polarization (Fig. 4A and Figs. S10, 11, **FBL**). PGE2-treated cells had rounder nuclei as compared to those in week 1 and of HPI4-treated cells, as also described in Fig. 2A, C (Fig. 4A and Figs. S10, 11, **LMNB1**). Segmentation of the LMNB1 signal revealed the development of invaginations starting from a few nuclei at week 2 and becoming the dominant phenotype by week 4 only in PGE2-treated cells (Fig. 4B), suggesting an impact of nuclear envelope invagination on transcriptional state of polarized RPE cells, as has been shown for gene expression regulation during hematopoietic stem cells lineage commitment^30^.

Segmentation of FBRL and LMNB1 signals also allowed us to perform quantification analysis. The average number of nucleoli detected in each nucleus declined from 3.3 ± 0.2 at week 1 to 2.1 ± 0.2 at week 4 with PGE2 and 1.9 ± 0.2 with HPI4. While we observed a steady decrease of about 15% per week in PGE2-treated cells, HPI4 induced a 36% decrease by week 2 that remained stable at week 3, followed by a 17% decrease at week 4. This rate change is easily observed when the data is fitted. PGE2 induced a linear reduction with an inflection point predicted before week 1, while HPI4 displayed an accelerating curve with a local minimum occurring at week 3.6 (Fig. 4C and Tables S11–S14). This suggests a progressive change in specific RPE transcriptional profile as cells continue to polarize, and a different transcriptional path is followed by cells that do not polarize. The average nucleolus location was calculated relative to the nucleus centroid. We found that the distance on the X/Y plane to the nucleus centroid transiently decreased by 20% at week 2 in the presence of HPI4, after which it gradually increased, reaching 5.2 ± 1.3 μm at week four (~50% increase from week 1). Conversely, PGE2 induced a slight but stable decrease from week 2 (9% decrease) that reached a maximum difference of 20% at week 3, and stabilized at 6% at week 4 (3.3 ± 0.5 μm). GLM analysis showed that both treatments induced a nonlinear change in FBRL distribution with a local minimum at week 2.9 for PGE2 and 1.9 for HPI4 (Fig. 4C and Tables S11–S14). The distribution of nucleoli on the Z axis showed that, on average, nucleoli were found 0.45 ± 0.05 μm below the nuclear centroid at week 1 into polarization. They moved further towards the centroid of the nucleus when cell polarization was blocked with HPI4. HPI4 caused the average nucleolus location to shift to 0.21 ± 0.10 μm below the nuclear centroid at weeks 2 and 3, and to 0.31 ± 0.11 μm at week 4. The data modeling using GLMs showed a decelerating rate change with a local minimum at week 2.8. In contrast, PGE2 did not prompt a change of location on the Z axis, except for week 3, where nucleoli moved to 0.38 ± 0.05 μm from the centroid (Fig. 4B and Tables S11–S14). These movements probably result from the overall morphological changes of nuclei induced by the two treatments. Overall, this analysis revealed that RPE polarization not only affects the overall cell shape and size, and unifies the cytoskeleton, it induces non-stochastic changes in nuclear size, nuclear envelope shape, and relative size and number of nucleoli. All these features are likely to affect RPE gene expression and align with cellular needs for polarization.

### Cell junction proteins consolidate to the lateral membranes of polarized iRPE cells

**Fig. 5:**
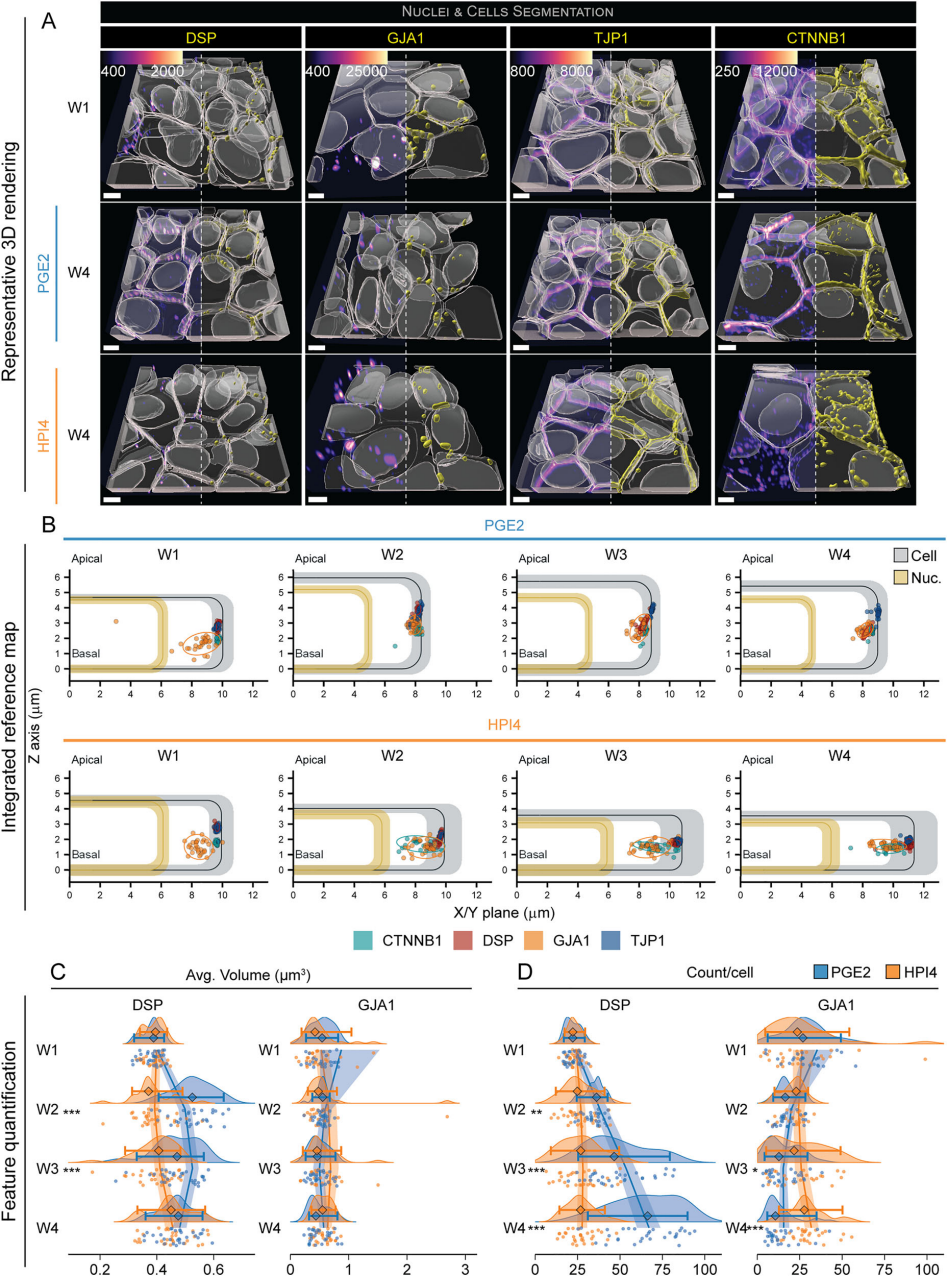
Cellular junctions strengthen with RPE polarization. **A** Representative 3D reconstructions of an iRPE cell expressing GFP-tagged TJP1 (tight junctions), CTNNB1 (adherens junctions), DSP (desmosomes), and GJA1 (gap junctions). Images from W1 constitute the baseline, while images at W4 show the endpoint for cells that were grown in the presence of PGE2 or HPI4 (magenta = phalloidin, cyan = Hoechst, yellow = GFP-tagged protein; scale bar = 5 μm). Note that the signal from phalloidin was stripped from the top of the cells in these images to better show the reconstruction of the GFP-tagged protein. **B** Integrated reference maps showing the location of the structure of interest within the cell. The colored points correspond to the average location of each structure of interest from each field of view (*n* = 30). Each group of points is surrounded by an ellipse, which represents the 90% confidence interval. Cells and nuclei dimensions from Fig. 2 are used as a reference. **C**, **D** Raincloud plots indicating the changes of DSP and GJA1 over time and between treatments for **C** volume and **D** count per cell (the points indicate the average values for each FOV; the diamonds represent the median, and the error bars indicate the 5th and 95th percentiles. Two-sided Welch’s *t*-test was performed to compare PGE2- and HPI4-treated cells at each timepoint. *N* = 30; ***P < 0.005, **P < 0.01, *P < 0.05). The solid lines indicate the generalized linear model maximum likelihood fit for the two treatments with week and the square of week as dependent variables.

Cellular junctions play a fundamental role in the establishment of a barrier function in an epithelium and are thought to “solidify” and mature as an epithelium polarizes^31^. To determine how junctional complexes mature with RPE polarization, we monitored the changes in localization and morphological features of four proteins involved in different types of cellular junctions: tight junction protein ZO1 (ZO1, line: TJP1, tight junctions), β-catenin (CTNB1, line: CTNNB1, adherens junctions), desmoplakin (DESP, line: DSP, desmosomes), and connexin 43 (CXA1, line: GJA1, gap junctions). At week 1 of polarization, junctional proteins were mainly found at or closer to cell borders, and a small fraction was visible in the cytoplasm. In cells polarized in the presence of PGE2, all junctional proteins were completely localized at the cell boundaries (Fig. 5A and Figs. S12, 13). In contrast, a large fraction of intracellular CTNB1, CXA1, and ZO1 proteins were still visible in HPI4-treated cells, even at W4 (Fig. 5A and Fig. S12, 13). To describe the relative location of junctional proteins within RPE cells, the average location of each junctional structure for each field of view was calculated from segmented images and plotted against the average cell for each condition that was calculated in Fig. 2B. Thirty data points, each corresponding to an imaged field of view, were pictorially presented with an ellipse delineating the 90% confidence interval for each junctional structure (Fig. 5B). All junctional proteins localized at the lateral cell membrane in the PGE2 condition, while significantly more variability in location was observed with HPI4 treatment, especially for CTNB1 and CXA1. A relatively large intracellular fraction was detected for these two proteins: 21% of CTNB1 points were found more than 1 µm away from the lateral membrane at week two with HPI4 treatment. This number decreased to 4% by week 4. In comparison, more than 96% of CTNB1 points were located within 1 µm from the lateral border in PGE2, already at week 2. About 20% of the CXA1 points were found more than 1 µm away from the lateral membrane with HPI4 across all weeks, while more than 93% of the points were located within 1 µm from the lateral border in PGE2 at all timepoints (Fig. 5B and Table S19). In fully polarized iRPE cells, ZO1 localized more apically on average, followed by CTNB1 and CXA1, while DESP was found more basally (median distance from bottom of the cell: ZO1 = 3.71 ± 0.25 µm; CTNB1 = 2.58 ± 0.18 µm; CXA1 = 2.54 ± 0.26 µm; DESP = 2.38 ± 0.23 µm).

We then compared the volume and number of particles of DESP and CXA1 junctional complexes. For these two proteins that assemble into discrete objects, we hypothesized that if they played a role in promoting RPE polarization, their average number and possibly their volume would increase in the presence of PGE2 and not HPI4. We discovered that full polarization of RPE led to an increase in the average volume and number of DESP structures per cell (volume: 0.38 ± 0.03 μm^3^ week 1 vs 0.47 ± 0.06 μm^3^ week 4; average count per cell: 22.7 ± 4.2 week 1 vs 64.5 ± 19.4 week 4). But the same phenotype was not evident for CXA1 (volume: 0.64 ± 0.77 μm^3^ week 1 vs 0.50 ± 0.18 μm^3^ week 4; average count per cell: 30.3 ± 24.7 week 1 vs 13.8 ± 9.3 week 4) (Fig. 5C, D, Fig. S14B, D, and Tables S11, S12). In fact, the average volume of gap junction segmented objects increased by 39% at week 2 with PGE2, and it gradually decreased by week 4, displaying a decelerating trajectory with an observed local maximum at week 2.9. In contrast, HPI4 treatment led to sporadic volume increase for CXA1 at week 4. Interestingly, the week 4 CXA1 volume in HPI4- treated cells is similar to CXA1 volume in fully polarized cells, suggesting this volume is not directly linked to RPE polarization (Fig. 5C, Fig. S14A, B, and Tables S11–S14). The average number of segmented desmosome objects per cell gradually increased in the presence of PGE2 (68% week 2, 28% week 3, 32% week 4). By week 4, the number of segmented desmosome objects was 1.8-fold higher than at week 1. The negative squared week coefficient indicates a decelerating curve with a predicted inflection point at week 5.4, suggesting that the number of desmosomes will stabilize between week 5 and 6 (Tables S13–S14). However, we did not observe any change in segmented desmosome objects number when cell polarization was inhibited with HPI4. Unlike the volume of gap junction segmented objects, their number seemed to change with cell polarization. The average number of segmented gap junction objects decreased in polarized cells by 41% at week 2, 13% at week 3 and 12% at week 4, with a predicted local minimum at week 3.4. No change was detected in the HPI4 condition, possibly reflecting a reduced functional role for gap junctions in polarized cells. (Fig. 5D, Fig. S14C, D, and Tables S11–S14). Taken together, we observed an increase in segmented desmosome objects per cell, and a decrease in segmented gap junction objects per cell, suggesting that the mechanical support provided by desmosomes plays an important role in RPE polarization, as similarly described for cell polarization in the epidermis^32^.

To evaluate how ZO1 and CTNB1 assemble at the cell border during RPE polarization to form tight and adherens junctions, we measured the volume and the number of segmented particles per cell. For these filamentous structures, we postulated that an increase in average volume of segmented objects as compared to week 1 or HPI4 treatment, in conjunction with a decreased number of objects, would represent polymerization of globular units into stable filaments. We noticed that the average volume of segmented ZO1 particles increased with both treatments from 1.21 ± 0.19 μm^3^ at week 1. A sudden volume increase was observed with PGE2 at week 3 (2.50 ± 0.81 μm^3^), which remained stable at week 4. HPI4 induced a similar increase starting at week 3, which stabilized at week 4 (1.47 ± 0.43 μm^3^). At week 4, the average ZO1 volume was 50% higher than in HPI4. Instead, the volume of CTNB1 particles decreased for both treatments. From 3.16 ± 0.77 μm^3^ at week 1, PGE2 induced a transient volume increase at week 2, followed by a decline at week 3, which stabilized at week 4 (1.68 ± 0.31 μm^3^). When polarization was inhibited, a decrease in CTNB1 volume was detected only at week 4 (1.37 ± 1.79 μm^3^) and was not significantly different from the volume in polarized cells at week 4 (Fig. S14E, F and Tables S11, S12). The number of segmented ZO1 particles decreased steadily from 25.6 ± 2.6 at week 1 and reached a plateau at week 3 in both polarized and non-polarized conditions (15.4 ± 2.2 PGE2, 21.7 ± 2.7 HPI4). However, HPI4-treated cells had 66% more segmented ZO1 particles by week 4. We detected 32.0 ± 5.1 segmented CTNB1 particles at week 1, which stabilized at 36.2 ± 6.6 at week 3 in polarized cells, after a 38% transient decrease at week 2. Instead, the number of CTNB1 particles remained constant until week 3 in non-polarized cells, and drastically increased to 62.4 ± 29.6 at week 4, which is 65% more than the same timepoint with PGE2 (Fig. S14E, F and Tables S11, S12). Overall, this data shows that a significant increase in ZO1 volume and a reduction of both ZO1 and CTNB1 count per cell directly correlate with RPE cell polarization, indicating consolidation of filamentous structures and a prominent role of these proteins in RPE cell polarization—as has also been suggested previously in other epithelial systems^33^.

### AI-driven RPE polarization analysis reveals ER and mitochondria fusion in mature cells

**Fig. 6:**
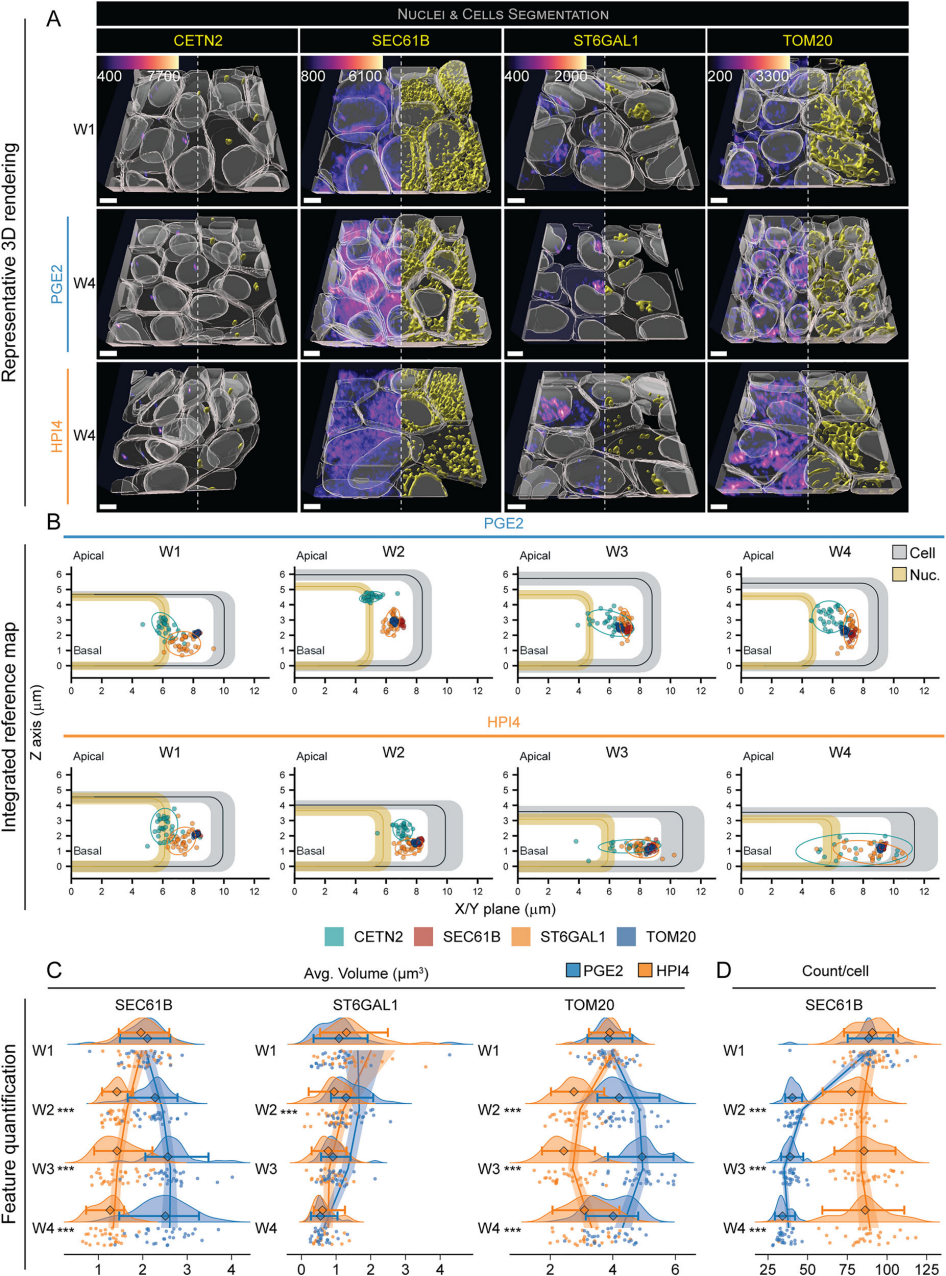
Polarization-associated changes in larger organelles. **A** Representative 3D reconstructions of iRPE cells expressing fluorescently-tagged CETN2 (centrioles), SEC61B (ER), ST6GAL1 (Golgi apparatus), and TOM20 (mitochondria). Images from W1 constitute the baseline, while images at W4 show the endpoint for cells that were grown in the presence of PGE2 or HPI4 (magenta = phalloidin, cyan = Hoechst, yellow = GFP-tagged protein; scale bar = 5 μm). Note that the signal from phalloidin was stripped from the top of the cells in these images to better show the reconstruction of the GFP-tagged protein. **B** Integrated reference maps showing the location of the structure of interest within the cell. The colored points correspond to the average location of each structure of interest from each field of view (*n* = 30). Each group of points is surrounded by an ellipse, which represents the 90% confidence interval. Cells and nuclei dimensions from Fig. 2 are used as a reference. **C**, **D** Raincloud plots indicating the changes over time and between treatments for **C** volume and **D** count per cell (the points indicate the average values for each FOV; the diamonds represent the median, and the error bars indicate the 5th and 95th percentiles. Two-sided Welch’s *t*-test was performed to compare PGE2- and HPI4-treated cells at each timepoint. *N* = 30; ***P < 0.005). The solid lines indicate the generalized linear model maximum likelihood fit for the two treatments with week and the square of week as dependent variables.

Cellular polarization involves cytoskeleton-driven centriole positioning towards the apical membrane^34^, polarization of the components of the secretory pathway through the Golgi apparatus and the endoplasmic reticulum (ER) that add membranes to the apical or basolateral surfaces, and differential distribution of channels and receptors^35^. Polarization also induces structural changes in mitochondria to meet the energy demand associated with a polarized functional cell^36^. We used cells expressing a GFP-tag conjugated to centrin-2 (CETN2, line: CETN2, centrioles), SEC61 beta subunit (SC61B, line: SEC61B, endoplasmic reticulum (ER), sialyltransferase 1 (SIAT1, line: ST6GAL1, Golgi apparatus), and translocase of the outer mitochondrial membrane (TOM20, line: TOM20, mitochondria) to image these relatively large cellular organelles. Representative 3D rendered images at weeks 1 and 4 of polarization are shown in Fig. 6A and Figs. S15, 16. SC61B and TOM20 showed major changes by week 4. At complete polarization in the presence of PGE2, SC61B, and TOM20 translocated from lateral locations, and were compactly distributed around and above the nucleus, which wasn’t seen in HPI4-treated samples. In cases where cells failed polarization, these two organelles continued to be dispersed in the cytoplasm and did not occupy the space above the nucleus. The distance from the apical and lateral membranes of each structure was plotted in relation to the average cell for each condition to describe their average distribution (Fig. 6B). The distance of the structure’s centroid from the cell centroid was also plotted to support the data (Fig. S17E, F). Both these analyses revealed that RPE polarization by PGE2 treatment induced translocation of CETN2 and SIAT1 towards the apical membrane of the cell, while their lateral location remained stable. In contrast, as also seen by 3D rendered images, PGE2 promoted movement of SC61B and TOM20 toward the center of the cell, but not towards the apical membrane (Fig. 6A, B and Fig. S17E, F). In cells treated with HPI4, all organelles were dispersed laterally to the nuclei as in week 1 and even more in the case of TOM20 (Fig. 6B and Fig. S17E, F). Overall, this analysis shows an intracellular polarization phenotype in various large organelles that is associated with the maintenance of polarized cellular structure and likely its polarized function.

Cell polarization in iRPE cells would require extra energy and membrane turnover to differentially deliver proteins to the apical or basolateral surfaces. To investigate if mitochondria and the components of the secretory pathway undergo fusion or fragmentation depending on the polarization status, proxy measurements of the energy state of a cell^36,37^, we calculated the average volume and number of segmented SC61B, SIAT1, and TOM20 objects. An increase in average volume of segmented objects, together with a reduced count and a constant total organelle volume, would indicate organelle fusion. We found that the average volume of SC61B and TOM20 segmented objects increased significantly from week 1 to week 4 of polarization in the presence of PGE2, while it decreased with HPI4. In polarized cells, the average volume of ER segmented objects showed a significant increase at week 3 (30% higher than week 1) and remained 25% higher at week 4 compared to week 1 (2.0 ± 0.4 μm^3^ week 1 vs 2.5 ± 0.6 μm^3^ week 4). Conversely, HPI4 treatment induced a 30% reduction in the volume of ER segmented objects at week 2 and an additional 20% reduction at week 4 (1.2 ± 0.3 μm^3^), suggesting significant differences in protein synthesis and secretory capacities of polarized and non-polarized cells (Fig. 6C, Fig. S17A, and Tables S11, S12). Similarly, the average volume of mitochondrial segmented objects increased during cell polarization by 10% at week 2 and 14% at week 3 (3.9 ± 0.5 μm^3^ week 1 vs 4.9 ± 0.6 μm^3^ week 3), while it decreased in non-polarized cells by 28% at week 2 and 11% at week 3 (2.5 ± 0.6 μm^3^ week 3). Of note, and unexpectedly, the volume of TOM20 decreased drastically to 3.9 ± 0.7 μm^3^ at week 4 of PGE2-treated cells and increased to 3.2 ± 0.7 μm^3^ at week 4 with HPI4 (Fig. 6C, Fig. S17B, and Tables S11, S12). GLM curve fitting exhibited two non-stochastically opposite rate changes for volumes of these organelles for the two states of polarization. PGE2 produced a decelerating curve with local maxima at weeks 4.2 for SC61B and 2.8 for TOM20, while HPI4 generated an accelerating curve with local minima at weeks 3.4 for SC61B and 2.7 for TOM20 (Tables S13, S14). Interestingly, the average volume of Golgi apparatus segmented objects decreased for both PGE2 and HPI4-treated cells and showed a 57% decrease at week 4 in the presence of PGE2, while a gradual decrease was observed with HPI4. At week 4, the average volume was similar between the two treatments (0.6 ± 0.2 μm^3^ PGE2 vs 0.7 ± 0.3 μm^3^ HPI4). This suggests the Golgi apparatus average volume is not associated with a polarized phenotype of RPE cells, but possibly related to the proliferation status of the cell. Similar to SC61B and TOM20, curve fitting showed a volume deceleration in the presence of PGE2 and acceleration with HPI4, although the rate change was milder (Tables S13, S14). CETN2 volume slightly increased (13%) at week 4 in polarized cells, while no change was detected with HPI4 (Fig. S18B). This change could be related to the length of the procentriole, which has been described to vary with cell state and phases of cell cycle, becoming longer in G1, in various other cell culture models, including RPE-1 cell line^38,39^. Interestingly, the number of SC61B and TOM20 objects decreased when cell polarization was induced with PGE2 (SC61B: 89.4 ± 12.3 objects per cell at week 1 vs 35.3 ± 4.4 objects per cell at week 4; TOM20: 31.6 ± 2.7 objects per cell at week 1 vs 16.8 ± 2.1 objects per cell at week 4) (Fig. 6D, Fig. S18A, and Tables S11, S12). Fitted mathematical model predictions indicate accelerating trends for objects/cells for both treatments, suggesting a strong impact of polarization states on the numbers of these organelles (Tables S13, S14). A difference in the number of objects was not as evident for SIAT1 and CETN2 (Fig. S18A and Tables S11, S12). To establish if the ER and mitochondria biomass was maintained with RPE polarization, we calculated the organelles' total volume (Fig. S18C). We found that the total ER volume per cell gradually decreased with time, reaching a 50% decrease with PGE2 (82.0 ± 20.9 μm^3^) and 40% decrease with HPI4 (99.4 ± 37.3 μm^3^). Mitochondrial total volume per cell decreased similarly for both treatments until week 3; however, the total volume decreased further with PGE2 (44% reduction, 62.0 ± 11.7 μm^3^), while it increased with HPI4 to the same volume as in week 1 (108.7 ± 23.6 μm^3^). Golgi’s total volume remained constant for both treatments until week 4, where we observed a significant reduction as compared to week one (3.8 ± 2.1 μm^3^ PGE2, 5.4 ± 4.9 μm^3^ HPI4). Overall, a lower number of mitochondria with larger average volume suggests that mitochondria fuse during cell polarization, possibly indicating a higher level of oxidative phosphorylation, a phenomenon already observed in hepatocytes^36^. The loss of mitochondrial biomass suggests that fusion is preceded by mitophagy or some other mechanism of mitochondrial degradation. An alternative explanation could be that smaller mitochondria are selectively degraded, leaving fewer but larger mitochondria, potentially more efficient in ATP production. In addition, cell polarization reduced the ER fragmentation, hinting at the formation of a stable ER network at week 4^40^ and an increase in the synthesis of membrane lipids^41^. The reduction in ER mass was observed independent of the polarization status of the cell, hinting that this phenomenon could be related to the proliferation status of a cell or is used for cytoplasmic space optimization.

### RPE polarization regulates the number of vesicular organelles

**Fig. 7:**
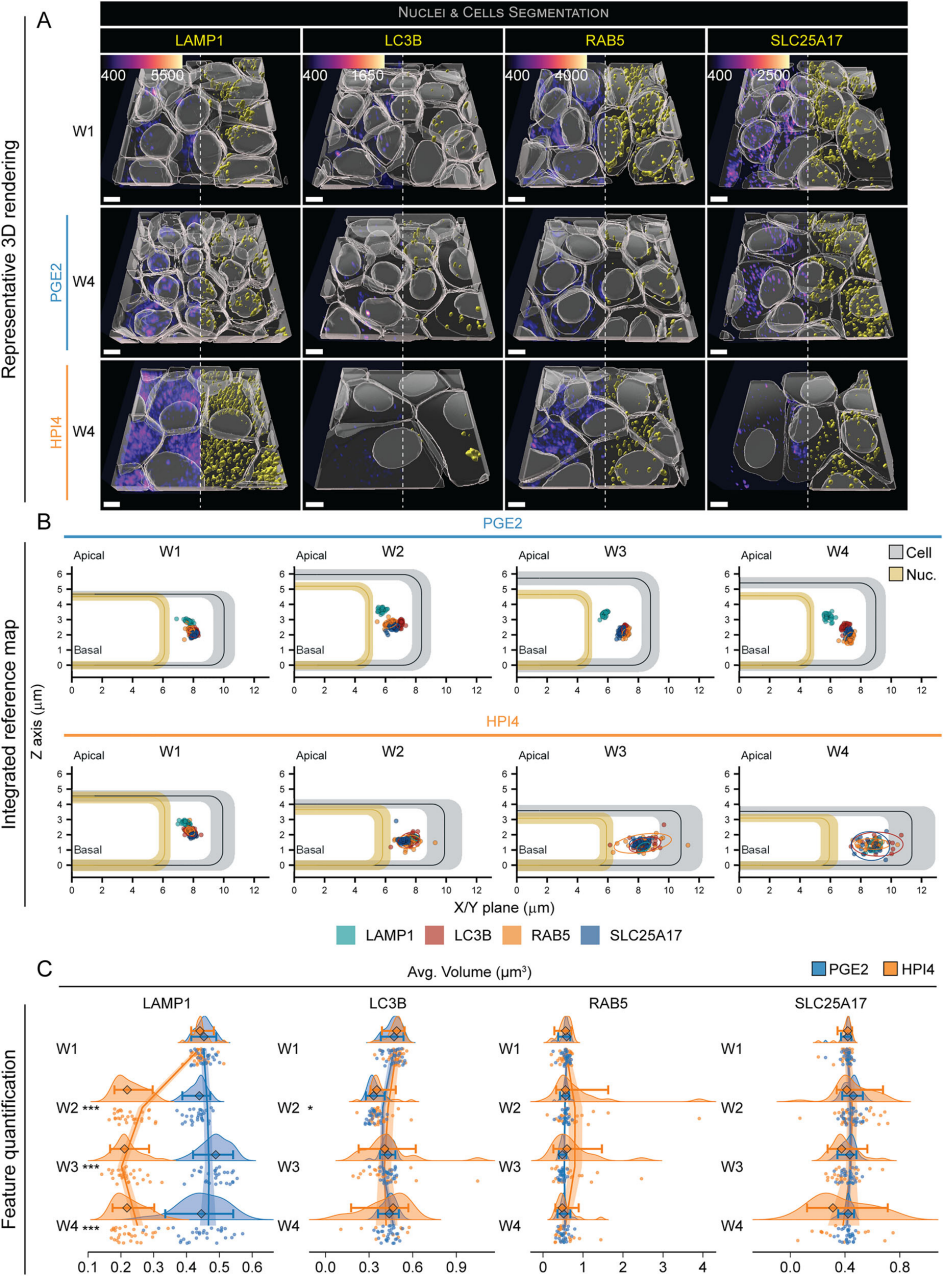
Vesicular organelle location and number change with RPE polarization. **A** Representative 3D reconstructions of iRPE cells expressing GFP-tagged LAMP1 (lysosomes), LC3B (autophagosomes), RAB5 (endosomes), and SLC25A17 (peroxisomes). Images from W1 constitute the baseline, while images at W4 show the endpoint for cells that were grown in the presence of PGE2 or HPI4 (magenta = phalloidin, cyan = Hoechst, yellow = GFP-tagged protein; scale bar = 5 μm). Note that the signal from phalloidin was stripped from the top of the cells in these images to better show the reconstruction of the GFP-tagged protein. **B** Integrated reference maps showing the location of the structure of interest within the cell. The colored points correspond to the average location of each structure of interest from each field of view (*n* = 30). Each group of points is surrounded by an ellipse, which represents the 90% confidence interval. Cells and nuclei dimensions from Fig. 2 are used as a reference. **C** Raincloud plots indicating the changes over time and between treatments for volume (the points indicate the average values for each FOV; the diamonds represent the median, and the error bars indicate the 5th and 95th percentiles. Two-sided Welch’s *t*-test was performed to compare PGE2- and HPI4-treated cells at each timepoint. *N* = 30; ***P < 0.005, *P < 0.05). The solid lines indicate the generalized linear model maximum likelihood fit for the two treatments with week and the square of week as dependent variables.

A fine regulation of lysosome, endosome, autophagosome, and peroxisome biogenesis and trafficking is necessary to maintain functional homeostasis of epithelial cells^4^. These vesicular organelles are involved in specific functions such as protein and lipid degradation or their transport and secretion to the apical or basolateral domains^42^. We imaged iRPE cells that express GFP-labeled lysosomal associated membrane protein 1 (LAMP1, line: LAMP1, lysosomes), autophagy-related protein LC3B (MLP3B, line: LC3B, autophagosomes), Ras-related protein Rab-5A (RAB5A, line: RAB5, endosomes), and peroxisomal membrane protein PMP34 (PM34, line: SLC25A17, peroxisomes) to evaluate the morphometric changes that occur during RPE polarization for each of these organelles. We hypothesized that the segregation of a pool of vesicles in a specific cytoplasmic domain during cell polarization would signify the development of a specific function, potentially via a new interaction with another intracellular organelle. Instead, an increase in their abundance or volume might indicate a higher activity of that specific organelle. 3D reconstructions revealed that, among these vesicular organelles, lysosomes undergo the largest change in subcellular distribution upon polarization; they translocate towards the center of the cell and apically to the nucleus when cells were treated with PGE2 (Fig. 7A and Figs. S19, 20). When comparing the abundance of these organelles in polarized RPE cells, we found that peroxisomes were the most numerous, suggesting an important role of these organelles in cellular lipid handling and metabolism in RPE cells (PGE2 week 4: 90.8 ± 16.5 objects per cell, as compared to 22.6 ± 7.8 (endosomes), 9.4 ± 2.5 (autophagosomes), 18.3 ± 3.8 (lysosomes); Fig. 7A, Figs. S19, 20, S15G, and Tables S11, S12). The average distance from the apical and lateral RPE cell membrane shows that, on average, lysosomes are translocated centrally and apically to the nucleus during cell polarization. This movement didn’t happen in the presence of HPI4. The location of autophagosomes and peroxisomes remained constant across timepoints during polarization, while the endosomes' average distribution slightly shifted towards the basal side of the cell (Fig. 7B and Fig. S21E, F). Comparing the average volume of each vesicular organelle, we discovered that lysosomes were the only organelles to show a drastic difference in volume. When apical/basal polarity was disrupted, the average lysosome volume decreased by half, starting from week 2, as compared to the PGE2 condition and to week 1, and remained constantly low until week 4 (0.45 ± 0.02 μm^3^ week 1 vs PGE2 0.45 ± 0.07 μm^3^ vs HPI4 0.23 ± 0.05 μm^3^; Fig. 7C, Fig. S21A–D, and Tables S11, S12). While PGE2 didn’t induce significant changes in LAMP1 volume over time, GLM predictions showed a slight nonlinear decrease in volume with an accelerating rate change (Tables S13, S14). Interestingly, cell polarization induced a slight decrease in the average number of lysosomes and endosomes per cell by week 4 (37 and 33% compared to week 1, respectively), and a slight increase in the number of autophagosomes and peroxisomes (77 and 33% compared to week 1, respectively) (Fig. S21G and Tables S11, S12). The different balance in the number and size of these vesicular organelles between polarized and non-polarized cells probably reflects the differences in the cellular metabolic status, as they act as critical intracellular signaling hubs^43^. It also highlights a likely critical role played by lysosomes and peroxisomes in polarized RPE cells, as has been suggested by the literature^44,45^.

### Average pairwise spatial interaction maps of subcellular structures

**Fig. 8:**
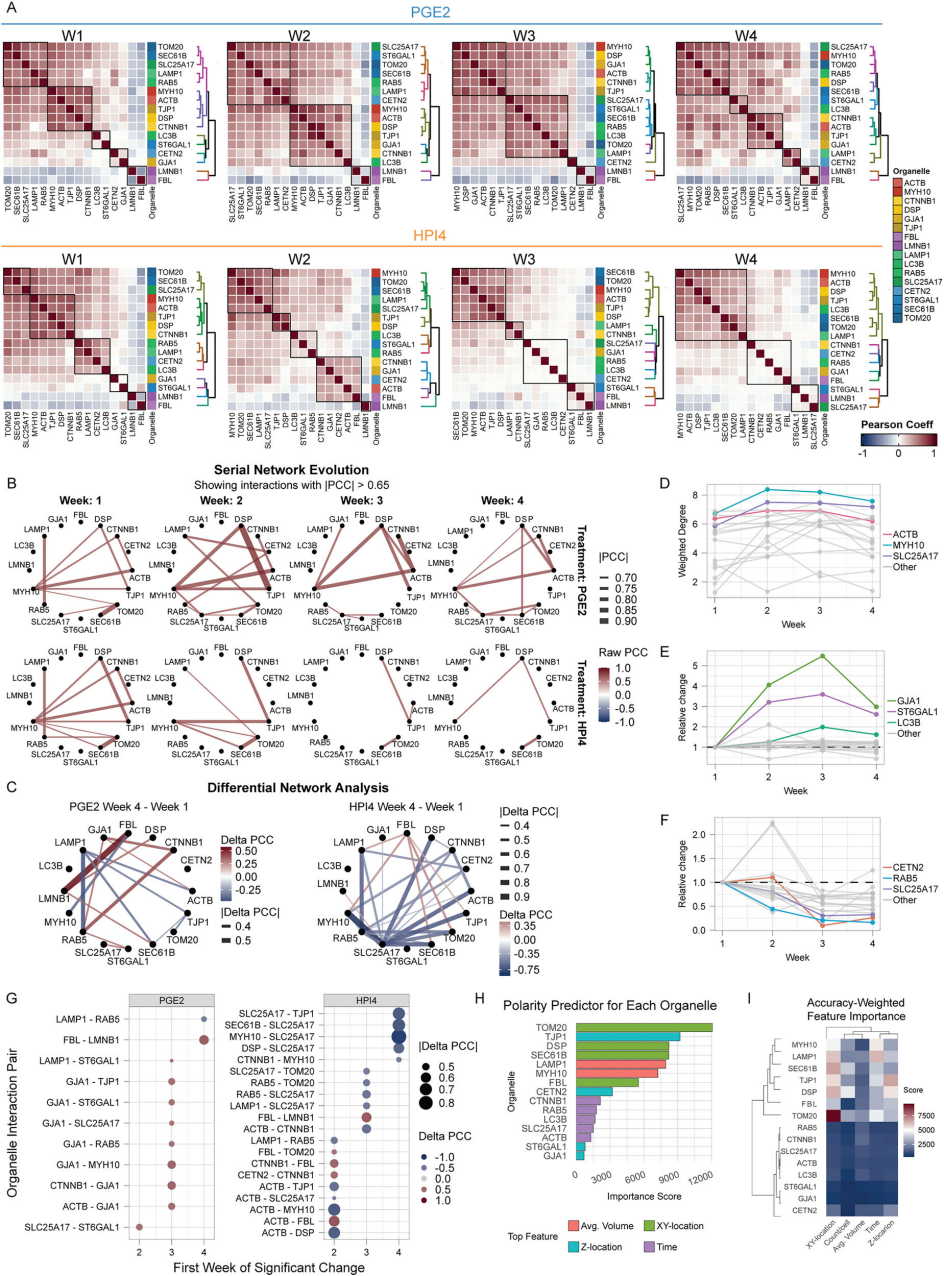
Spatial interaction maps of integrated cellular structures. **A** Each correlation heatmap shows the pairwise interaction between intracellular structures within each timepoint and treatment condition. Pearson correlation coefficient (PCC) was used to measure the spatial interaction of morphed signals of the intracellular structures after cell alignment with hexagon-based template matching. A clustering algorithm was applied to each triangle heatmap to generate dendrograms displaying the distance between clusters. A color code based on structure location was assigned to each intracellular structure. **B** Serial network evolution graph showing changes in organelle connectivity with time. Line color indicates PCC values, while line width corresponds to absolute values. **C** Network graph representing the difference between PCC values at week 4 and week 1 with PGE2 (left) and HPI4 (right). **D** Line graph displaying organelles weighted degree of centrality of PGE2-treated cells across weeks. Colored lines are the top three organelles with the highest average value. **E** Line graph showing the relative change of weighted degree to week 1 for PGE2 and **F** HPI4. **G** Dot plot shows the first week of significant change in interaction. The color indicates PCC difference to week 1, and dot size displays the absolute change. **H** Organelle-specific features were extracted from Random Forest predictions and ranked by importance. **I** Heatmap showing feature importance score weighted by model accuracy.

In addition to the individual features of intracellular organelles, their relative location with each other is important in regulating their function^46^. To estimate the spatial interactions between subcellular organelles and structures, we morphed the 3D signal of fluorescently labeled proteins onto an average cell at each condition. We then calculated a pairwise Pearson correlation coefficient (PCC) of the average signal. The organelles were clustered by spatial similarity within each condition to determine their relative proximities (Fig. 8 and Table S15). As expected, LMNB1 (nuclear envelope) and FBRL (nucleolus) showed a negative correlation with most of the other organelles in both conditions; interestingly, these values appear less negative at week 4 with HPI4. This suggests that the nucleolus and the nuclear membrane maintain a distinct subcellular location relative to other organelles. At week 1 of polarization, other cellular structures could be clustered in three groups based on their relative proximities, one with cytoskeletal and junctional proteins, one with mitochondria, ER and vesicular organelles, and one heterogeneous group formed by gap junctions, centrioles, Golgi, and autophagosomes, reflecting the intracellular spatial heterogeneity of organelles at this stage. In the presence of PGE2, this heterogeneity seems to sort out, and two main clusters emerge at week 2 and 3, one constituted by cytoskeletal and junctional organelles, suggesting that cytoskeletal and junctional proteins interact to promote RPE polarization; and the other by all the cytoplasmic organelles, suggesting resorting of organelles towards their “final” destinations. Interestingly, in fully polarized cells at week 4, three specific interactions emerged—peroxisomes (PM34)-myosin (MYH10)-mitochondria (TOM20)-endosomes (RAB5A)-desmosomes (DESP)-ER (SC61B); lysosomes (LAMP1) -centriole (CETN2); and Golgi (SIAT1)-autophagosomes (MLP3B). As reported in the integrated reference maps above (Fig. 7B), the average lysosome location appears to shift apically and centrally. These spatial interaction maps indicate that lysosomes and centrioles occupy a similar intracellular domain at week 4 with PGE2, suggesting a key role of lysosomal apical location not only in RPE polarization but also in regulating primary cilia function. Interestingly, a static cloud of lysosomes has been reported around the microtubule-organizing center, further supporting the interaction between the two organelles^47^. The interaction between Golgi and autophagosomes suggests a yet undiscovered role for autophagy during cell polarization and for the Golgi complex as a site for autophagy regulation in RPE^48^. We validated these specific spatial interactions between lysosomes and centrioles, Golgi and autophagosomes, by immunostaining of respective markers in polarized cells (Fig. S22A, B). Indeed, the immunostaining confirmed that the organelle pairs occupy a similar intracellular domain. Lastly, the proximity of peroxisomes, with mitochondria, myosin, endosomes, desmosomes, and ER, suggests a highly dynamic metabolic change occurring in polarized RPE cells. To understand the spatial organization of organelles in this group, we co-stained ER and mitochondria, since they also underwent similar morphometric changes (Fig. 6 and S17). We observed that this pair partially interacted basally, around the nucleus; however, mitochondria mostly localized laterally to the nucleus, while ER expanded apically to the nucleus (Fig. S22C). A validation of these proximity maps was provided by cells treated with HPI4 where an overall reduction in organelles communication was observed—as most Pearson coefficient values were closer to 0, starting from week 2, and more obvious at weeks 3 and 4. Expectedly, baseline interactions that were still visible at weeks 3 and 4 were between cytoskeletal structures, tight junctions, desmosomes, ER, and mitochondria. Overall, the loss of apical-basal polarity led to a drastic reduction in spatial organelle communication, suggesting an impairment of asymmetric organelle segregation. To confirm our in vitro discoveries in native human RPE, we labeled RPE cell borders and nuclei in tissue from age-matched healthy and AMD donors. We frequently observed round, regular nuclei with “wrinkled” membranes in healthy tissue, while RPE at the edge of geographic atrophy in AMD donors presented dysmorphic morphology with flat, elongated nuclei with smooth borders (Fig. S22D**)**. We also co-stained tissues with markers for lysosomes and centrioles, and ER and mitochondria. Similar to our observations in iRPE, the organelle pairs occupy a similar intracellular domain; however, mitochondria are mostly localized basally around the nucleus, while ER expanded apically to the nucleus (Fig. S22E, F).

To further investigate which organelles become a hub of polarization, we constructed a serial network analysis graph based on PCC to visualize longitudinal changes of organelle connectivity. We observed that the actomyosin cytoskeleton (MYH10 and ACTB) constitute a major hub throughout polarization, connecting two mostly independent network, one involving cytoplasmic organelles (peroxisomes, ER, and mitochondria), and the other constituted by junctional proteins (desmosomes, adherens, and tight junctions). When polarization is blocked, the connection between myosin and actin is lost as early as the second week, and the remaining interactions that are still observed at later timepoints are the ones between desmosomes and tight junctions, and between the ER and mitochondria. We then calculated the difference in PCC between weeks 1 and 4 to examine which interaction changed the most. The most different positive interaction in polarized cells occurred between the nuclear envelope and nucleoli (LMNB1 and FBL), while lysosomes (LAMP1) appeared to develop the most negative interaction, likely due to local segregation to the apical intracellular domain. Cell polarity inhibition mostly results in negative interactions, with peroxisomes appearing at the center of a hub. To further establish which organelles and structures become central hubs of polarization, we calculated a dynamic “weighted degree of centrality” on PGE2-treated cells by summing the strength of connection for each organelle at each timepoint. We found that the actomyosin cytoskeleton appears again as the most connected structure overall across all timepoints, together with peroxisomes. To understand which organelle developed the strongest connections over time, we plotted the relative change of centrality to week 1. We observed that gap junctions, autophagosomes, and Golgi showed the most prominent increase in connectivity. Conversely, we calculated the organelles' dynamic degree of centrality when polarization was prevented to find which organelles display the largest change of connectivity. We found that centrioles, endosomes, and peroxisomes showed the largest loss of connectivity, suggesting that interactions involving these organelles may be important for RPE health. In order to establish a temporal sequence of specific interactions that arise or vanish, we calculated the first week of significant change in PCC to week 1 (threshold = 0.45). When polarization is induced, the first interaction that we observed at week 2 is between peroxisomes and Golgi, followed at week 3 by interactions between cytoskeleton and cellular junctions (GJA1, ACTB, CTNNB1, MYH10, and TJP1), and at week 4 by nuclear consolidation and spatial segregation of endosomes and lysosomes. When polarization is inhibited, we first observed increased correlation between nucleoli and actin, beta-catenin, and mitochondria, reflecting the increased variability in intracellular organelles location, and a decrease in interaction between cytoskeleton and cell junctions (ACTB, DSP, MYH10, and TJP1). At weeks 3 and 4, most of the loss in connectivity involves interactions with peroxisomes, consistent with the previous differential network analysis (Fig. 8C), indicating that peroxisomes are at the center of the mechanisms of polarity loss. We then asked which organelle, and which features constitute the best morphological and spatial biomarker of polarization that can potentially be used to diagnose RPE cell dysfunction in disease models. To address this question, we trained fifteen separate supervised machine learning models (Random Forest classifiers, one for each intracellular structure except for LMNB1, for which we did not calculate shape metrics), to predict cell polarity. All models showed a level of accuracy above 0.68, with Golgi being the most useful organelle for predicting cell polarity (accuracy = 0.90) (Fig. S23A). To further understand what the most predictive feature for each organelle is, we extracted the features with the highest importance score. We found that the lateral distribution of mitochondria was the single most important feature, followed by tight junction location along the apical-basal axis. Lastly, we multiplied each feature’s importance score by the accuracy of the model it came from to obtain the true impact score (accuracy-weighed importance). A feature will have a high score only if it is very important for its model and if it is part of a model that was actually predictive of polarity. The analysis clearly divided organelles into two clusters by importance and highlighted the importance of mitochondrial lateral distribution as the most predictive feature. As cells treated with HPI4 showed significant variability across many of the analyzed cell and intracellular organelle data, we reasoned that analyzing organelle features between cells that have completely lost polarizations and cells that have only partially lost polarization may help identify biomarkers of cell dysfunction and possible therapeutic targets. Using cell height, we divided HPI4-treated cells into low, medium, and high polarity groups and re-trained a classifier model for each organelle to predict cell polarity from organelle features. Golgi was the most predictive organelle overall (accuracy = 0.80) (Fig. S23B). Subsequently, we evaluated which feature was the most important for each organelle separately. Feature extraction revealed that the organelle location along the Z axis was the most useful metric, specifically for mitochondria, tight junctions, desmosomes, lysosomes, and ER, followed by the average volume of their segmented particles (Fig. S23C, D).

Lastly, to provide a comprehensive view of the overall space occupied by each organelle in relation to all others and to the cell volume at each specific condition, we calculated a relative percentage of the intracellular volume that is occupied by each organelle (Table S16). The nucleus occupies most of the intracellular volume in both treatments (30–33%), and the volume remained stable with time. Other structures that occupy major percentages of volume are the actomyosin cytoskeleton (ACTB, MYH10), ER, and mitochondria, with 5–14% of volume occupied by each. Despite ER and mitochondria showed a reduction in volume with time (Fig. S18), we observed a relatively stable volume when calculated in relation to cell volume for both PGE2 and HPI4 conditions. The volume occupied by vesicular organelles ranges from 0.3 to 4.5%, with peroxisomes being the most abundant, confirming our previous data. We also observed that DESP volume increased from ~1% at week 1 to 4.4% at week 4 when cells polarized, while CXA1 volume decreased from 2.2 to 1%, further underscoring the relatively different importance of these two proteins in the establishment of cell polarity.

## Discussion

RPE cell culture models have been successfully used to model retinal diseases where RPE cells lose apical/basal polarity, such as in L-ORD and AMD^7,17^, or where RPE cells fail to polarize, such as in ciliopathies^11^. This study developed an AI algorithm (POLARIS) to generate a digital twin of polarized RPE cells and develop an atlas of intracellular organelles and cytoskeletal structures in fully polarized and non-polarized RPE cells. These integrated AI-driven maps will provide reference when studying disease cellular phenotypes of the RPE and will help discover subcellular anomalies. Quantitative measurements of organelle location, count, and morphology were obtained from thousands of RPE cells for each condition to ensure statistical robustness of the data. Mathematical modeling using GLMs was employed to investigate if the transitions between cell states during polarization could be predicted. The significant interaction terms across multiple models and variables indicated that cell state transition towards a polarized state is non-stochastic and follows a deterministic path. In contrast, more stochasticity is seen in cell state transition towards a non-polarized state. Non-stochastic and deterministic nature of RPE polarization is particularly evident with organelle-organelle spatial proximity. As cells polarize, specific organelles come to juxtapose, but they continue to be randomly distributed if cells do not polarize.

POLARIS helped determine that RPE cells shrink laterally to polarize along the apical/basal axis. This change in shape, referred to as anisotropic contraction, occurs when forces are generated only along one axis, and typically occurs in cells that establish an axis of polarity^49^. Interestingly, as the cell contracted laterally to polarize, the nucleus also contracted laterally and became more circular with a lower aspect ratio. This phenomenon of the nucleus following changes in cell shape, is known to coordinate cell morphology with gene expression in other cell types^50–53^. Interestingly, round nuclei that occupy a large fraction of the intracellular space, as we have found in fully polarized RPE cells, were described to affect tissue morphogenesis in the vertebrate retina through a mechanism called “nuclear jamming”^54^. Tight packing of neighboring nuclei limits tissue movements during development and may mechanically influence RPE apical-basal polarization. Changes in shape metrics, such as volume and aspect ratio, influence intracellular signal transduction, cargo transport, induce signaling gradients that affect the energy and time requirements for cargo transport, and induce gene expression changes^55,56^. Two key observations support relevant shape metrics changes in RPE polarization affecting cellular physiology. First, during RPE polarization, the ratio of cytoplasmic to nuclear volume increased from week 1 to week 4, because the cell maintained its volume, whereas the nuclear volume decreased with time, suggesting an effect on gene expression due to changes in signaling gradients. Furthermore, we discovered the development of invaginations of the nuclear envelope when cells polarize. A similar invagination of the nuclear envelope was described in hematopoietic stem cells^30^. These invaginations are caused by microtubules during cell differentiation, regulate gene expression and commitment of cells towards a specific hematopoietic lineage^30^. Another study demonstrated nuclear invaginations in epithelial cells were caused by actin and intermediate filaments that were directly linked to desmosomes and hemidesmosomes, thereby mechanically linking the nucleus to the extracellular environment^57^. An interesting idea coming from these observations is that changes in cell shape during embryonic RPE specification could directly regulate the expression of genes important for inducing epithelial phenotype or even of RPE signature genes. In support of this idea, a recent study carried out an electron microscopy-based volumetric reconstruction of one human parafoveal RPE cell and found that nuclear pores were aligned in distinct rows that seemed to correspond to areas adjacent to euchromatin^58^. In future studies, it would be interesting to examine if nuclear invagination is present in RPE from human tissues and if the spatial distribution of nuclear pores correlates with the invagination of the nuclear envelope. Nucleoli, that are the sites of ribosomal biogenesis and reflect the cell transcriptional activity, were found to decrease in average number per nucleus (from 3.3 at week 1 to an average of 2.1 at week 4) with the maturation status of the RPE. This suggests overall reduced transcriptional needs of fully mature RPE cells. Additionally, we measured a decrease in nuclear volume with polarization, which is associated with chromatin condensation and cell differentiation^59^. Interestingly, two nucleoli were also detected in a mature human parafoveal RPE cell^58^. Overall, our data provides input into how morphometric changes in cell structure and volume might be regulating gene expression changes and affecting cell differentiation state and cell function.

Among the intracellular organelles, the ones that showed the most different changes were the ER, mitochondria, and lysosomes. As RPE cells polarized, the ER and mitochondria moved towards the center of the cell, around the nucleus. A similar change in location was not observed for the Golgi apparatus, suggesting specificity in these translocations. In addition, when apical/basal polarization was blocked with HPI4, the ER location stayed unchanged over time, and mitochondrial location became even more eccentric, underscoring the specificity of these centripetal translocations during polarization. These observations further support a polarization-induced change in cellular metabolism and physiology. The ER and mitochondria location change is likely to enhance the crosstalk between these two organelles. The interaction between the ER and mitochondria in RPE cells has not been well studied; however, this organelle communication was reported to regulate Ca^2+^ transfer from ER to mitochondria, autophagy, and inflammasome formation in other cell types^60^. Recent studies characterized the location, number, and morphology of mitochondria in human perifoveal RPE cells^58,61^. Here, RPE cells contained between 475 and 734 ovoid mitochondria located towards the basal side of the cell. In our study, mitochondria were also localized towards the basal side of the cell (below the cell centroid, around the nucleus), but the number was significantly lower (week 4 PGE2: 16.8 per cell). We discovered ovoid mitochondria, but also some that were more elongated. This discrepancy in mitochondria shape and number may be related to image resolution (use of electron microscope in the previous study vs light microscope in our study) and the possible absorption of light by pigment granules that could impact our ability to correctly separate organelles when they are very close to each other. Another possibility is potential differences between specific RPE cell type (parafoveal) imaged in that study vs a non-parafoveal cells imaged in our in vitro study. Different types of RPE cells may have different physiology, as suggested recently^62^. It is also worth noting that the previous study imaged less than 8 cells whereas we imaged 10,000 cells.

In our study, lysosomes also underwent large changes with cell polarization as compared to other small vesicular organelles. Lysosomes became localized toward the center and apical side of the cell and decreased in number as cells polarized. Lysosomes have been described to be present throughout the RPE cytoplasm; however, their location could change between perinuclear and peripheral cytoplasmic regions according to the status of the cell^63^. Perinuclear lysosomes, as seen in our polarized cells, were described to form a “static cloud” positioned in close proximity to the centrosome^47^. This lysosome population was described to increase the efficiency of endosomes fusion with lysosomes and serves as a hub where fused vesicles are then sorted to their final location^47^. Our observation that lysosomes concentrate in an apical perinuclear region in close proximity to the centrosomes in polarized cells, supports the idea that lysosomal positioning is linked to healthy functional cells, whereas peripheral lysosomes, as seen in our non-polarized cells, are linked with stressed cells. In addition, we observed that lysosome volume decreased when we blocked cell polarization. As lysosome volume fluctuates with pH^64^, it is possible that lysosomal pH dysregulation could account for the observed difference in volume. Interestingly, the protein βA3/A1-crystallin was reported to regulate RPE apical/basal polarity and lysosomal pH^8,65^, thereby providing evidence that the two mechanisms are linked.

Organelle and intracellular structure dynamics are known to change considerably within a short time, especially for highly dynamic organelles like the vesicles of the trafficking machinery^66^. For this reason, our study aimed at determining the average status of these organelles during RPE polarization. To obtain quantitative data about the average status of organelles, our study made use of high-content imaging technology, where 10 thousand cells were imaged per line per timepoint per treatment. This was made possible by seeding cells in 96-well glass-bottom plates. However, these plates do not support long-term culture of cells. For this reason, we focused on up to 4 weeks on imaging. Our GLM data clearly support RPE polarization reaching saturation between weeks 3 and 4. Furthermore, the height of cells grown in 96-well glass-bottom plates was similar to the height measured on cells grown on porous plastic transwell® inserts (6.79 µm), suggesting similar polarization between these two culture conditions for these timepoints. Although our spatial proximity data captures dynamic changes in the location of organelles during polarization, it is limited to weekly measurements. In the future, it would be interesting to analyze how the intracellular machinery changes when RPE performs other important functions, such as phagocytosis of photoreceptor outer segments, or visual pigment recycling.

One of the strengths of this study is the AI-based, unbiased quantification of a large dataset collected longitudinally as cellular features polarize. As we analyzed over half a million cells, we noticed some degree of variability in the distribution of measurement points. We believe most of the variability stems from biological differences between cells, especially for the conditions where cell polarization is blocked with HPI4, as cell size and shape become stochastically irregular. However, other factors can also contribute to this variability: (1) RPE cells are phenotypically heterogeneous depending on their location in the retina^20^, hinting at morphometric variability of their intracellular organelles and structures. Although our iRPE phenotype seems to be closer to macular RPE cells^62^, additional research is needed to evaluate the similarity to specific RPE subtypes, especially since we noted some differences to the published single-cell morphometric data for parafoveal RPE cells; (2) the activity of RPE cells in vivo is influenced by circadian rhythm^67^. Although RPE cells in culture were shown to express gene regulators of the circadian clock, their expression is not synchronized without the addition of a synchronizing agent^68^. This asynchrony averages out any individual cell’s phenotype dictated by the circadian clock, but it might contribute to baseline cell-to-cell variability. This factor likely doesn’t diminish the comparative analysis of a polarized vs a non-polarized RPE cell presented here; (3) The high-content approach that we adopted in this study comes with a minor trade-off in image resolution. Some of the variability present in our quantification may be explained by our inability to distinguish small organelles that are in close proximity to each other. The size of small organelles like lysosomes, endosomes, autophagosomes, and peroxisomes ranges between 0.05 to 1 µm^69,70^. With the resolution used in our study, we wouldn’t be able to distinguish organelles on the lower end of this range. Nevertheless, we were able to detect relative changes in volume even in small organelles like lysosomes when compared between HPI4 and PGE2-treated cells, suggesting that the image resolution used in this study is sufficient to detect at least some subtle changes; (4) We noticed that the Mask R-CNN algorithm (POLARIS) wasn’t as efficient in correctly detecting cell boundaries in conditions where cells were more elongated and irregular. To reduce the variability of image segmentation quality between timepoints, we trained POLARIS on images from all timepoints and included significantly more images from week one to compensate for the higher degree of variability. Furthermore, we used segmented images from week one and week 3 for “the human in the loop” process of parameter selection, to ensure robust segmentation quality.

Despite observing different number of cells between HPI4 and PGE2-treated cells, we attribute most of the observed organelle morphological and positional changes to differential cell polarization, not proliferation. Since both compounds were added right after the first timepoint (week 1), when the RPE monolayer is already fully confluent, the difference in cell number is mainly due to the dividing cells that fill the space left by polarization. Additionally, both PGE2 and HPI4 inhibit cell proliferation. PGE2 does so via canonical WNT inhibition, while HPI4 inhibits the dynein motor needed for cell division, completely inhibiting cell proliferation^11^. The most dramatic change in cell density between the two conditions occurs between week 1 and week 2, as can be observed by the quantification of cell cross-sectional area shown in Fig. 2C. If the observed changes of organelles structures were solely dependent on the difference in cell density, we would only see differences between week 1 and week 2, which is not the case with most organelles. The observed relationship between cell polarization and proliferation is paralleled by in vivo observations. In fast-developing species such as zebrafish, where the eye develops in a little more than a day, RPE proliferation is limited to the initial stages, and RPE cells rely on lateral stretching to fully envelop the outer retina. In contrast, in slow-developing species such as the mouse and human, the slow growth of the optic vesicle gives time for the RPE cells to proliferate to keep the pace of eye growth. As a result, RPE cells don’t stretch laterally, but become a monolayer of cuboidal cells. Indeed, there is a clear correlation between the rate of proliferation and length of RPE apical-basal axis^71,72^.

This study provides significant advancements in the field of quantitative cell biology in studying cell polarity. We used an iPSC-generated, fully polarized, and post-mitotic cell type to generate a longitudinal and holistic view of RPE polarization, a developmental process that is not amenable to studying in human tissues in vivo. Since iPSCs can be derived from patients, and given that several diseases—such as ciliopathies^73^—are linked to disruptions in apical-basal polarity in RPE and other epithelial tissues^74^, our findings are directly applicable to advancing the understanding of disease-related changes in subcellular structures and facilitating mechanism-based drug discovery. In addition, while most studies focus on the role of a few specific proteins or organelles in regulating cell polarity, this work was designed to generate a holistic view of changes occurring in most intracellular organelles when polarization is induced or impaired. In our approach, we embraced systems biology-based new technologies, which are unbiased, large-scale, quantitative and multivariate, to address classical cell biology questions. Our work also extends the technical pipeline developed by the Allen Institute^75,76^. We quantified and compared longitudinal phenotypic changes upon chemical perturbation in a cell type that presents exceptional challenges. Specifically, RPE cells develop melanosomes, which absorb light at various wavelengths, quenching the fluorescent signals, and grow apical processes, making cell border segmentation more difficult. Our high-content imaging and AI-based pipeline offers a viable approach for anyone that needs to deal with these challenges, for instance for several diseased and degenerative cells that show lipofuscin and lipid-based accumulations, light-absorbing melanin, and changes in shape and size. Lastly, by differentiating sixteen of the fluorophore-tagged iPSC lines developed by the Allen Institute for Cell Science^15^ into terminally differentiated RPE cells, our work demonstrates the utility of this precious resource for the entire cell biology community.

In this study, we used a small molecule treatment to mimic the RPE phenotype of ciliopathy patients; however, POLARIS ability to pick up on subtle differences of intracellular phenotypes in other diseases, such as AMD and ciliopathies, remains to be tested. Our analysis suggests that because of the intrinsic cell-to-cell variability, subtle intracellular changes will likely be hard to detect if comparison is done at the single-cell level. Therefore, we recommend that these kinds of studies should be carried out using a quantitative, population-based approach. We believe this is a major strength of our study as it provides an unbiased, large dataset-based approach to analyzing changes seen at the cellular and intracellular level.

In summary, with the use of endogenously tagged proteins, high-content imaging, AI-based algorithms for image analysis, and mathematical modeling, this study provides a quantitative reference map of RPE cell, nucleus, and intracellular organelles and structures during the acquisition of a polarized phenotype. Among the most interesting findings, we noted that apical/basal polarization induced invaginations of the nuclear envelope, movements of ER, mitochondria, and lysosomes to specific cellular compartments, and a change in their number and volume. This 3D map will be a useful reference to discover the subcellular basis of diseases that affect RPE polarity, and most importantly, will promote the generation of quantitative, high-content data-driven models to study the biology of epithelial and non-epithelial cells.

## Methods

### iPSC line validation for RPE differentiation

All the iPSC lines used in this study were generated and characterized by the Allen Institute for Cell Science^15,75,77^ and were purchased from the Coriell Institute (Allen Cell Collection). Every iPSC line was generated from the same human parental WTC-11 iPSC line, using CRISPR/Cas9-mediated gene editing, so that each expresses a single fluorescent protein tagged to an organelle-specific protein marker to visualize specific organelles or cellular structures. The complete list of the 16 cell lines used in this study can be found in Table S1 (line name abbreviations were used in the figures and supplementary tables; protein and organelle names were used when appropriate in the text). To ensure that all the iPSC lines successfully differentiated to mature RPE, we used our previously published protocol^16–18^. In fully mature RPE, we analyzed the expression of RPE maturity markers using mRNA from iRPE isolated using the NucleoSpin RNA isolation kit (Machery-Nagel, #740955). The RNA was quantified using a nanodrop spectrophotometer (ND 1000), and retro-transcribed to cDNA with the iScript™ cDNA Synthesis kit (Biorad, #1708890). cDNA was diluted in DNAse/RNAse-free water to 1 ng/µl. To evaluate the expression of RPE signature genes, we used custom 96-well gene expression plates^7^, in which we loaded 2 ng of cDNA into each well with 4 μl of SYBR green and water. qPCR was performed on a ViiA 7 Real-Time PCR System (Thermo Fisher Scientific). ΔCt (threshold cycle) normalization was performed using the average Ct of multiple stably expressed reference genes: B2M, HPRT1, RPL13A, and GAPDH. ΔΔCt was derived by subtracting the ΔCt values obtained from the previous step between RPE and iPSC samples. Fold change of expression was calculated as 2^−ΔCt (RPE / iPSC)^ (Fig. S1A) to compare the relative expression of RPE markers to undifferentiated iPSCs.

Trans-epithelial electrical resistance (TER) measurements were performed to assess the confluence, mechanical integrity, and maturation of the monolayer using an EVOM2 and an EndOhm chamber (World Precision Instruments) (Fig. S1B).

Tissues used for immunostaining were fixed in 4% PFA. Samples were blocked and permeabilized for 1 h at room temperature in PBS with 1% bovine albumin serum (BSA, MP Biomedicals, 160069), 0.5% Triton X-100 (Sigma, T9284), 0.5% Tween 20 (Thermo Fisher Scientific, AAJ20605AP). Primary antibodies (LAMP1, Abcam, ab24170; Pericentrin, Abcam, ab28144; TOMM20, Proteintech, 11802-1-AP; Calnexin, Abcam, ab112995; LC3B, CST, #2775; TGN46, Millipore, SAB4200355) were diluted 1:100 and incubated overnight in PBS with 1% BSA. Secondary antibodies were diluted 1:500 in PBS with 1% BSA and added for 1 h at room temperature along with phalloidin-iFluor 647 (Abcam, ab176759), and Hoechst 33342 (10 µg/mL, Thermo Fisher Scientific, H3570). Flatmounts and cryosections from human donors were treated with TrueBlack Lipofuscin Autofluorescence Quencher (Biotium, 23007) for 2 min at 1:20 dilution in 70% ethanol and rinsed in PBS. Tissues used for cryosectioning were placed in 15% sucrose, followed by 30% sucrose until they sank. Samples were then embedded in OCT for cutting. Stained samples were imaged with Zeiss Airyscan 2.

### High-content imaging data acquisition

At day 40, immature iRPE cells were plated (~300 cells/mm^2^) on glass-bottom 96-well plates (Brooks, MGB096-1-2-LG-L) and were allowed to reach confluence and establish the monolayer for 7 days in RPE maturation media (RPE-MM) before high-content imaging was initiated. Seven days after seeding, either 50 µM of Prostaglandin E2 (PGE2, Tocris, #2296) or 30 µM of Hedgehog Pathway Inhibitor 4 (HPI4, Tocris, #4529) were added to RPE-MM to respectively induce or prevent cell polarization along the apical-basal axis^11^. Media containing the small molecules was refreshed three times a week. Four timepoints were used to monitor morphological changes of RPE structures and organelles during the initial 4-week period of polarization. At the end of each week, iRPE cells from each line were fixed in 4% PFA for 20 min. Since PGE2 and HPI4 were added at the end of the first week, no morphological differences between cells in the wells designated for PGE2 and HPI4 in the first timepoint (W1) were expected. Samples were stained for 30 min at room temperature with a PBS solution containing 1% BSA, 0.1% Triton X-100 (Sigma, T9284), Alexa Fluor 555 phalloidin (0.26 µM, Thermo Fisher Scientific, A34055), and Hoechst 33342 (10 µg/mL, Thermo Fisher Scientific, H3570) to highlight the actin cytoskeleton and nuclear DNA, respectively. High-content automated imaging was performed with a 60x water immersion objective (NA 1.2) on a Yokogawa CellVoyager 7000 spinning-disk confocal microscope equipped with two 16-bit sCMOS cameras (2560 × 2160 pixels). A 2 × 2 camera binning was applied to all channels to improve the signal-to-noise ratio. Tridimensional Z-stacks were acquired for six fields of view within each well and five wells per condition (cell line, drug treatment, and timepoint). Six fields of view were randomly distributed within each well. A total of 30 imaging stacks, ~10,000 iRPE cells, were collected per condition. The homogeneity of iRPE maturation across the five wells treated with PGE2 was confirmed by visual inspection of cell morphology and degree of pigmentation. iRPE cells cultured in the presence of HPI4 showed irregular morphology and reduced pigmentation, but no noticeable variation across the five wells. Images of all four timepoints for one cell line were collected using cells obtained from the same batch of cryopreserved immature RPE cells. The dimension of a voxel in this image acquisition was 0.217 µm (X) × 0.217 µm (Y) × 0.500 µm (Z). A Yokogawa proprietary software applied dark and shading corrections on the fly, as well as geometric correction using nearest neighbor interpolation. Data was collected across 28 days of culture for the four timepoints per cell line, and 16 h of imaging per cell line, over a period of 10 months for all 16 cell lines.

### POLARIS development and cell borders, and nuclei analysis

To determine the subcellular location of the organelles and cellular structures of interest, we used cell and nucleus boundaries as a reference, which was developed using POLARIS. The phalloidin staining was used to define iRPE cell borders, while Hoechst 33342 was used to define nucleus borders. We used mask region convolutional neural network (Mask R-CNN)^78^, a framework for object instance segmentation, to develop an algorithm that we named POLARIS to predict cell and nucleus boundaries in two dimensions (2D) images. POLARIS was trained on 2D slices of 37 manually annotated stacks from our dataset, taken from each timepoint and treatment. The stacks for manual annotation were selected as follows: 13 stacks from week 1 (we selected higher number of stacks for week 1 because POLARIS wasn’t as efficient for week 1 samples), 10 from week 2, 7 from week 3, 7 from week 4. Of the stacks from week 2 to week 4, 16 were from PGE2-treated cells, and 8 from HPI4-treated cells (because week one samples also worked as a training set for HPI4 samples). Images from six different lines were used to reduce bias toward a single-cell line. This selection of stacks covers the full range of variations observed in the dataset. Representative images of iRPE cells stained with phalloidin and Hoechst 33342, and their binary segmentations are shown in Fig. S2.

### 3D integration of the POLARIS algorithm

To extend POLARIS ability to analyze cell images in 3D, an additional algorithm was developed to find and connect matching objects across z-planes. Each object on one z-plane was matched against all objects on the adjacent z-plane, and an intersection over union (IoU) score was calculated as a measure of similarity for adjacent z-planes (range: 0–1). If the score was above a threshold of 0.6, objects/segments in 2D z-planes were combined into a single 3D object. A range of thresholds from 0.5 to 0.8 was tested. Because of multiple matching objects, we assembled 3D objects based on the highest cumulative IoU out of all possible combinations. Additionally, if the top plane of a 3D object matched the bottom plane of another 3D object, and the gap was not larger than 1.5 µm (3 z-planes), these objects were combined, and the missing planes were interpolated in the closest z-planes outside of the gap. A final postprocessing step was applied to dilate the objects predicted by the POLARIS instance segmentation model since they were smaller by 2–3 pixels in each direction as compared to the ground truth data. This discrepancy occurred because the cell boundaries were defined on the exterior in the ground truth data, while POLARIS detected boundaries on the interior of the cell. The dilation operation was added to match the two cell boundary definitions, as highlighted by the improvement of the IoU score in two (week 1 and week 2) manually verified stacks that were not used as training data (Avg. IoU before postprocessing: DNA = 0.89 ± 0.038, actin = 0.88 ± 0.55. Avg. IoU after postprocessing: DNA = 0.98 ± 0.033, actin = 0.94 ± 0.64) (Fig. S3, loss metrics and precision-recall curves are available in Tables S2–S5).

### Validation of the POLARIS algorithm

As needed, imaging stacks were manually annotated by six operators. Manual annotations started with masks produced by a classical segmentation algorithm. These masks were generated by detecting the fluorescence gradient, followed by an edge detection algorithm, dilation, and erosion to close small gaps between cell borders. To ensure that minimal variation was introduced to the ground truth data by different operators, four of the 37 stacks were annotated by each person separately and subsequently compared using Pearson correlation and Sørensen–Dice coefficient (Fig. S4A). Cell borders and nuclei segmentation from each operator, classical segmentation algorithms and POLARIS were compared to each other. A high degree of correlation was found for all comparisons for nuclei segmentation (Pearson >0.81, Sørensen–Dice >0.83) and for cell border segmentation (Pearson >0.65, Sørensen–Dice >0.75). Coefficients were overall lower in the cell border matrix as compared to the nuclei border matrix, suggesting less agreement between cell border segmentations. The variability between different cell border segmentations (which showed higher variability, as compared to nuclear border segmentation) is also shown with plots of cell cross-sectional area, aspect ratio, and volume (Fig. S4B and Table S18). We calculated the percent difference between the segmentations with the lowest and the highest values for each shape metric, and discovered a 28% difference for cross-sectional area, 9% for aspect ratio, and 40% for volume.

### POLARIS integration with subcellular signals using classical 3D segmentation

The fluorescence signal from fluorophore-tagged organelles and cellular structures was segmented using classical segmentation algorithms. Fluorescence from each iRPE sample was analyzed with a unique combination of algorithms. To develop a segmentation pipeline that worked best for subcellular iRPE images, we started from the pipeline developed by the Allen Institute for Cell Science^75,76^ and modified the combinations of AI-algorithms based on their level of success for segmenting iRPE images. A detailed description of the algorithms used for each structure can be found in the Supplementary Information and Table S6. To ensure accurate organelle segmentation, we adopted a human-in-the-loop (HITL) approach, where segmentation quality was validated by three rounds of biology experts’ feedback. For each of the three rounds of HITL, two experts evaluated the quality of segmentation from eighteen 3D stacks, generated with different parameters, from two timepoints (W1 and W4) for each of the sixteen organelles (details available in Supplementary Information). The summary of the parameters used in the selection process can be found in Table S7. These data were integrated with 3D cellular data obtained using POLARIS to develop a 3D subcellular map.

### Feature analysis

The quantitative analysis of shape metrics was carried out using functions from Python libraries (see https://github.com/NIH-NEI/SegmentationAnalyzer). Some shape features, like cross-sectional area, minimum and maximum Feret’s diameters, and aspect ratio, were measured in 2D by calculating maximum intensity projections of binary segmentations since RPE cells grow in a monolayer on a flat surface. The other shape metrics, such as volume, height, sphericity, and centroid location, were calculated by maintaining the three dimensions of the image stacks (Fig. S5, feature names and definitions are available in Table S8 and at https://github.com/NIH-NEI/SegmentationAnalyzer).

### Organelle and cell structure location

To describe the location of intracellular organelles and structures, we generated integrated reference maps (Figs. 2, 5–7, B panels) that display the average cells and nuclei dimensions for each condition, calculated by averaging the dimensions of all individual cells and nuclei obtained from their segmentations over the entire dataset (individual cell and nuclei counts for all 16 cell lines are reported in Tables S9). Cell and nucleus heights were obtained by measuring the length along the Z axis, while the widths were calculated by averaging the maximum and minimum Feret’s diameters from images projected along the Z axis (Fig. S5). The location of the nucleus was calculated as the average distance between the cell and nucleus centroids along the Z axis and the X/Y plane. The integrated reference maps are shown as half cells and nuclei because organelle location was measured using absolute distances from the cell membrane or centroid. The black rectangles represent the average cell dimensions, while the surrounding gray bands represent the confidence interval estimated using two standard deviations from the average cell dimensions (95% of the data). The average nucleus dimension and its confidence interval are shown in yellow. The integrated reference maps were used as a reference onto which the locations of fluorophore-tagged organelles and structures in the 3D space were overlayed. The location of fluorophore-tagged organelles and structures within a cell was calculated as the distance on the X/Y axes of the closest voxel of the segmented object of the structure of interest from the closest lateral cell border and the distance on the Z axis from the apical cell border (Fig. S5). The location of fibrillarin was calculated in 3D by using the nucleus border as a reference. The distance between organelle and cell centroids was also calculated, as this distance is a more reliable measurement for organelles and structures that are far from cell borders (Fig. S5). Measurements of the distance between centroids are shown in Figs. S17E, F and S21E, F.

### Quality control

To verify that the results of the image analysis calculations are accurate, and the AI-algorithms are reliable, a synthetic RPE cell (hexagonal prism) of known dimensions was generated, and its shape metrics were calculated. No value discrepancies were found between calculated metrics and known values. To prevent the incorrect quantification of shape metrics, any cell touching the z-stack boundaries (top, bottom, and sides) was removed from the analysis at the segmentation stage. To eliminate segmentation artifacts, we established cutoff values based on empirical inspection of several images and the related image analysis measurements to exclude segmented cells that had an unreasonable volume lower than 50 µm^3^ or higher than 100,000 µm^3^, width lower than 1.5 µm, and height lower than 1.0 µm. The chosen object filtering values are very conservative, such that objects lying outside these cutoffs are expected to arise from segmentation errors rather than biological variability. Lastly, as the edges of our data distribution are likely caused by errors of the segmentation, we excluded from the analysis the top and bottom 5% of the shape metric values at the single-cell level.

### Organelle and cell structure spatial interaction maps

To analyze the average interaction between intracellular structures within AI-driven 3D cellular maps, individual cells were aligned using hexagon-based template matching. We used this method because RPE cells are hexagonal when projected onto the X/Y plane. Individual cell contours were computed from a maximum intensity projection of the cell segmentation and subsequently matched to a library of 2D elongated hexagons of different sizes at all possible rotations. The best matching rotation angle was used to re-orient the corresponding single-cell image. When the nucleus was polarized toward one side of the cell, an extra 180-degree rotation was applied to guarantee that all single-cell images have the nucleus either at the center or on the left side of the image. After aligning all the cells, we calculated average pairwise spatial interaction maps following the code from ref. ^75^. Briefly, using segmented images, an average cell shape and an average organelle shape were calculated for each condition, and each organelle shape was morphed onto that mean cell shape. For colocalization, we computed the correlation between a pair of organelles (A,B) as: avg_colocalization_A_B = np.corrcoef(avg_gfp_A.flatten(), avg_gfp_B.flatten()). A Pearson correlation coefficient was calculated to map the spatial interaction between the morphed signals of each intracellular structure.

### Statistical analysis

The data for statistical analysis were aggregated at the level of individual fields of view by averaging each measurement (shape metric) over all RPE cells. Each RPE cell condition was represented by a maximum of 30 data points [i.e., the number of fields of view (FOV) acquired per condition]. In addition to FOV-based analysis, we also performed statistical analysis using single cells as data points to determine if observed trends were biased or retained by FOV averaging (Figs. S25, S26 and Table S17). The data were evaluated for normality using Shapiro–Wilk’s test^79^. To compare the sample means between PGE2- and HPI4-treated cells at each timepoint, the two-sided Welch’s *t*-test^80^ was performed. To evaluate the variance between timepoints within each group (PGE2 or HPI4), one-way Analysis of Variance (ANOVA)^81^ was performed, followed by Tukey’s honest significance test (HSD)^82^ with a 95% confidence interval for pairwise comparison. Adjusted *P* values of <0.05 (*), <0,01 (**), and <0.001 (***) suggested significance. The statistical analysis was carried out in R version 4.2.1^83^. In all graphs, the boxplot limits represent the first and third quartiles, the central line shows the median, and the whiskers indicate the 5th and 95th percentiles. The graphs describing organelles’ location were generated with “ggplot”, “ggchicklet”, and “ggh4x” R packages^84–86^.

The data described in each graph was fitted using generalized linear models (GLM) with maximum likelihood estimation (MLE) with week and square of week as dependent variables, with the latter transforming the observed nonlinear dynamics to a problem which may be studied using linear methods, providing an estimation on the form y = k + mx + nx^2^, where y is the observed variable and x is the number of week (1 to 4). This allows for further evaluation of rate change by using the derivative y’ = m + 2nx; a positive n indicates accelerating dynamics, while negative indicates the opposite. Solving the latter equation setting rate to 0 allows us to find the inflection point of rate change. Furthermore, evaluating whether the contribution of each coefficient (m and n) is statistically significant allows us to evaluate whether the observed change of a specific dependent variable is stochastic or not. The GLMs were constructed using Python and the “statsmodels” package version 0.14.1. The data were fitted using assumed Gaussian distributions, except for the counted data, which was fitted using assumed Poisson distributions. For comparison, models were also fitted to strictly linear models (no square term) and compared to the corresponding nonlinear models using pseudo R-squared to evaluate model fit and Akaike Information Criterion (AIC) to compare the quality of the fit vs model complexity.

### Imaris visualization

To visualize 3D renderings of iRPE cells, nuclei, and organelles, we imported the fluorescence image stacks into Imaris, thresholded them in each channel, and generated surface meshes. The phalloidin signal was stripped on the apical side of the cells to facilitate the visualization of the organelles. Each field of view was cropped to visualize single cells. A field of view of 125 × 125 pixels was used for all images of cells at week 1 and of HPI4-treated cells at week 4, while an 80 × 80 pixels field of view was used for all images of PGE2-treated cells at week 4.

## Supplementary information

Ortolan_Supplementary Information & Tables

## Data Availability

The raw data were available at the “Retinal Pigment Epithelium (RPE) Segmentation and Intensity” Collection (10.25452/figshare.plus.c.7787951). This includes: (1) a demo dataset, and training data for Mask R-CNN-based segmentation of cells and nuclei (10.25452/figshare.plus.28832501); (2) the fluorescence intensity stacks (10.25452/figshare.plus.28824257); (3) the segmented stacks (10.25452/figshare.plus.28821213).

## Code availability

The POLARIS code developed in this study is available on GitHub (Full pipeline: https://github.com/NIH-NEI/RPE_Pipeline, Segmentation of RPE cell and nucleus borders: https://github.com/NIH-NEI/RPE_Segmentation; Segmentation of fluorescently-tagged organelles: https://github.com/NIH-NEI/RPEOrganelle_Segmenter; Cell, nucleus, and organelle shape metric calculations: https://github.com/NIH-NEI/SegmentationAnalyzer). Software distributions are available at the “Retinal Pigment Epithelium (RPE) Segmentation and Intensity” Collection (10.25452/figshare.plus.c.7787951). This includes: (1) a ready-to-go distribution for Windows for Mask R-CNN-based segmentation of cells and nuclei (10.25452/figshare.plus.28832501); (2) a Windows distribution for cell alignment and organelle morphing to calculate the average pairwise spatial interaction maps (10.25452/figshare.plus.28836248).
