# Supplementary material for: AI driven 3D subcellular RPE map discovers cell state transitions in establishment of apical-basal polarity
Source: NPJ Artif Intell. 2026 Feb 6;2(1):20. doi: 10.1038/s44387-026-00074-6 (PMC12880915; doi:10.1038/s44387-026-00074-6)
Supplement: Supplementary file 1 — Ortolan_Supplementary Information & Tables [file 44387_2026_74_MOESM1_ESM.pdf]

Supplementary Information for

AI Driven 3D Subcellular RPE Map Discovers Cell State Transitions in Establishment of Apical-Basal Polarity

Davide Ortolan[1$*], Pushkar Sathe[2$], Andrei Volkov[1], Dominik Reichert[1], Sheldon Sebastian[1], Arvydas Maminishkis[1], Nicholas J. Schaub[3], Bengt Ljungquist[3], Devika Bose[1], Jorge Ferrari[1], Nyusha Lin[1], Gianluca Pegoraro[4], Carl G. Simon Jr.[5], Ruchi Sharma[1], Peter Bajcsy[2*], and Kapil Bharti[1*]

1 Ocular and Stem Cell Translational Research Section, National Eye Institute, NIH, Bethesda, Maryland, USA.

2 Information Technology Laboratory, Information Systems Group, National Institute of Standards and Technology, Gaithersburg, Maryland, USA.

3 Information Resources Technology Branch, National Center for Advancing Translational Sciences, NIH, Bethesda, Maryland, USA.

4 High Throughput Imaging Facility (HiTIF), National Cancer Institute, NIH, Bethesda, Maryland, USA.

5 Materials Measurement Laboratory, Biosystems and Biomaterials Division, National Institute of Standards and Technology, Gaithersburg, Maryland, USA.

[$]These authors contributed equally.

*Corresponding authors: Kapil Bharti, Peter Bajcsy, Davide Ortolan

Email: kapil.bharti@nih.gov, peter.bajcsy@nist.gov, davide.ortolan@nih.gov

This file includes:

Supplementary text

Figures S1-S26

Tables S1- S19

Supplementary text

Human in the loop parameter selection

Each organelle or subcellular structure was segmented using a combination of classical segmentation algorithms depending on the morphology of each organelle (**Table S6**). The parameters used by the algorithms were selected using a human-in-the-loop approach. To optimize the selection of parameters used by the algorithm sequence, we determined an initial set of pixel values that corresponded to the measured dimensions of each organelle and structure (scale parameter). These values were converted to pixels based on the pixel/micron scale. We calculated a 50 % deviation on the upper and lower side of this set of values and used this range to carry out a first round of segmentation for each organelle. Segmentation was subsequently visually inspected by two experts, who selected the parameters that produced the best quality of segmentation. The range of parameters was then reduced by 50 % starting from the newly selected values, and a new round of segmentation was performed. A total of three rounds of segmentation with a human in the loop were carried out (**Figure S17**). For example, if the initial parameter was 1, the range of parameters for the first iteration will be 0.5, 1, 1.5. If 1.5 produced the best segmentation quality, we chose this value ± 25% (1.125, 1.5, 1.875) for the second iteration and so on. A range of cutoff values was also included in the parameter selection. These cutoff values are related to the distribution of intensities along cross sections, not to the size or shape of the objects of interest. We started from standard values for the selection process. For DSP and GJA1, a fourth round of parameter selection was carried out as the quality of the segmentation could still be improved. We were not successful in finding an algorithm that allowed us to close the segmentation of LMNB1. In this case, we

decided to stop the parameter selection at the first round and not to run the shape metric analysis for this structure.

Data type and requirements:

All stacks were in the OME-TIFF format, which is an open-source file format standard.

Segmentation algorithms for each organelle

TJP1 segmentation technique

Preprocessing

We performed auto contrast intensity normalization, which uses two scaling parameters for modifying the number of standard deviations on either side of the mean intensity. The algorithm uses 99.99% of maximum intensity as saturation intensity. Edge preserving smoothing (anisotropic diffusion filter) was performed to enhance structure edges.

Segmentation

For core segmentation, 2D Frangi vesselness was calculated for each individual frame in the stack. When compared to 3D Frangi vesselness this was found to be significantly better. Combining 2D with 3D was not significantly different from 2D vesselness, so the 2D approach was selected. Due to resolution and limitations of Frangi vesselness in intersecting filaments, some fragmentation of the segmentation is visible.

Postprocessing

No objects were removed in this phase since doing that could remove a large number of TJP1 true positive filament fragments. These fragments occurred because the filaments

are extremely thin and are not uniformly bright. As a result, parts of the filaments were not segmented.

CTNNB1 segmentation technique

Preprocessing

We performed auto contrast intensity normalization, which uses two scaling parameters for modifying the number of standard deviations on either side of the mean intensity. The algorithm uses 99.99% of maximum intensity as saturation intensity. A 3D gaussian smoothing was performed to reduce noise in the stack.

Segmentation

For core segmentation, 2D Frangi vesselness was calculated for each individual frame in the stack. When compared to 3D Frangi vesselness this was found to be significantly better. Combining 2D with 3D was not significantly different from 2D vesselness. Due to resolution and limitations of Frangi vesselness in intersecting filaments, some fragmentation of the segmentation is visible. Additionally, a 2D spot filter was used as CTNNB1 signal had some spot-like structures. To reduce the number of steps involved, both sets of parameters (vesselness and spot) were kept the same due to their similar scales. The spot filter uses a Laplacian of Gaussian (LoG), edge detection filter.

Postprocessing

Small objects were not removed to prevent the exclusion of real segmentation.

DSP segmentation technique

Preprocessing

We performed auto contrast intensity normalization, which uses two scaling parameters for modifying the number of standard deviations on either side of the mean intensity. The algorithm uses 99.99% of maximum intensity as saturation intensity. 2D slice-by-slice gaussian smoothing was performed to reduce noise in the stack.

Segmentation

A 3D LoG-based spot filter was used to perform the core segmentation. We used two sets of parameters to recognize small or large objects, and the two segmentations were combined. 3D watershed was used for separating connected objects.

Postprocessing

Further postprocessing was unnecessary as the segmentations were of acceptable quality.

GJA1 segmentation technique

Preprocessing

We performed auto contrast intensity normalization, which uses two scaling parameters for standard deviation on either side of the mean intensity. The algorithm uses 99.99% of maximum intensity as saturation intensity. 2D slice-by-slice gaussian smoothing was performed to reduce noise in the stack.

Segmentation

A 3D LoG-based spot filter was used to perform the core segmentation. We used two sets of parameters to recognize small or large objects, and the two segmentations were combined.

Postprocessing

Further postprocessing was unnecessary as the segmentations were of acceptable quality.

CETN2 segmentation technique

Preprocessing

We performed auto contrast intensity normalization, which uses two scaling parameters for modifying the number of standard deviations on either side of the mean intensity. The algorithm uses 99.99% of maximum intensity as saturation intensity. A 2D slice-by-slice gaussian smoothing was performed to reduce noise in the stack.

Segmentation

For core segmentation, a 3D LoG based spot filter was used to segment spot-like structures. 3D watershed was used to separate fused objects.

Postprocessing

Based on pixel connectivity, objects equal to or smaller than 2 pixels were removed. This value was chosen to remove a large number of false positive segmented objects.

SEC61B segmentation technique

Preprocessing

We performed auto contrast intensity normalization, which uses two scaling parameters for modifying the number of standard deviations on either side of the mean intensity. The algorithm uses 99.99% of maximum intensity as saturation intensity. Edge preserving smoothing (anisotropic diffusion filter) was performed to enhance edge-like structures.

Segmentation

2D Frangi vesselness was calculated for each individual frame in the stack.

Postprocessing

Based on pixel connectivity, objects equal to or smaller than 4 pixels are removed. Note that 5 pixels mean 5 connected voxels in 3D. This cutoff helped with removing large number of false positive segmented objects.

ST6GAL1 segmentation technique

Preprocessing

We performed auto contrast intensity normalization, which uses two scaling parameters for modifying the number of standard deviations on either side of the mean intensity. The algorithm uses 99.99% of maximum intensity as saturation intensity. A 3D gaussian smoothing was performed to reduce noise in the stack.

Segmentation

Segmentation was done using a 3D LoG filter. We experimented with combinations of masked object thresholding and topologically preserving thinning prior to the LoG filter but found they didn't improve the segmentation.

Postprocessing

Further postprocessing was unnecessary as the segmentations were of acceptable quality.

TOM20 segmentation technique

Preprocessing

We performed auto contrast intensity normalization, which uses two scaling parameters for modifying the number of standard deviations on either side of the mean intensity. The algorithm uses 99.99% of maximum intensity as saturation intensity. A 3D gaussian smoothing was performed to reduce noise in the stack.

Segmentation:

2D Frangi Vesselness using selected parameters was conducted on the normalized and smoothed images for each slice of the stack.

Postprocessing:

Based on pixel connectivity, objects equal to or smaller than 2 pixels were removed. This value was chosen to remove a large number of false positive segmented objects.

LAMP1 segmentation technique

Preprocessing

We performed auto contrast intensity normalization, which uses two scaling parameters for modifying the number of standard deviations on either side of the mean intensity. The algorithm uses 99.99% of maximum intensity as saturation intensity. 2D slice-by-slice gaussian smoothing was performed to reduce noise in the stack.

Segmentation

A 2D LoG Filter was used on each frame to obtain segmentation.

Postprocessing

Based on pixel connectivity, objects equal to or smaller than 3 pixels are removed. Note that 3 pixels means 3 connected voxels in 3D. This cutoff helped with removing a large

number of false positive segmented objects. Inversely, connected toroidal areas with holes in the segmentation were filled.

LC3B segmentation technique

Preprocessing

We performed auto contrast intensity normalization, which uses two scaling parameters for modifying the number of standard deviations on either side of the mean intensity. The algorithm uses 99.99% of maximum intensity as saturation intensity. 2D slice-by-slice gaussian smoothing was performed to reduce noise in the stack.

Segmentation

A 3D LoG Filter was used to obtain particle segmentation. Subsequently, 3D Watershed was performed to separate connected objects into individual particles.

Postprocessing

Based on pixel connectivity, objects equal to or smaller than 4 pixels are removed. Note that 4 pixels mean 4 connected voxels in 3D. This cutoff helped with removing a large number of false positive segmented objects.

RAB5 segmentation technique

Preprocessing

We performed auto contrast intensity normalization, which uses two scaling parameters for modifying the number of standard deviations on either side of the mean intensity. The algorithm uses 99.99% of maximum intensity as saturation intensity. 2D slice-by-slice gaussian smoothing was performed to reduce noise in the stack.

Segmentation

A 2D LoG filter was used to obtain segmentation of RAB5 particles.

Postprocessing

Based on pixel connectivity, objects equal to or smaller than 4 pixels are removed. Note that 4 pixels mean 4 connected voxels in 3D. This cutoff helped with removing a large number of false positive segmented objects. Additionally, any enclosed empty areas were filled slice-by-slice.

SLC25A17 segmentation technique

Preprocessing

We performed auto contrast intensity normalization, which uses two scaling parameters for modifying the number of standard deviations on either side of the mean intensity. The algorithm uses 99.99% of maximum intensity as saturation intensity. A 2D slice-by-slice gaussian smoothing was performed to reduce noise in the stack.

Segmentation

A 3D LoG Filter was used to obtain object segmentation. Subsequently, connected objects were separated using 3D watershed.

Postprocessing

Further postprocessing was unnecessary as the segmentations were of acceptable quality.

ACTB segmentation technique

Preprocessing

We performed auto contrast intensity normalization, which uses two scaling parameters for modifying the number of standard deviations on either side of the mean intensity. The algorithm uses 99.99% of maximum intensity as saturation intensity. Edge preserving smoothing (anisotropic diffusion filter) was performed to enhance edge-like structures.

Segmentation

2D Frangi vesselness was calculated for each individual frame in the stack.

Postprocessing

No objects were removed in this phase since that may remove a large number of actin filaments. These fragments occurred because the filaments are extremely thin and are not uniformly bright. As a result, parts of the filaments were not segmented.

MYH10 segmentation technique

Preprocessing

We performed auto contrast intensity normalization, which uses two scaling parameters for modifying the number of standard deviations on either side of the mean intensity. The algorithm uses 99.99% of maximum intensity as saturation intensity. Edge preserving smoothing (anisotropic diffusion filter) was performed to enhance edge-like structures.

Segmentation

2D Frangi vesselness was calculated for each individual frame in the stack.

Postprocessing

No objects were removed in this phase since that may remove a large number of myosin filaments. These fragments occurred because the filaments are extremely thin and are not uniformly bright. As a result, parts of the filaments were not segmented.

FBL segmentation technique

Preprocessing

We performed auto contrast intensity normalization, which uses two scaling parameters for modifying the number of standard deviations on either side of the mean intensity. The algorithm uses 99.99% of maximum intensity as saturation intensity. A 2D slice-by-slice gaussian smoothing was performed to reduce noise in the stack.

Segmentation

Following the preprocessing, a 2D LoG filter was used to obtain particle segmentation.

Postprocessing

Based on pixel connectivity, objects equal to or smaller than 5 pixels are removed. Note that 5 pixels mean 5 connected voxels in 3D. This cutoff helped with removing a large number of false positive segmented objects.

LMNB1 segmentation technique

Preprocessing

We performed auto contrast intensity normalization, which uses two scaling parameters for modifying the number of standard deviations on either side of the mean intensity. The algorithm uses 99.99% of maximum intensity as saturation intensity. A 3D Gaussian smoothing was performed to reduce noise in the stack.

Segmentation

The middle frame of each stack was obtained from the intensity distribution using an Otsu threshold. A 2D Frangi Vesselness filter was then used to obtain nuclei outlines in the

middle frame. The middle frame is subsequently filled using hole-filling. Logical XOR was used on the frame obtained from Frangi vesselness and the one from filling holes. The resultant array is used to generate a 3D seed (as described on the Allen Cell and Structure Segmenter, https://github.com/AllenCell/aics-segmentation). Watershed was done to obtain each region inside the object. Logical XOR was then conducted on this segmentation and the same segmentation after a pixel dilation to obtain the object boundary.

Postprocessing

No postprocessing was performed.

Note: Because many objects were not segmented and because we were not able to close the segmentation of the nuclear envelope, we did not use this signal for calculating shape metrics.

Supplementary figures

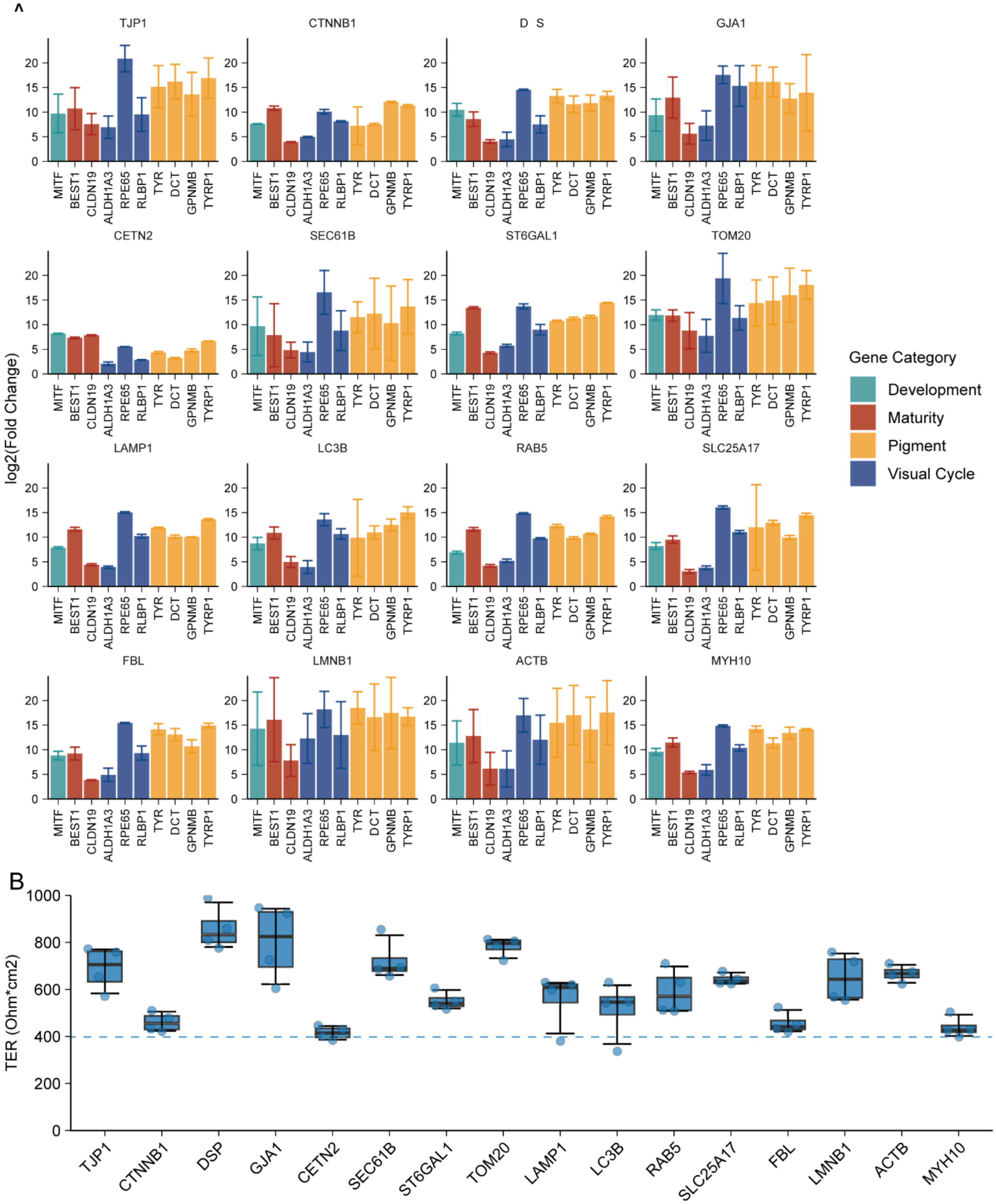

Figure S1: Validation of fully mature iPSC-RPE lines. (A) Differential expression of RPE signature genes in fully mature iPSC-RPE cells as compared to iPSC for each of the 16 tagged lines. (B) Boxplot showing the trans-epithelial electrical resistance of fully mature iPSC-RPE for each line. All lines have a TER of several hundred Ohms*cm^2, showing the good quality of the differentiations. The dashed line at 400 Ohms*cm^2 represents the quality control cutoff that was used for this study.

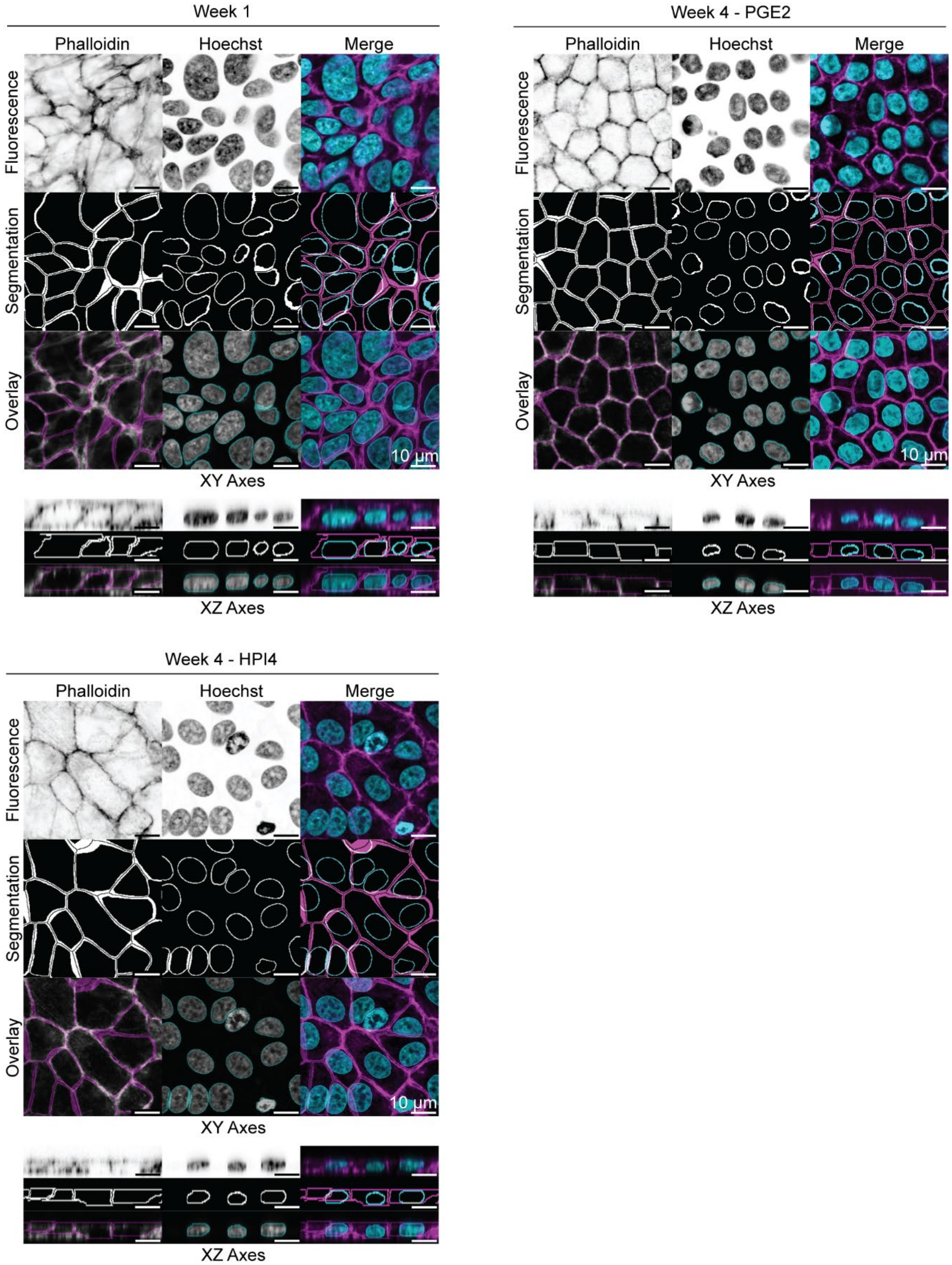

Figure S2: Representative images of phalloidin and Hoechst staining with their segmentation. Panels showing images of iPSC-RPE cells stained with phalloidin and

Hoechst and their respective AI-generated segmentations at week 1 (top left) and at week 4 with PGE2 (top right) or HPI4 (bottom left). Images in the top part of each panel were obtained from a representative X/Y plane of an image stack, while images in the bottom part of each panel show an XZ plane (magenta = phalloidin/cell border segmentation, cyan = Hoechst/nucleus border segmentation; scale bar = 10 µm).

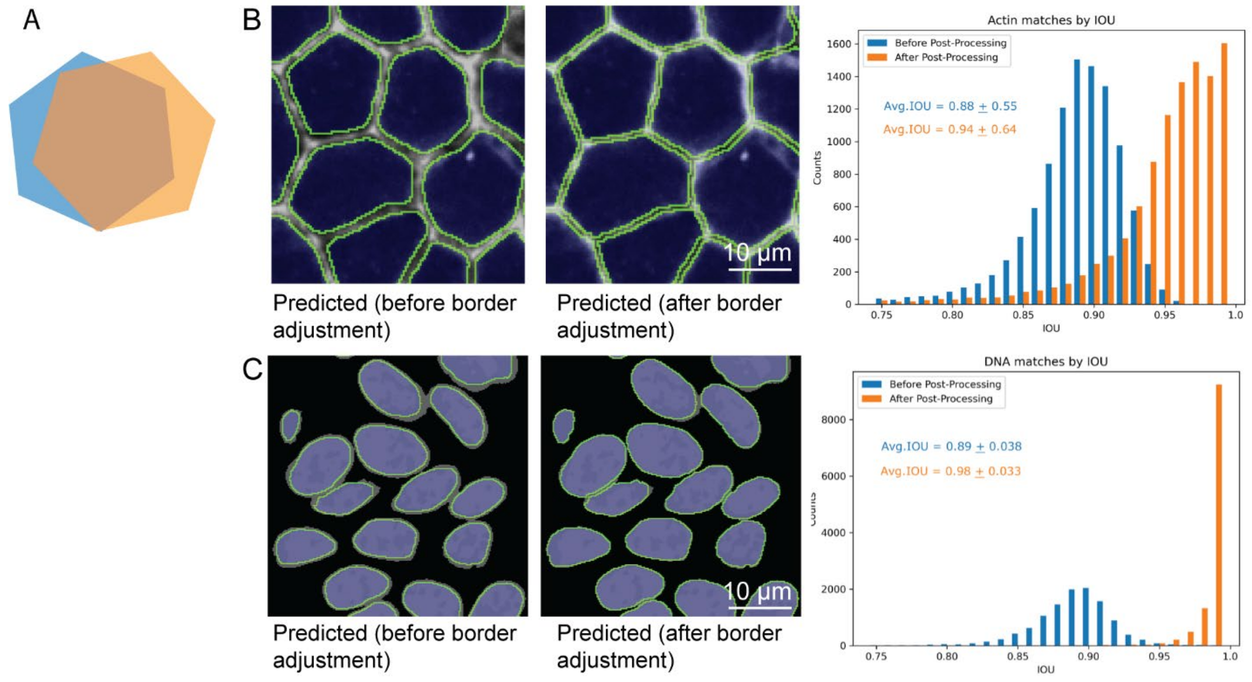

Figure S3: 3D assembly. (A) An IoU score was calculated to find the best match between 3D objects across different Z planes. If the score was above a threshold of 0.6, the objects were combined. (B) Representative images of cell border segmentation (blue masks with green outlines) from actin staining before and after dilation. Postprocessing dilation improved the IoU score, as shown in the bar graph. (C) Representative images of nuclei border segmentation (blue masks with green outlines) from Hoechst staining before and after dilation. Postprocessing dilation improved the IoU score, as shown in the bar graph. (Data from one stack at week 1 and one at week 2 that were not used for training).

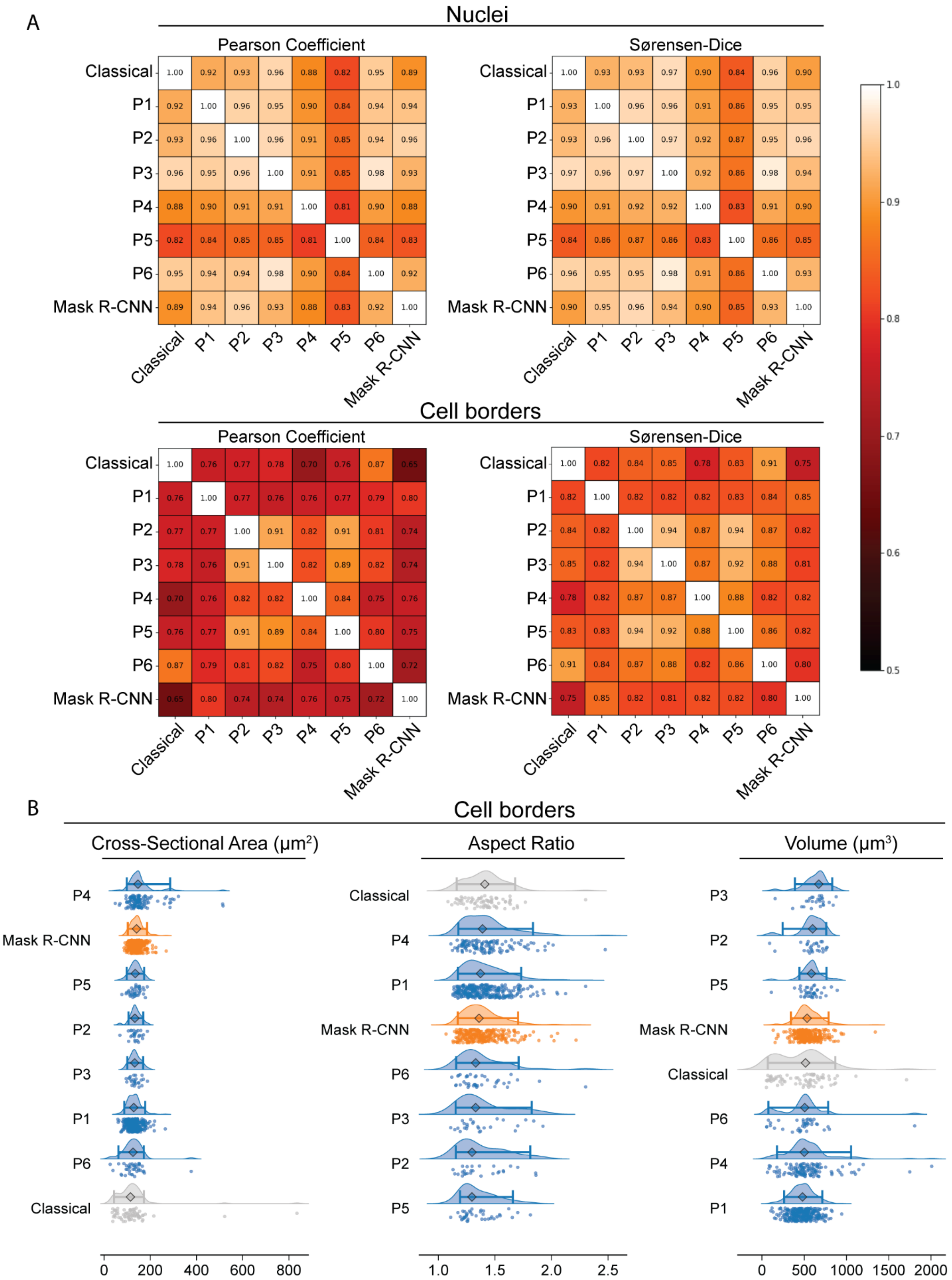
A
Nuclei
Pearson Coefficient
Classical, P1, P2, P3, P4, P5, P6, Mask R-CNN
Classical 1.00 0.92 0.93 0.96 0.88 0.82 0.95 0.89
P1 0.92 1.00 0.96 0.95 0.90 0.84 0.94 0.94
P2 0.93 0.96 1.00 0.96 0.91 0.85 0.94 0.96
P3 0.96 0.95 0.96 1.00 0.91 0.85 0.98 0.93
P4 0.88 0.90 0.91 0.91 1.00 0.81 0.90 0.88
P5 0.82 0.84 0.85 0.85 0.81 1.00 0.84 0.83
P6 0.95 0.94 0.94 0.98 0.90 0.84 1.00 0.92
Mask R-CNN 0.89 0.94 0.96 0.93 0.88 0.83 0.92 1.00
Sørensen-Dice
Classical 1.00 0.93 0.93 0.97 0.90 0.84 0.96 0.90
P1 0.93 1.00 0.96 0.96 0.91 0.86 0.95 0.95
P2 0.93 0.96 1.00 0.97 0.92 0.87 0.95 0.96
P3 0.97 0.96 0.97 1.00 0.92 0.86 0.98 0.94
P4 0.90 0.91 0.92 0.92 1.00 0.83 0.91 0.90
P5 0.84 0.86 0.87 0.86 0.83 1.00 0.86 0.85
P6 0.96 0.95 0.95 0.98 0.91 0.86 1.00 0.93
Mask R-CNN 0.90 0.95 0.96 0.94 0.90 0.85 0.93 1.00
Cell borders
Pearson Coefficient
Classical 1.00 0.76 0.77 0.78 0.70 0.76 0.87 0.65
P1 0.76 1.00 0.77 0.76 0.76 0.77 0.79 0.80
P2 0.77 0.77 1.00 0.91 0.82 0.91 0.81 0.74
P3 0.78 0.76 0.91 1.00 0.82 0.89 0.82 0.74
P4 0.70 0.76 0.82 0.82 1.00 0.84 0.75 0.76
P5 0.76 0.77 0.91 0.89 0.84 1.00 0.80 0.75
P6 0.87 0.79 0.81 0.82 0.75 0.80 1.00 0.72
Mask R-CNN 0.65 0.80 0.74 0.74 0.76 0.75 0.72 1.00
Sørensen-Dice
Classical 1.00 0.82 0.84 0.85 0.78 0.83 0.91 0.75
P1 0.82 1.00 0.82 0.82 0.82 0.83 0.84 0.85
P2 0.84 0.82 1.00 0.94 0.87 0.94 0.87 0.82
P3 0.85 0.82 0.94 1.00 0.87 0.92 0.88 0.81
P4 0.78 0.82 0.87 0.87 1.00 0.88 0.82 0.82
P5 0.83 0.83 0.94 0.92 0.88 1.00 0.86 0.82
P6 0.91 0.84 0.87 0.88 0.82 0.86 1.00 0.80
Mask R-CNN 0.75 0.85 0.82 0.81 0.82 0.82 0.80 1.00
1.0 0.9 0.8 0.7 0.6 0.5
B
Cell borders
Cross-Sectional Area (μm²)
P4, Mask R-CNN, P5, P2, P3, P1, P6, Classical
0 200 400 600 800
Aspect Ratio
Classical, P4, P1, Mask R-CNN, P6, P3, P2, P5
1.0 1.5 2.0 2.5
Volume (μm³)
P3, P2, P5, Mask R-CNN, Classical, P6, P4, P1
0 500 1000 1500 2000

Figure S4: Pearson correlation and Sorensen-Dice coefficient. (A) The four heatmaps show the degree of variation resulting from the generation of segmentation ground truth data by six different operators (P1-P6) for nuclei (top) and cell borders (bottom). The segmentation produced by classical algorithms and Mask R-CNN was also included as reference. (1.0 = high degree of correlation, 0.0 = low degree of correlation). (B) Measurements for cell cross-sectional area, aspect ratio, and volume performed on ground truth data are reported to show segmentation variability across six different operators, classical algorithms, and Mask R-CNN.

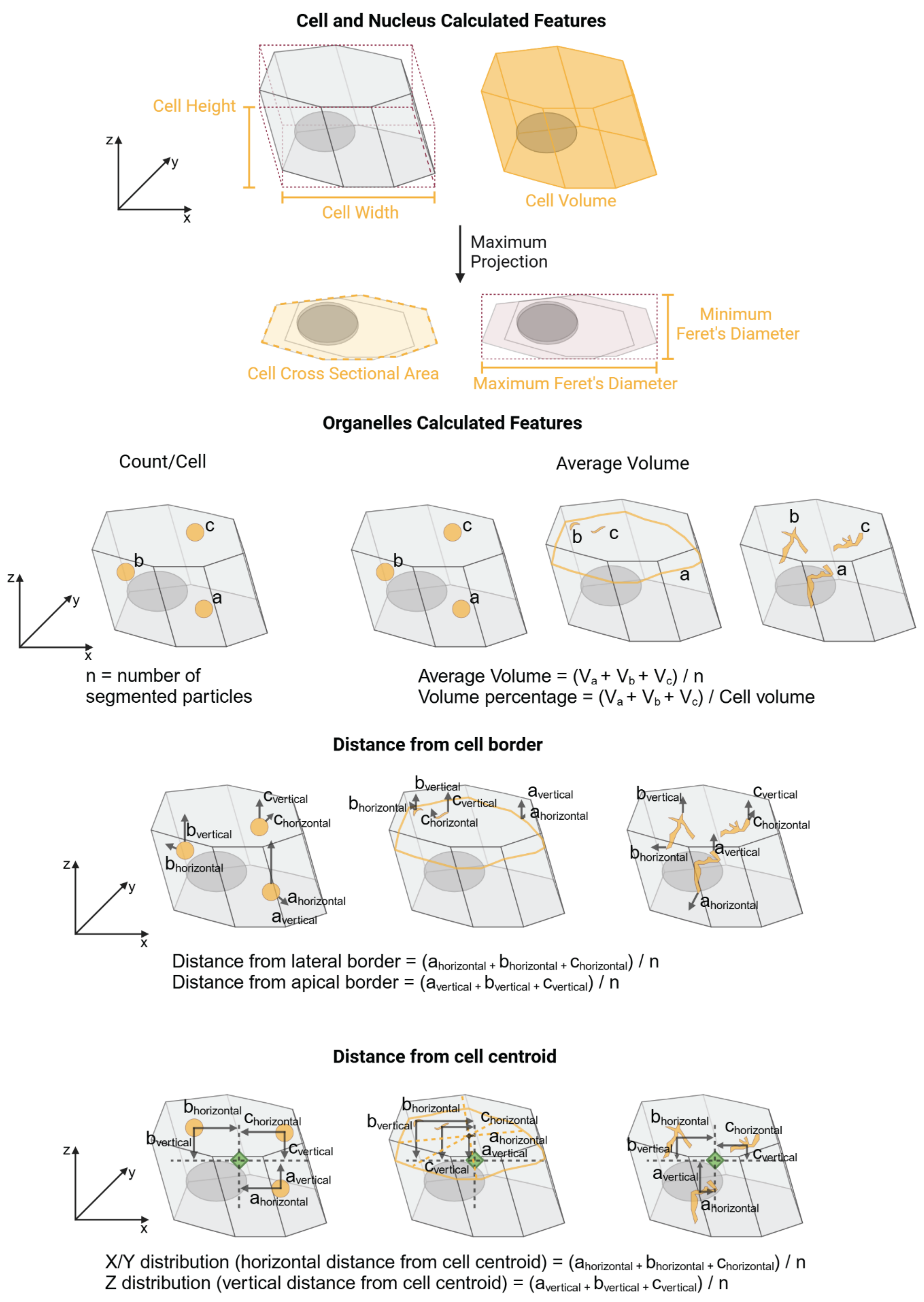
Cell and Nucleus Calculated Features
Cell Height
Cell Width
Cell Volume
Maximum Projection
Cell Cross Sectional Area
Minimum Feret's Diameter
Maximum Feret's Diameter
Organelles Calculated Features
Count/Cell
Average Volume
n = number of segmented particles
Average Volume = $(V_a + V_b + V_c) / n$
Volume percentage = $(V_a + V_b + V_c)$ / Cell volume
Distance from cell border
Distance from lateral border = $(a_{horizontal} + b_{horizontal} + c_{horizontal}) / n$
Distance from apical border = $(a_{vertical} + b_{vertical} + c_{vertical}) / n$
Distance from cell centroid
X/Y distribution (horizontal distance from cell centroid) = $(a_{horizontal} + b_{horizontal} + c_{horizontal}) / n$
Z distribution (vertical distance from cell centroid) = $(a_{vertical} + b_{vertical} + c_{vertical}) / n$

Figure S5: Visual representation of calculated parameters. Panel showing a visual representation of the calculated shape metrics on a mock 3D cell. The calculated object/parameter is displayed in yellow. The average distance of an organelle was calculated as 1) distance of the closest voxel of a segmented object from the cell borders or as 2) distance of the object centroid to the cell centroid. Both approaches are shown in the panel using three very different organelle types, as the interpretation of the organelle location may vary depending on its shape.

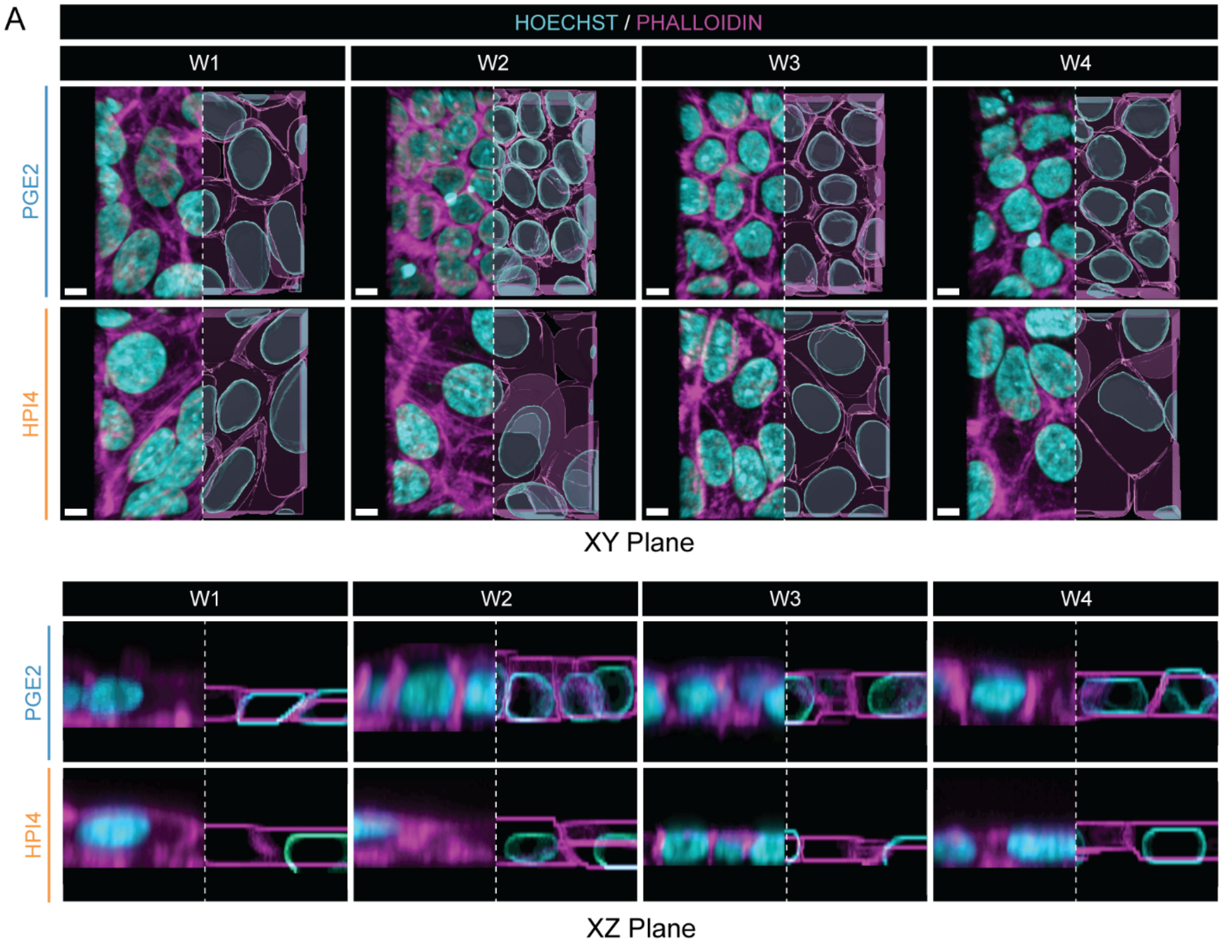

B

Cell

Cross-Sectional Area

	W1	W2	W3	W4
W1		—	***	***
W2	***		***	***
W3	***	***		***
W4	***	***	*	

Aspect Ratio

	W1	W2	W3	W4
W1		—	***	***
W2	***		***	***
W3	***	**		***
W4	***	***	—	

Volume

	W1	W2	W3	W4
W1		**	*	***
W2	***		***	***
W3	***	***		***
W4	***	***	—	

Nucleus

Cross-Sectional Area

	W1	W2	W3	W4
W1		***	***	*
W2	***		***	***
W3	***	—		—
W4	***	***	***	

Aspect Ratio

	W1	W2	W3	W4
W1		—	***	***
W2	***		***	***
W3	***	—		—
W4	***	—	—	

Volume

	W1	W2	W3	W4
W1		***	***	***
W2	***		***	***
W3	***	***		—
W4	***	***	**	

Figure S6: Cell and nucleus morphometric changes and statistics. (A) Representative fluorescence images of phalloidin and Hoechst-stained iRPE cells at

different timepoints and treatments, and corresponding cell and nucleus border segmentation (magenta = phalloidin/cell border segmentation, cyan = Hoechst/nucleus border segmentation; scale bar = 10 μm). (B) Results of Tukey's HSD pairwise comparison for cells and nuclei for three shape metrics: cross-sectional area, aspect ratio, and volume (N = 30; ***P < 0.005, **P < 0.01, *P < 0.05).

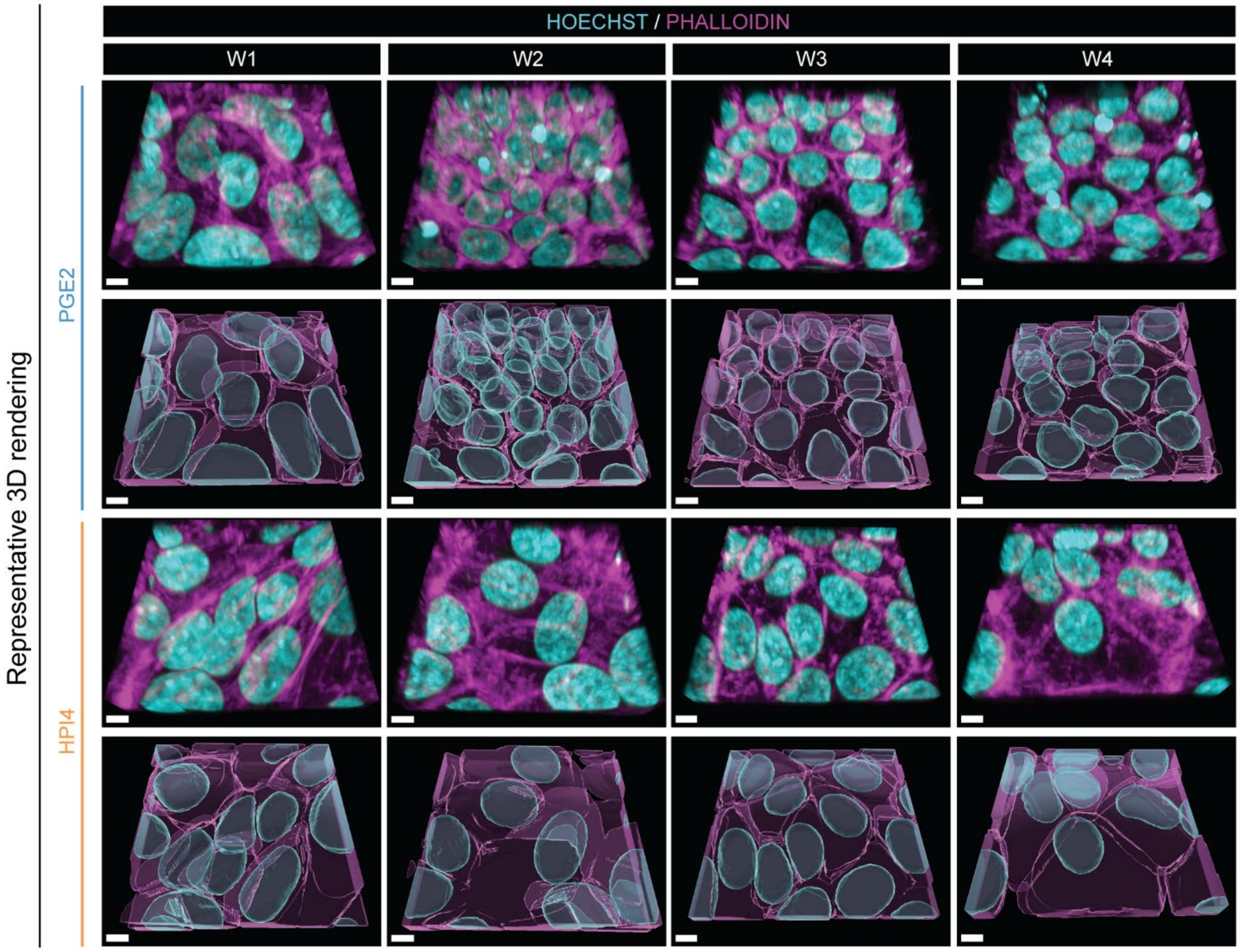

Figure S7: Side-by-side view of cell and nucleus morphometric changes. Representative fluorescence images of phalloidin and Hoechst-stained iRPE cells at different timepoints and treatments, and corresponding cell and nucleus border segmentation (magenta = phalloidin/cell border segmentation, cyan = Hoechst/nucleus border segmentation; scale bar = 10 μm).

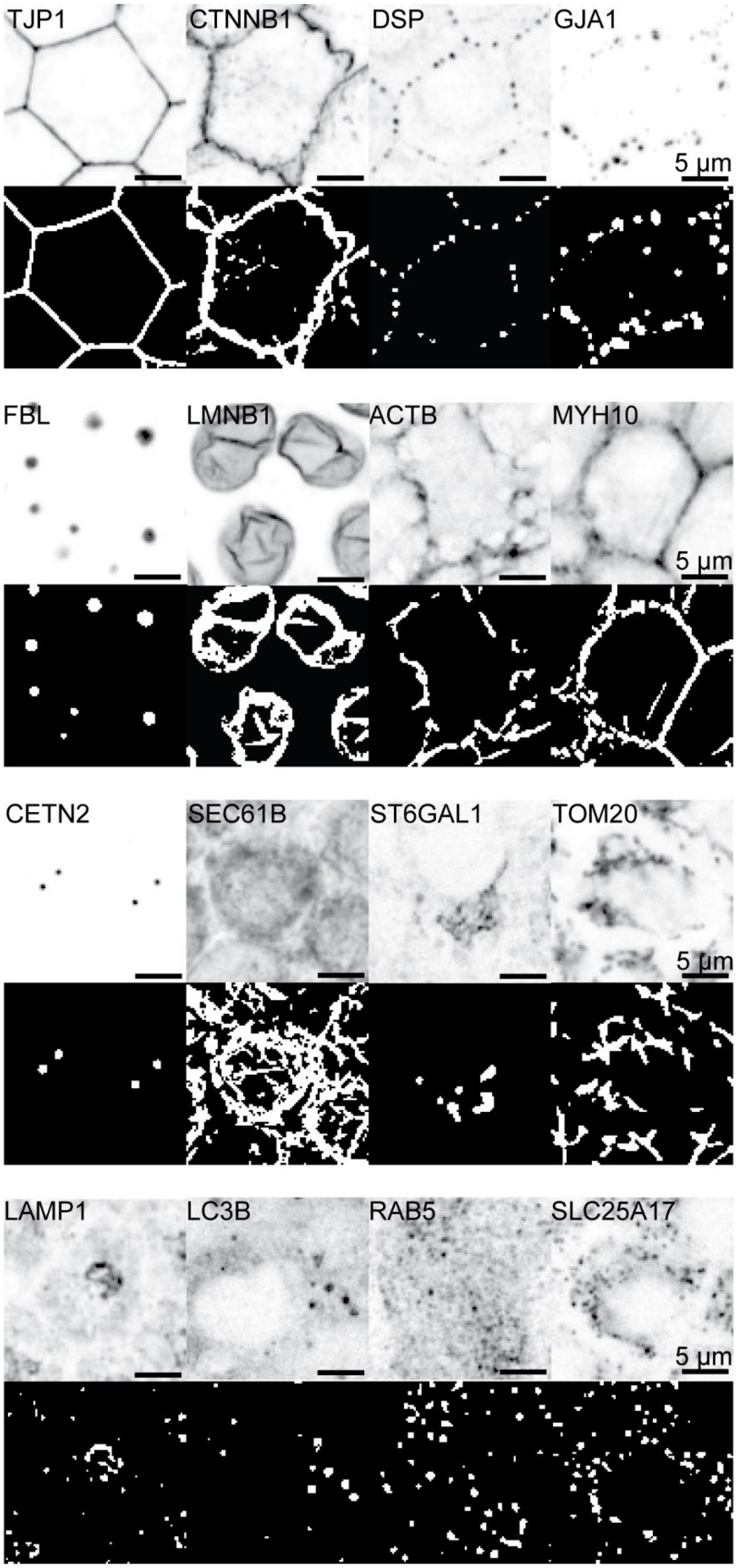
TJP1
CTNNB1
DSP
GJA1
5 µm
FBL
LMNB1
ACTB
MYH10
5 µm
CETN2
SEC61B
ST6GAL1
TOM20
5 µm
LAMP1
LC3B
RAB5
SLC25A17
5 µm

Figure S8: Representative fluorescence images of intracellular structures and their corresponding segmentation. Panel showing single X/Y planes of the proteins of interest and their corresponding binary segmentation (scale bars = 5 μm).

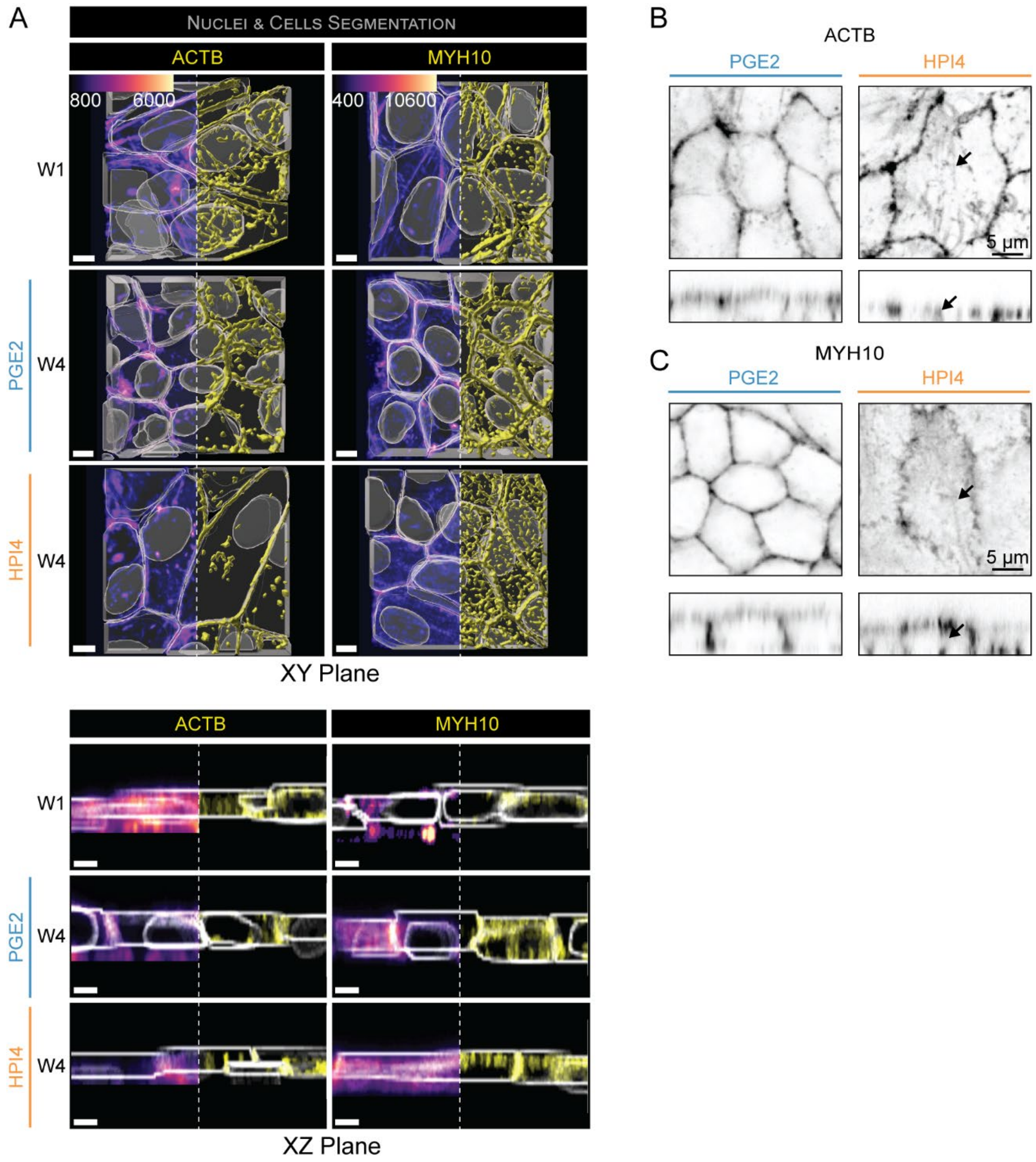

Figure S9: Top-down and side views showing phenotypic changes of cytoskeletal proteins. (A) Representative 3D renderings of iRPE cells expressing GFP-tagged ACTB (actin filaments) and MYH10 (actomyosin bundles), shown from a top-down view in the top panel and from a side view (5 µm average intensity projection) in the bottom panel. Raw intensities of the GFP-tagged proteins are shown on the left side of each image (magma color palette), while 3D reconstructions of the signal are shown on the right side

(yellow). Both raw intensities and reconstructions are overlayed onto the cell and nucleus border segmentations (grey) (scale bar = 10 µm). (B-C) Raw intensity images of GFP-tagged ACTB and MYH10 on the XY and XZ planes show organization of the actomyosin cytoskeleton along cell borders (lateral, apical, and basal) in PGE2-treated cells at week 4, as compared to disorganized filaments visible in the HPI4 condition (scale bar = 5 µm).

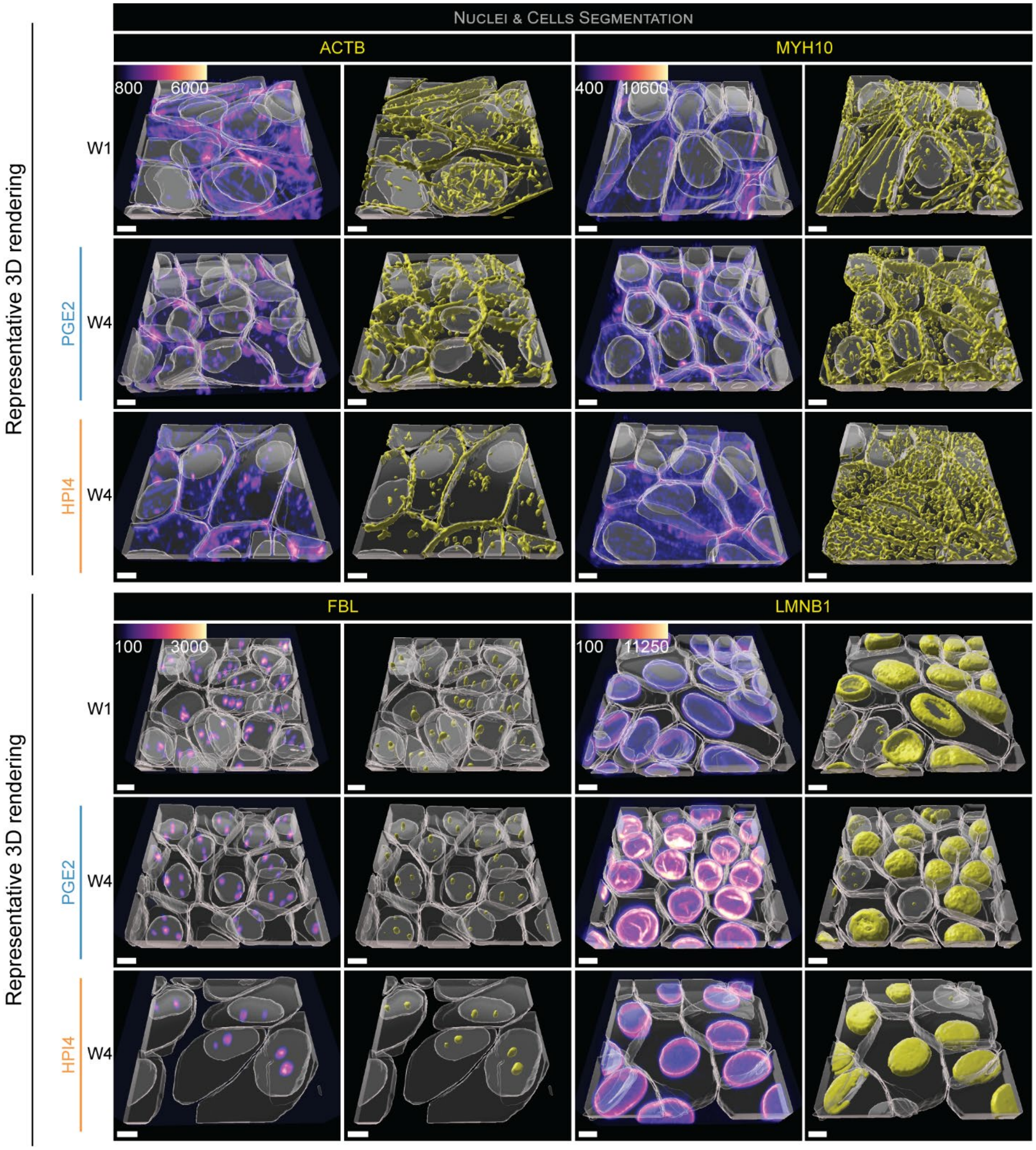

Figure S10: Side-by-side view of cytoskeletal and nuclear proteins. Representative 3D renderings of iRPE cells expressing GFP-tagged ACTB (actin filaments), MYH10 (actomyosin bundles), FBL (nucleolus), and LMNB1 (nuclear envelope) showing raw intensities (left, magma color palette) side-by-side with their corresponding segmentation

(right, yellow). Both raw intensities and reconstructions are overlayed onto the cell and nucleus border segmentations (grey) (scale bar = 10 μm).

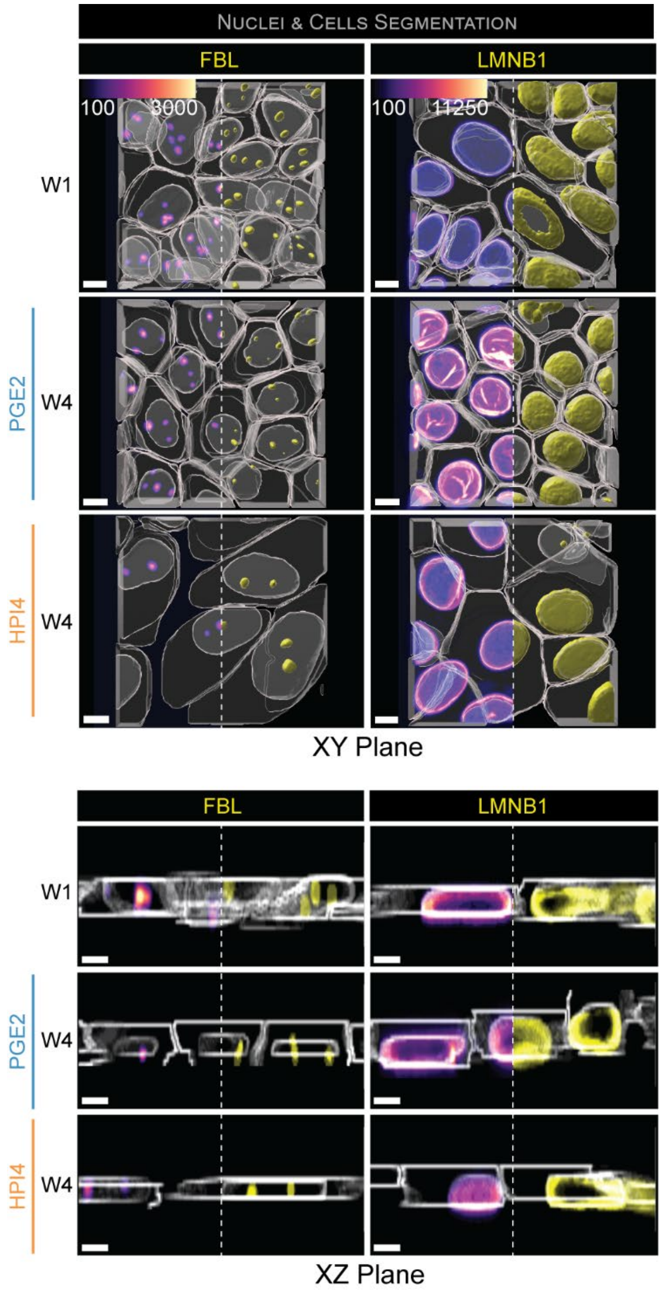

Figure S11: Top-down and side views showing phenotypic changes of nuclear proteins. Representative 3D renderings of iRPE cells expressing fluorescently tagged FBL (nucleolus) and LMNB1 (nuclear envelope), shown from a top-down view in the top panel and from a side view (5 µm average intensity projection) in the bottom panel. Raw intensities of the GFP-tagged proteins are shown on the left side of each image (magma color palette), while 3D reconstructions of the signal are shown on the right side (yellow).

Both raw intensities and reconstructions are overlayed onto the cell and nucleus border segmentations (grey) (scale bar = 10 μm).

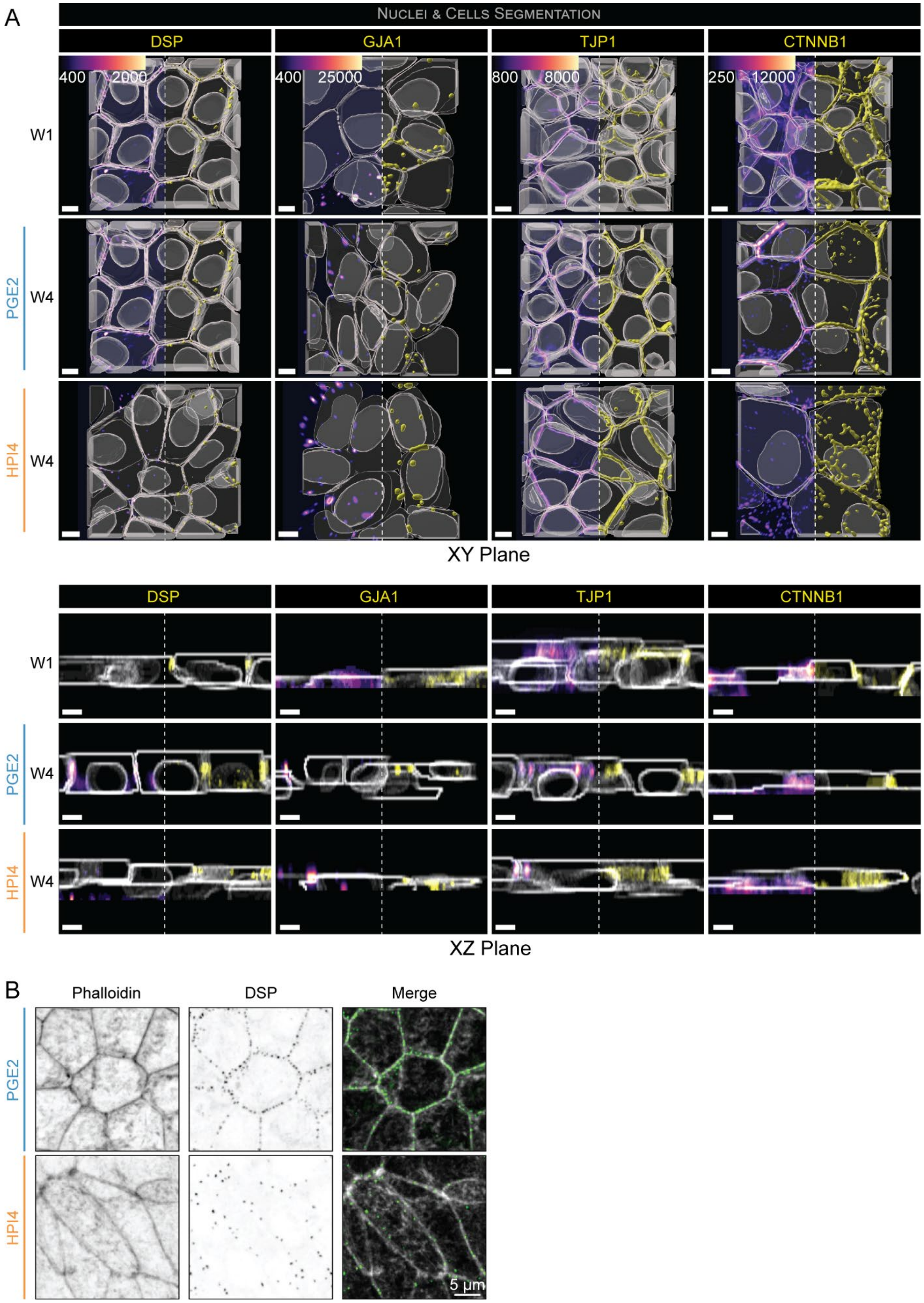
A
Nuclei & Cells Segmentation
DSP
GJA1
TJP1
CTNNB1
400
2000
400
25000
800
8000
250
12000
W1
PGE2
W4
HPI4
W4
XY Plane
DSP
GJA1
TJP1
CTNNB1
W1
PGE2
W4
HPI4
W4
XZ Plane
B
Phalloidin
DSP
Merge
PGE2
HPI4
5 µm

396

Figure S12: Top-down and side views showing phenotypic changes of junctional proteins. (A) Representative 3D renderings of iRPE cells expressing fluorescently tagged TJP1 (tight junctions), CTNNB1 (adherens junctions), DSP (desmosomes), and GJA1 (gap junctions), shown from a top-down view in the top panel and from a side view (5 μm average intensity projection) in the bottom panel. Raw intensities of the GFP-tagged proteins are shown on the left side of each image (magma color palette), while 3D reconstructions of the signal are shown on the right side (yellow). Both raw intensities and reconstructions are overlayed onto the cell and nucleus border segmentations (grey) (scale bar = 10 μm). (B) Representative images of GFP-tagged DSP comparing PGE2 and HPI4 conditions at week 4 (scale bar = 5 μm).

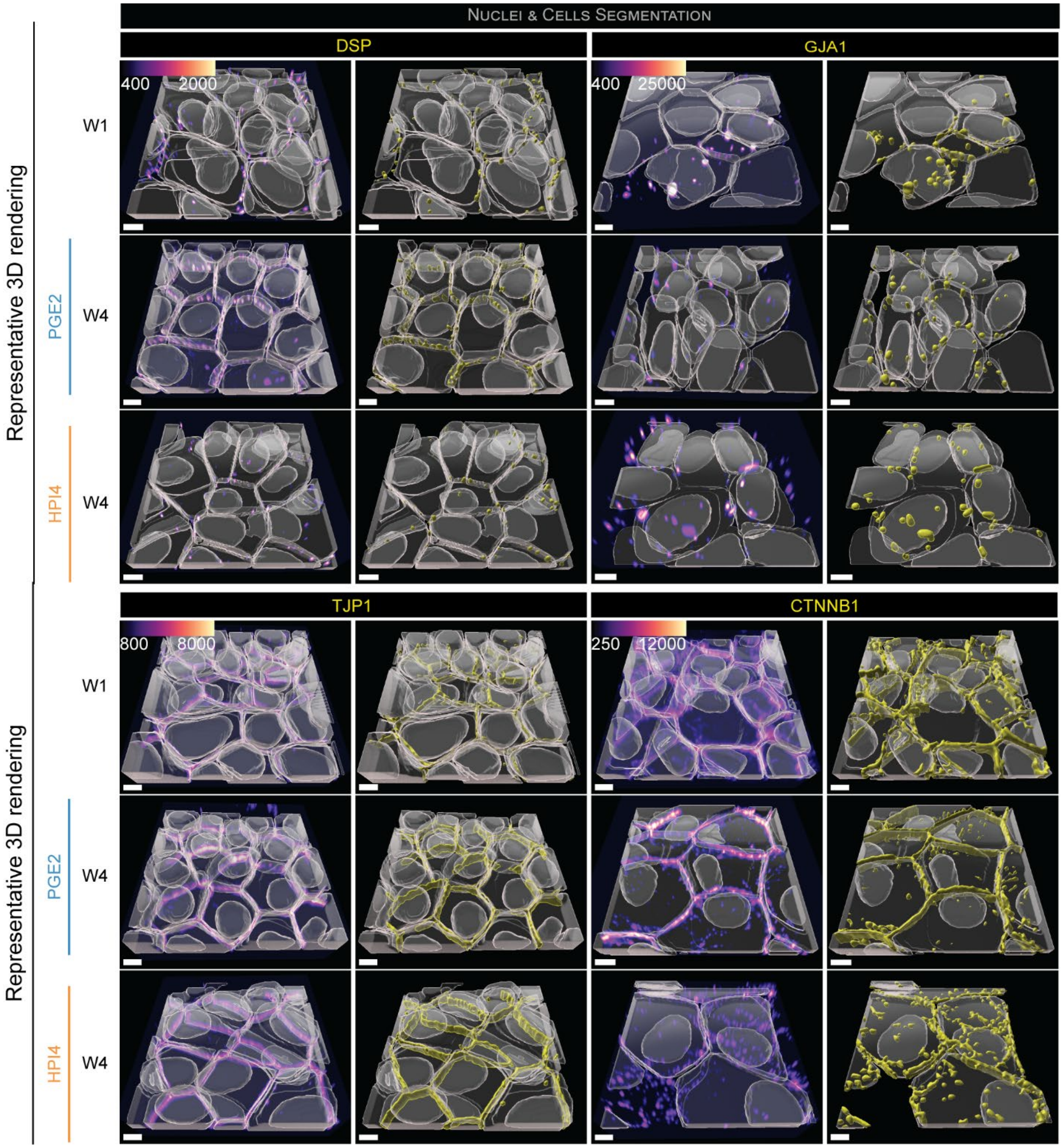

Figure S13: Side-by-side view of junctional proteins. Representative 3D renderings of iRPE cells expressing fluorescently tagged TJP1 (tight junctions), CTNNB1 (adherens junctions), DSP (desmosomes), and GJA1 (gap junctions) showing raw intensities (left, magma color palette) side-by-side with their corresponding segmentation (right, yellow).

Both raw intensities and reconstructions are overlayed onto the cell and nucleus border segmentations (grey) (scale bar = 10 µm).

A DSP - Avg. Volume

	W1	W2	W3	W4
W1		—	—	**
W2	***		—	**
W3	***	**		*
W4	***	**	—	

B GJA1 - Avg. Volume

	W1	W2	W3	W4
W1		—	—	—
W2	—		—	—
W3	—	—		—
W4	—	—	—	

C DSP - Count/cell

	W1	W2	W3	W4
W1		—	—	—
W2	**		—	—
W3	***	—		—
W4	***	***	**	

D GJA1 - Count/cell

	W1	W2	W3	W4
W1		—	—	—
W2	***		—	—
W3	***	—		—
W4	***	—	—	

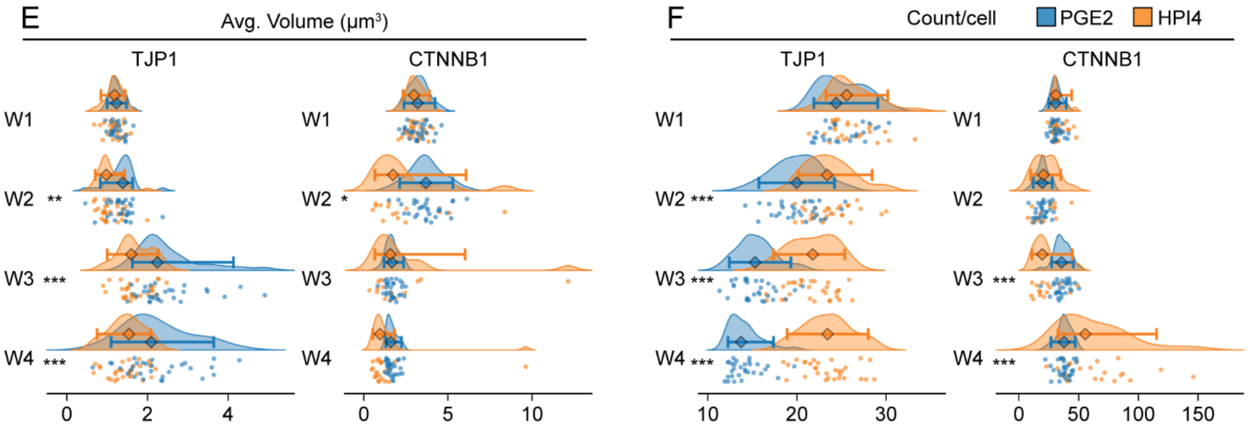

E TJP1

	W1	W2	W3	W4
W1		—	***	*
W2	—		***	***
W3	***	***		—
W4	***	***	—	

E CTNNB1

	W1	W2	W3	W4
W1		—	—	*
W2	*		—	—
W3	***	***		—
W4	***	***	—	

F TJP1

	W1	W2	W3	W4
W1		**	***	**
W2	***		*	—
W3	***	***		—
W4	***	***	—	

F CTNNB1

	W1	W2	W3	W4
W1		—	—	***
W2	***		—	***
W3	**	***		***
W4	***	***	—	

Figure S14: Shape metrics and multiple comparisons for cell junctions. (A-D) Results of Tukey's HSD pairwise comparison related to the graphs of **Figure 5D-G**. (N = 30; ***P < 0.005, **P < 0.01, *P < 0.05). (E) Raincloud plots indicating the changes over time and between treatments of TJP1 and CTNNB1 volume and tables showing the results of Tukey's HSD pairwise comparison. (F) Raincloud plots indicating the changes over time and between treatments of TJP1 and CTNNB1 count/cell and tables showing the results of Tukey's HSD pairwise comparison. (The points in the raincloud plots indicate the average values per cell for each FOV; the diamonds represent the median and the error bars indicate the 5th and 95th percentiles. N = 30; ***P < 0.005, **P < 0.01, *P < 0.05).

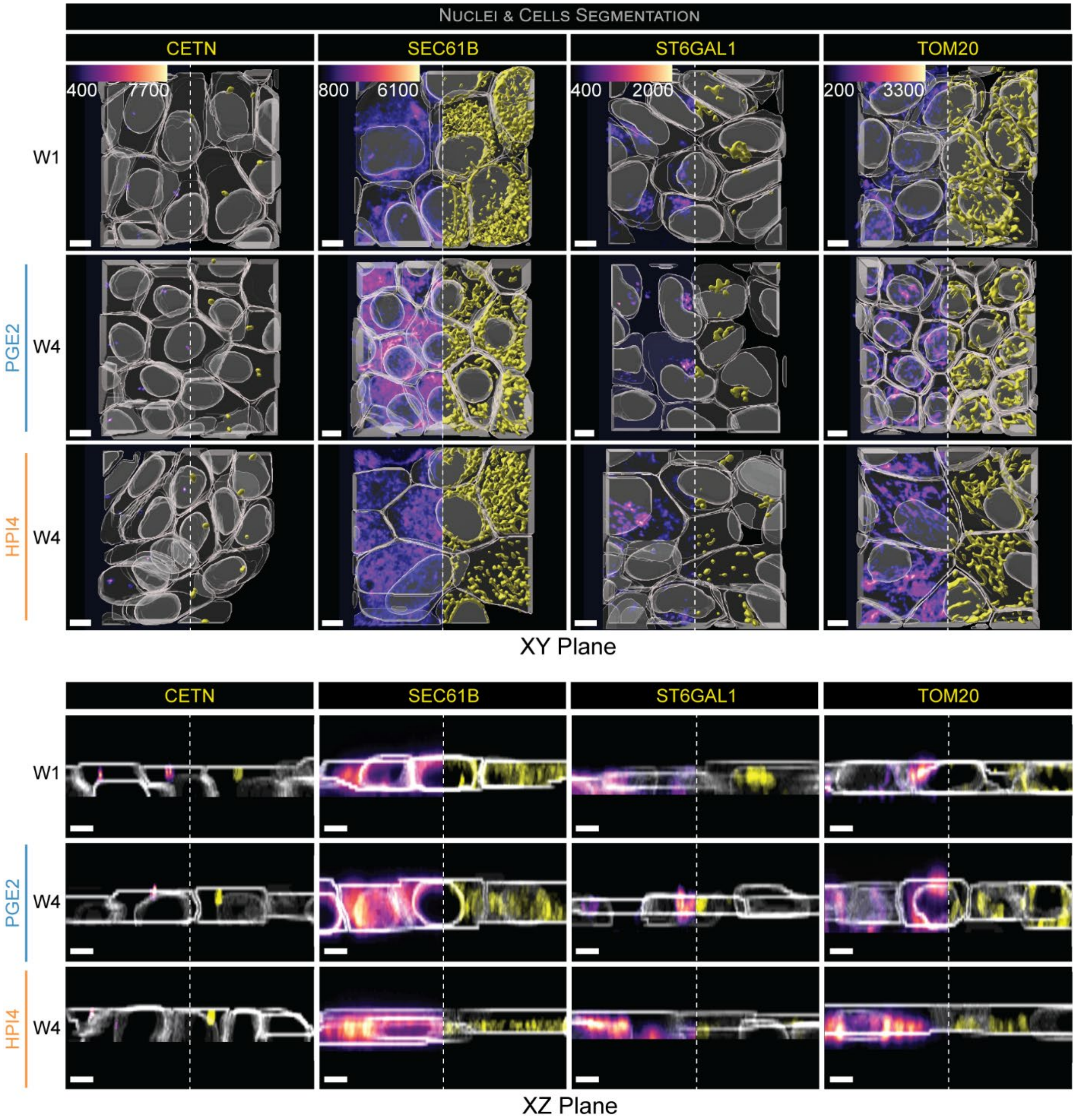

Figure S15: Top-down and side views showing phenotypic changes of centrioles and larger organelles. Representative 3D renderings of iRPE cells expressing fluorescently tagged CETN2 (centrioles), SEC61B (ER), ST6GAL1 (Golgi apparatus), and TOM20 (mitochondria), shown from a top-down view in the top panel and from a side view (5 µm average intensity projection) in the bottom panel. Raw intensities of the GFP-tagged proteins are shown on the left side of each image (magma color palette), while 3D

reconstructions of the signal are shown on the right side (yellow). Both raw intensities and reconstructions are overlayed onto the cell and nucleus border segmentations (grey) (scale bar = 10 μm).

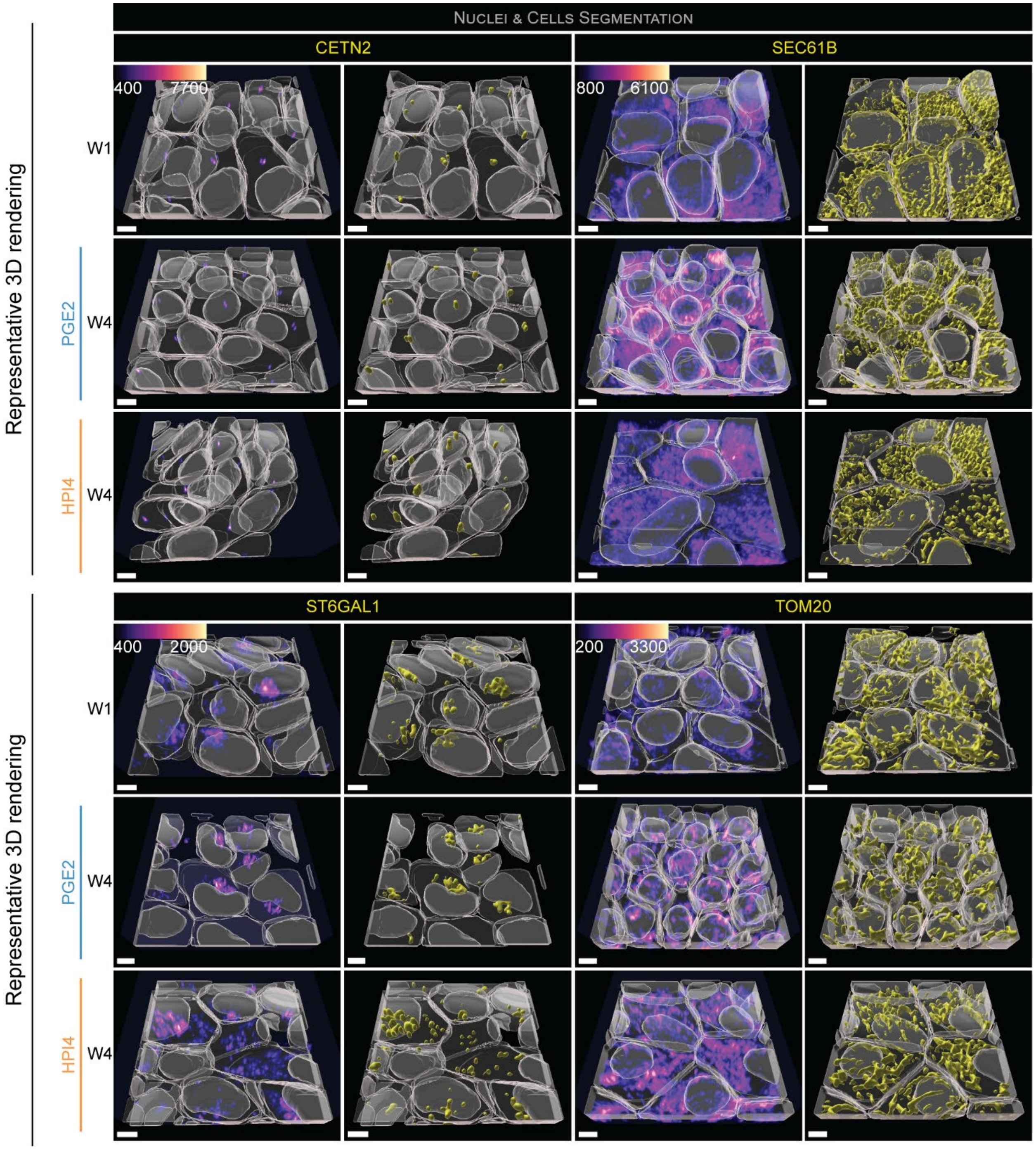

Figure S16: Side-by-side view of centrioles and larger organelles. Representative 3D renderings of iRPE cells expressing fluorescently tagged CETN2 (centrioles), SEC61B (ER), ST6GAL1 (Golgi apparatus), and TOM20 (mitochondria) showing raw intensities (left, magma color palette) side-by-side with their corresponding segmentation

(right, yellow). Both raw intensities and reconstructions are overlayed onto the cell and nucleus border segmentations (grey) (scale bar = 10 μm).

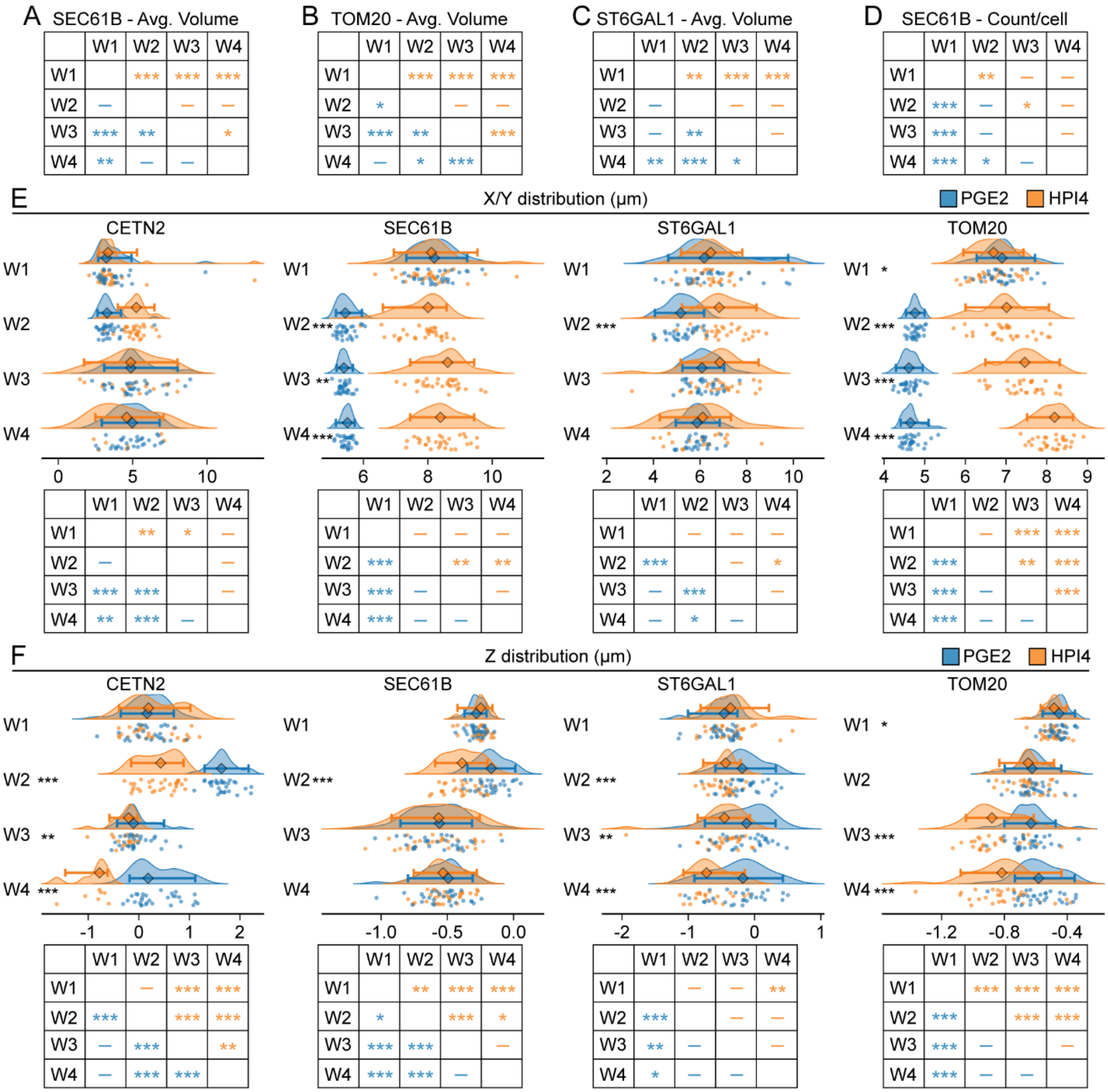

Figure S17: Shape metrics and multiple comparisons for centrioles and large organelles. (A-D) Results of Tukey's HSD pairwise comparison related to the graphs of **Figure 6D-G**. (N = 30; ***P < 0.005, **P < 0.01, *P < 0.05). (E) Raincloud plots showing the changes in organelle location on the X/Y plane. Distances from organelles and cell centroids were used. The tables indicate Tukey's HSD pairwise comparison. (F)

Raincloud plots showing the changes in organelle location on the Z axis. Distances from organelles and cell centroids were used. The tables indicate Tukey's HSD pairwise comparison. (The points in the raincloud plots indicate the average values per cell for each FOV; the diamonds represent the median and the error bars indicate the 5th and 95th percentiles. N = 30; ***P < 0.005, **P < 0.01, *P < 0.05).

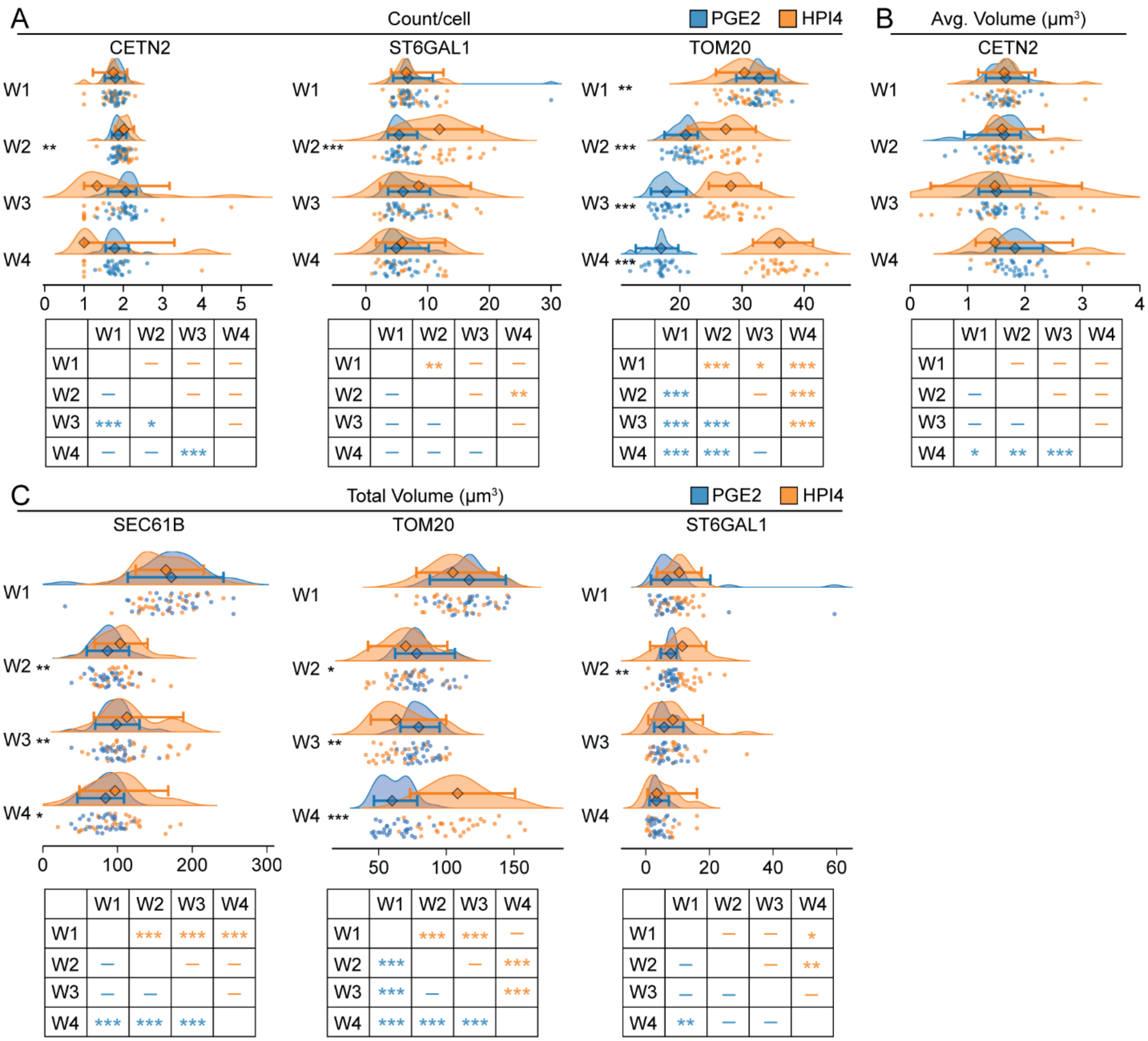

Figure S18: Shape metrics and multiple comparisons for centrioles and large organelles. (A) Raincloud plots showing the number of objects per cell and tables indicating Tukey's HSD pairwise comparison. (B) Raincloud plots showing centrioles average volume and its relative table of Tukey's HSD pairwise comparison. (C) Raincloud plots showing the total ER, mitochondria, and Golgi volume per cell and tables indicating Tukey's HSD pairwise comparison. (The points in the raincloud plots indicate the average values per cell for each FOV; the diamonds represent the median and the error bars indicate the 5th and 95th percentiles. N = 30; ***P < 0.005, **P < 0.01, *P < 0.05).

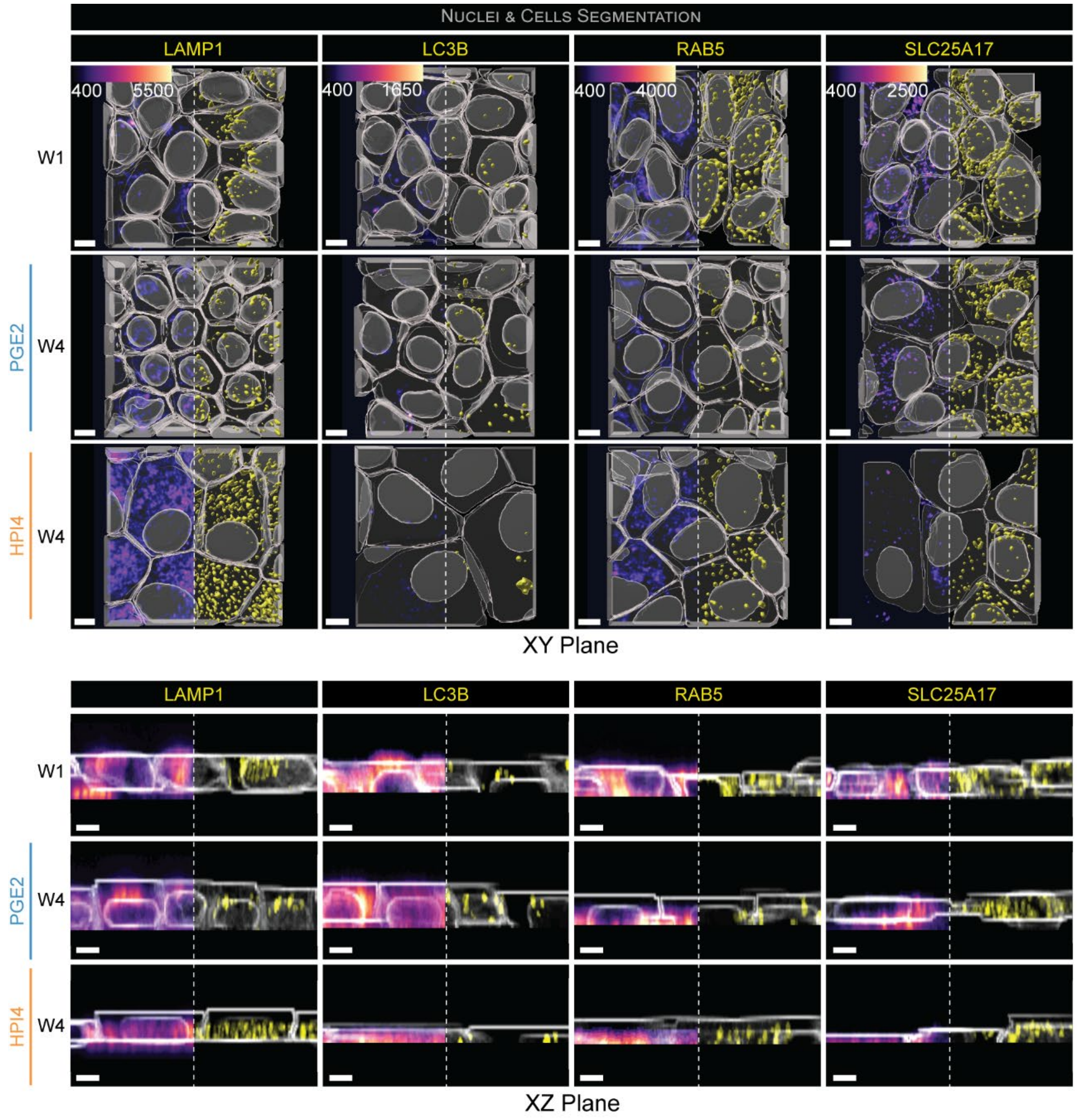

Figure S19: Top-down and side views showing phenotypic changes of vesicular organelles. Representative 3D renderings of iRPE cells expressing fluorescently tagged LAMP1 (lysosomes), LC3B (autophagosomes), RAB5 (endosomes), and SLC25A17 (peroxisomes), shown from a top-down view in the top panel and from a side view (5 µm average intensity projection) in the bottom panel. Raw intensities of the GFP-tagged proteins are shown on the left side of each image (magma color palette), while 3D reconstructions of the signal are shown on the right side (yellow). Both raw intensities and

reconstructions are overlayed onto the cell and nucleus border segmentations (grey) (scale bar = 10 μm).

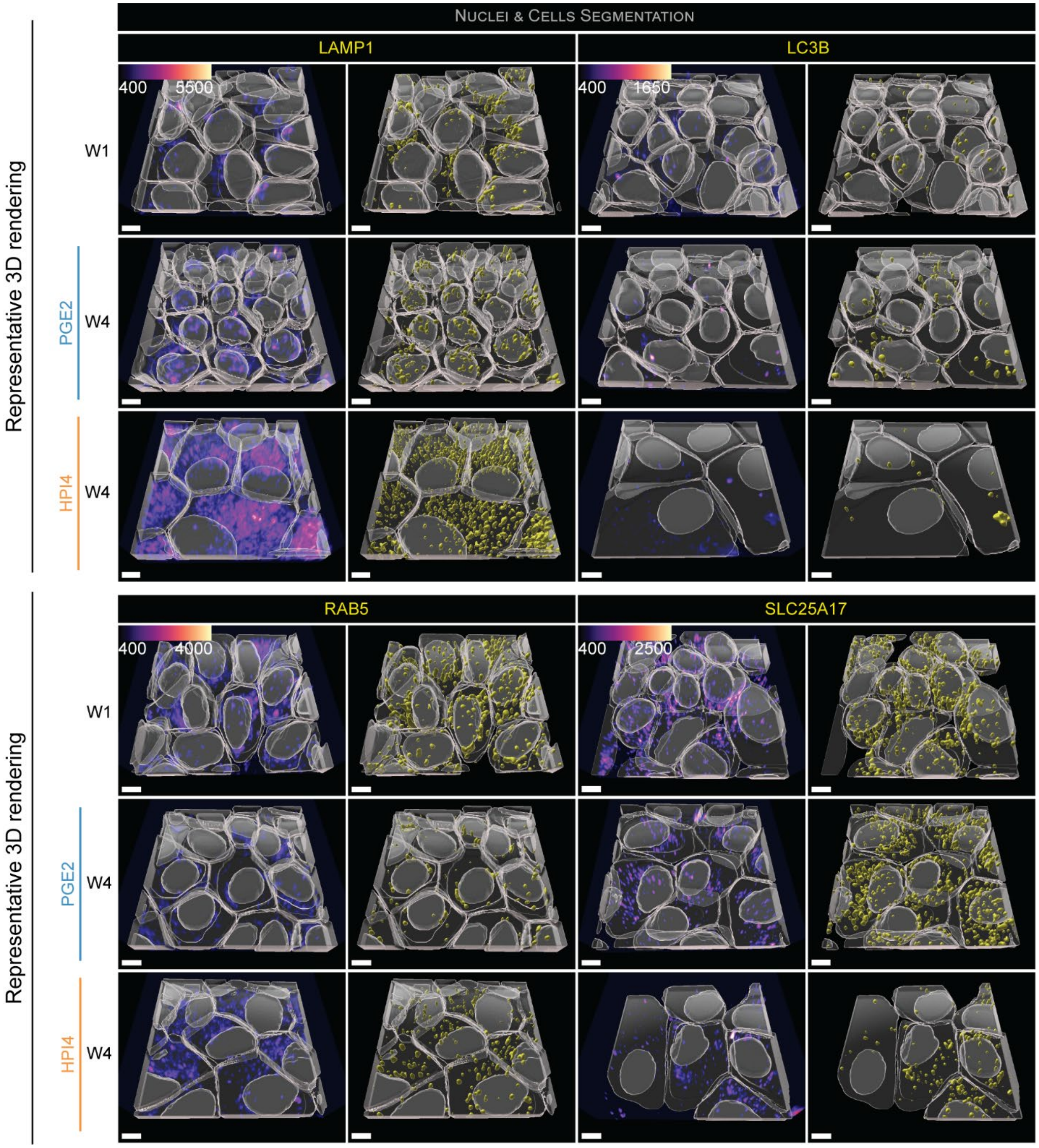

Figure S20: Side-by-side view of vesicular organelles. Representative 3D renderings of iRPE cells expressing fluorescently tagged LAMP1 (lysosomes), LC3B (autophagosomes), RAB5 (endosomes), and SLC25A17 (peroxisomes) showing raw intensities (left, magma color palette) side-by-side with their corresponding segmentation

(right, yellow). Both raw intensities and reconstructions are overlayed onto the cell and nucleus border segmentations (grey) (scale bar = 10 µm).

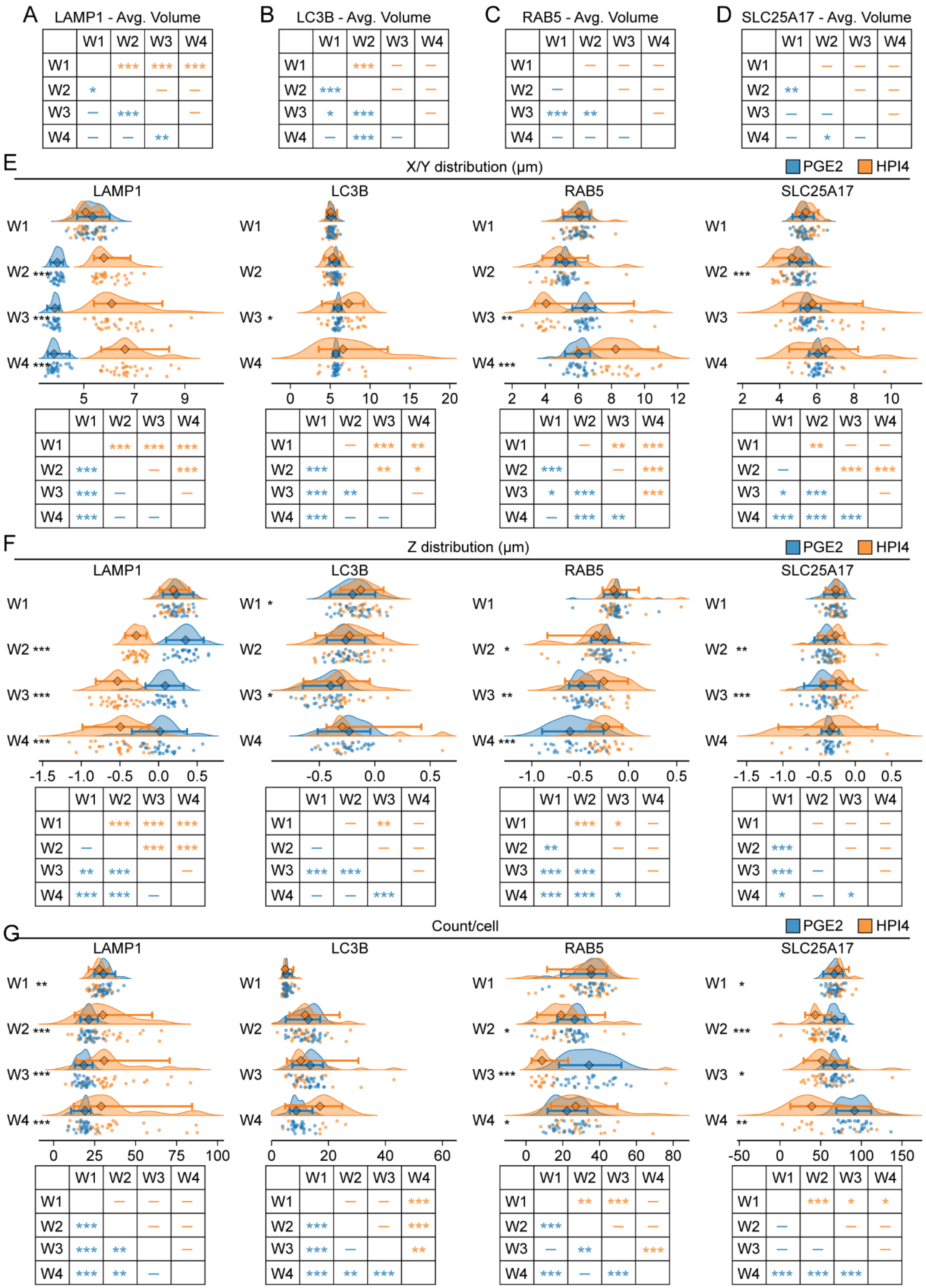

A LAMP1 - Avg. Volume

	W1	W2	W3	W4
W1		***	***	***
W2	*		—	—
W3	—	***		—
W4	—	—	**	

B LC3B - Avg. Volume

	W1	W2	W3	W4
W1		***	—	—
W2	***		—	—
W3	*	***		—
W4	—	***	—	

C RAB5 - Avg. Volume

	W1	W2	W3	W4
W1		—	—	—
W2	—		—	—
W3	***	**		—
W4	—	—	—	

D SLC25A17 - Avg. Volume

	W1	W2	W3	W4
W1		—	—	—
W2	**		—	—
W3	—	—		—
W4	—	*	—	

E X/Y distribution (µm)

LAMP1

	W1	W2	W3	W4
W1		***	***	***
W2	***		—	***
W3	***	—		—
W4	***	—	—	

LC3B

	W1	W2	W3	W4
W1		—	***	**
W2	***		**	*
W3	***	**		—
W4	***	—	—	

RAB5

	W1	W2	W3	W4
W1		—	**	***
W2	***		—	***
W3	*	***		***
W4	—	***	**	

SLC25A17

	W1	W2	W3	W4
W1		**	—	—
W2	—		***	***
W3	*	***		—
W4	***	***	***	

F Z distribution (µm)

LAMP1

	W1	W2	W3	W4
W1		***	***	***
W2	—		***	***
W3	**	***		—
W4	***	***	—	

LC3B

	W1	W2	W3	W4
W1		—	**	—
W2	—		—	—
W3	***	***		—
W4	—	—	***	

RAB5

	W1	W2	W3	W4
W1		***	*	—
W2	**		—	—
W3	***	***		—
W4	***	***	*	

SLC25A17

	W1	W2	W3	W4
W1		—	—	—
W2	***		—	—
W3	***	—		—
W4	*	—	*	

G Count/cell

LAMP1

	W1	W2	W3	W4
W1		—	—	—
W2	***		—	—
W3	***	**		—
W4	***	**	—	

LC3B

	W1	W2	W3	W4
W1		—	—	***
W2	***		—	***
W3	***	—		**
W4	***	**	***	

RAB5

	W1	W2	W3	W4
W1		**	***	—
W2	***		—	—
W3	—	**		***
W4	***	—	***	

SLC25A17

	W1	W2	W3	W4
W1		***	*	*
W2	—		—	—
W3	—	—		—
W4	***	***	***	

Figure S21: Shape metrics and multiple comparisons for vesicular organelles. (A-D) Results of Tukey's HSD pairwise comparison related to the graphs of **Figure 7D-G**. ($N = 30$; ***$P < 0.005$, **$P < 0.01$, *$P < 0.05$). (E) Raincloud plots showing the changes in organelle location on the X/Y plane. Distances from organelles and cell centroids were used. The tables indicate Tukey's HSD pairwise comparison. (F) Raincloud plots showing the changes in organelle location on the Z axis. Distances from organelles and cell centroids were used. The tables indicate Tukey's HSD pairwise comparison. (G) Raincloud plots showing the number of objects per cell and tables indicating Tukey's HSD pairwise comparison. (The points in the raincloud plots indicate the average values per cell for each FOV; the diamonds represent the median and the error bars indicate the 5th and 95th percentiles. $N = 30$; ***$P < 0.005$, **$P < 0.01$, *$P < 0.05$).

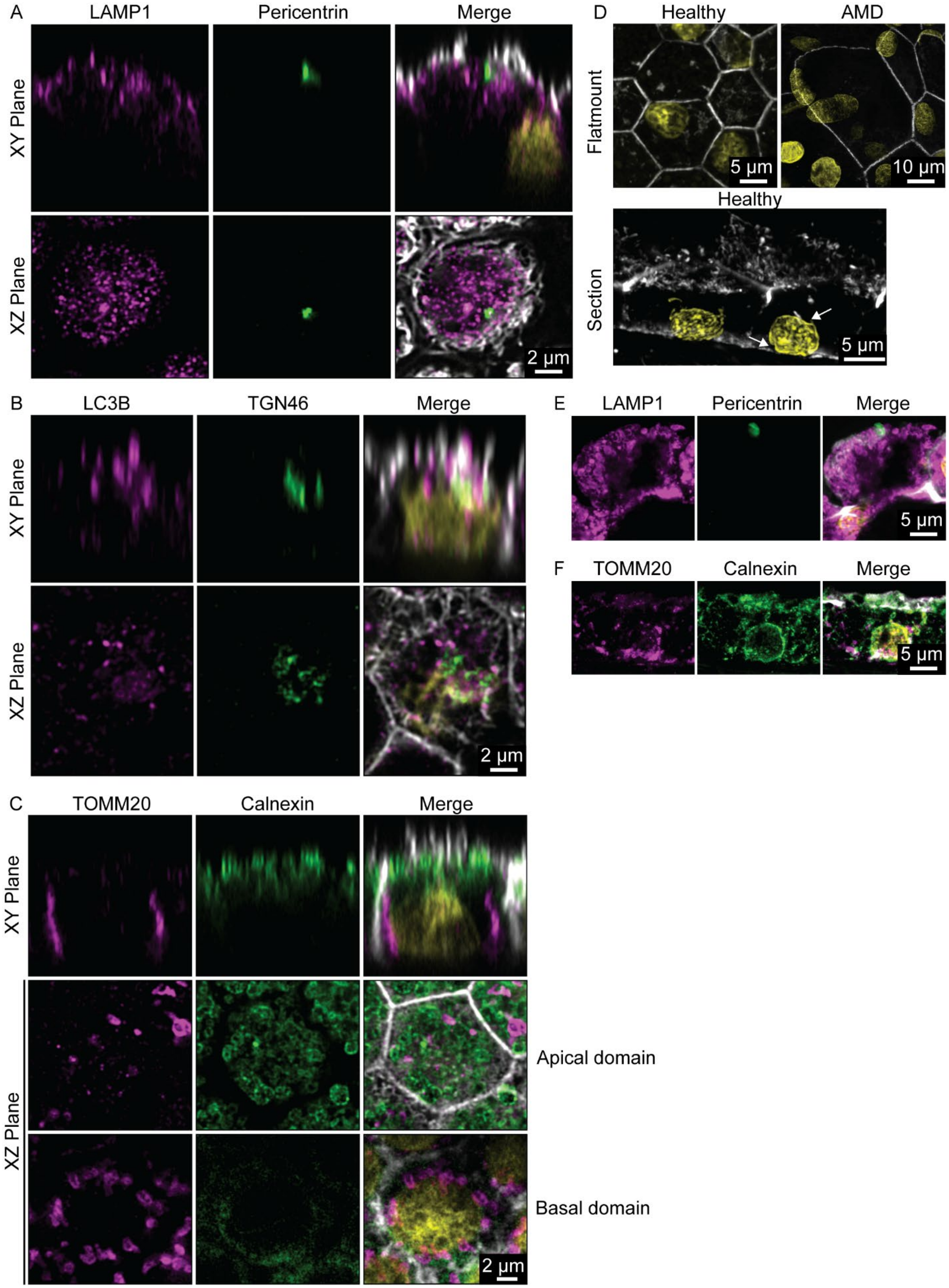
A
LAMP1
Pericentrin
Merge
XY Plane
XZ Plane
2 µm
B
LC3B
TGN46
Merge
XY Plane
XZ Plane
2 µm
C
TOMM20
Calnexin
Merge
XY Plane
XZ Plane
Apical domain
Basal domain
2 µm
D
Healthy
AMD
Flatmount
5 µm
10 µm
Healthy
Section
5 µm
E
LAMP1
Pericentrin
Merge
5 µm
F
TOMM20
Calnexin
Merge
5 µm

501

Figure S22: Co-labeling of organelle pair in iPSC-derived and native human RPE. Co-labeling of (A) LAMP1 and pericentrin, (B) LC3B and TGN46, and (C) TOMM20 and Calnexin in iRPE cells shows the intracellular location of these organelle pairs. Cell borders are shown with phalloidin stain (white), nuclei are shown with Hoechst (yellow). (D) Cell border and nuclei staining of RPE flatmounts (top) and sections (bottom) from age-matched healthy and AMD donors displays differences in cell and nuclei morphology (arrows highlight depressions of nuclear envelope). (E) Co-labeling of LAMP1 and pericentrin, and (F) TOMM20 and Calnexin in sections of human RPE cells shows the intracellular location of these organelle pairs.

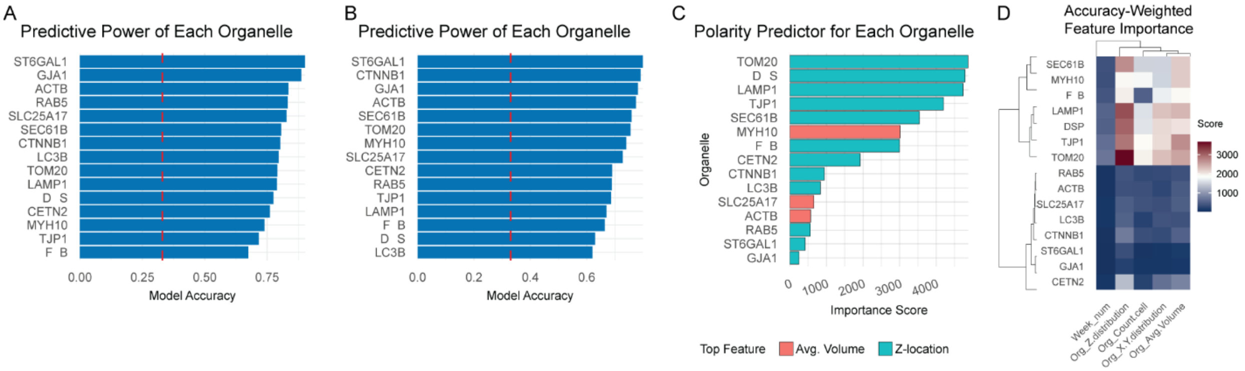

Figure S23: Random Forest accuracy and feature extraction. (A) Random Forest models are ranked by accuracy of polarization prediction between PGE2 and HPI4. (B) Random Forest models are ranked by accuracy of prediction between low, medium, and high polarization among HPI4 cells. (C) Organelle features were extracted and ranked by their importance in predicting cell polarization status in HPI4 data. (D) Heatmap showing the importance scores of organelle features weighted by the model accuracy for HPI4 data.

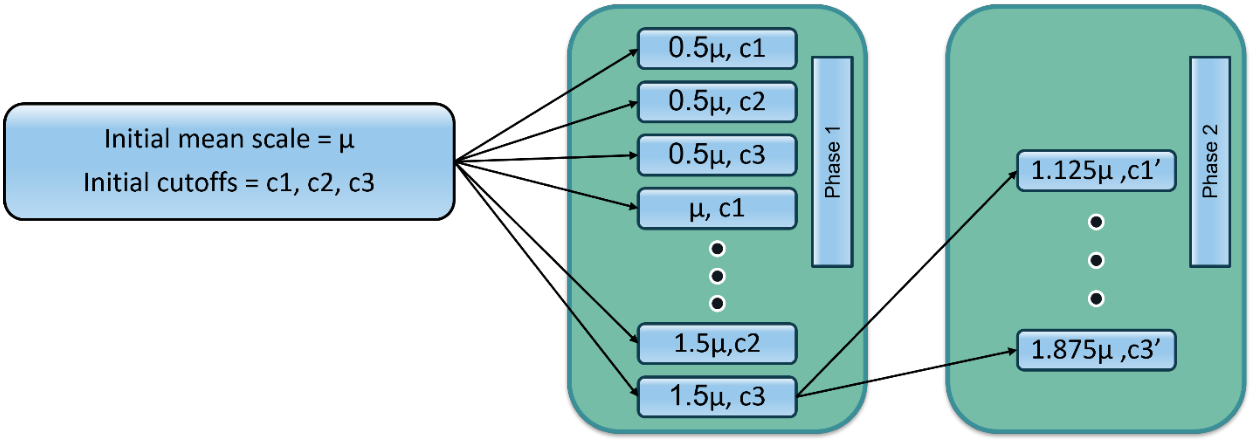

Figure S24: Parameter selection process for organelle segmentation. Schematic representation of the parameter selection process used for organelle segmentation with a human-in-the-loop approach.

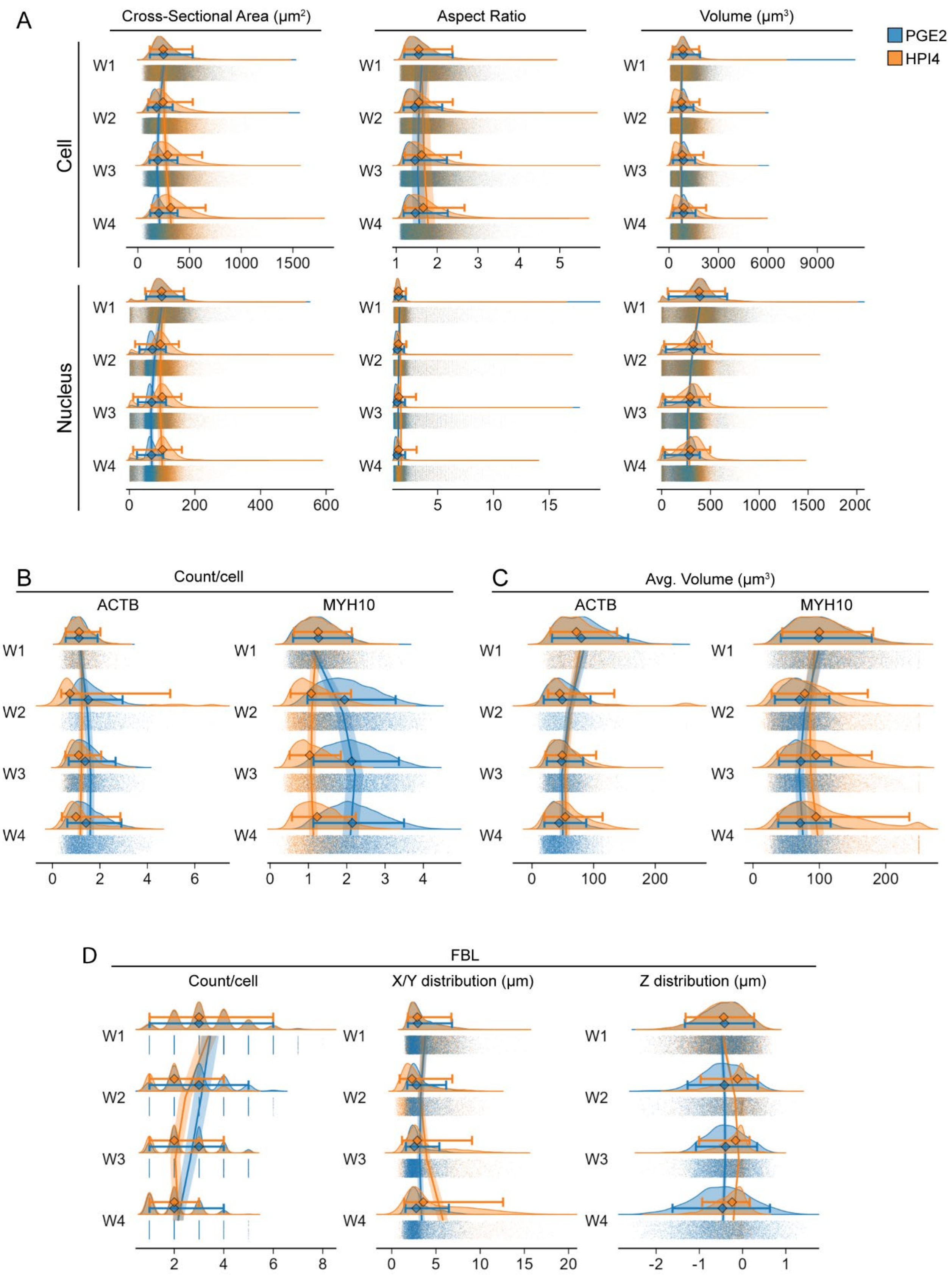
A
Cross-Sectional Area (µm²)
Aspect Ratio
Volume (µm³)
PGE2
HPI4
Cell
Nucleus
W1
W2
W3
W4
B
Count/cell
ACTB
MYH10
C
Avg. Volume (µm³)
ACTB
MYH10
D
FBL
Count/cell
X/Y distribution (µm)
Z distribution (µm)

Figure S25: Graphs from figures 2-4 plotted using single cell data. The raincloud plots from figures 2 (panel A), 3 (panels B-C), and 4 (panel D) are shown here using the average measurements within single cells, instead of further averaging the data at the FOV level. Although it is harder to see changes of trajectories because of the larger spread of data, the conclusions remain unchanged independently of the level at which averaging is performed. (The points in the raincloud plots indicate the average values per cell; the diamonds represent the median and the error bars indicate the 5th and 95th percentiles. N = 30; ***P < 0.005, **P < 0.01, *P < 0.05).

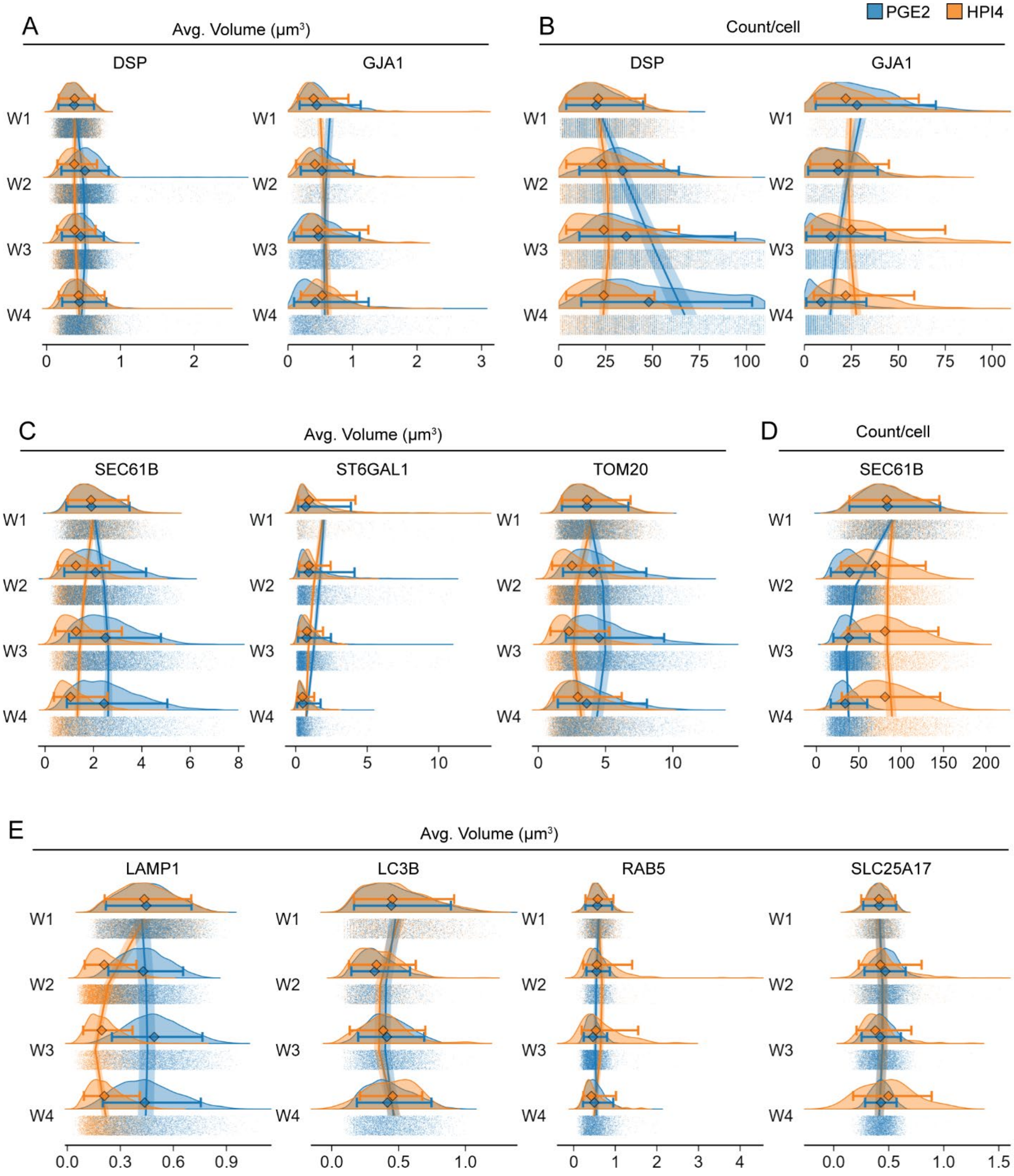

Figure S26: Graphs from figures 5-7 plotted using single cell data. The raincloud plots from figures 5 (panel A-B), 6 (panels C-D), and 7 (panel E) are shown here using the average measurements within single cells, instead of further averaging the data at the

FOV level. Although it is harder to see changes of trajectories because of the larger spread of data, the conclusions remain unchanged independently of the level at which averaging is performed. (The points in the raincloud plots indicate the average values per cell; the diamonds represent the median and the error bars indicate the 5th and 95th percentiles. N = 30; ***P < 0.005, **P < 0.01, *P < 0.05).

Supplementary tables

Table S1: Complete list of the 16 cell lines used in this study.

Table S2: Mask R-CNN training for cell border segmentation – intersection over union.

Table S3: Mask R-CNN training for cell border segmentation – loss metrics.

Table S4: Mask R-CNN training for nucleus border segmentation – intersection over union.

Table S5: Mask R-CNN training for nucleus border segmentation – loss metrics.

Table S6: Summary of image processing algorithms used to segment fluorophore-tagged organelles.

Table S7: Summary of the parameters used in the selection process for the segmentation of fluorophore-tagged organelles.

Table S8: Feature names and definitions.

Table S9: Raw values for cell and nucleus shape metrics used for Figure 2 plots.

Table S10: Summary of shape metrics for cells and nuclei.

Table S11: Summary of shape metrics for fluorophore-tagged organelles.

Table S12: Raw values for fluorophore-tagged organelle shape metrics.

Table S13: Summary of results from GLM. “ACC” = “Acceleration”, “DEC” = “Deceleration”.

Table S14: Uncertainty quantification for GLM.

Table S15: Raw values for the average pairwise spatial interaction maps of Figure 8.

Table S16: Percentage of cytoplasmic volume occupied by each organelle for each condition. The volume percentages were colored to highlight patterns. For PGE2, low values were colored in blue and high values in red. For HPI4, low values were colored in

purple and high values in orange. The volume percentages were summed within each condition, and the summed values are shown in grey.

Table S17: Summary of results from GLM performed on single-cell analysis of Figures S18-19.

Table S18: Raw values of cell shape metrics calculated from ground-truth data generated by six operators, classical segmentation, and Mask R-CNN (Figure S4B).

Table S19: Percentage of junctional organelles located in cytoplasm vs cell border.

Table S1

Catalog number	Clone	Gene	Gene symbol	Protein	Fluorophore	Tagged alleles	Line Abbreviation	Protein Abbreviation	Structure
AICS-0016	184	Actin beta	ACTB	Beta-actin	mEGFP	mono	ACTB	ACTB	Actin filaments
AICS-0024	80	Myosin heavy chain 10	MYH10	Non-muscle myosin heavy chain IIB	mEGFP	mono	MYH10	MYH10	Actomyosin bundles
AICS-0014	6	Fibrillarin	FBL	Fibrillarin	mEGFP	mono	FBL	FBRL	Nucleolus (Dense Fibrillar Component)
AICS-0013	210	Lamin B1	LMNB1	Lamin B1	mEGFP	mono	LMNB1	LMNB1	Nuclear envelope
AICS-0017	65	Desmoplakin	DSP	Desmoplakin	mEGFP	mono	DSP	DESP	Desmosomes
AICS-0053	16	Gap junction protein alpha 1	GJA1	Connexin-43	mEGFP	mono	GJA1	CXA1	Gap junctions
AICS-0023	20	Tight junction protein 1	TJP1	Tight junction protein ZO-1	mEGFP	mono	TJP1	ZO1	Tight junctions
AICS-0058	67	Catenin beta 1	CTNNB1	Beta-catenin	mEGFP	mono	CTNNB1	CTNB1	Adherens junctions
AICS-0032	19	Centrin 2	CETN2	Centrin-2	mTagRFP-T	mono	CETN2	CETN2	Centrioles
AICS-0010	55	Sec61 translocon beta subunit	SEC61B	Sec61 beta	mEGFP	mono	SEC61B	SC61B	Endoplasmic reticulum
AICS-0025	44	ST6 beta-galactoside alpha-2,6-sialyltransferase 1	ST6GAL1	Sialyltransferase 1	mEGFP	bi	ST6GAL1	SIAT1	Golgi apparatus
AICS-0011	27	Translocase of outer mitochondrial membrane 20	TOMM20	Tom20	mEGFP	mono	TOM20	TOM20	Mitochondria
AICS-0022	37	Lysosomal associated membrane protein mono 1	LAMP1	LAMP-1	mEGFP	mono	LAMP1	LAMP1	Lysosome
AICS-0030	22	Microtubule-associated protein 1, light chain 3 beta	LC3B	Autophagy-related protein LC3 B	mEGFP	mono	LC3B	MLP3B	Autophagosomes
AICS-0040	35	RAS-associated protein RAB5A	RAB5	Ras-related protein Rab-5A	mEGFP	bi	RAB5	RAB5A	Endosomes
AICS-0033	115	Solute carrier family 25 (mitochondrial carrier) member 17	SLC25A17	Peroxisomal membrane protein PMP34	mEGFP	mono	SLC25A17	PM34	Peroxisomes

Table S2

Epoch	bbox_AP_95	bbox_AP_50	bbox_AP_75	bbox_AR_50	segm_AP_95	segm_AP_50	segm_AP_75	segm_AR_50
1	0.4295	0.6666	0.5024	0.475	0.4644	0.6665	0.5406	0.5039
2	0.4949	0.7227	0.5943	0.539	0.53	0.7222	0.6196	0.5669
3	0.4979	0.7296	0.5924	0.544	0.5244	0.7301	0.6161	0.5652
4	0.5325	0.7442	0.6366	0.575	0.5505	0.7443	0.6501	0.588
5	0.5207	0.718	0.6218	0.5601	0.5457	0.7184	0.6392	0.5787
6	0.5474	0.7569	0.6465	0.5876	0.5559	0.7558	0.6511	0.5916
7	0.545	0.7274	0.6473	0.5842	0.5615	0.7271	0.6578	0.5928
8	0.5586	0.7525	0.6562	0.5949	0.5776	0.7527	0.6693	0.6054
9	0.5504	0.7403	0.6568	0.5894	0.5653	0.7399	0.6663	0.5997
10	0.5524	0.7605	0.6572	0.5919	0.5773	0.7519	0.6779	0.6091
11	0.5737	0.7599	0.6786	0.6113	0.5913	0.7596	0.6807	0.6238
12	0.5711	0.7714	0.6854	0.6105	0.5947	0.7717	0.6987	0.6261
13	0.562	0.7405	0.66	0.5983	0.5696	0.7403	0.67	0.6016
14	0.5729	0.7599	0.6762	0.6108	0.5871	0.7597	0.6877	0.6203
15	0.5661	0.7641	0.6763	0.6031	0.5817	0.7635	0.6788	0.6156
16	0.5895	0.754	0.6951	0.6235	0.6031	0.7537	0.704	0.631
17	0.5671	0.7337	0.6742	0.6015	0.5877	0.7338	0.6849	0.6145
18	0.5631	0.7348	0.6645	0.5987	0.5784	0.7344	0.6757	0.6075
19	0.5747	0.7627	0.6888	0.6105	0.5951	0.7628	0.7013	0.6254
20	0.5801	0.7644	0.6915	0.6159	0.6048	0.7642	0.7041	0.6334
21	0.5848	0.7607	0.6931	0.6198	0.5804	0.7604	0.692	0.6125
22	0.5785	0.7543	0.6815	0.6138	0.5846	0.7543	0.6921	0.6153
23	0.5524	0.7263	0.6561	0.584	0.5662	0.7264	0.6573	0.5925
24	0.5761	0.774	0.6903	0.6151	0.5916	0.7743	0.7008	0.6254
25	0.5794	0.7468	0.6762	0.6103	0.582	0.7463	0.6851	0.6089
26	0.5921	0.7731	0.7003	0.627	0.5967	0.7728	0.7013	0.6267
27	0.6127	0.7955	0.7203	0.6511	0.6179	0.7952	0.7202	0.6511
28	0.5974	0.7751	0.7024	0.6328	0.6005	0.7758	0.7028	0.6327
29	0.5933	0.7662	0.6947	0.6249	0.5943	0.7569	0.6968	0.6229
30	0.5978	0.7562	0.6976	0.6298	0.5977	0.756	0.6977	0.6255
31	0.6069	0.7667	0.7167	0.6377	0.6069	0.7666	0.7172	0.6347
32	0.597	0.7844	0.7007	0.6329	0.598	0.784	0.6998	0.63
33	0.5935	0.7474	0.6903	0.6214	0.5965	0.7473	0.6908	0.6207
34	0.6101	0.7775	0.7259	0.6426	0.6123	0.7773	0.7164	0.6416
35	0.6011	0.7683	0.7065	0.6323	0.6066	0.768	0.708	0.6324
36	0.6167	0.7768	0.7275	0.6489	0.6193	0.777	0.7287	0.6484
37	0.614	0.7866	0.7255	0.6458	0.6199	0.787	0.7286	0.6464
38	0.5816	0.748	0.6867	0.6147	0.5849	0.748	0.6874	0.6143
39	0.6039	0.7668	0.6985	0.6346	0.6058	0.7667	0.698	0.6317
40	0.6148	0.7958	0.722	0.6489	0.6168	0.7954	0.7234	0.6475
41	0.5972	0.7477	0.6981	0.6264	0.6006	0.7478	0.6992	0.6255
42	0.5953	0.7588	0.696	0.6265	0.5943	0.7586	0.6972	0.6212
43	0.6231	0.794	0.7322	0.659	0.6206	0.7937	0.733	0.6527
44	0.6042	0.7588	0.7109	0.633	0.6106	0.7588	0.7114	0.6351
45	0.5896	0.7577	0.6874	0.6212	0.587	0.7576	0.688	0.6144
46	0.5973	0.7576	0.7074	0.6289	0.601	0.7572	0.7083	0.6286
47	0.6178	0.7773	0.7282	0.6468	0.6197	0.7774	0.7288	0.6446
48	0.6189	0.7956	0.7231	0.6544	0.6207	0.7954	0.7239	0.652
49	0.5934	0.7493	0.6996	0.6219	0.5977	0.7494	0.7002	0.622

50	0.5921	0.7489	0.6987	0.6227	0.5971	0.7491	0.6999	0.6219

Table S3

Epoch	loss	loss_box_reg	loss_classifier	loss_mask	loss_objectness	loss_rpn_box_reg	lr
1	1.390013	0.475291	0.30618	0.367236	0.149609	0.091698	0.0005
2	1.009977	0.36064	0.229052	0.27458	0.081098	0.064606	0.0005
3	0.90551	0.32164	0.208144	0.257728	0.064804	0.053195	0.0005
4	0.860277	0.303176	0.197201	0.249956	0.05763	0.052314	0.0005
5	0.821657	0.288986	0.188595	0.242924	0.053507	0.047644	0.0005
6	0.794918	0.276706	0.184504	0.238441	0.049281	0.045986	0.0005
7	0.772949	0.270093	0.178235	0.233557	0.046377	0.044687	0.0005
8	0.75342	0.263652	0.175095	0.228927	0.044029	0.041717	0.0005
9	0.745991	0.259206	0.171755	0.228068	0.043746	0.043216	0.0005
10	0.729923	0.252697	0.169218	0.224735	0.041653	0.041619	0.0005
11	0.719104	0.251139	0.166438	0.221872	0.040374	0.039281	0.0005
12	0.706536	0.245844	0.160323	0.220362	0.040923	0.039085	0.0005
13	0.698825	0.243929	0.161759	0.216013	0.038334	0.03879	0.0005
14	0.686356	0.237822	0.157937	0.215808	0.03679	0.037999	0.0005
15	0.677597	0.235874	0.155017	0.214012	0.035781	0.036914	0.0005
16	0.680397	0.23676	0.154674	0.214866	0.037258	0.036839	0.0005
17	0.672932	0.234279	0.154343	0.212378	0.035273	0.03666	0.0005
18	0.661789	0.230121	0.149563	0.210864	0.035635	0.035606	0.0005
19	0.658655	0.2295	0.14865	0.209536	0.034976	0.035993	0.0005
20	0.65557	0.227103	0.150166	0.208739	0.034777	0.034785	0.0005
21	0.650836	0.225454	0.149092	0.207784	0.033493	0.035013	0.0005
22	0.648505	0.225866	0.148855	0.205712	0.032897	0.035175	0.0005
23	0.644111	0.22274	0.1471	0.205739	0.033378	0.035153	0.0005
24	0.635886	0.220515	0.144359	0.204857	0.03189	0.034265	0.0005
25	0.632323	0.219074	0.142814	0.205528	0.031534	0.033372	0.0005
26	0.601284	0.206649	0.133774	0.199153	0.029957	0.03175	0.000158
27	0.59772	0.205985	0.133871	0.1994	0.028047	0.030417	0.000158
28	0.59259	0.204935	0.132599	0.197689	0.027507	0.029861	0.000158
29	0.594463	0.205865	0.134304	0.199082	0.027121	0.028091	0.000158
30	0.598204	0.206768	0.133098	0.197991	0.028328	0.032019	0.000158
31	0.591233	0.204087	0.131915	0.198212	0.027001	0.030018	0.000158
32	0.593029	0.204171	0.132534	0.196313	0.027961	0.03205	0.000158
33	0.593133	0.204016	0.133199	0.197672	0.027342	0.030904	0.000158
34	0.586192	0.202459	0.130712	0.19616	0.027074	0.029787	0.000158
35	0.587044	0.201987	0.130887	0.196868	0.027318	0.029984	0.000158
36	0.59008	0.203342	0.132113	0.197191	0.026598	0.030837	0.000158
37	0.587424	0.202108	0.13151	0.195969	0.027573	0.030264	0.000158
38	0.578297	0.199462	0.128782	0.195586	0.026109	0.028358	0.000158
39	0.580884	0.200134	0.12887	0.196061	0.026641	0.029178	0.000158
40	0.578169	0.199117	0.128183	0.196115	0.02657	0.028185	0.000158
41	0.579391	0.200744	0.128909	0.195495	0.025638	0.028604	0.000158
42	0.572362	0.196868	0.128157	0.19373	0.025053	0.028554	0.000158
43	0.568922	0.197383	0.125448	0.194036	0.023786	0.028269	0.000158
44	0.574779	0.198328	0.129333	0.194119	0.025077	0.027922	0.000158

45	0.572423	0.196663	0.126591	0.193382	0.026795	0.028992	0.000158
46	0.572729	0.19643	0.126824	0.19337	0.026778	0.029327	0.000158
47	0.56584	0.194309	0.124642	0.193966	0.024837	0.028086	0.000158
48	0.566921	0.194031	0.125056	0.192743	0.026619	0.028472	0.000158
49	0.567973	0.194579	0.125724	0.193164	0.025622	0.028884	0.000158
50	0.570723	0.196559	0.125588	0.192081	0.026427	0.030068	0.000158

Table S4

Epoch	bbox_AP_95	bbox_AP_50	bbox_AP_75	bbox_AR_50	segm_AP_95	segm_AP_50	segm_AP_75	segm_AR_50
1	0.6531	0.7404	0.7195	0.6729	0.6369	0.7404	0.7199	0.6526
2	0.6452	0.7295	0.7173	0.6705	0.5913	0.7295	0.7058	0.6175
3	0.6838	0.7603	0.7393	0.7036	0.673	0.7603	0.7394	0.6901
4	0.689	0.7414	0.7302	0.7052	0.6522	0.7414	0.7301	0.6717
5	0.7016	0.7707	0.76	0.7198	0.6654	0.7707	0.7597	0.6834
6	0.665	0.7307	0.7104	0.6794	0.6251	0.7307	0.7097	0.6423
7	0.6847	0.7317	0.7207	0.6981	0.6626	0.7317	0.7207	0.6798
8	0.6924	0.7414	0.7405	0.7064	0.6731	0.7414	0.7311	0.688
9	0.7004	0.7517	0.7413	0.714	0.6824	0.7517	0.7413	0.6966
10	0.7063	0.7713	0.7603	0.7247	0.6754	0.7714	0.7603	0.6944
11	0.7109	0.7611	0.7508	0.7266	0.6613	0.7611	0.7507	0.6772
12	0.7277	0.7805	0.7701	0.7431	0.7043	0.7805	0.7699	0.7217
13	0.7118	0.7612	0.7507	0.7239	0.6752	0.7611	0.7505	0.6925
14	0.6658	0.7218	0.7116	0.6824	0.6457	0.7218	0.7116	0.6608
15	0.7191	0.781	0.7704	0.7351	0.7284	0.7811	0.7702	0.7394
16	0.6972	0.7419	0.7314	0.709	0.6694	0.7419	0.7315	0.687
17	0.7237	0.7808	0.7702	0.741	0.696	0.7808	0.7699	0.7176
18	0.7129	0.7518	0.7414	0.7234	0.691	0.7517	0.7413	0.7068
19	0.7016	0.7419	0.7317	0.7119	0.6757	0.7419	0.7316	0.6917
20	0.6803	0.7221	0.7118	0.6903	0.6496	0.7221	0.7116	0.6656
21	0.7308	0.7809	0.7701	0.745	0.6932	0.7809	0.77	0.7122
22	0.6857	0.7319	0.7216	0.6983	0.6512	0.7319	0.7216	0.6669
23	0.682	0.7221	0.7212	0.6939	0.663	0.7221	0.7116	0.6782
24	0.715	0.7608	0.7507	0.726	0.6709	0.7608	0.741	0.6887
25	0.7326	0.7811	0.7706	0.7439	0.7011	0.781	0.7708	0.7165
26	0.7374	0.7813	0.7709	0.7492	0.7093	0.7813	0.7709	0.7257
27	0.7192	0.7616	0.7509	0.7311	0.6889	0.7615	0.7507	0.7067
28	0.7521	0.8002	0.7901	0.7645	0.7133	0.8002	0.7898	0.7315
29	0.6974	0.7322	0.7221	0.707	0.6604	0.7322	0.7221	0.6769
30	0.7004	0.7323	0.7222	0.7069	0.6594	0.7323	0.722	0.6751
31	0.7321	0.781	0.77	0.7433	0.6927	0.7809	0.7602	0.7122
32	0.7652	0.8106	0.8003	0.7765	0.732	0.8106	0.8002	0.7487
33	0.7188	0.7613	0.7511	0.7287	0.6724	0.7614	0.7508	0.6878
34	0.7091	0.7418	0.7317	0.7164	0.6655	0.7418	0.7316	0.6813
35	0.7123	0.7608	0.7407	0.7239	0.68	0.7608	0.7405	0.6994
36	0.7193	0.7715	0.761	0.7329	0.7002	0.7715	0.7609	0.7166
37	0.7054	0.742	0.7411	0.7156	0.66	0.742	0.7314	0.6777
38	0.694	0.732	0.7216	0.7021	0.6479	0.7319	0.7214	0.6628
39	0.7331	0.7813	0.7611	0.7441	0.6978	0.7813	0.7609	0.716
40	0.7475	0.7912	0.7811	0.7585	0.7013	0.7912	0.7809	0.7199
41	0.7071	0.7419	0.7412	0.7169	0.6747	0.7418	0.7318	0.6916
42	0.7224	0.7619	0.7516	0.7326	0.6827	0.7619	0.7515	0.7011
43	0.758	0.8014	0.791	0.7697	0.7171	0.8014	0.7907	0.7354
44	0.7062	0.7419	0.7317	0.7135	0.6669	0.7419	0.7315	0.6821
45	0.7119	0.7516	0.7413	0.7227	0.6756	0.7516	0.7411	0.6928
46	0.7027	0.7418	0.7314	0.712	0.6613	0.7418	0.7313	0.6783
47	0.7191	0.7617	0.7515	0.7298	0.6818	0.7617	0.7512	0.6984
48	0.7032	0.7421	0.7415	0.7158	0.6778	0.7421	0.7413	0.694
49	0.7178	0.7615	0.751	0.7295	0.6736	0.7615	0.7508	0.6916

50	0.7341	0.7813	0.7703	0.744	0.7012	0.7812	0.7607	0.7192

Table S5

Epoch	loss	loss_box_reg	loss_classifier	loss_mask	loss_objectness	loss_rpn_box_reg	lr
1	0.838751	0.247404	0.178508	0.224367	0.110804	0.077668	0.0005
2	0.555887	0.16461	0.128982	0.162902	0.051898	0.047495	0.0005
3	0.484692	0.141295	0.110086	0.153483	0.041102	0.038727	0.0005
4	0.462795	0.132746	0.104503	0.148404	0.040035	0.037107	0.0005
5	0.43166	0.124083	0.099344	0.144988	0.031864	0.03138	0.0005
6	0.418412	0.118358	0.094182	0.143669	0.031823	0.03038	0.0005
7	0.409054	0.115222	0.092906	0.141597	0.029989	0.02934	0.0005
8	0.399813	0.113408	0.087677	0.141212	0.027572	0.029944	0.0005
9	0.394873	0.112442	0.08765	0.140607	0.025091	0.029084	0.0005
10	0.3818	0.107146	0.082956	0.138579	0.025028	0.028092	0.0005
11	0.3781	0.106928	0.081935	0.138556	0.02377	0.026911	0.0005
12	0.376011	0.106031	0.08207	0.138074	0.022454	0.027382	0.0005
13	0.370705	0.103683	0.081366	0.136299	0.023469	0.025887	0.0005
14	0.373373	0.104068	0.082525	0.136419	0.024398	0.025962	0.0005
15	0.362779	0.100991	0.077861	0.135901	0.022435	0.02559	0.0005
16	0.355331	0.098443	0.076725	0.134096	0.021226	0.024841	0.0005
17	0.350147	0.096747	0.075761	0.132874	0.021587	0.023178	0.0005
18	0.347773	0.097648	0.074658	0.132665	0.019446	0.023356	0.0005
19	0.351188	0.097118	0.074765	0.133109	0.020374	0.02582	0.0005
20	0.345223	0.096263	0.072415	0.131966	0.020113	0.024467	0.0005
21	0.340227	0.092859	0.074593	0.131051	0.019914	0.02181	0.0005
22	0.344072	0.095411	0.073859	0.130965	0.019084	0.024753	0.0005
23	0.338139	0.094781	0.072423	0.131024	0.01736	0.022551	0.0005
24	0.336335	0.093842	0.07124	0.13036	0.01821	0.022683	0.0005
25	0.334131	0.092108	0.070192	0.130768	0.018163	0.022901	0.0005
26	0.316468	0.087154	0.065106	0.127756	0.015692	0.020761	0.000158
27	0.311263	0.084467	0.063395	0.126112	0.015046	0.022243	0.000158
28	0.312731	0.085776	0.063157	0.128449	0.015608	0.01974	0.000158
29	0.310941	0.085041	0.063248	0.126777	0.015176	0.020699	0.000158
30	0.312916	0.08609	0.064738	0.127314	0.015304	0.019469	0.000158
31	0.30959	0.084898	0.062595	0.125664	0.015289	0.021143	0.000158
32	0.310305	0.084961	0.062438	0.127185	0.014992	0.020729	0.000158
33	0.309201	0.08544	0.062527	0.126337	0.014086	0.020811	0.000158
34	0.309739	0.084947	0.062434	0.126841	0.015415	0.020101	0.000158
35	0.306661	0.083547	0.061823	0.125593	0.015196	0.020503	0.000158
36	0.306705	0.083888	0.060305	0.127112	0.01459	0.020809	0.000158
37	0.305681	0.084627	0.06161	0.126253	0.01384	0.019351	0.000158
38	0.308847	0.084944	0.061405	0.126855	0.01373	0.021912	0.000158
39	0.305934	0.083875	0.061566	0.125822	0.015141	0.01953	0.000158
40	0.301826	0.083492	0.059704	0.126031	0.013668	0.018931	0.000158
41	0.304734	0.084464	0.060748	0.126273	0.014491	0.018758	0.000158
42	0.300847	0.082414	0.060558	0.125763	0.01319	0.018922	0.000158
43	0.302303	0.082805	0.061036	0.125687	0.014052	0.018723	0.000158
44	0.303313	0.08309	0.061844	0.124864	0.013365	0.02015	0.000158

45	0.301908	0.082666	0.060653	0.125518	0.013518	0.019553	0.000158
46	0.299288	0.082414	0.060461	0.124934	0.013512	0.017968	0.000158
47	0.302496	0.083233	0.059628	0.12581	0.014387	0.019437	0.000158
48	0.300508	0.083268	0.058676	0.125377	0.013117	0.020071	0.000158
49	0.296942	0.081331	0.058592	0.124522	0.013263	0.019235	0.000158
50	0.294984	0.080339	0.057156	0.124168	0.013231	0.020089	0.000158

Table S6

Organelle	Pre-processing	Segmentation	Post-processing
ACTB	1) Auto contrast normalization 2) Edge-preserving smoothing	2D Frangi vesselness	-
MYH10	1) Auto contrast normalization 2) Edge-preserving smoothing	2D Frangi vesselness	-
FBL	1) Auto contrast normalization 2) 3D Gaussian smoothing	3D LoG based spot filter	Remove small objects
LMNB1	1) Auto contrast normalization 2) 3D Gaussian smoothing	1) Obtain middle frame from intensity distribution 2) Obtain enclosing cell outline from middle frame with 2D Frangi vesselness. Find cell interior 3) Seeded watershed and cell interior are used to obtain LMNB1 layer	-
DSP	1) Auto contrast normalization 2) 2D Gaussian smoothing	1) 3D LoG based spot filter 2) 3D Watershed	-
GJA1	1) Auto contrast normalization 2) 2D Gaussian smoothing	3D LoG based spot filter	-
TJP1	1) Auto contrast normalization 2) Edge-preserving smoothing	2D Frangi vesselness	Remove small objects
CTNNB1	1) Auto contrast normalization 2) 3D Gaussian smoothing	2D LoG based spot filter	-
CETN2	1) Auto contrast normalization 2) 2D Gaussian smoothing	1) 3D LoG based spot filter 2) 3D Watershed	Remove small objects

SEC61B	1) Auto contrast normalization 2) Edge-preserving smoothing	2D Frangi vesselness	Remove small objects
ST6GAL1	1) Auto contrast normalization 2) 3D Gaussian smoothing	3D LoG based spot filter	-
TOM20	1) Auto contrast normalization 2) 3D Gaussian smoothing	2D Frangi vesselness	Remove small objects
LAMP1	1) Auto contrast normalization 2) Edge-preserving smoothing	2D LoG based spot filter	1) Fill holes 2) Remove small objects
LC3B	1) Auto contrast normalization 2) 2D Gaussian smoothing	1) 3D LoG based spot filter 2) 3D Watershed	Remove small objects
RAB5	1) Auto contrast normalization 2) 2D Gaussian smoothing	2D LoG based spot filter	1) Fill holes 2) Remove small objects
SLC25A17	1) Auto contrast normalization 2) 2D Gaussian smoothing	3D LoG based spot filter	-

Table S7

		initial μ	σ	selected μ	selected σ	selected μ	selected σ	selected μ	selected σ	selected μ	selected σ
	Channel	phase 0		phase 1		phase 2		phase 3		extra phase - for seleced	
pixel	ACTB	5		2.5		2.5		2.1875			
parameter		1.666667		0.833333333	0.05	0.833333333	0.05	0.729166667	0.05		
pixel	MYH10	5		2.5		1.875		1.640625			
parameter		1.666667		0.833333333	0.01	0.625	0.01	0.546875	0.01		
pixel	FBL	7.5		7.5		7.5		6.5625			
parameter		2.5		2.5	0.05	2.5	0.03	2.1875	0.05		
pixel	LMNB1	3		NA		NA		NA			
parameter		1		NA	NA	NA	NA	NA	NA		
pixel	DSP	4.5		2.25		1.6875		1.6875		1.8984375	
parameter		1.5		0.75	0.01	0.5625	0.01	0.5625	0.03	0.6328125	0.01
pixel	GJA1	8.5		4.25		4.25		4.78125		3.1875	
parameter		2.833333		1.416666667	0.05	1.416666667	0.03	1.59375	0.03	1.0625	0.01
pixel	TJP1	3		1.5		1.5		1.5			
parameter		1		0.5	0.1	0.5	0.1	0.5	0.1		
pixel	CTNNB1	3.25		1.625		2.03125		1.77734375			
parameter		1.083333		0.541666667	0.05	0.677083333	0.05	0.592447917	0.05		
pixel	CETN2	4.5		6.75		5.0625		5.0625			
parameter		1.5		2.25	0.1	1.6875	0.1	1.6875	0.075		
pixel	SEC61B	3		1.5		1.875		1.875			
parameter		1		0.5	0.05	0.625	0.075	0.625	0.075		
pixel	ST6GAL1	3.75		5.625		5.625		5.625			
parameter		1.25		1.875	0.1	1.875	0.075	1.875	0.0875		
pixel	TOM20	2.75		2.75		3.4375		3.4375			
parameter		0.916667		0.916666667	0.1	1.145833333	0.1	1.145833333	0.12		
pixel	LAMP1	3.5		3.5		3.5		3.0625			
parameter		1.166667		1.166666667	0.05	1.166666667	0.07	1.020833333	0.05		
pixel	LC3B	4.5		4.5		3.375		3.375			
parameter		1.5		1.5	0.1	1.125	0.1	1.125	0.075		
pixel	RAB5	3.25		4.875		6.09375		5.33203125			
parameter		1.083333		1.625	0.1	2.03125	0.05	1.77734375	0.07		
pixel	SLC25A17	2.75		2.75		2.75		2.75		2.40625	
parameter		0.916667		0.916666667	0.05	0.916666667	0.05	0.916666667	0.05	0.802083333	0.03

Table S8

Feature name in the manuscript	Feature name in the code	Brief description
Volume	Volume	Total 3D space occupied by the cell or organelle.
Centroid	Centroid	The geometric center of the organelle in (x, y, z) coordinates. Also the approximate center of mass.
Width	X/Y span	The extent of the organelle along the x- and y-axis
Height	Z span	The extent of the organelle along the z-axis.
Cross-sectional area	MIP Area	Maximum intensity projection area of the organelle in 2D in the XY plane.
Aspect ratio	2D Aspect Ratio	Ratio of major to minor axis in the 2D projection.
Minimum Feret's diameter	Min feret	The shortest distance between two parallel tangents to the organelle.
Maximum Feret's diameter	Max feret	The longest distance between two parallel tangents to the organelle.
Volume percentage	Volume fraction	Ratio of the organelle's volume to the total cell volume.
Z-distribution	Z-distribution	Distribution of organelle positions along the z-axis within the cell, with respect to the cell centroid.
X/Y-distribution	Radial distribution 2D	Distribution of organelle positions from the cell centroid in the xy-plane.
Distance from lateral border	Mean 2D distance to wall	Average shortest distance from the organelle to the cell membrane in 2D (XY plane).
Distance from apical border	Mean Top z-distance	Mean distance of the organelle from the top of the cell along the z-axis.

Table S9

Week	Treatment	Well	FOV	N	Metric	mean	median	sd
W1	HPI4	well_1	F001	2006	Cell AR	1.611741	1.525532	0.361197
W1	HPI4	well_1	F002	1797	Cell AR	1.62973	1.533333	0.376823
W1	HPI4	well_1	F003	2325	Cell AR	1.62515	1.54386	0.362421
W1	HPI4	well_1	F004	2112	Cell AR	1.626038	1.539663	0.367287
W1	HPI4	well_1	F005	2475	Cell AR	1.61919	1.542553	0.350214
W1	HPI4	well_1	F006	1903	Cell AR	1.654616	1.58	0.374006
W1	HPI4	well_2	F001	1728	Cell AR	1.669375	1.591229	0.383772
W1	HPI4	well_2	F002	2072	Cell AR	1.668421	1.573431	0.428992
W1	HPI4	well_2	F003	2175	Cell AR	1.658579	1.566667	0.41403
W1	HPI4	well_2	F004	2271	Cell AR	1.65312	1.565789	0.394568
W1	HPI4	well_2	F005	2464	Cell AR	1.616316	1.545455	0.351241
W1	HPI4	well_2	F006	2103	Cell AR	1.635089	1.551282	0.371235
W1	HPI4	well_3	F001	1564	Cell AR	1.672203	1.571429	0.420405
W1	HPI4	well_3	F002	1898	Cell AR	1.649983	1.56012	0.386242
W1	HPI4	well_3	F003	1917	Cell AR	1.650318	1.555556	0.400563
W1	HPI4	well_3	F004	2384	Cell AR	1.660523	1.574242	0.390455
W1	HPI4	well_3	F005	2264	Cell AR	1.622399	1.536232	0.369442
W1	HPI4	well_3	F006	2274	Cell AR	1.634351	1.545455	0.387242
W1	HPI4	well_4	F001	1973	Cell AR	1.648606	1.56383	0.391357
W1	HPI4	well_4	F002	2276	Cell AR	1.64386	1.565217	0.381616
W1	HPI4	well_4	F003	2401	Cell AR	1.638112	1.559322	0.372618
W1	HPI4	well_4	F004	2109	Cell AR	1.647627	1.553846	0.384586
W1	HPI4	well_4	F005	2355	Cell AR	1.64662	1.555556	0.392472
W1	HPI4	well_4	F006	2283	Cell AR	1.641868	1.547945	0.380046
W1	HPI4	well_5	F001	2187	Cell AR	1.621755	1.542373	0.355008
W1	HPI4	well_5	F002	2196	Cell AR	1.631835	1.535714	0.379772
W1	HPI4	well_5	F003	2472	Cell AR	1.615102	1.533333	0.368672
W1	HPI4	well_5	F004	2165	Cell AR	1.602332	1.508065	0.359038
W1	HPI4	well_5	F005	2000	Cell AR	1.62645	1.529857	0.393519
W1	HPI4	well_5	F006	1889	Cell AR	1.646555	1.55	0.407838
W1	PGE2	well_1	F001	2346	Cell AR	1.647404	1.5495	0.407044
W1	PGE2	well_1	F002	2124	Cell AR	1.641	1.55717	0.385623
W1	PGE2	well_1	F003	2450	Cell AR	1.649918	1.561005	0.392591
W1	PGE2	well_1	F004	2589	Cell AR	1.590532	1.5	0.351703
W1	PGE2	well_1	F005	2970	Cell AR	1.595264	1.51411	0.351374
W1	PGE2	well_1	F006	2939	Cell AR	1.586359	1.5	0.353715
W1	PGE2	well_2	F001	2002	Cell AR	1.624373	1.538462	0.366371
W1	PGE2	well_2	F002	2113	Cell AR	1.643764	1.557895	0.367191
W1	PGE2	well_2	F003	2227	Cell AR	1.638275	1.557143	0.365139
W1	PGE2	well_2	F004	2400	Cell AR	1.630247	1.542615	0.369136
W1	PGE2	well_2	F005	2511	Cell AR	1.620307	1.542857	0.35256
W1	PGE2	well_2	F006	1903	Cell AR	1.646687	1.548387	0.402047
W1	PGE2	well_3	F001	2114	Cell AR	1.658101	1.569426	0.397277
W1	PGE2	well_3	F002	2013	Cell AR	1.659632	1.577465	0.379599

W1	PGE2	well_3	F003	2208	Cell AR	1.678904	1.578947	0.407868
W1	PGE2	well_3	F004	2146	Cell AR	1.653282	1.574014	0.376912
W1	PGE2	well_3	F005	2403	Cell AR	1.619592	1.539683	0.366746
W1	PGE2	well_3	F006	2147	Cell AR	1.633163	1.552941	0.373106
W1	PGE2	well_4	F001	1891	Cell AR	1.657675	1.567164	0.394335
W1	PGE2	well_4	F002	1986	Cell AR	1.669902	1.571429	0.404885
W1	PGE2	well_4	F003	1905	Cell AR	1.688021	1.603774	0.420306
W1	PGE2	well_4	F004	2397	Cell AR	1.637547	1.548387	0.37318
W1	PGE2	well_4	F005	2079	Cell AR	1.658455	1.566038	0.389857
W1	PGE2	well_4	F006	2134	Cell AR	1.670233	1.576271	0.40407
W1	PGE2	well_5	F001	1894	Cell AR	1.642352	1.548166	0.385937
W1	PGE2	well_5	F002	2205	Cell AR	1.649476	1.56	0.389251
W1	PGE2	well_5	F003	1891	Cell AR	1.653938	1.557895	0.404053
W1	PGE2	well_5	F004	2105	Cell AR	1.644238	1.560976	0.380495
W1	PGE2	well_5	F005	2176	Cell AR	1.632569	1.555556	0.361778
W1	PGE2	well_5	F006	1968	Cell AR	1.675797	1.587129	0.402701
W2	HPI4	well_1	F001	1889	Cell AR	1.651832	1.56	0.396318
W2	HPI4	well_1	F002	2032	Cell AR	1.64194	1.551372	0.374142
W2	HPI4	well_1	F003	2276	Cell AR	1.630701	1.541667	0.379996
W2	HPI4	well_1	F004	2401	Cell AR	1.664733	1.58	0.386708
W2	HPI4	well_1	F005	1985	Cell AR	1.673268	1.58209	0.40299
W2	HPI4	well_1	F006	1738	Cell AR	1.666677	1.574119	0.436087
W2	HPI4	well_2	F001	1684	Cell AR	1.618294	1.544304	0.377418
W2	HPI4	well_2	F002	1846	Cell AR	1.653682	1.560976	0.398637
W2	HPI4	well_2	F003	2013	Cell AR	1.644544	1.566667	0.377938
W2	HPI4	well_2	F004	2491	Cell AR	1.617285	1.537037	0.358332
W2	HPI4	well_2	F005	2106	Cell AR	1.668862	1.588235	0.385798
W2	HPI4	well_2	F006	1974	Cell AR	1.629701	1.547247	0.369944
W2	HPI4	well_3	F001	1972	Cell AR	1.596851	1.515625	0.348699
W2	HPI4	well_3	F002	1767	Cell AR	1.610231	1.529412	0.36309
W2	HPI4	well_3	F003	1332	Cell AR	1.717113	1.597168	0.466788
W2	HPI4	well_3	F004	2224	Cell AR	1.644816	1.56338	0.381501
W2	HPI4	well_3	F005	2099	Cell AR	1.651335	1.557143	0.408886
W2	HPI4	well_3	F006	2048	Cell AR	1.622739	1.535718	0.375902
W2	HPI4	well_4	F001	1980	Cell AR	1.584136	1.508336	0.340103
W2	HPI4	well_4	F002	1990	Cell AR	1.578639	1.494764	0.354303
W2	HPI4	well_4	F003	2045	Cell AR	1.670545	1.589744	0.39244
W2	HPI4	well_4	F004	2328	Cell AR	1.606234	1.517444	0.364528
W2	HPI4	well_4	F005	1893	Cell AR	1.6422	1.54717	0.403823
W2	HPI4	well_4	F006	1970	Cell AR	1.627334	1.533908	0.389795
W2	HPI4	well_5	F001	1814	Cell AR	1.645784	1.5545	0.408245
W2	HPI4	well_5	F002	1701	Cell AR	1.65371	1.571429	0.38775
W2	HPI4	well_5	F003	1841	Cell AR	1.617238	1.527778	0.371024
W2	HPI4	well_5	F004	2312	Cell AR	1.629818	1.540112	0.376484
W2	HPI4	well_5	F005	2140	Cell AR	1.648519	1.557255	0.406411
W2	HPI4	well_5	F006	1915	Cell AR	1.647712	1.567164	0.390728
W2	PGE2	well_1	F001	2508	Cell AR	1.55505	1.47285	0.339344

W2	PGE2	well_1	F002	2196	Cell AR	1.541341	1.459238	0.355761
W2	PGE2	well_1	F003	3078	Cell AR	1.585203	1.490909	0.363816
W2	PGE2	well_1	F004	2519	Cell AR	1.52219	1.458333	0.301431
W2	PGE2	well_1	F005	3120	Cell AR	1.528441	1.455882	0.3153
W2	PGE2	well_1	F006	3092	Cell AR	1.485433	1.427619	0.265264
W2	PGE2	well_2	F001	2524	Cell AR	1.507545	1.4375	0.291062
W2	PGE2	well_2	F002	3220	Cell AR	1.512475	1.442623	0.29828
W2	PGE2	well_2	F003	3754	Cell AR	1.535256	1.456331	0.317084
W2	PGE2	well_2	F004	3308	Cell AR	1.51592	1.438596	0.30303
W2	PGE2	well_2	F005	2732	Cell AR	1.514032	1.440927	0.312525
W2	PGE2	well_2	F006	2641	Cell AR	1.518885	1.458333	0.281448
W2	PGE2	well_3	F001	2301	Cell AR	1.540521	1.464286	0.327757
W2	PGE2	well_3	F002	3625	Cell AR	1.569193	1.491228	0.34138
W2	PGE2	well_3	F003	3166	Cell AR	1.525213	1.453333	0.316266
W2	PGE2	well_3	F004	3460	Cell AR	1.503987	1.4375	0.288269
W2	PGE2	well_3	F005	2810	Cell AR	1.512007	1.441709	0.3071
W2	PGE2	well_3	F006	2454	Cell AR	1.544514	1.472213	0.331485
W2	PGE2	well_4	F001	2778	Cell AR	1.533053	1.461538	0.323109
W2	PGE2	well_4	F002	3296	Cell AR	1.517816	1.4375	0.317151
W2	PGE2	well_4	F003	3146	Cell AR	1.534	1.452606	0.328229
W2	PGE2	well_4	F004	3610	Cell AR	1.526573	1.45614	0.308515
W2	PGE2	well_4	F005	3154	Cell AR	1.522216	1.442857	0.31722
W2	PGE2	well_4	F006	2634	Cell AR	1.528536	1.454545	0.301809
W2	PGE2	well_5	F001	3092	Cell AR	1.546931	1.462963	0.322474
W2	PGE2	well_5	F002	3496	Cell AR	1.536264	1.459238	0.322262
W2	PGE2	well_5	F003	3256	Cell AR	1.579172	1.5	0.349038
W2	PGE2	well_5	F004	2836	Cell AR	1.502388	1.437928	0.290218
W2	PGE2	well_5	F005	2895	Cell AR	1.500282	1.432836	0.276907
W2	PGE2	well_5	F006	3259	Cell AR	1.508184	1.440678	0.302732
W3	HPI4	well_1	F001	876	Cell AR	1.672931	1.5625	0.44432
W3	HPI4	well_1	F002	1131	Cell AR	1.735562	1.649573	0.436898
W3	HPI4	well_1	F003	933	Cell AR	1.72368	1.619835	0.420327
W3	HPI4	well_1	F004	1484	Cell AR	1.686655	1.588235	0.397705
W3	HPI4	well_1	F005	1385	Cell AR	1.755835	1.645833	0.466115
W3	HPI4	well_1	F006	955	Cell AR	1.799235	1.670588	0.531356
W3	HPI4	well_2	F001	819	Cell AR	1.741024	1.647059	0.457646
W3	HPI4	well_2	F002	1035	Cell AR	1.72685	1.625	0.436602
W3	HPI4	well_2	F003	1013	Cell AR	1.710689	1.590361	0.424821
W3	HPI4	well_2	F004	1327	Cell AR	1.79173	1.683544	0.465984
W3	HPI4	well_2	F005	1421	Cell AR	1.709327	1.62037	0.426037
W3	HPI4	well_2	F006	1153	Cell AR	1.729622	1.614286	0.477337
W3	HPI4	well_3	F001	874	Cell AR	1.692909	1.59517	0.454501
W3	HPI4	well_3	F002	979	Cell AR	1.739902	1.630252	0.463543
W3	HPI4	well_3	F003	721	Cell AR	1.744907	1.659574	0.430436
W3	HPI4	well_3	F004	1130	Cell AR	1.702583	1.609265	0.431735
W3	HPI4	well_3	F005	1348	Cell AR	1.69621	1.585556	0.425952
W3	HPI4	well_3	F006	1161	Cell AR	1.707196	1.612245	0.421663

W3	HPI4	well_4	F001	815	Cell AR	1.710087	1.612903	0.433951
W3	HPI4	well_4	F002	994	Cell AR	1.737521	1.633498	0.45229
W3	HPI4	well_4	F003	827	Cell AR	1.750342	1.666667	0.438585
W3	HPI4	well_4	F004	853	Cell AR	1.706967	1.608108	0.426335
W3	HPI4	well_4	F005	1488	Cell AR	1.696979	1.607719	0.4208
W3	HPI4	well_4	F006	1371	Cell AR	1.725322	1.625	0.436287
W3	HPI4	well_5	F001	734	Cell AR	1.766131	1.656716	0.484309
W3	HPI4	well_5	F002	665	Cell AR	1.722368	1.630137	0.423482
W3	HPI4	well_5	F003	933	Cell AR	1.703135	1.615942	0.41382
W3	HPI4	well_5	F004	650	Cell AR	1.730577	1.630602	0.441189
W3	HPI4	well_5	F005	1139	Cell AR	1.64977	1.553191	0.398731
W3	HPI4	well_5	F006	1081	Cell AR	1.726312	1.612903	0.449337
W3	PGE2	well_1	F001	2916	Cell AR	1.543442	1.461538	0.343647
W3	PGE2	well_1	F002	3725	Cell AR	1.544471	1.468085	0.313829
W3	PGE2	well_1	F003	3755	Cell AR	1.534746	1.445946	0.334799
W3	PGE2	well_1	F004	3148	Cell AR	1.516112	1.436131	0.329625
W3	PGE2	well_1	F005	3514	Cell AR	1.549808	1.449127	0.374056
W3	PGE2	well_1	F006	3064	Cell AR	1.543755	1.464027	0.315669
W3	PGE2	well_2	F001	3084	Cell AR	1.61927	1.493054	0.43677
W3	PGE2	well_2	F002	3211	Cell AR	1.521523	1.440678	0.320186
W3	PGE2	well_2	F003	3552	Cell AR	1.5331	1.458082	0.319828
W3	PGE2	well_2	F004	3708	Cell AR	1.549612	1.458824	0.350975
W3	PGE2	well_2	F005	3916	Cell AR	1.557783	1.461538	0.38211
W3	PGE2	well_2	F006	4006	Cell AR	1.553574	1.461538	0.360037
W3	PGE2	well_3	F001	3250	Cell AR	1.606974	1.480891	0.442477
W3	PGE2	well_3	F002	2986	Cell AR	1.589289	1.487011	0.386111
W3	PGE2	well_3	F003	3965	Cell AR	1.559037	1.479167	0.339763
W3	PGE2	well_3	F004	3537	Cell AR	1.554811	1.464286	0.354746
W3	PGE2	well_3	F005	3998	Cell AR	1.511654	1.438356	0.297485
W3	PGE2	well_3	F006	3740	Cell AR	1.533496	1.454545	0.318526
W3	PGE2	well_4	F001	2792	Cell AR	1.529135	1.447761	0.323205
W3	PGE2	well_4	F002	3433	Cell AR	1.547877	1.461538	0.352434
W3	PGE2	well_4	F003	2859	Cell AR	1.536452	1.454545	0.338789
W3	PGE2	well_4	F004	3317	Cell AR	1.602132	1.493151	0.404844
W3	PGE2	well_4	F005	3655	Cell AR	1.546885	1.457627	0.339139
W3	PGE2	well_4	F006	3418	Cell AR	1.578827	1.484123	0.379829
W3	PGE2	well_5	F001	2468	Cell AR	1.577618	1.492537	0.355798
W3	PGE2	well_5	F002	2542	Cell AR	1.561885	1.460317	0.374305
W3	PGE2	well_5	F003	3154	Cell AR	1.549472	1.462963	0.34743
W3	PGE2	well_5	F004	2966	Cell AR	1.541243	1.448849	0.344341
W3	PGE2	well_5	F005	3566	Cell AR	1.54598	1.459016	0.341283
W3	PGE2	well_5	F006	2826	Cell AR	1.541054	1.45	0.358035
W4	HPI4	well_1	F001	600	Cell AR	1.808413	1.698082	0.496472
W4	HPI4	well_1	F002	975	Cell AR	1.759594	1.663934	0.468671
W4	HPI4	well_1	F003	928	Cell AR	1.731773	1.61416	0.472324
W4	HPI4	well_1	F004	1146	Cell AR	1.771015	1.675866	0.462999
W4	HPI4	well_1	F005	1258	Cell AR	1.714983	1.625	0.419803

W4	HPI4	well_1	F006	1063	Cell AR	1.792035	1.671875	0.495826
W4	HPI4	well_2	F001	745	Cell AR	1.760723	1.680556	0.453568
W4	HPI4	well_2	F002	1025	Cell AR	1.861833	1.72973	0.535857
W4	HPI4	well_2	F003	1060	Cell AR	1.7846	1.666667	0.473674
W4	HPI4	well_2	F004	1165	Cell AR	1.77451	1.662651	0.492579
W4	HPI4	well_2	F005	1104	Cell AR	1.717235	1.619211	0.429981
W4	HPI4	well_2	F006	1042	Cell AR	1.837403	1.722222	0.496476
W4	HPI4	well_3	F001	943	Cell AR	1.74259	1.666667	0.425626
W4	HPI4	well_3	F002	975	Cell AR	1.741594	1.642857	0.456983
W4	HPI4	well_3	F003	1033	Cell AR	1.763189	1.666667	0.455614
W4	HPI4	well_3	F004	1257	Cell AR	1.787855	1.7	0.467015
W4	HPI4	well_3	F005	1209	Cell AR	1.772188	1.692308	0.445124
W4	HPI4	well_3	F006	904	Cell AR	1.772	1.691608	0.458248
W4	HPI4	well_4	F001	906	Cell AR	1.741989	1.659333	0.431115
W4	HPI4	well_4	F002	1112	Cell AR	1.762722	1.655944	0.454204
W4	HPI4	well_4	F003	792	Cell AR	1.791606	1.687123	0.469012
W4	HPI4	well_4	F004	1199	Cell AR	1.733871	1.647887	0.453676
W4	HPI4	well_4	F005	1315	Cell AR	1.733427	1.612903	0.479762
W4	HPI4	well_4	F006	971	Cell AR	1.729902	1.643678	0.432599
W4	HPI4	well_5	F001	920	Cell AR	1.756944	1.644009	0.483647
W4	HPI4	well_5	F002	673	Cell AR	1.695192	1.604167	0.399722
W4	HPI4	well_5	F003	860	Cell AR	1.702845	1.627845	0.407807
W4	HPI4	well_5	F004	1099	Cell AR	1.743517	1.648936	0.452578
W4	HPI4	well_5	F005	1325	Cell AR	1.779148	1.676056	0.495063
W4	HPI4	well_5	F006	1116	Cell AR	1.823913	1.707879	0.531732
W4	PGE2	well_1	F001	2246	Cell AR	1.539569	1.464789	0.329111
W4	PGE2	well_1	F002	1936	Cell AR	1.549054	1.475079	0.323458
W4	PGE2	well_1	F003	2992	Cell AR	1.529275	1.4625	0.29626
W4	PGE2	well_1	F004	2919	Cell AR	1.496269	1.410714	0.322376
W4	PGE2	well_1	F005	3158	Cell AR	1.54113	1.449638	0.356965
W4	PGE2	well_1	F006	3063	Cell AR	1.514069	1.428571	0.326886
W4	PGE2	well_2	F001	2465	Cell AR	1.592021	1.5	0.357657
W4	PGE2	well_2	F002	3049	Cell AR	1.573303	1.471698	0.38057
W4	PGE2	well_2	F003	2809	Cell AR	1.581145	1.485714	0.357166
W4	PGE2	well_2	F004	3032	Cell AR	1.540969	1.469542	0.318034
W4	PGE2	well_2	F005	3481	Cell AR	1.549722	1.457831	0.349467
W4	PGE2	well_2	F006	3022	Cell AR	1.532911	1.462825	0.314493
W4	PGE2	well_3	F001	2445	Cell AR	1.570115	1.483871	0.345514
W4	PGE2	well_3	F002	3299	Cell AR	1.563874	1.484375	0.339743
W4	PGE2	well_3	F003	3466	Cell AR	1.565299	1.477936	0.353367
W4	PGE2	well_3	F004	3334	Cell AR	1.592873	1.48	0.408387
W4	PGE2	well_3	F005	2848	Cell AR	1.613113	1.5	0.430679
W4	PGE2	well_3	F006	3272	Cell AR	1.568229	1.476923	0.360645
W4	PGE2	well_4	F001	2332	Cell AR	1.618443	1.507246	0.410833
W4	PGE2	well_4	F002	2958	Cell AR	1.566569	1.480891	0.342518
W4	PGE2	well_4	F003	3074	Cell AR	1.59868	1.508197	0.374874
W4	PGE2	well_4	F004	3036	Cell AR	1.596577	1.492537	0.389789

W4	PGE2	well_4	F005	3745	Cell AR	1.522227	1.444444	0.317761
W4	PGE2	well_4	F006	2576	Cell AR	1.548407	1.464789	0.33407
W4	PGE2	well_5	F001	1990	Cell AR	1.569915	1.491803	0.346775
W4	PGE2	well_5	F002	2402	Cell AR	1.58281	1.473684	0.396245
W4	PGE2	well_5	F003	2893	Cell AR	1.506865	1.428571	0.317179
W4	PGE2	well_5	F004	2858	Cell AR	1.568817	1.467914	0.373638
W4	PGE2	well_5	F005	2650	Cell AR	1.566188	1.465517	0.374406
W4	PGE2	well_5	F006	2271	Cell AR	1.577683	1.46875	0.391419
W1	HPI4	well_1	F001	2006	Cell Area	293.5095	259.3446	149.2306
W1	HPI4	well_1	F002	1797	Cell Area	276.5639	244.7214	139.5146
W1	HPI4	well_1	F003	2325	Cell Area	255.2812	228.6194	127.7966
W1	HPI4	well_1	F004	2112	Cell Area	283.8109	257.1617	125.8026
W1	HPI4	well_1	F005	2475	Cell Area	262.5507	239.0411	117.7828
W1	HPI4	well_1	F006	1903	Cell Area	284.1528	254.7206	143.4213
W1	HPI4	well_2	F001	1728	Cell Area	282.8113	246.5288	146.1285
W1	HPI4	well_2	F002	2072	Cell Area	287.8158	259.6967	133.3503
W1	HPI4	well_2	F003	2175	Cell Area	280.6857	249.8853	135.8768
W1	HPI4	well_2	F004	2271	Cell Area	282.1024	255.7064	126.0344
W1	HPI4	well_2	F005	2464	Cell Area	268.7757	245.449	125.6918
W1	HPI4	well_2	F006	2103	Cell Area	276.6807	243.7825	139.6691
W1	HPI4	well_3	F001	1564	Cell Area	301.0126	266.8322	148.0265
W1	HPI4	well_3	F002	1898	Cell Area	283.2985	260.1192	124.1519
W1	HPI4	well_3	F003	1917	Cell Area	283.2905	252.8897	142.0565
W1	HPI4	well_3	F004	2384	Cell Area	264.9999	241.7404	119.6011
W1	HPI4	well_3	F005	2264	Cell Area	267.281	239.9096	130.1916
W1	HPI4	well_3	F006	2274	Cell Area	266.9227	242.2099	126.9557
W1	HPI4	well_4	F001	1973	Cell Area	267.0634	238.4778	135.5281
W1	HPI4	well_4	F002	2276	Cell Area	254.2477	233.5486	115.538
W1	HPI4	well_4	F003	2401	Cell Area	262.1525	237.3981	123.4504
W1	HPI4	well_4	F004	2109	Cell Area	273.4687	247.9606	132.4944
W1	HPI4	well_4	F005	2355	Cell Area	272.1569	244.6744	134.5265
W1	HPI4	well_4	F006	2283	Cell Area	266.6601	239.6044	129.0461
W1	HPI4	well_5	F001	2187	Cell Area	270.5686	238.4778	142.2062
W1	HPI4	well_5	F002	2196	Cell Area	263.8402	233.5017	131.6594
W1	HPI4	well_5	F003	2472	Cell Area	254.6644	230.4972	124.4253
W1	HPI4	well_5	F004	2165	Cell Area	252.5204	225.0986	127.3609
W1	HPI4	well_5	F005	2000	Cell Area	284.1305	254.0633	140.0503
W1	HPI4	well_5	F006	1889	Cell Area	280.1601	245.5194	144.8324
W1	PGE2	well_1	F001	2346	Cell Area	274.8253	243.1722	134.0043
W1	PGE2	well_1	F002	2124	Cell Area	295.0486	262.0908	146.0485
W1	PGE2	well_1	F003	2450	Cell Area	280.5056	258.2179	125.9749
W1	PGE2	well_1	F004	2589	Cell Area	263.2656	235.6611	127.7821
W1	PGE2	well_1	F005	2970	Cell Area	247.8928	220.4746	120.78
W1	PGE2	well_1	F006	2939	Cell Area	256.332	226.8356	125.0371
W1	PGE2	well_2	F001	2002	Cell Area	273.5365	248.1953	131.3481
W1	PGE2	well_2	F002	2113	Cell Area	267.3368	243.7356	120.9826
W1	PGE2	well_2	F003	2227	Cell Area	269.539	234.8161	134.9976

W1	PGE2	well_2	F004	2400	Cell Area	269.207	240.0974	129.3284
W1	PGE2	well_2	F005	2511	Cell Area	273.5676	250.4956	119.8054
W1	PGE2	well_2	F006	1903	Cell Area	285.6005	254.9083	135.3346
W1	PGE2	well_3	F001	2114	Cell Area	285.4512	257.725	132.3082
W1	PGE2	well_3	F002	2013	Cell Area	284.1932	259.6497	132.5938
W1	PGE2	well_3	F003	2208	Cell Area	276.6937	247.3503	132.2089
W1	PGE2	well_3	F004	2146	Cell Area	289.0254	264.3676	133.7957
W1	PGE2	well_3	F005	2403	Cell Area	276.3969	239.8392	147.4526
W1	PGE2	well_3	F006	2147	Cell Area	285.1277	254.1572	138.3528
W1	PGE2	well_4	F001	1891	Cell Area	294.8956	272.2308	134.7365
W1	PGE2	well_4	F002	1986	Cell Area	275.0174	250.3547	119.6309
W1	PGE2	well_4	F003	1905	Cell Area	282.3191	253.7347	134.0748
W1	PGE2	well_4	F004	2397	Cell Area	261.4376	232.8914	126.3198
W1	PGE2	well_4	F005	2079	Cell Area	284.7685	258.8517	138.4244
W1	PGE2	well_4	F006	2134	Cell Area	274.9029	245.5194	130.8862
W1	PGE2	well_5	F001	1894	Cell Area	290.5578	254.6267	150.8049
W1	PGE2	well_5	F002	2205	Cell Area	281.8568	255.0492	136.3546
W1	PGE2	well_5	F003	1891	Cell Area	292.2864	268.5222	129.1658
W1	PGE2	well_5	F004	2105	Cell Area	289.0998	260.5886	138.3949
W1	PGE2	well_5	F005	2176	Cell Area	273.9511	249.4158	125.1403
W1	PGE2	well_5	F006	1968	Cell Area	272.3144	247.0217	121.9797
W2	HPI4	well_1	F001	1889	Cell Area	280.7239	258.3822	131.0046
W2	HPI4	well_1	F002	2032	Cell Area	292.7426	260.9876	150.5679
W2	HPI4	well_1	F003	2276	Cell Area	255.7758	223.8546	137.9574
W2	HPI4	well_1	F004	2401	Cell Area	260.8593	232.7506	133.9843
W2	HPI4	well_1	F005	1985	Cell Area	294.8984	271.6206	143.5327
W2	HPI4	well_1	F006	1738	Cell Area	295.6178	272.9585	142.9048
W2	HPI4	well_2	F001	1684	Cell Area	285.8948	257.9597	136.332
W2	HPI4	well_2	F002	1846	Cell Area	290.8148	265.4239	138.8709
W2	HPI4	well_2	F003	2013	Cell Area	269.8443	243.6417	132.7067
W2	HPI4	well_2	F004	2491	Cell Area	255.013	226.7886	129.7815
W2	HPI4	well_2	F005	2106	Cell Area	281.0961	252.8193	134.925
W2	HPI4	well_2	F006	1974	Cell Area	272.5281	250.9415	132.1598
W2	HPI4	well_3	F001	1972	Cell Area	266.6883	242.914	125.3258
W2	HPI4	well_3	F002	1767	Cell Area	288.6138	261.6683	142.5776
W2	HPI4	well_3	F003	1332	Cell Area	312.6394	282.9576	168.2459
W2	HPI4	well_3	F004	2224	Cell Area	265.8684	243.8764	123.343
W2	HPI4	well_3	F005	2099	Cell Area	268.4062	242.515	131.2925
W2	HPI4	well_3	F006	2048	Cell Area	271.685	245.3786	135.5181
W2	HPI4	well_4	F001	1980	Cell Area	253.7414	230.1921	120.4285
W2	HPI4	well_4	F002	1990	Cell Area	242.4746	216.8833	121.932
W2	HPI4	well_4	F003	2045	Cell Area	259.5841	238.6656	125.507
W2	HPI4	well_4	F004	2328	Cell Area	244.0738	217.5406	123.7635
W2	HPI4	well_4	F005	1893	Cell Area	260.6917	234.2528	131.6957
W2	HPI4	well_4	F006	1970	Cell Area	274.5239	234.2528	150.2649
W2	HPI4	well_5	F001	1814	Cell Area	269.8828	243.6886	130.0876
W2	HPI4	well_5	F002	1701	Cell Area	294.7834	265.5178	150.7005

W2	HPI4	well_5	F003	1841	Cell Area	274.6379	249.1342	138.1062
W2	HPI4	well_5	F004	2312	Cell Area	249.945	227.0938	120.5749
W2	HPI4	well_5	F005	2140	Cell Area	261.2264	240.0974	127.9384
W2	HPI4	well_5	F006	1915	Cell Area	260.89	236.1306	130.5594
W2	PGE2	well_1	F001	2508	Cell Area	209.2774	194.2326	86.56842
W2	PGE2	well_1	F002	2196	Cell Area	195.5486	181.276	85.8128
W2	PGE2	well_1	F003	3078	Cell Area	209.102	196.5799	83.37474
W2	PGE2	well_1	F004	2519	Cell Area	203.8991	189.1861	84.02477
W2	PGE2	well_1	F005	3120	Cell Area	200.8896	187.6135	80.36923
W2	PGE2	well_1	F006	3092	Cell Area	187.8207	175.5957	75.66787
W2	PGE2	well_2	F001	2524	Cell Area	192.7286	182.8486	74.42172
W2	PGE2	well_2	F002	3220	Cell Area	181.8535	170.1971	69.88883
W2	PGE2	well_2	F003	3754	Cell Area	189.6471	178.8114	74.06341
W2	PGE2	well_2	F004	3308	Cell Area	189.8428	177.3561	78.39762
W2	PGE2	well_2	F005	2732	Cell Area	195.0827	181.3699	82.48008
W2	PGE2	well_2	F006	2641	Cell Area	196.3065	187.4492	73.8228
W2	PGE2	well_3	F001	2301	Cell Area	192.6164	175.1028	89.77242
W2	PGE2	well_3	F002	3625	Cell Area	192.4229	177.7786	77.28633
W2	PGE2	well_3	F003	3166	Cell Area	201.9416	188.1768	81.94986
W2	PGE2	well_3	F004	3460	Cell Area	178.9575	167.2396	73.94576
W2	PGE2	well_3	F005	2810	Cell Area	187.9396	175.7131	73.53081
W2	PGE2	well_3	F006	2454	Cell Area	193.6139	179.5625	79.44156
W2	PGE2	well_4	F001	2778	Cell Area	207.6047	191.1108	87.70662
W2	PGE2	well_4	F002	3296	Cell Area	189.2659	173.5067	76.80955
W2	PGE2	well_4	F003	3146	Cell Area	194.3561	181.6046	74.49487
W2	PGE2	well_4	F004	3610	Cell Area	201.3498	186.1817	87.83635
W2	PGE2	well_4	F005	3154	Cell Area	194.232	177.8256	80.45642
W2	PGE2	well_4	F006	2634	Cell Area	201.1252	185.1958	83.03106
W2	PGE2	well_5	F001	3092	Cell Area	197.8109	180.0319	85.96055
W2	PGE2	well_5	F002	3496	Cell Area	200.6449	186.8389	75.59446
W2	PGE2	well_5	F003	3256	Cell Area	201.3402	189.4443	75.79397
W2	PGE2	well_5	F004	2836	Cell Area	188.2048	177.1683	72.66245
W2	PGE2	well_5	F005	2895	Cell Area	187.8942	177.4031	69.91617
W2	PGE2	well_5	F006	3259	Cell Area	187.3084	173.4128	76.66969
W3	HPI4	well_1	F001	876	Cell Area	334.2032	289.9993	173.552
W3	HPI4	well_1	F002	1131	Cell Area	319.3044	283.6853	161.797
W3	HPI4	well_1	F003	933	Cell Area	338.1782	303.7775	168.8546
W3	HPI4	well_1	F004	1484	Cell Area	274.411	248.6882	135.2311
W3	HPI4	well_1	F005	1385	Cell Area	310.9502	284.1078	145.1784
W3	HPI4	well_1	F006	955	Cell Area	341.3174	314.34	158.7443
W3	HPI4	well_2	F001	819	Cell Area	330.4552	299.8811	165.4674
W3	HPI4	well_2	F002	1035	Cell Area	329.077	308.6597	141.9772
W3	HPI4	well_2	F003	1013	Cell Area	336.1548	301.2894	181.6457
W3	HPI4	well_2	F004	1327	Cell Area	301.4749	281.1972	140.9665
W3	HPI4	well_2	F005	1421	Cell Area	310.3725	281.6667	153.1389
W3	HPI4	well_2	F006	1153	Cell Area	333.9308	306.6411	164.2521
W3	HPI4	well_3	F001	874	Cell Area	327.8421	305.045	169.7731

W3	HPI4	well_3	F002	979	Cell Area	344.5288	326.6864	155.1651
W3	HPI4	well_3	F003	721	Cell Area	336.2364	308.6128	168.9022
W3	HPI4	well_3	F004	1130	Cell Area	300.8022	275.7282	148.8355
W3	HPI4	well_3	F005	1348	Cell Area	282.1103	250.7772	148.3754
W3	HPI4	well_3	F006	1161	Cell Area	303.2717	265.6117	164.6925
W3	HPI4	well_4	F001	815	Cell Area	322.8955	304.4347	166.7175
W3	HPI4	well_4	F002	994	Cell Area	344.4501	309.2935	166.5056
W3	HPI4	well_4	F003	827	Cell Area	339.6336	309.3639	168.755
W3	HPI4	well_4	F004	853	Cell Area	321.5541	288.5675	149.8004
W3	HPI4	well_4	F005	1488	Cell Area	289.2074	259.0864	145.8754
W3	HPI4	well_4	F006	1371	Cell Area	295.8713	264.8606	146.0838
W3	HPI4	well_5	F001	734	Cell Area	362.1131	332.3667	174.6985
W3	HPI4	well_5	F002	665	Cell Area	327.3123	303.5897	157.3527
W3	HPI4	well_5	F003	933	Cell Area	315.0881	284.9058	158.9814
W3	HPI4	well_5	F004	650	Cell Area	325.4753	298.7075	170.8618
W3	HPI4	well_5	F005	1139	Cell Area	320.0433	294.4356	158.912
W3	HPI4	well_5	F006	1081	Cell Area	318.6895	285.8447	161.1906
W3	PGE2	well_1	F001	2916	Cell Area	214.7406	197.8004	87.37637
W3	PGE2	well_1	F002	3725	Cell Area	204.8277	191.4394	79.39845
W3	PGE2	well_1	F003	3755	Cell Area	207.9199	192.9417	79.42591
W3	PGE2	well_1	F004	3148	Cell Area	198.64	175.314	89.82522
W3	PGE2	well_1	F005	3514	Cell Area	206.9269	185.5714	86.76149
W3	PGE2	well_1	F006	3064	Cell Area	223.3419	203.2929	96.05645
W3	PGE2	well_2	F001	3084	Cell Area	221.2144	202.1428	86.21225
W3	PGE2	well_2	F002	3211	Cell Area	218.6564	196.0869	94.60606
W3	PGE2	well_2	F003	3552	Cell Area	210.5805	193.1294	85.10072
W3	PGE2	well_2	F004	3708	Cell Area	210.0008	193.505	84.23103
W3	PGE2	well_2	F005	3916	Cell Area	213.7781	193.5989	88.7248
W3	PGE2	well_2	F006	4006	Cell Area	211.4803	196.2747	76.1686
W3	PGE2	well_3	F001	3250	Cell Area	218.3502	191.9793	101.5517
W3	PGE2	well_3	F002	2986	Cell Area	221.683	203.3868	91.36827
W3	PGE2	well_3	F003	3965	Cell Area	208.7951	185.7592	90.58201
W3	PGE2	well_3	F004	3537	Cell Area	214.5302	187.6839	97.81319
W3	PGE2	well_3	F005	3998	Cell Area	208.8273	189.5851	86.54628
W3	PGE2	well_3	F006	3740	Cell Area	220.4166	201.1804	87.02237
W3	PGE2	well_4	F001	2792	Cell Area	214.5619	194.8664	86.62805
W3	PGE2	well_4	F002	3433	Cell Area	214.1895	192.1906	90.7361
W3	PGE2	well_4	F003	2859	Cell Area	216.2926	198.0586	84.78959
W3	PGE2	well_4	F004	3317	Cell Area	222.8757	198.7628	99.38937
W3	PGE2	well_4	F005	3655	Cell Area	212.8535	195.0072	83.54255
W3	PGE2	well_4	F006	3418	Cell Area	218.571	198.2699	88.76647
W3	PGE2	well_5	F001	2468	Cell Area	219.81	196.1808	100.3211
W3	PGE2	well_5	F002	2542	Cell Area	232.3896	208.4803	102.1634
W3	PGE2	well_5	F003	3154	Cell Area	212.8798	190.8526	86.83815
W3	PGE2	well_5	F004	2966	Cell Area	208.5146	185.3601	95.91693
W3	PGE2	well_5	F005	3566	Cell Area	203.2627	184.656	84.52769
W3	PGE2	well_5	F006	2826	Cell Area	222.3979	197.8708	95.67426

W4	HPI4	well_1	F001	600	Cell Area	373.8276	342.2719	168.4054
W4	HPI4	well_1	F002	975	Cell Area	335.9154	310.6314	151.8212
W4	HPI4	well_1	F003	928	Cell Area	321.9909	291.7363	147.3218
W4	HPI4	well_1	F004	1146	Cell Area	332.4326	312.5326	146.4512
W4	HPI4	well_1	F005	1258	Cell Area	310.9692	272.3951	170.9163
W4	HPI4	well_1	F006	1063	Cell Area	332.2593	299.5056	168.7004
W4	HPI4	well_2	F001	745	Cell Area	389.2303	363.6786	165.3229
W4	HPI4	well_2	F002	1025	Cell Area	361.086	338.3756	160.1578
W4	HPI4	well_2	F003	1060	Cell Area	345.5866	312.5326	169.4877
W4	HPI4	well_2	F004	1165	Cell Area	339.794	309.0353	161.6044
W4	HPI4	well_2	F005	1104	Cell Area	322.3831	297.0175	155.5045
W4	HPI4	well_2	F006	1042	Cell Area	335.7543	310.3732	147.8637
W4	HPI4	well_3	F001	943	Cell Area	351.6992	327.9539	169.3255
W4	HPI4	well_3	F002	975	Cell Area	363.1464	336.4039	168.033
W4	HPI4	well_3	F003	1033	Cell Area	368.3121	338	174.4478
W4	HPI4	well_3	F004	1257	Cell Area	318.7689	297.6747	147.8649
W4	HPI4	well_3	F005	1209	Cell Area	319.1213	299.6933	139.303
W4	HPI4	well_3	F006	904	Cell Area	337.2084	303.2846	172.5061
W4	HPI4	well_4	F001	906	Cell Area	373.1647	339.3614	181.0318
W4	HPI4	well_4	F002	1112	Cell Area	355.6626	328.9632	161.4699
W4	HPI4	well_4	F003	792	Cell Area	391.2643	360.7915	186.3768
W4	HPI4	well_4	F004	1199	Cell Area	344.6837	318.1425	171.0753
W4	HPI4	well_4	F005	1315	Cell Area	307.4052	280.2114	150.3702
W4	HPI4	well_4	F006	971	Cell Area	366.8371	336.31	176.4341
W4	HPI4	well_5	F001	920	Cell Area	400.3913	375.579	177.9461
W4	HPI4	well_5	F002	673	Cell Area	393.8486	362.6928	185.7115
W4	HPI4	well_5	F003	860	Cell Area	382.4745	343.3517	191.6713
W4	HPI4	well_5	F004	1099	Cell Area	372.6819	339.8778	190.3193
W4	HPI4	well_5	F005	1325	Cell Area	334.9992	306.7819	154.5254
W4	HPI4	well_5	F006	1116	Cell Area	343.1536	319.011	153.6278
W4	PGE2	well_1	F001	2246	Cell Area	230.9538	216.1557	84.80788
W4	PGE2	well_1	F002	1936	Cell Area	234.1623	219.9817	86.15034
W4	PGE2	well_1	F003	2992	Cell Area	227.3016	212.2828	83.18291
W4	PGE2	well_1	F004	2919	Cell Area	217.0852	192.6131	88.69195
W4	PGE2	well_1	F005	3158	Cell Area	223.2404	205.2176	91.7357
W4	PGE2	well_1	F006	3063	Cell Area	222.701	207.3067	79.55992
W4	PGE2	well_2	F001	2465	Cell Area	241.2101	226.7417	89.23477
W4	PGE2	well_2	F002	3049	Cell Area	225.8033	206.1331	91.63911
W4	PGE2	well_2	F003	2809	Cell Area	225.4359	204.2083	90.32384
W4	PGE2	well_2	F004	3032	Cell Area	222.5831	202.8235	85.62185
W4	PGE2	well_2	F005	3481	Cell Area	212.586	194.5378	80.45688
W4	PGE2	well_2	F006	3022	Cell Area	231.2344	210.7101	90.1333
W4	PGE2	well_3	F001	2445	Cell Area	233.3401	219.2775	86.75549
W4	PGE2	well_3	F002	3299	Cell Area	220.627	205.6636	82.35189
W4	PGE2	well_3	F003	3466	Cell Area	216.9246	196.9085	83.04463
W4	PGE2	well_3	F004	3334	Cell Area	214.358	192.3783	86.4844
W4	PGE2	well_3	F005	2848	Cell Area	229.4296	206.3678	94.68993

W4	PGE2	well_3	F006	3272	Cell Area	223.1271	205.0299	85.49522
W4	PGE2	well_4	F001	2332	Cell Area	215.2333	198.8567	81.41027
W4	PGE2	well_4	F002	2958	Cell Area	219.2146	202.8235	83.40517
W4	PGE2	well_4	F003	3074	Cell Area	220.6308	203.3868	79.53405
W4	PGE2	well_4	F004	3036	Cell Area	211.6066	193.4581	80.37637
W4	PGE2	well_4	F005	3745	Cell Area	213.5992	199.6547	76.27509
W4	PGE2	well_4	F006	2576	Cell Area	223.7563	207.3771	81.95633
W4	PGE2	well_5	F001	1990	Cell Area	228.4455	210.6867	92.44372
W4	PGE2	well_5	F002	2402	Cell Area	221.163	195.7818	94.8444
W4	PGE2	well_5	F003	2893	Cell Area	200.412	183.4119	75.39716
W4	PGE2	well_5	F004	2858	Cell Area	211.8516	188.0829	90.40325
W4	PGE2	well_5	F005	2650	Cell Area	213.3584	196.1574	82.28596
W4	PGE2	well_5	F006	2271	Cell Area	228.7256	204.2083	101.5215
W1	HPI4	well_1	F001	2006	Cell Height	4.476321	4.5	1.875173
W1	HPI4	well_1	F002	1797	Cell Height	4.409015	4.5	1.85219
W1	HPI4	well_1	F003	2325	Cell Height	4.751398	4.5	1.95829
W1	HPI4	well_1	F004	2112	Cell Height	4.702178	4.5	1.900681
W1	HPI4	well_1	F005	2475	Cell Height	4.812323	5	1.900897
W1	HPI4	well_1	F006	1903	Cell Height	4.60536	4.5	1.978409
W1	HPI4	well_2	F001	1728	Cell Height	4.298322	4	1.803934
W1	HPI4	well_2	F002	2072	Cell Height	4.421573	4.5	1.784842
W1	HPI4	well_2	F003	2175	Cell Height	4.502529	4.5	1.871288
W1	HPI4	well_2	F004	2271	Cell Height	4.667327	4.5	2.040387
W1	HPI4	well_2	F005	2464	Cell Height	4.82569	4.5	1.976351
W1	HPI4	well_2	F006	2103	Cell Height	4.971945	5	2.077855
W1	HPI4	well_3	F001	1564	Cell Height	4.200448	4	1.830101
W1	HPI4	well_3	F002	1898	Cell Height	4.567966	4.5	1.885317
W1	HPI4	well_3	F003	1917	Cell Height	4.617632	4.5	1.856437
W1	HPI4	well_3	F004	2384	Cell Height	4.69505	4.5	2.021631
W1	HPI4	well_3	F005	2264	Cell Height	4.599823	4.5	1.892252
W1	HPI4	well_3	F006	2274	Cell Height	4.753298	4.5	2.008736
W1	HPI4	well_4	F001	1973	Cell Height	4.469336	4.5	2.028186
W1	HPI4	well_4	F002	2276	Cell Height	4.607206	4.5	1.884855
W1	HPI4	well_4	F003	2401	Cell Height	4.567888	4.5	2.028026
W1	HPI4	well_4	F004	2109	Cell Height	4.328118	4.5	1.758005
W1	HPI4	well_4	F005	2355	Cell Height	4.513163	4.5	1.917599
W1	HPI4	well_4	F006	2283	Cell Height	4.458388	4.5	1.796145
W1	HPI4	well_5	F001	2187	Cell Height	4.327846	4	1.988408
W1	HPI4	well_5	F002	2196	Cell Height	4.45674	4.5	1.867342
W1	HPI4	well_5	F003	2472	Cell Height	4.822411	5	2.142773
W1	HPI4	well_5	F004	2165	Cell Height	4.638106	4.5	2.079375
W1	HPI4	well_5	F005	2000	Cell Height	4.29125	4	1.785957
W1	HPI4	well_5	F006	1889	Cell Height	4.359185	4.5	1.842162
W1	PGE2	well_1	F001	2346	Cell Height	4.768755	4.5	1.959755
W1	PGE2	well_1	F002	2124	Cell Height	4.553672	4.5	1.929807
W1	PGE2	well_1	F003	2450	Cell Height	4.729388	4.5	2.075404
W1	PGE2	well_1	F004	2589	Cell Height	4.818463	4.5	2.041463

W1	PGE2	well_1	F005	2970	Cell Height	5.019697	5	2.147131
W1	PGE2	well_1	F006	2939	Cell Height	4.909153	5	2.119133
W1	PGE2	well_2	F001	2002	Cell Height	4.634116	4.5	1.813347
W1	PGE2	well_2	F002	2113	Cell Height	4.750355	5	1.949172
W1	PGE2	well_2	F003	2227	Cell Height	4.582847	4.5	1.863245
W1	PGE2	well_2	F004	2400	Cell Height	4.8025	5	2.015869
W1	PGE2	well_2	F005	2511	Cell Height	4.815014	5	2.006148
W1	PGE2	well_2	F006	1903	Cell Height	4.67341	4.5	1.953155
W1	PGE2	well_3	F001	2114	Cell Height	4.631977	4.5	1.817473
W1	PGE2	well_3	F002	2013	Cell Height	4.632886	4.5	1.85411
W1	PGE2	well_3	F003	2208	Cell Height	4.746377	4.5	1.917413
W1	PGE2	well_3	F004	2146	Cell Height	4.555452	4.5	1.872684
W1	PGE2	well_3	F005	2403	Cell Height	4.704744	4.5	2.072908
W1	PGE2	well_3	F006	2147	Cell Height	4.766884	4.5	2.053124
W1	PGE2	well_4	F001	1891	Cell Height	4.490746	4.5	1.94053
W1	PGE2	well_4	F002	1986	Cell Height	4.613797	4.5	1.796411
W1	PGE2	well_4	F003	1905	Cell Height	4.492651	4.5	1.924848
W1	PGE2	well_4	F004	2397	Cell Height	4.856279	5	2.084929
W1	PGE2	well_4	F005	2079	Cell Height	4.581289	4.5	1.99129
W1	PGE2	well_4	F006	2134	Cell Height	4.836223	5	2.058282
W1	PGE2	well_5	F001	1894	Cell Height	4.464097	4.5	1.854031
W1	PGE2	well_5	F002	2205	Cell Height	4.614966	4.5	1.995299
W1	PGE2	well_5	F003	1891	Cell Height	4.478054	4.5	1.924135
W1	PGE2	well_5	F004	2105	Cell Height	4.567933	4.5	1.930443
W1	PGE2	well_5	F005	2176	Cell Height	4.564338	4.5	1.90281
W1	PGE2	well_5	F006	1968	Cell Height	4.617886	4.5	2.011046
W2	HPI4	well_1	F001	1889	Cell Height	3.950503	4	1.6988
W2	HPI4	well_1	F002	2032	Cell Height	4.115896	4	1.711742
W2	HPI4	well_1	F003	2276	Cell Height	4.068981	3.5	1.924529
W2	HPI4	well_1	F004	2401	Cell Height	4.205956	4	2.014934
W2	HPI4	well_1	F005	1985	Cell Height	3.982368	4	1.767964
W2	HPI4	well_1	F006	1738	Cell Height	3.842635	3.5	1.616465
W2	HPI4	well_2	F001	1684	Cell Height	3.829869	3.5	1.601675
W2	HPI4	well_2	F002	1846	Cell Height	4.16468	4	1.791948
W2	HPI4	well_2	F003	2013	Cell Height	4.083706	4	1.896704
W2	HPI4	well_2	F004	2491	Cell Height	4.130068	4	1.832697
W2	HPI4	well_2	F005	2106	Cell Height	4.144112	4	1.850276
W2	HPI4	well_2	F006	1974	Cell Height	4.213019	4	1.913708
W2	HPI4	well_3	F001	1972	Cell Height	3.929767	4	1.665376
W2	HPI4	well_3	F002	1767	Cell Height	4.120826	4	1.77563
W2	HPI4	well_3	F003	1332	Cell Height	3.577703	3.5	1.576668
W2	HPI4	well_3	F004	2224	Cell Height	4.136915	4	1.891877
W2	HPI4	well_3	F005	2099	Cell Height	4.249404	4	1.987111
W2	HPI4	well_3	F006	2048	Cell Height	4.154541	4	1.932083
W2	HPI4	well_4	F001	1980	Cell Height	3.879798	3.5	1.651693
W2	HPI4	well_4	F002	1990	Cell Height	4.118342	4	1.887934
W2	HPI4	well_4	F003	2045	Cell Height	3.860391	3.5	1.635721

W2	HPI4	well_4	F004	2328	Cell Height	4.07195	4	1.869932
W2	HPI4	well_4	F005	1893	Cell Height	3.874802	3.5	1.660196
W2	HPI4	well_4	F006	1970	Cell Height	4.236294	4	1.944997
W2	HPI4	well_5	F001	1814	Cell Height	3.799614	3.5	1.632781
W2	HPI4	well_5	F002	1701	Cell Height	3.945032	3.5	1.74037
W2	HPI4	well_5	F003	1841	Cell Height	3.980989	3.5	1.809972
W2	HPI4	well_5	F004	2312	Cell Height	4.095156	4	1.868232
W2	HPI4	well_5	F005	2140	Cell Height	4.074065	4	1.890221
W2	HPI4	well_5	F006	1915	Cell Height	3.948825	4	1.692457
W2	PGE2	well_1	F001	2508	Cell Height	5.791467	5.5	2.695188
W2	PGE2	well_1	F002	2196	Cell Height	5.905738	6	2.574493
W2	PGE2	well_1	F003	3078	Cell Height	5.837232	6	2.500371
W2	PGE2	well_1	F004	2519	Cell Height	5.383089	5.5	2.271077
W2	PGE2	well_1	F005	3120	Cell Height	5.504487	5.5	2.304451
W2	PGE2	well_1	F006	3092	Cell Height	6.051746	6	2.604821
W2	PGE2	well_2	F001	2524	Cell Height	5.978011	6	2.404706
W2	PGE2	well_2	F002	3220	Cell Height	6.133851	6	2.65421
W2	PGE2	well_2	F003	3754	Cell Height	5.837906	6	2.380483
W2	PGE2	well_2	F004	3308	Cell Height	5.892684	6	2.348552
W2	PGE2	well_2	F005	2732	Cell Height	6.019949	6.5	2.462473
W2	PGE2	well_2	F006	2641	Cell Height	5.639909	5.5	2.318368
W2	PGE2	well_3	F001	2301	Cell Height	5.896567	6	2.617614
W2	PGE2	well_3	F002	3625	Cell Height	5.916	6	2.522261
W2	PGE2	well_3	F003	3166	Cell Height	6.072489	6	2.382739
W2	PGE2	well_3	F004	3460	Cell Height	6.045665	6	2.551764
W2	PGE2	well_3	F005	2810	Cell Height	5.921352	6	2.565283
W2	PGE2	well_3	F006	2454	Cell Height	5.903219	6	2.567734
W2	PGE2	well_4	F001	2778	Cell Height	5.898488	6	2.334155
W2	PGE2	well_4	F002	3296	Cell Height	6.439775	7	2.369495
W2	PGE2	well_4	F003	3146	Cell Height	6.135569	6.5	2.384331
W2	PGE2	well_4	F004	3610	Cell Height	5.922853	6	2.323651
W2	PGE2	well_4	F005	3154	Cell Height	6.412651	6.5	2.618773
W2	PGE2	well_4	F006	2634	Cell Height	6.150152	6.5	2.535346
W2	PGE2	well_5	F001	3092	Cell Height	6.051746	6	2.477085
W2	PGE2	well_5	F002	3496	Cell Height	5.952803	6	2.241003
W2	PGE2	well_5	F003	3256	Cell Height	5.755068	6	2.303001
W2	PGE2	well_5	F004	2836	Cell Height	6.046721	6.5	2.225201
W2	PGE2	well_5	F005	2895	Cell Height	6.306045	6.5	2.398337
W2	PGE2	well_5	F006	3259	Cell Height	6.128874	6.5	2.404363
W3	HPI4	well_1	F001	876	Cell Height	3.900114	3.5	1.812499
W3	HPI4	well_1	F002	1131	Cell Height	3.720159	3.5	1.634847
W3	HPI4	well_1	F003	933	Cell Height	3.424973	3.5	1.42677
W3	HPI4	well_1	F004	1484	Cell Height	3.544474	3.5	1.529231
W3	HPI4	well_1	F005	1385	Cell Height	3.754874	3.5	1.717815
W3	HPI4	well_1	F006	955	Cell Height	3.533508	3.5	1.622021
W3	HPI4	well_2	F001	819	Cell Height	3.53663	3.5	1.598212
W3	HPI4	well_2	F002	1035	Cell Height	3.637198	3.5	1.60437

W3	HPI4	well_2	F003	1013	Cell Height	3.679171	3.5	1.613686
W3	HPI4	well_2	F004	1327	Cell Height	3.828561	3.5	1.709645
W3	HPI4	well_2	F005	1421	Cell Height	3.590781	3.5	1.545163
W3	HPI4	well_2	F006	1153	Cell Height	3.777103	3.5	1.710673
W3	HPI4	well_3	F001	874	Cell Height	3.747712	3.5	1.726128
W3	HPI4	well_3	F002	979	Cell Height	3.686415	3.5	1.553816
W3	HPI4	well_3	F003	721	Cell Height	3.527739	3.5	1.453657
W3	HPI4	well_3	F004	1130	Cell Height	3.319912	3	1.414246
W3	HPI4	well_3	F005	1348	Cell Height	3.800816	3.5	1.770577
W3	HPI4	well_3	F006	1161	Cell Height	3.860896	3.5	1.74452
W3	HPI4	well_4	F001	815	Cell Height	3.290798	3	1.444545
W3	HPI4	well_4	F002	994	Cell Height	3.318913	3	1.3292
W3	HPI4	well_4	F003	827	Cell Height	3.288996	3	1.411827
W3	HPI4	well_4	F004	853	Cell Height	3.361665	3	1.339704
W3	HPI4	well_4	F005	1488	Cell Height	3.813508	3.5	1.660997
W3	HPI4	well_4	F006	1371	Cell Height	3.911014	3.5	1.770221
W3	HPI4	well_5	F001	734	Cell Height	3.379428	3	1.507181
W3	HPI4	well_5	F002	665	Cell Height	3.223308	3	1.389553
W3	HPI4	well_5	F003	933	Cell Height	3.612004	3.5	1.530816
W3	HPI4	well_5	F004	650	Cell Height	3.262308	3	1.419394
W3	HPI4	well_5	F005	1139	Cell Height	3.649693	3.5	1.663663
W3	HPI4	well_5	F006	1081	Cell Height	3.666975	3.5	1.675286
W3	PGE2	well_1	F001	2916	Cell Height	5.628944	6	2.404542
W3	PGE2	well_1	F002	3725	Cell Height	5.633423	5.5	2.463547
W3	PGE2	well_1	F003	3755	Cell Height	5.763382	6	2.437444
W3	PGE2	well_1	F004	3148	Cell Height	6.327827	6.5	2.669734
W3	PGE2	well_1	F005	3514	Cell Height	5.96144	6	2.636106
W3	PGE2	well_1	F006	3064	Cell Height	5.631038	5.5	2.314036
W3	PGE2	well_2	F001	3084	Cell Height	5.540532	5.5	2.548169
W3	PGE2	well_2	F002	3211	Cell Height	5.858144	5.5	2.660203
W3	PGE2	well_2	F003	3552	Cell Height	5.803632	5.5	2.494025
W3	PGE2	well_2	F004	3708	Cell Height	5.717638	5.5	2.493388
W3	PGE2	well_2	F005	3916	Cell Height	5.747063	5.5	2.384053
W3	PGE2	well_2	F006	4006	Cell Height	5.952197	6	2.326158
W3	PGE2	well_3	F001	3250	Cell Height	5.686154	5.5	2.567307
W3	PGE2	well_3	F002	2986	Cell Height	5.09645	5	2.404297
W3	PGE2	well_3	F003	3965	Cell Height	5.895586	6	2.52449
W3	PGE2	well_3	F004	3537	Cell Height	5.855386	6	2.502475
W3	PGE2	well_3	F005	3998	Cell Height	6.046898	6	2.51617
W3	PGE2	well_3	F006	3740	Cell Height	5.824866	6	2.370463
W3	PGE2	well_4	F001	2792	Cell Height	5.847421	5.5	2.639582
W3	PGE2	well_4	F002	3433	Cell Height	6.165307	6.5	2.670903
W3	PGE2	well_4	F003	2859	Cell Height	5.657398	5.5	2.476388
W3	PGE2	well_4	F004	3317	Cell Height	5.579138	5.5	2.607171
W3	PGE2	well_4	F005	3655	Cell Height	5.65513	5.5	2.509372
W3	PGE2	well_4	F006	3418	Cell Height	5.658133	5.5	2.398162
W3	PGE2	well_5	F001	2468	Cell Height	5.268233	5	2.427709

W3	PGE2	well_5	F002	2542	Cell Height	5.528521	5	2.48899
W3	PGE2	well_5	F003	3154	Cell Height	5.725904	6	2.50013
W3	PGE2	well_5	F004	2966	Cell Height	5.534558	5.5	2.47263
W3	PGE2	well_5	F005	3566	Cell Height	5.643999	5.5	2.521065
W3	PGE2	well_5	F006	2826	Cell Height	5.487615	5.5	2.505538
W4	HPI4	well_1	F001	600	Cell Height	3.2025	3	1.286663
W4	HPI4	well_1	F002	975	Cell Height	3.944615	4	1.654907
W4	HPI4	well_1	F003	928	Cell Height	3.668103	3.5	1.54273
W4	HPI4	well_1	F004	1146	Cell Height	3.630454	3.5	1.519235
W4	HPI4	well_1	F005	1258	Cell Height	3.400636	3	1.519583
W4	HPI4	well_1	F006	1063	Cell Height	3.511289	3.5	1.564184
W4	HPI4	well_2	F001	745	Cell Height	3.424832	3.5	1.352164
W4	HPI4	well_2	F002	1025	Cell Height	3.881463	4	1.606368
W4	HPI4	well_2	F003	1060	Cell Height	3.598585	3.5	1.505874
W4	HPI4	well_2	F004	1165	Cell Height	3.61588	3.5	1.464458
W4	HPI4	well_2	F005	1104	Cell Height	3.331975	3	1.357155
W4	HPI4	well_2	F006	1042	Cell Height	3.37572	3.5	1.385445
W4	HPI4	well_3	F001	943	Cell Height	3.400848	3	1.44649
W4	HPI4	well_3	F002	975	Cell Height	3.704103	3.5	1.522043
W4	HPI4	well_3	F003	1033	Cell Height	3.454985	3.5	1.382216
W4	HPI4	well_3	F004	1257	Cell Height	3.757359	4	1.518593
W4	HPI4	well_3	F005	1209	Cell Height	3.578164	3.5	1.401827
W4	HPI4	well_3	F006	904	Cell Height	3.616704	3.5	1.517501
W4	HPI4	well_4	F001	906	Cell Height	3.350442	3	1.370762
W4	HPI4	well_4	F002	1112	Cell Height	3.544065	3.5	1.497325
W4	HPI4	well_4	F003	792	Cell Height	3.487374	3.5	1.502368
W4	HPI4	well_4	F004	1199	Cell Height	3.580901	3.5	1.509196
W4	HPI4	well_4	F005	1315	Cell Height	3.315209	3	1.398275
W4	HPI4	well_4	F006	971	Cell Height	3.611226	3.5	1.548856
W4	HPI4	well_5	F001	920	Cell Height	3.56413	3.5	1.506864
W4	HPI4	well_5	F002	673	Cell Height	3.465082	3.5	1.457702
W4	HPI4	well_5	F003	860	Cell Height	3.593023	3.5	1.551055
W4	HPI4	well_5	F004	1099	Cell Height	3.50455	3.5	1.536436
W4	HPI4	well_5	F005	1325	Cell Height	3.45283	3.5	1.479671
W4	HPI4	well_5	F006	1116	Cell Height	3.750896	3.5	1.640641
W4	PGE2	well_1	F001	2246	Cell Height	5.557435	5.5	1.981214
W4	PGE2	well_1	F002	1936	Cell Height	5.174329	5.5	1.981297
W4	PGE2	well_1	F003	2992	Cell Height	5.541444	5.5	2.017838
W4	PGE2	well_1	F004	2919	Cell Height	5.875471	6	2.274059
W4	PGE2	well_1	F005	3158	Cell Height	5.788157	6	2.049381
W4	PGE2	well_1	F006	3063	Cell Height	5.677604	5.5	2.039084
W4	PGE2	well_2	F001	2465	Cell Height	5.063692	5	2.015615
W4	PGE2	well_2	F002	3049	Cell Height	5.491473	5.5	2.091084
W4	PGE2	well_2	F003	2809	Cell Height	5.383232	5.5	2.220582
W4	PGE2	well_2	F004	3032	Cell Height	5.433707	5.5	2.024078
W4	PGE2	well_2	F005	3481	Cell Height	5.445849	5.5	2.067923
W4	PGE2	well_2	F006	3022	Cell Height	5.469722	5.5	2.035231

W4	PGE2	well_3	F001	2445	Cell Height	5.141922	5.5	2.03174
W4	PGE2	well_3	F002	3299	Cell Height	5.546984	5.5	1.93208
W4	PGE2	well_3	F003	3466	Cell Height	5.253318	5.5	2.033457
W4	PGE2	well_3	F004	3334	Cell Height	5.45036	5.5	2.405554
W4	PGE2	well_3	F005	2848	Cell Height	5.372893	5.5	2.237637
W4	PGE2	well_3	F006	3272	Cell Height	5.628667	5.75	2.175524
W4	PGE2	well_4	F001	2332	Cell Height	5.195969	5.5	2.059126
W4	PGE2	well_4	F002	2958	Cell Height	5.519101	5.5	2.079228
W4	PGE2	well_4	F003	3074	Cell Height	5.396714	5.5	2.023974
W4	PGE2	well_4	F004	3036	Cell Height	5.407609	5.5	2.076648
W4	PGE2	well_4	F005	3745	Cell Height	5.372363	5.5	1.961978
W4	PGE2	well_4	F006	2576	Cell Height	5.330357	5.5	2.07143
W4	PGE2	well_5	F001	1990	Cell Height	4.871106	5	2.139455
W4	PGE2	well_5	F002	2402	Cell Height	5.304538	5.5	2.315043
W4	PGE2	well_5	F003	2893	Cell Height	5.530591	6	1.982508
W4	PGE2	well_5	F004	2858	Cell Height	5.483905	5.5	2.263045
W4	PGE2	well_5	F005	2650	Cell Height	5.400377	6	2.115814
W4	PGE2	well_5	F006	2271	Cell Height	5.365037	5.5	2.235564
W1	HPI4	well_1	F001	2006	Cell Volume	949.8536	859.5176	540.6072
W1	HPI4	well_1	F002	1797	Cell Volume	894.143	831.3626	515.8425
W1	HPI4	well_1	F003	2325	Cell Volume	875.3147	815.7301	501.543
W1	HPI4	well_1	F004	2112	Cell Volume	963.9555	906.286	501.3429
W1	HPI4	well_1	F005	2475	Cell Volume	907.1015	856.5483	481.1405
W1	HPI4	well_1	F006	1903	Cell Volume	943.4551	866.2424	539.9014
W1	HPI4	well_2	F001	1728	Cell Volume	907.2676	810.9535	548.6735
W1	HPI4	well_2	F002	2072	Cell Volume	926.8953	856.0554	504.1417
W1	HPI4	well_2	F003	2175	Cell Volume	909.4754	854.5297	487.9657
W1	HPI4	well_2	F004	2271	Cell Volume	943.7419	892.7425	500.5504
W1	HPI4	well_2	F005	2464	Cell Volume	921.4829	873.1901	474.9175
W1	HPI4	well_2	F006	2103	Cell Volume	968.2353	877.2274	545.7492
W1	HPI4	well_3	F001	1564	Cell Volume	912.875	836.3857	505.3287
W1	HPI4	well_3	F002	1898	Cell Volume	952.3293	885.959	511.1687
W1	HPI4	well_3	F003	1917	Cell Volume	949.3774	865.8903	545.9728
W1	HPI4	well_3	F004	2384	Cell Volume	889.6352	844.3545	470.8802
W1	HPI4	well_3	F005	2264	Cell Volume	878.2518	830.4942	477.0493
W1	HPI4	well_3	F006	2274	Cell Volume	921.9531	850.0113	534.1553
W1	HPI4	well_4	F001	1973	Cell Volume	860.3124	806.1065	490.8237
W1	HPI4	well_4	F002	2276	Cell Volume	857.5159	805.7544	471.7589
W1	HPI4	well_4	F003	2401	Cell Volume	872.1954	806.2474	507.1081
W1	HPI4	well_4	F004	2109	Cell Volume	864.6818	789.3474	481.9908
W1	HPI4	well_4	F005	2355	Cell Volume	882.363	836.8786	477.5495
W1	HPI4	well_4	F006	2283	Cell Volume	872.8206	805.5901	495.385
W1	HPI4	well_5	F001	2187	Cell Volume	849.0533	778.9022	515.9248
W1	HPI4	well_5	F002	2196	Cell Volume	856.6424	813.7937	471.0039
W1	HPI4	well_5	F003	2472	Cell Volume	869.3803	819.3683	487.9056
W1	HPI4	well_5	F004	2165	Cell Volume	842.3765	779.0431	491.7599
W1	HPI4	well_5	F005	2000	Cell Volume	888.4189	818.8872	499.8817

W1	HPI4	well_5	F006	1889	Cell Volume	882.8477	819.0163	497.2351
W1	PGE2	well_1	F001	2346	Cell Volume	938.9891	846.1267	519.5051
W1	PGE2	well_1	F002	2124	Cell Volume	981.1593	884.1282	573.4416
W1	PGE2	well_1	F003	2450	Cell Volume	957.6149	894.9137	520.8312
W1	PGE2	well_1	F004	2589	Cell Volume	916.0918	846.9247	532.2085
W1	PGE2	well_1	F005	2970	Cell Volume	872.7234	808.0782	487.9966
W1	PGE2	well_1	F006	2939	Cell Volume	893.5979	840.3056	484.5342
W1	PGE2	well_2	F001	2002	Cell Volume	930.9871	869.5167	558.9463
W1	PGE2	well_2	F002	2113	Cell Volume	917.0133	864.3646	480.9336
W1	PGE2	well_2	F003	2227	Cell Volume	890.3436	799.7221	530.9945
W1	PGE2	well_2	F004	2400	Cell Volume	931.7316	844.6949	538.7676
W1	PGE2	well_2	F005	2511	Cell Volume	954.9344	891.3576	530.8402
W1	PGE2	well_2	F006	1903	Cell Volume	954.6109	886.8744	505.5206
W1	PGE2	well_3	F001	2114	Cell Volume	959.1466	893.1415	513.1715
W1	PGE2	well_3	F002	2013	Cell Volume	949.5391	884.2925	520.437
W1	PGE2	well_3	F003	2208	Cell Volume	958.4601	887.3556	524.8734
W1	PGE2	well_3	F004	2146	Cell Volume	968.0796	890.7004	542.4124
W1	PGE2	well_3	F005	2403	Cell Volume	927.2122	848.3096	541.8404
W1	PGE2	well_3	F006	2147	Cell Volume	963.0751	906.1217	529.8153
W1	PGE2	well_4	F001	1891	Cell Volume	960.1377	912.1775	526.7118
W1	PGE2	well_4	F002	1986	Cell Volume	925.8855	864.3528	487.2117
W1	PGE2	well_4	F003	1905	Cell Volume	907.2021	849.5301	497.3063
W1	PGE2	well_4	F004	2397	Cell Volume	900.5438	844.0611	505.2119
W1	PGE2	well_4	F005	2079	Cell Volume	931.5478	888.4236	502.8553
W1	PGE2	well_4	F006	2134	Cell Volume	940.4852	886.3815	510.1743
W1	PGE2	well_5	F001	1894	Cell Volume	936.0146	858.2266	527.5261
W1	PGE2	well_5	F002	2205	Cell Volume	931.8569	864.4585	521.6098
W1	PGE2	well_5	F003	1891	Cell Volume	952.8451	894.7846	509.4618
W1	PGE2	well_5	F004	2105	Cell Volume	965.4256	898.0942	556.053
W1	PGE2	well_5	F005	2176	Cell Volume	915.2393	842.9344	525.0791
W1	PGE2	well_5	F006	1968	Cell Volume	901.7683	853.5439	473.6596
W2	HPI4	well_1	F001	1889	Cell Volume	864.0853	773.5271	522.9367
W2	HPI4	well_1	F002	2032	Cell Volume	924.1005	835.2003	569.8605
W2	HPI4	well_1	F003	2276	Cell Volume	782.7016	671.0239	512.8523
W2	HPI4	well_1	F004	2401	Cell Volume	823.4342	706.5374	542.8381
W2	HPI4	well_1	F005	1985	Cell Volume	902.8516	797.1636	549.8783
W2	HPI4	well_1	F006	1738	Cell Volume	908.5245	849.0607	548.3325
W2	HPI4	well_2	F001	1684	Cell Volume	870.8469	756.5919	547.1697
W2	HPI4	well_2	F002	1846	Cell Volume	953.0489	830.3768	604.1487
W2	HPI4	well_2	F003	2013	Cell Volume	847.1046	748.3649	540.8855
W2	HPI4	well_2	F004	2491	Cell Volume	816.3227	698.3925	543.9637
W2	HPI4	well_2	F005	2106	Cell Volume	898.4205	779.5477	572.7252
W2	HPI4	well_2	F006	1974	Cell Volume	880.3363	799.0062	546.5001
W2	HPI4	well_3	F001	1972	Cell Volume	809.768	754.0803	480.8152
W2	HPI4	well_3	F002	1767	Cell Volume	917.623	830.9636	567.3423
W2	HPI4	well_3	F003	1332	Cell Volume	878.2647	759.4672	573.9621
W2	HPI4	well_3	F004	2224	Cell Volume	826.7366	739.6567	493.2548

W2	HPI4	well_3	F005	2099	Cell Volume	855.8576	774.0669	526.4217
W2	HPI4	well_3	F006	2048	Cell Volume	852.3597	761.0868	521.9254
W2	HPI4	well_4	F001	1980	Cell Volume	766.8992	693.2051	457.6711
W2	HPI4	well_4	F002	1990	Cell Volume	765.3492	668.172	489.5488
W2	HPI4	well_4	F003	2045	Cell Volume	778.5685	701.5847	466.2439
W2	HPI4	well_4	F004	2328	Cell Volume	760.847	658.8888	492.5526
W2	HPI4	well_4	F005	1893	Cell Volume	781.4936	698.2517	481.8278
W2	HPI4	well_4	F006	1970	Cell Volume	862.6608	761.169	522.6619
W2	HPI4	well_5	F001	1814	Cell Volume	811.8438	744.9614	485.4867
W2	HPI4	well_5	F002	1701	Cell Volume	894.8774	795.9665	547.3429
W2	HPI4	well_5	F003	1841	Cell Volume	845.2532	752.9419	525.6002
W2	HPI4	well_5	F004	2312	Cell Volume	800.3067	681.7272	526.4138
W2	HPI4	well_5	F005	2140	Cell Volume	817.8807	745.2783	504.7235
W2	HPI4	well_5	F006	1915	Cell Volume	813.6981	709.7765	537.6463
W2	PGE2	well_1	F001	2508	Cell Volume	864.6205	872.8615	433.9827
W2	PGE2	well_1	F002	2196	Cell Volume	834.5226	830.0951	421.288
W2	PGE2	well_1	F003	3078	Cell Volume	861.9021	864.9983	394.4741
W2	PGE2	well_1	F004	2519	Cell Volume	814.1226	807.7965	416.1164
W2	PGE2	well_1	F005	3120	Cell Volume	807.3367	813.9228	383.0678
W2	PGE2	well_1	F006	3092	Cell Volume	819.0619	821.5747	395.1239
W2	PGE2	well_2	F001	2524	Cell Volume	834.7066	838.2752	389.0072
W2	PGE2	well_2	F002	3220	Cell Volume	798.3688	793.7132	382.9612
W2	PGE2	well_2	F003	3754	Cell Volume	799.1643	800.8253	375.8997
W2	PGE2	well_2	F004	3308	Cell Volume	800.2094	783.2915	379.771
W2	PGE2	well_2	F005	2732	Cell Volume	844.9709	855.3278	400.2389
W2	PGE2	well_2	F006	2641	Cell Volume	810.0573	809.2518	391.4058
W2	PGE2	well_3	F001	2301	Cell Volume	807.8009	806.9046	390.8524
W2	PGE2	well_3	F002	3625	Cell Volume	800.7348	798.9944	380.6112
W2	PGE2	well_3	F003	3166	Cell Volume	875.5516	853.5791	411.6181
W2	PGE2	well_3	F004	3460	Cell Volume	768.2436	764.3494	379.3144
W2	PGE2	well_3	F005	2810	Cell Volume	800.7619	796.3773	389.5264
W2	PGE2	well_3	F006	2454	Cell Volume	820.4783	824.4149	391.2686
W2	PGE2	well_4	F001	2778	Cell Volume	879.5881	876.934	403.8656
W2	PGE2	well_4	F002	3296	Cell Volume	872.9734	865.6086	398.4721
W2	PGE2	well_4	F003	3146	Cell Volume	853.2928	860.1631	386.9143
W2	PGE2	well_4	F004	3610	Cell Volume	850.0738	843.756	392.9705
W2	PGE2	well_4	F005	3154	Cell Volume	873.1188	873.6244	390.2475
W2	PGE2	well_4	F006	2634	Cell Volume	875.0773	877.1217	401.2923
W2	PGE2	well_5	F001	3092	Cell Volume	846.8426	833.2404	404.4158
W2	PGE2	well_5	F002	3496	Cell Volume	869.3922	872.8498	381.5094
W2	PGE2	well_5	F003	3256	Cell Volume	827.6178	814.6739	394.2542
W2	PGE2	well_5	F004	2836	Cell Volume	827.5664	815.5776	395.2434
W2	PGE2	well_5	F005	2895	Cell Volume	864.9753	870.6786	386.9328
W2	PGE2	well_5	F006	3259	Cell Volume	834.8384	826.6213	406.1815
W3	HPI4	well_1	F001	876	Cell Volume	1029.145	883.1776	668.7983
W3	HPI4	well_1	F002	1131	Cell Volume	952.904	821.4808	638.1399
W3	HPI4	well_1	F003	933	Cell Volume	971.7485	846.0328	633.6705

W3	HPI4	well_1	F004	1484	Cell Volume	787.0896	670.3315	504.0761
W3	HPI4	well_1	F005	1385	Cell Volume	919.7615	794.3469	580.6803
W3	HPI4	well_1	F006	955	Cell Volume	995.1485	829.9074	651.4576
W3	HPI4	well_2	F001	819	Cell Volume	965.7689	782.7751	656.0383
W3	HPI4	well_2	F002	1035	Cell Volume	990.7007	863.6135	620.909
W3	HPI4	well_2	F003	1013	Cell Volume	971.5242	871.594	603.5164
W3	HPI4	well_2	F004	1327	Cell Volume	925.6978	793.854	596.8109
W3	HPI4	well_2	F005	1421	Cell Volume	904.2243	765.8047	620.6302
W3	HPI4	well_2	F006	1153	Cell Volume	1006.85	880.3022	649.6112
W3	HPI4	well_3	F001	874	Cell Volume	990.9404	854.0368	666.6165
W3	HPI4	well_3	F002	979	Cell Volume	1028.912	951.7517	608.1715
W3	HPI4	well_3	F003	721	Cell Volume	983.6947	864.3881	619.3501
W3	HPI4	well_3	F004	1130	Cell Volume	829.408	703.0517	561.2198
W3	HPI4	well_3	F005	1348	Cell Volume	836.6772	740.8655	547.2971
W3	HPI4	well_3	F006	1161	Cell Volume	915.6152	779.3717	615.9589
W3	HPI4	well_4	F001	815	Cell Volume	889.5254	780.1463	597.4024
W3	HPI4	well_4	F002	994	Cell Volume	939.4696	817.1385	587.199
W3	HPI4	well_4	F003	827	Cell Volume	912.1052	806.0831	592.4304
W3	HPI4	well_4	F004	853	Cell Volume	886.4655	736.9104	562.1338
W3	HPI4	well_4	F005	1488	Cell Volume	867.1462	772.33	536.0482
W3	HPI4	well_4	F006	1371	Cell Volume	913.2926	813.0543	580.3951
W3	HPI4	well_5	F001	734	Cell Volume	1010.021	913.9731	625.3853
W3	HPI4	well_5	F002	665	Cell Volume	871.0339	742.356	544.0332
W3	HPI4	well_5	F003	933	Cell Volume	917.7945	818.805	602.602
W3	HPI4	well_5	F004	650	Cell Volume	887.897	753.1649	605.4344
W3	HPI4	well_5	F005	1139	Cell Volume	947.0491	827.4663	613.4167
W3	HPI4	well_5	F006	1081	Cell Volume	938.822	827.2315	618.9181
W3	PGE2	well_1	F001	2916	Cell Volume	899.3433	864.8692	450.0962
W3	PGE2	well_1	F002	3725	Cell Volume	859.2362	846.1267	431.0499
W3	PGE2	well_1	F003	3755	Cell Volume	891.5059	864.7401	437.2403
W3	PGE2	well_1	F004	3148	Cell Volume	900.2234	881.2763	405.952
W3	PGE2	well_1	F005	3514	Cell Volume	886.8711	875.6665	399.5377
W3	PGE2	well_1	F006	3064	Cell Volume	918.0875	916.2734	420.9597
W3	PGE2	well_2	F001	3084	Cell Volume	891.9375	862.4281	418.583
W3	PGE2	well_2	F002	3211	Cell Volume	929.682	918.4211	450.0323
W3	PGE2	well_2	F003	3552	Cell Volume	893.7755	871.7114	430.3233
W3	PGE2	well_2	F004	3708	Cell Volume	866.4279	844.9765	387.6495
W3	PGE2	well_2	F005	3916	Cell Volume	895.9438	857.6281	429.1053
W3	PGE2	well_2	F006	4006	Cell Volume	920.4366	917.1419	386.8145
W3	PGE2	well_3	F001	3250	Cell Volume	884.8186	854.072	423.8898
W3	PGE2	well_3	F002	2986	Cell Volume	825.7127	809.6978	399.6751
W3	PGE2	well_3	F003	3965	Cell Volume	869.4961	863.5196	368.9585
W3	PGE2	well_3	F004	3537	Cell Volume	897.2594	881.5697	394.6377
W3	PGE2	well_3	F005	3998	Cell Volume	916.9584	891.698	416.9192
W3	PGE2	well_3	F006	3740	Cell Volume	940.0856	931.9294	414.0194
W3	PGE2	well_4	F001	2792	Cell Volume	909.3587	908.7623	407.9827
W3	PGE2	well_4	F002	3433	Cell Volume	936.9297	932.4106	416.2559

W3	PGE2	well_4	F003	2859	Cell Volume	898.1987	885.959	413.5078
W3	PGE2	well_4	F004	3317	Cell Volume	872.0935	863.6369	402.4182
W3	PGE2	well_4	F005	3655	Cell Volume	872.6482	880.3257	392.9015
W3	PGE2	well_4	F006	3418	Cell Volume	907.6596	886.8744	426.9041
W3	PGE2	well_5	F001	2468	Cell Volume	852.6398	834.7896	440.6452
W3	PGE2	well_5	F002	2542	Cell Volume	944.9904	926.6129	461.7662
W3	PGE2	well_5	F003	3154	Cell Volume	874.6725	870.3031	394.5544
W3	PGE2	well_5	F004	2966	Cell Volume	828.9818	824.3327	390.3504
W3	PGE2	well_5	F005	3566	Cell Volume	826.6639	830.1069	379.4346
W3	PGE2	well_5	F006	2826	Cell Volume	887.2344	886.8392	409.8647
W4	HPI4	well_1	F001	600	Cell Volume	1015.539	869.7867	639.319
W4	HPI4	well_1	F002	975	Cell Volume	1097.788	1008.86	658.0257
W4	HPI4	well_1	F003	928	Cell Volume	995.2154	890.4422	613.1961
W4	HPI4	well_1	F004	1146	Cell Volume	994.8204	890.5126	603.9977
W4	HPI4	well_1	F005	1258	Cell Volume	894.0629	723.3787	666.6417
W4	HPI4	well_1	F006	1063	Cell Volume	941.5263	807.0689	583.4806
W4	HPI4	well_2	F001	745	Cell Volume	1101.282	1012.38	606.0451
W4	HPI4	well_2	F002	1025	Cell Volume	1143.781	1042.167	666.1261
W4	HPI4	well_2	F003	1060	Cell Volume	1023.661	915.0059	639.974
W4	HPI4	well_2	F004	1165	Cell Volume	1020.263	877.4151	665.5287
W4	HPI4	well_2	F005	1104	Cell Volume	888.739	769.4312	539.2208
W4	HPI4	well_2	F006	1042	Cell Volume	930.8922	822.7014	557.1215
W4	HPI4	well_3	F001	943	Cell Volume	984.2802	849.9996	632.2674
W4	HPI4	well_3	F002	975	Cell Volume	1071.389	990.1522	618.6754
W4	HPI4	well_3	F003	1033	Cell Volume	1061.688	962.5958	658.2305
W4	HPI4	well_3	F004	1257	Cell Volume	1001.606	872.3921	642.6392
W4	HPI4	well_3	F005	1209	Cell Volume	939.0347	857.0178	560.7187
W4	HPI4	well_3	F006	904	Cell Volume	1002.314	883.9991	628.4561
W4	HPI4	well_4	F001	906	Cell Volume	1055.686	926.3195	680.8098
W4	HPI4	well_4	F002	1112	Cell Volume	1053.582	926.4369	668.9978
W4	HPI4	well_4	F003	792	Cell Volume	1128.615	974.8483	717.1392
W4	HPI4	well_4	F004	1199	Cell Volume	1047.489	871.4532	714.9543
W4	HPI4	well_4	F005	1315	Cell Volume	857.6684	706.4904	580.4593
W4	HPI4	well_4	F006	971	Cell Volume	1112.872	1018.648	701.5554
W4	HPI4	well_5	F001	920	Cell Volume	1186.457	1063.984	733.6986
W4	HPI4	well_5	F002	673	Cell Volume	1124.057	970.0131	718.9338
W4	HPI4	well_5	F003	860	Cell Volume	1095.051	978.287	667.0893
W4	HPI4	well_5	F004	1099	Cell Volume	1078.44	908.3281	724.7368
W4	HPI4	well_5	F005	1325	Cell Volume	968.5151	807.3975	655.7587
W4	HPI4	well_5	F006	1116	Cell Volume	1048.262	941.0718	620.1522
W4	PGE2	well_1	F001	2246	Cell Volume	972.6079	973.9564	423.2136
W4	PGE2	well_1	F002	1936	Cell Volume	949.3352	926.4017	483.1391
W4	PGE2	well_1	F003	2992	Cell Volume	951.1752	927.7631	412.8985
W4	PGE2	well_1	F004	2919	Cell Volume	952.274	947.8553	424.0893
W4	PGE2	well_1	F005	3158	Cell Volume	966.3323	939.0649	440.8227
W4	PGE2	well_1	F006	3063	Cell Volume	967.1652	913.2572	448.2318
W4	PGE2	well_2	F001	2465	Cell Volume	925.1522	903.9153	435.6946

W4	PGE2	well_2	F002	3049	Cell Volume	921.1186	902.2957	409.3435
W4	PGE2	well_2	F003	2809	Cell Volume	902.2685	871.1715	444.9156
W4	PGE2	well_2	F004	3032	Cell Volume	909.9875	889.9493	398.7101
W4	PGE2	well_2	F005	3481	Cell Volume	867.3428	867.2751	370.3325
W4	PGE2	well_2	F006	3022	Cell Volume	966.4175	914.7008	459.6549
W4	PGE2	well_3	F001	2445	Cell Volume	934.2705	890.5596	478.1851
W4	PGE2	well_3	F002	3299	Cell Volume	924.6952	914.7125	387.5495
W4	PGE2	well_3	F003	3466	Cell Volume	868.1847	845.6807	401.7842
W4	PGE2	well_3	F004	3334	Cell Volume	859.9334	858.3088	409.8911
W4	PGE2	well_3	F005	2848	Cell Volume	916.8421	882.9076	445.6304
W4	PGE2	well_3	F006	3272	Cell Volume	942.486	943.1256	419.9076
W4	PGE2	well_4	F001	2332	Cell Volume	836.8494	828.5929	370.5376
W4	PGE2	well_4	F002	2958	Cell Volume	917.1843	891.9562	417.2832
W4	PGE2	well_4	F003	3074	Cell Volume	895.8732	866.7118	375.1618
W4	PGE2	well_4	F004	3036	Cell Volume	862.8652	842.1951	391.0743
W4	PGE2	well_4	F005	3745	Cell Volume	870.652	870.9603	363.8939
W4	PGE2	well_4	F006	2576	Cell Volume	932.3029	888.8109	466.0624
W4	PGE2	well_5	F001	1990	Cell Volume	851.2471	823.3938	428.1064
W4	PGE2	well_5	F002	2402	Cell Volume	857.2307	846.0915	390.8781
W4	PGE2	well_5	F003	2893	Cell Volume	845.2317	838.5217	352.0051
W4	PGE2	well_5	F004	2858	Cell Volume	855.8513	850.4456	378.0425
W4	PGE2	well_5	F005	2650	Cell Volume	872.6684	880.6191	383.3874
W4	PGE2	well_5	F006	2271	Cell Volume	935.3399	922.7635	463.7274
W1	HPI4	well_1	F001	2006	Cell Width	20.48315	19.71667	5.44736
W1	HPI4	well_1	F002	1797	Cell Width	19.94768	19.06667	5.323894
W1	HPI4	well_1	F003	2325	Cell Width	19.14658	18.41667	5.039065
W1	HPI4	well_1	F004	2112	Cell Width	20.30183	19.71667	4.98264
W1	HPI4	well_1	F005	2475	Cell Width	19.52731	18.95833	4.835804
W1	HPI4	well_1	F006	1903	Cell Width	20.24439	19.5	5.393298
W1	HPI4	well_2	F001	1728	Cell Width	20.1836	19.39167	5.404048
W1	HPI4	well_2	F002	2072	Cell Width	20.46982	19.825	5.278514
W1	HPI4	well_2	F003	2175	Cell Width	20.12818	19.39167	5.37579
W1	HPI4	well_2	F004	2271	Cell Width	20.28467	19.71667	5.029098
W1	HPI4	well_2	F005	2464	Cell Width	19.73742	19.06667	4.936948
W1	HPI4	well_2	F006	2103	Cell Width	19.9851	19.06667	5.394676
W1	HPI4	well_3	F001	1564	Cell Width	20.86546	20.04167	5.629307
W1	HPI4	well_3	F002	1898	Cell Width	20.34172	19.825	4.961677
W1	HPI4	well_3	F003	1917	Cell Width	20.1734	19.39167	5.439625
W1	HPI4	well_3	F004	2384	Cell Width	19.63128	19.175	4.923436
W1	HPI4	well_3	F005	2264	Cell Width	19.61967	18.95833	5.142195
W1	HPI4	well_3	F006	2274	Cell Width	19.69247	19.06667	5.153327
W1	HPI4	well_4	F001	1973	Cell Width	19.60625	18.95833	5.325622
W1	HPI4	well_4	F002	2276	Cell Width	19.26691	18.85	4.869273
W1	HPI4	well_4	F003	2401	Cell Width	19.45199	18.74167	5.034351
W1	HPI4	well_4	F004	2109	Cell Width	19.86224	19.28333	5.152177
W1	HPI4	well_4	F005	2355	Cell Width	19.82997	19.06667	5.374414
W1	HPI4	well_4	F006	2283	Cell Width	19.59614	18.85	5.140988

W1	HPI4	well_5	F001	2187	Cell Width	19.61081	18.74167	5.500477
W1	HPI4	well_5	F002	2196	Cell Width	19.52975	18.74167	5.156633
W1	HPI4	well_5	F003	2472	Cell Width	19.11597	18.525	5.034743
W1	HPI4	well_5	F004	2165	Cell Width	19.0393	18.30833	5.053482
W1	HPI4	well_5	F005	2000	Cell Width	20.16804	19.39167	5.29964
W1	HPI4	well_5	F006	1889	Cell Width	20.10056	19.28333	5.589352
W1	PGE2	well_1	F001	2346	Cell Width	19.96552	19.06667	5.451789
W1	PGE2	well_1	F002	2124	Cell Width	20.55508	19.71667	5.542205
W1	PGE2	well_1	F003	2450	Cell Width	20.20479	19.60833	5.12924
W1	PGE2	well_1	F004	2589	Cell Width	19.42058	18.63333	5.034546
W1	PGE2	well_1	F005	2970	Cell Width	18.84449	18.09167	4.894066
W1	PGE2	well_1	F006	2939	Cell Width	19.16479	18.41667	4.941907
W1	PGE2	well_2	F001	2002	Cell Width	19.91596	19.28333	5.05278
W1	PGE2	well_2	F002	2113	Cell Width	19.74435	19.06667	4.930549
W1	PGE2	well_2	F003	2227	Cell Width	19.75899	18.95833	5.17729
W1	PGE2	well_2	F004	2400	Cell Width	19.74601	18.95833	5.079722
W1	PGE2	well_2	F005	2511	Cell Width	19.95633	19.5	4.857423
W1	PGE2	well_2	F006	1903	Cell Width	20.33798	19.71667	5.293485
W1	PGE2	well_3	F001	2114	Cell Width	20.399	19.71667	5.131992
W1	PGE2	well_3	F002	2013	Cell Width	20.35623	19.71667	5.210192
W1	PGE2	well_3	F003	2208	Cell Width	20.11551	19.39167	5.295793
W1	PGE2	well_3	F004	2146	Cell Width	20.45657	19.825	5.287828
W1	PGE2	well_3	F005	2403	Cell Width	19.86129	18.85	5.585408
W1	PGE2	well_3	F006	2147	Cell Width	20.25182	19.39167	5.341919
W1	PGE2	well_4	F001	1891	Cell Width	20.67333	20.04167	5.253016
W1	PGE2	well_4	F002	1986	Cell Width	20.08552	19.5	4.986727
W1	PGE2	well_4	F003	1905	Cell Width	20.23945	19.60833	5.327636
W1	PGE2	well_4	F004	2397	Cell Width	19.45634	18.74167	5.062438
W1	PGE2	well_4	F005	2079	Cell Width	20.31315	19.60833	5.404218
W1	PGE2	well_4	F006	2134	Cell Width	19.97973	19.175	5.208669
W1	PGE2	well_5	F001	1894	Cell Width	20.37141	19.5	5.647303
W1	PGE2	well_5	F002	2205	Cell Width	20.13909	19.5	5.315132
W1	PGE2	well_5	F003	1891	Cell Width	20.62836	20.04167	5.169185
W1	PGE2	well_5	F004	2105	Cell Width	20.44855	19.71667	5.288136
W1	PGE2	well_5	F005	2176	Cell Width	19.90237	19.28333	5.00319
W1	PGE2	well_5	F006	1968	Cell Width	19.97264	19.28333	4.918861
W2	HPI4	well_1	F001	1889	Cell Width	20.16124	19.60833	5.41659
W2	HPI4	well_1	F002	2032	Cell Width	20.47015	19.825	5.736374
W2	HPI4	well_1	F003	2276	Cell Width	19.05715	18.30833	5.561865
W2	HPI4	well_1	F004	2401	Cell Width	19.37858	18.525	5.468782
W2	HPI4	well_1	F005	1985	Cell Width	20.6986	20.25833	5.667544
W2	HPI4	well_1	F006	1738	Cell Width	20.70351	19.93333	5.796348
W2	HPI4	well_2	F001	1684	Cell Width	20.21285	19.60833	5.465246
W2	HPI4	well_2	F002	1846	Cell Width	20.51731	19.93333	5.544112
W2	HPI4	well_2	F003	2013	Cell Width	19.74314	19.06667	5.368221
W2	HPI4	well_2	F004	2491	Cell Width	19.08645	18.41667	5.318194
W2	HPI4	well_2	F005	2106	Cell Width	20.18683	19.39167	5.389167

W2	HPI4	well_2	F006	1974	Cell Width	19.79169	19.28333	5.338803
W2	HPI4	well_3	F001	1972	Cell Width	19.50983	18.95833	5.011612
W2	HPI4	well_3	F002	1767	Cell Width	20.29383	19.60833	5.5931
W2	HPI4	well_3	F003	1332	Cell Width	21.20828	20.475	6.673208
W2	HPI4	well_3	F004	2224	Cell Width	19.64131	19.06667	5.213364
W2	HPI4	well_3	F005	2099	Cell Width	19.65334	18.95833	5.593682
W2	HPI4	well_3	F006	2048	Cell Width	19.68197	19.06667	5.478387
W2	HPI4	well_4	F001	1980	Cell Width	19.05031	18.41667	4.919882
W2	HPI4	well_4	F002	1990	Cell Width	18.53578	17.875	5.05275
W2	HPI4	well_4	F003	2045	Cell Width	19.37811	18.85	5.239659
W2	HPI4	well_4	F004	2328	Cell Width	18.70937	17.98333	5.116926
W2	HPI4	well_4	F005	1893	Cell Width	19.35733	18.525	5.406648
W2	HPI4	well_4	F006	1970	Cell Width	19.77721	18.74167	5.82144
W2	HPI4	well_5	F001	1814	Cell Width	19.70084	19.0125	5.36986
W2	HPI4	well_5	F002	1701	Cell Width	20.58059	20.04167	5.855612
W2	HPI4	well_5	F003	1841	Cell Width	19.82541	19.28333	5.428685
W2	HPI4	well_5	F004	2312	Cell Width	18.97159	18.41667	5.218376
W2	HPI4	well_5	F005	2140	Cell Width	19.40554	18.95833	5.470736
W2	HPI4	well_5	F006	1915	Cell Width	19.37537	18.63333	5.502601
W2	PGE2	well_1	F001	2508	Cell Width	17.35312	16.9	4.088634
W2	PGE2	well_1	F002	2196	Cell Width	16.7559	16.14167	4.023619
W2	PGE2	well_1	F003	3078	Cell Width	17.42294	17.00833	3.945018
W2	PGE2	well_1	F004	2519	Cell Width	17.07155	16.68333	3.814156
W2	PGE2	well_1	F005	3120	Cell Width	16.98052	16.575	3.825434
W2	PGE2	well_1	F006	3092	Cell Width	16.37102	16.03333	3.528567
W2	PGE2	well_2	F001	2524	Cell Width	16.62865	16.35833	3.550319
W2	PGE2	well_2	F002	3220	Cell Width	16.15755	15.70833	3.45813
W2	PGE2	well_2	F003	3754	Cell Width	16.51186	16.25	3.643678
W2	PGE2	well_2	F004	3308	Cell Width	16.48504	16.03333	3.704488
W2	PGE2	well_2	F005	2732	Cell Width	16.70443	16.25	3.721076
W2	PGE2	well_2	F006	2641	Cell Width	16.80471	16.575	3.548562
W2	PGE2	well_3	F001	2301	Cell Width	16.56361	16.03333	4.085103
W2	PGE2	well_3	F002	3625	Cell Width	16.69122	16.14167	3.840466
W2	PGE2	well_3	F003	3166	Cell Width	17.05251	16.68333	3.912662
W2	PGE2	well_3	F004	3460	Cell Width	16.0116	15.6	3.465232
W2	PGE2	well_3	F005	2810	Cell Width	16.42642	16.03333	3.596703
W2	PGE2	well_3	F006	2454	Cell Width	16.66479	16.25	3.770857
W2	PGE2	well_4	F001	2778	Cell Width	17.2629	16.68333	4.025255
W2	PGE2	well_4	F002	3296	Cell Width	16.48589	16.03333	3.710883
W2	PGE2	well_4	F003	3146	Cell Width	16.75744	16.25	3.635404
W2	PGE2	well_4	F004	3610	Cell Width	16.93292	16.46667	3.950342
W2	PGE2	well_4	F005	3154	Cell Width	16.66551	16.14167	3.812094
W2	PGE2	well_4	F006	2634	Cell Width	16.99209	16.46667	3.761753
W2	PGE2	well_5	F001	3092	Cell Width	16.83753	16.25	4.026291
W2	PGE2	well_5	F002	3496	Cell Width	17.01971	16.575	3.601075
W2	PGE2	well_5	F003	3256	Cell Width	17.12715	16.68333	3.803065
W2	PGE2	well_5	F004	2836	Cell Width	16.4423	16.0875	3.432125

W2	PGE2	well_5	F005	2895	Cell Width	16.42528	16.14167	3.340559
W2	PGE2	well_5	F006	3259	Cell Width	16.39144	16.03333	3.606124
W3	HPI4	well_1	F001	876	Cell Width	21.85378	20.90833	6.458424
W3	HPI4	well_1	F002	1131	Cell Width	21.5978	20.69167	6.193863
W3	HPI4	well_1	F003	933	Cell Width	22.12856	21.45	6.229419
W3	HPI4	well_1	F004	1484	Cell Width	19.96319	19.28333	5.409132
W3	HPI4	well_1	F005	1385	Cell Width	21.45047	20.69167	5.815612
W3	HPI4	well_1	F006	955	Cell Width	22.49533	21.775	6.392947
W3	HPI4	well_2	F001	819	Cell Width	21.85873	21.23333	6.316008
W3	HPI4	well_2	F002	1035	Cell Width	21.99135	21.55833	5.557855
W3	HPI4	well_2	F003	1013	Cell Width	22.00995	21.125	6.559917
W3	HPI4	well_2	F004	1327	Cell Width	21.18068	20.475	5.655493
W3	HPI4	well_2	F005	1421	Cell Width	21.25239	20.69167	5.870313
W3	HPI4	well_2	F006	1153	Cell Width	22.02906	21.45	6.115801
W3	HPI4	well_3	F001	874	Cell Width	21.61523	21.23333	6.598259
W3	HPI4	well_3	F002	979	Cell Width	22.50445	21.99167	6.04538
W3	HPI4	well_3	F003	721	Cell Width	22.187	21.66667	6.326661
W3	HPI4	well_3	F004	1130	Cell Width	20.81802	20.15	5.834135
W3	HPI4	well_3	F005	1348	Cell Width	20.18432	19.39167	5.80727
W3	HPI4	well_3	F006	1161	Cell Width	20.88034	19.93333	6.343986
W3	HPI4	well_4	F001	815	Cell Width	21.55116	21.45	6.332273
W3	HPI4	well_4	F002	994	Cell Width	22.47699	21.66667	6.123864
W3	HPI4	well_4	F003	827	Cell Width	22.16314	21.45	6.241401
W3	HPI4	well_4	F004	853	Cell Width	21.6433	21.125	5.706811
W3	HPI4	well_4	F005	1488	Cell Width	20.52727	19.6625	5.80609
W3	HPI4	well_4	F006	1371	Cell Width	20.80537	20.15	5.787724
W3	HPI4	well_5	F001	734	Cell Width	23.01272	22.31667	6.626124
W3	HPI4	well_5	F002	665	Cell Width	21.73786	21.125	5.974909
W3	HPI4	well_5	F003	933	Cell Width	21.33064	20.69167	5.858008
W3	HPI4	well_5	F004	650	Cell Width	21.57533	21.01667	6.443182
W3	HPI4	well_5	F005	1139	Cell Width	21.44876	20.8	5.895275
W3	HPI4	well_5	F006	1081	Cell Width	21.47545	20.58333	6.307677
W3	PGE2	well_1	F001	2916	Cell Width	17.58589	16.9	4.031653
W3	PGE2	well_1	F002	3725	Cell Width	17.18693	16.79167	3.66504
W3	PGE2	well_1	F003	3755	Cell Width	17.30644	16.68333	3.7801
W3	PGE2	well_1	F004	3148	Cell Width	16.80677	16.03333	3.979927
W3	PGE2	well_1	F005	3514	Cell Width	17.2467	16.35833	4.095842
W3	PGE2	well_1	F006	3064	Cell Width	17.92567	17.33333	4.138833
W3	PGE2	well_2	F001	3084	Cell Width	18.00782	17.225	4.289515
W3	PGE2	well_2	F002	3211	Cell Width	17.67763	16.9	4.140368
W3	PGE2	well_2	F003	3552	Cell Width	17.40055	16.79167	3.823837
W3	PGE2	well_2	F004	3708	Cell Width	17.397	16.79167	3.990839
W3	PGE2	well_2	F005	3916	Cell Width	17.55335	16.79167	4.147499
W3	PGE2	well_2	F006	4006	Cell Width	17.55133	16.9	3.74174
W3	PGE2	well_3	F001	3250	Cell Width	17.78883	16.84583	4.690008
W3	PGE2	well_3	F002	2986	Cell Width	17.86869	17.225	4.264085
W3	PGE2	well_3	F003	3965	Cell Width	17.34746	16.575	4.138955

W3	PGE2	well_3	F004	3537	Cell Width	17.50547	16.575	4.37582
W3	PGE2	well_3	F005	3998	Cell Width	17.28404	16.575	3.872402
W3	PGE2	well_3	F006	3740	Cell Width	17.82231	17.11667	3.880241
W3	PGE2	well_4	F001	2792	Cell Width	17.56901	16.95417	3.994157
W3	PGE2	well_4	F002	3433	Cell Width	17.52832	16.79167	4.169908
W3	PGE2	well_4	F003	2859	Cell Width	17.63787	17.00833	3.918728
W3	PGE2	well_4	F004	3317	Cell Width	17.95868	17.00833	4.572525
W3	PGE2	well_4	F005	3655	Cell Width	17.53011	16.79167	3.893538
W3	PGE2	well_4	F006	3418	Cell Width	17.81548	17.11667	4.141793
W3	PGE2	well_5	F001	2468	Cell Width	17.78304	16.9	4.479885
W3	PGE2	well_5	F002	2542	Cell Width	18.28694	17.55	4.547349
W3	PGE2	well_5	F003	3154	Cell Width	17.55113	16.79167	4.078301
W3	PGE2	well_5	F004	2966	Cell Width	17.27657	16.46667	4.243012
W3	PGE2	well_5	F005	3566	Cell Width	17.10527	16.46667	3.894734
W3	PGE2	well_5	F006	2826	Cell Width	17.87005	17.11667	4.299259
W4	HPI4	well_1	F001	600	Cell Width	23.47132	23.18333	6.249197
W4	HPI4	well_1	F002	975	Cell Width	22.28056	21.775	5.831677
W4	HPI4	well_1	F003	928	Cell Width	21.81072	21.2875	5.799875
W4	HPI4	well_1	F004	1146	Cell Width	22.19132	21.775	5.781514
W4	HPI4	well_1	F005	1258	Cell Width	21.07574	20.25833	6.234312
W4	HPI4	well_1	F006	1063	Cell Width	22.02652	21.34167	6.320648
W4	HPI4	well_2	F001	745	Cell Width	23.99023	23.50833	6.024843
W4	HPI4	well_2	F002	1025	Cell Width	23.33352	22.64167	6.183972
W4	HPI4	well_2	F003	1060	Cell Width	22.63165	21.88333	6.423224
W4	HPI4	well_2	F004	1165	Cell Width	22.44871	21.88333	6.199945
W4	HPI4	well_2	F005	1104	Cell Width	21.61083	21.125	5.898446
W4	HPI4	well_2	F006	1042	Cell Width	22.45713	21.66667	5.834366
W4	HPI4	well_3	F001	943	Cell Width	22.66499	22.20833	6.220178
W4	HPI4	well_3	F002	975	Cell Width	23.04433	22.64167	6.051074
W4	HPI4	well_3	F003	1033	Cell Width	23.27185	22.75	6.337092
W4	HPI4	well_3	F004	1257	Cell Width	21.75699	21.125	5.841318
W4	HPI4	well_3	F005	1209	Cell Width	21.77733	21.34167	5.53025
W4	HPI4	well_3	F006	904	Cell Width	22.27017	21.66667	6.217563
W4	HPI4	well_4	F001	906	Cell Width	23.21921	22.53333	6.371074
W4	HPI4	well_4	F002	1112	Cell Width	22.84976	22.1	6.015949
W4	HPI4	well_4	F003	792	Cell Width	23.9444	23.4	6.553589
W4	HPI4	well_4	F004	1199	Cell Width	22.38624	21.88333	6.336954
W4	HPI4	well_4	F005	1315	Cell Width	21.20977	20.58333	6.015978
W4	HPI4	well_4	F006	971	Cell Width	23.09162	22.425	6.371853
W4	HPI4	well_5	F001	920	Cell Width	24.21097	23.8875	6.32457
W4	HPI4	well_5	F002	673	Cell Width	23.76492	23.18333	6.299506
W4	HPI4	well_5	F003	860	Cell Width	23.47407	22.85833	6.329373
W4	HPI4	well_5	F004	1099	Cell Width	23.19092	22.425	6.781062
W4	HPI4	well_5	F005	1325	Cell Width	22.28175	21.66667	6.036195
W4	HPI4	well_5	F006	1116	Cell Width	22.72311	22.1	6.186562
W4	PGE2	well_1	F001	2246	Cell Width	18.29497	17.76667	3.88722
W4	PGE2	well_1	F002	1936	Cell Width	18.38516	17.875	3.87192

W4	PGE2	well_1	F003	2992	Cell Width	18.13374	17.55	3.685942
W4	PGE2	well_1	F004	2919	Cell Width	17.62675	16.79167	4.046949
W4	PGE2	well_1	F005	3158	Cell Width	17.94937	17.33333	4.067994
W4	PGE2	well_1	F006	3063	Cell Width	17.89753	17.33333	3.672242
W4	PGE2	well_2	F001	2465	Cell Width	18.76883	18.41667	4.083528
W4	PGE2	well_2	F002	3049	Cell Width	18.08108	17.33333	4.285715
W4	PGE2	well_2	F003	2809	Cell Width	18.05399	17.44167	4.163305
W4	PGE2	well_2	F004	3032	Cell Width	17.92734	17.225	3.933106
W4	PGE2	well_2	F005	3481	Cell Width	17.57409	16.9	3.885263
W4	PGE2	well_2	F006	3022	Cell Width	18.23061	17.60417	3.973294
W4	PGE2	well_3	F001	2445	Cell Width	18.44299	17.98333	3.980238
W4	PGE2	well_3	F002	3299	Cell Width	17.90925	17.44167	3.829069
W4	PGE2	well_3	F003	3466	Cell Width	17.76104	17.0625	3.950907
W4	PGE2	well_3	F004	3334	Cell Width	17.63205	16.79167	4.198575
W4	PGE2	well_3	F005	2848	Cell Width	18.30765	17.55	4.482335
W4	PGE2	well_3	F006	3272	Cell Width	18.02942	17.44167	3.985704
W4	PGE2	well_4	F001	2332	Cell Width	17.74558	17.11667	3.939592
W4	PGE2	well_4	F002	2958	Cell Width	17.81897	17.225	3.90797
W4	PGE2	well_4	F003	3074	Cell Width	17.95785	17.33333	3.811269
W4	PGE2	well_4	F004	3036	Cell Width	17.55478	16.9	3.96431
W4	PGE2	well_4	F005	3745	Cell Width	17.57953	17.11667	3.626411
W4	PGE2	well_4	F006	2576	Cell Width	18.00739	17.55	3.795873
W4	PGE2	well_5	F001	1990	Cell Width	18.16195	17.55	4.122022
W4	PGE2	well_5	F002	2402	Cell Width	17.88276	16.79167	4.335062
W4	PGE2	well_5	F003	2893	Cell Width	16.95639	16.25	3.611566
W4	PGE2	well_5	F004	2858	Cell Width	17.48086	16.52083	4.187435
W4	PGE2	well_5	F005	2650	Cell Width	17.59689	17.00833	3.969691
W4	PGE2	well_5	F006	2271	Cell Width	18.12649	17.225	4.514983
W1	HPI4	well_1	F001	1779	Nucleus AR	1.613084	1.5	0.716356
W1	HPI4	well_1	F002	1534	Nucleus AR	1.622149	1.5	0.621093
W1	HPI4	well_1	F003	1957	Nucleus AR	1.576657	1.485714	0.560105
W1	HPI4	well_1	F004	1826	Nucleus AR	1.591329	1.466667	0.723801
W1	HPI4	well_1	F005	2136	Nucleus AR	1.579655	1.48	0.58336
W1	HPI4	well_1	F006	1670	Nucleus AR	1.642267	1.5139	0.620015
W1	HPI4	well_2	F001	1519	Nucleus AR	1.638747	1.511111	0.695236
W1	HPI4	well_2	F002	1767	Nucleus AR	1.624624	1.509091	0.695576
W1	HPI4	well_2	F003	1910	Nucleus AR	1.619432	1.5	0.688051
W1	HPI4	well_2	F004	1930	Nucleus AR	1.598673	1.479167	0.615762
W1	HPI4	well_2	F005	2173	Nucleus AR	1.595398	1.468085	0.763239
W1	HPI4	well_2	F006	1819	Nucleus AR	1.586512	1.469388	0.625154
W1	HPI4	well_3	F001	1380	Nucleus AR	1.694698	1.529412	0.858806
W1	HPI4	well_3	F002	1707	Nucleus AR	1.630631	1.5	0.695642
W1	HPI4	well_3	F003	1664	Nucleus AR	1.617073	1.5	0.729283
W1	HPI4	well_3	F004	2029	Nucleus AR	1.647805	1.512821	0.702514
W1	HPI4	well_3	F005	1922	Nucleus AR	1.576562	1.5	0.500621
W1	HPI4	well_3	F006	1966	Nucleus AR	1.617227	1.5	0.65667
W1	HPI4	well_4	F001	1621	Nucleus AR	1.629309	1.5	0.692854

W1	HPI4	well_4	F002	1883	Nucleus AR	1.609907	1.490196	0.652383
W1	HPI4	well_4	F003	1990	Nucleus AR	1.587205	1.488889	0.565839
W1	HPI4	well_4	F004	1800	Nucleus AR	1.611992	1.5	0.669297
W1	HPI4	well_4	F005	1968	Nucleus AR	1.619651	1.5	0.724155
W1	HPI4	well_4	F006	1865	Nucleus AR	1.628562	1.487805	0.762271
W1	HPI4	well_5	F001	1731	Nucleus AR	1.65825	1.488372	0.827591
W1	HPI4	well_5	F002	1807	Nucleus AR	1.62788	1.487179	0.725221
W1	HPI4	well_5	F003	2045	Nucleus AR	1.571968	1.46	0.601282
W1	HPI4	well_5	F004	1765	Nucleus AR	1.558075	1.465116	0.490668
W1	HPI4	well_5	F005	1696	Nucleus AR	1.606913	1.487805	0.666862
W1	HPI4	well_5	F006	1567	Nucleus AR	1.588536	1.488889	0.611269
W1	PGE2	well_1	F001	2088	Nucleus AR	1.582236	1.488372	0.571219
W1	PGE2	well_1	F002	1885	Nucleus AR	1.639535	1.5	0.773121
W1	PGE2	well_1	F003	2089	Nucleus AR	1.592887	1.5	0.526336
W1	PGE2	well_1	F004	2227	Nucleus AR	1.588634	1.479167	0.593003
W1	PGE2	well_1	F005	2492	Nucleus AR	1.56739	1.458333	0.598127
W1	PGE2	well_1	F006	2566	Nucleus AR	1.594414	1.47619	0.715084
W1	PGE2	well_2	F001	1729	Nucleus AR	1.594708	1.491228	0.618249
W1	PGE2	well_2	F002	1816	Nucleus AR	1.58018	1.4861	0.580557
W1	PGE2	well_2	F003	1887	Nucleus AR	1.592389	1.5	0.574506
W1	PGE2	well_2	F004	2061	Nucleus AR	1.573333	1.480769	0.514783
W1	PGE2	well_2	F005	2098	Nucleus AR	1.562566	1.475595	0.514231
W1	PGE2	well_2	F006	1676	Nucleus AR	1.612305	1.48863	0.677698
W1	PGE2	well_3	F001	1851	Nucleus AR	1.610624	1.5	0.632701
W1	PGE2	well_3	F002	1798	Nucleus AR	1.618509	1.489362	0.711528
W1	PGE2	well_3	F003	1905	Nucleus AR	1.592206	1.487805	0.608312
W1	PGE2	well_3	F004	1902	Nucleus AR	1.607661	1.5	0.627026
W1	PGE2	well_3	F005	2032	Nucleus AR	1.586248	1.479167	0.604878
W1	PGE2	well_3	F006	1874	Nucleus AR	1.619413	1.479583	0.719967
W1	PGE2	well_4	F001	1627	Nucleus AR	1.616927	1.5	0.609368
W1	PGE2	well_4	F002	1758	Nucleus AR	1.578333	1.488088	0.517703
W1	PGE2	well_4	F003	1669	Nucleus AR	1.656807	1.534884	0.675578
W1	PGE2	well_4	F004	2033	Nucleus AR	1.587653	1.488889	0.591486
W1	PGE2	well_4	F005	1768	Nucleus AR	1.622013	1.52381	0.584771
W1	PGE2	well_4	F006	1816	Nucleus AR	1.606564	1.5	0.633529
W1	PGE2	well_5	F001	1629	Nucleus AR	1.614355	1.509804	0.693337
W1	PGE2	well_5	F002	1888	Nucleus AR	1.616393	1.5	0.749068
W1	PGE2	well_5	F003	1649	Nucleus AR	1.608345	1.488889	0.706529
W1	PGE2	well_5	F004	1777	Nucleus AR	1.611752	1.5	0.644384
W1	PGE2	well_5	F005	1851	Nucleus AR	1.637658	1.5	0.725131
W1	PGE2	well_5	F006	1706	Nucleus AR	1.623472	1.523268	0.591655
W2	HPI4	well_1	F001	1616	Nucleus AR	1.616188	1.5	0.670497
W2	HPI4	well_1	F002	1675	Nucleus AR	1.624493	1.512195	0.666252
W2	HPI4	well_1	F003	1796	Nucleus AR	1.635591	1.510204	0.697458
W2	HPI4	well_1	F004	1739	Nucleus AR	1.682932	1.512195	0.893454
W2	HPI4	well_1	F005	1621	Nucleus AR	1.641838	1.5	0.728094
W2	HPI4	well_1	F006	1471	Nucleus AR	1.656805	1.487179	0.870598

W2	HPI4	well_2	F001	1461	Nucleus AR	1.589842	1.478261	0.625221
W2	HPI4	well_2	F002	1570	Nucleus AR	1.612248	1.489362	0.611142
W2	HPI4	well_2	F003	1570	Nucleus AR	1.661859	1.5	0.8428
W2	HPI4	well_2	F004	1870	Nucleus AR	1.577992	1.465116	0.650175
W2	HPI4	well_2	F005	1644	Nucleus AR	1.621187	1.510204	0.643618
W2	HPI4	well_2	F006	1614	Nucleus AR	1.619874	1.487179	0.696054
W2	HPI4	well_3	F001	1751	Nucleus AR	1.597591	1.475	0.697077
W2	HPI4	well_3	F002	1471	Nucleus AR	1.622075	1.466667	0.778351
W2	HPI4	well_3	F003	1127	Nucleus AR	1.643974	1.46875	0.842959
W2	HPI4	well_3	F004	1733	Nucleus AR	1.601836	1.490196	0.583404
W2	HPI4	well_3	F005	1760	Nucleus AR	1.608616	1.488372	0.685344
W2	HPI4	well_3	F006	1654	Nucleus AR	1.643606	1.5	0.715616
W2	HPI4	well_4	F001	1628	Nucleus AR	1.594222	1.473684	0.700842
W2	HPI4	well_4	F002	1545	Nucleus AR	1.594604	1.452381	0.72035
W2	HPI4	well_4	F003	1541	Nucleus AR	1.630699	1.488372	0.700035
W2	HPI4	well_4	F004	1727	Nucleus AR	1.600827	1.464286	0.721193
W2	HPI4	well_4	F005	1511	Nucleus AR	1.641663	1.5	0.742002
W2	HPI4	well_4	F006	1590	Nucleus AR	1.650767	1.5	0.825759
W2	HPI4	well_5	F001	1496	Nucleus AR	1.610309	1.473684	0.744757
W2	HPI4	well_5	F002	1370	Nucleus AR	1.642773	1.496032	0.784911
W2	HPI4	well_5	F003	1387	Nucleus AR	1.603697	1.468085	0.667306
W2	HPI4	well_5	F004	1683	Nucleus AR	1.611868	1.487805	0.679775
W2	HPI4	well_5	F005	1752	Nucleus AR	1.61579	1.477273	0.695967
W2	HPI4	well_5	F006	1445	Nucleus AR	1.643629	1.510638	0.682209
W2	PGE2	well_1	F001	2222	Nucleus AR	1.530617	1.410256	0.614922
W2	PGE2	well_1	F002	1940	Nucleus AR	1.48863	1.368421	0.590003
W2	PGE2	well_1	F003	2718	Nucleus AR	1.518491	1.421053	0.521446
W2	PGE2	well_1	F004	2149	Nucleus AR	1.491194	1.4	0.488591
W2	PGE2	well_1	F005	2692	Nucleus AR	1.503924	1.394737	0.549499
W2	PGE2	well_1	F006	2744	Nucleus AR	1.46269	1.361111	0.532978
W2	PGE2	well_2	F001	2270	Nucleus AR	1.470781	1.382353	0.472464
W2	PGE2	well_2	F002	2877	Nucleus AR	1.469037	1.382979	0.43172
W2	PGE2	well_2	F003	3288	Nucleus AR	1.509301	1.393939	0.579195
W2	PGE2	well_2	F004	2973	Nucleus AR	1.507325	1.4	0.561656
W2	PGE2	well_2	F005	2444	Nucleus AR	1.460431	1.368421	0.484516
W2	PGE2	well_2	F006	2304	Nucleus AR	1.506491	1.404762	0.50844
W2	PGE2	well_3	F001	2012	Nucleus AR	1.49146	1.378378	0.571939
W2	PGE2	well_3	F002	3189	Nucleus AR	1.514229	1.411765	0.515392
W2	PGE2	well_3	F003	2876	Nucleus AR	1.456021	1.365854	0.521403
W2	PGE2	well_3	F004	2935	Nucleus AR	1.458725	1.371429	0.491798
W2	PGE2	well_3	F005	2421	Nucleus AR	1.46929	1.375	0.460056
W2	PGE2	well_3	F006	2111	Nucleus AR	1.491196	1.375	0.574347
W2	PGE2	well_4	F001	2452	Nucleus AR	1.479795	1.384615	0.504973
W2	PGE2	well_4	F002	3055	Nucleus AR	1.466554	1.371429	0.475569
W2	PGE2	well_4	F003	2843	Nucleus AR	1.472168	1.371429	0.554549
W2	PGE2	well_4	F004	3219	Nucleus AR	1.464785	1.37931	0.420467
W2	PGE2	well_4	F005	2886	Nucleus AR	1.47094	1.371429	0.501716

W2	PGE2	well_4	F006	2381	Nucleus AR	1.496681	1.384615	0.580287
W2	PGE2	well_5	F001	2804	Nucleus AR	1.501799	1.4	0.533655
W2	PGE2	well_5	F002	3207	Nucleus AR	1.473063	1.384615	0.532433
W2	PGE2	well_5	F003	2872	Nucleus AR	1.500306	1.4	0.49704
W2	PGE2	well_5	F004	2580	Nucleus AR	1.460117	1.365854	0.488421
W2	PGE2	well_5	F005	2615	Nucleus AR	1.458562	1.368421	0.525781
W2	PGE2	well_5	F006	2953	Nucleus AR	1.451936	1.368421	0.443331
W3	HPI4	well_1	F001	798	Nucleus AR	1.677051	1.512195	0.79556
W3	HPI4	well_1	F002	1010	Nucleus AR	1.739678	1.534109	0.902809
W3	HPI4	well_1	F003	908	Nucleus AR	1.741108	1.518065	0.88484
W3	HPI4	well_1	F004	1248	Nucleus AR	1.739195	1.514286	0.868818
W3	HPI4	well_1	F005	1235	Nucleus AR	1.772289	1.548387	0.960981
W3	HPI4	well_1	F006	914	Nucleus AR	1.82033	1.55	1.00422
W3	HPI4	well_2	F001	797	Nucleus AR	1.838278	1.536585	1.242246
W3	HPI4	well_2	F002	971	Nucleus AR	1.785326	1.534884	1.016996
W3	HPI4	well_2	F003	933	Nucleus AR	1.768675	1.52459	0.962046
W3	HPI4	well_2	F004	1129	Nucleus AR	1.745256	1.552632	0.873619
W3	HPI4	well_2	F005	1253	Nucleus AR	1.749421	1.520833	0.90834
W3	HPI4	well_2	F006	1058	Nucleus AR	1.74152	1.5	0.924466
W3	HPI4	well_3	F001	795	Nucleus AR	1.742863	1.527778	0.983423
W3	HPI4	well_3	F002	885	Nucleus AR	1.740514	1.531915	0.818969
W3	HPI4	well_3	F003	621	Nucleus AR	1.715035	1.512195	0.818241
W3	HPI4	well_3	F004	986	Nucleus AR	1.726178	1.5	0.852339
W3	HPI4	well_3	F005	1121	Nucleus AR	1.703715	1.510638	0.864259
W3	HPI4	well_3	F006	1047	Nucleus AR	1.701281	1.5	0.868815
W3	HPI4	well_4	F001	722	Nucleus AR	1.699267	1.5	0.843503
W3	HPI4	well_4	F002	918	Nucleus AR	1.735151	1.544657	0.934126
W3	HPI4	well_4	F003	708	Nucleus AR	1.736877	1.525658	0.931725
W3	HPI4	well_4	F004	785	Nucleus AR	1.792194	1.489362	1.086268
W3	HPI4	well_4	F005	1210	Nucleus AR	1.659072	1.488372	0.840394
W3	HPI4	well_4	F006	1129	Nucleus AR	1.719854	1.5	0.98949
W3	HPI4	well_5	F001	712	Nucleus AR	1.744435	1.537749	0.851038
W3	HPI4	well_5	F002	558	Nucleus AR	1.66336	1.487805	0.834019
W3	HPI4	well_5	F003	812	Nucleus AR	1.729762	1.489579	1.089596
W3	HPI4	well_5	F004	517	Nucleus AR	1.79465	1.52	1.00976
W3	HPI4	well_5	F005	976	Nucleus AR	1.659815	1.47619	0.821275
W3	HPI4	well_5	F006	931	Nucleus AR	1.7144	1.52381	0.803965
W3	PGE2	well_1	F001	2659	Nucleus AR	1.511875	1.382353	0.584475
W3	PGE2	well_1	F002	3405	Nucleus AR	1.504348	1.378378	0.613623
W3	PGE2	well_1	F003	3522	Nucleus AR	1.476417	1.368421	0.489293
W3	PGE2	well_1	F004	3028	Nucleus AR	1.471321	1.355197	0.610171
W3	PGE2	well_1	F005	3384	Nucleus AR	1.510998	1.375	0.664639
W3	PGE2	well_1	F006	2846	Nucleus AR	1.493494	1.390774	0.574383
W3	PGE2	well_2	F001	2912	Nucleus AR	1.533883	1.372093	0.663851
W3	PGE2	well_2	F002	2973	Nucleus AR	1.468532	1.358974	0.57567
W3	PGE2	well_2	F003	3367	Nucleus AR	1.470867	1.368421	0.526511
W3	PGE2	well_2	F004	3533	Nucleus AR	1.484999	1.382353	0.504741

W3	PGE2	well_2	F005	3705	Nucleus AR	1.501787	1.382353	0.562572
W3	PGE2	well_2	F006	3844	Nucleus AR	1.450074	1.342105	0.477509
W3	PGE2	well_3	F001	3127	Nucleus AR	1.530271	1.368421	0.693889
W3	PGE2	well_3	F002	2777	Nucleus AR	1.539842	1.404762	0.613274
W3	PGE2	well_3	F003	3810	Nucleus AR	1.483727	1.361111	0.578794
W3	PGE2	well_3	F004	3332	Nucleus AR	1.488828	1.361111	0.657109
W3	PGE2	well_3	F005	3851	Nucleus AR	1.460382	1.342857	0.580842
W3	PGE2	well_3	F006	3599	Nucleus AR	1.479131	1.368421	0.557714
W3	PGE2	well_4	F001	2774	Nucleus AR	1.476616	1.342105	0.603439
W3	PGE2	well_4	F002	3347	Nucleus AR	1.480983	1.352941	0.580504
W3	PGE2	well_4	F003	2729	Nucleus AR	1.481179	1.357143	0.618876
W3	PGE2	well_4	F004	3120	Nucleus AR	1.515534	1.375	0.667109
W3	PGE2	well_4	F005	3555	Nucleus AR	1.521169	1.380952	0.679388
W3	PGE2	well_4	F006	3216	Nucleus AR	1.503417	1.384615	0.585657
W3	PGE2	well_5	F001	2321	Nucleus AR	1.537083	1.404762	0.642793
W3	PGE2	well_5	F002	2453	Nucleus AR	1.514251	1.377778	0.628967
W3	PGE2	well_5	F003	3024	Nucleus AR	1.496889	1.361111	0.61507
W3	PGE2	well_5	F004	2780	Nucleus AR	1.459513	1.351351	0.535122
W3	PGE2	well_5	F005	3314	Nucleus AR	1.469736	1.354839	0.583121
W3	PGE2	well_5	F006	2620	Nucleus AR	1.467572	1.35	0.587336
W4	HPI4	well_1	F001	540	Nucleus AR	1.922259	1.56125	1.278334
W4	HPI4	well_1	F002	888	Nucleus AR	1.749469	1.510875	1.015964
W4	HPI4	well_1	F003	835	Nucleus AR	1.73774	1.5	0.943216
W4	HPI4	well_1	F004	991	Nucleus AR	1.712771	1.521739	0.874057
W4	HPI4	well_1	F005	1090	Nucleus AR	1.767434	1.511912	0.964122
W4	HPI4	well_1	F006	991	Nucleus AR	1.761411	1.551724	0.859664
W4	HPI4	well_2	F001	653	Nucleus AR	1.753284	1.522727	0.950281
W4	HPI4	well_2	F002	918	Nucleus AR	1.646246	1.488372	0.682765
W4	HPI4	well_2	F003	956	Nucleus AR	1.646197	1.489125	0.80614
W4	HPI4	well_2	F004	1031	Nucleus AR	1.701811	1.525	0.76933
W4	HPI4	well_2	F005	1024	Nucleus AR	1.753853	1.514333	0.982044
W4	HPI4	well_2	F006	933	Nucleus AR	1.725278	1.543478	0.800676
W4	HPI4	well_3	F001	759	Nucleus AR	1.738727	1.5	0.980007
W4	HPI4	well_3	F002	840	Nucleus AR	1.689997	1.503521	0.855008
W4	HPI4	well_3	F003	927	Nucleus AR	1.758307	1.5	1.035101
W4	HPI4	well_3	F004	1041	Nucleus AR	1.735846	1.509804	0.969455
W4	HPI4	well_3	F005	1065	Nucleus AR	1.682989	1.486486	0.903292
W4	HPI4	well_3	F006	813	Nucleus AR	1.747111	1.522727	0.882886
W4	HPI4	well_4	F001	772	Nucleus AR	1.682109	1.490181	0.91588
W4	HPI4	well_4	F002	962	Nucleus AR	1.729091	1.5	0.978334
W4	HPI4	well_4	F003	701	Nucleus AR	1.758886	1.520833	1.057536
W4	HPI4	well_4	F004	1062	Nucleus AR	1.685575	1.487805	0.837769
W4	HPI4	well_4	F005	1118	Nucleus AR	1.726824	1.476732	0.933312
W4	HPI4	well_4	F006	845	Nucleus AR	1.747855	1.47619	0.983591
W4	HPI4	well_5	F001	791	Nucleus AR	1.643315	1.478261	0.715703
W4	HPI4	well_5	F002	598	Nucleus AR	1.765013	1.504545	0.989759
W4	HPI4	well_5	F003	752	Nucleus AR	1.734063	1.548319	0.90296

W4	HPI4	well_5	F004	949	Nucleus AR	1.726749	1.5	0.939415
W4	HPI4	well_5	F005	1195	Nucleus AR	1.738021	1.511628	0.932081
W4	HPI4	well_5	F006	1013	Nucleus AR	1.719219	1.5	0.988454
W4	PGE2	well_1	F001	2025	Nucleus AR	1.468828	1.361111	0.482507
W4	PGE2	well_1	F002	1830	Nucleus AR	1.518026	1.370497	0.670865
W4	PGE2	well_1	F003	2816	Nucleus AR	1.489279	1.375	0.596668
W4	PGE2	well_1	F004	2679	Nucleus AR	1.459617	1.347826	0.552233
W4	PGE2	well_1	F005	2927	Nucleus AR	1.479851	1.371429	0.502395
W4	PGE2	well_1	F006	2858	Nucleus AR	1.482865	1.358974	0.551196
W4	PGE2	well_2	F001	2324	Nucleus AR	1.55046	1.428571	0.624996
W4	PGE2	well_2	F002	2806	Nucleus AR	1.49109	1.363636	0.605834
W4	PGE2	well_2	F003	2502	Nucleus AR	1.503749	1.390244	0.598855
W4	PGE2	well_2	F004	2867	Nucleus AR	1.480128	1.371429	0.533619
W4	PGE2	well_2	F005	3262	Nucleus AR	1.471292	1.361407	0.522834
W4	PGE2	well_2	F006	2745	Nucleus AR	1.452972	1.357143	0.49327
W4	PGE2	well_3	F001	2165	Nucleus AR	1.484455	1.375	0.583315
W4	PGE2	well_3	F002	3094	Nucleus AR	1.451966	1.357143	0.521495
W4	PGE2	well_3	F003	3177	Nucleus AR	1.460201	1.35	0.534605
W4	PGE2	well_3	F004	3019	Nucleus AR	1.506975	1.380952	0.608576
W4	PGE2	well_3	F005	2629	Nucleus AR	1.556958	1.405405	0.725402
W4	PGE2	well_3	F006	2911	Nucleus AR	1.458316	1.358974	0.534862
W4	PGE2	well_4	F001	2186	Nucleus AR	1.528201	1.379665	0.616125
W4	PGE2	well_4	F002	2767	Nucleus AR	1.473172	1.35	0.584684
W4	PGE2	well_4	F003	2924	Nucleus AR	1.513702	1.389566	0.625479
W4	PGE2	well_4	F004	2783	Nucleus AR	1.50201	1.375	0.582405
W4	PGE2	well_4	F005	3493	Nucleus AR	1.470196	1.365854	0.502168
W4	PGE2	well_4	F006	2292	Nucleus AR	1.477294	1.368421	0.611276
W4	PGE2	well_5	F001	1871	Nucleus AR	1.559176	1.424242	0.646664
W4	PGE2	well_5	F002	2315	Nucleus AR	1.534301	1.375	0.676577
W4	PGE2	well_5	F003	2729	Nucleus AR	1.477096	1.34375	0.617074
W4	PGE2	well_5	F004	2705	Nucleus AR	1.496545	1.342857	0.64335
W4	PGE2	well_5	F005	2441	Nucleus AR	1.487138	1.35	0.619561
W4	PGE2	well_5	F006	2162	Nucleus AR	1.532109	1.351351	0.799432
W1	HPI4	well_1	F001	1779	Nucleus Area	107.7141	103.935	39.86734
W1	HPI4	well_1	F002	1534	Nucleus Area	103.641	96.33	43.69243
W1	HPI4	well_1	F003	1957	Nucleus Area	97.11685	92.80917	35.06794
W1	HPI4	well_1	F004	1826	Nucleus Area	105.7413	102.1511	38.57593
W1	HPI4	well_1	F005	2136	Nucleus Area	99.10783	96.18917	33.62239
W1	HPI4	well_1	F006	1670	Nucleus Area	103.1688	100.5315	37.86967
W1	HPI4	well_2	F001	1519	Nucleus Area	106.8347	102.5267	41.71585
W1	HPI4	well_2	F002	1767	Nucleus Area	106.4822	103.8881	38.16651
W1	HPI4	well_2	F003	1910	Nucleus Area	102.9026	99.21708	38.02764
W1	HPI4	well_2	F004	1930	Nucleus Area	104.3242	102.0103	34.71524
W1	HPI4	well_2	F005	2173	Nucleus Area	97.7522	94.64	35.04762
W1	HPI4	well_2	F006	1819	Nucleus Area	100.8865	96.09528	38.90877
W1	HPI4	well_3	F001	1380	Nucleus Area	109.5768	106.2118	44.30843
W1	HPI4	well_3	F002	1707	Nucleus Area	103.2714	102.6675	34.84455

W1	HPI4	well_3	F003	1664	Nucleus Area	102.8869	98.51292	39.24445
W1	HPI4	well_3	F004	2029	Nucleus Area	101.1018	97.73833	36.36217
W1	HPI4	well_3	F005	1922	Nucleus Area	102.5028	96.51778	38.14683
W1	HPI4	well_3	F006	1966	Nucleus Area	99.89802	95.53194	40.10582
W1	HPI4	well_4	F001	1621	Nucleus Area	102.3002	98.25472	37.99658
W1	HPI4	well_4	F002	1883	Nucleus Area	98.28015	94.73389	34.30879
W1	HPI4	well_4	F003	1990	Nucleus Area	102.0327	96.89333	38.22872
W1	HPI4	well_4	F004	1800	Nucleus Area	104.7202	101.8225	37.59952
W1	HPI4	well_4	F005	1968	Nucleus Area	101.3501	96.79944	41.05944
W1	HPI4	well_4	F006	1865	Nucleus Area	100.9487	96.51778	38.50736
W1	HPI4	well_5	F001	1731	Nucleus Area	99.92175	95.76667	40.50752
W1	HPI4	well_5	F002	1807	Nucleus Area	100.227	96.65861	37.38853
W1	HPI4	well_5	F003	2045	Nucleus Area	97.16862	92.38667	37.04546
W1	HPI4	well_5	F004	1765	Nucleus Area	98.29268	93.13778	37.01425
W1	HPI4	well_5	F005	1696	Nucleus Area	107.3417	102.2215	42.13357
W1	HPI4	well_5	F006	1567	Nucleus Area	104.9247	99.85083	40.28468
W1	PGE2	well_1	F001	2088	Nucleus Area	101.3214	96.40042	37.34132
W1	PGE2	well_1	F002	1885	Nucleus Area	106.9448	103.935	41.37852
W1	PGE2	well_1	F003	2089	Nucleus Area	103.7103	101.5878	34.90098
W1	PGE2	well_1	F004	2227	Nucleus Area	100.8502	95.9075	38.13396
W1	PGE2	well_1	F005	2492	Nucleus Area	94.30822	88.39639	35.56723
W1	PGE2	well_1	F006	2566	Nucleus Area	96.51604	90.46194	36.81482
W1	PGE2	well_2	F001	1729	Nucleus Area	102.4816	99.33444	35.60186
W1	PGE2	well_2	F002	1816	Nucleus Area	101.5291	96.28306	36.87683
W1	PGE2	well_2	F003	1887	Nucleus Area	99.94669	94.87472	36.55129
W1	PGE2	well_2	F004	2061	Nucleus Area	99.67037	95.01556	37.96238
W1	PGE2	well_2	F005	2098	Nucleus Area	102.0097	98.95889	35.211
W1	PGE2	well_2	F006	1676	Nucleus Area	104.347	100.2968	38.43502
W1	PGE2	well_3	F001	1851	Nucleus Area	106.327	102.4797	38.35937
W1	PGE2	well_3	F002	1798	Nucleus Area	106.0759	100.4376	41.45942
W1	PGE2	well_3	F003	1905	Nucleus Area	102.4528	98.48944	35.3542
W1	PGE2	well_3	F004	1902	Nucleus Area	106.9404	105.3199	38.62236
W1	PGE2	well_3	F005	2032	Nucleus Area	100.5951	94.00625	40.40915
W1	PGE2	well_3	F006	1874	Nucleus Area	103.5789	99.78042	37.96944
W1	PGE2	well_4	F001	1627	Nucleus Area	109.3566	106.1414	39.7504
W1	PGE2	well_4	F002	1758	Nucleus Area	102.5582	100.156	33.4735
W1	PGE2	well_4	F003	1669	Nucleus Area	105.3645	101.3061	40.63817
W1	PGE2	well_4	F004	2033	Nucleus Area	98.24525	92.5275	36.68533
W1	PGE2	well_4	F005	1768	Nucleus Area	105.5306	101.9399	38.55828
W1	PGE2	well_4	F006	1816	Nucleus Area	101.3702	96.42389	40.01692
W1	PGE2	well_5	F001	1629	Nucleus Area	107.0642	101.4939	41.3566
W1	PGE2	well_5	F002	1888	Nucleus Area	105.3841	100.6724	39.75875
W1	PGE2	well_5	F003	1649	Nucleus Area	105.9359	103.7003	37.07553
W1	PGE2	well_5	F004	1777	Nucleus Area	106.5762	104.1697	38.01135
W1	PGE2	well_5	F005	1851	Nucleus Area	103.2991	100.2733	37.21598
W1	PGE2	well_5	F006	1706	Nucleus Area	102.7265	98.67722	38.07112
W2	HPI4	well_1	F001	1616	Nucleus Area	89.39262	92.41014	39.47686

W2	HPI4	well_1	F002	1675	Nucleus Area	99.16771	98.25472	37.70138
W2	HPI4	well_1	F003	1796	Nucleus Area	90.89613	90.72014	36.00458
W2	HPI4	well_1	F004	1739	Nucleus Area	94.90434	92.85611	38.44557
W2	HPI4	well_1	F005	1621	Nucleus Area	95.85459	98.02	37.26174
W2	HPI4	well_1	F006	1471	Nucleus Area	97.86161	99.61611	38.17943
W2	HPI4	well_2	F001	1461	Nucleus Area	90.36565	92.43361	43.56403
W2	HPI4	well_2	F002	1570	Nucleus Area	96.59806	98.39556	36.65873
W2	HPI4	well_2	F003	1570	Nucleus Area	95.76084	96.35347	37.21591
W2	HPI4	well_2	F004	1870	Nucleus Area	90.47553	91.6825	35.68266
W2	HPI4	well_2	F005	1644	Nucleus Area	96.19048	97.94958	37.46872
W2	HPI4	well_2	F006	1614	Nucleus Area	93.79119	96.28306	37.724
W2	HPI4	well_3	F001	1751	Nucleus Area	86.22676	89.94556	39.06568
W2	HPI4	well_3	F002	1471	Nucleus Area	93.6804	94.49917	38.79987
W2	HPI4	well_3	F003	1127	Nucleus Area	100.0146	101.5878	45.48251
W2	HPI4	well_3	F004	1733	Nucleus Area	93.97427	94.40528	36.14872
W2	HPI4	well_3	F005	1760	Nucleus Area	89.29103	88.53722	37.55194
W2	HPI4	well_3	F006	1654	Nucleus Area	93.26805	94.94514	38.71754
W2	HPI4	well_4	F001	1628	Nucleus Area	82.542	86.37778	35.87274
W2	HPI4	well_4	F002	1545	Nucleus Area	86.28665	85.29806	35.57724
W2	HPI4	well_4	F003	1541	Nucleus Area	92.53475	95.25028	34.29017
W2	HPI4	well_4	F004	1727	Nucleus Area	89.9486	89.9925	35.20644
W2	HPI4	well_4	F005	1511	Nucleus Area	94.25817	96.33	40.02721
W2	HPI4	well_4	F006	1590	Nucleus Area	97.40612	94.31139	39.51522
W2	HPI4	well_5	F001	1496	Nucleus Area	90.6169	90.95486	39.93362
W2	HPI4	well_5	F002	1370	Nucleus Area	98.50757	100.5315	41.1559
W2	HPI4	well_5	F003	1387	Nucleus Area	98.42798	98.53639	36.63786
W2	HPI4	well_5	F004	1683	Nucleus Area	94.47231	95.9075	37.89251
W2	HPI4	well_5	F005	1752	Nucleus Area	89.79341	93.70111	37.43812
W2	HPI4	well_5	F006	1445	Nucleus Area	94.20987	98.25472	38.0489
W2	PGE2	well_1	F001	2222	Nucleus Area	74.67217	73.37417	26.14372
W2	PGE2	well_1	F002	1940	Nucleus Area	71.80668	69.59514	24.0338
W2	PGE2	well_1	F003	2718	Nucleus Area	75.35982	73.86708	23.85187
W2	PGE2	well_1	F004	2149	Nucleus Area	75.55917	73.23333	24.24484
W2	PGE2	well_1	F005	2692	Nucleus Area	72.96623	71.4025	24.09524
W2	PGE2	well_1	F006	2744	Nucleus Area	68.21993	68.77361	22.2684
W2	PGE2	well_2	F001	2270	Nucleus Area	71.11686	70.72181	22.89771
W2	PGE2	well_2	F002	2877	Nucleus Area	70.02063	68.06944	23.40891
W2	PGE2	well_2	F003	3288	Nucleus Area	70.8878	69.21958	24.11391
W2	PGE2	well_2	F004	2973	Nucleus Area	72.19912	69.43083	24.66124
W2	PGE2	well_2	F005	2444	Nucleus Area	71.1196	69.29	23.63085
W2	PGE2	well_2	F006	2304	Nucleus Area	72.5951	72.88125	22.75043
W2	PGE2	well_3	F001	2012	Nucleus Area	71.60626	68.58583	28.1405
W2	PGE2	well_3	F002	3189	Nucleus Area	72.80336	71.12083	24.51628
W2	PGE2	well_3	F003	2876	Nucleus Area	72.77479	70.51056	22.67507
W2	PGE2	well_3	F004	2935	Nucleus Area	67.54731	66.47333	20.55959
W2	PGE2	well_3	F005	2421	Nucleus Area	69.97557	68.49194	22.55108
W2	PGE2	well_3	F006	2111	Nucleus Area	72.81123	69.7125	25.97451

W2	PGE2	well_4	F001	2452	Nucleus Area	74.48176	71.825	26.31713
W2	PGE2	well_4	F002	3055	Nucleus Area	70.77907	68.91444	23.19506
W2	PGE2	well_4	F003	2843	Nucleus Area	70.59312	68.8675	21.36146
W2	PGE2	well_4	F004	3219	Nucleus Area	74.18471	71.54333	24.87135
W2	PGE2	well_4	F005	2886	Nucleus Area	71.33387	68.77361	23.89786
W2	PGE2	well_4	F006	2381	Nucleus Area	73.62409	71.54333	26.22604
W2	PGE2	well_5	F001	2804	Nucleus Area	72.49704	69.99417	24.29734
W2	PGE2	well_5	F002	3207	Nucleus Area	72.49361	71.16778	22.13126
W2	PGE2	well_5	F003	2872	Nucleus Area	73.47924	71.14431	23.33123
W2	PGE2	well_5	F004	2580	Nucleus Area	69.33614	67.76431	23.76467
W2	PGE2	well_5	F005	2615	Nucleus Area	70.00824	69.43083	22.47151
W2	PGE2	well_5	F006	2953	Nucleus Area	70.65667	69.10222	23.2801
W3	HPI4	well_1	F001	798	Nucleus Area	99.40986	101.7521	44.62267
W3	HPI4	well_1	F002	1010	Nucleus Area	99.22512	101.2122	41.08651
W3	HPI4	well_1	F003	908	Nucleus Area	96.69728	101.9633	44.06601
W3	HPI4	well_1	F004	1248	Nucleus Area	90.42877	93.16125	35.3169
W3	HPI4	well_1	F005	1235	Nucleus Area	96.78964	98.4425	40.27079
W3	HPI4	well_1	F006	914	Nucleus Area	98.469	103.5594	42.25551
W3	HPI4	well_2	F001	797	Nucleus Area	94.46377	98.30167	44.0254
W3	HPI4	well_2	F002	971	Nucleus Area	97.84532	102.5267	39.82675
W3	HPI4	well_2	F003	933	Nucleus Area	102.1968	102.8553	46.25567
W3	HPI4	well_2	F004	1129	Nucleus Area	97.33696	99.42833	38.13842
W3	HPI4	well_2	F005	1253	Nucleus Area	94.25924	98.20778	40.58018
W3	HPI4	well_2	F006	1058	Nucleus Area	99.12515	100.109	41.00043
W3	HPI4	well_3	F001	795	Nucleus Area	99.17082	104.1697	41.69073
W3	HPI4	well_3	F002	885	Nucleus Area	101.0527	105.3433	38.33076
W3	HPI4	well_3	F003	621	Nucleus Area	103.6678	104.1697	42.01239
W3	HPI4	well_3	F004	986	Nucleus Area	91.22286	95.74319	40.48474
W3	HPI4	well_3	F005	1121	Nucleus Area	92.85176	91.02528	45.58451
W3	HPI4	well_3	F006	1047	Nucleus Area	91.70317	91.21306	40.96415
W3	HPI4	well_4	F001	722	Nucleus Area	99.22294	102.5267	42.75458
W3	HPI4	well_4	F002	918	Nucleus Area	104.9151	104.8269	44.14966
W3	HPI4	well_4	F003	708	Nucleus Area	106.3471	107.6201	43.22872
W3	HPI4	well_4	F004	785	Nucleus Area	94.52674	99.2875	43.32348
W3	HPI4	well_4	F005	1210	Nucleus Area	97.01644	95.57889	38.70975
W3	HPI4	well_4	F006	1129	Nucleus Area	95.60716	95.81361	42.20635
W3	HPI4	well_5	F001	712	Nucleus Area	104.3442	107.2211	52.20238
W3	HPI4	well_5	F002	558	Nucleus Area	100.8409	104.8269	39.92426
W3	HPI4	well_5	F003	812	Nucleus Area	96.54142	100.954	40.21697
W3	HPI4	well_5	F004	517	Nucleus Area	102.7154	103.2308	49.3655
W3	HPI4	well_5	F005	976	Nucleus Area	98.61897	100.4846	39.76231
W3	HPI4	well_5	F006	931	Nucleus Area	101.5777	103.7942	42.75003
W3	PGE2	well_1	F001	2659	Nucleus Area	71.47586	68.82056	24.68037
W3	PGE2	well_1	F002	3405	Nucleus Area	70.03139	69.43083	23.21653
W3	PGE2	well_1	F003	3522	Nucleus Area	70.19509	67.78778	22.85587
W3	PGE2	well_1	F004	3028	Nucleus Area	66.08841	62.53	24.22634
W3	PGE2	well_1	F005	3384	Nucleus Area	69.13574	65.65181	24.05119

W3	PGE2	well_1	F006	2846	Nucleus Area	73.03063	70.72181	23.31753
W3	PGE2	well_2	F001	2912	Nucleus Area	72.34737	69.61861	23.59183
W3	PGE2	well_2	F002	2973	Nucleus Area	71.90315	69.33694	23.6346
W3	PGE2	well_2	F003	3367	Nucleus Area	69.09511	67.22444	22.41414
W3	PGE2	well_2	F004	3533	Nucleus Area	71.12554	67.83472	24.29911
W3	PGE2	well_2	F005	3705	Nucleus Area	71.44465	67.50611	25.24045
W3	PGE2	well_2	F006	3844	Nucleus Area	69.88711	68.75014	20.19212
W3	PGE2	well_3	F001	3127	Nucleus Area	71.46209	66.89583	27.55926
W3	PGE2	well_3	F002	2777	Nucleus Area	74.1885	72.2475	26.86814
W3	PGE2	well_3	F003	3810	Nucleus Area	68.83866	64.92417	24.22781
W3	PGE2	well_3	F004	3332	Nucleus Area	71.14573	65.86306	26.25971
W3	PGE2	well_3	F005	3851	Nucleus Area	69.33751	65.86306	24.64552
W3	PGE2	well_3	F006	3599	Nucleus Area	74.22455	71.77806	24.23326
W3	PGE2	well_4	F001	2774	Nucleus Area	69.50623	67.78778	23.76794
W3	PGE2	well_4	F002	3347	Nucleus Area	70.69968	66.14472	26.82474
W3	PGE2	well_4	F003	2729	Nucleus Area	71.82368	69.33694	24.42541
W3	PGE2	well_4	F004	3120	Nucleus Area	72.44068	69.24306	26.35971
W3	PGE2	well_4	F005	3555	Nucleus Area	71.89006	69.75944	24.46786
W3	PGE2	well_4	F006	3216	Nucleus Area	72.66946	71.02694	23.37076
W3	PGE2	well_5	F001	2321	Nucleus Area	71.24336	69.38389	26.25398
W3	PGE2	well_5	F002	2453	Nucleus Area	73.13677	71.07389	25.63588
W3	PGE2	well_5	F003	3024	Nucleus Area	69.54056	67.38875	25.3971
W3	PGE2	well_5	F004	2780	Nucleus Area	68.67575	65.74569	25.49293
W3	PGE2	well_5	F005	3314	Nucleus Area	68.5779	65.86306	23.05692
W3	PGE2	well_5	F006	2620	Nucleus Area	72.00197	69.38389	23.67764
W4	HPI4	well_1	F001	540	Nucleus Area	102.1874	107.4793	51.879
W4	HPI4	well_1	F002	888	Nucleus Area	93.69228	96.98722	42.70109
W4	HPI4	well_1	F003	835	Nucleus Area	95.1886	96.79944	38.36756
W4	HPI4	well_1	F004	991	Nucleus Area	97.45363	99.05278	37.13905
W4	HPI4	well_1	F005	1090	Nucleus Area	92.95241	94.35833	46.04762
W4	HPI4	well_1	F006	991	Nucleus Area	96.90338	98.58333	43.23918
W4	HPI4	well_2	F001	653	Nucleus Area	102.3037	104.8739	48.27126
W4	HPI4	well_2	F002	918	Nucleus Area	93.32642	95.27375	38.26164
W4	HPI4	well_2	F003	956	Nucleus Area	98.91514	99.61611	42.7519
W4	HPI4	well_2	F004	1031	Nucleus Area	98.53347	99.2875	37.70983
W4	HPI4	well_2	F005	1024	Nucleus Area	94.52511	98.30167	40.14398
W4	HPI4	well_2	F006	933	Nucleus Area	100.9128	101.4	39.16585
W4	HPI4	well_3	F001	759	Nucleus Area	101.0428	103.3247	44.54295
W4	HPI4	well_3	F002	840	Nucleus Area	102.0972	104.1697	40.30719
W4	HPI4	well_3	F003	927	Nucleus Area	102.5011	104.5453	46.41374
W4	HPI4	well_3	F004	1041	Nucleus Area	94.09912	97.78528	36.59783
W4	HPI4	well_3	F005	1065	Nucleus Area	93.11644	95.29722	36.17173
W4	HPI4	well_3	F006	813	Nucleus Area	96.64279	96.7525	43.46352
W4	HPI4	well_4	F001	772	Nucleus Area	107.3039	105.9536	49.41479
W4	HPI4	well_4	F002	962	Nucleus Area	103.2563	105.5546	43.23377
W4	HPI4	well_4	F003	701	Nucleus Area	109.7031	112.7606	44.75699
W4	HPI4	well_4	F004	1062	Nucleus Area	95.2402	99.49875	40.50474

W4	HPI4	well_4	F005	1118	Nucleus Area	91.12609	94.49917	41.11226
W4	HPI4	well_4	F006	845	Nucleus Area	97.48889	100.4142	44.67649
W4	HPI4	well_5	F001	791	Nucleus Area	108.1713	108.3008	46.81033
W4	HPI4	well_5	F002	598	Nucleus Area	111.5964	114.8026	47.37106
W4	HPI4	well_5	F003	752	Nucleus Area	111.507	108.8642	49.11746
W4	HPI4	well_5	F004	949	Nucleus Area	107.4803	108.6294	46.33532
W4	HPI4	well_5	F005	1195	Nucleus Area	95.45491	100.3672	40.35054
W4	HPI4	well_5	F006	1013	Nucleus Area	94.92973	98.16083	38.41751
W4	PGE2	well_1	F001	2025	Nucleus Area	69.89191	69.7125	20.76411
W4	PGE2	well_1	F002	1830	Nucleus Area	69.62926	70.18194	21.30168
W4	PGE2	well_1	F003	2816	Nucleus Area	70.88344	69.75944	22.21588
W4	PGE2	well_1	F004	2679	Nucleus Area	66.4771	65.29972	21.5207
W4	PGE2	well_1	F005	2927	Nucleus Area	68.02285	67.41222	24.12928
W4	PGE2	well_1	F006	2858	Nucleus Area	71.02658	69.66556	22.76866
W4	PGE2	well_2	F001	2324	Nucleus Area	72.0374	72.71694	24.75911
W4	PGE2	well_2	F002	2806	Nucleus Area	68.7322	67.45917	22.27094
W4	PGE2	well_2	F003	2502	Nucleus Area	68.94696	68.13986	23.61795
W4	PGE2	well_2	F004	2867	Nucleus Area	66.86148	65.4875	22.56993
W4	PGE2	well_2	F005	3262	Nucleus Area	66.90074	65.86306	21.40347
W4	PGE2	well_2	F006	2745	Nucleus Area	69.79195	69.52472	20.59292
W4	PGE2	well_3	F001	2165	Nucleus Area	70.05117	70.22889	22.74166
W4	PGE2	well_3	F002	3094	Nucleus Area	69.2881	68.96139	21.01571
W4	PGE2	well_3	F003	3177	Nucleus Area	67.47643	66.94278	21.30574
W4	PGE2	well_3	F004	3019	Nucleus Area	67.79274	65.81611	22.6585
W4	PGE2	well_3	F005	2629	Nucleus Area	70.01059	68.25722	23.49648
W4	PGE2	well_3	F006	2911	Nucleus Area	71.02906	70.18194	21.06546
W4	PGE2	well_4	F001	2186	Nucleus Area	66.41232	65.53444	20.60317
W4	PGE2	well_4	F002	2767	Nucleus Area	67.00693	65.53444	22.33442
W4	PGE2	well_4	F003	2924	Nucleus Area	67.87638	67.01319	21.2455
W4	PGE2	well_4	F004	2783	Nucleus Area	67.44827	64.68944	22.88187
W4	PGE2	well_4	F005	3493	Nucleus Area	67.14799	65.39361	20.29133
W4	PGE2	well_4	F006	2292	Nucleus Area	68.91399	67.97556	21.70324
W4	PGE2	well_5	F001	1871	Nucleus Area	70.9304	71.07389	23.6149
W4	PGE2	well_5	F002	2315	Nucleus Area	69.65158	67.31833	24.45903
W4	PGE2	well_5	F003	2729	Nucleus Area	63.20778	61.87278	22.41681
W4	PGE2	well_5	F004	2705	Nucleus Area	65.38747	62.85861	22.45116
W4	PGE2	well_5	F005	2441	Nucleus Area	65.22137	64.26694	21.66321
W4	PGE2	well_5	F006	2162	Nucleus Area	68.74584	67.6	24.47874
W1	HPI4	well_1	F001	1779	Nucleus Height	4.422709	4.5	1.751606
W1	HPI4	well_1	F002	1534	Nucleus Height	4.419166	4.5	1.746859
W1	HPI4	well_1	F003	1957	Nucleus Height	4.754727	5	1.752557
W1	HPI4	well_1	F004	1826	Nucleus Height	4.66621	4.5	1.724252
W1	HPI4	well_1	F005	2136	Nucleus Height	4.787453	5	1.70397
W1	HPI4	well_1	F006	1670	Nucleus Height	4.573952	4.5	1.822668
W1	HPI4	well_2	F001	1519	Nucleus Height	4.282752	4.5	1.711545
W1	HPI4	well_2	F002	1767	Nucleus Height	4.398415	4.5	1.62964
W1	HPI4	well_2	F003	1910	Nucleus Height	4.351832	4.5	1.688492

W1	HPI4	well_2	F004	1930	Nucleus Height	4.710363	4.5	1.851973
W1	HPI4	well_2	F005	2173	Nucleus Height	4.795674	5	1.725763
W1	HPI4	well_2	F006	1819	Nucleus Height	4.96619	5	1.792705
W1	HPI4	well_3	F001	1380	Nucleus Height	4.155797	4	1.704727
W1	HPI4	well_3	F002	1707	Nucleus Height	4.520797	4.5	1.746041
W1	HPI4	well_3	F003	1664	Nucleus Height	4.541166	4.5	1.657892
W1	HPI4	well_3	F004	2029	Nucleus Height	4.699606	4.5	1.829136
W1	HPI4	well_3	F005	1922	Nucleus Height	4.686524	5	1.74476
W1	HPI4	well_3	F006	1966	Nucleus Height	4.805697	5	1.790529
W1	HPI4	well_4	F001	1621	Nucleus Height	4.545651	4.5	1.853945
W1	HPI4	well_4	F002	1883	Nucleus Height	4.67897	4.5	1.726703
W1	HPI4	well_4	F003	1990	Nucleus Height	4.559548	4.5	1.797251
W1	HPI4	well_4	F004	1800	Nucleus Height	4.315	4.5	1.602943
W1	HPI4	well_4	F005	1968	Nucleus Height	4.54878	4.5	1.745183
W1	HPI4	well_4	F006	1865	Nucleus Height	4.526273	4.5	1.635381
W1	HPI4	well_5	F001	1731	Nucleus Height	4.380416	4.5	1.85208
W1	HPI4	well_5	F002	1807	Nucleus Height	4.489762	4.5	1.694327
W1	HPI4	well_5	F003	2045	Nucleus Height	4.829584	5	1.865704
W1	HPI4	well_5	F004	1765	Nucleus Height	4.670255	5	1.85326
W1	HPI4	well_5	F005	1696	Nucleus Height	4.339917	4.5	1.641603
W1	HPI4	well_5	F006	1567	Nucleus Height	4.451181	4.5	1.701741
W1	PGE2	well_1	F001	2088	Nucleus Height	4.563937	4.5	1.597976
W1	PGE2	well_1	F002	1885	Nucleus Height	4.327321	4.5	1.681365
W1	PGE2	well_1	F003	2089	Nucleus Height	4.575634	4.5	1.794061
W1	PGE2	well_1	F004	2227	Nucleus Height	4.678491	4.5	1.759878
W1	PGE2	well_1	F005	2492	Nucleus Height	4.977929	5	1.833909
W1	PGE2	well_1	F006	2566	Nucleus Height	4.717654	5	1.730102
W1	PGE2	well_2	F001	1729	Nucleus Height	4.65587	4.5	1.614952
W1	PGE2	well_2	F002	1816	Nucleus Height	4.790198	5	1.796241
W1	PGE2	well_2	F003	1887	Nucleus Height	4.63858	4.5	1.726303
W1	PGE2	well_2	F004	2061	Nucleus Height	4.777293	5	1.758444
W1	PGE2	well_2	F005	2098	Nucleus Height	4.833889	5	1.777148
W1	PGE2	well_2	F006	1676	Nucleus Height	4.618437	4.5	1.767491
W1	PGE2	well_3	F001	1851	Nucleus Height	4.622636	4.5	1.63784
W1	PGE2	well_3	F002	1798	Nucleus Height	4.573137	4.5	1.70332
W1	PGE2	well_3	F003	1905	Nucleus Height	4.72336	5	1.675439
W1	PGE2	well_3	F004	1902	Nucleus Height	4.503417	4.5	1.69362
W1	PGE2	well_3	F005	2032	Nucleus Height	4.651083	5	1.859625
W1	PGE2	well_3	F006	1874	Nucleus Height	4.743863	5	1.845624
W1	PGE2	well_4	F001	1627	Nucleus Height	4.489551	4.5	1.764559
W1	PGE2	well_4	F002	1758	Nucleus Height	4.628555	4.5	1.642743
W1	PGE2	well_4	F003	1669	Nucleus Height	4.415219	4.5	1.782333
W1	PGE2	well_4	F004	2033	Nucleus Height	4.891294	5	1.829531
W1	PGE2	well_4	F005	1768	Nucleus Height	4.583428	4.5	1.795178
W1	PGE2	well_4	F006	1816	Nucleus Height	4.886289	5	1.825254
W1	PGE2	well_5	F001	1629	Nucleus Height	4.453039	4.5	1.703472
W1	PGE2	well_5	F002	1888	Nucleus Height	4.486758	4.5	1.7551

W1	PGE2	well_5	F003	1649	Nucleus Height	4.454518	4.5	1.737051
W1	PGE2	well_5	F004	1777	Nucleus Height	4.612831	4.5	1.776683
W1	PGE2	well_5	F005	1851	Nucleus Height	4.590492	4.5	1.709764
W1	PGE2	well_5	F006	1706	Nucleus Height	4.623388	4.5	1.781508
W2	HPI4	well_1	F001	1616	Nucleus Height	3.58354	3.5	1.374969
W2	HPI4	well_1	F002	1675	Nucleus Height	3.80597	4	1.427654
W2	HPI4	well_1	F003	1796	Nucleus Height	3.878619	4	1.613063
W2	HPI4	well_1	F004	1739	Nucleus Height	4.125072	4	1.680037
W2	HPI4	well_1	F005	1621	Nucleus Height	3.815854	4	1.455891
W2	HPI4	well_1	F006	1471	Nucleus Height	3.525153	3.5	1.294433
W2	HPI4	well_2	F001	1461	Nucleus Height	3.544148	3.5	1.325566
W2	HPI4	well_2	F002	1570	Nucleus Height	3.764013	3.5	1.341193
W2	HPI4	well_2	F003	1570	Nucleus Height	3.817516	4	1.467834
W2	HPI4	well_2	F004	1870	Nucleus Height	3.964171	4	1.472523
W2	HPI4	well_2	F005	1644	Nucleus Height	3.938564	4	1.441873
W2	HPI4	well_2	F006	1614	Nucleus Height	3.892503	4	1.474751
W2	HPI4	well_3	F001	1751	Nucleus Height	3.673615	3.5	1.412338
W2	HPI4	well_3	F002	1471	Nucleus Height	3.787899	4	1.413089
W2	HPI4	well_3	F003	1127	Nucleus Height	3.492014	3.5	1.376802
W2	HPI4	well_3	F004	1733	Nucleus Height	4.116272	4	1.609517
W2	HPI4	well_3	F005	1760	Nucleus Height	4.032386	4	1.673312
W2	HPI4	well_3	F006	1654	Nucleus Height	3.937424	4	1.620493
W2	HPI4	well_4	F001	1628	Nucleus Height	3.822789	4	1.441787
W2	HPI4	well_4	F002	1545	Nucleus Height	3.972492	4	1.575513
W2	HPI4	well_4	F003	1541	Nucleus Height	3.879299	4	1.435191
W2	HPI4	well_4	F004	1727	Nucleus Height	4.008107	4	1.498819
W2	HPI4	well_4	F005	1511	Nucleus Height	3.790205	4	1.475517
W2	HPI4	well_4	F006	1590	Nucleus Height	4.061006	4	1.602504
W2	HPI4	well_5	F001	1496	Nucleus Height	3.762701	4	1.445963
W2	HPI4	well_5	F002	1370	Nucleus Height	3.882482	4	1.443628
W2	HPI4	well_5	F003	1387	Nucleus Height	3.923937	4	1.492822
W2	HPI4	well_5	F004	1683	Nucleus Height	4.042187	4	1.53429
W2	HPI4	well_5	F005	1752	Nucleus Height	3.916096	4	1.56474
W2	HPI4	well_5	F006	1445	Nucleus Height	3.766436	4	1.45439
W2	PGE2	well_1	F001	2222	Nucleus Height	5.193969	5.5	2.092137
W2	PGE2	well_1	F002	1940	Nucleus Height	5.188402	5.5	2.059944
W2	PGE2	well_1	F003	2718	Nucleus Height	5.377483	5.5	1.999374
W2	PGE2	well_1	F004	2149	Nucleus Height	4.986273	5.5	1.757578
W2	PGE2	well_1	F005	2692	Nucleus Height	5.028975	5	1.847819
W2	PGE2	well_1	F006	2744	Nucleus Height	5.372085	5.5	2.037705
W2	PGE2	well_2	F001	2270	Nucleus Height	5.422907	6	1.892379
W2	PGE2	well_2	F002	2877	Nucleus Height	5.36114	5.5	2.024346
W2	PGE2	well_2	F003	3288	Nucleus Height	5.280262	5.5	1.855438
W2	PGE2	well_2	F004	2973	Nucleus Height	5.362597	5.5	1.926267
W2	PGE2	well_2	F005	2444	Nucleus Height	5.363953	6	1.923902
W2	PGE2	well_2	F006	2304	Nucleus Height	5.0625	5	1.860355
W2	PGE2	well_3	F001	2012	Nucleus Height	5.428429	6	2.124154

W2	PGE2	well_3	F002	3189	Nucleus Height	5.386485	5.5	1.98484
W2	PGE2	well_3	F003	2876	Nucleus Height	5.398818	5.5	1.877978
W2	PGE2	well_3	F004	2935	Nucleus Height	5.611755	6	1.928541
W2	PGE2	well_3	F005	2421	Nucleus Height	5.479554	6	1.94514
W2	PGE2	well_3	F006	2111	Nucleus Height	5.574135	6	1.983659
W2	PGE2	well_4	F001	2452	Nucleus Height	5.345024	5.5	1.89015
W2	PGE2	well_4	F002	3055	Nucleus Height	5.621768	6	1.893463
W2	PGE2	well_4	F003	2843	Nucleus Height	5.402744	6	1.915699
W2	PGE2	well_4	F004	3219	Nucleus Height	5.347468	5.5	1.859592
W2	PGE2	well_4	F005	2886	Nucleus Height	5.71535	6	2.02749
W2	PGE2	well_4	F006	2381	Nucleus Height	5.537799	6	2.042058
W2	PGE2	well_5	F001	2804	Nucleus Height	5.377675	5.5	1.97564
W2	PGE2	well_5	F002	3207	Nucleus Height	5.271437	5.5	1.88453
W2	PGE2	well_5	F003	2872	Nucleus Height	5.341574	5.5	1.838993
W2	PGE2	well_5	F004	2580	Nucleus Height	5.502519	6	1.769012
W2	PGE2	well_5	F005	2615	Nucleus Height	5.669407	6	1.787587
W2	PGE2	well_5	F006	2953	Nucleus Height	5.446664	6	1.883807
W3	HPI4	well_1	F001	798	Nucleus Height	3.33208	3	1.399722
W3	HPI4	well_1	F002	1010	Nucleus Height	3.203465	3	1.330044
W3	HPI4	well_1	F003	908	Nucleus Height	2.860683	2.5	1.254156
W3	HPI4	well_1	F004	1248	Nucleus Height	3.21875	3	1.313303
W3	HPI4	well_1	F005	1235	Nucleus Height	3.289069	3	1.338043
W3	HPI4	well_1	F006	914	Nucleus Height	2.960613	3	1.302993
W3	HPI4	well_2	F001	797	Nucleus Height	3.018193	3	1.276225
W3	HPI4	well_2	F002	971	Nucleus Height	3.053553	3	1.264999
W3	HPI4	well_2	F003	933	Nucleus Height	3.266881	3	1.304312
W3	HPI4	well_2	F004	1129	Nucleus Height	3.41674	3.5	1.347994
W3	HPI4	well_2	F005	1253	Nucleus Height	3.234238	3	1.360382
W3	HPI4	well_2	F006	1058	Nucleus Height	3.286389	3	1.409263
W3	HPI4	well_3	F001	795	Nucleus Height	3.243396	3	1.384355
W3	HPI4	well_3	F002	885	Nucleus Height	3.261582	3.5	1.335299
W3	HPI4	well_3	F003	621	Nucleus Height	3.21256	3	1.16774
W3	HPI4	well_3	F004	986	Nucleus Height	3.032454	3	1.224314
W3	HPI4	well_3	F005	1121	Nucleus Height	3.431311	3.5	1.405096
W3	HPI4	well_3	F006	1047	Nucleus Height	3.418816	3.5	1.483368
W3	HPI4	well_4	F001	722	Nucleus Height	3.022161	3	1.121066
W3	HPI4	well_4	F002	918	Nucleus Height	3.057734	3	1.14201
W3	HPI4	well_4	F003	708	Nucleus Height	2.991525	3	1.159762
W3	HPI4	well_4	F004	785	Nucleus Height	3.154777	3	1.156418
W3	HPI4	well_4	F005	1210	Nucleus Height	3.559091	3.5	1.329821
W3	HPI4	well_4	F006	1129	Nucleus Height	3.625332	3.5	1.409824
W3	HPI4	well_5	F001	712	Nucleus Height	3.087079	3	1.19986
W3	HPI4	well_5	F002	558	Nucleus Height	3.091398	3	1.234483
W3	HPI4	well_5	F003	812	Nucleus Height	3.371921	3.5	1.223828
W3	HPI4	well_5	F004	517	Nucleus Height	3.017408	3	1.169992
W3	HPI4	well_5	F005	976	Nucleus Height	3.402664	3.5	1.300396
W3	HPI4	well_5	F006	931	Nucleus Height	3.380773	3.5	1.364593

W3	PGE2	well_1	F001	2659	Nucleus Height	4.798232	5	1.857408
W3	PGE2	well_1	F002	3405	Nucleus Height	4.781204	5	1.929234
W3	PGE2	well_1	F003	3522	Nucleus Height	4.7818	5	1.789121
W3	PGE2	well_1	F004	3028	Nucleus Height	5.189234	5.5	1.914256
W3	PGE2	well_1	F005	3384	Nucleus Height	4.936466	5.5	1.875117
W3	PGE2	well_1	F006	2846	Nucleus Height	4.71785	5	1.769785
W3	PGE2	well_2	F001	2912	Nucleus Height	4.740041	5	1.778359
W3	PGE2	well_2	F002	2973	Nucleus Height	4.835856	5	1.80589
W3	PGE2	well_2	F003	3367	Nucleus Height	4.834422	5	1.783177
W3	PGE2	well_2	F004	3533	Nucleus Height	4.811492	5	1.794626
W3	PGE2	well_2	F005	3705	Nucleus Height	4.817274	5	1.742176
W3	PGE2	well_2	F006	3844	Nucleus Height	4.937175	5	1.66679
W3	PGE2	well_3	F001	3127	Nucleus Height	4.735529	5	1.912663
W3	PGE2	well_3	F002	2777	Nucleus Height	4.380086	4.5	1.879576
W3	PGE2	well_3	F003	3810	Nucleus Height	4.941339	5	1.843876
W3	PGE2	well_3	F004	3332	Nucleus Height	4.972389	5.5	1.880589
W3	PGE2	well_3	F005	3851	Nucleus Height	4.926383	5	1.872798
W3	PGE2	well_3	F006	3599	Nucleus Height	4.841623	5	1.760794
W3	PGE2	well_4	F001	2774	Nucleus Height	4.75739	5	2.035851
W3	PGE2	well_4	F002	3347	Nucleus Height	5.024798	5.5	2.049517
W3	PGE2	well_4	F003	2729	Nucleus Height	4.778307	5	1.842882
W3	PGE2	well_4	F004	3120	Nucleus Height	4.712179	5	1.961479
W3	PGE2	well_4	F005	3555	Nucleus Height	4.65865	4.5	1.885064
W3	PGE2	well_4	F006	3216	Nucleus Height	4.75342	5	1.7723
W3	PGE2	well_5	F001	2321	Nucleus Height	4.508402	4.5	1.964127
W3	PGE2	well_5	F002	2453	Nucleus Height	4.463718	4.5	1.896514
W3	PGE2	well_5	F003	3024	Nucleus Height	4.794147	5	1.900094
W3	PGE2	well_5	F004	2780	Nucleus Height	4.782914	5	1.873494
W3	PGE2	well_5	F005	3314	Nucleus Height	4.817894	5	1.917507
W3	PGE2	well_5	F006	2620	Nucleus Height	4.746565	5	1.968511
W4	HPI4	well_1	F001	540	Nucleus Height	2.821296	2.5	1.073811
W4	HPI4	well_1	F002	888	Nucleus Height	3.460023	3.5	1.272079
W4	HPI4	well_1	F003	835	Nucleus Height	3.166467	3	1.115006
W4	HPI4	well_1	F004	991	Nucleus Height	3.326942	3.5	1.233198
W4	HPI4	well_1	F005	1090	Nucleus Height	2.969725	3	1.209467
W4	HPI4	well_1	F006	991	Nucleus Height	3.105449	3	1.241224
W4	HPI4	well_2	F001	653	Nucleus Height	3.232772	3.5	1.198234
W4	HPI4	well_2	F002	918	Nucleus Height	3.440087	3.5	1.21814
W4	HPI4	well_2	F003	956	Nucleus Height	3.135983	3	1.155602
W4	HPI4	well_2	F004	1031	Nucleus Height	3.156159	3	1.126192
W4	HPI4	well_2	F005	1024	Nucleus Height	3.085938	3	1.154039
W4	HPI4	well_2	F006	933	Nucleus Height	3.124866	3	1.190855
W4	HPI4	well_3	F001	759	Nucleus Height	3.317523	3.5	1.186432
W4	HPI4	well_3	F002	840	Nucleus Height	3.371429	3.5	1.170564
W4	HPI4	well_3	F003	927	Nucleus Height	3.167206	3	1.156297
W4	HPI4	well_3	F004	1041	Nucleus Height	3.470221	3.5	1.215911
W4	HPI4	well_3	F005	1065	Nucleus Height	3.410798	3.5	1.190906

W4	HPI4	well_3	F006	813	Nucleus Height	3.292743	3.5	1.190235
W4	HPI4	well_4	F001	772	Nucleus Height	3.146373	3	1.158043
W4	HPI4	well_4	F002	962	Nucleus Height	3.197505	3.5	1.142762
W4	HPI4	well_4	F003	701	Nucleus Height	3.256776	3	1.173079
W4	HPI4	well_4	F004	1062	Nucleus Height	3.282486	3.5	1.22311
W4	HPI4	well_4	F005	1118	Nucleus Height	3.075581	3	1.214786
W4	HPI4	well_4	F006	845	Nucleus Height	3.223077	3.5	1.256594
W4	HPI4	well_5	F001	791	Nucleus Height	3.30531	3.5	1.194406
W4	HPI4	well_5	F002	598	Nucleus Height	3.189799	3.5	1.193722
W4	HPI4	well_5	F003	752	Nucleus Height	3.310505	3.5	1.224064
W4	HPI4	well_5	F004	949	Nucleus Height	3.258693	3.5	1.300049
W4	HPI4	well_5	F005	1195	Nucleus Height	3.109623	3	1.257686
W4	HPI4	well_5	F006	1013	Nucleus Height	3.420039	3.5	1.247932
W4	PGE2	well_1	F001	2025	Nucleus Height	4.814568	5	1.530022
W4	PGE2	well_1	F002	1830	Nucleus Height	4.605464	4.5	1.65549
W4	PGE2	well_1	F003	2816	Nucleus Height	4.733665	5	1.584477
W4	PGE2	well_1	F004	2679	Nucleus Height	4.821202	5	1.717204
W4	PGE2	well_1	F005	2927	Nucleus Height	4.841134	5	1.597763
W4	PGE2	well_1	F006	2858	Nucleus Height	4.741777	5	1.557377
W4	PGE2	well_2	F001	2324	Nucleus Height	4.394363	4.5	1.621903
W4	PGE2	well_2	F002	2806	Nucleus Height	4.788133	5	1.622073
W4	PGE2	well_2	F003	2502	Nucleus Height	4.639488	5	1.667937
W4	PGE2	well_2	F004	2867	Nucleus Height	4.657133	5	1.691896
W4	PGE2	well_2	F005	3262	Nucleus Height	4.687462	5	1.657514
W4	PGE2	well_2	F006	2745	Nucleus Height	4.754645	5	1.623724
W4	PGE2	well_3	F001	2165	Nucleus Height	4.418245	4.5	1.659779
W4	PGE2	well_3	F002	3094	Nucleus Height	4.781997	5	1.58305
W4	PGE2	well_3	F003	3177	Nucleus Height	4.561221	4.5	1.593669
W4	PGE2	well_3	F004	3019	Nucleus Height	4.681517	5	1.753348
W4	PGE2	well_3	F005	2629	Nucleus Height	4.619247	5	1.703851
W4	PGE2	well_3	F006	2911	Nucleus Height	4.890587	5	1.684261
W4	PGE2	well_4	F001	2186	Nucleus Height	4.613907	5	1.818559
W4	PGE2	well_4	F002	2767	Nucleus Height	4.713589	5	1.664
W4	PGE2	well_4	F003	2924	Nucleus Height	4.652018	5	1.671814
W4	PGE2	well_4	F004	2783	Nucleus Height	4.717571	5	1.694444
W4	PGE2	well_4	F005	3493	Nucleus Height	4.748354	5	1.610195
W4	PGE2	well_4	F006	2292	Nucleus Height	4.639398	5	1.703491
W4	PGE2	well_5	F001	1871	Nucleus Height	4.343399	4.5	1.852173
W4	PGE2	well_5	F002	2315	Nucleus Height	4.615551	5	1.756005
W4	PGE2	well_5	F003	2729	Nucleus Height	4.864969	5.5	1.707431
W4	PGE2	well_5	F004	2705	Nucleus Height	4.787616	5	1.844023
W4	PGE2	well_5	F005	2441	Nucleus Height	4.69746	5	1.776881
W4	PGE2	well_5	F006	2162	Nucleus Height	4.57123	5	1.861037
W1	HPI4	well_1	F001	1779	Nucleus Volume	390.8401	397.0092	173.9885
W1	HPI4	well_1	F002	1534	Nucleus Volume	378.064	385.7777	184.91
W1	HPI4	well_1	F003	1957	Nucleus Volume	373.7757	375.0392	158.7353
W1	HPI4	well_1	F004	1826	Nucleus Volume	401.8916	402.7716	174.0447

W1	HPI4	well_1	F005	2136	Nucleus Volume	386.5565	391.4345	160.8531
W1	HPI4	well_1	F006	1670	Nucleus Volume	385.9238	397.8542	168.0215
W1	HPI4	well_2	F001	1519	Nucleus Volume	378.4406	379.7806	177.8523
W1	HPI4	well_2	F002	1767	Nucleus Volume	387.3703	393.9578	163.8678
W1	HPI4	well_2	F003	1910	Nucleus Volume	366.2291	370.3565	161.4399
W1	HPI4	well_2	F004	1930	Nucleus Volume	396.8968	405.8699	163.2993
W1	HPI4	well_2	F005	2173	Nucleus Volume	378.6737	379.5224	154.1267
W1	HPI4	well_2	F006	1819	Nucleus Volume	402.875	392.08	172.148
W1	HPI4	well_3	F001	1380	Nucleus Volume	371.4702	378.8299	169.4421
W1	HPI4	well_3	F002	1707	Nucleus Volume	384.5675	391.5636	166.8974
W1	HPI4	well_3	F003	1664	Nucleus Volume	381.4283	379.7806	164.3987
W1	HPI4	well_3	F004	2029	Nucleus Volume	380.0393	383.63	160.34
W1	HPI4	well_3	F005	1922	Nucleus Volume	384.9747	388.3362	160.6189
W1	HPI4	well_3	F006	1966	Nucleus Volume	389.523	387.5968	178.7256
W1	HPI4	well_4	F001	1621	Nucleus Volume	374.3261	385.3669	161.2119
W1	HPI4	well_4	F002	1883	Nucleus Volume	370.7744	377.3394	152.2444
W1	HPI4	well_4	F003	1990	Nucleus Volume	374.0957	374.6284	171.606
W1	HPI4	well_4	F004	1800	Nucleus Volume	367.6025	373.9712	156.4063
W1	HPI4	well_4	F005	1968	Nucleus Volume	373.0652	379.7101	163.8058
W1	HPI4	well_4	F006	1865	Nucleus Volume	374.2316	382.2217	164.5299
W1	HPI4	well_5	F001	1731	Nucleus Volume	354.9235	370.6968	163.9366
W1	HPI4	well_5	F002	1807	Nucleus Volume	365.6512	378.6539	156.1348
W1	HPI4	well_5	F003	2045	Nucleus Volume	371.348	373.7717	160.0163
W1	HPI4	well_5	F004	1765	Nucleus Volume	365.0343	370.0631	161.3879
W1	HPI4	well_5	F005	1696	Nucleus Volume	384.0759	382.9728	170.6309
W1	HPI4	well_5	F006	1567	Nucleus Volume	375.2299	376.9874	160.0996
W1	PGE2	well_1	F001	2088	Nucleus Volume	373.5284	373.5956	154.0575
W1	PGE2	well_1	F002	1885	Nucleus Volume	381.1753	386.8457	171.8428
W1	PGE2	well_1	F003	2089	Nucleus Volume	386.5218	392.7138	168.0169
W1	PGE2	well_1	F004	2227	Nucleus Volume	383.4217	386.6344	176.9252
W1	PGE2	well_1	F005	2492	Nucleus Volume	370.4077	369.7814	157.0597
W1	PGE2	well_1	F006	2566	Nucleus Volume	364.292	368.9129	153.2758
W1	PGE2	well_2	F001	1729	Nucleus Volume	390.9514	393.3475	168.0751
W1	PGE2	well_2	F002	1816	Nucleus Volume	395.99	394.9319	176.8394
W1	PGE2	well_2	F003	1887	Nucleus Volume	375.7777	374.1942	167.2541
W1	PGE2	well_2	F004	2061	Nucleus Volume	386.834	387.5499	172.5511
W1	PGE2	well_2	F005	2098	Nucleus Volume	398.701	402.3843	163.1993
W1	PGE2	well_2	F006	1676	Nucleus Volume	393.1306	401.3163	170.9787
W1	PGE2	well_3	F001	1851	Nucleus Volume	404.1971	409.7546	165.9896
W1	PGE2	well_3	F002	1798	Nucleus Volume	397.1827	395.8825	180.7095
W1	PGE2	well_3	F003	1905	Nucleus Volume	397.1823	394.3803	163.7703
W1	PGE2	well_3	F004	1902	Nucleus Volume	398.5737	406.8206	178.2558
W1	PGE2	well_3	F005	2032	Nucleus Volume	377.442	380.5434	173.1361
W1	PGE2	well_3	F006	1874	Nucleus Volume	397.3467	402.9124	170.0423
W1	PGE2	well_4	F001	1627	Nucleus Volume	403.6799	415.2471	178.5795
W1	PGE2	well_4	F002	1758	Nucleus Volume	393.0334	395.3778	160.9295
W1	PGE2	well_4	F003	1669	Nucleus Volume	380.5133	389.6154	173.7242

W1	PGE2	well_4	F004	2033	Nucleus Volume	384.3165	387.0804	164.6746
W1	PGE2	well_4	F005	1768	Nucleus Volume	393.1539	402.8185	165.4001
W1	PGE2	well_4	F006	1816	Nucleus Volume	398.8172	396.9974	169.7373
W1	PGE2	well_5	F001	1629	Nucleus Volume	388.387	395.8356	171.0756
W1	PGE2	well_5	F002	1888	Nucleus Volume	385.8852	386.5288	169.4641
W1	PGE2	well_5	F003	1649	Nucleus Volume	390.6482	396.2581	167.0229
W1	PGE2	well_5	F004	1777	Nucleus Volume	402.0108	404.755	170.186
W1	PGE2	well_5	F005	1851	Nucleus Volume	391.9183	397.2674	173.2943
W1	PGE2	well_5	F006	1706	Nucleus Volume	384.3828	391.2819	165.2137
W2	HPI4	well_1	F001	1616	Nucleus Volume	275.4556	290.1284	142.5005
W2	HPI4	well_1	F002	1675	Nucleus Volume	322.4127	346.4969	147.7765
W2	HPI4	well_1	F003	1796	Nucleus Volume	292.7961	309.0353	140.3852
W2	HPI4	well_1	F004	1739	Nucleus Volume	321.45	338.7981	153.8271
W2	HPI4	well_1	F005	1621	Nucleus Volume	314.1617	339.0328	149.4826
W2	HPI4	well_1	F006	1471	Nucleus Volume	302.8078	322.3675	148.7993
W2	HPI4	well_2	F001	1461	Nucleus Volume	283.2928	290.3044	166.0584
W2	HPI4	well_2	F002	1570	Nucleus Volume	309.0403	328.6111	139.7498
W2	HPI4	well_2	F003	1570	Nucleus Volume	311.1828	332.4253	146.3796
W2	HPI4	well_2	F004	1870	Nucleus Volume	303.713	321.5342	143.6609
W2	HPI4	well_2	F005	1644	Nucleus Volume	322.1412	341.9903	153.0633
W2	HPI4	well_2	F006	1614	Nucleus Volume	309.4822	337.9765	144.7189
W2	HPI4	well_3	F001	1751	Nucleus Volume	274.0593	293.8018	150.8529
W2	HPI4	well_3	F002	1471	Nucleus Volume	305.716	330.7471	150.2022
W2	HPI4	well_3	F003	1127	Nucleus Volume	302.7433	303.3785	164.2476
W2	HPI4	well_3	F004	1733	Nucleus Volume	323.7955	349.2667	149.9532
W2	HPI4	well_3	F005	1760	Nucleus Volume	302.3325	324.9729	150.3637
W2	HPI4	well_3	F006	1654	Nucleus Volume	312.2251	333.3056	156.3891
W2	HPI4	well_4	F001	1628	Nucleus Volume	269.4623	283.3449	137.7208
W2	HPI4	well_4	F002	1545	Nucleus Volume	285.673	295.1632	135.4
W2	HPI4	well_4	F003	1541	Nucleus Volume	304.1821	324.5974	135.6855
W2	HPI4	well_4	F004	1727	Nucleus Volume	301.974	323.7054	142.1332
W2	HPI4	well_4	F005	1511	Nucleus Volume	303.6695	323.3533	146.0162
W2	HPI4	well_4	F006	1590	Nucleus Volume	326.6923	331.4395	152.9576
W2	HPI4	well_5	F001	1496	Nucleus Volume	294.1734	310.5844	151.4917
W2	HPI4	well_5	F002	1370	Nucleus Volume	325.9377	341.1453	159.0016
W2	HPI4	well_5	F003	1387	Nucleus Volume	323.3789	341.826	140.5349
W2	HPI4	well_5	F004	1683	Nucleus Volume	321.7876	333.4933	152.4616
W2	HPI4	well_5	F005	1752	Nucleus Volume	299.0492	316.7811	147.5076
W2	HPI4	well_5	F006	1445	Nucleus Volume	307.3344	332.3197	157.7782
W2	PGE2	well_1	F001	2222	Nucleus Volume	306.2689	331.24	125.8139
W2	PGE2	well_1	F002	1940	Nucleus Volume	291.3328	314.7508	119.7143
W2	PGE2	well_1	F003	2718	Nucleus Volume	313.9985	335.066	115.0635
W2	PGE2	well_1	F004	2149	Nucleus Volume	300.5146	323.8697	111.7332
W2	PGE2	well_1	F005	2692	Nucleus Volume	288.7097	311.1008	108.0492
W2	PGE2	well_1	F006	2744	Nucleus Volume	288.4541	317.1097	115.5566
W2	PGE2	well_2	F001	2270	Nucleus Volume	304.5324	328.5524	115.4029
W2	PGE2	well_2	F002	2877	Nucleus Volume	292.4365	315.4432	118.3632

W2	PGE2	well_2	F003	3288	Nucleus Volume	296.0955	316.4173	113.5658
W2	PGE2	well_2	F004	2973	Nucleus Volume	300.9336	314.9503	118.4427
W2	PGE2	well_2	F005	2444	Nucleus Volume	297.6538	323.5059	109.8578
W2	PGE2	well_2	F006	2304	Nucleus Volume	292.3878	315.4784	116.8833
W2	PGE2	well_3	F001	2012	Nucleus Volume	305.039	333.7281	126.7956
W2	PGE2	well_3	F002	3189	Nucleus Volume	307.6295	329.9725	118.6374
W2	PGE2	well_3	F003	2876	Nucleus Volume	308.4793	328.0713	110.0083
W2	PGE2	well_3	F004	2935	Nucleus Volume	294.1518	317.4853	106.4613
W2	PGE2	well_3	F005	2421	Nucleus Volume	300.5866	324.48	110.7402
W2	PGE2	well_3	F006	2111	Nucleus Volume	313.967	337.1081	113.6662
W2	PGE2	well_4	F001	2452	Nucleus Volume	314.323	336.0283	127.4642
W2	PGE2	well_4	F002	3055	Nucleus Volume	313.5203	332.2493	120.4882
W2	PGE2	well_4	F003	2843	Nucleus Volume	300.9726	327.1793	111.0392
W2	PGE2	well_4	F004	3219	Nucleus Volume	310.3502	332.7188	110.2386
W2	PGE2	well_4	F005	2886	Nucleus Volume	314.6338	337.0494	113.7711
W2	PGE2	well_4	F006	2381	Nucleus Volume	317.4233	342.9057	118.9787
W2	PGE2	well_5	F001	2804	Nucleus Volume	305.9671	322.3558	121.3642
W2	PGE2	well_5	F002	3207	Nucleus Volume	303.4862	331.8503	113.1015
W2	PGE2	well_5	F003	2872	Nucleus Volume	307.8131	326.7333	112.5819
W2	PGE2	well_5	F004	2580	Nucleus Volume	301.7439	319.0814	117.0014
W2	PGE2	well_5	F005	2615	Nucleus Volume	313.8062	337.0846	109.4805
W2	PGE2	well_5	F006	2953	Nucleus Volume	304.3506	326.5925	118.5365
W3	HPI4	well_1	F001	798	Nucleus Volume	289.9939	303.7775	159.5944
W3	HPI4	well_1	F002	1010	Nucleus Volume	277.3946	287.4995	150.4291
W3	HPI4	well_1	F003	908	Nucleus Volume	253.1386	254.0281	155.6845
W3	HPI4	well_1	F004	1248	Nucleus Volume	254.2621	274.3551	129.0637
W3	HPI4	well_1	F005	1235	Nucleus Volume	276.0665	292.0414	138.2758
W3	HPI4	well_1	F006	914	Nucleus Volume	257.6636	271.3858	148.0236
W3	HPI4	well_2	F001	797	Nucleus Volume	256.056	266.9496	150.4059
W3	HPI4	well_2	F002	971	Nucleus Volume	271.2565	280.2114	151.1515
W3	HPI4	well_2	F003	933	Nucleus Volume	288.5248	299.4117	150.6259
W3	HPI4	well_2	F004	1129	Nucleus Volume	290.6977	300.6792	151.2496
W3	HPI4	well_2	F005	1253	Nucleus Volume	267.8067	278.5214	147.2673
W3	HPI4	well_2	F006	1058	Nucleus Volume	280.8682	296.196	143.8275
W3	HPI4	well_3	F001	795	Nucleus Volume	279.7415	301.8528	140.4043
W3	HPI4	well_3	F002	885	Nucleus Volume	292.4328	313.2368	153.3739
W3	HPI4	well_3	F003	621	Nucleus Volume	291.7933	318.7997	142.4334
W3	HPI4	well_3	F004	986	Nucleus Volume	247.7237	261.5862	143.4154
W3	HPI4	well_3	F005	1121	Nucleus Volume	271.7416	287.6286	148.1596
W3	HPI4	well_3	F006	1047	Nucleus Volume	270.0345	287.746	146.6941
W3	HPI4	well_4	F001	722	Nucleus Volume	270.5766	271.9022	156.0791
W3	HPI4	well_4	F002	918	Nucleus Volume	287.1357	300.5149	153.188
W3	HPI4	well_4	F003	708	Nucleus Volume	282.4768	283.4858	150.6218
W3	HPI4	well_4	F004	785	Nucleus Volume	270.5363	275.2588	160.185
W3	HPI4	well_4	F005	1210	Nucleus Volume	293.6399	305.9722	142.6807
W3	HPI4	well_4	F006	1129	Nucleus Volume	294.5488	307.9086	153.0868
W3	HPI4	well_5	F001	712	Nucleus Volume	291.153	293.4497	187.0396

W3	HPI4	well_5	F002	558	Nucleus Volume	279.2318	292.5813	147.3598
W3	HPI4	well_5	F003	812	Nucleus Volume	286.5874	298.3085	150.5302
W3	HPI4	well_5	F004	517	Nucleus Volume	275.697	281.2676	161.1844
W3	HPI4	well_5	F005	976	Nucleus Volume	295.0605	305.6435	151.3657
W3	HPI4	well_5	F006	931	Nucleus Volume	300.7455	309.0118	156.3436
W3	PGE2	well_1	F001	2659	Nucleus Volume	270.4778	294.5764	102.4704
W3	PGE2	well_1	F002	3405	Nucleus Volume	268.8914	294.5529	107.3768
W3	PGE2	well_1	F003	3522	Nucleus Volume	269.0001	288.8022	105.3166
W3	PGE2	well_1	F004	3028	Nucleus Volume	267.7812	291.0908	98.08865
W3	PGE2	well_1	F005	3384	Nucleus Volume	268.4412	291.9827	100.5408
W3	PGE2	well_1	F006	2846	Nucleus Volume	275.6661	296.4776	97.37227
W3	PGE2	well_2	F001	2912	Nucleus Volume	272.7728	297.6513	96.4163
W3	PGE2	well_2	F002	2973	Nucleus Volume	274.9042	298.7544	100.3372
W3	PGE2	well_2	F003	3367	Nucleus Volume	266.9403	288.6614	99.17419
W3	PGE2	well_2	F004	3533	Nucleus Volume	270.6328	288.1919	99.66736
W3	PGE2	well_2	F005	3705	Nucleus Volume	271.137	286.1029	98.54656
W3	PGE2	well_2	F006	3844	Nucleus Volume	275.9057	298.907	90.12018
W3	PGE2	well_3	F001	3127	Nucleus Volume	266.2394	287.9572	109.0839
W3	PGE2	well_3	F002	2777	Nucleus Volume	263.1672	289.4829	115.1351
W3	PGE2	well_3	F003	3810	Nucleus Volume	267.2349	285.6804	95.79164
W3	PGE2	well_3	F004	3332	Nucleus Volume	274.6705	292.9685	97.93952
W3	PGE2	well_3	F005	3851	Nucleus Volume	268.7321	289.8819	100.3282
W3	PGE2	well_3	F006	3599	Nucleus Volume	286.7662	309.7864	98.73186
W3	PGE2	well_4	F001	2774	Nucleus Volume	264.579	298.2498	109.8459
W3	PGE2	well_4	F002	3347	Nucleus Volume	279.2495	307.1106	113.1754
W3	PGE2	well_4	F003	2729	Nucleus Volume	274.6219	298.0738	108.7773
W3	PGE2	well_4	F004	3120	Nucleus Volume	266.8712	288.9078	103.7864
W3	PGE2	well_4	F005	3555	Nucleus Volume	267.123	293.6844	104.5067
W3	PGE2	well_4	F006	3216	Nucleus Volume	276.1161	298.8718	101.8067
W3	PGE2	well_5	F001	2321	Nucleus Volume	255.1012	279.9767	113.0541
W3	PGE2	well_5	F002	2453	Nucleus Volume	263.3332	289.3656	114.5187
W3	PGE2	well_5	F003	3024	Nucleus Volume	266.0035	293.7079	113.8624
W3	PGE2	well_5	F004	2780	Nucleus Volume	258.9281	284.7063	100.6128
W3	PGE2	well_5	F005	3314	Nucleus Volume	259.146	282.3356	96.35953
W3	PGE2	well_5	F006	2620	Nucleus Volume	269.4376	294.3182	102.6455
W4	HPI4	well_1	F001	540	Nucleus Volume	263.2661	269.4376	161.2308
W4	HPI4	well_1	F002	888	Nucleus Volume	282.5784	290.2575	154.7585
W4	HPI4	well_1	F003	835	Nucleus Volume	265.1642	278.5683	130.125
W4	HPI4	well_1	F004	991	Nucleus Volume	283.7318	303.6836	142.5806
W4	HPI4	well_1	F005	1090	Nucleus Volume	246.1248	240.9776	150.7438
W4	HPI4	well_1	F006	991	Nucleus Volume	265.4154	274.7893	146.5211
W4	HPI4	well_2	F001	653	Nucleus Volume	296.1628	313.9175	160.3565
W4	HPI4	well_2	F002	918	Nucleus Volume	279.3164	288.3563	143.1027
W4	HPI4	well_2	F003	956	Nucleus Volume	273.0896	282.4647	142.4887
W4	HPI4	well_2	F004	1031	Nucleus Volume	272.6396	282.5821	133.899
W4	HPI4	well_2	F005	1024	Nucleus Volume	261.9773	278.4979	142.0964
W4	HPI4	well_2	F006	933	Nucleus Volume	279.8655	289.835	140.0794

W4	HPI4	well_3	F001	759	Nucleus Volume	293.9334	309.9272	147.2225
W4	HPI4	well_3	F002	840	Nucleus Volume	304.0204	327.3084	142.9439
W4	HPI4	well_3	F003	927	Nucleus Volume	290.5974	308.2607	161.148
W4	HPI4	well_3	F004	1041	Nucleus Volume	284.9131	307.2749	140.1681
W4	HPI4	well_3	F005	1065	Nucleus Volume	280.7641	300.5149	133.1107
W4	HPI4	well_3	F006	813	Nucleus Volume	275.0731	296.2899	137.8122
W4	HPI4	well_4	F001	772	Nucleus Volume	302.4035	319.2809	160.8787
W4	HPI4	well_4	F002	962	Nucleus Volume	296.3958	310.1619	154.1113
W4	HPI4	well_4	F003	701	Nucleus Volume	316.841	332.5779	160.5732
W4	HPI4	well_4	F004	1062	Nucleus Volume	280.4054	285.7508	154.3944
W4	HPI4	well_4	F005	1118	Nucleus Volume	251.9276	258.6639	147.311
W4	HPI4	well_4	F006	845	Nucleus Volume	281.0185	309.3169	152.2746
W4	HPI4	well_5	F001	791	Nucleus Volume	314.9977	338.9858	160.5362
W4	HPI4	well_5	F002	598	Nucleus Volume	324.3141	339.8778	174.4995
W4	HPI4	well_5	F003	752	Nucleus Volume	323.9392	332.5427	167.7468
W4	HPI4	well_5	F004	949	Nucleus Volume	308.2345	319.1518	167.8825
W4	HPI4	well_5	F005	1195	Nucleus Volume	260.4582	270.6113	140.1502
W4	HPI4	well_5	F006	1013	Nucleus Volume	284.796	313.894	141.3324
W4	PGE2	well_1	F001	2025	Nucleus Volume	279.0006	298.4258	105.1509
W4	PGE2	well_1	F002	1830	Nucleus Volume	269.0588	291.9592	107.8297
W4	PGE2	well_1	F003	2816	Nucleus Volume	277.4512	292.0531	109.3384
W4	PGE2	well_1	F004	2679	Nucleus Volume	263.9219	287.746	107.7545
W4	PGE2	well_1	F005	2927	Nucleus Volume	270.6745	286.0325	110.4535
W4	PGE2	well_1	F006	2858	Nucleus Volume	278.7768	294.8933	107.0532
W4	PGE2	well_2	F001	2324	Nucleus Volume	263.4141	283.3097	112.4131
W4	PGE2	well_2	F002	2806	Nucleus Volume	269.4926	284.2369	105.1057
W4	PGE2	well_2	F003	2502	Nucleus Volume	260.7057	280.9742	106.1649
W4	PGE2	well_2	F004	2867	Nucleus Volume	254.9936	274.1086	103.2323
W4	PGE2	well_2	F005	3262	Nucleus Volume	255.8498	275.1414	98.89104
W4	PGE2	well_2	F006	2745	Nucleus Volume	270.713	289.5064	99.74988
W4	PGE2	well_3	F001	2165	Nucleus Volume	256.3642	273.7331	110.4202
W4	PGE2	well_3	F002	3094	Nucleus Volume	269.6471	283.2041	95.34927
W4	PGE2	well_3	F003	3177	Nucleus Volume	252.2139	266.2924	100.0034
W4	PGE2	well_3	F004	3019	Nucleus Volume	254.8141	274.7189	101.1306
W4	PGE2	well_3	F005	2629	Nucleus Volume	261.6996	278.1693	102.3169
W4	PGE2	well_3	F006	2911	Nucleus Volume	280.9205	301.0782	99.03881
W4	PGE2	well_4	F001	2186	Nucleus Volume	247.9992	265.8933	102.4001
W4	PGE2	well_4	F002	2767	Nucleus Volume	255.547	272.7942	102.1094
W4	PGE2	well_4	F003	2924	Nucleus Volume	254.7923	274.3081	92.99509
W4	PGE2	well_4	F004	2783	Nucleus Volume	255.7992	273.3575	96.94261
W4	PGE2	well_4	F005	3493	Nucleus Volume	259.4171	275.47	93.05758
W4	PGE2	well_4	F006	2292	Nucleus Volume	261.4484	282.7581	106.6045
W4	PGE2	well_5	F001	1871	Nucleus Volume	249.826	277.606	109.2209
W4	PGE2	well_5	F002	2315	Nucleus Volume	258.8289	285.7508	100.8034
W4	PGE2	well_5	F003	2729	Nucleus Volume	248.0965	266.7618	98.83837
W4	PGE2	well_5	F004	2705	Nucleus Volume	247.9052	271.4093	98.55835
W4	PGE2	well_5	F005	2441	Nucleus Volume	247.5025	274.8363	99.58893

W4	PGE2	well_5	F006	2162	Nucleus Volume	255.5016	285.1875	112.9412
W1	HPI4	well_1	F001	1779	Nucleus Width	11.94121	11.91667	2.532064
W1	HPI4	well_1	F002	1534	Nucleus Width	11.69816	11.59167	2.689471
W1	HPI4	well_1	F003	1957	Nucleus Width	11.35441	11.26667	2.352325
W1	HPI4	well_1	F004	1826	Nucleus Width	11.84019	11.80833	2.461564
W1	HPI4	well_1	F005	2136	Nucleus Width	11.48927	11.48333	2.253212
W1	HPI4	well_1	F006	1670	Nucleus Width	11.7059	11.80833	2.511209
W1	HPI4	well_2	F001	1519	Nucleus Width	11.90768	11.80833	2.635542
W1	HPI4	well_2	F002	1767	Nucleus Width	11.88644	11.91667	2.503801
W1	HPI4	well_2	F003	1910	Nucleus Width	11.66035	11.59167	2.526746
W1	HPI4	well_2	F004	1930	Nucleus Width	11.78611	11.80833	2.321507
W1	HPI4	well_2	F005	2173	Nucleus Width	11.38193	11.26667	2.336954
W1	HPI4	well_2	F006	1819	Nucleus Width	11.55593	11.48333	2.463979
W1	HPI4	well_3	F001	1380	Nucleus Width	12.05091	12.025	2.869785
W1	HPI4	well_3	F002	1707	Nucleus Width	11.72221	11.91667	2.361616
W1	HPI4	well_3	F003	1664	Nucleus Width	11.67155	11.59167	2.493381
W1	HPI4	well_3	F004	2029	Nucleus Width	11.61687	11.7	2.45091
W1	HPI4	well_3	F005	1922	Nucleus Width	11.65784	11.59167	2.423351
W1	HPI4	well_3	F006	1966	Nucleus Width	11.49232	11.375	2.56223
W1	HPI4	well_4	F001	1621	Nucleus Width	11.66585	11.59167	2.511495
W1	HPI4	well_4	F002	1883	Nucleus Width	11.44973	11.375	2.302212
W1	HPI4	well_4	F003	1990	Nucleus Width	11.65536	11.59167	2.432676
W1	HPI4	well_4	F004	1800	Nucleus Width	11.81923	11.7	2.422367
W1	HPI4	well_4	F005	1968	Nucleus Width	11.56668	11.48333	2.645959
W1	HPI4	well_4	F006	1865	Nucleus Width	11.57732	11.48333	2.522091
W1	HPI4	well_5	F001	1731	Nucleus Width	11.48427	11.375	2.693745
W1	HPI4	well_5	F002	1807	Nucleus Width	11.53501	11.48333	2.484924
W1	HPI4	well_5	F003	2045	Nucleus Width	11.33892	11.26667	2.4082
W1	HPI4	well_5	F004	1765	Nucleus Width	11.41704	11.26667	2.4211
W1	HPI4	well_5	F005	1696	Nucleus Width	11.91999	11.91667	2.585605
W1	HPI4	well_5	F006	1567	Nucleus Width	11.79471	11.59167	2.523619
W1	PGE2	well_1	F001	2088	Nucleus Width	11.57906	11.48333	2.412745
W1	PGE2	well_1	F002	1885	Nucleus Width	11.88517	11.91667	2.667758
W1	PGE2	well_1	F003	2089	Nucleus Width	11.76985	11.7	2.334667
W1	PGE2	well_1	F004	2227	Nucleus Width	11.56292	11.48333	2.46863
W1	PGE2	well_1	F005	2492	Nucleus Width	11.18546	10.94167	2.379881
W1	PGE2	well_1	F006	2566	Nucleus Width	11.31239	11.15833	2.382972
W1	PGE2	well_2	F001	1729	Nucleus Width	11.68853	11.7	2.302588
W1	PGE2	well_2	F002	1816	Nucleus Width	11.61284	11.48333	2.365016
W1	PGE2	well_2	F003	1887	Nucleus Width	11.54499	11.375	2.34415
W1	PGE2	well_2	F004	2061	Nucleus Width	11.49295	11.375	2.445485
W1	PGE2	well_2	F005	2098	Nucleus Width	11.64542	11.7	2.290647
W1	PGE2	well_2	F006	1676	Nucleus Width	11.74311	11.7	2.512601
W1	PGE2	well_3	F001	1851	Nucleus Width	11.88184	11.91667	2.454781
W1	PGE2	well_3	F002	1798	Nucleus Width	11.87292	11.7	2.568148
W1	PGE2	well_3	F003	1905	Nucleus Width	11.68544	11.59167	2.281476
W1	PGE2	well_3	F004	1902	Nucleus Width	11.91222	11.91667	2.535469

W1	PGE2	well_3	F005	2032	Nucleus Width	11.51015	11.26667	2.544169
W1	PGE2	well_3	F006	1874	Nucleus Width	11.71989	11.7	2.498888
W1	PGE2	well_4	F001	1627	Nucleus Width	12.04844	12.025	2.5046
W1	PGE2	well_4	F002	1758	Nucleus Width	11.7024	11.7	2.188188
W1	PGE2	well_4	F003	1669	Nucleus Width	11.83456	11.80833	2.628144
W1	PGE2	well_4	F004	2033	Nucleus Width	11.41965	11.26667	2.393122
W1	PGE2	well_4	F005	1768	Nucleus Width	11.86036	11.80833	2.436209
W1	PGE2	well_4	F006	1816	Nucleus Width	11.56619	11.5375	2.529213
W1	PGE2	well_5	F001	1629	Nucleus Width	11.90057	11.80833	2.591216
W1	PGE2	well_5	F002	1888	Nucleus Width	11.81832	11.7	2.515483
W1	PGE2	well_5	F003	1649	Nucleus Width	11.85623	11.91667	2.364626
W1	PGE2	well_5	F004	1777	Nucleus Width	11.87814	11.91667	2.489036
W1	PGE2	well_5	F005	1851	Nucleus Width	11.72312	11.7	2.430627
W1	PGE2	well_5	F006	1706	Nucleus Width	11.69651	11.59167	2.481542
W2	HPI4	well_1	F001	1616	Nucleus Width	10.74551	11.15833	2.886972
W2	HPI4	well_1	F002	1675	Nucleus Width	11.42791	11.59167	2.54742
W2	HPI4	well_1	F003	1796	Nucleus Width	10.96296	11.15833	2.52062
W2	HPI4	well_1	F004	1739	Nucleus Width	11.20923	11.26667	2.578472
W2	HPI4	well_1	F005	1621	Nucleus Width	11.22349	11.48333	2.622324
W2	HPI4	well_1	F006	1471	Nucleus Width	11.31336	11.59167	2.660015
W2	HPI4	well_2	F001	1461	Nucleus Width	10.75155	11.15833	3.082908
W2	HPI4	well_2	F002	1570	Nucleus Width	11.27108	11.48333	2.61293
W2	HPI4	well_2	F003	1570	Nucleus Width	11.23762	11.48333	2.595259
W2	HPI4	well_2	F004	1870	Nucleus Width	10.91166	11.15833	2.467099
W2	HPI4	well_2	F005	1644	Nucleus Width	11.23925	11.48333	2.616433
W2	HPI4	well_2	F006	1614	Nucleus Width	11.04893	11.375	2.685367
W2	HPI4	well_3	F001	1751	Nucleus Width	10.53085	11.05	2.916886
W2	HPI4	well_3	F002	1471	Nucleus Width	11.03078	11.26667	2.74872
W2	HPI4	well_3	F003	1127	Nucleus Width	11.3948	11.7	2.95771
W2	HPI4	well_3	F004	1733	Nucleus Width	11.11564	11.375	2.563588
W2	HPI4	well_3	F005	1760	Nucleus Width	10.79837	11.05	2.680533
W2	HPI4	well_3	F006	1654	Nucleus Width	11.05216	11.375	2.737327
W2	HPI4	well_4	F001	1628	Nucleus Width	10.3262	10.83333	2.759828
W2	HPI4	well_4	F002	1545	Nucleus Width	10.6539	10.725	2.53542
W2	HPI4	well_4	F003	1541	Nucleus Width	11.04648	11.375	2.47842
W2	HPI4	well_4	F004	1727	Nucleus Width	10.88207	11.05	2.492436
W2	HPI4	well_4	F005	1511	Nucleus Width	11.09811	11.375	2.750148
W2	HPI4	well_4	F006	1590	Nucleus Width	11.31429	11.375	2.641055
W2	HPI4	well_5	F001	1496	Nucleus Width	10.8497	11.05	2.72414
W2	HPI4	well_5	F002	1370	Nucleus Width	11.32083	11.7	2.830804
W2	HPI4	well_5	F003	1387	Nucleus Width	11.36828	11.48333	2.455597
W2	HPI4	well_5	F004	1683	Nucleus Width	11.14437	11.375	2.588387
W2	HPI4	well_5	F005	1752	Nucleus Width	10.80792	11.15833	2.73528
W2	HPI4	well_5	F006	1445	Nucleus Width	11.10121	11.48333	2.740302
W2	PGE2	well_1	F001	2222	Nucleus Width	9.945946	9.966667	2.154653
W2	PGE2	well_1	F002	1940	Nucleus Width	9.758879	9.641667	2.023531
W2	PGE2	well_1	F003	2718	Nucleus Width	10.02163	9.966667	1.989

W2	PGE2	well_1	F004	2149	Nucleus Width	10.02877	9.966667	1.983856
W2	PGE2	well_1	F005	2692	Nucleus Width	9.840103	9.75	2.064978
W2	PGE2	well_1	F006	2744	Nucleus Width	9.475969	9.641667	1.969473
W2	PGE2	well_2	F001	2270	Nucleus Width	9.708432	9.75	1.912979
W2	PGE2	well_2	F002	2877	Nucleus Width	9.647466	9.533333	1.971261
W2	PGE2	well_2	F003	3288	Nucleus Width	9.696888	9.641667	2.076844
W2	PGE2	well_2	F004	2973	Nucleus Width	9.78684	9.641667	2.04051
W2	PGE2	well_2	F005	2444	Nucleus Width	9.701684	9.75	1.964553
W2	PGE2	well_2	F006	2304	Nucleus Width	9.828758	9.858333	1.968887
W2	PGE2	well_3	F001	2012	Nucleus Width	9.715271	9.641667	2.130056
W2	PGE2	well_3	F002	3189	Nucleus Width	9.834791	9.858333	2.04249
W2	PGE2	well_3	F003	2876	Nucleus Width	9.814412	9.75	1.890512
W2	PGE2	well_3	F004	2935	Nucleus Width	9.48295	9.425	1.797116
W2	PGE2	well_3	F005	2421	Nucleus Width	9.63406	9.641667	1.899491
W2	PGE2	well_3	F006	2111	Nucleus Width	9.826875	9.75	2.070781
W2	PGE2	well_4	F001	2452	Nucleus Width	9.915283	9.858333	2.035508
W2	PGE2	well_4	F002	3055	Nucleus Width	9.680177	9.641667	1.967861
W2	PGE2	well_4	F003	2843	Nucleus Width	9.682249	9.641667	1.822381
W2	PGE2	well_4	F004	3219	Nucleus Width	9.903195	9.858333	1.991963
W2	PGE2	well_4	F005	2886	Nucleus Width	9.715878	9.641667	1.980799
W2	PGE2	well_4	F006	2381	Nucleus Width	9.862565	9.75	2.092412
W2	PGE2	well_5	F001	2804	Nucleus Width	9.811778	9.75	2.028828
W2	PGE2	well_5	F002	3207	Nucleus Width	9.806109	9.75	1.837966
W2	PGE2	well_5	F003	2872	Nucleus Width	9.886473	9.858333	1.981831
W2	PGE2	well_5	F004	2580	Nucleus Width	9.574399	9.533333	1.909881
W2	PGE2	well_5	F005	2615	Nucleus Width	9.619959	9.641667	1.826926
W2	PGE2	well_5	F006	2953	Nucleus Width	9.672593	9.641667	1.879195
W3	HPI4	well_1	F001	798	Nucleus Width	11.31282	11.80833	3.06066
W3	HPI4	well_1	F002	1010	Nucleus Width	11.40493	11.80833	2.84282
W3	HPI4	well_1	F003	908	Nucleus Width	11.1785	11.7	3.121425
W3	HPI4	well_1	F004	1248	Nucleus Width	10.93116	11.375	2.620244
W3	HPI4	well_1	F005	1235	Nucleus Width	11.26947	11.59167	2.821273
W3	HPI4	well_1	F006	914	Nucleus Width	11.37488	11.80833	2.925069
W3	HPI4	well_2	F001	797	Nucleus Width	11.05421	11.59167	3.090512
W3	HPI4	well_2	F002	971	Nucleus Width	11.33104	11.80833	2.818548
W3	HPI4	well_2	F003	933	Nucleus Width	11.51805	11.7	2.980341
W3	HPI4	well_2	F004	1129	Nucleus Width	11.33364	11.59167	2.678355
W3	HPI4	well_2	F005	1253	Nucleus Width	11.08536	11.59167	2.904434
W3	HPI4	well_2	F006	1058	Nucleus Width	11.3837	11.7	2.809942
W3	HPI4	well_3	F001	795	Nucleus Width	11.35619	11.80833	2.983321
W3	HPI4	well_3	F002	885	Nucleus Width	11.55139	11.80833	2.652265
W3	HPI4	well_3	F003	621	Nucleus Width	11.6679	11.91667	2.686268
W3	HPI4	well_3	F004	986	Nucleus Width	10.8986	11.375	2.878037
W3	HPI4	well_3	F005	1121	Nucleus Width	10.98139	11.15833	2.977522
W3	HPI4	well_3	F006	1047	Nucleus Width	10.92501	11.15833	2.851666
W3	HPI4	well_4	F001	722	Nucleus Width	11.34979	11.7	2.953692
W3	HPI4	well_4	F002	918	Nucleus Width	11.7085	12.025	2.84443

W3	HPI4	well_4	F003	708	Nucleus Width	11.78905	12.13333	2.823788
W3	HPI4	well_4	F004	785	Nucleus Width	11.04545	11.59167	3.079428
W3	HPI4	well_4	F005	1210	Nucleus Width	11.29075	11.48333	2.613041
W3	HPI4	well_4	F006	1129	Nucleus Width	11.16716	11.375	2.751851
W3	HPI4	well_5	F001	712	Nucleus Width	11.52426	12.025	3.431941
W3	HPI4	well_5	F002	558	Nucleus Width	11.44645	11.80833	2.763249
W3	HPI4	well_5	F003	812	Nucleus Width	11.22237	11.59167	2.722707
W3	HPI4	well_5	F004	517	Nucleus Width	11.51267	11.80833	3.155468
W3	HPI4	well_5	F005	976	Nucleus Width	11.33249	11.59167	2.747527
W3	HPI4	well_5	F006	931	Nucleus Width	11.5158	11.80833	2.937329
W3	PGE2	well_1	F001	2659	Nucleus Width	9.741077	9.641667	2.088318
W3	PGE2	well_1	F002	3405	Nucleus Width	9.640999	9.75	1.998017
W3	PGE2	well_1	F003	3522	Nucleus Width	9.659999	9.641667	1.960704
W3	PGE2	well_1	F004	3028	Nucleus Width	9.348866	9.208333	2.013909
W3	PGE2	well_1	F005	3384	Nucleus Width	9.594767	9.425	2.083795
W3	PGE2	well_1	F006	2846	Nucleus Width	9.842651	9.858333	1.931249
W3	PGE2	well_2	F001	2912	Nucleus Width	9.828125	9.641667	2.082858
W3	PGE2	well_2	F002	2973	Nucleus Width	9.754883	9.641667	1.975631
W3	PGE2	well_2	F003	3367	Nucleus Width	9.578893	9.533333	1.885505
W3	PGE2	well_2	F004	3533	Nucleus Width	9.714277	9.641667	2.036664
W3	PGE2	well_2	F005	3705	Nucleus Width	9.741462	9.641667	2.087332
W3	PGE2	well_2	F006	3844	Nucleus Width	9.641554	9.641667	1.852481
W3	PGE2	well_3	F001	3127	Nucleus Width	9.727343	9.641667	2.262765
W3	PGE2	well_3	F002	2777	Nucleus Width	9.902104	9.858333	2.254261
W3	PGE2	well_3	F003	3810	Nucleus Width	9.54792	9.425	2.03357
W3	PGE2	well_3	F004	3332	Nucleus Width	9.689266	9.533333	2.104013
W3	PGE2	well_3	F005	3851	Nucleus Width	9.572267	9.533333	2.02603
W3	PGE2	well_3	F006	3599	Nucleus Width	9.912485	9.858333	2.04322
W3	PGE2	well_4	F001	2774	Nucleus Width	9.581876	9.533333	2.055612
W3	PGE2	well_4	F002	3347	Nucleus Width	9.656685	9.533333	2.200264
W3	PGE2	well_4	F003	2729	Nucleus Width	9.747221	9.641667	2.072685
W3	PGE2	well_4	F004	3120	Nucleus Width	9.791007	9.75	2.161001
W3	PGE2	well_4	F005	3555	Nucleus Width	9.768741	9.75	2.136324
W3	PGE2	well_4	F006	3216	Nucleus Width	9.829566	9.75	2.00219
W3	PGE2	well_5	F001	2321	Nucleus Width	9.724655	9.858333	2.184759
W3	PGE2	well_5	F002	2453	Nucleus Width	9.838283	9.858333	2.148691
W3	PGE2	well_5	F003	3024	Nucleus Width	9.572454	9.533333	2.168048
W3	PGE2	well_5	F004	2780	Nucleus Width	9.519382	9.425	2.063624
W3	PGE2	well_5	F005	3314	Nucleus Width	9.54484	9.425	1.930982
W3	PGE2	well_5	F006	2620	Nucleus Width	9.769517	9.641667	1.96853
W4	HPI4	well_1	F001	540	Nucleus Width	11.46247	12.025	3.399403
W4	HPI4	well_1	F002	888	Nucleus Width	11.01669	11.375	2.893238
W4	HPI4	well_1	F003	835	Nucleus Width	11.16651	11.48333	2.714587
W4	HPI4	well_1	F004	991	Nucleus Width	11.34319	11.7	2.590769
W4	HPI4	well_1	F005	1090	Nucleus Width	10.99544	11.375	2.971398
W4	HPI4	well_1	F006	991	Nucleus Width	11.25213	11.59167	2.935003
W4	HPI4	well_2	F001	653	Nucleus Width	11.41946	11.91667	3.220699

W4	HPI4	well_2	F002	918	Nucleus Width	11.02262	11.26667	2.662491
W4	HPI4	well_2	F003	956	Nucleus Width	11.32956	11.59167	2.861724
W4	HPI4	well_2	F004	1031	Nucleus Width	11.40631	11.7	2.601038
W4	HPI4	well_2	F005	1024	Nucleus Width	11.13157	11.59167	2.78236
W4	HPI4	well_2	F006	933	Nucleus Width	11.5739	11.80833	2.609311
W4	HPI4	well_3	F001	759	Nucleus Width	11.46264	11.80833	2.866038
W4	HPI4	well_3	F002	840	Nucleus Width	11.53363	11.91667	2.682635
W4	HPI4	well_3	F003	927	Nucleus Width	11.50986	11.80833	3.002367
W4	HPI4	well_3	F004	1041	Nucleus Width	11.11515	11.59167	2.655742
W4	HPI4	well_3	F005	1065	Nucleus Width	11.03352	11.375	2.577407
W4	HPI4	well_3	F006	813	Nucleus Width	11.18858	11.59167	2.974099
W4	HPI4	well_4	F001	772	Nucleus Width	11.75108	12.025	3.067898
W4	HPI4	well_4	F002	962	Nucleus Width	11.57185	11.91667	2.883692
W4	HPI4	well_4	F003	701	Nucleus Width	11.92949	12.35	2.920921
W4	HPI4	well_4	F004	1062	Nucleus Width	11.12202	11.59167	2.825788
W4	HPI4	well_4	F005	1118	Nucleus Width	10.85872	11.375	2.95504
W4	HPI4	well_4	F006	845	Nucleus Width	11.20231	11.80833	3.034122
W4	HPI4	well_5	F001	791	Nucleus Width	11.8145	12.13333	2.940451
W4	HPI4	well_5	F002	598	Nucleus Width	12.00616	12.35	3.029087
W4	HPI4	well_5	F003	752	Nucleus Width	12.04978	12.24167	3.017361
W4	HPI4	well_5	F004	949	Nucleus Width	11.82032	12.13333	3.015595
W4	HPI4	well_5	F005	1195	Nucleus Width	11.16024	11.7	2.901839
W4	HPI4	well_5	F006	1013	Nucleus Width	11.12058	11.48333	2.753679
W4	PGE2	well_1	F001	2025	Nucleus Width	9.640383	9.75	1.874548
W4	PGE2	well_1	F002	1830	Nucleus Width	9.621066	9.75	1.919988
W4	PGE2	well_1	F003	2816	Nucleus Width	9.710452	9.75	1.943686
W4	PGE2	well_1	F004	2679	Nucleus Width	9.388404	9.425	1.891474
W4	PGE2	well_1	F005	2927	Nucleus Width	9.505797	9.533333	2.0117
W4	PGE2	well_1	F006	2858	Nucleus Width	9.7149	9.75	2.01127
W4	PGE2	well_2	F001	2324	Nucleus Width	9.778155	9.858333	2.123111
W4	PGE2	well_2	F002	2806	Nucleus Width	9.564876	9.425	1.948744
W4	PGE2	well_2	F003	2502	Nucleus Width	9.560005	9.641667	2.042936
W4	PGE2	well_2	F004	2867	Nucleus Width	9.420919	9.425	1.978746
W4	PGE2	well_2	F005	3262	Nucleus Width	9.429351	9.425	1.910716
W4	PGE2	well_2	F006	2745	Nucleus Width	9.63259	9.641667	1.838604
W4	PGE2	well_3	F001	2165	Nucleus Width	9.639065	9.75	1.939967
W4	PGE2	well_3	F002	3094	Nucleus Width	9.596639	9.641667	1.79961
W4	PGE2	well_3	F003	3177	Nucleus Width	9.461929	9.533333	1.90133
W4	PGE2	well_3	F004	3019	Nucleus Width	9.51087	9.425	1.993539
W4	PGE2	well_3	F005	2629	Nucleus Width	9.685387	9.641667	2.090294
W4	PGE2	well_3	F006	2911	Nucleus Width	9.725885	9.75	1.844665
W4	PGE2	well_4	F001	2186	Nucleus Width	9.430253	9.533333	1.917813
W4	PGE2	well_4	F002	2767	Nucleus Width	9.429385	9.316667	1.967684
W4	PGE2	well_4	F003	2924	Nucleus Width	9.521329	9.533333	1.93933
W4	PGE2	well_4	F004	2783	Nucleus Width	9.480081	9.425	2.007303
W4	PGE2	well_4	F005	3493	Nucleus Width	9.469196	9.425	1.829591
W4	PGE2	well_4	F006	2292	Nucleus Width	9.57989	9.641667	1.87898

W4	PGE2	well_5	F001	1871	Nucleus Width	9.741257	9.858333	2.084494
W4	PGE2	well_5	F002	2315	Nucleus Width	9.603247	9.641667	2.132456
W4	PGE2	well_5	F003	2729	Nucleus Width	9.145771	9.1	1.987655
W4	PGE2	well_5	F004	2705	Nucleus Width	9.33553	9.208333	2.03272
W4	PGE2	well_5	F005	2441	Nucleus Width	9.31356	9.316667	1.937633
W4	PGE2	well_5	F006	2162	Nucleus Width	9.540649	9.533333	2.13755

Table S10

Week	Treatment	mean	median	sd	Metric
W1	HPI4	246.6472	245.8339	11.34097	Cell Area
W1	PGE2	250.7003	249.3649	10.68075	Cell Area
W2	HPI4	244.4281	242.8142	16.34732	Cell Area
W2	PGE2	172.8955	172.357	7.178978	Cell Area
W3	HPI4	292.0758	295.4343	19.09591	Cell Area
W3	PGE2	191.6926	191.68	6.676774	Cell Area
W4	HPI4	319.6179	315.2461	25.19483	Cell Area
W4	PGE2	198.326	198.9845	7.699555	Cell Area
W1	HPI4	1.679921	1.681907	0.018349	Cell AR
W1	PGE2	1.685288	1.687944	0.025577	Cell AR
W2	HPI4	1.678297	1.682012	0.029985	Cell AR
W2	PGE2	1.567948	1.564439	0.024423	Cell AR
W3	HPI4	1.766743	1.768786	0.033366	Cell AR
W3	PGE2	1.59376	1.588844	0.027571	Cell AR
W4	HPI4	1.807365	1.805635	0.040745	Cell AR
W4	PGE2	1.602266	1.606304	0.032263	Cell AR
W1	HPI4	797.8425	795.0863	33.73205	Cell Volume
W1	PGE2	829.2331	831.0227	26.10755	Cell Volume
W2	HPI4	746.1454	749.1143	49.77023	Cell Volume
W2	PGE2	720.3617	718.8938	27.40783	Cell Volume
W3	HPI4	838.633	837.6534	56.09944	Cell Volume
W3	PGE2	776.6818	781.1513	29.12124	Cell Volume
W4	HPI4	925.0055	932.3818	76.98063	Cell Volume
W4	PGE2	792.0652	799.0138	38.17704	Cell Volume
W1	HPI4	4.526433	4.53479	0.186622	Cell Height
W1	PGE2	4.647321	4.621119	0.135903	Cell Height
W2	HPI4	3.99131	4.033761	0.1513	Cell Height
W2	PGE2	5.890957	5.891883	0.226267	Cell Height
W3	HPI4	3.570576	3.615438	0.206586	Cell Height
W3	PGE2	5.664749	5.653717	0.239069	Cell Height
W4	HPI4	3.510862	3.530555	0.165295	Cell Height
W4	PGE2	5.347095	5.354152	0.205777	Cell Height
W1	HPI4	19.00265	18.97801	0.439202	Cell Width
W1	PGE2	19.1883	19.24135	0.421734	Cell Width
W2	HPI4	18.85436	18.80384	0.651762	Cell Width
W2	PGE2	15.88731	15.87316	0.341789	Cell Width
W3	HPI4	20.72633	20.75613	0.692645	Cell Width
W3	PGE2	16.75183	16.72643	0.309419	Cell Width
W4	HPI4	21.78827	21.79297	0.830107	Cell Width
W4	PGE2	17.08859	17.10526	0.337246	Cell Width
W1	HPI4	101.5993	101.6037	3.238789	Nucleus Area
W1	PGE2	102.3619	102.2659	3.345728	Nucleus Area
W2	HPI4	92.18443	92.9629	4.199631	Nucleus Area
W2	PGE2	71.33851	71.41983	2.003204	Nucleus Area

W3	HPI4	96.97971	97.20465	4.104461	Nucleus Area
W3	PGE2	70.49165	70.7332	1.808936	Nucleus Area
W4	HPI4	98.45421	96.90384	6.000127	Nucleus Area
W4	PGE2	68.02148	68.36978	1.992393	Nucleus Area
W1	HPI4	1.629125	1.63155	0.033965	Nucleus AR
W1	PGE2	1.619778	1.618706	0.025149	Nucleus AR
W2	HPI4	1.651916	1.65225	0.025768	Nucleus AR
W2	PGE2	1.498438	1.495292	0.024674	Nucleus AR
W3	HPI4	1.781539	1.777162	0.05473	Nucleus AR
W3	PGE2	1.510796	1.507347	0.026322	Nucleus AR
W4	HPI4	1.764641	1.759558	0.061678	Nucleus AR
W4	PGE2	1.514803	1.508481	0.034067	Nucleus AR
W1	HPI4	372.7557	372.872	11.02692	Nucleus Volume
W1	PGE2	383.1021	385.4627	10.17176	Nucleus Volume
W2	HPI4	298.4583	298.8461	15.55892	Nucleus Volume
W2	PGE2	297.8123	298.7622	8.394385	Nucleus Volume
W3	HPI4	272.3898	274.0259	14.12415	Nucleus Volume
W3	PGE2	265.3363	264.4524	6.465467	Nucleus Volume
W4	HPI4	279.1706	276.1707	20.3864	Nucleus Volume
W4	PGE2	257.1566	255.465	9.841911	Nucleus Volume
W1	HPI4	4.533628	4.525423	0.191695	Nucleus Height
W1	PGE2	4.612521	4.603486	0.148627	Nucleus Height
W2	HPI4	3.81835	3.840905	0.167647	Nucleus Height
W2	PGE2	5.342801	5.335938	0.177721	Nucleus Height
W3	HPI4	3.182476	3.210514	0.187634	Nucleus Height
W3	PGE2	4.750954	4.750512	0.159101	Nucleus Height
W4	HPI4	3.191963	3.179871	0.14795	Nucleus Height
W4	PGE2	4.629422	4.64574	0.134717	Nucleus Height
W1	HPI4	11.58391	11.58974	0.182939	Nucleus Width
W1	PGE2	11.63386	11.65692	0.190566	Nucleus Width
W2	HPI4	10.94718	10.98229	0.272187	Nucleus Width
W2	PGE2	9.708055	9.716487	0.14012	Nucleus Width
W3	HPI4	11.18941	11.22528	0.239738	Nucleus Width
W3	PGE2	9.642591	9.657397	0.125086	Nucleus Width
W4	HPI4	11.25569	11.23235	0.329308	Nucleus Width
W4	PGE2	9.48755	9.490372	0.141559	Nucleus Width

Table S11

Line	Week	Treatment	mean	median	sd	Metric
ACTB	W1	PGE2	7.999321	7.993082	0.608366	X/Y distribution
ACTB	W1	HPI4	7.661851	7.645076	0.578039	X/Y distribution
ACTB	W2	PGE2	5.298201	5.229909	0.514801	X/Y distribution
ACTB	W2	HPI4	4.474	4.399867	1.624012	X/Y distribution
ACTB	W3	PGE2	7.150573	7.072148	0.403022	X/Y distribution
ACTB	W3	HPI4	7.858906	7.980864	0.860769	X/Y distribution
ACTB	W4	PGE2	7.357982	7.354374	0.378458	X/Y distribution
ACTB	W4	HPI4	7.101141	7.284834	1.265883	X/Y distribution
MYH10	W1	PGE2	8.288095	8.211405	0.726817	X/Y distribution
MYH10	W1	HPI4	8.42357	8.387502	0.560209	X/Y distribution
MYH10	W2	PGE2	5.759156	5.759465	0.275571	X/Y distribution
MYH10	W2	HPI4	7.114658	7.079687	0.607834	X/Y distribution
MYH10	W3	PGE2	5.799152	5.808292	0.254491	X/Y distribution
MYH10	W3	HPI4	7.716332	7.682444	0.702619	X/Y distribution
MYH10	W4	PGE2	5.88016	5.879538	0.288658	X/Y distribution
MYH10	W4	HPI4	8.126193	7.828607	1.050439	X/Y distribution
FBL	W1	PGE2	3.496021	3.499388	0.25881	X/Y distribution
FBL	W1	HPI4	3.471524	3.401697	0.382949	X/Y distribution
FBL	W2	PGE2	3.249522	3.223048	0.260309	X/Y distribution
FBL	W2	HPI4	2.768808	2.837889	0.569911	X/Y distribution
FBL	W3	PGE2	2.840976	2.827199	0.205732	X/Y distribution
FBL	W3	HPI4	3.445489	3.425931	0.683878	X/Y distribution
FBL	W4	PGE2	3.253748	3.283865	0.460977	X/Y distribution
FBL	W4	HPI4	5.248879	5.396442	1.263963	X/Y distribution
DSP	W1	PGE2	8.693746	8.642735	0.90515	X/Y distribution
DSP	W1	HPI4	8.689141	8.671229	0.722538	X/Y distribution
DSP	W2	PGE2	6.163519	6.06301	0.449289	X/Y distribution
DSP	W2	HPI4	7.976204	7.89295	0.951557	X/Y distribution
DSP	W3	PGE2	5.975327	6.059813	0.416205	X/Y distribution
DSP	W3	HPI4	8.620872	8.400218	1.61008	X/Y distribution
DSP	W4	PGE2	6.060471	6.064627	0.469211	X/Y distribution
DSP	W4	HPI4	8.444856	8.391807	0.980873	X/Y distribution
GJA1	W1	PGE2	7.853266	8.121644	1.52088	X/Y distribution
GJA1	W1	HPI4	7.643336	7.696331	1.066213	X/Y distribution
GJA1	W2	PGE2	6.763049	6.710809	0.853453	X/Y distribution
GJA1	W2	HPI4	6.69809	6.79079	1.666817	X/Y distribution
GJA1	W3	PGE2	7.376215	7.406686	0.856876	X/Y distribution
GJA1	W3	HPI4	6.314873	6.26627	1.610526	X/Y distribution
GJA1	W4	PGE2	7.023247	6.944946	0.872895	X/Y distribution
GJA1	W4	HPI4	8.681295	8.462218	1.477104	X/Y distribution
TJP1	W1	PGE2	7.59996	7.422312	0.589346	X/Y distribution
TJP1	W1	HPI4	7.377716	7.306474	0.619195	X/Y distribution
TJP1	W2	PGE2	6.689496	6.248021	1.109729	X/Y distribution
TJP1	W2	HPI4	7.627083	7.527967	0.49323	X/Y distribution

TJP1	W3	PGE2	6.145458	6.173138	0.198401	X/Y distribution
TJP1	W3	HPI4	8.703424	8.647357	0.709516	X/Y distribution
TJP1	W4	PGE2	6.708812	6.665043	0.520656	X/Y distribution
TJP1	W4	HPI4	9.361892	9.348104	0.752678	X/Y distribution
CTNNB1	W1	PGE2	6.986235	6.950445	0.436584	X/Y distribution
CTNNB1	W1	HPI4	7.023402	7.009501	0.421112	X/Y distribution
CTNNB1	W2	PGE2	5.992248	6.020617	0.777113	X/Y distribution
CTNNB1	W2	HPI4	4.581246	4.013463	1.499905	X/Y distribution
CTNNB1	W3	PGE2	7.270658	7.273669	0.444791	X/Y distribution
CTNNB1	W3	HPI4	4.848887	4.445379	1.435614	X/Y distribution
CTNNB1	W4	PGE2	7.158428	7.156523	0.391081	X/Y distribution
CTNNB1	W4	HPI4	9.76031	10.18562	2.586719	X/Y distribution
CETN2	W1	PGE2	3.718543	3.219556	1.415504	X/Y distribution
CETN2	W1	HPI4	3.680193	3.337471	1.946887	X/Y distribution
CETN2	W2	PGE2	3.395476	3.268633	0.738926	X/Y distribution
CETN2	W2	HPI4	5.153006	5.233947	0.75334	X/Y distribution
CETN2	W3	PGE2	5.110347	4.876212	1.570655	X/Y distribution
CETN2	W3	HPI4	4.999421	4.859668	1.926319	X/Y distribution
CETN2	W4	PGE2	4.916062	4.965908	1.223457	X/Y distribution
CETN2	W4	HPI4	4.51464	4.58562	1.92121	X/Y distribution
SEC61B	W1	PGE2	8.23142	8.198237	0.604541	X/Y distribution
SEC61B	W1	HPI4	8.102059	8.110551	0.856113	X/Y distribution
SEC61B	W2	PGE2	5.460078	5.434322	0.258745	X/Y distribution
SEC61B	W2	HPI4	7.779698	8.005598	0.689307	X/Y distribution
SEC61B	W3	PGE2	5.394098	5.394413	0.165452	X/Y distribution
SEC61B	W3	HPI4	8.426639	8.614289	0.642344	X/Y distribution
SEC61B	W4	PGE2	5.474878	5.502656	0.174207	X/Y distribution
SEC61B	W4	HPI4	8.412412	8.390734	0.620241	X/Y distribution
ST6GAL1	W1	PGE2	6.525392	6.180542	1.522937	X/Y distribution
ST6GAL1	W1	HPI4	6.492782	6.459179	0.945393	X/Y distribution
ST6GAL1	W2	PGE2	5.199753	5.168461	0.627976	X/Y distribution
ST6GAL1	W2	HPI4	6.927928	6.814449	1.07438	X/Y distribution
ST6GAL1	W3	PGE2	6.134848	6.076455	0.65112	X/Y distribution
ST6GAL1	W3	HPI4	6.705346	6.848704	1.213498	X/Y distribution
ST6GAL1	W4	PGE2	5.890911	5.869534	0.545531	X/Y distribution
ST6GAL1	W4	HPI4	5.938951	6.112371	1.203076	X/Y distribution
TOM20	W1	PGE2	6.925465	6.896542	0.435788	X/Y distribution
TOM20	W1	HPI4	6.672381	6.691665	0.478885	X/Y distribution
TOM20	W2	PGE2	4.765545	4.762037	0.139261	X/Y distribution
TOM20	W2	HPI4	6.998304	7.013266	0.62272	X/Y distribution
TOM20	W3	PGE2	4.623998	4.609516	0.204193	X/Y distribution
TOM20	W3	HPI4	7.428276	7.46013	0.562114	X/Y distribution
TOM20	W4	PGE2	4.671732	4.650626	0.222386	X/Y distribution
TOM20	W4	HPI4	8.126977	8.19049	0.380392	X/Y distribution
LAMP1	W1	PGE2	5.350178	5.347927	0.45091	X/Y distribution
LAMP1	W1	HPI4	5.155106	5.072539	0.393083	X/Y distribution
LAMP1	W2	PGE2	3.949721	3.946111	0.168478	X/Y distribution

LAMP1	W2	HPI4	5.970587	5.794298	0.537366	X/Y distribution
LAMP1	W3	PGE2	3.833359	3.853416	0.160305	X/Y distribution
LAMP1	W3	HPI4	6.401117	6.102867	0.944975	X/Y distribution
LAMP1	W4	PGE2	3.879483	3.819189	0.258595	X/Y distribution
LAMP1	W4	HPI4	6.739844	6.630087	0.78563	X/Y distribution
LC3B	W1	PGE2	5.158682	5.085295	0.374684	X/Y distribution
LC3B	W1	HPI4	5.086854	5.075382	0.447051	X/Y distribution
LC3B	W2	PGE2	5.620774	5.674705	0.378884	X/Y distribution
LC3B	W2	HPI4	5.372097	5.309214	0.780407	X/Y distribution
LC3B	W3	PGE2	5.951903	5.9956	0.323875	X/Y distribution
LC3B	W3	HPI4	6.983472	7.294795	1.807982	X/Y distribution
LC3B	W4	PGE2	5.743837	5.708257	0.245428	X/Y distribution
LC3B	W4	HPI4	7.09243	6.596253	3.494701	X/Y distribution
RAB5	W1	PGE2	6.020903	6.103036	0.48855	X/Y distribution
RAB5	W1	HPI4	5.967938	6.024844	0.674972	X/Y distribution
RAB5	W2	PGE2	5.181714	5.22884	0.470715	X/Y distribution
RAB5	W2	HPI4	5.06772	4.857977	1.210446	X/Y distribution
RAB5	W3	PGE2	6.390409	6.434056	0.419555	X/Y distribution
RAB5	W3	HPI4	4.704265	4.052152	1.975493	X/Y distribution
RAB5	W4	PGE2	5.960581	6.011931	0.570845	X/Y distribution
RAB5	W4	HPI4	8.279909	8.252202	1.439009	X/Y distribution
SLC25A17	W1	PGE2	5.282329	5.248673	0.401541	X/Y distribution
SLC25A17	W1	HPI4	5.466914	5.391687	0.406124	X/Y distribution
SLC25A17	W2	PGE2	5.134053	5.093752	0.400926	X/Y distribution
SLC25A17	W2	HPI4	4.582454	4.65351	0.615648	X/Y distribution
SLC25A17	W3	PGE2	5.546964	5.493785	0.356836	X/Y distribution
SLC25A17	W3	HPI4	6.01576	5.763907	1.479938	X/Y distribution
SLC25A17	W4	PGE2	6.019456	6.056123	0.27369	X/Y distribution
SLC25A17	W4	HPI4	6.222104	6.490793	1.409692	X/Y distribution
ACTB	W1	PGE2	1.143991	1.163942	0.158345	Avg. Volume
ACTB	W1	HPI4	1.200079	1.151524	0.180418	Avg. Volume
ACTB	W2	PGE2	1.627259	1.661222	0.282735	Avg. Volume
ACTB	W2	HPI4	1.544752	0.711579	1.851111	Avg. Volume
ACTB	W3	PGE2	1.482323	1.425829	0.211961	Avg. Volume
ACTB	W3	HPI4	1.071215	1.065257	0.227639	Avg. Volume
ACTB	W4	PGE2	1.528482	1.587626	0.269415	Avg. Volume
ACTB	W4	HPI4	1.033385	0.907911	0.450973	Avg. Volume
MYH10	W1	PGE2	1.271709	1.25704	0.146307	Avg. Volume
MYH10	W1	HPI4	1.277801	1.285359	0.145831	Avg. Volume
MYH10	W2	PGE2	1.992359	2.010487	0.270925	Avg. Volume
MYH10	W2	HPI4	1.161497	1.174101	0.138049	Avg. Volume
MYH10	W3	PGE2	2.169272	2.19618	0.172539	Avg. Volume
MYH10	W3	HPI4	1.08309	1.071898	0.173121	Avg. Volume
MYH10	W4	PGE2	2.207228	2.201543	0.253551	Avg. Volume
MYH10	W4	HPI4	1.248506	1.212858	0.286006	Avg. Volume
FBL	W1	PGE2	5.153083	5.128439	0.535695	Avg. Volume
FBL	W1	HPI4	5.154413	5.149073	0.493135	Avg. Volume

FBL	W2	PGE2	4.552862	4.59941	0.503223	Avg. Volume
FBL	W2	HPI4	6.339517	6.479428	1.310185	Avg. Volume
FBL	W3	PGE2	6.050995	6.016479	0.476648	Avg. Volume
FBL	W3	HPI4	8.738051	8.884743	1.955745	Avg. Volume
FBL	W4	PGE2	3.192355	3.208565	0.453001	Avg. Volume
FBL	W4	HPI4	4.027771	3.955832	1.14012	Avg. Volume
DSP	W1	PGE2	0.380548	0.388553	0.033427	Avg. Volume
DSP	W1	HPI4	0.388447	0.394896	0.034113	Avg. Volume
DSP	W2	PGE2	0.527068	0.524892	0.069748	Avg. Volume
DSP	W2	HPI4	0.386782	0.370741	0.066957	Avg. Volume
DSP	W3	PGE2	0.470532	0.471265	0.079654	Avg. Volume
DSP	W3	HPI4	0.397958	0.406742	0.070434	Avg. Volume
DSP	W4	PGE2	0.470327	0.475464	0.062219	Avg. Volume
DSP	W4	HPI4	0.447791	0.450188	0.073528	Avg. Volume
GJA1	W1	PGE2	0.785799	0.557067	1.258691	Avg. Volume
GJA1	W1	HPI4	0.481977	0.421473	0.278042	Avg. Volume
GJA1	W2	PGE2	0.538343	0.553887	0.102151	Avg. Volume
GJA1	W2	HPI4	0.569507	0.478849	0.47946	Avg. Volume
GJA1	W3	PGE2	0.495439	0.460741	0.160837	Avg. Volume
GJA1	W3	HPI4	0.546988	0.456217	0.268988	Avg. Volume
GJA1	W4	PGE2	0.500994	0.437234	0.177939	Avg. Volume
GJA1	W4	HPI4	0.566894	0.55081	0.144351	Avg. Volume
TJP1	W1	PGE2	1.243816	1.23024	0.17098	Avg. Volume
TJP1	W1	HPI4	1.181825	1.185302	0.203893	Avg. Volume
TJP1	W2	PGE2	1.343131	1.383632	0.335916	Avg. Volume
TJP1	W2	HPI4	1.073935	0.977323	0.281245	Avg. Volume
TJP1	W3	PGE2	2.502049	2.241683	0.805917	Avg. Volume
TJP1	W3	HPI4	1.698175	1.594555	0.408371	Avg. Volume
TJP1	W4	PGE2	2.247562	2.09513	0.866373	Avg. Volume
TJP1	W4	HPI4	1.473042	1.539548	0.426649	Avg. Volume
CTNNB1	W1	PGE2	3.24078	3.19628	0.598093	Avg. Volume
CTNNB1	W1	HPI4	3.07751	2.986283	0.500887	Avg. Volume
CTNNB1	W2	PGE2	3.711934	3.690175	1.044314	Avg. Volume
CTNNB1	W2	HPI4	2.387014	1.739552	2.115306	Avg. Volume
CTNNB1	W3	PGE2	1.709891	1.670674	0.398697	Avg. Volume
CTNNB1	W3	HPI4	2.278804	1.579592	2.863998	Avg. Volume
CTNNB1	W4	PGE2	1.676844	1.607074	0.308562	Avg. Volume
CTNNB1	W4	HPI4	1.373445	0.961524	1.792573	Avg. Volume
CETN2	W1	PGE2	1.640048	1.665026	0.253088	Avg. Volume
CETN2	W1	HPI4	1.670975	1.637823	0.377122	Avg. Volume
CETN2	W2	PGE2	1.58092	1.63532	0.331474	Avg. Volume
CETN2	W2	HPI4	1.661021	1.59845	0.315696	Avg. Volume
CETN2	W3	PGE2	1.536347	1.503813	0.287	Avg. Volume
CETN2	W3	HPI4	1.643904	1.474205	0.825827	Avg. Volume
CETN2	W4	PGE2	1.847705	1.827503	0.288702	Avg. Volume
CETN2	W4	HPI4	1.723532	1.47875	0.699569	Avg. Volume
SEC61B	W1	PGE2	2.045515	2.105764	0.413579	Avg. Volume

SEC61B	W1	HPI4	1.97642	1.956465	0.371854	Avg. Volume
SEC61B	W2	PGE2	2.23993	2.2789	0.37174	Avg. Volume
SEC61B	W2	HPI4	1.424174	1.417242	0.239599	Avg. Volume
SEC61B	W3	PGE2	2.635173	2.563899	0.466075	Avg. Volume
SEC61B	W3	HPI4	1.476274	1.419498	0.431207	Avg. Volume
SEC61B	W4	PGE2	2.457829	2.50981	0.564193	Avg. Volume
SEC61B	W4	HPI4	1.195176	1.263405	0.308156	Avg. Volume
ST6GAL1	W1	PGE2	1.09183	1.084123	0.748676	Avg. Volume
ST6GAL1	W1	HPI4	1.404694	1.296205	0.671341	Avg. Volume
ST6GAL1	W2	PGE2	1.392369	1.287421	0.444635	Avg. Volume
ST6GAL1	W2	HPI4	0.936131	0.941542	0.373967	Avg. Volume
ST6GAL1	W3	PGE2	0.953714	0.907448	0.328203	Avg. Volume
ST6GAL1	W3	HPI4	0.805659	0.77173	0.335654	Avg. Volume
ST6GAL1	W4	PGE2	0.605979	0.552034	0.238364	Avg. Volume
ST6GAL1	W4	HPI4	0.672341	0.60552	0.309142	Avg. Volume
TOM20	W1	PGE2	3.88246	3.854016	0.458433	Avg. Volume
TOM20	W1	HPI4	3.859425	3.90326	0.445906	Avg. Volume
TOM20	W2	PGE2	4.347876	4.202047	0.659689	Avg. Volume
TOM20	W2	HPI4	2.813705	2.756008	0.521648	Avg. Volume
TOM20	W3	PGE2	4.916073	4.936354	0.593053	Avg. Volume
TOM20	W3	HPI4	2.511965	2.43153	0.574445	Avg. Volume
TOM20	W4	PGE2	3.933182	4.010203	0.659698	Avg. Volume
TOM20	W4	HPI4	3.175705	3.091077	0.677279	Avg. Volume
LAMP1	W1	PGE2	0.453987	0.453065	0.023674	Avg. Volume
LAMP1	W1	HPI4	0.442164	0.440845	0.023536	Avg. Volume
LAMP1	W2	PGE2	0.433268	0.438823	0.027206	Avg. Volume
LAMP1	W2	HPI4	0.227098	0.219531	0.040631	Avg. Volume
LAMP1	W3	PGE2	0.487202	0.488816	0.041029	Avg. Volume
LAMP1	W3	HPI4	0.217787	0.211166	0.039322	Avg. Volume
LAMP1	W4	PGE2	0.445859	0.44505	0.069043	Avg. Volume
LAMP1	W4	HPI4	0.227305	0.218704	0.046106	Avg. Volume
LC3B	W1	PGE2	0.465276	0.471513	0.052163	Avg. Volume
LC3B	W1	HPI4	0.481962	0.489373	0.048941	Avg. Volume
LC3B	W2	PGE2	0.33489	0.330926	0.039871	Avg. Volume
LC3B	W2	HPI4	0.363536	0.352632	0.065833	Avg. Volume
LC3B	W3	PGE2	0.427827	0.42995	0.04081	Avg. Volume
LC3B	W3	HPI4	0.419323	0.406852	0.172593	Avg. Volume
LC3B	W4	PGE2	0.436449	0.437993	0.048378	Avg. Volume
LC3B	W4	HPI4	0.417834	0.464624	0.146344	Avg. Volume
RAB5	W1	PGE2	0.573103	0.59056	0.099573	Avg. Volume
RAB5	W1	HPI4	0.551033	0.564772	0.136424	Avg. Volume
RAB5	W2	PGE2	0.560797	0.570557	0.082842	Avg. Volume
RAB5	W2	HPI4	0.837121	0.560803	0.785132	Avg. Volume
RAB5	W3	PGE2	0.480571	0.482202	0.059453	Avg. Volume
RAB5	W3	HPI4	0.720985	0.599778	0.525274	Avg. Volume
RAB5	W4	PGE2	0.516187	0.514985	0.102612	Avg. Volume
RAB5	W4	HPI4	0.546166	0.479829	0.244303	Avg. Volume

SLC25A17	W1	PGE2	0.414033	0.420085	0.036642	Avg. Volume
SLC25A17	W1	HPI4	0.407784	0.419535	0.047366	Avg. Volume
SLC25A17	W2	PGE2	0.450263	0.459874	0.051821	Avg. Volume
SLC25A17	W2	HPI4	0.463	0.412677	0.131497	Avg. Volume
SLC25A17	W3	PGE2	0.423998	0.434714	0.047569	Avg. Volume
SLC25A17	W3	HPI4	0.398216	0.374036	0.095567	Avg. Volume
SLC25A17	W4	PGE2	0.417567	0.422653	0.038048	Avg. Volume
SLC25A17	W4	HPI4	0.374223	0.311627	0.207255	Avg. Volume
ACTB	W1	PGE2	0.002333	0.010551	0.071647	Z distribution
ACTB	W1	HPI4	0.034671	0.042294	0.08425	Z distribution
ACTB	W2	PGE2	0.071911	0.080081	0.055381	Z distribution
ACTB	W2	HPI4	-0.02585	-0.03702	0.306863	Z distribution
ACTB	W3	PGE2	0.070705	0.080477	0.127209	Z distribution
ACTB	W3	HPI4	-0.08625	-0.0842	0.088621	Z distribution
ACTB	W4	PGE2	-0.09894	-0.05195	0.150017	Z distribution
ACTB	W4	HPI4	-0.1135	0.011311	0.318321	Z distribution
MYH10	W1	PGE2	-0.15354	-0.13567	0.109165	Z distribution
MYH10	W1	HPI4	-0.15645	-0.13214	0.113376	Z distribution
MYH10	W2	PGE2	0.25471	0.257161	0.0805	Z distribution
MYH10	W2	HPI4	0.060774	0.051954	0.092892	Z distribution
MYH10	W3	PGE2	0.014896	0.009792	0.162185	Z distribution
MYH10	W3	HPI4	0.128339	0.126206	0.067209	Z distribution
MYH10	W4	PGE2	-0.09068	-0.0725	0.126746	Z distribution
MYH10	W4	HPI4	0.128737	0.124864	0.08755	Z distribution
FBL	W1	PGE2	-0.44908	-0.45788	0.050531	Z distribution
FBL	W1	HPI4	-0.46493	-0.46225	0.044405	Z distribution
FBL	W2	PGE2	-0.44495	-0.42313	0.078531	Z distribution
FBL	W2	HPI4	-0.2042	-0.17585	0.103965	Z distribution
FBL	W3	PGE2	-0.3834	-0.38929	0.052131	Z distribution
FBL	W3	HPI4	-0.21045	-0.20069	0.09534	Z distribution
FBL	W4	PGE2	-0.4466	-0.45675	0.153142	Z distribution
FBL	W4	HPI4	-0.3101	-0.28732	0.108525	Z distribution
DSP	W1	PGE2	0.549585	0.505339	0.282926	Z distribution
DSP	W1	HPI4	0.569849	0.538081	0.201661	Z distribution
DSP	W2	PGE2	1.258493	1.289161	0.30926	Z distribution
DSP	W2	HPI4	0.074151	0.008122	0.33497	Z distribution
DSP	W3	PGE2	0.562705	0.538635	0.508644	Z distribution
DSP	W3	HPI4	-0.02792	-0.00585	0.13925	Z distribution
DSP	W4	PGE2	0.057681	-0.03502	0.439236	Z distribution
DSP	W4	HPI4	-0.06974	-0.0563	0.227127	Z distribution
GJA1	W1	PGE2	-0.38421	-0.34846	0.222045	Z distribution
GJA1	W1	HPI4	-0.43705	-0.418	0.27406	Z distribution
GJA1	W2	PGE2	-0.01528	-0.03591	0.132482	Z distribution
GJA1	W2	HPI4	-0.35599	-0.34026	0.199647	Z distribution
GJA1	W3	PGE2	-0.12067	-0.05012	0.300234	Z distribution
GJA1	W3	HPI4	-0.23836	-0.18817	0.223852	Z distribution
GJA1	W4	PGE2	-0.09677	-0.1312	0.191622	Z distribution

GJA1	W4	HPI4	-0.08172	-0.0481	0.128599	Z distribution
TJP1	W1	PGE2	-0.05602	-0.07174	0.114496	Z distribution
TJP1	W1	HPI4	-0.04114	-0.03376	0.123749	Z distribution
TJP1	W2	PGE2	0.354251	0.377269	0.24981	Z distribution
TJP1	W2	HPI4	-0.07054	-0.05882	0.09263	Z distribution
TJP1	W3	PGE2	0.927932	0.90597	0.265617	Z distribution
TJP1	W3	HPI4	-0.09765	-0.10095	0.075318	Z distribution
TJP1	W4	PGE2	0.9519	0.969002	0.311131	Z distribution
TJP1	W4	HPI4	0.028209	0.011401	0.128634	Z distribution
CTNNB1	W1	PGE2	-0.63854	-0.6689	0.142516	Z distribution
CTNNB1	W1	HPI4	-0.68065	-0.689	0.118296	Z distribution
CTNNB1	W2	PGE2	-0.47742	-0.48768	0.154646	Z distribution
CTNNB1	W2	HPI4	-0.25863	-0.13854	0.380323	Z distribution
CTNNB1	W3	PGE2	-0.4253	-0.40115	0.133781	Z distribution
CTNNB1	W3	HPI4	-0.22485	-0.21772	0.191594	Z distribution
CTNNB1	W4	PGE2	-0.33386	-0.29833	0.099838	Z distribution
CTNNB1	W4	HPI4	-0.33565	-0.27694	0.158229	Z distribution
CETN2	W1	PGE2	0.189742	0.161574	0.382012	Z distribution
CETN2	W1	HPI4	0.300507	0.190814	0.498935	Z distribution
CETN2	W2	PGE2	1.660531	1.633432	0.258869	Z distribution
CETN2	W2	HPI4	0.382778	0.431581	0.376923	Z distribution
CETN2	W3	PGE2	-0.05065	-0.10725	0.304101	Z distribution
CETN2	W3	HPI4	-0.27104	-0.20168	0.23754	Z distribution
CETN2	W4	PGE2	0.364738	0.182473	0.438683	Z distribution
CETN2	W4	HPI4	-0.89917	-0.78051	0.351101	Z distribution
SEC61B	W1	PGE2	-0.27755	-0.28126	0.060679	Z distribution
SEC61B	W1	HPI4	-0.25719	-0.24862	0.075728	Z distribution
SEC61B	W2	PGE2	-0.16701	-0.16936	0.114916	Z distribution
SEC61B	W2	HPI4	-0.39031	-0.38969	0.135964	Z distribution
SEC61B	W3	PGE2	-0.57907	-0.55999	0.203777	Z distribution
SEC61B	W3	HPI4	-0.57877	-0.56638	0.241061	Z distribution
SEC61B	W4	PGE2	-0.5356	-0.49364	0.167791	Z distribution
SEC61B	W4	HPI4	-0.51733	-0.53284	0.139926	Z distribution
ST6GAL1	W1	PGE2	-0.49656	-0.45994	0.23066	Z distribution
ST6GAL1	W1	HPI4	-0.35763	-0.36363	0.322819	Z distribution
ST6GAL1	W2	PGE2	-0.15298	-0.1864	0.292805	Z distribution
ST6GAL1	W2	HPI4	-0.47604	-0.43636	0.179348	Z distribution
ST6GAL1	W3	PGE2	-0.17409	-0.12284	0.382819	Z distribution
ST6GAL1	W3	HPI4	-0.48312	-0.45587	0.365579	Z distribution
ST6GAL1	W4	PGE2	-0.22891	-0.17752	0.412919	Z distribution
ST6GAL1	W4	HPI4	-0.66529	-0.72638	0.287806	Z distribution
TOM20	W1	PGE2	-0.45828	-0.45161	0.060977	Z distribution
TOM20	W1	HPI4	-0.49231	-0.48376	0.059451	Z distribution
TOM20	W2	PGE2	-0.62262	-0.62487	0.111237	Z distribution
TOM20	W2	HPI4	-0.63884	-0.64875	0.10707	Z distribution
TOM20	W3	PGE2	-0.63987	-0.63023	0.113684	Z distribution
TOM20	W3	HPI4	-0.84505	-0.87929	0.157785	Z distribution

TOM20	W4	PGE2	-0.57366	-0.58224	0.133682	Z distribution
TOM20	W4	HPI4	-0.80745	-0.81654	0.205356	Z distribution
LAMP1	W1	PGE2	0.233997	0.225429	0.126273	Z distribution
LAMP1	W1	HPI4	0.205223	0.190746	0.128954	Z distribution
LAMP1	W2	PGE2	0.344092	0.349528	0.144763	Z distribution
LAMP1	W2	HPI4	-0.28635	-0.28903	0.093715	Z distribution
LAMP1	W3	PGE2	0.076347	0.08599	0.161622	Z distribution
LAMP1	W3	HPI4	-0.53724	-0.53041	0.16437	Z distribution
LAMP1	W4	PGE2	0.02099	0.018418	0.234206	Z distribution
LAMP1	W4	HPI4	-0.50963	-0.49534	0.289022	Z distribution
LC3B	W1	PGE2	-0.1928	-0.19985	0.132877	Z distribution
LC3B	W1	HPI4	-0.12183	-0.12764	0.130098	Z distribution
LC3B	W2	PGE2	-0.26204	-0.26038	0.106346	Z distribution
LC3B	W2	HPI4	-0.21961	-0.22759	0.183254	Z distribution
LC3B	W3	PGE2	-0.43467	-0.39772	0.128423	Z distribution
LC3B	W3	HPI4	-0.32975	-0.30625	0.204771	Z distribution
LC3B	W4	PGE2	-0.24195	-0.23195	0.150213	Z distribution
LC3B	W4	HPI4	-0.18653	-0.29412	0.320868	Z distribution
RAB5	W1	PGE2	-0.13488	-0.1271	0.128825	Z distribution
RAB5	W1	HPI4	-0.12707	-0.15225	0.155053	Z distribution
RAB5	W2	PGE2	-0.25083	-0.242	0.090862	Z distribution
RAB5	W2	HPI4	-0.36768	-0.32883	0.220094	Z distribution
RAB5	W3	PGE2	-0.47251	-0.48611	0.096584	Z distribution
RAB5	W3	HPI4	-0.29324	-0.25589	0.213357	Z distribution
RAB5	W4	PGE2	-0.57847	-0.60571	0.207854	Z distribution
RAB5	W4	HPI4	-0.24239	-0.23529	0.137132	Z distribution
SLC25A17	W1	PGE2	-0.27645	-0.26771	0.101331	Z distribution
SLC25A17	W1	HPI4	-0.27983	-0.27001	0.078605	Z distribution
SLC25A17	W2	PGE2	-0.41501	-0.41186	0.103661	Z distribution
SLC25A17	W2	HPI4	-0.29055	-0.2717	0.168758	Z distribution
SLC25A17	W3	PGE2	-0.44969	-0.43517	0.138067	Z distribution
SLC25A17	W3	HPI4	-0.24312	-0.2238	0.174741	Z distribution
SLC25A17	W4	PGE2	-0.36222	-0.35462	0.086832	Z distribution
SLC25A17	W4	HPI4	-0.34962	-0.31721	0.450703	Z distribution
ACTB	W1	PGE2	87.23362	86.56887	14.05822	Count/cell
ACTB	W1	HPI4	80.06511	78.88889	12.4295	Count/cell
ACTB	W2	PGE2	49.30337	50.25467	9.230974	Count/cell
ACTB	W2	HPI4	70.44414	62	57.58975	Count/cell
ACTB	W3	PGE2	51.99168	50.86075	7.218139	Count/cell
ACTB	W3	HPI4	61.68898	58.02372	24.04927	Count/cell
ACTB	W4	PGE2	48.46465	47.5716	7.10428	Count/cell
ACTB	W4	HPI4	59.19822	61.66667	16.56637	Count/cell
MYH10	W1	PGE2	107.8578	111.6847	17.05805	Count/cell
MYH10	W1	HPI4	108.1583	110.3304	14.57664	Count/cell
MYH10	W2	PGE2	72.29838	72.51908	7.817025	Count/cell
MYH10	W2	HPI4	86.98671	86.176	26.04653	Count/cell
MYH10	W3	PGE2	75.23054	74.65337	6.728169	Count/cell

MYH10	W3	HPI4	105.1951	103.8493	20.81065	Count/cell
MYH10	W4	PGE2	75.40474	75.03264	10.10306	Count/cell
MYH10	W4	HPI4	118.8849	112.5983	40.34169	Count/cell
FBL	W1	PGE2	3.328321	3.319609	0.151966	Count/cell
FBL	W1	HPI4	3.287919	3.265517	0.19514	Count/cell
FBL	W2	PGE2	2.83646	2.809712	0.163528	Count/cell
FBL	W2	HPI4	2.132831	2.133019	0.199569	Count/cell
FBL	W3	PGE2	2.594694	2.627847	0.119541	Count/cell
FBL	W3	HPI4	2.25038	2.233459	0.32354	Count/cell
FBL	W4	PGE2	2.053592	2.017857	0.195164	Count/cell
FBL	W4	HPI4	1.921168	1.950617	0.222177	Count/cell
DSP	W1	PGE2	22.4279	22.16986	4.306592	Count/cell
DSP	W1	HPI4	22.96419	22.19681	4.13484	Count/cell
DSP	W2	PGE2	38.18739	36.4845	19.04854	Count/cell
DSP	W2	HPI4	25.81432	24.84809	8.809618	Count/cell
DSP	W3	PGE2	48.76091	46.54559	17.75294	Count/cell
DSP	W3	HPI4	26.92075	26.86005	12.04335	Count/cell
DSP	W4	PGE2	64.4891	66.2561	19.40443	Count/cell
DSP	W4	HPI4	26.78821	26.79248	8.47539	Count/cell
GJA1	W1	PGE2	35.02399	27	26.59997	Count/cell
GJA1	W1	HPI4	25.48729	23.6	19.78163	Count/cell
GJA1	W2	PGE2	17.85682	16.47123	6.153537	Count/cell
GJA1	W2	HPI4	21.07016	22.90244	6.55076	Count/cell
GJA1	W3	PGE2	15.57391	12.76003	9.399503	Count/cell
GJA1	W3	HPI4	23.63975	21.66667	15.29987	Count/cell
GJA1	W4	PGE2	13.82282	10.66856	9.319185	Count/cell
GJA1	W4	HPI4	29.27808	27.69231	10.88306	Count/cell
TJP1	W1	PGE2	25.12116	24.39857	2.571314	Count/cell
TJP1	W1	HPI4	26.07301	25.58716	2.579621	Count/cell
TJP1	W2	PGE2	20.00504	19.95988	2.681085	Count/cell
TJP1	W2	HPI4	23.64381	23.38156	2.614834	Count/cell
TJP1	W3	PGE2	15.39179	15.29952	2.172855	Count/cell
TJP1	W3	HPI4	21.69841	21.7694	2.678675	Count/cell
TJP1	W4	PGE2	14.12039	13.72738	1.826884	Count/cell
TJP1	W4	HPI4	23.41669	23.41759	2.770092	Count/cell
CTNNB1	W1	PGE2	31.23372	30.66318	4.489316	Count/cell
CTNNB1	W1	HPI4	32.77639	30.96461	5.571062	Count/cell
CTNNB1	W2	PGE2	19.66947	19.56006	5.16662	Count/cell
CTNNB1	W2	HPI4	22.27003	20.5	9.869678	Count/cell
CTNNB1	W3	PGE2	36.18025	35.68531	6.594703	Count/cell
CTNNB1	W3	HPI4	22.76287	19.61538	11.3811	Count/cell
CTNNB1	W4	PGE2	37.81126	37.76974	6.303202	Count/cell
CTNNB1	W4	HPI4	62.36833	55.5	29.5605	Count/cell
CETN2	W1	PGE2	1.807603	1.797101	0.166936	Count/cell
CETN2	W1	HPI4	1.744743	1.748265	0.273297	Count/cell
CETN2	W2	PGE2	1.884705	1.869191	0.136725	Count/cell
CETN2	W2	HPI4	2.009673	2.018717	0.191751	Count/cell

CETN2	W3	PGE2	2.041925	2.05719	0.270289	Count/cell
CETN2	W3	HPI4	1.657942	1.333333	0.918382	Count/cell
CETN2	W4	PGE2	1.812202	1.783198	0.239868	Count/cell
CETN2	W4	HPI4	1.55	1	1.051529	Count/cell
SEC61B	W1	PGE2	88.85381	88.42036	12.73156	Count/cell
SEC61B	W1	HPI4	89.77024	90.77002	11.94917	Count/cell
SEC61B	W2	PGE2	40.94056	40.4002	3.90342	Count/cell
SEC61B	W2	HPI4	76.55362	77.67026	11.05403	Count/cell
SEC61B	W3	PGE2	39.14335	39.01614	4.511668	Count/cell
SEC61B	W3	HPI4	87.28651	85.50027	12.31061	Count/cell
SEC61B	W4	PGE2	35.32954	34.20253	4.426611	Count/cell
SEC61B	W4	HPI4	85.20879	86.26874	17.23908	Count/cell
ST6GAL1	W1	PGE2	7.651994	6.8	4.633276	Count/cell
ST6GAL1	W1	HPI4	7.130191	6.52197	2.576658	Count/cell
ST6GAL1	W2	PGE2	5.760102	5.373271	1.569807	Count/cell
ST6GAL1	W2	HPI4	11.19277	11.90909	4.946318	Count/cell
ST6GAL1	W3	PGE2	6.590594	6.02737	2.525899	Count/cell
ST6GAL1	W3	HPI4	8.633952	8.536232	4.942221	Count/cell
ST6GAL1	W4	PGE2	5.609627	4.926732	2.406982	Count/cell
ST6GAL1	W4	HPI4	6.583755	5.904762	4.054568	Count/cell
TOM20	W1	PGE2	32.74479	32.73789	2.125116	Count/cell
TOM20	W1	HPI4	30.41775	30.37972	3.33696	Count/cell
TOM20	W2	PGE2	20.59847	20.92666	1.680753	Count/cell
TOM20	W2	HPI4	26.69942	27.37056	3.571065	Count/cell
TOM20	W3	PGE2	17.86991	17.81696	1.768584	Count/cell
TOM20	W3	HPI4	28.14307	28.16973	2.842487	Count/cell
TOM20	W4	PGE2	16.75126	16.93805	2.067822	Count/cell
TOM20	W4	HPI4	36.23014	36.01703	3.089113	Count/cell
LAMP1	W1	PGE2	30.83033	30.39027	4.284323	Count/cell
LAMP1	W1	HPI4	27.45415	27.77545	3.869381	Count/cell
LAMP1	W2	PGE2	21.55159	21.50674	3.540155	Count/cell
LAMP1	W2	HPI4	31.58389	30.05895	14.45797	Count/cell
LAMP1	W3	PGE2	18.16013	18.4093	3.514018	Count/cell
LAMP1	W3	HPI4	34.83054	30.84806	17.7006	Count/cell
LAMP1	W4	PGE2	18.2536	19.19027	3.829334	Count/cell
LAMP1	W4	HPI4	34.92709	28.97793	21.39582	Count/cell
LC3B	W1	PGE2	5.367347	5.194995	1.01324	Count/cell
LC3B	W1	HPI4	5.221037	4.761529	1.252366	Count/cell
LC3B	W2	PGE2	12.18473	12.93609	4.064754	Count/cell
LC3B	W2	HPI4	12.46629	11.79167	5.539167	Count/cell
LC3B	W3	PGE2	13.2353	13.50359	3.365832	Count/cell
LC3B	W3	HPI4	13.23853	10.25	8.730159	Count/cell
LC3B	W4	PGE2	9.412286	8.649202	2.538833	Count/cell
LC3B	W4	HPI4	31.4	18	36.02166	Count/cell
RAB5	W1	PGE2	34.65063	35.41157	8.276418	Count/cell
RAB5	W1	HPI4	32.81838	35.3284	10.86322	Count/cell
RAB5	W2	PGE2	25.75901	26.45047	6.008182	Count/cell

RAB5	W2	HPI4	20.10093	19	11.35283	Count/cell
RAB5	W3	PGE2	34.34977	34.2662	11.36197	Count/cell
RAB5	W3	HPI4	12.69899	8.666667	15.81981	Count/cell
RAB5	W4	PGE2	22.64071	22.2309	7.843066	Count/cell
RAB5	W4	HPI4	29.8052	26.97727	13.86868	Count/cell
SLC25A17	W1	PGE2	65.56802	66.84302	7.410307	Count/cell
SLC25A17	W1	HPI4	70.78701	71.50346	8.270478	Count/cell
SLC25A17	W2	PGE2	66.84068	67.3736	8.795055	Count/cell
SLC25A17	W2	HPI4	44.91907	43.42118	12.35468	Count/cell
SLC25A17	W3	PGE2	67.6567	67.39285	8.904761	Count/cell
SLC25A17	W3	HPI4	56.28552	52.08333	22.80802	Count/cell
SLC25A17	W4	PGE2	90.76213	91.60691	16.47659	Count/cell
SLC25A17	W4	HPI4	53.0461	39	35.46659	Count/cell
SEC61B	W1	PGE2	171.4417	172.0137	45.17494	Total volume
SEC61B	W1	HPI4	163.8206	164.6681	34.449	Total volume
SEC61B	W2	PGE2	85.96147	86.66584	19.2981	Total volume
SEC61B	W2	HPI4	102.5832	103.5944	25.6545	Total volume
SEC61B	W3	PGE2	97.59546	98.70536	21.65321	Total volume
SEC61B	W3	HPI4	119.4401	112.6265	37.98787	Total volume
SEC61B	W4	PGE2	82.03102	84.01057	20.9532	Total volume
SEC61B	W4	HPI4	99.42329	96.66341	37.33582	Total volume
TOM20	W1	PGE2	115.4925	116.8629	16.45209	Total volume
TOM20	W1	HPI4	106.1988	104.8505	19.8118	Total volume
TOM20	W2	PGE2	80.90515	78.10982	14.28264	Total volume
TOM20	W2	HPI4	70.95261	70.0411	17.64267	Total volume
TOM20	W3	PGE2	80.25414	79.63676	9.872483	Total volume
TOM20	W3	HPI4	67.79997	62.88541	18.71027	Total volume
TOM20	W4	PGE2	61.98744	59.94874	11.70895	Total volume
TOM20	W4	HPI4	108.7245	108.3384	23.59795	Total volume
ST6GAL1	W1	PGE2	9.043688	6.719457	10.75382	Total volume
ST6GAL1	W1	HPI4	10.32422	10.49898	4.250052	Total volume
ST6GAL1	W2	PGE2	7.543003	7.823259	1.587777	Total volume
ST6GAL1	W2	HPI4	11.12083	11.42315	5.711846	Total volume
ST6GAL1	W3	PGE2	6.62963	5.765025	3.083651	Total volume
ST6GAL1	W3	HPI4	8.06619	8.48912	6.903117	Total volume
ST6GAL1	W4	PGE2	3.757352	3.304835	2.090295	Total volume
ST6GAL1	W4	HPI4	5.363109	3.443245	4.867119	Total volume

Table S12

Line	Week	Treatment	Well	FOV	N	Metric	mean	median	sd
ACTB	W1	HPI4	well_1	F001	11	Avg. Volume	1.179882	1.055598	0.377163
ACTB	W1	HPI4	well_1	F002	37	Avg. Volume	1.238346	1.137785	0.406375
ACTB	W1	HPI4	well_1	F003	50	Avg. Volume	1.035052	0.974515	0.414034
ACTB	W1	HPI4	well_1	F004	18	Avg. Volume	1.151401	1.043772	0.293295
ACTB	W1	HPI4	well_1	F005	14	Avg. Volume	1.6934	1.652335	0.763822
ACTB	W1	HPI4	well_1	F006	39	Avg. Volume	1.087573	0.952972	0.498557
ACTB	W1	HPI4	well_2	F001	100	Avg. Volume	1.477983	1.369848	0.575525
ACTB	W1	HPI4	well_2	F002	60	Avg. Volume	1.124462	1.083457	0.431082
ACTB	W1	HPI4	well_2	F003	68	Avg. Volume	1.173658	1.140203	0.427458
ACTB	W1	HPI4	well_2	F004	33	Avg. Volume	1.379085	1.271591	0.467166
ACTB	W1	HPI4	well_2	F005	19	Avg. Volume	1.099781	1.087485	0.364999
ACTB	W1	HPI4	well_2	F006	46	Avg. Volume	1.426192	1.337084	0.467436
ACTB	W1	HPI4	well_3	F001	137	Avg. Volume	0.959917	0.865384	0.411388
ACTB	W1	HPI4	well_3	F002	23	Avg. Volume	1.310345	1.17223	0.43036
ACTB	W1	HPI4	well_3	F003	21	Avg. Volume	1.150047	1.214967	0.392116
ACTB	W1	HPI4	well_3	F004	59	Avg. Volume	1.117436	1.03338	0.3476
ACTB	W1	HPI4	well_3	F005	58	Avg. Volume	0.99112	0.87324	0.417163
ACTB	W1	HPI4	well_3	F006	67	Avg. Volume	1.544919	1.566462	0.424512
ACTB	W1	HPI4	well_4	F001	46	Avg. Volume	0.986325	0.888463	0.362765
ACTB	W1	HPI4	well_4	F002	95	Avg. Volume	1.512402	1.418765	0.55735
ACTB	W1	HPI4	well_4	F003	51	Avg. Volume	1.151647	1.056749	0.4462
ACTB	W1	HPI4	well_4	F004	94	Avg. Volume	1.060209	0.96537	0.366488
ACTB	W1	HPI4	well_4	F005	34	Avg. Volume	1.218665	1.19642	0.418205
ACTB	W1	HPI4	well_4	F006	56	Avg. Volume	1.026103	0.997379	0.320478
ACTB	W1	HPI4	well_5	F001	85	Avg. Volume	1.196587	1.141713	0.401728
ACTB	W1	HPI4	well_5	F002	62	Avg. Volume	1.121125	1.106037	0.325475
ACTB	W1	HPI4	well_5	F003	75	Avg. Volume	1.057894	1.071515	0.368244
ACTB	W1	HPI4	well_5	F004	61	Avg. Volume	1.151901	1.147531	0.363011
ACTB	W1	HPI4	well_5	F005	130	Avg. Volume	1.270311	1.220462	0.41525
ACTB	W1	HPI4	well_5	F006	96	Avg. Volume	1.108591	1.107469	0.439033
ACTB	W1	PGE2	well_1	F001	35	Avg. Volume	1.308477	1.210136	0.385398
ACTB	W1	PGE2	well_1	F002	17	Avg. Volume	1.265108	1.238009	0.531562
ACTB	W1	PGE2	well_1	F003	46	Avg. Volume	1.439059	1.311225	0.38872
ACTB	W1	PGE2	well_1	F004	84	Avg. Volume	1.194886	1.181243	0.41788
ACTB	W1	PGE2	well_1	F005	130	Avg. Volume	1.035223	0.936715	0.381923
ACTB	W1	PGE2	well_1	F006	74	Avg. Volume	1.35604	1.23776	0.425717
ACTB	W1	PGE2	well_2	F001	37	Avg. Volume	1.276121	1.310177	0.395236
ACTB	W1	PGE2	well_2	F002	18	Avg. Volume	1.006234	0.924918	0.314039
ACTB	W1	PGE2	well_2	F003	39	Avg. Volume	1.232238	1.103567	0.595058
ACTB	W1	PGE2	well_2	F004	46	Avg. Volume	0.99391	0.938907	0.291513
ACTB	W1	PGE2	well_2	F005	43	Avg. Volume	1.215436	1.149527	0.381989
ACTB	W1	PGE2	well_2	F006	48	Avg. Volume	1.269269	1.152432	0.401369
ACTB	W1	PGE2	well_3	F001	59	Avg. Volume	1.105218	1.098429	0.369337
ACTB	W1	PGE2	well_3	F002	79	Avg. Volume	0.858219	0.821314	0.327442
ACTB	W1	PGE2	well_3	F003	31	Avg. Volume	0.943247	0.957914	0.307275
ACTB	W1	PGE2	well_3	F004	29	Avg. Volume	1.038707	0.991276	0.28978

ACTB	W1	PGE2	well_3	F005	74	Avg. Volume	1.123094	1.106877	0.37939
ACTB	W1	PGE2	well_3	F006	90	Avg. Volume	1.217545	1.188805	0.409736
ACTB	W1	PGE2	well_4	F001	31	Avg. Volume	1.23184	1.170209	0.329339
ACTB	W1	PGE2	well_4	F002	28	Avg. Volume	1.143687	1.146247	0.452783
ACTB	W1	PGE2	well_4	F003	58	Avg. Volume	1.095548	1.016985	0.457874
ACTB	W1	PGE2	well_4	F004	16	Avg. Volume	1.022192	0.987415	0.397253
ACTB	W1	PGE2	well_4	F005	19	Avg. Volume	1.126049	1.166066	0.356469
ACTB	W1	PGE2	well_4	F006	9	Avg. Volume	1.398466	1.443385	0.28034
ACTB	W1	PGE2	well_5	F001	26	Avg. Volume	0.971932	0.865528	0.314493
ACTB	W1	PGE2	well_5	F002	31	Avg. Volume	0.98468	1.008854	0.361201
ACTB	W1	PGE2	well_5	F003	11	Avg. Volume	0.780034	0.710773	0.213504
ACTB	W1	PGE2	well_5	F004	30	Avg. Volume	1.184197	1.113989	0.378012
ACTB	W1	PGE2	well_5	F005	22	Avg. Volume	1.185117	1.220999	0.317521
ACTB	W1	PGE2	well_5	F006	60	Avg. Volume	1.317957	1.274661	0.366213
ACTB	W2	HPI4	well_1	F001	1	Avg. Volume	4.961022	4.961022	NA
ACTB	W2	HPI4	well_1	F002	1	Avg. Volume	0.711579	0.711579	NA
ACTB	W2	HPI4	well_1	F005	3	Avg. Volume	1.419635	1.419635	NA
ACTB	W2	HPI4	well_1	F006	3	Avg. Volume	0.890394	0.890394	NA
ACTB	W2	HPI4	well_2	F006	3	Avg. Volume	2.223	2.223	NA
ACTB	W2	HPI4	well_3	F001	5	Avg. Volume	0.706582	0.514751	0.408283
ACTB	W2	HPI4	well_3	F002	1	Avg. Volume	6.704449	6.704449	NA
ACTB	W2	HPI4	well_3	F003	1	Avg. Volume	0.385104	0.385104	NA
ACTB	W2	HPI4	well_3	F006	1	Avg. Volume	0.675383	0.675383	NA
ACTB	W2	HPI4	well_4	F002	1	Avg. Volume	0.058681	0.058681	NA
ACTB	W2	HPI4	well_4	F006	3	Avg. Volume	0.871863	0.871863	NA
ACTB	W2	HPI4	well_5	F001	14	Avg. Volume	0.599003	0.51151	0.280856
ACTB	W2	HPI4	well_5	F003	1	Avg. Volume	0.616741	0.616741	NA
ACTB	W2	HPI4	well_5	F004	3	Avg. Volume	0.632546	0.632546	NA
ACTB	W2	HPI4	well_5	F006	16	Avg. Volume	1.715295	1.183803	1.460833
ACTB	W2	PGE2	well_1	F001	126	Avg. Volume	1.449504	1.367818	0.428232
ACTB	W2	PGE2	well_1	F002	139	Avg. Volume	1.479263	1.396597	0.477205
ACTB	W2	PGE2	well_1	F003	180	Avg. Volume	1.687633	1.574573	0.556668
ACTB	W2	PGE2	well_1	F004	97	Avg. Volume	1.235466	1.010244	0.702562
ACTB	W2	PGE2	well_1	F005	101	Avg. Volume	1.740463	1.628033	0.732782
ACTB	W2	PGE2	well_1	F006	72	Avg. Volume	1.326601	0.904486	0.939874
ACTB	W2	PGE2	well_2	F001	59	Avg. Volume	1.975152	1.888731	0.666889
ACTB	W2	PGE2	well_2	F002	91	Avg. Volume	1.500276	1.411486	0.542026
ACTB	W2	PGE2	well_2	F003	142	Avg. Volume	2.046905	2.008975	0.690067
ACTB	W2	PGE2	well_2	F004	60	Avg. Volume	1.825775	1.604163	0.754802
ACTB	W2	PGE2	well_2	F005	36	Avg. Volume	1.895418	1.856024	0.796326
ACTB	W2	PGE2	well_2	F006	123	Avg. Volume	1.874139	1.867945	0.581703
ACTB	W2	PGE2	well_3	F001	67	Avg. Volume	1.233862	1.134761	0.526002
ACTB	W2	PGE2	well_3	F002	90	Avg. Volume	1.543508	1.514127	0.57883
ACTB	W2	PGE2	well_3	F003	99	Avg. Volume	2.232171	2.089587	0.825255
ACTB	W2	PGE2	well_3	F004	20	Avg. Volume	1.80011	1.861557	0.388634
ACTB	W2	PGE2	well_3	F005	83	Avg. Volume	1.214383	1.14075	0.459691
ACTB	W2	PGE2	well_3	F006	102	Avg. Volume	1.830078	1.78707	0.587666
ACTB	W2	PGE2	well_4	F001	36	Avg. Volume	1.528109	1.468892	0.595433
ACTB	W2	PGE2	well_4	F002	38	Avg. Volume	1.83491	1.582365	0.902849

ACTB	W2	PGE2	well_4	F003	38	Avg. Volume	1.73767	1.445626	0.948458
ACTB	W2	PGE2	well_4	F004	53	Avg. Volume	1.478443	1.241319	0.577499
ACTB	W2	PGE2	well_4	F005	67	Avg. Volume	1.820414	1.533376	0.894802
ACTB	W2	PGE2	well_4	F006	56	Avg. Volume	1.781551	1.522219	0.753512
ACTB	W2	PGE2	well_5	F001	15	Avg. Volume	1.63481	1.449913	1.069379
ACTB	W2	PGE2	well_5	F002	21	Avg. Volume	1.152497	0.929006	0.601389
ACTB	W2	PGE2	well_5	F003	20	Avg. Volume	1.921065	2.071243	0.639847
ACTB	W2	PGE2	well_5	F004	36	Avg. Volume	1.206817	1.054117	0.690997
ACTB	W2	PGE2	well_5	F005	29	Avg. Volume	1.308268	1.273759	0.25267
ACTB	W2	PGE2	well_5	F006	35	Avg. Volume	1.522496	1.360503	0.550429
ACTB	W3	HPI4	well_1	F001	43	Avg. Volume	0.910881	0.833264	0.453625
ACTB	W3	HPI4	well_1	F002	36	Avg. Volume	1.05058	0.996146	0.395826
ACTB	W3	HPI4	well_1	F003	78	Avg. Volume	1.202151	1.14313	0.455917
ACTB	W3	HPI4	well_1	F004	122	Avg. Volume	1.607305	1.445593	0.557085
ACTB	W3	HPI4	well_1	F005	68	Avg. Volume	1.528361	1.347293	0.645884
ACTB	W3	HPI4	well_1	F006	59	Avg. Volume	0.906802	0.873688	0.337252
ACTB	W3	HPI4	well_2	F001	86	Avg. Volume	1.026355	0.887204	0.426193
ACTB	W3	HPI4	well_2	F002	58	Avg. Volume	1.115877	1.086987	0.34966
ACTB	W3	HPI4	well_2	F003	52	Avg. Volume	1.277711	1.29006	0.382571
ACTB	W3	HPI4	well_2	F004	55	Avg. Volume	1.007811	0.970744	0.310726
ACTB	W3	HPI4	well_2	F005	111	Avg. Volume	1.266376	1.204821	0.480394
ACTB	W3	HPI4	well_2	F006	95	Avg. Volume	1.223148	1.12456	0.411724
ACTB	W3	HPI4	well_3	F001	32	Avg. Volume	1.098819	0.940297	0.400541
ACTB	W3	HPI4	well_3	F002	37	Avg. Volume	1.09044	1.118647	0.37163
ACTB	W3	HPI4	well_3	F003	6	Avg. Volume	0.575597	0.550094	0.140671
ACTB	W3	HPI4	well_3	F004	37	Avg. Volume	1.193784	1.047122	0.59934
ACTB	W3	HPI4	well_3	F005	12	Avg. Volume	1.029756	0.979765	0.403466
ACTB	W3	HPI4	well_3	F006	59	Avg. Volume	1.183249	1.082894	0.405627
ACTB	W3	HPI4	well_4	F001	103	Avg. Volume	1.046535	1.04158	0.378731
ACTB	W3	HPI4	well_4	F002	91	Avg. Volume	1.416499	1.382849	0.514683
ACTB	W3	HPI4	well_4	F003	21	Avg. Volume	0.955692	0.819457	0.487818
ACTB	W3	HPI4	well_4	F004	9	Avg. Volume	1.024809	0.897565	0.311932
ACTB	W3	HPI4	well_4	F005	58	Avg. Volume	1.201155	1.17118	0.41746
ACTB	W3	HPI4	well_4	F006	70	Avg. Volume	1.088192	1.018108	0.391644
ACTB	W3	HPI4	well_5	F001	20	Avg. Volume	0.925861	0.868157	0.335024
ACTB	W3	HPI4	well_5	F002	8	Avg. Volume	0.837167	0.826022	0.17939
ACTB	W3	HPI4	well_5	F003	4	Avg. Volume	0.639889	0.639889	0.237175
ACTB	W3	HPI4	well_5	F004	5	Avg. Volume	1.079934	1.100799	0.251318
ACTB	W3	HPI4	well_5	F005	26	Avg. Volume	0.804114	0.675768	0.322205
ACTB	W3	HPI4	well_5	F006	18	Avg. Volume	0.821596	0.699955	0.359695
ACTB	W3	PGE2	well_1	F001	162	Avg. Volume	1.346349	1.27818	0.503314
ACTB	W3	PGE2	well_1	F002	232	Avg. Volume	1.376874	1.236943	0.627343
ACTB	W3	PGE2	well_1	F003	294	Avg. Volume	1.356682	1.315314	0.467813
ACTB	W3	PGE2	well_1	F004	58	Avg. Volume	1.451941	1.338516	0.453666
ACTB	W3	PGE2	well_1	F005	80	Avg. Volume	1.387987	1.258411	0.519077
ACTB	W3	PGE2	well_1	F006	281	Avg. Volume	1.144145	1.090841	0.429607
ACTB	W3	PGE2	well_2	F001	113	Avg. Volume	1.399718	1.277063	0.57361
ACTB	W3	PGE2	well_2	F002	160	Avg. Volume	1.198481	1.100464	0.515644
ACTB	W3	PGE2	well_2	F003	190	Avg. Volume	1.482312	1.424417	0.540141

ACTB	W3	PGE2	well_2	F004	155	Avg. Volume	1.936892	1.875737	0.605615
ACTB	W3	PGE2	well_2	F005	288	Avg. Volume	1.825504	1.653536	0.755996
ACTB	W3	PGE2	well_2	F006	191	Avg. Volume	1.864029	1.800598	0.65822
ACTB	W3	PGE2	well_3	F001	110	Avg. Volume	1.542443	1.442829	0.641938
ACTB	W3	PGE2	well_3	F002	211	Avg. Volume	1.312649	1.224112	0.459547
ACTB	W3	PGE2	well_3	F003	201	Avg. Volume	1.555375	1.413734	0.654956
ACTB	W3	PGE2	well_3	F004	83	Avg. Volume	1.69289	1.702081	0.634432
ACTB	W3	PGE2	well_3	F005	217	Avg. Volume	1.396829	1.323434	0.534663
ACTB	W3	PGE2	well_3	F006	227	Avg. Volume	1.613459	1.525373	0.62338
ACTB	W3	PGE2	well_4	F001	176	Avg. Volume	1.66063	1.540816	0.603344
ACTB	W3	PGE2	well_4	F002	159	Avg. Volume	1.387197	1.298357	0.472941
ACTB	W3	PGE2	well_4	F003	251	Avg. Volume	1.792037	1.606543	0.719565
ACTB	W3	PGE2	well_4	F004	161	Avg. Volume	1.207247	1.093135	0.545565
ACTB	W3	PGE2	well_4	F005	196	Avg. Volume	1.204347	1.110558	0.440654
ACTB	W3	PGE2	well_4	F006	196	Avg. Volume	1.477679	1.366512	0.543174
ACTB	W3	PGE2	well_5	F001	105	Avg. Volume	1.506458	1.442453	0.42364
ACTB	W3	PGE2	well_5	F002	132	Avg. Volume	1.384046	1.301209	0.476913
ACTB	W3	PGE2	well_5	F003	223	Avg. Volume	1.365678	1.296495	0.505916
ACTB	W3	PGE2	well_5	F004	102	Avg. Volume	1.683084	1.578891	0.512584
ACTB	W3	PGE2	well_5	F005	222	Avg. Volume	1.219398	1.122318	0.492339
ACTB	W3	PGE2	well_5	F006	204	Avg. Volume	1.69734	1.596372	0.557003
ACTB	W4	HPI4	well_1	F001	36	Avg. Volume	0.956221	0.946571	0.254031
ACTB	W4	HPI4	well_1	F002	4	Avg. Volume	0.741658	0.741658	0.117039
ACTB	W4	HPI4	well_1	F003	12	Avg. Volume	1.00613	1.036717	0.226476
ACTB	W4	HPI4	well_1	F004	23	Avg. Volume	1.639209	1.565597	0.905472
ACTB	W4	HPI4	well_1	F005	39	Avg. Volume	1.361145	1.297374	0.493336
ACTB	W4	HPI4	well_1	F006	27	Avg. Volume	0.805366	0.742261	0.27839
ACTB	W4	HPI4	well_2	F001	25	Avg. Volume	0.711118	0.604317	0.363518
ACTB	W4	HPI4	well_2	F003	4	Avg. Volume	0.886204	0.886204	0.224245
ACTB	W4	HPI4	well_2	F004	10	Avg. Volume	1.746521	1.610522	0.530947
ACTB	W4	HPI4	well_2	F005	38	Avg. Volume	2.389563	2.253278	0.940065
ACTB	W4	HPI4	well_2	F006	13	Avg. Volume	1.207737	1.190377	0.480913
ACTB	W4	HPI4	well_3	F001	8	Avg. Volume	1.186721	1.100981	0.309869
ACTB	W4	HPI4	well_3	F002	3	Avg. Volume	0.513259	0.513259	NA
ACTB	W4	HPI4	well_3	F003	1	Avg. Volume	0.800526	0.800526	NA
ACTB	W4	HPI4	well_3	F004	1	Avg. Volume	0.372133	0.372133	NA
ACTB	W4	HPI4	well_3	F006	5	Avg. Volume	0.735173	0.7249	0.173265
ACTB	W4	HPI4	well_4	F001	14	Avg. Volume	0.929617	0.874528	0.298034
ACTB	W4	HPI4	well_4	F002	23	Avg. Volume	0.684663	0.692232	0.230011
ACTB	W4	HPI4	well_4	F003	3	Avg. Volume	1.019365	1.019365	NA
ACTB	W4	HPI4	well_4	F004	6	Avg. Volume	0.827575	0.834235	0.233835
ACTB	W4	HPI4	well_4	F005	19	Avg. Volume	1.015318	1.116736	0.341417
ACTB	W4	HPI4	well_4	F006	4	Avg. Volume	1.599083	1.599083	0.145173
ACTB	W4	HPI4	well_5	F001	3	Avg. Volume	1.442238	1.442238	NA
ACTB	W4	HPI4	well_5	F002	8	Avg. Volume	1.579352	1.636618	1.055756
ACTB	W4	HPI4	well_5	F003	1	Avg. Volume	0.657583	0.657583	NA
ACTB	W4	HPI4	well_5	F004	5	Avg. Volume	0.495188	0.356497	0.334514
ACTB	W4	HPI4	well_5	F005	3	Avg. Volume	0.869184	0.869184	NA
ACTB	W4	HPI4	well_5	F006	17	Avg. Volume	0.756941	0.698768	0.471205

ACTB	W4	PGE2	well_1	F001	231	Avg. Volume	1.427312	1.347159	0.514837
ACTB	W4	PGE2	well_1	F002	289	Avg. Volume	1.22782	1.168581	0.540928
ACTB	W4	PGE2	well_1	F003	254	Avg. Volume	1.780907	1.595662	0.814668
ACTB	W4	PGE2	well_1	F004	280	Avg. Volume	1.255116	1.161837	0.520217
ACTB	W4	PGE2	well_1	F005	291	Avg. Volume	1.211128	1.120339	0.53029
ACTB	W4	PGE2	well_1	F006	318	Avg. Volume	1.724393	1.637143	0.644834
ACTB	W4	PGE2	well_2	F001	235	Avg. Volume	1.749492	1.594208	0.691644
ACTB	W4	PGE2	well_2	F002	265	Avg. Volume	1.107565	1.028083	0.410569
ACTB	W4	PGE2	well_2	F003	228	Avg. Volume	1.605676	1.515032	0.564155
ACTB	W4	PGE2	well_2	F004	337	Avg. Volume	1.973694	1.840643	0.835296
ACTB	W4	PGE2	well_2	F005	295	Avg. Volume	1.714311	1.568933	0.728675
ACTB	W4	PGE2	well_2	F006	285	Avg. Volume	1.821252	1.723102	0.6301
ACTB	W4	PGE2	well_3	F001	297	Avg. Volume	1.307913	1.222627	0.700264
ACTB	W4	PGE2	well_3	F002	253	Avg. Volume	2.04998	1.95341	0.819807
ACTB	W4	PGE2	well_3	F003	245	Avg. Volume	1.68772	1.563713	0.682825
ACTB	W4	PGE2	well_3	F004	286	Avg. Volume	1.623788	1.501457	0.679076
ACTB	W4	PGE2	well_3	F005	248	Avg. Volume	1.790707	1.653616	0.74877
ACTB	W4	PGE2	well_3	F006	338	Avg. Volume	1.299065	1.178406	0.665413
ACTB	W4	PGE2	well_4	F001	322	Avg. Volume	1.649138	1.483976	0.777275
ACTB	W4	PGE2	well_4	F002	230	Avg. Volume	1.440903	1.255098	0.641178
ACTB	W4	PGE2	well_4	F003	289	Avg. Volume	1.623928	1.536813	0.694677
ACTB	W4	PGE2	well_4	F004	120	Avg. Volume	1.671229	1.567749	0.703735
ACTB	W4	PGE2	well_4	F005	239	Avg. Volume	1.918784	1.785649	0.749564
ACTB	W4	PGE2	well_4	F006	154	Avg. Volume	1.224586	1.160465	0.433951
ACTB	W4	PGE2	well_5	F001	95	Avg. Volume	1.115293	1.112178	0.409554
ACTB	W4	PGE2	well_5	F002	188	Avg. Volume	1.569576	1.503278	0.601886
ACTB	W4	PGE2	well_5	F003	264	Avg. Volume	1.331034	1.248918	0.499905
ACTB	W4	PGE2	well_5	F004	204	Avg. Volume	1.151506	1.078289	0.505981
ACTB	W4	PGE2	well_5	F005	255	Avg. Volume	1.492875	1.410941	0.603793
ACTB	W4	PGE2	well_5	F006	207	Avg. Volume	1.30776	1.166905	0.605402
ACTB	W1	HPI4	well_1	F001	11	Count/cell	79.11111	77	20.60003
ACTB	W1	HPI4	well_1	F002	37	Count/cell	78.66667	71	32.47948
ACTB	W1	HPI4	well_1	F003	50	Count/cell	83.75	75.5	34.46813
ACTB	W1	HPI4	well_1	F004	18	Count/cell	96.0625	93.5	34.67077
ACTB	W1	HPI4	well_1	F005	14	Count/cell	71.66667	67	27.41709
ACTB	W1	HPI4	well_1	F006	39	Count/cell	85.62857	76	36.98337
ACTB	W1	HPI4	well_2	F001	100	Count/cell	67.31111	69	27.86147
ACTB	W1	HPI4	well_2	F002	60	Count/cell	71.31481	68.5	30.81829
ACTB	W1	HPI4	well_2	F003	68	Count/cell	67.66667	60	31.1876
ACTB	W1	HPI4	well_2	F004	33	Count/cell	93.27586	90	40.90922
ACTB	W1	HPI4	well_2	F005	19	Count/cell	97.94118	109	31.02312
ACTB	W1	HPI4	well_2	F006	46	Count/cell	94.275	79.5	36.59689
ACTB	W1	HPI4	well_3	F001	137	Count/cell	70.77236	63	31.10791
ACTB	W1	HPI4	well_3	F002	23	Count/cell	83.31579	78	29.76398
ACTB	W1	HPI4	well_3	F003	21	Count/cell	103.8947	108	42.92357
ACTB	W1	HPI4	well_3	F004	59	Count/cell	83.18868	71	41.61456
ACTB	W1	HPI4	well_3	F005	58	Count/cell	80.13462	77	37.47681
ACTB	W1	HPI4	well_3	F006	67	Count/cell	94.50847	95	35.93259
ACTB	W1	HPI4	well_4	F001	46	Count/cell	68.55	70	27.37859

ACTB	W1	HPI4	well_4	F002	95	Count/cell	77.96512	78	27.42024
ACTB	W1	HPI4	well_4	F003	51	Count/cell	79.46667	71	34.47436
ACTB	W1	HPI4	well_4	F004	94	Count/cell	64.14118	64	24.78006
ACTB	W1	HPI4	well_4	F005	34	Count/cell	103.9	100.5	37.99397
ACTB	W1	HPI4	well_4	F006	56	Count/cell	62.05882	58	25.84292
ACTB	W1	HPI4	well_5	F001	85	Count/cell	73.57333	68	31.49568
ACTB	W1	HPI4	well_5	F002	62	Count/cell	73.94444	71	24.02783
ACTB	W1	HPI4	well_5	F003	75	Count/cell	94.17391	91	43.12975
ACTB	W1	HPI4	well_5	F004	61	Count/cell	70.27273	67	25.929
ACTB	W1	HPI4	well_5	F005	130	Count/cell	71.38793	69.5	25.06367
ACTB	W1	HPI4	well_5	F006	96	Count/cell	60.03448	55	28.68654
ACTB	W1	PGE2	well_1	F001	35	Count/cell	99	105	45.65862
ACTB	W1	PGE2	well_1	F002	17	Count/cell	88.73333	88	34.61723
ACTB	W1	PGE2	well_1	F003	46	Count/cell	87.1	86.5	34.59198
ACTB	W1	PGE2	well_1	F004	84	Count/cell	75.31081	71	36.95385
ACTB	W1	PGE2	well_1	F005	130	Count/cell	69.56897	69	26.56419
ACTB	W1	PGE2	well_1	F006	74	Count/cell	80.48485	77	37.52589
ACTB	W1	PGE2	well_2	F001	37	Count/cell	89.02941	82.5	37.69695
ACTB	W1	PGE2	well_2	F002	18	Count/cell	110.9375	105	43.18868
ACTB	W1	PGE2	well_2	F003	39	Count/cell	76.45714	55	45.96824
ACTB	W1	PGE2	well_2	F004	46	Count/cell	91.975	90.5	38.04012
ACTB	W1	PGE2	well_2	F005	43	Count/cell	92.16216	87	33.36125
ACTB	W1	PGE2	well_2	F006	48	Count/cell	70.02381	69.5	25.16142
ACTB	W1	PGE2	well_3	F001	59	Count/cell	86.03774	82	30.71016
ACTB	W1	PGE2	well_3	F002	79	Count/cell	81.40845	79	30.06402
ACTB	W1	PGE2	well_3	F003	31	Count/cell	95.66667	73	53.93087
ACTB	W1	PGE2	well_3	F004	29	Count/cell	83	81	32.50897
ACTB	W1	PGE2	well_3	F005	74	Count/cell	86.01515	84	40.25744
ACTB	W1	PGE2	well_3	F006	90	Count/cell	97.64198	87	45.32971
ACTB	W1	PGE2	well_4	F001	31	Count/cell	73.17857	65	31.34167
ACTB	W1	PGE2	well_4	F002	28	Count/cell	68.8	73	27.26261
ACTB	W1	PGE2	well_4	F003	58	Count/cell	83.42308	83.5	36.16647
ACTB	W1	PGE2	well_4	F004	16	Count/cell	75.64286	63	47.1244
ACTB	W1	PGE2	well_4	F005	19	Count/cell	119.7647	118	41.78596
ACTB	W1	PGE2	well_4	F006	9	Count/cell	92.71429	91	22.3809
ACTB	W1	PGE2	well_5	F001	26	Count/cell	69.77273	70	28.30603
ACTB	W1	PGE2	well_5	F002	31	Count/cell	68.03704	64	24.66309
ACTB	W1	PGE2	well_5	F003	11	Count/cell	122.1111	125	33.80253
ACTB	W1	PGE2	well_5	F004	30	Count/cell	91.03846	89.5	35.8558
ACTB	W1	PGE2	well_5	F005	22	Count/cell	95.5	92	39.06443
ACTB	W1	PGE2	well_5	F006	60	Count/cell	96.47273	87	42.74509
ACTB	W2	HPI4	well_1	F001	1	Count/cell	28	28	NA
ACTB	W2	HPI4	well_1	F002	1	Count/cell	38	38	NA
ACTB	W2	HPI4	well_1	F004	2	Count/cell	250	250	0
ACTB	W2	HPI4	well_1	F005	3	Count/cell	27	27	NA
ACTB	W2	HPI4	well_1	F006	3	Count/cell	81	81	NA
ACTB	W2	HPI4	well_2	F006	3	Count/cell	65	65	NA
ACTB	W2	HPI4	well_3	F001	5	Count/cell	75.66667	70	35.8376
ACTB	W2	HPI4	well_3	F002	1	Count/cell	30	30	NA

ACTB	W2	HPI4	well_3	F003	1	Count/cell	59	59	NA
ACTB	W2	HPI4	well_3	F006	1	Count/cell	137	137	NA
ACTB	W2	HPI4	well_4	F002	1	Count/cell	2	2	NA
ACTB	W2	HPI4	well_4	F006	3	Count/cell	90	90	NA
ACTB	W2	HPI4	well_5	F001	14	Count/cell	47.15385	44	17.69579
ACTB	W2	HPI4	well_5	F003	1	Count/cell	69	69	NA
ACTB	W2	HPI4	well_5	F004	3	Count/cell	83	83	NA
ACTB	W2	HPI4	well_5	F006	16	Count/cell	45.28571	40	17.04035
ACTB	W2	PGE2	well_1	F001	126	Count/cell	67.58036	66	21.87501
ACTB	W2	PGE2	well_1	F002	139	Count/cell	69.3254	66	30.53439
ACTB	W2	PGE2	well_1	F003	180	Count/cell	67.67284	68	23.03274
ACTB	W2	PGE2	well_1	F004	97	Count/cell	50.32184	47	22.87148
ACTB	W2	PGE2	well_1	F005	101	Count/cell	56.40659	50	31.14981
ACTB	W2	PGE2	well_1	F006	72	Count/cell	37.0625	36.5	17.43093
ACTB	W2	PGE2	well_2	F001	59	Count/cell	38.9434	38	11.33479
ACTB	W2	PGE2	well_2	F002	91	Count/cell	51.17073	51.5	19.97023
ACTB	W2	PGE2	well_2	F003	142	Count/cell	50.53543	51	19.4874
ACTB	W2	PGE2	well_2	F004	60	Count/cell	55.98148	53.5	21.44452
ACTB	W2	PGE2	well_2	F005	36	Count/cell	50.1875	48.5	24.49152
ACTB	W2	PGE2	well_2	F006	123	Count/cell	51.02752	49	17.04268
ACTB	W2	PGE2	well_3	F001	67	Count/cell	41.71186	42	15.32302
ACTB	W2	PGE2	well_3	F002	90	Count/cell	46.4	44.5	19.06923
ACTB	W2	PGE2	well_3	F003	99	Count/cell	47	43	19.2472
ACTB	W2	PGE2	well_3	F004	20	Count/cell	42.72222	39	16.94561
ACTB	W2	PGE2	well_3	F005	83	Count/cell	54.36986	48	23.76535
ACTB	W2	PGE2	well_3	F006	102	Count/cell	52.21111	52.5	17.4951
ACTB	W2	PGE2	well_4	F001	36	Count/cell	37.25	37.5	16.64429
ACTB	W2	PGE2	well_4	F002	38	Count/cell	35.94286	31	16.34903
ACTB	W2	PGE2	well_4	F003	38	Count/cell	35.94286	35	19.6797
ACTB	W2	PGE2	well_4	F004	53	Count/cell	44.95745	39	22.36939
ACTB	W2	PGE2	well_4	F005	67	Count/cell	38.8	36.5	19.71646
ACTB	W2	PGE2	well_4	F006	56	Count/cell	42.76	39.5	16.63971
ACTB	W2	PGE2	well_5	F001	15	Count/cell	43.69231	35	24.34264
ACTB	W2	PGE2	well_5	F002	21	Count/cell	55.57895	55	28.23771
ACTB	W2	PGE2	well_5	F003	20	Count/cell	51.11111	52	19.64655
ACTB	W2	PGE2	well_5	F004	36	Count/cell	60.875	64	25.67382
ACTB	W2	PGE2	well_5	F005	29	Count/cell	51.56	50	14.45706
ACTB	W2	PGE2	well_5	F006	35	Count/cell	50	48	20.05825
ACTB	W3	HPI4	well_1	F001	43	Count/cell	39.13514	36	15.14222
ACTB	W3	HPI4	well_1	F002	36	Count/cell	88	87	30.78542
ACTB	W3	HPI4	well_1	F003	78	Count/cell	35.29577	31	13.981
ACTB	W3	HPI4	well_1	F004	122	Count/cell	41.89815	38	17.77636
ACTB	W3	HPI4	well_1	F005	68	Count/cell	39.28333	35	13.63208
ACTB	W3	HPI4	well_1	F006	59	Count/cell	47.7037	45.5	20.06088
ACTB	W3	HPI4	well_2	F001	86	Count/cell	44.48052	44	14.95309
ACTB	W3	HPI4	well_2	F002	58	Count/cell	59.40385	60.5	19.46768
ACTB	W3	HPI4	well_2	F003	52	Count/cell	57.95652	58.5	16.66661
ACTB	W3	HPI4	well_2	F004	55	Count/cell	73.18367	69	26.09731
ACTB	W3	HPI4	well_2	F005	111	Count/cell	42.47475	42	15.55526

ACTB	W3	HPI4	well_2	F006	95	Count/cell	54.18605	50	21.80853
ACTB	W3	HPI4	well_3	F001	32	Count/cell	53.89286	54	18.54592
ACTB	W3	HPI4	well_3	F002	37	Count/cell	58.09091	53	20.92899
ACTB	W3	HPI4	well_3	F003	6	Count/cell	57	54.5	19.0263
ACTB	W3	HPI4	well_3	F004	37	Count/cell	72.90909	72	34.61246
ACTB	W3	HPI4	well_3	F005	12	Count/cell	32.7	29	15.29742
ACTB	W3	HPI4	well_3	F006	59	Count/cell	47.07547	45	16.35995
ACTB	W3	HPI4	well_4	F001	103	Count/cell	57.10638	55.5	26.4386
ACTB	W3	HPI4	well_4	F002	91	Count/cell	63.2439	59.5	27.18458
ACTB	W3	HPI4	well_4	F003	21	Count/cell	52.78947	43	26.42091
ACTB	W3	HPI4	well_4	F004	9	Count/cell	74	75	16.91153
ACTB	W3	HPI4	well_4	F005	58	Count/cell	59.5	56	14.85947
ACTB	W3	HPI4	well_4	F006	70	Count/cell	58.28125	57	21.6747
ACTB	W3	HPI4	well_5	F001	20	Count/cell	65.27778	55.5	35.365
ACTB	W3	HPI4	well_5	F002	8	Count/cell	84.66667	77.5	21.85101
ACTB	W3	HPI4	well_5	F003	4	Count/cell	160	160	52.3259
ACTB	W3	HPI4	well_5	F004	5	Count/cell	59.66667	61	23.02897
ACTB	W3	HPI4	well_5	F005	26	Count/cell	85.21739	87	31.26421
ACTB	W3	HPI4	well_5	F006	18	Count/cell	86.25	94	31.90716
ACTB	W3	PGE2	well_1	F001	162	Count/cell	54.15972	55	20.35924
ACTB	W3	PGE2	well_1	F002	232	Count/cell	45.69048	44.5	18.73115
ACTB	W3	PGE2	well_1	F003	294	Count/cell	43.59623	43	13.79391
ACTB	W3	PGE2	well_1	F004	58	Count/cell	72.13462	66.5	29.14805
ACTB	W3	PGE2	well_1	F005	80	Count/cell	52.55556	51.5	18.47487
ACTB	W3	PGE2	well_1	F006	281	Count/cell	40.49012	40	15.46286
ACTB	W3	PGE2	well_2	F001	113	Count/cell	50.65347	47	19.56908
ACTB	W3	PGE2	well_2	F002	160	Count/cell	56.51724	55	23.38866
ACTB	W3	PGE2	well_2	F003	190	Count/cell	51.46784	51	18.20158
ACTB	W3	PGE2	well_2	F004	155	Count/cell	55.48201	52	17.50791
ACTB	W3	PGE2	well_2	F005	288	Count/cell	49.62934	48	17.19163
ACTB	W3	PGE2	well_2	F006	191	Count/cell	52.74857	49	18.45419
ACTB	W3	PGE2	well_3	F001	110	Count/cell	54.02041	54	19.51102
ACTB	W3	PGE2	well_3	F002	211	Count/cell	50.32292	49.5	18.05489
ACTB	W3	PGE2	well_3	F003	201	Count/cell	49.48619	49	16.62682
ACTB	W3	PGE2	well_3	F004	83	Count/cell	66.77027	68	24.09089
ACTB	W3	PGE2	well_3	F005	217	Count/cell	45.44388	44	16.58086
ACTB	W3	PGE2	well_3	F006	227	Count/cell	45.87685	44	15.20551
ACTB	W3	PGE2	well_4	F001	176	Count/cell	48.26415	48	16.45187
ACTB	W3	PGE2	well_4	F002	159	Count/cell	63.58333	63	21.02995
ACTB	W3	PGE2	well_4	F003	251	Count/cell	51.30531	47	18.723
ACTB	W3	PGE2	well_4	F004	161	Count/cell	51.06803	48	23.16695
ACTB	W3	PGE2	well_4	F005	196	Count/cell	45.16477	44.5	14.87629
ACTB	W3	PGE2	well_4	F006	196	Count/cell	46.11173	45	15.29004
ACTB	W3	PGE2	well_5	F001	105	Count/cell	59.31183	58	15.86422
ACTB	W3	PGE2	well_5	F002	132	Count/cell	60.68908	57	19.41812
ACTB	W3	PGE2	well_5	F003	223	Count/cell	48.0199	46	14.8526
ACTB	W3	PGE2	well_5	F004	102	Count/cell	57.73626	57	15.05459
ACTB	W3	PGE2	well_5	F005	222	Count/cell	42.75253	42	14.99727
ACTB	W3	PGE2	well_5	F006	204	Count/cell	48.6978	48	14.55688

ACTB	W4	HPI4	well_1	F001	36	Count/cell	77.125	70	32.42236
ACTB	W4	HPI4	well_1	F002	4	Count/cell	62	62	4.242641
ACTB	W4	HPI4	well_1	F003	12	Count/cell	78.4	68	35.85836
ACTB	W4	HPI4	well_1	F004	23	Count/cell	36.05263	29	16.62486
ACTB	W4	HPI4	well_1	F005	39	Count/cell	51.37143	47	21.42303
ACTB	W4	HPI4	well_1	F006	27	Count/cell	60.26087	61	25.49727
ACTB	W4	HPI4	well_2	F001	25	Count/cell	49.47619	49	18.63228
ACTB	W4	HPI4	well_2	F003	4	Count/cell	77.5	77.5	20.5061
ACTB	W4	HPI4	well_2	F004	10	Count/cell	36.5	38	11.73517
ACTB	W4	HPI4	well_2	F005	38	Count/cell	41.85294	43	14.7856
ACTB	W4	HPI4	well_2	F006	13	Count/cell	80	71	24.37622
ACTB	W4	HPI4	well_3	F001	8	Count/cell	82.16667	95.5	40.082
ACTB	W4	HPI4	well_3	F002	3	Count/cell	14	14	NA
ACTB	W4	HPI4	well_3	F003	1	Count/cell	57	57	NA
ACTB	W4	HPI4	well_3	F004	1	Count/cell	48	48	NA
ACTB	W4	HPI4	well_3	F006	5	Count/cell	61.33333	60	28.0238
ACTB	W4	HPI4	well_4	F001	14	Count/cell	73.66667	67.5	31.77573
ACTB	W4	HPI4	well_4	F002	23	Count/cell	44.63158	49	17.15035
ACTB	W4	HPI4	well_4	F003	3	Count/cell	45	45	NA
ACTB	W4	HPI4	well_4	F004	6	Count/cell	81.75	78.5	25.59134
ACTB	W4	HPI4	well_4	F005	19	Count/cell	66.52941	58	32.734
ACTB	W4	HPI4	well_4	F006	4	Count/cell	56.5	56.5	28.99138
ACTB	W4	HPI4	well_5	F001	3	Count/cell	69	69	NA
ACTB	W4	HPI4	well_5	F002	8	Count/cell	43.5	32	34.4369
ACTB	W4	HPI4	well_5	F003	1	Count/cell	65	65	NA
ACTB	W4	HPI4	well_5	F004	5	Count/cell	68.66667	78	25.32456
ACTB	W4	HPI4	well_5	F005	3	Count/cell	66	66	NA
ACTB	W4	HPI4	well_5	F006	17	Count/cell	64.26667	56	44.62937
ACTB	W4	PGE2	well_1	F001	231	Count/cell	57.92754	54	25.54094
ACTB	W4	PGE2	well_1	F002	289	Count/cell	43.04231	40.5	17.01266
ACTB	W4	PGE2	well_1	F003	254	Count/cell	55.83843	52	25.46937
ACTB	W4	PGE2	well_1	F004	280	Count/cell	47.34127	43	22.91459
ACTB	W4	PGE2	well_1	F005	291	Count/cell	53.30682	49	24.93293
ACTB	W4	PGE2	well_1	F006	318	Count/cell	41.40559	39	16.11199
ACTB	W4	PGE2	well_2	F001	235	Count/cell	57.29858	56	19.72962
ACTB	W4	PGE2	well_2	F002	265	Count/cell	45.03704	41	21.04174
ACTB	W4	PGE2	well_2	F003	228	Count/cell	58.14146	53	22.59586
ACTB	W4	PGE2	well_2	F004	337	Count/cell	44.44737	42	16.56781
ACTB	W4	PGE2	well_2	F005	295	Count/cell	43.42804	41	18.36264
ACTB	W4	PGE2	well_2	F006	285	Count/cell	57.03101	55	22.73051
ACTB	W4	PGE2	well_3	F001	297	Count/cell	39.89963	38	16.83505
ACTB	W4	PGE2	well_3	F002	253	Count/cell	51.28696	49.5	18.31398
ACTB	W4	PGE2	well_3	F003	245	Count/cell	50.00909	47	20.08907
ACTB	W4	PGE2	well_3	F004	286	Count/cell	44.6965	40	21.522
ACTB	W4	PGE2	well_3	F005	248	Count/cell	63.8964	62	19.53262
ACTB	W4	PGE2	well_3	F006	338	Count/cell	36.79479	34	16.31252
ACTB	W4	PGE2	well_4	F001	322	Count/cell	32.33681	31	11.50584
ACTB	W4	PGE2	well_4	F002	230	Count/cell	47.80193	44	20.20488
ACTB	W4	PGE2	well_4	F003	289	Count/cell	45.49807	45	14.19394

ACTB	W4	PGE2	well_4	F004	120	Count/cell	46.58182	45	21.10541
ACTB	W4	PGE2	well_4	F005	239	Count/cell	52.01852	50	20.02254
ACTB	W4	PGE2	well_4	F006	154	Count/cell	55.55797	52	20.10085
ACTB	W4	PGE2	well_5	F001	95	Count/cell	50.76471	47	21.8385
ACTB	W4	PGE2	well_5	F002	188	Count/cell	49.125	48	17.51208
ACTB	W4	PGE2	well_5	F003	264	Count/cell	42.84322	38	20.71377
ACTB	W4	PGE2	well_5	F004	204	Count/cell	53.2967	49.5	22.49812
ACTB	W4	PGE2	well_5	F005	255	Count/cell	44.7	40.5	18.64191
ACTB	W4	PGE2	well_5	F006	207	Count/cell	42.58602	38	18.82481
ACTB	W1	HPI4	well_1	F001	11	X/Y distribution	8.434782	7.971447	2.107614
ACTB	W1	HPI4	well_1	F002	37	X/Y distribution	7.167289	7.311092	1.172323
ACTB	W1	HPI4	well_1	F003	50	X/Y distribution	7.914017	7.625	1.307048
ACTB	W1	HPI4	well_1	F004	18	X/Y distribution	7.854878	7.834374	1.241433
ACTB	W1	HPI4	well_1	F005	14	X/Y distribution	6.857136	6.233466	1.349161
ACTB	W1	HPI4	well_1	F006	39	X/Y distribution	7.683776	7.635682	1.681859
ACTB	W1	HPI4	well_2	F001	100	X/Y distribution	7.074555	6.949082	1.173827
ACTB	W1	HPI4	well_2	F002	60	X/Y distribution	8.005755	8.031935	1.44848
ACTB	W1	HPI4	well_2	F003	68	X/Y distribution	7.151767	7.153692	1.137118
ACTB	W1	HPI4	well_2	F004	33	X/Y distribution	7.522087	7.682722	1.591534
ACTB	W1	HPI4	well_2	F005	19	X/Y distribution	8.128878	8.009375	1.638011
ACTB	W1	HPI4	well_2	F006	46	X/Y distribution	7.634093	7.610871	1.263398
ACTB	W1	HPI4	well_3	F001	137	X/Y distribution	8.604879	8.503706	1.664278
ACTB	W1	HPI4	well_3	F002	23	X/Y distribution	7.722145	7.355155	1.471815
ACTB	W1	HPI4	well_3	F003	21	X/Y distribution	8.225239	8.360886	1.699628
ACTB	W1	HPI4	well_3	F004	59	X/Y distribution	7.399838	7.492823	1.149492
ACTB	W1	HPI4	well_3	F005	58	X/Y distribution	8.747522	8.589032	1.850208
ACTB	W1	HPI4	well_3	F006	67	X/Y distribution	7.463741	7.67847	1.23323
ACTB	W1	HPI4	well_4	F001	46	X/Y distribution	8.35341	8.04787	1.525245
ACTB	W1	HPI4	well_4	F002	95	X/Y distribution	7.096177	7.132485	1.048424
ACTB	W1	HPI4	well_4	F003	51	X/Y distribution	7.427901	7.477595	1.508522
ACTB	W1	HPI4	well_4	F004	94	X/Y distribution	7.770278	7.537668	1.300601
ACTB	W1	HPI4	well_4	F005	34	X/Y distribution	7.588316	7.637667	1.262355
ACTB	W1	HPI4	well_4	F006	56	X/Y distribution	8.644724	8.614715	1.188257
ACTB	W1	HPI4	well_5	F001	85	X/Y distribution	7.103656	7.127648	1.245375
ACTB	W1	HPI4	well_5	F002	62	X/Y distribution	6.452193	6.158725	0.957231
ACTB	W1	HPI4	well_5	F003	75	X/Y distribution	8.123283	8.303433	1.424509
ACTB	W1	HPI4	well_5	F004	61	X/Y distribution	6.997711	7.072029	0.93422
ACTB	W1	HPI4	well_5	F005	130	X/Y distribution	7.049436	7.039918	1.040478
ACTB	W1	HPI4	well_5	F006	96	X/Y distribution	7.656058	7.456397	1.608605
ACTB	W1	PGE2	well_1	F001	35	X/Y distribution	7.794721	7.606258	1.639284
ACTB	W1	PGE2	well_1	F002	17	X/Y distribution	7.961504	7.916133	1.384656
ACTB	W1	PGE2	well_1	F003	46	X/Y distribution	7.411387	7.307221	1.129648
ACTB	W1	PGE2	well_1	F004	84	X/Y distribution	7.9333	7.831054	1.613945
ACTB	W1	PGE2	well_1	F005	130	X/Y distribution	8.19592	8.093656	1.396434
ACTB	W1	PGE2	well_1	F006	74	X/Y distribution	7.366834	7.187015	1.216115
ACTB	W1	PGE2	well_2	F001	37	X/Y distribution	7.850888	7.736889	1.201082
ACTB	W1	PGE2	well_2	F002	18	X/Y distribution	8.192127	7.903084	1.592181
ACTB	W1	PGE2	well_2	F003	39	X/Y distribution	7.428081	7.297992	1.895432
ACTB	W1	PGE2	well_2	F004	46	X/Y distribution	8.467121	8.224644	1.448933

ACTB	W1	PGE2	well_2	F005	43	X/Y distribution	8.383397	8.523079	1.485327
ACTB	W1	PGE2	well_2	F006	48	X/Y distribution	7.680103	7.723363	1.516645
ACTB	W1	PGE2	well_3	F001	59	X/Y distribution	7.706085	7.757705	1.388694
ACTB	W1	PGE2	well_3	F002	79	X/Y distribution	8.753623	8.470185	1.408484
ACTB	W1	PGE2	well_3	F003	31	X/Y distribution	8.121381	7.388689	2.068805
ACTB	W1	PGE2	well_3	F004	29	X/Y distribution	8.2019	8.132307	1.352733
ACTB	W1	PGE2	well_3	F005	74	X/Y distribution	7.527087	7.449494	1.466383
ACTB	W1	PGE2	well_3	F006	90	X/Y distribution	8.057262	8.22494	1.283722
ACTB	W1	PGE2	well_4	F001	31	X/Y distribution	7.073014	6.732277	1.093791
ACTB	W1	PGE2	well_4	F002	28	X/Y distribution	7.134579	7.094948	1.560413
ACTB	W1	PGE2	well_4	F003	58	X/Y distribution	9.071075	9.338149	1.847268
ACTB	W1	PGE2	well_4	F004	16	X/Y distribution	8.024659	8.269286	1.511666
ACTB	W1	PGE2	well_4	F005	19	X/Y distribution	9.46441	9.450263	1.281961
ACTB	W1	PGE2	well_4	F006	9	X/Y distribution	7.476106	7.288173	1.08773
ACTB	W1	PGE2	well_5	F001	26	X/Y distribution	6.846758	6.732672	1.231099
ACTB	W1	PGE2	well_5	F002	31	X/Y distribution	7.788661	8.056591	1.221159
ACTB	W1	PGE2	well_5	F003	11	X/Y distribution	9.092346	9.412744	1.678098
ACTB	W1	PGE2	well_5	F004	30	X/Y distribution	8.256504	7.675105	1.713816
ACTB	W1	PGE2	well_5	F005	22	X/Y distribution	8.503986	8.466971	1.677065
ACTB	W1	PGE2	well_5	F006	60	X/Y distribution	8.214813	8.043316	1.607811
ACTB	W2	HPI4	well_1	F001	1	X/Y distribution	2.206875	2.206875	NA
ACTB	W2	HPI4	well_1	F002	1	X/Y distribution	4.399867	4.399867	NA
ACTB	W2	HPI4	well_1	F005	3	X/Y distribution	2.76245	2.76245	NA
ACTB	W2	HPI4	well_1	F006	3	X/Y distribution	4.873791	4.873791	NA
ACTB	W2	HPI4	well_2	F006	3	X/Y distribution	3.280668	3.280668	NA
ACTB	W2	HPI4	well_3	F001	5	X/Y distribution	4.080347	4.304612	0.455867
ACTB	W2	HPI4	well_3	F002	1	X/Y distribution	4.700473	4.700473	NA
ACTB	W2	HPI4	well_3	F003	1	X/Y distribution	2.665887	2.665887	NA
ACTB	W2	HPI4	well_3	F006	1	X/Y distribution	6.224498	6.224498	NA
ACTB	W2	HPI4	well_4	F002	1	X/Y distribution	5.642374	5.642374	NA
ACTB	W2	HPI4	well_4	F006	3	X/Y distribution	8.768072	8.768072	NA
ACTB	W2	HPI4	well_5	F001	14	X/Y distribution	3.81335	3.826254	0.640386
ACTB	W2	HPI4	well_5	F003	1	X/Y distribution	5.048374	5.048374	NA
ACTB	W2	HPI4	well_5	F004	3	X/Y distribution	4.088722	4.088722	NA
ACTB	W2	HPI4	well_5	F006	16	X/Y distribution	4.554258	4.331901	0.884728
ACTB	W2	PGE2	well_1	F001	126	X/Y distribution	6.26935	6.223136	1.029729
ACTB	W2	PGE2	well_1	F002	139	X/Y distribution	6.179089	5.988351	1.291868
ACTB	W2	PGE2	well_1	F003	180	X/Y distribution	6.142522	6.042442	1.18996
ACTB	W2	PGE2	well_1	F004	97	X/Y distribution	6.335373	6.301279	1.881818
ACTB	W2	PGE2	well_1	F005	101	X/Y distribution	5.522293	5.420829	1.497733
ACTB	W2	PGE2	well_1	F006	72	X/Y distribution	5.692801	5.747986	1.648354
ACTB	W2	PGE2	well_2	F001	59	X/Y distribution	4.516069	4.463327	0.877197
ACTB	W2	PGE2	well_2	F002	91	X/Y distribution	5.408103	5.335421	0.970074
ACTB	W2	PGE2	well_2	F003	142	X/Y distribution	5.310129	5.360597	0.86614
ACTB	W2	PGE2	well_2	F004	60	X/Y distribution	5.247404	5.247818	1.072692
ACTB	W2	PGE2	well_2	F005	36	X/Y distribution	5.143633	5.057654	1.102813
ACTB	W2	PGE2	well_2	F006	123	X/Y distribution	5.0547	5.100394	0.893163
ACTB	W2	PGE2	well_3	F001	67	X/Y distribution	5.202657	5.094794	1.031479
ACTB	W2	PGE2	well_3	F002	90	X/Y distribution	5.579965	5.609464	1.156675

ACTB	W2	PGE2	well_3	F003	99	X/Y distribution	4.878815	4.884635	1.070429
ACTB	W2	PGE2	well_3	F004	20	X/Y distribution	4.783868	4.730197	0.692873
ACTB	W2	PGE2	well_3	F005	83	X/Y distribution	5.866913	5.94562	1.209542
ACTB	W2	PGE2	well_3	F006	102	X/Y distribution	5.137394	5.046906	1.021643
ACTB	W2	PGE2	well_4	F001	36	X/Y distribution	4.757921	4.611299	1.089296
ACTB	W2	PGE2	well_4	F002	38	X/Y distribution	4.841432	4.543887	1.348124
ACTB	W2	PGE2	well_4	F003	38	X/Y distribution	4.774524	4.839571	1.428524
ACTB	W2	PGE2	well_4	F004	53	X/Y distribution	5.386805	5.227369	1.407248
ACTB	W2	PGE2	well_4	F005	67	X/Y distribution	4.719226	4.428781	1.28397
ACTB	W2	PGE2	well_4	F006	56	X/Y distribution	4.558461	4.382954	1.234273
ACTB	W2	PGE2	well_5	F001	15	X/Y distribution	4.94568	4.242095	1.859984
ACTB	W2	PGE2	well_5	F002	21	X/Y distribution	5.560623	5.665487	1.167261
ACTB	W2	PGE2	well_5	F003	20	X/Y distribution	5.21567	5.05805	1.472652
ACTB	W2	PGE2	well_5	F004	36	X/Y distribution	5.841857	5.767037	1.453721
ACTB	W2	PGE2	well_5	F005	29	X/Y distribution	5.244147	5.028007	1.018196
ACTB	W2	PGE2	well_5	F006	35	X/Y distribution	4.828611	4.758571	0.927946
ACTB	W3	HPI4	well_1	F001	43	X/Y distribution	6.945892	6.870361	1.062315
ACTB	W3	HPI4	well_1	F002	36	X/Y distribution	9.512491	9.481137	1.697619
ACTB	W3	HPI4	well_1	F003	78	X/Y distribution	7.01704	6.764698	1.2608
ACTB	W3	HPI4	well_1	F004	122	X/Y distribution	8.1849	8.271421	1.703791
ACTB	W3	HPI4	well_1	F005	68	X/Y distribution	8.097556	7.978154	1.381922
ACTB	W3	HPI4	well_1	F006	59	X/Y distribution	8.599317	8.389136	1.494954
ACTB	W3	HPI4	well_2	F001	86	X/Y distribution	8.004564	7.894078	1.425966
ACTB	W3	HPI4	well_2	F002	58	X/Y distribution	8.268621	8.007017	1.59544
ACTB	W3	HPI4	well_2	F003	52	X/Y distribution	8.584258	8.391872	1.217539
ACTB	W3	HPI4	well_2	F004	55	X/Y distribution	8.802123	8.679568	1.506604
ACTB	W3	HPI4	well_2	F005	111	X/Y distribution	7.82207	7.532274	1.522586
ACTB	W3	HPI4	well_2	F006	95	X/Y distribution	8.580112	8.563112	1.585768
ACTB	W3	HPI4	well_3	F001	32	X/Y distribution	7.244076	6.846509	1.429772
ACTB	W3	HPI4	well_3	F002	37	X/Y distribution	7.423471	7.839162	1.558206
ACTB	W3	HPI4	well_3	F003	6	X/Y distribution	7.068234	7.027901	0.518091
ACTB	W3	HPI4	well_3	F004	37	X/Y distribution	7.379897	6.9029	1.772321
ACTB	W3	HPI4	well_3	F005	12	X/Y distribution	6.141036	5.721011	2.050493
ACTB	W3	HPI4	well_3	F006	59	X/Y distribution	8.054293	7.940964	1.248587
ACTB	W3	HPI4	well_4	F001	103	X/Y distribution	6.735403	6.453951	1.596617
ACTB	W3	HPI4	well_4	F002	91	X/Y distribution	6.909684	6.677105	1.310899
ACTB	W3	HPI4	well_4	F003	21	X/Y distribution	6.571848	6.881904	1.351011
ACTB	W3	HPI4	well_4	F004	9	X/Y distribution	9.245248	10.03971	1.610734
ACTB	W3	HPI4	well_4	F005	58	X/Y distribution	7.952779	8.023537	1.417892
ACTB	W3	HPI4	well_4	F006	70	X/Y distribution	9.011645	8.92534	1.615191
ACTB	W3	HPI4	well_5	F001	20	X/Y distribution	6.6296	7.08618	1.696651
ACTB	W3	HPI4	well_5	F002	8	X/Y distribution	8.203777	8.747437	2.007695
ACTB	W3	HPI4	well_5	F003	4	X/Y distribution	8.783547	8.783547	1.392304
ACTB	W3	HPI4	well_5	F004	5	X/Y distribution	7.608461	7.980656	0.85794
ACTB	W3	HPI4	well_5	F005	26	X/Y distribution	7.957163	8.193673	2.213124
ACTB	W3	HPI4	well_5	F006	18	X/Y distribution	8.428084	9.050799	2.40645
ACTB	W3	PGE2	well_1	F001	162	X/Y distribution	6.565803	6.498616	1.040527
ACTB	W3	PGE2	well_1	F002	232	X/Y distribution	7.288794	7.201448	1.121806
ACTB	W3	PGE2	well_1	F003	294	X/Y distribution	6.913737	6.847352	0.952472

ACTB	W3	PGE2	well_1	F004	58	X/Y distribution	7.38267	7.389669	1.090772
ACTB	W3	PGE2	well_1	F005	80	X/Y distribution	6.432492	6.272046	0.981321
ACTB	W3	PGE2	well_1	F006	281	X/Y distribution	6.895066	6.795469	0.915347
ACTB	W3	PGE2	well_2	F001	113	X/Y distribution	6.695168	6.523088	1.153346
ACTB	W3	PGE2	well_2	F002	160	X/Y distribution	7.840707	7.714085	1.363256
ACTB	W3	PGE2	well_2	F003	190	X/Y distribution	6.951697	6.671987	1.268299
ACTB	W3	PGE2	well_2	F004	155	X/Y distribution	6.825472	6.765544	1.089811
ACTB	W3	PGE2	well_2	F005	288	X/Y distribution	7.032233	6.985531	1.05087
ACTB	W3	PGE2	well_2	F006	191	X/Y distribution	7.029917	6.982117	1.085961
ACTB	W3	PGE2	well_3	F001	110	X/Y distribution	7.412421	7.243214	1.164517
ACTB	W3	PGE2	well_3	F002	211	X/Y distribution	7.809508	7.614035	1.159783
ACTB	W3	PGE2	well_3	F003	201	X/Y distribution	7.297418	7.322542	1.09456
ACTB	W3	PGE2	well_3	F004	83	X/Y distribution	6.845641	6.715464	1.057473
ACTB	W3	PGE2	well_3	F005	217	X/Y distribution	7.192713	7.081841	0.991112
ACTB	W3	PGE2	well_3	F006	227	X/Y distribution	7.112063	7.044114	0.963054
ACTB	W3	PGE2	well_4	F001	176	X/Y distribution	6.618544	6.562588	0.945023
ACTB	W3	PGE2	well_4	F002	159	X/Y distribution	7.933508	7.830379	1.458804
ACTB	W3	PGE2	well_4	F003	251	X/Y distribution	6.922643	6.817974	1.071698
ACTB	W3	PGE2	well_4	F004	161	X/Y distribution	7.601856	7.31749	1.414569
ACTB	W3	PGE2	well_4	F005	196	X/Y distribution	7.900439	7.838777	1.154505
ACTB	W3	PGE2	well_4	F006	196	X/Y distribution	6.952316	6.974351	0.977419
ACTB	W3	PGE2	well_5	F001	105	X/Y distribution	7.371199	7.36962	1.037348
ACTB	W3	PGE2	well_5	F002	132	X/Y distribution	7.313181	7.109941	1.244726
ACTB	W3	PGE2	well_5	F003	223	X/Y distribution	7.364963	7.218079	1.227428
ACTB	W3	PGE2	well_5	F004	102	X/Y distribution	6.647071	6.648391	0.979214
ACTB	W3	PGE2	well_5	F005	222	X/Y distribution	7.025396	6.964403	1.155185
ACTB	W3	PGE2	well_5	F006	204	X/Y distribution	7.342552	7.167347	1.055962
ACTB	W4	HPI4	well_1	F001	36	X/Y distribution	8.955368	9.062145	1.470221
ACTB	W4	HPI4	well_1	F002	4	X/Y distribution	7.577184	7.577184	0.200643
ACTB	W4	HPI4	well_1	F003	12	X/Y distribution	7.737104	7.81805	0.815404
ACTB	W4	HPI4	well_1	F004	23	X/Y distribution	6.993961	7.043921	1.590852
ACTB	W4	HPI4	well_1	F005	39	X/Y distribution	8.864401	8.76027	1.905723
ACTB	W4	HPI4	well_1	F006	27	X/Y distribution	8.647697	8.286148	1.846214
ACTB	W4	HPI4	well_2	F001	25	X/Y distribution	7.122879	7.171021	1.410994
ACTB	W4	HPI4	well_2	F003	4	X/Y distribution	8.272624	8.272624	0.364529
ACTB	W4	HPI4	well_2	F004	10	X/Y distribution	6.304146	5.942358	1.202048
ACTB	W4	HPI4	well_2	F005	38	X/Y distribution	6.400737	6.494595	1.397872
ACTB	W4	HPI4	well_2	F006	13	X/Y distribution	8.173539	8.079746	2.400768
ACTB	W4	HPI4	well_3	F001	8	X/Y distribution	7.377758	7.656312	1.831439
ACTB	W4	HPI4	well_3	F002	3	X/Y distribution	5.101864	5.101864	NA
ACTB	W4	HPI4	well_3	F003	1	X/Y distribution	5.232291	5.232291	NA
ACTB	W4	HPI4	well_3	F004	1	X/Y distribution	4.430873	4.430873	NA
ACTB	W4	HPI4	well_3	F006	5	X/Y distribution	7.372284	7.514911	1.249032
ACTB	W4	HPI4	well_4	F001	14	X/Y distribution	8.372148	8.387489	1.444052
ACTB	W4	HPI4	well_4	F002	23	X/Y distribution	6.935832	7.055018	1.025273
ACTB	W4	HPI4	well_4	F003	3	X/Y distribution	6.110986	6.110986	NA
ACTB	W4	HPI4	well_4	F004	6	X/Y distribution	8.464025	8.505107	2.084177
ACTB	W4	HPI4	well_4	F005	19	X/Y distribution	7.501235	7.238072	1.337773
ACTB	W4	HPI4	well_4	F006	4	X/Y distribution	7.895321	7.895321	0.208831

ACTB	W4	HPI4	well_5	F001	3	X/Y distribution	5.730365	5.730365	NA
ACTB	W4	HPI4	well_5	F002	8	X/Y distribution	6.307589	5.235813	2.32109
ACTB	W4	HPI4	well_5	F003	1	X/Y distribution	5.13073	5.13073	NA
ACTB	W4	HPI4	well_5	F004	5	X/Y distribution	8.63485	9.179372	1.162659
ACTB	W4	HPI4	well_5	F005	3	X/Y distribution	5.986784	5.986784	NA
ACTB	W4	HPI4	well_5	F006	17	X/Y distribution	7.197384	7.351544	2.212657
ACTB	W4	PGE2	well_1	F001	231	X/Y distribution	8.095218	8.007962	1.233386
ACTB	W4	PGE2	well_1	F002	289	X/Y distribution	7.189617	7.101268	1.01944
ACTB	W4	PGE2	well_1	F003	254	X/Y distribution	7.759397	7.667493	1.065033
ACTB	W4	PGE2	well_1	F004	280	X/Y distribution	7.214125	7.125799	1.05935
ACTB	W4	PGE2	well_1	F005	291	X/Y distribution	7.566452	7.479333	1.101715
ACTB	W4	PGE2	well_1	F006	318	X/Y distribution	6.572114	6.320008	1.12838
ACTB	W4	PGE2	well_2	F001	235	X/Y distribution	7.705473	7.618378	1.227181
ACTB	W4	PGE2	well_2	F002	265	X/Y distribution	7.738641	7.606211	1.197288
ACTB	W4	PGE2	well_2	F003	228	X/Y distribution	7.560386	7.621765	1.198644
ACTB	W4	PGE2	well_2	F004	337	X/Y distribution	6.749931	6.633669	0.946791
ACTB	W4	PGE2	well_2	F005	295	X/Y distribution	7.10831	7.049658	1.096885
ACTB	W4	PGE2	well_2	F006	285	X/Y distribution	7.199901	7.353328	1.037783
ACTB	W4	PGE2	well_3	F001	297	X/Y distribution	7.283331	7.306092	1.120951
ACTB	W4	PGE2	well_3	F002	253	X/Y distribution	7.306105	7.308291	0.988189
ACTB	W4	PGE2	well_3	F003	245	X/Y distribution	7.538882	7.291101	1.245141
ACTB	W4	PGE2	well_3	F004	286	X/Y distribution	7.402643	7.341773	1.150191
ACTB	W4	PGE2	well_3	F005	248	X/Y distribution	7.788786	7.77282	1.065549
ACTB	W4	PGE2	well_3	F006	338	X/Y distribution	7.023092	6.98981	1.08877
ACTB	W4	PGE2	well_4	F001	322	X/Y distribution	6.792681	6.711128	0.977133
ACTB	W4	PGE2	well_4	F002	230	X/Y distribution	7.233971	7.229412	0.820648
ACTB	W4	PGE2	well_4	F003	289	X/Y distribution	6.830043	6.648267	1.343225
ACTB	W4	PGE2	well_4	F004	120	X/Y distribution	6.964617	6.798128	1.225758
ACTB	W4	PGE2	well_4	F005	239	X/Y distribution	7.061389	7.003921	1.084745
ACTB	W4	PGE2	well_4	F006	154	X/Y distribution	7.430656	7.412572	1.168189
ACTB	W4	PGE2	well_5	F001	95	X/Y distribution	7.416974	7.423868	1.067549
ACTB	W4	PGE2	well_5	F002	188	X/Y distribution	7.486496	7.391008	1.257229
ACTB	W4	PGE2	well_5	F003	264	X/Y distribution	7.302948	7.098272	1.119332
ACTB	W4	PGE2	well_5	F004	204	X/Y distribution	8.066731	7.966291	1.273113
ACTB	W4	PGE2	well_5	F005	255	X/Y distribution	7.523268	7.346901	1.166275
ACTB	W4	PGE2	well_5	F006	207	X/Y distribution	7.827269	7.696426	1.255248
ACTB	W1	HPI4	well_1	F001	11	Z distribution	-0.13711	-0.13901	0.305596
ACTB	W1	HPI4	well_1	F002	37	Z distribution	0.104508	0.132961	0.170695
ACTB	W1	HPI4	well_1	F003	50	Z distribution	0.065772	0.10247	0.166197
ACTB	W1	HPI4	well_1	F004	18	Z distribution	0.201602	0.211542	0.172399
ACTB	W1	HPI4	well_1	F005	14	Z distribution	-0.15507	-0.15646	0.24155
ACTB	W1	HPI4	well_1	F006	39	Z distribution	0.042714	0.119605	0.255896
ACTB	W1	HPI4	well_2	F001	100	Z distribution	0.043572	0.051383	0.283607
ACTB	W1	HPI4	well_2	F002	60	Z distribution	0.046064	0.075732	0.221993
ACTB	W1	HPI4	well_2	F003	68	Z distribution	0.015104	-0.00478	0.163231
ACTB	W1	HPI4	well_2	F004	33	Z distribution	0.035456	0.055657	0.227512
ACTB	W1	HPI4	well_2	F005	19	Z distribution	-0.02275	0.025312	0.188723
ACTB	W1	HPI4	well_2	F006	46	Z distribution	0.134822	0.174098	0.20362
ACTB	W1	HPI4	well_3	F001	137	Z distribution	-0.03254	0.019238	0.256375

ACTB	W1	HPI4	well_3	F002	23	Z distribution	0.030111	0.108181	0.231498
ACTB	W1	HPI4	well_3	F003	21	Z distribution	-0.09183	-0.10974	0.213543
ACTB	W1	HPI4	well_3	F004	59	Z distribution	-0.07554	0.021244	0.209873
ACTB	W1	HPI4	well_3	F005	58	Z distribution	-0.01336	0.068379	0.231384
ACTB	W1	HPI4	well_3	F006	67	Z distribution	0.068875	0.081341	0.191859
ACTB	W1	HPI4	well_4	F001	46	Z distribution	0.01166	0.00847	0.198139
ACTB	W1	HPI4	well_4	F002	95	Z distribution	-0.04067	-0.00997	0.256091
ACTB	W1	HPI4	well_4	F003	51	Z distribution	0.01935	0.044902	0.202631
ACTB	W1	HPI4	well_4	F004	94	Z distribution	0.061653	0.092188	0.247719
ACTB	W1	HPI4	well_4	F005	34	Z distribution	-0.00313	0.022691	0.147336
ACTB	W1	HPI4	well_4	F006	56	Z distribution	0.052386	0.066358	0.189934
ACTB	W1	HPI4	well_5	F001	85	Z distribution	0.10716	0.133962	0.189617
ACTB	W1	HPI4	well_5	F002	62	Z distribution	0.16688	0.180777	0.199357
ACTB	W1	HPI4	well_5	F003	75	Z distribution	0.08092	0.127337	0.183342
ACTB	W1	HPI4	well_5	F004	61	Z distribution	0.13362	0.156097	0.191876
ACTB	W1	HPI4	well_5	F005	130	Z distribution	0.148019	0.150505	0.207814
ACTB	W1	HPI4	well_5	F006	96	Z distribution	0.041873	0.043729	0.219057
ACTB	W1	PGE2	well_1	F001	35	Z distribution	-0.13817	-0.11979	0.280554
ACTB	W1	PGE2	well_1	F002	17	Z distribution	0.007033	0.100322	0.214447
ACTB	W1	PGE2	well_1	F003	46	Z distribution	0.043883	0.030062	0.187291
ACTB	W1	PGE2	well_1	F004	84	Z distribution	0.000224	0.052178	0.193264
ACTB	W1	PGE2	well_1	F005	130	Z distribution	-0.05571	-0.01609	0.286108
ACTB	W1	PGE2	well_1	F006	74	Z distribution	0.014069	0.043974	0.182062
ACTB	W1	PGE2	well_2	F001	37	Z distribution	0.083503	0.107234	0.184473
ACTB	W1	PGE2	well_2	F002	18	Z distribution	-0.02316	-0.0083	0.201508
ACTB	W1	PGE2	well_2	F003	39	Z distribution	-0.0469	0.020491	0.228854
ACTB	W1	PGE2	well_2	F004	46	Z distribution	0.015473	0.014193	0.156679
ACTB	W1	PGE2	well_2	F005	43	Z distribution	0.079674	0.05171	0.149728
ACTB	W1	PGE2	well_2	F006	48	Z distribution	0.109656	0.159151	0.218883
ACTB	W1	PGE2	well_3	F001	59	Z distribution	0.005687	0.01248	0.187106
ACTB	W1	PGE2	well_3	F002	79	Z distribution	0.015811	0.041935	0.190795
ACTB	W1	PGE2	well_3	F003	31	Z distribution	-0.1188	-0.03587	0.279227
ACTB	W1	PGE2	well_3	F004	29	Z distribution	0.06801	0.111229	0.198728
ACTB	W1	PGE2	well_3	F005	74	Z distribution	-0.03947	0.002975	0.274932
ACTB	W1	PGE2	well_3	F006	90	Z distribution	-0.07702	-0.06208	0.205809
ACTB	W1	PGE2	well_4	F001	31	Z distribution	0.017584	0.06702	0.24332
ACTB	W1	PGE2	well_4	F002	28	Z distribution	0.086877	0.125268	0.179241
ACTB	W1	PGE2	well_4	F003	58	Z distribution	-0.1386	-0.0633	0.278458
ACTB	W1	PGE2	well_4	F004	16	Z distribution	0.038671	0.041395	0.236273
ACTB	W1	PGE2	well_4	F005	19	Z distribution	-0.04695	-0.01785	0.183059
ACTB	W1	PGE2	well_4	F006	9	Z distribution	-0.02018	0.043782	0.287742
ACTB	W1	PGE2	well_5	F001	26	Z distribution	0.136762	0.150761	0.123956
ACTB	W1	PGE2	well_5	F002	31	Z distribution	0.054462	0.12512	0.179389
ACTB	W1	PGE2	well_5	F003	11	Z distribution	-0.09281	-0.06296	0.119255
ACTB	W1	PGE2	well_5	F004	30	Z distribution	0.086945	0.071884	0.135961
ACTB	W1	PGE2	well_5	F005	22	Z distribution	0.021814	0.059462	0.174831
ACTB	W1	PGE2	well_5	F006	60	Z distribution	-0.01838	-0.03635	0.212338
ACTB	W2	HPI4	well_1	F001	1	Z distribution	0.786609	0.786609	NA
ACTB	W2	HPI4	well_1	F002	1	Z distribution	0.076339	0.076339	NA

ACTB	W2	HPI4	well_1	F005	3	Z distribution	0.17123	0.17123	NA
ACTB	W2	HPI4	well_1	F006	3	Z distribution	-0.25516	-0.25516	NA
ACTB	W2	HPI4	well_2	F006	3	Z distribution	-0.07006	-0.07006	NA
ACTB	W2	HPI4	well_3	F001	5	Z distribution	-0.01703	-0.01819	0.090676
ACTB	W2	HPI4	well_3	F002	1	Z distribution	0.084146	0.084146	NA
ACTB	W2	HPI4	well_3	F003	1	Z distribution	-0.03702	-0.03702	NA
ACTB	W2	HPI4	well_3	F006	1	Z distribution	-0.70514	-0.70514	NA
ACTB	W2	HPI4	well_4	F002	1	Z distribution	0.100133	0.100133	NA
ACTB	W2	HPI4	well_4	F006	3	Z distribution	-0.14178	-0.14178	NA
ACTB	W2	HPI4	well_5	F001	14	Z distribution	-0.24465	-0.19315	0.230202
ACTB	W2	HPI4	well_5	F003	1	Z distribution	-0.01284	-0.01284	NA
ACTB	W2	HPI4	well_5	F004	3	Z distribution	-0.04135	-0.04135	NA
ACTB	W2	HPI4	well_5	F006	16	Z distribution	-0.08117	-0.07597	0.156251
ACTB	W2	PGE2	well_1	F001	126	Z distribution	0.080728	0.093093	0.284899
ACTB	W2	PGE2	well_1	F002	139	Z distribution	0.073208	0.078556	0.290914
ACTB	W2	PGE2	well_1	F003	180	Z distribution	0.038905	0.051228	0.255991
ACTB	W2	PGE2	well_1	F004	97	Z distribution	0.02293	0.019748	0.235388
ACTB	W2	PGE2	well_1	F005	101	Z distribution	0.104425	0.09648	0.23214
ACTB	W2	PGE2	well_1	F006	72	Z distribution	0.031824	0.017283	0.233951
ACTB	W2	PGE2	well_2	F001	59	Z distribution	0.147872	0.137002	0.229208
ACTB	W2	PGE2	well_2	F002	91	Z distribution	-0.07291	-0.06652	0.308107
ACTB	W2	PGE2	well_2	F003	142	Z distribution	0.110387	0.144888	0.308833
ACTB	W2	PGE2	well_2	F004	60	Z distribution	0.084321	0.082113	0.236693
ACTB	W2	PGE2	well_2	F005	36	Z distribution	0.055618	0.087969	0.238113
ACTB	W2	PGE2	well_2	F006	123	Z distribution	0.033527	0.030117	0.246573
ACTB	W2	PGE2	well_3	F001	67	Z distribution	0.062794	0.088433	0.259452
ACTB	W2	PGE2	well_3	F002	90	Z distribution	0.109294	0.140822	0.299891
ACTB	W2	PGE2	well_3	F003	99	Z distribution	0.173114	0.187073	0.286902
ACTB	W2	PGE2	well_3	F004	20	Z distribution	0.079435	0.109923	0.166527
ACTB	W2	PGE2	well_3	F005	83	Z distribution	0.071862	0.072225	0.216788
ACTB	W2	PGE2	well_3	F006	102	Z distribution	0.083821	0.082759	0.198279
ACTB	W2	PGE2	well_4	F001	36	Z distribution	0.091613	0.083235	0.169631
ACTB	W2	PGE2	well_4	F002	38	Z distribution	0.125904	0.108521	0.273309
ACTB	W2	PGE2	well_4	F003	38	Z distribution	0.11138	0.082133	0.181266
ACTB	W2	PGE2	well_4	F004	53	Z distribution	0.11595	0.126977	0.24417
ACTB	W2	PGE2	well_4	F005	67	Z distribution	0.147617	0.158443	0.244587
ACTB	W2	PGE2	well_4	F006	56	Z distribution	0.037528	0.065425	0.215562
ACTB	W2	PGE2	well_5	F001	15	Z distribution	0.016675	0.045423	0.189251
ACTB	W2	PGE2	well_5	F002	21	Z distribution	0.029565	0.031682	0.223401
ACTB	W2	PGE2	well_5	F003	20	Z distribution	0.090282	0.085801	0.17903
ACTB	W2	PGE2	well_5	F004	36	Z distribution	-0.07304	-0.09367	0.260715
ACTB	W2	PGE2	well_5	F005	29	Z distribution	0.09958	0.090562	0.156816
ACTB	W2	PGE2	well_5	F006	35	Z distribution	0.073113	0.13443	0.186145
ACTB	W3	HPI4	well_1	F001	43	Z distribution	-0.0558	-0.00654	0.245408
ACTB	W3	HPI4	well_1	F002	36	Z distribution	-0.16408	-0.12991	0.229785
ACTB	W3	HPI4	well_1	F003	78	Z distribution	-0.19922	-0.12278	0.24916
ACTB	W3	HPI4	well_1	F004	122	Z distribution	-0.11964	-0.09569	0.280005
ACTB	W3	HPI4	well_1	F005	68	Z distribution	-0.08165	-0.07568	0.131376
ACTB	W3	HPI4	well_1	F006	59	Z distribution	-0.14816	-0.13508	0.19839

ACTB	W3	HPI4	well_2	F001	86	Z distribution	0.016019	0.034128	0.166325
ACTB	W3	HPI4	well_2	F002	58	Z distribution	-0.09625	-0.05247	0.183088
ACTB	W3	HPI4	well_2	F003	52	Z distribution	-0.08345	-0.06748	0.154714
ACTB	W3	HPI4	well_2	F004	55	Z distribution	0.063698	0.090036	0.204953
ACTB	W3	HPI4	well_2	F005	111	Z distribution	-0.08479	-0.06147	0.17993
ACTB	W3	HPI4	well_2	F006	95	Z distribution	-0.06365	-0.06335	0.196142
ACTB	W3	HPI4	well_3	F001	32	Z distribution	0.087884	0.100103	0.12776
ACTB	W3	HPI4	well_3	F002	37	Z distribution	-0.23299	-0.11836	0.301107
ACTB	W3	HPI4	well_3	F003	6	Z distribution	-0.20875	-0.26347	0.165607
ACTB	W3	HPI4	well_3	F004	37	Z distribution	-0.14798	-0.13758	0.274862
ACTB	W3	HPI4	well_3	F005	12	Z distribution	-0.18675	-0.18529	0.132885
ACTB	W3	HPI4	well_3	F006	59	Z distribution	-0.05782	-0.04656	0.129204
ACTB	W3	HPI4	well_4	F001	103	Z distribution	0.077104	0.11798	0.23664
ACTB	W3	HPI4	well_4	F002	91	Z distribution	-0.08716	-0.06564	0.254419
ACTB	W3	HPI4	well_4	F003	21	Z distribution	-0.20672	-0.22404	0.271757
ACTB	W3	HPI4	well_4	F004	9	Z distribution	-0.15194	-0.15904	0.056771
ACTB	W3	HPI4	well_4	F005	58	Z distribution	0.002177	0.008471	0.16523
ACTB	W3	HPI4	well_4	F006	70	Z distribution	-0.08289	-0.04609	0.207186
ACTB	W3	HPI4	well_5	F001	20	Z distribution	-0.17259	-0.14587	0.212675
ACTB	W3	HPI4	well_5	F002	8	Z distribution	-0.03587	0.0013	0.211046
ACTB	W3	HPI4	well_5	F003	4	Z distribution	0.067474	0.067474	0.08308
ACTB	W3	HPI4	well_5	F004	5	Z distribution	-0.09333	-0.02163	0.152083
ACTB	W3	HPI4	well_5	F005	26	Z distribution	-0.08362	-0.03308	0.170322
ACTB	W3	HPI4	well_5	F006	18	Z distribution	-0.05678	-0.02159	0.117317
ACTB	W3	PGE2	well_1	F001	162	Z distribution	-0.0015	-0.00168	0.305571
ACTB	W3	PGE2	well_1	F002	232	Z distribution	-0.07506	-0.04309	0.321053
ACTB	W3	PGE2	well_1	F003	294	Z distribution	-0.0287	-0.03671	0.253225
ACTB	W3	PGE2	well_1	F004	58	Z distribution	0.056058	0.043324	0.222901
ACTB	W3	PGE2	well_1	F005	80	Z distribution	0.122481	0.121292	0.249927
ACTB	W3	PGE2	well_1	F006	281	Z distribution	-0.20334	-0.14178	0.401846
ACTB	W3	PGE2	well_2	F001	113	Z distribution	0.135299	0.106764	0.283313
ACTB	W3	PGE2	well_2	F002	160	Z distribution	0.091445	0.151025	0.313566
ACTB	W3	PGE2	well_2	F003	190	Z distribution	0.208912	0.200586	0.21755
ACTB	W3	PGE2	well_2	F004	155	Z distribution	0.224318	0.209795	0.273518
ACTB	W3	PGE2	well_2	F005	288	Z distribution	-0.18528	-0.15648	0.348082
ACTB	W3	PGE2	well_2	F006	191	Z distribution	0.096488	0.092439	0.270332
ACTB	W3	PGE2	well_3	F001	110	Z distribution	0.026781	0.040202	0.28006
ACTB	W3	PGE2	well_3	F002	211	Z distribution	0.073619	0.071061	0.198101
ACTB	W3	PGE2	well_3	F003	201	Z distribution	0.183848	0.166408	0.254115
ACTB	W3	PGE2	well_3	F004	83	Z distribution	0.268026	0.256985	0.272995
ACTB	W3	PGE2	well_3	F005	217	Z distribution	0.000282	0.005014	0.257785
ACTB	W3	PGE2	well_3	F006	227	Z distribution	-0.12318	-0.06376	0.311582
ACTB	W3	PGE2	well_4	F001	176	Z distribution	0.118057	0.115058	0.252709
ACTB	W3	PGE2	well_4	F002	159	Z distribution	-0.05952	-0.00109	0.336556
ACTB	W3	PGE2	well_4	F003	251	Z distribution	0.087335	0.115161	0.305432
ACTB	W3	PGE2	well_4	F004	161	Z distribution	0.171262	0.162601	0.265428
ACTB	W3	PGE2	well_4	F005	196	Z distribution	0.070171	0.085417	0.231565
ACTB	W3	PGE2	well_4	F006	196	Z distribution	0.061139	0.060069	0.251401
ACTB	W3	PGE2	well_5	F001	105	Z distribution	0.107736	0.105163	0.187071

ACTB	W3	PGE2	well_5	F002	132	Z distribution	0.027877	0.062951	0.32236
ACTB	W3	PGE2	well_5	F003	223	Z distribution	0.148201	0.117842	0.222746
ACTB	W3	PGE2	well_5	F004	102	Z distribution	0.29569	0.288723	0.172633
ACTB	W3	PGE2	well_5	F005	222	Z distribution	0.273235	0.267159	0.288435
ACTB	W3	PGE2	well_5	F006	204	Z distribution	-0.05052	-0.0346	0.243532
ACTB	W4	HPI4	well_1	F001	36	Z distribution	0.236829	0.261793	0.16291
ACTB	W4	HPI4	well_1	F002	4	Z distribution	0.071613	0.071613	0.245607
ACTB	W4	HPI4	well_1	F003	12	Z distribution	0.067601	0.062846	0.157319
ACTB	W4	HPI4	well_1	F004	23	Z distribution	-0.00296	-0.01234	0.114459
ACTB	W4	HPI4	well_1	F005	39	Z distribution	-0.05738	-0.03948	0.203308
ACTB	W4	HPI4	well_1	F006	27	Z distribution	0.124582	0.184034	0.247886
ACTB	W4	HPI4	well_2	F001	25	Z distribution	-0.04202	0.103386	0.398444
ACTB	W4	HPI4	well_2	F003	4	Z distribution	0.223474	0.223474	0.069926
ACTB	W4	HPI4	well_2	F004	10	Z distribution	0.04899	0.144596	0.210573
ACTB	W4	HPI4	well_2	F005	38	Z distribution	0.052277	0.056764	0.278263
ACTB	W4	HPI4	well_2	F006	13	Z distribution	0.136262	0.12767	0.166339
ACTB	W4	HPI4	well_3	F001	8	Z distribution	-0.04201	-0.00266	0.144812
ACTB	W4	HPI4	well_3	F002	3	Z distribution	-0.72869	-0.72869	NA
ACTB	W4	HPI4	well_3	F003	1	Z distribution	-0.25731	-0.25731	NA
ACTB	W4	HPI4	well_3	F004	1	Z distribution	-0.32301	-0.32301	NA
ACTB	W4	HPI4	well_3	F006	5	Z distribution	-0.46403	-0.43756	0.057117
ACTB	W4	HPI4	well_4	F001	14	Z distribution	0.144321	0.165862	0.175282
ACTB	W4	HPI4	well_4	F002	23	Z distribution	0.041568	0.163493	0.307667
ACTB	W4	HPI4	well_4	F003	3	Z distribution	-0.49108	-0.49108	NA
ACTB	W4	HPI4	well_4	F004	6	Z distribution	-0.13739	-0.02771	0.351545
ACTB	W4	HPI4	well_4	F005	19	Z distribution	0.074494	0.117496	0.233373
ACTB	W4	HPI4	well_4	F006	4	Z distribution	0.171443	0.171443	0.084982
ACTB	W4	HPI4	well_5	F001	3	Z distribution	0.025579	0.025579	NA
ACTB	W4	HPI4	well_5	F002	8	Z distribution	-0.46798	-0.5105	0.260384
ACTB	W4	HPI4	well_5	F003	1	Z distribution	0.200311	0.200311	NA
ACTB	W4	HPI4	well_5	F004	5	Z distribution	-0.59176	-0.69911	0.199878
ACTB	W4	HPI4	well_5	F005	3	Z distribution	-1.03194	-1.03194	NA
ACTB	W4	HPI4	well_5	F006	17	Z distribution	-0.15986	-0.31471	0.35904
ACTB	W4	PGE2	well_1	F001	231	Z distribution	-0.02172	-0.0071	0.341829
ACTB	W4	PGE2	well_1	F002	289	Z distribution	-0.10109	-0.06627	0.384131
ACTB	W4	PGE2	well_1	F003	254	Z distribution	0.004262	0.036846	0.38514
ACTB	W4	PGE2	well_1	F004	280	Z distribution	0.056245	0.073286	0.403146
ACTB	W4	PGE2	well_1	F005	291	Z distribution	-0.02991	-0.0171	0.32519
ACTB	W4	PGE2	well_1	F006	318	Z distribution	-0.02784	0.008802	0.40828
ACTB	W4	PGE2	well_2	F001	235	Z distribution	-0.03746	-0.03581	0.385312
ACTB	W4	PGE2	well_2	F002	265	Z distribution	-0.05434	-0.01826	0.40652
ACTB	W4	PGE2	well_2	F003	228	Z distribution	-0.20191	-0.16478	0.400957
ACTB	W4	PGE2	well_2	F004	337	Z distribution	-0.22013	-0.20196	0.435174
ACTB	W4	PGE2	well_2	F005	295	Z distribution	-0.29493	-0.24202	0.390279
ACTB	W4	PGE2	well_2	F006	285	Z distribution	0.084539	0.080857	0.394977
ACTB	W4	PGE2	well_3	F001	297	Z distribution	-0.2642	-0.19501	0.430662
ACTB	W4	PGE2	well_3	F002	253	Z distribution	-0.34596	-0.32226	0.405028
ACTB	W4	PGE2	well_3	F003	245	Z distribution	-0.07376	-0.04976	0.354064
ACTB	W4	PGE2	well_3	F004	286	Z distribution	0.061924	0.068474	0.319692

ACTB	W4	PGE2	well_3	F005	248	Z distribution	-0.34925	-0.32102	0.509804
ACTB	W4	PGE2	well_3	F006	338	Z distribution	-0.04574	-0.01049	0.443883
ACTB	W4	PGE2	well_4	F001	322	Z distribution	-0.16118	-0.09871	0.452068
ACTB	W4	PGE2	well_4	F002	230	Z distribution	-0.32454	-0.26168	0.503532
ACTB	W4	PGE2	well_4	F003	289	Z distribution	-0.06268	-0.06228	0.324198
ACTB	W4	PGE2	well_4	F004	120	Z distribution	-0.13751	-0.10065	0.358187
ACTB	W4	PGE2	well_4	F005	239	Z distribution	-0.32191	-0.26494	0.458147
ACTB	W4	PGE2	well_4	F006	154	Z distribution	-0.35595	-0.30167	0.502615
ACTB	W4	PGE2	well_5	F001	95	Z distribution	0.104344	0.087689	0.26354
ACTB	W4	PGE2	well_5	F002	188	Z distribution	0.095534	0.068602	0.289915
ACTB	W4	PGE2	well_5	F003	264	Z distribution	-0.03924	-0.01067	0.319885
ACTB	W4	PGE2	well_5	F004	204	Z distribution	0.096877	0.09197	0.256054
ACTB	W4	PGE2	well_5	F005	255	Z distribution	0.048797	0.096934	0.320286
ACTB	W4	PGE2	well_5	F006	207	Z distribution	-0.04956	-0.02011	0.324243
MYH10	W1	HPI4	well_1	F001	148	Avg. Volume	1.278495	1.213337	0.482081
MYH10	W1	HPI4	well_1	F002	167	Avg. Volume	1.263494	1.214865	0.368837
MYH10	W1	HPI4	well_1	F003	161	Avg. Volume	1.262601	1.262064	0.439768
MYH10	W1	HPI4	well_1	F004	280	Avg. Volume	1.710755	1.685441	0.617352
MYH10	W1	HPI4	well_1	F005	180	Avg. Volume	1.210795	1.197203	0.355128
MYH10	W1	HPI4	well_1	F006	104	Avg. Volume	1.050675	0.988459	0.334249
MYH10	W1	HPI4	well_2	F001	124	Avg. Volume	1.193262	1.082377	0.443811
MYH10	W1	HPI4	well_2	F002	150	Avg. Volume	1.339982	1.290193	0.399149
MYH10	W1	HPI4	well_2	F003	192	Avg. Volume	1.166999	1.117324	0.448548
MYH10	W1	HPI4	well_2	F004	203	Avg. Volume	1.377677	1.374077	0.398997
MYH10	W1	HPI4	well_2	F005	166	Avg. Volume	1.350272	1.312412	0.358983
MYH10	W1	HPI4	well_2	F006	129	Avg. Volume	1.326624	1.227597	0.487478
MYH10	W1	HPI4	well_3	F001	118	Avg. Volume	1.17591	1.137544	0.407685
MYH10	W1	HPI4	well_3	F002	194	Avg. Volume	1.372341	1.384581	0.445015
MYH10	W1	HPI4	well_3	F003	115	Avg. Volume	1.244043	1.230806	0.429646
MYH10	W1	HPI4	well_3	F004	98	Avg. Volume	1.124746	1.083849	0.337073
MYH10	W1	HPI4	well_3	F005	123	Avg. Volume	1.319325	1.322949	0.41024
MYH10	W1	HPI4	well_3	F006	212	Avg. Volume	1.334813	1.302097	0.470654
MYH10	W1	HPI4	well_4	F001	81	Avg. Volume	1.361774	1.419986	0.380106
MYH10	W1	HPI4	well_4	F002	266	Avg. Volume	1.436766	1.318738	0.661863
MYH10	W1	HPI4	well_4	F003	158	Avg. Volume	1.063867	1.042806	0.342824
MYH10	W1	HPI4	well_4	F004	186	Avg. Volume	1.263296	1.23715	0.374289
MYH10	W1	HPI4	well_4	F005	126	Avg. Volume	1.40214	1.382406	0.405393
MYH10	W1	HPI4	well_4	F006	114	Avg. Volume	1.260476	1.199326	0.449151
MYH10	W1	HPI4	well_5	F001	167	Avg. Volume	0.950573	0.911472	0.389989
MYH10	W1	HPI4	well_5	F002	143	Avg. Volume	1.292222	1.265392	0.384428
MYH10	W1	HPI4	well_5	F003	222	Avg. Volume	1.336847	1.286043	0.487206
MYH10	W1	HPI4	well_5	F004	184	Avg. Volume	1.302017	1.280985	0.39456
MYH10	W1	HPI4	well_5	F005	164	Avg. Volume	1.477575	1.484044	0.415361
MYH10	W1	HPI4	well_5	F006	148	Avg. Volume	1.083672	1.029499	0.352433
MYH10	W1	PGE2	well_1	F001	145	Avg. Volume	1.476207	1.436657	0.479088
MYH10	W1	PGE2	well_1	F002	91	Avg. Volume	1.291156	1.254153	0.361572
MYH10	W1	PGE2	well_1	F003	250	Avg. Volume	1.180189	1.148247	0.42588
MYH10	W1	PGE2	well_1	F004	393	Avg. Volume	1.75064	1.70203	0.586491
MYH10	W1	PGE2	well_1	F005	286	Avg. Volume	1.461163	1.453429	0.471176

MYH10	W1	PGE2	well_1	F006	145	Avg. Volume	1.045821	1.068831	0.385226
MYH10	W1	PGE2	well_2	F001	219	Avg. Volume	1.270976	1.257172	0.47379
MYH10	W1	PGE2	well_2	F002	184	Avg. Volume	1.226215	1.220492	0.503776
MYH10	W1	PGE2	well_2	F003	177	Avg. Volume	1.112653	1.063585	0.403893
MYH10	W1	PGE2	well_2	F004	158	Avg. Volume	1.144212	1.07057	0.395526
MYH10	W1	PGE2	well_2	F005	232	Avg. Volume	1.385675	1.328064	0.482401
MYH10	W1	PGE2	well_2	F006	221	Avg. Volume	1.239483	1.213011	0.442387
MYH10	W1	PGE2	well_3	F001	129	Avg. Volume	1.074061	1.075145	0.322075
MYH10	W1	PGE2	well_3	F002	131	Avg. Volume	1.183649	1.149766	0.388575
MYH10	W1	PGE2	well_3	F003	222	Avg. Volume	1.526843	1.486196	0.561548
MYH10	W1	PGE2	well_3	F004	175	Avg. Volume	1.259182	1.269065	0.363681
MYH10	W1	PGE2	well_3	F005	132	Avg. Volume	1.183485	1.141949	0.357266
MYH10	W1	PGE2	well_3	F006	147	Avg. Volume	1.247591	1.208819	0.441658
MYH10	W1	PGE2	well_4	F001	129	Avg. Volume	1.097537	1.061795	0.38461
MYH10	W1	PGE2	well_4	F002	210	Avg. Volume	1.316906	1.324518	0.438792
MYH10	W1	PGE2	well_4	F003	116	Avg. Volume	1.255798	1.219394	0.406244
MYH10	W1	PGE2	well_4	F004	99	Avg. Volume	1.161765	1.11367	0.345268
MYH10	W1	PGE2	well_4	F005	153	Avg. Volume	1.393564	1.382362	0.433722
MYH10	W1	PGE2	well_4	F006	223	Avg. Volume	1.263944	1.236693	0.441626
MYH10	W1	PGE2	well_5	F001	105	Avg. Volume	1.320737	1.334268	0.354232
MYH10	W1	PGE2	well_5	F002	132	Avg. Volume	1.258282	1.268939	0.434081
MYH10	W1	PGE2	well_5	F003	233	Avg. Volume	1.18805	1.116445	0.489039
MYH10	W1	PGE2	well_5	F004	179	Avg. Volume	1.226526	1.277036	0.429763
MYH10	W1	PGE2	well_5	F005	190	Avg. Volume	1.269779	1.214365	0.432672
MYH10	W1	PGE2	well_5	F006	168	Avg. Volume	1.339186	1.358939	0.44841
MYH10	W2	HPI4	well_1	F001	163	Avg. Volume	1.015173	0.911421	0.376308
MYH10	W2	HPI4	well_1	F002	160	Avg. Volume	1.279057	1.203932	0.453473
MYH10	W2	HPI4	well_1	F003	150	Avg. Volume	1.023415	1.01081	0.302424
MYH10	W2	HPI4	well_1	F004	250	Avg. Volume	0.967006	0.914938	0.349118
MYH10	W2	HPI4	well_1	F005	219	Avg. Volume	1.00908	0.978148	0.336279
MYH10	W2	HPI4	well_1	F006	165	Avg. Volume	1.159265	1.095752	0.402168
MYH10	W2	HPI4	well_2	F001	156	Avg. Volume	1.38693	1.197309	0.632239
MYH10	W2	HPI4	well_2	F002	155	Avg. Volume	1.090029	1.0447	0.420054
MYH10	W2	HPI4	well_2	F003	191	Avg. Volume	1.079846	1.014814	0.424335
MYH10	W2	HPI4	well_2	F004	180	Avg. Volume	1.189867	1.08197	0.507275
MYH10	W2	HPI4	well_2	F005	148	Avg. Volume	1.257809	1.234842	0.464965
MYH10	W2	HPI4	well_2	F006	133	Avg. Volume	1.24076	1.089661	0.610943
MYH10	W2	HPI4	well_3	F001	100	Avg. Volume	1.056123	1.015717	0.436577
MYH10	W2	HPI4	well_3	F002	212	Avg. Volume	1.184263	1.048259	0.532535
MYH10	W2	HPI4	well_3	F003	218	Avg. Volume	1.239827	1.013235	0.711226
MYH10	W2	HPI4	well_3	F004	125	Avg. Volume	0.979446	0.80588	0.465934
MYH10	W2	HPI4	well_3	F005	183	Avg. Volume	1.482387	1.431586	0.468469
MYH10	W2	HPI4	well_3	F006	113	Avg. Volume	1.284893	1.070437	0.636881
MYH10	W2	HPI4	well_4	F001	170	Avg. Volume	1.123874	1.010001	0.524665
MYH10	W2	HPI4	well_4	F002	209	Avg. Volume	1.357868	1.162254	0.661722
MYH10	W2	HPI4	well_4	F003	235	Avg. Volume	1.25274	1.209476	0.425914
MYH10	W2	HPI4	well_4	F004	333	Avg. Volume	1.19296	1.133531	0.445881
MYH10	W2	HPI4	well_4	F005	198	Avg. Volume	1.121507	1.069018	0.385077
MYH10	W2	HPI4	well_4	F006	91	Avg. Volume	1.202246	1.128638	0.421348

MYH10	W2	HPI4	well_5	F001	121	Avg. Volume	0.972006	0.854551	0.422574
MYH10	W2	HPI4	well_5	F002	156	Avg. Volume	1.137672	1.024282	0.507072
MYH10	W2	HPI4	well_5	F003	252	Avg. Volume	1.27999	1.237216	0.447565
MYH10	W2	HPI4	well_5	F004	306	Avg. Volume	1.237092	1.133873	0.544012
MYH10	W2	HPI4	well_5	F005	250	Avg. Volume	1.163938	1.049568	0.463603
MYH10	W2	HPI4	well_5	F006	160	Avg. Volume	0.877829	0.782382	0.422152
MYH10	W2	PGE2	well_1	F001	49	Avg. Volume	1.965293	1.81717	0.681524
MYH10	W2	PGE2	well_1	F002	70	Avg. Volume	2.010487	1.8843	0.653175
MYH10	W2	PGE2	well_1	F003	149	Avg. Volume	1.889927	1.825533	0.711432
MYH10	W2	PGE2	well_1	F004	318	Avg. Volume	1.993788	1.891558	0.711145
MYH10	W2	PGE2	well_1	F005	207	Avg. Volume	2.224666	2.134016	0.654118
MYH10	W2	PGE2	well_1	F006	103	Avg. Volume	1.949123	1.904338	0.52745
MYH10	W2	PGE2	well_2	F001	257	Avg. Volume	1.936053	1.842718	0.599798
MYH10	W2	PGE2	well_2	F002	384	Avg. Volume	1.450218	1.362395	0.520473
MYH10	W2	PGE2	well_2	F003	118	Avg. Volume	2.043712	2.075069	0.60424
MYH10	W2	PGE2	well_2	F004	254	Avg. Volume	1.743183	1.643236	0.676251
MYH10	W2	PGE2	well_2	F005	42	Avg. Volume	1.393367	1.322129	0.364618
MYH10	W2	PGE2	well_3	F001	261	Avg. Volume	2.104121	1.961398	0.827675
MYH10	W2	PGE2	well_3	F002	398	Avg. Volume	1.667122	1.612017	0.550308
MYH10	W2	PGE2	well_3	F003	206	Avg. Volume	2.111727	2.042262	0.625137
MYH10	W2	PGE2	well_3	F004	360	Avg. Volume	1.802356	1.75107	0.706225
MYH10	W2	PGE2	well_3	F005	277	Avg. Volume	2.072119	2.090572	0.586127
MYH10	W2	PGE2	well_3	F006	218	Avg. Volume	2.058657	1.963865	0.672383
MYH10	W2	PGE2	well_4	F001	175	Avg. Volume	2.095451	2.0475	0.504558
MYH10	W2	PGE2	well_4	F002	320	Avg. Volume	2.360704	2.328768	0.753463
MYH10	W2	PGE2	well_4	F003	319	Avg. Volume	2.066799	2.053218	0.596104
MYH10	W2	PGE2	well_4	F004	356	Avg. Volume	1.713004	1.652522	0.613142
MYH10	W2	PGE2	well_4	F005	347	Avg. Volume	2.301011	2.22467	0.711259
MYH10	W2	PGE2	well_4	F006	382	Avg. Volume	1.775093	1.728442	0.623624
MYH10	W2	PGE2	well_5	F001	173	Avg. Volume	1.992928	1.960266	0.700477
MYH10	W2	PGE2	well_5	F002	335	Avg. Volume	2.376991	2.36033	0.723554
MYH10	W2	PGE2	well_5	F003	391	Avg. Volume	2.186215	2.153153	0.591395
MYH10	W2	PGE2	well_5	F004	414	Avg. Volume	2.514468	2.465228	0.633156
MYH10	W2	PGE2	well_5	F005	292	Avg. Volume	1.631205	1.580333	0.498315
MYH10	W2	PGE2	well_5	F006	329	Avg. Volume	2.348633	2.261065	0.778391
MYH10	W3	HPI4	well_1	F001	139	Avg. Volume	0.739494	0.723752	0.251761
MYH10	W3	HPI4	well_1	F002	206	Avg. Volume	0.984259	0.963148	0.336992
MYH10	W3	HPI4	well_1	F003	196	Avg. Volume	1.212899	1.193224	0.390747
MYH10	W3	HPI4	well_1	F004	181	Avg. Volume	1.034349	1.004552	0.299609
MYH10	W3	HPI4	well_1	F005	110	Avg. Volume	1.067994	1.02599	0.320286
MYH10	W3	HPI4	well_1	F006	154	Avg. Volume	1.29645	1.258333	0.439845
MYH10	W3	HPI4	well_2	F001	97	Avg. Volume	0.883229	0.813467	0.353879
MYH10	W3	HPI4	well_2	F002	182	Avg. Volume	1.07436	1.033236	0.361102
MYH10	W3	HPI4	well_2	F003	163	Avg. Volume	1.272773	1.244028	0.395072
MYH10	W3	HPI4	well_2	F004	189	Avg. Volume	1.123692	1.113776	0.35826
MYH10	W3	HPI4	well_2	F005	267	Avg. Volume	1.018183	0.95632	0.396679
MYH10	W3	HPI4	well_2	F006	122	Avg. Volume	1.193649	1.157403	0.384878
MYH10	W3	HPI4	well_3	F001	60	Avg. Volume	0.929891	0.82729	0.430741
MYH10	W3	HPI4	well_3	F002	164	Avg. Volume	1.069436	1.042928	0.350026

MYH10	W3	HPI4	well_3	F003	203	Avg. Volume	1.048275	0.971389	0.388001
MYH10	W3	HPI4	well_3	F004	228	Avg. Volume	1.213717	1.19632	0.409122
MYH10	W3	HPI4	well_3	F005	341	Avg. Volume	1.20099	1.14705	0.502527
MYH10	W3	HPI4	well_3	F006	131	Avg. Volume	0.883057	0.864438	0.317912
MYH10	W3	HPI4	well_4	F001	99	Avg. Volume	0.715277	0.666133	0.208048
MYH10	W3	HPI4	well_4	F002	175	Avg. Volume	1.063325	0.965521	0.333565
MYH10	W3	HPI4	well_4	F003	152	Avg. Volume	1.02346	0.95124	0.46261
MYH10	W3	HPI4	well_4	F004	188	Avg. Volume	1.158668	1.107799	0.432929
MYH10	W3	HPI4	well_4	F005	166	Avg. Volume	1.445635	1.526502	0.523119
MYH10	W3	HPI4	well_4	F006	78	Avg. Volume	1.321281	1.378052	0.387723
MYH10	W3	HPI4	well_5	F001	62	Avg. Volume	1.216512	1.214553	0.373234
MYH10	W3	HPI4	well_5	F002	128	Avg. Volume	1.146988	1.137355	0.367725
MYH10	W3	HPI4	well_5	F003	105	Avg. Volume	1.305828	1.270345	0.394278
MYH10	W3	HPI4	well_5	F004	63	Avg. Volume	0.910569	0.824104	0.340241
MYH10	W3	HPI4	well_5	F005	179	Avg. Volume	0.854961	0.778061	0.382919
MYH10	W3	HPI4	well_5	F006	169	Avg. Volume	1.083502	1.030583	0.437329
MYH10	W3	PGE2	well_1	F001	354	Avg. Volume	2.199947	2.211468	0.569357
MYH10	W3	PGE2	well_1	F002	355	Avg. Volume	2.362708	2.310738	0.666318
MYH10	W3	PGE2	well_1	F003	397	Avg. Volume	2.495162	2.502553	0.716611
MYH10	W3	PGE2	well_1	F004	275	Avg. Volume	2.098988	1.98542	0.653409
MYH10	W3	PGE2	well_1	F005	227	Avg. Volume	1.924772	1.916804	0.616401
MYH10	W3	PGE2	well_2	F001	271	Avg. Volume	2.246018	2.16479	0.635883
MYH10	W3	PGE2	well_2	F002	245	Avg. Volume	2.281741	2.246292	0.571252
MYH10	W3	PGE2	well_2	F003	364	Avg. Volume	2.176047	2.143704	0.684195
MYH10	W3	PGE2	well_2	F004	225	Avg. Volume	2.25624	2.177129	0.538044
MYH10	W3	PGE2	well_2	F005	358	Avg. Volume	1.783806	1.710839	0.621788
MYH10	W3	PGE2	well_2	F006	351	Avg. Volume	2.363131	2.336045	0.685518
MYH10	W3	PGE2	well_3	F001	250	Avg. Volume	2.104612	2.072777	0.689542
MYH10	W3	PGE2	well_3	F002	124	Avg. Volume	2.045439	2.039756	0.54693
MYH10	W3	PGE2	well_3	F003	255	Avg. Volume	1.844558	1.772508	0.542258
MYH10	W3	PGE2	well_3	F004	171	Avg. Volume	2.078091	2.016949	0.73743
MYH10	W3	PGE2	well_3	F005	312	Avg. Volume	2.310421	2.197202	0.747201
MYH10	W3	PGE2	well_3	F006	265	Avg. Volume	2.27742	2.264243	0.642881
MYH10	W3	PGE2	well_4	F001	110	Avg. Volume	2.241992	2.257459	0.567616
MYH10	W3	PGE2	well_4	F002	178	Avg. Volume	2.182964	2.047785	0.629852
MYH10	W3	PGE2	well_4	F003	273	Avg. Volume	2.41279	2.360829	0.68636
MYH10	W3	PGE2	well_4	F004	73	Avg. Volume	2.127037	2.159714	0.590537
MYH10	W3	PGE2	well_4	F005	155	Avg. Volume	2.21297	2.113861	0.586131
MYH10	W3	PGE2	well_4	F006	390	Avg. Volume	2.143376	2.096069	0.694197
MYH10	W3	PGE2	well_5	F001	252	Avg. Volume	2.19618	2.156691	0.79388
MYH10	W3	PGE2	well_5	F002	254	Avg. Volume	2.293589	2.290215	0.73973
MYH10	W3	PGE2	well_5	F003	338	Avg. Volume	2.051424	1.969995	0.778426
MYH10	W3	PGE2	well_5	F004	200	Avg. Volume	2.332189	2.242809	0.550276
MYH10	W3	PGE2	well_5	F005	285	Avg. Volume	1.864349	1.830833	0.66809
MYH10	W3	PGE2	well_5	F006	198	Avg. Volume	2.00093	1.981519	0.56166
MYH10	W4	HPI4	well_1	F001	104	Avg. Volume	1.195465	1.187013	0.346768
MYH10	W4	HPI4	well_1	F002	93	Avg. Volume	1.173861	1.120609	0.396387
MYH10	W4	HPI4	well_1	F003	177	Avg. Volume	1.06746	1.030719	0.347076
MYH10	W4	HPI4	well_1	F004	250	Avg. Volume	1.304618	1.236247	0.502581

MYH10	W4	HPI4	well_1	F005	205	Avg. Volume	1.103541	1.027225	0.418696
MYH10	W4	HPI4	well_1	F006	174	Avg. Volume	1.341217	1.380746	0.415439
MYH10	W4	HPI4	well_2	F001	48	Avg. Volume	0.829943	0.733779	0.277701
MYH10	W4	HPI4	well_2	F002	58	Avg. Volume	0.833045	0.743568	0.315772
MYH10	W4	HPI4	well_2	F003	165	Avg. Volume	1.209549	1.216413	0.388694
MYH10	W4	HPI4	well_2	F004	223	Avg. Volume	1.264764	1.235848	0.436169
MYH10	W4	HPI4	well_2	F005	239	Avg. Volume	1.213205	1.166242	0.390169
MYH10	W4	HPI4	well_2	F006	162	Avg. Volume	1.410945	1.361266	0.485177
MYH10	W4	HPI4	well_3	F001	33	Avg. Volume	0.413489	0.409317	0.119971
MYH10	W4	HPI4	well_3	F002	46	Avg. Volume	1.334163	1.3487	0.328789
MYH10	W4	HPI4	well_3	F003	159	Avg. Volume	1.05103	1.065639	0.359316
MYH10	W4	HPI4	well_3	F004	172	Avg. Volume	1.742833	1.692776	0.607979
MYH10	W4	HPI4	well_3	F005	164	Avg. Volume	1.652274	1.685259	0.655918
MYH10	W4	HPI4	well_3	F006	162	Avg. Volume	1.345642	1.321	0.490058
MYH10	W4	HPI4	well_4	F001	114	Avg. Volume	1.21251	1.128053	0.484044
MYH10	W4	HPI4	well_4	F002	157	Avg. Volume	1.120948	1.079189	0.373734
MYH10	W4	HPI4	well_4	F003	114	Avg. Volume	1.052177	1.056156	0.373298
MYH10	W4	HPI4	well_4	F004	202	Avg. Volume	1.131421	1.063231	0.489864
MYH10	W4	HPI4	well_4	F005	139	Avg. Volume	1.25176	1.117347	0.578993
MYH10	W4	HPI4	well_4	F006	115	Avg. Volume	1.822784	1.888391	0.58495
MYH10	W4	HPI4	well_5	F001	131	Avg. Volume	1.657114	1.655534	0.497528
MYH10	W4	HPI4	well_5	F002	140	Avg. Volume	1.388018	1.404023	0.416678
MYH10	W4	HPI4	well_5	F003	145	Avg. Volume	1.449616	1.396933	0.485449
MYH10	W4	HPI4	well_5	F004	207	Avg. Volume	1.117319	1.065729	0.468306
MYH10	W4	HPI4	well_5	F005	237	Avg. Volume	1.583693	1.497914	0.575748
MYH10	W4	HPI4	well_5	F006	90	Avg. Volume	1.180788	1.120358	0.469745
MYH10	W4	PGE2	well_1	F001	315	Avg. Volume	2.282869	2.235077	0.746511
MYH10	W4	PGE2	well_1	F002	196	Avg. Volume	2.43803	2.438725	0.736708
MYH10	W4	PGE2	well_1	F003	251	Avg. Volume	1.8768	1.88169	0.564171
MYH10	W4	PGE2	well_1	F004	174	Avg. Volume	2.549437	2.54393	0.561016
MYH10	W4	PGE2	well_1	F005	327	Avg. Volume	2.648635	2.538797	0.766958
MYH10	W4	PGE2	well_1	F006	257	Avg. Volume	2.178398	2.153576	0.683054
MYH10	W4	PGE2	well_2	F001	168	Avg. Volume	1.9757	1.988891	0.498248
MYH10	W4	PGE2	well_2	F002	291	Avg. Volume	2.359755	2.266996	0.658657
MYH10	W4	PGE2	well_2	F003	88	Avg. Volume	1.954839	1.974917	0.532122
MYH10	W4	PGE2	well_2	F004	151	Avg. Volume	2.250216	2.169316	0.576406
MYH10	W4	PGE2	well_2	F005	404	Avg. Volume	2.211288	2.162556	0.696202
MYH10	W4	PGE2	well_2	F006	316	Avg. Volume	1.76921	1.703153	0.640047
MYH10	W4	PGE2	well_3	F001	215	Avg. Volume	2.095587	2.040703	0.491824
MYH10	W4	PGE2	well_3	F002	290	Avg. Volume	2.516538	2.444343	0.687095
MYH10	W4	PGE2	well_3	F003	354	Avg. Volume	2.322598	2.219986	0.807399
MYH10	W4	PGE2	well_3	F004	253	Avg. Volume	2.651679	2.584973	0.886254
MYH10	W4	PGE2	well_3	F005	224	Avg. Volume	1.944618	1.938404	0.622013
MYH10	W4	PGE2	well_3	F006	283	Avg. Volume	2.102311	2.03067	0.669922
MYH10	W4	PGE2	well_4	F001	268	Avg. Volume	1.993586	1.970614	0.588465
MYH10	W4	PGE2	well_4	F002	162	Avg. Volume	2.258841	2.219899	0.518577
MYH10	W4	PGE2	well_4	F003	297	Avg. Volume	2.465762	2.457024	0.644085
MYH10	W4	PGE2	well_4	F004	239	Avg. Volume	2.628147	2.526289	0.769653
MYH10	W4	PGE2	well_4	F005	349	Avg. Volume	2.088873	2.041841	0.611551

MYH10	W4	PGE2	well_4	F006	291	Avg. Volume	2.191798	2.116111	0.755305
MYH10	W4	PGE2	well_5	F001	248	Avg. Volume	1.821703	1.84638	0.459612
MYH10	W4	PGE2	well_5	F002	375	Avg. Volume	2.037197	1.969385	0.697397
MYH10	W4	PGE2	well_5	F003	176	Avg. Volume	2.277382	2.183908	0.615551
MYH10	W4	PGE2	well_5	F004	367	Avg. Volume	1.993802	1.899083	0.730622
MYH10	W4	PGE2	well_5	F005	179	Avg. Volume	2.402385	2.355605	0.551596
MYH10	W4	PGE2	well_5	F006	210	Avg. Volume	1.928869	1.92625	0.494293
MYH10	W1	HPI4	well_1	F001	148	Count/cell	106.2727	102	48.74346
MYH10	W1	HPI4	well_1	F002	167	Count/cell	111.1074	107	35.359
MYH10	W1	HPI4	well_1	F003	161	Count/cell	94.70946	89.5	41.00942
MYH10	W1	HPI4	well_1	F004	280	Count/cell	78.47843	76	31.1209
MYH10	W1	HPI4	well_1	F005	180	Count/cell	96.8642	90	36.13476
MYH10	W1	HPI4	well_1	F006	104	Count/cell	110.1739	109.5	36.81155
MYH10	W1	HPI4	well_2	F001	124	Count/cell	124.2973	124	52.93608
MYH10	W1	HPI4	well_2	F002	150	Count/cell	113.0896	110.5	42.19173
MYH10	W1	HPI4	well_2	F003	192	Count/cell	98.69767	96	36.35242
MYH10	W1	HPI4	well_2	F004	203	Count/cell	99.24862	97	32.29463
MYH10	W1	HPI4	well_2	F005	166	Count/cell	113.5068	108	36.39625
MYH10	W1	HPI4	well_2	F006	129	Count/cell	110.487	105	47.28847
MYH10	W1	HPI4	well_3	F001	118	Count/cell	115.3019	111.5	37.85515
MYH10	W1	HPI4	well_3	F002	194	Count/cell	101.9543	98	40.8241
MYH10	W1	HPI4	well_3	F003	115	Count/cell	121.4951	116	41.96097
MYH10	W1	HPI4	well_3	F004	98	Count/cell	117.4205	112.5	36.24112
MYH10	W1	HPI4	well_3	F005	123	Count/cell	123.7248	116	39.50688
MYH10	W1	HPI4	well_3	F006	212	Count/cell	97.13158	97	38.92713
MYH10	W1	HPI4	well_4	F001	81	Count/cell	136.2603	136	39.65687
MYH10	W1	HPI4	well_4	F002	266	Count/cell	71.99582	70	30.57674
MYH10	W1	HPI4	well_4	F003	158	Count/cell	107.8264	103.5	44.44582
MYH10	W1	HPI4	well_4	F004	186	Count/cell	93.89759	87.5	36.09191
MYH10	W1	HPI4	well_4	F005	126	Count/cell	125.5982	122	49.46278
MYH10	W1	HPI4	well_4	F006	114	Count/cell	131.3235	131	51.27699
MYH10	W1	HPI4	well_5	F001	167	Count/cell	94.8543	88	34.5094
MYH10	W1	HPI4	well_5	F002	143	Count/cell	110.6484	103	42.91772
MYH10	W1	HPI4	well_5	F003	222	Count/cell	93.88442	93	30.1391
MYH10	W1	HPI4	well_5	F004	184	Count/cell	112.0843	110	39.88216
MYH10	W1	HPI4	well_5	F005	164	Count/cell	122.4452	122	37.93298
MYH10	W1	HPI4	well_5	F006	148	Count/cell	109.9697	106	39.46163
MYH10	W1	PGE2	well_1	F001	145	Count/cell	112.3566	110	37.98166
MYH10	W1	PGE2	well_1	F002	91	Count/cell	124.0854	114.5	43.67959
MYH10	W1	PGE2	well_1	F003	250	Count/cell	85.09821	83	33.19489
MYH10	W1	PGE2	well_1	F004	393	Count/cell	72.90395	70	24.23719
MYH10	W1	PGE2	well_1	F005	286	Count/cell	88.8062	86	35.63719
MYH10	W1	PGE2	well_1	F006	145	Count/cell	118.6434	114	46.85263
MYH10	W1	PGE2	well_2	F001	219	Count/cell	100.4394	97	36.9982
MYH10	W1	PGE2	well_2	F002	184	Count/cell	85.7006	81	34.15689
MYH10	W1	PGE2	well_2	F003	177	Count/cell	105.8938	99	48.26481
MYH10	W1	PGE2	well_2	F004	158	Count/cell	116.5	112	42.46875
MYH10	W1	PGE2	well_2	F005	232	Count/cell	94.66346	93	34.30838
MYH10	W1	PGE2	well_2	F006	221	Count/cell	96.51759	96	39.95766

MYH10	W1	PGE2	well_3	F001	129	Count/cell	120.1695	116.5	46.57432
MYH10	W1	PGE2	well_3	F002	131	Count/cell	123.8051	123	38.19143
MYH10	W1	PGE2	well_3	F003	222	Count/cell	74.09091	71	28.34114
MYH10	W1	PGE2	well_3	F004	175	Count/cell	111.0127	105	40.28759
MYH10	W1	PGE2	well_3	F005	132	Count/cell	122.2966	122.5	41.71378
MYH10	W1	PGE2	well_3	F006	147	Count/cell	116.1221	114	40.45834
MYH10	W1	PGE2	well_4	F001	129	Count/cell	126.918	117	53.03867
MYH10	W1	PGE2	well_4	F002	210	Count/cell	98.47872	98	36.25732
MYH10	W1	PGE2	well_4	F003	116	Count/cell	121.8462	116	35.23121
MYH10	W1	PGE2	well_4	F004	99	Count/cell	130.0787	127	46.80599
MYH10	W1	PGE2	well_4	F005	153	Count/cell	124.2464	115.5	42.65903
MYH10	W1	PGE2	well_4	F006	223	Count/cell	93.74372	90	35.22496
MYH10	W1	PGE2	well_5	F001	105	Count/cell	127.2366	121	39.90516
MYH10	W1	PGE2	well_5	F002	132	Count/cell	128.4746	125.5	46.79903
MYH10	W1	PGE2	well_5	F003	233	Count/cell	81.74286	79	34.54365
MYH10	W1	PGE2	well_5	F004	179	Count/cell	110.9691	107.5	41.77081
MYH10	W1	PGE2	well_5	F005	190	Count/cell	106.4912	105	41.10437
MYH10	W1	PGE2	well_5	F006	168	Count/cell	116.404	113	37.01228
MYH10	W2	HPI4	well_1	F001	163	Count/cell	107.0753	103.5	45.63031
MYH10	W2	HPI4	well_1	F002	160	Count/cell	143.4868	146.5	56.61207
MYH10	W2	HPI4	well_1	F003	150	Count/cell	111.7612	110	43.00904
MYH10	W2	HPI4	well_1	F004	250	Count/cell	76.75556	74	33.7961
MYH10	W2	HPI4	well_1	F005	219	Count/cell	109.9645	107	49.41994
MYH10	W2	HPI4	well_1	F006	165	Count/cell	70.08725	67	28.30145
MYH10	W2	HPI4	well_2	F001	156	Count/cell	65.70714	62	28.01657
MYH10	W2	HPI4	well_2	F002	155	Count/cell	108.2429	99.5	55.35911
MYH10	W2	HPI4	well_2	F003	191	Count/cell	66.23392	60	33.19219
MYH10	W2	HPI4	well_2	F004	180	Count/cell	88.81481	81.5	43.7354
MYH10	W2	HPI4	well_2	F005	148	Count/cell	103.5263	98	37.82808
MYH10	W2	HPI4	well_2	F006	133	Count/cell	46.75833	39	27.09615
MYH10	W2	HPI4	well_3	F001	100	Count/cell	95.3	87	45.17667
MYH10	W2	HPI4	well_3	F002	212	Count/cell	71.87435	66	30.28818
MYH10	W2	HPI4	well_3	F003	218	Count/cell	51.71717	47	26.51617
MYH10	W2	HPI4	well_3	F004	125	Count/cell	63.84685	57	32.08517
MYH10	W2	HPI4	well_3	F005	183	Count/cell	87.38037	82	44.90033
MYH10	W2	HPI4	well_3	F006	113	Count/cell	74.13592	67	38.91974
MYH10	W2	HPI4	well_4	F001	170	Count/cell	61.37255	60	27.4377
MYH10	W2	HPI4	well_4	F002	209	Count/cell	55.84492	53	21.25378
MYH10	W2	HPI4	well_4	F003	235	Count/cell	93.62085	92	37.25266
MYH10	W2	HPI4	well_4	F004	333	Count/cell	82.33223	76	35.40032
MYH10	W2	HPI4	well_4	F005	198	Count/cell	91.64045	87	39.3141
MYH10	W2	HPI4	well_4	F006	91	Count/cell	166.7209	161.5	60.57677
MYH10	W2	HPI4	well_5	F001	121	Count/cell	96.26364	89.5	40.6612
MYH10	W2	HPI4	well_5	F002	156	Count/cell	84.97163	78	40.67694
MYH10	W2	HPI4	well_5	F003	252	Count/cell	100.7225	100	38.68282
MYH10	W2	HPI4	well_5	F004	306	Count/cell	69.22464	66	33.17064
MYH10	W2	HPI4	well_5	F005	250	Count/cell	95.54464	85	44.0013
MYH10	W2	HPI4	well_5	F006	160	Count/cell	68.67361	64.5	30.26037
MYH10	W2	PGE2	well_1	F001	49	Count/cell	60.53488	48	31.1405

MYH10	W2	PGE2	well_1	F002	70	Count/cell	79.14516	77.5	27.51741
MYH10	W2	PGE2	well_1	F003	149	Count/cell	61.4812	59	21.33059
MYH10	W2	PGE2	well_1	F004	318	Count/cell	67.86063	67	22.45309
MYH10	W2	PGE2	well_1	F005	207	Count/cell	74.15676	70	24.81173
MYH10	W2	PGE2	well_1	F006	103	Count/cell	79.57143	79	23.83841
MYH10	W2	PGE2	well_2	F001	257	Count/cell	84	82	24.77259
MYH10	W2	PGE2	well_2	F002	384	Count/cell	75.99128	75	25.50098
MYH10	W2	PGE2	well_2	F003	118	Count/cell	78.79245	81	23.53426
MYH10	W2	PGE2	well_2	F004	254	Count/cell	62.06987	63	21.13147
MYH10	W2	PGE2	well_2	F005	42	Count/cell	83.94444	78.5	39.70602
MYH10	W2	PGE2	well_3	F001	261	Count/cell	61.60593	59	23.56308
MYH10	W2	PGE2	well_3	F002	398	Count/cell	68.07778	66	25.46601
MYH10	W2	PGE2	well_3	F003	206	Count/cell	79.11413	77	23.3971
MYH10	W2	PGE2	well_3	F004	360	Count/cell	61.40979	60	23.7618
MYH10	W2	PGE2	well_3	F005	277	Count/cell	80	78	23.04431
MYH10	W2	PGE2	well_3	F006	218	Count/cell	69.27778	68	24.8232
MYH10	W2	PGE2	well_4	F001	175	Count/cell	85.35669	88	19.0892
MYH10	W2	PGE2	well_4	F002	320	Count/cell	68.83333	66.5	26.38604
MYH10	W2	PGE2	well_4	F003	319	Count/cell	83.15278	83	20.89623
MYH10	W2	PGE2	well_4	F004	356	Count/cell	66.61059	65	24.47041
MYH10	W2	PGE2	well_4	F005	347	Count/cell	69.377	67	23.05953
MYH10	W2	PGE2	well_4	F006	382	Count/cell	75.4186	76	24.58102
MYH10	W2	PGE2	well_5	F001	173	Count/cell	65.73248	68	22.33325
MYH10	W2	PGE2	well_5	F002	335	Count/cell	74.20915	72	25.08155
MYH10	W2	PGE2	well_5	F003	391	Count/cell	77.36752	77	24.65358
MYH10	W2	PGE2	well_5	F004	414	Count/cell	68.58133	66	20.72479
MYH10	W2	PGE2	well_5	F005	292	Count/cell	72.51908	71	24.19805
MYH10	W2	PGE2	well_5	F006	329	Count/cell	62.46102	59	20.50114
MYH10	W3	HPI4	well_1	F001	139	Count/cell	97.04	92	38.02766
MYH10	W3	HPI4	well_1	F002	206	Count/cell	82.18478	77.5	36.64359
MYH10	W3	HPI4	well_1	F003	196	Count/cell	110.7841	109.5	46.40454
MYH10	W3	HPI4	well_1	F004	181	Count/cell	95.6319	91	43.21765
MYH10	W3	HPI4	well_1	F005	110	Count/cell	129.6531	126.5	47.9235
MYH10	W3	HPI4	well_1	F006	154	Count/cell	96.50725	89	38.62703
MYH10	W3	HPI4	well_2	F001	97	Count/cell	103.4023	99	45.23928
MYH10	W3	HPI4	well_2	F002	182	Count/cell	79.88957	77	30.50987
MYH10	W3	HPI4	well_2	F003	163	Count/cell	119.3724	116	40.15473
MYH10	W3	HPI4	well_2	F004	189	Count/cell	96.05917	96	32.01408
MYH10	W3	HPI4	well_2	F005	267	Count/cell	73.77406	72	33.96042
MYH10	W3	HPI4	well_2	F006	122	Count/cell	144.8348	144	60.4428
MYH10	W3	HPI4	well_3	F001	60	Count/cell	104.2963	102	42.5395
MYH10	W3	HPI4	well_3	F002	164	Count/cell	90.39726	89	36.07473
MYH10	W3	HPI4	well_3	F003	203	Count/cell	80.1044	77.5	33.77051
MYH10	W3	HPI4	well_3	F004	228	Count/cell	98.60294	94	38.9035
MYH10	W3	HPI4	well_3	F005	341	Count/cell	69.61093	64	34.57259
MYH10	W3	HPI4	well_3	F006	131	Count/cell	106.6102	108.5	36.84503
MYH10	W3	HPI4	well_4	F001	99	Count/cell	118.9438	116	48.9228
MYH10	W3	HPI4	well_4	F002	175	Count/cell	111.8165	110	38.63298
MYH10	W3	HPI4	well_4	F003	152	Count/cell	89.30882	91.5	36.35127

MYH10	W3	HPI4	well_4	F004	188	Count/cell	87.09524	85	31.49513
MYH10	W3	HPI4	well_4	F005	166	Count/cell	107.027	102	42.70846
MYH10	W3	HPI4	well_4	F006	78	Count/cell	157.973	152.5	59.48753
MYH10	W3	HPI4	well_5	F001	62	Count/cell	119.8364	120	49.56394
MYH10	W3	HPI4	well_5	F002	128	Count/cell	115.9035	112.5	33.69934
MYH10	W3	HPI4	well_5	F003	105	Count/cell	135.7097	124	40.76299
MYH10	W3	HPI4	well_5	F004	63	Count/cell	127.5273	125	40.85152
MYH10	W3	HPI4	well_5	F005	179	Count/cell	94.34783	86	40.35208
MYH10	W3	HPI4	well_5	F006	169	Count/cell	111.6093	114	40.89384
MYH10	W3	PGE2	well_1	F001	354	Count/cell	71.35	71	19.23219
MYH10	W3	PGE2	well_1	F002	355	Count/cell	84.52038	82	24.85073
MYH10	W3	PGE2	well_1	F003	397	Count/cell	71.13536	68	23.52145
MYH10	W3	PGE2	well_1	F004	275	Count/cell	82.19028	79	25.21131
MYH10	W3	PGE2	well_1	F005	227	Count/cell	71.14286	70	23.15283
MYH10	W3	PGE2	well_2	F001	271	Count/cell	78.62295	75.5	24.50072
MYH10	W3	PGE2	well_2	F002	245	Count/cell	82.75	83	24.20774
MYH10	W3	PGE2	well_2	F003	364	Count/cell	74.65337	72	21.42133
MYH10	W3	PGE2	well_2	F004	225	Count/cell	83.09453	83	18.55629
MYH10	W3	PGE2	well_2	F005	358	Count/cell	71.81424	72	23.28975
MYH10	W3	PGE2	well_2	F006	351	Count/cell	72.52201	70	22.37967
MYH10	W3	PGE2	well_3	F001	250	Count/cell	63.96875	62	20.90645
MYH10	W3	PGE2	well_3	F002	124	Count/cell	85.01818	80	27.64287
MYH10	W3	PGE2	well_3	F003	255	Count/cell	75.04803	71	29.60844
MYH10	W3	PGE2	well_3	F004	171	Count/cell	76.70588	76	26.94143
MYH10	W3	PGE2	well_3	F005	312	Count/cell	67.21708	64	21.89078
MYH10	W3	PGE2	well_3	F006	265	Count/cell	73.75314	73	23.07118
MYH10	W3	PGE2	well_4	F001	110	Count/cell	82.64286	77	22.33819
MYH10	W3	PGE2	well_4	F002	178	Count/cell	75.86335	72	28.40543
MYH10	W3	PGE2	well_4	F003	273	Count/cell	72.84898	68	24.2145
MYH10	W3	PGE2	well_4	F004	73	Count/cell	78.36923	85	24.81152
MYH10	W3	PGE2	well_4	F005	155	Count/cell	92.56115	88	33.74931
MYH10	W3	PGE2	well_4	F006	390	Count/cell	67.4136	65	21.50645
MYH10	W3	PGE2	well_5	F001	252	Count/cell	69.26872	67	20.49828
MYH10	W3	PGE2	well_5	F002	254	Count/cell	75.94783	72	24.25262
MYH10	W3	PGE2	well_5	F003	338	Count/cell	73.40328	71	21.71143
MYH10	W3	PGE2	well_5	F004	200	Count/cell	75.47253	74	21.81836
MYH10	W3	PGE2	well_5	F005	285	Count/cell	64.11328	64	23.2399
MYH10	W3	PGE2	well_5	F006	198	Count/cell	68.27374	64	21.55964
MYH10	W4	HPI4	well_1	F001	104	Count/cell	122.1848	112	44.87417
MYH10	W4	HPI4	well_1	F002	93	Count/cell	103.3373	100	37.57927
MYH10	W4	HPI4	well_1	F003	177	Count/cell	79.38365	79	32.82882
MYH10	W4	HPI4	well_1	F004	250	Count/cell	75.40265	73.5	32.54893
MYH10	W4	HPI4	well_1	F005	205	Count/cell	80.04865	75	32.4952
MYH10	W4	HPI4	well_1	F006	174	Count/cell	115.141	107	40.86839
MYH10	W4	HPI4	well_2	F001	48	Count/cell	165.4889	177	58.82007
MYH10	W4	HPI4	well_2	F002	58	Count/cell	168.3455	177	59.4469
MYH10	W4	HPI4	well_2	F003	165	Count/cell	77.72297	70.5	37.84049
MYH10	W4	HPI4	well_2	F004	223	Count/cell	90.87	82.5	43.09733
MYH10	W4	HPI4	well_2	F005	239	Count/cell	89.05116	83	30.88178

MYH10	W4	HPI4	well_2	F006	162	Count/cell	103.3472	96	36.72108
MYH10	W4	HPI4	well_3	F001	33	Count/cell	146.6452	116	69.04518
MYH10	W4	HPI4	well_3	F002	46	Count/cell	205.6279	217	48.76622
MYH10	W4	HPI4	well_3	F003	159	Count/cell	110.0556	103	47.75063
MYH10	W4	HPI4	well_3	F004	172	Count/cell	96.75974	90	42.74182
MYH10	W4	HPI4	well_3	F005	164	Count/cell	106.3469	98	40.68567
MYH10	W4	HPI4	well_3	F006	162	Count/cell	123.1528	120	45.09901
MYH10	W4	HPI4	well_4	F001	114	Count/cell	148.3241	142	57.69543
MYH10	W4	HPI4	well_4	F002	157	Count/cell	116.1867	98.5	59.51588
MYH10	W4	HPI4	well_4	F003	114	Count/cell	125.4815	117	56.4977
MYH10	W4	HPI4	well_4	F004	202	Count/cell	47.52778	45	20.00347
MYH10	W4	HPI4	well_4	F005	139	Count/cell	54.952	53	24.90526
MYH10	W4	HPI4	well_4	F006	115	Count/cell	162.156	157	66.85434
MYH10	W4	HPI4	well_5	F001	131	Count/cell	170.6452	167.5	60.76226
MYH10	W4	HPI4	well_5	F002	140	Count/cell	151.9248	144	54.51392
MYH10	W4	HPI4	well_5	F003	145	Count/cell	153.2482	147	60.27874
MYH10	W4	HPI4	well_5	F004	207	Count/cell	89.60541	82	35.80034
MYH10	W4	HPI4	well_5	F005	237	Count/cell	90.47887	86	43.33607
MYH10	W4	HPI4	well_5	F006	90	Count/cell	197.1059	198	50.61691
MYH10	W4	PGE2	well_1	F001	315	Count/cell	83.70877	81	21.70796
MYH10	W4	PGE2	well_1	F002	196	Count/cell	81.29213	79	30.35262
MYH10	W4	PGE2	well_1	F003	251	Count/cell	80.47111	76	26.85461
MYH10	W4	PGE2	well_1	F004	174	Count/cell	82.80769	81.5	18.26759
MYH10	W4	PGE2	well_1	F005	327	Count/cell	77.78983	78	22.54587
MYH10	W4	PGE2	well_1	F006	257	Count/cell	75.27273	72	24.16787
MYH10	W4	PGE2	well_2	F001	168	Count/cell	86.42667	86.5	24.49362
MYH10	W4	PGE2	well_2	F002	291	Count/cell	71.27586	70	18.15893
MYH10	W4	PGE2	well_2	F003	88	Count/cell	81.76923	79	26.28017
MYH10	W4	PGE2	well_2	F004	151	Count/cell	90.66176	91.5	21.71472
MYH10	W4	PGE2	well_2	F005	404	Count/cell	57.71625	56	19.04573
MYH10	W4	PGE2	well_2	F006	316	Count/cell	70.24296	66	24.41848
MYH10	W4	PGE2	well_3	F001	215	Count/cell	92.55102	90	28.2493
MYH10	W4	PGE2	well_3	F002	290	Count/cell	71.69962	70	19.40816
MYH10	W4	PGE2	well_3	F003	354	Count/cell	64.57188	62	22.97636
MYH10	W4	PGE2	well_3	F004	253	Count/cell	62.92952	60	19.94826
MYH10	W4	PGE2	well_3	F005	224	Count/cell	80.255	78	20.17461
MYH10	W4	PGE2	well_3	F006	283	Count/cell	64.92941	62	19.87686
MYH10	W4	PGE2	well_4	F001	268	Count/cell	68.77593	68	20.60177
MYH10	W4	PGE2	well_4	F002	162	Count/cell	94.08966	91	30.11757
MYH10	W4	PGE2	well_4	F003	297	Count/cell	72.00375	69	19.15793
MYH10	W4	PGE2	well_4	F004	239	Count/cell	63.30556	60	20.03367
MYH10	W4	PGE2	well_4	F005	349	Count/cell	72.96508	71	24.54261
MYH10	W4	PGE2	well_4	F006	291	Count/cell	61.31034	60	18.29017
MYH10	W4	PGE2	well_5	F001	248	Count/cell	89.8296	88	22.9258
MYH10	W4	PGE2	well_5	F002	375	Count/cell	63.57227	64	18.98746
MYH10	W4	PGE2	well_5	F003	176	Count/cell	81.03774	78	23.01977
MYH10	W4	PGE2	well_5	F004	367	Count/cell	63.48328	61	21.38912
MYH10	W4	PGE2	well_5	F005	179	Count/cell	80.60494	78.5	21.15848
MYH10	W4	PGE2	well_5	F006	210	Count/cell	74.79255	73	21.03307

MYH10	W1	HPI4	well_1	F001	148	X/Y distribution	8.613931	8.219996	1.718857
MYH10	W1	HPI4	well_1	F002	167	X/Y distribution	8.404341	8.224778	1.297928
MYH10	W1	HPI4	well_1	F003	161	X/Y distribution	8.242076	8.067532	1.592777
MYH10	W1	HPI4	well_1	F004	280	X/Y distribution	7.255143	7.13917	1.249743
MYH10	W1	HPI4	well_1	F005	180	X/Y distribution	8.469455	8.220058	1.464295
MYH10	W1	HPI4	well_1	F006	104	X/Y distribution	8.89159	8.686124	1.403977
MYH10	W1	HPI4	well_2	F001	124	X/Y distribution	9.113113	9.235182	1.627597
MYH10	W1	HPI4	well_2	F002	150	X/Y distribution	8.197775	8.059265	1.325654
MYH10	W1	HPI4	well_2	F003	192	X/Y distribution	7.72785	7.585005	1.199647
MYH10	W1	HPI4	well_2	F004	203	X/Y distribution	7.910269	7.856536	1.233026
MYH10	W1	HPI4	well_2	F005	166	X/Y distribution	8.295643	8.143716	1.516896
MYH10	W1	HPI4	well_2	F006	129	X/Y distribution	9.045179	8.757556	1.634843
MYH10	W1	HPI4	well_3	F001	118	X/Y distribution	8.888688	8.780236	1.644527
MYH10	W1	HPI4	well_3	F002	194	X/Y distribution	7.936503	7.941659	1.298627
MYH10	W1	HPI4	well_3	F003	115	X/Y distribution	8.966563	8.949547	1.781683
MYH10	W1	HPI4	well_3	F004	98	X/Y distribution	9.12735	8.849574	1.634226
MYH10	W1	HPI4	well_3	F005	123	X/Y distribution	8.913884	8.713531	1.530104
MYH10	W1	HPI4	well_3	F006	212	X/Y distribution	8.025905	7.818361	1.396915
MYH10	W1	HPI4	well_4	F001	81	X/Y distribution	9.274219	9.100159	1.561277
MYH10	W1	HPI4	well_4	F002	266	X/Y distribution	7.430473	7.409521	1.183801
MYH10	W1	HPI4	well_4	F003	158	X/Y distribution	8.551694	8.156688	1.621233
MYH10	W1	HPI4	well_4	F004	186	X/Y distribution	8.120953	7.954757	1.373827
MYH10	W1	HPI4	well_4	F005	126	X/Y distribution	8.760734	8.871487	1.610356
MYH10	W1	HPI4	well_4	F006	114	X/Y distribution	9.485229	9.443498	1.923224
MYH10	W1	HPI4	well_5	F001	167	X/Y distribution	8.234649	7.87733	1.454067
MYH10	W1	HPI4	well_5	F002	143	X/Y distribution	8.561061	8.413757	1.505505
MYH10	W1	HPI4	well_5	F003	222	X/Y distribution	7.475619	7.284693	1.352481
MYH10	W1	HPI4	well_5	F004	184	X/Y distribution	8.305638	8.208938	1.292641
MYH10	W1	HPI4	well_5	F005	164	X/Y distribution	8.110919	8.047309	1.361333
MYH10	W1	HPI4	well_5	F006	148	X/Y distribution	8.370663	8.151862	1.757677
MYH10	W1	PGE2	well_1	F001	145	X/Y distribution	8.935981	8.848497	1.484469
MYH10	W1	PGE2	well_1	F002	91	X/Y distribution	9.694621	9.695868	1.615568
MYH10	W1	PGE2	well_1	F003	250	X/Y distribution	7.660049	7.529195	1.27941
MYH10	W1	PGE2	well_1	F004	393	X/Y distribution	6.173193	6.175639	0.929347
MYH10	W1	PGE2	well_1	F005	286	X/Y distribution	7.296918	7.167878	1.208397
MYH10	W1	PGE2	well_1	F006	145	X/Y distribution	8.784547	8.77266	1.432858
MYH10	W1	PGE2	well_2	F001	219	X/Y distribution	7.770585	7.586005	1.176495
MYH10	W1	PGE2	well_2	F002	184	X/Y distribution	8.151087	7.851132	1.560229
MYH10	W1	PGE2	well_2	F003	177	X/Y distribution	8.790421	8.580067	1.713961
MYH10	W1	PGE2	well_2	F004	158	X/Y distribution	8.704494	8.498171	1.489691
MYH10	W1	PGE2	well_2	F005	232	X/Y distribution	7.601225	7.550843	1.08722
MYH10	W1	PGE2	well_2	F006	221	X/Y distribution	7.973581	7.875521	1.330035
MYH10	W1	PGE2	well_3	F001	129	X/Y distribution	8.923815	8.783061	1.792078
MYH10	W1	PGE2	well_3	F002	131	X/Y distribution	8.723769	8.50261	1.335308
MYH10	W1	PGE2	well_3	F003	222	X/Y distribution	7.819997	7.748734	1.241938
MYH10	W1	PGE2	well_3	F004	175	X/Y distribution	8.271723	8.235208	1.321761
MYH10	W1	PGE2	well_3	F005	132	X/Y distribution	8.928763	8.647458	1.627437
MYH10	W1	PGE2	well_3	F006	147	X/Y distribution	8.71978	8.468648	1.481189
MYH10	W1	PGE2	well_4	F001	129	X/Y distribution	8.387675	8.309528	1.497239

MYH10	W1	PGE2	well_4	F002	210	X/Y distribution	7.80097	7.734195	1.133423
MYH10	W1	PGE2	well_4	F003	116	X/Y distribution	9.012562	8.756825	1.615989
MYH10	W1	PGE2	well_4	F004	99	X/Y distribution	9.354872	9.600045	1.739303
MYH10	W1	PGE2	well_4	F005	153	X/Y distribution	7.995696	7.793543	1.356897
MYH10	W1	PGE2	well_4	F006	223	X/Y distribution	7.746227	7.611654	1.312879
MYH10	W1	PGE2	well_5	F001	105	X/Y distribution	9.257358	9.082876	1.775282
MYH10	W1	PGE2	well_5	F002	132	X/Y distribution	8.577715	8.594472	1.505553
MYH10	W1	PGE2	well_5	F003	233	X/Y distribution	7.552003	7.31367	1.372982
MYH10	W1	PGE2	well_5	F004	179	X/Y distribution	8.100573	7.907264	1.300797
MYH10	W1	PGE2	well_5	F005	190	X/Y distribution	7.832813	7.71977	1.438396
MYH10	W1	PGE2	well_5	F006	168	X/Y distribution	8.099846	7.831767	1.295335
MYH10	W2	HPI4	well_1	F001	163	X/Y distribution	7.705112	7.495179	1.647332
MYH10	W2	HPI4	well_1	F002	160	X/Y distribution	7.983918	7.845108	1.453169
MYH10	W2	HPI4	well_1	F003	150	X/Y distribution	7.582252	7.498229	1.261298
MYH10	W2	HPI4	well_1	F004	250	X/Y distribution	7.166084	7.051174	1.392123
MYH10	W2	HPI4	well_1	F005	219	X/Y distribution	7.079739	6.986322	1.289918
MYH10	W2	HPI4	well_1	F006	165	X/Y distribution	6.438155	6.385121	1.266636
MYH10	W2	HPI4	well_2	F001	156	X/Y distribution	7.177795	7.12174	1.596112
MYH10	W2	HPI4	well_2	F002	155	X/Y distribution	7.303851	7.273154	1.704913
MYH10	W2	HPI4	well_2	F003	191	X/Y distribution	6.649411	6.611015	1.265099
MYH10	W2	HPI4	well_2	F004	180	X/Y distribution	7.079634	6.90289	1.609273
MYH10	W2	HPI4	well_2	F005	148	X/Y distribution	7.668808	7.468545	1.274211
MYH10	W2	HPI4	well_2	F006	133	X/Y distribution	6.127704	6.154151	1.625286
MYH10	W2	HPI4	well_3	F001	100	X/Y distribution	7.667818	7.208299	2.02043
MYH10	W2	HPI4	well_3	F002	212	X/Y distribution	7.560451	7.501653	1.700656
MYH10	W2	HPI4	well_3	F003	218	X/Y distribution	6.748731	6.704667	1.716204
MYH10	W2	HPI4	well_3	F004	125	X/Y distribution	6.586864	6.263236	2.025679
MYH10	W2	HPI4	well_3	F005	183	X/Y distribution	6.775434	6.749355	1.501544
MYH10	W2	HPI4	well_3	F006	113	X/Y distribution	7.030551	7.125661	1.631382
MYH10	W2	HPI4	well_4	F001	170	X/Y distribution	6.443931	6.184004	1.397756
MYH10	W2	HPI4	well_4	F002	209	X/Y distribution	6.321138	6.187966	1.470885
MYH10	W2	HPI4	well_4	F003	235	X/Y distribution	6.879665	6.734943	1.300535
MYH10	W2	HPI4	well_4	F004	333	X/Y distribution	6.377891	6.352305	1.159403
MYH10	W2	HPI4	well_4	F005	198	X/Y distribution	6.90769	6.907561	1.316407
MYH10	W2	HPI4	well_4	F006	91	X/Y distribution	8.790906	8.81537	1.635181
MYH10	W2	HPI4	well_5	F001	121	X/Y distribution	8.188688	8.075746	1.848799
MYH10	W2	HPI4	well_5	F002	156	X/Y distribution	7.359242	7.168552	1.63843
MYH10	W2	HPI4	well_5	F003	252	X/Y distribution	7.193716	6.996666	1.248399
MYH10	W2	HPI4	well_5	F004	306	X/Y distribution	6.395647	6.302916	1.305751
MYH10	W2	HPI4	well_5	F005	250	X/Y distribution	6.91829	6.883251	1.348029
MYH10	W2	HPI4	well_5	F006	160	X/Y distribution	7.330617	7.013464	1.637323
MYH10	W2	PGE2	well_1	F001	49	X/Y distribution	5.531303	5.293762	1.188216
MYH10	W2	PGE2	well_1	F002	70	X/Y distribution	5.951083	5.887142	0.838032
MYH10	W2	PGE2	well_1	F003	149	X/Y distribution	5.348519	5.143345	0.787148
MYH10	W2	PGE2	well_1	F004	318	X/Y distribution	5.439383	5.450792	0.694032
MYH10	W2	PGE2	well_1	F005	207	X/Y distribution	5.822735	5.780096	0.783179
MYH10	W2	PGE2	well_1	F006	103	X/Y distribution	5.875963	5.81697	0.831254
MYH10	W2	PGE2	well_2	F001	257	X/Y distribution	6.271197	6.138258	0.894251
MYH10	W2	PGE2	well_2	F002	384	X/Y distribution	6.080633	6.075509	0.843112

MYH10	W2	PGE2	well_2	F003	118	X/Y distribution	5.798576	5.719176	0.708915
MYH10	W2	PGE2	well_2	F004	254	X/Y distribution	5.458878	5.280501	0.85698
MYH10	W2	PGE2	well_2	F005	42	X/Y distribution	6.101219	6.003086	1.307669
MYH10	W2	PGE2	well_3	F001	261	X/Y distribution	5.770481	5.709441	0.925213
MYH10	W2	PGE2	well_3	F002	398	X/Y distribution	6.076459	6.003628	1.031907
MYH10	W2	PGE2	well_3	F003	206	X/Y distribution	5.898596	5.877335	0.77587
MYH10	W2	PGE2	well_3	F004	360	X/Y distribution	5.56842	5.48295	0.87222
MYH10	W2	PGE2	well_3	F005	277	X/Y distribution	6.041404	5.978331	0.831172
MYH10	W2	PGE2	well_3	F006	218	X/Y distribution	5.734971	5.711247	0.916764
MYH10	W2	PGE2	well_4	F001	175	X/Y distribution	5.98466	6.021404	0.669717
MYH10	W2	PGE2	well_4	F002	320	X/Y distribution	5.515661	5.49368	0.830089
MYH10	W2	PGE2	well_4	F003	319	X/Y distribution	6.04276	6.055953	0.672693
MYH10	W2	PGE2	well_4	F004	356	X/Y distribution	5.590613	5.516569	0.747628
MYH10	W2	PGE2	well_4	F005	347	X/Y distribution	5.585772	5.526427	0.841924
MYH10	W2	PGE2	well_4	F006	382	X/Y distribution	6.201448	6.111243	0.966838
MYH10	W2	PGE2	well_5	F001	173	X/Y distribution	5.503483	5.403846	0.727489
MYH10	W2	PGE2	well_5	F002	335	X/Y distribution	5.751313	5.67899	0.830743
MYH10	W2	PGE2	well_5	F003	391	X/Y distribution	5.759465	5.770508	0.780539
MYH10	W2	PGE2	well_5	F004	414	X/Y distribution	5.394303	5.305993	0.687525
MYH10	W2	PGE2	well_5	F005	292	X/Y distribution	5.696208	5.655042	0.799583
MYH10	W2	PGE2	well_5	F006	329	X/Y distribution	5.220023	5.09961	0.797615
MYH10	W3	HPI4	well_1	F001	139	X/Y distribution	7.930692	7.513916	1.451656
MYH10	W3	HPI4	well_1	F002	206	X/Y distribution	7.353703	7.313007	1.260471
MYH10	W3	HPI4	well_1	F003	196	X/Y distribution	7.852507	7.635187	1.363175
MYH10	W3	HPI4	well_1	F004	181	X/Y distribution	7.438431	7.254994	1.321258
MYH10	W3	HPI4	well_1	F005	110	X/Y distribution	8.231398	8.133223	1.648147
MYH10	W3	HPI4	well_1	F006	154	X/Y distribution	7.048313	6.882802	1.27276
MYH10	W3	HPI4	well_2	F001	97	X/Y distribution	9.483724	9.38412	2.210674
MYH10	W3	HPI4	well_2	F002	182	X/Y distribution	7.527956	7.390843	1.262045
MYH10	W3	HPI4	well_2	F003	163	X/Y distribution	7.842073	7.797089	1.206542
MYH10	W3	HPI4	well_2	F004	189	X/Y distribution	7.68186	7.675386	1.187641
MYH10	W3	HPI4	well_2	F005	267	X/Y distribution	6.463946	6.297716	1.315831
MYH10	W3	HPI4	well_2	F006	122	X/Y distribution	8.213057	8.218042	1.492551
MYH10	W3	HPI4	well_3	F001	60	X/Y distribution	8.112932	7.570563	1.719702
MYH10	W3	HPI4	well_3	F002	164	X/Y distribution	7.677873	7.746548	1.372881
MYH10	W3	HPI4	well_3	F003	203	X/Y distribution	7.015971	6.876026	1.342781
MYH10	W3	HPI4	well_3	F004	228	X/Y distribution	7.124494	6.96411	1.163362
MYH10	W3	HPI4	well_3	F005	341	X/Y distribution	6.288394	6.203552	1.320904
MYH10	W3	HPI4	well_3	F006	131	X/Y distribution	8.040353	7.962339	1.269466
MYH10	W3	HPI4	well_4	F001	99	X/Y distribution	9.130542	9.06288	1.469407
MYH10	W3	HPI4	well_4	F002	175	X/Y distribution	7.433663	7.369505	1.123345
MYH10	W3	HPI4	well_4	F003	152	X/Y distribution	7.197647	7.298399	1.306004
MYH10	W3	HPI4	well_4	F004	188	X/Y distribution	6.778049	6.695746	0.93885
MYH10	W3	HPI4	well_4	F005	166	X/Y distribution	7.192178	7.138677	1.362187
MYH10	W3	HPI4	well_4	F006	78	X/Y distribution	8.040671	8.097309	1.387424
MYH10	W3	HPI4	well_5	F001	62	X/Y distribution	8.612042	8.712023	1.48364
MYH10	W3	HPI4	well_5	F002	128	X/Y distribution	7.608366	7.509268	1.13729
MYH10	W3	HPI4	well_5	F003	105	X/Y distribution	8.17174	8.075825	1.120331
MYH10	W3	HPI4	well_5	F004	63	X/Y distribution	8.454234	8.524203	1.442017

MYH10	W3	HPI4	well_5	F005	179	X/Y distribution	7.683027	7.613395	1.2158
MYH10	W3	HPI4	well_5	F006	169	X/Y distribution	7.860132	7.818179	1.423566
MYH10	W3	PGE2	well_1	F001	354	X/Y distribution	5.284515	5.201172	0.61556
MYH10	W3	PGE2	well_1	F002	355	X/Y distribution	5.784863	5.726949	0.661
MYH10	W3	PGE2	well_1	F003	397	X/Y distribution	5.343452	5.310543	0.667967
MYH10	W3	PGE2	well_1	F004	275	X/Y distribution	5.95887	5.871911	0.835646
MYH10	W3	PGE2	well_1	F005	227	X/Y distribution	5.60623	5.49091	0.799912
MYH10	W3	PGE2	well_2	F001	271	X/Y distribution	5.927637	5.841446	0.826555
MYH10	W3	PGE2	well_2	F002	245	X/Y distribution	5.947124	5.819684	0.796468
MYH10	W3	PGE2	well_2	F003	364	X/Y distribution	5.872765	5.782934	0.835765
MYH10	W3	PGE2	well_2	F004	225	X/Y distribution	5.867078	5.893211	0.615227
MYH10	W3	PGE2	well_2	F005	358	X/Y distribution	5.78556	5.701344	0.897816
MYH10	W3	PGE2	well_2	F006	351	X/Y distribution	5.574127	5.52186	0.68814
MYH10	W3	PGE2	well_3	F001	250	X/Y distribution	5.417122	5.272542	0.802
MYH10	W3	PGE2	well_3	F002	124	X/Y distribution	5.844208	5.677186	0.864952
MYH10	W3	PGE2	well_3	F003	255	X/Y distribution	5.899288	5.803151	0.799407
MYH10	W3	PGE2	well_3	F004	171	X/Y distribution	5.770812	5.556472	0.896629
MYH10	W3	PGE2	well_3	F005	312	X/Y distribution	5.587493	5.486215	0.785318
MYH10	W3	PGE2	well_3	F006	265	X/Y distribution	5.681059	5.647748	0.681822
MYH10	W3	PGE2	well_4	F001	110	X/Y distribution	6.000729	5.932513	0.677202
MYH10	W3	PGE2	well_4	F002	178	X/Y distribution	6.3492	6.228661	0.900227
MYH10	W3	PGE2	well_4	F003	273	X/Y distribution	5.772952	5.757031	0.716603
MYH10	W3	PGE2	well_4	F004	73	X/Y distribution	5.485714	5.592039	0.770701
MYH10	W3	PGE2	well_4	F005	155	X/Y distribution	6.172943	6.111546	0.904677
MYH10	W3	PGE2	well_4	F006	390	X/Y distribution	5.902426	5.779981	0.879085
MYH10	W3	PGE2	well_5	F001	252	X/Y distribution	5.701347	5.587402	0.842135
MYH10	W3	PGE2	well_5	F002	254	X/Y distribution	6.230936	6.246622	1.039328
MYH10	W3	PGE2	well_5	F003	338	X/Y distribution	6.075963	6.020469	0.858233
MYH10	W3	PGE2	well_5	F004	200	X/Y distribution	5.545599	5.496154	0.613999
MYH10	W3	PGE2	well_5	F005	285	X/Y distribution	5.808292	5.728732	0.929557
MYH10	W3	PGE2	well_5	F006	198	X/Y distribution	5.97712	5.893268	0.805432
MYH10	W4	HPI4	well_1	F001	104	X/Y distribution	8.27976	8.23223	1.331687
MYH10	W4	HPI4	well_1	F002	93	X/Y distribution	7.824404	7.622504	1.302953
MYH10	W4	HPI4	well_1	F003	177	X/Y distribution	7.072417	6.993477	1.253008
MYH10	W4	HPI4	well_1	F004	250	X/Y distribution	7.081685	7.107163	1.24839
MYH10	W4	HPI4	well_1	F005	205	X/Y distribution	7.313222	7.160289	1.378432
MYH10	W4	HPI4	well_1	F006	174	X/Y distribution	7.767426	7.673501	1.151417
MYH10	W4	HPI4	well_2	F001	48	X/Y distribution	9.372625	9.340995	1.595331
MYH10	W4	HPI4	well_2	F002	58	X/Y distribution	9.945146	9.982851	2.095271
MYH10	W4	HPI4	well_2	F003	165	X/Y distribution	6.909112	6.796083	1.531064
MYH10	W4	HPI4	well_2	F004	223	X/Y distribution	7.19284	7.000331	1.408393
MYH10	W4	HPI4	well_2	F005	239	X/Y distribution	7.060289	7.032407	1.148928
MYH10	W4	HPI4	well_2	F006	162	X/Y distribution	7.868706	7.880811	1.434067
MYH10	W4	HPI4	well_3	F001	33	X/Y distribution	10.87122	11.10479	1.628785
MYH10	W4	HPI4	well_3	F002	46	X/Y distribution	10.40395	10.15554	1.146778
MYH10	W4	HPI4	well_3	F003	159	X/Y distribution	7.83281	7.670507	1.587049
MYH10	W4	HPI4	well_3	F004	172	X/Y distribution	7.720129	7.540789	1.488684
MYH10	W4	HPI4	well_3	F005	164	X/Y distribution	8.10509	8.012981	1.387016
MYH10	W4	HPI4	well_3	F006	162	X/Y distribution	7.749995	7.491493	1.309515

MYH10	W4	HPI4	well_4	F001	114	X/Y distribution	8.462605	8.370966	1.340169
MYH10	W4	HPI4	well_4	F002	157	X/Y distribution	7.791583	7.705671	1.504332
MYH10	W4	HPI4	well_4	F003	114	X/Y distribution	8.505277	8.389022	1.488571
MYH10	W4	HPI4	well_4	F004	202	X/Y distribution	7.15811	6.907973	1.614081
MYH10	W4	HPI4	well_4	F005	139	X/Y distribution	7.297129	7.084223	1.783224
MYH10	W4	HPI4	well_4	F006	115	X/Y distribution	8.871866	8.825047	1.876141
MYH10	W4	HPI4	well_5	F001	131	X/Y distribution	8.654173	8.741851	1.409275
MYH10	W4	HPI4	well_5	F002	140	X/Y distribution	8.602492	8.554512	1.225743
MYH10	W4	HPI4	well_5	F003	145	X/Y distribution	8.484285	8.302146	1.547344
MYH10	W4	HPI4	well_5	F004	207	X/Y distribution	7.301034	7.240969	1.25173
MYH10	W4	HPI4	well_5	F005	237	X/Y distribution	6.821194	6.688121	1.425891
MYH10	W4	HPI4	well_5	F006	90	X/Y distribution	9.465226	9.341576	1.259513
MYH10	W4	PGE2	well_1	F001	315	X/Y distribution	6.137548	6.057096	0.72457
MYH10	W4	PGE2	well_1	F002	196	X/Y distribution	6.199373	6.004256	0.905942
MYH10	W4	PGE2	well_1	F003	251	X/Y distribution	6.018079	5.930727	0.738177
MYH10	W4	PGE2	well_1	F004	174	X/Y distribution	5.509152	5.500089	0.531684
MYH10	W4	PGE2	well_1	F005	327	X/Y distribution	5.783266	5.717659	0.795175
MYH10	W4	PGE2	well_1	F006	257	X/Y distribution	5.772568	5.711525	0.800493
MYH10	W4	PGE2	well_2	F001	168	X/Y distribution	6.087529	6.010797	0.623337
MYH10	W4	PGE2	well_2	F002	291	X/Y distribution	5.959967	5.875786	0.69933
MYH10	W4	PGE2	well_2	F003	88	X/Y distribution	5.453982	5.44662	0.703374
MYH10	W4	PGE2	well_2	F004	151	X/Y distribution	6.156262	6.103667	0.700785
MYH10	W4	PGE2	well_2	F005	404	X/Y distribution	5.462015	5.38547	0.769404
MYH10	W4	PGE2	well_2	F006	316	X/Y distribution	5.861761	5.764454	0.818212
MYH10	W4	PGE2	well_3	F001	215	X/Y distribution	6.334547	6.19505	0.726421
MYH10	W4	PGE2	well_3	F002	290	X/Y distribution	5.730493	5.625545	0.692584
MYH10	W4	PGE2	well_3	F003	354	X/Y distribution	5.908831	5.868808	0.827977
MYH10	W4	PGE2	well_3	F004	253	X/Y distribution	5.546319	5.469661	0.731728
MYH10	W4	PGE2	well_3	F005	224	X/Y distribution	6.213479	6.157174	0.697844
MYH10	W4	PGE2	well_3	F006	283	X/Y distribution	5.705061	5.682581	0.618062
MYH10	W4	PGE2	well_4	F001	268	X/Y distribution	6.264693	6.194786	0.69915
MYH10	W4	PGE2	well_4	F002	162	X/Y distribution	6.285992	6.18492	0.809712
MYH10	W4	PGE2	well_4	F003	297	X/Y distribution	5.848586	5.74728	0.786846
MYH10	W4	PGE2	well_4	F004	239	X/Y distribution	5.402766	5.360791	0.607917
MYH10	W4	PGE2	well_4	F005	349	X/Y distribution	5.812125	5.727658	0.631531
MYH10	W4	PGE2	well_4	F006	291	X/Y distribution	5.425786	5.362013	0.73758
MYH10	W4	PGE2	well_5	F001	248	X/Y distribution	6.264203	6.185728	0.685914
MYH10	W4	PGE2	well_5	F002	375	X/Y distribution	5.516966	5.436921	0.734169
MYH10	W4	PGE2	well_5	F003	176	X/Y distribution	6.095095	5.971792	0.797449
MYH10	W4	PGE2	well_5	F004	367	X/Y distribution	5.897315	5.903028	0.751656
MYH10	W4	PGE2	well_5	F005	179	X/Y distribution	5.759769	5.753028	0.631002
MYH10	W4	PGE2	well_5	F006	210	X/Y distribution	5.991286	5.899171	0.828031
MYH10	W1	HPI4	well_1	F001	148	Z distribution	-0.09397	-0.04957	0.271187
MYH10	W1	HPI4	well_1	F002	167	Z distribution	-0.22357	-0.22855	0.269327
MYH10	W1	HPI4	well_1	F003	161	Z distribution	-0.49866	-0.40247	0.420134
MYH10	W1	HPI4	well_1	F004	280	Z distribution	-0.25276	-0.1843	0.41195
MYH10	W1	HPI4	well_1	F005	180	Z distribution	-0.30814	-0.25802	0.341505
MYH10	W1	HPI4	well_1	F006	104	Z distribution	-0.27695	-0.28081	0.269077
MYH10	W1	HPI4	well_2	F001	124	Z distribution	-0.03045	-0.03935	0.258215

MYH10	W1	HPI4	well_2	F002	150	Z distribution	-0.02191	0.027085	0.261429
MYH10	W1	HPI4	well_2	F003	192	Z distribution	-0.0931	-0.08834	0.284831
MYH10	W1	HPI4	well_2	F004	203	Z distribution	-0.04189	0.010645	0.275802
MYH10	W1	HPI4	well_2	F005	166	Z distribution	-0.17771	-0.15967	0.249194
MYH10	W1	HPI4	well_2	F006	129	Z distribution	-0.14761	-0.09852	0.335632
MYH10	W1	HPI4	well_3	F001	118	Z distribution	-0.06024	-0.04326	0.195609
MYH10	W1	HPI4	well_3	F002	194	Z distribution	-0.13615	-0.1306	0.272779
MYH10	W1	HPI4	well_3	F003	115	Z distribution	-0.22508	-0.20609	0.272061
MYH10	W1	HPI4	well_3	F004	98	Z distribution	-0.11195	-0.08994	0.264228
MYH10	W1	HPI4	well_3	F005	123	Z distribution	-0.17385	-0.14	0.256797
MYH10	W1	HPI4	well_3	F006	212	Z distribution	-0.21153	-0.19291	0.321314
MYH10	W1	HPI4	well_4	F001	81	Z distribution	-0.0106	0.029776	0.20498
MYH10	W1	HPI4	well_4	F002	266	Z distribution	-0.27635	-0.21589	0.39545
MYH10	W1	HPI4	well_4	F003	158	Z distribution	-0.04223	-0.06333	0.28672
MYH10	W1	HPI4	well_4	F004	186	Z distribution	-0.2451	-0.18616	0.322726
MYH10	W1	HPI4	well_4	F005	126	Z distribution	-0.1942	-0.20759	0.238242
MYH10	W1	HPI4	well_4	F006	114	Z distribution	-0.10892	-0.08346	0.260018
MYH10	W1	HPI4	well_5	F001	167	Z distribution	0.023432	0.004304	0.259481
MYH10	W1	HPI4	well_5	F002	143	Z distribution	-0.31818	-0.27213	0.303839
MYH10	W1	HPI4	well_5	F003	222	Z distribution	-0.09187	-0.06499	0.266448
MYH10	W1	HPI4	well_5	F004	184	Z distribution	-0.12637	-0.10341	0.232975
MYH10	W1	HPI4	well_5	F005	164	Z distribution	-0.08951	-0.08522	0.210133
MYH10	W1	HPI4	well_5	F006	148	Z distribution	-0.12814	-0.10298	0.301378
MYH10	W1	PGE2	well_1	F001	145	Z distribution	-0.19272	-0.1489	0.263144
MYH10	W1	PGE2	well_1	F002	91	Z distribution	-0.30984	-0.30799	0.289507
MYH10	W1	PGE2	well_1	F003	250	Z distribution	-0.07881	-0.06598	0.31064
MYH10	W1	PGE2	well_1	F004	393	Z distribution	0.046829	0.068253	0.336943
MYH10	W1	PGE2	well_1	F005	286	Z distribution	-0.0779	-0.0687	0.294543
MYH10	W1	PGE2	well_1	F006	145	Z distribution	0.044346	0.073836	0.210307
MYH10	W1	PGE2	well_2	F001	219	Z distribution	-0.04119	-0.02764	0.28106
MYH10	W1	PGE2	well_2	F002	184	Z distribution	-0.0837	-0.07502	0.266777
MYH10	W1	PGE2	well_2	F003	177	Z distribution	-0.31886	-0.27063	0.36488
MYH10	W1	PGE2	well_2	F004	158	Z distribution	-0.15941	-0.12878	0.322509
MYH10	W1	PGE2	well_2	F005	232	Z distribution	-0.1067	-0.10722	0.290947
MYH10	W1	PGE2	well_2	F006	221	Z distribution	-0.09167	-0.03896	0.33701
MYH10	W1	PGE2	well_3	F001	129	Z distribution	-0.1223	-0.09659	0.298487
MYH10	W1	PGE2	well_3	F002	131	Z distribution	-0.24007	-0.18391	0.263558
MYH10	W1	PGE2	well_3	F003	222	Z distribution	-0.42712	-0.37006	0.489225
MYH10	W1	PGE2	well_3	F004	175	Z distribution	-0.1172	-0.08601	0.287238
MYH10	W1	PGE2	well_3	F005	132	Z distribution	-0.36987	-0.31227	0.34309
MYH10	W1	PGE2	well_3	F006	147	Z distribution	-0.06907	-0.03987	0.253991
MYH10	W1	PGE2	well_4	F001	129	Z distribution	-0.06361	-0.00979	0.284917
MYH10	W1	PGE2	well_4	F002	210	Z distribution	-0.15217	-0.11841	0.256945
MYH10	W1	PGE2	well_4	F003	116	Z distribution	-0.24575	-0.20302	0.285995
MYH10	W1	PGE2	well_4	F004	99	Z distribution	-0.257	-0.23239	0.252657
MYH10	W1	PGE2	well_4	F005	153	Z distribution	-0.12059	-0.1075	0.233234
MYH10	W1	PGE2	well_4	F006	223	Z distribution	-0.16079	-0.16074	0.27161
MYH10	W1	PGE2	well_5	F001	105	Z distribution	-0.1073	-0.08392	0.235238
MYH10	W1	PGE2	well_5	F002	132	Z distribution	-0.16893	-0.16321	0.254596

MYH10	W1	PGE2	well_5	F003	233	Z distribution	-0.22162	-0.18089	0.368261
MYH10	W1	PGE2	well_5	F004	179	Z distribution	-0.15452	-0.10958	0.306741
MYH10	W1	PGE2	well_5	F005	190	Z distribution	-0.08949	-0.06848	0.298578
MYH10	W1	PGE2	well_5	F006	168	Z distribution	-0.14903	-0.10002	0.290424
MYH10	W2	HPI4	well_1	F001	163	Z distribution	0.135222	0.162331	0.213951
MYH10	W2	HPI4	well_1	F002	160	Z distribution	0.096128	0.168838	0.297754
MYH10	W2	HPI4	well_1	F003	150	Z distribution	0.179956	0.187059	0.221586
MYH10	W2	HPI4	well_1	F004	250	Z distribution	0.026446	0.056377	0.287531
MYH10	W2	HPI4	well_1	F005	219	Z distribution	0.244119	0.252147	0.243339
MYH10	W2	HPI4	well_1	F006	165	Z distribution	0.030088	0.044171	0.247459
MYH10	W2	HPI4	well_2	F001	156	Z distribution	-0.08907	-0.05442	0.397538
MYH10	W2	HPI4	well_2	F002	155	Z distribution	0.085762	0.070368	0.264034
MYH10	W2	HPI4	well_2	F003	191	Z distribution	-0.00744	0.039962	0.21752
MYH10	W2	HPI4	well_2	F004	180	Z distribution	0.100617	0.090996	0.254574
MYH10	W2	HPI4	well_2	F005	148	Z distribution	0.134672	0.11308	0.238316
MYH10	W2	HPI4	well_2	F006	133	Z distribution	-0.08067	-0.09318	0.237752
MYH10	W2	HPI4	well_3	F001	100	Z distribution	0.04627	0.064632	0.253221
MYH10	W2	HPI4	well_3	F002	212	Z distribution	-0.05604	-0.03934	0.292234
MYH10	W2	HPI4	well_3	F003	218	Z distribution	-0.09783	-0.06571	0.246571
MYH10	W2	HPI4	well_3	F004	125	Z distribution	0.053018	0.082502	0.212831
MYH10	W2	HPI4	well_3	F005	183	Z distribution	0.145011	0.177847	0.282395
MYH10	W2	HPI4	well_3	F006	113	Z distribution	0.050889	0.061863	0.222944
MYH10	W2	HPI4	well_4	F001	170	Z distribution	0.11187	0.144862	0.313212
MYH10	W2	HPI4	well_4	F002	209	Z distribution	-0.06575	-0.02772	0.320629
MYH10	W2	HPI4	well_4	F003	235	Z distribution	0.039691	0.097314	0.2488
MYH10	W2	HPI4	well_4	F004	333	Z distribution	0.132152	0.145304	0.301399
MYH10	W2	HPI4	well_4	F005	198	Z distribution	0.171302	0.17666	0.21701
MYH10	W2	HPI4	well_4	F006	91	Z distribution	-0.02393	0.031293	0.242577
MYH10	W2	HPI4	well_5	F001	121	Z distribution	-0.08215	-0.04837	0.278159
MYH10	W2	HPI4	well_5	F002	156	Z distribution	0.037435	0.053806	0.241345
MYH10	W2	HPI4	well_5	F003	252	Z distribution	0.040083	0.057123	0.238173
MYH10	W2	HPI4	well_5	F004	306	Z distribution	0.159349	0.161795	0.250726
MYH10	W2	HPI4	well_5	F005	250	Z distribution	0.172698	0.180807	0.244664
MYH10	W2	HPI4	well_5	F006	160	Z distribution	0.133335	0.120288	0.29589
MYH10	W2	PGE2	well_1	F001	49	Z distribution	0.258538	0.251317	0.229367
MYH10	W2	PGE2	well_1	F002	70	Z distribution	0.122886	0.165643	0.2422
MYH10	W2	PGE2	well_1	F003	149	Z distribution	0.124508	0.175505	0.350134
MYH10	W2	PGE2	well_1	F004	318	Z distribution	0.312584	0.281793	0.325781
MYH10	W2	PGE2	well_1	F005	207	Z distribution	0.282686	0.267842	0.263607
MYH10	W2	PGE2	well_1	F006	103	Z distribution	0.36862	0.32588	0.307855
MYH10	W2	PGE2	well_2	F001	257	Z distribution	0.346138	0.348377	0.272141
MYH10	W2	PGE2	well_2	F002	384	Z distribution	0.223082	0.229176	0.278525
MYH10	W2	PGE2	well_2	F003	118	Z distribution	0.257161	0.278522	0.267758
MYH10	W2	PGE2	well_2	F004	254	Z distribution	0.209143	0.180291	0.340803
MYH10	W2	PGE2	well_2	F005	42	Z distribution	0.333509	0.296229	0.2722
MYH10	W2	PGE2	well_3	F001	261	Z distribution	0.183035	0.180805	0.324475
MYH10	W2	PGE2	well_3	F002	398	Z distribution	0.218121	0.177875	0.336366
MYH10	W2	PGE2	well_3	F003	206	Z distribution	0.261991	0.262197	0.263063
MYH10	W2	PGE2	well_3	F004	360	Z distribution	0.160213	0.119535	0.332527

MYH10	W2	PGE2	well_3	F005	277	Z distribution	0.256198	0.238001	0.248995
MYH10	W2	PGE2	well_3	F006	218	Z distribution	0.271518	0.272866	0.263485
MYH10	W2	PGE2	well_4	F001	175	Z distribution	0.165141	0.162823	0.247165
MYH10	W2	PGE2	well_4	F002	320	Z distribution	0.29106	0.301569	0.39302
MYH10	W2	PGE2	well_4	F003	319	Z distribution	0.39214	0.397851	0.269651
MYH10	W2	PGE2	well_4	F004	356	Z distribution	0.185691	0.181913	0.355036
MYH10	W2	PGE2	well_4	F005	347	Z distribution	0.41045	0.401234	0.357701
MYH10	W2	PGE2	well_4	F006	382	Z distribution	0.182226	0.152411	0.318242
MYH10	W2	PGE2	well_5	F001	173	Z distribution	0.234403	0.18917	0.298953
MYH10	W2	PGE2	well_5	F002	335	Z distribution	0.35764	0.37618	0.29642
MYH10	W2	PGE2	well_5	F003	391	Z distribution	0.217786	0.211122	0.306797
MYH10	W2	PGE2	well_5	F004	414	Z distribution	0.352712	0.353124	0.376247
MYH10	W2	PGE2	well_5	F005	292	Z distribution	0.144895	0.128502	0.306063
MYH10	W2	PGE2	well_5	F006	329	Z distribution	0.262523	0.243147	0.421316
MYH10	W3	HPI4	well_1	F001	139	Z distribution	0.067991	0.070118	0.256572
MYH10	W3	HPI4	well_1	F002	206	Z distribution	-0.00525	0.014314	0.250045
MYH10	W3	HPI4	well_1	F003	196	Z distribution	0.018427	0.047911	0.260573
MYH10	W3	HPI4	well_1	F004	181	Z distribution	0.139258	0.155923	0.258419
MYH10	W3	HPI4	well_1	F005	110	Z distribution	0.141073	0.175285	0.156366
MYH10	W3	HPI4	well_1	F006	154	Z distribution	0.046045	0.088192	0.22029
MYH10	W3	HPI4	well_2	F001	97	Z distribution	0.125501	0.158249	0.221812
MYH10	W3	HPI4	well_2	F002	182	Z distribution	0.033891	0.04639	0.238616
MYH10	W3	HPI4	well_2	F003	163	Z distribution	-0.00214	0.056608	0.247433
MYH10	W3	HPI4	well_2	F004	189	Z distribution	0.194922	0.205846	0.224259
MYH10	W3	HPI4	well_2	F005	267	Z distribution	0.190782	0.206808	0.269322
MYH10	W3	HPI4	well_2	F006	122	Z distribution	0.22201	0.251964	0.216846
MYH10	W3	HPI4	well_3	F001	60	Z distribution	0.108951	0.137555	0.189678
MYH10	W3	HPI4	well_3	F002	164	Z distribution	0.126911	0.115862	0.212095
MYH10	W3	HPI4	well_3	F003	203	Z distribution	0.119086	0.127115	0.254589
MYH10	W3	HPI4	well_3	F004	228	Z distribution	0.118112	0.132728	0.197383
MYH10	W3	HPI4	well_3	F005	341	Z distribution	0.132256	0.121352	0.2705
MYH10	W3	HPI4	well_3	F006	131	Z distribution	0.257932	0.272075	0.194189
MYH10	W3	HPI4	well_4	F001	99	Z distribution	0.15834	0.181855	0.201646
MYH10	W3	HPI4	well_4	F002	175	Z distribution	0.160213	0.168243	0.183574
MYH10	W3	HPI4	well_4	F003	152	Z distribution	0.198954	0.224827	0.214827
MYH10	W3	HPI4	well_4	F004	188	Z distribution	0.174268	0.188112	0.189159
MYH10	W3	HPI4	well_4	F005	166	Z distribution	0.112157	0.146122	0.245389
MYH10	W3	HPI4	well_4	F006	78	Z distribution	0.101524	0.12048	0.209769
MYH10	W3	HPI4	well_5	F001	62	Z distribution	0.106062	0.102747	0.185799
MYH10	W3	HPI4	well_5	F002	128	Z distribution	0.121947	0.170648	0.208602
MYH10	W3	HPI4	well_5	F003	105	Z distribution	0.090456	0.092378	0.181648
MYH10	W3	HPI4	well_5	F004	63	Z distribution	0.196268	0.187879	0.140782
MYH10	W3	HPI4	well_5	F005	179	Z distribution	0.241236	0.21012	0.289026
MYH10	W3	HPI4	well_5	F006	169	Z distribution	0.153001	0.135883	0.218527
MYH10	W3	PGE2	well_1	F001	354	Z distribution	0.143555	0.144348	0.27874
MYH10	W3	PGE2	well_1	F002	355	Z distribution	0.162579	0.161648	0.26603
MYH10	W3	PGE2	well_1	F003	397	Z distribution	0.151455	0.163047	0.336181
MYH10	W3	PGE2	well_1	F004	275	Z distribution	0.109861	0.12275	0.332467
MYH10	W3	PGE2	well_1	F005	227	Z distribution	0.187192	0.171308	0.331286

MYH10	W3	PGE2	well_2	F001	271	Z distribution	0.198632	0.184794	0.284125
MYH10	W3	PGE2	well_2	F002	245	Z distribution	0.146483	0.120734	0.284154
MYH10	W3	PGE2	well_2	F003	364	Z distribution	0.198327	0.225827	0.294298
MYH10	W3	PGE2	well_2	F004	225	Z distribution	-0.02533	-0.01543	0.26076
MYH10	W3	PGE2	well_2	F005	358	Z distribution	0.160358	0.161864	0.290978
MYH10	W3	PGE2	well_2	F006	351	Z distribution	0.292828	0.323284	0.304858
MYH10	W3	PGE2	well_3	F001	250	Z distribution	0.086589	0.075953	0.283404
MYH10	W3	PGE2	well_3	F002	124	Z distribution	0.009792	0.0317	0.241358
MYH10	W3	PGE2	well_3	F003	255	Z distribution	-0.05027	-0.02921	0.279166
MYH10	W3	PGE2	well_3	F004	171	Z distribution	0.126276	0.125896	0.30535
MYH10	W3	PGE2	well_3	F005	312	Z distribution	-0.09145	-0.1005	0.361817
MYH10	W3	PGE2	well_3	F006	265	Z distribution	-0.06682	-0.06171	0.301941
MYH10	W3	PGE2	well_4	F001	110	Z distribution	-0.22892	-0.16469	0.381813
MYH10	W3	PGE2	well_4	F002	178	Z distribution	-0.169	-0.12556	0.389319
MYH10	W3	PGE2	well_4	F003	273	Z distribution	-0.08098	-0.08229	0.385231
MYH10	W3	PGE2	well_4	F004	73	Z distribution	0.068605	0.052536	0.24533
MYH10	W3	PGE2	well_4	F005	155	Z distribution	-0.04081	-0.00463	0.329795
MYH10	W3	PGE2	well_4	F006	390	Z distribution	-0.00921	0.001084	0.386331
MYH10	W3	PGE2	well_5	F001	252	Z distribution	0.109252	0.123832	0.342944
MYH10	W3	PGE2	well_5	F002	254	Z distribution	-0.09182	-0.06821	0.373198
MYH10	W3	PGE2	well_5	F003	338	Z distribution	-0.17325	-0.14236	0.406611
MYH10	W3	PGE2	well_5	F004	200	Z distribution	-0.0693	-0.05266	0.302961
MYH10	W3	PGE2	well_5	F005	285	Z distribution	-0.18558	-0.15257	0.348729
MYH10	W3	PGE2	well_5	F006	198	Z distribution	-0.43704	-0.40792	0.455275
MYH10	W4	HPI4	well_1	F001	104	Z distribution	0.224895	0.221424	0.152317
MYH10	W4	HPI4	well_1	F002	93	Z distribution	0.19993	0.176878	0.145727
MYH10	W4	HPI4	well_1	F003	177	Z distribution	0.26418	0.27316	0.201765
MYH10	W4	HPI4	well_1	F004	250	Z distribution	0.092504	0.104748	0.211926
MYH10	W4	HPI4	well_1	F005	205	Z distribution	0.242133	0.240613	0.178795
MYH10	W4	HPI4	well_1	F006	174	Z distribution	0.218579	0.22648	0.149678
MYH10	W4	HPI4	well_2	F001	48	Z distribution	0.11594	0.120677	0.127753
MYH10	W4	HPI4	well_2	F002	58	Z distribution	0.23067	0.22234	0.153634
MYH10	W4	HPI4	well_2	F003	165	Z distribution	0.109575	0.116863	0.257676
MYH10	W4	HPI4	well_2	F004	223	Z distribution	0.137076	0.175699	0.25478
MYH10	W4	HPI4	well_2	F005	239	Z distribution	0.302524	0.303364	0.192641
MYH10	W4	HPI4	well_2	F006	162	Z distribution	0.171562	0.197687	0.151236
MYH10	W4	HPI4	well_3	F001	33	Z distribution	0.106955	0.095785	0.149113
MYH10	W4	HPI4	well_3	F002	46	Z distribution	0.026746	0.022771	0.153658
MYH10	W4	HPI4	well_3	F003	159	Z distribution	0.145049	0.157744	0.195114
MYH10	W4	HPI4	well_3	F004	172	Z distribution	0.106667	0.153153	0.244907
MYH10	W4	HPI4	well_3	F005	164	Z distribution	0.156121	0.173976	0.166651
MYH10	W4	HPI4	well_3	F006	162	Z distribution	0.103478	0.14458	0.238733
MYH10	W4	HPI4	well_4	F001	114	Z distribution	0.213915	0.217464	0.153903
MYH10	W4	HPI4	well_4	F002	157	Z distribution	0.03243	0.044721	0.229814
MYH10	W4	HPI4	well_4	F003	114	Z distribution	0.143523	0.179009	0.255169
MYH10	W4	HPI4	well_4	F004	202	Z distribution	0.055002	0.047174	0.254563
MYH10	W4	HPI4	well_4	F005	139	Z distribution	0.121343	0.138577	0.26256
MYH10	W4	HPI4	well_4	F006	115	Z distribution	-0.02166	0.052697	0.262588
MYH10	W4	HPI4	well_5	F001	131	Z distribution	-0.07352	0.016033	0.309673

MYH10	W4	HPI4	well_5	F002	140	Z distribution	0.128386	0.192331	0.235044
MYH10	W4	HPI4	well_5	F003	145	Z distribution	0.038856	0.070283	0.230361
MYH10	W4	HPI4	well_5	F004	207	Z distribution	0.182604	0.168689	0.213468
MYH10	W4	HPI4	well_5	F005	237	Z distribution	0.042456	0.09064	0.268948
MYH10	W4	HPI4	well_5	F006	90	Z distribution	0.04419	0.049044	0.141706
MYH10	W4	PGE2	well_1	F001	315	Z distribution	0.104643	0.104685	0.310843
MYH10	W4	PGE2	well_1	F002	196	Z distribution	-0.01033	0.028314	0.403262
MYH10	W4	PGE2	well_1	F003	251	Z distribution	-0.1379	-0.0771	0.408045
MYH10	W4	PGE2	well_1	F004	174	Z distribution	-0.14013	-0.13032	0.285691
MYH10	W4	PGE2	well_1	F005	327	Z distribution	-0.20204	-0.15987	0.354848
MYH10	W4	PGE2	well_1	F006	257	Z distribution	-0.30171	-0.27155	0.466361
MYH10	W4	PGE2	well_2	F001	168	Z distribution	-0.05891	-0.03699	0.30493
MYH10	W4	PGE2	well_2	F002	291	Z distribution	-0.34678	-0.3013	0.413948
MYH10	W4	PGE2	well_2	F003	88	Z distribution	0.163244	0.129363	0.299658
MYH10	W4	PGE2	well_2	F004	151	Z distribution	-0.04477	-0.00303	0.298278
MYH10	W4	PGE2	well_2	F005	404	Z distribution	-0.15311	-0.12548	0.435295
MYH10	W4	PGE2	well_2	F006	316	Z distribution	-0.05974	-0.03167	0.327083
MYH10	W4	PGE2	well_3	F001	215	Z distribution	-0.24435	-0.24162	0.378465
MYH10	W4	PGE2	well_3	F002	290	Z distribution	-0.06605	-0.0485	0.362958
MYH10	W4	PGE2	well_3	F003	354	Z distribution	-0.07895	0.024451	0.471877
MYH10	W4	PGE2	well_3	F004	253	Z distribution	-0.17503	-0.12729	0.425308
MYH10	W4	PGE2	well_3	F005	224	Z distribution	-0.05717	-0.02219	0.308448
MYH10	W4	PGE2	well_3	F006	283	Z distribution	-0.21844	-0.16658	0.419169
MYH10	W4	PGE2	well_4	F001	268	Z distribution	-0.13627	-0.10079	0.387768
MYH10	W4	PGE2	well_4	F002	162	Z distribution	-0.02011	-0.02505	0.248613
MYH10	W4	PGE2	well_4	F003	297	Z distribution	0.104262	0.078793	0.314723
MYH10	W4	PGE2	well_4	F004	239	Z distribution	-0.16158	-0.11654	0.415869
MYH10	W4	PGE2	well_4	F005	349	Z distribution	-0.01215	-0.01001	0.360225
MYH10	W4	PGE2	well_4	F006	291	Z distribution	0.051252	0.114308	0.376041
MYH10	W4	PGE2	well_5	F001	248	Z distribution	0.064215	0.082281	0.27575
MYH10	W4	PGE2	well_5	F002	375	Z distribution	-0.02917	-0.01082	0.371756
MYH10	W4	PGE2	well_5	F003	176	Z distribution	-0.09242	-0.0405	0.332644
MYH10	W4	PGE2	well_5	F004	367	Z distribution	0.034361	0.028463	0.345977
MYH10	W4	PGE2	well_5	F005	179	Z distribution	-0.20527	-0.16431	0.380848
MYH10	W4	PGE2	well_5	F006	210	Z distribution	-0.29007	-0.20316	0.397487
FBL	W1	HPI4	well_1	F001	368	Avg. Volume	5.124188	4.600556	2.410859
FBL	W1	HPI4	well_1	F002	368	Avg. Volume	5.22938	4.534051	2.782454
FBL	W1	HPI4	well_1	F003	421	Avg. Volume	4.701206	4.225	2.347623
FBL	W1	HPI4	well_1	F004	346	Avg. Volume	5.455232	5.049462	2.448039
FBL	W1	HPI4	well_1	F005	343	Avg. Volume	4.835888	4.514491	2.093512
FBL	W1	HPI4	well_1	F006	332	Avg. Volume	5.389774	5.091516	2.561562
FBL	W1	HPI4	well_2	F001	332	Avg. Volume	5.035494	4.562413	2.418421
FBL	W1	HPI4	well_2	F002	204	Avg. Volume	6.07676	5.821111	2.572468
FBL	W1	HPI4	well_2	F003	431	Avg. Volume	4.252352	3.927685	2.036249
FBL	W1	HPI4	well_2	F004	310	Avg. Volume	5.452364	5.058264	2.791314
FBL	W1	HPI4	well_2	F005	331	Avg. Volume	4.949903	4.424514	2.526658
FBL	W1	HPI4	well_2	F006	360	Avg. Volume	4.677192	4.219132	2.498626
FBL	W1	HPI4	well_3	F001	279	Avg. Volume	4.527678	4.154583	2.214211
FBL	W1	HPI4	well_3	F002	266	Avg. Volume	5.58357	5.203792	2.543119

FBL	W1	HPI4	well_3	F003	410	Avg. Volume	5.052561	4.700313	2.337198
FBL	W1	HPI4	well_3	F004	317	Avg. Volume	5.375478	4.776597	2.849787
FBL	W1	HPI4	well_3	F005	372	Avg. Volume	4.07779	4.04337	1.943806
FBL	W1	HPI4	well_3	F006	285	Avg. Volume	5.603799	4.991759	2.66248
FBL	W1	HPI4	well_4	F001	311	Avg. Volume	5.575537	5.093472	2.525747
FBL	W1	HPI4	well_4	F002	331	Avg. Volume	5.833749	5.621597	2.648228
FBL	W1	HPI4	well_4	F003	396	Avg. Volume	4.693568	4.327691	2.413296
FBL	W1	HPI4	well_4	F004	445	Avg. Volume	4.922897	4.545787	2.526628
FBL	W1	HPI4	well_4	F005	228	Avg. Volume	5.645008	5.196163	2.220002
FBL	W1	HPI4	well_4	F006	324	Avg. Volume	5.425848	5.058683	2.471707
FBL	W1	HPI4	well_5	F001	179	Avg. Volume	5.173958	4.536007	2.638353
FBL	W1	HPI4	well_5	F002	290	Avg. Volume	6.099816	5.771233	2.596044
FBL	W1	HPI4	well_5	F003	412	Avg. Volume	4.963057	4.553611	2.394283
FBL	W1	HPI4	well_5	F004	444	Avg. Volume	4.536025	4.064606	2.437575
FBL	W1	HPI4	well_5	F005	309	Avg. Volume	5.292941	4.955573	2.496526
FBL	W1	HPI4	well_5	F006	280	Avg. Volume	5.069376	4.761927	2.20072
FBL	W1	PGE2	well_1	F001	448	Avg. Volume	5.100781	4.815718	2.249173
FBL	W1	PGE2	well_1	F002	186	Avg. Volume	5.984357	5.475874	2.665801
FBL	W1	PGE2	well_1	F003	265	Avg. Volume	5.291869	4.680361	2.782446
FBL	W1	PGE2	well_1	F004	334	Avg. Volume	5.150174	4.827454	2.498374
FBL	W1	PGE2	well_1	F005	429	Avg. Volume	4.327888	3.951157	2.020246
FBL	W1	PGE2	well_1	F006	379	Avg. Volume	4.623994	4.213264	2.120389
FBL	W1	PGE2	well_2	F001	240	Avg. Volume	6.230851	5.73309	2.995809
FBL	W1	PGE2	well_2	F002	367	Avg. Volume	5.536358	5.210833	2.677334
FBL	W1	PGE2	well_2	F003	415	Avg. Volume	4.8384	4.489063	2.461428
FBL	W1	PGE2	well_2	F004	430	Avg. Volume	4.690967	4.227934	2.242459
FBL	W1	PGE2	well_2	F005	333	Avg. Volume	4.668462	4.3095	2.134814
FBL	W1	PGE2	well_2	F006	258	Avg. Volume	5.693915	5.102274	2.655856
FBL	W1	PGE2	well_3	F001	325	Avg. Volume	5.324396	5.02775	2.511733
FBL	W1	PGE2	well_3	F002	254	Avg. Volume	5.850728	5.275382	3.023754
FBL	W1	PGE2	well_3	F003	353	Avg. Volume	5.319862	4.805938	2.450689
FBL	W1	PGE2	well_3	F004	376	Avg. Volume	4.364136	3.992234	2.076767
FBL	W1	PGE2	well_3	F005	364	Avg. Volume	5.16623	4.850926	2.416391
FBL	W1	PGE2	well_3	F006	177	Avg. Volume	5.887965	5.163889	3.062292
FBL	W1	PGE2	well_4	F001	264	Avg. Volume	4.392868	3.987931	2.192174
FBL	W1	PGE2	well_4	F002	360	Avg. Volume	5.106704	4.776597	2.338285
FBL	W1	PGE2	well_4	F003	338	Avg. Volume	4.472449	4.189792	2.137523
FBL	W1	PGE2	well_4	F004	350	Avg. Volume	5.469774	5.086626	2.404023
FBL	W1	PGE2	well_4	F005	225	Avg. Volume	6.098957	5.897396	2.630424
FBL	W1	PGE2	well_4	F006	328	Avg. Volume	5.542018	5.076846	2.67322
FBL	W1	PGE2	well_5	F001	415	Avg. Volume	4.606002	4.193704	2.318705
FBL	W1	PGE2	well_5	F002	307	Avg. Volume	5.230041	4.741389	2.618124
FBL	W1	PGE2	well_5	F003	282	Avg. Volume	4.954367	4.759971	2.021182
FBL	W1	PGE2	well_5	F004	319	Avg. Volume	4.924986	4.624028	2.171985
FBL	W1	PGE2	well_5	F005	340	Avg. Volume	5.027008	4.66217	2.388459
FBL	W1	PGE2	well_5	F006	312	Avg. Volume	4.715994	4.322997	2.29522
FBL	W2	HPI4	well_1	F001	172	Avg. Volume	4.786542	4.584907	2.78449
FBL	W2	HPI4	well_1	F002	162	Avg. Volume	8.060519	7.616736	3.047274
FBL	W2	HPI4	well_1	F003	151	Avg. Volume	6.588447	5.915	3.288556

FBL	W2	HPI4	well_1	F004	174	Avg. Volume	6.486241	6.049965	3.085608
FBL	W2	HPI4	well_1	F005	175	Avg. Volume	6.795731	6.56831	3.089343
FBL	W2	HPI4	well_1	F006	149	Avg. Volume	7.323177	6.443125	3.12675
FBL	W2	HPI4	well_2	F001	128	Avg. Volume	4.305571	3.732083	2.857918
FBL	W2	HPI4	well_2	F002	177	Avg. Volume	5.955638	5.695926	2.645894
FBL	W2	HPI4	well_2	F003	105	Avg. Volume	6.462495	5.785903	3.085187
FBL	W2	HPI4	well_2	F004	201	Avg. Volume	7.128235	6.537014	3.476519
FBL	W2	HPI4	well_2	F005	215	Avg. Volume	6.299415	6.149722	2.485729
FBL	W2	HPI4	well_2	F006	111	Avg. Volume	5.792028	5.431472	2.4806
FBL	W2	HPI4	well_3	F001	110	Avg. Volume	3.513278	2.894907	2.338591
FBL	W2	HPI4	well_3	F002	32	Avg. Volume	7.592426	7.428958	3.854522
FBL	W2	HPI4	well_3	F003	113	Avg. Volume	6.212741	5.891528	3.478356
FBL	W2	HPI4	well_3	F004	166	Avg. Volume	5.391617	4.770729	3.006923
FBL	W2	HPI4	well_3	F005	109	Avg. Volume	6.472615	5.809375	3.440999
FBL	W2	HPI4	well_3	F006	81	Avg. Volume	5.213057	4.835278	2.960918
FBL	W2	HPI4	well_4	F001	126	Avg. Volume	4.443465	3.716435	2.977848
FBL	W2	HPI4	well_4	F002	207	Avg. Volume	6.746396	6.267083	2.948552
FBL	W2	HPI4	well_4	F003	163	Avg. Volume	7.242207	7.14338	3.310703
FBL	W2	HPI4	well_4	F004	149	Avg. Volume	7.692418	6.439213	4.454518
FBL	W2	HPI4	well_4	F005	84	Avg. Volume	7.803273	7.501331	2.79748
FBL	W2	HPI4	well_4	F006	101	Avg. Volume	7.289306	6.724792	3.295889
FBL	W2	HPI4	well_5	F001	68	Avg. Volume	3.448682	3.268507	1.913233
FBL	W2	HPI4	well_5	F002	89	Avg. Volume	6.181623	6.531146	3.054555
FBL	W2	HPI4	well_5	F003	85	Avg. Volume	9.13583	8.841204	3.400452
FBL	W2	HPI4	well_5	F004	135	Avg. Volume	6.803225	6.384444	3.449468
FBL	W2	HPI4	well_5	F005	185	Avg. Volume	6.857574	6.466597	3.226988
FBL	W2	HPI4	well_5	F006	95	Avg. Volume	6.16173	5.809375	3.514562
FBL	W2	PGE2	well_1	F001	128	Avg. Volume	4.727693	4.117028	2.580398
FBL	W2	PGE2	well_1	F002	40	Avg. Volume	3.052405	2.922292	1.442834
FBL	W2	PGE2	well_1	F003	61	Avg. Volume	4.454928	4.166319	2.274442
FBL	W2	PGE2	well_1	F004	117	Avg. Volume	4.008577	3.661667	2.013004
FBL	W2	PGE2	well_1	F005	222	Avg. Volume	4.862796	4.491019	1.966943
FBL	W2	PGE2	well_1	F006	71	Avg. Volume	4.735244	4.260208	2.527349
FBL	W2	PGE2	well_2	F001	74	Avg. Volume	4.246475	3.63526	2.328742
FBL	W2	PGE2	well_2	F002	128	Avg. Volume	4.700156	4.439184	2.416864
FBL	W2	PGE2	well_2	F003	170	Avg. Volume	5.307436	5.107164	2.622522
FBL	W2	PGE2	well_2	F004	108	Avg. Volume	4.450806	4.233802	1.847882
FBL	W2	PGE2	well_2	F005	76	Avg. Volume	4.323175	3.884653	2.087346
FBL	W2	PGE2	well_2	F006	52	Avg. Volume	4.214667	3.985388	1.809247
FBL	W2	PGE2	well_3	F001	54	Avg. Volume	3.488559	3.41912	1.859945
FBL	W2	PGE2	well_3	F002	286	Avg. Volume	4.684894	4.408866	2.146324
FBL	W2	PGE2	well_3	F003	300	Avg. Volume	4.835502	4.393218	2.203001
FBL	W2	PGE2	well_3	F004	172	Avg. Volume	5.00474	4.870486	2.125308
FBL	W2	PGE2	well_3	F005	201	Avg. Volume	4.495443	3.8025	2.653219
FBL	W2	PGE2	well_3	F006	149	Avg. Volume	4.99308	4.788333	2.538049
FBL	W2	PGE2	well_4	F001	222	Avg. Volume	4.104745	3.679271	2.080089
FBL	W2	PGE2	well_4	F002	164	Avg. Volume	5.289202	4.616204	2.99444
FBL	W2	PGE2	well_4	F003	208	Avg. Volume	4.509187	4.297373	1.928751
FBL	W2	PGE2	well_4	F004	262	Avg. Volume	4.691395	4.26412	2.114372

FBL	W2	PGE2	well_4	F005	83	Avg. Volume	4.513927	4.091991	1.897309
FBL	W2	PGE2	well_4	F006	170	Avg. Volume	4.492346	4.041134	2.183805
FBL	W2	PGE2	well_5	F001	159	Avg. Volume	4.856826	4.248472	2.260337
FBL	W2	PGE2	well_5	F002	211	Avg. Volume	4.997296	4.545787	2.328666
FBL	W2	PGE2	well_5	F003	182	Avg. Volume	5.130036	4.755081	2.188467
FBL	W2	PGE2	well_5	F004	132	Avg. Volume	5.136932	4.831366	2.169673
FBL	W2	PGE2	well_5	F005	171	Avg. Volume	3.970914	3.567778	1.920177
FBL	W2	PGE2	well_5	F006	349	Avg. Volume	4.306465	4.102944	1.835937
FBL	W3	HPI4	well_1	F001	56	Avg. Volume	5.896887	5.480764	3.060144
FBL	W3	HPI4	well_1	F002	54	Avg. Volume	9.750581	8.759051	4.427638
FBL	W3	HPI4	well_1	F003	55	Avg. Volume	10.19577	9.189375	3.733685
FBL	W3	HPI4	well_1	F004	281	Avg. Volume	5.164554	4.377569	2.802598
FBL	W3	HPI4	well_1	F005	269	Avg. Volume	6.110918	5.445556	3.312711
FBL	W3	HPI4	well_1	F006	124	Avg. Volume	6.231561	5.645069	2.972948
FBL	W3	HPI4	well_2	F001	31	Avg. Volume	5.161426	3.720347	3.769144
FBL	W3	HPI4	well_2	F002	34	Avg. Volume	10.3082	9.623611	4.123733
FBL	W3	HPI4	well_2	F003	61	Avg. Volume	12.23143	11.00065	5.757384
FBL	W3	HPI4	well_2	F004	112	Avg. Volume	9.077821	8.41088	3.257019
FBL	W3	HPI4	well_2	F005	198	Avg. Volume	7.283389	6.328698	3.818948
FBL	W3	HPI4	well_2	F006	85	Avg. Volume	8.950975	8.062708	3.93883
FBL	W3	HPI4	well_3	F001	50	Avg. Volume	7.878042	7.258785	4.161252
FBL	W3	HPI4	well_3	F002	10	Avg. Volume	13.04762	11.8476	4.592181
FBL	W3	HPI4	well_3	F003	29	Avg. Volume	10.69327	8.802083	5.278816
FBL	W3	HPI4	well_3	F004	79	Avg. Volume	8.740182	7.968819	4.105398
FBL	W3	HPI4	well_3	F005	76	Avg. Volume	7.283535	6.530755	2.71281
FBL	W3	HPI4	well_3	F006	51	Avg. Volume	10.47374	9.694028	4.117302
FBL	W3	HPI4	well_4	F001	35	Avg. Volume	10.11546	9.694028	3.685843
FBL	W3	HPI4	well_4	F002	47	Avg. Volume	10.10569	8.191806	5.868474
FBL	W3	HPI4	well_4	F003	52	Avg. Volume	7.568905	7.326267	3.462558
FBL	W3	HPI4	well_4	F004	140	Avg. Volume	9.34101	8.414792	4.184805
FBL	W3	HPI4	well_4	F005	134	Avg. Volume	8.563871	7.847546	3.791925
FBL	W3	HPI4	well_4	F006	119	Avg. Volume	8.818511	8.191806	3.257994
FBL	W3	HPI4	well_5	F001	94	Avg. Volume	9.536242	8.813819	3.832306
FBL	W3	HPI4	well_5	F002	63	Avg. Volume	7.154483	6.728704	3.266844
FBL	W3	HPI4	well_5	F003	98	Avg. Volume	8.763256	8.068576	3.551697
FBL	W3	HPI4	well_5	F004	55	Avg. Volume	10.99488	10.11653	5.174443
FBL	W3	HPI4	well_5	F005	31	Avg. Volume	9.263414	8.19963	4.690259
FBL	W3	HPI4	well_5	F006	13	Avg. Volume	7.435893	7.933611	3.193159
FBL	W3	PGE2	well_1	F001	296	Avg. Volume	5.64521	5.381007	2.386252
FBL	W3	PGE2	well_1	F002	303	Avg. Volume	6.089969	5.586389	2.819335
FBL	W3	PGE2	well_1	F003	353	Avg. Volume	6.232541	5.87588	2.648109
FBL	W3	PGE2	well_1	F004	286	Avg. Volume	6.655272	6.091042	2.564065
FBL	W3	PGE2	well_1	F005	312	Avg. Volume	5.392396	5.037726	2.350519
FBL	W3	PGE2	well_1	F006	346	Avg. Volume	5.648217	5.217679	2.434086
FBL	W3	PGE2	well_2	F001	350	Avg. Volume	4.987538	4.569259	2.379265
FBL	W3	PGE2	well_2	F002	317	Avg. Volume	5.978591	5.692014	2.537082
FBL	W3	PGE2	well_2	F003	299	Avg. Volume	5.608953	5.023056	2.476744
FBL	W3	PGE2	well_2	F004	292	Avg. Volume	6.738102	6.226007	2.920386
FBL	W3	PGE2	well_2	F005	287	Avg. Volume	6.086783	5.762431	2.423168

FBL	W3	PGE2	well_2	F006	365	Avg. Volume	6.047087	5.586389	2.21178
FBL	W3	PGE2	well_3	F001	201	Avg. Volume	5.326151	4.976111	2.009649
FBL	W3	PGE2	well_3	F002	259	Avg. Volume	5.928593	5.367315	2.6412
FBL	W3	PGE2	well_3	F003	288	Avg. Volume	6.153419	5.997153	2.412554
FBL	W3	PGE2	well_3	F004	302	Avg. Volume	6.922167	6.163414	2.837943
FBL	W3	PGE2	well_3	F005	273	Avg. Volume	5.855407	5.578565	2.247167
FBL	W3	PGE2	well_3	F006	335	Avg. Volume	5.985872	5.539444	2.633996
FBL	W3	PGE2	well_4	F001	173	Avg. Volume	5.954691	5.390787	2.559156
FBL	W3	PGE2	well_4	F002	252	Avg. Volume	6.12304	5.455336	3.066391
FBL	W3	PGE2	well_4	F003	198	Avg. Volume	6.570271	5.8661	3.353902
FBL	W3	PGE2	well_4	F004	208	Avg. Volume	5.877588	5.328194	2.668396
FBL	W3	PGE2	well_4	F005	202	Avg. Volume	5.738204	5.47294	2.287127
FBL	W3	PGE2	well_4	F006	278	Avg. Volume	6.224819	5.629421	2.956159
FBL	W3	PGE2	well_5	F001	231	Avg. Volume	6.470521	5.903264	3.086963
FBL	W3	PGE2	well_5	F002	169	Avg. Volume	7.107539	6.3375	2.895579
FBL	W3	PGE2	well_5	F003	335	Avg. Volume	5.585815	5.336019	2.299645
FBL	W3	PGE2	well_5	F004	220	Avg. Volume	6.420585	5.731134	2.949788
FBL	W3	PGE2	well_5	F005	214	Avg. Volume	5.786508	5.449468	2.211708
FBL	W3	PGE2	well_5	F006	223	Avg. Volume	6.387991	5.695926	3.061742
FBL	W4	HPI4	well_1	F001	46	Avg. Volume	3.116918	2.611285	2.280962
FBL	W4	HPI4	well_1	F002	48	Avg. Volume	1.923986	1.776065	1.435704
FBL	W4	HPI4	well_1	F003	32	Avg. Volume	4.223044	3.452373	3.303541
FBL	W4	HPI4	well_1	F004	15	Avg. Volume	4.509977	4.154583	2.965659
FBL	W4	HPI4	well_1	F005	84	Avg. Volume	3.282712	2.951632	2.164169
FBL	W4	HPI4	well_1	F006	43	Avg. Volume	2.321176	2.139884	1.665353
FBL	W4	HPI4	well_2	F001	94	Avg. Volume	3.549428	2.699306	2.368378
FBL	W4	HPI4	well_2	F002	51	Avg. Volume	3.508054	2.863611	2.484204
FBL	W4	HPI4	well_2	F003	110	Avg. Volume	3.955832	3.286111	2.303469
FBL	W4	HPI4	well_2	F004	15	Avg. Volume	2.091285	2.065556	1.64212
FBL	W4	HPI4	well_2	F005	47	Avg. Volume	3.92074	3.286111	2.291446
FBL	W4	HPI4	well_2	F006	39	Avg. Volume	5.544027	5.609861	2.581715
FBL	W4	HPI4	well_3	F001	17	Avg. Volume	7.410963	4.940903	6.083951
FBL	W4	HPI4	well_3	F002	26	Avg. Volume	4.049492	3.890521	2.118446
FBL	W4	HPI4	well_3	F003	86	Avg. Volume	3.836154	3.359462	2.425303
FBL	W4	HPI4	well_3	F004	65	Avg. Volume	2.969116	2.511528	2.442273
FBL	W4	HPI4	well_3	F005	88	Avg. Volume	4.712976	3.679271	3.503994
FBL	W4	HPI4	well_3	F006	49	Avg. Volume	4.993706	3.76338	3.329804
FBL	W4	HPI4	well_4	F001	46	Avg. Volume	4.427203	3.074861	3.642892
FBL	W4	HPI4	well_4	F002	48	Avg. Volume	3.789553	3.548218	2.895115
FBL	W4	HPI4	well_4	F003	53	Avg. Volume	4.225458	4.717917	2.612657
FBL	W4	HPI4	well_4	F004	86	Avg. Volume	4.210169	3.943333	2.533275
FBL	W4	HPI4	well_4	F005	107	Avg. Volume	3.390624	2.769722	2.500289
FBL	W4	HPI4	well_4	F006	80	Avg. Volume	3.603844	3.246991	2.525739
FBL	W4	HPI4	well_5	F001	43	Avg. Volume	4.325814	3.556042	2.393607
FBL	W4	HPI4	well_5	F003	22	Avg. Volume	6.195145	4.82941	4.378102
FBL	W4	HPI4	well_5	F004	69	Avg. Volume	3.425075	2.652361	2.577933
FBL	W4	HPI4	well_5	F005	102	Avg. Volume	4.501125	4.412778	2.426264
FBL	W4	HPI4	well_5	F006	54	Avg. Volume	4.791756	4.078299	3.24967
FBL	W4	PGE2	well_1	F001	434	Avg. Volume	2.53701	2.124236	1.586824

FBL	W4	PGE2	well_1	F003	341	Avg. Volume	3.314193	2.699306	2.11702
FBL	W4	PGE2	well_1	F004	376	Avg. Volume	3.333347	2.884736	1.975035
FBL	W4	PGE2	well_1	F005	367	Avg. Volume	3.875394	3.466065	2.373497
FBL	W4	PGE2	well_1	F006	385	Avg. Volume	2.943193	2.581944	1.874902
FBL	W4	PGE2	well_2	F001	206	Avg. Volume	3.348368	2.871435	2.163781
FBL	W4	PGE2	well_2	F002	259	Avg. Volume	3.208565	2.816667	2.018054
FBL	W4	PGE2	well_2	F003	339	Avg. Volume	3.569999	2.74625	2.569472
FBL	W4	PGE2	well_2	F004	279	Avg. Volume	3.147045	2.769722	1.881097
FBL	W4	PGE2	well_2	F005	331	Avg. Volume	3.126417	2.664097	1.988621
FBL	W4	PGE2	well_2	F006	327	Avg. Volume	3.158607	2.699306	1.840408
FBL	W4	PGE2	well_3	F001	305	Avg. Volume	2.712992	2.147708	1.772471
FBL	W4	PGE2	well_3	F002	274	Avg. Volume	3.277905	2.757986	2.068927
FBL	W4	PGE2	well_3	F003	263	Avg. Volume	3.051124	2.546736	1.952218
FBL	W4	PGE2	well_3	F004	302	Avg. Volume	3.630125	2.926204	2.518285
FBL	W4	PGE2	well_3	F005	258	Avg. Volume	2.764539	2.32375	2.02562
FBL	W4	PGE2	well_3	F006	306	Avg. Volume	3.403578	3.004444	2.043265
FBL	W4	PGE2	well_4	F001	214	Avg. Volume	3.262311	2.936962	1.507841
FBL	W4	PGE2	well_4	F002	259	Avg. Volume	2.977129	2.347222	2.002963
FBL	W4	PGE2	well_4	F003	273	Avg. Volume	2.937913	2.206389	2.314285
FBL	W4	PGE2	well_4	F004	305	Avg. Volume	3.876206	3.245035	2.505629
FBL	W4	PGE2	well_4	F005	294	Avg. Volume	3.04809	2.517396	2.181158
FBL	W4	PGE2	well_4	F006	279	Avg. Volume	3.257753	2.863611	2.044799
FBL	W4	PGE2	well_5	F001	57	Avg. Volume	2.412975	2.124236	1.730961
FBL	W4	PGE2	well_5	F002	79	Avg. Volume	4.555209	4.279769	2.0332
FBL	W4	PGE2	well_5	F003	220	Avg. Volume	3.331615	2.593681	2.39999
FBL	W4	PGE2	well_5	F004	142	Avg. Volume	2.477151	1.807361	2.046536
FBL	W4	PGE2	well_5	F005	261	Avg. Volume	2.674829	2.130104	1.799645
FBL	W4	PGE2	well_5	F006	78	Avg. Volume	3.364706	3.261335	2.02542
FBL	W1	HPI4	well_1	F001	368	Count/cell	3.321023	3	1.29069
FBL	W1	HPI4	well_1	F002	368	Count/cell	3.209632	3	1.268597
FBL	W1	HPI4	well_1	F003	421	Count/cell	3.230024	3	1.274419
FBL	W1	HPI4	well_1	F004	346	Count/cell	3.075301	3	1.157032
FBL	W1	HPI4	well_1	F005	343	Count/cell	3.428144	3	1.254153
FBL	W1	HPI4	well_1	F006	332	Count/cell	3.235294	3	1.192781
FBL	W1	HPI4	well_2	F001	332	Count/cell	3.380795	3	1.098487
FBL	W1	HPI4	well_2	F002	204	Count/cell	3.324468	3	1.037292
FBL	W1	HPI4	well_2	F003	431	Count/cell	3.159806	3	1.21824
FBL	W1	HPI4	well_2	F004	310	Count/cell	3.133333	3	1.288834
FBL	W1	HPI4	well_2	F005	331	Count/cell	3.559748	4	1.396667
FBL	W1	HPI4	well_2	F006	360	Count/cell	3.12828	3	1.197409
FBL	W1	HPI4	well_3	F001	279	Count/cell	3.334559	3	1.425331
FBL	W1	HPI4	well_3	F002	266	Count/cell	3.566802	3	1.123742
FBL	W1	HPI4	well_3	F003	410	Count/cell	3.21393	3	1.239019
FBL	W1	HPI4	well_3	F004	317	Count/cell	3.072848	3	1.286781
FBL	W1	HPI4	well_3	F005	372	Count/cell	3.35989	3	1.435341
FBL	W1	HPI4	well_3	F006	285	Count/cell	3.281022	3	1.271498
FBL	W1	HPI4	well_4	F001	311	Count/cell	3.057627	3	1.267088
FBL	W1	HPI4	well_4	F002	331	Count/cell	3.176101	3	1.240914
FBL	W1	HPI4	well_4	F003	396	Count/cell	3.275862	3	1.379283

FBL	W1	HPI4	well_4	F004	445	Count/cell	3.255172	3	1.422884
FBL	W1	HPI4	well_4	F005	228	Count/cell	3.614679	3.5	1.118823
FBL	W1	HPI4	well_4	F006	324	Count/cell	3.301948	3	1.2173
FBL	W1	HPI4	well_5	F001	179	Count/cell	3.898204	4	1.645127
FBL	W1	HPI4	well_5	F002	290	Count/cell	3.120567	3	1.198903
FBL	W1	HPI4	well_5	F003	412	Count/cell	3.005102	3	1.126284
FBL	W1	HPI4	well_5	F004	444	Count/cell	3.069444	3	1.303143
FBL	W1	HPI4	well_5	F005	309	Count/cell	3.40636	3	1.108108
FBL	W1	HPI4	well_5	F006	280	Count/cell	3.441606	3	1.236748
FBL	W1	PGE2	well_1	F001	448	Count/cell	3.05467	3	1.182722
FBL	W1	PGE2	well_1	F002	186	Count/cell	3.298246	3	0.993477
FBL	W1	PGE2	well_1	F003	265	Count/cell	3.518828	3	1.21556
FBL	W1	PGE2	well_1	F004	334	Count/cell	3.119122	3	1.207254
FBL	W1	PGE2	well_1	F005	429	Count/cell	3.106024	3	1.130746
FBL	W1	PGE2	well_1	F006	379	Count/cell	3.414835	3	1.373058
FBL	W1	PGE2	well_2	F001	240	Count/cell	3.481982	3	1.209742
FBL	W1	PGE2	well_2	F002	367	Count/cell	3.294618	3	1.421587
FBL	W1	PGE2	well_2	F003	415	Count/cell	3.266497	3	1.27115
FBL	W1	PGE2	well_2	F004	430	Count/cell	3.320574	3	1.465055
FBL	W1	PGE2	well_2	F005	333	Count/cell	3.24375	3	1.170413
FBL	W1	PGE2	well_2	F006	258	Count/cell	3.258065	3	1.333899
FBL	W1	PGE2	well_3	F001	325	Count/cell	3.187097	3	1.247844
FBL	W1	PGE2	well_3	F002	254	Count/cell	3.28	3	1.428904
FBL	W1	PGE2	well_3	F003	353	Count/cell	3.196481	3	1.236499
FBL	W1	PGE2	well_3	F004	376	Count/cell	3.331522	3	1.411856
FBL	W1	PGE2	well_3	F005	364	Count/cell	3.128571	3	1.249887
FBL	W1	PGE2	well_3	F006	177	Count/cell	3.462428	3	1.412168
FBL	W1	PGE2	well_4	F001	264	Count/cell	3.420233	3	1.378709
FBL	W1	PGE2	well_4	F002	360	Count/cell	3.408163	3	1.214986
FBL	W1	PGE2	well_4	F003	338	Count/cell	3.444785	3	1.390635
FBL	W1	PGE2	well_4	F004	350	Count/cell	3.36646	3	1.153024
FBL	W1	PGE2	well_4	F005	225	Count/cell	3.339713	3	1.085039
FBL	W1	PGE2	well_4	F006	328	Count/cell	3.182109	3	1.161094
FBL	W1	PGE2	well_5	F001	415	Count/cell	3.190955	3	1.251183
FBL	W1	PGE2	well_5	F002	307	Count/cell	3.318644	3	1.28842
FBL	W1	PGE2	well_5	F003	282	Count/cell	3.536122	3	1.040051
FBL	W1	PGE2	well_5	F004	319	Count/cell	3.571429	3	1.222653
FBL	W1	PGE2	well_5	F005	340	Count/cell	3.404908	3	1.414814
FBL	W1	PGE2	well_5	F006	312	Count/cell	3.702797	4	1.281087
FBL	W2	HPI4	well_1	F001	172	Count/cell	1.909639	2	0.858627
FBL	W2	HPI4	well_1	F002	162	Count/cell	2.283019	2	0.886838
FBL	W2	HPI4	well_1	F003	151	Count/cell	2.425676	2	1.149334
FBL	W2	HPI4	well_1	F004	174	Count/cell	2.291667	2	1.074217
FBL	W2	HPI4	well_1	F005	175	Count/cell	2.142857	2	0.890977
FBL	W2	HPI4	well_1	F006	149	Count/cell	2.048951	2	0.833529
FBL	W2	HPI4	well_2	F001	128	Count/cell	1.64	1	0.733968
FBL	W2	HPI4	well_2	F002	177	Count/cell	2.136095	2	0.865578
FBL	W2	HPI4	well_2	F003	105	Count/cell	2.089109	2	0.861383
FBL	W2	HPI4	well_2	F004	201	Count/cell	2.035354	2	0.851253

FBL	W2	HPI4	well_2	F005	215	Count/cell	2.171429	2	0.788156
FBL	W2	HPI4	well_2	F006	111	Count/cell	2.333333	2	0.895144
FBL	W2	HPI4	well_3	F001	110	Count/cell	1.675926	2	0.721223
FBL	W2	HPI4	well_3	F002	32	Count/cell	2.21875	2	0.87009
FBL	W2	HPI4	well_3	F003	113	Count/cell	2.336449	2	1.054585
FBL	W2	HPI4	well_3	F004	166	Count/cell	2.062893	2	0.959203
FBL	W2	HPI4	well_3	F005	109	Count/cell	1.990654	2	0.936627
FBL	W2	HPI4	well_3	F006	81	Count/cell	2.05	2	0.855363
FBL	W2	HPI4	well_4	F001	126	Count/cell	2.008264	2	0.899035
FBL	W2	HPI4	well_4	F002	207	Count/cell	2.103448	2	0.875485
FBL	W2	HPI4	well_4	F003	163	Count/cell	2.201299	2	0.9385
FBL	W2	HPI4	well_4	F004	149	Count/cell	1.951724	2	0.876634
FBL	W2	HPI4	well_4	F005	84	Count/cell	2.2875	2	0.859699
FBL	W2	HPI4	well_4	F006	101	Count/cell	2.469388	2	1.027499
FBL	W2	HPI4	well_5	F001	68	Count/cell	2.104478	2	1.046413
FBL	W2	HPI4	well_5	F002	89	Count/cell	2.232558	2	0.953932
FBL	W2	HPI4	well_5	F003	85	Count/cell	2.518519	2	0.895979
FBL	W2	HPI4	well_5	F004	135	Count/cell	2.167939	2	0.805297
FBL	W2	HPI4	well_5	F005	185	Count/cell	2.129944	2	1.05532
FBL	W2	HPI4	well_5	F006	95	Count/cell	1.968085	2	0.873162
FBL	W2	PGE2	well_1	F001	128	Count/cell	2.68	3	1.12594
FBL	W2	PGE2	well_1	F002	40	Count/cell	3.236842	3	1.172528
FBL	W2	PGE2	well_1	F003	61	Count/cell	3	3	1.354006
FBL	W2	PGE2	well_1	F004	117	Count/cell	2.765766	3	1.026512
FBL	W2	PGE2	well_1	F005	222	Count/cell	2.780374	3	1.008596
FBL	W2	PGE2	well_1	F006	71	Count/cell	2.882353	3	1.252215
FBL	W2	PGE2	well_2	F001	74	Count/cell	2.819444	3	1.091922
FBL	W2	PGE2	well_2	F002	128	Count/cell	2.818182	3	1.072381
FBL	W2	PGE2	well_2	F003	170	Count/cell	2.478528	2	0.898129
FBL	W2	PGE2	well_2	F004	108	Count/cell	2.761905	3	0.893608
FBL	W2	PGE2	well_2	F005	76	Count/cell	2.739726	3	1.202167
FBL	W2	PGE2	well_2	F006	52	Count/cell	2.882353	3	1.088982
FBL	W2	PGE2	well_3	F001	54	Count/cell	2.846154	3	1.126936
FBL	W2	PGE2	well_3	F002	286	Count/cell	3.125899	3	1.28669
FBL	W2	PGE2	well_3	F003	300	Count/cell	2.850694	3	1.10523
FBL	W2	PGE2	well_3	F004	172	Count/cell	3.072289	3	1.223835
FBL	W2	PGE2	well_3	F005	201	Count/cell	2.769634	3	1.178455
FBL	W2	PGE2	well_3	F006	149	Count/cell	2.618056	3	1.044443
FBL	W2	PGE2	well_4	F001	222	Count/cell	2.862559	3	1.15679
FBL	W2	PGE2	well_4	F002	164	Count/cell	2.594937	3	1.016037
FBL	W2	PGE2	well_4	F003	208	Count/cell	3.068627	3	1.229841
FBL	W2	PGE2	well_4	F004	262	Count/cell	2.795276	3	1.016376
FBL	W2	PGE2	well_4	F005	83	Count/cell	2.797468	3	1.125232
FBL	W2	PGE2	well_4	F006	170	Count/cell	2.801242	3	1.144661
FBL	W2	PGE2	well_5	F001	159	Count/cell	2.684211	3	1.025639
FBL	W2	PGE2	well_5	F002	211	Count/cell	2.835	3	1.064632
FBL	W2	PGE2	well_5	F003	182	Count/cell	2.775281	3	1.055016
FBL	W2	PGE2	well_5	F004	132	Count/cell	2.784615	3	1.174566
FBL	W2	PGE2	well_5	F005	171	Count/cell	2.869048	3	1.018156

FBL	W2	PGE2	well_5	F006	349	Count/cell	3.097345	3	1.242477
FBL	W3	HPI4	well_1	F001	56	Count/cell	2.2	2	0.847655
FBL	W3	HPI4	well_1	F002	54	Count/cell	2.392157	2	0.918225
FBL	W3	HPI4	well_1	F003	55	Count/cell	1.865385	2	0.741722
FBL	W3	HPI4	well_1	F004	281	Count/cell	2.172161	2	1.102985
FBL	W3	HPI4	well_1	F005	269	Count/cell	2.202335	2	1.002884
FBL	W3	HPI4	well_1	F006	124	Count/cell	2.042017	2	0.896272
FBL	W3	HPI4	well_2	F001	31	Count/cell	1.4	1	0.498273
FBL	W3	HPI4	well_2	F002	34	Count/cell	1.84375	2	0.723316
FBL	W3	HPI4	well_2	F003	61	Count/cell	2.416667	2	1.02992
FBL	W3	HPI4	well_2	F004	112	Count/cell	2.559633	2	1.013165
FBL	W3	HPI4	well_2	F005	198	Count/cell	2.252632	2	0.986345
FBL	W3	HPI4	well_2	F006	85	Count/cell	2.214286	2	0.90609
FBL	W3	HPI4	well_3	F001	50	Count/cell	1.978723	2	0.846716
FBL	W3	HPI4	well_3	F002	10	Count/cell	1.9	2	0.737865
FBL	W3	HPI4	well_3	F003	29	Count/cell	2.793103	3	1.346406
FBL	W3	HPI4	well_3	F004	79	Count/cell	2.467532	2	1.020635
FBL	W3	HPI4	well_3	F005	76	Count/cell	2.708333	2.5	1.080395
FBL	W3	HPI4	well_3	F006	51	Count/cell	2.32	2	0.843704
FBL	W3	HPI4	well_4	F001	35	Count/cell	2.147059	2	0.783634
FBL	W3	HPI4	well_4	F002	47	Count/cell	2.311111	2	1.10417
FBL	W3	HPI4	well_4	F003	52	Count/cell	2.82	3	1.155113
FBL	W3	HPI4	well_4	F004	140	Count/cell	2.133333	2	0.887727
FBL	W3	HPI4	well_4	F005	134	Count/cell	1.960938	2	0.826741
FBL	W3	HPI4	well_4	F006	119	Count/cell	2.295652	2	0.964037
FBL	W3	HPI4	well_5	F001	94	Count/cell	1.879121	2	0.757704
FBL	W3	HPI4	well_5	F002	63	Count/cell	2.40678	2	0.930677
FBL	W3	HPI4	well_5	F003	98	Count/cell	2.814433	3	1.130357
FBL	W3	HPI4	well_5	F004	55	Count/cell	2.301887	2	0.911147
FBL	W3	HPI4	well_5	F005	31	Count/cell	2.129032	2	1.05647
FBL	W3	HPI4	well_5	F006	13	Count/cell	2.583333	3	0.514929
FBL	W3	PGE2	well_1	F001	296	Count/cell	2.698582	3	1.09898
FBL	W3	PGE2	well_1	F002	303	Count/cell	2.587031	3	1.061517
FBL	W3	PGE2	well_1	F003	353	Count/cell	2.536657	2	1.038653
FBL	W3	PGE2	well_1	F004	286	Count/cell	2.492647	2.5	0.859042
FBL	W3	PGE2	well_1	F005	312	Count/cell	2.695652	3	1.035129
FBL	W3	PGE2	well_1	F006	346	Count/cell	2.678466	3	1.023327
FBL	W3	PGE2	well_2	F001	350	Count/cell	2.648968	3	1.045174
FBL	W3	PGE2	well_2	F002	317	Count/cell	2.626198	3	1.099629
FBL	W3	PGE2	well_2	F003	299	Count/cell	2.710884	3	1.058728
FBL	W3	PGE2	well_2	F004	292	Count/cell	2.442105	2	1.011022
FBL	W3	PGE2	well_2	F005	287	Count/cell	2.452899	2	0.870502
FBL	W3	PGE2	well_2	F006	365	Count/cell	2.540845	3	0.953965
FBL	W3	PGE2	well_3	F001	201	Count/cell	2.728205	3	1.001532
FBL	W3	PGE2	well_3	F002	259	Count/cell	2.633858	3	0.987969
FBL	W3	PGE2	well_3	F003	288	Count/cell	2.629496	3	1.028191
FBL	W3	PGE2	well_3	F004	302	Count/cell	2.410169	2	0.950062
FBL	W3	PGE2	well_3	F005	273	Count/cell	2.630597	2	1.002673
FBL	W3	PGE2	well_3	F006	335	Count/cell	2.561728	2	0.982455

FBL	W3	PGE2	well_4	F001	173	Count/cell	2.711765	3	1.045918
FBL	W3	PGE2	well_4	F002	252	Count/cell	2.453061	2	0.911471
FBL	W3	PGE2	well_4	F003	198	Count/cell	2.420213	2	0.9751
FBL	W3	PGE2	well_4	F004	208	Count/cell	2.707921	3	1.001907
FBL	W3	PGE2	well_4	F005	202	Count/cell	2.65641	3	0.973832
FBL	W3	PGE2	well_4	F006	278	Count/cell	2.597786	3	1.027688
FBL	W3	PGE2	well_5	F001	231	Count/cell	2.454955	2	1.135701
FBL	W3	PGE2	well_5	F002	169	Count/cell	2.337349	2	0.849629
FBL	W3	PGE2	well_5	F003	335	Count/cell	2.644377	3	1.035045
FBL	W3	PGE2	well_5	F004	220	Count/cell	2.716279	3	1.114251
FBL	W3	PGE2	well_5	F005	214	Count/cell	2.855072	3	1.004002
FBL	W3	PGE2	well_5	F006	223	Count/cell	2.580645	3	1.047127
FBL	W4	HPI4	well_1	F001	46	Count/cell	1.744186	2	0.758853
FBL	W4	HPI4	well_1	F002	48	Count/cell	1.866667	2	0.786245
FBL	W4	HPI4	well_1	F003	32	Count/cell	2.032258	2	0.795147
FBL	W4	HPI4	well_1	F004	15	Count/cell	1.928571	2	0.730046
FBL	W4	HPI4	well_1	F005	84	Count/cell	1.950617	2	0.864599
FBL	W4	HPI4	well_1	F006	43	Count/cell	2.023256	2	1.011561
FBL	W4	HPI4	well_2	F001	94	Count/cell	1.688889	2	0.664416
FBL	W4	HPI4	well_2	F002	51	Count/cell	2.117647	2	0.951779
FBL	W4	HPI4	well_2	F003	110	Count/cell	1.682243	2	0.623413
FBL	W4	HPI4	well_2	F004	15	Count/cell	1.642857	1.5	0.744946
FBL	W4	HPI4	well_2	F005	47	Count/cell	1.777778	2	0.735122
FBL	W4	HPI4	well_2	F006	39	Count/cell	2.051282	2	0.916191
FBL	W4	HPI4	well_3	F001	17	Count/cell	1.294118	1	0.469668
FBL	W4	HPI4	well_3	F002	26	Count/cell	2.08	2	0.702377
FBL	W4	HPI4	well_3	F003	86	Count/cell	2.119048	2	0.869909
FBL	W4	HPI4	well_3	F004	65	Count/cell	1.983871	2	0.914222
FBL	W4	HPI4	well_3	F005	88	Count/cell	1.741176	2	0.657429
FBL	W4	HPI4	well_3	F006	49	Count/cell	2.173913	2	0.797339
FBL	W4	HPI4	well_4	F001	46	Count/cell	2.065217	2	0.928611
FBL	W4	HPI4	well_4	F002	48	Count/cell	1.958333	2	0.742576
FBL	W4	HPI4	well_4	F003	53	Count/cell	2.235294	2	1.011696
FBL	W4	HPI4	well_4	F004	86	Count/cell	1.841463	2	0.838422
FBL	W4	HPI4	well_4	F005	107	Count/cell	1.847619	2	0.874653
FBL	W4	HPI4	well_4	F006	80	Count/cell	1.792208	2	0.848182
FBL	W4	HPI4	well_5	F001	43	Count/cell	2.302326	2	1.124084
FBL	W4	HPI4	well_5	F003	22	Count/cell	2.190476	2	0.813575
FBL	W4	HPI4	well_5	F004	69	Count/cell	1.723077	2	0.625192
FBL	W4	HPI4	well_5	F005	102	Count/cell	1.71134	2	0.62847
FBL	W4	HPI4	well_5	F006	54	Count/cell	2.148148	2	0.898792
FBL	W4	PGE2	well_1	F001	434	Count/cell	2.322275	2	1.079028
FBL	W4	PGE2	well_1	F003	341	Count/cell	1.981982	2	0.884764
FBL	W4	PGE2	well_1	F004	376	Count/cell	2.183562	2	0.884391
FBL	W4	PGE2	well_1	F005	367	Count/cell	2.019126	2	0.832336
FBL	W4	PGE2	well_1	F006	385	Count/cell	1.937173	2	0.823685
FBL	W4	PGE2	well_2	F001	206	Count/cell	2.169154	2	0.938746
FBL	W4	PGE2	well_2	F002	259	Count/cell	2.117647	2	0.865959
FBL	W4	PGE2	well_2	F003	339	Count/cell	1.883234	2	0.828266

FBL	W4	PGE2	well_2	F004	279	Count/cell	2.110294	2	0.890066
FBL	W4	PGE2	well_2	F005	331	Count/cell	1.845426	2	0.749316
FBL	W4	PGE2	well_2	F006	327	Count/cell	2.160883	2	0.880152
FBL	W4	PGE2	well_3	F001	305	Count/cell	2.325503	2	1.097021
FBL	W4	PGE2	well_3	F002	274	Count/cell	2.01476	2	0.843144
FBL	W4	PGE2	well_3	F003	263	Count/cell	1.923077	2	0.884141
FBL	W4	PGE2	well_3	F004	302	Count/cell	2.046823	2	0.914728
FBL	W4	PGE2	well_3	F005	258	Count/cell	1.976096	2	0.898569
FBL	W4	PGE2	well_3	F006	306	Count/cell	2.076412	2	0.850965
FBL	W4	PGE2	well_4	F001	214	Count/cell	2.696078	3	1.015051
FBL	W4	PGE2	well_4	F002	259	Count/cell	2.015564	2	0.824284
FBL	W4	PGE2	well_4	F003	273	Count/cell	1.847015	2	0.862727
FBL	W4	PGE2	well_4	F004	305	Count/cell	2.093023	2	0.88956
FBL	W4	PGE2	well_4	F005	294	Count/cell	1.802867	2	0.759017
FBL	W4	PGE2	well_4	F006	279	Count/cell	1.944444	2	0.866919
FBL	W4	PGE2	well_5	F001	57	Count/cell	2.017857	2	0.962781
FBL	W4	PGE2	well_5	F002	79	Count/cell	2.253333	2	0.806952
FBL	W4	PGE2	well_5	F003	220	Count/cell	1.938679	2	0.88752
FBL	W4	PGE2	well_5	F004	142	Count/cell	1.671533	2	0.676382
FBL	W4	PGE2	well_5	F005	261	Count/cell	1.996124	2	0.862076
FBL	W4	PGE2	well_5	F006	78	Count/cell	2.184211	2	0.975849
FBL	W1	HPI4	well_1	F001	368	X/Y distribution	3.566093	3.069316	1.54821
FBL	W1	HPI4	well_1	F002	368	X/Y distribution	3.530399	3.059298	1.558275
FBL	W1	HPI4	well_1	F003	421	X/Y distribution	3.301434	2.72163	1.479509
FBL	W1	HPI4	well_1	F004	346	X/Y distribution	3.317051	2.89048	1.424482
FBL	W1	HPI4	well_1	F005	343	X/Y distribution	3.268681	2.751059	1.323488
FBL	W1	HPI4	well_1	F006	332	X/Y distribution	3.393345	2.890672	1.501958
FBL	W1	HPI4	well_2	F001	332	X/Y distribution	3.410049	3.05898	1.237687
FBL	W1	HPI4	well_2	F002	204	X/Y distribution	3.348303	3.026911	1.211374
FBL	W1	HPI4	well_2	F003	431	X/Y distribution	3.067988	2.594554	1.30731
FBL	W1	HPI4	well_2	F004	310	X/Y distribution	3.516323	2.937059	1.632102
FBL	W1	HPI4	well_2	F005	331	X/Y distribution	3.64124	3.080142	1.617836
FBL	W1	HPI4	well_2	F006	360	X/Y distribution	3.208145	2.782139	1.382652
FBL	W1	HPI4	well_3	F001	279	X/Y distribution	3.672984	3.030364	1.817005
FBL	W1	HPI4	well_3	F002	266	X/Y distribution	3.590145	3.097053	1.462659
FBL	W1	HPI4	well_3	F003	410	X/Y distribution	3.117462	2.621258	1.284722
FBL	W1	HPI4	well_3	F004	317	X/Y distribution	3.603073	2.918037	1.849659
FBL	W1	HPI4	well_3	F005	372	X/Y distribution	3.899797	3.190657	1.837753
FBL	W1	HPI4	well_3	F006	285	X/Y distribution	3.310347	2.894964	1.234564
FBL	W1	HPI4	well_4	F001	311	X/Y distribution	3.855492	3.228966	1.859857
FBL	W1	HPI4	well_4	F002	331	X/Y distribution	3.690579	3.063822	1.819649
FBL	W1	HPI4	well_4	F003	396	X/Y distribution	3.518745	3.087643	1.491375
FBL	W1	HPI4	well_4	F004	445	X/Y distribution	3.364619	2.866024	1.463609
FBL	W1	HPI4	well_4	F005	228	X/Y distribution	3.613074	2.877892	1.836593
FBL	W1	HPI4	well_4	F006	324	X/Y distribution	3.223841	2.827959	1.19622
FBL	W1	HPI4	well_5	F001	179	X/Y distribution	5.10581	3.795571	3.156846
FBL	W1	HPI4	well_5	F002	290	X/Y distribution	3.078988	2.651944	1.322275
FBL	W1	HPI4	well_5	F003	412	X/Y distribution	3.063358	2.633026	1.328555
FBL	W1	HPI4	well_5	F004	444	X/Y distribution	3.128098	2.603692	1.450622

FBL	W1	HPI4	well_5	F005	309	X/Y distribution	3.245344	2.872917	1.254891
FBL	W1	HPI4	well_5	F006	280	X/Y distribution	3.494923	2.82753	1.674635
FBL	W1	PGE2	well_1	F001	448	X/Y distribution	2.818105	2.427281	1.140591
FBL	W1	PGE2	well_1	F002	186	X/Y distribution	3.932521	3.221807	1.946305
FBL	W1	PGE2	well_1	F003	265	X/Y distribution	3.696886	3.151701	1.652119
FBL	W1	PGE2	well_1	F004	334	X/Y distribution	3.439973	2.930653	1.520342
FBL	W1	PGE2	well_1	F005	429	X/Y distribution	3.162281	2.732291	1.31543
FBL	W1	PGE2	well_1	F006	379	X/Y distribution	3.435396	3.022341	1.366936
FBL	W1	PGE2	well_2	F001	240	X/Y distribution	3.402181	3.066307	1.226988
FBL	W1	PGE2	well_2	F002	367	X/Y distribution	3.885181	3.263916	1.902564
FBL	W1	PGE2	well_2	F003	415	X/Y distribution	3.404175	2.96032	1.448388
FBL	W1	PGE2	well_2	F004	430	X/Y distribution	3.196722	2.778455	1.24579
FBL	W1	PGE2	well_2	F005	333	X/Y distribution	3.720117	3.219801	1.610478
FBL	W1	PGE2	well_2	F006	258	X/Y distribution	3.599222	3.06348	1.607737
FBL	W1	PGE2	well_3	F001	325	X/Y distribution	3.625653	2.930506	1.790438
FBL	W1	PGE2	well_3	F002	254	X/Y distribution	3.326799	2.838116	1.374574
FBL	W1	PGE2	well_3	F003	353	X/Y distribution	3.224676	2.836857	1.380538
FBL	W1	PGE2	well_3	F004	376	X/Y distribution	3.479473	3.035455	1.483383
FBL	W1	PGE2	well_3	F005	364	X/Y distribution	3.329818	2.918586	1.437279
FBL	W1	PGE2	well_3	F006	177	X/Y distribution	3.52043	2.947318	1.529595
FBL	W1	PGE2	well_4	F001	264	X/Y distribution	4.011308	3.339695	1.862659
FBL	W1	PGE2	well_4	F002	360	X/Y distribution	3.613063	3.138829	1.626871
FBL	W1	PGE2	well_4	F003	338	X/Y distribution	3.682858	3.188513	1.639217
FBL	W1	PGE2	well_4	F004	350	X/Y distribution	3.135538	2.711424	1.357029
FBL	W1	PGE2	well_4	F005	225	X/Y distribution	3.619923	2.898751	1.912427
FBL	W1	PGE2	well_4	F006	328	X/Y distribution	3.399577	2.762121	1.703131
FBL	W1	PGE2	well_5	F001	415	X/Y distribution	3.362193	2.838686	1.44283
FBL	W1	PGE2	well_5	F002	307	X/Y distribution	3.519302	2.951955	1.511055
FBL	W1	PGE2	well_5	F003	282	X/Y distribution	3.260647	2.776221	1.397821
FBL	W1	PGE2	well_5	F004	319	X/Y distribution	3.768091	3.204428	1.653979
FBL	W1	PGE2	well_5	F005	340	X/Y distribution	3.639082	3.110526	1.66345
FBL	W1	PGE2	well_5	F006	312	X/Y distribution	3.669445	2.983529	1.657604
FBL	W2	HPI4	well_1	F001	172	X/Y distribution	2.523081	2.161102	1.456437
FBL	W2	HPI4	well_1	F002	162	X/Y distribution	2.868658	2.507568	1.320375
FBL	W2	HPI4	well_1	F003	151	X/Y distribution	3.516363	2.943094	1.996899
FBL	W2	HPI4	well_1	F004	174	X/Y distribution	3.515903	2.73652	2.321648
FBL	W2	HPI4	well_1	F005	175	X/Y distribution	3.514108	2.716008	2.493462
FBL	W2	HPI4	well_1	F006	149	X/Y distribution	3.161039	2.543367	1.962591
FBL	W2	HPI4	well_2	F001	128	X/Y distribution	1.545907	1.280166	1.082711
FBL	W2	HPI4	well_2	F002	177	X/Y distribution	2.920581	2.473047	1.670577
FBL	W2	HPI4	well_2	F003	105	X/Y distribution	3.103015	2.57647	1.868764
FBL	W2	HPI4	well_2	F004	201	X/Y distribution	2.207999	1.796384	1.36929
FBL	W2	HPI4	well_2	F005	215	X/Y distribution	2.142063	1.833477	1.103966
FBL	W2	HPI4	well_2	F006	111	X/Y distribution	2.782737	2.486982	1.187838
FBL	W2	HPI4	well_3	F001	110	X/Y distribution	1.211342	1.252083	0.417806
FBL	W2	HPI4	well_3	F002	32	X/Y distribution	2.458622	2.301129	1.296093
FBL	W2	HPI4	well_3	F003	113	X/Y distribution	3.44812	2.811579	2.140551
FBL	W2	HPI4	well_3	F004	166	X/Y distribution	2.80712	2.169612	1.886639
FBL	W2	HPI4	well_3	F005	109	X/Y distribution	2.651225	2.074538	1.773153

FBL	W2	HPI4	well_3	F006	81	X/Y distribution	2.615504	2.105458	1.723537
FBL	W2	HPI4	well_4	F001	126	X/Y distribution	2.39924	1.815497	1.891051
FBL	W2	HPI4	well_4	F002	207	X/Y distribution	2.445394	2.048586	1.3928
FBL	W2	HPI4	well_4	F003	163	X/Y distribution	3.048522	2.588505	1.852718
FBL	W2	HPI4	well_4	F004	149	X/Y distribution	2.968348	2.360274	1.974404
FBL	W2	HPI4	well_4	F005	84	X/Y distribution	3.123525	2.895174	1.281205
FBL	W2	HPI4	well_4	F006	101	X/Y distribution	3.838408	2.943697	2.275654
FBL	W2	HPI4	well_5	F001	68	X/Y distribution	2.197329	1.820041	1.363548
FBL	W2	HPI4	well_5	F002	89	X/Y distribution	2.604962	2.550816	1.080372
FBL	W2	HPI4	well_5	F003	85	X/Y distribution	3.098891	2.915193	1.143601
FBL	W2	HPI4	well_5	F004	135	X/Y distribution	2.892036	2.085719	2.062084
FBL	W2	HPI4	well_5	F005	185	X/Y distribution	2.889004	2.265496	1.973455
FBL	W2	HPI4	well_5	F006	95	X/Y distribution	2.565194	2.1069	1.726519
FBL	W2	PGE2	well_1	F001	128	X/Y distribution	3.365652	2.912552	1.615769
FBL	W2	PGE2	well_1	F002	40	X/Y distribution	3.694006	2.951534	1.684278
FBL	W2	PGE2	well_1	F003	61	X/Y distribution	3.525948	2.948335	1.934784
FBL	W2	PGE2	well_1	F004	117	X/Y distribution	3.811303	2.841786	2.338012
FBL	W2	PGE2	well_1	F005	222	X/Y distribution	2.987765	2.728056	1.014342
FBL	W2	PGE2	well_1	F006	71	X/Y distribution	2.963084	2.68715	1.202601
FBL	W2	PGE2	well_2	F001	74	X/Y distribution	3.418964	2.931577	1.475809
FBL	W2	PGE2	well_2	F002	128	X/Y distribution	3.294795	2.91484	1.285043
FBL	W2	PGE2	well_2	F003	170	X/Y distribution	2.93873	2.667711	1.126806
FBL	W2	PGE2	well_2	F004	108	X/Y distribution	2.828188	2.534822	0.922331
FBL	W2	PGE2	well_2	F005	76	X/Y distribution	3.292509	2.916951	1.327153
FBL	W2	PGE2	well_2	F006	52	X/Y distribution	3.606715	2.896172	1.580389
FBL	W2	PGE2	well_3	F001	54	X/Y distribution	3.375063	3.019543	1.354433
FBL	W2	PGE2	well_3	F002	286	X/Y distribution	3.189884	2.937139	1.085533
FBL	W2	PGE2	well_3	F003	300	X/Y distribution	2.875317	2.545885	1.118994
FBL	W2	PGE2	well_3	F004	172	X/Y distribution	3.227531	2.758148	1.334219
FBL	W2	PGE2	well_3	F005	201	X/Y distribution	3.212036	2.764403	1.323375
FBL	W2	PGE2	well_3	F006	149	X/Y distribution	3.328554	2.882034	1.461814
FBL	W2	PGE2	well_4	F001	222	X/Y distribution	3.776157	3.087378	1.744472
FBL	W2	PGE2	well_4	F002	164	X/Y distribution	3.288498	2.844732	1.468089
FBL	W2	PGE2	well_4	F003	208	X/Y distribution	3.084273	2.802355	1.030777
FBL	W2	PGE2	well_4	F004	262	X/Y distribution	2.878811	2.665229	0.990645
FBL	W2	PGE2	well_4	F005	83	X/Y distribution	3.038202	2.647102	1.18992
FBL	W2	PGE2	well_4	F006	170	X/Y distribution	3.073324	2.67514	1.249072
FBL	W2	PGE2	well_5	F001	159	X/Y distribution	3.115777	2.754646	1.318387
FBL	W2	PGE2	well_5	F002	211	X/Y distribution	3.313771	2.915108	1.493858
FBL	W2	PGE2	well_5	F003	182	X/Y distribution	3.104035	2.824939	1.195669
FBL	W2	PGE2	well_5	F004	132	X/Y distribution	3.458604	2.961637	1.510107
FBL	W2	PGE2	well_5	F005	171	X/Y distribution	3.218565	2.803146	1.39023
FBL	W2	PGE2	well_5	F006	349	X/Y distribution	3.199594	2.922574	1.127111
FBL	W3	HPI4	well_1	F001	56	X/Y distribution	3.187003	2.960711	1.622197
FBL	W3	HPI4	well_1	F002	54	X/Y distribution	4.265247	3.302512	3.195237
FBL	W3	HPI4	well_1	F003	55	X/Y distribution	2.692684	2.669509	1.063076
FBL	W3	HPI4	well_1	F004	281	X/Y distribution	4.896407	4.403995	2.894328
FBL	W3	HPI4	well_1	F005	269	X/Y distribution	4.324495	3.567413	2.649705
FBL	W3	HPI4	well_1	F006	124	X/Y distribution	4.070345	2.909395	2.93252

FBL	W3	HPI4	well_2	F001	31	X/Y distribution	1.667846	1.302129	2.187536
FBL	W3	HPI4	well_2	F002	34	X/Y distribution	3.163452	2.819286	1.880582
FBL	W3	HPI4	well_2	F003	61	X/Y distribution	3.637204	3.054304	2.239566
FBL	W3	HPI4	well_2	F004	112	X/Y distribution	3.159772	2.851251	1.490046
FBL	W3	HPI4	well_2	F005	198	X/Y distribution	4.205147	3.072119	2.757312
FBL	W3	HPI4	well_2	F006	85	X/Y distribution	2.733431	2.447463	1.234481
FBL	W3	HPI4	well_3	F001	50	X/Y distribution	2.790022	2.670702	1.602986
FBL	W3	HPI4	well_3	F002	10	X/Y distribution	2.542995	2.54361	1.381794
FBL	W3	HPI4	well_3	F003	29	X/Y distribution	2.833978	2.790251	0.887762
FBL	W3	HPI4	well_3	F004	79	X/Y distribution	4.435339	2.842739	3.499892
FBL	W3	HPI4	well_3	F005	76	X/Y distribution	3.86389	3.311196	1.855939
FBL	W3	HPI4	well_3	F006	51	X/Y distribution	3.083649	2.766065	1.601305
FBL	W3	HPI4	well_4	F001	35	X/Y distribution	4.003484	3.000411	2.690486
FBL	W3	HPI4	well_4	F002	47	X/Y distribution	3.242809	2.718427	1.852766
FBL	W3	HPI4	well_4	F003	52	X/Y distribution	4.344055	2.937499	2.736592
FBL	W3	HPI4	well_4	F004	140	X/Y distribution	3.457989	2.894132	2.036633
FBL	W3	HPI4	well_4	F005	134	X/Y distribution	3.570204	2.71782	2.587037
FBL	W3	HPI4	well_4	F006	119	X/Y distribution	3.336054	2.828353	1.747965
FBL	W3	HPI4	well_5	F001	94	X/Y distribution	3.474302	2.79789	2.31152
FBL	W3	HPI4	well_5	F002	63	X/Y distribution	3.476808	2.804639	1.993279
FBL	W3	HPI4	well_5	F003	98	X/Y distribution	3.393873	2.862596	1.853737
FBL	W3	HPI4	well_5	F004	55	X/Y distribution	2.874161	2.608126	1.436293
FBL	W3	HPI4	well_5	F005	31	X/Y distribution	3.103571	2.176602	2.451775
FBL	W3	HPI4	well_5	F006	13	X/Y distribution	3.534454	2.89927	2.00645
FBL	W3	PGE2	well_1	F001	296	X/Y distribution	3.288502	2.752705	1.507767
FBL	W3	PGE2	well_1	F002	303	X/Y distribution	2.954208	2.662129	1.250243
FBL	W3	PGE2	well_1	F003	353	X/Y distribution	2.828907	2.56685	1.121911
FBL	W3	PGE2	well_1	F004	286	X/Y distribution	2.576789	2.419425	0.876104
FBL	W3	PGE2	well_1	F005	312	X/Y distribution	2.878239	2.571465	1.128699
FBL	W3	PGE2	well_1	F006	346	X/Y distribution	3.087537	2.720967	1.313427
FBL	W3	PGE2	well_2	F001	350	X/Y distribution	3.000488	2.641012	1.204004
FBL	W3	PGE2	well_2	F002	317	X/Y distribution	2.790326	2.545469	0.977543
FBL	W3	PGE2	well_2	F003	299	X/Y distribution	2.803222	2.549217	1.122812
FBL	W3	PGE2	well_2	F004	292	X/Y distribution	2.907745	2.495677	1.297531
FBL	W3	PGE2	well_2	F005	287	X/Y distribution	2.461063	2.389102	0.714705
FBL	W3	PGE2	well_2	F006	365	X/Y distribution	2.645648	2.506686	0.915682
FBL	W3	PGE2	well_3	F001	201	X/Y distribution	3.013306	2.686949	1.121037
FBL	W3	PGE2	well_3	F002	259	X/Y distribution	2.689324	2.522239	0.854572
FBL	W3	PGE2	well_3	F003	288	X/Y distribution	2.89979	2.652263	1.01134
FBL	W3	PGE2	well_3	F004	302	X/Y distribution	2.454439	2.416858	0.6476
FBL	W3	PGE2	well_3	F005	273	X/Y distribution	2.553011	2.42863	0.732902
FBL	W3	PGE2	well_3	F006	335	X/Y distribution	2.798096	2.557924	1.036174
FBL	W3	PGE2	well_4	F001	173	X/Y distribution	2.831026	2.577715	1.10418
FBL	W3	PGE2	well_4	F002	252	X/Y distribution	2.753025	2.588504	0.956239
FBL	W3	PGE2	well_4	F003	198	X/Y distribution	2.800638	2.54847	1.082769
FBL	W3	PGE2	well_4	F004	208	X/Y distribution	3.021018	2.721916	1.0628
FBL	W3	PGE2	well_4	F005	202	X/Y distribution	2.889413	2.652543	0.933965
FBL	W3	PGE2	well_4	F006	278	X/Y distribution	2.731906	2.48507	1.011192
FBL	W3	PGE2	well_5	F001	231	X/Y distribution	3.302189	2.674214	1.725852

FBL	W3	PGE2	well_5	F002	169	X/Y distribution	2.661393	2.431087	0.967453
FBL	W3	PGE2	well_5	F003	335	X/Y distribution	2.82549	2.439401	1.214565
FBL	W3	PGE2	well_5	F004	220	X/Y distribution	2.787133	2.451434	1.228945
FBL	W3	PGE2	well_5	F005	214	X/Y distribution	3.102976	2.81821	1.161405
FBL	W3	PGE2	well_5	F006	223	X/Y distribution	2.892445	2.408135	1.33261
FBL	W4	HPI4	well_1	F001	46	X/Y distribution	5.880468	4.560395	3.661949
FBL	W4	HPI4	well_1	F002	48	X/Y distribution	6.159636	5.00647	3.911445
FBL	W4	HPI4	well_1	F003	32	X/Y distribution	8.555841	9.428249	4.437333
FBL	W4	HPI4	well_1	F004	15	X/Y distribution	5.97416	2.709851	5.335186
FBL	W4	HPI4	well_1	F005	84	X/Y distribution	5.703654	3.434022	4.191016
FBL	W4	HPI4	well_1	F006	43	X/Y distribution	5.953647	4.894384	3.542116
FBL	W4	HPI4	well_2	F001	94	X/Y distribution	5.207371	4.841099	2.957579
FBL	W4	HPI4	well_2	F002	51	X/Y distribution	4.415151	2.914283	3.246291
FBL	W4	HPI4	well_2	F003	110	X/Y distribution	3.498433	2.776284	2.403479
FBL	W4	HPI4	well_2	F004	15	X/Y distribution	5.514238	3.914852	3.613944
FBL	W4	HPI4	well_2	F005	47	X/Y distribution	3.892983	2.841262	2.941558
FBL	W4	HPI4	well_2	F006	39	X/Y distribution	6.037366	3.349427	4.541406
FBL	W4	HPI4	well_3	F001	17	X/Y distribution	3.776937	2.456418	3.74966
FBL	W4	HPI4	well_3	F002	26	X/Y distribution	4.09032	3.48607	2.160476
FBL	W4	HPI4	well_3	F003	86	X/Y distribution	5.500049	3.439218	3.690515
FBL	W4	HPI4	well_3	F004	65	X/Y distribution	7.502194	7.071704	3.953072
FBL	W4	HPI4	well_3	F005	88	X/Y distribution	4.401225	2.719111	3.36465
FBL	W4	HPI4	well_3	F006	49	X/Y distribution	5.396442	3.575151	3.539329
FBL	W4	HPI4	well_4	F001	46	X/Y distribution	4.84285	3.214587	3.018138
FBL	W4	HPI4	well_4	F002	48	X/Y distribution	4.462495	3.117357	3.735291
FBL	W4	HPI4	well_4	F003	53	X/Y distribution	5.993874	4.470453	4.140117
FBL	W4	HPI4	well_4	F004	86	X/Y distribution	5.03463	3.380432	3.419046
FBL	W4	HPI4	well_4	F005	107	X/Y distribution	5.729162	5.267307	3.688652
FBL	W4	HPI4	well_4	F006	80	X/Y distribution	7.68464	7.978953	4.552898
FBL	W4	HPI4	well_5	F001	43	X/Y distribution	4.472529	3.359349	2.953209
FBL	W4	HPI4	well_5	F003	22	X/Y distribution	4.03053	3.003546	2.160414
FBL	W4	HPI4	well_5	F004	69	X/Y distribution	5.442371	4.664598	2.935849
FBL	W4	HPI4	well_5	F005	102	X/Y distribution	3.745673	2.895457	2.804161
FBL	W4	HPI4	well_5	F006	54	X/Y distribution	3.318634	2.584604	2.504781
FBL	W4	PGE2	well_1	F001	434	X/Y distribution	3.620227	2.929171	1.833656
FBL	W4	PGE2	well_1	F003	341	X/Y distribution	2.931484	2.526415	1.298342
FBL	W4	PGE2	well_1	F004	376	X/Y distribution	2.870045	2.679116	1.054488
FBL	W4	PGE2	well_1	F005	367	X/Y distribution	2.798142	2.607437	1.081447
FBL	W4	PGE2	well_1	F006	385	X/Y distribution	3.283865	2.915975	1.336973
FBL	W4	PGE2	well_2	F001	206	X/Y distribution	3.613631	3.114227	1.607456
FBL	W4	PGE2	well_2	F002	259	X/Y distribution	3.100719	2.815115	1.229746
FBL	W4	PGE2	well_2	F003	339	X/Y distribution	3.329761	2.759651	1.73042
FBL	W4	PGE2	well_2	F004	279	X/Y distribution	3.095764	2.723357	1.294959
FBL	W4	PGE2	well_2	F005	331	X/Y distribution	2.976518	2.6427	1.235978
FBL	W4	PGE2	well_2	F006	327	X/Y distribution	3.340097	2.793574	1.534725
FBL	W4	PGE2	well_3	F001	305	X/Y distribution	3.950542	3.422502	1.832708
FBL	W4	PGE2	well_3	F002	274	X/Y distribution	3.459571	2.891801	1.654375
FBL	W4	PGE2	well_3	F003	263	X/Y distribution	3.334303	2.86649	1.358721
FBL	W4	PGE2	well_3	F004	302	X/Y distribution	2.832714	2.47328	1.289729

FBL	W4	PGE2	well_3	F005	258	X/Y distribution	3.294355	2.826063	1.482182
FBL	W4	PGE2	well_3	F006	306	X/Y distribution	3.286351	2.888355	1.411835
FBL	W4	PGE2	well_4	F001	214	X/Y distribution	2.970133	2.741789	1.032504
FBL	W4	PGE2	well_4	F002	259	X/Y distribution	3.051264	2.795448	1.191708
FBL	W4	PGE2	well_4	F003	273	X/Y distribution	2.734305	2.536504	1.162521
FBL	W4	PGE2	well_4	F004	305	X/Y distribution	3.05226	2.547953	1.486124
FBL	W4	PGE2	well_4	F005	294	X/Y distribution	2.712634	2.455823	1.170538
FBL	W4	PGE2	well_4	F006	279	X/Y distribution	3.821896	3.228421	1.872726
FBL	W4	PGE2	well_5	F001	57	X/Y distribution	4.936966	4.896604	2.382968
FBL	W4	PGE2	well_5	F002	79	X/Y distribution	2.635358	2.318848	1.265599
FBL	W4	PGE2	well_5	F003	220	X/Y distribution	3.096805	2.680421	1.437544
FBL	W4	PGE2	well_5	F004	142	X/Y distribution	3.322075	2.669172	2.022646
FBL	W4	PGE2	well_5	F005	261	X/Y distribution	3.424862	2.840377	1.731164
FBL	W4	PGE2	well_5	F006	78	X/Y distribution	3.482046	3.094464	1.403016
FBL	W1	HPI4	well_1	F001	368	Z distribution	-0.50008	-0.48508	0.47824
FBL	W1	HPI4	well_1	F002	368	Z distribution	-0.50845	-0.45911	0.563063
FBL	W1	HPI4	well_1	F003	421	Z distribution	-0.47775	-0.46285	0.477654
FBL	W1	HPI4	well_1	F004	346	Z distribution	-0.47796	-0.42292	0.467895
FBL	W1	HPI4	well_1	F005	343	Z distribution	-0.46345	-0.42819	0.430348
FBL	W1	HPI4	well_1	F006	332	Z distribution	-0.45619	-0.43952	0.553399
FBL	W1	HPI4	well_2	F001	332	Z distribution	-0.41268	-0.40797	0.453796
FBL	W1	HPI4	well_2	F002	204	Z distribution	-0.407	-0.41037	0.344248
FBL	W1	HPI4	well_2	F003	431	Z distribution	-0.49551	-0.47844	0.475544
FBL	W1	HPI4	well_2	F004	310	Z distribution	-0.52052	-0.46968	0.513282
FBL	W1	HPI4	well_2	F005	331	Z distribution	-0.42317	-0.43837	0.491271
FBL	W1	HPI4	well_2	F006	360	Z distribution	-0.47783	-0.51509	0.508557
FBL	W1	HPI4	well_3	F001	279	Z distribution	-0.39779	-0.33341	0.407236
FBL	W1	HPI4	well_3	F002	266	Z distribution	-0.43641	-0.38195	0.39167
FBL	W1	HPI4	well_3	F003	410	Z distribution	-0.56379	-0.54138	0.49834
FBL	W1	HPI4	well_3	F004	317	Z distribution	-0.46105	-0.44868	0.509943
FBL	W1	HPI4	well_3	F005	372	Z distribution	-0.46464	-0.39015	0.577988
FBL	W1	HPI4	well_3	F006	285	Z distribution	-0.39425	-0.35414	0.38727
FBL	W1	HPI4	well_4	F001	311	Z distribution	-0.4559	-0.40104	0.480623
FBL	W1	HPI4	well_4	F002	331	Z distribution	-0.49561	-0.48738	0.481758
FBL	W1	HPI4	well_4	F003	396	Z distribution	-0.44552	-0.37352	0.476837
FBL	W1	HPI4	well_4	F004	445	Z distribution	-0.42771	-0.38817	0.576275
FBL	W1	HPI4	well_4	F005	228	Z distribution	-0.48642	-0.45409	0.353032
FBL	W1	HPI4	well_4	F006	324	Z distribution	-0.40781	-0.37986	0.442507
FBL	W1	HPI4	well_5	F001	179	Z distribution	-0.41791	-0.3641	0.407878
FBL	W1	HPI4	well_5	F002	290	Z distribution	-0.44773	-0.41649	0.407061
FBL	W1	HPI4	well_5	F003	412	Z distribution	-0.47692	-0.44817	0.536291
FBL	W1	HPI4	well_5	F004	444	Z distribution	-0.54483	-0.52035	0.525604
FBL	W1	HPI4	well_5	F005	309	Z distribution	-0.46098	-0.42688	0.429374
FBL	W1	HPI4	well_5	F006	280	Z distribution	-0.5421	-0.54514	0.374358
FBL	W1	PGE2	well_1	F001	448	Z distribution	-0.5164	-0.5145	0.464326
FBL	W1	PGE2	well_1	F002	186	Z distribution	-0.48422	-0.46996	0.396799
FBL	W1	PGE2	well_1	F003	265	Z distribution	-0.50347	-0.45554	0.402089
FBL	W1	PGE2	well_1	F004	334	Z distribution	-0.43929	-0.44651	0.447043
FBL	W1	PGE2	well_1	F005	429	Z distribution	-0.50524	-0.45996	0.50775

FBL	W1	PGE2	well_1	F006	379	Z distribution	-0.42921	-0.42252	0.466294
FBL	W1	PGE2	well_2	F001	240	Z distribution	-0.28954	-0.25219	0.376035
FBL	W1	PGE2	well_2	F002	367	Z distribution	-0.49415	-0.43068	0.62436
FBL	W1	PGE2	well_2	F003	415	Z distribution	-0.46033	-0.42432	0.514006
FBL	W1	PGE2	well_2	F004	430	Z distribution	-0.48952	-0.44078	0.516366
FBL	W1	PGE2	well_2	F005	333	Z distribution	-0.41839	-0.33813	0.526162
FBL	W1	PGE2	well_2	F006	258	Z distribution	-0.39784	-0.3697	0.457051
FBL	W1	PGE2	well_3	F001	325	Z distribution	-0.46051	-0.41981	0.499378
FBL	W1	PGE2	well_3	F002	254	Z distribution	-0.43549	-0.38961	0.428246
FBL	W1	PGE2	well_3	F003	353	Z distribution	-0.46894	-0.45159	0.428025
FBL	W1	PGE2	well_3	F004	376	Z distribution	-0.48162	-0.46548	0.449979
FBL	W1	PGE2	well_3	F005	364	Z distribution	-0.45556	-0.43125	0.537691
FBL	W1	PGE2	well_3	F006	177	Z distribution	-0.33502	-0.30231	0.354092
FBL	W1	PGE2	well_4	F001	264	Z distribution	-0.45278	-0.40367	0.521541
FBL	W1	PGE2	well_4	F002	360	Z distribution	-0.43192	-0.39707	0.482582
FBL	W1	PGE2	well_4	F003	338	Z distribution	-0.47367	-0.46119	0.552504
FBL	W1	PGE2	well_4	F004	350	Z distribution	-0.49687	-0.47081	0.445057
FBL	W1	PGE2	well_4	F005	225	Z distribution	-0.39036	-0.35918	0.369954
FBL	W1	PGE2	well_4	F006	328	Z distribution	-0.4602	-0.39634	0.473311
FBL	W1	PGE2	well_5	F001	415	Z distribution	-0.48016	-0.41326	0.506833
FBL	W1	PGE2	well_5	F002	307	Z distribution	-0.45523	-0.40585	0.48488
FBL	W1	PGE2	well_5	F003	282	Z distribution	-0.44777	-0.41972	0.358613
FBL	W1	PGE2	well_5	F004	319	Z distribution	-0.43904	-0.39545	0.516179
FBL	W1	PGE2	well_5	F005	340	Z distribution	-0.49467	-0.47523	0.537682
FBL	W1	PGE2	well_5	F006	312	Z distribution	-0.3851	-0.3127	0.465767
FBL	W2	HPI4	well_1	F001	172	Z distribution	-0.24587	-0.09725	0.460783
FBL	W2	HPI4	well_1	F002	162	Z distribution	-0.23511	-0.21119	0.265647
FBL	W2	HPI4	well_1	F003	151	Z distribution	-0.24843	-0.21379	0.371641
FBL	W2	HPI4	well_1	F004	174	Z distribution	-0.1757	-0.10078	0.340719
FBL	W2	HPI4	well_1	F005	175	Z distribution	-0.15867	-0.10157	0.338654
FBL	W2	HPI4	well_1	F006	149	Z distribution	-0.176	-0.11695	0.227034
FBL	W2	HPI4	well_2	F001	128	Z distribution	-0.41024	-0.42714	0.460886
FBL	W2	HPI4	well_2	F002	177	Z distribution	-0.1995	-0.13377	0.311708
FBL	W2	HPI4	well_2	F003	105	Z distribution	-0.16478	-0.09241	0.294259
FBL	W2	HPI4	well_2	F004	201	Z distribution	-0.19251	-0.11154	0.442568
FBL	W2	HPI4	well_2	F005	215	Z distribution	-0.07706	-0.04141	0.332115
FBL	W2	HPI4	well_2	F006	111	Z distribution	-0.11371	-0.04087	0.284342
FBL	W2	HPI4	well_3	F001	110	Z distribution	-0.39127	-0.36453	0.452645
FBL	W2	HPI4	well_3	F002	32	Z distribution	-0.06779	-0.03001	0.190627
FBL	W2	HPI4	well_3	F003	113	Z distribution	-0.13724	-0.10149	0.302709
FBL	W2	HPI4	well_3	F004	166	Z distribution	-0.29322	-0.16909	0.421518
FBL	W2	HPI4	well_3	F005	109	Z distribution	-0.16139	-0.04935	0.411382
FBL	W2	HPI4	well_3	F006	81	Z distribution	-0.12436	-0.06401	0.587069
FBL	W2	HPI4	well_4	F001	126	Z distribution	-0.37835	-0.26498	0.514567
FBL	W2	HPI4	well_4	F002	207	Z distribution	-0.13329	-0.09132	0.365869
FBL	W2	HPI4	well_4	F003	163	Z distribution	-0.23346	-0.20522	0.354349
FBL	W2	HPI4	well_4	F004	149	Z distribution	-0.25988	-0.15365	0.395825
FBL	W2	HPI4	well_4	F005	84	Z distribution	-0.06524	-0.04049	0.183793
FBL	W2	HPI4	well_4	F006	101	Z distribution	-0.14249	-0.10213	0.27671

FBL	W2	HPI4	well_5	F001	68	Z distribution	-0.49526	-0.46376	0.547128
FBL	W2	HPI4	well_5	F002	89	Z distribution	-0.21281	-0.06146	0.404622
FBL	W2	HPI4	well_5	F003	85	Z distribution	-0.16591	-0.14945	0.156965
FBL	W2	HPI4	well_5	F004	135	Z distribution	-0.14949	-0.10048	0.409586
FBL	W2	HPI4	well_5	F005	185	Z distribution	-0.21571	-0.11153	0.478148
FBL	W2	HPI4	well_5	F006	95	Z distribution	-0.10135	-0.04305	0.388624
FBL	W2	PGE2	well_1	F001	128	Z distribution	-0.45961	-0.42885	0.464238
FBL	W2	PGE2	well_1	F002	40	Z distribution	-0.50113	-0.40471	0.645706
FBL	W2	PGE2	well_1	F003	61	Z distribution	-0.42001	-0.47386	0.426176
FBL	W2	PGE2	well_1	F004	117	Z distribution	-0.68325	-0.64695	0.579119
FBL	W2	PGE2	well_1	F005	222	Z distribution	-0.41309	-0.36264	0.497084
FBL	W2	PGE2	well_1	F006	71	Z distribution	-0.4334	-0.50946	0.421011
FBL	W2	PGE2	well_2	F001	74	Z distribution	-0.38021	-0.40127	0.453136
FBL	W2	PGE2	well_2	F002	128	Z distribution	-0.50849	-0.56061	0.461001
FBL	W2	PGE2	well_2	F003	170	Z distribution	-0.41355	-0.44227	0.444189
FBL	W2	PGE2	well_2	F004	108	Z distribution	-0.46876	-0.47435	0.36247
FBL	W2	PGE2	well_2	F005	76	Z distribution	-0.39732	-0.31898	0.488283
FBL	W2	PGE2	well_2	F006	52	Z distribution	-0.53352	-0.53926	0.484236
FBL	W2	PGE2	well_3	F001	54	Z distribution	-0.5759	-0.52238	0.517579
FBL	W2	PGE2	well_3	F002	286	Z distribution	-0.38577	-0.33801	0.518326
FBL	W2	PGE2	well_3	F003	300	Z distribution	-0.42505	-0.39798	0.490445
FBL	W2	PGE2	well_3	F004	172	Z distribution	-0.34233	-0.31976	0.419476
FBL	W2	PGE2	well_3	F005	201	Z distribution	-0.42122	-0.38605	0.615098
FBL	W2	PGE2	well_3	F006	149	Z distribution	-0.50264	-0.44039	0.575903
FBL	W2	PGE2	well_4	F001	222	Z distribution	-0.5564	-0.44384	0.650678
FBL	W2	PGE2	well_4	F002	164	Z distribution	-0.49138	-0.46679	0.57229
FBL	W2	PGE2	well_4	F003	208	Z distribution	-0.35159	-0.37748	0.496373
FBL	W2	PGE2	well_4	F004	262	Z distribution	-0.46778	-0.48472	0.408375
FBL	W2	PGE2	well_4	F005	83	Z distribution	-0.5124	-0.54249	0.479141
FBL	W2	PGE2	well_4	F006	170	Z distribution	-0.37772	-0.38445	0.407116
FBL	W2	PGE2	well_5	F001	159	Z distribution	-0.3774	-0.36265	0.475936
FBL	W2	PGE2	well_5	F002	211	Z distribution	-0.34742	-0.36073	0.457353
FBL	W2	PGE2	well_5	F003	182	Z distribution	-0.43798	-0.38391	0.457189
FBL	W2	PGE2	well_5	F004	132	Z distribution	-0.33163	-0.401	0.496555
FBL	W2	PGE2	well_5	F005	171	Z distribution	-0.41392	-0.41956	0.42203
FBL	W2	PGE2	well_5	F006	349	Z distribution	-0.41766	-0.42285	0.526313
FBL	W3	HPI4	well_1	F001	56	Z distribution	-0.24474	-0.18984	0.282006
FBL	W3	HPI4	well_1	F002	54	Z distribution	-0.16788	-0.12226	0.195556
FBL	W3	HPI4	well_1	F003	55	Z distribution	-0.0864	-0.04982	0.130238
FBL	W3	HPI4	well_1	F004	281	Z distribution	-0.43912	-0.27273	0.581335
FBL	W3	HPI4	well_1	F005	269	Z distribution	-0.29984	-0.187	0.511212
FBL	W3	HPI4	well_1	F006	124	Z distribution	-0.3292	-0.30726	0.298008
FBL	W3	HPI4	well_2	F001	31	Z distribution	-0.25009	-0.23184	0.714635
FBL	W3	HPI4	well_2	F002	34	Z distribution	-0.05043	-0.02475	0.222903
FBL	W3	HPI4	well_2	F003	61	Z distribution	-0.15469	-0.07444	0.270833
FBL	W3	HPI4	well_2	F004	112	Z distribution	-0.24543	-0.22706	0.20516
FBL	W3	HPI4	well_2	F005	198	Z distribution	-0.34319	-0.25793	0.41548
FBL	W3	HPI4	well_2	F006	85	Z distribution	-0.15409	-0.08833	0.209779
FBL	W3	HPI4	well_3	F001	50	Z distribution	-0.29594	-0.08835	0.589563

FBL	W3	HPI4	well_3	F002	10	Z distribution	-0.07944	-0.08211	0.022957
FBL	W3	HPI4	well_3	F003	29	Z distribution	-0.14698	-0.08827	0.147103
FBL	W3	HPI4	well_3	F004	79	Z distribution	-0.32417	-0.27404	0.305348
FBL	W3	HPI4	well_3	F005	76	Z distribution	-0.20467	-0.10514	0.320936
FBL	W3	HPI4	well_3	F006	51	Z distribution	-0.17888	-0.14648	0.172716
FBL	W3	HPI4	well_4	F001	35	Z distribution	-0.08285	-0.05285	0.171698
FBL	W3	HPI4	well_4	F002	47	Z distribution	-0.13803	-0.11201	0.119249
FBL	W3	HPI4	well_4	F003	52	Z distribution	-0.23905	-0.18365	0.232941
FBL	W3	HPI4	well_4	F004	140	Z distribution	-0.26872	-0.23912	0.284622
FBL	W3	HPI4	well_4	F005	134	Z distribution	-0.31368	-0.21504	0.349321
FBL	W3	HPI4	well_4	F006	119	Z distribution	-0.1878	-0.11453	0.236006
FBL	W3	HPI4	well_5	F001	94	Z distribution	-0.1967	-0.16896	0.251432
FBL	W3	HPI4	well_5	F002	63	Z distribution	-0.07664	-0.03204	0.226887
FBL	W3	HPI4	well_5	F003	98	Z distribution	-0.26876	-0.22957	0.265492
FBL	W3	HPI4	well_5	F004	55	Z distribution	-0.09086	-0.03901	0.140843
FBL	W3	HPI4	well_5	F005	31	Z distribution	-0.26492	-0.14044	0.341508
FBL	W3	HPI4	well_5	F006	13	Z distribution	-0.1904	-0.12019	0.245668
FBL	W3	PGE2	well_1	F001	296	Z distribution	-0.44729	-0.3947	0.453899
FBL	W3	PGE2	well_1	F002	303	Z distribution	-0.38401	-0.39783	0.441612
FBL	W3	PGE2	well_1	F003	353	Z distribution	-0.40736	-0.40109	0.436589
FBL	W3	PGE2	well_1	F004	286	Z distribution	-0.38902	-0.40774	0.380975
FBL	W3	PGE2	well_1	F005	312	Z distribution	-0.4026	-0.43102	0.469011
FBL	W3	PGE2	well_1	F006	346	Z distribution	-0.29036	-0.33782	0.459731
FBL	W3	PGE2	well_2	F001	350	Z distribution	-0.3916	-0.3859	0.439059
FBL	W3	PGE2	well_2	F002	317	Z distribution	-0.34777	-0.34832	0.44932
FBL	W3	PGE2	well_2	F003	299	Z distribution	-0.3704	-0.36712	0.393218
FBL	W3	PGE2	well_2	F004	292	Z distribution	-0.3499	-0.37762	0.403942
FBL	W3	PGE2	well_2	F005	287	Z distribution	-0.3549	-0.39778	0.380344
FBL	W3	PGE2	well_2	F006	365	Z distribution	-0.43071	-0.45385	0.411248
FBL	W3	PGE2	well_3	F001	201	Z distribution	-0.34435	-0.35171	0.397704
FBL	W3	PGE2	well_3	F002	259	Z distribution	-0.31312	-0.30707	0.385182
FBL	W3	PGE2	well_3	F003	288	Z distribution	-0.3897	-0.37907	0.380306
FBL	W3	PGE2	well_3	F004	302	Z distribution	-0.52986	-0.52074	0.385254
FBL	W3	PGE2	well_3	F005	273	Z distribution	-0.42495	-0.46512	0.419093
FBL	W3	PGE2	well_3	F006	335	Z distribution	-0.43303	-0.45922	0.44213
FBL	W3	PGE2	well_4	F001	173	Z distribution	-0.43676	-0.42194	0.430174
FBL	W3	PGE2	well_4	F002	252	Z distribution	-0.41995	-0.40567	0.410885
FBL	W3	PGE2	well_4	F003	198	Z distribution	-0.32538	-0.37141	0.408689
FBL	W3	PGE2	well_4	F004	208	Z distribution	-0.45052	-0.44785	0.426836
FBL	W3	PGE2	well_4	F005	202	Z distribution	-0.35886	-0.31578	0.45691
FBL	W3	PGE2	well_4	F006	278	Z distribution	-0.33791	-0.31161	0.406946
FBL	W3	PGE2	well_5	F001	231	Z distribution	-0.39096	-0.38905	0.454867
FBL	W3	PGE2	well_5	F002	169	Z distribution	-0.38955	-0.40487	0.410094
FBL	W3	PGE2	well_5	F003	335	Z distribution	-0.4114	-0.34994	0.46169
FBL	W3	PGE2	well_5	F004	220	Z distribution	-0.34511	-0.37235	0.439245
FBL	W3	PGE2	well_5	F005	214	Z distribution	-0.34423	-0.3482	0.439147
FBL	W3	PGE2	well_5	F006	223	Z distribution	-0.29027	-0.31174	0.430749
FBL	W4	HPI4	well_1	F001	46	Z distribution	-0.35799	-0.18926	0.461584
FBL	W4	HPI4	well_1	F002	48	Z distribution	-0.41996	-0.38004	0.362917

FBL	W4	HPI4	well_1	F003	32	Z distribution	-0.61045	-0.4349	0.548941
FBL	W4	HPI4	well_1	F004	15	Z distribution	-0.28805	-0.21401	0.285292
FBL	W4	HPI4	well_1	F005	84	Z distribution	-0.25427	-0.198	0.345536
FBL	W4	HPI4	well_1	F006	43	Z distribution	-0.27451	-0.2172	0.312229
FBL	W4	HPI4	well_2	F001	94	Z distribution	-0.42659	-0.27259	0.439509
FBL	W4	HPI4	well_2	F002	51	Z distribution	-0.26801	-0.22059	0.325617
FBL	W4	HPI4	well_2	F003	110	Z distribution	-0.28732	-0.2535	0.27441
FBL	W4	HPI4	well_2	F004	15	Z distribution	-0.37612	-0.28422	0.334995
FBL	W4	HPI4	well_2	F005	47	Z distribution	-0.25774	-0.20236	0.285896
FBL	W4	HPI4	well_2	F006	39	Z distribution	-0.31248	-0.30206	0.377854
FBL	W4	HPI4	well_3	F001	17	Z distribution	-0.1662	-0.15636	0.179441
FBL	W4	HPI4	well_3	F002	26	Z distribution	-0.33351	-0.31259	0.192839
FBL	W4	HPI4	well_3	F003	86	Z distribution	-0.25771	-0.23632	0.279163
FBL	W4	HPI4	well_3	F004	65	Z distribution	-0.56643	-0.57109	0.423632
FBL	W4	HPI4	well_3	F005	88	Z distribution	-0.24124	-0.18916	0.273529
FBL	W4	HPI4	well_3	F006	49	Z distribution	-0.19847	-0.17826	0.29881
FBL	W4	HPI4	well_4	F001	46	Z distribution	-0.22461	-0.16494	0.278639
FBL	W4	HPI4	well_4	F002	48	Z distribution	-0.36979	-0.3652	0.262486
FBL	W4	HPI4	well_4	F003	53	Z distribution	-0.3939	-0.39885	0.299335
FBL	W4	HPI4	well_4	F004	86	Z distribution	-0.28889	-0.20699	0.382828
FBL	W4	HPI4	well_4	F005	107	Z distribution	-0.24487	-0.23453	0.345554
FBL	W4	HPI4	well_4	F006	80	Z distribution	-0.33806	-0.28311	0.37373
FBL	W4	HPI4	well_5	F001	43	Z distribution	-0.26253	-0.20749	0.257306
FBL	W4	HPI4	well_5	F003	22	Z distribution	-0.09822	-0.11336	0.167739
FBL	W4	HPI4	well_5	F004	69	Z distribution	-0.40382	-0.4035	0.345869
FBL	W4	HPI4	well_5	F005	102	Z distribution	-0.23143	-0.2018	0.297731
FBL	W4	HPI4	well_5	F006	54	Z distribution	-0.23977	-0.19282	0.279246
FBL	W4	PGE2	well_1	F001	434	Z distribution	-0.38532	-0.40659	0.690854
FBL	W4	PGE2	well_1	F003	341	Z distribution	-0.45058	-0.51947	0.680693
FBL	W4	PGE2	well_1	F004	376	Z distribution	-0.54113	-0.46837	0.68275
FBL	W4	PGE2	well_1	F005	367	Z distribution	-0.50224	-0.52004	0.600314
FBL	W4	PGE2	well_1	F006	385	Z distribution	-0.55762	-0.51185	0.758386
FBL	W4	PGE2	well_2	F001	206	Z distribution	-0.352	-0.40313	0.575184
FBL	W4	PGE2	well_2	F002	259	Z distribution	-0.56261	-0.54393	0.680751
FBL	W4	PGE2	well_2	F003	339	Z distribution	-0.45656	-0.44477	0.766513
FBL	W4	PGE2	well_2	F004	279	Z distribution	-0.41002	-0.46067	0.699576
FBL	W4	PGE2	well_2	F005	331	Z distribution	-0.5646	-0.50869	0.742095
FBL	W4	PGE2	well_2	F006	327	Z distribution	-0.54682	-0.51784	0.6989
FBL	W4	PGE2	well_3	F001	305	Z distribution	-0.65424	-0.63517	0.745508
FBL	W4	PGE2	well_3	F002	274	Z distribution	-0.35079	-0.37712	0.635455
FBL	W4	PGE2	well_3	F003	263	Z distribution	-0.42649	-0.50386	0.694666
FBL	W4	PGE2	well_3	F004	302	Z distribution	-0.62621	-0.60711	0.663223
FBL	W4	PGE2	well_3	F005	258	Z distribution	-0.57927	-0.61543	0.599905
FBL	W4	PGE2	well_3	F006	306	Z distribution	-0.52652	-0.49626	0.619636
FBL	W4	PGE2	well_4	F001	214	Z distribution	-0.11161	-0.07446	0.415909
FBL	W4	PGE2	well_4	F002	259	Z distribution	-0.42925	-0.39644	0.508031
FBL	W4	PGE2	well_4	F003	273	Z distribution	-0.5392	-0.58428	0.566595
FBL	W4	PGE2	well_4	F004	305	Z distribution	-0.54715	-0.49719	0.57848
FBL	W4	PGE2	well_4	F005	294	Z distribution	-0.42458	-0.43784	0.646161

FBL	W4	PGE2	well_4	F006	279	Z distribution	-0.49598	-0.47945	0.768036
FBL	W4	PGE2	well_5	F001	57	Z distribution	-0.32579	-0.28525	0.772351
FBL	W4	PGE2	well_5	F002	79	Z distribution	0.110608	0.091398	0.411604
FBL	W4	PGE2	well_5	F003	220	Z distribution	-0.33613	-0.2939	0.632224
FBL	W4	PGE2	well_5	F004	142	Z distribution	-0.4405	-0.40669	0.633224
FBL	W4	PGE2	well_5	F005	261	Z distribution	-0.46191	-0.52234	0.654004
FBL	W4	PGE2	well_5	F006	78	Z distribution	-0.45675	-0.49845	0.732207
DSP	W1	HPI4	well_1	F001	150	Avg. Volume	0.353184	0.351738	0.133406
DSP	W1	HPI4	well_1	F002	168	Avg. Volume	0.342643	0.344259	0.118146
DSP	W1	HPI4	well_1	F003	384	Avg. Volume	0.447592	0.434882	0.160796
DSP	W1	HPI4	well_1	F004	145	Avg. Volume	0.404583	0.396557	0.1294
DSP	W1	HPI4	well_1	F005	293	Avg. Volume	0.373931	0.371209	0.132887
DSP	W1	HPI4	well_1	F006	148	Avg. Volume	0.408527	0.400188	0.139254
DSP	W1	HPI4	well_2	F001	172	Avg. Volume	0.343438	0.31042	0.143673
DSP	W1	HPI4	well_2	F002	258	Avg. Volume	0.342267	0.325134	0.153025
DSP	W1	HPI4	well_2	F003	152	Avg. Volume	0.419899	0.425632	0.174133
DSP	W1	HPI4	well_2	F004	352	Avg. Volume	0.352707	0.334479	0.130743
DSP	W1	HPI4	well_2	F005	236	Avg. Volume	0.433074	0.426079	0.151523
DSP	W1	HPI4	well_2	F006	315	Avg. Volume	0.389307	0.390226	0.130334
DSP	W1	HPI4	well_3	F001	223	Avg. Volume	0.411776	0.401099	0.163867
DSP	W1	HPI4	well_3	F002	266	Avg. Volume	0.438583	0.439895	0.160192
DSP	W1	HPI4	well_3	F003	191	Avg. Volume	0.420809	0.4056	0.142045
DSP	W1	HPI4	well_3	F004	324	Avg. Volume	0.406176	0.388708	0.146654
DSP	W1	HPI4	well_3	F005	209	Avg. Volume	0.362484	0.351614	0.13687
DSP	W1	HPI4	well_3	F006	213	Avg. Volume	0.413452	0.410562	0.146247
DSP	W1	HPI4	well_4	F001	205	Avg. Volume	0.423167	0.413698	0.139454
DSP	W1	HPI4	well_4	F002	106	Avg. Volume	0.353624	0.323154	0.183347
DSP	W1	HPI4	well_4	F003	359	Avg. Volume	0.395524	0.382459	0.149775
DSP	W1	HPI4	well_4	F004	181	Avg. Volume	0.352923	0.351594	0.173711
DSP	W1	HPI4	well_4	F005	252	Avg. Volume	0.377304	0.370666	0.130488
DSP	W1	HPI4	well_4	F006	188	Avg. Volume	0.419675	0.423268	0.149287
DSP	W1	HPI4	well_5	F001	172	Avg. Volume	0.395413	0.380585	0.151725
DSP	W1	HPI4	well_5	F002	265	Avg. Volume	0.327709	0.319222	0.136344
DSP	W1	HPI4	well_5	F003	231	Avg. Volume	0.339041	0.317565	0.141052
DSP	W1	HPI4	well_5	F004	361	Avg. Volume	0.394378	0.384853	0.151445
DSP	W1	HPI4	well_5	F005	178	Avg. Volume	0.392883	0.379515	0.150924
DSP	W1	HPI4	well_5	F006	201	Avg. Volume	0.417329	0.41244	0.158123
DSP	W1	PGE2	well_1	F001	104	Avg. Volume	0.31381	0.290948	0.106942
DSP	W1	PGE2	well_1	F002	161	Avg. Volume	0.340155	0.330464	0.143126
DSP	W1	PGE2	well_1	F003	332	Avg. Volume	0.385741	0.384793	0.131036
DSP	W1	PGE2	well_1	F004	188	Avg. Volume	0.389434	0.38327	0.143365
DSP	W1	PGE2	well_1	F005	84	Avg. Volume	0.414665	0.406016	0.168354
DSP	W1	PGE2	well_1	F006	287	Avg. Volume	0.389454	0.374089	0.149175
DSP	W1	PGE2	well_2	F001	249	Avg. Volume	0.424184	0.408022	0.128619
DSP	W1	PGE2	well_2	F002	245	Avg. Volume	0.390316	0.368709	0.142045
DSP	W1	PGE2	well_2	F003	228	Avg. Volume	0.368879	0.360619	0.127528
DSP	W1	PGE2	well_2	F004	256	Avg. Volume	0.315844	0.303771	0.139988
DSP	W1	PGE2	well_2	F005	347	Avg. Volume	0.325267	0.290555	0.160917
DSP	W1	PGE2	well_2	F006	203	Avg. Volume	0.375509	0.359907	0.138727

DSP	W1	PGE2	well_3	F001	192	Avg. Volume	0.370332	0.384602	0.127044
DSP	W1	PGE2	well_3	F002	229	Avg. Volume	0.426635	0.408417	0.164049
DSP	W1	PGE2	well_3	F003	224	Avg. Volume	0.344425	0.321665	0.135136
DSP	W1	PGE2	well_3	F004	215	Avg. Volume	0.397298	0.38284	0.151403
DSP	W1	PGE2	well_3	F005	112	Avg. Volume	0.367337	0.364923	0.148765
DSP	W1	PGE2	well_3	F006	261	Avg. Volume	0.435183	0.442495	0.138098
DSP	W1	PGE2	well_4	F001	266	Avg. Volume	0.392666	0.377779	0.133368
DSP	W1	PGE2	well_4	F002	227	Avg. Volume	0.387673	0.3887	0.113247
DSP	W1	PGE2	well_4	F003	296	Avg. Volume	0.400326	0.390315	0.154806
DSP	W1	PGE2	well_4	F004	364	Avg. Volume	0.398746	0.390168	0.148499
DSP	W1	PGE2	well_4	F005	366	Avg. Volume	0.414904	0.413581	0.139572
DSP	W1	PGE2	well_4	F006	218	Avg. Volume	0.407061	0.409087	0.156783
DSP	W1	PGE2	well_5	F001	112	Avg. Volume	0.393719	0.377068	0.133688
DSP	W1	PGE2	well_5	F002	127	Avg. Volume	0.368715	0.360368	0.163568
DSP	W1	PGE2	well_5	F003	233	Avg. Volume	0.381736	0.369035	0.139377
DSP	W1	PGE2	well_5	F004	203	Avg. Volume	0.421515	0.416941	0.149769
DSP	W1	PGE2	well_5	F005	230	Avg. Volume	0.331086	0.321765	0.141075
DSP	W1	PGE2	well_5	F006	276	Avg. Volume	0.343817	0.319669	0.15089
DSP	W2	HPI4	well_1	F001	235	Avg. Volume	0.349681	0.326477	0.133658
DSP	W2	HPI4	well_1	F002	179	Avg. Volume	0.399865	0.380585	0.14113
DSP	W2	HPI4	well_1	F003	363	Avg. Volume	0.311139	0.313941	0.16829
DSP	W2	HPI4	well_1	F004	200	Avg. Volume	0.31498	0.302351	0.147969
DSP	W2	HPI4	well_1	F005	286	Avg. Volume	0.3652	0.352617	0.136754
DSP	W2	HPI4	well_1	F006	115	Avg. Volume	0.402456	0.366427	0.137124
DSP	W2	HPI4	well_2	F001	147	Avg. Volume	0.354948	0.33928	0.153977
DSP	W2	HPI4	well_2	F002	177	Avg. Volume	0.473223	0.46185	0.155356
DSP	W2	HPI4	well_2	F003	329	Avg. Volume	0.4024	0.39316	0.151948
DSP	W2	HPI4	well_2	F004	272	Avg. Volume	0.372313	0.351431	0.137024
DSP	W2	HPI4	well_2	F005	185	Avg. Volume	0.362197	0.355916	0.11987
DSP	W2	HPI4	well_2	F006	275	Avg. Volume	0.327024	0.305139	0.140785
DSP	W2	HPI4	well_3	F001	144	Avg. Volume	0.37071	0.359691	0.118337
DSP	W2	HPI4	well_3	F002	183	Avg. Volume	0.46253	0.460116	0.173054
DSP	W2	HPI4	well_3	F003	55	Avg. Volume	0.357905	0.356225	0.197964
DSP	W2	HPI4	well_3	F004	288	Avg. Volume	0.383146	0.375995	0.143991
DSP	W2	HPI4	well_3	F005	289	Avg. Volume	0.318592	0.316875	0.141564
DSP	W2	HPI4	well_3	F006	277	Avg. Volume	0.393738	0.38025	0.142474
DSP	W2	HPI4	well_4	F001	222	Avg. Volume	0.366677	0.355323	0.114493
DSP	W2	HPI4	well_4	F002	154	Avg. Volume	0.363454	0.357625	0.140097
DSP	W2	HPI4	well_4	F003	24	Avg. Volume	0.370771	0.380086	0.176001
DSP	W2	HPI4	well_4	F004	202	Avg. Volume	0.210223	0.201861	0.103007
DSP	W2	HPI4	well_4	F005	182	Avg. Volume	0.437606	0.429912	0.15855
DSP	W2	HPI4	well_4	F006	340	Avg. Volume	0.559828	0.570802	0.182942
DSP	W2	HPI4	well_5	F001	237	Avg. Volume	0.463067	0.427455	0.157099
DSP	W2	HPI4	well_5	F002	197	Avg. Volume	0.369536	0.36079	0.12817
DSP	W2	HPI4	well_5	F003	84	Avg. Volume	0.394518	0.393998	0.131905
DSP	W2	HPI4	well_5	F004	149	Avg. Volume	0.37064	0.359418	0.119676
DSP	W2	HPI4	well_5	F005	262	Avg. Volume	0.470673	0.454647	0.155384
DSP	W2	HPI4	well_5	F006	160	Avg. Volume	0.504433	0.502427	0.205538
DSP	W2	PGE2	well_1	F001	286	Avg. Volume	0.531796	0.539624	0.169112

DSP	W2	PGE2	well_1	F002	342	Avg. Volume	0.514411	0.525997	0.180179
DSP	W2	PGE2	well_1	F003	375	Avg. Volume	0.438565	0.4225	0.170521
DSP	W2	PGE2	well_1	F004	315	Avg. Volume	0.589465	0.602943	0.180312
DSP	W2	PGE2	well_1	F005	340	Avg. Volume	0.596407	0.609744	0.196767
DSP	W2	PGE2	well_1	F006	393	Avg. Volume	0.576938	0.575628	0.162108
DSP	W2	PGE2	well_2	F001	331	Avg. Volume	0.652715	0.660039	0.149437
DSP	W2	PGE2	well_2	F002	375	Avg. Volume	0.539789	0.540979	0.13371
DSP	W2	PGE2	well_2	F003	352	Avg. Volume	0.632571	0.183814	0.795115
DSP	W2	PGE2	well_2	F004	308	Avg. Volume	0.545197	0.541532	0.171932
DSP	W2	PGE2	well_2	F005	416	Avg. Volume	0.574401	0.570587	0.188709
DSP	W2	PGE2	well_2	F006	396	Avg. Volume	0.529975	0.548844	0.175864
DSP	W2	PGE2	well_3	F001	127	Avg. Volume	0.381226	0.345447	0.185011
DSP	W2	PGE2	well_3	F002	303	Avg. Volume	0.493548	0.487049	0.152472
DSP	W2	PGE2	well_3	F003	337	Avg. Volume	0.482832	0.46475	0.188679
DSP	W2	PGE2	well_3	F004	339	Avg. Volume	0.597479	0.586806	0.174999
DSP	W2	PGE2	well_3	F005	373	Avg. Volume	0.606895	0.593512	0.182652
DSP	W2	PGE2	well_3	F006	334	Avg. Volume	0.512398	0.523962	0.164404
DSP	W2	PGE2	well_4	F001	302	Avg. Volume	0.449739	0.427797	0.159082
DSP	W2	PGE2	well_4	F002	292	Avg. Volume	0.51981	0.521381	0.146811
DSP	W2	PGE2	well_4	F003	391	Avg. Volume	0.476129	0.462924	0.199248
DSP	W2	PGE2	well_4	F004	338	Avg. Volume	0.410068	0.414046	0.158215
DSP	W2	PGE2	well_4	F005	421	Avg. Volume	0.514289	0.505386	0.177198
DSP	W2	PGE2	well_4	F006	14	Avg. Volume	0.637497	0.645077	0.182483
DSP	W2	PGE2	well_5	F001	186	Avg. Volume	0.469376	0.464852	0.161314
DSP	W2	PGE2	well_5	F002	329	Avg. Volume	0.557682	0.582111	0.165117
DSP	W2	PGE2	well_5	F003	221	Avg. Volume	0.40322	0.395417	0.150521
DSP	W2	PGE2	well_5	F004	331	Avg. Volume	0.504101	0.505163	0.165173
DSP	W2	PGE2	well_5	F005	306	Avg. Volume	0.510964	0.490725	0.161589
DSP	W2	PGE2	well_5	F006	245	Avg. Volume	0.562545	0.549641	0.180663
DSP	W3	HPI4	well_1	F001	242	Avg. Volume	0.477289	0.473356	0.137547
DSP	W3	HPI4	well_1	F002	315	Avg. Volume	0.342897	0.324529	0.147739
DSP	W3	HPI4	well_1	F003	90	Avg. Volume	0.479676	0.448336	0.19322
DSP	W3	HPI4	well_1	F004	370	Avg. Volume	0.380083	0.377512	0.141258
DSP	W3	HPI4	well_1	F005	345	Avg. Volume	0.389116	0.394533	0.157665
DSP	W3	HPI4	well_1	F006	191	Avg. Volume	0.42323	0.410764	0.165379
DSP	W3	HPI4	well_2	F001	219	Avg. Volume	0.462519	0.454122	0.164697
DSP	W3	HPI4	well_2	F002	195	Avg. Volume	0.485313	0.481914	0.166703
DSP	W3	HPI4	well_2	F003	260	Avg. Volume	0.290776	0.266114	0.137856
DSP	W3	HPI4	well_2	F004	310	Avg. Volume	0.34175	0.329514	0.146843
DSP	W3	HPI4	well_2	F005	212	Avg. Volume	0.347417	0.333473	0.133204
DSP	W3	HPI4	well_2	F006	279	Avg. Volume	0.286227	0.282687	0.122337
DSP	W3	HPI4	well_3	F001	244	Avg. Volume	0.436652	0.427716	0.130483
DSP	W3	HPI4	well_3	F002	205	Avg. Volume	0.389195	0.374287	0.144069
DSP	W3	HPI4	well_3	F003	87	Avg. Volume	0.173072	0.135804	0.107123
DSP	W3	HPI4	well_3	F004	106	Avg. Volume	0.412942	0.390492	0.159609
DSP	W3	HPI4	well_3	F005	327	Avg. Volume	0.432303	0.418358	0.138612
DSP	W3	HPI4	well_3	F006	366	Avg. Volume	0.428848	0.4239	0.156369
DSP	W3	HPI4	well_4	F001	180	Avg. Volume	0.452629	0.448906	0.145927
DSP	W3	HPI4	well_4	F002	99	Avg. Volume	0.351256	0.329589	0.138868

DSP	W3	HPI4	well_4	F003	42	Avg. Volume	0.49118	0.425853	0.260293
DSP	W3	HPI4	well_4	F004	91	Avg. Volume	0.400542	0.396894	0.156275
DSP	W3	HPI4	well_4	F005	507	Avg. Volume	0.316328	0.304629	0.143324
DSP	W3	HPI4	well_4	F006	305	Avg. Volume	0.416919	0.400833	0.157297
DSP	W3	HPI4	well_5	F001	152	Avg. Volume	0.399442	0.396814	0.144923
DSP	W3	HPI4	well_5	F002	59	Avg. Volume	0.39409	0.332899	0.189636
DSP	W3	HPI4	well_5	F003	3	Avg. Volume	0.477269	0.477269	NA
DSP	W3	HPI4	well_5	F004	29	Avg. Volume	0.443427	0.436583	0.132809
DSP	W3	HPI4	well_5	F005	205	Avg. Volume	0.434283	0.422813	0.145208
DSP	W3	HPI4	well_5	F006	292	Avg. Volume	0.382072	0.372035	0.160299
DSP	W3	PGE2	well_1	F001	382	Avg. Volume	0.420231	0.410397	0.127757
DSP	W3	PGE2	well_1	F002	355	Avg. Volume	0.563609	0.57754	0.166175
DSP	W3	PGE2	well_1	F003	336	Avg. Volume	0.535382	0.549672	0.152167
DSP	W3	PGE2	well_1	F004	209	Avg. Volume	0.5462	0.529533	0.156133
DSP	W3	PGE2	well_1	F005	332	Avg. Volume	0.581598	0.55941	0.154461
DSP	W3	PGE2	well_1	F006	167	Avg. Volume	0.418168	0.410074	0.15432
DSP	W3	PGE2	well_2	F001	308	Avg. Volume	0.48282	0.474604	0.152654
DSP	W3	PGE2	well_2	F002	328	Avg. Volume	0.559108	0.535825	0.18417
DSP	W3	PGE2	well_2	F003	194	Avg. Volume	0.503729	0.502138	0.213263
DSP	W3	PGE2	well_2	F004	384	Avg. Volume	0.487061	0.487587	0.150006
DSP	W3	PGE2	well_2	F005	348	Avg. Volume	0.431735	0.412432	0.140956
DSP	W3	PGE2	well_2	F006	217	Avg. Volume	0.391193	0.383047	0.16159
DSP	W3	PGE2	well_3	F001	345	Avg. Volume	0.523037	0.513918	0.171884
DSP	W3	PGE2	well_3	F002	72	Avg. Volume	0.255313	0.244744	0.165229
DSP	W3	PGE2	well_3	F003	339	Avg. Volume	0.457477	0.447711	0.137186
DSP	W3	PGE2	well_3	F004	371	Avg. Volume	0.291517	0.271339	0.135101
DSP	W3	PGE2	well_3	F005	291	Avg. Volume	0.446137	0.425072	0.142783
DSP	W3	PGE2	well_3	F006	358	Avg. Volume	0.41729	0.399965	0.125598
DSP	W3	PGE2	well_4	F001	309	Avg. Volume	0.492315	0.477269	0.167055
DSP	W3	PGE2	well_4	F002	416	Avg. Volume	0.3756	0.366301	0.122596
DSP	W3	PGE2	well_4	F003	236	Avg. Volume	0.535354	0.52668	0.156616
DSP	W3	PGE2	well_4	F004	315	Avg. Volume	0.561848	0.558117	0.174261
DSP	W3	PGE2	well_4	F005	361	Avg. Volume	0.438102	0.410373	0.173731
DSP	W3	PGE2	well_4	F006	234	Avg. Volume	0.445249	0.42392	0.160618
DSP	W3	PGE2	well_5	F001	136	Avg. Volume	0.566857	0.552204	0.169357
DSP	W3	PGE2	well_5	F002	246	Avg. Volume	0.427476	0.418289	0.136712
DSP	W3	PGE2	well_5	F003	436	Avg. Volume	0.427371	0.41596	0.155701
DSP	W3	PGE2	well_5	F004	265	Avg. Volume	0.459711	0.455855	0.19132
DSP	W3	PGE2	well_5	F005	174	Avg. Volume	0.546461	0.517312	0.216364
DSP	W3	PGE2	well_5	F006	327	Avg. Volume	0.528014	0.517111	0.141108
DSP	W4	HPI4	well_1	F001	42	Avg. Volume	0.498406	0.49859	0.113867
DSP	W4	HPI4	well_1	F002	282	Avg. Volume	0.580981	0.574502	0.229423
DSP	W4	HPI4	well_1	F003	219	Avg. Volume	0.546058	0.560818	0.197907
DSP	W4	HPI4	well_1	F004	188	Avg. Volume	0.468221	0.451503	0.169479
DSP	W4	HPI4	well_1	F005	292	Avg. Volume	0.436609	0.421433	0.199026
DSP	W4	HPI4	well_1	F006	197	Avg. Volume	0.351673	0.335086	0.142382
DSP	W4	HPI4	well_2	F001	47	Avg. Volume	0.560672	0.561953	0.144751
DSP	W4	HPI4	well_2	F002	250	Avg. Volume	0.507101	0.509613	0.183127
DSP	W4	HPI4	well_2	F003	192	Avg. Volume	0.406867	0.390813	0.166901

DSP	W4	HPI4	well_2	F004	204	Avg. Volume	0.453544	0.443613	0.159436
DSP	W4	HPI4	well_2	F005	227	Avg. Volume	0.341899	0.324939	0.196729
DSP	W4	HPI4	well_2	F006	198	Avg. Volume	0.448217	0.441938	0.1593
DSP	W4	HPI4	well_3	F001	139	Avg. Volume	0.416685	0.383699	0.152777
DSP	W4	HPI4	well_3	F002	147	Avg. Volume	0.532429	0.539861	0.149722
DSP	W4	HPI4	well_3	F003	97	Avg. Volume	0.436827	0.427102	0.136007
DSP	W4	HPI4	well_3	F004	160	Avg. Volume	0.578134	0.354652	0.563564
DSP	W4	HPI4	well_3	F005	258	Avg. Volume	0.52699	0.522372	0.187696
DSP	W4	HPI4	well_3	F006	169	Avg. Volume	0.458444	0.465532	0.183789
DSP	W4	HPI4	well_4	F001	49	Avg. Volume	0.452158	0.462301	0.134764
DSP	W4	HPI4	well_4	F002	170	Avg. Volume	0.456041	0.428787	0.190936
DSP	W4	HPI4	well_4	F003	84	Avg. Volume	0.322767	0.327842	0.11156
DSP	W4	HPI4	well_4	F004	138	Avg. Volume	0.454681	0.444249	0.186537
DSP	W4	HPI4	well_4	F005	248	Avg. Volume	0.331368	0.313236	0.146293
DSP	W4	HPI4	well_4	F006	128	Avg. Volume	0.432248	0.444184	0.169739
DSP	W4	HPI4	well_5	F001	24	Avg. Volume	0.33772	0.374429	0.106749
DSP	W4	HPI4	well_5	F002	42	Avg. Volume	0.44125	0.469458	0.134383
DSP	W4	HPI4	well_5	F003	168	Avg. Volume	0.431015	0.423079	0.175922
DSP	W4	HPI4	well_5	F004	41	Avg. Volume	0.467049	0.481181	0.216676
DSP	W4	HPI4	well_5	F005	86	Avg. Volume	0.425518	0.425686	0.129306
DSP	W4	HPI4	well_5	F006	105	Avg. Volume	0.332169	0.309833	0.156159
DSP	W4	PGE2	well_1	F001	330	Avg. Volume	0.494057	0.492599	0.18825
DSP	W4	PGE2	well_1	F002	157	Avg. Volume	0.59527	0.547256	0.207879
DSP	W4	PGE2	well_1	F003	217	Avg. Volume	0.52764	0.515889	0.16312
DSP	W4	PGE2	well_1	F004	306	Avg. Volume	0.355798	0.329665	0.122728
DSP	W4	PGE2	well_1	F005	236	Avg. Volume	0.457908	0.425846	0.172975
DSP	W4	PGE2	well_1	F006	281	Avg. Volume	0.485762	0.485938	0.184527
DSP	W4	PGE2	well_2	F001	129	Avg. Volume	0.465232	0.432731	0.158431
DSP	W4	PGE2	well_2	F002	366	Avg. Volume	0.45289	0.409227	0.214038
DSP	W4	PGE2	well_2	F003	348	Avg. Volume	0.562831	0.521867	0.219685
DSP	W4	PGE2	well_2	F004	256	Avg. Volume	0.481298	0.4706	0.133336
DSP	W4	PGE2	well_2	F005	277	Avg. Volume	0.505332	0.465078	0.196992
DSP	W4	PGE2	well_2	F006	330	Avg. Volume	0.447099	0.434053	0.13324
DSP	W4	PGE2	well_3	F001	203	Avg. Volume	0.552737	0.520396	0.244683
DSP	W4	PGE2	well_3	F002	169	Avg. Volume	0.386652	0.331964	0.199549
DSP	W4	PGE2	well_3	F003	277	Avg. Volume	0.545261	0.491755	0.222479
DSP	W4	PGE2	well_3	F004	335	Avg. Volume	0.427075	0.401844	0.147725
DSP	W4	PGE2	well_3	F005	288	Avg. Volume	0.425697	0.379454	0.193738
DSP	W4	PGE2	well_3	F006	318	Avg. Volume	0.468525	0.422927	0.194668
DSP	W4	PGE2	well_4	F001	103	Avg. Volume	0.536457	0.526071	0.149548
DSP	W4	PGE2	well_4	F002	239	Avg. Volume	0.482172	0.469444	0.158679
DSP	W4	PGE2	well_4	F003	373	Avg. Volume	0.477534	0.449468	0.17186
DSP	W4	PGE2	well_4	F004	221	Avg. Volume	0.365344	0.343216	0.148702
DSP	W4	PGE2	well_4	F005	259	Avg. Volume	0.480141	0.468559	0.158471
DSP	W4	PGE2	well_4	F006	202	Avg. Volume	0.473393	0.441654	0.175766
DSP	W4	PGE2	well_5	F001	81	Avg. Volume	0.449485	0.421561	0.169414
DSP	W4	PGE2	well_5	F002	215	Avg. Volume	0.39985	0.386509	0.143872
DSP	W4	PGE2	well_5	F003	304	Avg. Volume	0.414184	0.396759	0.183997
DSP	W4	PGE2	well_5	F004	187	Avg. Volume	0.480148	0.453257	0.152715

DSP	W4	PGE2	well_5	F005	321	Avg. Volume	0.558385	0.548947	0.151935
DSP	W4	PGE2	well_5	F006	263	Avg. Volume	0.355639	0.354511	0.128981
DSP	W1	HPI4	well_1	F001	150	Count/cell	17.83824	14	12.33317
DSP	W1	HPI4	well_1	F002	168	Count/cell	22.10256	20	14.60145
DSP	W1	HPI4	well_1	F003	384	Count/cell	21.66382	22	11.09573
DSP	W1	HPI4	well_1	F004	145	Count/cell	32.70769	32.5	13.44668
DSP	W1	HPI4	well_1	F005	293	Count/cell	25.31061	24	11.75196
DSP	W1	HPI4	well_1	F006	148	Count/cell	20.48507	20	10.17866
DSP	W1	HPI4	well_2	F001	172	Count/cell	22.1871	20	13.4284
DSP	W1	HPI4	well_2	F002	258	Count/cell	17.79487	17	10.20312
DSP	W1	HPI4	well_2	F003	152	Count/cell	20.78676	19	11.39227
DSP	W1	HPI4	well_2	F004	352	Count/cell	18.59568	17	10.5055
DSP	W1	HPI4	well_2	F005	236	Count/cell	28.39252	26	12.86934
DSP	W1	HPI4	well_2	F006	315	Count/cell	25.26056	24	13.23913
DSP	W1	HPI4	well_3	F001	223	Count/cell	23.355	21	11.09661
DSP	W1	HPI4	well_3	F002	266	Count/cell	16.85714	14	9.651297
DSP	W1	HPI4	well_3	F003	191	Count/cell	25.81503	26	10.46869
DSP	W1	HPI4	well_3	F004	324	Count/cell	20.16897	19	9.93695
DSP	W1	HPI4	well_3	F005	209	Count/cell	24.18325	24	11.69449
DSP	W1	HPI4	well_3	F006	213	Count/cell	26.48969	24.5	12.97277
DSP	W1	HPI4	well_4	F001	205	Count/cell	30.34239	29.5	13.71319
DSP	W1	HPI4	well_4	F002	106	Count/cell	13.89109	9	11.5887
DSP	W1	HPI4	well_4	F003	359	Count/cell	20.31231	19	12.8311
DSP	W1	HPI4	well_4	F004	181	Count/cell	21.76163	19	16.62402
DSP	W1	HPI4	well_4	F005	252	Count/cell	27.84716	27	12.25168
DSP	W1	HPI4	well_4	F006	188	Count/cell	23.8869	23	10.60585
DSP	W1	HPI4	well_5	F001	172	Count/cell	25.97452	26	14.68121
DSP	W1	HPI4	well_5	F002	265	Count/cell	21.97166	20	13.33051
DSP	W1	HPI4	well_5	F003	231	Count/cell	20.38389	16	14.96407
DSP	W1	HPI4	well_5	F004	361	Count/cell	22.9052	21	11.95929
DSP	W1	HPI4	well_5	F005	178	Count/cell	27.44785	27	13.64278
DSP	W1	HPI4	well_5	F006	201	Count/cell	22.20652	22	11.43051
DSP	W1	PGE2	well_1	F001	104	Count/cell	18.58696	17.5	8.681358
DSP	W1	PGE2	well_1	F002	161	Count/cell	22.99324	22	15.62289
DSP	W1	PGE2	well_1	F003	332	Count/cell	20.53	20	9.890831
DSP	W1	PGE2	well_1	F004	188	Count/cell	26.27746	24	15.86364
DSP	W1	PGE2	well_1	F005	84	Count/cell	26.63158	23	18.73951
DSP	W1	PGE2	well_1	F006	287	Count/cell	18.55939	18	10.54192
DSP	W1	PGE2	well_2	F001	249	Count/cell	24.95516	22	12.01005
DSP	W1	PGE2	well_2	F002	245	Count/cell	17.38326	17	9.351933
DSP	W1	PGE2	well_2	F003	228	Count/cell	25.71569	25	11.84121
DSP	W1	PGE2	well_2	F004	256	Count/cell	16.51852	15	11.69881
DSP	W1	PGE2	well_2	F005	347	Count/cell	16.69937	16	9.380906
DSP	W1	PGE2	well_2	F006	203	Count/cell	20.04396	19	9.642691
DSP	W1	PGE2	well_3	F001	192	Count/cell	20.58382	19	11.10417
DSP	W1	PGE2	well_3	F002	229	Count/cell	18.88462	18	10.38143
DSP	W1	PGE2	well_3	F003	224	Count/cell	22.095	20	12.4693
DSP	W1	PGE2	well_3	F004	215	Count/cell	17.9697	15	10.86087
DSP	W1	PGE2	well_3	F005	112	Count/cell	29.31683	29	15.37916

DSP	W1	PGE2	well_3	F006	261	Count/cell	22.24473	20	10.64365
DSP	W1	PGE2	well_4	F001	266	Count/cell	29.20747	28	15.65429
DSP	W1	PGE2	well_4	F002	227	Count/cell	28.26471	28	11.42025
DSP	W1	PGE2	well_4	F003	296	Count/cell	22.90299	22	12.5384
DSP	W1	PGE2	well_4	F004	364	Count/cell	17.20846	16	8.777488
DSP	W1	PGE2	well_4	F005	366	Count/cell	19.31343	18	10.67709
DSP	W1	PGE2	well_4	F006	218	Count/cell	25.455	25	13.17406
DSP	W1	PGE2	well_5	F001	112	Count/cell	31.58	32	13.97919
DSP	W1	PGE2	well_5	F002	127	Count/cell	17.43333	12	15.40694
DSP	W1	PGE2	well_5	F003	233	Count/cell	26.6	26	12.32813
DSP	W1	PGE2	well_5	F004	203	Count/cell	26.1768	25	12.41378
DSP	W1	PGE2	well_5	F005	230	Count/cell	22.65385	21	13.18451
DSP	W1	PGE2	well_5	F006	276	Count/cell	20.05159	19	11.39377
DSP	W2	HPI4	well_1	F001	235	Count/cell	19.45327	18	10.85522
DSP	W2	HPI4	well_1	F002	179	Count/cell	26.96933	25	14.97546
DSP	W2	HPI4	well_1	F003	363	Count/cell	9.486957	7	8.304557
DSP	W2	HPI4	well_1	F004	200	Count/cell	22.07609	20	14.10982
DSP	W2	HPI4	well_1	F005	286	Count/cell	25.03788	22	16.97903
DSP	W2	HPI4	well_1	F006	115	Count/cell	37.78095	36	19.07071
DSP	W2	HPI4	well_2	F001	147	Count/cell	21.2	21	12.14207
DSP	W2	HPI4	well_2	F002	177	Count/cell	43.42138	41	17.83908
DSP	W2	HPI4	well_2	F003	329	Count/cell	23.15132	22	12.64899
DSP	W2	HPI4	well_2	F004	272	Count/cell	25.83468	22	15.19911
DSP	W2	HPI4	well_2	F005	185	Count/cell	34.1018	34	17.8456
DSP	W2	HPI4	well_2	F006	275	Count/cell	20.57031	15.5	15.82561
DSP	W2	HPI4	well_3	F001	144	Count/cell	44.26563	41	17.88435
DSP	W2	HPI4	well_3	F002	183	Count/cell	35.05521	36	13.94489
DSP	W2	HPI4	well_3	F003	55	Count/cell	13.5	12.5	10.41963
DSP	W2	HPI4	well_3	F004	288	Count/cell	27.44361	26	12.91739
DSP	W2	HPI4	well_3	F005	289	Count/cell	24.02273	21	14.79516
DSP	W2	HPI4	well_3	F006	277	Count/cell	33.836	29	21.3758
DSP	W2	HPI4	well_4	F001	222	Count/cell	24.65829	24	11.00155
DSP	W2	HPI4	well_4	F002	154	Count/cell	16.91781	15.5	11.1346
DSP	W2	HPI4	well_4	F003	24	Count/cell	11.14286	11	5.790386
DSP	W2	HPI4	well_4	F004	202	Count/cell	15.78534	13	12.18934
DSP	W2	HPI4	well_4	F005	182	Count/cell	21.24848	20	11.05662
DSP	W2	HPI4	well_4	F006	340	Count/cell	27.00645	26	13.43786
DSP	W2	HPI4	well_5	F001	237	Count/cell	21	19	11.10107
DSP	W2	HPI4	well_5	F002	197	Count/cell	29.63277	28	14.76235
DSP	W2	HPI4	well_5	F003	84	Count/cell	19.33784	18	8.762118
DSP	W2	HPI4	well_5	F004	149	Count/cell	35.77778	36	16.84152
DSP	W2	HPI4	well_5	F005	262	Count/cell	35.44444	34.5	16.90161
DSP	W2	HPI4	well_5	F006	160	Count/cell	29.27027	29	14.18085
DSP	W2	PGE2	well_1	F001	286	Count/cell	41.10769	39	21.37087
DSP	W2	PGE2	well_1	F002	342	Count/cell	31.05195	31	14.60235
DSP	W2	PGE2	well_1	F003	375	Count/cell	32.76261	34	14.04955
DSP	W2	PGE2	well_1	F004	315	Count/cell	42.97183	42	16.07357
DSP	W2	PGE2	well_1	F005	340	Count/cell	35.98382	34	17.06338
DSP	W2	PGE2	well_1	F006	393	Count/cell	37.66289	35	14.71653

DSP	W2	PGE2	well_2	F001	331	Count/cell	37.75168	35	13.82547
DSP	W2	PGE2	well_2	F002	375	Count/cell	43.71302	37.5	22.19225
DSP	W2	PGE2	well_2	F003	352	Count/cell	25.39564	19	18.15306
DSP	W2	PGE2	well_2	F004	308	Count/cell	40.54348	40	15.64451
DSP	W2	PGE2	well_2	F005	416	Count/cell	36.936	36	14.1297
DSP	W2	PGE2	well_2	F006	396	Count/cell	37.94382	38	15.71819
DSP	W2	PGE2	well_3	F001	127	Count/cell	23.50413	27	15.97922
DSP	W2	PGE2	well_3	F002	303	Count/cell	36.81618	35	13.12005
DSP	W2	PGE2	well_3	F003	337	Count/cell	41.22039	41	18.50094
DSP	W2	PGE2	well_3	F004	339	Count/cell	30.56494	31	10.18745
DSP	W2	PGE2	well_3	F005	373	Count/cell	33.33929	32	10.34759
DSP	W2	PGE2	well_3	F006	334	Count/cell	36.15282	35	10.80354
DSP	W2	PGE2	well_4	F001	302	Count/cell	33.25556	33	10.36605
DSP	W2	PGE2	well_4	F002	292	Count/cell	37.10943	35	12.22141
DSP	W2	PGE2	well_4	F003	391	Count/cell	28.3711	28	12.56013
DSP	W2	PGE2	well_4	F004	338	Count/cell	32.9085	33	15.86329
DSP	W2	PGE2	well_4	F005	421	Count/cell	37.2231	36	15.19107
DSP	W2	PGE2	well_4	F006	14	Count/cell	134.8333	152	50.8274
DSP	W2	PGE2	well_5	F001	186	Count/cell	23.94611	23	9.086106
DSP	W2	PGE2	well_5	F002	329	Count/cell	42.21017	43	16.08428
DSP	W2	PGE2	well_5	F003	221	Count/cell	26.87065	24	15.94939
DSP	W2	PGE2	well_5	F004	331	Count/cell	37.38538	38	15.35939
DSP	W2	PGE2	well_5	F005	306	Count/cell	33.05455	30	14.32116
DSP	W2	PGE2	well_5	F006	245	Count/cell	33.03167	31	13.81777
DSP	W3	HPI4	well_1	F001	242	Count/cell	34.58525	35	14.03149
DSP	W3	HPI4	well_1	F002	315	Count/cell	22.20979	23	13.03967
DSP	W3	HPI4	well_1	F003	90	Count/cell	14.34568	15	7.242514
DSP	W3	HPI4	well_1	F004	370	Count/cell	21.15964	20	9.979819
DSP	W3	HPI4	well_1	F005	345	Count/cell	27.24841	26	14.82169
DSP	W3	HPI4	well_1	F006	191	Count/cell	41.37209	41	17.89576
DSP	W3	HPI4	well_2	F001	219	Count/cell	28.60606	26.5	15.20841
DSP	W3	HPI4	well_2	F002	195	Count/cell	50.10734	50	22.43994
DSP	W3	HPI4	well_2	F003	260	Count/cell	17.44538	13	12.67756
DSP	W3	HPI4	well_2	F004	310	Count/cell	19.41786	18	10.91514
DSP	W3	HPI4	well_2	F005	212	Count/cell	28.87895	27	15.4194
DSP	W3	HPI4	well_2	F006	279	Count/cell	19.16406	16.5	11.94023
DSP	W3	HPI4	well_3	F001	244	Count/cell	33.97297	29	20.05894
DSP	W3	HPI4	well_3	F002	205	Count/cell	32.19565	33	15.83435
DSP	W3	HPI4	well_3	F003	87	Count/cell	6.45122	4.5	5.484327
DSP	W3	HPI4	well_3	F004	106	Count/cell	10.57	9	7.231043
DSP	W3	HPI4	well_3	F005	327	Count/cell	31.71477	32	12.39973
DSP	W3	HPI4	well_3	F006	366	Count/cell	31.15616	29	19.01251
DSP	W3	HPI4	well_4	F001	180	Count/cell	42.64417	41	20.35812
DSP	W3	HPI4	well_4	F002	99	Count/cell	32.55556	29	19.00864
DSP	W3	HPI4	well_4	F003	42	Count/cell	8.205128	7	5.82273
DSP	W3	HPI4	well_4	F004	91	Count/cell	15.0122	13	8.745361
DSP	W3	HPI4	well_4	F005	507	Count/cell	14.37447	12	9.916936
DSP	W3	HPI4	well_4	F006	305	Count/cell	35.07273	33	16.84369
DSP	W3	HPI4	well_5	F001	152	Count/cell	48.7029	47	24.28673

DSP	W3	HPI4	well_5	F002	59	Count/cell	26.4717	23	15.73165
DSP	W3	HPI4	well_5	F003	3	Count/cell	24	24	0
DSP	W3	HPI4	well_5	F004	29	Count/cell	15.48	16	7.953616
DSP	W3	HPI4	well_5	F005	205	Count/cell	50.42703	49	24.38942
DSP	W3	HPI4	well_5	F006	292	Count/cell	24.07547	21	16.87841
DSP	W3	PGE2	well_1	F001	382	Count/cell	33.74269	34	13.3229
DSP	W3	PGE2	well_1	F002	355	Count/cell	49.8625	44	23.83156
DSP	W3	PGE2	well_1	F003	336	Count/cell	32.75248	30	14.22064
DSP	W3	PGE2	well_1	F004	209	Count/cell	43.51596	38	23.05109
DSP	W3	PGE2	well_1	F005	332	Count/cell	76.58054	73.5	29.15941
DSP	W3	PGE2	well_1	F006	167	Count/cell	81.91275	82	38.95249
DSP	W3	PGE2	well_2	F001	308	Count/cell	37.2491	31	20.10118
DSP	W3	PGE2	well_2	F002	328	Count/cell	81.16216	81	35.48694
DSP	W3	PGE2	well_2	F003	194	Count/cell	29.17978	27	18.32418
DSP	W3	PGE2	well_2	F004	384	Count/cell	32.55942	31	12.70541
DSP	W3	PGE2	well_2	F005	348	Count/cell	70.85577	58	41.1395
DSP	W3	PGE2	well_2	F006	217	Count/cell	45.25628	35	33.86917
DSP	W3	PGE2	well_3	F001	345	Count/cell	53.06796	43	34.9189
DSP	W3	PGE2	well_3	F002	72	Count/cell	40.30882	17.5	42.80928
DSP	W3	PGE2	well_3	F003	339	Count/cell	39.70164	32	22.2144
DSP	W3	PGE2	well_3	F004	371	Count/cell	19.55263	18	11.35208
DSP	W3	PGE2	well_3	F005	291	Count/cell	48.25283	41	26.95859
DSP	W3	PGE2	well_3	F006	358	Count/cell	63.1677	57.5	30.19062
DSP	W3	PGE2	well_4	F001	309	Count/cell	59.64158	50	34.80026
DSP	W3	PGE2	well_4	F002	416	Count/cell	26.18085	24	10.56304
DSP	W3	PGE2	well_4	F003	236	Count/cell	47.83491	42.5	24.02598
DSP	W3	PGE2	well_4	F004	315	Count/cell	52.90845	49	27.76425
DSP	W3	PGE2	well_4	F005	361	Count/cell	34.62006	30	19.86981
DSP	W3	PGE2	well_4	F006	234	Count/cell	70.52857	66	34.80735
DSP	W3	PGE2	well_5	F001	136	Count/cell	39.12903	34	18.83474
DSP	W3	PGE2	well_5	F002	246	Count/cell	52.66364	42	34.86655
DSP	W3	PGE2	well_5	F003	436	Count/cell	24.699	20	16.07267
DSP	W3	PGE2	well_5	F004	265	Count/cell	35.59917	29	25.70872
DSP	W3	PGE2	well_5	F005	174	Count/cell	77.15385	75.5	42.08734
DSP	W3	PGE2	well_5	F006	327	Count/cell	63.18707	51.5	33.12942
DSP	W4	HPI4	well_1	F001	42	Count/cell	47.97222	47.5	15.339
DSP	W4	HPI4	well_1	F002	282	Count/cell	34.17063	33	12.62415
DSP	W4	HPI4	well_1	F003	219	Count/cell	33.59091	34	13.02007
DSP	W4	HPI4	well_1	F004	188	Count/cell	27.82558	29	12.25431
DSP	W4	HPI4	well_1	F005	292	Count/cell	13.15523	11	8.828545
DSP	W4	HPI4	well_1	F006	197	Count/cell	27.38547	24	15.253
DSP	W4	HPI4	well_2	F001	47	Count/cell	42.7561	42	10.86228
DSP	W4	HPI4	well_2	F002	250	Count/cell	30.87054	31	10.80509
DSP	W4	HPI4	well_2	F003	192	Count/cell	19.46243	18	11.03404
DSP	W4	HPI4	well_2	F004	204	Count/cell	22.30601	20	10.40521
DSP	W4	HPI4	well_2	F005	227	Count/cell	17.74074	12	17.28065
DSP	W4	HPI4	well_2	F006	198	Count/cell	28.28889	29	14.15181
DSP	W4	HPI4	well_3	F001	139	Count/cell	28.9127	29	12.88101
DSP	W4	HPI4	well_3	F002	147	Count/cell	39.12977	38	9.63918

DSP	W4	HPI4	well_3	F003	97	Count/cell	37.18391	37	16.98873
DSP	W4	HPI4	well_3	F004	160	Count/cell	22.1931	21	14.1413
DSP	W4	HPI4	well_3	F005	258	Count/cell	18.14163	17	9.60476
DSP	W4	HPI4	well_3	F006	169	Count/cell	26.75163	25	14.18964
DSP	W4	HPI4	well_4	F001	49	Count/cell	33.83721	34	13.28252
DSP	W4	HPI4	well_4	F002	170	Count/cell	24.48052	24	9.790098
DSP	W4	HPI4	well_4	F003	84	Count/cell	23.63158	22.5	11.07772
DSP	W4	HPI4	well_4	F004	138	Count/cell	22.66929	21	11.06335
DSP	W4	HPI4	well_4	F005	248	Count/cell	14.2287	13	8.824959
DSP	W4	HPI4	well_4	F006	128	Count/cell	33.30833	30	18.4877
DSP	W4	HPI4	well_5	F001	24	Count/cell	23.25	16.5	16.19251
DSP	W4	HPI4	well_5	F002	42	Count/cell	26.83333	25	10.84304
DSP	W4	HPI4	well_5	F003	168	Count/cell	25.81333	25	13.4266
DSP	W4	HPI4	well_5	F004	41	Count/cell	14.35	13.5	7.879054
DSP	W4	HPI4	well_5	F005	86	Count/cell	26.8961	25	13.77506
DSP	W4	HPI4	well_5	F006	105	Count/cell	16.51042	14	10.10888
DSP	W4	PGE2	well_1	F001	330	Count/cell	48.77104	43	30.3786
DSP	W4	PGE2	well_1	F002	157	Count/cell	94.02113	89	48.23001
DSP	W4	PGE2	well_1	F003	217	Count/cell	66.77949	65	34.64336
DSP	W4	PGE2	well_1	F004	306	Count/cell	67.45255	62.5	32.03969
DSP	W4	PGE2	well_1	F005	236	Count/cell	69.52093	60	44.48467
DSP	W4	PGE2	well_1	F006	281	Count/cell	51.85771	39	35.0912
DSP	W4	PGE2	well_2	F001	129	Count/cell	84.81034	82.5	34.24945
DSP	W4	PGE2	well_2	F002	366	Count/cell	79.40909	79	42.98723
DSP	W4	PGE2	well_2	F003	348	Count/cell	86.31429	87	35.58295
DSP	W4	PGE2	well_2	F004	256	Count/cell	48.86957	40	28.56404
DSP	W4	PGE2	well_2	F005	277	Count/cell	83.028	83.5	37.2919
DSP	W4	PGE2	well_2	F006	330	Count/cell	58.56954	52	30.88151
DSP	W4	PGE2	well_3	F001	203	Count/cell	85.34254	82	38.20477
DSP	W4	PGE2	well_3	F002	169	Count/cell	55.09677	47	44.24486
DSP	W4	PGE2	well_3	F003	277	Count/cell	82.50201	79	37.45849
DSP	W4	PGE2	well_3	F004	335	Count/cell	63.02632	57	35.96541
DSP	W4	PGE2	well_3	F005	288	Count/cell	88.29119	89	36.06255
DSP	W4	PGE2	well_3	F006	318	Count/cell	91.09441	94.5	33.06126
DSP	W4	PGE2	well_4	F001	103	Count/cell	83.31522	85.5	35.58921
DSP	W4	PGE2	well_4	F002	239	Count/cell	65.73272	59	48.60882
DSP	W4	PGE2	well_4	F003	373	Count/cell	62.16176	59	33.96033
DSP	W4	PGE2	well_4	F004	221	Count/cell	25.27053	21	18.7791
DSP	W4	PGE2	well_4	F005	259	Count/cell	67.72961	62	35.07959
DSP	W4	PGE2	well_4	F006	202	Count/cell	76.33889	70	34.2963
DSP	W4	PGE2	well_5	F001	81	Count/cell	41.72603	35	28.08087
DSP	W4	PGE2	well_5	F002	215	Count/cell	58.63731	50	35.80318
DSP	W4	PGE2	well_5	F003	304	Count/cell	42.51103	31	34.53454
DSP	W4	PGE2	well_5	F004	187	Count/cell	43.58333	40.5	23.10699
DSP	W4	PGE2	well_5	F005	321	Count/cell	35.76976	33	14.72806
DSP	W4	PGE2	well_5	F006	263	Count/cell	27.13983	25	14.90172
DSP	W1	HPI4	well_1	F001	150	X/Y distribution	9.281068	9.098704	1.971684
DSP	W1	HPI4	well_1	F002	168	X/Y distribution	9.778081	9.704683	2.158244
DSP	W1	HPI4	well_1	F003	384	X/Y distribution	7.495293	7.344293	1.340431

DSP	W1	HPI4	well_1	F004	145	X/Y distribution	10.14763	10.00545	1.881931
DSP	W1	HPI4	well_1	F005	293	X/Y distribution	8.127597	8.03874	1.38375
DSP	W1	HPI4	well_1	F006	148	X/Y distribution	9.49667	9.2481	1.991916
DSP	W1	HPI4	well_2	F001	172	X/Y distribution	9.261776	8.880604	1.940215
DSP	W1	HPI4	well_2	F002	258	X/Y distribution	8.202219	8.165431	1.770743
DSP	W1	HPI4	well_2	F003	152	X/Y distribution	9.816218	9.919556	2.062869
DSP	W1	HPI4	well_2	F004	352	X/Y distribution	7.782864	7.779907	1.570029
DSP	W1	HPI4	well_2	F005	236	X/Y distribution	8.602787	8.580092	1.676126
DSP	W1	HPI4	well_2	F006	315	X/Y distribution	7.969258	7.888375	1.492055
DSP	W1	HPI4	well_3	F001	223	X/Y distribution	8.682669	8.549206	1.615091
DSP	W1	HPI4	well_3	F002	266	X/Y distribution	8.498555	8.377069	1.538911
DSP	W1	HPI4	well_3	F003	191	X/Y distribution	9.327791	9.23079	2.019939
DSP	W1	HPI4	well_3	F004	324	X/Y distribution	7.510016	7.439756	1.535437
DSP	W1	HPI4	well_3	F005	209	X/Y distribution	9.027261	8.967566	1.640387
DSP	W1	HPI4	well_3	F006	213	X/Y distribution	8.78672	8.598731	1.636322
DSP	W1	HPI4	well_4	F001	205	X/Y distribution	9.183554	8.929331	1.825796
DSP	W1	HPI4	well_4	F002	106	X/Y distribution	8.222841	7.876734	1.602734
DSP	W1	HPI4	well_4	F003	359	X/Y distribution	7.637011	7.490553	1.607685
DSP	W1	HPI4	well_4	F004	181	X/Y distribution	8.690677	8.237592	2.193799
DSP	W1	HPI4	well_4	F005	252	X/Y distribution	8.659789	8.672325	1.561782
DSP	W1	HPI4	well_4	F006	188	X/Y distribution	9.022801	9.032585	1.553479
DSP	W1	HPI4	well_5	F001	172	X/Y distribution	9.251141	9.185435	1.963516
DSP	W1	HPI4	well_5	F002	265	X/Y distribution	8.304357	8.222617	1.630812
DSP	W1	HPI4	well_5	F003	231	X/Y distribution	8.439227	8.253302	1.963715
DSP	W1	HPI4	well_5	F004	361	X/Y distribution	7.552388	7.515036	1.603111
DSP	W1	HPI4	well_5	F005	178	X/Y distribution	9.3445	9.417102	1.866156
DSP	W1	HPI4	well_5	F006	201	X/Y distribution	8.571479	8.6109	1.678167
DSP	W1	PGE2	well_1	F001	104	X/Y distribution	9.82905	9.585806	1.62179
DSP	W1	PGE2	well_1	F002	161	X/Y distribution	9.407342	9.407223	2.072012
DSP	W1	PGE2	well_1	F003	332	X/Y distribution	7.732561	7.694634	1.389952
DSP	W1	PGE2	well_1	F004	188	X/Y distribution	9.162046	9.000314	1.733589
DSP	W1	PGE2	well_1	F005	84	X/Y distribution	10.17729	9.984877	2.243681
DSP	W1	PGE2	well_1	F006	287	X/Y distribution	7.837116	7.817488	1.33191
DSP	W1	PGE2	well_2	F001	249	X/Y distribution	8.168546	8.016014	1.622884
DSP	W1	PGE2	well_2	F002	245	X/Y distribution	8.310207	8.279435	1.525948
DSP	W1	PGE2	well_2	F003	228	X/Y distribution	8.880249	8.85487	1.609483
DSP	W1	PGE2	well_2	F004	256	X/Y distribution	8.209838	8.018867	1.657482
DSP	W1	PGE2	well_2	F005	347	X/Y distribution	7.499788	7.356528	1.513498
DSP	W1	PGE2	well_2	F006	203	X/Y distribution	8.287919	8.215968	1.584629
DSP	W1	PGE2	well_3	F001	192	X/Y distribution	8.961631	8.818026	1.739074
DSP	W1	PGE2	well_3	F002	229	X/Y distribution	8.035831	7.764601	1.429643
DSP	W1	PGE2	well_3	F003	224	X/Y distribution	8.768577	8.630287	1.738333
DSP	W1	PGE2	well_3	F004	215	X/Y distribution	8.878553	8.714553	1.771544
DSP	W1	PGE2	well_3	F005	112	X/Y distribution	10.77042	10.57753	2.065442
DSP	W1	PGE2	well_3	F006	261	X/Y distribution	7.869709	7.846195	1.508438
DSP	W1	PGE2	well_4	F001	266	X/Y distribution	8.676203	8.456552	1.513551
DSP	W1	PGE2	well_4	F002	227	X/Y distribution	8.609266	8.551433	1.399222
DSP	W1	PGE2	well_4	F003	296	X/Y distribution	8.029282	7.906351	1.876093
DSP	W1	PGE2	well_4	F004	364	X/Y distribution	7.011915	6.87477	1.437701

DSP	W1	PGE2	well_4	F005	366	X/Y distribution	7.63271	7.522427	1.415073
DSP	W1	PGE2	well_4	F006	218	X/Y distribution	8.974168	8.968643	1.611223
DSP	W1	PGE2	well_5	F001	112	X/Y distribution	10.71602	10.65758	2.202211
DSP	W1	PGE2	well_5	F002	127	X/Y distribution	9.647444	9.364735	1.839128
DSP	W1	PGE2	well_5	F003	233	X/Y distribution	8.74616	8.611011	1.806475
DSP	W1	PGE2	well_5	F004	203	X/Y distribution	9.262384	9.093395	1.788545
DSP	W1	PGE2	well_5	F005	230	X/Y distribution	8.493581	8.197477	2.228617
DSP	W1	PGE2	well_5	F006	276	X/Y distribution	8.226567	8.119504	1.606349
DSP	W2	HPI4	well_1	F001	235	X/Y distribution	7.535027	7.333075	1.65136
DSP	W2	HPI4	well_1	F002	179	X/Y distribution	8.194489	8.001551	1.786939
DSP	W2	HPI4	well_1	F003	363	X/Y distribution	6.329936	6.198508	1.487137
DSP	W2	HPI4	well_1	F004	200	X/Y distribution	7.872116	7.538279	2.065273
DSP	W2	HPI4	well_1	F005	286	X/Y distribution	7.329798	7.154545	1.75578
DSP	W2	HPI4	well_1	F006	115	X/Y distribution	9.063471	8.785932	1.680903
DSP	W2	HPI4	well_2	F001	147	X/Y distribution	8.309343	8.255178	1.60549
DSP	W2	HPI4	well_2	F002	177	X/Y distribution	8.784301	8.584907	1.865001
DSP	W2	HPI4	well_2	F003	329	X/Y distribution	7.152323	7.059872	1.503631
DSP	W2	HPI4	well_2	F004	272	X/Y distribution	8.101104	7.796244	2.040119
DSP	W2	HPI4	well_2	F005	185	X/Y distribution	8.761411	8.533419	1.784368
DSP	W2	HPI4	well_2	F006	275	X/Y distribution	7.456148	7.173952	1.953033
DSP	W2	HPI4	well_3	F001	144	X/Y distribution	9.551451	9.478407	1.554424
DSP	W2	HPI4	well_3	F002	183	X/Y distribution	9.308386	9.389274	1.558394
DSP	W2	HPI4	well_3	F003	55	X/Y distribution	9.634588	9.264509	1.868772
DSP	W2	HPI4	well_3	F004	288	X/Y distribution	7.871818	7.673049	1.477267
DSP	W2	HPI4	well_3	F005	289	X/Y distribution	7.632093	7.428727	1.802185
DSP	W2	HPI4	well_3	F006	277	X/Y distribution	7.649985	7.46871	1.618845
DSP	W2	HPI4	well_4	F001	222	X/Y distribution	7.866221	7.971219	1.299149
DSP	W2	HPI4	well_4	F002	154	X/Y distribution	7.964887	7.964048	1.78994
DSP	W2	HPI4	well_4	F003	24	X/Y distribution	9.308646	9.461877	1.952751
DSP	W2	HPI4	well_4	F004	202	X/Y distribution	7.542664	7.302576	1.58301
DSP	W2	HPI4	well_4	F005	182	X/Y distribution	6.31001	6.1457	1.163485
DSP	W2	HPI4	well_4	F006	340	X/Y distribution	6.593475	6.611003	1.140513
DSP	W2	HPI4	well_5	F001	237	X/Y distribution	7.913784	7.900267	1.437515
DSP	W2	HPI4	well_5	F002	197	X/Y distribution	8.675808	8.794783	1.790699
DSP	W2	HPI4	well_5	F003	84	X/Y distribution	9.077069	8.972459	1.74625
DSP	W2	HPI4	well_5	F004	149	X/Y distribution	8.201554	8.216016	1.585883
DSP	W2	HPI4	well_5	F005	262	X/Y distribution	6.925376	6.826091	1.302727
DSP	W2	HPI4	well_5	F006	160	X/Y distribution	6.368854	6.352806	1.197704
DSP	W2	PGE2	well_1	F001	286	X/Y distribution	6.552754	6.459289	1.091595
DSP	W2	PGE2	well_1	F002	342	X/Y distribution	6.14354	6.088232	0.853674
DSP	W2	PGE2	well_1	F003	375	X/Y distribution	6.700591	6.620067	1.226879
DSP	W2	PGE2	well_1	F004	315	X/Y distribution	6.321488	6.252742	0.936515
DSP	W2	PGE2	well_1	F005	340	X/Y distribution	5.895335	5.737723	1.000231
DSP	W2	PGE2	well_1	F006	393	X/Y distribution	5.878109	5.85409	0.799231
DSP	W2	PGE2	well_2	F001	331	X/Y distribution	5.982479	5.941162	0.668827
DSP	W2	PGE2	well_2	F002	375	X/Y distribution	5.650714	5.610475	0.63674
DSP	W2	PGE2	well_2	F003	352	X/Y distribution	5.9487	5.885972	1.014575
DSP	W2	PGE2	well_2	F004	308	X/Y distribution	6.492911	6.454572	1.045652
DSP	W2	PGE2	well_2	F005	416	X/Y distribution	5.934629	5.862353	0.784611

DSP	W2	PGE2	well_2	F006	396	X/Y distribution	6.52516	6.475567	0.954627
DSP	W2	PGE2	well_3	F001	127	X/Y distribution	5.974759	5.763993	1.015726
DSP	W2	PGE2	well_3	F002	303	X/Y distribution	6.742878	6.659496	0.987871
DSP	W2	PGE2	well_3	F003	337	X/Y distribution	7.198479	7.118376	1.248991
DSP	W2	PGE2	well_3	F004	339	X/Y distribution	5.689368	5.612527	0.73964
DSP	W2	PGE2	well_3	F005	373	X/Y distribution	5.722373	5.595991	0.905649
DSP	W2	PGE2	well_3	F006	334	X/Y distribution	5.945323	5.859472	0.865265
DSP	W2	PGE2	well_4	F001	302	X/Y distribution	5.975834	5.962272	0.80749
DSP	W2	PGE2	well_4	F002	292	X/Y distribution	6.265678	6.030102	1.095679
DSP	W2	PGE2	well_4	F003	391	X/Y distribution	6.424162	6.261751	1.044728
DSP	W2	PGE2	well_4	F004	338	X/Y distribution	6.476736	6.303725	1.212392
DSP	W2	PGE2	well_4	F005	421	X/Y distribution	6.148205	6.036018	0.971194
DSP	W2	PGE2	well_4	F006	14	X/Y distribution	5.144679	5.23901	0.468952
DSP	W2	PGE2	well_5	F001	186	X/Y distribution	5.749823	5.714005	0.873409
DSP	W2	PGE2	well_5	F002	329	X/Y distribution	6.577321	6.487691	0.913414
DSP	W2	PGE2	well_5	F003	221	X/Y distribution	6.805553	6.646671	1.150602
DSP	W2	PGE2	well_5	F004	331	X/Y distribution	6.654712	6.454622	1.092661
DSP	W2	PGE2	well_5	F005	306	X/Y distribution	5.714794	5.614413	0.734952
DSP	W2	PGE2	well_5	F006	245	X/Y distribution	5.668473	5.65428	0.717644
DSP	W3	HPI4	well_1	F001	242	X/Y distribution	7.659003	7.510928	1.228804
DSP	W3	HPI4	well_1	F002	315	X/Y distribution	7.536864	7.409465	1.396218
DSP	W3	HPI4	well_1	F003	90	X/Y distribution	9.283737	9.028403	1.805382
DSP	W3	HPI4	well_1	F004	370	X/Y distribution	7.146096	7.053633	1.307081
DSP	W3	HPI4	well_1	F005	345	X/Y distribution	7.57615	7.504811	1.435379
DSP	W3	HPI4	well_1	F006	191	X/Y distribution	8.833241	8.747765	1.77027
DSP	W3	HPI4	well_2	F001	219	X/Y distribution	8.06247	7.934057	1.5296
DSP	W3	HPI4	well_2	F002	195	X/Y distribution	8.900547	8.853589	1.300534
DSP	W3	HPI4	well_2	F003	260	X/Y distribution	7.554385	7.212242	1.676382
DSP	W3	HPI4	well_2	F004	310	X/Y distribution	7.765316	7.738501	1.333541
DSP	W3	HPI4	well_2	F005	212	X/Y distribution	9.031144	8.91821	1.646846
DSP	W3	HPI4	well_2	F006	279	X/Y distribution	7.669867	7.538758	1.479929
DSP	W3	HPI4	well_3	F001	244	X/Y distribution	7.821914	7.696206	1.490114
DSP	W3	HPI4	well_3	F002	205	X/Y distribution	8.711947	8.633722	1.87134
DSP	W3	HPI4	well_3	F003	87	X/Y distribution	8.959314	8.808094	2.237852
DSP	W3	HPI4	well_3	F004	106	X/Y distribution	8.217716	8.017034	1.892627
DSP	W3	HPI4	well_3	F005	327	X/Y distribution	7.240993	7.19597	1.139999
DSP	W3	HPI4	well_3	F006	366	X/Y distribution	6.695622	6.598833	1.261799
DSP	W3	HPI4	well_4	F001	180	X/Y distribution	8.738491	8.647621	1.500553
DSP	W3	HPI4	well_4	F002	99	X/Y distribution	10.31256	10.54159	2.274802
DSP	W3	HPI4	well_4	F003	42	X/Y distribution	10.24751	9.914215	2.238354
DSP	W3	HPI4	well_4	F004	91	X/Y distribution	9.099237	9.143235	2.060498
DSP	W3	HPI4	well_4	F005	507	X/Y distribution	6.652456	6.513062	1.173364
DSP	W3	HPI4	well_4	F006	305	X/Y distribution	7.519877	7.422025	1.391688
DSP	W3	HPI4	well_5	F001	152	X/Y distribution	9.22242	9.240023	1.751167
DSP	W3	HPI4	well_5	F002	59	X/Y distribution	9.959904	10.08051	1.929606
DSP	W3	HPI4	well_5	F003	3	X/Y distribution	14.76101	14.76101	NA
DSP	W3	HPI4	well_5	F004	29	X/Y distribution	11.36192	11.49052	1.909297
DSP	W3	HPI4	well_5	F005	205	X/Y distribution	8.582719	8.603531	1.630849
DSP	W3	HPI4	well_5	F006	292	X/Y distribution	7.501725	7.384336	1.526229

DSP	W3	PGE2	well_1	F001	382	X/Y distribution	6.815091	6.795671	0.93913
DSP	W3	PGE2	well_1	F002	355	X/Y distribution	6.35548	6.255314	0.83719
DSP	W3	PGE2	well_1	F003	336	X/Y distribution	6.49354	6.341584	0.913504
DSP	W3	PGE2	well_1	F004	209	X/Y distribution	5.939241	5.862509	0.776255
DSP	W3	PGE2	well_1	F005	332	X/Y distribution	5.54006	5.463502	0.63416
DSP	W3	PGE2	well_1	F006	167	X/Y distribution	4.988063	4.941291	0.558197
DSP	W3	PGE2	well_2	F001	308	X/Y distribution	6.188541	6.206455	0.783059
DSP	W3	PGE2	well_2	F002	328	X/Y distribution	5.395444	5.407519	0.446336
DSP	W3	PGE2	well_2	F003	194	X/Y distribution	5.853669	5.825042	0.931187
DSP	W3	PGE2	well_2	F004	384	X/Y distribution	6.218151	6.203167	0.809646
DSP	W3	PGE2	well_2	F005	348	X/Y distribution	6.081985	6.017798	0.770503
DSP	W3	PGE2	well_2	F006	217	X/Y distribution	5.476803	5.381901	0.801294
DSP	W3	PGE2	well_3	F001	345	X/Y distribution	6.410531	6.332027	0.904285
DSP	W3	PGE2	well_3	F002	72	X/Y distribution	5.213947	5.062518	0.977726
DSP	W3	PGE2	well_3	F003	339	X/Y distribution	5.868275	5.760759	0.865101
DSP	W3	PGE2	well_3	F004	371	X/Y distribution	6.419066	6.36691	0.907068
DSP	W3	PGE2	well_3	F005	291	X/Y distribution	6.150495	6.093497	0.681087
DSP	W3	PGE2	well_3	F006	358	X/Y distribution	6.037641	5.980999	0.668632
DSP	W3	PGE2	well_4	F001	309	X/Y distribution	6.101039	6.088686	0.676525
DSP	W3	PGE2	well_4	F002	416	X/Y distribution	6.086537	6.089942	0.726247
DSP	W3	PGE2	well_4	F003	236	X/Y distribution	6.240365	6.176285	0.82314
DSP	W3	PGE2	well_4	F004	315	X/Y distribution	6.154128	6.025259	0.731878
DSP	W3	PGE2	well_4	F005	361	X/Y distribution	6.257363	6.234649	0.861154
DSP	W3	PGE2	well_4	F006	234	X/Y distribution	5.817664	5.805094	0.562592
DSP	W3	PGE2	well_5	F001	136	X/Y distribution	6.344573	6.236721	0.950953
DSP	W3	PGE2	well_5	F002	246	X/Y distribution	5.909543	5.864057	0.80669
DSP	W3	PGE2	well_5	F003	436	X/Y distribution	6.010153	5.935623	0.744162
DSP	W3	PGE2	well_5	F004	265	X/Y distribution	5.840495	5.770258	0.663003
DSP	W3	PGE2	well_5	F005	174	X/Y distribution	5.199072	5.059772	0.679213
DSP	W3	PGE2	well_5	F006	327	X/Y distribution	5.852858	5.826276	0.736102
DSP	W4	HPI4	well_1	F001	42	X/Y distribution	9.058198	9.019318	1.356077
DSP	W4	HPI4	well_1	F002	282	X/Y distribution	7.678466	7.612437	1.331439
DSP	W4	HPI4	well_1	F003	219	X/Y distribution	8.364109	8.204167	1.442266
DSP	W4	HPI4	well_1	F004	188	X/Y distribution	9.13491	9.172632	1.595028
DSP	W4	HPI4	well_1	F005	292	X/Y distribution	7.504222	7.36402	1.754894
DSP	W4	HPI4	well_1	F006	197	X/Y distribution	8.650715	8.443324	1.784059
DSP	W4	HPI4	well_2	F001	47	X/Y distribution	10.21988	9.934201	1.382346
DSP	W4	HPI4	well_2	F002	250	X/Y distribution	8.356835	8.320014	1.450815
DSP	W4	HPI4	well_2	F003	192	X/Y distribution	8.239692	8.082343	1.73759
DSP	W4	HPI4	well_2	F004	204	X/Y distribution	8.260789	8.15329	1.883434
DSP	W4	HPI4	well_2	F005	227	X/Y distribution	7.933554	7.855005	2.042051
DSP	W4	HPI4	well_2	F006	198	X/Y distribution	8.454078	8.432967	1.50157
DSP	W4	HPI4	well_3	F001	139	X/Y distribution	8.109078	8.032429	1.47518
DSP	W4	HPI4	well_3	F002	147	X/Y distribution	8.998157	8.810839	1.159125
DSP	W4	HPI4	well_3	F003	97	X/Y distribution	9.847491	9.565329	2.014652
DSP	W4	HPI4	well_3	F004	160	X/Y distribution	7.651045	7.532219	2.184613
DSP	W4	HPI4	well_3	F005	258	X/Y distribution	8.23274	8.327545	1.347107
DSP	W4	HPI4	well_3	F006	169	X/Y distribution	8.012286	8.183094	1.634959
DSP	W4	HPI4	well_4	F001	49	X/Y distribution	8.341382	8.425647	1.711514

DSP	W4	HPI4	well_4	F002	170	X/Y distribution	8.975542	8.990794	1.547039
DSP	W4	HPI4	well_4	F003	84	X/Y distribution	8.822993	8.41855	1.888452
DSP	W4	HPI4	well_4	F004	138	X/Y distribution	7.833157	7.70911	1.756221
DSP	W4	HPI4	well_4	F005	248	X/Y distribution	7.787565	7.476256	1.916195
DSP	W4	HPI4	well_4	F006	128	X/Y distribution	8.770599	8.867614	1.815715
DSP	W4	HPI4	well_5	F001	24	X/Y distribution	9.391081	8.797275	2.011165
DSP	W4	HPI4	well_5	F002	42	X/Y distribution	8.561933	7.876502	2.124334
DSP	W4	HPI4	well_5	F003	168	X/Y distribution	9.142743	9.019814	1.657548
DSP	W4	HPI4	well_5	F004	41	X/Y distribution	4.707896	4.144335	1.827798
DSP	W4	HPI4	well_5	F005	86	X/Y distribution	9.88503	9.765583	2.458209
DSP	W4	HPI4	well_5	F006	105	X/Y distribution	8.419504	8.383459	1.551253
DSP	W4	PGE2	well_1	F001	330	X/Y distribution	6.534713	6.509497	0.722324
DSP	W4	PGE2	well_1	F002	157	X/Y distribution	6.189501	6.106908	0.722422
DSP	W4	PGE2	well_1	F003	217	X/Y distribution	5.747777	5.691686	0.564853
DSP	W4	PGE2	well_1	F004	306	X/Y distribution	5.441233	5.370546	0.507337
DSP	W4	PGE2	well_1	F005	236	X/Y distribution	6.155018	5.980637	0.809682
DSP	W4	PGE2	well_1	F006	281	X/Y distribution	6.624421	6.587582	0.706885
DSP	W4	PGE2	well_2	F001	129	X/Y distribution	5.910807	5.850442	0.537063
DSP	W4	PGE2	well_2	F002	366	X/Y distribution	5.426376	5.376661	0.530765
DSP	W4	PGE2	well_2	F003	348	X/Y distribution	5.859624	5.837694	0.503647
DSP	W4	PGE2	well_2	F004	256	X/Y distribution	6.053231	6.032317	0.63002
DSP	W4	PGE2	well_2	F005	277	X/Y distribution	5.688617	5.666998	0.477603
DSP	W4	PGE2	well_2	F006	330	X/Y distribution	6.483568	6.33928	0.692895
DSP	W4	PGE2	well_3	F001	203	X/Y distribution	6.224894	6.237957	0.583287
DSP	W4	PGE2	well_3	F002	169	X/Y distribution	5.691851	5.692687	0.723034
DSP	W4	PGE2	well_3	F003	277	X/Y distribution	5.788798	5.709971	0.576063
DSP	W4	PGE2	well_3	F004	335	X/Y distribution	5.429537	5.416085	0.45755
DSP	W4	PGE2	well_3	F005	288	X/Y distribution	5.147283	5.134241	0.461901
DSP	W4	PGE2	well_3	F006	318	X/Y distribution	5.324341	5.264482	0.480144
DSP	W4	PGE2	well_4	F001	103	X/Y distribution	6.472	6.449918	0.650037
DSP	W4	PGE2	well_4	F002	239	X/Y distribution	6.17718	6.100879	0.770182
DSP	W4	PGE2	well_4	F003	373	X/Y distribution	6.008812	5.952903	0.723621
DSP	W4	PGE2	well_4	F004	221	X/Y distribution	6.671682	6.575711	1.008927
DSP	W4	PGE2	well_4	F005	259	X/Y distribution	5.768808	5.609442	0.836732
DSP	W4	PGE2	well_4	F006	202	X/Y distribution	6.584603	6.446448	0.868557
DSP	W4	PGE2	well_5	F001	81	X/Y distribution	6.719374	6.583853	0.87416
DSP	W4	PGE2	well_5	F002	215	X/Y distribution	5.862522	5.880823	0.485222
DSP	W4	PGE2	well_5	F003	304	X/Y distribution	6.119334	6.057292	0.881088
DSP	W4	PGE2	well_5	F004	187	X/Y distribution	6.076022	5.987788	0.721165
DSP	W4	PGE2	well_5	F005	321	X/Y distribution	6.770877	6.713836	0.787289
DSP	W4	PGE2	well_5	F006	263	X/Y distribution	6.861316	6.792929	1.010332
DSP	W1	HPI4	well_1	F001	150	Z distribution	0.467263	0.461454	0.510252
DSP	W1	HPI4	well_1	F002	168	Z distribution	0.272413	0.264429	0.472996
DSP	W1	HPI4	well_1	F003	384	Z distribution	0.791964	0.740714	0.756471
DSP	W1	HPI4	well_1	F004	145	Z distribution	0.221413	0.19895	0.38149
DSP	W1	HPI4	well_1	F005	293	Z distribution	0.522311	0.403628	0.609704
DSP	W1	HPI4	well_1	F006	148	Z distribution	0.347368	0.31966	0.453451
DSP	W1	HPI4	well_2	F001	172	Z distribution	0.354573	0.27383	0.533595
DSP	W1	HPI4	well_2	F002	258	Z distribution	0.553851	0.524959	0.703702

DSP	W1	HPI4	well_2	F003	152	Z distribution	0.2253	0.21809	0.460631
DSP	W1	HPI4	well_2	F004	352	Z distribution	0.743663	0.601822	0.849107
DSP	W1	HPI4	well_2	F005	236	Z distribution	0.493105	0.427149	0.5251
DSP	W1	HPI4	well_2	F006	315	Z distribution	0.669488	0.643951	0.682703
DSP	W1	HPI4	well_3	F001	223	Z distribution	0.747321	0.645511	0.574546
DSP	W1	HPI4	well_3	F002	266	Z distribution	0.867537	0.719369	0.738725
DSP	W1	HPI4	well_3	F003	191	Z distribution	0.431625	0.347456	0.519868
DSP	W1	HPI4	well_3	F004	324	Z distribution	0.792579	0.745494	0.789021
DSP	W1	HPI4	well_3	F005	209	Z distribution	0.674451	0.665936	0.568955
DSP	W1	HPI4	well_3	F006	213	Z distribution	0.585681	0.575524	0.533067
DSP	W1	HPI4	well_4	F001	205	Z distribution	0.453114	0.379923	0.556571
DSP	W1	HPI4	well_4	F002	106	Z distribution	0.973321	1.089796	0.630346
DSP	W1	HPI4	well_4	F003	359	Z distribution	0.829377	0.69688	0.811765
DSP	W1	HPI4	well_4	F004	181	Z distribution	0.383773	0.260027	0.717181
DSP	W1	HPI4	well_4	F005	252	Z distribution	0.486398	0.433077	0.483461
DSP	W1	HPI4	well_4	F006	188	Z distribution	0.414008	0.337431	0.458588
DSP	W1	HPI4	well_5	F001	172	Z distribution	0.495235	0.375601	0.569381
DSP	W1	HPI4	well_5	F002	265	Z distribution	0.638863	0.567018	0.734257
DSP	W1	HPI4	well_5	F003	231	Z distribution	0.770936	0.523343	0.87562
DSP	W1	HPI4	well_5	F004	361	Z distribution	0.733967	0.555001	0.867926
DSP	W1	HPI4	well_5	F005	178	Z distribution	0.405471	0.358648	0.518688
DSP	W1	HPI4	well_5	F006	201	Z distribution	0.749094	0.73246	0.691479
DSP	W1	PGE2	well_1	F001	104	Z distribution	0.483828	0.518866	0.447848
DSP	W1	PGE2	well_1	F002	161	Z distribution	0.147178	0.169974	0.572205
DSP	W1	PGE2	well_1	F003	332	Z distribution	1.03515	0.996797	0.803935
DSP	W1	PGE2	well_1	F004	188	Z distribution	0.375142	0.358968	0.476056
DSP	W1	PGE2	well_1	F005	84	Z distribution	0.204891	0.22471	0.573595
DSP	W1	PGE2	well_1	F006	287	Z distribution	0.868332	0.907572	0.660933
DSP	W1	PGE2	well_2	F001	249	Z distribution	0.663425	0.656205	0.600589
DSP	W1	PGE2	well_2	F002	245	Z distribution	0.776263	0.70081	0.747294
DSP	W1	PGE2	well_2	F003	228	Z distribution	0.463086	0.436	0.533309
DSP	W1	PGE2	well_2	F004	256	Z distribution	0.54773	0.513832	0.585599
DSP	W1	PGE2	well_2	F005	347	Z distribution	1.103784	1.036254	0.92884
DSP	W1	PGE2	well_2	F006	203	Z distribution	0.642948	0.61806	0.599291
DSP	W1	PGE2	well_3	F001	192	Z distribution	0.402835	0.243268	0.614527
DSP	W1	PGE2	well_3	F002	229	Z distribution	0.77914	0.764855	0.542306
DSP	W1	PGE2	well_3	F003	224	Z distribution	0.354654	0.271176	0.588944
DSP	W1	PGE2	well_3	F004	215	Z distribution	0.590913	0.466823	0.674187
DSP	W1	PGE2	well_3	F005	112	Z distribution	0.019281	-0.03169	0.464571
DSP	W1	PGE2	well_3	F006	261	Z distribution	0.80792	0.740661	0.732388
DSP	W1	PGE2	well_4	F001	266	Z distribution	0.328948	0.294031	0.475858
DSP	W1	PGE2	well_4	F002	227	Z distribution	0.49338	0.471023	0.52089
DSP	W1	PGE2	well_4	F003	296	Z distribution	0.766355	0.664511	0.791273
DSP	W1	PGE2	well_4	F004	364	Z distribution	0.93061	0.96151	0.902601
DSP	W1	PGE2	well_4	F005	366	Z distribution	0.918337	0.898124	0.747481
DSP	W1	PGE2	well_4	F006	218	Z distribution	0.40548	0.386155	0.466703
DSP	W1	PGE2	well_5	F001	112	Z distribution	-0.0198	-0.02867	0.376149
DSP	W1	PGE2	well_5	F002	127	Z distribution	0.377846	0.349913	0.474027
DSP	W1	PGE2	well_5	F003	233	Z distribution	0.389871	0.294015	0.602299

DSP	W1	PGE2	well_5	F004	203	Z distribution	0.418824	0.384631	0.580685
DSP	W1	PGE2	well_5	F005	230	Z distribution	0.517298	0.376788	0.704445
DSP	W1	PGE2	well_5	F006	276	Z distribution	0.693902	0.557419	0.759213
DSP	W2	HPI4	well_1	F001	235	Z distribution	0.216305	0.184469	0.52428
DSP	W2	HPI4	well_1	F002	179	Z distribution	-0.19147	-0.15249	0.456515
DSP	W2	HPI4	well_1	F003	363	Z distribution	-0.30582	-0.25201	0.519782
DSP	W2	HPI4	well_1	F004	200	Z distribution	-0.29859	-0.19977	0.513867
DSP	W2	HPI4	well_1	F005	286	Z distribution	-0.10852	-0.07132	0.571022
DSP	W2	HPI4	well_1	F006	115	Z distribution	-0.13254	-0.09147	0.466749
DSP	W2	HPI4	well_2	F001	147	Z distribution	0.056815	0.051031	0.456984
DSP	W2	HPI4	well_2	F002	177	Z distribution	-0.37918	-0.40517	0.441358
DSP	W2	HPI4	well_2	F003	329	Z distribution	-0.21565	-0.27823	0.571225
DSP	W2	HPI4	well_2	F004	272	Z distribution	-0.03367	-0.06234	0.435431
DSP	W2	HPI4	well_2	F005	185	Z distribution	-0.1786	-0.16348	0.417183
DSP	W2	HPI4	well_2	F006	275	Z distribution	0.252148	0.130326	0.648528
DSP	W2	HPI4	well_3	F001	144	Z distribution	0.044667	0.065784	0.375268
DSP	W2	HPI4	well_3	F002	183	Z distribution	-0.20605	-0.2237	0.391472
DSP	W2	HPI4	well_3	F003	55	Z distribution	-0.40007	-0.42949	0.44714
DSP	W2	HPI4	well_3	F004	288	Z distribution	-0.02874	-0.03004	0.364128
DSP	W2	HPI4	well_3	F005	289	Z distribution	0.466154	0.406704	0.571286
DSP	W2	HPI4	well_3	F006	277	Z distribution	0.809746	0.768236	0.650751
DSP	W2	HPI4	well_4	F001	222	Z distribution	0.333513	0.28944	0.492052
DSP	W2	HPI4	well_4	F002	154	Z distribution	0.034789	-0.02156	0.403676
DSP	W2	HPI4	well_4	F003	24	Z distribution	-0.05476	-0.05381	0.247164
DSP	W2	HPI4	well_4	F004	202	Z distribution	0.266194	0.199402	0.608068
DSP	W2	HPI4	well_4	F005	182	Z distribution	0.791682	0.784428	0.498274
DSP	W2	HPI4	well_4	F006	340	Z distribution	0.83014	0.847485	0.634748
DSP	W2	HPI4	well_5	F001	237	Z distribution	0.460173	0.3999	0.463905
DSP	W2	HPI4	well_5	F002	197	Z distribution	0.049407	-0.03468	0.458866
DSP	W2	HPI4	well_5	F003	84	Z distribution	-0.08282	-0.09527	0.294527
DSP	W2	HPI4	well_5	F004	149	Z distribution	-0.01854	0.007548	0.41527
DSP	W2	HPI4	well_5	F005	262	Z distribution	0.105387	0.1094	0.454161
DSP	W2	HPI4	well_5	F006	160	Z distribution	0.142432	0.191764	0.404548
DSP	W2	PGE2	well_1	F001	286	Z distribution	1.055678	1.034424	0.6725
DSP	W2	PGE2	well_1	F002	342	Z distribution	1.571657	1.674558	0.791788
DSP	W2	PGE2	well_1	F003	375	Z distribution	1.25819	1.2241	0.794869
DSP	W2	PGE2	well_1	F004	315	Z distribution	1.223095	1.290903	0.630117
DSP	W2	PGE2	well_1	F005	340	Z distribution	1.454638	1.44908	0.890629
DSP	W2	PGE2	well_1	F006	393	Z distribution	1.588761	1.612109	0.744358
DSP	W2	PGE2	well_2	F001	331	Z distribution	1.46852	1.524736	0.701885
DSP	W2	PGE2	well_2	F002	375	Z distribution	1.331517	1.381399	0.897921
DSP	W2	PGE2	well_2	F003	352	Z distribution	0.792648	0.932927	1.067814
DSP	W2	PGE2	well_2	F004	308	Z distribution	1.088008	1.120288	0.682115
DSP	W2	PGE2	well_2	F005	416	Z distribution	1.323329	1.353086	0.805091
DSP	W2	PGE2	well_2	F006	396	Z distribution	1.439064	1.430996	0.798797
DSP	W2	PGE2	well_3	F001	127	Z distribution	1.35041	1.335023	0.984284
DSP	W2	PGE2	well_3	F002	303	Z distribution	1.282855	1.373867	0.668287
DSP	W2	PGE2	well_3	F003	337	Z distribution	0.956253	1.00312	0.732225
DSP	W2	PGE2	well_3	F004	339	Z distribution	1.486626	1.561928	0.776468

DSP	W2	PGE2	well_3	F005	373	Z distribution	1.741738	1.83955	0.810584
DSP	W2	PGE2	well_3	F006	334	Z distribution	1.293962	1.427319	0.865319
DSP	W2	PGE2	well_4	F001	302	Z distribution	0.916806	1.011845	0.712872
DSP	W2	PGE2	well_4	F002	292	Z distribution	1.199058	1.246435	0.714396
DSP	W2	PGE2	well_4	F003	391	Z distribution	1.274624	1.365604	0.855686
DSP	W2	PGE2	well_4	F004	338	Z distribution	1.084183	1.135527	0.783584
DSP	W2	PGE2	well_4	F005	421	Z distribution	1.575682	1.599017	0.891848
DSP	W2	PGE2	well_4	F006	14	Z distribution	0.089515	0.055309	0.191099
DSP	W2	PGE2	well_5	F001	186	Z distribution	1.15024	1.292827	0.781102
DSP	W2	PGE2	well_5	F002	329	Z distribution	1.284361	1.262984	0.681381
DSP	W2	PGE2	well_5	F003	221	Z distribution	1.467444	1.480053	0.837109
DSP	W2	PGE2	well_5	F004	331	Z distribution	1.120435	1.147711	0.700738
DSP	W2	PGE2	well_5	F005	306	Z distribution	1.492794	1.612264	0.851679
DSP	W2	PGE2	well_5	F006	245	Z distribution	1.392691	1.499032	0.884036
DSP	W3	HPI4	well_1	F001	242	Z distribution	-0.02966	-0.04399	0.425596
DSP	W3	HPI4	well_1	F002	315	Z distribution	-0.05961	-0.07243	0.501359
DSP	W3	HPI4	well_1	F003	90	Z distribution	0.255219	0.263987	0.415389
DSP	W3	HPI4	well_1	F004	370	Z distribution	-0.19716	-0.21187	0.389174
DSP	W3	HPI4	well_1	F005	345	Z distribution	0.066045	0.041162	0.494867
DSP	W3	HPI4	well_1	F006	191	Z distribution	0.126683	0.106107	0.509063
DSP	W3	HPI4	well_2	F001	219	Z distribution	0.007092	0.06027	0.488957
DSP	W3	HPI4	well_2	F002	195	Z distribution	-0.21551	-0.21152	0.572558
DSP	W3	HPI4	well_2	F003	260	Z distribution	0.022703	0.052983	0.557998
DSP	W3	HPI4	well_2	F004	310	Z distribution	-0.07021	-0.07986	0.577577
DSP	W3	HPI4	well_2	F005	212	Z distribution	-0.18981	-0.19003	0.447446
DSP	W3	HPI4	well_2	F006	279	Z distribution	0.040022	-0.0172	0.566598
DSP	W3	HPI4	well_3	F001	244	Z distribution	-0.11218	-0.13606	0.465542
DSP	W3	HPI4	well_3	F002	205	Z distribution	-0.0029	0.046134	0.504749
DSP	W3	HPI4	well_3	F003	87	Z distribution	-0.32027	-0.27808	0.508183
DSP	W3	HPI4	well_3	F004	106	Z distribution	0.096019	0.086853	0.429276
DSP	W3	HPI4	well_3	F005	327	Z distribution	-0.15608	-0.15814	0.501971
DSP	W3	HPI4	well_3	F006	366	Z distribution	0.112427	0.057975	0.505578
DSP	W3	HPI4	well_4	F001	180	Z distribution	-0.19462	-0.13623	0.467707
DSP	W3	HPI4	well_4	F002	99	Z distribution	-0.16813	-0.12724	0.462986
DSP	W3	HPI4	well_4	F003	42	Z distribution	0.05093	-0.00288	0.364487
DSP	W3	HPI4	well_4	F004	91	Z distribution	0.16342	0.116	0.420141
DSP	W3	HPI4	well_4	F005	507	Z distribution	-0.12979	-0.14827	0.561887
DSP	W3	HPI4	well_4	F006	305	Z distribution	0.098118	0.116888	0.50879
DSP	W3	HPI4	well_5	F001	152	Z distribution	-0.10817	-0.06863	0.477845
DSP	W3	HPI4	well_5	F002	59	Z distribution	-0.00029	0.049678	0.357539
DSP	W3	HPI4	well_5	F003	3	Z distribution	0.010285	0.010285	NA
DSP	W3	HPI4	well_5	F004	29	Z distribution	-0.0088	0.045882	0.28403
DSP	W3	HPI4	well_5	F005	205	Z distribution	-0.1476	-0.09291	0.518558
DSP	W3	HPI4	well_5	F006	292	Z distribution	0.224308	0.227866	0.566461
DSP	W3	PGE2	well_1	F001	382	Z distribution	0.754481	0.746619	0.673927
DSP	W3	PGE2	well_1	F002	355	Z distribution	0.36355	0.416831	0.736032
DSP	W3	PGE2	well_1	F003	336	Z distribution	0.892849	0.910531	0.70276
DSP	W3	PGE2	well_1	F004	209	Z distribution	0.482821	0.443801	0.749542
DSP	W3	PGE2	well_1	F005	332	Z distribution	0.007849	-0.02951	0.501113

DSP	W3	PGE2	well_1	F006	167	Z distribution	0.175018	0.18539	0.370253
DSP	W3	PGE2	well_2	F001	308	Z distribution	1.200562	1.156002	0.997448
DSP	W3	PGE2	well_2	F002	328	Z distribution	-0.09757	-0.20731	0.707063
DSP	W3	PGE2	well_2	F003	194	Z distribution	0.989669	0.868585	0.765923
DSP	W3	PGE2	well_2	F004	384	Z distribution	0.93202	0.955051	0.822888
DSP	W3	PGE2	well_2	F005	348	Z distribution	-0.09656	-0.17359	0.76812
DSP	W3	PGE2	well_2	F006	217	Z distribution	0.359779	0.204287	0.943575
DSP	W3	PGE2	well_3	F001	345	Z distribution	0.594448	0.521555	0.810117
DSP	W3	PGE2	well_3	F002	72	Z distribution	0.201733	0.058421	0.568065
DSP	W3	PGE2	well_3	F003	339	Z distribution	0.830185	0.691689	0.821946
DSP	W3	PGE2	well_3	F004	371	Z distribution	1.683852	1.644676	0.800674
DSP	W3	PGE2	well_3	F005	291	Z distribution	0.423481	0.377086	0.765485
DSP	W3	PGE2	well_3	F006	358	Z distribution	-0.12664	-0.09959	0.616949
DSP	W3	PGE2	well_4	F001	309	Z distribution	0.21962	0.169886	0.654762
DSP	W3	PGE2	well_4	F002	416	Z distribution	1.482653	1.585923	0.790893
DSP	W3	PGE2	well_4	F003	236	Z distribution	0.764576	0.744469	0.690946
DSP	W3	PGE2	well_4	F004	315	Z distribution	0.37082	0.257608	0.679007
DSP	W3	PGE2	well_4	F005	361	Z distribution	0.905235	0.922807	0.764541
DSP	W3	PGE2	well_4	F006	234	Z distribution	-0.38078	-0.45384	0.668011
DSP	W3	PGE2	well_5	F001	136	Z distribution	1.137079	1.277607	0.699775
DSP	W3	PGE2	well_5	F002	246	Z distribution	0.193703	0.028415	0.962041
DSP	W3	PGE2	well_5	F003	436	Z distribution	0.944897	0.930099	0.935011
DSP	W3	PGE2	well_5	F004	265	Z distribution	1.061581	1.057417	0.959937
DSP	W3	PGE2	well_5	F005	174	Z distribution	-0.02529	-0.04299	0.555829
DSP	W3	PGE2	well_5	F006	327	Z distribution	0.635541	0.570486	0.679213
DSP	W4	HPI4	well_1	F001	42	Z distribution	-0.18643	-0.12755	0.338324
DSP	W4	HPI4	well_1	F002	282	Z distribution	0.095809	0.119915	0.459212
DSP	W4	HPI4	well_1	F003	219	Z distribution	-0.36771	-0.43491	0.436364
DSP	W4	HPI4	well_1	F004	188	Z distribution	-0.04525	-0.04144	0.428225
DSP	W4	HPI4	well_1	F005	292	Z distribution	-0.17472	-0.21692	0.475513
DSP	W4	HPI4	well_1	F006	197	Z distribution	0.058349	0.077417	0.464838
DSP	W4	HPI4	well_2	F001	47	Z distribution	0.07633	0.093881	0.367015
DSP	W4	HPI4	well_2	F002	250	Z distribution	0.177081	0.247651	0.438071
DSP	W4	HPI4	well_2	F003	192	Z distribution	-0.09379	-0.13669	0.514229
DSP	W4	HPI4	well_2	F004	204	Z distribution	-0.1805	-0.14345	0.413692
DSP	W4	HPI4	well_2	F005	227	Z distribution	-0.0472	-0.07182	0.498099
DSP	W4	HPI4	well_2	F006	198	Z distribution	0.371171	0.368399	0.40492
DSP	W4	HPI4	well_3	F001	139	Z distribution	0.350652	0.375509	0.356205
DSP	W4	HPI4	well_3	F002	147	Z distribution	0.021301	-0.02921	0.496167
DSP	W4	HPI4	well_3	F003	97	Z distribution	-0.15618	-0.05835	0.364197
DSP	W4	HPI4	well_3	F004	160	Z distribution	0.028989	0.045958	0.410409
DSP	W4	HPI4	well_3	F005	258	Z distribution	0.132617	0.142021	0.434433
DSP	W4	HPI4	well_3	F006	169	Z distribution	0.052803	0.11698	0.417029
DSP	W4	HPI4	well_4	F001	49	Z distribution	-0.0654	-0.07167	0.384421
DSP	W4	HPI4	well_4	F002	170	Z distribution	-0.30022	-0.36717	0.480423
DSP	W4	HPI4	well_4	F003	84	Z distribution	-0.21751	-0.22329	0.459222
DSP	W4	HPI4	well_4	F004	138	Z distribution	-0.28883	-0.22116	0.53426
DSP	W4	HPI4	well_4	F005	248	Z distribution	-0.0691	-0.05388	0.444117
DSP	W4	HPI4	well_4	F006	128	Z distribution	-0.23374	-0.224	0.479157

DSP	W4	HPI4	well_5	F001	24	Z distribution	0.001025	0.090909	0.327473
DSP	W4	HPI4	well_5	F002	42	Z distribution	-0.64087	-0.72808	0.401335
DSP	W4	HPI4	well_5	F003	168	Z distribution	0.162655	0.22037	0.470826
DSP	W4	HPI4	well_5	F004	41	Z distribution	-0.4137	-0.31572	0.421116
DSP	W4	HPI4	well_5	F005	86	Z distribution	-0.33119	-0.31439	0.402174
DSP	W4	HPI4	well_5	F006	105	Z distribution	0.191453	0.184386	0.359472
DSP	W4	PGE2	well_1	F001	330	Z distribution	0.443155	0.323706	0.760421
DSP	W4	PGE2	well_1	F002	157	Z distribution	-0.09322	-0.1169	0.536891
DSP	W4	PGE2	well_1	F003	217	Z distribution	0.425294	0.314677	0.715878
DSP	W4	PGE2	well_1	F004	306	Z distribution	-0.58491	-0.64356	0.552117
DSP	W4	PGE2	well_1	F005	236	Z distribution	-0.37572	-0.45905	0.577243
DSP	W4	PGE2	well_1	F006	281	Z distribution	0.473143	0.353258	0.868792
DSP	W4	PGE2	well_2	F001	129	Z distribution	-0.32376	-0.43255	0.491638
DSP	W4	PGE2	well_2	F002	366	Z distribution	-0.00601	-0.14811	0.619238
DSP	W4	PGE2	well_2	F003	348	Z distribution	-0.4288	-0.53705	0.607676
DSP	W4	PGE2	well_2	F004	256	Z distribution	0.411812	0.290961	0.734207
DSP	W4	PGE2	well_2	F005	277	Z distribution	-0.40974	-0.46671	0.493529
DSP	W4	PGE2	well_2	F006	330	Z distribution	0.241643	0.218288	0.708164
DSP	W4	PGE2	well_3	F001	203	Z distribution	-0.25963	-0.29576	0.432408
DSP	W4	PGE2	well_3	F002	169	Z distribution	0.223434	0.060127	0.779569
DSP	W4	PGE2	well_3	F003	277	Z distribution	-0.38001	-0.40892	0.490706
DSP	W4	PGE2	well_3	F004	335	Z distribution	-0.01129	-0.20414	0.719704
DSP	W4	PGE2	well_3	F005	288	Z distribution	-0.29075	-0.26932	0.406993
DSP	W4	PGE2	well_3	F006	318	Z distribution	-0.58197	-0.62543	0.437613
DSP	W4	PGE2	well_4	F001	103	Z distribution	-0.17576	-0.23429	0.439025
DSP	W4	PGE2	well_4	F002	239	Z distribution	0.10834	-0.14798	1.043399
DSP	W4	PGE2	well_4	F003	373	Z distribution	-0.05875	-0.1352	0.648214
DSP	W4	PGE2	well_4	F004	221	Z distribution	1.117555	1.178532	1.020415
DSP	W4	PGE2	well_4	F005	259	Z distribution	-0.16127	-0.20065	0.43895
DSP	W4	PGE2	well_4	F006	202	Z distribution	-0.18598	-0.20227	0.532264
DSP	W4	PGE2	well_5	F001	81	Z distribution	0.264468	0.218187	0.688514
DSP	W4	PGE2	well_5	F002	215	Z distribution	-0.08715	-0.17203	0.621219
DSP	W4	PGE2	well_5	F003	304	Z distribution	0.357393	0.06848	1.004727
DSP	W4	PGE2	well_5	F004	187	Z distribution	0.371792	0.25389	0.795577
DSP	W4	PGE2	well_5	F005	321	Z distribution	0.659061	0.713548	0.63477
DSP	W4	PGE2	well_5	F006	263	Z distribution	1.048079	1.082061	0.747676
GJA1	W1	HPI4	well_1	F001	9	Avg. Volume	1.147423	0.810384	0.865959
GJA1	W1	HPI4	well_1	F002	13	Avg. Volume	0.430226	0.365184	0.14347
GJA1	W1	HPI4	well_1	F003	4	Avg. Volume	0.382386	0.382386	0.099409
GJA1	W1	HPI4	well_1	F004	15	Avg. Volume	0.861637	0.783545	0.440518
GJA1	W1	HPI4	well_1	F005	1	Avg. Volume	0.404058	0.404058	NA
GJA1	W1	HPI4	well_1	F006	4	Avg. Volume	0.287535	0.287535	0.19087
GJA1	W1	HPI4	well_2	F001	3	Avg. Volume	0.547685	0.547685	NA
GJA1	W1	HPI4	well_2	F002	6	Avg. Volume	0.282086	0.261715	0.05889
GJA1	W1	HPI4	well_2	F003	6	Avg. Volume	0.276349	0.271199	0.060118
GJA1	W1	HPI4	well_2	F004	7	Avg. Volume	0.534944	0.490309	0.342084
GJA1	W1	HPI4	well_2	F005	5	Avg. Volume	0.290883	0.279814	0.098934
GJA1	W1	HPI4	well_2	F006	4	Avg. Volume	0.379698	0.379698	0.193961
GJA1	W1	HPI4	well_3	F002	7	Avg. Volume	0.384271	0.293738	0.273842

GJA1	W1	HPI4	well_3	F003	3	Avg. Volume	0.41272	0.41272	NA
GJA1	W1	HPI4	well_3	F004	1	Avg. Volume	0.099757	0.099757	NA
GJA1	W1	HPI4	well_3	F005	7	Avg. Volume	0.432023	0.375556	0.238055
GJA1	W1	HPI4	well_3	F006	7	Avg. Volume	0.305791	0.289831	0.107285
GJA1	W1	HPI4	well_4	F001	19	Avg. Volume	0.456017	0.396894	0.178012
GJA1	W1	HPI4	well_4	F002	1	Avg. Volume	1.425641	1.425641	NA
GJA1	W1	HPI4	well_4	F003	14	Avg. Volume	0.467472	0.472984	0.18075
GJA1	W1	HPI4	well_4	F004	11	Avg. Volume	0.516415	0.505556	0.19327
GJA1	W1	HPI4	well_4	F006	4	Avg. Volume	0.33013	0.33013	0.050964
GJA1	W1	HPI4	well_5	F001	3	Avg. Volume	0.144187	0.144187	NA
GJA1	W1	HPI4	well_5	F002	66	Avg. Volume	0.339669	0.32917	0.151989
GJA1	W1	HPI4	well_5	F003	6	Avg. Volume	0.607215	0.604475	0.125609
GJA1	W1	HPI4	well_5	F004	7	Avg. Volume	0.641995	0.596471	0.284816
GJA1	W1	HPI4	well_5	F005	29	Avg. Volume	0.476022	0.406852	0.258464
GJA1	W1	HPI4	well_5	F006	5	Avg. Volume	0.631121	0.736581	0.192481
GJA1	W1	PGE2	well_1	F002	1	Avg. Volume	0.596586	0.596586	NA
GJA1	W1	PGE2	well_1	F003	11	Avg. Volume	0.740774	0.778006	0.48245
GJA1	W1	PGE2	well_1	F004	4	Avg. Volume	0.678137	0.678137	0.171469
GJA1	W1	PGE2	well_1	F005	4	Avg. Volume	0.775357	0.775357	0.345013
GJA1	W1	PGE2	well_1	F006	22	Avg. Volume	0.542865	0.514706	0.288722
GJA1	W1	PGE2	well_2	F001	1	Avg. Volume	0.323917	0.323917	NA
GJA1	W1	PGE2	well_2	F002	1	Avg. Volume	0.183083	0.183083	NA
GJA1	W1	PGE2	well_2	F003	1	Avg. Volume	0.793026	0.793026	NA
GJA1	W1	PGE2	well_2	F004	22	Avg. Volume	0.395257	0.366012	0.159105
GJA1	W1	PGE2	well_2	F005	21	Avg. Volume	0.508759	0.459385	0.201974
GJA1	W1	PGE2	well_2	F006	4	Avg. Volume	0.689879	0.689879	0.012989
GJA1	W1	PGE2	well_3	F002	5	Avg. Volume	0.383435	0.410146	0.046802
GJA1	W1	PGE2	well_3	F004	7	Avg. Volume	0.400173	0.385615	0.050974
GJA1	W1	PGE2	well_3	F005	50	Avg. Volume	0.441994	0.424065	0.226606
GJA1	W1	PGE2	well_3	F006	4	Avg. Volume	0.277168	0.277168	0.047856
GJA1	W1	PGE2	well_4	F001	4	Avg. Volume	0.26532	0.26532	0.064611
GJA1	W1	PGE2	well_4	F002	3	Avg. Volume	6.891271	6.891271	NA
GJA1	W1	PGE2	well_4	F003	13	Avg. Volume	0.333298	0.294811	0.122092
GJA1	W1	PGE2	well_4	F004	4	Avg. Volume	0.615893	0.615893	0.236616
GJA1	W1	PGE2	well_4	F006	1	Avg. Volume	0.543739	0.543739	NA
GJA1	W1	PGE2	well_5	F001	10	Avg. Volume	0.825617	0.84202	0.403873
GJA1	W1	PGE2	well_5	F002	7	Avg. Volume	0.531079	0.454442	0.247528
GJA1	W1	PGE2	well_5	F003	5	Avg. Volume	0.700714	0.68843	0.406436
GJA1	W1	PGE2	well_5	F004	1	Avg. Volume	0.824136	0.824136	NA
GJA1	W1	PGE2	well_5	F005	23	Avg. Volume	0.598891	0.41288	0.319122
GJA1	W1	PGE2	well_5	F006	16	Avg. Volume	0.570395	0.486915	0.38633
GJA1	W2	HPI4	well_1	F001	1	Avg. Volume	0.582405	0.582405	NA
GJA1	W2	HPI4	well_1	F002	3	Avg. Volume	0.342694	0.342694	NA
GJA1	W2	HPI4	well_2	F003	1	Avg. Volume	0.238243	0.238243	NA
GJA1	W2	HPI4	well_2	F004	58	Avg. Volume	0.340151	0.298026	0.16985
GJA1	W2	HPI4	well_2	F005	1	Avg. Volume	0.48067	0.48067	NA
GJA1	W2	HPI4	well_2	F006	8	Avg. Volume	0.581112	0.598848	0.169445
GJA1	W2	HPI4	well_3	F001	15	Avg. Volume	0.544015	0.478833	0.448852
GJA1	W2	HPI4	well_3	F002	13	Avg. Volume	0.470776	0.430324	0.205368

GJA1	W2	HPI4	well_3	F004	42	Avg. Volume	0.361006	0.342549	0.174015
GJA1	W2	HPI4	well_3	F005	18	Avg. Volume	0.533552	0.575069	0.31505
GJA1	W2	HPI4	well_3	F006	12	Avg. Volume	0.40386	0.418309	0.27215
GJA1	W2	HPI4	well_4	F001	41	Avg. Volume	0.815809	0.712331	0.453325
GJA1	W2	HPI4	well_4	F002	11	Avg. Volume	0.440329	0.48936	0.149537
GJA1	W2	HPI4	well_4	F003	4	Avg. Volume	0.355732	0.355732	0.009066
GJA1	W2	HPI4	well_4	F004	43	Avg. Volume	0.293748	0.305139	0.170789
GJA1	W2	HPI4	well_4	F005	25	Avg. Volume	0.451496	0.430072	0.177244
GJA1	W2	HPI4	well_4	F006	18	Avg. Volume	0.559256	0.46678	0.317169
GJA1	W2	HPI4	well_5	F001	12	Avg. Volume	0.478849	0.401538	0.226827
GJA1	W2	HPI4	well_5	F002	23	Avg. Volume	0.630941	0.610278	0.217943
GJA1	W2	HPI4	well_5	F003	1	Avg. Volume	2.689525	2.689525	NA
GJA1	W2	HPI4	well_5	F004	47	Avg. Volume	0.393886	0.362515	0.17178
GJA1	W2	HPI4	well_5	F005	28	Avg. Volume	0.586773	0.595345	0.255172
GJA1	W2	HPI4	well_5	F006	20	Avg. Volume	0.523842	0.576156	0.180425
GJA1	W2	PGE2	well_1	F001	102	Avg. Volume	0.521732	0.482284	0.213383
GJA1	W2	PGE2	well_1	F002	48	Avg. Volume	0.603423	0.547819	0.232128
GJA1	W2	PGE2	well_1	F003	76	Avg. Volume	0.560737	0.44206	0.374286
GJA1	W2	PGE2	well_1	F004	23	Avg. Volume	0.478869	0.43256	0.208352
GJA1	W2	PGE2	well_1	F005	189	Avg. Volume	0.508559	0.521083	0.215211
GJA1	W2	PGE2	well_1	F006	101	Avg. Volume	0.674211	0.646269	0.251177
GJA1	W2	PGE2	well_2	F001	14	Avg. Volume	0.559802	0.561347	0.297982
GJA1	W2	PGE2	well_2	F002	21	Avg. Volume	0.491012	0.458326	0.177175
GJA1	W2	PGE2	well_2	F003	25	Avg. Volume	0.587079	0.514824	0.355243
GJA1	W2	PGE2	well_2	F004	20	Avg. Volume	0.384872	0.281667	0.288561
GJA1	W2	PGE2	well_2	F005	50	Avg. Volume	0.523023	0.505044	0.197675
GJA1	W2	PGE2	well_2	F006	12	Avg. Volume	0.598432	0.533993	0.168627
GJA1	W2	PGE2	well_3	F001	28	Avg. Volume	0.535674	0.452941	0.277801
GJA1	W2	PGE2	well_3	F002	13	Avg. Volume	0.415606	0.463043	0.133187
GJA1	W2	PGE2	well_3	F003	25	Avg. Volume	0.549195	0.609211	0.224245
GJA1	W2	PGE2	well_3	F004	47	Avg. Volume	0.75921	0.735463	0.223313
GJA1	W2	PGE2	well_3	F005	85	Avg. Volume	0.51188	0.516389	0.211638
GJA1	W2	PGE2	well_3	F006	24	Avg. Volume	0.467524	0.436868	0.20358
GJA1	W2	PGE2	well_4	F001	6	Avg. Volume	0.369255	0.368201	0.055789
GJA1	W2	PGE2	well_4	F002	25	Avg. Volume	0.573997	0.508565	0.272406
GJA1	W2	PGE2	well_4	F003	50	Avg. Volume	0.634311	0.595782	0.212071
GJA1	W2	PGE2	well_4	F004	7	Avg. Volume	0.233875	0.21125	0.079064
GJA1	W2	PGE2	well_4	F005	24	Avg. Volume	0.473457	0.469609	0.164469
GJA1	W2	PGE2	well_4	F006	40	Avg. Volume	0.571622	0.522536	0.254537
GJA1	W2	PGE2	well_5	F001	59	Avg. Volume	0.586621	0.559066	0.345838
GJA1	W2	PGE2	well_5	F002	18	Avg. Volume	0.558579	0.567106	0.209596
GJA1	W2	PGE2	well_5	F003	19	Avg. Volume	0.606964	0.640456	0.18426
GJA1	W2	PGE2	well_5	F004	6	Avg. Volume	0.674955	0.693291	0.149222
GJA1	W2	PGE2	well_5	F005	18	Avg. Volume	0.506253	0.51915	0.252653
GJA1	W2	PGE2	well_5	F006	18	Avg. Volume	0.629551	0.593847	0.269675
GJA1	W3	HPI4	well_1	F001	1	Avg. Volume	0.446706	0.446706	NA
GJA1	W3	HPI4	well_1	F003	4	Avg. Volume	0.748764	0.748764	0.335267
GJA1	W3	HPI4	well_1	F005	6	Avg. Volume	0.544947	0.544947	0.059773
GJA1	W3	HPI4	well_1	F006	5	Avg. Volume	0.632372	0.662905	0.238752

GJA1	W3	HPI4	well_2	F001	5	Avg. Volume	0.421851	0.268979	0.316098
GJA1	W3	HPI4	well_2	F002	4	Avg. Volume	0.376667	0.376667	0.074439
GJA1	W3	HPI4	well_2	F004	11	Avg. Volume	0.657895	0.370453	0.440082
GJA1	W3	HPI4	well_2	F005	3	Avg. Volume	0.368849	0.368849	NA
GJA1	W3	HPI4	well_2	F006	8	Avg. Volume	0.678011	0.746338	0.331687
GJA1	W3	HPI4	well_3	F001	4	Avg. Volume	0.549711	0.549711	0.291936
GJA1	W3	HPI4	well_3	F002	8	Avg. Volume	0.3637	0.379738	0.119723
GJA1	W3	HPI4	well_3	F003	4	Avg. Volume	0.835936	0.835936	0.067492
GJA1	W3	HPI4	well_3	F004	4	Avg. Volume	0.409259	0.409259	0.235768
GJA1	W3	HPI4	well_3	F005	9	Avg. Volume	0.357782	0.244111	0.304118
GJA1	W3	HPI4	well_3	F006	1	Avg. Volume	0.483528	0.483528	NA
GJA1	W3	HPI4	well_4	F001	4	Avg. Volume	0.443527	0.443527	0.086445
GJA1	W3	HPI4	well_4	F002	1	Avg. Volume	0.17213	0.17213	NA
GJA1	W3	HPI4	well_4	F004	4	Avg. Volume	0.176042	0.176042	0.041493
GJA1	W3	HPI4	well_4	F005	1	Avg. Volume	0.772155	0.772155	NA
GJA1	W3	HPI4	well_4	F006	4	Avg. Volume	1.502987	1.502987	0.576665
GJA1	W3	HPI4	well_5	F001	4	Avg. Volume	0.447682	0.447682	0.109116
GJA1	W3	HPI4	well_5	F002	19	Avg. Volume	0.516454	0.474139	0.254044
GJA1	W3	HPI4	well_5	F004	16	Avg. Volume	0.431571	0.442189	0.125239
GJA1	W3	HPI4	well_5	F005	14	Avg. Volume	0.879957	0.93933	0.369289
GJA1	W3	HPI4	well_5	F006	16	Avg. Volume	0.456217	0.358204	0.422329
GJA1	W3	PGE2	well_1	F001	14	Avg. Volume	0.435515	0.44709	0.141768
GJA1	W3	PGE2	well_1	F002	198	Avg. Volume	0.43497	0.352083	0.327911
GJA1	W3	PGE2	well_1	F003	188	Avg. Volume	0.471415	0.459437	0.175224
GJA1	W3	PGE2	well_1	F004	100	Avg. Volume	0.474909	0.42819	0.259297
GJA1	W3	PGE2	well_1	F005	99	Avg. Volume	0.392917	0.328611	0.296502
GJA1	W3	PGE2	well_1	F006	321	Avg. Volume	0.730152	0.725833	0.223975
GJA1	W3	PGE2	well_2	F001	189	Avg. Volume	0.677143	0.683866	0.216874
GJA1	W3	PGE2	well_2	F002	201	Avg. Volume	0.450066	0.399028	0.286113
GJA1	W3	PGE2	well_2	F003	252	Avg. Volume	0.446798	0.420544	0.183315
GJA1	W3	PGE2	well_2	F004	273	Avg. Volume	0.630032	0.590159	0.305092
GJA1	W3	PGE2	well_2	F005	270	Avg. Volume	0.57497	0.512575	0.307712
GJA1	W3	PGE2	well_2	F006	199	Avg. Volume	0.651424	0.660978	0.227828
GJA1	W3	PGE2	well_3	F001	151	Avg. Volume	0.598475	0.523095	0.36413
GJA1	W3	PGE2	well_3	F002	177	Avg. Volume	0.348639	0.234722	0.32774
GJA1	W3	PGE2	well_3	F003	95	Avg. Volume	0.232932	0.164306	0.22874
GJA1	W3	PGE2	well_3	F004	184	Avg. Volume	0.296941	0.264649	0.193296
GJA1	W3	PGE2	well_3	F005	111	Avg. Volume	0.343951	0.281667	0.315095
GJA1	W3	PGE2	well_3	F006	164	Avg. Volume	0.795906	0.752892	0.321554
GJA1	W3	PGE2	well_4	F001	190	Avg. Volume	0.615721	0.556752	0.303787
GJA1	W3	PGE2	well_4	F002	170	Avg. Volume	0.761868	0.680694	0.355166
GJA1	W3	PGE2	well_4	F003	89	Avg. Volume	0.343341	0.326935	0.195521
GJA1	W3	PGE2	well_4	F004	179	Avg. Volume	0.268627	0.21125	0.18365
GJA1	W3	PGE2	well_4	F005	304	Avg. Volume	0.781543	0.710035	0.395629
GJA1	W3	PGE2	well_4	F006	248	Avg. Volume	0.439971	0.413024	0.176331
GJA1	W3	PGE2	well_5	F001	198	Avg. Volume	0.567889	0.537607	0.240307
GJA1	W3	PGE2	well_5	F002	208	Avg. Volume	0.371719	0.336994	0.159692
GJA1	W3	PGE2	well_5	F003	150	Avg. Volume	0.546337	0.534811	0.30567
GJA1	W3	PGE2	well_5	F004	70	Avg. Volume	0.257509	0.224942	0.144833

GJA1	W3	PGE2	well_5	F005	97	Avg. Volume	0.392586	0.317657	0.311394
GJA1	W3	PGE2	well_5	F006	111	Avg. Volume	0.5289	0.537433	0.252724
GJA1	W4	HPI4	well_1	F001	7	Avg. Volume	0.685429	0.601128	0.360672
GJA1	W4	HPI4	well_1	F002	16	Avg. Volume	0.672163	0.675222	0.174626
GJA1	W4	HPI4	well_1	F003	37	Avg. Volume	0.637352	0.591349	0.229108
GJA1	W4	HPI4	well_1	F004	28	Avg. Volume	0.543496	0.523079	0.189884
GJA1	W4	HPI4	well_1	F005	8	Avg. Volume	0.699802	0.679668	0.10997
GJA1	W4	HPI4	well_1	F006	17	Avg. Volume	0.471107	0.507587	0.266205
GJA1	W4	HPI4	well_2	F001	9	Avg. Volume	0.337076	0.364025	0.082008
GJA1	W4	HPI4	well_2	F002	12	Avg. Volume	0.635182	0.604902	0.29785
GJA1	W4	HPI4	well_2	F003	24	Avg. Volume	0.56237	0.571157	0.198459
GJA1	W4	HPI4	well_2	F004	44	Avg. Volume	0.375501	0.389136	0.215399
GJA1	W4	HPI4	well_2	F005	15	Avg. Volume	0.740469	0.656635	0.199994
GJA1	W4	HPI4	well_2	F006	3	Avg. Volume	0.547083	0.547083	NA
GJA1	W4	HPI4	well_3	F001	9	Avg. Volume	0.350078	0.379747	0.123139
GJA1	W4	HPI4	well_3	F002	8	Avg. Volume	0.615158	0.558304	0.189655
GJA1	W4	HPI4	well_3	F003	15	Avg. Volume	0.407609	0.410764	0.174518
GJA1	W4	HPI4	well_3	F004	13	Avg. Volume	0.471725	0.510521	0.253436
GJA1	W4	HPI4	well_3	F005	5	Avg. Volume	0.655663	0.695784	0.189143
GJA1	W4	HPI4	well_3	F006	21	Avg. Volume	0.445326	0.352083	0.225803
GJA1	W4	HPI4	well_4	F002	25	Avg. Volume	0.401744	0.411142	0.191488
GJA1	W4	HPI4	well_4	F003	8	Avg. Volume	0.744798	0.704517	0.162664
GJA1	W4	HPI4	well_4	F004	25	Avg. Volume	0.773066	0.638966	0.440036
GJA1	W4	HPI4	well_4	F005	70	Avg. Volume	0.55081	0.495199	0.215067
GJA1	W4	HPI4	well_4	F006	50	Avg. Volume	0.500107	0.482108	0.200812
GJA1	W4	HPI4	well_5	F001	11	Avg. Volume	0.866352	0.699472	0.600189
GJA1	W4	HPI4	well_5	F002	15	Avg. Volume	0.441088	0.4225	0.237611
GJA1	W4	HPI4	well_5	F003	17	Avg. Volume	0.644519	0.51678	0.361416
GJA1	W4	HPI4	well_5	F004	29	Avg. Volume	0.49116	0.425588	0.189296
GJA1	W4	HPI4	well_5	F005	91	Avg. Volume	0.774822	0.745243	0.30519
GJA1	W4	HPI4	well_5	F006	10	Avg. Volume	0.398863	0.272422	0.316816
GJA1	W4	PGE2	well_1	F001	43	Avg. Volume	0.799316	0.727639	0.348342
GJA1	W4	PGE2	well_1	F002	113	Avg. Volume	0.37643	0.305139	0.261914
GJA1	W4	PGE2	well_1	F003	306	Avg. Volume	0.665604	0.532037	0.516112
GJA1	W4	PGE2	well_1	F004	34	Avg. Volume	0.494361	0.444101	0.241704
GJA1	W4	PGE2	well_1	F005	55	Avg. Volume	0.363472	0.340347	0.208474
GJA1	W4	PGE2	well_1	F006	329	Avg. Volume	0.637705	0.577677	0.40875
GJA1	W4	PGE2	well_2	F001	200	Avg. Volume	0.812586	0.733692	0.547445
GJA1	W4	PGE2	well_2	F002	159	Avg. Volume	0.47239	0.44206	0.204944
GJA1	W4	PGE2	well_2	F003	277	Avg. Volume	0.323437	0.301618	0.173378
GJA1	W4	PGE2	well_2	F004	252	Avg. Volume	0.698483	0.629447	0.396588
GJA1	W4	PGE2	well_2	F005	168	Avg. Volume	0.76229	0.555509	0.672766
GJA1	W4	PGE2	well_2	F006	175	Avg. Volume	0.540092	0.532037	0.238668
GJA1	W4	PGE2	well_3	F001	85	Avg. Volume	0.616704	0.597924	0.24298
GJA1	W4	PGE2	well_3	F002	306	Avg. Volume	0.233076	0.214603	0.125322
GJA1	W4	PGE2	well_3	F003	220	Avg. Volume	0.321712	0.284917	0.173558
GJA1	W4	PGE2	well_3	F004	40	Avg. Volume	0.35966	0.264063	0.28559
GJA1	W4	PGE2	well_3	F005	19	Avg. Volume	0.318092	0.251153	0.281622
GJA1	W4	PGE2	well_3	F006	144	Avg. Volume	0.394757	0.362515	0.209465

GJA1	W4	PGE2	well_4	F001	100	Avg. Volume	0.421084	0.350127	0.302143
GJA1	W4	PGE2	well_4	F002	72	Avg. Volume	0.359012	0.271887	0.329061
GJA1	W4	PGE2	well_4	F003	154	Avg. Volume	0.413509	0.339043	0.268667
GJA1	W4	PGE2	well_4	F004	292	Avg. Volume	0.632912	0.547152	0.362058
GJA1	W4	PGE2	well_4	F005	212	Avg. Volume	0.443488	0.402536	0.227821
GJA1	W4	PGE2	well_4	F006	60	Avg. Volume	0.880329	0.80588	0.708218
GJA1	W4	PGE2	well_5	F001	197	Avg. Volume	0.336987	0.305139	0.187813
GJA1	W4	PGE2	well_5	F002	177	Avg. Volume	0.59442	0.538557	0.298096
GJA1	W4	PGE2	well_5	F003	124	Avg. Volume	0.33022	0.281667	0.212532
GJA1	W4	PGE2	well_5	F004	124	Avg. Volume	0.671815	0.668769	0.280784
GJA1	W4	PGE2	well_5	F005	91	Avg. Volume	0.324886	0.281667	0.187024
GJA1	W4	PGE2	well_5	F006	97	Avg. Volume	0.43098	0.4225	0.195693
GJA1	W1	HPI4	well_1	F001	9	Count/cell	60	63	25.33114
GJA1	W1	HPI4	well_1	F002	13	Count/cell	32	30	14.90637
GJA1	W1	HPI4	well_1	F003	4	Count/cell	26	26	16.97056
GJA1	W1	HPI4	well_1	F004	15	Count/cell	43.53846	44	18.97637
GJA1	W1	HPI4	well_1	F005	1	Count/cell	14	14	NA
GJA1	W1	HPI4	well_1	F006	4	Count/cell	15	15	0
GJA1	W1	HPI4	well_2	F001	3	Count/cell	2	2	NA
GJA1	W1	HPI4	well_2	F002	6	Count/cell	7	6.5	2.160247
GJA1	W1	HPI4	well_2	F003	6	Count/cell	15	13.5	12.35584
GJA1	W1	HPI4	well_2	F004	7	Count/cell	7.166667	6.5	4.535049
GJA1	W1	HPI4	well_2	F005	5	Count/cell	26	30	7.81025
GJA1	W1	HPI4	well_2	F006	4	Count/cell	43	51	13.85641
GJA1	W1	HPI4	well_3	F002	7	Count/cell	23.4	21	10.52616
GJA1	W1	HPI4	well_3	F003	3	Count/cell	12	12	NA
GJA1	W1	HPI4	well_3	F004	1	Count/cell	4	4	NA
GJA1	W1	HPI4	well_3	F005	7	Count/cell	29	26	15.45962
GJA1	W1	HPI4	well_3	F006	7	Count/cell	15.2	13	7.42967
GJA1	W1	HPI4	well_4	F001	19	Count/cell	24.11765	26	12.55927
GJA1	W1	HPI4	well_4	F002	1	Count/cell	99	99	NA
GJA1	W1	HPI4	well_4	F003	14	Count/cell	38.83333	31	23.5713
GJA1	W1	HPI4	well_4	F004	11	Count/cell	31.77778	22	21.04031
GJA1	W1	HPI4	well_4	F006	4	Count/cell	14	14	4.242641
GJA1	W1	HPI4	well_5	F001	3	Count/cell	6	6	NA
GJA1	W1	HPI4	well_5	F002	66	Count/cell	19.22034	16	11.22508
GJA1	W1	HPI4	well_5	F003	6	Count/cell	37.75	36.5	5.85235
GJA1	W1	HPI4	well_5	F004	7	Count/cell	23.8	23	9.176056
GJA1	W1	HPI4	well_5	F005	29	Count/cell	20.84	19	11.19405
GJA1	W1	HPI4	well_5	F006	5	Count/cell	24	25	2.645751
GJA1	W1	PGE2	well_1	F002	1	Count/cell	12	12	NA
GJA1	W1	PGE2	well_1	F003	11	Count/cell	33.44444	34	8.427798
GJA1	W1	PGE2	well_1	F004	4	Count/cell	23.66667	10	23.67136
GJA1	W1	PGE2	well_1	F005	4	Count/cell	115	115	9.899495
GJA1	W1	PGE2	well_1	F006	22	Count/cell	34.63158	34	14.24863
GJA1	W1	PGE2	well_2	F001	1	Count/cell	5	5	NA
GJA1	W1	PGE2	well_2	F002	1	Count/cell	5	5	NA
GJA1	W1	PGE2	well_2	F003	1	Count/cell	14	14	NA
GJA1	W1	PGE2	well_2	F004	22	Count/cell	21.61111	20	13.20044

GJA1	W1	PGE2	well_2	F005	21	Count/cell	26.4	21.5	14.66252
GJA1	W1	PGE2	well_2	F006	4	Count/cell	25.5	25.5	3.535534
GJA1	W1	PGE2	well_3	F002	5	Count/cell	19	19	11
GJA1	W1	PGE2	well_3	F004	7	Count/cell	26.8	26	5.263079
GJA1	W1	PGE2	well_3	F005	50	Count/cell	26.29545	20	20.33736
GJA1	W1	PGE2	well_3	F006	4	Count/cell	31	31	1.414214
GJA1	W1	PGE2	well_4	F001	4	Count/cell	27	27	8.485281
GJA1	W1	PGE2	well_4	F002	3	Count/cell	27	27	NA
GJA1	W1	PGE2	well_4	F003	13	Count/cell	21	19	15.15376
GJA1	W1	PGE2	well_4	F004	4	Count/cell	36	36	0
GJA1	W1	PGE2	well_4	F006	1	Count/cell	115	115	NA
GJA1	W1	PGE2	well_5	F001	10	Count/cell	39	40	15.52878
GJA1	W1	PGE2	well_5	F002	7	Count/cell	58	70	35.81899
GJA1	W1	PGE2	well_5	F003	5	Count/cell	47	41	17.77639
GJA1	W1	PGE2	well_5	F004	1	Count/cell	36	36	NA
GJA1	W1	PGE2	well_5	F005	23	Count/cell	49.63158	45	18.12368
GJA1	W1	PGE2	well_5	F006	16	Count/cell	35.64286	37.5	20.8939
GJA1	W2	HPI4	well_1	F001	1	Count/cell	16	16	NA
GJA1	W2	HPI4	well_1	F002	3	Count/cell	25	25	NA
GJA1	W2	HPI4	well_2	F003	1	Count/cell	20	20	NA
GJA1	W2	HPI4	well_2	F004	58	Count/cell	11.71154	10	6.926325
GJA1	W2	HPI4	well_2	F005	1	Count/cell	23	23	NA
GJA1	W2	HPI4	well_2	F006	8	Count/cell	30.16667	31.5	6.112828
GJA1	W2	HPI4	well_3	F001	15	Count/cell	20.78571	17.5	17.96654
GJA1	W2	HPI4	well_3	F002	13	Count/cell	18.63636	21	11.73263
GJA1	W2	HPI4	well_3	F004	42	Count/cell	17.5	16	10.01013
GJA1	W2	HPI4	well_3	F005	18	Count/cell	16.70588	15	10.39089
GJA1	W2	HPI4	well_3	F006	12	Count/cell	10.1	7	7.781031
GJA1	W2	HPI4	well_4	F001	41	Count/cell	33.51351	30	19.39304
GJA1	W2	HPI4	well_4	F002	11	Count/cell	27.22222	25	18.43758
GJA1	W2	HPI4	well_4	F003	4	Count/cell	10.33333	7	5.773503
GJA1	W2	HPI4	well_4	F004	43	Count/cell	9	7	7.009152
GJA1	W2	HPI4	well_4	F005	25	Count/cell	27.57143	25	14.02701
GJA1	W2	HPI4	well_4	F006	18	Count/cell	26.125	26.5	10.61995
GJA1	W2	HPI4	well_5	F001	12	Count/cell	20.8	19	9.704524
GJA1	W2	HPI4	well_5	F002	23	Count/cell	24.78947	24	10.86063
GJA1	W2	HPI4	well_5	F003	1	Count/cell	24	24	NA
GJA1	W2	HPI4	well_5	F004	47	Count/cell	22.90244	25	12.09505
GJA1	W2	HPI4	well_5	F005	28	Count/cell	23.91667	26.5	10.10775
GJA1	W2	HPI4	well_5	F006	20	Count/cell	24.83333	27	12.27743
GJA1	W2	PGE2	well_1	F001	102	Count/cell	23.3956	22	11.19809
GJA1	W2	PGE2	well_1	F002	48	Count/cell	28.5	27	9.635428
GJA1	W2	PGE2	well_1	F003	76	Count/cell	9.902778	6.5	7.966761
GJA1	W2	PGE2	well_1	F004	23	Count/cell	14.05263	13	6.620454
GJA1	W2	PGE2	well_1	F005	189	Count/cell	15.06145	11	11.77798
GJA1	W2	PGE2	well_1	F006	101	Count/cell	26.98913	27	9.036549
GJA1	W2	PGE2	well_2	F001	14	Count/cell	9	9.5	3.357488
GJA1	W2	PGE2	well_2	F002	21	Count/cell	15.5	16	5.472419
GJA1	W2	PGE2	well_2	F003	25	Count/cell	14.90909	15	6.186845

GJA1	W2	PGE2	well_2	F004	20	Count/cell	14.15789	14	11.77692
GJA1	W2	PGE2	well_2	F005	50	Count/cell	15.65909	15	8.14389
GJA1	W2	PGE2	well_2	F006	12	Count/cell	28.5	28.5	8.045012
GJA1	W2	PGE2	well_3	F001	28	Count/cell	14.44	12	9.032718
GJA1	W2	PGE2	well_3	F002	13	Count/cell	17.72727	13	9.00101
GJA1	W2	PGE2	well_3	F003	25	Count/cell	27.61905	30	10.4378
GJA1	W2	PGE2	well_3	F004	47	Count/cell	25.5122	26	6.538815
GJA1	W2	PGE2	well_3	F005	85	Count/cell	19.89474	18	13.89732
GJA1	W2	PGE2	well_3	F006	24	Count/cell	14.85	15	6.611593
GJA1	W2	PGE2	well_4	F001	6	Count/cell	18.25	19	6.396614
GJA1	W2	PGE2	well_4	F002	25	Count/cell	20.09524	20	8.496498
GJA1	W2	PGE2	well_4	F003	50	Count/cell	28.18182	26	8.999413
GJA1	W2	PGE2	well_4	F004	7	Count/cell	6.166667	5	4.020779
GJA1	W2	PGE2	well_4	F005	24	Count/cell	17.15	15	6.714282
GJA1	W2	PGE2	well_4	F006	40	Count/cell	13.69444	12	8.070473
GJA1	W2	PGE2	well_5	F001	59	Count/cell	15.79245	16	10.01798
GJA1	W2	PGE2	well_5	F002	18	Count/cell	21.5625	20.5	5.573374
GJA1	W2	PGE2	well_5	F003	19	Count/cell	19.5	17.5	8.806749
GJA1	W2	PGE2	well_5	F004	6	Count/cell	10.6	8	5.98331
GJA1	W2	PGE2	well_5	F005	18	Count/cell	17.6875	13	14.40703
GJA1	W2	PGE2	well_5	F006	18	Count/cell	11.35294	10	6.936688
GJA1	W3	HPI4	well_1	F001	1	Count/cell	32	32	NA
GJA1	W3	HPI4	well_1	F003	4	Count/cell	6.5	6.5	3.535534
GJA1	W3	HPI4	well_1	F005	6	Count/cell	11.2	9	6.140033
GJA1	W3	HPI4	well_1	F006	5	Count/cell	47.33333	39	24.58319
GJA1	W3	HPI4	well_2	F001	5	Count/cell	21.66667	17	13.61372
GJA1	W3	HPI4	well_2	F002	4	Count/cell	35	35	8.485281
GJA1	W3	HPI4	well_2	F004	11	Count/cell	27.3	28.5	10.61498
GJA1	W3	HPI4	well_2	F005	3	Count/cell	5	5	NA
GJA1	W3	HPI4	well_2	F006	8	Count/cell	10.16667	9	8.818541
GJA1	W3	HPI4	well_3	F001	4	Count/cell	16	16	0
GJA1	W3	HPI4	well_3	F002	8	Count/cell	37.42857	28	39.3779
GJA1	W3	HPI4	well_3	F003	4	Count/cell	38.5	38.5	10.6066
GJA1	W3	HPI4	well_3	F004	4	Count/cell	7	7	1.414214
GJA1	W3	HPI4	well_3	F005	9	Count/cell	16	15	11.48913
GJA1	W3	HPI4	well_3	F006	1	Count/cell	5	5	NA
GJA1	W3	HPI4	well_4	F001	4	Count/cell	7.5	7.5	6.363961
GJA1	W3	HPI4	well_4	F002	1	Count/cell	6	6	NA
GJA1	W3	HPI4	well_4	F004	4	Count/cell	12	12	0
GJA1	W3	HPI4	well_4	F005	1	Count/cell	29	29	NA
GJA1	W3	HPI4	well_4	F006	4	Count/cell	51	51	2.828427
GJA1	W3	HPI4	well_5	F001	4	Count/cell	25.5	25.5	0.707107
GJA1	W3	HPI4	well_5	F002	19	Count/cell	49.52941	48	27.10193
GJA1	W3	HPI4	well_5	F004	16	Count/cell	17.07143	13.5	14.11199
GJA1	W3	HPI4	well_5	F005	14	Count/cell	44.08333	37	21.00848
GJA1	W3	HPI4	well_5	F006	16	Count/cell	33.21429	32.5	21.02079
GJA1	W3	PGE2	well_1	F001	14	Count/cell	14.61538	14	8.312024
GJA1	W3	PGE2	well_1	F002	198	Count/cell	5.388298	4	4.517733
GJA1	W3	PGE2	well_1	F003	188	Count/cell	31.16071	29	14.30436

GJA1	W3	PGE2	well_1	F004	100	Count/cell	19.90323	19	12.84952
GJA1	W3	PGE2	well_1	F005	99	Count/cell	8.308511	6	7.399545
GJA1	W3	PGE2	well_1	F006	321	Count/cell	27.4433	27	9.624289
GJA1	W3	PGE2	well_2	F001	189	Count/cell	25.82558	25.5	10.023
GJA1	W3	PGE2	well_2	F002	201	Count/cell	9.413613	6	8.342764
GJA1	W3	PGE2	well_2	F003	252	Count/cell	27.69163	25	16.28268
GJA1	W3	PGE2	well_2	F004	273	Count/cell	12.5315	11	7.019805
GJA1	W3	PGE2	well_2	F005	270	Count/cell	14.99203	12	10.01958
GJA1	W3	PGE2	well_2	F006	199	Count/cell	21.14917	21	9.554805
GJA1	W3	PGE2	well_3	F001	151	Count/cell	10.20979	10	6.470794
GJA1	W3	PGE2	well_3	F002	177	Count/cell	5.380952	3	5.9718
GJA1	W3	PGE2	well_3	F003	95	Count/cell	3.366667	2	3.258351
GJA1	W3	PGE2	well_3	F004	184	Count/cell	7	4	7.272649
GJA1	W3	PGE2	well_3	F005	111	Count/cell	3.367925	2	3.591857
GJA1	W3	PGE2	well_3	F006	164	Count/cell	22.62162	22	10.81345
GJA1	W3	PGE2	well_4	F001	190	Count/cell	12.98857	12	7.040106
GJA1	W3	PGE2	well_4	F002	170	Count/cell	12.35294	12	5.191682
GJA1	W3	PGE2	well_4	F003	89	Count/cell	7.5	4	7.607067
GJA1	W3	PGE2	well_4	F004	179	Count/cell	7.217647	5	6.056415
GJA1	W3	PGE2	well_4	F005	304	Count/cell	8.942446	8	4.501536
GJA1	W3	PGE2	well_4	F006	248	Count/cell	26.13839	25	11.25663
GJA1	W3	PGE2	well_5	F001	198	Count/cell	38.16111	36	17.82697
GJA1	W3	PGE2	well_5	F002	208	Count/cell	21.91489	18	14.2079
GJA1	W3	PGE2	well_5	F003	150	Count/cell	24.34752	22	15.44205
GJA1	W3	PGE2	well_5	F004	70	Count/cell	6.941176	3.5	7.693537
GJA1	W3	PGE2	well_5	F005	97	Count/cell	7.827957	6	6.962046
GJA1	W3	PGE2	well_5	F006	111	Count/cell	22.51485	21	13.36459
GJA1	W4	HPI4	well_1	F001	7	Count/cell	38.4	38	13.3529
GJA1	W4	HPI4	well_1	F002	16	Count/cell	40.14286	42.5	12.76327
GJA1	W4	HPI4	well_1	F003	37	Count/cell	39.15152	37	18.1919
GJA1	W4	HPI4	well_1	F004	28	Count/cell	26.5	23.5	15.02462
GJA1	W4	HPI4	well_1	F005	8	Count/cell	28.16667	26	6.853223
GJA1	W4	HPI4	well_1	F006	17	Count/cell	25.6	22	15.44021
GJA1	W4	HPI4	well_2	F001	9	Count/cell	24.14286	17	17.25716
GJA1	W4	HPI4	well_2	F002	12	Count/cell	21.5	22	13.89044
GJA1	W4	HPI4	well_2	F003	24	Count/cell	19	18.5	13.13493
GJA1	W4	HPI4	well_2	F004	44	Count/cell	11	8	8.826098
GJA1	W4	HPI4	well_2	F005	15	Count/cell	50.78571	49.5	22.14351
GJA1	W4	HPI4	well_2	F006	3	Count/cell	31	31	NA
GJA1	W4	HPI4	well_3	F001	9	Count/cell	30.42857	28	14.3278
GJA1	W4	HPI4	well_3	F002	8	Count/cell	26.33333	26	12.43651
GJA1	W4	HPI4	well_3	F003	15	Count/cell	27.61538	20	23.92885
GJA1	W4	HPI4	well_3	F004	13	Count/cell	14	11	12.32883
GJA1	W4	HPI4	well_3	F005	5	Count/cell	36.33333	34	4.932883
GJA1	W4	HPI4	well_3	F006	21	Count/cell	34.42105	26	23.29834
GJA1	W4	HPI4	well_4	F002	25	Count/cell	21.09091	18	14.45159
GJA1	W4	HPI4	well_4	F003	8	Count/cell	29.5	25.5	12.27599
GJA1	W4	HPI4	well_4	F004	25	Count/cell	11.86364	8	7.655035
GJA1	W4	HPI4	well_4	F005	70	Count/cell	27.66667	27	12.3654

GJA1	W4	HPI4	well_4	F006	50	Count/cell	28.65909	27	11.33413
GJA1	W4	HPI4	well_5	F001	11	Count/cell	55.7	56.5	43.69859
GJA1	W4	HPI4	well_5	F002	15	Count/cell	27.69231	25	22.85237
GJA1	W4	HPI4	well_5	F003	17	Count/cell	49.33333	47	30.26234
GJA1	W4	HPI4	well_5	F004	29	Count/cell	26.72	21	15.91781
GJA1	W4	HPI4	well_5	F005	91	Count/cell	15.31707	16	5.293551
GJA1	W4	HPI4	well_5	F006	10	Count/cell	31	20	23.57359
GJA1	W4	PGE2	well_1	F001	43	Count/cell	42.51351	41	17.46683
GJA1	W4	PGE2	well_1	F002	113	Count/cell	16.51402	10	15.71585
GJA1	W4	PGE2	well_1	F003	306	Count/cell	7.401361	7	4.967691
GJA1	W4	PGE2	well_1	F004	34	Count/cell	35.43333	31.5	19.04565
GJA1	W4	PGE2	well_1	F005	55	Count/cell	11.1	8	8.694943
GJA1	W4	PGE2	well_1	F006	329	Count/cell	8.785942	8	6.037452
GJA1	W4	PGE2	well_2	F001	200	Count/cell	8.885417	9	6.021549
GJA1	W4	PGE2	well_2	F002	159	Count/cell	16.85517	17	9.619843
GJA1	W4	PGE2	well_2	F003	277	Count/cell	8.532319	7	6.726805
GJA1	W4	PGE2	well_2	F004	252	Count/cell	12.0083	9	9.800932
GJA1	W4	PGE2	well_2	F005	168	Count/cell	6.31875	5	5.51199
GJA1	W4	PGE2	well_2	F006	175	Count/cell	14.01807	13	9.545726
GJA1	W4	PGE2	well_3	F001	85	Count/cell	34.37333	32	13.53451
GJA1	W4	PGE2	well_3	F002	306	Count/cell	9.436426	7	8.276176
GJA1	W4	PGE2	well_3	F003	220	Count/cell	15.77114	13	11.94267
GJA1	W4	PGE2	well_3	F004	40	Count/cell	6.131579	4	5.850253
GJA1	W4	PGE2	well_3	F005	19	Count/cell	4.555556	3.5	3.791976
GJA1	W4	PGE2	well_3	F006	144	Count/cell	12.29412	8.5	10.66466
GJA1	W4	PGE2	well_4	F001	100	Count/cell	10.23711	7	9.550058
GJA1	W4	PGE2	well_4	F002	72	Count/cell	6.426471	3	6.230201
GJA1	W4	PGE2	well_4	F003	154	Count/cell	8.280822	5	7.728803
GJA1	W4	PGE2	well_4	F004	292	Count/cell	11.86331	11	7.233797
GJA1	W4	PGE2	well_4	F005	212	Count/cell	18.5	17	11.27943
GJA1	W4	PGE2	well_4	F006	60	Count/cell	5.701754	5	3.863589
GJA1	W4	PGE2	well_5	F001	197	Count/cell	8.44385	7	6.355998
GJA1	W4	PGE2	well_5	F002	177	Count/cell	14.06173	14	6.641383
GJA1	W4	PGE2	well_5	F003	124	Count/cell	7.245763	5	6.594399
GJA1	W4	PGE2	well_5	F004	124	Count/cell	24.27027	25	10.12912
GJA1	W4	PGE2	well_5	F005	91	Count/cell	8.827586	7	7.564001
GJA1	W4	PGE2	well_5	F006	97	Count/cell	19.89773	18.5	12.04784
GJA1	W1	HPI4	well_1	F001	9	X/Y distribution	9.721608	8.816103	1.96274
GJA1	W1	HPI4	well_1	F002	13	X/Y distribution	8.122752	8.343664	1.675106
GJA1	W1	HPI4	well_1	F003	4	X/Y distribution	7.151777	7.151777	0.931236
GJA1	W1	HPI4	well_1	F004	15	X/Y distribution	7.692735	7.776975	1.296154
GJA1	W1	HPI4	well_1	F005	1	X/Y distribution	7.466212	7.466212	NA
GJA1	W1	HPI4	well_1	F006	4	X/Y distribution	7.945561	7.945561	0.988848
GJA1	W1	HPI4	well_2	F001	3	X/Y distribution	4.767994	4.767994	NA
GJA1	W1	HPI4	well_2	F002	6	X/Y distribution	8.444393	8.489461	1.77526
GJA1	W1	HPI4	well_2	F003	6	X/Y distribution	6.714451	6.756939	0.813521
GJA1	W1	HPI4	well_2	F004	7	X/Y distribution	8.284279	7.667792	2.816157
GJA1	W1	HPI4	well_2	F005	5	X/Y distribution	7.361812	6.16173	2.600287
GJA1	W1	HPI4	well_2	F006	4	X/Y distribution	8.001354	8.001354	0.523997

GJA1	W1	HPI4	well_3	F002	7	X/Y distribution	7.428364	5.818704	3.387403
GJA1	W1	HPI4	well_3	F003	3	X/Y distribution	5.348659	5.348659	NA
GJA1	W1	HPI4	well_3	F004	1	X/Y distribution	8.959008	8.959008	NA
GJA1	W1	HPI4	well_3	F005	7	X/Y distribution	8.528327	7.370239	3.337278
GJA1	W1	HPI4	well_3	F006	7	X/Y distribution	7.566156	7.311661	1.215175
GJA1	W1	HPI4	well_4	F001	19	X/Y distribution	7.191391	7.259	1.393624
GJA1	W1	HPI4	well_4	F002	1	X/Y distribution	8.304098	8.304098	NA
GJA1	W1	HPI4	well_4	F003	14	X/Y distribution	8.518962	9.054892	1.867719
GJA1	W1	HPI4	well_4	F004	11	X/Y distribution	7.699926	6.955636	2.56814
GJA1	W1	HPI4	well_4	F006	4	X/Y distribution	5.857326	5.857326	2.902898
GJA1	W1	HPI4	well_5	F001	3	X/Y distribution	7.170602	7.170602	NA
GJA1	W1	HPI4	well_5	F002	66	X/Y distribution	8.553067	8.316672	1.574101
GJA1	W1	HPI4	well_5	F003	6	X/Y distribution	8.718849	8.773245	2.008789
GJA1	W1	HPI4	well_5	F004	7	X/Y distribution	8.109225	6.979665	1.689826
GJA1	W1	HPI4	well_5	F005	29	X/Y distribution	7.440581	7.342945	1.86659
GJA1	W1	HPI4	well_5	F006	5	X/Y distribution	6.943942	6.124974	1.428176
GJA1	W1	PGE2	well_1	F002	1	X/Y distribution	7.643102	7.643102	NA
GJA1	W1	PGE2	well_1	F003	11	X/Y distribution	6.882474	6.900043	1.57495
GJA1	W1	PGE2	well_1	F004	4	X/Y distribution	5.912731	5.912731	0.531828
GJA1	W1	PGE2	well_1	F005	4	X/Y distribution	10.73268	10.73268	0.258864
GJA1	W1	PGE2	well_1	F006	22	X/Y distribution	8.304356	7.85136	2.290078
GJA1	W1	PGE2	well_2	F001	1	X/Y distribution	8.464845	8.464845	NA
GJA1	W1	PGE2	well_2	F002	1	X/Y distribution	2.468616	2.468616	NA
GJA1	W1	PGE2	well_2	F003	1	X/Y distribution	6.880001	6.880001	NA
GJA1	W1	PGE2	well_2	F004	22	X/Y distribution	8.500202	8.406051	1.432894
GJA1	W1	PGE2	well_2	F005	21	X/Y distribution	8.412575	7.863659	2.373325
GJA1	W1	PGE2	well_2	F006	4	X/Y distribution	8.1603	8.1603	0.573428
GJA1	W1	PGE2	well_3	F002	5	X/Y distribution	6.716383	6.781002	0.382148
GJA1	W1	PGE2	well_3	F004	7	X/Y distribution	6.792929	6.550863	1.374001
GJA1	W1	PGE2	well_3	F005	50	X/Y distribution	8.553331	8.385163	1.729092
GJA1	W1	PGE2	well_3	F006	4	X/Y distribution	7.900533	7.900533	0.437403
GJA1	W1	PGE2	well_4	F001	4	X/Y distribution	8.916345	8.916345	1.112871
GJA1	W1	PGE2	well_4	F002	3	X/Y distribution	8.590676	8.590676	NA
GJA1	W1	PGE2	well_4	F003	13	X/Y distribution	8.571738	8.187858	1.731268
GJA1	W1	PGE2	well_4	F004	4	X/Y distribution	7.156271	7.156271	0.01352
GJA1	W1	PGE2	well_4	F006	1	X/Y distribution	10.01831	10.01831	NA
GJA1	W1	PGE2	well_5	F001	10	X/Y distribution	7.552109	7.661694	0.909403
GJA1	W1	PGE2	well_5	F002	7	X/Y distribution	9.483817	9.51645	1.566298
GJA1	W1	PGE2	well_5	F003	5	X/Y distribution	7.347202	6.989369	1.714685
GJA1	W1	PGE2	well_5	F004	1	X/Y distribution	8.103216	8.103216	NA
GJA1	W1	PGE2	well_5	F005	23	X/Y distribution	8.140071	7.838151	1.510007
GJA1	W1	PGE2	well_5	F006	16	X/Y distribution	7.9801	7.126382	2.322477
GJA1	W2	HPI4	well_1	F001	1	X/Y distribution	4.249861	4.249861	NA
GJA1	W2	HPI4	well_1	F002	3	X/Y distribution	9.151418	9.151418	NA
GJA1	W2	HPI4	well_2	F003	1	X/Y distribution	10.10235	10.10235	NA
GJA1	W2	HPI4	well_2	F004	58	X/Y distribution	5.0654	4.593553	1.718002
GJA1	W2	HPI4	well_2	F005	1	X/Y distribution	7.054602	7.054602	NA
GJA1	W2	HPI4	well_2	F006	8	X/Y distribution	9.015619	9.508885	1.313017
GJA1	W2	HPI4	well_3	F001	15	X/Y distribution	4.488153	4.481492	1.651635

GJA1	W2	HPI4	well_3	F002	13	X/Y distribution	5.688624	5.625758	1.116129
GJA1	W2	HPI4	well_3	F004	42	X/Y distribution	6.378546	6.478114	1.323234
GJA1	W2	HPI4	well_3	F005	18	X/Y distribution	7.25613	6.675323	1.663498
GJA1	W2	HPI4	well_3	F006	12	X/Y distribution	7.610766	6.734302	1.828448
GJA1	W2	HPI4	well_4	F001	41	X/Y distribution	5.239857	4.950806	1.087979
GJA1	W2	HPI4	well_4	F002	11	X/Y distribution	8.721321	9.042032	2.002937
GJA1	W2	HPI4	well_4	F003	4	X/Y distribution	4.78039	4.78039	0.782908
GJA1	W2	HPI4	well_4	F004	43	X/Y distribution	7.004647	6.657882	2.206857
GJA1	W2	HPI4	well_4	F005	25	X/Y distribution	6.787136	6.631023	2.157238
GJA1	W2	HPI4	well_4	F006	18	X/Y distribution	7.917238	7.965804	2.086842
GJA1	W2	HPI4	well_5	F001	12	X/Y distribution	7.051344	7.675472	1.93353
GJA1	W2	HPI4	well_5	F002	23	X/Y distribution	4.984208	4.748412	0.784879
GJA1	W2	HPI4	well_5	F003	1	X/Y distribution	4.284212	4.284212	NA
GJA1	W2	HPI4	well_5	F004	47	X/Y distribution	6.79079	6.744852	1.496888
GJA1	W2	HPI4	well_5	F005	28	X/Y distribution	6.414794	6.294814	1.353032
GJA1	W2	HPI4	well_5	F006	20	X/Y distribution	8.018673	7.938544	1.916556
GJA1	W2	PGE2	well_1	F001	102	X/Y distribution	6.574041	6.370257	1.114176
GJA1	W2	PGE2	well_1	F002	48	X/Y distribution	6.775456	6.47376	1.357146
GJA1	W2	PGE2	well_1	F003	76	X/Y distribution	8.573954	8.792324	1.889448
GJA1	W2	PGE2	well_1	F004	23	X/Y distribution	6.146793	6.35443	0.92172
GJA1	W2	PGE2	well_1	F005	189	X/Y distribution	6.221519	6.074547	0.900358
GJA1	W2	PGE2	well_1	F006	101	X/Y distribution	6.646162	6.679196	1.10305
GJA1	W2	PGE2	well_2	F001	14	X/Y distribution	5.336108	4.840442	1.471918
GJA1	W2	PGE2	well_2	F002	21	X/Y distribution	6.042041	5.77733	1.254507
GJA1	W2	PGE2	well_2	F003	25	X/Y distribution	6.24264	6.591314	1.147585
GJA1	W2	PGE2	well_2	F004	20	X/Y distribution	7.48585	7.210527	1.771733
GJA1	W2	PGE2	well_2	F005	50	X/Y distribution	7.059307	6.698349	1.426289
GJA1	W2	PGE2	well_2	F006	12	X/Y distribution	7.126212	7.424485	1.039844
GJA1	W2	PGE2	well_3	F001	28	X/Y distribution	6.852774	7.038195	1.099528
GJA1	W2	PGE2	well_3	F002	13	X/Y distribution	7.642582	7.828416	1.975202
GJA1	W2	PGE2	well_3	F003	25	X/Y distribution	8.401071	8.176996	1.586125
GJA1	W2	PGE2	well_3	F004	47	X/Y distribution	6.208776	6.341102	0.814651
GJA1	W2	PGE2	well_3	F005	85	X/Y distribution	6.974593	6.932065	1.254842
GJA1	W2	PGE2	well_3	F006	24	X/Y distribution	6.359621	6.278172	1.224943
GJA1	W2	PGE2	well_4	F001	6	X/Y distribution	7.916818	7.744928	1.37114
GJA1	W2	PGE2	well_4	F002	25	X/Y distribution	5.716035	5.759395	1.025665
GJA1	W2	PGE2	well_4	F003	50	X/Y distribution	7.602907	7.390718	1.460126
GJA1	W2	PGE2	well_4	F004	7	X/Y distribution	7.008425	6.753732	1.317808
GJA1	W2	PGE2	well_4	F005	24	X/Y distribution	6.988694	7.067101	1.265034
GJA1	W2	PGE2	well_4	F006	40	X/Y distribution	7.031638	7.013059	1.376613
GJA1	W2	PGE2	well_5	F001	59	X/Y distribution	6.040383	5.804064	1.124473
GJA1	W2	PGE2	well_5	F002	18	X/Y distribution	8.084994	7.548361	1.809863
GJA1	W2	PGE2	well_5	F003	19	X/Y distribution	6.528583	6.751224	1.617335
GJA1	W2	PGE2	well_5	F004	6	X/Y distribution	6.563952	6.615241	0.473021
GJA1	W2	PGE2	well_5	F005	18	X/Y distribution	5.439958	5.431884	1.001694
GJA1	W2	PGE2	well_5	F006	18	X/Y distribution	5.299587	5.462733	1.236427
GJA1	W3	HPI4	well_1	F001	1	X/Y distribution	3.882108	3.882108	NA
GJA1	W3	HPI4	well_1	F003	4	X/Y distribution	5.092142	5.092142	0.259524
GJA1	W3	HPI4	well_1	F005	6	X/Y distribution	5.469513	5.503892	0.684307

GJA1	W3	HPI4	well_1	F006	5	X/Y distribution	7.286857	6.946401	1.859558
GJA1	W3	HPI4	well_2	F001	5	X/Y distribution	7.801386	7.895633	0.319527
GJA1	W3	HPI4	well_2	F002	4	X/Y distribution	7.677018	7.677018	0.095577
GJA1	W3	HPI4	well_2	F004	11	X/Y distribution	5.436804	5.444998	0.466971
GJA1	W3	HPI4	well_2	F005	3	X/Y distribution	5.177567	5.177567	NA
GJA1	W3	HPI4	well_2	F006	8	X/Y distribution	6.26627	6.419777	1.379552
GJA1	W3	HPI4	well_3	F001	4	X/Y distribution	6.865691	6.865691	3.11187
GJA1	W3	HPI4	well_3	F002	8	X/Y distribution	8.004185	7.90214	2.650504
GJA1	W3	HPI4	well_3	F003	4	X/Y distribution	6.859166	6.859166	0.971342
GJA1	W3	HPI4	well_3	F004	4	X/Y distribution	6.273815	6.273815	0.675854
GJA1	W3	HPI4	well_3	F005	9	X/Y distribution	8.326546	6.667465	3.290806
GJA1	W3	HPI4	well_3	F006	1	X/Y distribution	6.062738	6.062738	NA
GJA1	W3	HPI4	well_4	F001	4	X/Y distribution	5.724517	5.724517	0.111127
GJA1	W3	HPI4	well_4	F002	1	X/Y distribution	2.538519	2.538519	NA
GJA1	W3	HPI4	well_4	F004	4	X/Y distribution	4.84628	4.84628	0.63741
GJA1	W3	HPI4	well_4	F005	1	X/Y distribution	3.895172	3.895172	NA
GJA1	W3	HPI4	well_4	F006	4	X/Y distribution	5.630488	5.630488	0.461866
GJA1	W3	HPI4	well_5	F001	4	X/Y distribution	8.556254	8.556254	0.303912
GJA1	W3	HPI4	well_5	F002	19	X/Y distribution	8.199742	8.30486	1.511967
GJA1	W3	HPI4	well_5	F004	16	X/Y distribution	7.423299	7.307869	1.982813
GJA1	W3	HPI4	well_5	F005	14	X/Y distribution	5.7561	5.104427	1.495299
GJA1	W3	HPI4	well_5	F006	16	X/Y distribution	8.819644	8.506234	2.471789
GJA1	W3	PGE2	well_1	F001	14	X/Y distribution	7.19129	7.449783	1.385852
GJA1	W3	PGE2	well_1	F002	198	X/Y distribution	6.786551	6.713664	1.339444
GJA1	W3	PGE2	well_1	F003	188	X/Y distribution	6.067002	6.041525	1.117136
GJA1	W3	PGE2	well_1	F004	100	X/Y distribution	8.262483	8.182686	1.617441
GJA1	W3	PGE2	well_1	F005	99	X/Y distribution	7.858418	7.847064	1.469331
GJA1	W3	PGE2	well_1	F006	321	X/Y distribution	6.943373	6.991221	0.767468
GJA1	W3	PGE2	well_2	F001	189	X/Y distribution	8.534987	8.449802	1.172388
GJA1	W3	PGE2	well_2	F002	201	X/Y distribution	7.354542	7.107504	1.419737
GJA1	W3	PGE2	well_2	F003	252	X/Y distribution	6.587805	6.502507	1.265745
GJA1	W3	PGE2	well_2	F004	273	X/Y distribution	7.172641	7.049467	1.124755
GJA1	W3	PGE2	well_2	F005	270	X/Y distribution	7.211537	7.149898	1.237041
GJA1	W3	PGE2	well_2	F006	199	X/Y distribution	7.587574	7.602598	1.221368
GJA1	W3	PGE2	well_3	F001	151	X/Y distribution	8.5666	8.301256	2.01971
GJA1	W3	PGE2	well_3	F002	177	X/Y distribution	7.407596	7.404491	1.859884
GJA1	W3	PGE2	well_3	F003	95	X/Y distribution	7.596069	7.756881	2.132468
GJA1	W3	PGE2	well_3	F004	184	X/Y distribution	7.054512	6.741323	1.83619
GJA1	W3	PGE2	well_3	F005	111	X/Y distribution	7.405776	7.12331	1.802384
GJA1	W3	PGE2	well_3	F006	164	X/Y distribution	8.148256	8.141351	1.199239
GJA1	W3	PGE2	well_4	F001	190	X/Y distribution	7.570697	7.496429	1.152475
GJA1	W3	PGE2	well_4	F002	170	X/Y distribution	8.061432	7.892063	1.197079
GJA1	W3	PGE2	well_4	F003	89	X/Y distribution	7.598389	7.268329	2.002355
GJA1	W3	PGE2	well_4	F004	179	X/Y distribution	7.88828	7.751164	1.942679
GJA1	W3	PGE2	well_4	F005	304	X/Y distribution	6.46059	6.405913	1.249262
GJA1	W3	PGE2	well_4	F006	248	X/Y distribution	6.954947	6.789702	1.296032
GJA1	W3	PGE2	well_5	F001	198	X/Y distribution	6.021118	5.995586	1.209722
GJA1	W3	PGE2	well_5	F002	208	X/Y distribution	6.936129	6.713123	1.270628
GJA1	W3	PGE2	well_5	F003	150	X/Y distribution	5.137107	4.886945	1.146127

GJA1	W3	PGE2	well_5	F004	70	X/Y distribution	7.563905	7.834454	1.55989
GJA1	W3	PGE2	well_5	F005	97	X/Y distribution	7.829367	7.797043	1.821096
GJA1	W3	PGE2	well_5	F006	111	X/Y distribution	9.52747	9.381611	1.641167
GJA1	W4	HPI4	well_1	F001	7	X/Y distribution	7.373534	6.370179	2.098852
GJA1	W4	HPI4	well_1	F002	16	X/Y distribution	8.220253	7.642971	2.765818
GJA1	W4	HPI4	well_1	F003	37	X/Y distribution	6.034342	5.888101	0.811608
GJA1	W4	HPI4	well_1	F004	28	X/Y distribution	8.693498	8.874744	1.396188
GJA1	W4	HPI4	well_1	F005	8	X/Y distribution	8.462218	8.748171	1.123275
GJA1	W4	HPI4	well_1	F006	17	X/Y distribution	12.27239	12.14659	3.29623
GJA1	W4	HPI4	well_2	F001	9	X/Y distribution	10.4119	10.72501	1.911381
GJA1	W4	HPI4	well_2	F002	12	X/Y distribution	8.232488	7.673332	2.134155
GJA1	W4	HPI4	well_2	F003	24	X/Y distribution	11.78355	12.10219	2.332481
GJA1	W4	HPI4	well_2	F004	44	X/Y distribution	8.627676	7.97778	2.331592
GJA1	W4	HPI4	well_2	F005	15	X/Y distribution	7.979513	8.104088	1.064175
GJA1	W4	HPI4	well_2	F006	3	X/Y distribution	9.286094	9.286094	NA
GJA1	W4	HPI4	well_3	F001	9	X/Y distribution	7.90369	7.871325	0.974449
GJA1	W4	HPI4	well_3	F002	8	X/Y distribution	9.321682	9.347744	2.155912
GJA1	W4	HPI4	well_3	F003	15	X/Y distribution	10.82632	10.49525	2.23144
GJA1	W4	HPI4	well_3	F004	13	X/Y distribution	8.832827	8.245365	2.276803
GJA1	W4	HPI4	well_3	F005	5	X/Y distribution	8.177607	7.929146	1.514014
GJA1	W4	HPI4	well_3	F006	21	X/Y distribution	9.790478	9.9158	2.817741
GJA1	W4	HPI4	well_4	F002	25	X/Y distribution	10.71296	10.86682	2.183479
GJA1	W4	HPI4	well_4	F003	8	X/Y distribution	8.200636	7.882464	0.934559
GJA1	W4	HPI4	well_4	F004	25	X/Y distribution	8.220993	7.938747	1.190564
GJA1	W4	HPI4	well_4	F005	70	X/Y distribution	6.383422	6.03169	1.396895
GJA1	W4	HPI4	well_4	F006	50	X/Y distribution	6.670823	6.623069	1.352177
GJA1	W4	HPI4	well_5	F001	11	X/Y distribution	7.893087	8.356158	2.165941
GJA1	W4	HPI4	well_5	F002	15	X/Y distribution	7.880329	8.489602	2.137918
GJA1	W4	HPI4	well_5	F003	17	X/Y distribution	8.925485	8.54512	2.042345
GJA1	W4	HPI4	well_5	F004	29	X/Y distribution	8.65588	8.56197	1.452203
GJA1	W4	HPI4	well_5	F005	91	X/Y distribution	6.931881	6.97777	0.925361
GJA1	W4	HPI4	well_5	F006	10	X/Y distribution	9.051983	8.913425	1.116633
GJA1	W4	PGE2	well_1	F001	43	X/Y distribution	5.413421	5.368402	1.267233
GJA1	W4	PGE2	well_1	F002	113	X/Y distribution	5.466457	5.411637	1.266187
GJA1	W4	PGE2	well_1	F003	306	X/Y distribution	6.760048	6.713861	1.028822
GJA1	W4	PGE2	well_1	F004	34	X/Y distribution	7.302865	7.107877	1.507183
GJA1	W4	PGE2	well_1	F005	55	X/Y distribution	6.559851	6.706322	1.40733
GJA1	W4	PGE2	well_1	F006	329	X/Y distribution	6.347358	6.228773	1.0028
GJA1	W4	PGE2	well_2	F001	200	X/Y distribution	6.906857	7.02506	1.200824
GJA1	W4	PGE2	well_2	F002	159	X/Y distribution	7.467844	7.33307	1.077048
GJA1	W4	PGE2	well_2	F003	277	X/Y distribution	6.983035	6.87125	1.555758
GJA1	W4	PGE2	well_2	F004	252	X/Y distribution	7.072951	7.015687	1.130079
GJA1	W4	PGE2	well_2	F005	168	X/Y distribution	7.07491	7.152121	1.596456
GJA1	W4	PGE2	well_2	F006	175	X/Y distribution	6.721805	6.703116	0.894468
GJA1	W4	PGE2	well_3	F001	85	X/Y distribution	6.842934	6.800549	1.241426
GJA1	W4	PGE2	well_3	F002	306	X/Y distribution	6.24471	6.187166	1.399849
GJA1	W4	PGE2	well_3	F003	220	X/Y distribution	6.795451	6.690811	1.339093
GJA1	W4	PGE2	well_3	F004	40	X/Y distribution	5.400276	5.429036	1.588955
GJA1	W4	PGE2	well_3	F005	19	X/Y distribution	7.738273	7.76364	1.920662

GJA1	W4	PGE2	well_3	F006	144	X/Y distribution	7.446891	7.261901	1.464314
GJA1	W4	PGE2	well_4	F001	100	X/Y distribution	8.693452	8.548925	2.121192
GJA1	W4	PGE2	well_4	F002	72	X/Y distribution	8.017252	7.949474	1.602907
GJA1	W4	PGE2	well_4	F003	154	X/Y distribution	7.995952	7.837038	1.522101
GJA1	W4	PGE2	well_4	F004	292	X/Y distribution	6.87087	6.859541	1.012843
GJA1	W4	PGE2	well_4	F005	212	X/Y distribution	6.142003	6.184022	1.081929
GJA1	W4	PGE2	well_4	F006	60	X/Y distribution	6.802658	6.927268	1.336777
GJA1	W4	PGE2	well_5	F001	197	X/Y distribution	7.007342	6.863231	1.225685
GJA1	W4	PGE2	well_5	F002	177	X/Y distribution	7.950897	7.706821	1.260723
GJA1	W4	PGE2	well_5	F003	124	X/Y distribution	6.79672	6.702136	1.326161
GJA1	W4	PGE2	well_5	F004	124	X/Y distribution	7.567815	7.25291	1.472755
GJA1	W4	PGE2	well_5	F005	91	X/Y distribution	7.012338	7.073686	1.295602
GJA1	W4	PGE2	well_5	F006	97	X/Y distribution	9.294171	9.139731	2.098023
GJA1	W1	HPI4	well_1	F001	9	Z distribution	-0.2867	-0.33995	0.181957
GJA1	W1	HPI4	well_1	F002	13	Z distribution	-0.41819	-0.41703	0.160443
GJA1	W1	HPI4	well_1	F003	4	Z distribution	-0.55192	-0.55192	0.002516
GJA1	W1	HPI4	well_1	F004	15	Z distribution	-0.20208	-0.16072	0.345785
GJA1	W1	HPI4	well_1	F005	1	Z distribution	-0.65797	-0.65797	NA
GJA1	W1	HPI4	well_1	F006	4	Z distribution	-0.13599	-0.13599	0.136965
GJA1	W1	HPI4	well_2	F001	3	Z distribution	-0.67205	-0.67205	NA
GJA1	W1	HPI4	well_2	F002	6	Z distribution	-0.38542	-0.38422	0.055636
GJA1	W1	HPI4	well_2	F003	6	Z distribution	-0.61434	-0.59005	0.232398
GJA1	W1	HPI4	well_2	F004	7	Z distribution	-0.35762	-0.34073	0.098622
GJA1	W1	HPI4	well_2	F005	5	Z distribution	-0.41782	-0.43883	0.084521
GJA1	W1	HPI4	well_2	F006	4	Z distribution	-0.35794	-0.35794	0.10023
GJA1	W1	HPI4	well_3	F002	7	Z distribution	-0.55538	-0.69992	0.256109
GJA1	W1	HPI4	well_3	F003	3	Z distribution	-0.11151	-0.11151	NA
GJA1	W1	HPI4	well_3	F004	1	Z distribution	-1.31664	-1.31664	NA
GJA1	W1	HPI4	well_3	F005	7	Z distribution	-0.48565	-0.5066	0.144985
GJA1	W1	HPI4	well_3	F006	7	Z distribution	-0.4727	-0.50317	0.214226
GJA1	W1	HPI4	well_4	F001	19	Z distribution	-0.25729	-0.24696	0.229624
GJA1	W1	HPI4	well_4	F002	1	Z distribution	0.124162	0.124162	NA
GJA1	W1	HPI4	well_4	F003	14	Z distribution	-0.42443	-0.41095	0.285722
GJA1	W1	HPI4	well_4	F004	11	Z distribution	-0.20994	-0.10535	0.179643
GJA1	W1	HPI4	well_4	F006	4	Z distribution	-0.68824	-0.68824	0.309348
GJA1	W1	HPI4	well_5	F001	3	Z distribution	-0.89811	-0.89811	NA
GJA1	W1	HPI4	well_5	F002	66	Z distribution	-0.39633	-0.36642	0.356663
GJA1	W1	HPI4	well_5	F003	6	Z distribution	-0.15259	-0.05531	0.237424
GJA1	W1	HPI4	well_5	F004	7	Z distribution	-0.31384	-0.25483	0.13398
GJA1	W1	HPI4	well_5	F005	29	Z distribution	-0.59524	-0.6389	0.379429
GJA1	W1	HPI4	well_5	F006	5	Z distribution	-0.42576	-0.47171	0.162904
GJA1	W1	PGE2	well_1	F002	1	Z distribution	-0.69846	-0.69846	NA
GJA1	W1	PGE2	well_1	F003	11	Z distribution	-0.26293	-0.32068	0.221403
GJA1	W1	PGE2	well_1	F004	4	Z distribution	-0.17412	-0.17412	0.124772
GJA1	W1	PGE2	well_1	F005	4	Z distribution	-0.3503	-0.3503	0.047901
GJA1	W1	PGE2	well_1	F006	22	Z distribution	-0.30692	-0.28699	0.173886
GJA1	W1	PGE2	well_2	F001	1	Z distribution	-0.98161	-0.98161	NA
GJA1	W1	PGE2	well_2	F002	1	Z distribution	-0.0825	-0.0825	NA
GJA1	W1	PGE2	well_2	F003	1	Z distribution	-0.62334	-0.62334	NA

GJA1	W1	PGE2	well_2	F004	22	Z distribution	-0.35239	-0.39353	0.224989
GJA1	W1	PGE2	well_2	F005	21	Z distribution	-0.34381	-0.37824	0.27965
GJA1	W1	PGE2	well_2	F006	4	Z distribution	-0.34667	-0.34667	0.244061
GJA1	W1	PGE2	well_3	F002	5	Z distribution	-0.35025	-0.36735	0.047702
GJA1	W1	PGE2	well_3	F004	7	Z distribution	-0.45728	-0.47013	0.134953
GJA1	W1	PGE2	well_3	F005	50	Z distribution	-0.1181	-0.07031	0.295469
GJA1	W1	PGE2	well_3	F006	4	Z distribution	-0.24376	-0.24376	0.113931
GJA1	W1	PGE2	well_4	F001	4	Z distribution	-0.47653	-0.47653	0.168645
GJA1	W1	PGE2	well_4	F002	3	Z distribution	-0.12227	-0.12227	NA
GJA1	W1	PGE2	well_4	F003	13	Z distribution	-0.53817	-0.55167	0.161967
GJA1	W1	PGE2	well_4	F004	4	Z distribution	-0.17347	-0.17347	0.263437
GJA1	W1	PGE2	well_4	F006	1	Z distribution	-0.78956	-0.78956	NA
GJA1	W1	PGE2	well_5	F001	10	Z distribution	-0.46013	-0.4877	0.216928
GJA1	W1	PGE2	well_5	F002	7	Z distribution	-0.1805	-0.17518	0.102411
GJA1	W1	PGE2	well_5	F003	5	Z distribution	-0.56503	-0.57636	0.048266
GJA1	W1	PGE2	well_5	F004	1	Z distribution	-0.53315	-0.53315	NA
GJA1	W1	PGE2	well_5	F005	23	Z distribution	-0.19858	-0.14602	0.244492
GJA1	W1	PGE2	well_5	F006	16	Z distribution	-0.25961	-0.25214	0.245587
GJA1	W2	HPI4	well_1	F001	1	Z distribution	-0.74433	-0.74433	NA
GJA1	W2	HPI4	well_1	F002	3	Z distribution	-0.43273	-0.43273	NA
GJA1	W2	HPI4	well_2	F003	1	Z distribution	-0.37306	-0.37306	NA
GJA1	W2	HPI4	well_2	F004	58	Z distribution	-0.30999	-0.32163	0.234829
GJA1	W2	HPI4	well_2	F005	1	Z distribution	-0.52345	-0.52345	NA
GJA1	W2	HPI4	well_2	F006	8	Z distribution	-0.27337	-0.26614	0.179567
GJA1	W2	HPI4	well_3	F001	15	Z distribution	-0.53006	-0.4025	0.467583
GJA1	W2	HPI4	well_3	F002	13	Z distribution	-0.71016	-0.68025	0.25709
GJA1	W2	HPI4	well_3	F004	42	Z distribution	-0.277	-0.2304	0.314734
GJA1	W2	HPI4	well_3	F005	18	Z distribution	-0.32051	-0.10586	0.407759
GJA1	W2	HPI4	well_3	F006	12	Z distribution	-0.26079	-0.2935	0.432613
GJA1	W2	HPI4	well_4	F001	41	Z distribution	-0.07804	-0.05361	0.234306
GJA1	W2	HPI4	well_4	F002	11	Z distribution	-0.45435	-0.47242	0.270076
GJA1	W2	HPI4	well_4	F003	4	Z distribution	-0.34026	-0.34026	0.112637
GJA1	W2	HPI4	well_4	F004	43	Z distribution	-0.44778	-0.45873	0.339745
GJA1	W2	HPI4	well_4	F005	25	Z distribution	-0.28272	-0.20066	0.199276
GJA1	W2	HPI4	well_4	F006	18	Z distribution	-0.39396	-0.40175	0.222948
GJA1	W2	HPI4	well_5	F001	12	Z distribution	-0.57602	-0.55832	0.258871
GJA1	W2	HPI4	well_5	F002	23	Z distribution	-0.14769	-0.06411	0.223478
GJA1	W2	HPI4	well_5	F003	1	Z distribution	-0.48887	-0.48887	NA
GJA1	W2	HPI4	well_5	F004	47	Z distribution	-0.26323	-0.21067	0.282757
GJA1	W2	HPI4	well_5	F005	28	Z distribution	0.079014	0.027879	0.19162
GJA1	W2	HPI4	well_5	F006	20	Z distribution	-0.03831	0.056433	0.30774
GJA1	W2	PGE2	well_1	F001	102	Z distribution	-0.09148	-0.06478	0.271923
GJA1	W2	PGE2	well_1	F002	48	Z distribution	-0.03998	-0.03471	0.213122
GJA1	W2	PGE2	well_1	F003	76	Z distribution	0.138637	0.211209	0.626189
GJA1	W2	PGE2	well_1	F004	23	Z distribution	0.021737	0.052673	0.230977
GJA1	W2	PGE2	well_1	F005	189	Z distribution	0.046477	0.104866	0.485654
GJA1	W2	PGE2	well_1	F006	101	Z distribution	0.252355	0.211986	0.295848
GJA1	W2	PGE2	well_2	F001	14	Z distribution	-0.09904	-0.0947	0.325117
GJA1	W2	PGE2	well_2	F002	21	Z distribution	-0.19559	-0.17548	0.297717

GJA1	W2	PGE2	well_2	F003	25	Z distribution	-0.00355	0.017601	0.132112
GJA1	W2	PGE2	well_2	F004	20	Z distribution	-0.15992	-0.17643	0.359114
GJA1	W2	PGE2	well_2	F005	50	Z distribution	0.120955	0.103868	0.361876
GJA1	W2	PGE2	well_2	F006	12	Z distribution	-0.00632	0.004558	0.119584
GJA1	W2	PGE2	well_3	F001	28	Z distribution	-0.25626	-0.19266	0.292511
GJA1	W2	PGE2	well_3	F002	13	Z distribution	-0.21066	-0.22847	0.130961
GJA1	W2	PGE2	well_3	F003	25	Z distribution	-0.14335	-0.18741	0.191916
GJA1	W2	PGE2	well_3	F004	47	Z distribution	0.192546	0.249973	0.254114
GJA1	W2	PGE2	well_3	F005	85	Z distribution	-0.06829	-0.01173	0.374288
GJA1	W2	PGE2	well_3	F006	24	Z distribution	-0.10339	-0.11868	0.218643
GJA1	W2	PGE2	well_4	F001	6	Z distribution	-0.21591	-0.24073	0.160976
GJA1	W2	PGE2	well_4	F002	25	Z distribution	-0.04283	-0.09524	0.188618
GJA1	W2	PGE2	well_4	F003	50	Z distribution	-0.03185	-0.02226	0.185363
GJA1	W2	PGE2	well_4	F004	7	Z distribution	-0.04395	-0.03336	0.204016
GJA1	W2	PGE2	well_4	F005	24	Z distribution	-0.01953	-0.00121	0.196403
GJA1	W2	PGE2	well_4	F006	40	Z distribution	0.187475	0.095262	0.422884
GJA1	W2	PGE2	well_5	F001	59	Z distribution	0.126664	0.077871	0.268347
GJA1	W2	PGE2	well_5	F002	18	Z distribution	-0.07113	-0.04004	0.104753
GJA1	W2	PGE2	well_5	F003	19	Z distribution	0.05121	0.07251	0.222371
GJA1	W2	PGE2	well_5	F004	6	Z distribution	0.107038	0.084825	0.063873
GJA1	W2	PGE2	well_5	F005	18	Z distribution	-0.05523	-0.07055	0.256496
GJA1	W2	PGE2	well_5	F006	18	Z distribution	0.154865	0.173429	0.207322
GJA1	W3	HPI4	well_1	F001	1	Z distribution	-0.0843	-0.0843	NA
GJA1	W3	HPI4	well_1	F003	4	Z distribution	-0.34642	-0.34642	0.174398
GJA1	W3	HPI4	well_1	F005	6	Z distribution	-0.14797	-0.14412	0.081939
GJA1	W3	HPI4	well_1	F006	5	Z distribution	-0.24398	-0.24651	0.112821
GJA1	W3	HPI4	well_2	F001	5	Z distribution	-0.39167	-0.23646	0.270135
GJA1	W3	HPI4	well_2	F002	4	Z distribution	-0.05284	-0.05284	0.008033
GJA1	W3	HPI4	well_2	F004	11	Z distribution	0.052053	0.053118	0.192278
GJA1	W3	HPI4	well_2	F005	3	Z distribution	-0.68561	-0.68561	NA
GJA1	W3	HPI4	well_2	F006	8	Z distribution	-0.47905	-0.44516	0.248314
GJA1	W3	HPI4	well_3	F001	4	Z distribution	-0.18661	-0.18661	0.037073
GJA1	W3	HPI4	well_3	F002	8	Z distribution	-0.36726	-0.26058	0.305719
GJA1	W3	HPI4	well_3	F003	4	Z distribution	-0.0162	-0.0162	0.008541
GJA1	W3	HPI4	well_3	F004	4	Z distribution	-0.40583	-0.40583	0.003621
GJA1	W3	HPI4	well_3	F005	9	Z distribution	-0.13196	-0.13146	0.131402
GJA1	W3	HPI4	well_3	F006	1	Z distribution	-0.06521	-0.06521	NA
GJA1	W3	HPI4	well_4	F001	4	Z distribution	-0.18817	-0.18817	0.303007
GJA1	W3	HPI4	well_4	F002	1	Z distribution	-0.05709	-0.05709	NA
GJA1	W3	HPI4	well_4	F004	4	Z distribution	0.062276	0.062276	0.100459
GJA1	W3	HPI4	well_4	F005	1	Z distribution	-0.90288	-0.90288	NA
GJA1	W3	HPI4	well_4	F006	4	Z distribution	-0.28056	-0.28056	0.247324
GJA1	W3	HPI4	well_5	F001	4	Z distribution	-0.10764	-0.10764	0.083457
GJA1	W3	HPI4	well_5	F002	19	Z distribution	-0.27212	-0.30331	0.228342
GJA1	W3	HPI4	well_5	F004	16	Z distribution	-0.35397	-0.30897	0.387351
GJA1	W3	HPI4	well_5	F005	14	Z distribution	-0.20536	-0.19752	0.14937
GJA1	W3	HPI4	well_5	F006	16	Z distribution	-0.10052	-0.08117	0.256541
GJA1	W3	PGE2	well_1	F001	14	Z distribution	-0.13005	-0.05281	0.362084
GJA1	W3	PGE2	well_1	F002	198	Z distribution	-0.34948	-0.20997	0.884932

GJA1	W3	PGE2	well_1	F003	188	Z distribution	0.068676	0.106963	0.416816
GJA1	W3	PGE2	well_1	F004	100	Z distribution	0.060843	0.091301	0.365173
GJA1	W3	PGE2	well_1	F005	99	Z distribution	0.030083	0.165967	0.736113
GJA1	W3	PGE2	well_1	F006	321	Z distribution	0.495358	0.451776	0.411434
GJA1	W3	PGE2	well_2	F001	189	Z distribution	0.252054	0.228909	0.282512
GJA1	W3	PGE2	well_2	F002	201	Z distribution	-0.37197	-0.24192	0.599393
GJA1	W3	PGE2	well_2	F003	252	Z distribution	0.109064	0.15155	0.37039
GJA1	W3	PGE2	well_2	F004	273	Z distribution	0.130673	0.162	0.43832
GJA1	W3	PGE2	well_2	F005	270	Z distribution	0.188121	0.206811	0.426575
GJA1	W3	PGE2	well_2	F006	199	Z distribution	0.024365	0.075463	0.434026
GJA1	W3	PGE2	well_3	F001	151	Z distribution	-0.01967	0.062217	0.45175
GJA1	W3	PGE2	well_3	F002	177	Z distribution	-0.57798	-0.43366	0.793846
GJA1	W3	PGE2	well_3	F003	95	Z distribution	-0.62845	-0.53084	0.758178
GJA1	W3	PGE2	well_3	F004	184	Z distribution	-0.58482	-0.52982	0.556258
GJA1	W3	PGE2	well_3	F005	111	Z distribution	-0.54411	-0.37828	0.804931
GJA1	W3	PGE2	well_3	F006	164	Z distribution	0.035778	0.088032	0.331472
GJA1	W3	PGE2	well_4	F001	190	Z distribution	0.167313	0.187397	0.384983
GJA1	W3	PGE2	well_4	F002	170	Z distribution	0.278473	0.247506	0.321214
GJA1	W3	PGE2	well_4	F003	89	Z distribution	-0.37331	-0.20546	0.562513
GJA1	W3	PGE2	well_4	F004	179	Z distribution	-0.29183	-0.27381	0.437567
GJA1	W3	PGE2	well_4	F005	304	Z distribution	0.22136	0.241407	0.510666
GJA1	W3	PGE2	well_4	F006	248	Z distribution	-0.06506	-0.04198	0.376131
GJA1	W3	PGE2	well_5	F001	198	Z distribution	-0.27102	-0.23776	0.315282
GJA1	W3	PGE2	well_5	F002	208	Z distribution	-0.23105	-0.18325	0.334229
GJA1	W3	PGE2	well_5	F003	150	Z distribution	-0.32511	-0.26659	0.31158
GJA1	W3	PGE2	well_5	F004	70	Z distribution	-0.52672	-0.45372	0.427587
GJA1	W3	PGE2	well_5	F005	97	Z distribution	-0.35647	-0.19236	0.603997
GJA1	W3	PGE2	well_5	F006	111	Z distribution	-0.03518	-0.00136	0.337477
GJA1	W4	HPI4	well_1	F001	7	Z distribution	-0.0481	-0.0002	0.120425
GJA1	W4	HPI4	well_1	F002	16	Z distribution	-0.04456	-0.01416	0.196843
GJA1	W4	HPI4	well_1	F003	37	Z distribution	-0.02623	-0.01407	0.15753
GJA1	W4	HPI4	well_1	F004	28	Z distribution	-0.09855	0.006564	0.280186
GJA1	W4	HPI4	well_1	F005	8	Z distribution	0.046318	0.04423	0.112243
GJA1	W4	HPI4	well_1	F006	17	Z distribution	-0.25966	-0.30185	0.192231
GJA1	W4	HPI4	well_2	F001	9	Z distribution	-0.05712	-0.01289	0.109396
GJA1	W4	HPI4	well_2	F002	12	Z distribution	-0.3114	-0.1739	0.319553
GJA1	W4	HPI4	well_2	F003	24	Z distribution	-0.10247	-0.10999	0.294032
GJA1	W4	HPI4	well_2	F004	44	Z distribution	-0.16619	-0.07359	0.369078
GJA1	W4	HPI4	well_2	F005	15	Z distribution	-0.00143	-0.00021	0.147041
GJA1	W4	HPI4	well_2	F006	3	Z distribution	-0.13609	-0.13609	NA
GJA1	W4	HPI4	well_3	F001	9	Z distribution	-0.1675	-0.18256	0.1139
GJA1	W4	HPI4	well_3	F002	8	Z distribution	-0.11161	-0.0931	0.154254
GJA1	W4	HPI4	well_3	F003	15	Z distribution	-0.31109	-0.31413	0.324317
GJA1	W4	HPI4	well_3	F004	13	Z distribution	-0.30621	-0.13701	0.358832
GJA1	W4	HPI4	well_3	F005	5	Z distribution	0.021409	0.030999	0.030663
GJA1	W4	HPI4	well_3	F006	21	Z distribution	-0.24786	-0.16313	0.283606
GJA1	W4	HPI4	well_4	F002	25	Z distribution	-0.28809	-0.24299	0.238296
GJA1	W4	HPI4	well_4	F003	8	Z distribution	0.141945	0.124838	0.145246
GJA1	W4	HPI4	well_4	F004	25	Z distribution	0.009993	0.02848	0.135783

GJA1	W4	HPI4	well_4	F005	70	Z distribution	-0.05866	-0.05064	0.249465
GJA1	W4	HPI4	well_4	F006	50	Z distribution	0.100179	0.043065	0.279538
GJA1	W4	HPI4	well_5	F001	11	Z distribution	-0.01608	0.189196	0.317901
GJA1	W4	HPI4	well_5	F002	15	Z distribution	0.012476	0.035273	0.177301
GJA1	W4	HPI4	well_5	F003	17	Z distribution	0.041838	0.041328	0.199891
GJA1	W4	HPI4	well_5	F004	29	Z distribution	-0.01959	-0.02099	0.208332
GJA1	W4	HPI4	well_5	F005	91	Z distribution	-0.01978	-0.02081	0.154861
GJA1	W4	HPI4	well_5	F006	10	Z distribution	0.054362	0.060173	0.217853
GJA1	W4	PGE2	well_1	F001	43	Z distribution	-0.38387	-0.3542	0.222737
GJA1	W4	PGE2	well_1	F002	113	Z distribution	-0.16387	-0.0567	0.47246
GJA1	W4	PGE2	well_1	F003	306	Z distribution	0.411224	0.447855	0.729824
GJA1	W4	PGE2	well_1	F004	34	Z distribution	-0.01161	-0.03647	0.242722
GJA1	W4	PGE2	well_1	F005	55	Z distribution	-0.11401	0.010642	0.603143
GJA1	W4	PGE2	well_1	F006	329	Z distribution	0.138887	0.201123	0.626295
GJA1	W4	PGE2	well_2	F001	200	Z distribution	0.202496	0.195093	0.449775
GJA1	W4	PGE2	well_2	F002	159	Z distribution	-0.02561	0.002846	0.431831
GJA1	W4	PGE2	well_2	F003	277	Z distribution	-0.25429	-0.20909	0.580395
GJA1	W4	PGE2	well_2	F004	252	Z distribution	0.190336	0.236696	0.63553
GJA1	W4	PGE2	well_2	F005	168	Z distribution	-0.08644	0.090309	0.870187
GJA1	W4	PGE2	well_2	F006	175	Z distribution	-0.10528	-0.09328	0.370925
GJA1	W4	PGE2	well_3	F001	85	Z distribution	-0.33004	-0.34441	0.359804
GJA1	W4	PGE2	well_3	F002	306	Z distribution	0.101891	0.144472	0.579921
GJA1	W4	PGE2	well_3	F003	220	Z distribution	-0.22238	-0.19466	0.479929
GJA1	W4	PGE2	well_3	F004	40	Z distribution	-0.18722	-0.17702	0.614404
GJA1	W4	PGE2	well_3	F005	19	Z distribution	-0.3295	-0.17293	0.835065
GJA1	W4	PGE2	well_3	F006	144	Z distribution	-0.17497	-0.0723	0.586668
GJA1	W4	PGE2	well_4	F001	100	Z distribution	-0.25498	-0.16661	0.411376
GJA1	W4	PGE2	well_4	F002	72	Z distribution	-0.1484	-0.08133	0.550282
GJA1	W4	PGE2	well_4	F003	154	Z distribution	-0.10818	0.020744	0.642787
GJA1	W4	PGE2	well_4	F004	292	Z distribution	0.082098	0.111337	0.484338
GJA1	W4	PGE2	well_4	F005	212	Z distribution	-0.18817	-0.15844	0.407614
GJA1	W4	PGE2	well_4	F006	60	Z distribution	0.002669	0.025023	0.365272
GJA1	W4	PGE2	well_5	F001	197	Z distribution	-0.32154	-0.27576	0.461017
GJA1	W4	PGE2	well_5	F002	177	Z distribution	0.136022	0.111411	0.372973
GJA1	W4	PGE2	well_5	F003	124	Z distribution	-0.31881	-0.1975	0.589816
GJA1	W4	PGE2	well_5	F004	124	Z distribution	0.004006	0.004389	0.348751
GJA1	W4	PGE2	well_5	F005	91	Z distribution	-0.26135	-0.16321	0.491744
GJA1	W4	PGE2	well_5	F006	97	Z distribution	-0.18216	-0.16094	0.396967
TJP1	W1	HPI4	well_1	F001	316	Avg. Volume	1.169469	1.029578	0.75689
TJP1	W1	HPI4	well_1	F003	262	Avg. Volume	1.085756	0.910432	0.719392
TJP1	W1	HPI4	well_1	F004	176	Avg. Volume	1.582504	1.389676	0.904219
TJP1	W1	HPI4	well_1	F005	276	Avg. Volume	0.922221	0.775453	0.629683
TJP1	W1	HPI4	well_1	F006	288	Avg. Volume	1.200646	1.039065	0.751412
TJP1	W1	HPI4	well_2	F001	70	Avg. Volume	0.825231	0.638005	0.641606
TJP1	W1	HPI4	well_2	F002	239	Avg. Volume	1.399359	1.354769	0.607333
TJP1	W1	HPI4	well_2	F003	181	Avg. Volume	1.185302	1.104661	0.659108
TJP1	W1	HPI4	well_2	F004	255	Avg. Volume	1.085141	0.924952	0.660592
TJP1	W1	HPI4	well_2	F005	202	Avg. Volume	0.677024	0.574026	0.452257
TJP1	W1	HPI4	well_2	F006	394	Avg. Volume	1.019729	0.856538	0.742653

TJP1	W1	HPI4	well_3	F003	196	Avg. Volume	0.88946	0.725854	0.610701
TJP1	W1	HPI4	well_3	F004	283	Avg. Volume	1.373883	1.189259	0.837675
TJP1	W1	HPI4	well_3	F005	288	Avg. Volume	1.144624	0.980368	0.776493
TJP1	W1	HPI4	well_3	F006	371	Avg. Volume	1.222828	1.082852	0.790563
TJP1	W1	HPI4	well_4	F001	280	Avg. Volume	1.455263	1.313816	0.909471
TJP1	W1	HPI4	well_4	F002	250	Avg. Volume	1.420889	1.210386	0.977117
TJP1	W1	HPI4	well_4	F003	254	Avg. Volume	1.282907	1.125971	0.810913
TJP1	W1	HPI4	well_4	F004	205	Avg. Volume	1.137448	0.92232	0.718266
TJP1	W1	HPI4	well_4	F005	363	Avg. Volume	1.13689	1.056715	0.665255
TJP1	W1	HPI4	well_4	F006	330	Avg. Volume	1.295852	1.105585	0.897156
TJP1	W1	HPI4	well_5	F001	283	Avg. Volume	1.283249	1.09491	0.90173
TJP1	W1	HPI4	well_5	F002	150	Avg. Volume	1.409544	1.051587	1.106165
TJP1	W1	HPI4	well_5	F003	200	Avg. Volume	1.259902	1.129209	0.820081
TJP1	W1	HPI4	well_5	F004	189	Avg. Volume	1.103135	0.955026	0.738577
TJP1	W1	HPI4	well_5	F005	260	Avg. Volume	1.268044	1.114815	0.850358
TJP1	W1	HPI4	well_5	F006	227	Avg. Volume	1.072979	0.996102	0.640198
TJP1	W1	PGE2	well_1	F001	345	Avg. Volume	1.447401	1.318356	0.879434
TJP1	W1	PGE2	well_1	F002	238	Avg. Volume	1.136411	0.99561	0.68991
TJP1	W1	PGE2	well_1	F003	317	Avg. Volume	1.102665	0.976444	0.688633
TJP1	W1	PGE2	well_1	F004	257	Avg. Volume	1.152973	1.032778	0.714759
TJP1	W1	PGE2	well_1	F005	397	Avg. Volume	1.296826	1.114931	0.886985
TJP1	W1	PGE2	well_1	F006	464	Avg. Volume	1.080514	0.945246	0.667209
TJP1	W1	PGE2	well_2	F004	278	Avg. Volume	1.239643	1.129705	0.741034
TJP1	W1	PGE2	well_2	F005	237	Avg. Volume	1.324877	1.177693	0.756666
TJP1	W1	PGE2	well_3	F001	204	Avg. Volume	1.429107	1.255532	0.783203
TJP1	W1	PGE2	well_3	F002	175	Avg. Volume	1.139875	1.025414	0.674666
TJP1	W1	PGE2	well_3	F003	293	Avg. Volume	1.482502	1.282904	0.856856
TJP1	W1	PGE2	well_3	F004	232	Avg. Volume	1.450491	1.322413	0.915185
TJP1	W1	PGE2	well_3	F005	357	Avg. Volume	1.146035	1.042611	0.719455
TJP1	W1	PGE2	well_3	F006	301	Avg. Volume	1.097948	0.938155	0.737512
TJP1	W1	PGE2	well_4	F001	220	Avg. Volume	1.650844	1.4528	1.052437
TJP1	W1	PGE2	well_4	F003	186	Avg. Volume	1.102254	0.991049	0.691535
TJP1	W1	PGE2	well_4	F004	249	Avg. Volume	0.97888	0.922859	0.483548
TJP1	W1	PGE2	well_4	F005	281	Avg. Volume	1.23024	1.072345	0.877706
TJP1	W1	PGE2	well_4	F006	327	Avg. Volume	1.235618	1.0982	0.872444
TJP1	W1	PGE2	well_5	F001	296	Avg. Volume	1.134551	0.999995	0.653824
TJP1	W1	PGE2	well_5	F002	377	Avg. Volume	1.284559	1.165503	0.849928
TJP1	W1	PGE2	well_5	F003	286	Avg. Volume	0.951285	0.799436	0.665394
TJP1	W1	PGE2	well_5	F004	247	Avg. Volume	1.458159	1.229777	0.777014
TJP1	W1	PGE2	well_5	F005	294	Avg. Volume	1.325659	1.185906	0.890307
TJP1	W1	PGE2	well_5	F006	269	Avg. Volume	1.216091	1.065435	0.752543
TJP1	W2	HPI4	well_1	F001	266	Avg. Volume	0.910311	0.637103	0.723713
TJP1	W2	HPI4	well_1	F002	322	Avg. Volume	1.251277	1.031742	1.037005
TJP1	W2	HPI4	well_1	F003	270	Avg. Volume	0.671673	0.454043	0.560874
TJP1	W2	HPI4	well_1	F004	354	Avg. Volume	0.975802	0.62283	0.914931
TJP1	W2	HPI4	well_1	F005	220	Avg. Volume	0.773222	0.609463	0.525021
TJP1	W2	HPI4	well_1	F006	204	Avg. Volume	1.380469	1.213863	0.972878
TJP1	W2	HPI4	well_2	F001	286	Avg. Volume	1.995777	1.415819	1.856238
TJP1	W2	HPI4	well_2	F002	208	Avg. Volume	1.369764	1.197071	0.94673

TJP1	W2	HPI4	well_2	F003	234	Avg. Volume	0.931121	0.732073	0.66081
TJP1	W2	HPI4	well_2	F004	436	Avg. Volume	0.978845	0.79468	0.786999
TJP1	W2	HPI4	well_2	F005	393	Avg. Volume	0.959996	0.791908	0.750279
TJP1	W2	HPI4	well_2	F006	322	Avg. Volume	0.946612	0.811099	0.68348
TJP1	W2	HPI4	well_3	F001	359	Avg. Volume	1.079766	0.838096	0.930346
TJP1	W2	HPI4	well_3	F002	306	Avg. Volume	1.243827	1.033212	0.984856
TJP1	W2	HPI4	well_3	F003	227	Avg. Volume	0.866146	0.711991	0.561664
TJP1	W2	HPI4	well_3	F004	270	Avg. Volume	0.928491	0.787884	0.623814
TJP1	W2	HPI4	well_3	F005	288	Avg. Volume	0.950031	0.649398	0.852168
TJP1	W2	HPI4	well_3	F006	294	Avg. Volume	1.274827	0.994229	0.976687
TJP1	W2	HPI4	well_4	F001	332	Avg. Volume	1.460289	1.248396	1.135361
TJP1	W2	HPI4	well_4	F002	221	Avg. Volume	0.701731	0.477073	0.628679
TJP1	W2	HPI4	well_4	F003	390	Avg. Volume	0.935325	0.687422	0.745527
TJP1	W2	HPI4	well_4	F004	306	Avg. Volume	0.701209	0.53057	0.515234
TJP1	W2	HPI4	well_4	F005	328	Avg. Volume	0.920308	0.804253	0.591584
TJP1	W2	HPI4	well_4	F006	307	Avg. Volume	0.894355	0.725292	0.696151
TJP1	W2	HPI4	well_5	F001	338	Avg. Volume	1.373345	1.192126	0.978938
TJP1	W2	HPI4	well_5	F002	286	Avg. Volume	1.004243	0.827896	0.736227
TJP1	W2	HPI4	well_5	F003	265	Avg. Volume	1.084201	0.950625	0.691045
TJP1	W2	HPI4	well_5	F004	336	Avg. Volume	1.004211	0.772165	0.778007
TJP1	W2	HPI4	well_5	F005	356	Avg. Volume	1.26558	1.061192	0.950218
TJP1	W2	HPI4	well_5	F006	358	Avg. Volume	1.385291	1.163034	1.153037
TJP1	W2	PGE2	well_1	F001	368	Avg. Volume	1.399069	1.04949	1.080602
TJP1	W2	PGE2	well_1	F002	217	Avg. Volume	1.581933	1.227262	1.211487
TJP1	W2	PGE2	well_1	F003	424	Avg. Volume	1.260525	0.9997	0.953722
TJP1	W2	PGE2	well_1	F004	325	Avg. Volume	1.361369	1.17417	0.913282
TJP1	W2	PGE2	well_1	F005	224	Avg. Volume	1.091191	0.963715	0.742873
TJP1	W2	PGE2	well_1	F006	427	Avg. Volume	1.269411	0.989187	1.064024
TJP1	W2	PGE2	well_2	F001	331	Avg. Volume	1.462647	1.190998	1.079899
TJP1	W2	PGE2	well_2	F002	534	Avg. Volume	1.487926	1.117678	1.293977
TJP1	W2	PGE2	well_2	F003	398	Avg. Volume	1.368194	1.016478	1.086069
TJP1	W2	PGE2	well_2	F004	461	Avg. Volume	0.997744	0.837588	0.644059
TJP1	W2	PGE2	well_2	F005	388	Avg. Volume	1.188223	0.995764	0.842718
TJP1	W2	PGE2	well_2	F006	310	Avg. Volume	1.297602	1.023576	0.954809
TJP1	W2	PGE2	well_3	F001	93	Avg. Volume	1.467026	1.210496	0.889413
TJP1	W2	PGE2	well_3	F002	478	Avg. Volume	1.314146	0.946439	1.2447
TJP1	W2	PGE2	well_3	F003	491	Avg. Volume	1.459759	1.132986	1.219914
TJP1	W2	PGE2	well_3	F004	550	Avg. Volume	0.987384	0.748151	0.819488
TJP1	W2	PGE2	well_3	F005	458	Avg. Volume	1.485029	0.994369	1.469459
TJP1	W2	PGE2	well_3	F006	38	Avg. Volume	0.456438	0.3765	0.392432
TJP1	W2	PGE2	well_4	F001	313	Avg. Volume	1.50773	1.136379	1.187087
TJP1	W2	PGE2	well_4	F002	455	Avg. Volume	2.375625	1.857566	1.775335
TJP1	W2	PGE2	well_4	F003	445	Avg. Volume	1.592971	1.200213	1.347558
TJP1	W2	PGE2	well_4	F004	329	Avg. Volume	1.406712	1.101238	1.132085
TJP1	W2	PGE2	well_4	F005	375	Avg. Volume	1.272738	1.031101	0.926753
TJP1	W2	PGE2	well_4	F006	41	Avg. Volume	0.696867	0.506458	0.507379
TJP1	W2	PGE2	well_5	F001	518	Avg. Volume	1.626403	1.242072	1.390219
TJP1	W2	PGE2	well_5	F002	406	Avg. Volume	1.102202	0.907563	0.790016
TJP1	W2	PGE2	well_5	F003	326	Avg. Volume	1.032052	0.715932	0.936454

TJP1	W2	PGE2	well_5	F004	392	Avg. Volume	1.566258	1.240985	1.209257
TJP1	W2	PGE2	well_5	F005	397	Avg. Volume	1.5455	1.163049	1.255925
TJP1	W2	PGE2	well_5	F006	471	Avg. Volume	1.633242	1.257835	1.377306
TJP1	W3	HPI4	well_1	F001	48	Avg. Volume	2.150638	1.861962	1.320902
TJP1	W3	HPI4	well_1	F002	74	Avg. Volume	1.57733	1.356155	0.951872
TJP1	W3	HPI4	well_1	F003	81	Avg. Volume	2.473025	1.688194	2.074958
TJP1	W3	HPI4	well_1	F004	127	Avg. Volume	2.074567	1.408892	1.800483
TJP1	W3	HPI4	well_1	F005	74	Avg. Volume	1.492238	1.000084	1.215936
TJP1	W3	HPI4	well_1	F006	66	Avg. Volume	1.745408	1.420599	1.105577
TJP1	W3	HPI4	well_2	F001	74	Avg. Volume	1.588636	1.397413	1.098826
TJP1	W3	HPI4	well_2	F002	183	Avg. Volume	1.053836	0.698043	0.924248
TJP1	W3	HPI4	well_2	F003	105	Avg. Volume	1.65343	1.384861	1.168198
TJP1	W3	HPI4	well_2	F004	154	Avg. Volume	1.466257	1.118825	1.233984
TJP1	W3	HPI4	well_2	F005	200	Avg. Volume	0.955292	0.698992	0.771626
TJP1	W3	HPI4	well_2	F006	127	Avg. Volume	1.369318	1.192227	1.022426
TJP1	W3	HPI4	well_3	F001	88	Avg. Volume	2.228627	1.994042	1.317558
TJP1	W3	HPI4	well_3	F002	154	Avg. Volume	2.152392	1.826417	1.56456
TJP1	W3	HPI4	well_3	F003	74	Avg. Volume	1.430904	1.19312	1.048471
TJP1	W3	HPI4	well_3	F004	181	Avg. Volume	0.900855	0.654288	0.760297
TJP1	W3	HPI4	well_3	F005	158	Avg. Volume	1.450478	1.308001	0.91439
TJP1	W3	HPI4	well_3	F006	179	Avg. Volume	1.334566	1.007428	1.048747
TJP1	W3	HPI4	well_4	F001	94	Avg. Volume	2.277403	1.847231	1.599353
TJP1	W3	HPI4	well_4	F002	198	Avg. Volume	1.393184	1.205925	0.966836
TJP1	W3	HPI4	well_4	F003	141	Avg. Volume	1.560177	1.212487	1.088805
TJP1	W3	HPI4	well_4	F004	62	Avg. Volume	1.600473	1.196791	1.291356
TJP1	W3	HPI4	well_4	F005	161	Avg. Volume	2.105906	1.938973	1.280399
TJP1	W3	HPI4	well_4	F006	151	Avg. Volume	1.710107	1.452516	1.140609
TJP1	W3	HPI4	well_5	F001	47	Avg. Volume	1.823088	1.615182	1.380428
TJP1	W3	HPI4	well_5	F002	145	Avg. Volume	2.006911	1.503092	1.60522
TJP1	W3	HPI4	well_5	F003	187	Avg. Volume	1.525631	1.01224	1.490723
TJP1	W3	HPI4	well_5	F004	176	Avg. Volume	2.258122	1.582636	2.085733
TJP1	W3	HPI4	well_5	F005	190	Avg. Volume	1.474629	1.263029	1.178386
TJP1	W3	HPI4	well_5	F006	192	Avg. Volume	2.11183	1.744999	1.679128
TJP1	W3	PGE2	well_1	F001	390	Avg. Volume	1.91042	1.385448	1.584129
TJP1	W3	PGE2	well_1	F002	404	Avg. Volume	2.651041	1.813574	2.555477
TJP1	W3	PGE2	well_1	F003	375	Avg. Volume	2.145992	1.440608	1.948963
TJP1	W3	PGE2	well_1	F004	417	Avg. Volume	2.050035	1.436747	1.788371
TJP1	W3	PGE2	well_1	F005	358	Avg. Volume	2.075141	1.426022	1.96513
TJP1	W3	PGE2	well_1	F006	223	Avg. Volume	2.821425	2.020567	2.530157
TJP1	W3	PGE2	well_2	F001	211	Avg. Volume	2.18793	1.785628	1.440315
TJP1	W3	PGE2	well_2	F002	366	Avg. Volume	2.226559	1.248103	2.31468
TJP1	W3	PGE2	well_2	F003	284	Avg. Volume	4.307192	2.566296	4.603272
TJP1	W3	PGE2	well_2	F004	413	Avg. Volume	2.256807	1.542647	2.075257
TJP1	W3	PGE2	well_2	F005	350	Avg. Volume	1.653122	1.253123	1.353488
TJP1	W3	PGE2	well_2	F006	289	Avg. Volume	3.485627	2.015258	3.372154
TJP1	W3	PGE2	well_3	F001	426	Avg. Volume	2.541426	1.71722	2.330481
TJP1	W3	PGE2	well_3	F002	388	Avg. Volume	1.731699	1.271869	1.486652
TJP1	W3	PGE2	well_3	F003	391	Avg. Volume	2.440893	1.598067	2.324719
TJP1	W3	PGE2	well_3	F004	330	Avg. Volume	2.258798	1.605485	1.988536

TJP1	W3	PGE2	well_3	F005	468	Avg. Volume	3.464868	2.384493	3.272295
TJP1	W3	PGE2	well_3	F006	185	Avg. Volume	4.910181	3.095399	4.81756
TJP1	W3	PGE2	well_4	F001	250	Avg. Volume	2.333508	1.69652	1.991719
TJP1	W3	PGE2	well_4	F002	418	Avg. Volume	2.095498	1.561949	1.572101
TJP1	W3	PGE2	well_4	F003	218	Avg. Volume	2.894068	1.870652	2.676423
TJP1	W3	PGE2	well_4	F004	444	Avg. Volume	1.498929	1.177034	1.107876
TJP1	W3	PGE2	well_4	F005	419	Avg. Volume	1.94092	1.386426	1.69874
TJP1	W3	PGE2	well_4	F006	226	Avg. Volume	3.911375	2.622238	3.687359
TJP1	W3	PGE2	well_5	F001	333	Avg. Volume	2.058706	1.295667	2.027727
TJP1	W3	PGE2	well_5	F002	314	Avg. Volume	2.05328	1.451953	1.967398
TJP1	W3	PGE2	well_5	F003	345	Avg. Volume	2.620902	1.873248	2.396885
TJP1	W3	PGE2	well_5	F004	289	Avg. Volume	1.60054	1.274404	1.196202
TJP1	W3	PGE2	well_5	F005	414	Avg. Volume	2.01968	1.310018	1.949519
TJP1	W3	PGE2	well_5	F006	141	Avg. Volume	2.914912	1.985208	2.20298
TJP1	W4	HPI4	well_1	F001	89	Avg. Volume	1.558689	1.193076	1.341393
TJP1	W4	HPI4	well_1	F002	154	Avg. Volume	2.042214	1.764644	1.464123
TJP1	W4	HPI4	well_1	F003	42	Avg. Volume	1.223585	0.995723	0.921309
TJP1	W4	HPI4	well_1	F004	191	Avg. Volume	1.379076	1.052203	1.148471
TJP1	W4	HPI4	well_1	F005	125	Avg. Volume	1.085235	0.73373	0.894956
TJP1	W4	HPI4	well_1	F006	194	Avg. Volume	0.701153	0.515718	0.572298
TJP1	W4	HPI4	well_2	F001	162	Avg. Volume	1.881609	1.710513	1.341797
TJP1	W4	HPI4	well_2	F002	145	Avg. Volume	2.20475	1.675556	1.83968
TJP1	W4	HPI4	well_2	F003	133	Avg. Volume	1.195281	1.064785	0.832883
TJP1	W4	HPI4	well_2	F004	111	Avg. Volume	1.25356	1.048156	0.921787
TJP1	W4	HPI4	well_2	F005	128	Avg. Volume	1.202115	0.98825	0.91416
TJP1	W4	HPI4	well_2	F006	136	Avg. Volume	1.740624	1.462366	1.267363
TJP1	W4	HPI4	well_3	F001	195	Avg. Volume	2.070897	1.676091	1.626052
TJP1	W4	HPI4	well_3	F002	183	Avg. Volume	1.552499	1.091458	1.353003
TJP1	W4	HPI4	well_3	F003	174	Avg. Volume	1.541409	1.072048	1.371337
TJP1	W4	HPI4	well_3	F004	182	Avg. Volume	1.592833	1.294467	1.212614
TJP1	W4	HPI4	well_3	F005	160	Avg. Volume	2.094777	1.686114	1.672226
TJP1	W4	HPI4	well_3	F006	28	Avg. Volume	1.651588	1.197297	1.483427
TJP1	W4	HPI4	well_4	F001	136	Avg. Volume	1.7895	1.417622	1.540889
TJP1	W4	HPI4	well_4	F002	121	Avg. Volume	1.537687	1.176849	1.254986
TJP1	W4	HPI4	well_4	F003	115	Avg. Volume	1.312727	1.009827	0.944201
TJP1	W4	HPI4	well_4	F004	187	Avg. Volume	2.070575	1.590498	1.766001
TJP1	W4	HPI4	well_4	F005	230	Avg. Volume	1.024768	0.759859	0.862535
TJP1	W4	HPI4	well_4	F006	103	Avg. Volume	0.810054	0.632073	0.586816
TJP1	W4	HPI4	well_5	F001	174	Avg. Volume	1.615317	1.336713	1.253839
TJP1	W4	HPI4	well_5	F002	136	Avg. Volume	0.665918	0.464228	0.55862
TJP1	W4	HPI4	well_5	F003	27	Avg. Volume	0.824548	0.526169	0.642766
TJP1	W4	HPI4	well_5	F004	197	Avg. Volume	1.388119	1.11996	1.061757
TJP1	W4	HPI4	well_5	F005	190	Avg. Volume	1.815946	1.656105	1.394529
TJP1	W4	HPI4	well_5	F006	232	Avg. Volume	1.364199	0.959465	1.186835
TJP1	W4	PGE2	well_1	F001	174	Avg. Volume	2.361304	1.755952	1.914595
TJP1	W4	PGE2	well_1	F002	117	Avg. Volume	3.066484	1.709205	3.454479
TJP1	W4	PGE2	well_1	F003	66	Avg. Volume	2.601999	2.103049	1.67278
TJP1	W4	PGE2	well_1	F004	238	Avg. Volume	1.506593	1.101238	1.306685
TJP1	W4	PGE2	well_1	F005	389	Avg. Volume	2.105148	1.480706	2.013985

TJP1	W4	PGE2	well_1	F006	259	Avg. Volume	3.594326	2.387765	3.297474
TJP1	W4	PGE2	well_2	F001	129	Avg. Volume	0.638884	0.481963	0.556637
TJP1	W4	PGE2	well_2	F002	174	Avg. Volume	1.079279	0.794563	0.883772
TJP1	W4	PGE2	well_2	F003	190	Avg. Volume	2.341851	1.956019	1.705607
TJP1	W4	PGE2	well_2	F004	279	Avg. Volume	2.533907	1.368857	2.763845
TJP1	W4	PGE2	well_2	F005	366	Avg. Volume	1.629215	1.002733	1.563192
TJP1	W4	PGE2	well_2	F006	257	Avg. Volume	1.887342	1.191215	2.193958
TJP1	W4	PGE2	well_3	F001	63	Avg. Volume	4.277713	2.292454	4.57045
TJP1	W4	PGE2	well_3	F002	418	Avg. Volume	3.644218	2.118729	3.864175
TJP1	W4	PGE2	well_3	F003	344	Avg. Volume	2.374887	1.416157	2.304353
TJP1	W4	PGE2	well_3	F004	364	Avg. Volume	1.734413	1.078487	1.702729
TJP1	W4	PGE2	well_3	F005	149	Avg. Volume	1.675024	1.292439	1.330797
TJP1	W4	PGE2	well_3	F006	333	Avg. Volume	3.363676	1.953224	3.617466
TJP1	W4	PGE2	well_4	F001	295	Avg. Volume	1.325503	1.001481	1.009355
TJP1	W4	PGE2	well_4	F002	280	Avg. Volume	1.824548	1.16411	1.916397
TJP1	W4	PGE2	well_4	F003	189	Avg. Volume	1.579377	1.189259	1.457693
TJP1	W4	PGE2	well_4	F004	198	Avg. Volume	3.641865	2.005204	3.998202
TJP1	W4	PGE2	well_4	F005	273	Avg. Volume	1.676304	1.05625	1.673874
TJP1	W4	PGE2	well_4	F006	209	Avg. Volume	2.085112	1.613063	1.628457
TJP1	W4	PGE2	well_5	F001	295	Avg. Volume	1.89536	1.275324	1.850673
TJP1	W4	PGE2	well_5	F002	185	Avg. Volume	2.93073	1.918854	2.577409
TJP1	W4	PGE2	well_5	F003	303	Avg. Volume	2.851737	1.895165	2.76678
TJP1	W4	PGE2	well_5	F004	319	Avg. Volume	1.96051	1.377639	1.728624
TJP1	W4	PGE2	well_5	F005	289	Avg. Volume	2.117482	1.538497	1.753248
TJP1	W4	PGE2	well_5	F006	244	Avg. Volume	1.122076	0.874731	0.874355
TJP1	W1	HPI4	well_1	F001	316	Count/cell	28.23448	26	13.96313
TJP1	W1	HPI4	well_1	F003	262	Count/cell	24.03419	23	10.90689
TJP1	W1	HPI4	well_1	F004	176	Count/cell	28.1625	28	13.10283
TJP1	W1	HPI4	well_1	F005	276	Count/cell	26.608	25	13.39747
TJP1	W1	HPI4	well_1	F006	288	Count/cell	27.18321	26	13.9247
TJP1	W1	HPI4	well_2	F001	70	Count/cell	25.65079	23	13.24966
TJP1	W1	HPI4	well_2	F002	239	Count/cell	33.22791	31	14.29722
TJP1	W1	HPI4	well_2	F003	181	Count/cell	30.32515	27	15.70637
TJP1	W1	HPI4	well_2	F004	255	Count/cell	28.06897	26.5	13.45457
TJP1	W1	HPI4	well_2	F005	202	Count/cell	24.25683	22	14.54454
TJP1	W1	HPI4	well_2	F006	394	Count/cell	23.08757	20	13.12916
TJP1	W1	HPI4	well_3	F003	196	Count/cell	26.92697	23.5	15.29522
TJP1	W1	HPI4	well_3	F004	283	Count/cell	25.01167	23	12.71702
TJP1	W1	HPI4	well_3	F005	288	Count/cell	24.2471	23	11.94532
TJP1	W1	HPI4	well_3	F006	371	Count/cell	23.98824	22	13.67188
TJP1	W1	HPI4	well_4	F001	280	Count/cell	21.33597	19	11.12812
TJP1	W1	HPI4	well_4	F002	250	Count/cell	24.36564	23	13.42245
TJP1	W1	HPI4	well_4	F003	254	Count/cell	25.7013	24	14.05307
TJP1	W1	HPI4	well_4	F004	205	Count/cell	29.88587	27	14.91004
TJP1	W1	HPI4	well_4	F005	363	Count/cell	25.58716	23	13.16941
TJP1	W1	HPI4	well_4	F006	330	Count/cell	24.59197	23	12.77164
TJP1	W1	HPI4	well_5	F001	283	Count/cell	24.30435	23	12.17165
TJP1	W1	HPI4	well_5	F002	150	Count/cell	23.66667	22	12.96674
TJP1	W1	HPI4	well_5	F003	200	Count/cell	25.26519	24	13.15617

TJP1	W1	HPI4	well_5	F004	189	Count/cell	26.60355	24	13.2212
TJP1	W1	HPI4	well_5	F005	260	Count/cell	24.62553	21	13.16091
TJP1	W1	HPI4	well_5	F006	227	Count/cell	29.02451	26	15.03835
TJP1	W1	PGE2	well_1	F001	345	Count/cell	24.32051	23	12.27105
TJP1	W1	PGE2	well_1	F002	238	Count/cell	26.67907	24	13.2758
TJP1	W1	PGE2	well_1	F003	317	Count/cell	26.14286	25	13.81511
TJP1	W1	PGE2	well_1	F004	257	Count/cell	25.94444	25	13.35264
TJP1	W1	PGE2	well_1	F005	397	Count/cell	21.90782	20	11.96704
TJP1	W1	PGE2	well_1	F006	464	Count/cell	24.39857	23	11.69063
TJP1	W1	PGE2	well_2	F004	278	Count/cell	26.58167	25	12.40372
TJP1	W1	PGE2	well_2	F005	237	Count/cell	27.64953	27	13.38956
TJP1	W1	PGE2	well_3	F001	204	Count/cell	29.13441	28.5	12.755
TJP1	W1	PGE2	well_3	F002	175	Count/cell	28.78344	27	13.87089
TJP1	W1	PGE2	well_3	F003	293	Count/cell	23.70849	21	12.61421
TJP1	W1	PGE2	well_3	F004	232	Count/cell	22.88038	22	10.70496
TJP1	W1	PGE2	well_3	F005	357	Count/cell	27.39692	25	15.89178
TJP1	W1	PGE2	well_3	F006	301	Count/cell	24.65201	24	12.71433
TJP1	W1	PGE2	well_4	F001	220	Count/cell	22.19095	21	11.729
TJP1	W1	PGE2	well_4	F003	186	Count/cell	23.06627	21	12.00865
TJP1	W1	PGE2	well_4	F004	249	Count/cell	30.31696	31	14.35965
TJP1	W1	PGE2	well_4	F005	281	Count/cell	22.09412	21	11.2456
TJP1	W1	PGE2	well_4	F006	327	Count/cell	21.90169	21	10.8509
TJP1	W1	PGE2	well_5	F001	296	Count/cell	28.12177	25	14.97219
TJP1	W1	PGE2	well_5	F002	377	Count/cell	24.17941	23	12.16626
TJP1	W1	PGE2	well_5	F003	286	Count/cell	23.21509	21	13.28144
TJP1	W1	PGE2	well_5	F004	247	Count/cell	27.4646	26.5	12.29891
TJP1	W1	PGE2	well_5	F005	294	Count/cell	23.65169	22	12.77106
TJP1	W1	PGE2	well_5	F006	269	Count/cell	21.64634	19	11.68467
TJP1	W2	HPI4	well_1	F001	266	Count/cell	20.87815	19.5	11.37943
TJP1	W2	HPI4	well_1	F002	322	Count/cell	21.56463	19	12.58097
TJP1	W2	HPI4	well_1	F003	270	Count/cell	21.62963	19	12.42875
TJP1	W2	HPI4	well_1	F004	354	Count/cell	22.13836	20	11.57664
TJP1	W2	HPI4	well_1	F005	220	Count/cell	25.68159	23	13.86103
TJP1	W2	HPI4	well_1	F006	204	Count/cell	23.03279	22	12.24696
TJP1	W2	HPI4	well_2	F001	286	Count/cell	19.78682	18	11.32218
TJP1	W2	HPI4	well_2	F002	208	Count/cell	29.99462	27.5	14.43887
TJP1	W2	HPI4	well_2	F003	234	Count/cell	26.01887	23	13.03185
TJP1	W2	HPI4	well_2	F004	436	Count/cell	20.92405	19	10.74954
TJP1	W2	HPI4	well_2	F005	393	Count/cell	25.07003	24	12.50969
TJP1	W2	HPI4	well_2	F006	322	Count/cell	23.13149	23	11.57419
TJP1	W2	HPI4	well_3	F001	359	Count/cell	19.75153	18.5	11.00725
TJP1	W2	HPI4	well_3	F002	306	Count/cell	22.8777	21	12.40645
TJP1	W2	HPI4	well_3	F003	227	Count/cell	24.6	23	12.1153
TJP1	W2	HPI4	well_3	F004	270	Count/cell	24.85772	24	13.32912
TJP1	W2	HPI4	well_3	F005	288	Count/cell	20.6391	20	11.25474
TJP1	W2	HPI4	well_3	F006	294	Count/cell	25.03383	23	12.9193
TJP1	W2	HPI4	well_4	F001	332	Count/cell	21.75333	20	11.6133
TJP1	W2	HPI4	well_4	F002	221	Count/cell	20.62871	19	12.51887
TJP1	W2	HPI4	well_4	F003	390	Count/cell	23.32768	22	12.09101

TJP1	W2	HPI4	well_4	F004	306	Count/cell	22.08029	21	11.50509
TJP1	W2	HPI4	well_4	F005	328	Count/cell	29.48333	28	15.31486
TJP1	W2	HPI4	well_4	F006	307	Count/cell	27.14079	25	13.78556
TJP1	W2	HPI4	well_5	F001	338	Count/cell	23.76144	22	12.22462
TJP1	W2	HPI4	well_5	F002	286	Count/cell	24	22	11.50516
TJP1	W2	HPI4	well_5	F003	265	Count/cell	26.74583	25.5	11.90926
TJP1	W2	HPI4	well_5	F004	336	Count/cell	23.58497	21.5	11.89052
TJP1	W2	HPI4	well_5	F005	356	Count/cell	25.76161	24	12.09058
TJP1	W2	HPI4	well_5	F006	358	Count/cell	23.43544	21	13.9625
TJP1	W2	PGE2	well_1	F001	368	Count/cell	20.22222	18	11.92213
TJP1	W2	PGE2	well_1	F002	217	Count/cell	22.02551	20	12.14715
TJP1	W2	PGE2	well_1	F003	424	Count/cell	22.51546	20	13.60944
TJP1	W2	PGE2	well_1	F004	325	Count/cell	25.01356	24	12.70598
TJP1	W2	PGE2	well_1	F005	224	Count/cell	22.19403	19	13.46169
TJP1	W2	PGE2	well_1	F006	427	Count/cell	18.40209	17	10.53964
TJP1	W2	PGE2	well_2	F001	331	Count/cell	20.85149	19	11.89807
TJP1	W2	PGE2	well_2	F002	534	Count/cell	14.19348	12	9.249668
TJP1	W2	PGE2	well_2	F003	398	Count/cell	19.47253	18	11.75258
TJP1	W2	PGE2	well_2	F004	461	Count/cell	25.40964	24	11.80215
TJP1	W2	PGE2	well_2	F005	388	Count/cell	21.02266	20	11.46385
TJP1	W2	PGE2	well_2	F006	310	Count/cell	17.13978	15	10.33635
TJP1	W2	PGE2	well_3	F001	93	Count/cell	23.20482	24	9.003738
TJP1	W2	PGE2	well_3	F002	478	Count/cell	15.79271	14	9.371595
TJP1	W2	PGE2	well_3	F003	491	Count/cell	18.29075	17	11.02352
TJP1	W2	PGE2	well_3	F004	550	Count/cell	17.67129	16	10.98912
TJP1	W2	PGE2	well_3	F005	458	Count/cell	15.66588	14	9.754698
TJP1	W2	PGE2	well_3	F006	38	Count/cell	22.65714	18	11.80215
TJP1	W2	PGE2	well_4	F001	313	Count/cell	21.79514	21	11.5029
TJP1	W2	PGE2	well_4	F002	455	Count/cell	19.01205	17	11.27746
TJP1	W2	PGE2	well_4	F003	445	Count/cell	19.64764	18	12.19493
TJP1	W2	PGE2	well_4	F004	329	Count/cell	19.52961	18.5	10.31064
TJP1	W2	PGE2	well_4	F005	375	Count/cell	21.90323	20	11.36452
TJP1	W2	PGE2	well_4	F006	41	Count/cell	21.72973	18	12.01076
TJP1	W2	PGE2	well_5	F001	518	Count/cell	16.87835	15	10.91324
TJP1	W2	PGE2	well_5	F002	406	Count/cell	19.69755	18	11.01581
TJP1	W2	PGE2	well_5	F003	326	Count/cell	18.02365	16.5	10.12445
TJP1	W2	PGE2	well_5	F004	392	Count/cell	20.47091	20	11.74615
TJP1	W2	PGE2	well_5	F005	397	Count/cell	21.43137	20	11.86696
TJP1	W2	PGE2	well_5	F006	471	Count/cell	18.28706	17	10.9537
TJP1	W3	HPI4	well_1	F001	48	Count/cell	21.95238	19	9.484139
TJP1	W3	HPI4	well_1	F002	74	Count/cell	26.19697	26	10.85317
TJP1	W3	HPI4	well_1	F003	81	Count/cell	19.76712	19	12.02257
TJP1	W3	HPI4	well_1	F004	127	Count/cell	23.34513	22	12.13228
TJP1	W3	HPI4	well_1	F005	74	Count/cell	24.75758	25	10.84302
TJP1	W3	HPI4	well_1	F006	66	Count/cell	24.63934	21	12.99363
TJP1	W3	HPI4	well_2	F001	74	Count/cell	23.69697	23	10.73738
TJP1	W3	HPI4	well_2	F002	183	Count/cell	19.05294	17	11.28504
TJP1	W3	HPI4	well_2	F003	105	Count/cell	21.96842	21	11.30943
TJP1	W3	HPI4	well_2	F004	154	Count/cell	20.55714	21	11.79213

TJP1	W3	HPI4	well_2	F005	200	Count/cell	19.18919	16	11.94015
TJP1	W3	HPI4	well_2	F006	127	Count/cell	20.47788	19	9.749631
TJP1	W3	HPI4	well_3	F001	88	Count/cell	24.3875	24	11.76295
TJP1	W3	HPI4	well_3	F002	154	Count/cell	20.36691	18	11.12651
TJP1	W3	HPI4	well_3	F003	74	Count/cell	24.65152	22.5	13.46162
TJP1	W3	HPI4	well_3	F004	181	Count/cell	18.43558	16	10.63249
TJP1	W3	HPI4	well_3	F005	158	Count/cell	23.47917	21	12.64356
TJP1	W3	HPI4	well_3	F006	179	Count/cell	21.58642	20	11.08894
TJP1	W3	HPI4	well_4	F001	94	Count/cell	19.48837	17.5	10.26329
TJP1	W3	HPI4	well_4	F002	198	Count/cell	23.64804	22	11.05421
TJP1	W3	HPI4	well_4	F003	141	Count/cell	21.46154	19	10.6692
TJP1	W3	HPI4	well_4	F004	62	Count/cell	24.5	21	13.17194
TJP1	W3	HPI4	well_4	F005	161	Count/cell	23.17808	21	11.85585
TJP1	W3	HPI4	well_4	F006	151	Count/cell	21.35294	18	11.08454
TJP1	W3	HPI4	well_5	F001	47	Count/cell	25.85366	20	13.12738
TJP1	W3	HPI4	well_5	F002	145	Count/cell	18.61069	17	10.70339
TJP1	W3	HPI4	well_5	F003	187	Count/cell	18.2907	17	11.06691
TJP1	W3	HPI4	well_5	F004	176	Count/cell	16.52761	16	9.623988
TJP1	W3	HPI4	well_5	F005	190	Count/cell	22.93714	21	12.285
TJP1	W3	HPI4	well_5	F006	192	Count/cell	16.59538	15	9.884118
TJP1	W3	PGE2	well_1	F001	390	Count/cell	19.78177	18	11.4594
TJP1	W3	PGE2	well_1	F002	404	Count/cell	16.0989	15	9.407874
TJP1	W3	PGE2	well_1	F003	375	Count/cell	15.93842	14	9.023451
TJP1	W3	PGE2	well_1	F004	417	Count/cell	14.40256	14	8.384371
TJP1	W3	PGE2	well_1	F005	358	Count/cell	16.78221	14	10.64324
TJP1	W3	PGE2	well_1	F006	223	Count/cell	15.18593	14	8.243798
TJP1	W3	PGE2	well_2	F001	211	Count/cell	20.64021	19	11.47095
TJP1	W3	PGE2	well_2	F002	366	Count/cell	15.68437	14	10.08737
TJP1	W3	PGE2	well_2	F003	284	Count/cell	14.35769	12	9.850962
TJP1	W3	PGE2	well_2	F004	413	Count/cell	14.39474	12	9.775149
TJP1	W3	PGE2	well_2	F005	350	Count/cell	18.75472	18	9.276147
TJP1	W3	PGE2	well_2	F006	289	Count/cell	12.23019	10	8.120065
TJP1	W3	PGE2	well_3	F001	426	Count/cell	14.24219	12	8.656556
TJP1	W3	PGE2	well_3	F002	388	Count/cell	14.24011	12.5	8.486048
TJP1	W3	PGE2	well_3	F003	391	Count/cell	14.59437	13	9.675467
TJP1	W3	PGE2	well_3	F004	330	Count/cell	12.88487	11	7.827971
TJP1	W3	PGE2	well_3	F005	468	Count/cell	12.63765	10	8.914716
TJP1	W3	PGE2	well_3	F006	185	Count/cell	11.31138	9	7.922748
TJP1	W3	PGE2	well_4	F001	250	Count/cell	14.4115	12	8.853055
TJP1	W3	PGE2	well_4	F002	418	Count/cell	17.39221	16	9.795005
TJP1	W3	PGE2	well_4	F003	218	Count/cell	14.45771	13	8.719487
TJP1	W3	PGE2	well_4	F004	444	Count/cell	17.31266	16	9.305298
TJP1	W3	PGE2	well_4	F005	419	Count/cell	17.63402	15	11.36544
TJP1	W3	PGE2	well_4	F006	226	Count/cell	13.12919	12	8.568029
TJP1	W3	PGE2	well_5	F001	333	Count/cell	15.41311	13	9.977598
TJP1	W3	PGE2	well_5	F002	314	Count/cell	15.80282	14	8.987026
TJP1	W3	PGE2	well_5	F003	345	Count/cell	12.91746	11	8.394693
TJP1	W3	PGE2	well_5	F004	289	Count/cell	16.32692	15	8.575626
TJP1	W3	PGE2	well_5	F005	414	Count/cell	16.05744	14	9.541688

TJP1	W3	PGE2	well_5	F006	141	Count/cell	16.73643	15	10.46387
TJP1	W4	HPI4	well_1	F001	89	Count/cell	20.91139	19	12.27901
TJP1	W4	HPI4	well_1	F002	154	Count/cell	23.32609	22	10.63815
TJP1	W4	HPI4	well_1	F003	42	Count/cell	27.13514	26	9.80692
TJP1	W4	HPI4	well_1	F004	191	Count/cell	22.31977	22	10.98628
TJP1	W4	HPI4	well_1	F005	125	Count/cell	25.28319	23	13.17565
TJP1	W4	HPI4	well_1	F006	194	Count/cell	18.49153	17	11.17023
TJP1	W4	HPI4	well_2	F001	162	Count/cell	24.71429	25	11.97028
TJP1	W4	HPI4	well_2	F002	145	Count/cell	20.90152	18	11.80041
TJP1	W4	HPI4	well_2	F003	133	Count/cell	25.05785	22	12.52417
TJP1	W4	HPI4	well_2	F004	111	Count/cell	25.43564	25	12.2388
TJP1	W4	HPI4	well_2	F005	128	Count/cell	24.17241	24.5	12.56515
TJP1	W4	HPI4	well_2	F006	136	Count/cell	27.18699	26	15.10257
TJP1	W4	HPI4	well_3	F001	195	Count/cell	19.93889	18	10.36386
TJP1	W4	HPI4	well_3	F002	183	Count/cell	23.50909	21	12.62335
TJP1	W4	HPI4	well_3	F003	174	Count/cell	23.85897	21	13.64927
TJP1	W4	HPI4	well_3	F004	182	Count/cell	24.30061	21	13.57882
TJP1	W4	HPI4	well_3	F005	160	Count/cell	20.86111	19	10.7043
TJP1	W4	HPI4	well_3	F006	28	Count/cell	19	20	10.2632
TJP1	W4	HPI4	well_4	F001	136	Count/cell	24.696	25	12.75528
TJP1	W4	HPI4	well_4	F002	121	Count/cell	28.19266	25	16.71353
TJP1	W4	HPI4	well_4	F003	115	Count/cell	27.74528	25	14.20668
TJP1	W4	HPI4	well_4	F004	187	Count/cell	22.04118	20	12.43794
TJP1	W4	HPI4	well_4	F005	230	Count/cell	18.80288	17	10.26154
TJP1	W4	HPI4	well_4	F006	103	Count/cell	24.84615	24	12.33416
TJP1	W4	HPI4	well_5	F001	174	Count/cell	28.66242	26	16.57754
TJP1	W4	HPI4	well_5	F002	136	Count/cell	21.88	19	13.13614
TJP1	W4	HPI4	well_5	F003	27	Count/cell	22.34783	23	8.445594
TJP1	W4	HPI4	well_5	F004	197	Count/cell	23.24581	21	12.17503
TJP1	W4	HPI4	well_5	F005	190	Count/cell	22.15029	21	11.27151
TJP1	W4	HPI4	well_5	F006	232	Count/cell	21.48585	20	12.37314
TJP1	W4	PGE2	well_1	F001	174	Count/cell	17.87342	15.5	10.32759
TJP1	W4	PGE2	well_1	F002	117	Count/cell	14.15094	12	9.502674
TJP1	W4	PGE2	well_1	F003	66	Count/cell	14.66102	15	6.781339
TJP1	W4	PGE2	well_1	F004	238	Count/cell	14.5	14	8.015682
TJP1	W4	PGE2	well_1	F005	389	Count/cell	13.67967	12	8.69151
TJP1	W4	PGE2	well_1	F006	259	Count/cell	14.18828	12	8.815696
TJP1	W4	PGE2	well_2	F001	129	Count/cell	16.44828	15	11.17088
TJP1	W4	PGE2	well_2	F002	174	Count/cell	19.69182	18	10.55183
TJP1	W4	PGE2	well_2	F003	190	Count/cell	12.71348	11	7.706622
TJP1	W4	PGE2	well_2	F004	279	Count/cell	12.35496	11	7.368772
TJP1	W4	PGE2	well_2	F005	366	Count/cell	12.99413	12	7.663126
TJP1	W4	PGE2	well_2	F006	257	Count/cell	12.27542	11	7.243221
TJP1	W4	PGE2	well_3	F001	63	Count/cell	14.72881	15	9.95651
TJP1	W4	PGE2	well_3	F002	418	Count/cell	12.88665	11	9.178353
TJP1	W4	PGE2	well_3	F003	344	Count/cell	12.72063	11	7.40619
TJP1	W4	PGE2	well_3	F004	364	Count/cell	13.48494	12	7.719189
TJP1	W4	PGE2	well_3	F005	149	Count/cell	11.88806	11	5.988285
TJP1	W4	PGE2	well_3	F006	333	Count/cell	12.92834	11	8.397094

TJP1	W4	PGE2	well_4	F001	295	Count/cell	15.14444	14	7.387663
TJP1	W4	PGE2	well_4	F002	280	Count/cell	15.58527	14	9.094537
TJP1	W4	PGE2	well_4	F003	189	Count/cell	16.73529	15	10.00742
TJP1	W4	PGE2	well_4	F004	198	Count/cell	12.69231	11	7.513071
TJP1	W4	PGE2	well_4	F005	273	Count/cell	12.95238	12	7.264888
TJP1	W4	PGE2	well_4	F006	209	Count/cell	16.00535	14	8.855772
TJP1	W4	PGE2	well_5	F001	295	Count/cell	13.21852	11	8.391026
TJP1	W4	PGE2	well_5	F002	185	Count/cell	12.26316	11	7.071549
TJP1	W4	PGE2	well_5	F003	303	Count/cell	12.24373	11	7.246291
TJP1	W4	PGE2	well_5	F004	319	Count/cell	13.77509	13	7.662676
TJP1	W4	PGE2	well_5	F005	289	Count/cell	14.17537	13	7.33591
TJP1	W4	PGE2	well_5	F006	244	Count/cell	14.65179	13	8.240456
TJP1	W1	HPI4	well_1	F001	316	X/Y distribution	7.77456	7.701646	1.422996
TJP1	W1	HPI4	well_1	F003	262	X/Y distribution	7.608963	7.238828	1.748438
TJP1	W1	HPI4	well_1	F004	176	X/Y distribution	8.960352	8.754015	1.969342
TJP1	W1	HPI4	well_1	F005	276	X/Y distribution	7.306474	7.110611	1.581325
TJP1	W1	HPI4	well_1	F006	288	X/Y distribution	7.628777	7.557064	1.617413
TJP1	W1	HPI4	well_2	F001	70	X/Y distribution	6.40072	6.192859	1.16772
TJP1	W1	HPI4	well_2	F002	239	X/Y distribution	8.657116	8.27983	1.760633
TJP1	W1	HPI4	well_2	F003	181	X/Y distribution	8.385319	8.175145	1.868741
TJP1	W1	HPI4	well_2	F004	255	X/Y distribution	7.88432	7.593168	1.735892
TJP1	W1	HPI4	well_2	F005	202	X/Y distribution	7.012546	7.0251	1.180828
TJP1	W1	HPI4	well_2	F006	394	X/Y distribution	6.850697	6.804087	1.376404
TJP1	W1	HPI4	well_3	F003	196	X/Y distribution	7.402865	7.321249	1.788152
TJP1	W1	HPI4	well_3	F004	283	X/Y distribution	7.412406	7.374659	1.319749
TJP1	W1	HPI4	well_3	F005	288	X/Y distribution	7.310728	7.001601	1.581107
TJP1	W1	HPI4	well_3	F006	371	X/Y distribution	6.644854	6.554314	1.376893
TJP1	W1	HPI4	well_4	F001	280	X/Y distribution	6.626757	6.473835	1.401999
TJP1	W1	HPI4	well_4	F002	250	X/Y distribution	7.0948	7.072762	1.445039
TJP1	W1	HPI4	well_4	F003	254	X/Y distribution	7.504834	7.517895	1.601658
TJP1	W1	HPI4	well_4	F004	205	X/Y distribution	8.184467	8.000733	1.596648
TJP1	W1	HPI4	well_4	F005	363	X/Y distribution	6.881193	6.711973	1.416809
TJP1	W1	HPI4	well_4	F006	330	X/Y distribution	7.141108	7.107781	1.497919
TJP1	W1	HPI4	well_5	F001	283	X/Y distribution	6.77105	6.583298	1.392061
TJP1	W1	HPI4	well_5	F002	150	X/Y distribution	6.940349	6.755833	1.345256
TJP1	W1	HPI4	well_5	F003	200	X/Y distribution	7.199373	7.138588	1.361238
TJP1	W1	HPI4	well_5	F004	189	X/Y distribution	7.119626	7.067435	1.411167
TJP1	W1	HPI4	well_5	F005	260	X/Y distribution	7.422306	7.317224	1.567769
TJP1	W1	HPI4	well_5	F006	227	X/Y distribution	7.071759	7.079777	1.187332
TJP1	W1	PGE2	well_1	F001	345	X/Y distribution	7.269085	7.036928	1.499864
TJP1	W1	PGE2	well_1	F002	238	X/Y distribution	7.873925	7.662138	1.520688
TJP1	W1	PGE2	well_1	F003	317	X/Y distribution	7.476227	7.331133	1.505912
TJP1	W1	PGE2	well_1	F004	257	X/Y distribution	7.674265	7.522993	1.857191
TJP1	W1	PGE2	well_1	F005	397	X/Y distribution	6.770607	6.691608	1.468623
TJP1	W1	PGE2	well_1	F006	464	X/Y distribution	6.659524	6.575144	1.203183
TJP1	W1	PGE2	well_2	F004	278	X/Y distribution	7.657441	7.408311	1.61745
TJP1	W1	PGE2	well_2	F005	237	X/Y distribution	8.753225	8.467123	1.787586
TJP1	W1	PGE2	well_3	F001	204	X/Y distribution	9.045038	8.80632	1.824866
TJP1	W1	PGE2	well_3	F002	175	X/Y distribution	7.667433	7.63253	1.458035

TJP1	W1	PGE2	well_3	F003	293	X/Y distribution	7.413513	7.078238	1.652603
TJP1	W1	PGE2	well_3	F004	232	X/Y distribution	8.567549	8.522599	1.934891
TJP1	W1	PGE2	well_3	F005	357	X/Y distribution	7.085534	6.945658	1.33646
TJP1	W1	PGE2	well_3	F006	301	X/Y distribution	7.394513	7.167472	1.478283
TJP1	W1	PGE2	well_4	F001	220	X/Y distribution	7.776771	7.714759	1.890773
TJP1	W1	PGE2	well_4	F003	186	X/Y distribution	7.420563	7.364598	1.618669
TJP1	W1	PGE2	well_4	F004	249	X/Y distribution	7.976613	7.918286	1.717622
TJP1	W1	PGE2	well_4	F005	281	X/Y distribution	7.387633	7.174577	1.589415
TJP1	W1	PGE2	well_4	F006	327	X/Y distribution	7.159486	6.905911	1.640663
TJP1	W1	PGE2	well_5	F001	296	X/Y distribution	7.811688	7.56136	1.574032
TJP1	W1	PGE2	well_5	F002	377	X/Y distribution	7.11873	6.948522	1.41952
TJP1	W1	PGE2	well_5	F003	286	X/Y distribution	7.024134	6.986701	1.451931
TJP1	W1	PGE2	well_5	F004	247	X/Y distribution	8.38525	8.183147	1.878404
TJP1	W1	PGE2	well_5	F005	294	X/Y distribution	7.422312	7.298199	1.39819
TJP1	W1	PGE2	well_5	F006	269	X/Y distribution	7.20794	7.016696	1.31213
TJP1	W2	HPI4	well_1	F001	266	X/Y distribution	8.014737	7.805627	1.724134
TJP1	W2	HPI4	well_1	F002	322	X/Y distribution	7.129176	6.890958	1.886961
TJP1	W2	HPI4	well_1	F003	270	X/Y distribution	6.995538	6.817497	1.532827
TJP1	W2	HPI4	well_1	F004	354	X/Y distribution	7.045229	7.032378	1.561396
TJP1	W2	HPI4	well_1	F005	220	X/Y distribution	8.192129	7.555113	2.059101
TJP1	W2	HPI4	well_1	F006	204	X/Y distribution	8.20275	7.874874	2.013027
TJP1	W2	HPI4	well_2	F001	286	X/Y distribution	7.981361	7.824469	1.84268
TJP1	W2	HPI4	well_2	F002	208	X/Y distribution	8.851293	8.692811	1.946327
TJP1	W2	HPI4	well_2	F003	234	X/Y distribution	7.931845	7.607265	1.815159
TJP1	W2	HPI4	well_2	F004	436	X/Y distribution	6.885093	6.755858	1.329504
TJP1	W2	HPI4	well_2	F005	393	X/Y distribution	7.440749	7.283955	1.551121
TJP1	W2	HPI4	well_2	F006	322	X/Y distribution	7.578243	7.437334	1.519379
TJP1	W2	HPI4	well_3	F001	359	X/Y distribution	7.158165	7.003025	1.400433
TJP1	W2	HPI4	well_3	F002	306	X/Y distribution	7.958703	7.57629	1.835782
TJP1	W2	HPI4	well_3	F003	227	X/Y distribution	8.043949	7.792648	1.801818
TJP1	W2	HPI4	well_3	F004	270	X/Y distribution	8.18416	7.884719	1.754161
TJP1	W2	HPI4	well_3	F005	288	X/Y distribution	6.661664	6.434735	1.536613
TJP1	W2	HPI4	well_3	F006	294	X/Y distribution	7.893638	7.669917	1.808023
TJP1	W2	HPI4	well_4	F001	332	X/Y distribution	7.468686	7.442665	1.574411
TJP1	W2	HPI4	well_4	F002	221	X/Y distribution	7.944871	7.632834	1.983878
TJP1	W2	HPI4	well_4	F003	390	X/Y distribution	7.158754	6.971974	1.499342
TJP1	W2	HPI4	well_4	F004	306	X/Y distribution	7.181473	7.013779	1.55383
TJP1	W2	HPI4	well_4	F005	328	X/Y distribution	7.477692	7.302467	1.651084
TJP1	W2	HPI4	well_4	F006	307	X/Y distribution	7.461066	7.213902	1.607434
TJP1	W2	HPI4	well_5	F001	338	X/Y distribution	7.688951	7.407535	1.799367
TJP1	W2	HPI4	well_5	F002	286	X/Y distribution	8.040687	7.813364	1.846658
TJP1	W2	HPI4	well_5	F003	265	X/Y distribution	8.072777	7.924905	1.719406
TJP1	W2	HPI4	well_5	F004	336	X/Y distribution	7.456288	7.324637	1.524384
TJP1	W2	HPI4	well_5	F005	356	X/Y distribution	7.400416	7.207227	1.57296
TJP1	W2	HPI4	well_5	F006	358	X/Y distribution	7.312422	7.180243	1.714339
TJP1	W2	PGE2	well_1	F001	368	X/Y distribution	6.551937	6.41276	1.231054
TJP1	W2	PGE2	well_1	F002	217	X/Y distribution	6.272329	6.195879	1.09539
TJP1	W2	PGE2	well_1	F003	424	X/Y distribution	6.549837	6.493044	1.1767
TJP1	W2	PGE2	well_1	F004	325	X/Y distribution	7.014808	6.991842	1.075191

TJP1	W2	PGE2	well_1	F005	224	X/Y distribution	6.489857	6.456117	1.051539
TJP1	W2	PGE2	well_1	F006	427	X/Y distribution	5.910646	5.834318	1.057525
TJP1	W2	PGE2	well_2	F001	331	X/Y distribution	6.331108	6.197989	1.123619
TJP1	W2	PGE2	well_2	F002	534	X/Y distribution	5.622504	5.549061	1.132824
TJP1	W2	PGE2	well_2	F003	398	X/Y distribution	6.104351	6.021202	1.071421
TJP1	W2	PGE2	well_2	F004	461	X/Y distribution	6.164769	6.122073	0.855131
TJP1	W2	PGE2	well_2	F005	388	X/Y distribution	6.223713	6.210918	1.008049
TJP1	W2	PGE2	well_2	F006	310	X/Y distribution	5.785245	5.822476	1.013656
TJP1	W2	PGE2	well_3	F001	93	X/Y distribution	9.865457	9.662074	1.693194
TJP1	W2	PGE2	well_3	F002	478	X/Y distribution	5.904361	5.815685	1.168625
TJP1	W2	PGE2	well_3	F003	491	X/Y distribution	5.963769	5.910614	1.089286
TJP1	W2	PGE2	well_3	F004	550	X/Y distribution	6.101207	6.038902	1.166515
TJP1	W2	PGE2	well_3	F005	458	X/Y distribution	6.207145	6.172377	1.177865
TJP1	W2	PGE2	well_3	F006	38	X/Y distribution	9.320766	9.115778	1.795663
TJP1	W2	PGE2	well_4	F001	313	X/Y distribution	8.035675	7.936636	1.710942
TJP1	W2	PGE2	well_4	F002	455	X/Y distribution	5.944628	5.871715	1.017591
TJP1	W2	PGE2	well_4	F003	445	X/Y distribution	6.169956	6.123286	1.073151
TJP1	W2	PGE2	well_4	F004	329	X/Y distribution	7.614723	7.552822	1.613065
TJP1	W2	PGE2	well_4	F005	375	X/Y distribution	6.970812	6.774905	1.647566
TJP1	W2	PGE2	well_4	F006	41	X/Y distribution	9.570127	9.453823	2.334916
TJP1	W2	PGE2	well_5	F001	518	X/Y distribution	6.022801	5.871641	1.288434
TJP1	W2	PGE2	well_5	F002	406	X/Y distribution	6.531359	6.345888	1.272697
TJP1	W2	PGE2	well_5	F003	326	X/Y distribution	6.088751	5.912362	1.123253
TJP1	W2	PGE2	well_5	F004	392	X/Y distribution	6.47356	6.460146	1.121892
TJP1	W2	PGE2	well_5	F005	397	X/Y distribution	6.774726	6.873723	1.190883
TJP1	W2	PGE2	well_5	F006	471	X/Y distribution	6.103966	6.062139	1.115105
TJP1	W3	HPI4	well_1	F001	48	X/Y distribution	9.885556	10.05096	1.896479
TJP1	W3	HPI4	well_1	F002	74	X/Y distribution	9.472628	9.567069	1.624835
TJP1	W3	HPI4	well_1	F003	81	X/Y distribution	8.647461	8.574653	2.332113
TJP1	W3	HPI4	well_1	F004	127	X/Y distribution	9.032607	8.911764	1.985262
TJP1	W3	HPI4	well_1	F005	74	X/Y distribution	8.556662	8.494217	1.508468
TJP1	W3	HPI4	well_1	F006	66	X/Y distribution	9.084708	9.131887	1.530453
TJP1	W3	HPI4	well_2	F001	74	X/Y distribution	10.05477	10.1494	2.002904
TJP1	W3	HPI4	well_2	F002	183	X/Y distribution	8.155401	7.934831	1.870579
TJP1	W3	HPI4	well_2	F003	105	X/Y distribution	8.668382	8.476535	1.720246
TJP1	W3	HPI4	well_2	F004	154	X/Y distribution	7.885732	7.455236	1.950202
TJP1	W3	HPI4	well_2	F005	200	X/Y distribution	7.422	7.368898	1.858465
TJP1	W3	HPI4	well_2	F006	127	X/Y distribution	8.502791	8.561307	1.743145
TJP1	W3	HPI4	well_3	F001	88	X/Y distribution	10.14482	10.1793	1.804841
TJP1	W3	HPI4	well_3	F002	154	X/Y distribution	8.922005	8.780647	2.047254
TJP1	W3	HPI4	well_3	F003	74	X/Y distribution	8.974379	9.19931	2.195139
TJP1	W3	HPI4	well_3	F004	181	X/Y distribution	7.655574	7.470656	1.716134
TJP1	W3	HPI4	well_3	F005	158	X/Y distribution	8.573802	8.249243	2.160967
TJP1	W3	HPI4	well_3	F006	179	X/Y distribution	8.855068	8.740361	2.355672
TJP1	W3	HPI4	well_4	F001	94	X/Y distribution	8.995083	9.22587	1.946728
TJP1	W3	HPI4	well_4	F002	198	X/Y distribution	8.647253	8.462515	1.803976
TJP1	W3	HPI4	well_4	F003	141	X/Y distribution	8.628082	8.357379	1.846591
TJP1	W3	HPI4	well_4	F004	62	X/Y distribution	8.799082	8.800497	1.925514
TJP1	W3	HPI4	well_4	F005	161	X/Y distribution	9.638237	9.796279	2.203703

TJP1	W3	HPI4	well_4	F006	151	X/Y distribution	8.276454	8.059416	2.052617
TJP1	W3	HPI4	well_5	F001	47	X/Y distribution	9.581846	9.302916	1.974512
TJP1	W3	HPI4	well_5	F002	145	X/Y distribution	8.32256	8.317334	1.919953
TJP1	W3	HPI4	well_5	F003	187	X/Y distribution	8.047996	7.938809	2.020429
TJP1	W3	HPI4	well_5	F004	176	X/Y distribution	7.921919	7.982941	2.054096
TJP1	W3	HPI4	well_5	F005	190	X/Y distribution	7.991526	7.751067	1.773072
TJP1	W3	HPI4	well_5	F006	192	X/Y distribution	7.758331	7.65367	1.915564
TJP1	W3	PGE2	well_1	F001	390	X/Y distribution	6.233535	6.225718	0.861747
TJP1	W3	PGE2	well_1	F002	404	X/Y distribution	6.151625	6.193909	1.081039
TJP1	W3	PGE2	well_1	F003	375	X/Y distribution	6.258499	6.21097	1.010703
TJP1	W3	PGE2	well_1	F004	417	X/Y distribution	5.946564	5.888023	1.020841
TJP1	W3	PGE2	well_1	F005	358	X/Y distribution	6.217639	6.170424	1.1656
TJP1	W3	PGE2	well_1	F006	223	X/Y distribution	6.277592	6.269659	1.06602
TJP1	W3	PGE2	well_2	F001	211	X/Y distribution	6.766479	6.679298	1.083322
TJP1	W3	PGE2	well_2	F002	366	X/Y distribution	6.169649	6.03871	1.230369
TJP1	W3	PGE2	well_2	F003	284	X/Y distribution	6.285126	6.336895	1.272718
TJP1	W3	PGE2	well_2	F004	413	X/Y distribution	5.891567	5.867252	1.157927
TJP1	W3	PGE2	well_2	F005	350	X/Y distribution	5.97666	5.923438	0.866619
TJP1	W3	PGE2	well_2	F006	289	X/Y distribution	6.125627	6.14632	1.12608
TJP1	W3	PGE2	well_3	F001	426	X/Y distribution	6.176627	6.112474	1.108525
TJP1	W3	PGE2	well_3	F002	388	X/Y distribution	6.471817	6.430482	1.187314
TJP1	W3	PGE2	well_3	F003	391	X/Y distribution	6.136303	6.09995	1.175747
TJP1	W3	PGE2	well_3	F004	330	X/Y distribution	5.841147	5.86345	0.996524
TJP1	W3	PGE2	well_3	F005	468	X/Y distribution	5.84432	5.818528	1.170506
TJP1	W3	PGE2	well_3	F006	185	X/Y distribution	5.923934	6.050189	1.205449
TJP1	W3	PGE2	well_4	F001	250	X/Y distribution	6.069957	6.042385	1.002209
TJP1	W3	PGE2	well_4	F002	418	X/Y distribution	5.804883	5.83174	0.894655
TJP1	W3	PGE2	well_4	F003	218	X/Y distribution	6.300757	6.254481	1.217286
TJP1	W3	PGE2	well_4	F004	444	X/Y distribution	6.188212	6.083313	1.044107
TJP1	W3	PGE2	well_4	F005	419	X/Y distribution	6.193238	6.249525	1.048402
TJP1	W3	PGE2	well_4	F006	226	X/Y distribution	6.018761	5.960198	1.168301
TJP1	W3	PGE2	well_5	F001	333	X/Y distribution	6.267964	6.248921	1.124448
TJP1	W3	PGE2	well_5	F002	314	X/Y distribution	6.224483	6.268055	1.028228
TJP1	W3	PGE2	well_5	F003	345	X/Y distribution	6.136846	6.237126	1.104621
TJP1	W3	PGE2	well_5	F004	289	X/Y distribution	6.192505	6.190845	0.9067
TJP1	W3	PGE2	well_5	F005	414	X/Y distribution	6.013264	6.113462	1.120939
TJP1	W3	PGE2	well_5	F006	141	X/Y distribution	6.258168	6.103902	1.185726
TJP1	W4	HPI4	well_1	F001	89	X/Y distribution	9.041482	8.684498	2.231329
TJP1	W4	HPI4	well_1	F002	154	X/Y distribution	9.660824	9.565468	2.081593
TJP1	W4	HPI4	well_1	F003	42	X/Y distribution	10.30943	10.51444	1.765866
TJP1	W4	HPI4	well_1	F004	191	X/Y distribution	8.514488	8.242968	2.039686
TJP1	W4	HPI4	well_1	F005	125	X/Y distribution	9.161374	8.83107	2.085355
TJP1	W4	HPI4	well_1	F006	194	X/Y distribution	7.522431	7.355255	1.852293
TJP1	W4	HPI4	well_2	F001	162	X/Y distribution	9.268308	8.887545	1.991419
TJP1	W4	HPI4	well_2	F002	145	X/Y distribution	10.35717	10.52459	2.264163
TJP1	W4	HPI4	well_2	F003	133	X/Y distribution	9.392761	9.463703	2.293762
TJP1	W4	HPI4	well_2	F004	111	X/Y distribution	9.810335	9.467462	2.095631
TJP1	W4	HPI4	well_2	F005	128	X/Y distribution	9.277759	8.991112	2.330368
TJP1	W4	HPI4	well_2	F006	136	X/Y distribution	9.399166	9.300812	2.029677

TJP1	W4	HPI4	well_3	F001	195	X/Y distribution	9.637717	9.737724	2.183529
TJP1	W4	HPI4	well_3	F002	183	X/Y distribution	8.989947	8.959887	1.847194
TJP1	W4	HPI4	well_3	F003	174	X/Y distribution	8.977412	8.834927	1.986587
TJP1	W4	HPI4	well_3	F004	182	X/Y distribution	9.149822	9.27269	2.047256
TJP1	W4	HPI4	well_3	F005	160	X/Y distribution	9.412326	9.503929	2.121864
TJP1	W4	HPI4	well_3	F006	28	X/Y distribution	10.30935	10.27876	2.145639
TJP1	W4	HPI4	well_4	F001	136	X/Y distribution	9.907429	9.778924	2.490234
TJP1	W4	HPI4	well_4	F002	121	X/Y distribution	9.798705	10.25353	2.351952
TJP1	W4	HPI4	well_4	F003	115	X/Y distribution	9.901801	9.6845	2.415776
TJP1	W4	HPI4	well_4	F004	187	X/Y distribution	9.504749	9.547691	2.271516
TJP1	W4	HPI4	well_4	F005	230	X/Y distribution	7.820326	7.704189	1.89972
TJP1	W4	HPI4	well_4	F006	103	X/Y distribution	9.303447	8.907733	2.336302
TJP1	W4	HPI4	well_5	F001	174	X/Y distribution	10.05378	10.25037	2.284836
TJP1	W4	HPI4	well_5	F002	136	X/Y distribution	8.913556	8.645953	2.176405
TJP1	W4	HPI4	well_5	F003	27	X/Y distribution	11.14847	11.17313	1.55844
TJP1	W4	HPI4	well_5	F004	197	X/Y distribution	8.897807	8.715207	2.140513
TJP1	W4	HPI4	well_5	F005	190	X/Y distribution	9.234555	9.245119	2.129592
TJP1	W4	HPI4	well_5	F006	232	X/Y distribution	8.180039	7.978289	1.96683
TJP1	W4	PGE2	well_1	F001	174	X/Y distribution	6.84859	6.810409	0.918789
TJP1	W4	PGE2	well_1	F002	117	X/Y distribution	6.851284	6.960364	1.26624
TJP1	W4	PGE2	well_1	F003	66	X/Y distribution	6.52657	6.551276	0.773527
TJP1	W4	PGE2	well_1	F004	238	X/Y distribution	6.441216	6.391908	0.869623
TJP1	W4	PGE2	well_1	F005	389	X/Y distribution	6.386871	6.497575	1.038991
TJP1	W4	PGE2	well_1	F006	259	X/Y distribution	6.866318	6.837458	1.30022
TJP1	W4	PGE2	well_2	F001	129	X/Y distribution	8.369734	8.208764	1.727118
TJP1	W4	PGE2	well_2	F002	174	X/Y distribution	8.220847	8.220603	1.526136
TJP1	W4	PGE2	well_2	F003	190	X/Y distribution	6.669468	6.772604	1.142421
TJP1	W4	PGE2	well_2	F004	279	X/Y distribution	6.309765	6.301749	1.005219
TJP1	W4	PGE2	well_2	F005	366	X/Y distribution	6.407186	6.355641	1.084234
TJP1	W4	PGE2	well_2	F006	257	X/Y distribution	6.358303	6.323878	0.864047
TJP1	W4	PGE2	well_3	F001	63	X/Y distribution	7.188132	7.105936	1.640445
TJP1	W4	PGE2	well_3	F002	418	X/Y distribution	6.240585	6.269823	1.385896
TJP1	W4	PGE2	well_3	F003	344	X/Y distribution	6.755689	6.726025	1.297568
TJP1	W4	PGE2	well_3	F004	364	X/Y distribution	6.401475	6.453816	1.134154
TJP1	W4	PGE2	well_3	F005	149	X/Y distribution	6.175427	6.169139	0.977827
TJP1	W4	PGE2	well_3	F006	333	X/Y distribution	6.029877	6.035552	1.025934
TJP1	W4	PGE2	well_4	F001	295	X/Y distribution	6.713212	6.61646	0.926841
TJP1	W4	PGE2	well_4	F002	280	X/Y distribution	6.814301	6.765133	1.108265
TJP1	W4	PGE2	well_4	F003	189	X/Y distribution	7.022619	7.059286	1.253487
TJP1	W4	PGE2	well_4	F004	198	X/Y distribution	6.678822	6.73115	1.092381
TJP1	W4	PGE2	well_4	F005	273	X/Y distribution	6.660618	6.769132	1.094356
TJP1	W4	PGE2	well_4	F006	209	X/Y distribution	7.282601	7.190147	1.053024
TJP1	W4	PGE2	well_5	F001	295	X/Y distribution	6.729756	6.689187	1.181979
TJP1	W4	PGE2	well_5	F002	185	X/Y distribution	6.232775	6.303121	0.952197
TJP1	W4	PGE2	well_5	F003	303	X/Y distribution	6.578305	6.591414	1.13835
TJP1	W4	PGE2	well_5	F004	319	X/Y distribution	6.358885	6.381358	0.932974
TJP1	W4	PGE2	well_5	F005	289	X/Y distribution	6.446213	6.397962	0.981952
TJP1	W4	PGE2	well_5	F006	244	X/Y distribution	6.698928	6.644954	0.901604
TJP1	W1	HPI4	well_1	F001	316	Z distribution	-0.01366	-0.04312	0.57391

TJP1	W1	HPI4	well_1	F003	262	Z distribution	-0.04874	-0.10783	0.575012
TJP1	W1	HPI4	well_1	F004	176	Z distribution	-0.3329	-0.34166	0.568867
TJP1	W1	HPI4	well_1	F005	276	Z distribution	0.16297	0.025454	0.695358
TJP1	W1	HPI4	well_1	F006	288	Z distribution	-0.0754	-0.0755	0.592209
TJP1	W1	HPI4	well_2	F001	70	Z distribution	0.019714	-0.02745	0.512716
TJP1	W1	HPI4	well_2	F002	239	Z distribution	-0.21628	-0.19374	0.454189
TJP1	W1	HPI4	well_2	F003	181	Z distribution	-0.2837	-0.23358	0.499199
TJP1	W1	HPI4	well_2	F004	255	Z distribution	-0.01223	-0.02258	0.58848
TJP1	W1	HPI4	well_2	F005	202	Z distribution	0.105007	0.006049	0.715503
TJP1	W1	HPI4	well_2	F006	394	Z distribution	0.167115	0.113407	0.71614
TJP1	W1	HPI4	well_3	F003	196	Z distribution	-0.11056	-0.07855	0.5563
TJP1	W1	HPI4	well_3	F004	283	Z distribution	-0.01039	-0.04779	0.574366
TJP1	W1	HPI4	well_3	F005	288	Z distribution	-0.06704	-0.08695	0.500793
TJP1	W1	HPI4	well_3	F006	371	Z distribution	0.154271	-0.02846	0.743336
TJP1	W1	HPI4	well_4	F001	280	Z distribution	-0.09629	-0.08422	0.597103
TJP1	W1	HPI4	well_4	F002	250	Z distribution	0.058424	0.034892	0.538097
TJP1	W1	HPI4	well_4	F003	254	Z distribution	-0.10257	-0.15739	0.511567
TJP1	W1	HPI4	well_4	F004	205	Z distribution	-0.03376	-0.03654	0.457724
TJP1	W1	HPI4	well_4	F005	363	Z distribution	-0.11711	-0.17113	0.563253
TJP1	W1	HPI4	well_4	F006	330	Z distribution	0.093333	0.0492	0.601611
TJP1	W1	HPI4	well_5	F001	283	Z distribution	-0.0958	-0.04812	0.497651
TJP1	W1	HPI4	well_5	F002	150	Z distribution	0.004071	-0.05037	0.516559
TJP1	W1	HPI4	well_5	F003	200	Z distribution	-0.15902	-0.12684	0.501669
TJP1	W1	HPI4	well_5	F004	189	Z distribution	-0.03129	-0.07414	0.510456
TJP1	W1	HPI4	well_5	F005	260	Z distribution	0.021437	-0.0177	0.531922
TJP1	W1	HPI4	well_5	F006	227	Z distribution	-0.09029	-0.10432	0.491017
TJP1	W1	PGE2	well_1	F001	345	Z distribution	-0.07174	-0.15986	0.765248
TJP1	W1	PGE2	well_1	F002	238	Z distribution	-0.17138	-0.18806	0.632483
TJP1	W1	PGE2	well_1	F003	317	Z distribution	-0.13827	-0.16845	0.712225
TJP1	W1	PGE2	well_1	F004	257	Z distribution	-0.18258	-0.18644	0.647858
TJP1	W1	PGE2	well_1	F005	397	Z distribution	0.058284	0.011148	0.73573
TJP1	W1	PGE2	well_1	F006	464	Z distribution	0.041646	-0.02378	0.707541
TJP1	W1	PGE2	well_2	F004	278	Z distribution	-0.0328	-0.05192	0.66984
TJP1	W1	PGE2	well_2	F005	237	Z distribution	0.001049	0.012996	0.650477
TJP1	W1	PGE2	well_3	F001	204	Z distribution	-0.24768	-0.21755	0.470603
TJP1	W1	PGE2	well_3	F002	175	Z distribution	-0.06796	-0.10557	0.616271
TJP1	W1	PGE2	well_3	F003	293	Z distribution	0.081046	0.049733	0.573583
TJP1	W1	PGE2	well_3	F004	232	Z distribution	-0.13299	-0.12144	0.575688
TJP1	W1	PGE2	well_3	F005	357	Z distribution	-0.18027	-0.15891	0.681188
TJP1	W1	PGE2	well_3	F006	301	Z distribution	-0.08735	-0.11899	0.584932
TJP1	W1	PGE2	well_4	F001	220	Z distribution	-0.10013	-0.14027	0.567556
TJP1	W1	PGE2	well_4	F003	186	Z distribution	0.111048	0.034081	0.664043
TJP1	W1	PGE2	well_4	F004	249	Z distribution	-0.0764	-0.06168	0.62309
TJP1	W1	PGE2	well_4	F005	281	Z distribution	0.023171	-0.03253	0.727258
TJP1	W1	PGE2	well_4	F006	327	Z distribution	-0.08464	-0.1329	0.690376
TJP1	W1	PGE2	well_5	F001	296	Z distribution	-0.10056	-0.09012	0.534876
TJP1	W1	PGE2	well_5	F002	377	Z distribution	0.027719	-0.00178	0.719636
TJP1	W1	PGE2	well_5	F003	286	Z distribution	0.144035	0.086784	0.618817
TJP1	W1	PGE2	well_5	F004	247	Z distribution	-0.31113	-0.30218	0.504111

TJP1	W1	PGE2	well_5	F005	294	Z distribution	0.033596	0.036477	0.800292
TJP1	W1	PGE2	well_5	F006	269	Z distribution	0.063905	-0.09133	0.638805
TJP1	W2	HPI4	well_1	F001	266	Z distribution	0.05872	0.062053	0.400779
TJP1	W2	HPI4	well_1	F002	322	Z distribution	-0.17488	-0.16097	0.452941
TJP1	W2	HPI4	well_1	F003	270	Z distribution	-0.07138	-0.04668	0.414325
TJP1	W2	HPI4	well_1	F004	354	Z distribution	-0.18776	-0.18754	0.395175
TJP1	W2	HPI4	well_1	F005	220	Z distribution	-0.05814	-0.03264	0.368327
TJP1	W2	HPI4	well_1	F006	204	Z distribution	-0.08493	-0.03928	0.432436
TJP1	W2	HPI4	well_2	F001	286	Z distribution	0.138177	0.096096	0.415322
TJP1	W2	HPI4	well_2	F002	208	Z distribution	0.014077	0.021542	0.489795
TJP1	W2	HPI4	well_2	F003	234	Z distribution	-0.17151	-0.18288	0.414349
TJP1	W2	HPI4	well_2	F004	436	Z distribution	-0.03155	-0.04448	0.444051
TJP1	W2	HPI4	well_2	F005	393	Z distribution	-0.10923	-0.09362	0.366571
TJP1	W2	HPI4	well_2	F006	322	Z distribution	-0.02038	-0.02156	0.411687
TJP1	W2	HPI4	well_3	F001	359	Z distribution	0.071395	0.047938	0.423553
TJP1	W2	HPI4	well_3	F002	306	Z distribution	-0.03679	-0.06998	0.410257
TJP1	W2	HPI4	well_3	F003	227	Z distribution	-0.09186	-0.06909	0.34746
TJP1	W2	HPI4	well_3	F004	270	Z distribution	0.042639	0.059635	0.4107
TJP1	W2	HPI4	well_3	F005	288	Z distribution	-0.10083	-0.04199	0.566725
TJP1	W2	HPI4	well_3	F006	294	Z distribution	-0.10665	-0.1056	0.466302
TJP1	W2	HPI4	well_4	F001	332	Z distribution	-0.01571	0.000901	0.44338
TJP1	W2	HPI4	well_4	F002	221	Z distribution	-0.01806	-0.05442	0.450837
TJP1	W2	HPI4	well_4	F003	390	Z distribution	-0.15824	-0.12691	0.446695
TJP1	W2	HPI4	well_4	F004	306	Z distribution	0.000705	0.013924	0.407065
TJP1	W2	HPI4	well_4	F005	328	Z distribution	-0.28698	-0.2743	0.393188
TJP1	W2	HPI4	well_4	F006	307	Z distribution	-0.03059	0.023297	0.45563
TJP1	W2	HPI4	well_5	F001	338	Z distribution	-0.05949	-0.05332	0.397894
TJP1	W2	HPI4	well_5	F002	286	Z distribution	-0.04697	-0.01514	0.43548
TJP1	W2	HPI4	well_5	F003	265	Z distribution	-0.15024	-0.12912	0.434308
TJP1	W2	HPI4	well_5	F004	336	Z distribution	-0.04658	-0.05453	0.4523
TJP1	W2	HPI4	well_5	F005	356	Z distribution	-0.19156	-0.12565	0.46641
TJP1	W2	HPI4	well_5	F006	358	Z distribution	-0.19176	-0.18285	0.475177
TJP1	W2	PGE2	well_1	F001	368	Z distribution	0.445844	0.387654	0.852264
TJP1	W2	PGE2	well_1	F002	217	Z distribution	0.283872	0.250279	0.52359
TJP1	W2	PGE2	well_1	F003	424	Z distribution	0.47805	0.357091	0.720502
TJP1	W2	PGE2	well_1	F004	325	Z distribution	0.473802	0.479516	0.595751
TJP1	W2	PGE2	well_1	F005	224	Z distribution	0.085532	0.00202	0.752975
TJP1	W2	PGE2	well_1	F006	427	Z distribution	0.473855	0.37805	0.848753
TJP1	W2	PGE2	well_2	F001	331	Z distribution	0.258382	0.199249	0.689117
TJP1	W2	PGE2	well_2	F002	534	Z distribution	0.651108	0.575845	0.89991
TJP1	W2	PGE2	well_2	F003	398	Z distribution	0.299772	0.239427	0.670869
TJP1	W2	PGE2	well_2	F004	461	Z distribution	0.859532	0.818348	0.799331
TJP1	W2	PGE2	well_2	F005	388	Z distribution	0.346728	0.324251	0.782458
TJP1	W2	PGE2	well_2	F006	310	Z distribution	0.370016	0.255851	0.725026
TJP1	W2	PGE2	well_3	F001	93	Z distribution	-0.06749	-0.10694	0.332553
TJP1	W2	PGE2	well_3	F002	478	Z distribution	0.336824	0.266709	0.869385
TJP1	W2	PGE2	well_3	F003	491	Z distribution	0.53368	0.447744	0.756004
TJP1	W2	PGE2	well_3	F004	550	Z distribution	0.384522	0.366004	0.817078
TJP1	W2	PGE2	well_3	F005	458	Z distribution	0.303121	0.172157	0.871467

TJP1	W2	PGE2	well_3	F006	38	Z distribution	-0.03859	0.052973	0.446413
TJP1	W2	PGE2	well_4	F001	313	Z distribution	-0.10441	-0.08329	0.505984
TJP1	W2	PGE2	well_4	F002	455	Z distribution	0.72235	0.730606	0.722229
TJP1	W2	PGE2	well_4	F003	445	Z distribution	0.44427	0.348674	0.736171
TJP1	W2	PGE2	well_4	F004	329	Z distribution	0.250202	0.143038	0.592499
TJP1	W2	PGE2	well_4	F005	375	Z distribution	0.176144	0.09358	0.585705
TJP1	W2	PGE2	well_4	F006	41	Z distribution	-0.20612	-0.18885	0.448397
TJP1	W2	PGE2	well_5	F001	518	Z distribution	0.563105	0.477758	0.867409
TJP1	W2	PGE2	well_5	F002	406	Z distribution	0.131032	0.080416	0.663804
TJP1	W2	PGE2	well_5	F003	326	Z distribution	0.485127	0.445816	0.681309
TJP1	W2	PGE2	well_5	F004	392	Z distribution	0.562221	0.495516	0.708473
TJP1	W2	PGE2	well_5	F005	397	Z distribution	0.612674	0.565877	0.703415
TJP1	W2	PGE2	well_5	F006	471	Z distribution	0.512395	0.469417	0.72929
TJP1	W3	HPI4	well_1	F001	48	Z distribution	-0.03879	-0.01519	0.310484
TJP1	W3	HPI4	well_1	F002	74	Z distribution	0.075981	0.053148	0.258584
TJP1	W3	HPI4	well_1	F003	81	Z distribution	-0.06777	-0.06305	0.320519
TJP1	W3	HPI4	well_1	F004	127	Z distribution	-0.18578	-0.16308	0.405972
TJP1	W3	HPI4	well_1	F005	74	Z distribution	-0.00771	0.075837	0.350353
TJP1	W3	HPI4	well_1	F006	66	Z distribution	-0.26992	-0.27432	0.313889
TJP1	W3	HPI4	well_2	F001	74	Z distribution	-0.1426	-0.18373	0.411131
TJP1	W3	HPI4	well_2	F002	183	Z distribution	-0.06431	-0.05514	0.382928
TJP1	W3	HPI4	well_2	F003	105	Z distribution	-0.15077	-0.17256	0.335865
TJP1	W3	HPI4	well_2	F004	154	Z distribution	-0.21368	-0.22694	0.366974
TJP1	W3	HPI4	well_2	F005	200	Z distribution	-0.02772	-0.04149	0.351463
TJP1	W3	HPI4	well_2	F006	127	Z distribution	0.034049	0.005696	0.325726
TJP1	W3	HPI4	well_3	F001	88	Z distribution	-0.17982	-0.16814	0.354796
TJP1	W3	HPI4	well_3	F002	154	Z distribution	-0.00279	-0.0006	0.363533
TJP1	W3	HPI4	well_3	F003	74	Z distribution	-0.11681	-0.10747	0.395613
TJP1	W3	HPI4	well_3	F004	181	Z distribution	-0.08761	-0.07951	0.445705
TJP1	W3	HPI4	well_3	F005	158	Z distribution	-0.06736	-0.03545	0.418138
TJP1	W3	HPI4	well_3	F006	179	Z distribution	-0.09545	-0.04215	0.384839
TJP1	W3	HPI4	well_4	F001	94	Z distribution	-0.10645	-0.02265	0.362001
TJP1	W3	HPI4	well_4	F002	198	Z distribution	-0.08997	-0.10778	0.351754
TJP1	W3	HPI4	well_4	F003	141	Z distribution	-0.16589	-0.15674	0.37567
TJP1	W3	HPI4	well_4	F004	62	Z distribution	-0.02256	-0.00509	0.345018
TJP1	W3	HPI4	well_4	F005	161	Z distribution	-0.13331	-0.10869	0.383168
TJP1	W3	HPI4	well_4	F006	151	Z distribution	-0.12694	-0.09541	0.387187
TJP1	W3	HPI4	well_5	F001	47	Z distribution	-0.1308	-0.07426	0.24143
TJP1	W3	HPI4	well_5	F002	145	Z distribution	-0.16385	-0.14142	0.418862
TJP1	W3	HPI4	well_5	F003	187	Z distribution	-0.11768	-0.10887	0.362197
TJP1	W3	HPI4	well_5	F004	176	Z distribution	-0.02915	-0.03561	0.32248
TJP1	W3	HPI4	well_5	F005	190	Z distribution	-0.14696	-0.09894	0.399982
TJP1	W3	HPI4	well_5	F006	192	Z distribution	-0.08696	-0.08428	0.338689
TJP1	W3	PGE2	well_1	F001	390	Z distribution	0.873282	0.836814	0.811785
TJP1	W3	PGE2	well_1	F002	404	Z distribution	0.855727	0.841191	0.743133
TJP1	W3	PGE2	well_1	F003	375	Z distribution	0.90281	0.972112	0.776423
TJP1	W3	PGE2	well_1	F004	417	Z distribution	1.003765	1.029757	0.914831
TJP1	W3	PGE2	well_1	F005	358	Z distribution	1.05694	1.261678	0.951278
TJP1	W3	PGE2	well_1	F006	223	Z distribution	1.420277	1.589812	0.785886

TJP1	W3	PGE2	well_2	F001	211	Z distribution	0.522054	0.518886	0.775185
TJP1	W3	PGE2	well_2	F002	366	Z distribution	0.352377	0.332449	0.856163
TJP1	W3	PGE2	well_2	F003	284	Z distribution	1.106902	1.150706	0.669573
TJP1	W3	PGE2	well_2	F004	413	Z distribution	0.90913	0.985534	1.15727
TJP1	W3	PGE2	well_2	F005	350	Z distribution	1.115968	1.13181	0.738349
TJP1	W3	PGE2	well_2	F006	289	Z distribution	1.459752	1.584011	0.690969
TJP1	W3	PGE2	well_3	F001	426	Z distribution	0.874403	0.941255	0.835848
TJP1	W3	PGE2	well_3	F002	388	Z distribution	0.402547	0.298788	1.075631
TJP1	W3	PGE2	well_3	F003	391	Z distribution	1.115455	1.238149	0.807181
TJP1	W3	PGE2	well_3	F004	330	Z distribution	1.211701	1.352215	0.947194
TJP1	W3	PGE2	well_3	F005	468	Z distribution	1.107983	1.202993	0.924832
TJP1	W3	PGE2	well_3	F006	185	Z distribution	1.332533	1.455659	0.561734
TJP1	W3	PGE2	well_4	F001	250	Z distribution	0.808583	0.809977	0.827064
TJP1	W3	PGE2	well_4	F002	418	Z distribution	0.613501	0.568021	0.886777
TJP1	W3	PGE2	well_4	F003	218	Z distribution	0.991844	1.03656	0.61071
TJP1	W3	PGE2	well_4	F004	444	Z distribution	0.72439	0.610253	0.877046
TJP1	W3	PGE2	well_4	F005	419	Z distribution	0.788673	0.76265	1.026057
TJP1	W3	PGE2	well_4	F006	226	Z distribution	1.038302	1.236313	0.883904
TJP1	W3	PGE2	well_5	F001	333	Z distribution	0.699074	0.694884	0.841364
TJP1	W3	PGE2	well_5	F002	314	Z distribution	0.980538	1.187021	0.978855
TJP1	W3	PGE2	well_5	F003	345	Z distribution	0.863772	0.98888	0.869842
TJP1	W3	PGE2	well_5	F004	289	Z distribution	1.135797	1.317455	0.893596
TJP1	W3	PGE2	well_5	F005	414	Z distribution	0.812755	0.827348	0.857579
TJP1	W3	PGE2	well_5	F006	141	Z distribution	0.757128	0.84447	0.691671
TJP1	W4	HPI4	well_1	F001	89	Z distribution	0.097878	0.053318	0.377207
TJP1	W4	HPI4	well_1	F002	154	Z distribution	0.101063	0.134048	0.398004
TJP1	W4	HPI4	well_1	F003	42	Z distribution	-0.19014	-0.13649	0.317705
TJP1	W4	HPI4	well_1	F004	191	Z distribution	-0.08125	-0.04821	0.412148
TJP1	W4	HPI4	well_1	F005	125	Z distribution	-0.06131	-0.01723	0.357833
TJP1	W4	HPI4	well_1	F006	194	Z distribution	-0.03847	-0.04093	0.412685
TJP1	W4	HPI4	well_2	F001	162	Z distribution	0.258186	0.283789	0.420457
TJP1	W4	HPI4	well_2	F002	145	Z distribution	0.235968	0.205712	0.369811
TJP1	W4	HPI4	well_2	F003	133	Z distribution	-0.06234	-0.05258	0.351071
TJP1	W4	HPI4	well_2	F004	111	Z distribution	-0.12607	-0.10997	0.37354
TJP1	W4	HPI4	well_2	F005	128	Z distribution	-0.15571	-0.10754	0.292488
TJP1	W4	HPI4	well_2	F006	136	Z distribution	0.035916	0.054722	0.299645
TJP1	W4	HPI4	well_3	F001	195	Z distribution	0.117246	0.15193	0.351815
TJP1	W4	HPI4	well_3	F002	183	Z distribution	-0.06576	0.008081	0.362055
TJP1	W4	HPI4	well_3	F003	174	Z distribution	-0.02327	-0.01223	0.369633
TJP1	W4	HPI4	well_3	F004	182	Z distribution	0.1935	0.224856	0.426511
TJP1	W4	HPI4	well_3	F005	160	Z distribution	0.099669	0.110631	0.388414
TJP1	W4	HPI4	well_3	F006	28	Z distribution	-0.05159	-0.09366	0.264693
TJP1	W4	HPI4	well_4	F001	136	Z distribution	0.049095	-0.00345	0.391968
TJP1	W4	HPI4	well_4	F002	121	Z distribution	-0.00232	0.014859	0.357173
TJP1	W4	HPI4	well_4	F003	115	Z distribution	-0.05987	-0.0761	0.336738
TJP1	W4	HPI4	well_4	F004	187	Z distribution	-0.00077	-0.02944	0.422949
TJP1	W4	HPI4	well_4	F005	230	Z distribution	0.023575	-0.0214	0.367213
TJP1	W4	HPI4	well_4	F006	103	Z distribution	-0.07704	-0.04847	0.250869
TJP1	W4	HPI4	well_5	F001	174	Z distribution	0.03109	0.052603	0.392368

TJP1	W4	HPI4	well_5	F002	136	Z distribution	0.153007	0.158451	0.346566
TJP1	W4	HPI4	well_5	F003	27	Z distribution	0.343277	0.304713	0.362024
TJP1	W4	HPI4	well_5	F004	197	Z distribution	-0.1318	-0.12859	0.351501
TJP1	W4	HPI4	well_5	F005	190	Z distribution	0.112048	0.057348	0.382418
TJP1	W4	HPI4	well_5	F006	232	Z distribution	0.122458	0.132019	0.492732
TJP1	W4	PGE2	well_1	F001	174	Z distribution	0.714389	0.693305	0.675503
TJP1	W4	PGE2	well_1	F002	117	Z distribution	0.589398	0.752025	0.943845
TJP1	W4	PGE2	well_1	F003	66	Z distribution	1.021301	1.12633	0.697655
TJP1	W4	PGE2	well_1	F004	238	Z distribution	1.018368	1.110286	0.811341
TJP1	W4	PGE2	well_1	F005	389	Z distribution	1.145299	1.247623	0.781944
TJP1	W4	PGE2	well_1	F006	259	Z distribution	0.974614	1.061613	0.695795
TJP1	W4	PGE2	well_2	F001	129	Z distribution	0.5975	0.621679	0.479348
TJP1	W4	PGE2	well_2	F002	174	Z distribution	0.513241	0.548257	0.487727
TJP1	W4	PGE2	well_2	F003	190	Z distribution	0.518862	0.413499	0.775146
TJP1	W4	PGE2	well_2	F004	279	Z distribution	1.020474	1.111118	0.93901
TJP1	W4	PGE2	well_2	F005	366	Z distribution	0.894657	0.792016	0.911699
TJP1	W4	PGE2	well_2	F006	257	Z distribution	0.959885	1.057425	0.838231
TJP1	W4	PGE2	well_3	F001	63	Z distribution	0.961653	0.993365	0.57497
TJP1	W4	PGE2	well_3	F002	418	Z distribution	1.741219	1.849013	0.823007
TJP1	W4	PGE2	well_3	F003	344	Z distribution	0.819963	0.8933	0.726015
TJP1	W4	PGE2	well_3	F004	364	Z distribution	0.976845	1.177681	1.044169
TJP1	W4	PGE2	well_3	F005	149	Z distribution	1.458984	1.574287	0.6509
TJP1	W4	PGE2	well_3	F006	333	Z distribution	1.314384	1.486483	0.880805
TJP1	W4	PGE2	well_4	F001	295	Z distribution	1.188593	1.458918	1.059053
TJP1	W4	PGE2	well_4	F002	280	Z distribution	0.96339	1.101199	0.883234
TJP1	W4	PGE2	well_4	F003	189	Z distribution	0.16828	0.14242	0.902216
TJP1	W4	PGE2	well_4	F004	198	Z distribution	1.137773	1.209124	0.644765
TJP1	W4	PGE2	well_4	F005	273	Z distribution	0.727917	0.786474	0.897569
TJP1	W4	PGE2	well_4	F006	209	Z distribution	0.814523	0.888361	0.694587
TJP1	W4	PGE2	well_5	F001	295	Z distribution	1.093954	1.231366	0.903566
TJP1	W4	PGE2	well_5	F002	185	Z distribution	1.33141	1.371217	0.617918
TJP1	W4	PGE2	well_5	F003	303	Z distribution	0.781888	0.889146	0.905479
TJP1	W4	PGE2	well_5	F004	319	Z distribution	1.108533	1.330429	0.935616
TJP1	W4	PGE2	well_5	F005	289	Z distribution	1.128296	1.392573	1.007274
TJP1	W4	PGE2	well_5	F006	244	Z distribution	0.87142	0.961372	1.04493
CTNNB1	W1	HPI4	well_1	F001	91	Avg. Volume	2.662127	2.550046	1.046662
CTNNB1	W1	HPI4	well_1	F002	73	Avg. Volume	2.756665	2.410858	1.48014
CTNNB1	W1	HPI4	well_1	F003	164	Avg. Volume	3.838574	3.377511	2.051643
CTNNB1	W1	HPI4	well_1	F004	154	Avg. Volume	3.461687	3.187045	1.747538
CTNNB1	W1	HPI4	well_1	F005	155	Avg. Volume	3.356368	2.846007	1.669289
CTNNB1	W1	HPI4	well_1	F006	135	Avg. Volume	2.826261	2.377649	1.601269
CTNNB1	W1	HPI4	well_2	F001	169	Avg. Volume	2.944222	2.767114	1.437647
CTNNB1	W1	HPI4	well_2	F002	212	Avg. Volume	3.434511	3.08995	1.780126
CTNNB1	W1	HPI4	well_2	F003	128	Avg. Volume	2.706032	2.384506	1.374822
CTNNB1	W1	HPI4	well_2	F004	168	Avg. Volume	2.860204	2.696828	1.307773
CTNNB1	W1	HPI4	well_2	F005	228	Avg. Volume	3.387294	3.045784	1.454767
CTNNB1	W1	HPI4	well_2	F006	121	Avg. Volume	2.853337	2.482562	1.445302
CTNNB1	W1	HPI4	well_3	F001	126	Avg. Volume	4.112208	3.815441	2.065345
CTNNB1	W1	HPI4	well_3	F002	206	Avg. Volume	3.541784	3.057078	1.763066

CTNNB1	W1	HPI4	well_3	F003	72	Avg. Volume	2.476783	2.221059	1.31529
CTNNB1	W1	HPI4	well_3	F004	148	Avg. Volume	2.81642	2.490403	1.467678
CTNNB1	W1	HPI4	well_3	F005	121	Avg. Volume	3.222172	2.521733	2.104012
CTNNB1	W1	HPI4	well_3	F006	34	Avg. Volume	2.957573	2.258463	1.96464
CTNNB1	W1	HPI4	well_4	F001	76	Avg. Volume	2.587559	2.333855	1.29652
CTNNB1	W1	HPI4	well_4	F002	69	Avg. Volume	3.221172	2.94677	1.648275
CTNNB1	W1	HPI4	well_4	F003	76	Avg. Volume	3.607941	3.215741	1.971632
CTNNB1	W1	HPI4	well_4	F004	108	Avg. Volume	3.112519	2.694127	1.664111
CTNNB1	W1	HPI4	well_4	F005	137	Avg. Volume	3.744679	2.912023	2.532839
CTNNB1	W1	HPI4	well_4	F006	118	Avg. Volume	2.927516	2.798734	1.281704
CTNNB1	W1	HPI4	well_5	F001	74	Avg. Volume	3.014993	2.612458	1.565449
CTNNB1	W1	HPI4	well_5	F002	202	Avg. Volume	3.935027	3.38455	2.275481
CTNNB1	W1	HPI4	well_5	F003	105	Avg. Volume	2.258196	1.980469	1.022685
CTNNB1	W1	HPI4	well_5	F004	134	Avg. Volume	2.419845	2.086306	1.278584
CTNNB1	W1	HPI4	well_5	F005	117	Avg. Volume	2.061994	1.907569	0.929003
CTNNB1	W1	HPI4	well_5	F006	164	Avg. Volume	3.219627	2.7885	1.610464
CTNNB1	W1	PGE2	well_1	F001	162	Avg. Volume	3.162878	2.795589	1.621796
CTNNB1	W1	PGE2	well_1	F002	155	Avg. Volume	3.490332	3.228048	1.811739
CTNNB1	W1	PGE2	well_1	F003	202	Avg. Volume	3.715182	3.134685	2.327418
CTNNB1	W1	PGE2	well_1	F004	166	Avg. Volume	3.95895	3.442384	2.069306
CTNNB1	W1	PGE2	well_1	F005	69	Avg. Volume	3.159714	2.72692	1.437129
CTNNB1	W1	PGE2	well_1	F006	227	Avg. Volume	4.176447	3.532135	2.229075
CTNNB1	W1	PGE2	well_2	F001	134	Avg. Volume	4.620183	4.243056	2.869756
CTNNB1	W1	PGE2	well_2	F002	138	Avg. Volume	3.108251	2.594424	1.756372
CTNNB1	W1	PGE2	well_2	F003	107	Avg. Volume	3.436141	2.899886	2.138872
CTNNB1	W1	PGE2	well_2	F004	177	Avg. Volume	4.281104	3.307497	2.903854
CTNNB1	W1	PGE2	well_2	F005	47	Avg. Volume	2.660779	2.355366	1.546093
CTNNB1	W1	PGE2	well_2	F006	80	Avg. Volume	3.119537	2.530149	1.866764
CTNNB1	W1	PGE2	well_3	F001	106	Avg. Volume	2.732586	2.283841	1.787327
CTNNB1	W1	PGE2	well_3	F002	70	Avg. Volume	2.895942	2.381194	1.780314
CTNNB1	W1	PGE2	well_3	F003	152	Avg. Volume	3.717371	3.188966	2.0747
CTNNB1	W1	PGE2	well_3	F004	95	Avg. Volume	2.937966	2.609203	1.445734
CTNNB1	W1	PGE2	well_3	F005	100	Avg. Volume	3.269211	2.840161	1.870106
CTNNB1	W1	PGE2	well_3	F006	118	Avg. Volume	3.482769	3.019161	1.869066
CTNNB1	W1	PGE2	well_4	F001	39	Avg. Volume	2.590336	1.907118	1.604617
CTNNB1	W1	PGE2	well_4	F002	96	Avg. Volume	3.729297	3.078418	2.028926
CTNNB1	W1	PGE2	well_4	F003	111	Avg. Volume	3.462983	2.976429	1.899933
CTNNB1	W1	PGE2	well_4	F004	202	Avg. Volume	3.229682	3.028373	1.441105
CTNNB1	W1	PGE2	well_4	F005	75	Avg. Volume	2.451108	2.081678	1.032837
CTNNB1	W1	PGE2	well_4	F006	63	Avg. Volume	2.988722	2.637917	1.469372
CTNNB1	W1	PGE2	well_5	F001	136	Avg. Volume	3.532246	3.131724	1.640446
CTNNB1	W1	PGE2	well_5	F002	137	Avg. Volume	2.805859	2.598815	1.305979
CTNNB1	W1	PGE2	well_5	F003	70	Avg. Volume	2.109151	1.879702	0.863051
CTNNB1	W1	PGE2	well_5	F004	87	Avg. Volume	2.341358	2.133457	1.117961
CTNNB1	W1	PGE2	well_5	F005	83	Avg. Volume	2.444153	2.170825	1.185019
CTNNB1	W1	PGE2	well_5	F006	89	Avg. Volume	3.613168	3.371998	1.316864
CTNNB1	W2	HPI4	well_1	F001	7	Avg. Volume	2.544847	2.382989	0.601353
CTNNB1	W2	HPI4	well_1	F005	3	Avg. Volume	1.739552	1.739552	NA
CTNNB1	W2	HPI4	well_2	F001	1	Avg. Volume	1.453974	1.453974	NA

CTNNB1	W2	HPI4	well_2	F005	3	Avg. Volume	0.995436	0.995436	NA
CTNNB1	W2	HPI4	well_2	F006	3	Avg. Volume	2.48284	2.48284	NA
CTNNB1	W2	HPI4	well_3	F001	1	Avg. Volume	8.373715	8.373715	NA
CTNNB1	W2	HPI4	well_3	F002	4	Avg. Volume	5.091289	5.091289	2.623627
CTNNB1	W2	HPI4	well_3	F005	8	Avg. Volume	4.526296	4.313384	2.188953
CTNNB1	W2	HPI4	well_3	F006	3	Avg. Volume	0.532819	0.532819	NA
CTNNB1	W2	HPI4	well_4	F001	5	Avg. Volume	0.713025	0.766759	0.256184
CTNNB1	W2	HPI4	well_4	F003	1	Avg. Volume	1.955236	1.955236	NA
CTNNB1	W2	HPI4	well_4	F005	1	Avg. Volume	2.023306	2.023306	NA
CTNNB1	W2	HPI4	well_5	F004	6	Avg. Volume	0.719574	0.785727	0.167916
CTNNB1	W2	HPI4	well_5	F005	78	Avg. Volume	1.1469	0.798132	0.944745
CTNNB1	W2	HPI4	well_5	F006	11	Avg. Volume	1.506409	1.48575	0.559173
CTNNB1	W2	PGE2	well_1	F001	91	Avg. Volume	2.062605	1.640922	1.389115
CTNNB1	W2	PGE2	well_1	F002	67	Avg. Volume	3.428246	2.783659	2.698894
CTNNB1	W2	PGE2	well_1	F003	74	Avg. Volume	2.670201	2.440476	1.521063
CTNNB1	W2	PGE2	well_1	F004	96	Avg. Volume	3.690898	2.874589	2.39948
CTNNB1	W2	PGE2	well_1	F005	95	Avg. Volume	3.68927	3.306132	2.079486
CTNNB1	W2	PGE2	well_1	F006	121	Avg. Volume	3.012433	2.306679	2.091593
CTNNB1	W2	PGE2	well_2	F001	117	Avg. Volume	4.00407	3.437377	2.330874
CTNNB1	W2	PGE2	well_2	F002	91	Avg. Volume	4.264477	3.622546	2.261801
CTNNB1	W2	PGE2	well_2	F003	161	Avg. Volume	3.764225	3.191283	1.911847
CTNNB1	W2	PGE2	well_2	F004	91	Avg. Volume	4.49059	3.556944	2.922649
CTNNB1	W2	PGE2	well_2	F005	68	Avg. Volume	3.559264	3.128721	1.936301
CTNNB1	W2	PGE2	well_2	F006	141	Avg. Volume	3.161683	2.687214	1.710183
CTNNB1	W2	PGE2	well_3	F001	88	Avg. Volume	4.931928	4.027966	3.090704
CTNNB1	W2	PGE2	well_3	F002	50	Avg. Volume	4.495802	3.88918	2.238082
CTNNB1	W2	PGE2	well_3	F003	24	Avg. Volume	3.800116	3.779497	2.265259
CTNNB1	W2	PGE2	well_3	F004	42	Avg. Volume	3.769378	3.163761	2.108756
CTNNB1	W2	PGE2	well_3	F005	51	Avg. Volume	2.406858	1.630341	2.102579
CTNNB1	W2	PGE2	well_3	F006	34	Avg. Volume	5.193367	4.425066	4.017384
CTNNB1	W2	PGE2	well_4	F001	10	Avg. Volume	2.990495	2.981196	1.112655
CTNNB1	W2	PGE2	well_4	F002	8	Avg. Volume	2.231237	1.520517	2.109294
CTNNB1	W2	PGE2	well_4	F003	10	Avg. Volume	3.176036	3.17113	1.940516
CTNNB1	W2	PGE2	well_4	F004	73	Avg. Volume	3.2963	3.018889	2.189774
CTNNB1	W2	PGE2	well_4	F005	66	Avg. Volume	4.428933	3.722173	3.52432
CTNNB1	W2	PGE2	well_4	F006	24	Avg. Volume	5.28938	4.467902	2.87119
CTNNB1	W2	PGE2	well_5	F001	3	Avg. Volume	1.49179	1.49179	NA
CTNNB1	W2	PGE2	well_5	F002	27	Avg. Volume	3.892851	3.883349	1.772561
CTNNB1	W2	PGE2	well_5	F003	73	Avg. Volume	3.053061	2.755378	1.388777
CTNNB1	W2	PGE2	well_5	F004	38	Avg. Volume	6.126794	5.179146	4.049909
CTNNB1	W2	PGE2	well_5	F005	65	Avg. Volume	3.689453	3.312517	1.978783
CTNNB1	W2	PGE2	well_5	F006	41	Avg. Volume	5.296275	3.865875	4.264844
CTNNB1	W3	HPI4	well_1	F001	17	Avg. Volume	3.394273	3.189377	1.646533
CTNNB1	W3	HPI4	well_1	F005	1	Avg. Volume	1.14388	1.14388	NA
CTNNB1	W3	HPI4	well_1	F006	1	Avg. Volume	1.89473	1.89473	NA
CTNNB1	W3	HPI4	well_2	F001	12	Avg. Volume	0.873606	0.566016	0.844025
CTNNB1	W3	HPI4	well_2	F004	1	Avg. Volume	0.336895	0.336895	NA
CTNNB1	W3	HPI4	well_2	F006	6	Avg. Volume	0.813672	0.753182	0.323979
CTNNB1	W3	HPI4	well_3	F001	66	Avg. Volume	2.449101	2.098097	1.38983

CTNNB1	W3	HPI4	well_3	F002	1	Avg. Volume	1.610642	1.610642	NA
CTNNB1	W3	HPI4	well_3	F003	1	Avg. Volume	1.025682	1.025682	NA
CTNNB1	W3	HPI4	well_3	F004	3	Avg. Volume	12.13983	12.13983	NA
CTNNB1	W3	HPI4	well_3	F005	4	Avg. Volume	0.793948	0.793948	0.047302
CTNNB1	W3	HPI4	well_4	F001	30	Avg. Volume	3.205927	2.754963	1.981454
CTNNB1	W3	HPI4	well_5	F001	3	Avg. Volume	1.664394	1.664394	NA
CTNNB1	W3	HPI4	well_5	F005	8	Avg. Volume	1.579592	1.465763	0.485298
CTNNB1	W3	HPI4	well_5	F006	7	Avg. Volume	1.255889	1.20633	0.616939
CTNNB1	W3	PGE2	well_1	F001	85	Avg. Volume	1.7533	1.692272	0.714107
CTNNB1	W3	PGE2	well_1	F002	138	Avg. Volume	1.418051	1.259249	0.613621
CTNNB1	W3	PGE2	well_1	F003	91	Avg. Volume	1.45349	1.344623	0.572309
CTNNB1	W3	PGE2	well_1	F004	45	Avg. Volume	1.376967	1.209797	0.600646
CTNNB1	W3	PGE2	well_1	F005	144	Avg. Volume	1.569401	1.452466	0.716345
CTNNB1	W3	PGE2	well_1	F006	138	Avg. Volume	2.223555	1.869625	1.216355
CTNNB1	W3	PGE2	well_2	F001	248	Avg. Volume	1.701667	1.532823	0.956526
CTNNB1	W3	PGE2	well_2	F002	175	Avg. Volume	2.423536	2.185345	1.219433
CTNNB1	W3	PGE2	well_2	F003	190	Avg. Volume	2.294852	1.893595	1.462706
CTNNB1	W3	PGE2	well_2	F004	137	Avg. Volume	1.610058	1.47943	0.670185
CTNNB1	W3	PGE2	well_2	F005	220	Avg. Volume	2.269951	1.99983	1.208373
CTNNB1	W3	PGE2	well_2	F006	276	Avg. Volume	2.493579	2.209796	1.39823
CTNNB1	W3	PGE2	well_3	F001	181	Avg. Volume	1.771563	1.636227	0.848096
CTNNB1	W3	PGE2	well_3	F002	217	Avg. Volume	1.354742	1.272194	0.551689
CTNNB1	W3	PGE2	well_3	F003	219	Avg. Volume	1.37535	1.209602	0.64064
CTNNB1	W3	PGE2	well_3	F004	217	Avg. Volume	1.337343	1.222756	0.595767
CTNNB1	W3	PGE2	well_3	F005	233	Avg. Volume	1.593997	1.37213	0.819284
CTNNB1	W3	PGE2	well_3	F006	201	Avg. Volume	1.934246	1.777906	0.784803
CTNNB1	W3	PGE2	well_4	F001	102	Avg. Volume	2.105571	1.905938	0.921346
CTNNB1	W3	PGE2	well_4	F002	192	Avg. Volume	1.714725	1.448549	0.915776
CTNNB1	W3	PGE2	well_4	F003	193	Avg. Volume	1.935306	1.712168	0.966912
CTNNB1	W3	PGE2	well_4	F004	200	Avg. Volume	1.254942	1.116044	0.628784
CTNNB1	W3	PGE2	well_4	F005	224	Avg. Volume	1.629681	1.371113	0.928605
CTNNB1	W3	PGE2	well_4	F006	233	Avg. Volume	1.732638	1.563945	0.949437
CTNNB1	W3	PGE2	well_5	F001	64	Avg. Volume	1.799559	1.671377	0.820165
CTNNB1	W3	PGE2	well_5	F002	129	Avg. Volume	1.172018	1.052897	0.529062
CTNNB1	W3	PGE2	well_5	F003	75	Avg. Volume	1.350524	1.229459	0.637572
CTNNB1	W3	PGE2	well_5	F004	95	Avg. Volume	0.835882	0.722162	0.442772
CTNNB1	W3	PGE2	well_5	F005	151	Avg. Volume	1.639681	1.376239	0.869188
CTNNB1	W3	PGE2	well_5	F006	233	Avg. Volume	2.170544	1.889179	1.27429
CTNNB1	W4	HPI4	well_1	F004	5	Avg. Volume	0.738318	0.8556	0.207952
CTNNB1	W4	HPI4	well_1	F005	17	Avg. Volume	1.029995	0.974097	0.441433
CTNNB1	W4	HPI4	well_1	F006	12	Avg. Volume	0.893871	0.830634	0.455094
CTNNB1	W4	HPI4	well_2	F001	8	Avg. Volume	1.047922	1.245658	0.552461
CTNNB1	W4	HPI4	well_2	F002	1	Avg. Volume	0.477073	0.477073	NA
CTNNB1	W4	HPI4	well_2	F003	7	Avg. Volume	1.080199	1.115621	0.254329
CTNNB1	W4	HPI4	well_2	F004	4	Avg. Volume	1.545575	1.545575	0.105965
CTNNB1	W4	HPI4	well_2	F005	8	Avg. Volume	0.76915	0.763311	0.30765
CTNNB1	W4	HPI4	well_2	F006	7	Avg. Volume	0.872943	0.928012	0.138666
CTNNB1	W4	HPI4	well_3	F001	4	Avg. Volume	0.536176	0.536176	0.001807
CTNNB1	W4	HPI4	well_3	F002	6	Avg. Volume	0.659043	0.657563	0.231775

CTNNB1	W4	HPI4	well_3	F003	1	Avg. Volume	9.614426	9.614426	NA
CTNNB1	W4	HPI4	well_3	F004	9	Avg. Volume	0.920788	0.882459	0.230534
CTNNB1	W4	HPI4	well_3	F005	1	Avg. Volume	1.503187	1.503187	NA
CTNNB1	W4	HPI4	well_3	F006	5	Avg. Volume	0.705038	0.659206	0.082993
CTNNB1	W4	HPI4	well_4	F001	13	Avg. Volume	0.53037	0.416098	0.26664
CTNNB1	W4	HPI4	well_4	F002	3	Avg. Volume	1.872088	1.872088	NA
CTNNB1	W4	HPI4	well_4	F004	14	Avg. Volume	0.795846	0.844995	0.305822
CTNNB1	W4	HPI4	well_4	F006	5	Avg. Volume	1.272654	1.505491	0.40918
CTNNB1	W4	HPI4	well_5	F001	3	Avg. Volume	1.235125	1.235125	NA
CTNNB1	W4	HPI4	well_5	F003	13	Avg. Volume	1.00226	0.947887	0.378238
CTNNB1	W4	HPI4	well_5	F004	42	Avg. Volume	1.428079	1.446205	0.462825
CTNNB1	W4	HPI4	well_5	F005	8	Avg. Volume	0.893729	0.838894	0.154413
CTNNB1	W4	HPI4	well_5	F006	14	Avg. Volume	1.538837	1.531189	0.675061
CTNNB1	W4	PGE2	well_1	F001	28	Avg. Volume	2.070502	1.481953	1.700724
CTNNB1	W4	PGE2	well_1	F002	113	Avg. Volume	2.37467	2.167416	1.061583
CTNNB1	W4	PGE2	well_1	F003	89	Avg. Volume	2.288012	1.718101	1.755302
CTNNB1	W4	PGE2	well_1	F004	58	Avg. Volume	1.631618	1.311009	0.824427
CTNNB1	W4	PGE2	well_1	F005	43	Avg. Volume	2.041974	1.429672	1.267696
CTNNB1	W4	PGE2	well_1	F006	53	Avg. Volume	1.474618	1.417722	0.606062
CTNNB1	W4	PGE2	well_2	F001	79	Avg. Volume	1.935575	1.556502	1.123346
CTNNB1	W4	PGE2	well_2	F002	218	Avg. Volume	1.711851	1.503202	0.907422
CTNNB1	W4	PGE2	well_2	F003	141	Avg. Volume	2.184901	1.872469	1.20182
CTNNB1	W4	PGE2	well_2	F004	156	Avg. Volume	1.755088	1.53566	0.886967
CTNNB1	W4	PGE2	well_2	F005	184	Avg. Volume	1.369459	1.192914	0.760214
CTNNB1	W4	PGE2	well_2	F006	145	Avg. Volume	1.841089	1.56237	1.025656
CTNNB1	W4	PGE2	well_3	F001	186	Avg. Volume	1.374792	1.154677	0.770303
CTNNB1	W4	PGE2	well_3	F002	301	Avg. Volume	1.445859	1.207841	0.829368
CTNNB1	W4	PGE2	well_3	F003	288	Avg. Volume	1.736942	1.614324	0.771694
CTNNB1	W4	PGE2	well_3	F004	196	Avg. Volume	1.468183	1.275637	0.667351
CTNNB1	W4	PGE2	well_3	F005	251	Avg. Volume	1.503051	1.244615	0.822518
CTNNB1	W4	PGE2	well_3	F006	240	Avg. Volume	1.564539	1.344193	0.794903
CTNNB1	W4	PGE2	well_4	F001	119	Avg. Volume	2.116101	1.636209	1.339872
CTNNB1	W4	PGE2	well_4	F002	189	Avg. Volume	1.652821	1.379831	0.985643
CTNNB1	W4	PGE2	well_4	F003	176	Avg. Volume	1.805952	1.640417	0.823856
CTNNB1	W4	PGE2	well_4	F004	114	Avg. Volume	1.582531	1.234941	0.958477
CTNNB1	W4	PGE2	well_4	F005	207	Avg. Volume	1.346207	1.129182	0.683781
CTNNB1	W4	PGE2	well_4	F006	268	Avg. Volume	1.465283	1.264056	0.813389
CTNNB1	W4	PGE2	well_5	F001	110	Avg. Volume	1.726473	1.464085	0.973521
CTNNB1	W4	PGE2	well_5	F002	188	Avg. Volume	1.273664	1.126262	0.650119
CTNNB1	W4	PGE2	well_5	F003	189	Avg. Volume	1.414476	1.053207	0.924833
CTNNB1	W4	PGE2	well_5	F004	152	Avg. Volume	1.469354	1.098779	0.894212
CTNNB1	W4	PGE2	well_5	F005	243	Avg. Volume	1.296418	1.117763	0.755731
CTNNB1	W4	PGE2	well_5	F006	200	Avg. Volume	1.383325	1.241196	0.689843
CTNNB1	W1	HPI4	well_1	F001	91	Count/cell	36.7439	35	17.9573
CTNNB1	W1	HPI4	well_1	F002	73	Count/cell	29.91045	29	15.35019
CTNNB1	W1	HPI4	well_1	F003	164	Count/cell	24.16438	22.5	11.65364
CTNNB1	W1	HPI4	well_1	F004	154	Count/cell	30.55396	30	13.24582
CTNNB1	W1	HPI4	well_1	F005	155	Count/cell	33.02878	30	14.59548
CTNNB1	W1	HPI4	well_1	F006	135	Count/cell	31.29508	30.5	14.34964

CTNNB1	W1	HPI4	well_2	F001	169	Count/cell	29.39474	26	14.0067
CTNNB1	W1	HPI4	well_2	F002	212	Count/cell	29.19372	28	12.31085
CTNNB1	W1	HPI4	well_2	F003	128	Count/cell	40.07018	38	15.63223
CTNNB1	W1	HPI4	well_2	F004	168	Count/cell	32.37748	30	14.49264
CTNNB1	W1	HPI4	well_2	F005	228	Count/cell	40.82524	39.5	16.24413
CTNNB1	W1	HPI4	well_2	F006	121	Count/cell	31.96364	31	13.38555
CTNNB1	W1	HPI4	well_3	F001	126	Count/cell	27.46957	25	13.61393
CTNNB1	W1	HPI4	well_3	F002	206	Count/cell	32.94054	32	14.1399
CTNNB1	W1	HPI4	well_3	F003	72	Count/cell	31.6875	31.5	14.64379
CTNNB1	W1	HPI4	well_3	F004	148	Count/cell	37.62406	34	17.58512
CTNNB1	W1	HPI4	well_3	F005	121	Count/cell	31.50442	30	16.03017
CTNNB1	W1	HPI4	well_3	F006	34	Count/cell	30.19355	25	18.54619
CTNNB1	W1	HPI4	well_4	F001	76	Count/cell	29.91176	30.5	14.8653
CTNNB1	W1	HPI4	well_4	F002	69	Count/cell	29.93443	28	14.87825
CTNNB1	W1	HPI4	well_4	F003	76	Count/cell	27.68571	28	13.17721
CTNNB1	W1	HPI4	well_4	F004	108	Count/cell	29.07292	27	14.21878
CTNNB1	W1	HPI4	well_4	F005	137	Count/cell	30.63415	30	14.39391
CTNNB1	W1	HPI4	well_4	F006	118	Count/cell	31.8785	29	13.32023
CTNNB1	W1	HPI4	well_5	F001	74	Count/cell	29.27273	27	12.1168
CTNNB1	W1	HPI4	well_5	F002	202	Count/cell	28.45	25	14.96375
CTNNB1	W1	HPI4	well_5	F003	105	Count/cell	45.90323	45	16.54379
CTNNB1	W1	HPI4	well_5	F004	134	Count/cell	42.01667	43	18.8586
CTNNB1	W1	HPI4	well_5	F005	117	Count/cell	47.9619	45	22.37956
CTNNB1	W1	HPI4	well_5	F006	164	Count/cell	29.62838	27	13.57082
CTNNB1	W1	PGE2	well_1	F001	162	Count/cell	36.94483	35	15.52005
CTNNB1	W1	PGE2	well_1	F002	155	Count/cell	30.03546	27	13.76248
CTNNB1	W1	PGE2	well_1	F003	202	Count/cell	26.33149	23	13.42098
CTNNB1	W1	PGE2	well_1	F004	166	Count/cell	28.94595	27.5	13.03412
CTNNB1	W1	PGE2	well_1	F005	69	Count/cell	30.44444	25	15.67799
CTNNB1	W1	PGE2	well_1	F006	227	Count/cell	29.74757	28	13.74345
CTNNB1	W1	PGE2	well_2	F001	134	Count/cell	23.95833	23	11.019
CTNNB1	W1	PGE2	well_2	F002	138	Count/cell	28.48	25	15.09946
CTNNB1	W1	PGE2	well_2	F003	107	Count/cell	30.44792	29.5	14.03145
CTNNB1	W1	PGE2	well_2	F004	177	Count/cell	27.31056	26	13.86824
CTNNB1	W1	PGE2	well_2	F005	47	Count/cell	40.65854	37	17.54937
CTNNB1	W1	PGE2	well_2	F006	80	Count/cell	31.05405	27	18.38619
CTNNB1	W1	PGE2	well_3	F001	106	Count/cell	32.92553	30.5	16.19985
CTNNB1	W1	PGE2	well_3	F002	70	Count/cell	34.39683	35	13.25743
CTNNB1	W1	PGE2	well_3	F003	152	Count/cell	27.81618	28	12.44913
CTNNB1	W1	PGE2	well_3	F004	95	Count/cell	23.11628	22	9.046831
CTNNB1	W1	PGE2	well_3	F005	100	Count/cell	32.27473	30	14.94736
CTNNB1	W1	PGE2	well_3	F006	118	Count/cell	30.8972	29	13.12346
CTNNB1	W1	PGE2	well_4	F001	39	Count/cell	34.48571	32	16.83723
CTNNB1	W1	PGE2	well_4	F002	96	Count/cell	27.70455	26	12.75693
CTNNB1	W1	PGE2	well_4	F003	111	Count/cell	26.09091	27	12.72238
CTNNB1	W1	PGE2	well_4	F004	202	Count/cell	30.87845	30	13.03187
CTNNB1	W1	PGE2	well_4	F005	75	Count/cell	35.98529	34	14.56687
CTNNB1	W1	PGE2	well_4	F006	63	Count/cell	30.98182	29	15.14925
CTNNB1	W1	PGE2	well_5	F001	136	Count/cell	29.94309	27	14.09936

CTNNB1	W1	PGE2	well_5	F002	137	Count/cell	38.41129	36	16.16933
CTNNB1	W1	PGE2	well_5	F003	70	Count/cell	41.67188	40.5	18.23572
CTNNB1	W1	PGE2	well_5	F004	87	Count/cell	33.02564	30.5	14.79204
CTNNB1	W1	PGE2	well_5	F005	83	Count/cell	34.45946	34.5	14.68966
CTNNB1	W1	PGE2	well_5	F006	89	Count/cell	27.5875	28	10.50701
CTNNB1	W2	HPI4	well_1	F001	7	Count/cell	29.6	32	10.78425
CTNNB1	W2	HPI4	well_1	F005	3	Count/cell	26	26	NA
CTNNB1	W2	HPI4	well_2	F001	1	Count/cell	18	18	NA
CTNNB1	W2	HPI4	well_2	F005	3	Count/cell	11	11	NA
CTNNB1	W2	HPI4	well_2	F006	3	Count/cell	18	18	NA
CTNNB1	W2	HPI4	well_3	F001	1	Count/cell	16	16	NA
CTNNB1	W2	HPI4	well_3	F002	4	Count/cell	20.5	20.5	3.535534
CTNNB1	W2	HPI4	well_3	F005	8	Count/cell	26.66667	27	2.94392
CTNNB1	W2	HPI4	well_3	F006	3	Count/cell	30	30	NA
CTNNB1	W2	HPI4	well_4	F001	5	Count/cell	7.5	5	7.549834
CTNNB1	W2	HPI4	well_4	F003	1	Count/cell	30	30	NA
CTNNB1	W2	HPI4	well_4	F005	1	Count/cell	15	15	NA
CTNNB1	W2	HPI4	well_5	F004	6	Count/cell	31	32.5	15.38397
CTNNB1	W2	HPI4	well_5	F005	78	Count/cell	10.78378	9	6.617267
CTNNB1	W2	HPI4	well_5	F006	11	Count/cell	44	44	13.57387
CTNNB1	W2	PGE2	well_1	F001	91	Count/cell	20.70732	18.5	11.56569
CTNNB1	W2	PGE2	well_1	F002	67	Count/cell	18.96721	14	14.37471
CTNNB1	W2	PGE2	well_1	F003	74	Count/cell	29.04478	25	14.47509
CTNNB1	W2	PGE2	well_1	F004	96	Count/cell	26.04651	22	16.46666
CTNNB1	W2	PGE2	well_1	F005	95	Count/cell	26.24419	23.5	14.61018
CTNNB1	W2	PGE2	well_1	F006	121	Count/cell	21.2	18	11.74484
CTNNB1	W2	PGE2	well_2	F001	117	Count/cell	21.42857	20	10.67605
CTNNB1	W2	PGE2	well_2	F002	91	Count/cell	22.87654	21	11.63119
CTNNB1	W2	PGE2	well_2	F003	161	Count/cell	27.72414	26	13.10825
CTNNB1	W2	PGE2	well_2	F004	91	Count/cell	19.39286	18	10.56594
CTNNB1	W2	PGE2	well_2	F005	68	Count/cell	22.86885	21	11.79332
CTNNB1	W2	PGE2	well_2	F006	141	Count/cell	28.08527	26	12.98559
CTNNB1	W2	PGE2	well_3	F001	88	Count/cell	13.88462	12	8.865489
CTNNB1	W2	PGE2	well_3	F002	50	Count/cell	19.72727	14.5	13.61491
CTNNB1	W2	PGE2	well_3	F003	24	Count/cell	18.05	13	13.21672
CTNNB1	W2	PGE2	well_3	F004	42	Count/cell	21.22222	16.5	11.31146
CTNNB1	W2	PGE2	well_3	F005	51	Count/cell	13.47826	10.5	9.472156
CTNNB1	W2	PGE2	well_3	F006	34	Count/cell	12.56667	12	7.518636
CTNNB1	W2	PGE2	well_4	F001	10	Count/cell	17.875	15	9.264949
CTNNB1	W2	PGE2	well_4	F002	8	Count/cell	19.16667	16.5	10.18659
CTNNB1	W2	PGE2	well_4	F003	10	Count/cell	19	18.5	12.80625
CTNNB1	W2	PGE2	well_4	F004	73	Count/cell	25.26154	23	16.27237
CTNNB1	W2	PGE2	well_4	F005	66	Count/cell	16.17241	14.5	10.75588
CTNNB1	W2	PGE2	well_4	F006	24	Count/cell	15.05	13.5	9.811245
CTNNB1	W2	PGE2	well_5	F001	3	Count/cell	8	8	NA
CTNNB1	W2	PGE2	well_5	F002	27	Count/cell	14.25	13	6.001811
CTNNB1	W2	PGE2	well_5	F003	73	Count/cell	22.34328	18	13.08892
CTNNB1	W2	PGE2	well_5	F004	38	Count/cell	11.4	6	10.53342
CTNNB1	W2	PGE2	well_5	F005	65	Count/cell	18.2931	17.5	8.33721

CTNNB1	W2	PGE2	well_5	F006	41	Count/cell	19.75676	18	13.95908
CTNNB1	W3	HPI4	well_1	F001	17	Count/cell	24	25	6.866066
CTNNB1	W3	HPI4	well_1	F005	1	Count/cell	15	15	NA
CTNNB1	W3	HPI4	well_1	F006	1	Count/cell	18	18	NA
CTNNB1	W3	HPI4	well_2	F001	12	Count/cell	10.54545	7	5.106146
CTNNB1	W3	HPI4	well_2	F004	1	Count/cell	17	17	NA
CTNNB1	W3	HPI4	well_2	F006	6	Count/cell	25.75	27.5	12.44655
CTNNB1	W3	HPI4	well_3	F001	66	Count/cell	23.46552	22	9.291036
CTNNB1	W3	HPI4	well_3	F002	1	Count/cell	21	21	NA
CTNNB1	W3	HPI4	well_3	F003	1	Count/cell	43	43	NA
CTNNB1	W3	HPI4	well_3	F004	3	Count/cell	12	12	NA
CTNNB1	W3	HPI4	well_3	F005	4	Count/cell	17.5	17.5	7.778175
CTNNB1	W3	HPI4	well_4	F001	30	Count/cell	19.61538	16	8.832109
CTNNB1	W3	HPI4	well_5	F001	3	Count/cell	11	11	NA
CTNNB1	W3	HPI4	well_5	F005	8	Count/cell	49.16667	47.5	12.93703
CTNNB1	W3	HPI4	well_5	F006	7	Count/cell	34.4	32	5.770615
CTNNB1	W3	PGE2	well_1	F001	85	Count/cell	40.04	40	16.93185
CTNNB1	W3	PGE2	well_1	F002	138	Count/cell	33.90323	30.5	12.93223
CTNNB1	W3	PGE2	well_1	F003	91	Count/cell	32.96471	31	14.30821
CTNNB1	W3	PGE2	well_1	F004	45	Count/cell	33.66667	37	14.88258
CTNNB1	W3	PGE2	well_1	F005	144	Count/cell	43.96875	43.5	16.63264
CTNNB1	W3	PGE2	well_1	F006	138	Count/cell	50.32258	45	27.19474
CTNNB1	W3	PGE2	well_2	F001	248	Count/cell	39.06757	37	16.88663
CTNNB1	W3	PGE2	well_2	F002	175	Count/cell	38.71338	36	15.70279
CTNNB1	W3	PGE2	well_2	F003	190	Count/cell	30.74138	30	15.39375
CTNNB1	W3	PGE2	well_2	F004	137	Count/cell	31.71545	32	12.83982
CTNNB1	W3	PGE2	well_2	F005	220	Count/cell	35.50249	34	17.66865
CTNNB1	W3	PGE2	well_2	F006	276	Count/cell	43.25403	42.5	19.44274
CTNNB1	W3	PGE2	well_3	F001	181	Count/cell	42.14634	40	18.97536
CTNNB1	W3	PGE2	well_3	F002	217	Count/cell	40.85641	40	14.12317
CTNNB1	W3	PGE2	well_3	F003	219	Count/cell	42.15228	42	18.33078
CTNNB1	W3	PGE2	well_3	F004	217	Count/cell	39.96447	37	16.98931
CTNNB1	W3	PGE2	well_3	F005	233	Count/cell	37.17619	36.5	17.41397
CTNNB1	W3	PGE2	well_3	F006	201	Count/cell	47.02762	47	17.72708
CTNNB1	W3	PGE2	well_4	F001	102	Count/cell	35.86813	35	16.88142
CTNNB1	W3	PGE2	well_4	F002	192	Count/cell	36.12069	34	16.79569
CTNNB1	W3	PGE2	well_4	F003	193	Count/cell	31.86782	31	14.19353
CTNNB1	W3	PGE2	well_4	F004	200	Count/cell	32.64088	32	13.74321
CTNNB1	W3	PGE2	well_4	F005	224	Count/cell	32.07463	31	13.06022
CTNNB1	W3	PGE2	well_4	F006	233	Count/cell	42.80476	41	19.17073
CTNNB1	W3	PGE2	well_5	F001	64	Count/cell	29.51724	28	14.74014
CTNNB1	W3	PGE2	well_5	F002	129	Count/cell	25.96522	24	10.05331
CTNNB1	W3	PGE2	well_5	F003	75	Count/cell	29.13433	26	14.36736
CTNNB1	W3	PGE2	well_5	F004	95	Count/cell	18.2069	16	10.64014
CTNNB1	W3	PGE2	well_5	F005	151	Count/cell	34.27206	31	15.71056
CTNNB1	W3	PGE2	well_5	F006	233	Count/cell	33.75117	32	15.20548
CTNNB1	W4	HPI4	well_1	F004	5	Count/cell	36.66667	31	23.02897
CTNNB1	W4	HPI4	well_1	F005	17	Count/cell	42.93333	37	22.32701
CTNNB1	W4	HPI4	well_1	F006	12	Count/cell	63.9	53.5	22.71783

CTNNB1	W4	HPI4	well_2	F001	8	Count/cell	65.5	56.5	25.16148
CTNNB1	W4	HPI4	well_2	F002	1	Count/cell	80	80	NA
CTNNB1	W4	HPI4	well_2	F003	7	Count/cell	83	81	18.31666
CTNNB1	W4	HPI4	well_2	F004	4	Count/cell	119	119	24.04163
CTNNB1	W4	HPI4	well_2	F005	8	Count/cell	32.33333	15.5	31.48121
CTNNB1	W4	HPI4	well_2	F006	7	Count/cell	60	42	31.81981
CTNNB1	W4	HPI4	well_3	F001	4	Count/cell	51	51	38.18377
CTNNB1	W4	HPI4	well_3	F002	6	Count/cell	69.25	62	18.15443
CTNNB1	W4	HPI4	well_3	F003	1	Count/cell	23	23	NA
CTNNB1	W4	HPI4	well_3	F004	9	Count/cell	40.42857	39	12.29983
CTNNB1	W4	HPI4	well_3	F005	1	Count/cell	146	146	NA
CTNNB1	W4	HPI4	well_3	F006	5	Count/cell	85.33333	98	22.81082
CTNNB1	W4	HPI4	well_4	F001	13	Count/cell	35.09091	28	27.84764
CTNNB1	W4	HPI4	well_4	F002	3	Count/cell	42	42	NA
CTNNB1	W4	HPI4	well_4	F004	14	Count/cell	38.33333	33.5	16.48875
CTNNB1	W4	HPI4	well_4	F006	5	Count/cell	48.66667	46	6.429101
CTNNB1	W4	HPI4	well_5	F001	3	Count/cell	87	87	NA
CTNNB1	W4	HPI4	well_5	F003	13	Count/cell	41.27273	42	27.73118
CTNNB1	W4	HPI4	well_5	F004	42	Count/cell	93.25	91	27.83356
CTNNB1	W4	HPI4	well_5	F005	8	Count/cell	71.71429	74	7.111359
CTNNB1	W4	HPI4	well_5	F006	14	Count/cell	41.16667	41	15.79317
CTNNB1	W4	PGE2	well_1	F001	28	Count/cell	22.16667	18	11.48786
CTNNB1	W4	PGE2	well_1	F002	113	Count/cell	42.29703	43	15.64579
CTNNB1	W4	PGE2	well_1	F003	89	Count/cell	35.34177	35	15.29445
CTNNB1	W4	PGE2	well_1	F004	58	Count/cell	32.65385	32	12.61634
CTNNB1	W4	PGE2	well_1	F005	43	Count/cell	26.56757	25	13.1139
CTNNB1	W4	PGE2	well_1	F006	53	Count/cell	27.02128	24	13.39125
CTNNB1	W4	PGE2	well_2	F001	79	Count/cell	33.90141	30	17.20395
CTNNB1	W4	PGE2	well_2	F002	218	Count/cell	37.70051	35	14.029
CTNNB1	W4	PGE2	well_2	F003	141	Count/cell	39.04724	37	15.60263
CTNNB1	W4	PGE2	well_2	F004	156	Count/cell	29.06429	29	11.14858
CTNNB1	W4	PGE2	well_2	F005	184	Count/cell	34.10909	33	12.9153
CTNNB1	W4	PGE2	well_2	F006	145	Count/cell	41.22481	39	17.91309
CTNNB1	W4	PGE2	well_3	F001	186	Count/cell	35.17964	34	12.87021
CTNNB1	W4	PGE2	well_3	F002	301	Count/cell	38.64207	38	15.27601
CTNNB1	W4	PGE2	well_3	F003	288	Count/cell	45.35632	44	15.93857
CTNNB1	W4	PGE2	well_3	F004	196	Count/cell	48.16292	50	15.55894
CTNNB1	W4	PGE2	well_3	F005	251	Count/cell	37.65487	37	14.83436
CTNNB1	W4	PGE2	well_3	F006	240	Count/cell	48.13242	47	18.30014
CTNNB1	W4	PGE2	well_4	F001	119	Count/cell	37.75926	37.5	17.71681
CTNNB1	W4	PGE2	well_4	F002	189	Count/cell	44.19527	43	19.40829
CTNNB1	W4	PGE2	well_4	F003	176	Count/cell	45.89241	43.5	18.74896
CTNNB1	W4	PGE2	well_4	F004	114	Count/cell	42.30392	40	18.5049
CTNNB1	W4	PGE2	well_4	F005	207	Count/cell	42.48663	40	16.07053
CTNNB1	W4	PGE2	well_4	F006	268	Count/cell	37.9751	37	15.23235
CTNNB1	W4	PGE2	well_5	F001	110	Count/cell	39.42857	37.5	18.10849
CTNNB1	W4	PGE2	well_5	F002	188	Count/cell	44.62722	43	19.35254
CTNNB1	W4	PGE2	well_5	F003	189	Count/cell	36.69006	36	15.9968
CTNNB1	W4	PGE2	well_5	F004	152	Count/cell	37.15441	33.5	18.13157

CTNNB1	W4	PGE2	well_5	F005	243	Count/cell	33.8211	33	13.05042
CTNNB1	W4	PGE2	well_5	F006	200	Count/cell	37.78022	35	17.40153
CTNNB1	W1	HPI4	well_1	F001	91	X/Y distribution	7.212391	7.231181	1.404105
CTNNB1	W1	HPI4	well_1	F002	73	X/Y distribution	6.733303	6.43405	1.563404
CTNNB1	W1	HPI4	well_1	F003	164	X/Y distribution	6.196865	6.205049	1.192577
CTNNB1	W1	HPI4	well_1	F004	154	X/Y distribution	6.765106	6.718122	1.048686
CTNNB1	W1	HPI4	well_1	F005	155	X/Y distribution	7.175293	7.19742	1.18441
CTNNB1	W1	HPI4	well_1	F006	135	X/Y distribution	6.64441	6.752498	1.203851
CTNNB1	W1	HPI4	well_2	F001	169	X/Y distribution	6.660796	6.547654	1.334801
CTNNB1	W1	HPI4	well_2	F002	212	X/Y distribution	6.720348	6.701792	0.907018
CTNNB1	W1	HPI4	well_2	F003	128	X/Y distribution	7.360524	7.243639	1.246014
CTNNB1	W1	HPI4	well_2	F004	168	X/Y distribution	7.091062	6.993589	1.210675
CTNNB1	W1	HPI4	well_2	F005	228	X/Y distribution	7.423221	7.343223	1.28175
CTNNB1	W1	HPI4	well_2	F006	121	X/Y distribution	7.006612	6.89937	1.128839
CTNNB1	W1	HPI4	well_3	F001	126	X/Y distribution	6.731626	6.854508	1.185125
CTNNB1	W1	HPI4	well_3	F002	206	X/Y distribution	6.750138	6.632037	1.063234
CTNNB1	W1	HPI4	well_3	F003	72	X/Y distribution	7.355255	7.254059	1.15284
CTNNB1	W1	HPI4	well_3	F004	148	X/Y distribution	7.405701	7.379379	1.247994
CTNNB1	W1	HPI4	well_3	F005	121	X/Y distribution	7.331518	7.403967	1.397031
CTNNB1	W1	HPI4	well_3	F006	34	X/Y distribution	7.559476	7.584378	1.57029
CTNNB1	W1	HPI4	well_4	F001	76	X/Y distribution	6.76798	6.908521	1.373266
CTNNB1	W1	HPI4	well_4	F002	69	X/Y distribution	6.466916	6.497697	1.326053
CTNNB1	W1	HPI4	well_4	F003	76	X/Y distribution	7.104235	7.268296	1.326606
CTNNB1	W1	HPI4	well_4	F004	108	X/Y distribution	6.636198	6.620207	1.14404
CTNNB1	W1	HPI4	well_4	F005	137	X/Y distribution	7.29642	7.256986	1.270177
CTNNB1	W1	HPI4	well_4	F006	118	X/Y distribution	6.98025	6.870895	1.2155
CTNNB1	W1	HPI4	well_5	F001	74	X/Y distribution	7.012389	6.982304	1.232229
CTNNB1	W1	HPI4	well_5	F002	202	X/Y distribution	6.42774	6.345603	1.381379
CTNNB1	W1	HPI4	well_5	F003	105	X/Y distribution	8.218055	8.227117	1.227511
CTNNB1	W1	HPI4	well_5	F004	134	X/Y distribution	7.423003	7.373922	1.390532
CTNNB1	W1	HPI4	well_5	F005	117	X/Y distribution	7.466792	7.502585	1.207688
CTNNB1	W1	HPI4	well_5	F006	164	X/Y distribution	6.778452	6.760768	1.140684
CTNNB1	W1	PGE2	well_1	F001	162	X/Y distribution	7.509139	7.490739	1.359461
CTNNB1	W1	PGE2	well_1	F002	155	X/Y distribution	6.413196	6.270669	1.249269
CTNNB1	W1	PGE2	well_1	F003	202	X/Y distribution	6.284417	6.199438	1.23298
CTNNB1	W1	PGE2	well_1	F004	166	X/Y distribution	6.671219	6.75824	1.09339
CTNNB1	W1	PGE2	well_1	F005	69	X/Y distribution	6.981825	7.131566	1.263486
CTNNB1	W1	PGE2	well_1	F006	227	X/Y distribution	6.775755	6.71964	1.089862
CTNNB1	W1	PGE2	well_2	F001	134	X/Y distribution	6.674068	6.557169	1.254225
CTNNB1	W1	PGE2	well_2	F002	138	X/Y distribution	6.768415	6.670085	1.278402
CTNNB1	W1	PGE2	well_2	F003	107	X/Y distribution	6.859218	6.642129	1.424889
CTNNB1	W1	PGE2	well_2	F004	177	X/Y distribution	6.360887	6.281342	1.072684
CTNNB1	W1	PGE2	well_2	F005	47	X/Y distribution	7.555075	7.350892	1.684118
CTNNB1	W1	PGE2	well_2	F006	80	X/Y distribution	6.938816	7.025435	1.353896
CTNNB1	W1	PGE2	well_3	F001	106	X/Y distribution	6.894923	6.854641	1.325153
CTNNB1	W1	PGE2	well_3	F002	70	X/Y distribution	7.821685	7.959611	1.365312
CTNNB1	W1	PGE2	well_3	F003	152	X/Y distribution	6.757116	6.619184	1.23687
CTNNB1	W1	PGE2	well_3	F004	95	X/Y distribution	6.68542	6.411306	1.094425
CTNNB1	W1	PGE2	well_3	F005	100	X/Y distribution	7.431315	7.455335	1.197666

CTNNB1	W1	PGE2	well_3	F006	118	X/Y distribution	7.10146	6.822169	1.321364
CTNNB1	W1	PGE2	well_4	F001	39	X/Y distribution	7.594693	7.572892	1.795503
CTNNB1	W1	PGE2	well_4	F002	96	X/Y distribution	6.771732	6.661218	1.125934
CTNNB1	W1	PGE2	well_4	F003	111	X/Y distribution	6.242124	6.087692	1.019778
CTNNB1	W1	PGE2	well_4	F004	202	X/Y distribution	7.099403	7.150355	1.04656
CTNNB1	W1	PGE2	well_4	F005	75	X/Y distribution	7.258915	7.004761	1.219329
CTNNB1	W1	PGE2	well_4	F006	63	X/Y distribution	7.071455	7.213885	1.436907
CTNNB1	W1	PGE2	well_5	F001	136	X/Y distribution	6.457407	6.453916	1.274082
CTNNB1	W1	PGE2	well_5	F002	137	X/Y distribution	7.202466	7.220624	1.194036
CTNNB1	W1	PGE2	well_5	F003	70	X/Y distribution	7.853469	7.673081	1.279328
CTNNB1	W1	PGE2	well_5	F004	87	X/Y distribution	7.387795	7.360292	1.456622
CTNNB1	W1	PGE2	well_5	F005	83	X/Y distribution	7.201574	7.190517	1.260068
CTNNB1	W1	PGE2	well_5	F006	89	X/Y distribution	6.962074	6.978782	1.049537
CTNNB1	W2	HPI4	well_1	F001	7	X/Y distribution	4.322071	4.093622	0.712363
CTNNB1	W2	HPI4	well_1	F005	3	X/Y distribution	3.889319	3.889319	NA
CTNNB1	W2	HPI4	well_2	F001	1	X/Y distribution	3.764909	3.764909	NA
CTNNB1	W2	HPI4	well_2	F005	3	X/Y distribution	3.71147	3.71147	NA
CTNNB1	W2	HPI4	well_2	F006	3	X/Y distribution	3.698664	3.698664	NA
CTNNB1	W2	HPI4	well_3	F001	1	X/Y distribution	4.034361	4.034361	NA
CTNNB1	W2	HPI4	well_3	F002	4	X/Y distribution	4.415266	4.415266	0.516904
CTNNB1	W2	HPI4	well_3	F005	8	X/Y distribution	3.853541	3.85375	0.26375
CTNNB1	W2	HPI4	well_3	F006	3	X/Y distribution	3.979607	3.979607	NA
CTNNB1	W2	HPI4	well_4	F001	5	X/Y distribution	3.857138	3.879471	0.637002
CTNNB1	W2	HPI4	well_4	F003	1	X/Y distribution	4.584992	4.584992	NA
CTNNB1	W2	HPI4	well_4	F005	1	X/Y distribution	4.013463	4.013463	NA
CTNNB1	W2	HPI4	well_5	F004	6	X/Y distribution	6.326628	5.875147	1.396905
CTNNB1	W2	HPI4	well_5	F005	78	X/Y distribution	4.830636	4.671895	0.839602
CTNNB1	W2	HPI4	well_5	F006	11	X/Y distribution	9.436631	9.953201	2.360157
CTNNB1	W2	PGE2	well_1	F001	91	X/Y distribution	5.871466	5.611	1.910794
CTNNB1	W2	PGE2	well_1	F002	67	X/Y distribution	5.468791	5.083998	1.903807
CTNNB1	W2	PGE2	well_1	F003	74	X/Y distribution	7.151915	7.217087	1.75212
CTNNB1	W2	PGE2	well_1	F004	96	X/Y distribution	6.532055	6.564733	1.871909
CTNNB1	W2	PGE2	well_1	F005	95	X/Y distribution	6.608192	6.60713	1.664888
CTNNB1	W2	PGE2	well_1	F006	121	X/Y distribution	6.026879	5.635353	1.714491
CTNNB1	W2	PGE2	well_2	F001	117	X/Y distribution	6.194502	6.187916	1.394819
CTNNB1	W2	PGE2	well_2	F002	91	X/Y distribution	6.445588	6.551159	1.257077
CTNNB1	W2	PGE2	well_2	F003	161	X/Y distribution	6.692855	6.57442	1.315269
CTNNB1	W2	PGE2	well_2	F004	91	X/Y distribution	6.264588	6.159309	1.785205
CTNNB1	W2	PGE2	well_2	F005	68	X/Y distribution	6.736402	6.771532	1.650172
CTNNB1	W2	PGE2	well_2	F006	141	X/Y distribution	7.00191	7.040326	1.361351
CTNNB1	W2	PGE2	well_3	F001	88	X/Y distribution	5.196585	4.693634	1.778985
CTNNB1	W2	PGE2	well_3	F002	50	X/Y distribution	5.972919	5.731347	1.850763
CTNNB1	W2	PGE2	well_3	F003	24	X/Y distribution	5.82037	5.574548	1.45584
CTNNB1	W2	PGE2	well_3	F004	42	X/Y distribution	6.161483	5.983919	1.675359
CTNNB1	W2	PGE2	well_3	F005	51	X/Y distribution	5.015453	4.48614	1.819674
CTNNB1	W2	PGE2	well_3	F006	34	X/Y distribution	5.156919	4.894568	1.508518
CTNNB1	W2	PGE2	well_4	F001	10	X/Y distribution	4.955053	4.91177	1.304619
CTNNB1	W2	PGE2	well_4	F002	8	X/Y distribution	6.782369	6.958434	1.228807
CTNNB1	W2	PGE2	well_4	F003	10	X/Y distribution	6.868431	6.839021	1.572125

CTNNB1	W2	PGE2	well_4	F004	73	X/Y distribution	6.944734	7.076927	2.32271
CTNNB1	W2	PGE2	well_4	F005	66	X/Y distribution	5.825043	5.732337	2.084846
CTNNB1	W2	PGE2	well_4	F006	24	X/Y distribution	5.774869	6.346328	1.730758
CTNNB1	W2	PGE2	well_5	F001	3	X/Y distribution	3.655797	3.655797	NA
CTNNB1	W2	PGE2	well_5	F002	27	X/Y distribution	5.801407	6.049097	1.399278
CTNNB1	W2	PGE2	well_5	F003	73	X/Y distribution	5.991555	6.00973	1.761967
CTNNB1	W2	PGE2	well_5	F004	38	X/Y distribution	4.75686	4.187124	2.006692
CTNNB1	W2	PGE2	well_5	F005	65	X/Y distribution	6.014355	5.905237	1.417429
CTNNB1	W2	PGE2	well_5	F006	41	X/Y distribution	6.0781	5.938614	2.003595
CTNNB1	W3	HPI4	well_1	F001	17	X/Y distribution	4.224642	3.969291	0.691941
CTNNB1	W3	HPI4	well_1	F005	1	X/Y distribution	5.362109	5.362109	NA
CTNNB1	W3	HPI4	well_1	F006	1	X/Y distribution	4.921751	4.921751	NA
CTNNB1	W3	HPI4	well_2	F001	12	X/Y distribution	4.445379	4.118599	0.966245
CTNNB1	W3	HPI4	well_2	F004	1	X/Y distribution	5.385593	5.385593	NA
CTNNB1	W3	HPI4	well_2	F006	6	X/Y distribution	5.524511	5.403772	0.570406
CTNNB1	W3	HPI4	well_3	F001	66	X/Y distribution	3.836116	3.869972	0.542982
CTNNB1	W3	HPI4	well_3	F002	1	X/Y distribution	4.324283	4.324283	NA
CTNNB1	W3	HPI4	well_3	F003	1	X/Y distribution	9.123659	9.123659	NA
CTNNB1	W3	HPI4	well_3	F004	3	X/Y distribution	2.800577	2.800577	NA
CTNNB1	W3	HPI4	well_3	F005	4	X/Y distribution	3.914546	3.914546	0.049907
CTNNB1	W3	HPI4	well_4	F001	30	X/Y distribution	4.000844	4.166688	0.737639
CTNNB1	W3	HPI4	well_5	F001	3	X/Y distribution	3.906066	3.906066	NA
CTNNB1	W3	HPI4	well_5	F005	8	X/Y distribution	5.825258	5.835368	0.394431
CTNNB1	W3	HPI4	well_5	F006	7	X/Y distribution	5.137972	4.609571	0.873021
CTNNB1	W3	PGE2	well_1	F001	85	X/Y distribution	8.211736	8.182221	1.510405
CTNNB1	W3	PGE2	well_1	F002	138	X/Y distribution	6.980956	6.756475	1.351106
CTNNB1	W3	PGE2	well_1	F003	91	X/Y distribution	7.012274	6.795773	1.577569
CTNNB1	W3	PGE2	well_1	F004	45	X/Y distribution	7.326083	6.956066	1.643421
CTNNB1	W3	PGE2	well_1	F005	144	X/Y distribution	8.044819	7.789408	1.508233
CTNNB1	W3	PGE2	well_1	F006	138	X/Y distribution	8.050047	7.676347	1.844575
CTNNB1	W3	PGE2	well_2	F001	248	X/Y distribution	6.935357	6.91793	1.122501
CTNNB1	W3	PGE2	well_2	F002	175	X/Y distribution	7.282543	7.087104	1.443217
CTNNB1	W3	PGE2	well_2	F003	190	X/Y distribution	6.888035	6.784871	1.329948
CTNNB1	W3	PGE2	well_2	F004	137	X/Y distribution	6.947975	6.819186	1.423859
CTNNB1	W3	PGE2	well_2	F005	220	X/Y distribution	7.264794	7.281188	1.471058
CTNNB1	W3	PGE2	well_2	F006	276	X/Y distribution	7.103896	6.942793	1.333912
CTNNB1	W3	PGE2	well_3	F001	181	X/Y distribution	7.689769	7.664784	1.478353
CTNNB1	W3	PGE2	well_3	F002	217	X/Y distribution	7.44099	7.327107	1.177021
CTNNB1	W3	PGE2	well_3	F003	219	X/Y distribution	7.506124	7.419092	1.478114
CTNNB1	W3	PGE2	well_3	F004	217	X/Y distribution	7.60826	7.577115	1.457377
CTNNB1	W3	PGE2	well_3	F005	233	X/Y distribution	7.215895	6.990751	1.466725
CTNNB1	W3	PGE2	well_3	F006	201	X/Y distribution	7.533894	7.435465	1.448945
CTNNB1	W3	PGE2	well_4	F001	102	X/Y distribution	7.673008	7.519088	1.464272
CTNNB1	W3	PGE2	well_4	F002	192	X/Y distribution	7.354663	7.206319	1.539818
CTNNB1	W3	PGE2	well_4	F003	193	X/Y distribution	7.161168	7.020221	1.547326
CTNNB1	W3	PGE2	well_4	F004	200	X/Y distribution	7.514561	7.344943	1.484046
CTNNB1	W3	PGE2	well_4	F005	224	X/Y distribution	6.803159	6.501866	1.405511
CTNNB1	W3	PGE2	well_4	F006	233	X/Y distribution	7.350342	7.291741	1.341977
CTNNB1	W3	PGE2	well_5	F001	64	X/Y distribution	7.339407	7.24019	1.515602

CTNNB1	W3	PGE2	well_5	F002	129	X/Y distribution	7.05882	6.782104	1.315941
CTNNB1	W3	PGE2	well_5	F003	75	X/Y distribution	6.575045	6.295411	1.747144
CTNNB1	W3	PGE2	well_5	F004	95	X/Y distribution	6.019377	5.804217	1.626957
CTNNB1	W3	PGE2	well_5	F005	151	X/Y distribution	7.254571	7.193977	1.509672
CTNNB1	W3	PGE2	well_5	F006	233	X/Y distribution	6.972177	6.954175	1.345111
CTNNB1	W4	HPI4	well_1	F004	5	X/Y distribution	12.42417	15.77588	6.726142
CTNNB1	W4	HPI4	well_1	F005	17	X/Y distribution	9.217476	9.185229	1.80276
CTNNB1	W4	HPI4	well_1	F006	12	X/Y distribution	10.73527	10.60223	2.082834
CTNNB1	W4	HPI4	well_2	F001	8	X/Y distribution	10.24927	10.04996	0.99218
CTNNB1	W4	HPI4	well_2	F002	1	X/Y distribution	10.2215	10.2215	NA
CTNNB1	W4	HPI4	well_2	F003	7	X/Y distribution	10.47679	10.35116	0.612423
CTNNB1	W4	HPI4	well_2	F004	4	X/Y distribution	13.40936	13.40936	1.960311
CTNNB1	W4	HPI4	well_2	F005	8	X/Y distribution	6.976022	7.100515	2.092882
CTNNB1	W4	HPI4	well_2	F006	7	X/Y distribution	6.979272	6.65422	0.747921
CTNNB1	W4	HPI4	well_3	F001	4	X/Y distribution	12.26865	12.26865	0.569603
CTNNB1	W4	HPI4	well_3	F002	6	X/Y distribution	8.37445	7.7228	2.91369
CTNNB1	W4	HPI4	well_3	F003	1	X/Y distribution	4.598584	4.598584	NA
CTNNB1	W4	HPI4	well_3	F004	9	X/Y distribution	10.1369	10.59691	2.485918
CTNNB1	W4	HPI4	well_3	F005	1	X/Y distribution	15.4977	15.4977	NA
CTNNB1	W4	HPI4	well_3	F006	5	X/Y distribution	11.48026	11.49764	0.923682
CTNNB1	W4	HPI4	well_4	F001	13	X/Y distribution	6.111027	5.405174	2.140175
CTNNB1	W4	HPI4	well_4	F002	3	X/Y distribution	6.022934	6.022934	NA
CTNNB1	W4	HPI4	well_4	F004	14	X/Y distribution	10.75276	10.61663	2.254414
CTNNB1	W4	HPI4	well_4	F006	5	X/Y distribution	6.939351	7.010835	0.22333
CTNNB1	W4	HPI4	well_5	F001	3	X/Y distribution	10.10416	10.10416	NA
CTNNB1	W4	HPI4	well_5	F003	13	X/Y distribution	11.72172	11.42499	3.777163
CTNNB1	W4	HPI4	well_5	F004	42	X/Y distribution	11.21024	11.42675	2.006268
CTNNB1	W4	HPI4	well_5	F005	8	X/Y distribution	10.14975	9.827848	1.06378
CTNNB1	W4	HPI4	well_5	F006	14	X/Y distribution	8.189837	7.603421	2.766249
CTNNB1	W4	PGE2	well_1	F001	28	X/Y distribution	6.694164	6.419917	2.026603
CTNNB1	W4	PGE2	well_1	F002	113	X/Y distribution	7.310671	7.205579	1.282021
CTNNB1	W4	PGE2	well_1	F003	89	X/Y distribution	7.291694	7.057178	1.519911
CTNNB1	W4	PGE2	well_1	F004	58	X/Y distribution	7.151592	6.931067	1.391125
CTNNB1	W4	PGE2	well_1	F005	43	X/Y distribution	7.032193	6.578081	1.979535
CTNNB1	W4	PGE2	well_1	F006	53	X/Y distribution	6.245799	6.219986	1.637387
CTNNB1	W4	PGE2	well_2	F001	79	X/Y distribution	7.364095	7.014122	1.790318
CTNNB1	W4	PGE2	well_2	F002	218	X/Y distribution	6.759642	6.762827	1.133941
CTNNB1	W4	PGE2	well_2	F003	141	X/Y distribution	7.2034	7.036763	1.437436
CTNNB1	W4	PGE2	well_2	F004	156	X/Y distribution	7.338362	7.405277	1.483663
CTNNB1	W4	PGE2	well_2	F005	184	X/Y distribution	6.721031	6.501786	1.342899
CTNNB1	W4	PGE2	well_2	F006	145	X/Y distribution	7.409638	7.37188	1.305649
CTNNB1	W4	PGE2	well_3	F001	186	X/Y distribution	6.846076	6.705025	1.214472
CTNNB1	W4	PGE2	well_3	F002	301	X/Y distribution	6.685807	6.497522	1.280441
CTNNB1	W4	PGE2	well_3	F003	288	X/Y distribution	6.775488	6.70276	1.126661
CTNNB1	W4	PGE2	well_3	F004	196	X/Y distribution	7.364754	7.13247	1.184455
CTNNB1	W4	PGE2	well_3	F005	251	X/Y distribution	6.721797	6.655393	1.318433
CTNNB1	W4	PGE2	well_3	F006	240	X/Y distribution	7.118988	6.941513	1.353822
CTNNB1	W4	PGE2	well_4	F001	119	X/Y distribution	7.843657	8.000091	1.71946
CTNNB1	W4	PGE2	well_4	F002	189	X/Y distribution	7.681475	7.6775	1.270596

CTNNB1	W4	PGE2	well_4	F003	176	X/Y distribution	7.582935	7.383697	1.447476
CTNNB1	W4	PGE2	well_4	F004	114	X/Y distribution	7.761701	7.819206	1.631982
CTNNB1	W4	PGE2	well_4	F005	207	X/Y distribution	7.368317	7.184312	1.465396
CTNNB1	W4	PGE2	well_4	F006	268	X/Y distribution	6.830459	6.688924	1.230219
CTNNB1	W4	PGE2	well_5	F001	110	X/Y distribution	7.8464	7.696048	1.956735
CTNNB1	W4	PGE2	well_5	F002	188	X/Y distribution	7.552886	7.469377	1.543569
CTNNB1	W4	PGE2	well_5	F003	189	X/Y distribution	6.923978	6.644882	1.365559
CTNNB1	W4	PGE2	well_5	F004	152	X/Y distribution	7.121925	6.878221	1.783715
CTNNB1	W4	PGE2	well_5	F005	243	X/Y distribution	7.161454	6.88778	1.483965
CTNNB1	W4	PGE2	well_5	F006	200	X/Y distribution	7.042464	6.868212	1.521518
CTNNB1	W1	HPI4	well_1	F001	91	Z distribution	-0.56249	-0.52029	0.323952
CTNNB1	W1	HPI4	well_1	F002	73	Z distribution	-0.49725	-0.47374	0.398916
CTNNB1	W1	HPI4	well_1	F003	164	Z distribution	-0.89389	-0.87085	0.521819
CTNNB1	W1	HPI4	well_1	F004	154	Z distribution	-0.81487	-0.79784	0.501429
CTNNB1	W1	HPI4	well_1	F005	155	Z distribution	-0.81717	-0.83074	0.481947
CTNNB1	W1	HPI4	well_1	F006	135	Z distribution	-0.72313	-0.74365	0.445687
CTNNB1	W1	HPI4	well_2	F001	169	Z distribution	-0.7344	-0.7048	0.494836
CTNNB1	W1	HPI4	well_2	F002	212	Z distribution	-0.73324	-0.7002	0.418333
CTNNB1	W1	HPI4	well_2	F003	128	Z distribution	-0.62257	-0.60363	0.385799
CTNNB1	W1	HPI4	well_2	F004	168	Z distribution	-0.77076	-0.71513	0.50374
CTNNB1	W1	HPI4	well_2	F005	228	Z distribution	-0.71927	-0.67906	0.414593
CTNNB1	W1	HPI4	well_2	F006	121	Z distribution	-0.71711	-0.70262	0.448269
CTNNB1	W1	HPI4	well_3	F001	126	Z distribution	-0.79605	-0.80828	0.465723
CTNNB1	W1	HPI4	well_3	F002	206	Z distribution	-0.7387	-0.68548	0.425319
CTNNB1	W1	HPI4	well_3	F003	72	Z distribution	-0.77415	-0.76652	0.439895
CTNNB1	W1	HPI4	well_3	F004	148	Z distribution	-0.51331	-0.44229	0.45624
CTNNB1	W1	HPI4	well_3	F005	121	Z distribution	-0.6328	-0.54988	0.492068
CTNNB1	W1	HPI4	well_3	F006	34	Z distribution	-0.55972	-0.59796	0.297226
CTNNB1	W1	HPI4	well_4	F001	76	Z distribution	-0.63639	-0.55182	0.434127
CTNNB1	W1	HPI4	well_4	F002	69	Z distribution	-0.71992	-0.72636	0.422116
CTNNB1	W1	HPI4	well_4	F003	76	Z distribution	-0.60238	-0.53583	0.422828
CTNNB1	W1	HPI4	well_4	F004	108	Z distribution	-0.6765	-0.54262	0.532981
CTNNB1	W1	HPI4	well_4	F005	137	Z distribution	-0.65048	-0.58261	0.451747
CTNNB1	W1	HPI4	well_4	F006	118	Z distribution	-0.57017	-0.52161	0.349669
CTNNB1	W1	HPI4	well_5	F001	74	Z distribution	-0.46044	-0.41771	0.364574
CTNNB1	W1	HPI4	well_5	F002	202	Z distribution	-0.96735	-0.86548	0.618259
CTNNB1	W1	HPI4	well_5	F003	105	Z distribution	-0.51826	-0.44815	0.32155
CTNNB1	W1	HPI4	well_5	F004	134	Z distribution	-0.69851	-0.62613	0.480852
CTNNB1	W1	HPI4	well_5	F005	117	Z distribution	-0.6187	-0.63712	0.432136
CTNNB1	W1	HPI4	well_5	F006	164	Z distribution	-0.67949	-0.69292	0.447886
CTNNB1	W1	PGE2	well_1	F001	162	Z distribution	-0.70695	-0.67599	0.412493
CTNNB1	W1	PGE2	well_1	F002	155	Z distribution	-0.73793	-0.64039	0.522362
CTNNB1	W1	PGE2	well_1	F003	202	Z distribution	-0.91514	-0.81178	0.564227
CTNNB1	W1	PGE2	well_1	F004	166	Z distribution	-0.64612	-0.63043	0.460895
CTNNB1	W1	PGE2	well_1	F005	69	Z distribution	-0.56796	-0.47575	0.411523
CTNNB1	W1	PGE2	well_1	F006	227	Z distribution	-0.66043	-0.61089	0.459028
CTNNB1	W1	PGE2	well_2	F001	134	Z distribution	-0.51784	-0.50874	0.432029
CTNNB1	W1	PGE2	well_2	F002	138	Z distribution	-0.77683	-0.69949	0.515003
CTNNB1	W1	PGE2	well_2	F003	107	Z distribution	-0.71024	-0.74418	0.412274

CTNNB1	W1	PGE2	well_2	F004	177	Z distribution	-0.72577	-0.62231	0.537042
CTNNB1	W1	PGE2	well_2	F005	47	Z distribution	-0.67737	-0.7047	0.432539
CTNNB1	W1	PGE2	well_2	F006	80	Z distribution	-0.72688	-0.64522	0.463693
CTNNB1	W1	PGE2	well_3	F001	106	Z distribution	-0.55954	-0.5141	0.363954
CTNNB1	W1	PGE2	well_3	F002	70	Z distribution	-0.53228	-0.54413	0.308509
CTNNB1	W1	PGE2	well_3	F003	152	Z distribution	-0.74918	-0.74293	0.517475
CTNNB1	W1	PGE2	well_3	F004	95	Z distribution	-0.23233	-0.21128	0.223318
CTNNB1	W1	PGE2	well_3	F005	100	Z distribution	-0.58558	-0.53893	0.443437
CTNNB1	W1	PGE2	well_3	F006	118	Z distribution	-0.64296	-0.59856	0.396801
CTNNB1	W1	PGE2	well_4	F001	39	Z distribution	-0.46038	-0.33115	0.371832
CTNNB1	W1	PGE2	well_4	F002	96	Z distribution	-0.71958	-0.6915	0.428928
CTNNB1	W1	PGE2	well_4	F003	111	Z distribution	-0.80612	-0.74648	0.58005
CTNNB1	W1	PGE2	well_4	F004	202	Z distribution	-0.5593	-0.53546	0.360397
CTNNB1	W1	PGE2	well_4	F005	75	Z distribution	-0.7487	-0.68439	0.458764
CTNNB1	W1	PGE2	well_4	F006	63	Z distribution	-0.6922	-0.57967	0.426507
CTNNB1	W1	PGE2	well_5	F001	136	Z distribution	-0.77374	-0.76905	0.419422
CTNNB1	W1	PGE2	well_5	F002	137	Z distribution	-0.8016	-0.77604	0.499159
CTNNB1	W1	PGE2	well_5	F003	70	Z distribution	-0.43334	-0.44993	0.254872
CTNNB1	W1	PGE2	well_5	F004	87	Z distribution	-0.47709	-0.39827	0.301658
CTNNB1	W1	PGE2	well_5	F005	83	Z distribution	-0.46506	-0.43598	0.336006
CTNNB1	W1	PGE2	well_5	F006	89	Z distribution	-0.54768	-0.49996	0.373714
CTNNB1	W2	HPI4	well_1	F001	7	Z distribution	-0.03154	-0.07057	0.1549
CTNNB1	W2	HPI4	well_1	F005	3	Z distribution	0.093297	0.093297	NA
CTNNB1	W2	HPI4	well_2	F001	1	Z distribution	-0.03577	-0.03577	NA
CTNNB1	W2	HPI4	well_2	F005	3	Z distribution	-0.55504	-0.55504	NA
CTNNB1	W2	HPI4	well_2	F006	3	Z distribution	-0.09924	-0.09924	NA
CTNNB1	W2	HPI4	well_3	F001	1	Z distribution	0.251186	0.251186	NA
CTNNB1	W2	HPI4	well_3	F002	4	Z distribution	-0.26271	-0.26271	0.285726
CTNNB1	W2	HPI4	well_3	F005	8	Z distribution	-0.02996	-0.06105	0.141284
CTNNB1	W2	HPI4	well_3	F006	3	Z distribution	-0.13854	-0.13854	NA
CTNNB1	W2	HPI4	well_4	F001	5	Z distribution	-0.47683	-0.30129	0.332189
CTNNB1	W2	HPI4	well_4	F003	1	Z distribution	-0.13032	-0.13032	NA
CTNNB1	W2	HPI4	well_4	F005	1	Z distribution	-1.3757	-1.3757	NA
CTNNB1	W2	HPI4	well_5	F004	6	Z distribution	-0.26117	-0.28861	0.214854
CTNNB1	W2	HPI4	well_5	F005	78	Z distribution	-0.40538	-0.30299	0.512177
CTNNB1	W2	HPI4	well_5	F006	11	Z distribution	-0.42171	-0.38637	0.140037
CTNNB1	W2	PGE2	well_1	F001	91	Z distribution	-0.41357	-0.37242	0.287619
CTNNB1	W2	PGE2	well_1	F002	67	Z distribution	-0.43598	-0.37551	0.30791
CTNNB1	W2	PGE2	well_1	F003	74	Z distribution	-0.25981	-0.16086	0.295058
CTNNB1	W2	PGE2	well_1	F004	96	Z distribution	-0.51155	-0.45065	0.329798
CTNNB1	W2	PGE2	well_1	F005	95	Z distribution	-0.49121	-0.44887	0.386139
CTNNB1	W2	PGE2	well_1	F006	121	Z distribution	-0.49645	-0.37494	0.457572
CTNNB1	W2	PGE2	well_2	F001	117	Z distribution	-0.54639	-0.49146	0.413481
CTNNB1	W2	PGE2	well_2	F002	91	Z distribution	-0.63603	-0.53217	0.411263
CTNNB1	W2	PGE2	well_2	F003	161	Z distribution	-0.5994	-0.53488	0.389149
CTNNB1	W2	PGE2	well_2	F004	91	Z distribution	-0.46646	-0.43094	0.349369
CTNNB1	W2	PGE2	well_2	F005	68	Z distribution	-0.44588	-0.40361	0.329076
CTNNB1	W2	PGE2	well_2	F006	141	Z distribution	-0.53155	-0.51874	0.355032
CTNNB1	W2	PGE2	well_3	F001	88	Z distribution	-0.50473	-0.4806	0.295053

CTNNB1	W2	PGE2	well_3	F002	50	Z distribution	-0.58599	-0.57389	0.366045
CTNNB1	W2	PGE2	well_3	F003	24	Z distribution	-0.25918	-0.22866	0.275135
CTNNB1	W2	PGE2	well_3	F004	42	Z distribution	-0.49244	-0.48349	0.315171
CTNNB1	W2	PGE2	well_3	F005	51	Z distribution	-0.31595	-0.21048	0.273529
CTNNB1	W2	PGE2	well_3	F006	34	Z distribution	-0.38884	-0.29252	0.310229
CTNNB1	W2	PGE2	well_4	F001	10	Z distribution	-0.49772	-0.48247	0.215563
CTNNB1	W2	PGE2	well_4	F002	8	Z distribution	-0.34229	-0.34048	0.048768
CTNNB1	W2	PGE2	well_4	F003	10	Z distribution	-0.35154	-0.20276	0.426334
CTNNB1	W2	PGE2	well_4	F004	73	Z distribution	-0.50597	-0.50775	0.366216
CTNNB1	W2	PGE2	well_4	F005	66	Z distribution	-0.42476	-0.42162	0.243972
CTNNB1	W2	PGE2	well_4	F006	24	Z distribution	-0.48508	-0.49671	0.333023
CTNNB1	W2	PGE2	well_5	F001	3	Z distribution	-1.13125	-1.13125	NA
CTNNB1	W2	PGE2	well_5	F002	27	Z distribution	-0.36637	-0.38627	0.269734
CTNNB1	W2	PGE2	well_5	F003	73	Z distribution	-0.43427	-0.41514	0.288461
CTNNB1	W2	PGE2	well_5	F004	38	Z distribution	-0.37042	-0.32681	0.294329
CTNNB1	W2	PGE2	well_5	F005	65	Z distribution	-0.49029	-0.44889	0.285693
CTNNB1	W2	PGE2	well_5	F006	41	Z distribution	-0.54117	-0.51991	0.308046
CTNNB1	W3	HPI4	well_1	F001	17	Z distribution	-0.13057	-0.12146	0.176972
CTNNB1	W3	HPI4	well_1	F005	1	Z distribution	-0.47797	-0.47797	NA
CTNNB1	W3	HPI4	well_1	F006	1	Z distribution	-0.01898	-0.01898	NA
CTNNB1	W3	HPI4	well_2	F001	12	Z distribution	-0.23443	-0.19652	0.28744
CTNNB1	W3	HPI4	well_2	F004	1	Z distribution	0.090291	0.090291	NA
CTNNB1	W3	HPI4	well_2	F006	6	Z distribution	-0.33032	-0.34955	0.157005
CTNNB1	W3	HPI4	well_3	F001	66	Z distribution	-0.17693	-0.15999	0.240614
CTNNB1	W3	HPI4	well_3	F002	1	Z distribution	-0.11922	-0.11922	NA
CTNNB1	W3	HPI4	well_3	F003	1	Z distribution	-0.54265	-0.54265	NA
CTNNB1	W3	HPI4	well_3	F004	3	Z distribution	0.022126	0.022126	NA
CTNNB1	W3	HPI4	well_3	F005	4	Z distribution	-0.21772	-0.21772	0.011103
CTNNB1	W3	HPI4	well_4	F001	30	Z distribution	-0.06292	-0.00674	0.278012
CTNNB1	W3	HPI4	well_5	F001	3	Z distribution	-0.42127	-0.42127	NA
CTNNB1	W3	HPI4	well_5	F005	8	Z distribution	-0.40757	-0.39152	0.099075
CTNNB1	W3	HPI4	well_5	F006	7	Z distribution	-0.34465	-0.47122	0.215545
CTNNB1	W3	PGE2	well_1	F001	85	Z distribution	-0.46336	-0.35313	0.390368
CTNNB1	W3	PGE2	well_1	F002	138	Z distribution	-0.26464	-0.20655	0.228902
CTNNB1	W3	PGE2	well_1	F003	91	Z distribution	-0.33187	-0.27267	0.275245
CTNNB1	W3	PGE2	well_1	F004	45	Z distribution	-0.37458	-0.3324	0.215834
CTNNB1	W3	PGE2	well_1	F005	144	Z distribution	-0.34319	-0.27531	0.281132
CTNNB1	W3	PGE2	well_1	F006	138	Z distribution	-0.50283	-0.48028	0.381141
CTNNB1	W3	PGE2	well_2	F001	248	Z distribution	-0.37474	-0.32481	0.319619
CTNNB1	W3	PGE2	well_2	F002	175	Z distribution	-0.58757	-0.48251	0.457557
CTNNB1	W3	PGE2	well_2	F003	190	Z distribution	-0.42542	-0.30674	0.357825
CTNNB1	W3	PGE2	well_2	F004	137	Z distribution	-0.29478	-0.237	0.237411
CTNNB1	W3	PGE2	well_2	F005	220	Z distribution	-0.54623	-0.45769	0.399729
CTNNB1	W3	PGE2	well_2	F006	276	Z distribution	-0.54864	-0.47428	0.441212
CTNNB1	W3	PGE2	well_3	F001	181	Z distribution	-0.48669	-0.44016	0.356646
CTNNB1	W3	PGE2	well_3	F002	217	Z distribution	-0.3502	-0.28274	0.296271
CTNNB1	W3	PGE2	well_3	F003	219	Z distribution	-0.37689	-0.28735	0.332237
CTNNB1	W3	PGE2	well_3	F004	217	Z distribution	-0.3253	-0.27122	0.279963
CTNNB1	W3	PGE2	well_3	F005	233	Z distribution	-0.43208	-0.34025	0.359526

CTNNB1	W3	PGE2	well_3	F006	201	Z distribution	-0.51606	-0.45903	0.385485
CTNNB1	W3	PGE2	well_4	F001	102	Z distribution	-0.43442	-0.37635	0.237506
CTNNB1	W3	PGE2	well_4	F002	192	Z distribution	-0.36938	-0.32687	0.283861
CTNNB1	W3	PGE2	well_4	F003	193	Z distribution	-0.43486	-0.30273	0.373266
CTNNB1	W3	PGE2	well_4	F004	200	Z distribution	-0.23237	-0.16662	0.232256
CTNNB1	W3	PGE2	well_4	F005	224	Z distribution	-0.36114	-0.25949	0.306034
CTNNB1	W3	PGE2	well_4	F006	233	Z distribution	-0.45429	-0.36956	0.366489
CTNNB1	W3	PGE2	well_5	F001	64	Z distribution	-0.2554	-0.24658	0.187573
CTNNB1	W3	PGE2	well_5	F002	129	Z distribution	-0.34568	-0.26715	0.300848
CTNNB1	W3	PGE2	well_5	F003	75	Z distribution	-0.72156	-0.61017	0.528806
CTNNB1	W3	PGE2	well_5	F004	95	Z distribution	-0.80454	-0.74553	0.536664
CTNNB1	W3	PGE2	well_5	F005	151	Z distribution	-0.2446	-0.19513	0.235551
CTNNB1	W3	PGE2	well_5	F006	233	Z distribution	-0.55562	-0.42565	0.455265
CTNNB1	W4	HPI4	well_1	F004	5	Z distribution	-0.47383	-0.31689	0.321562
CTNNB1	W4	HPI4	well_1	F005	17	Z distribution	-0.2137	-0.18179	0.205507
CTNNB1	W4	HPI4	well_1	F006	12	Z distribution	-0.54818	-0.58292	0.263197
CTNNB1	W4	HPI4	well_2	F001	8	Z distribution	-0.51831	-0.41499	0.236758
CTNNB1	W4	HPI4	well_2	F002	1	Z distribution	-0.29895	-0.29895	NA
CTNNB1	W4	HPI4	well_2	F003	7	Z distribution	-0.2622	-0.12445	0.230163
CTNNB1	W4	HPI4	well_2	F004	4	Z distribution	-0.49114	-0.49114	0.082524
CTNNB1	W4	HPI4	well_2	F005	8	Z distribution	-0.4755	-0.44409	0.189715
CTNNB1	W4	HPI4	well_2	F006	7	Z distribution	-0.09291	-0.10967	0.142461
CTNNB1	W4	HPI4	well_3	F001	4	Z distribution	-0.17931	-0.17931	0.156552
CTNNB1	W4	HPI4	well_3	F002	6	Z distribution	-0.60839	-0.53197	0.236937
CTNNB1	W4	HPI4	well_3	F003	1	Z distribution	-0.73684	-0.73684	NA
CTNNB1	W4	HPI4	well_3	F004	9	Z distribution	-0.23483	-0.23526	0.165281
CTNNB1	W4	HPI4	well_3	F005	1	Z distribution	-0.1855	-0.1855	NA
CTNNB1	W4	HPI4	well_3	F006	5	Z distribution	-0.28161	-0.34923	0.120052
CTNNB1	W4	HPI4	well_4	F001	13	Z distribution	-0.22532	-0.14298	0.191236
CTNNB1	W4	HPI4	well_4	F002	3	Z distribution	-0.3118	-0.3118	NA
CTNNB1	W4	HPI4	well_4	F004	14	Z distribution	-0.24035	-0.23944	0.103397
CTNNB1	W4	HPI4	well_4	F006	5	Z distribution	-0.2943	-0.36583	0.234585
CTNNB1	W4	HPI4	well_5	F001	3	Z distribution	-0.27227	-0.27227	NA
CTNNB1	W4	HPI4	well_5	F003	13	Z distribution	-0.22798	-0.07558	0.442136
CTNNB1	W4	HPI4	well_5	F004	42	Z distribution	-0.39023	-0.34889	0.221444
CTNNB1	W4	HPI4	well_5	F005	8	Z distribution	-0.23251	-0.25938	0.086428
CTNNB1	W4	HPI4	well_5	F006	14	Z distribution	-0.25972	-0.21201	0.236558
CTNNB1	W4	PGE2	well_1	F001	28	Z distribution	-0.27437	-0.20238	0.222316
CTNNB1	W4	PGE2	well_1	F002	113	Z distribution	-0.63984	-0.60192	0.398692
CTNNB1	W4	PGE2	well_1	F003	89	Z distribution	-0.37023	-0.26117	0.342671
CTNNB1	W4	PGE2	well_1	F004	58	Z distribution	-0.20899	-0.16599	0.248164
CTNNB1	W4	PGE2	well_1	F005	43	Z distribution	-0.27777	-0.19575	0.262379
CTNNB1	W4	PGE2	well_1	F006	53	Z distribution	-0.49633	-0.29453	0.431517
CTNNB1	W4	PGE2	well_2	F001	79	Z distribution	-0.34129	-0.30503	0.313098
CTNNB1	W4	PGE2	well_2	F002	218	Z distribution	-0.29091	-0.20651	0.331066
CTNNB1	W4	PGE2	well_2	F003	141	Z distribution	-0.31887	-0.20387	0.329915
CTNNB1	W4	PGE2	well_2	F004	156	Z distribution	-0.22035	-0.1475	0.266044
CTNNB1	W4	PGE2	well_2	F005	184	Z distribution	-0.26546	-0.16814	0.294974
CTNNB1	W4	PGE2	well_2	F006	145	Z distribution	-0.37752	-0.29801	0.354812

CTNNB1	W4	PGE2	well_3	F001	186	Z distribution	-0.21272	-0.17921	0.19441
CTNNB1	W4	PGE2	well_3	F002	301	Z distribution	-0.25013	-0.18537	0.310926
CTNNB1	W4	PGE2	well_3	F003	288	Z distribution	-0.44786	-0.3475	0.388788
CTNNB1	W4	PGE2	well_3	F004	196	Z distribution	-0.33482	-0.235	0.374605
CTNNB1	W4	PGE2	well_3	F005	251	Z distribution	-0.28581	-0.20432	0.300314
CTNNB1	W4	PGE2	well_3	F006	240	Z distribution	-0.40957	-0.29552	0.385744
CTNNB1	W4	PGE2	well_4	F001	119	Z distribution	-0.45954	-0.36875	0.344469
CTNNB1	W4	PGE2	well_4	F002	189	Z distribution	-0.45614	-0.30981	0.416487
CTNNB1	W4	PGE2	well_4	F003	176	Z distribution	-0.47271	-0.33915	0.41595
CTNNB1	W4	PGE2	well_4	F004	114	Z distribution	-0.36361	-0.31653	0.369414
CTNNB1	W4	PGE2	well_4	F005	207	Z distribution	-0.28751	-0.23301	0.249828
CTNNB1	W4	PGE2	well_4	F006	268	Z distribution	-0.27805	-0.1941	0.315363
CTNNB1	W4	PGE2	well_5	F001	110	Z distribution	-0.33145	-0.29187	0.293561
CTNNB1	W4	PGE2	well_5	F002	188	Z distribution	-0.26907	-0.1881	0.257246
CTNNB1	W4	PGE2	well_5	F003	189	Z distribution	-0.30499	-0.24296	0.29168
CTNNB1	W4	PGE2	well_5	F004	152	Z distribution	-0.29166	-0.24776	0.283152
CTNNB1	W4	PGE2	well_5	F005	243	Z distribution	-0.22965	-0.16221	0.287189
CTNNB1	W4	PGE2	well_5	F006	200	Z distribution	-0.2486	-0.19759	0.270733
CETN2	W1	HPI4	well_1	F001	7	Avg. Volume	1.366083	1.337917	0.742934
CETN2	W1	HPI4	well_1	F002	3	Avg. Volume	1.079722	1.079722	NA
CETN2	W1	HPI4	well_1	F003	1	Avg. Volume	3.051389	3.051389	NA
CETN2	W1	HPI4	well_1	F004	83	Avg. Volume	1.799854	1.484618	0.882199
CETN2	W1	HPI4	well_1	F005	88	Avg. Volume	1.609026	1.431806	0.697806
CETN2	W1	HPI4	well_1	F006	17	Avg. Volume	1.433057	1.549167	0.653975
CETN2	W1	HPI4	well_2	F001	9	Avg. Volume	0.928271	0.856736	0.461232
CETN2	W1	HPI4	well_2	F002	75	Avg. Volume	1.615584	1.513958	0.589441
CETN2	W1	HPI4	well_2	F003	114	Avg. Volume	1.827209	1.678264	0.66683
CETN2	W1	HPI4	well_2	F004	65	Avg. Volume	1.80384	1.666528	0.722399
CETN2	W1	HPI4	well_2	F005	4	Avg. Volume	1.889514	1.889514	0.514518
CETN2	W1	HPI4	well_2	F006	19	Avg. Volume	1.893311	1.47875	0.999363
CETN2	W1	HPI4	well_3	F001	14	Avg. Volume	1.403117	1.42985	0.566261
CETN2	W1	HPI4	well_3	F002	17	Avg. Volume	1.779873	1.525694	0.956961
CETN2	W1	HPI4	well_3	F003	71	Avg. Volume	1.840644	1.678264	0.760967
CETN2	W1	HPI4	well_3	F004	39	Avg. Volume	2.366456	2.32375	0.897214
CETN2	W1	HPI4	well_3	F005	21	Avg. Volume	1.702074	1.490486	1.105191
CETN2	W1	HPI4	well_3	F006	33	Avg. Volume	1.62997	1.584375	0.739672
CETN2	W1	HPI4	well_4	F001	25	Avg. Volume	1.530724	1.349653	0.752921
CETN2	W1	HPI4	well_4	F002	86	Avg. Volume	1.637823	1.47875	0.915464
CETN2	W1	HPI4	well_4	F003	113	Avg. Volume	1.710625	1.537431	0.695989
CETN2	W1	HPI4	well_4	F004	87	Avg. Volume	1.787534	1.678264	0.704802
CETN2	W1	HPI4	well_4	F005	21	Avg. Volume	1.569437	1.393663	0.705749
CETN2	W1	HPI4	well_4	F006	35	Avg. Volume	1.362272	1.337917	0.675648
CETN2	W1	HPI4	well_5	F001	155	Avg. Volume	1.65856	1.451366	0.771783
CETN2	W1	HPI4	well_5	F002	12	Avg. Volume	1.345545	1.43474	0.531577
CETN2	W1	HPI4	well_5	F003	120	Avg. Volume	1.581459	1.441586	0.743694
CETN2	W1	HPI4	well_5	F004	110	Avg. Volume	1.577533	1.506134	0.804018
CETN2	W1	HPI4	well_5	F006	41	Avg. Volume	1.677778	1.572639	0.819686
CETN2	W1	PGE2	well_1	F001	51	Avg. Volume	1.560642	1.384861	0.639691
CETN2	W1	PGE2	well_1	F002	98	Avg. Volume	2.296166	2.194653	0.850522

CETN2	W1	PGE2	well_1	F003	56	Avg. Volume	1.898668	1.666528	0.947902
CETN2	W1	PGE2	well_1	F004	71	Avg. Volume	1.374833	1.255764	0.627566
CETN2	W1	PGE2	well_1	F005	106	Avg. Volume	1.71253	1.511024	0.738042
CETN2	W1	PGE2	well_1	F006	99	Avg. Volume	1.87965	1.725208	0.821849
CETN2	W1	PGE2	well_2	F002	34	Avg. Volume	1.448497	1.431806	0.58516
CETN2	W1	PGE2	well_2	F003	41	Avg. Volume	1.665026	1.69	0.802721
CETN2	W1	PGE2	well_2	F004	26	Avg. Volume	1.717918	1.619583	0.639364
CETN2	W1	PGE2	well_2	F005	93	Avg. Volume	1.700735	1.560903	0.692642
CETN2	W1	PGE2	well_2	F006	57	Avg. Volume	1.98789	1.783889	0.874899
CETN2	W1	PGE2	well_3	F001	9	Avg. Volume	1.296002	1.361389	0.400627
CETN2	W1	PGE2	well_3	F003	3	Avg. Volume	1.431806	1.431806	NA
CETN2	W1	PGE2	well_3	F004	78	Avg. Volume	1.484266	1.396597	0.688214
CETN2	W1	PGE2	well_3	F005	71	Avg. Volume	1.745421	1.584375	0.72922
CETN2	W1	PGE2	well_3	F006	16	Avg. Volume	1.16928	1.130579	0.667775
CETN2	W1	PGE2	well_4	F001	21	Avg. Volume	1.713781	1.467014	0.739692
CETN2	W1	PGE2	well_4	F002	5	Avg. Volume	1.357477	1.255764	0.295119
CETN2	W1	PGE2	well_4	F003	22	Avg. Volume	1.70707	1.637188	0.903563
CETN2	W1	PGE2	well_4	F004	57	Avg. Volume	1.418804	1.244028	0.683081
CETN2	W1	PGE2	well_4	F005	54	Avg. Volume	1.504504	1.44941	0.587054
CETN2	W1	PGE2	well_5	F001	31	Avg. Volume	1.399698	1.349653	0.733959
CETN2	W1	PGE2	well_5	F002	71	Avg. Volume	1.627805	1.47875	0.672224
CETN2	W1	PGE2	well_5	F003	69	Avg. Volume	1.623495	1.47875	0.809207
CETN2	W1	PGE2	well_5	F004	39	Avg. Volume	1.769358	1.666528	0.69456
CETN2	W1	PGE2	well_5	F005	24	Avg. Volume	1.695086	1.684132	0.610057
CETN2	W1	PGE2	well_5	F006	4	Avg. Volume	2.094896	2.094896	0.074688
CETN2	W2	HPI4	well_1	F001	269	Avg. Volume	1.871464	1.643056	0.944525
CETN2	W2	HPI4	well_1	F002	308	Avg. Volume	1.626931	1.423981	0.965988
CETN2	W2	HPI4	well_1	F003	488	Avg. Volume	1.409747	1.302708	0.776703
CETN2	W2	HPI4	well_1	F004	439	Avg. Volume	1.342207	1.208819	0.777678
CETN2	W2	HPI4	well_1	F005	246	Avg. Volume	1.79163	1.570292	0.895416
CETN2	W2	HPI4	well_1	F006	184	Avg. Volume	2.129007	1.912986	1.052092
CETN2	W2	HPI4	well_2	F001	181	Avg. Volume	1.878744	1.783889	0.703374
CETN2	W2	HPI4	well_2	F002	259	Avg. Volume	1.631466	1.490486	0.849406
CETN2	W2	HPI4	well_2	F003	174	Avg. Volume	1.604682	1.549167	0.880265
CETN2	W2	HPI4	well_2	F004	365	Avg. Volume	1.396579	1.2675	0.76495
CETN2	W2	HPI4	well_2	F005	290	Avg. Volume	1.454205	1.324225	0.752907
CETN2	W2	HPI4	well_2	F006	221	Avg. Volume	1.592217	1.425938	0.823098
CETN2	W2	HPI4	well_3	F001	290	Avg. Volume	1.325067	1.197083	0.750911
CETN2	W2	HPI4	well_3	F002	30	Avg. Volume	2.465637	2.147708	1.503015
CETN2	W2	HPI4	well_3	F003	25	Avg. Volume	2.648822	2.652361	0.786626
CETN2	W2	HPI4	well_3	F004	354	Avg. Volume	1.534531	1.408333	0.809901
CETN2	W2	HPI4	well_3	F005	383	Avg. Volume	1.310852	1.214688	0.670829
CETN2	W2	HPI4	well_3	F006	224	Avg. Volume	1.473122	1.361389	0.782713
CETN2	W2	HPI4	well_4	F001	184	Avg. Volume	1.786321	1.537431	0.869369
CETN2	W2	HPI4	well_4	F002	16	Avg. Volume	1.831432	1.778021	1.363781
CETN2	W2	HPI4	well_4	F003	155	Avg. Volume	1.659065	1.543299	0.867788
CETN2	W2	HPI4	well_4	F004	191	Avg. Volume	1.45858	1.298796	0.686272
CETN2	W2	HPI4	well_4	F005	239	Avg. Volume	1.457506	1.302708	0.759481
CETN2	W2	HPI4	well_4	F006	183	Avg. Volume	1.641714	1.47875	0.828388

CETN2	W2	HPI4	well_5	F001	193	Avg. Volume	1.952435	1.672396	0.872117
CETN2	W2	HPI4	well_5	F002	117	Avg. Volume	1.463818	1.255764	0.88748
CETN2	W2	HPI4	well_5	F003	75	Avg. Volume	1.689048	1.361389	0.956001
CETN2	W2	HPI4	well_5	F004	192	Avg. Volume	1.491888	1.384861	0.818624
CETN2	W2	HPI4	well_5	F005	158	Avg. Volume	1.468197	1.361389	0.745273
CETN2	W2	HPI4	well_5	F006	200	Avg. Volume	1.443713	1.337917	0.771826
CETN2	W2	PGE2	well_1	F001	274	Avg. Volume	1.408892	1.302708	0.702987
CETN2	W2	PGE2	well_1	F002	26	Avg. Volume	0.606543	0.630397	0.486098
CETN2	W2	PGE2	well_1	F003	350	Avg. Volume	1.65159	1.502222	0.726894
CETN2	W2	PGE2	well_1	F004	217	Avg. Volume	1.871609	1.760417	0.682987
CETN2	W2	PGE2	well_1	F005	311	Avg. Volume	1.697615	1.572639	0.692096
CETN2	W2	PGE2	well_1	F006	259	Avg. Volume	1.582159	1.408333	0.730154
CETN2	W2	PGE2	well_2	F001	245	Avg. Volume	1.841373	1.69	0.738461
CETN2	W2	PGE2	well_2	F002	363	Avg. Volume	1.369721	1.220556	0.651365
CETN2	W2	PGE2	well_2	F003	327	Avg. Volume	1.918291	1.725208	0.725167
CETN2	W2	PGE2	well_2	F004	392	Avg. Volume	1.55106	1.443542	0.645169
CETN2	W2	PGE2	well_2	F005	367	Avg. Volume	1.924356	1.69	0.751504
CETN2	W2	PGE2	well_2	F006	357	Avg. Volume	1.516088	1.361389	0.741553
CETN2	W2	PGE2	well_3	F001	303	Avg. Volume	1.55483	1.431806	0.736635
CETN2	W2	PGE2	well_3	F002	285	Avg. Volume	1.216972	1.103194	0.672192
CETN2	W2	PGE2	well_3	F003	373	Avg. Volume	1.798611	1.607847	0.707985
CETN2	W2	PGE2	well_3	F004	342	Avg. Volume	1.868169	1.69	0.731866
CETN2	W2	PGE2	well_3	F005	188	Avg. Volume	1.805669	1.713472	0.691738
CETN2	W2	PGE2	well_3	F006	261	Avg. Volume	1.526531	1.377037	0.70746
CETN2	W2	PGE2	well_4	F001	324	Avg. Volume	1.776267	1.613715	0.655389
CETN2	W2	PGE2	well_4	F002	325	Avg. Volume	1.533473	1.384861	0.638925
CETN2	W2	PGE2	well_4	F003	392	Avg. Volume	1.626183	1.502222	0.635072
CETN2	W2	PGE2	well_4	F004	346	Avg. Volume	1.922675	1.766285	0.709035
CETN2	W2	PGE2	well_4	F005	341	Avg. Volume	1.644456	1.472882	0.769081
CETN2	W2	PGE2	well_4	F006	345	Avg. Volume	1.356378	1.204907	0.664816
CETN2	W2	PGE2	well_5	F001	304	Avg. Volume	1.317469	1.191215	0.674387
CETN2	W2	PGE2	well_5	F002	446	Avg. Volume	1.800559	1.643056	0.712271
CETN2	W2	PGE2	well_5	F003	344	Avg. Volume	1.741204	1.596111	0.737839
CETN2	W2	PGE2	well_5	F004	90	Avg. Volume	2.079551	2.042083	0.740887
CETN2	W2	PGE2	well_5	F005	337	Avg. Volume	0.785273	0.657222	0.504395
CETN2	W2	PGE2	well_5	F006	372	Avg. Volume	1.134027	1.079722	0.508721
CETN2	W3	HPI4	well_1	F001	3	Avg. Volume	0.845	0.845	NA
CETN2	W3	HPI4	well_1	F002	23	Avg. Volume	1.169081	1.103194	0.510389
CETN2	W3	HPI4	well_1	F003	1	Avg. Volume	1.854306	1.854306	NA
CETN2	W3	HPI4	well_1	F004	6	Avg. Volume	0.873036	0.948669	0.439588
CETN2	W3	HPI4	well_1	F005	9	Avg. Volume	1.246662	1.431806	0.591123
CETN2	W3	HPI4	well_1	F006	19	Avg. Volume	1.474205	1.384861	0.813584
CETN2	W3	HPI4	well_2	F001	1	Avg. Volume	3.192222	3.192222	NA
CETN2	W3	HPI4	well_2	F002	7	Avg. Volume	2.966889	2.722778	1.680732
CETN2	W3	HPI4	well_2	F005	4	Avg. Volume	1.924722	1.924722	0.165974
CETN2	W3	HPI4	well_3	F001	1	Avg. Volume	2.652361	2.652361	NA
CETN2	W3	HPI4	well_3	F005	1	Avg. Volume	2.253333	2.253333	NA
CETN2	W3	HPI4	well_4	F001	4	Avg. Volume	1.366083	1.366083	0.922814
CETN2	W3	HPI4	well_4	F002	1	Avg. Volume	0.375556	0.375556	NA

CETN2	W3	HPI4	well_4	F005	3	Avg. Volume	2.370694	2.370694	NA
CETN2	W3	HPI4	well_4	F006	5	Avg. Volume	1.404421	1.384861	0.089636
CETN2	W3	HPI4	well_5	F001	6	Avg. Volume	1.476794	1.838657	0.935173
CETN2	W3	HPI4	well_5	F003	1	Avg. Volume	0.187778	0.187778	NA
CETN2	W3	HPI4	well_5	F004	1	Avg. Volume	1.298796	1.298796	NA
CETN2	W3	HPI4	well_5	F006	12	Avg. Volume	2.302234	1.795625	1.74589
CETN2	W3	PGE2	well_1	F001	21	Avg. Volume	1.437653	1.290972	0.472457
CETN2	W3	PGE2	well_1	F002	36	Avg. Volume	1.582725	1.425938	0.645863
CETN2	W3	PGE2	well_1	F003	28	Avg. Volume	1.480299	1.502222	0.720668
CETN2	W3	PGE2	well_1	F004	40	Avg. Volume	1.341698	1.399531	0.537
CETN2	W3	PGE2	well_1	F005	47	Avg. Volume	1.281822	1.314444	0.535714
CETN2	W3	PGE2	well_1	F006	37	Avg. Volume	1.050832	0.962361	0.496571
CETN2	W3	PGE2	well_2	F001	7	Avg. Volume	1.488921	1.549167	0.386239
CETN2	W3	PGE2	well_2	F002	13	Avg. Volume	1.969177	1.736944	0.830838
CETN2	W3	PGE2	well_2	F003	39	Avg. Volume	1.669132	1.572639	0.73198
CETN2	W3	PGE2	well_2	F004	26	Avg. Volume	1.537057	1.420069	0.781586
CETN2	W3	PGE2	well_2	F005	33	Avg. Volume	1.966473	1.490486	1.237277
CETN2	W3	PGE2	well_2	F006	19	Avg. Volume	1.650765	1.43963	0.731165
CETN2	W3	PGE2	well_3	F001	53	Avg. Volume	1.344101	1.255764	0.621618
CETN2	W3	PGE2	well_3	F002	66	Avg. Volume	1.648478	1.543299	0.644308
CETN2	W3	PGE2	well_3	F003	125	Avg. Volume	1.650605	1.455278	0.689837
CETN2	W3	PGE2	well_3	F004	75	Avg. Volume	1.217846	1.114931	0.487768
CETN2	W3	PGE2	well_3	F005	59	Avg. Volume	1.337031	1.208819	0.517403
CETN2	W3	PGE2	well_3	F006	302	Avg. Volume	1.262328	1.079722	0.622846
CETN2	W3	PGE2	well_4	F001	7	Avg. Volume	1.542125	1.713472	0.862089
CETN2	W3	PGE2	well_4	F002	10	Avg. Volume	2.199543	2.429375	0.930077
CETN2	W3	PGE2	well_4	F003	9	Avg. Volume	1.205746	1.255764	0.224093
CETN2	W3	PGE2	well_4	F004	64	Avg. Volume	1.954342	1.731076	0.773881
CETN2	W3	PGE2	well_4	F005	48	Avg. Volume	2.202058	1.830833	0.98693
CETN2	W3	PGE2	well_4	F006	64	Avg. Volume	1.569845	1.457234	0.616444
CETN2	W3	PGE2	well_5	F001	47	Avg. Volume	1.46065	1.361389	0.524164
CETN2	W3	PGE2	well_5	F002	57	Avg. Volume	1.490958	1.314444	0.722433
CETN2	W3	PGE2	well_5	F003	43	Avg. Volume	1.310744	1.118843	0.680673
CETN2	W3	PGE2	well_5	F004	48	Avg. Volume	1.516669	1.332049	0.709173
CETN2	W3	PGE2	well_5	F005	51	Avg. Volume	1.184403	1.126667	0.571883
CETN2	W3	PGE2	well_5	F006	57	Avg. Volume	1.536387	1.377037	0.618634
CETN2	W4	HPI4	well_1	F005	6	Avg. Volume	1.05625	1.079722	0.468661
CETN2	W4	HPI4	well_1	F006	4	Avg. Volume	1.408333	1.408333	0.298753
CETN2	W4	HPI4	well_2	F005	3	Avg. Volume	3.098333	3.098333	NA
CETN2	W4	HPI4	well_3	F002	1	Avg. Volume	2.206389	2.206389	NA
CETN2	W4	HPI4	well_3	F006	3	Avg. Volume	1.47875	1.47875	NA
CETN2	W4	HPI4	well_5	F001	1	Avg. Volume	1.337917	1.337917	NA
CETN2	W4	HPI4	well_5	F005	1	Avg. Volume	1.47875	1.47875	NA
CETN2	W4	PGE2	well_1	F001	40	Avg. Volume	2.080443	1.787801	0.860313
CETN2	W4	PGE2	well_1	F002	125	Avg. Volume	2.026107	1.830833	0.832027
CETN2	W4	PGE2	well_1	F003	209	Avg. Volume	2.069821	1.778021	0.889656
CETN2	W4	PGE2	well_1	F004	165	Avg. Volume	1.766197	1.65088	0.788311
CETN2	W4	PGE2	well_1	F005	232	Avg. Volume	1.835792	1.619583	0.774541
CETN2	W4	PGE2	well_1	F006	188	Avg. Volume	2.370016	2.229861	0.832578

CETN2	W4	PGE2	well_2	F001	110	Avg. Volume	2.006616	1.854306	0.780743
CETN2	W4	PGE2	well_2	F002	152	Avg. Volume	1.585041	1.46408	0.726833
CETN2	W4	PGE2	well_2	F003	67	Avg. Volume	2.282077	1.971667	0.942388
CETN2	W4	PGE2	well_2	F004	68	Avg. Volume	2.126518	1.985359	1.008852
CETN2	W4	PGE2	well_2	F005	156	Avg. Volume	1.819214	1.674352	0.89043
CETN2	W4	PGE2	well_2	F006	127	Avg. Volume	2.232008	2.065556	0.835715
CETN2	W4	PGE2	well_3	F001	18	Avg. Volume	1.046103	0.991115	0.423692
CETN2	W4	PGE2	well_3	F002	27	Avg. Volume	1.679761	1.654792	0.488293
CETN2	W4	PGE2	well_3	F003	14	Avg. Volume	1.809969	1.519826	0.95175
CETN2	W4	PGE2	well_3	F004	65	Avg. Volume	1.797286	1.69	0.930839
CETN2	W4	PGE2	well_3	F005	35	Avg. Volume	2.013266	2.006875	1.056564
CETN2	W4	PGE2	well_3	F006	58	Avg. Volume	1.93849	1.795625	0.795802
CETN2	W4	PGE2	well_4	F001	7	Avg. Volume	1.741639	1.502222	0.584135
CETN2	W4	PGE2	well_4	F002	10	Avg. Volume	1.592444	1.69	0.316206
CETN2	W4	PGE2	well_4	F003	8	Avg. Volume	1.666528	1.461146	0.538993
CETN2	W4	PGE2	well_4	F004	18	Avg. Volume	1.528384	1.290972	0.871438
CETN2	W4	PGE2	well_4	F005	11	Avg. Volume	1.898207	1.909074	0.717546
CETN2	W4	PGE2	well_4	F006	21	Avg. Volume	1.523738	1.197083	0.73741
CETN2	W4	PGE2	well_5	F001	8	Avg. Volume	2.34331	2.177049	0.533634
CETN2	W4	PGE2	well_5	F002	16	Avg. Volume	1.7885	1.995139	0.824313
CETN2	W4	PGE2	well_5	F003	21	Avg. Volume	1.907015	1.619583	0.739552
CETN2	W4	PGE2	well_5	F004	15	Avg. Volume	1.889213	1.924722	1.191329
CETN2	W4	PGE2	well_5	F005	20	Avg. Volume	1.454952	1.345741	0.670453
CETN2	W4	PGE2	well_5	F006	25	Avg. Volume	1.612504	1.384861	0.73751
CETN2	W1	HPI4	well_1	F001	7	Count/cell	1.5	1.5	0.547723
CETN2	W1	HPI4	well_1	F002	3	Count/cell	2	2	0
CETN2	W1	HPI4	well_1	F003	1	Count/cell	1	1	NA
CETN2	W1	HPI4	well_1	F004	83	Count/cell	1.707317	2	0.693672
CETN2	W1	HPI4	well_1	F005	88	Count/cell	1.752941	2	0.670925
CETN2	W1	HPI4	well_1	F006	17	Count/cell	1.941176	2	1.297622
CETN2	W1	HPI4	well_2	F001	9	Count/cell	2.142857	2	0.377964
CETN2	W1	HPI4	well_2	F002	75	Count/cell	1.716216	2	0.652083
CETN2	W1	HPI4	well_2	F003	114	Count/cell	1.648649	2	0.612723
CETN2	W1	HPI4	well_2	F004	65	Count/cell	1.754098	2	0.745112
CETN2	W1	HPI4	well_2	F005	4	Count/cell	1.5	1.5	0.57735
CETN2	W1	HPI4	well_2	F006	19	Count/cell	1.722222	2	0.751904
CETN2	W1	HPI4	well_3	F001	14	Count/cell	2	2	0.877058
CETN2	W1	HPI4	well_3	F002	17	Count/cell	2	2	0.816497
CETN2	W1	HPI4	well_3	F003	71	Count/cell	1.704225	2	0.684405
CETN2	W1	HPI4	well_3	F004	39	Count/cell	1.564103	1	0.717997
CETN2	W1	HPI4	well_3	F005	21	Count/cell	2.05	2	0.944513
CETN2	W1	HPI4	well_3	F006	33	Count/cell	1.575758	1	0.662868
CETN2	W1	HPI4	well_4	F001	25	Count/cell	1.72	2	0.678233
CETN2	W1	HPI4	well_4	F002	86	Count/cell	1.670732	2	0.667682
CETN2	W1	HPI4	well_4	F003	113	Count/cell	1.891892	2	0.790297
CETN2	W1	HPI4	well_4	F004	87	Count/cell	1.647059	2	0.630681
CETN2	W1	HPI4	well_4	F005	21	Count/cell	2.3	2	1.218282
CETN2	W1	HPI4	well_4	F006	35	Count/cell	1.794118	2	0.640994
CETN2	W1	HPI4	well_5	F001	155	Count/cell	1.851351	2	0.673753

CETN2	W1	HPI4	well_5	F002	12	Count/cell	1.818182	2	0.603023
CETN2	W1	HPI4	well_5	F003	120	Count/cell	1.779661	2	0.68107
CETN2	W1	HPI4	well_5	F004	110	Count/cell	1.846154	2	0.720965
CETN2	W1	HPI4	well_5	F005	2	Count/cell	1	1	0
CETN2	W1	HPI4	well_5	F006	41	Count/cell	1.74359	2	0.751068
CETN2	W1	PGE2	well_1	F001	51	Count/cell	1.854167	2	0.798658
CETN2	W1	PGE2	well_1	F002	98	Count/cell	1.694737	2	0.620118
CETN2	W1	PGE2	well_1	F003	56	Count/cell	1.792453	2	0.71679
CETN2	W1	PGE2	well_1	F004	71	Count/cell	1.911765	2	0.706796
CETN2	W1	PGE2	well_1	F005	106	Count/cell	2.048077	2	0.817049
CETN2	W1	PGE2	well_1	F006	99	Count/cell	1.861702	2	0.727362
CETN2	W1	PGE2	well_2	F002	34	Count/cell	1.852941	2	0.702047
CETN2	W1	PGE2	well_2	F003	41	Count/cell	1.564103	1	0.717997
CETN2	W1	PGE2	well_2	F004	26	Count/cell	2.125	2	0.946963
CETN2	W1	PGE2	well_2	F005	93	Count/cell	1.788889	2	0.661874
CETN2	W1	PGE2	well_2	F006	57	Count/cell	1.690909	2	0.716802
CETN2	W1	PGE2	well_3	F001	9	Count/cell	1.555556	2	0.527046
CETN2	W1	PGE2	well_3	F003	3	Count/cell	2	2	0
CETN2	W1	PGE2	well_3	F004	78	Count/cell	1.68	2	0.791167
CETN2	W1	PGE2	well_3	F005	71	Count/cell	1.710145	2	0.841942
CETN2	W1	PGE2	well_3	F006	16	Count/cell	1.933333	2	0.798809
CETN2	W1	PGE2	well_4	F001	21	Count/cell	2.05	2	0.604805
CETN2	W1	PGE2	well_4	F002	5	Count/cell	2	2	0
CETN2	W1	PGE2	well_4	F003	22	Count/cell	1.7	2	0.732695
CETN2	W1	PGE2	well_4	F004	57	Count/cell	1.796296	2	0.736633
CETN2	W1	PGE2	well_4	F005	54	Count/cell	1.882353	2	0.682556
CETN2	W1	PGE2	well_5	F001	31	Count/cell	1.517241	1	0.687682
CETN2	W1	PGE2	well_5	F002	71	Count/cell	1.705882	2	0.599971
CETN2	W1	PGE2	well_5	F003	69	Count/cell	1.797101	2	0.65484
CETN2	W1	PGE2	well_5	F004	39	Count/cell	1.923077	2	0.928627
CETN2	W1	PGE2	well_5	F005	24	Count/cell	1.869565	2	0.757049
CETN2	W1	PGE2	well_5	F006	4	Count/cell	1.5	1.5	0.57735
CETN2	W2	HPI4	well_1	F001	269	Count/cell	1.878788	2	0.872306
CETN2	W2	HPI4	well_1	F002	308	Count/cell	1.939394	2	0.93525
CETN2	W2	HPI4	well_1	F003	488	Count/cell	1.903766	2	0.805606
CETN2	W2	HPI4	well_1	F004	439	Count/cell	2.079254	2	1.060446
CETN2	W2	HPI4	well_1	F005	246	Count/cell	1.864979	2	0.862788
CETN2	W2	HPI4	well_1	F006	184	Count/cell	1.818681	2	0.870056
CETN2	W2	HPI4	well_2	F001	181	Count/cell	2.127907	2	0.895688
CETN2	W2	HPI4	well_2	F002	259	Count/cell	2	2	0.852803
CETN2	W2	HPI4	well_2	F003	174	Count/cell	1.863905	2	0.851713
CETN2	W2	HPI4	well_2	F004	365	Count/cell	2.079772	2	0.943801
CETN2	W2	HPI4	well_2	F005	290	Count/cell	2.185455	2	0.942782
CETN2	W2	HPI4	well_2	F006	221	Count/cell	2.313084	2	1.150675
CETN2	W2	HPI4	well_3	F001	290	Count/cell	2.131673	2	1.102154
CETN2	W2	HPI4	well_3	F002	30	Count/cell	1.321429	1	0.475595
CETN2	W2	HPI4	well_3	F003	25	Count/cell	2.083333	2	1.017955
CETN2	W2	HPI4	well_3	F004	354	Count/cell	2.127907	2	1.064087
CETN2	W2	HPI4	well_3	F005	383	Count/cell	2.140496	2	0.913125

CETN2	W2	HPI4	well_3	F006	224	Count/cell	1.962264	2	0.963057
CETN2	W2	HPI4	well_4	F001	184	Count/cell	1.910112	2	0.825312
CETN2	W2	HPI4	well_4	F002	16	Count/cell	2.333333	2	1.397276
CETN2	W2	HPI4	well_4	F003	155	Count/cell	1.954248	2	0.868603
CETN2	W2	HPI4	well_4	F004	191	Count/cell	2.219251	2	1.067634
CETN2	W2	HPI4	well_4	F005	239	Count/cell	1.970085	2	0.90963
CETN2	W2	HPI4	well_4	F006	183	Count/cell	2	2	0.861892
CETN2	W2	HPI4	well_5	F001	193	Count/cell	1.889474	2	0.874927
CETN2	W2	HPI4	well_5	F002	117	Count/cell	1.769912	2	0.876365
CETN2	W2	HPI4	well_5	F003	75	Count/cell	2.094595	2	1.172387
CETN2	W2	HPI4	well_5	F004	192	Count/cell	2.037433	2	0.894241
CETN2	W2	HPI4	well_5	F005	158	Count/cell	2.12987	2	1.124308
CETN2	W2	HPI4	well_5	F006	200	Count/cell	2.159794	2	1.115242
CETN2	W2	PGE2	well_1	F001	274	Count/cell	2.2	2	0.899592
CETN2	W2	PGE2	well_1	F002	26	Count/cell	1.833333	1.5	1.129319
CETN2	W2	PGE2	well_1	F003	350	Count/cell	1.81791	2	0.700497
CETN2	W2	PGE2	well_1	F004	217	Count/cell	1.753623	2	0.647755
CETN2	W2	PGE2	well_1	F005	311	Count/cell	1.83557	2	0.674057
CETN2	W2	PGE2	well_1	F006	259	Count/cell	1.92	2	0.659804
CETN2	W2	PGE2	well_2	F001	245	Count/cell	1.887931	2	0.674477
CETN2	W2	PGE2	well_2	F002	363	Count/cell	1.769886	2	0.795931
CETN2	W2	PGE2	well_2	F003	327	Count/cell	1.806452	2	0.629345
CETN2	W2	PGE2	well_2	F004	392	Count/cell	2	2	0.89148
CETN2	W2	PGE2	well_2	F005	367	Count/cell	1.723647	2	0.590868
CETN2	W2	PGE2	well_2	F006	357	Count/cell	2.00578	2	0.861392
CETN2	W2	PGE2	well_3	F001	303	Count/cell	2.054422	2	0.922101
CETN2	W2	PGE2	well_3	F002	285	Count/cell	2.076642	2	0.912651
CETN2	W2	PGE2	well_3	F003	373	Count/cell	1.913747	2	0.807465
CETN2	W2	PGE2	well_3	F004	342	Count/cell	1.9375	2	0.764292
CETN2	W2	PGE2	well_3	F005	188	Count/cell	1.864865	2	0.85853
CETN2	W2	PGE2	well_3	F006	261	Count/cell	1.873518	2	0.816381
CETN2	W2	PGE2	well_4	F001	324	Count/cell	1.826367	2	0.569731
CETN2	W2	PGE2	well_4	F002	325	Count/cell	1.921136	2	0.809415
CETN2	W2	PGE2	well_4	F003	392	Count/cell	1.992188	2	0.766424
CETN2	W2	PGE2	well_4	F004	346	Count/cell	1.84985	2	0.703889
CETN2	W2	PGE2	well_4	F005	341	Count/cell	1.754601	2	0.652494
CETN2	W2	PGE2	well_4	F006	345	Count/cell	2.07622	2	0.890134
CETN2	W2	PGE2	well_5	F001	304	Count/cell	2.047782	2	0.874547
CETN2	W2	PGE2	well_5	F002	446	Count/cell	1.729792	2	0.658607
CETN2	W2	PGE2	well_5	F003	344	Count/cell	2.026946	2	0.860821
CETN2	W2	PGE2	well_5	F004	90	Count/cell	1.627907	2	0.686792
CETN2	W2	PGE2	well_5	F005	337	Count/cell	1.723602	2	0.651862
CETN2	W2	PGE2	well_5	F006	372	Count/cell	1.689944	2	0.636393
CETN2	W3	HPI4	well_1	F001	3	Count/cell	2	2	0
CETN2	W3	HPI4	well_1	F002	23	Count/cell	1.714286	2	0.64365
CETN2	W3	HPI4	well_1	F003	1	Count/cell	2	2	NA
CETN2	W3	HPI4	well_1	F004	6	Count/cell	4.75	4	3.40343
CETN2	W3	HPI4	well_1	F005	9	Count/cell	1.875	1	1.726888
CETN2	W3	HPI4	well_1	F006	19	Count/cell	1.722222	2	0.574513

CETN2	W3	HPI4	well_2	F001	1	Count/cell	1	1	NA
CETN2	W3	HPI4	well_2	F002	7	Count/cell	1	1	0
CETN2	W3	HPI4	well_2	F005	4	Count/cell	1.333333	1	0.57735
CETN2	W3	HPI4	well_3	F001	1	Count/cell	1	1	NA
CETN2	W3	HPI4	well_3	F005	1	Count/cell	1	1	NA
CETN2	W3	HPI4	well_4	F001	4	Count/cell	1.333333	1	0.57735
CETN2	W3	HPI4	well_4	F002	1	Count/cell	1	1	NA
CETN2	W3	HPI4	well_4	F005	3	Count/cell	1	1	0
CETN2	W3	HPI4	well_4	F006	5	Count/cell	1.5	1.5	0.57735
CETN2	W3	HPI4	well_5	F001	6	Count/cell	1	1	0
CETN2	W3	HPI4	well_5	F003	1	Count/cell	2	2	NA
CETN2	W3	HPI4	well_5	F004	1	Count/cell	3	3	NA
CETN2	W3	HPI4	well_5	F006	12	Count/cell	1.272727	1	0.467099
CETN2	W3	PGE2	well_1	F001	21	Count/cell	2.05	2	0.604805
CETN2	W3	PGE2	well_1	F002	36	Count/cell	2.058824	2	0.600059
CETN2	W3	PGE2	well_1	F003	28	Count/cell	1.75	1	1.004619
CETN2	W3	PGE2	well_1	F004	40	Count/cell	2.184211	2	0.896096
CETN2	W3	PGE2	well_1	F005	47	Count/cell	2.326087	2	1.193582
CETN2	W3	PGE2	well_1	F006	37	Count/cell	2.055556	2	0.860048
CETN2	W3	PGE2	well_2	F001	7	Count/cell	2.333333	2	0.516398
CETN2	W3	PGE2	well_2	F002	13	Count/cell	1.923077	2	0.862316
CETN2	W3	PGE2	well_2	F003	39	Count/cell	1.756757	2	0.862986
CETN2	W3	PGE2	well_2	F004	26	Count/cell	2.291667	2	0.999094
CETN2	W3	PGE2	well_2	F005	33	Count/cell	1.645161	2	0.660726
CETN2	W3	PGE2	well_2	F006	19	Count/cell	2	2	0.840168
CETN2	W3	PGE2	well_3	F001	53	Count/cell	2.115385	2	0.899975
CETN2	W3	PGE2	well_3	F002	66	Count/cell	1.96875	2	0.853913
CETN2	W3	PGE2	well_3	F003	125	Count/cell	2.310924	2	0.908975
CETN2	W3	PGE2	well_3	F004	75	Count/cell	2.197183	2	0.838702
CETN2	W3	PGE2	well_3	F005	59	Count/cell	2.186441	2	0.937248
CETN2	W3	PGE2	well_3	F006	302	Count/cell	2.286195	2	1.047315
CETN2	W3	PGE2	well_4	F001	7	Count/cell	1.571429	2	0.534522
CETN2	W3	PGE2	well_4	F002	10	Count/cell	1.333333	1	0.5
CETN2	W3	PGE2	well_4	F003	9	Count/cell	2.285714	2	0.48795
CETN2	W3	PGE2	well_4	F004	64	Count/cell	1.885246	2	0.732538
CETN2	W3	PGE2	well_4	F005	48	Count/cell	1.644444	2	0.608857
CETN2	W3	PGE2	well_4	F006	64	Count/cell	2.032258	2	0.809123
CETN2	W3	PGE2	well_5	F001	47	Count/cell	1.911111	2	0.900056
CETN2	W3	PGE2	well_5	F002	57	Count/cell	2.236364	2	1.170053
CETN2	W3	PGE2	well_5	F003	43	Count/cell	2.219512	2	0.908631
CETN2	W3	PGE2	well_5	F004	48	Count/cell	2.065217	2	1.20004
CETN2	W3	PGE2	well_5	F005	51	Count/cell	2.58	2	1.415512
CETN2	W3	PGE2	well_5	F006	57	Count/cell	2.053571	2	0.882551
CETN2	W4	HPI4	well_1	F004	2	Count/cell	1	1	0
CETN2	W4	HPI4	well_1	F005	6	Count/cell	1.4	1	0.547723
CETN2	W4	HPI4	well_1	F006	4	Count/cell	1	1	0
CETN2	W4	HPI4	well_2	F005	3	Count/cell	1	1	0
CETN2	W4	HPI4	well_3	F002	1	Count/cell	1	1	NA
CETN2	W4	HPI4	well_3	F006	3	Count/cell	1	1	0

CETN2	W4	HPI4	well_5	F001	1	Count/cell	2	2	NA
CETN2	W4	HPI4	well_5	F005	1	Count/cell	4	4	NA
CETN2	W4	PGE2	well_1	F001	40	Count/cell	1.684211	2	0.574469
CETN2	W4	PGE2	well_1	F002	125	Count/cell	1.926829	2	0.841259
CETN2	W4	PGE2	well_1	F003	209	Count/cell	1.678392	2	0.617166
CETN2	W4	PGE2	well_1	F004	165	Count/cell	1.974522	2	0.823914
CETN2	W4	PGE2	well_1	F005	232	Count/cell	1.951542	2	0.837366
CETN2	W4	PGE2	well_1	F006	188	Count/cell	1.565217	2	0.596975
CETN2	W4	PGE2	well_2	F001	110	Count/cell	1.773585	2	0.590125
CETN2	W4	PGE2	well_2	F002	152	Count/cell	2.114094	2	1.023575
CETN2	W4	PGE2	well_2	F003	67	Count/cell	1.742424	2	0.615457
CETN2	W4	PGE2	well_2	F004	68	Count/cell	1.615385	2	0.677595
CETN2	W4	PGE2	well_2	F005	156	Count/cell	1.940397	2	0.850347
CETN2	W4	PGE2	well_2	F006	127	Count/cell	1.788618	2	0.603976
CETN2	W4	PGE2	well_3	F001	18	Count/cell	2.611111	2	1.144752
CETN2	W4	PGE2	well_3	F002	27	Count/cell	1.84	2	0.6245
CETN2	W4	PGE2	well_3	F003	14	Count/cell	1.615385	1	0.869718
CETN2	W4	PGE2	well_3	F004	65	Count/cell	1.888889	2	0.935175
CETN2	W4	PGE2	well_3	F005	35	Count/cell	1.848485	2	0.939455
CETN2	W4	PGE2	well_3	F006	58	Count/cell	1.8	2	0.649786
CETN2	W4	PGE2	well_4	F001	7	Count/cell	1.714286	2	0.48795
CETN2	W4	PGE2	well_4	F002	10	Count/cell	1.777778	2	0.440959
CETN2	W4	PGE2	well_4	F003	8	Count/cell	1.625	2	0.517549
CETN2	W4	PGE2	well_4	F004	18	Count/cell	1.647059	2	0.701888
CETN2	W4	PGE2	well_4	F005	11	Count/cell	2	2	0.894427
CETN2	W4	PGE2	well_4	F006	21	Count/cell	2.047619	2	0.86465
CETN2	W4	PGE2	well_5	F001	8	Count/cell	1.285714	1	0.48795
CETN2	W4	PGE2	well_5	F002	16	Count/cell	1.533333	1	0.743223
CETN2	W4	PGE2	well_5	F003	21	Count/cell	1.619048	2	0.669043
CETN2	W4	PGE2	well_5	F004	15	Count/cell	1.857143	1.5	1.09945
CETN2	W4	PGE2	well_5	F005	20	Count/cell	2.15	2	0.988087
CETN2	W4	PGE2	well_5	F006	25	Count/cell	1.75	2	0.675664
CETN2	W1	HPI4	well_1	F001	7	X/Y distribution	3.580692	3.735819	1.768504
CETN2	W1	HPI4	well_1	F002	3	X/Y distribution	2.056805	2.056805	NA
CETN2	W1	HPI4	well_1	F003	1	X/Y distribution	13.19321	13.19321	NA
CETN2	W1	HPI4	well_1	F004	83	X/Y distribution	2.956441	2.737769	1.437923
CETN2	W1	HPI4	well_1	F005	88	X/Y distribution	2.949313	2.782082	1.695698
CETN2	W1	HPI4	well_1	F006	17	X/Y distribution	3.447872	3.029455	1.552402
CETN2	W1	HPI4	well_2	F001	9	X/Y distribution	3.619431	3.143402	1.293475
CETN2	W1	HPI4	well_2	F002	75	X/Y distribution	3.066411	2.599623	1.496656
CETN2	W1	HPI4	well_2	F003	114	X/Y distribution	2.760306	2.607964	1.261828
CETN2	W1	HPI4	well_2	F004	65	X/Y distribution	3.575486	3.138241	1.831599
CETN2	W1	HPI4	well_2	F005	4	X/Y distribution	3.510998	3.510998	0.229565
CETN2	W1	HPI4	well_2	F006	19	X/Y distribution	3.735853	4.233214	1.601652
CETN2	W1	HPI4	well_3	F001	14	X/Y distribution	3.779343	3.853676	1.420295
CETN2	W1	HPI4	well_3	F002	17	X/Y distribution	4.285707	4.300629	1.880174
CETN2	W1	HPI4	well_3	F003	71	X/Y distribution	3.337471	2.96513	1.546183
CETN2	W1	HPI4	well_3	F004	39	X/Y distribution	2.749825	2.637227	1.593536
CETN2	W1	HPI4	well_3	F005	21	X/Y distribution	2.67277	2.284373	1.773611

CETN2	W1	HPI4	well_3	F006	33	X/Y distribution	2.834881	2.910177	1.177364
CETN2	W1	HPI4	well_4	F001	25	X/Y distribution	3.398883	3.079104	1.941066
CETN2	W1	HPI4	well_4	F002	86	X/Y distribution	3.076757	2.810058	1.47431
CETN2	W1	HPI4	well_4	F003	113	X/Y distribution	3.140727	2.745635	1.530769
CETN2	W1	HPI4	well_4	F004	87	X/Y distribution	2.868209	2.515686	1.473197
CETN2	W1	HPI4	well_4	F005	21	X/Y distribution	3.745021	3.776529	1.815154
CETN2	W1	HPI4	well_4	F006	35	X/Y distribution	3.091491	2.614839	1.514014
CETN2	W1	HPI4	well_5	F001	155	X/Y distribution	3.556859	3.44629	1.607551
CETN2	W1	HPI4	well_5	F002	12	X/Y distribution	5.946288	4.181982	3.387054
CETN2	W1	HPI4	well_5	F003	120	X/Y distribution	3.123389	2.733248	1.672997
CETN2	W1	HPI4	well_5	F004	110	X/Y distribution	3.144433	2.791721	1.719127
CETN2	W1	HPI4	well_5	F006	41	X/Y distribution	3.520739	3.085725	1.50915
CETN2	W1	PGE2	well_1	F001	51	X/Y distribution	3.44081	3.350749	1.763862
CETN2	W1	PGE2	well_1	F002	98	X/Y distribution	3.065956	2.898345	1.512169
CETN2	W1	PGE2	well_1	F003	56	X/Y distribution	3.219556	2.869059	1.604474
CETN2	W1	PGE2	well_1	F004	71	X/Y distribution	2.883616	2.666956	1.397146
CETN2	W1	PGE2	well_1	F005	106	X/Y distribution	4.232919	3.727538	2.166069
CETN2	W1	PGE2	well_1	F006	99	X/Y distribution	3.736788	3.440465	1.917375
CETN2	W1	PGE2	well_2	F002	34	X/Y distribution	3.08752	2.67551	1.293587
CETN2	W1	PGE2	well_2	F003	41	X/Y distribution	2.784915	2.24077	1.664122
CETN2	W1	PGE2	well_2	F004	26	X/Y distribution	4.738358	4.290788	2.311389
CETN2	W1	PGE2	well_2	F005	93	X/Y distribution	2.969781	2.84599	1.429457
CETN2	W1	PGE2	well_2	F006	57	X/Y distribution	3.050246	2.411626	1.614939
CETN2	W1	PGE2	well_3	F001	9	X/Y distribution	3.821014	4.102084	1.425116
CETN2	W1	PGE2	well_3	F003	3	X/Y distribution	9.89224	9.89224	NA
CETN2	W1	PGE2	well_3	F004	78	X/Y distribution	2.769714	2.628712	1.456045
CETN2	W1	PGE2	well_3	F005	71	X/Y distribution	4.252211	3.904109	1.977435
CETN2	W1	PGE2	well_3	F006	16	X/Y distribution	3.910603	4.109165	1.379314
CETN2	W1	PGE2	well_4	F001	21	X/Y distribution	3.104435	2.766018	1.829555
CETN2	W1	PGE2	well_4	F002	5	X/Y distribution	4.836861	4.452538	1.301851
CETN2	W1	PGE2	well_4	F003	22	X/Y distribution	4.950492	4.783113	1.511893
CETN2	W1	PGE2	well_4	F004	57	X/Y distribution	3.853543	3.073992	2.364939
CETN2	W1	PGE2	well_4	F005	54	X/Y distribution	3.038403	2.84661	1.515263
CETN2	W1	PGE2	well_5	F001	31	X/Y distribution	3.782181	3.950672	1.859915
CETN2	W1	PGE2	well_5	F002	71	X/Y distribution	2.639762	2.302939	1.431807
CETN2	W1	PGE2	well_5	F003	69	X/Y distribution	3.065368	2.801127	1.550582
CETN2	W1	PGE2	well_5	F004	39	X/Y distribution	2.878264	2.573934	1.314537
CETN2	W1	PGE2	well_5	F005	24	X/Y distribution	3.987227	3.28709	2.201246
CETN2	W1	PGE2	well_5	F006	4	X/Y distribution	2.407889	2.407889	1.016756
CETN2	W2	HPI4	well_1	F001	269	X/Y distribution	5.276009	5.149002	2.303763
CETN2	W2	HPI4	well_1	F002	308	X/Y distribution	5.039164	5.024054	2.113355
CETN2	W2	HPI4	well_1	F003	488	X/Y distribution	3.694969	3.493323	1.704297
CETN2	W2	HPI4	well_1	F004	439	X/Y distribution	4.635503	4.540201	1.89718
CETN2	W2	HPI4	well_1	F005	246	X/Y distribution	4.501589	4.463727	2.007758
CETN2	W2	HPI4	well_1	F006	184	X/Y distribution	5.785159	5.49836	2.71478
CETN2	W2	HPI4	well_2	F001	181	X/Y distribution	5.340582	5.433213	2.413207
CETN2	W2	HPI4	well_2	F002	259	X/Y distribution	4.762617	4.622134	2.059676
CETN2	W2	HPI4	well_2	F003	174	X/Y distribution	5.227398	5.265127	2.260928
CETN2	W2	HPI4	well_2	F004	365	X/Y distribution	5.037367	5.038081	2.185896

CETN2	W2	HPI4	well_2	F005	290	X/Y distribution	5.471368	5.264454	2.14428
CETN2	W2	HPI4	well_2	F006	221	X/Y distribution	6.770976	6.959939	2.316842
CETN2	W2	HPI4	well_3	F001	290	X/Y distribution	5.569269	5.356329	2.076576
CETN2	W2	HPI4	well_3	F002	30	X/Y distribution	3.095206	2.839254	1.190098
CETN2	W2	HPI4	well_3	F003	25	X/Y distribution	6.314057	6.385906	1.947432
CETN2	W2	HPI4	well_3	F004	354	X/Y distribution	5.123552	5.014635	2.086809
CETN2	W2	HPI4	well_3	F005	383	X/Y distribution	5.061202	4.894806	2.086669
CETN2	W2	HPI4	well_3	F006	224	X/Y distribution	5.716828	5.647505	2.099998
CETN2	W2	HPI4	well_4	F001	184	X/Y distribution	4.538707	4.350684	1.868468
CETN2	W2	HPI4	well_4	F002	16	X/Y distribution	6.577343	6.410047	2.574819
CETN2	W2	HPI4	well_4	F003	155	X/Y distribution	4.869411	4.726152	2.001018
CETN2	W2	HPI4	well_4	F004	191	X/Y distribution	5.240496	5.202328	2.118531
CETN2	W2	HPI4	well_4	F005	239	X/Y distribution	5.253167	5.06756	2.10171
CETN2	W2	HPI4	well_4	F006	183	X/Y distribution	4.361611	4.228681	2.363237
CETN2	W2	HPI4	well_5	F001	193	X/Y distribution	4.553106	4.504017	1.911995
CETN2	W2	HPI4	well_5	F002	117	X/Y distribution	4.773959	4.582732	2.119964
CETN2	W2	HPI4	well_5	F003	75	X/Y distribution	5.423874	5.045367	2.490222
CETN2	W2	HPI4	well_5	F004	192	X/Y distribution	5.336351	5.094535	2.135217
CETN2	W2	HPI4	well_5	F005	158	X/Y distribution	5.735962	5.565134	2.634733
CETN2	W2	HPI4	well_5	F006	200	X/Y distribution	5.503387	5.506317	2.318558
CETN2	W2	PGE2	well_1	F001	274	X/Y distribution	4.145648	3.976711	1.789225
CETN2	W2	PGE2	well_1	F002	26	X/Y distribution	6.46503	6.122082	2.087882
CETN2	W2	PGE2	well_1	F003	350	X/Y distribution	3.046369	2.855116	1.440204
CETN2	W2	PGE2	well_1	F004	217	X/Y distribution	2.569135	2.458416	1.232203
CETN2	W2	PGE2	well_1	F005	311	X/Y distribution	2.943423	2.792295	1.365311
CETN2	W2	PGE2	well_1	F006	259	X/Y distribution	3.097058	2.861233	1.399073
CETN2	W2	PGE2	well_2	F001	245	X/Y distribution	3.142974	2.95926	1.432173
CETN2	W2	PGE2	well_2	F002	363	X/Y distribution	3.049506	2.774889	1.364245
CETN2	W2	PGE2	well_2	F003	327	X/Y distribution	2.958343	2.734385	1.358208
CETN2	W2	PGE2	well_2	F004	392	X/Y distribution	3.488124	3.345704	1.571511
CETN2	W2	PGE2	well_2	F005	367	X/Y distribution	2.704609	2.43624	1.248657
CETN2	W2	PGE2	well_2	F006	357	X/Y distribution	3.703312	3.514794	1.703452
CETN2	W2	PGE2	well_3	F001	303	X/Y distribution	3.78189	3.541852	1.509015
CETN2	W2	PGE2	well_3	F002	285	X/Y distribution	4.25104	4.065014	1.891583
CETN2	W2	PGE2	well_3	F003	373	X/Y distribution	3.328496	3.11224	1.495563
CETN2	W2	PGE2	well_3	F004	342	X/Y distribution	3.297249	3.128336	1.446315
CETN2	W2	PGE2	well_3	F005	188	X/Y distribution	3.015889	2.755765	1.417453
CETN2	W2	PGE2	well_3	F006	261	X/Y distribution	3.583577	3.465806	1.658606
CETN2	W2	PGE2	well_4	F001	324	X/Y distribution	2.746238	2.570385	1.260146
CETN2	W2	PGE2	well_4	F002	325	X/Y distribution	3.185921	3.078868	1.484103
CETN2	W2	PGE2	well_4	F003	392	X/Y distribution	3.434934	3.309146	1.485041
CETN2	W2	PGE2	well_4	F004	346	X/Y distribution	3.242744	3.09037	1.427745
CETN2	W2	PGE2	well_4	F005	341	X/Y distribution	2.998372	2.798704	1.365206
CETN2	W2	PGE2	well_4	F006	345	X/Y distribution	4.031134	3.895988	1.673913
CETN2	W2	PGE2	well_5	F001	304	X/Y distribution	4.106789	4.000005	1.69141
CETN2	W2	PGE2	well_5	F002	446	X/Y distribution	2.562423	2.37054	1.269875
CETN2	W2	PGE2	well_5	F003	344	X/Y distribution	3.536086	3.351532	1.656869
CETN2	W2	PGE2	well_5	F004	90	X/Y distribution	3.420259	3.323213	1.719678
CETN2	W2	PGE2	well_5	F005	337	X/Y distribution	3.294523	3.045738	1.550028

CETN2	W2	PGE2	well_5	F006	372	X/Y distribution	2.733185	2.444206	1.328092
CETN2	W3	HPI4	well_1	F001	3	X/Y distribution	6.41003	6.41003	NA
CETN2	W3	HPI4	well_1	F002	23	X/Y distribution	5.952784	6.332283	2.364185
CETN2	W3	HPI4	well_1	F003	1	X/Y distribution	1.45864	1.45864	NA
CETN2	W3	HPI4	well_1	F004	6	X/Y distribution	5.888697	5.66544	1.839399
CETN2	W3	HPI4	well_1	F005	9	X/Y distribution	7.336017	7.577656	3.181376
CETN2	W3	HPI4	well_1	F006	19	X/Y distribution	5.462841	5.936844	2.656306
CETN2	W3	HPI4	well_2	F001	1	X/Y distribution	8.00714	8.00714	NA
CETN2	W3	HPI4	well_2	F002	7	X/Y distribution	3.000049	2.532934	1.508534
CETN2	W3	HPI4	well_2	F005	4	X/Y distribution	8.006988	8.006988	2.781205
CETN2	W3	HPI4	well_3	F001	1	X/Y distribution	3.596256	3.596256	NA
CETN2	W3	HPI4	well_3	F005	1	X/Y distribution	4.436828	4.436828	NA
CETN2	W3	HPI4	well_4	F001	4	X/Y distribution	7.226089	7.226089	0.35073
CETN2	W3	HPI4	well_4	F002	1	X/Y distribution	1.752456	1.752456	NA
CETN2	W3	HPI4	well_4	F005	3	X/Y distribution	4.69373	4.69373	NA
CETN2	W3	HPI4	well_4	F006	5	X/Y distribution	4.493359	4.932727	2.596572
CETN2	W3	HPI4	well_5	F001	6	X/Y distribution	5.136636	5.312258	0.600325
CETN2	W3	HPI4	well_5	F003	1	X/Y distribution	4.30881	4.30881	NA
CETN2	W3	HPI4	well_5	F004	1	X/Y distribution	2.961976	2.961976	NA
CETN2	W3	HPI4	well_5	F006	12	X/Y distribution	4.859668	5.470722	1.827557
CETN2	W3	PGE2	well_1	F001	21	X/Y distribution	5.238905	5.273123	1.802217
CETN2	W3	PGE2	well_1	F002	36	X/Y distribution	5.001748	4.656129	1.954902
CETN2	W3	PGE2	well_1	F003	28	X/Y distribution	4.509911	4.614673	1.933098
CETN2	W3	PGE2	well_1	F004	40	X/Y distribution	6.147528	6.232318	2.627043
CETN2	W3	PGE2	well_1	F005	47	X/Y distribution	5.950956	5.890479	1.984158
CETN2	W3	PGE2	well_1	F006	37	X/Y distribution	4.877478	5.098563	1.948908
CETN2	W3	PGE2	well_2	F001	7	X/Y distribution	3.612302	3.146953	1.986215
CETN2	W3	PGE2	well_2	F002	13	X/Y distribution	3.133033	2.750126	1.444886
CETN2	W3	PGE2	well_2	F003	39	X/Y distribution	3.433816	3.256906	1.54551
CETN2	W3	PGE2	well_2	F004	26	X/Y distribution	4.780432	4.947261	2.565071
CETN2	W3	PGE2	well_2	F005	33	X/Y distribution	4.874946	4.286558	2.43688
CETN2	W3	PGE2	well_2	F006	19	X/Y distribution	3.115818	2.815122	1.51193
CETN2	W3	PGE2	well_3	F001	53	X/Y distribution	5.101208	4.93122	2.193162
CETN2	W3	PGE2	well_3	F002	66	X/Y distribution	4.669827	4.741927	2.073612
CETN2	W3	PGE2	well_3	F003	125	X/Y distribution	5.822748	6.301352	2.514441
CETN2	W3	PGE2	well_3	F004	75	X/Y distribution	7.029735	6.804879	2.98903
CETN2	W3	PGE2	well_3	F005	59	X/Y distribution	7.277403	6.714314	2.807083
CETN2	W3	PGE2	well_3	F006	302	X/Y distribution	4.992244	4.787928	2.083323
CETN2	W3	PGE2	well_4	F001	7	X/Y distribution	3.634201	3.148969	1.869302
CETN2	W3	PGE2	well_4	F002	10	X/Y distribution	2.270373	2.015486	1.361571
CETN2	W3	PGE2	well_4	F003	9	X/Y distribution	4.810601	4.804546	2.937786
CETN2	W3	PGE2	well_4	F004	64	X/Y distribution	4.65257	3.886136	3.245829
CETN2	W3	PGE2	well_4	F005	48	X/Y distribution	3.056194	2.660232	1.597956
CETN2	W3	PGE2	well_4	F006	64	X/Y distribution	4.598904	4.149187	1.75634
CETN2	W3	PGE2	well_5	F001	47	X/Y distribution	4.599841	4.010883	2.244997
CETN2	W3	PGE2	well_5	F002	57	X/Y distribution	5.93093	5.228916	2.485817
CETN2	W3	PGE2	well_5	F003	43	X/Y distribution	8.866156	9.209183	2.768955
CETN2	W3	PGE2	well_5	F004	48	X/Y distribution	8.528699	7.931037	3.01835
CETN2	W3	PGE2	well_5	F005	51	X/Y distribution	7.410266	7.619338	2.468546

CETN2	W3	PGE2	well_5	F006	57	X/Y distribution	5.381645	5.379898	1.808456
CETN2	W4	HPI4	well_1	F005	6	X/Y distribution	4.58562	4.58407	0.648697
CETN2	W4	HPI4	well_1	F006	4	X/Y distribution	2.378735	2.378735	0.19149
CETN2	W4	HPI4	well_2	F005	3	X/Y distribution	6.921729	6.921729	NA
CETN2	W4	HPI4	well_3	F002	1	X/Y distribution	7.121556	7.121556	NA
CETN2	W4	HPI4	well_3	F006	3	X/Y distribution	3.197282	3.197282	NA
CETN2	W4	HPI4	well_5	F001	1	X/Y distribution	4.679177	4.679177	NA
CETN2	W4	HPI4	well_5	F005	1	X/Y distribution	2.718378	2.718378	NA
CETN2	W4	PGE2	well_1	F001	40	X/Y distribution	3.895653	3.667052	2.135288
CETN2	W4	PGE2	well_1	F002	125	X/Y distribution	5.304	5.054411	2.370983
CETN2	W4	PGE2	well_1	F003	209	X/Y distribution	4.27969	3.859018	2.139555
CETN2	W4	PGE2	well_1	F004	165	X/Y distribution	6.091692	5.87873	2.632975
CETN2	W4	PGE2	well_1	F005	232	X/Y distribution	5.347409	5.303143	2.185497
CETN2	W4	PGE2	well_1	F006	188	X/Y distribution	3.198644	2.873622	1.548192
CETN2	W4	PGE2	well_2	F001	110	X/Y distribution	4.863262	3.799441	2.604642
CETN2	W4	PGE2	well_2	F002	152	X/Y distribution	6.48517	6.367766	2.68473
CETN2	W4	PGE2	well_2	F003	67	X/Y distribution	4.242029	3.948809	1.909544
CETN2	W4	PGE2	well_2	F004	68	X/Y distribution	4.591301	4.162529	2.298607
CETN2	W4	PGE2	well_2	F005	156	X/Y distribution	5.587057	5.355015	2.727734
CETN2	W4	PGE2	well_2	F006	127	X/Y distribution	4.200124	3.755254	1.920778
CETN2	W4	PGE2	well_3	F001	18	X/Y distribution	7.007552	5.996211	4.26964
CETN2	W4	PGE2	well_3	F002	27	X/Y distribution	5.276548	4.882856	1.867859
CETN2	W4	PGE2	well_3	F003	14	X/Y distribution	4.029431	4.188352	1.880001
CETN2	W4	PGE2	well_3	F004	65	X/Y distribution	6.867304	6.345461	3.245048
CETN2	W4	PGE2	well_3	F005	35	X/Y distribution	5.655796	5.012235	3.052468
CETN2	W4	PGE2	well_3	F006	58	X/Y distribution	4.663876	4.69618	2.194464
CETN2	W4	PGE2	well_4	F001	7	X/Y distribution	2.680756	3.228249	1.206173
CETN2	W4	PGE2	well_4	F002	10	X/Y distribution	4.370417	4.061614	2.384308
CETN2	W4	PGE2	well_4	F003	8	X/Y distribution	2.518888	2.356029	0.6869
CETN2	W4	PGE2	well_4	F004	18	X/Y distribution	3.519744	2.792863	2.161499
CETN2	W4	PGE2	well_4	F005	11	X/Y distribution	5.772239	4.51392	3.233388
CETN2	W4	PGE2	well_4	F006	21	X/Y distribution	5.068555	4.077911	2.411458
CETN2	W4	PGE2	well_5	F001	8	X/Y distribution	3.860883	3.118614	2.412939
CETN2	W4	PGE2	well_5	F002	16	X/Y distribution	5.427231	5.783592	2.918042
CETN2	W4	PGE2	well_5	F003	21	X/Y distribution	3.640827	3.11587	2.230851
CETN2	W4	PGE2	well_5	F004	15	X/Y distribution	6.736092	6.868648	3.170231
CETN2	W4	PGE2	well_5	F005	20	X/Y distribution	6.646606	6.228151	4.056703
CETN2	W4	PGE2	well_5	F006	25	X/Y distribution	5.653075	4.950416	3.04043
CETN2	W1	HPI4	well_1	F001	7	Z distribution	-0.39673	-0.27362	0.2364
CETN2	W1	HPI4	well_1	F002	3	Z distribution	-0.34827	-0.34827	NA
CETN2	W1	HPI4	well_1	F003	1	Z distribution	-0.21427	-0.21427	NA
CETN2	W1	HPI4	well_1	F004	83	Z distribution	0.938649	0.822538	0.816511
CETN2	W1	HPI4	well_1	F005	88	Z distribution	0.694902	0.62706	0.933315
CETN2	W1	HPI4	well_1	F006	17	Z distribution	-0.38458	-0.30141	0.462437
CETN2	W1	HPI4	well_2	F001	9	Z distribution	-0.39763	-0.50871	0.169547
CETN2	W1	HPI4	well_2	F002	75	Z distribution	0.500426	0.361138	0.626515
CETN2	W1	HPI4	well_2	F003	114	Z distribution	0.89727	0.970586	0.713449
CETN2	W1	HPI4	well_2	F004	65	Z distribution	0.190814	0.087833	0.561403
CETN2	W1	HPI4	well_2	F005	4	Z distribution	-0.02331	-0.02331	0.001672

CETN2	W1	HPI4	well_2	F006	19	Z distribution	0.026649	-0.14475	0.350575
CETN2	W1	HPI4	well_3	F001	14	Z distribution	0.040729	0.082422	0.454919
CETN2	W1	HPI4	well_3	F002	17	Z distribution	0.192346	0.253468	0.371697
CETN2	W1	HPI4	well_3	F003	71	Z distribution	0.654552	0.619042	0.628045
CETN2	W1	HPI4	well_3	F004	39	Z distribution	0.25841	0.263242	0.528061
CETN2	W1	HPI4	well_3	F005	21	Z distribution	-0.01287	-0.03129	0.347665
CETN2	W1	HPI4	well_3	F006	33	Z distribution	-0.1813	-0.15751	0.60921
CETN2	W1	HPI4	well_4	F001	25	Z distribution	0.088005	0.119683	0.674593
CETN2	W1	HPI4	well_4	F002	86	Z distribution	0.952355	0.968698	0.751234
CETN2	W1	HPI4	well_4	F003	113	Z distribution	1.199041	1.194585	0.931261
CETN2	W1	HPI4	well_4	F004	87	Z distribution	1.042941	1.039081	0.684226
CETN2	W1	HPI4	well_4	F005	21	Z distribution	0.009596	0.00577	0.385203
CETN2	W1	HPI4	well_4	F006	35	Z distribution	0.140979	0.033724	0.362947
CETN2	W1	HPI4	well_5	F001	155	Z distribution	0.767245	0.717135	0.729114
CETN2	W1	HPI4	well_5	F002	12	Z distribution	-0.09094	-0.00953	0.540273
CETN2	W1	HPI4	well_5	F003	120	Z distribution	0.989005	1.011775	0.957027
CETN2	W1	HPI4	well_5	F004	110	Z distribution	0.908316	0.938462	0.880203
CETN2	W1	HPI4	well_5	F006	41	Z distribution	0.272369	0.145692	0.818737
CETN2	W1	PGE2	well_1	F001	51	Z distribution	0.062852	-0.0206	0.685536
CETN2	W1	PGE2	well_1	F002	98	Z distribution	0.443813	0.32468	0.673192
CETN2	W1	PGE2	well_1	F003	56	Z distribution	0.125087	0.102627	0.672857
CETN2	W1	PGE2	well_1	F004	71	Z distribution	0.498244	0.402096	0.77439
CETN2	W1	PGE2	well_1	F005	106	Z distribution	0.510849	0.517537	0.571995
CETN2	W1	PGE2	well_1	F006	99	Z distribution	0.567891	0.618841	0.755976
CETN2	W1	PGE2	well_2	F002	34	Z distribution	0.15037	0.063766	0.681794
CETN2	W1	PGE2	well_2	F003	41	Z distribution	0.161574	0.049004	0.484405
CETN2	W1	PGE2	well_2	F004	26	Z distribution	0.006951	0.107203	0.401729
CETN2	W1	PGE2	well_2	F005	93	Z distribution	0.946008	1.004804	0.879484
CETN2	W1	PGE2	well_2	F006	57	Z distribution	0.413223	0.290739	0.713448
CETN2	W1	PGE2	well_3	F001	9	Z distribution	-0.23367	-0.13452	0.44492
CETN2	W1	PGE2	well_3	F003	3	Z distribution	-0.8258	-0.8258	NA
CETN2	W1	PGE2	well_3	F004	78	Z distribution	0.717652	0.721302	0.682169
CETN2	W1	PGE2	well_3	F005	71	Z distribution	0.2436	0.1147	0.61201
CETN2	W1	PGE2	well_3	F006	16	Z distribution	-0.39691	-0.33165	0.433649
CETN2	W1	PGE2	well_4	F001	21	Z distribution	-0.05679	-0.09191	0.408543
CETN2	W1	PGE2	well_4	F002	5	Z distribution	-0.27423	-0.29693	0.119101
CETN2	W1	PGE2	well_4	F003	22	Z distribution	-0.00351	0.015598	0.454355
CETN2	W1	PGE2	well_4	F004	57	Z distribution	0.453801	0.24775	0.588083
CETN2	W1	PGE2	well_4	F005	54	Z distribution	0.627167	0.530458	0.583685
CETN2	W1	PGE2	well_5	F001	31	Z distribution	-0.18095	-0.14347	0.406985
CETN2	W1	PGE2	well_5	F002	71	Z distribution	0.367843	0.373101	0.605706
CETN2	W1	PGE2	well_5	F003	69	Z distribution	0.422461	0.352448	0.789053
CETN2	W1	PGE2	well_5	F004	39	Z distribution	0.320181	0.273365	0.651056
CETN2	W1	PGE2	well_5	F005	24	Z distribution	0.086209	0.033194	0.35601
CETN2	W1	PGE2	well_5	F006	4	Z distribution	-0.03088	-0.03088	0.105123
CETN2	W2	HPI4	well_1	F001	269	Z distribution	-0.12642	-0.12217	0.595448
CETN2	W2	HPI4	well_1	F002	308	Z distribution	0.783131	0.809006	0.613335
CETN2	W2	HPI4	well_1	F003	488	Z distribution	0.649226	0.590607	0.854365
CETN2	W2	HPI4	well_1	F004	439	Z distribution	0.619695	0.524028	1.014291

CETN2	W2	HPI4	well_1	F005	246	Z distribution	0.270713	0.322731	0.647515
CETN2	W2	HPI4	well_1	F006	184	Z distribution	-0.08755	-0.055	0.603854
CETN2	W2	HPI4	well_2	F001	181	Z distribution	0.48739	0.471088	0.413103
CETN2	W2	HPI4	well_2	F002	259	Z distribution	0.046852	0.179638	0.862078
CETN2	W2	HPI4	well_2	F003	174	Z distribution	-0.10388	-0.06998	0.654479
CETN2	W2	HPI4	well_2	F004	365	Z distribution	0.207147	0.205566	0.745015
CETN2	W2	HPI4	well_2	F005	290	Z distribution	0.740519	0.674402	0.849959
CETN2	W2	HPI4	well_2	F006	221	Z distribution	0.420882	0.511993	0.64637
CETN2	W2	HPI4	well_3	F001	290	Z distribution	0.76678	0.762058	0.745846
CETN2	W2	HPI4	well_3	F002	30	Z distribution	0.217938	0.114643	0.358514
CETN2	W2	HPI4	well_3	F003	25	Z distribution	-0.0201	-0.11037	0.338857
CETN2	W2	HPI4	well_3	F004	354	Z distribution	0.775846	0.747993	0.796633
CETN2	W2	HPI4	well_3	F005	383	Z distribution	0.924469	1.04427	0.829014
CETN2	W2	HPI4	well_3	F006	224	Z distribution	0.84761	0.868566	0.637396
CETN2	W2	HPI4	well_4	F001	184	Z distribution	0.735463	0.751737	0.602822
CETN2	W2	HPI4	well_4	F002	16	Z distribution	-0.14608	-0.11866	0.429139
CETN2	W2	HPI4	well_4	F003	155	Z distribution	0.025833	-0.02849	0.60702
CETN2	W2	HPI4	well_4	F004	191	Z distribution	0.517873	0.528286	0.696626
CETN2	W2	HPI4	well_4	F005	239	Z distribution	0.917574	1.048154	0.73062
CETN2	W2	HPI4	well_4	F006	183	Z distribution	0.733995	0.706219	0.66683
CETN2	W2	HPI4	well_5	F001	193	Z distribution	0.697719	0.676225	0.500465
CETN2	W2	HPI4	well_5	F002	117	Z distribution	-0.28564	-0.31347	0.623443
CETN2	W2	HPI4	well_5	F003	75	Z distribution	-0.15688	-0.09129	0.698339
CETN2	W2	HPI4	well_5	F004	192	Z distribution	0.311491	0.297001	0.645372
CETN2	W2	HPI4	well_5	F005	158	Z distribution	0.269472	0.231655	0.661325
CETN2	W2	HPI4	well_5	F006	200	Z distribution	0.442279	0.460068	0.718622
CETN2	W2	PGE2	well_1	F001	274	Z distribution	1.686516	1.73586	0.928645
CETN2	W2	PGE2	well_1	F002	26	Z distribution	1.097064	1.185712	0.565955
CETN2	W2	PGE2	well_1	F003	350	Z distribution	1.915368	1.945501	0.746533
CETN2	W2	PGE2	well_1	F004	217	Z distribution	1.633371	1.712733	0.650572
CETN2	W2	PGE2	well_1	F005	311	Z distribution	1.633493	1.720783	0.602234
CETN2	W2	PGE2	well_1	F006	259	Z distribution	1.296024	1.347483	0.787937
CETN2	W2	PGE2	well_2	F001	245	Z distribution	1.583332	1.609202	0.743901
CETN2	W2	PGE2	well_2	F002	363	Z distribution	1.946815	2.089877	1.111776
CETN2	W2	PGE2	well_2	F003	327	Z distribution	1.419696	1.492389	0.671278
CETN2	W2	PGE2	well_2	F004	392	Z distribution	2.110863	2.15132	0.682331
CETN2	W2	PGE2	well_2	F005	367	Z distribution	1.617923	1.738322	0.676481
CETN2	W2	PGE2	well_2	F006	357	Z distribution	1.699144	1.797417	0.747083
CETN2	W2	PGE2	well_3	F001	303	Z distribution	1.663597	1.644362	0.732025
CETN2	W2	PGE2	well_3	F002	285	Z distribution	1.799595	1.955644	1.197766
CETN2	W2	PGE2	well_3	F003	373	Z distribution	1.730698	1.772149	0.816092
CETN2	W2	PGE2	well_3	F004	342	Z distribution	1.446621	1.537451	0.717093
CETN2	W2	PGE2	well_3	F005	188	Z distribution	1.401562	1.512231	0.80149
CETN2	W2	PGE2	well_3	F006	261	Z distribution	1.53323	1.603774	0.872442
CETN2	W2	PGE2	well_4	F001	324	Z distribution	1.56744	1.68057	0.69457
CETN2	W2	PGE2	well_4	F002	325	Z distribution	1.694684	1.804219	0.778355
CETN2	W2	PGE2	well_4	F003	392	Z distribution	1.884763	1.959334	0.720853
CETN2	W2	PGE2	well_4	F004	346	Z distribution	1.435976	1.435559	0.768651
CETN2	W2	PGE2	well_4	F005	341	Z distribution	1.527197	1.631067	0.857064

CETN2	W2	PGE2	well_4	F006	345	Z distribution	1.630154	1.732696	0.881299
CETN2	W2	PGE2	well_5	F001	304	Z distribution	1.566535	1.684505	1.071361
CETN2	W2	PGE2	well_5	F002	446	Z distribution	2.218098	2.265867	0.730248
CETN2	W2	PGE2	well_5	F003	344	Z distribution	1.767393	1.804486	0.855517
CETN2	W2	PGE2	well_5	F004	90	Z distribution	1.780534	1.985384	0.778922
CETN2	W2	PGE2	well_5	F005	337	Z distribution	1.303487	1.351863	0.734872
CETN2	W2	PGE2	well_5	F006	372	Z distribution	2.224763	2.386576	0.849334
CETN2	W3	HPI4	well_1	F001	3	Z distribution	-0.16117	-0.16117	NA
CETN2	W3	HPI4	well_1	F002	23	Z distribution	-0.22269	-0.17374	0.582406
CETN2	W3	HPI4	well_1	F003	1	Z distribution	-0.12133	-0.12133	NA
CETN2	W3	HPI4	well_1	F004	6	Z distribution	-0.53368	-0.52795	0.250617
CETN2	W3	HPI4	well_1	F005	9	Z distribution	-0.40052	-0.36565	0.298221
CETN2	W3	HPI4	well_1	F006	19	Z distribution	-0.27558	-0.20464	0.235048
CETN2	W3	HPI4	well_2	F001	1	Z distribution	-0.06512	-0.06512	NA
CETN2	W3	HPI4	well_2	F002	7	Z distribution	-0.5002	-0.49487	0.320702
CETN2	W3	HPI4	well_2	F005	4	Z distribution	-0.18055	-0.18055	0.045126
CETN2	W3	HPI4	well_3	F001	1	Z distribution	-0.11167	-0.11167	NA
CETN2	W3	HPI4	well_3	F005	1	Z distribution	-0.00406	-0.00406	NA
CETN2	W3	HPI4	well_4	F001	4	Z distribution	-0.02722	-0.02722	0.002226
CETN2	W3	HPI4	well_4	F002	1	Z distribution	-1.0187	-1.0187	NA
CETN2	W3	HPI4	well_4	F005	3	Z distribution	-0.45187	-0.45187	NA
CETN2	W3	HPI4	well_4	F006	5	Z distribution	-0.20168	-0.23657	0.154907
CETN2	W3	HPI4	well_5	F001	6	Z distribution	-0.1849	-0.27804	0.545279
CETN2	W3	HPI4	well_5	F003	1	Z distribution	-0.26317	-0.26317	NA
CETN2	W3	HPI4	well_5	F004	1	Z distribution	-0.3203	-0.3203	NA
CETN2	W3	HPI4	well_5	F006	12	Z distribution	-0.10544	0.069916	0.469159
CETN2	W3	PGE2	well_1	F001	21	Z distribution	-0.46727	-0.61838	0.455573
CETN2	W3	PGE2	well_1	F002	36	Z distribution	-0.19351	-0.22107	0.449348
CETN2	W3	PGE2	well_1	F003	28	Z distribution	0.024013	-0.0092	0.408118
CETN2	W3	PGE2	well_1	F004	40	Z distribution	-0.36839	-0.44532	0.467207
CETN2	W3	PGE2	well_1	F005	47	Z distribution	-0.26987	-0.25941	0.326809
CETN2	W3	PGE2	well_1	F006	37	Z distribution	-0.14655	-0.07254	0.489865
CETN2	W3	PGE2	well_2	F001	7	Z distribution	-0.11837	0.003384	0.221881
CETN2	W3	PGE2	well_2	F002	13	Z distribution	-0.10505	-0.00243	0.480312
CETN2	W3	PGE2	well_2	F003	39	Z distribution	-0.1027	-0.17335	0.515995
CETN2	W3	PGE2	well_2	F004	26	Z distribution	0.536495	0.483389	0.542415
CETN2	W3	PGE2	well_2	F005	33	Z distribution	-0.04025	0.067816	0.415437
CETN2	W3	PGE2	well_2	F006	19	Z distribution	-0.09843	6.42E-05	0.57231
CETN2	W3	PGE2	well_3	F001	53	Z distribution	0.135318	0.180416	0.47334
CETN2	W3	PGE2	well_3	F002	66	Z distribution	0.042917	0.001777	0.58473
CETN2	W3	PGE2	well_3	F003	125	Z distribution	0.448557	0.489382	0.476308
CETN2	W3	PGE2	well_3	F004	75	Z distribution	-0.29164	-0.27351	0.527661
CETN2	W3	PGE2	well_3	F005	59	Z distribution	-0.38601	-0.43898	0.516934
CETN2	W3	PGE2	well_3	F006	302	Z distribution	0.821467	0.809482	0.675022
CETN2	W3	PGE2	well_4	F001	7	Z distribution	-0.01775	0.017393	0.267237
CETN2	W3	PGE2	well_4	F002	10	Z distribution	-0.12149	-0.01602	0.354044
CETN2	W3	PGE2	well_4	F003	9	Z distribution	-0.22683	-0.23901	0.270311
CETN2	W3	PGE2	well_4	F004	64	Z distribution	0.243034	0.309114	0.497178
CETN2	W3	PGE2	well_4	F005	48	Z distribution	0.301295	0.288942	0.530298

CETN2	W3	PGE2	well_4	F006	64	Z distribution	0.322041	0.27622	0.41373
CETN2	W3	PGE2	well_5	F001	47	Z distribution	-0.53988	-0.53124	0.41165
CETN2	W3	PGE2	well_5	F002	57	Z distribution	-0.1763	-0.20903	0.449253
CETN2	W3	PGE2	well_5	F003	43	Z distribution	-0.32818	-0.31618	0.410214
CETN2	W3	PGE2	well_5	F004	48	Z distribution	-0.18835	-0.19253	0.435448
CETN2	W3	PGE2	well_5	F005	51	Z distribution	-0.10944	-0.14005	0.503423
CETN2	W3	PGE2	well_5	F006	57	Z distribution	-0.09845	-0.17772	0.471437
CETN2	W4	HPI4	well_1	F005	6	Z distribution	-0.59634	-0.59683	0.139518
CETN2	W4	HPI4	well_1	F006	4	Z distribution	-0.78051	-0.78051	0.032659
CETN2	W4	HPI4	well_2	F005	3	Z distribution	-0.80517	-0.80517	NA
CETN2	W4	HPI4	well_3	F002	1	Z distribution	-1.06173	-1.06173	NA
CETN2	W4	HPI4	well_3	F006	3	Z distribution	-0.75148	-0.75148	NA
CETN2	W4	HPI4	well_5	F001	1	Z distribution	-0.67437	-0.67437	NA
CETN2	W4	HPI4	well_5	F005	1	Z distribution	-1.62459	-1.62459	NA
CETN2	W4	PGE2	well_1	F001	40	Z distribution	0.632666	0.612353	0.610369
CETN2	W4	PGE2	well_1	F002	125	Z distribution	0.65222	0.719872	0.555361
CETN2	W4	PGE2	well_1	F003	209	Z distribution	0.835236	0.926376	0.521855
CETN2	W4	PGE2	well_1	F004	165	Z distribution	0.728678	0.75718	0.511
CETN2	W4	PGE2	well_1	F005	232	Z distribution	1.162756	1.171722	0.6988
CETN2	W4	PGE2	well_1	F006	188	Z distribution	1.119558	1.191874	0.442582
CETN2	W4	PGE2	well_2	F001	110	Z distribution	0.724864	0.741044	0.559527
CETN2	W4	PGE2	well_2	F002	152	Z distribution	1.10626	1.187348	0.697009
CETN2	W4	PGE2	well_2	F003	67	Z distribution	0.681012	0.658201	0.493228
CETN2	W4	PGE2	well_2	F004	68	Z distribution	0.438917	0.55118	0.456655
CETN2	W4	PGE2	well_2	F005	156	Z distribution	1.078186	1.092553	0.777561
CETN2	W4	PGE2	well_2	F006	127	Z distribution	0.800054	0.807579	0.516698
CETN2	W4	PGE2	well_3	F001	18	Z distribution	-0.08055	-0.10458	0.436317
CETN2	W4	PGE2	well_3	F002	27	Z distribution	0.048544	0.012199	0.254713
CETN2	W4	PGE2	well_3	F003	14	Z distribution	-0.14091	-0.20596	0.25886
CETN2	W4	PGE2	well_3	F004	65	Z distribution	0.022749	-0.05594	0.494776
CETN2	W4	PGE2	well_3	F005	35	Z distribution	0.156194	0.109038	0.371199
CETN2	W4	PGE2	well_3	F006	58	Z distribution	0.574049	0.570773	0.693581
CETN2	W4	PGE2	well_4	F001	7	Z distribution	0.208752	0.176161	0.362889
CETN2	W4	PGE2	well_4	F002	10	Z distribution	0.138732	0.126561	0.208174
CETN2	W4	PGE2	well_4	F003	8	Z distribution	0.067192	0.068982	0.130585
CETN2	W4	PGE2	well_4	F004	18	Z distribution	-0.22175	-0.15418	0.302227
CETN2	W4	PGE2	well_4	F005	11	Z distribution	0.055229	0.015509	0.1853
CETN2	W4	PGE2	well_4	F006	21	Z distribution	0.266013	0.305323	0.507682
CETN2	W4	PGE2	well_5	F001	8	Z distribution	0.144502	0.11152	0.203583
CETN2	W4	PGE2	well_5	F002	16	Z distribution	-0.31131	-0.33415	0.467752
CETN2	W4	PGE2	well_5	F003	21	Z distribution	-0.05427	-0.11363	0.717483
CETN2	W4	PGE2	well_5	F004	15	Z distribution	0.081345	0.060206	0.30306
CETN2	W4	PGE2	well_5	F005	20	Z distribution	-0.02349	0.006273	0.221989
CETN2	W4	PGE2	well_5	F006	25	Z distribution	0.050714	0.037494	0.252495
SEC61B	W1	HPI4	well_1	F001	110	Avg. Volume	2.295994	2.113261	0.835575
SEC61B	W1	HPI4	well_1	F002	114	Avg. Volume	1.887419	1.736134	0.633428
SEC61B	W1	HPI4	well_1	F003	137	Avg. Volume	1.097731	1.066943	0.359021
SEC61B	W1	HPI4	well_1	F004	168	Avg. Volume	2.407177	2.326886	0.942765
SEC61B	W1	HPI4	well_1	F005	206	Avg. Volume	1.68066	1.598306	0.573058

SEC61B	W1	HPI4	well_1	F006	223	Avg. Volume	2.577138	2.488056	0.771419
SEC61B	W1	HPI4	well_2	F001	113	Avg. Volume	2.612034	2.587467	0.740285
SEC61B	W1	HPI4	well_2	F002	168	Avg. Volume	1.808374	1.714572	0.652025
SEC61B	W1	HPI4	well_2	F003	166	Avg. Volume	1.936076	1.922118	0.619422
SEC61B	W1	HPI4	well_2	F004	147	Avg. Volume	2.26086	2.147963	0.717377
SEC61B	W1	HPI4	well_2	F005	174	Avg. Volume	2.148652	2.129064	0.642618
SEC61B	W1	HPI4	well_2	F006	124	Avg. Volume	2.062243	1.924935	0.686407
SEC61B	W1	HPI4	well_3	F001	48	Avg. Volume	1.702981	1.596643	0.505177
SEC61B	W1	HPI4	well_3	F002	169	Avg. Volume	1.849673	1.775642	0.736254
SEC61B	W1	HPI4	well_3	F003	171	Avg. Volume	1.489234	1.344166	0.611476
SEC61B	W1	HPI4	well_3	F004	259	Avg. Volume	1.976853	1.901775	0.6983
SEC61B	W1	HPI4	well_3	F005	224	Avg. Volume	2.094706	2.032181	0.672551
SEC61B	W1	HPI4	well_3	F006	272	Avg. Volume	2.096141	2.029878	0.722971
SEC61B	W1	HPI4	well_4	F001	91	Avg. Volume	2.190949	2.105227	0.895334
SEC61B	W1	HPI4	well_4	F002	139	Avg. Volume	2.626839	2.574522	1.060305
SEC61B	W1	HPI4	well_4	F003	252	Avg. Volume	2.36966	2.310317	0.729425
SEC61B	W1	HPI4	well_4	F004	198	Avg. Volume	1.9236	1.801757	0.599416
SEC61B	W1	HPI4	well_4	F005	157	Avg. Volume	1.887597	1.757972	0.636472
SEC61B	W1	HPI4	well_4	F006	224	Avg. Volume	2.252806	2.186886	0.681331
SEC61B	W1	HPI4	well_5	F001	147	Avg. Volume	1.463204	1.439377	0.451443
SEC61B	W1	HPI4	well_5	F002	127	Avg. Volume	1.67613	1.649727	0.593701
SEC61B	W1	HPI4	well_5	F003	308	Avg. Volume	2.122585	1.99423	0.788269
SEC61B	W1	HPI4	well_5	F004	133	Avg. Volume	1.528459	1.486162	0.500374
SEC61B	W1	HPI4	well_5	F005	117	Avg. Volume	1.454597	1.337259	0.533912
SEC61B	W1	HPI4	well_5	F006	101	Avg. Volume	1.812222	1.78143	0.602316
SEC61B	W1	PGE2	well_1	F001	146	Avg. Volume	2.113293	1.998235	0.738603
SEC61B	W1	PGE2	well_1	F002	158	Avg. Volume	1.894857	1.787689	0.606201
SEC61B	W1	PGE2	well_1	F003	121	Avg. Volume	1.976066	1.784611	0.723888
SEC61B	W1	PGE2	well_1	F004	284	Avg. Volume	1.637448	1.586371	0.561193
SEC61B	W1	PGE2	well_1	F005	229	Avg. Volume	2.122883	2.049543	0.712126
SEC61B	W1	PGE2	well_1	F006	170	Avg. Volume	2.26159	2.10922	0.92197
SEC61B	W1	PGE2	well_2	F001	108	Avg. Volume	2.407132	2.297567	1.041144
SEC61B	W1	PGE2	well_2	F002	167	Avg. Volume	0.802586	0.754349	0.280724
SEC61B	W1	PGE2	well_2	F003	190	Avg. Volume	1.522036	1.397634	0.562539
SEC61B	W1	PGE2	well_2	F004	226	Avg. Volume	2.275557	2.280739	0.676794
SEC61B	W1	PGE2	well_2	F005	211	Avg. Volume	1.921064	1.812056	0.757067
SEC61B	W1	PGE2	well_2	F006	124	Avg. Volume	2.887651	2.936347	0.834571
SEC61B	W1	PGE2	well_3	F001	246	Avg. Volume	2.112386	2.064416	0.739712
SEC61B	W1	PGE2	well_3	F002	163	Avg. Volume	2.24031	2.119663	0.841268
SEC61B	W1	PGE2	well_3	F003	174	Avg. Volume	2.437174	2.336137	0.659009
SEC61B	W1	PGE2	well_3	F004	200	Avg. Volume	2.614311	2.570918	0.837402
SEC61B	W1	PGE2	well_3	F005	163	Avg. Volume	2.335074	2.252783	0.794272
SEC61B	W1	PGE2	well_3	F006	199	Avg. Volume	1.797917	1.744705	0.59817
SEC61B	W1	PGE2	well_4	F001	172	Avg. Volume	2.319155	2.227757	0.793605
SEC61B	W1	PGE2	well_4	F002	207	Avg. Volume	2.057936	2.051037	0.597287
SEC61B	W1	PGE2	well_4	F003	116	Avg. Volume	1.691093	1.590729	0.599576
SEC61B	W1	PGE2	well_4	F004	205	Avg. Volume	1.45287	1.401221	0.502151
SEC61B	W1	PGE2	well_4	F005	97	Avg. Volume	2.017062	1.958004	0.698038
SEC61B	W1	PGE2	well_4	F006	158	Avg. Volume	1.92446	1.834372	0.699996

SEC61B	W1	PGE2	well_5	F001	156	Avg. Volume	2.127389	2.042579	0.688899
SEC61B	W1	PGE2	well_5	F002	181	Avg. Volume	1.751785	1.649706	0.613065
SEC61B	W1	PGE2	well_5	F003	127	Avg. Volume	2.361221	2.426174	0.646456
SEC61B	W1	PGE2	well_5	F004	132	Avg. Volume	2.625477	2.41123	0.896004
SEC61B	W1	PGE2	well_5	F005	219	Avg. Volume	1.578528	1.511793	0.576352
SEC61B	W1	PGE2	well_5	F006	104	Avg. Volume	2.099142	2.039572	0.640813
SEC61B	W2	HPI4	well_1	F001	166	Avg. Volume	1.098191	0.980374	0.504508
SEC61B	W2	HPI4	well_1	F002	182	Avg. Volume	1.079585	0.971495	0.509735
SEC61B	W2	HPI4	well_1	F003	187	Avg. Volume	1.391496	1.310285	0.630571
SEC61B	W2	HPI4	well_1	F004	209	Avg. Volume	1.491716	1.392859	0.681464
SEC61B	W2	HPI4	well_1	F005	190	Avg. Volume	1.28605	1.156195	0.625437
SEC61B	W2	HPI4	well_1	F006	173	Avg. Volume	1.28017	1.081134	0.638895
SEC61B	W2	HPI4	well_2	F001	143	Avg. Volume	1.739132	1.590243	0.730768
SEC61B	W2	HPI4	well_2	F002	243	Avg. Volume	1.141521	0.998147	0.53346
SEC61B	W2	HPI4	well_2	F003	296	Avg. Volume	1.428881	1.37555	0.621011
SEC61B	W2	HPI4	well_2	F004	251	Avg. Volume	1.551049	1.516521	0.624926
SEC61B	W2	HPI4	well_2	F005	207	Avg. Volume	1.375059	1.298237	0.547138
SEC61B	W2	HPI4	well_2	F006	171	Avg. Volume	2.156342	2.021373	0.911294
SEC61B	W2	HPI4	well_3	F001	167	Avg. Volume	1.814143	1.786618	0.748006
SEC61B	W2	HPI4	well_3	F002	158	Avg. Volume	1.599844	1.546113	0.626551
SEC61B	W2	HPI4	well_3	F003	189	Avg. Volume	1.230325	1.118652	0.536012
SEC61B	W2	HPI4	well_3	F004	370	Avg. Volume	1.27111	1.203359	0.572629
SEC61B	W2	HPI4	well_3	F005	176	Avg. Volume	1.463045	1.268093	0.655386
SEC61B	W2	HPI4	well_3	F006	189	Avg. Volume	1.562402	1.570505	0.715719
SEC61B	W2	HPI4	well_4	F001	114	Avg. Volume	1.405603	1.305114	0.597901
SEC61B	W2	HPI4	well_4	F002	235	Avg. Volume	1.261349	1.113901	0.680513
SEC61B	W2	HPI4	well_4	F003	293	Avg. Volume	1.323653	1.216031	0.558357
SEC61B	W2	HPI4	well_4	F004	204	Avg. Volume	1.072353	0.856457	0.599389
SEC61B	W2	HPI4	well_4	F005	169	Avg. Volume	1.371648	1.307598	0.526144
SEC61B	W2	HPI4	well_4	F006	175	Avg. Volume	1.461609	1.329205	0.687736
SEC61B	W2	HPI4	well_5	F001	136	Avg. Volume	1.490347	1.416526	0.606888
SEC61B	W2	HPI4	well_5	F002	196	Avg. Volume	1.575478	1.514924	0.642969
SEC61B	W2	HPI4	well_5	F003	419	Avg. Volume	1.083632	0.945148	0.572328
SEC61B	W2	HPI4	well_5	F004	348	Avg. Volume	1.587786	1.47183	0.737693
SEC61B	W2	HPI4	well_5	F005	158	Avg. Volume	1.463224	1.373869	0.536292
SEC61B	W2	HPI4	well_5	F006	230	Avg. Volume	1.668483	1.487614	0.833252
SEC61B	W2	PGE2	well_1	F001	288	Avg. Volume	2.348723	2.23483	0.981276
SEC61B	W2	PGE2	well_1	F002	269	Avg. Volume	1.899654	1.734215	0.875248
SEC61B	W2	PGE2	well_1	F003	381	Avg. Volume	2.330465	2.156002	0.99713
SEC61B	W2	PGE2	well_1	F004	172	Avg. Volume	1.862352	1.754693	0.868404
SEC61B	W2	PGE2	well_1	F005	293	Avg. Volume	2.340687	2.276306	1.033375
SEC61B	W2	PGE2	well_1	F006	217	Avg. Volume	2.047759	1.940126	0.85275
SEC61B	W2	PGE2	well_2	F001	244	Avg. Volume	1.212239	1.012165	0.738417
SEC61B	W2	PGE2	well_2	F002	351	Avg. Volume	2.784942	2.722778	1.195198
SEC61B	W2	PGE2	well_2	F003	391	Avg. Volume	2.24623	2.082871	0.991638
SEC61B	W2	PGE2	well_2	F004	366	Avg. Volume	1.937469	1.85927	0.885463
SEC61B	W2	PGE2	well_2	F005	292	Avg. Volume	2.439704	2.21885	1.163726
SEC61B	W2	PGE2	well_2	F006	280	Avg. Volume	2.475747	2.296534	1.00022
SEC61B	W2	PGE2	well_3	F001	283	Avg. Volume	2.259419	2.146144	1.093138

SEC61B	W2	PGE2	well_3	F002	443	Avg. Volume	2.281771	2.199733	0.89564
SEC61B	W2	PGE2	well_3	F003	351	Avg. Volume	2.401532	2.186046	1.195496
SEC61B	W2	PGE2	well_3	F004	526	Avg. Volume	2.276028	2.115443	0.932143
SEC61B	W2	PGE2	well_3	F005	174	Avg. Volume	2.392058	2.305712	0.968922
SEC61B	W2	PGE2	well_3	F006	293	Avg. Volume	2.217081	1.99652	1.029662
SEC61B	W2	PGE2	well_4	F001	260	Avg. Volume	2.194959	2.10065	0.859862
SEC61B	W2	PGE2	well_4	F002	461	Avg. Volume	1.81498	1.770197	0.82558
SEC61B	W2	PGE2	well_4	F003	129	Avg. Volume	2.778665	2.639891	1.103641
SEC61B	W2	PGE2	well_4	F004	393	Avg. Volume	2.250342	2.064954	1.00913
SEC61B	W2	PGE2	well_4	F005	354	Avg. Volume	2.446279	2.321029	0.961008
SEC61B	W2	PGE2	well_4	F006	406	Avg. Volume	1.933516	1.729556	0.916807
SEC61B	W2	PGE2	well_5	F001	383	Avg. Volume	1.88114	1.748681	0.821005
SEC61B	W2	PGE2	well_5	F002	369	Avg. Volume	2.972662	2.816667	1.142478
SEC61B	W2	PGE2	well_5	F003	402	Avg. Volume	1.533394	1.425162	0.7544
SEC61B	W2	PGE2	well_5	F004	400	Avg. Volume	2.398219	2.345375	0.945149
SEC61B	W2	PGE2	well_5	F005	305	Avg. Volume	2.635661	2.418384	1.094815
SEC61B	W2	PGE2	well_5	F006	375	Avg. Volume	2.60424	2.416485	1.090615
SEC61B	W3	HPI4	well_1	F001	112	Avg. Volume	1.002372	0.835336	0.608987
SEC61B	W3	HPI4	well_1	F002	104	Avg. Volume	1.135801	1.011619	0.654732
SEC61B	W3	HPI4	well_1	F003	123	Avg. Volume	1.607654	1.455278	0.899343
SEC61B	W3	HPI4	well_1	F004	127	Avg. Volume	1.253158	1.155941	0.608859
SEC61B	W3	HPI4	well_1	F005	135	Avg. Volume	0.96216	0.713947	0.624408
SEC61B	W3	HPI4	well_1	F006	133	Avg. Volume	0.855615	0.658923	0.551533
SEC61B	W3	HPI4	well_2	F001	115	Avg. Volume	2.395244	2.197377	1.007736
SEC61B	W3	HPI4	well_2	F002	147	Avg. Volume	0.843587	0.711761	0.580124
SEC61B	W3	HPI4	well_2	F003	153	Avg. Volume	1.669245	1.451105	1.035398
SEC61B	W3	HPI4	well_2	F004	197	Avg. Volume	1.48495	1.418719	0.726538
SEC61B	W3	HPI4	well_2	F005	121	Avg. Volume	2.129337	2.14179	1.012214
SEC61B	W3	HPI4	well_2	F006	122	Avg. Volume	2.245345	2.130666	1.14331
SEC61B	W3	HPI4	well_3	F001	59	Avg. Volume	1.028633	0.962361	0.533268
SEC61B	W3	HPI4	well_3	F002	92	Avg. Volume	1.50588	1.392868	0.676332
SEC61B	W3	HPI4	well_3	F003	105	Avg. Volume	2.182171	2.09602	0.832256
SEC61B	W3	HPI4	well_3	F004	160	Avg. Volume	1.214472	1.051009	0.733538
SEC61B	W3	HPI4	well_3	F005	184	Avg. Volume	1.880666	1.743585	0.999731
SEC61B	W3	HPI4	well_3	F006	95	Avg. Volume	1.88349	1.672675	1.031677
SEC61B	W3	HPI4	well_4	F001	25	Avg. Volume	1.05161	0.846219	0.539598
SEC61B	W3	HPI4	well_4	F002	155	Avg. Volume	1.551907	1.429017	0.617088
SEC61B	W3	HPI4	well_4	F003	118	Avg. Volume	1.354047	1.169819	0.652149
SEC61B	W3	HPI4	well_4	F004	152	Avg. Volume	1.296396	1.244847	0.584258
SEC61B	W3	HPI4	well_4	F005	138	Avg. Volume	1.08234	0.997969	0.487383
SEC61B	W3	HPI4	well_4	F006	91	Avg. Volume	1.314832	1.135224	0.649146
SEC61B	W3	HPI4	well_5	F001	115	Avg. Volume	1.926984	1.844487	0.873632
SEC61B	W3	HPI4	well_5	F002	43	Avg. Volume	1.851712	1.773759	0.661559
SEC61B	W3	HPI4	well_5	F003	173	Avg. Volume	1.536304	1.449365	0.641736
SEC61B	W3	HPI4	well_5	F004	133	Avg. Volume	1.311454	1.169323	0.59776
SEC61B	W3	HPI4	well_5	F005	122	Avg. Volume	1.614027	1.366337	0.912906
SEC61B	W3	HPI4	well_5	F006	132	Avg. Volume	1.116826	1.074486	0.512505
SEC61B	W3	PGE2	well_1	F001	227	Avg. Volume	2.183934	1.935556	1.300271
SEC61B	W3	PGE2	well_1	F002	270	Avg. Volume	1.487667	1.364401	0.770982

SEC61B	W3	PGE2	well_1	F003	218	Avg. Volume	2.074764	1.807361	1.208943
SEC61B	W3	PGE2	well_1	F004	376	Avg. Volume	2.534666	2.473385	0.980263
SEC61B	W3	PGE2	well_1	F005	315	Avg. Volume	2.473864	2.381528	1.021605
SEC61B	W3	PGE2	well_1	F006	126	Avg. Volume	3.234513	3.238541	1.184354
SEC61B	W3	PGE2	well_2	F001	362	Avg. Volume	2.410781	2.188539	1.096297
SEC61B	W3	PGE2	well_2	F002	297	Avg. Volume	2.041383	1.841025	0.85757
SEC61B	W3	PGE2	well_2	F003	428	Avg. Volume	2.747527	2.606469	1.116225
SEC61B	W3	PGE2	well_2	F004	399	Avg. Volume	2.576446	2.437939	1.15149
SEC61B	W3	PGE2	well_2	F005	270	Avg. Volume	2.862448	2.735399	0.987124
SEC61B	W3	PGE2	well_2	F006	331	Avg. Volume	2.422127	2.300278	0.868685
SEC61B	W3	PGE2	well_3	F001	490	Avg. Volume	2.915898	2.851725	1.106727
SEC61B	W3	PGE2	well_3	F002	174	Avg. Volume	2.645621	2.648858	1.025307
SEC61B	W3	PGE2	well_3	F003	388	Avg. Volume	2.548976	2.403994	1.007803
SEC61B	W3	PGE2	well_3	F004	469	Avg. Volume	2.714176	2.634568	1.177212
SEC61B	W3	PGE2	well_3	F005	454	Avg. Volume	3.680952	3.528211	1.445178
SEC61B	W3	PGE2	well_3	F006	435	Avg. Volume	2.863764	2.811451	1.12635
SEC61B	W3	PGE2	well_4	F001	315	Avg. Volume	2.693096	2.570786	1.121339
SEC61B	W3	PGE2	well_4	F002	397	Avg. Volume	2.444583	2.367043	1.005246
SEC61B	W3	PGE2	well_4	F003	396	Avg. Volume	2.36278	2.265528	0.962634
SEC61B	W3	PGE2	well_4	F004	386	Avg. Volume	2.50275	2.391946	1.085645
SEC61B	W3	PGE2	well_4	F005	344	Avg. Volume	2.874612	2.74486	1.054484
SEC61B	W3	PGE2	well_4	F006	254	Avg. Volume	2.75053	2.616364	1.016387
SEC61B	W3	PGE2	well_5	F001	400	Avg. Volume	2.281996	2.15636	1.017882
SEC61B	W3	PGE2	well_5	F002	178	Avg. Volume	2.551351	2.435603	1.11417
SEC61B	W3	PGE2	well_5	F003	171	Avg. Volume	4.018244	3.811889	1.467852
SEC61B	W3	PGE2	well_5	F004	344	Avg. Volume	2.547006	2.367772	1.138644
SEC61B	W3	PGE2	well_5	F005	413	Avg. Volume	2.846246	2.667831	1.298026
SEC61B	W3	PGE2	well_5	F006	367	Avg. Volume	2.762475	2.593998	1.147148
SEC61B	W4	HPI4	well_1	F001	92	Avg. Volume	0.808377	0.683562	0.38948
SEC61B	W4	HPI4	well_1	F002	91	Avg. Volume	0.72243	0.695081	0.30333
SEC61B	W4	HPI4	well_1	F003	165	Avg. Volume	0.944153	0.894552	0.467953
SEC61B	W4	HPI4	well_1	F004	157	Avg. Volume	1.088465	0.967324	0.570993
SEC61B	W4	HPI4	well_1	F005	155	Avg. Volume	0.826864	0.688005	0.558725
SEC61B	W4	HPI4	well_1	F006	102	Avg. Volume	0.773458	0.708732	0.395521
SEC61B	W4	HPI4	well_2	F001	97	Avg. Volume	1.133291	1.092828	0.528654
SEC61B	W4	HPI4	well_2	F002	110	Avg. Volume	1.604186	1.471295	0.662103
SEC61B	W4	HPI4	well_2	F003	137	Avg. Volume	1.274515	1.109803	0.62924
SEC61B	W4	HPI4	well_2	F004	163	Avg. Volume	1.543413	1.444368	0.638839
SEC61B	W4	HPI4	well_2	F005	87	Avg. Volume	1.41453	1.130188	0.94528
SEC61B	W4	HPI4	well_2	F006	183	Avg. Volume	1.293185	1.207719	0.708268
SEC61B	W4	HPI4	well_3	F001	150	Avg. Volume	1.049424	0.921515	0.537047
SEC61B	W4	HPI4	well_3	F002	142	Avg. Volume	1.258337	1.117318	0.6919
SEC61B	W4	HPI4	well_3	F003	137	Avg. Volume	1.449647	1.324037	0.673779
SEC61B	W4	HPI4	well_3	F004	183	Avg. Volume	1.338231	1.328772	0.574824
SEC61B	W4	HPI4	well_3	F005	109	Avg. Volume	0.64472	0.470825	0.515845
SEC61B	W4	HPI4	well_3	F006	39	Avg. Volume	1.395933	1.259386	0.631422
SEC61B	W4	HPI4	well_4	F001	108	Avg. Volume	1.5161	1.351498	0.674263
SEC61B	W4	HPI4	well_4	F002	132	Avg. Volume	0.987928	0.91593	0.40843
SEC61B	W4	HPI4	well_4	F003	127	Avg. Volume	1.3865	1.211167	0.818105

SEC61B	W4	HPI4	well_4	F004	124	Avg. Volume	1.918193	1.690363	0.879315
SEC61B	W4	HPI4	well_4	F005	91	Avg. Volume	1.31225	1.021042	0.750476
SEC61B	W4	HPI4	well_4	F006	133	Avg. Volume	0.926628	0.71081	0.658569
SEC61B	W4	HPI4	well_5	F001	163	Avg. Volume	1.160341	1.076629	0.490339
SEC61B	W4	HPI4	well_5	F002	68	Avg. Volume	1.268473	1.199631	0.549193
SEC61B	W4	HPI4	well_5	F003	124	Avg. Volume	1.39002	1.214466	0.624873
SEC61B	W4	HPI4	well_5	F004	160	Avg. Volume	0.72373	0.627053	0.355174
SEC61B	W4	HPI4	well_5	F005	201	Avg. Volume	1.155789	1.014894	0.568027
SEC61B	W4	HPI4	well_5	F006	141	Avg. Volume	1.546166	1.447321	0.711729
SEC61B	W4	PGE2	well_1	F001	32	Avg. Volume	1.259296	1.140461	0.634272
SEC61B	W4	PGE2	well_1	F002	10	Avg. Volume	2.077247	1.60219	1.013253
SEC61B	W4	PGE2	well_1	F003	141	Avg. Volume	3.127344	2.748597	1.459874
SEC61B	W4	PGE2	well_1	F004	195	Avg. Volume	2.128224	2.007254	0.93305
SEC61B	W4	PGE2	well_1	F005	125	Avg. Volume	2.559876	2.29617	1.287363
SEC61B	W4	PGE2	well_1	F006	38	Avg. Volume	1.373391	1.188133	0.643795
SEC61B	W4	PGE2	well_2	F001	99	Avg. Volume	1.85635	1.6731	0.819342
SEC61B	W4	PGE2	well_2	F002	170	Avg. Volume	2.473042	2.329291	1.065098
SEC61B	W4	PGE2	well_2	F003	203	Avg. Volume	2.095675	1.998711	0.920134
SEC61B	W4	PGE2	well_2	F004	224	Avg. Volume	2.11989	1.998938	1.084525
SEC61B	W4	PGE2	well_2	F005	169	Avg. Volume	2.691386	2.606685	1.147813
SEC61B	W4	PGE2	well_2	F006	223	Avg. Volume	2.347754	2.204922	1.072377
SEC61B	W4	PGE2	well_3	F001	102	Avg. Volume	1.586354	1.386096	0.852937
SEC61B	W4	PGE2	well_3	F002	150	Avg. Volume	2.529751	2.439508	1.110921
SEC61B	W4	PGE2	well_3	F003	203	Avg. Volume	2.249002	1.94138	1.315808
SEC61B	W4	PGE2	well_3	F004	264	Avg. Volume	2.837363	2.688375	1.163956
SEC61B	W4	PGE2	well_3	F005	191	Avg. Volume	2.937094	2.882716	1.017171
SEC61B	W4	PGE2	well_3	F006	211	Avg. Volume	1.877181	1.727556	0.801096
SEC61B	W4	PGE2	well_4	F001	310	Avg. Volume	2.958153	2.708725	1.326614
SEC61B	W4	PGE2	well_4	F002	230	Avg. Volume	2.698978	2.513361	1.24345
SEC61B	W4	PGE2	well_4	F003	300	Avg. Volume	3.704369	3.612044	1.451459
SEC61B	W4	PGE2	well_4	F004	348	Avg. Volume	2.489868	2.298272	1.12842
SEC61B	W4	PGE2	well_4	F005	322	Avg. Volume	3.335297	3.028519	1.650315
SEC61B	W4	PGE2	well_4	F006	201	Avg. Volume	2.293226	2.1063	1.224899
SEC61B	W4	PGE2	well_5	F001	197	Avg. Volume	2.095832	1.85235	1.165666
SEC61B	W4	PGE2	well_5	F002	148	Avg. Volume	2.550154	2.44289	0.999423
SEC61B	W4	PGE2	well_5	F003	408	Avg. Volume	3.030197	2.960294	1.232615
SEC61B	W4	PGE2	well_5	F004	325	Avg. Volume	3.178798	3.082196	1.310258
SEC61B	W4	PGE2	well_5	F005	342	Avg. Volume	2.607105	2.456515	1.201397
SEC61B	W4	PGE2	well_5	F006	216	Avg. Volume	2.666682	2.647002	1.128517
SEC61B	W1	HPI4	well_1	F001	110	Count/cell	106.1327	100	32.11854
SEC61B	W1	HPI4	well_1	F002	114	Count/cell	97.52941	95.5	32.00177
SEC61B	W1	HPI4	well_1	F003	137	Count/cell	87.29268	85	31.09523
SEC61B	W1	HPI4	well_1	F004	168	Count/cell	88.74172	88	29.52907
SEC61B	W1	HPI4	well_1	F005	206	Count/cell	83.2663	79	29.84129
SEC61B	W1	HPI4	well_1	F006	223	Count/cell	79.09045	78	25.8119
SEC61B	W1	HPI4	well_2	F001	113	Count/cell	97.47573	96	31.61281
SEC61B	W1	HPI4	well_2	F002	168	Count/cell	87.25333	81.5	30.84061
SEC61B	W1	HPI4	well_2	F003	166	Count/cell	97.32667	94	31.08864
SEC61B	W1	HPI4	well_2	F004	147	Count/cell	99.65649	98	26.15303

SEC61B	W1	HPI4	well_2	F005	174	Count/cell	95.02564	95	32.85686
SEC61B	W1	HPI4	well_2	F006	124	Count/cell	95.32143	91	35.52419
SEC61B	W1	HPI4	well_3	F001	48	Count/cell	117.881	115	37.48053
SEC61B	W1	HPI4	well_3	F002	169	Count/cell	85.08497	83	30.88965
SEC61B	W1	HPI4	well_3	F003	171	Count/cell	93.24837	88	34.52426
SEC61B	W1	HPI4	well_3	F004	259	Count/cell	72.38627	71	27.31831
SEC61B	W1	HPI4	well_3	F005	224	Count/cell	77.76733	78	25.20129
SEC61B	W1	HPI4	well_3	F006	272	Count/cell	73.53279	72	28.31893
SEC61B	W1	HPI4	well_4	F001	91	Count/cell	95.2716	90	42.43525
SEC61B	W1	HPI4	well_4	F002	139	Count/cell	84.53175	79	32.12368
SEC61B	W1	HPI4	well_4	F003	252	Count/cell	77.23009	75	25.69047
SEC61B	W1	HPI4	well_4	F004	198	Count/cell	77.88202	76	24.39975
SEC61B	W1	HPI4	well_4	F005	157	Count/cell	97.88732	92	37.49971
SEC61B	W1	HPI4	well_4	F006	224	Count/cell	76.855	76.5	25.22589
SEC61B	W1	HPI4	well_5	F001	147	Count/cell	95.51908	93	34.26017
SEC61B	W1	HPI4	well_5	F002	127	Count/cell	80.35398	78	27.46554
SEC61B	W1	HPI4	well_5	F003	308	Count/cell	66.8556	62	28.8738
SEC61B	W1	HPI4	well_5	F004	133	Count/cell	92.79832	88	36.38648
SEC61B	W1	HPI4	well_5	F005	117	Count/cell	107.4476	102	37.4271
SEC61B	W1	HPI4	well_5	F006	101	Count/cell	106.4615	105	33.41001
SEC61B	W1	PGE2	well_1	F001	146	Count/cell	84.29771	86	23.23052
SEC61B	W1	PGE2	well_1	F002	158	Count/cell	87.83916	83	31.30532
SEC61B	W1	PGE2	well_1	F003	121	Count/cell	90.1	83	34.50886
SEC61B	W1	PGE2	well_1	F004	284	Count/cell	72.33071	67	29.16941
SEC61B	W1	PGE2	well_1	F005	229	Count/cell	86.56522	85	29.3614
SEC61B	W1	PGE2	well_1	F006	170	Count/cell	87.87013	83	33.91468
SEC61B	W1	PGE2	well_2	F001	108	Count/cell	100.1042	98.5	40.49756
SEC61B	W1	PGE2	well_2	F002	167	Count/cell	38.83444	35	18.31918
SEC61B	W1	PGE2	well_2	F003	190	Count/cell	90.8	84	33.08848
SEC61B	W1	PGE2	well_2	F004	226	Count/cell	79.11386	73.5	29.07858
SEC61B	W1	PGE2	well_2	F005	211	Count/cell	88.60847	86	26.94508
SEC61B	W1	PGE2	well_2	F006	124	Count/cell	99	96.5	32.61282
SEC61B	W1	PGE2	well_3	F001	246	Count/cell	78.81364	73.5	25.52386
SEC61B	W1	PGE2	well_3	F002	163	Count/cell	91.74483	85	36.91277
SEC61B	W1	PGE2	well_3	F003	174	Count/cell	89.28662	83	29.9827
SEC61B	W1	PGE2	well_3	F004	200	Count/cell	84.88333	80	27.75678
SEC61B	W1	PGE2	well_3	F005	163	Count/cell	102.4	96	37.0134
SEC61B	W1	PGE2	well_3	F006	199	Count/cell	87.88268	84	30.49724
SEC61B	W1	PGE2	well_4	F001	172	Count/cell	88.23226	85	29.01294
SEC61B	W1	PGE2	well_4	F002	207	Count/cell	91.19892	88.5	26.71771
SEC61B	W1	PGE2	well_4	F003	116	Count/cell	104.2762	98	35.05394
SEC61B	W1	PGE2	well_4	F004	205	Count/cell	86.13661	83	32.50575
SEC61B	W1	PGE2	well_4	F005	97	Count/cell	112.7931	107	34.9059
SEC61B	W1	PGE2	well_4	F006	158	Count/cell	88.07042	82	36.8169
SEC61B	W1	PGE2	well_5	F001	156	Count/cell	91.35714	89	30.67375
SEC61B	W1	PGE2	well_5	F002	181	Count/cell	84.37423	81	26.45147
SEC61B	W1	PGE2	well_5	F003	127	Count/cell	92.29204	88	27.59227
SEC61B	W1	PGE2	well_5	F004	132	Count/cell	103.3814	101.5	30.89351
SEC61B	W1	PGE2	well_5	F005	219	Count/cell	82.28788	78.5	27.71763

SEC61B	W1	PGE2	well_5	F006	104	Count/cell	100.7391	101.5	30.05668
SEC61B	W2	HPI4	well_1	F001	166	Count/cell	81.31757	76	28.0107
SEC61B	W2	HPI4	well_1	F002	182	Count/cell	73.62963	70.5	31.43245
SEC61B	W2	HPI4	well_1	F003	187	Count/cell	80.19162	79	26.21136
SEC61B	W2	HPI4	well_1	F004	209	Count/cell	80.93617	77.5	30.79892
SEC61B	W2	HPI4	well_1	F005	190	Count/cell	72.45882	66.5	31.1794
SEC61B	W2	HPI4	well_1	F006	173	Count/cell	81.33974	77.5	28.00357
SEC61B	W2	HPI4	well_2	F001	143	Count/cell	92.05512	85	32.46617
SEC61B	W2	HPI4	well_2	F002	243	Count/cell	71.64055	66	28.31911
SEC61B	W2	HPI4	well_2	F003	296	Count/cell	65.79401	63	24.66861
SEC61B	W2	HPI4	well_2	F004	251	Count/cell	71.29204	69	26.56821
SEC61B	W2	HPI4	well_2	F005	207	Count/cell	83.28191	82.5	27.94514
SEC61B	W2	HPI4	well_2	F006	171	Count/cell	86.26797	83	28.18524
SEC61B	W2	HPI4	well_3	F001	167	Count/cell	76.95973	74	23.32174
SEC61B	W2	HPI4	well_3	F002	158	Count/cell	84.89437	83	31.12722
SEC61B	W2	HPI4	well_3	F003	189	Count/cell	88.71598	89	34.89136
SEC61B	W2	HPI4	well_3	F004	370	Count/cell	59.22222	58	25.1288
SEC61B	W2	HPI4	well_3	F005	176	Count/cell	87.24528	83	33.00014
SEC61B	W2	HPI4	well_3	F006	189	Count/cell	74.02959	74	26.29445
SEC61B	W2	HPI4	well_4	F001	114	Count/cell	99.47059	98.5	32.8479
SEC61B	W2	HPI4	well_4	F002	235	Count/cell	59.79717	57	26.24297
SEC61B	W2	HPI4	well_4	F003	293	Count/cell	64.96226	62	25.84655
SEC61B	W2	HPI4	well_4	F004	204	Count/cell	67.63736	64	31.8654
SEC61B	W2	HPI4	well_4	F005	169	Count/cell	77.60265	77	26.14755
SEC61B	W2	HPI4	well_4	F006	175	Count/cell	87.38217	86	28.0879
SEC61B	W2	HPI4	well_5	F001	136	Count/cell	84.06557	82.5	29.33498
SEC61B	W2	HPI4	well_5	F002	196	Count/cell	71.38636	66.5	31.44244
SEC61B	W2	HPI4	well_5	F003	419	Count/cell	47.80952	44	22.01328
SEC61B	W2	HPI4	well_5	F004	348	Count/cell	64.01592	61	25.83747
SEC61B	W2	HPI4	well_5	F005	158	Count/cell	83.46897	82	28.30883
SEC61B	W2	HPI4	well_5	F006	230	Count/cell	77.73786	72	32.64591
SEC61B	W2	PGE2	well_1	F001	288	Count/cell	44.48659	42	14.92484
SEC61B	W2	PGE2	well_1	F002	269	Count/cell	45.44813	44	17.08186
SEC61B	W2	PGE2	well_1	F003	381	Count/cell	46.13953	43	17.30681
SEC61B	W2	PGE2	well_1	F004	172	Count/cell	35.54545	33	14.40456
SEC61B	W2	PGE2	well_1	F005	293	Count/cell	39.6203	40	15.11873
SEC61B	W2	PGE2	well_1	F006	217	Count/cell	38.04615	37	14.44659
SEC61B	W2	PGE2	well_2	F001	244	Count/cell	31.60274	28	15.89392
SEC61B	W2	PGE2	well_2	F002	351	Count/cell	40.05994	39	15.43974
SEC61B	W2	PGE2	well_2	F003	391	Count/cell	36.52975	35	14.01723
SEC61B	W2	PGE2	well_2	F004	366	Count/cell	36.28267	35	14.33577
SEC61B	W2	PGE2	well_2	F005	292	Count/cell	43.18561	42	13.99021
SEC61B	W2	PGE2	well_2	F006	280	Count/cell	40.28627	38	14.56213
SEC61B	W2	PGE2	well_3	F001	283	Count/cell	37.57422	36	13.74909
SEC61B	W2	PGE2	well_3	F002	443	Count/cell	41.33249	41	16.34153
SEC61B	W2	PGE2	well_3	F003	351	Count/cell	40.82911	40.5	15.98876
SEC61B	W2	PGE2	well_3	F004	526	Count/cell	39.41423	38.5	15.44422
SEC61B	W2	PGE2	well_3	F005	174	Count/cell	46.87821	46	15.03816
SEC61B	W2	PGE2	well_3	F006	293	Count/cell	39.07985	37	13.73486

SEC61B	W2	PGE2	well_4	F001	260	Count/cell	44.66383	43	15.59126
SEC61B	W2	PGE2	well_4	F002	461	Count/cell	37.94005	37	14.88994
SEC61B	W2	PGE2	well_4	F003	129	Count/cell	49.25862	49.5	19.54309
SEC61B	W2	PGE2	well_4	F004	393	Count/cell	40.51412	40	14.1632
SEC61B	W2	PGE2	well_4	F005	354	Count/cell	43.20872	41	16.01142
SEC61B	W2	PGE2	well_4	F006	406	Count/cell	36.82514	35	15.05541
SEC61B	W2	PGE2	well_5	F001	383	Count/cell	39.01744	37	15.74893
SEC61B	W2	PGE2	well_5	F002	369	Count/cell	43.51796	42	15.4583
SEC61B	W2	PGE2	well_5	F003	402	Count/cell	39.83056	37.5	16.82489
SEC61B	W2	PGE2	well_5	F004	400	Count/cell	42.04396	42	16.13522
SEC61B	W2	PGE2	well_5	F005	305	Count/cell	45.69708	45	13.18005
SEC61B	W2	PGE2	well_5	F006	375	Count/cell	43.35799	42	16.10018
SEC61B	W3	HPI4	well_1	F001	112	Count/cell	94.7	93	33.48345
SEC61B	W3	HPI4	well_1	F002	104	Count/cell	102.5109	103.5	25.29322
SEC61B	W3	HPI4	well_1	F003	123	Count/cell	78.22936	79	27.53168
SEC61B	W3	HPI4	well_1	F004	127	Count/cell	81.95614	76	30.18949
SEC61B	W3	HPI4	well_1	F005	135	Count/cell	85.57851	85	34.64721
SEC61B	W3	HPI4	well_1	F006	133	Count/cell	73.59664	71	28.02611
SEC61B	W3	HPI4	well_2	F001	115	Count/cell	87.84615	86.5	29.98568
SEC61B	W3	HPI4	well_2	F002	147	Count/cell	66.27481	61	26.38474
SEC61B	W3	HPI4	well_2	F003	153	Count/cell	67.58696	64	25.24471
SEC61B	W3	HPI4	well_2	F004	197	Count/cell	64.25424	63	25.48814
SEC61B	W3	HPI4	well_2	F005	121	Count/cell	85.42202	76	33.19479
SEC61B	W3	HPI4	well_2	F006	122	Count/cell	84.0367	80	31.77203
SEC61B	W3	HPI4	well_3	F001	59	Count/cell	88.83019	87	33.9676
SEC61B	W3	HPI4	well_3	F002	92	Count/cell	104.253	104	39.62379
SEC61B	W3	HPI4	well_3	F003	105	Count/cell	84.56989	82	25.87907
SEC61B	W3	HPI4	well_3	F004	160	Count/cell	78.19444	77.5	28.03925
SEC61B	W3	HPI4	well_3	F005	184	Count/cell	73.96951	73	29.69857
SEC61B	W3	HPI4	well_3	F006	95	Count/cell	109.5294	107	28.36563
SEC61B	W3	HPI4	well_4	F001	25	Count/cell	103.6667	94	35.56731
SEC61B	W3	HPI4	well_4	F002	155	Count/cell	85.40714	81	32.26417
SEC61B	W3	HPI4	well_4	F003	118	Count/cell	92.57547	86.5	35.54526
SEC61B	W3	HPI4	well_4	F004	152	Count/cell	75.75	68	32.14867
SEC61B	W3	HPI4	well_4	F005	138	Count/cell	91.3629	85.5	34.38598
SEC61B	W3	HPI4	well_4	F006	91	Count/cell	101.1111	101	36.13551
SEC61B	W3	HPI4	well_5	F001	115	Count/cell	106.2427	103	36.76941
SEC61B	W3	HPI4	well_5	F002	43	Count/cell	98.7027	94	25.24201
SEC61B	W3	HPI4	well_5	F003	173	Count/cell	82.22581	78	28.43095
SEC61B	W3	HPI4	well_5	F004	133	Count/cell	79.20168	80	27.8256
SEC61B	W3	HPI4	well_5	F005	122	Count/cell	92.01852	91	29.8457
SEC61B	W3	HPI4	well_5	F006	132	Count/cell	98.9916	98	37.94007
SEC61B	W3	PGE2	well_1	F001	227	Count/cell	36.47291	37	13.10442
SEC61B	W3	PGE2	well_1	F002	270	Count/cell	28.89431	28	11.94874
SEC61B	W3	PGE2	well_1	F003	218	Count/cell	34.7602	35	12.80619
SEC61B	W3	PGE2	well_1	F004	376	Count/cell	36.28152	35	11.28911
SEC61B	W3	PGE2	well_1	F005	315	Count/cell	34.59862	32	12.06168
SEC61B	W3	PGE2	well_1	F006	126	Count/cell	49.51304	51	17.43628
SEC61B	W3	PGE2	well_2	F001	362	Count/cell	41.21472	40	11.83229

SEC61B	W3	PGE2	well_2	F002	297	Count/cell	38.61338	37	12.96807
SEC61B	W3	PGE2	well_2	F003	428	Count/cell	42.53351	41	12.65223
SEC61B	W3	PGE2	well_2	F004	399	Count/cell	43.05525	42	13.91296
SEC61B	W3	PGE2	well_2	F005	270	Count/cell	40.14403	38	13.03681
SEC61B	W3	PGE2	well_2	F006	331	Count/cell	39.46667	38	14.13204
SEC61B	W3	PGE2	well_3	F001	490	Count/cell	38.77045	38	12.37915
SEC61B	W3	PGE2	well_3	F002	174	Count/cell	50.19745	47	15.54499
SEC61B	W3	PGE2	well_3	F003	388	Count/cell	40.6034	40	12.39071
SEC61B	W3	PGE2	well_3	F004	469	Count/cell	39.45327	39	11.98595
SEC61B	W3	PGE2	well_3	F005	454	Count/cell	33.84878	32	9.873605
SEC61B	W3	PGE2	well_3	F006	435	Count/cell	40.86768	39	11.87425
SEC61B	W3	PGE2	well_4	F001	315	Count/cell	44.03169	43	14.44433
SEC61B	W3	PGE2	well_4	F002	397	Count/cell	39.04749	38	11.63716
SEC61B	W3	PGE2	well_4	F003	396	Count/cell	41.59551	42	12.9238
SEC61B	W3	PGE2	well_4	F004	386	Count/cell	36.9711	36	10.79046
SEC61B	W3	PGE2	well_4	F005	344	Count/cell	40.32154	39	12.7208
SEC61B	W3	PGE2	well_4	F006	254	Count/cell	44.43534	43.5	13.43753
SEC61B	W3	PGE2	well_5	F001	400	Count/cell	38.50276	36	14.78531
SEC61B	W3	PGE2	well_5	F002	178	Count/cell	35.34161	33	12.08051
SEC61B	W3	PGE2	well_5	F003	171	Count/cell	35.5817	34	12.14258
SEC61B	W3	PGE2	well_5	F004	344	Count/cell	32.84295	31	11.95588
SEC61B	W3	PGE2	well_5	F005	413	Count/cell	37.35467	36	12.03935
SEC61B	W3	PGE2	well_5	F006	367	Count/cell	38.9848	38	11.9869
SEC61B	W4	HPI4	well_1	F001	92	Count/cell	89.55422	89	29.97082
SEC61B	W4	HPI4	well_1	F002	91	Count/cell	82.18519	82	30.07745
SEC61B	W4	HPI4	well_1	F003	165	Count/cell	78.78912	76	30.78566
SEC61B	W4	HPI4	well_1	F004	157	Count/cell	81.06294	79	34.34441
SEC61B	W4	HPI4	well_1	F005	155	Count/cell	64.03597	58	33.85506
SEC61B	W4	HPI4	well_1	F006	102	Count/cell	85.16304	86	31.70096
SEC61B	W4	HPI4	well_2	F001	97	Count/cell	112.9773	109.5	36.63174
SEC61B	W4	HPI4	well_2	F002	110	Count/cell	104.7959	107.5	34.76696
SEC61B	W4	HPI4	well_2	F003	137	Count/cell	94.50407	94	31.54839
SEC61B	W4	HPI4	well_2	F004	163	Count/cell	88.12414	84	24.17152
SEC61B	W4	HPI4	well_2	F005	87	Count/cell	73.96154	76	30.71843
SEC61B	W4	HPI4	well_2	F006	183	Count/cell	61.96319	59	25.02169
SEC61B	W4	HPI4	well_3	F001	150	Count/cell	95.84444	96	34.19345
SEC61B	W4	HPI4	well_3	F002	142	Count/cell	83.14844	77	32.14585
SEC61B	W4	HPI4	well_3	F003	137	Count/cell	86.68293	81	32.17277
SEC61B	W4	HPI4	well_3	F004	183	Count/cell	89.30061	85	30.35675
SEC61B	W4	HPI4	well_3	F005	109	Count/cell	31.83505	32	16.64124
SEC61B	W4	HPI4	well_3	F006	39	Count/cell	87.11429	87	27.41379
SEC61B	W4	HPI4	well_4	F001	108	Count/cell	118.125	122	44.44589
SEC61B	W4	HPI4	well_4	F002	132	Count/cell	94.97479	92	35.8645
SEC61B	W4	HPI4	well_4	F003	127	Count/cell	95.19469	86	39.97764
SEC61B	W4	HPI4	well_4	F004	124	Count/cell	100.5818	97.5	27.41639
SEC61B	W4	HPI4	well_4	F005	91	Count/cell	82.62963	77	40.04855
SEC61B	W4	HPI4	well_4	F006	133	Count/cell	57.00833	55.5	29.1226
SEC61B	W4	HPI4	well_5	F001	163	Count/cell	84.82069	77	32.58571
SEC61B	W4	HPI4	well_5	F002	68	Count/cell	108.3167	109.5	28.77233

SEC61B	W4	HPI4	well_5	F003	124	Count/cell	85.85455	82	32.3929
SEC61B	W4	HPI4	well_5	F004	160	Count/cell	68.4375	64.5	32.33494
SEC61B	W4	HPI4	well_5	F005	201	Count/cell	79.88398	77	30.70311
SEC61B	W4	HPI4	well_5	F006	141	Count/cell	89.3937	87	29.34176
SEC61B	W4	PGE2	well_1	F001	32	Count/cell	32.10714	30.5	11.69469
SEC61B	W4	PGE2	well_1	F002	10	Count/cell	34.25	34.5	11.68332
SEC61B	W4	PGE2	well_1	F003	141	Count/cell	32.74219	31	9.733655
SEC61B	W4	PGE2	well_1	F004	195	Count/cell	30.6236	30.5	10.54337
SEC61B	W4	PGE2	well_1	F005	125	Count/cell	31.09009	31	10.17086
SEC61B	W4	PGE2	well_1	F006	38	Count/cell	33.14706	31.5	9.655058
SEC61B	W4	PGE2	well_2	F001	99	Count/cell	31.95556	28.5	12.56883
SEC61B	W4	PGE2	well_2	F002	170	Count/cell	32.69935	31	12.05638
SEC61B	W4	PGE2	well_2	F003	203	Count/cell	46.51351	47	16.39628
SEC61B	W4	PGE2	well_2	F004	224	Count/cell	28.30882	28	9.760568
SEC61B	W4	PGE2	well_2	F005	169	Count/cell	34.16993	32	10.96033
SEC61B	W4	PGE2	well_2	F006	223	Count/cell	37.0495	36	13.27915
SEC61B	W4	PGE2	well_3	F001	102	Count/cell	33.15556	31	14.0328
SEC61B	W4	PGE2	well_3	F002	150	Count/cell	39.56296	38	13.07305
SEC61B	W4	PGE2	well_3	F003	203	Count/cell	28.01093	27	12.05299
SEC61B	W4	PGE2	well_3	F004	264	Count/cell	34.61925	33	11.50052
SEC61B	W4	PGE2	well_3	F005	191	Count/cell	39.71839	38.5	11.96507
SEC61B	W4	PGE2	well_3	F006	211	Count/cell	41.99476	40	16.11456
SEC61B	W4	PGE2	well_4	F001	310	Count/cell	33.23571	32	11.47484
SEC61B	W4	PGE2	well_4	F002	230	Count/cell	40.91304	39	13.35229
SEC61B	W4	PGE2	well_4	F003	300	Count/cell	35.78519	33	12.2892
SEC61B	W4	PGE2	well_4	F004	348	Count/cell	31.30769	31	10.86196
SEC61B	W4	PGE2	well_4	F005	322	Count/cell	33.90345	32	11.50708
SEC61B	W4	PGE2	well_4	F006	201	Count/cell	38.13587	37	15.05865
SEC61B	W4	PGE2	well_5	F001	197	Count/cell	39.99435	38	15.16931
SEC61B	W4	PGE2	well_5	F002	148	Count/cell	42.0597	40	14.9645
SEC61B	W4	PGE2	well_5	F003	408	Count/cell	34.23514	34	11.9865
SEC61B	W4	PGE2	well_5	F004	325	Count/cell	35.47603	34	11.93436
SEC61B	W4	PGE2	well_5	F005	342	Count/cell	32.40196	31	11.20503
SEC61B	W4	PGE2	well_5	F006	216	Count/cell	40.71939	39	12.33373
SEC61B	W1	HPI4	well_1	F001	110	X/Y distribution	9.237339	9.380144	1.502443
SEC61B	W1	HPI4	well_1	F002	114	X/Y distribution	8.552221	8.330674	1.422635
SEC61B	W1	HPI4	well_1	F003	137	X/Y distribution	8.131389	8.07851	1.313347
SEC61B	W1	HPI4	well_1	F004	168	X/Y distribution	7.840207	7.667374	1.390841
SEC61B	W1	HPI4	well_1	F005	206	X/Y distribution	7.591502	7.359899	1.347375
SEC61B	W1	HPI4	well_1	F006	223	X/Y distribution	7.577693	7.455039	1.546033
SEC61B	W1	HPI4	well_2	F001	113	X/Y distribution	8.46299	8.417454	1.367294
SEC61B	W1	HPI4	well_2	F002	168	X/Y distribution	7.915651	7.775333	1.368555
SEC61B	W1	HPI4	well_2	F003	166	X/Y distribution	8.200301	8.179733	1.302502
SEC61B	W1	HPI4	well_2	F004	147	X/Y distribution	8.295925	8.044598	1.408921
SEC61B	W1	HPI4	well_2	F005	174	X/Y distribution	8.037087	7.867144	1.420912
SEC61B	W1	HPI4	well_2	F006	124	X/Y distribution	8.396184	8.287307	1.482515
SEC61B	W1	HPI4	well_3	F001	48	X/Y distribution	10.7594	10.77558	1.757351
SEC61B	W1	HPI4	well_3	F002	169	X/Y distribution	8.089713	7.999943	1.268122
SEC61B	W1	HPI4	well_3	F003	171	X/Y distribution	8.158042	8.058585	1.293602

SEC61B	W1	HPI4	well_3	F004	259	X/Y distribution	7.184286	7.13838	1.211669
SEC61B	W1	HPI4	well_3	F005	224	X/Y distribution	7.307868	7.1691	1.228391
SEC61B	W1	HPI4	well_3	F006	272	X/Y distribution	6.99918	6.937643	1.278626
SEC61B	W1	HPI4	well_4	F001	91	X/Y distribution	8.256006	8.339921	1.557392
SEC61B	W1	HPI4	well_4	F002	139	X/Y distribution	7.47779	7.181592	1.412456
SEC61B	W1	HPI4	well_4	F003	252	X/Y distribution	6.89318	6.891085	1.099021
SEC61B	W1	HPI4	well_4	F004	198	X/Y distribution	7.67114	7.57533	0.999902
SEC61B	W1	HPI4	well_4	F005	157	X/Y distribution	8.544678	8.583768	1.563331
SEC61B	W1	HPI4	well_4	F006	224	X/Y distribution	7.658507	7.762779	1.271764
SEC61B	W1	HPI4	well_5	F001	147	X/Y distribution	8.645747	8.830504	1.414512
SEC61B	W1	HPI4	well_5	F002	127	X/Y distribution	8.20464	8.14341	1.357722
SEC61B	W1	HPI4	well_5	F003	308	X/Y distribution	6.36624	6.372923	1.085529
SEC61B	W1	HPI4	well_5	F004	133	X/Y distribution	8.020092	8.098449	1.49074
SEC61B	W1	HPI4	well_5	F005	117	X/Y distribution	8.801606	8.683873	1.51012
SEC61B	W1	HPI4	well_5	F006	101	X/Y distribution	9.785166	9.70245	1.706249
SEC61B	W1	PGE2	well_1	F001	146	X/Y distribution	8.743775	8.731369	1.26935
SEC61B	W1	PGE2	well_1	F002	158	X/Y distribution	8.529671	8.490778	1.163487
SEC61B	W1	PGE2	well_1	F003	121	X/Y distribution	9.238831	9.172507	1.453226
SEC61B	W1	PGE2	well_1	F004	284	X/Y distribution	7.232248	7.167347	1.290306
SEC61B	W1	PGE2	well_1	F005	229	X/Y distribution	7.578681	7.590464	1.28892
SEC61B	W1	PGE2	well_1	F006	170	X/Y distribution	8.465331	8.35393	1.459176
SEC61B	W1	PGE2	well_2	F001	108	X/Y distribution	9.198568	9.140916	1.468228
SEC61B	W1	PGE2	well_2	F002	167	X/Y distribution	7.690529	7.609931	1.114248
SEC61B	W1	PGE2	well_2	F003	190	X/Y distribution	8.366886	8.364043	1.405041
SEC61B	W1	PGE2	well_2	F004	226	X/Y distribution	7.39993	7.362194	1.302728
SEC61B	W1	PGE2	well_2	F005	211	X/Y distribution	7.571441	7.410258	1.385794
SEC61B	W1	PGE2	well_2	F006	124	X/Y distribution	8.629216	8.107963	1.692343
SEC61B	W1	PGE2	well_3	F001	246	X/Y distribution	7.657841	7.593174	1.048151
SEC61B	W1	PGE2	well_3	F002	163	X/Y distribution	8.160721	7.866945	1.639663
SEC61B	W1	PGE2	well_3	F003	174	X/Y distribution	8.093527	8.108114	1.305912
SEC61B	W1	PGE2	well_3	F004	200	X/Y distribution	7.72898	7.557319	1.327854
SEC61B	W1	PGE2	well_3	F005	163	X/Y distribution	8.43169	8.230894	1.657394
SEC61B	W1	PGE2	well_3	F006	199	X/Y distribution	7.818339	7.557432	1.4179
SEC61B	W1	PGE2	well_4	F001	172	X/Y distribution	8.092031	8.063878	1.316738
SEC61B	W1	PGE2	well_4	F002	207	X/Y distribution	8.052151	8.034853	1.353482
SEC61B	W1	PGE2	well_4	F003	116	X/Y distribution	8.975101	8.913537	1.186666
SEC61B	W1	PGE2	well_4	F004	205	X/Y distribution	7.834082	7.769259	1.272502
SEC61B	W1	PGE2	well_4	F005	97	X/Y distribution	9.690515	9.895121	1.590094
SEC61B	W1	PGE2	well_4	F006	158	X/Y distribution	8.014209	7.925137	1.648206
SEC61B	W1	PGE2	well_5	F001	156	X/Y distribution	8.303573	8.19993	1.274988
SEC61B	W1	PGE2	well_5	F002	181	X/Y distribution	8.235754	8.103774	1.215762
SEC61B	W1	PGE2	well_5	F003	127	X/Y distribution	8.770384	8.587184	1.312621
SEC61B	W1	PGE2	well_5	F004	132	X/Y distribution	8.538522	8.411176	1.381362
SEC61B	W1	PGE2	well_5	F005	219	X/Y distribution	7.272362	7.167401	1.271482
SEC61B	W1	PGE2	well_5	F006	104	X/Y distribution	8.627704	8.641307	1.593947
SEC61B	W2	HPI4	well_1	F001	166	X/Y distribution	7.986198	7.951022	1.166512
SEC61B	W2	HPI4	well_1	F002	182	X/Y distribution	7.728339	7.698514	1.485906
SEC61B	W2	HPI4	well_1	F003	187	X/Y distribution	8.096456	8.109127	1.213253
SEC61B	W2	HPI4	well_1	F004	209	X/Y distribution	8.024998	7.982533	1.530546

SEC61B	W2	HPI4	well_1	F005	190	X/Y distribution	7.349548	7.473603	1.762705
SEC61B	W2	HPI4	well_1	F006	173	X/Y distribution	8.197798	8.075104	1.409424
SEC61B	W2	HPI4	well_2	F001	143	X/Y distribution	8.800399	8.671648	1.327976
SEC61B	W2	HPI4	well_2	F002	243	X/Y distribution	7.103593	7.119657	1.256566
SEC61B	W2	HPI4	well_2	F003	296	X/Y distribution	7.103045	6.922735	1.249321
SEC61B	W2	HPI4	well_2	F004	251	X/Y distribution	7.23737	7.251989	1.317248
SEC61B	W2	HPI4	well_2	F005	207	X/Y distribution	8.091041	8.171303	1.390137
SEC61B	W2	HPI4	well_2	F006	171	X/Y distribution	8.431296	8.396654	1.218054
SEC61B	W2	HPI4	well_3	F001	167	X/Y distribution	8.147838	8.1289	1.28645
SEC61B	W2	HPI4	well_3	F002	158	X/Y distribution	8.590108	8.559026	1.484868
SEC61B	W2	HPI4	well_3	F003	189	X/Y distribution	8.236174	8.15523	1.570665
SEC61B	W2	HPI4	well_3	F004	370	X/Y distribution	6.650819	6.696134	1.227977
SEC61B	W2	HPI4	well_3	F005	176	X/Y distribution	8.434495	8.393241	1.401474
SEC61B	W2	HPI4	well_3	F006	189	X/Y distribution	7.965584	7.883953	1.407029
SEC61B	W2	HPI4	well_4	F001	114	X/Y distribution	8.576962	8.53581	1.273729
SEC61B	W2	HPI4	well_4	F002	235	X/Y distribution	6.957391	6.849638	1.407207
SEC61B	W2	HPI4	well_4	F003	293	X/Y distribution	7.14658	6.945265	1.393371
SEC61B	W2	HPI4	well_4	F004	204	X/Y distribution	7.614454	7.31356	1.623392
SEC61B	W2	HPI4	well_4	F005	169	X/Y distribution	7.920652	7.831327	1.283882
SEC61B	W2	HPI4	well_4	F006	175	X/Y distribution	8.403631	8.459713	1.426781
SEC61B	W2	HPI4	well_5	F001	136	X/Y distribution	8.233383	8.312177	1.369224
SEC61B	W2	HPI4	well_5	F002	196	X/Y distribution	8.038324	8.069274	1.355535
SEC61B	W2	HPI4	well_5	F003	419	X/Y distribution	5.902761	5.958951	1.116726
SEC61B	W2	HPI4	well_5	F004	348	X/Y distribution	6.55877	6.425804	1.235186
SEC61B	W2	HPI4	well_5	F005	158	X/Y distribution	8.26413	8.199067	1.428633
SEC61B	W2	HPI4	well_5	F006	230	X/Y distribution	7.598815	7.658368	1.629995
SEC61B	W2	PGE2	well_1	F001	288	X/Y distribution	5.626771	5.663613	0.704056
SEC61B	W2	PGE2	well_1	F002	269	X/Y distribution	5.490135	5.471648	0.793038
SEC61B	W2	PGE2	well_1	F003	381	X/Y distribution	5.925538	5.816857	0.933311
SEC61B	W2	PGE2	well_1	F004	172	X/Y distribution	5.201299	5.295276	0.848371
SEC61B	W2	PGE2	well_1	F005	293	X/Y distribution	5.33106	5.441664	0.801737
SEC61B	W2	PGE2	well_1	F006	217	X/Y distribution	5.054438	5.140374	0.722695
SEC61B	W2	PGE2	well_2	F001	244	X/Y distribution	5.452936	5.478592	0.869302
SEC61B	W2	PGE2	well_2	F002	351	X/Y distribution	5.34532	5.323219	0.783276
SEC61B	W2	PGE2	well_2	F003	391	X/Y distribution	5.189145	5.169281	0.710386
SEC61B	W2	PGE2	well_2	F004	366	X/Y distribution	5.13778	5.115165	0.720031
SEC61B	W2	PGE2	well_2	F005	292	X/Y distribution	5.677218	5.6425	0.685862
SEC61B	W2	PGE2	well_2	F006	280	X/Y distribution	5.363657	5.371391	0.761716
SEC61B	W2	PGE2	well_3	F001	283	X/Y distribution	5.463704	5.439697	0.805142
SEC61B	W2	PGE2	well_3	F002	443	X/Y distribution	5.182223	5.144882	0.707289
SEC61B	W2	PGE2	well_3	F003	351	X/Y distribution	5.355839	5.12466	1.050939
SEC61B	W2	PGE2	well_3	F004	526	X/Y distribution	5.482457	5.511369	0.902562
SEC61B	W2	PGE2	well_3	F005	174	X/Y distribution	5.155091	5.035365	0.672735
SEC61B	W2	PGE2	well_3	F006	293	X/Y distribution	5.186661	5.200314	0.742967
SEC61B	W2	PGE2	well_4	F001	260	X/Y distribution	5.729909	5.710583	0.866721
SEC61B	W2	PGE2	well_4	F002	461	X/Y distribution	5.415709	5.347366	0.868717
SEC61B	W2	PGE2	well_4	F003	129	X/Y distribution	5.97014	5.888882	1.004909
SEC61B	W2	PGE2	well_4	F004	393	X/Y distribution	5.267865	5.246302	0.713283
SEC61B	W2	PGE2	well_4	F005	354	X/Y distribution	5.346909	5.314436	0.747676

SEC61B	W2	PGE2	well_4	F006	406	X/Y distribution	5.273631	5.271016	0.749907
SEC61B	W2	PGE2	well_5	F001	383	X/Y distribution	5.614168	5.565184	0.858027
SEC61B	W2	PGE2	well_5	F002	369	X/Y distribution	5.50183	5.448987	0.711936
SEC61B	W2	PGE2	well_5	F003	402	X/Y distribution	6.047411	5.97532	1.135939
SEC61B	W2	PGE2	well_5	F004	400	X/Y distribution	5.649119	5.563271	0.813632
SEC61B	W2	PGE2	well_5	F005	305	X/Y distribution	5.783193	5.75138	0.803513
SEC61B	W2	PGE2	well_5	F006	375	X/Y distribution	5.581173	5.531207	0.800543
SEC61B	W3	HPI4	well_1	F001	112	X/Y distribution	8.866156	8.852768	1.615907
SEC61B	W3	HPI4	well_1	F002	104	X/Y distribution	8.920299	8.932146	1.234259
SEC61B	W3	HPI4	well_1	F003	123	X/Y distribution	8.145291	8.092603	1.545995
SEC61B	W3	HPI4	well_1	F004	127	X/Y distribution	7.94703	7.733759	1.406917
SEC61B	W3	HPI4	well_1	F005	135	X/Y distribution	8.708069	8.73689	1.415035
SEC61B	W3	HPI4	well_1	F006	133	X/Y distribution	8.31405	8.321591	1.36903
SEC61B	W3	HPI4	well_2	F001	115	X/Y distribution	8.946105	8.986712	1.46325
SEC61B	W3	HPI4	well_2	F002	147	X/Y distribution	7.66529	7.640571	1.214142
SEC61B	W3	HPI4	well_2	F003	153	X/Y distribution	7.285108	7.317595	1.396438
SEC61B	W3	HPI4	well_2	F004	197	X/Y distribution	7.031083	6.950057	1.444709
SEC61B	W3	HPI4	well_2	F005	121	X/Y distribution	8.608679	8.593096	1.624443
SEC61B	W3	HPI4	well_2	F006	122	X/Y distribution	8.669298	8.782313	1.521217
SEC61B	W3	HPI4	well_3	F001	59	X/Y distribution	8.762798	8.949859	1.638918
SEC61B	W3	HPI4	well_3	F002	92	X/Y distribution	8.961788	9.422911	1.777592
SEC61B	W3	HPI4	well_3	F003	105	X/Y distribution	8.844177	8.759692	1.488729
SEC61B	W3	HPI4	well_3	F004	160	X/Y distribution	7.852628	7.913426	1.455115
SEC61B	W3	HPI4	well_3	F005	184	X/Y distribution	8.015192	7.9316	1.534614
SEC61B	W3	HPI4	well_3	F006	95	X/Y distribution	9.788409	9.740104	1.486838
SEC61B	W3	HPI4	well_4	F001	25	X/Y distribution	9.180352	9.290027	1.39331
SEC61B	W3	HPI4	well_4	F002	155	X/Y distribution	8.294297	8.310502	1.755773
SEC61B	W3	HPI4	well_4	F003	118	X/Y distribution	8.773683	8.82947	1.476105
SEC61B	W3	HPI4	well_4	F004	152	X/Y distribution	7.647707	7.440381	1.426377
SEC61B	W3	HPI4	well_4	F005	138	X/Y distribution	8.652615	8.444631	1.466536
SEC61B	W3	HPI4	well_4	F006	91	X/Y distribution	8.619899	8.330611	1.5355
SEC61B	W3	HPI4	well_5	F001	115	X/Y distribution	9.646472	9.604591	1.819687
SEC61B	W3	HPI4	well_5	F002	43	X/Y distribution	7.960752	7.984177	1.023322
SEC61B	W3	HPI4	well_5	F003	173	X/Y distribution	7.860308	7.676285	1.190258
SEC61B	W3	HPI4	well_5	F004	133	X/Y distribution	7.706714	7.602504	1.320354
SEC61B	W3	HPI4	well_5	F005	122	X/Y distribution	8.447581	8.096583	1.299782
SEC61B	W3	HPI4	well_5	F006	132	X/Y distribution	8.677333	8.52486	1.54141
SEC61B	W3	PGE2	well_1	F001	227	X/Y distribution	5.786782	5.752469	0.665521
SEC61B	W3	PGE2	well_1	F002	270	X/Y distribution	5.271571	5.331739	0.694109
SEC61B	W3	PGE2	well_1	F003	218	X/Y distribution	5.333028	5.243193	0.75489
SEC61B	W3	PGE2	well_1	F004	376	X/Y distribution	5.273258	5.250699	0.557997
SEC61B	W3	PGE2	well_1	F005	315	X/Y distribution	5.304013	5.297971	0.494064
SEC61B	W3	PGE2	well_1	F006	126	X/Y distribution	5.374437	5.371297	0.61224
SEC61B	W3	PGE2	well_2	F001	362	X/Y distribution	5.633916	5.67179	0.635287
SEC61B	W3	PGE2	well_2	F002	297	X/Y distribution	5.565097	5.500688	0.703339
SEC61B	W3	PGE2	well_2	F003	428	X/Y distribution	5.42548	5.390757	0.590666
SEC61B	W3	PGE2	well_2	F004	399	X/Y distribution	5.56462	5.53802	0.664705
SEC61B	W3	PGE2	well_2	F005	270	X/Y distribution	5.391133	5.319814	0.657019
SEC61B	W3	PGE2	well_2	F006	331	X/Y distribution	5.431859	5.399182	0.596931

SEC61B	W3	PGE2	well_3	F001	490	X/Y distribution	5.249088	5.24041	0.595425
SEC61B	W3	PGE2	well_3	F002	174	X/Y distribution	5.703399	5.508318	0.764656
SEC61B	W3	PGE2	well_3	F003	388	X/Y distribution	5.298267	5.256578	0.598917
SEC61B	W3	PGE2	well_3	F004	469	X/Y distribution	5.445994	5.474976	0.606617
SEC61B	W3	PGE2	well_3	F005	454	X/Y distribution	5.260044	5.241151	0.593676
SEC61B	W3	PGE2	well_3	F006	435	X/Y distribution	5.462121	5.437973	0.56334
SEC61B	W3	PGE2	well_4	F001	315	X/Y distribution	5.502579	5.512733	0.683924
SEC61B	W3	PGE2	well_4	F002	397	X/Y distribution	5.403988	5.386553	0.581652
SEC61B	W3	PGE2	well_4	F003	396	X/Y distribution	5.506094	5.497952	0.649939
SEC61B	W3	PGE2	well_4	F004	386	X/Y distribution	5.253952	5.193776	0.667511
SEC61B	W3	PGE2	well_4	F005	344	X/Y distribution	5.359558	5.343469	0.609191
SEC61B	W3	PGE2	well_4	F006	254	X/Y distribution	5.531984	5.512042	0.643202
SEC61B	W3	PGE2	well_5	F001	400	X/Y distribution	5.136669	5.101632	0.735225
SEC61B	W3	PGE2	well_5	F002	178	X/Y distribution	5.397692	5.396672	0.55066
SEC61B	W3	PGE2	well_5	F003	171	X/Y distribution	5.469409	5.410106	0.721333
SEC61B	W3	PGE2	well_5	F004	344	X/Y distribution	5.021339	4.939073	0.657922
SEC61B	W3	PGE2	well_5	F005	413	X/Y distribution	5.208383	5.152113	0.630865
SEC61B	W3	PGE2	well_5	F006	367	X/Y distribution	5.257194	5.263128	0.653471
SEC61B	W4	HPI4	well_1	F001	92	X/Y distribution	8.514396	8.339409	1.288547
SEC61B	W4	HPI4	well_1	F002	91	X/Y distribution	8.366822	8.207925	1.262992
SEC61B	W4	HPI4	well_1	F003	165	X/Y distribution	7.754412	7.715081	1.439559
SEC61B	W4	HPI4	well_1	F004	157	X/Y distribution	7.938729	7.591111	1.340413
SEC61B	W4	HPI4	well_1	F005	155	X/Y distribution	7.692429	7.686201	1.447414
SEC61B	W4	HPI4	well_1	F006	102	X/Y distribution	8.394167	8.330984	1.594548
SEC61B	W4	HPI4	well_2	F001	97	X/Y distribution	9.221406	9.052064	1.144971
SEC61B	W4	HPI4	well_2	F002	110	X/Y distribution	9.533887	9.487415	1.568691
SEC61B	W4	HPI4	well_2	F003	137	X/Y distribution	8.581928	8.66055	1.389319
SEC61B	W4	HPI4	well_2	F004	163	X/Y distribution	8.856096	8.917632	1.286025
SEC61B	W4	HPI4	well_2	F005	87	X/Y distribution	7.928439	8.173112	1.578247
SEC61B	W4	HPI4	well_2	F006	183	X/Y distribution	7.348064	7.302785	1.353487
SEC61B	W4	HPI4	well_3	F001	150	X/Y distribution	8.535494	8.441907	1.313388
SEC61B	W4	HPI4	well_3	F002	142	X/Y distribution	8.34673	8.090483	1.469265
SEC61B	W4	HPI4	well_3	F003	137	X/Y distribution	8.387301	8.429766	1.490084
SEC61B	W4	HPI4	well_3	F004	183	X/Y distribution	8.091382	8.298206	1.527136
SEC61B	W4	HPI4	well_3	F005	109	X/Y distribution	7.325921	7.340736	1.759206
SEC61B	W4	HPI4	well_3	F006	39	X/Y distribution	8.657797	8.516461	1.480277
SEC61B	W4	HPI4	well_4	F001	108	X/Y distribution	9.524214	9.137456	1.931245
SEC61B	W4	HPI4	well_4	F002	132	X/Y distribution	8.723795	8.735269	1.394445
SEC61B	W4	HPI4	well_4	F003	127	X/Y distribution	9.117723	8.890973	1.687106
SEC61B	W4	HPI4	well_4	F004	124	X/Y distribution	9.336911	9.408192	1.243242
SEC61B	W4	HPI4	well_4	F005	91	X/Y distribution	8.441521	8.544251	1.933632
SEC61B	W4	HPI4	well_4	F006	133	X/Y distribution	7.574254	7.674091	1.537121
SEC61B	W4	HPI4	well_5	F001	163	X/Y distribution	8.008895	7.894696	1.46566
SEC61B	W4	HPI4	well_5	F002	68	X/Y distribution	9.168564	9.305052	1.142219
SEC61B	W4	HPI4	well_5	F003	124	X/Y distribution	8.248824	8.177365	1.577517
SEC61B	W4	HPI4	well_5	F004	160	X/Y distribution	7.730154	7.554474	1.355251
SEC61B	W4	HPI4	well_5	F005	201	X/Y distribution	8.029276	8.022772	1.225998
SEC61B	W4	HPI4	well_5	F006	141	X/Y distribution	8.992814	9.028738	1.475314
SEC61B	W4	PGE2	well_1	F001	32	X/Y distribution	5.550768	5.689929	0.91016

SEC61B	W4	PGE2	well_1	F002	10	X/Y distribution	5.6762	5.470633	1.336054
SEC61B	W4	PGE2	well_1	F003	141	X/Y distribution	5.385936	5.431205	0.627819
SEC61B	W4	PGE2	well_1	F004	195	X/Y distribution	5.417088	5.404199	0.65917
SEC61B	W4	PGE2	well_1	F005	125	X/Y distribution	5.60285	5.555596	0.700432
SEC61B	W4	PGE2	well_1	F006	38	X/Y distribution	5.490953	5.398524	0.705993
SEC61B	W4	PGE2	well_2	F001	99	X/Y distribution	5.590415	5.583676	0.743089
SEC61B	W4	PGE2	well_2	F002	170	X/Y distribution	5.101945	5.073443	0.63512
SEC61B	W4	PGE2	well_2	F003	203	X/Y distribution	5.602531	5.542521	0.843553
SEC61B	W4	PGE2	well_2	F004	224	X/Y distribution	5.094908	5.10379	0.737729
SEC61B	W4	PGE2	well_2	F005	169	X/Y distribution	5.28769	5.26292	0.693694
SEC61B	W4	PGE2	well_2	F006	223	X/Y distribution	5.426586	5.426643	0.704321
SEC61B	W4	PGE2	well_3	F001	102	X/Y distribution	5.327324	5.287289	0.755941
SEC61B	W4	PGE2	well_3	F002	150	X/Y distribution	5.634593	5.654532	0.734934
SEC61B	W4	PGE2	well_3	F003	203	X/Y distribution	5.310708	5.228005	0.748615
SEC61B	W4	PGE2	well_3	F004	264	X/Y distribution	5.477437	5.47839	0.617485
SEC61B	W4	PGE2	well_3	F005	191	X/Y distribution	5.547487	5.552718	0.606201
SEC61B	W4	PGE2	well_3	F006	211	X/Y distribution	5.544412	5.449655	0.71008
SEC61B	W4	PGE2	well_4	F001	310	X/Y distribution	5.401589	5.359203	0.732754
SEC61B	W4	PGE2	well_4	F002	230	X/Y distribution	5.76202	5.740176	0.723594
SEC61B	W4	PGE2	well_4	F003	300	X/Y distribution	5.688115	5.680372	0.66201
SEC61B	W4	PGE2	well_4	F004	348	X/Y distribution	5.195205	5.186577	0.641416
SEC61B	W4	PGE2	well_4	F005	322	X/Y distribution	5.618879	5.596525	0.684748
SEC61B	W4	PGE2	well_4	F006	201	X/Y distribution	5.514359	5.46851	0.702261
SEC61B	W4	PGE2	well_5	F001	197	X/Y distribution	5.523882	5.481834	0.667385
SEC61B	W4	PGE2	well_5	F002	148	X/Y distribution	5.527216	5.530957	0.72435
SEC61B	W4	PGE2	well_5	F003	408	X/Y distribution	5.331402	5.387431	0.708849
SEC61B	W4	PGE2	well_5	F004	325	X/Y distribution	5.422607	5.376712	0.680425
SEC61B	W4	PGE2	well_5	F005	342	X/Y distribution	5.396023	5.378106	0.669984
SEC61B	W4	PGE2	well_5	F006	216	X/Y distribution	5.795223	5.742542	0.632217
SEC61B	W1	HPI4	well_1	F001	110	Z distribution	-0.30808	-0.33121	0.232118
SEC61B	W1	HPI4	well_1	F002	114	Z distribution	-0.26417	-0.21825	0.286615
SEC61B	W1	HPI4	well_1	F003	137	Z distribution	-0.3553	-0.35998	0.272532
SEC61B	W1	HPI4	well_1	F004	168	Z distribution	-0.45814	-0.43076	0.351135
SEC61B	W1	HPI4	well_1	F005	206	Z distribution	-0.25878	-0.25174	0.286072
SEC61B	W1	HPI4	well_1	F006	223	Z distribution	-0.24989	-0.18651	0.306612
SEC61B	W1	HPI4	well_2	F001	113	Z distribution	-0.30009	-0.28097	0.264228
SEC61B	W1	HPI4	well_2	F002	168	Z distribution	-0.26634	-0.23258	0.288933
SEC61B	W1	HPI4	well_2	F003	166	Z distribution	-0.22355	-0.21478	0.245259
SEC61B	W1	HPI4	well_2	F004	147	Z distribution	-0.2813	-0.28397	0.281935
SEC61B	W1	HPI4	well_2	F005	174	Z distribution	-0.26847	-0.26042	0.251035
SEC61B	W1	HPI4	well_2	F006	124	Z distribution	-0.23847	-0.12772	0.321371
SEC61B	W1	HPI4	well_3	F001	48	Z distribution	-0.16853	-0.12564	0.209352
SEC61B	W1	HPI4	well_3	F002	169	Z distribution	-0.24753	-0.21434	0.269879
SEC61B	W1	HPI4	well_3	F003	171	Z distribution	-0.24971	-0.19955	0.285134
SEC61B	W1	HPI4	well_3	F004	259	Z distribution	-0.19989	-0.13152	0.311314
SEC61B	W1	HPI4	well_3	F005	224	Z distribution	-0.20164	-0.18027	0.308582
SEC61B	W1	HPI4	well_3	F006	272	Z distribution	-0.16673	-0.1252	0.306878
SEC61B	W1	HPI4	well_4	F001	91	Z distribution	-0.42381	-0.35973	0.33997
SEC61B	W1	HPI4	well_4	F002	139	Z distribution	-0.42329	-0.41334	0.310401

SEC61B	W1	HPI4	well_4	F003	252	Z distribution	-0.22746	-0.18303	0.313024
SEC61B	W1	HPI4	well_4	F004	198	Z distribution	-0.23635	-0.21896	0.302675
SEC61B	W1	HPI4	well_4	F005	157	Z distribution	-0.2583	-0.24384	0.295182
SEC61B	W1	HPI4	well_4	F006	224	Z distribution	-0.20871	-0.17619	0.296817
SEC61B	W1	HPI4	well_5	F001	147	Z distribution	-0.15072	-0.11655	0.252499
SEC61B	W1	HPI4	well_5	F002	127	Z distribution	-0.20632	-0.15468	0.285093
SEC61B	W1	HPI4	well_5	F003	308	Z distribution	-0.15366	-0.1386	0.326681
SEC61B	W1	HPI4	well_5	F004	133	Z distribution	-0.24256	-0.18354	0.30938
SEC61B	W1	HPI4	well_5	F005	117	Z distribution	-0.20445	-0.18927	0.265485
SEC61B	W1	HPI4	well_5	F006	101	Z distribution	-0.27358	-0.29324	0.28066
SEC61B	W1	PGE2	well_1	F001	146	Z distribution	-0.33133	-0.27455	0.292616
SEC61B	W1	PGE2	well_1	F002	158	Z distribution	-0.23114	-0.1924	0.30721
SEC61B	W1	PGE2	well_1	F003	121	Z distribution	-0.21815	-0.14487	0.292384
SEC61B	W1	PGE2	well_1	F004	284	Z distribution	-0.2149	-0.18503	0.291568
SEC61B	W1	PGE2	well_1	F005	229	Z distribution	-0.32001	-0.29162	0.299843
SEC61B	W1	PGE2	well_1	F006	170	Z distribution	-0.29518	-0.28408	0.299841
SEC61B	W1	PGE2	well_2	F001	108	Z distribution	-0.30391	-0.31594	0.304926
SEC61B	W1	PGE2	well_2	F002	167	Z distribution	-0.43917	-0.43659	0.334864
SEC61B	W1	PGE2	well_2	F003	190	Z distribution	-0.14556	-0.12695	0.264548
SEC61B	W1	PGE2	well_2	F004	226	Z distribution	-0.26537	-0.2154	0.331696
SEC61B	W1	PGE2	well_2	F005	211	Z distribution	-0.28684	-0.27301	0.313521
SEC61B	W1	PGE2	well_2	F006	124	Z distribution	-0.30627	-0.30876	0.264551
SEC61B	W1	PGE2	well_3	F001	246	Z distribution	-0.2879	-0.21885	0.333561
SEC61B	W1	PGE2	well_3	F002	163	Z distribution	-0.21707	-0.18763	0.269725
SEC61B	W1	PGE2	well_3	F003	174	Z distribution	-0.3021	-0.30609	0.294971
SEC61B	W1	PGE2	well_3	F004	200	Z distribution	-0.30569	-0.33168	0.344971
SEC61B	W1	PGE2	well_3	F005	163	Z distribution	-0.24604	-0.2292	0.258255
SEC61B	W1	PGE2	well_3	F006	199	Z distribution	-0.23064	-0.21858	0.277765
SEC61B	W1	PGE2	well_4	F001	172	Z distribution	-0.30034	-0.27135	0.28408
SEC61B	W1	PGE2	well_4	F002	207	Z distribution	-0.27569	-0.25589	0.291276
SEC61B	W1	PGE2	well_4	F003	116	Z distribution	-0.3043	-0.23324	0.29004
SEC61B	W1	PGE2	well_4	F004	205	Z distribution	-0.19558	-0.16182	0.24378
SEC61B	W1	PGE2	well_4	F005	97	Z distribution	-0.37641	-0.38721	0.281919
SEC61B	W1	PGE2	well_4	F006	158	Z distribution	-0.21625	-0.20877	0.327631
SEC61B	W1	PGE2	well_5	F001	156	Z distribution	-0.24627	-0.23239	0.293083
SEC61B	W1	PGE2	well_5	F002	181	Z distribution	-0.22976	-0.21111	0.341462
SEC61B	W1	PGE2	well_5	F003	127	Z distribution	-0.27353	-0.2588	0.252662
SEC61B	W1	PGE2	well_5	F004	132	Z distribution	-0.36345	-0.37199	0.258785
SEC61B	W1	PGE2	well_5	F005	219	Z distribution	-0.2466	-0.22701	0.304554
SEC61B	W1	PGE2	well_5	F006	104	Z distribution	-0.3511	-0.34733	0.320172
SEC61B	W2	HPI4	well_1	F001	166	Z distribution	-0.38612	-0.32194	0.371996
SEC61B	W2	HPI4	well_1	F002	182	Z distribution	-0.39195	-0.31532	0.429769
SEC61B	W2	HPI4	well_1	F003	187	Z distribution	-0.54001	-0.54992	0.476399
SEC61B	W2	HPI4	well_1	F004	209	Z distribution	-0.53686	-0.50426	0.473305
SEC61B	W2	HPI4	well_1	F005	190	Z distribution	-0.45257	-0.37869	0.395329
SEC61B	W2	HPI4	well_1	F006	173	Z distribution	-0.54474	-0.52174	0.376953
SEC61B	W2	HPI4	well_2	F001	143	Z distribution	-0.39062	-0.38543	0.367522
SEC61B	W2	HPI4	well_2	F002	243	Z distribution	-0.56215	-0.54603	0.441907
SEC61B	W2	HPI4	well_2	F003	296	Z distribution	-0.19535	-0.14042	0.317596

SEC61B	W2	HPI4	well_2	F004	251	Z distribution	-0.51309	-0.50097	0.473957
SEC61B	W2	HPI4	well_2	F005	207	Z distribution	-0.61893	-0.71461	0.51234
SEC61B	W2	HPI4	well_2	F006	171	Z distribution	-0.63328	-0.7074	0.369803
SEC61B	W2	HPI4	well_3	F001	167	Z distribution	-0.35448	-0.29261	0.419779
SEC61B	W2	HPI4	well_3	F002	158	Z distribution	-0.28813	-0.21282	0.328299
SEC61B	W2	HPI4	well_3	F003	189	Z distribution	-0.25254	-0.18622	0.323478
SEC61B	W2	HPI4	well_3	F004	370	Z distribution	-0.26459	-0.18904	0.348786
SEC61B	W2	HPI4	well_3	F005	176	Z distribution	-0.39989	-0.36495	0.394091
SEC61B	W2	HPI4	well_3	F006	189	Z distribution	-0.24753	-0.23394	0.344523
SEC61B	W2	HPI4	well_4	F001	114	Z distribution	-0.22581	-0.20536	0.294668
SEC61B	W2	HPI4	well_4	F002	235	Z distribution	-0.52369	-0.47197	0.499408
SEC61B	W2	HPI4	well_4	F003	293	Z distribution	-0.27665	-0.25411	0.375762
SEC61B	W2	HPI4	well_4	F004	204	Z distribution	-0.38876	-0.3362	0.393139
SEC61B	W2	HPI4	well_4	F005	169	Z distribution	-0.45113	-0.40799	0.3801
SEC61B	W2	HPI4	well_4	F006	175	Z distribution	-0.52503	-0.50871	0.407083
SEC61B	W2	HPI4	well_5	F001	136	Z distribution	-0.23525	-0.22747	0.307507
SEC61B	W2	HPI4	well_5	F002	196	Z distribution	-0.14515	-0.09672	0.359007
SEC61B	W2	HPI4	well_5	F003	419	Z distribution	-0.37557	-0.29897	0.407091
SEC61B	W2	HPI4	well_5	F004	348	Z distribution	-0.20078	-0.14672	0.391857
SEC61B	W2	HPI4	well_5	F005	158	Z distribution	-0.43689	-0.38155	0.395637
SEC61B	W2	HPI4	well_5	F006	230	Z distribution	-0.3519	-0.28687	0.371819
SEC61B	W2	PGE2	well_1	F001	288	Z distribution	-0.07707	-0.11082	0.410964
SEC61B	W2	PGE2	well_1	F002	269	Z distribution	-0.16771	-0.19334	0.487227
SEC61B	W2	PGE2	well_1	F003	381	Z distribution	0.001189	-0.02144	0.416042
SEC61B	W2	PGE2	well_1	F004	172	Z distribution	-0.22389	-0.26722	0.414032
SEC61B	W2	PGE2	well_1	F005	293	Z distribution	-0.36754	-0.31667	0.485955
SEC61B	W2	PGE2	well_1	F006	217	Z distribution	0.021807	0.027852	0.529437
SEC61B	W2	PGE2	well_2	F001	244	Z distribution	-0.32344	-0.28369	0.49028
SEC61B	W2	PGE2	well_2	F002	351	Z distribution	-0.17441	-0.17901	0.459415
SEC61B	W2	PGE2	well_2	F003	391	Z distribution	-0.17102	-0.15151	0.441086
SEC61B	W2	PGE2	well_2	F004	366	Z distribution	-0.37802	-0.32681	0.395109
SEC61B	W2	PGE2	well_2	F005	292	Z distribution	-0.10007	-0.07399	0.438106
SEC61B	W2	PGE2	well_2	F006	280	Z distribution	-0.29031	-0.28301	0.45421
SEC61B	W2	PGE2	well_3	F001	283	Z distribution	-0.14555	-0.15099	0.426189
SEC61B	W2	PGE2	well_3	F002	443	Z distribution	-0.26016	-0.25701	0.489657
SEC61B	W2	PGE2	well_3	F003	351	Z distribution	-0.24045	-0.24225	0.55139
SEC61B	W2	PGE2	well_3	F004	526	Z distribution	-0.06755	-0.06047	0.417295
SEC61B	W2	PGE2	well_3	F005	174	Z distribution	-0.3273	-0.25173	0.468049
SEC61B	W2	PGE2	well_3	F006	293	Z distribution	-0.20687	-0.26252	0.476929
SEC61B	W2	PGE2	well_4	F001	260	Z distribution	-0.19605	-0.16011	0.391661
SEC61B	W2	PGE2	well_4	F002	461	Z distribution	-0.03584	-0.06349	0.460625
SEC61B	W2	PGE2	well_4	F003	129	Z distribution	-0.00869	-0.00669	0.349082
SEC61B	W2	PGE2	well_4	F004	393	Z distribution	-0.24457	-0.20953	0.412402
SEC61B	W2	PGE2	well_4	F005	354	Z distribution	-0.15883	-0.1671	0.49291
SEC61B	W2	PGE2	well_4	F006	406	Z distribution	-0.13571	-0.13295	0.516648
SEC61B	W2	PGE2	well_5	F001	383	Z distribution	-0.13355	-0.1478	0.467354
SEC61B	W2	PGE2	well_5	F002	369	Z distribution	-0.25047	-0.27147	0.444369
SEC61B	W2	PGE2	well_5	F003	402	Z distribution	-0.20644	-0.20549	0.408556
SEC61B	W2	PGE2	well_5	F004	400	Z distribution	-0.0316	-0.03887	0.407835

SEC61B	W2	PGE2	well_5	F005	305	Z distribution	-0.16423	-0.1377	0.38499
SEC61B	W2	PGE2	well_5	F006	375	Z distribution	0.054045	0.075301	0.444449
SEC61B	W3	HPI4	well_1	F001	112	Z distribution	-0.85654	-0.76442	0.716182
SEC61B	W3	HPI4	well_1	F002	104	Z distribution	-1.11996	-1.15527	0.595671
SEC61B	W3	HPI4	well_1	F003	123	Z distribution	-0.57895	-0.53146	0.516281
SEC61B	W3	HPI4	well_1	F004	127	Z distribution	-0.74613	-0.68497	0.60388
SEC61B	W3	HPI4	well_1	F005	135	Z distribution	-0.53648	-0.37613	0.501427
SEC61B	W3	HPI4	well_1	F006	133	Z distribution	-0.79723	-0.8932	0.577104
SEC61B	W3	HPI4	well_2	F001	115	Z distribution	-0.74089	-0.7239	0.637095
SEC61B	W3	HPI4	well_2	F002	147	Z distribution	-0.89754	-0.89015	0.534486
SEC61B	W3	HPI4	well_2	F003	153	Z distribution	-0.55381	-0.4565	0.588211
SEC61B	W3	HPI4	well_2	F004	197	Z distribution	-0.76235	-0.78243	0.586169
SEC61B	W3	HPI4	well_2	F005	121	Z distribution	-0.79894	-0.80799	0.572446
SEC61B	W3	HPI4	well_2	F006	122	Z distribution	-0.9416	-1.06232	0.61266
SEC61B	W3	HPI4	well_3	F001	59	Z distribution	-0.23013	-0.18971	0.290974
SEC61B	W3	HPI4	well_3	F002	92	Z distribution	-0.29159	-0.2047	0.416225
SEC61B	W3	HPI4	well_3	F003	105	Z distribution	-0.49559	-0.43514	0.453158
SEC61B	W3	HPI4	well_3	F004	160	Z distribution	-0.53571	-0.45044	0.508243
SEC61B	W3	HPI4	well_3	F005	184	Z distribution	-0.61297	-0.65332	0.493661
SEC61B	W3	HPI4	well_3	F006	95	Z distribution	-0.80328	-0.78455	0.515432
SEC61B	W3	HPI4	well_4	F001	25	Z distribution	-0.70677	-0.53713	0.444174
SEC61B	W3	HPI4	well_4	F002	155	Z distribution	-0.46504	-0.46571	0.429019
SEC61B	W3	HPI4	well_4	F003	118	Z distribution	-0.28716	-0.18826	0.353365
SEC61B	W3	HPI4	well_4	F004	152	Z distribution	-0.28767	-0.21366	0.414056
SEC61B	W3	HPI4	well_4	F005	138	Z distribution	-0.48954	-0.4202	0.458181
SEC61B	W3	HPI4	well_4	F006	91	Z distribution	-0.35105	-0.30225	0.335802
SEC61B	W3	HPI4	well_5	F001	115	Z distribution	-0.40301	-0.34526	0.371306
SEC61B	W3	HPI4	well_5	F002	43	Z distribution	-0.09776	-0.02112	0.233595
SEC61B	W3	HPI4	well_5	F003	173	Z distribution	-0.29458	-0.19978	0.398947
SEC61B	W3	HPI4	well_5	F004	133	Z distribution	-0.64385	-0.59845	0.480601
SEC61B	W3	HPI4	well_5	F005	122	Z distribution	-0.425	-0.37815	0.370813
SEC61B	W3	HPI4	well_5	F006	132	Z distribution	-0.61199	-0.51454	0.48961
SEC61B	W3	PGE2	well_1	F001	227	Z distribution	-0.66655	-0.69486	0.906024
SEC61B	W3	PGE2	well_1	F002	270	Z distribution	-0.08808	0.051446	0.486602
SEC61B	W3	PGE2	well_1	F003	218	Z distribution	-0.42187	-0.40206	0.635064
SEC61B	W3	PGE2	well_1	F004	376	Z distribution	-0.77615	-0.77187	0.648599
SEC61B	W3	PGE2	well_1	F005	315	Z distribution	-1.046	-0.99617	0.610665
SEC61B	W3	PGE2	well_1	F006	126	Z distribution	-0.3062	-0.27749	0.523291
SEC61B	W3	PGE2	well_2	F001	362	Z distribution	-0.88265	-0.83595	0.529727
SEC61B	W3	PGE2	well_2	F002	297	Z distribution	-0.74897	-0.74803	0.627929
SEC61B	W3	PGE2	well_2	F003	428	Z distribution	-0.8105	-0.79047	0.598613
SEC61B	W3	PGE2	well_2	F004	399	Z distribution	-0.5753	-0.51105	0.596613
SEC61B	W3	PGE2	well_2	F005	270	Z distribution	-0.52278	-0.51477	0.523899
SEC61B	W3	PGE2	well_2	F006	331	Z distribution	-0.54468	-0.52509	0.604274
SEC61B	W3	PGE2	well_3	F001	490	Z distribution	-0.50445	-0.5059	0.569303
SEC61B	W3	PGE2	well_3	F002	174	Z distribution	-0.37984	-0.39619	0.528106
SEC61B	W3	PGE2	well_3	F003	388	Z distribution	-0.51493	-0.53091	0.576352
SEC61B	W3	PGE2	well_3	F004	469	Z distribution	-0.41727	-0.41239	0.533473
SEC61B	W3	PGE2	well_3	F005	454	Z distribution	-0.42757	-0.40904	0.56008

SEC61B	W3	PGE2	well_3	F006	435	Z distribution	-0.46209	-0.45435	0.537578
SEC61B	W3	PGE2	well_4	F001	315	Z distribution	-0.46801	-0.43915	0.499072
SEC61B	W3	PGE2	well_4	F002	397	Z distribution	-0.63713	-0.67342	0.592472
SEC61B	W3	PGE2	well_4	F003	396	Z distribution	-0.75011	-0.74907	0.49671
SEC61B	W3	PGE2	well_4	F004	386	Z distribution	-0.74465	-0.74855	0.558667
SEC61B	W3	PGE2	well_4	F005	344	Z distribution	-0.41594	-0.40252	0.508763
SEC61B	W3	PGE2	well_4	F006	254	Z distribution	-0.64696	-0.68591	0.468325
SEC61B	W3	PGE2	well_5	F001	400	Z distribution	-0.65124	-0.63235	0.594148
SEC61B	W3	PGE2	well_5	F002	178	Z distribution	-0.82022	-0.87454	0.537655
SEC61B	W3	PGE2	well_5	F003	171	Z distribution	-0.66913	-0.61875	0.48768
SEC61B	W3	PGE2	well_5	F004	344	Z distribution	-0.75889	-0.77217	0.645586
SEC61B	W3	PGE2	well_5	F005	413	Z distribution	-0.39088	-0.33459	0.558449
SEC61B	W3	PGE2	well_5	F006	367	Z distribution	-0.32312	-0.29528	0.55351
SEC61B	W4	HPI4	well_1	F001	92	Z distribution	-0.56639	-0.57291	0.447714
SEC61B	W4	HPI4	well_1	F002	91	Z distribution	-0.53263	-0.457	0.467866
SEC61B	W4	HPI4	well_1	F003	165	Z distribution	-0.59129	-0.5792	0.48623
SEC61B	W4	HPI4	well_1	F004	157	Z distribution	-0.4883	-0.46901	0.444246
SEC61B	W4	HPI4	well_1	F005	155	Z distribution	-0.56813	-0.571	0.443514
SEC61B	W4	HPI4	well_1	F006	102	Z distribution	-0.62728	-0.64053	0.450378
SEC61B	W4	HPI4	well_2	F001	97	Z distribution	-0.39046	-0.33047	0.388816
SEC61B	W4	HPI4	well_2	F002	110	Z distribution	-0.53304	-0.48682	0.450566
SEC61B	W4	HPI4	well_2	F003	137	Z distribution	-0.59312	-0.61082	0.407814
SEC61B	W4	HPI4	well_2	F004	163	Z distribution	-0.79625	-0.91825	0.48526
SEC61B	W4	HPI4	well_2	F005	87	Z distribution	-0.3869	-0.34317	0.406076
SEC61B	W4	HPI4	well_2	F006	183	Z distribution	-0.27452	-0.1825	0.315485
SEC61B	W4	HPI4	well_3	F001	150	Z distribution	-0.40386	-0.38985	0.414111
SEC61B	W4	HPI4	well_3	F002	142	Z distribution	-0.57365	-0.6633	0.472545
SEC61B	W4	HPI4	well_3	F003	137	Z distribution	-0.4816	-0.5108	0.39961
SEC61B	W4	HPI4	well_3	F004	183	Z distribution	-0.65046	-0.73236	0.438866
SEC61B	W4	HPI4	well_3	F005	109	Z distribution	-0.32676	-0.26761	0.396838
SEC61B	W4	HPI4	well_3	F006	39	Z distribution	-0.42628	-0.29088	0.417174
SEC61B	W4	HPI4	well_4	F001	108	Z distribution	-0.40182	-0.3115	0.412303
SEC61B	W4	HPI4	well_4	F002	132	Z distribution	-0.74693	-0.81703	0.497932
SEC61B	W4	HPI4	well_4	F003	127	Z distribution	-0.59728	-0.66351	0.492584
SEC61B	W4	HPI4	well_4	F004	124	Z distribution	-0.65772	-0.65717	0.430571
SEC61B	W4	HPI4	well_4	F005	91	Z distribution	-0.2819	-0.22199	0.316689
SEC61B	W4	HPI4	well_4	F006	133	Z distribution	-0.4067	-0.37027	0.4076
SEC61B	W4	HPI4	well_5	F001	163	Z distribution	-0.52019	-0.53272	0.373525
SEC61B	W4	HPI4	well_5	F002	68	Z distribution	-0.55796	-0.57966	0.476237
SEC61B	W4	HPI4	well_5	F003	124	Z distribution	-0.2717	-0.1504	0.38313
SEC61B	W4	HPI4	well_5	F004	160	Z distribution	-0.76011	-0.77534	0.593946
SEC61B	W4	HPI4	well_5	F005	201	Z distribution	-0.6049	-0.64498	0.519045
SEC61B	W4	HPI4	well_5	F006	141	Z distribution	-0.50186	-0.51166	0.419551
SEC61B	W4	PGE2	well_1	F001	32	Z distribution	-1.035	-1.11271	0.497799
SEC61B	W4	PGE2	well_1	F002	10	Z distribution	-0.69443	-0.69042	0.318671
SEC61B	W4	PGE2	well_1	F003	141	Z distribution	-0.58194	-0.55625	0.464535
SEC61B	W4	PGE2	well_1	F004	195	Z distribution	-0.46265	-0.3916	0.450279
SEC61B	W4	PGE2	well_1	F005	125	Z distribution	-0.47956	-0.48927	0.458673
SEC61B	W4	PGE2	well_1	F006	38	Z distribution	-0.60248	-0.60355	0.497446

SEC61B	W4	PGE2	well_2	F001	99	Z distribution	-0.82622	-0.83638	0.514454
SEC61B	W4	PGE2	well_2	F002	170	Z distribution	-0.43663	-0.3962	0.437898
SEC61B	W4	PGE2	well_2	F003	203	Z distribution	-0.31776	-0.29549	0.38351
SEC61B	W4	PGE2	well_2	F004	224	Z distribution	-0.61655	-0.57282	0.525658
SEC61B	W4	PGE2	well_2	F005	169	Z distribution	-0.44071	-0.4052	0.437776
SEC61B	W4	PGE2	well_2	F006	223	Z distribution	-0.46991	-0.40894	0.468077
SEC61B	W4	PGE2	well_3	F001	102	Z distribution	-0.76314	-0.65668	0.557607
SEC61B	W4	PGE2	well_3	F002	150	Z distribution	-0.45791	-0.43494	0.425723
SEC61B	W4	PGE2	well_3	F003	203	Z distribution	-0.55823	-0.51352	0.429197
SEC61B	W4	PGE2	well_3	F004	264	Z distribution	-0.47909	-0.49312	0.530335
SEC61B	W4	PGE2	well_3	F005	191	Z distribution	-0.50136	-0.49014	0.436102
SEC61B	W4	PGE2	well_3	F006	211	Z distribution	-0.61046	-0.53768	0.550993
SEC61B	W4	PGE2	well_4	F001	310	Z distribution	-0.39507	-0.41563	0.5172
SEC61B	W4	PGE2	well_4	F002	230	Z distribution	-0.63684	-0.6456	0.447876
SEC61B	W4	PGE2	well_4	F003	300	Z distribution	-0.24385	-0.20257	0.503813
SEC61B	W4	PGE2	well_4	F004	348	Z distribution	-0.30161	-0.29611	0.459781
SEC61B	W4	PGE2	well_4	F005	322	Z distribution	-0.52728	-0.49881	0.537175
SEC61B	W4	PGE2	well_4	F006	201	Z distribution	-0.67996	-0.66821	0.549871
SEC61B	W4	PGE2	well_5	F001	197	Z distribution	-0.69237	-0.66546	0.480036
SEC61B	W4	PGE2	well_5	F002	148	Z distribution	-0.59915	-0.57001	0.507142
SEC61B	W4	PGE2	well_5	F003	408	Z distribution	-0.38072	-0.38526	0.469945
SEC61B	W4	PGE2	well_5	F004	325	Z distribution	-0.31873	-0.32956	0.469292
SEC61B	W4	PGE2	well_5	F005	342	Z distribution	-0.47238	-0.51797	0.472329
SEC61B	W4	PGE2	well_5	F006	216	Z distribution	-0.48592	-0.49747	0.439778
SEC61B	W1	HPI4	well_1	F001	110	Total volume	221.5519	219.4066	87.00878
SEC61B	W1	HPI4	well_1	F002	114	Total volume	170.9714	151.079	77.61
SEC61B	W1	HPI4	well_1	F003	137	Total volume	92.04756	78.65542	46.38153
SEC61B	W1	HPI4	well_1	F004	168	Total volume	202.3082	200.2181	96.3049
SEC61B	W1	HPI4	well_1	F005	206	Total volume	129.4292	114.1689	61.70993
SEC61B	W1	HPI4	well_1	F006	223	Total volume	187.2475	177.1449	83.92634
SEC61B	W1	HPI4	well_2	F001	113	Total volume	241.8666	222.5871	91.15164
SEC61B	W1	HPI4	well_2	F002	168	Total volume	146.536	143.0045	66.77779
SEC61B	W1	HPI4	well_2	F003	166	Total volume	169.6602	157.4399	66.45193
SEC61B	W1	HPI4	well_2	F004	147	Total volume	207.9221	199.1383	72.97546
SEC61B	W1	HPI4	well_2	F005	174	Total volume	192.6976	184.1044	82.95764
SEC61B	W1	HPI4	well_2	F006	124	Total volume	185.205	172.6382	82.73969
SEC61B	W1	HPI4	well_3	F001	48	Total volume	180.9485	157.5456	84.22443
SEC61B	W1	HPI4	well_3	F002	169	Total volume	147.582	141.5375	74.60202
SEC61B	W1	HPI4	well_3	F003	171	Total volume	131.8668	120.3421	69.20837
SEC61B	W1	HPI4	well_3	F004	259	Total volume	132.168	125.905	63.54731
SEC61B	W1	HPI4	well_3	F005	224	Total volume	149.5481	147.9102	62.25598
SEC61B	W1	HPI4	well_3	F006	272	Total volume	142.3764	139.4954	69.07319
SEC61B	W1	HPI4	well_4	F001	91	Total volume	196.1075	174.8211	104.2496
SEC61B	W1	HPI4	well_4	F002	139	Total volume	207.5902	210.9683	99.35193
SEC61B	W1	HPI4	well_4	F003	252	Total volume	168.8818	171.5819	65.99616
SEC61B	W1	HPI4	well_4	F004	198	Total volume	137.6725	128.1349	59.86444
SEC61B	W1	HPI4	well_4	F005	157	Total volume	168.7605	149.4007	84.24599
SEC61B	W1	HPI4	well_4	F006	224	Total volume	160.5758	150.5039	70.09423
SEC61B	W1	HPI4	well_5	F001	147	Total volume	134.3199	122.7597	68.65654

SEC61B	W1	HPI4	well_5	F002	127	Total volume	121.2187	117.7367	52.48345
SEC61B	W1	HPI4	well_5	F003	308	Total volume	129.4169	122.9592	70.36781
SEC61B	W1	HPI4	well_5	F004	133	Total volume	128.2112	117.7132	60.41802
SEC61B	W1	HPI4	well_5	F005	117	Total volume	143.5029	127.4307	67.8928
SEC61B	W1	HPI4	well_5	F006	101	Total volume	186.4283	175.5488	91.17346
SEC61B	W2	HPI4	well_1	F001	146	Total volume	82.49439	73.47979	45.2619
SEC61B	W2	HPI4	well_1	F002	158	Total volume	74.93695	59.77201	50.56482
SEC61B	W2	HPI4	well_1	F003	121	Total volume	105.3349	98.11389	58.14907
SEC61B	W2	HPI4	well_1	F004	284	Total volume	111.4825	102.0338	63.75013
SEC61B	W2	HPI4	well_1	F005	229	Total volume	89.38057	65.54618	66.80019
SEC61B	W2	HPI4	well_1	F006	170	Total volume	94.96119	85.90833	53.48152
SEC61B	W2	HPI4	well_2	F001	108	Total volume	148.1334	137.1247	72.71117
SEC61B	W2	HPI4	well_2	F002	167	Total volume	79.44456	65.39361	50.51454
SEC61B	W2	HPI4	well_2	F003	190	Total volume	87.48459	78.11556	52.6558
SEC61B	W2	HPI4	well_2	F004	226	Total volume	101.8539	95.60236	52.40615
SEC61B	W2	HPI4	well_2	F005	211	Total volume	106.6414	91.14264	60.18217
SEC61B	W2	HPI4	well_2	F006	124	Total volume	171.5597	167.1926	81.47664
SEC61B	W2	HPI4	well_3	F001	246	Total volume	130.6458	121.4453	66.77106
SEC61B	W2	HPI4	well_3	F002	163	Total volume	127.3704	117.5137	66.29786
SEC61B	W2	HPI4	well_3	F003	174	Total volume	100.7546	92.29278	56.16733
SEC61B	W2	HPI4	well_3	F004	200	Total volume	70.59257	60.72264	45.17488
SEC61B	W2	HPI4	well_3	F005	163	Total volume	119.4825	104.5335	70.8111
SEC61B	W2	HPI4	well_3	F006	199	Total volume	110.6808	104.0993	64.73631
SEC61B	W2	HPI4	well_4	F001	172	Total volume	130.7893	126.3158	65.22728
SEC61B	W2	HPI4	well_4	F002	207	Total volume	70.8513	62.38917	44.54582
SEC61B	W2	HPI4	well_4	F003	116	Total volume	77.91111	71.36729	43.39624
SEC61B	W2	HPI4	well_4	F004	205	Total volume	68.59654	56.3216	47.19767
SEC61B	W2	HPI4	well_4	F005	97	Total volume	96.23922	90.415	48.62359
SEC61B	W2	HPI4	well_4	F006	158	Total volume	117.2107	97.92611	62.36032
SEC61B	W2	HPI4	well_5	F001	156	Total volume	115.8541	109.8852	54.64327
SEC61B	W2	HPI4	well_5	F002	181	Total volume	108.4569	91.09569	70.03447
SEC61B	W2	HPI4	well_5	F003	127	Total volume	50.92239	40.1375	38.54
SEC61B	W2	HPI4	well_5	F004	132	Total volume	93.44495	85.50931	55.28204
SEC61B	W2	HPI4	well_5	F005	219	Total volume	115.0022	112.9366	59.2201
SEC61B	W2	HPI4	well_5	F006	104	Total volume	118.9825	101.6582	74.74138
SEC61B	W3	HPI4	well_1	F001	166	Total volume	97.64374	80.46278	76.37007
SEC61B	W3	HPI4	well_1	F002	182	Total volume	108.5083	99.13493	58.77538
SEC61B	W3	HPI4	well_1	F003	187	Total volume	118.5229	115.9058	74.2253
SEC61B	W3	HPI4	well_1	F004	209	Total volume	99.18759	85.93181	58.09275
SEC61B	W3	HPI4	well_1	F005	190	Total volume	77.95532	61.33292	53.43751
SEC61B	W3	HPI4	well_1	F006	173	Total volume	60.51471	51.55674	42.10728
SEC61B	W3	HPI4	well_2	F001	143	Total volume	195.9409	192.4253	93.16702
SEC61B	W3	HPI4	well_2	F002	243	Total volume	52.48371	44.45639	36.74448
SEC61B	W3	HPI4	well_2	F003	296	Total volume	101.5275	82.50486	67.47383
SEC61B	W3	HPI4	well_2	F004	251	Total volume	90.43091	77.71653	61.18224
SEC61B	W3	HPI4	well_2	F005	207	Total volume	166.8188	165.1271	77.26609
SEC61B	W3	HPI4	well_2	F006	171	Total volume	171.2449	160.151	92.07377
SEC61B	W3	HPI4	well_3	F001	167	Total volume	87.03589	73.91403	55.51408
SEC61B	W3	HPI4	well_3	F002	158	Total volume	133.7387	124.7901	61.89423

SEC61B	W3	HPI4	well_3	F003	189	Total volume	168.8544	159.259	71.10535
SEC61B	W3	HPI4	well_3	F004	370	Total volume	87.41121	71.37903	58.16958
SEC61B	W3	HPI4	well_3	F005	176	Total volume	121.5387	112.5845	63.12733
SEC61B	W3	HPI4	well_3	F006	189	Total volume	193.7585	206.0861	98.75767
SEC61B	W3	HPI4	well_4	F001	114	Total volume	100.2443	84.80514	39.68113
SEC61B	W3	HPI4	well_4	F002	235	Total volume	121.3257	114.7792	63.27385
SEC61B	W3	HPI4	well_4	F003	293	Total volume	114.9541	99.82736	61.13932
SEC61B	W3	HPI4	well_4	F004	204	Total volume	87.11991	79.24222	51.59383
SEC61B	W3	HPI4	well_4	F005	169	Total volume	94.50787	84.32396	57.25024
SEC61B	W3	HPI4	well_4	F006	175	Total volume	122.3048	103.1135	66.82841
SEC61B	W3	HPI4	well_5	F001	136	Total volume	181.2999	177.0979	90.27486
SEC61B	W3	HPI4	well_5	F002	196	Total volume	166.9097	154.4942	66.98768
SEC61B	W3	HPI4	well_5	F003	419	Total volume	115.5477	109.2397	57.06893
SEC61B	W3	HPI4	well_5	F004	348	Total volume	98.76579	87.80958	57.72242
SEC61B	W3	HPI4	well_5	F005	158	Total volume	136.8081	118.8164	78.71698
SEC61B	W3	HPI4	well_5	F006	230	Total volume	110.299	105.7306	69.86215
SEC61B	W4	HPI4	well_1	F001	288	Total volume	74.73098	56.59153	50.46663
SEC61B	W4	HPI4	well_1	F002	269	Total volume	59.42037	54.08	34.6265
SEC61B	W4	HPI4	well_1	F003	381	Total volume	70.34705	64.17306	42.24996
SEC61B	W4	HPI4	well_1	F004	172	Total volume	86.2864	73.74972	58.9574
SEC61B	W4	HPI4	well_1	F005	293	Total volume	48.38993	44.55028	33.04401
SEC61B	W4	HPI4	well_1	F006	217	Total volume	66.6259	60.47618	44.16763
SEC61B	W4	HPI4	well_2	F001	244	Total volume	123.0859	112.6197	68.30356
SEC61B	W4	HPI4	well_2	F002	351	Total volume	164.3281	169.1643	87.22422
SEC61B	W4	HPI4	well_2	F003	391	Total volume	112.1478	103.4656	63.69385
SEC61B	W4	HPI4	well_2	F004	366	Total volume	125.173	119.2858	61.08296
SEC61B	W4	HPI4	well_2	F005	292	Total volume	89.68828	71.49639	57.94584
SEC61B	W4	HPI4	well_2	F006	280	Total volume	74.1391	68.30417	46.05195
SEC61B	W4	HPI4	well_3	F001	283	Total volume	95.89804	79.77035	60.88589
SEC61B	W4	HPI4	well_3	F002	443	Total volume	96.42165	88.38465	56.80514
SEC61B	W4	HPI4	well_3	F003	351	Total volume	118.0806	107.4089	67.89545
SEC61B	W4	HPI4	well_3	F004	526	Total volume	115.9983	101.4	69.03433
SEC61B	W4	HPI4	well_3	F005	174	Total volume	20.12083	14.31806	18.15269
SEC61B	W4	HPI4	well_3	F006	293	Total volume	111.4957	97.55056	59.42211
SEC61B	W4	HPI4	well_4	F001	260	Total volume	170.5766	145.9033	101.325
SEC61B	W4	HPI4	well_4	F002	461	Total volume	96.90517	85.81444	59.71656
SEC61B	W4	HPI4	well_4	F003	129	Total volume	128.7101	105.8245	85.67961
SEC61B	W4	HPI4	well_4	F004	393	Total volume	184.4641	179.2926	91.00531
SEC61B	W4	HPI4	well_4	F005	354	Total volume	99.42544	75.76833	70.97341
SEC61B	W4	HPI4	well_4	F006	406	Total volume	49.59306	39.08125	38.78754
SEC61B	W4	HPI4	well_5	F001	383	Total volume	93.42382	81.19042	56.9962
SEC61B	W4	HPI4	well_5	F002	369	Total volume	129.2471	125.8346	63.75854
SEC61B	W4	HPI4	well_5	F003	402	Total volume	111.0844	96.76424	61.49472
SEC61B	W4	HPI4	well_5	F004	400	Total volume	51.93008	41.42847	39.36912
SEC61B	W4	HPI4	well_5	F005	305	Total volume	84.92237	77.85736	48.08399
SEC61B	W4	HPI4	well_5	F006	375	Total volume	130.0385	124.2502	68.30456
SEC61B	W1	PGE2	well_1	F001	112	Total volume	170.0817	158.3906	79.68803
SEC61B	W1	PGE2	well_1	F002	104	Total volume	156.7457	138.3922	78.26755
SEC61B	W1	PGE2	well_1	F003	123	Total volume	171.6226	150.5743	99.06344

SEC61B	W1	PGE2	well_1	F004	127	Total volume	112.8672	101.8929	63.38441
SEC61B	W1	PGE2	well_1	F005	135	Total volume	172.4047	169.8685	79.7903
SEC61B	W1	PGE2	well_1	F006	133	Total volume	190.9268	175.1849	106.4441
SEC61B	W1	PGE2	well_2	F001	115	Total volume	225.4546	208.1282	126.7644
SEC61B	W1	PGE2	well_2	F002	147	Total volume	29.79491	27.55639	15.04059
SEC61B	W1	PGE2	well_2	F003	153	Total volume	128.8947	106.8925	74.41909
SEC61B	W1	PGE2	well_2	F004	197	Total volume	167.285	158.2732	76.60961
SEC61B	W1	PGE2	well_2	F005	121	Total volume	152.3355	148.5557	62.37605
SEC61B	W1	PGE2	well_2	F006	122	Total volume	255.3613	246.8339	96.76339
SEC61B	W1	PGE2	well_3	F001	59	Total volume	152.6017	138.404	73.95809
SEC61B	W1	PGE2	well_3	F002	92	Total volume	187.7679	178.8114	95.68913
SEC61B	W1	PGE2	well_3	F003	105	Total volume	201.6872	194.1622	76.06254
SEC61B	W1	PGE2	well_3	F004	160	Total volume	206.1934	215.9914	82.21684
SEC61B	W1	PGE2	well_3	F005	184	Total volume	219.6503	211.0857	98.3466
SEC61B	W1	PGE2	well_3	F006	95	Total volume	147.0658	132.4303	66.04046
SEC61B	W1	PGE2	well_4	F001	25	Total volume	191.555	187.4844	90.91685
SEC61B	W1	PGE2	well_4	F002	155	Total volume	173.1467	170.3614	65.40049
SEC61B	W1	PGE2	well_4	F003	118	Total volume	165.3081	129.3202	93.11977
SEC61B	W1	PGE2	well_4	F004	152	Total volume	114.9702	111.1644	57.00011
SEC61B	W1	PGE2	well_4	F005	138	Total volume	205.5759	180.8065	83.42372
SEC61B	W1	PGE2	well_4	F006	91	Total volume	154.7208	144.2485	79.05889
SEC61B	W1	PGE2	well_5	F001	115	Total volume	183.0436	180.6422	77.78919
SEC61B	W1	PGE2	well_5	F002	43	Total volume	138.9861	130.0361	69.22056
SEC61B	W1	PGE2	well_5	F003	173	Total volume	200.5049	195.1011	75.79384
SEC61B	W1	PGE2	well_5	F004	133	Total volume	255.3078	249.7327	108.4559
SEC61B	W1	PGE2	well_5	F005	122	Total volume	119.4072	111.6808	53.9643
SEC61B	W1	PGE2	well_5	F006	132	Total volume	191.9831	192.9886	61.71035
SEC61B	W2	PGE2	well_1	F001	227	Total volume	96.14231	90.05118	48.36694
SEC61B	W2	PGE2	well_1	F002	270	Total volume	84.26606	72.31792	52.50543
SEC61B	W2	PGE2	well_1	F003	218	Total volume	97.49606	87.86826	54.45474
SEC61B	W2	PGE2	well_1	F004	376	Total volume	62.77393	55.11278	37.56325
SEC61B	W2	PGE2	well_1	F005	315	Total volume	87.3664	86.09611	47.03025
SEC61B	W2	PGE2	well_1	F006	126	Total volume	72.8839	71.49639	39.02059
SEC61B	W2	PGE2	well_2	F001	362	Total volume	38.43214	29.31681	31.78998
SEC61B	W2	PGE2	well_2	F002	297	Total volume	105.7709	101.4939	53.08997
SEC61B	W2	PGE2	well_2	F003	428	Total volume	76.71678	74.54778	41.91459
SEC61B	W2	PGE2	well_2	F004	399	Total volume	66.88881	60.58181	40.42004
SEC61B	W2	PGE2	well_2	F005	270	Total volume	98.71431	86.68292	55.03145
SEC61B	W2	PGE2	well_2	F006	331	Total volume	88.37394	87.21104	40.56092
SEC61B	W2	PGE2	well_3	F001	490	Total volume	80.56682	76.56639	45.12882
SEC61B	W2	PGE2	well_3	F002	174	Total volume	87.30866	78.8784	50.06377
SEC61B	W2	PGE2	well_3	F003	388	Total volume	86.02301	81.44861	46.26023
SEC61B	W2	PGE2	well_3	F004	469	Total volume	81.80598	73.39764	46.66946
SEC61B	W2	PGE2	well_3	F005	454	Total volume	102.228	94.56958	47.81588
SEC61B	W2	PGE2	well_3	F006	435	Total volume	78.86095	67.76431	41.84377
SEC61B	W2	PGE2	well_4	F001	315	Total volume	92.89282	82.71611	48.65531
SEC61B	W2	PGE2	well_4	F002	397	Total volume	64.75172	60.20625	37.31572
SEC61B	W2	PGE2	well_4	F003	396	Total volume	129.6632	118.1005	76.54262
SEC61B	W2	PGE2	well_4	F004	386	Total volume	83.97164	77.90431	46.86699

SEC61B	W2	PGE2	well_4	F005	344	Total volume	98.68298	90.84924	52.44711
SEC61B	W2	PGE2	well_4	F006	254	Total volume	65.97074	57.11965	39.16364
SEC61B	W2	PGE2	well_5	F001	400	Total volume	69.7706	58.49278	42.56345
SEC61B	W2	PGE2	well_5	F002	178	Total volume	120.2073	113.4647	59.29161
SEC61B	W2	PGE2	well_5	F003	171	Total volume	55.54597	51.21639	34.38196
SEC61B	W2	PGE2	well_5	F004	344	Total volume	92.44391	86.27215	51.6744
SEC61B	W2	PGE2	well_5	F005	413	Total volume	109.7792	100.7193	54.06585
SEC61B	W2	PGE2	well_5	F006	367	Total volume	102.545	97.97306	48.67887
SEC61B	W3	PGE2	well_1	F001	92	Total volume	80.86007	66.56722	59.99808
SEC61B	W3	PGE2	well_1	F002	91	Total volume	38.33295	33.30708	26.45507
SEC61B	W3	PGE2	well_1	F003	165	Total volume	68.12956	59.26736	49.88743
SEC61B	W3	PGE2	well_1	F004	157	Total volume	86.08063	80.76792	40.21922
SEC61B	W3	PGE2	well_1	F005	155	Total volume	78.57355	75.39278	35.11219
SEC61B	W3	PGE2	well_1	F006	102	Total volume	155.8916	158.4258	73.53959
SEC61B	W3	PGE2	well_2	F001	97	Total volume	93.14988	86.51861	48.82759
SEC61B	W3	PGE2	well_2	F002	110	Total volume	72.50517	61.73194	36.41678
SEC61B	W3	PGE2	well_2	F003	137	Total volume	109.2161	106.9394	49.17558
SEC61B	W3	PGE2	well_2	F004	163	Total volume	103.6514	95.49674	54.87101
SEC61B	W3	PGE2	well_2	F005	87	Total volume	105.1683	101.2122	39.12355
SEC61B	W3	PGE2	well_2	F006	183	Total volume	90.53181	82.05889	43.10209
SEC61B	W3	PGE2	well_3	F001	150	Total volume	105.8012	99.87431	48.63377
SEC61B	W3	PGE2	well_3	F002	142	Total volume	122.259	117.4081	56.85987
SEC61B	W3	PGE2	well_3	F003	137	Total volume	97.1951	91.87028	48.8221
SEC61B	W3	PGE2	well_3	F004	183	Total volume	101.6371	96.79944	48.31657
SEC61B	W3	PGE2	well_3	F005	109	Total volume	116.8389	113.4413	46.97583
SEC61B	W3	PGE2	well_3	F006	39	Total volume	109.4356	103.7472	47.04676
SEC61B	W3	PGE2	well_4	F001	108	Total volume	110.1565	106.6343	51.47407
SEC61B	W3	PGE2	well_4	F002	132	Total volume	91.59091	84.68778	46.71405
SEC61B	W3	PGE2	well_4	F003	127	Total volume	92.95626	88.02083	45.99849
SEC61B	W3	PGE2	well_4	F004	124	Total volume	86.31416	80.81486	39.16744
SEC61B	W3	PGE2	well_4	F005	91	Total volume	109.7896	105.7658	50.9729
SEC61B	W3	PGE2	well_4	F006	133	Total volume	116.3448	112.9718	50.00814
SEC61B	W3	PGE2	well_5	F001	163	Total volume	82.46704	77.48181	45.78253
SEC61B	W3	PGE2	well_5	F002	68	Total volume	86.07645	71.49639	49.89394
SEC61B	W3	PGE2	well_5	F003	124	Total volume	135.0328	123.1118	63.12217
SEC61B	W3	PGE2	well_5	F004	160	Total volume	80.61939	73.44458	44.89613
SEC61B	W3	PGE2	well_5	F005	201	Total volume	101.0423	94.17056	51.3446
SEC61B	W3	PGE2	well_5	F006	141	Total volume	100.2156	93.65417	42.65655
SEC61B	W4	PGE2	well_1	F001	32	Total volume	38.06691	29.10556	27.04313
SEC61B	W4	PGE2	well_1	F002	10	Total volume	65.63714	61.33292	31.27616
SEC61B	W4	PGE2	well_1	F003	141	Total volume	93.89314	89.87514	41.71201
SEC61B	W4	PGE2	well_1	F004	195	Total volume	62.86773	60.32361	29.94791
SEC61B	W4	PGE2	well_1	F005	125	Total volume	77.84933	69.64208	43.48783
SEC61B	W4	PGE2	well_1	F006	38	Total volume	43.52164	39.93799	20.12551
SEC61B	W4	PGE2	well_2	F001	99	Total volume	54.01572	50.19535	23.35667
SEC61B	W4	PGE2	well_2	F002	170	Total volume	75.67197	72.98688	34.52702
SEC61B	W4	PGE2	well_2	F003	203	Total volume	90.22113	80.93222	46.82944
SEC61B	W4	PGE2	well_2	F004	224	Total volume	57.17574	57.46	30.21313
SEC61B	W4	PGE2	well_2	F005	169	Total volume	84.65653	88.63111	36.70928

SEC61B	W4	PGE2	well_2	F006	223	Total volume	83.3646	76.98889	47.06802
SEC61B	W4	PGE2	well_3	F001	102	Total volume	49.73529	43.97521	31.40849
SEC61B	W4	PGE2	well_3	F002	150	Total volume	96.51603	89.41743	52.88548
SEC61B	W4	PGE2	well_3	F003	203	Total volume	61.67177	55.37097	42.37205
SEC61B	W4	PGE2	well_3	F004	264	Total volume	92.27597	86.71813	43.71159
SEC61B	W4	PGE2	well_3	F005	191	Total volume	107.463	107.3854	42.43655
SEC61B	W4	PGE2	well_3	F006	211	Total volume	73.75991	65.22931	40.14485
SEC61B	W4	PGE2	well_4	F001	310	Total volume	93.04887	84.55868	47.62346
SEC61B	W4	PGE2	well_4	F002	230	Total volume	103.9622	99.4166	51.86588
SEC61B	W4	PGE2	well_4	F003	300	Total volume	121.5981	119.8492	47.1238
SEC61B	W4	PGE2	well_4	F004	348	Total volume	73.08249	69.34868	38.08661
SEC61B	W4	PGE2	well_4	F005	322	Total volume	109.6965	96.0366	60.8536
SEC61B	W4	PGE2	well_4	F006	201	Total volume	86.80974	80.67403	54.85339
SEC61B	W4	PGE2	well_5	F001	197	Total volume	81.21747	64.66597	55.67957
SEC61B	W4	PGE2	well_5	F002	148	Total volume	98.2028	94.05319	43.22078
SEC61B	W4	PGE2	well_5	F003	408	Total volume	96.40074	95.485	43.35423
SEC61B	W4	PGE2	well_5	F004	325	Total volume	104.207	96.84639	45.05186
SEC61B	W4	PGE2	well_5	F005	342	Total volume	79.65142	72.97514	41.06079
SEC61B	W4	PGE2	well_5	F006	216	Total volume	104.6897	105.7306	49.20669
ST6GAL1	W1	HPI4	well_1	F001	30	Avg. Volume	1.419805	1.186059	1.436994
ST6GAL1	W1	HPI4	well_1	F002	3	Avg. Volume	1.159167	1.159167	NA
ST6GAL1	W1	HPI4	well_1	F003	7	Avg. Volume	0.997213	1.109063	0.247552
ST6GAL1	W1	HPI4	well_1	F004	25	Avg. Volume	1.475012	0.948278	1.433993
ST6GAL1	W1	HPI4	well_1	F005	22	Avg. Volume	0.897847	0.873167	0.542054
ST6GAL1	W1	HPI4	well_1	F006	20	Avg. Volume	1.248483	0.575671	1.22452
ST6GAL1	W1	HPI4	well_2	F001	8	Avg. Volume	0.892084	0.957888	0.270731
ST6GAL1	W1	HPI4	well_2	F002	35	Avg. Volume	1.297544	0.891944	1.233732
ST6GAL1	W1	HPI4	well_2	F003	18	Avg. Volume	2.324814	1.263106	3.177106
ST6GAL1	W1	HPI4	well_2	F004	41	Avg. Volume	1.38241	0.946713	1.040967
ST6GAL1	W1	HPI4	well_2	F005	9	Avg. Volume	2.625461	0.516389	3.068449
ST6GAL1	W1	HPI4	well_2	F006	7	Avg. Volume	0.96441	0.657222	1.148903
ST6GAL1	W1	HPI4	well_3	F001	8	Avg. Volume	0.467161	0.353643	0.328697
ST6GAL1	W1	HPI4	well_3	F002	4	Avg. Volume	0.631042	0.631042	0.759649
ST6GAL1	W1	HPI4	well_3	F003	19	Avg. Volume	1.231391	1.096488	0.970126
ST6GAL1	W1	HPI4	well_3	F004	31	Avg. Volume	2.103455	1.191215	2.665763
ST6GAL1	W1	HPI4	well_3	F005	9	Avg. Volume	1.103133	0.697263	1.07996
ST6GAL1	W1	HPI4	well_3	F006	18	Avg. Volume	0.790837	0.464415	0.800675
ST6GAL1	W1	HPI4	well_4	F001	65	Avg. Volume	1.547103	1.154833	1.164089
ST6GAL1	W1	HPI4	well_4	F002	28	Avg. Volume	0.636974	0.504653	0.442848
ST6GAL1	W1	HPI4	well_4	F003	3	Avg. Volume	1.912083	1.912083	NA
ST6GAL1	W1	HPI4	well_4	F004	29	Avg. Volume	1.294867	0.686217	1.629685
ST6GAL1	W1	HPI4	well_4	F005	16	Avg. Volume	1.861693	1.325622	1.559626
ST6GAL1	W1	HPI4	well_4	F006	14	Avg. Volume	0.362757	0.362219	0.210779
ST6GAL1	W1	HPI4	well_5	F001	7	Avg. Volume	3.584678	3.520833	3.914056
ST6GAL1	W1	HPI4	well_5	F002	18	Avg. Volume	1.545586	0.987017	1.299479
ST6GAL1	W1	HPI4	well_5	F003	31	Avg. Volume	1.738036	1.262284	1.808384
ST6GAL1	W1	HPI4	well_5	F004	10	Avg. Volume	1.854431	0.677565	3.024079
ST6GAL1	W1	HPI4	well_5	F005	26	Avg. Volume	1.516126	0.83494	1.53117
ST6GAL1	W1	HPI4	well_5	F006	25	Avg. Volume	1.275222	0.447928	1.38938

ST6GAL1	W2	HPI4	well_1	F001	3	Avg. Volume	1.121332	1.121332	NA
ST6GAL1	W2	HPI4	well_1	F002	7	Avg. Volume	0.715696	0.714729	0.289424
ST6GAL1	W2	HPI4	well_1	F003	5	Avg. Volume	0.267714	0.258194	0.037495
ST6GAL1	W2	HPI4	well_1	F004	25	Avg. Volume	1.60747	1.240116	1.267122
ST6GAL1	W2	HPI4	well_1	F005	30	Avg. Volume	1.286382	1.087546	0.774894
ST6GAL1	W2	HPI4	well_1	F006	40	Avg. Volume	0.846532	0.7961	0.394298
ST6GAL1	W2	HPI4	well_2	F001	8	Avg. Volume	0.175419	0.117361	0.155322
ST6GAL1	W2	HPI4	well_2	F002	8	Avg. Volume	1.104132	1.25283	0.45049
ST6GAL1	W2	HPI4	well_2	F003	20	Avg. Volume	1.139649	0.947716	0.836083
ST6GAL1	W2	HPI4	well_2	F004	10	Avg. Volume	0.91333	1.008914	0.435129
ST6GAL1	W2	HPI4	well_2	F005	12	Avg. Volume	1.405597	1.322609	0.923516
ST6GAL1	W2	HPI4	well_2	F006	13	Avg. Volume	1.330489	1.18428	0.846537
ST6GAL1	W2	HPI4	well_3	F001	20	Avg. Volume	1.018385	0.931735	0.537456
ST6GAL1	W2	HPI4	well_3	F002	5	Avg. Volume	0.093889	0.093889	0.046944
ST6GAL1	W2	HPI4	well_3	F003	15	Avg. Volume	0.501897	0.443838	0.345783
ST6GAL1	W2	HPI4	well_3	F005	49	Avg. Volume	1.295083	1.013707	0.728445
ST6GAL1	W2	HPI4	well_3	F006	12	Avg. Volume	0.956511	0.967333	0.338385
ST6GAL1	W2	HPI4	well_4	F001	5	Avg. Volume	0.605566	0.804457	0.38276
ST6GAL1	W2	HPI4	well_4	F002	9	Avg. Volume	0.941542	1.047029	0.383921
ST6GAL1	W2	HPI4	well_4	F003	28	Avg. Volume	1.313165	1.2293	0.465833
ST6GAL1	W2	HPI4	well_4	F004	10	Avg. Volume	0.882137	0.843435	0.256668
ST6GAL1	W2	HPI4	well_4	F005	13	Avg. Volume	1.274582	0.983598	0.631888
ST6GAL1	W2	HPI4	well_4	F006	17	Avg. Volume	1.435193	1.036131	0.9752
ST6GAL1	W2	HPI4	well_5	F001	7	Avg. Volume	0.805488	0.725031	0.435923
ST6GAL1	W2	HPI4	well_5	F002	10	Avg. Volume	0.739026	0.702304	0.431656
ST6GAL1	W2	HPI4	well_5	F003	13	Avg. Volume	0.709514	0.655657	0.318749
ST6GAL1	W2	HPI4	well_5	F004	15	Avg. Volume	0.976115	0.539861	0.847516
ST6GAL1	W2	HPI4	well_5	F005	10	Avg. Volume	0.812718	0.831063	0.370544
ST6GAL1	W2	HPI4	well_5	F006	43	Avg. Volume	0.873251	0.865538	0.408614
ST6GAL1	W3	HPI4	well_1	F001	1	Avg. Volume	0.845	0.845	NA
ST6GAL1	W3	HPI4	well_1	F002	14	Avg. Volume	0.55747	0.248806	0.603642
ST6GAL1	W3	HPI4	well_1	F003	23	Avg. Volume	0.623994	0.518997	0.412007
ST6GAL1	W3	HPI4	well_1	F004	94	Avg. Volume	0.622088	0.507587	0.415472
ST6GAL1	W3	HPI4	well_1	F005	76	Avg. Volume	1.055871	1.01224	0.495968
ST6GAL1	W3	HPI4	well_1	F006	32	Avg. Volume	1.511867	1.625172	0.980096
ST6GAL1	W3	HPI4	well_2	F001	1	Avg. Volume	0.305139	0.305139	NA
ST6GAL1	W3	HPI4	well_2	F003	6	Avg. Volume	0.679139	0.632772	0.189735
ST6GAL1	W3	HPI4	well_2	F004	5	Avg. Volume	0.77173	0.649398	0.373141
ST6GAL1	W3	HPI4	well_2	F005	11	Avg. Volume	1.26975	0.942595	0.642829
ST6GAL1	W3	HPI4	well_2	F006	15	Avg. Volume	0.609485	0.524213	0.488929
ST6GAL1	W3	HPI4	well_3	F001	7	Avg. Volume	0.471792	0.359907	0.224143
ST6GAL1	W3	HPI4	well_3	F003	3	Avg. Volume	1.314444	1.314444	NA
ST6GAL1	W3	HPI4	well_3	F005	11	Avg. Volume	0.704534	0.661623	0.310656
ST6GAL1	W3	HPI4	well_3	F006	5	Avg. Volume	0.29262	0.21125	0.151215
ST6GAL1	W3	HPI4	well_4	F001	6	Avg. Volume	0.568028	0.539861	0.187033
ST6GAL1	W3	HPI4	well_4	F002	5	Avg. Volume	1.130579	1.067986	0.554254
ST6GAL1	W3	HPI4	well_4	F003	6	Avg. Volume	0.145723	0.123229	0.078069
ST6GAL1	W3	HPI4	well_4	F004	5	Avg. Volume	1.088919	0.809792	0.559468
ST6GAL1	W3	HPI4	well_4	F005	29	Avg. Volume	0.585393	0.617233	0.353504

ST6GAL1	W3	HPI4	well_4	F006	5	Avg. Volume	0.916862	0.934033	0.068521
ST6GAL1	W3	HPI4	well_5	F001	8	Avg. Volume	0.928948	0.843156	0.26824
ST6GAL1	W3	HPI4	well_5	F002	21	Avg. Volume	0.902856	0.826222	0.617187
ST6GAL1	W3	HPI4	well_5	F003	5	Avg. Volume	1.102098	0.97595	0.404073
ST6GAL1	W3	HPI4	well_5	F004	7	Avg. Volume	0.578543	0.563333	0.126917
ST6GAL1	W3	HPI4	well_5	F005	27	Avg. Volume	1.001388	1.062509	0.318902
ST6GAL1	W3	HPI4	well_5	F006	83	Avg. Volume	1.168536	1.051779	0.510455
ST6GAL1	W4	HPI4	well_1	F001	3	Avg. Volume	0.4225	0.4225	NA
ST6GAL1	W4	HPI4	well_1	F002	4	Avg. Volume	0.738201	0.738201	0.911195
ST6GAL1	W4	HPI4	well_1	F003	3	Avg. Volume	0.665046	0.665046	NA
ST6GAL1	W4	HPI4	well_1	F004	9	Avg. Volume	1.382456	1.049848	0.943919
ST6GAL1	W4	HPI4	well_1	F005	11	Avg. Volume	0.353639	0.21125	0.306995
ST6GAL1	W4	HPI4	well_1	F006	19	Avg. Volume	0.395468	0.357951	0.267771
ST6GAL1	W4	HPI4	well_2	F004	18	Avg. Volume	0.606407	0.558404	0.393455
ST6GAL1	W4	HPI4	well_2	F006	13	Avg. Volume	0.60552	0.677199	0.316794
ST6GAL1	W4	HPI4	well_3	F002	9	Avg. Volume	0.482159	0.349149	0.354929
ST6GAL1	W4	HPI4	well_3	F004	6	Avg. Volume	0.4225	0.305139	0.320118
ST6GAL1	W4	HPI4	well_3	F005	1	Avg. Volume	1.166569	1.166569	NA
ST6GAL1	W4	HPI4	well_4	F001	39	Avg. Volume	0.459677	0.404896	0.291982
ST6GAL1	W4	HPI4	well_4	F004	3	Avg. Volume	0.87937	0.87937	NA
ST6GAL1	W4	HPI4	well_4	F006	23	Avg. Volume	0.467215	0.445972	0.268058
ST6GAL1	W4	HPI4	well_5	F001	6	Avg. Volume	1.240339	1.183	0.172057
ST6GAL1	W4	HPI4	well_5	F003	9	Avg. Volume	0.355995	0.293403	0.187655
ST6GAL1	W4	HPI4	well_5	F004	16	Avg. Volume	0.563596	0.421718	0.38078
ST6GAL1	W4	HPI4	well_5	F005	13	Avg. Volume	0.905753	0.678561	0.726533
ST6GAL1	W4	HPI4	well_5	F006	5	Avg. Volume	0.662066	0.727639	0.276386
ST6GAL1	W1	PGE2	well_1	F001	8	Avg. Volume	0.623118	0.606256	0.350717
ST6GAL1	W1	PGE2	well_1	F002	8	Avg. Volume	0.476508	0.421522	0.315166
ST6GAL1	W1	PGE2	well_1	F003	9	Avg. Volume	1.084123	0.369154	1.36823
ST6GAL1	W1	PGE2	well_1	F004	12	Avg. Volume	1.281341	1.026351	1.257415
ST6GAL1	W1	PGE2	well_1	F005	23	Avg. Volume	0.82413	0.719815	0.422477
ST6GAL1	W1	PGE2	well_1	F006	33	Avg. Volume	1.138632	0.801968	1.043385
ST6GAL1	W1	PGE2	well_2	F001	8	Avg. Volume	0.691376	0.700966	0.385285
ST6GAL1	W1	PGE2	well_2	F002	11	Avg. Volume	1.201871	0.617421	1.534437
ST6GAL1	W1	PGE2	well_2	F003	20	Avg. Volume	1.765706	1.298014	1.548308
ST6GAL1	W1	PGE2	well_2	F004	4	Avg. Volume	0.504653	0.504653	0.182571
ST6GAL1	W1	PGE2	well_2	F005	19	Avg. Volume	1.11113	0.970185	0.868379
ST6GAL1	W1	PGE2	well_2	F006	14	Avg. Volume	1.119269	0.553064	1.803248
ST6GAL1	W1	PGE2	well_3	F001	13	Avg. Volume	1.254987	0.680694	1.159392
ST6GAL1	W1	PGE2	well_3	F002	5	Avg. Volume	0.287861	0.179954	0.198013
ST6GAL1	W1	PGE2	well_3	F003	1	Avg. Volume	1.204907	1.204907	NA
ST6GAL1	W1	PGE2	well_3	F004	19	Avg. Volume	0.687981	0.586806	0.541787
ST6GAL1	W1	PGE2	well_3	F005	18	Avg. Volume	1.066956	0.651589	1.016259
ST6GAL1	W1	PGE2	well_3	F006	8	Avg. Volume	1.449701	0.981166	1.213749
ST6GAL1	W1	PGE2	well_4	F001	11	Avg. Volume	0.896216	0.698299	0.642497
ST6GAL1	W1	PGE2	well_4	F002	6	Avg. Volume	0.578305	0.568363	0.225426
ST6GAL1	W1	PGE2	well_4	F003	7	Avg. Volume	0.29914	0.258194	0.171794
ST6GAL1	W1	PGE2	well_4	F004	1	Avg. Volume	2.004528	2.004528	NA
ST6GAL1	W1	PGE2	well_4	F005	88	Avg. Volume	1.380895	0.826558	1.575315

ST6GAL1	W1	PGE2	well_4	F006	7	Avg. Volume	1.342846	0.792188	1.266649
ST6GAL1	W1	PGE2	well_5	F001	7	Avg. Volume	1.374623	0.845	1.450095
ST6GAL1	W1	PGE2	well_5	F002	6	Avg. Volume	0.428368	0.416632	0.15287
ST6GAL1	W1	PGE2	well_5	F004	6	Avg. Volume	4.27319	3.46823	2.492579
ST6GAL1	W1	PGE2	well_5	F005	8	Avg. Volume	0.883501	0.866125	0.543718
ST6GAL1	W1	PGE2	well_5	F006	1	Avg. Volume	0.427194	0.427194	NA
ST6GAL1	W2	PGE2	well_1	F001	111	Avg. Volume	2.054857	1.189259	2.27999
ST6GAL1	W2	PGE2	well_1	F002	152	Avg. Volume	1.160274	0.704167	1.097945
ST6GAL1	W2	PGE2	well_1	F003	160	Avg. Volume	0.769755	0.667282	0.40053
ST6GAL1	W2	PGE2	well_1	F004	234	Avg. Volume	1.737716	1.232292	1.583424
ST6GAL1	W2	PGE2	well_1	F005	148	Avg. Volume	1.660981	1.151313	1.357717
ST6GAL1	W2	PGE2	well_1	F006	184	Avg. Volume	1.061222	0.834338	0.77893
ST6GAL1	W2	PGE2	well_2	F001	64	Avg. Volume	0.928808	0.61419	0.894394
ST6GAL1	W2	PGE2	well_2	F002	175	Avg. Volume	1.190909	0.877274	0.879848
ST6GAL1	W2	PGE2	well_2	F003	338	Avg. Volume	0.931423	0.742635	0.650982
ST6GAL1	W2	PGE2	well_2	F004	145	Avg. Volume	1.764815	1.334005	1.493045
ST6GAL1	W2	PGE2	well_2	F005	88	Avg. Volume	1.046654	0.719256	0.960475
ST6GAL1	W2	PGE2	well_2	F006	268	Avg. Volume	1.787701	1.312097	1.58242
ST6GAL1	W2	PGE2	well_3	F001	42	Avg. Volume	1.425114	0.913069	1.405596
ST6GAL1	W2	PGE2	well_3	F002	220	Avg. Volume	1.205306	0.809792	1.238752
ST6GAL1	W2	PGE2	well_3	F003	49	Avg. Volume	1.860662	1.126667	1.685291
ST6GAL1	W2	PGE2	well_3	F004	77	Avg. Volume	1.557897	0.946713	1.897639
ST6GAL1	W2	PGE2	well_3	F005	198	Avg. Volume	1.051956	0.793026	0.911895
ST6GAL1	W2	PGE2	well_3	F006	276	Avg. Volume	1.272037	0.995613	0.993765
ST6GAL1	W2	PGE2	well_4	F001	44	Avg. Volume	0.892119	0.444128	0.940614
ST6GAL1	W2	PGE2	well_4	F002	291	Avg. Volume	0.820875	0.712702	0.482916
ST6GAL1	W2	PGE2	well_4	F003	71	Avg. Volume	2.567914	1.722274	2.352442
ST6GAL1	W2	PGE2	well_4	F004	72	Avg. Volume	0.961471	0.718664	0.727832
ST6GAL1	W2	PGE2	well_4	F005	39	Avg. Volume	1.302805	0.918351	0.922655
ST6GAL1	W2	PGE2	well_4	F006	53	Avg. Volume	1.409733	0.710873	1.609041
ST6GAL1	W2	PGE2	well_5	F001	239	Avg. Volume	2.002763	1.240116	1.920049
ST6GAL1	W2	PGE2	well_5	F002	250	Avg. Volume	1.184906	1.009306	0.82031
ST6GAL1	W2	PGE2	well_5	F003	158	Avg. Volume	2.082686	1.395424	2.115232
ST6GAL1	W2	PGE2	well_5	F004	13	Avg. Volume	1.009306	0.782407	0.883002
ST6GAL1	W2	PGE2	well_5	F005	11	Avg. Volume	1.423899	0.872384	1.213718
ST6GAL1	W2	PGE2	well_5	F006	117	Avg. Volume	1.644516	0.906028	1.617296
ST6GAL1	W3	PGE2	well_1	F001	195	Avg. Volume	1.086764	0.850868	0.727859
ST6GAL1	W3	PGE2	well_1	F002	350	Avg. Volume	1.145942	1.072345	0.594858
ST6GAL1	W3	PGE2	well_1	F003	293	Avg. Volume	1.11839	0.988767	0.655478
ST6GAL1	W3	PGE2	well_1	F004	136	Avg. Volume	1.132231	1.066414	0.61907
ST6GAL1	W3	PGE2	well_1	F005	277	Avg. Volume	1.298111	1.009306	0.951749
ST6GAL1	W3	PGE2	well_1	F006	339	Avg. Volume	0.904096	0.784063	0.526928
ST6GAL1	W3	PGE2	well_2	F001	180	Avg. Volume	0.802357	0.637456	0.533217
ST6GAL1	W3	PGE2	well_2	F002	60	Avg. Volume	0.676123	0.592022	0.474135
ST6GAL1	W3	PGE2	well_2	F003	321	Avg. Volume	0.910801	0.83033	0.529556
ST6GAL1	W3	PGE2	well_2	F004	295	Avg. Volume	1.243576	1.009306	0.858314
ST6GAL1	W3	PGE2	well_2	F005	179	Avg. Volume	0.98594	0.827396	0.553668
ST6GAL1	W3	PGE2	well_2	F006	324	Avg. Volume	0.929053	0.795698	0.575125
ST6GAL1	W3	PGE2	well_3	F001	186	Avg. Volume	0.826663	0.515085	0.819259

ST6GAL1	W3	PGE2	well_3	F002	183	Avg. Volume	0.641205	0.46162	0.581002
ST6GAL1	W3	PGE2	well_3	F003	239	Avg. Volume	0.710831	0.584458	0.523971
ST6GAL1	W3	PGE2	well_3	F004	152	Avg. Volume	0.806302	0.668958	0.572146
ST6GAL1	W3	PGE2	well_3	F005	152	Avg. Volume	2.123328	0.988767	2.506777
ST6GAL1	W3	PGE2	well_3	F006	233	Avg. Volume	0.809947	0.511694	0.824178
ST6GAL1	W3	PGE2	well_4	F001	197	Avg. Volume	0.777543	0.542795	0.703396
ST6GAL1	W3	PGE2	well_4	F002	218	Avg. Volume	0.566904	0.406852	0.475887
ST6GAL1	W3	PGE2	well_4	F003	215	Avg. Volume	1.514553	1.075028	1.41088
ST6GAL1	W3	PGE2	well_4	F004	205	Avg. Volume	0.776418	0.469444	0.808869
ST6GAL1	W3	PGE2	well_4	F005	222	Avg. Volume	0.978208	0.791601	0.674466
ST6GAL1	W3	PGE2	well_4	F006	214	Avg. Volume	0.886336	0.785928	0.545066
ST6GAL1	W3	PGE2	well_5	F001	57	Avg. Volume	0.570189	0.469444	0.412279
ST6GAL1	W3	PGE2	well_5	F002	70	Avg. Volume	0.42904	0.399028	0.318956
ST6GAL1	W3	PGE2	well_5	F003	237	Avg. Volume	0.677114	0.542208	0.519702
ST6GAL1	W3	PGE2	well_5	F004	265	Avg. Volume	1.231865	0.771649	1.227115
ST6GAL1	W3	PGE2	well_5	F005	166	Avg. Volume	1.023409	0.800403	0.70846
ST6GAL1	W3	PGE2	well_5	F006	38	Avg. Volume	1.028191	0.795122	0.807442
ST6GAL1	W4	PGE2	well_1	F001	67	Avg. Volume	0.404415	0.299271	0.302108
ST6GAL1	W4	PGE2	well_1	F002	46	Avg. Volume	0.813934	0.760713	0.442969
ST6GAL1	W4	PGE2	well_1	F003	193	Avg. Volume	0.56557	0.457219	0.429181
ST6GAL1	W4	PGE2	well_1	F004	130	Avg. Volume	0.492291	0.494547	0.23913
ST6GAL1	W4	PGE2	well_1	F005	171	Avg. Volume	0.595878	0.477269	0.501167
ST6GAL1	W4	PGE2	well_1	F006	173	Avg. Volume	1.326682	0.903681	1.192555
ST6GAL1	W4	PGE2	well_2	F001	204	Avg. Volume	0.517278	0.382891	0.418949
ST6GAL1	W4	PGE2	well_2	F002	61	Avg. Volume	0.490978	0.40946	0.303642
ST6GAL1	W4	PGE2	well_2	F003	71	Avg. Volume	1.009009	0.764021	0.712577
ST6GAL1	W4	PGE2	well_2	F004	97	Avg. Volume	0.556168	0.382262	0.424164
ST6GAL1	W4	PGE2	well_2	F005	63	Avg. Volume	0.514998	0.458775	0.242681
ST6GAL1	W4	PGE2	well_2	F006	135	Avg. Volume	0.5479	0.488222	0.330434
ST6GAL1	W4	PGE2	well_3	F001	193	Avg. Volume	0.704487	0.523095	0.650691
ST6GAL1	W4	PGE2	well_3	F002	13	Avg. Volume	0.258325	0.105625	0.342272
ST6GAL1	W4	PGE2	well_3	F003	13	Avg. Volume	0.301215	0.309051	0.189423
ST6GAL1	W4	PGE2	well_3	F004	224	Avg. Volume	0.757219	0.602454	0.484843
ST6GAL1	W4	PGE2	well_3	F005	182	Avg. Volume	0.75109	0.523561	0.653165
ST6GAL1	W4	PGE2	well_3	F006	42	Avg. Volume	0.39816	0.285188	0.387657
ST6GAL1	W4	PGE2	well_4	F001	166	Avg. Volume	1.058879	0.816247	0.829838
ST6GAL1	W4	PGE2	well_4	F002	91	Avg. Volume	0.510509	0.404337	0.396106
ST6GAL1	W4	PGE2	well_4	F003	232	Avg. Volume	0.726017	0.608601	0.503974
ST6GAL1	W4	PGE2	well_4	F004	173	Avg. Volume	0.459475	0.39316	0.290782
ST6GAL1	W4	PGE2	well_4	F005	263	Avg. Volume	0.605035	0.452818	0.514137
ST6GAL1	W4	PGE2	well_4	F006	32	Avg. Volume	0.537064	0.429102	0.393188
ST6GAL1	W4	PGE2	well_5	F001	57	Avg. Volume	0.263817	0.187778	0.224311
ST6GAL1	W4	PGE2	well_5	F002	96	Avg. Volume	0.478388	0.336435	0.417697
ST6GAL1	W4	PGE2	well_5	F004	95	Avg. Volume	0.578964	0.481181	0.437319
ST6GAL1	W4	PGE2	well_5	F006	4	Avg. Volume	0.74366	0.74366	0.017915
ST6GAL1	W1	HPI4	well_1	F001	30	Count/cell	5.769231	6	2.657934
ST6GAL1	W1	HPI4	well_1	F002	3	Count/cell	13	13	NA
ST6GAL1	W1	HPI4	well_1	F003	7	Count/cell	9.8	10	4.868265
ST6GAL1	W1	HPI4	well_1	F004	25	Count/cell	5.772727	5	2.990968

ST6GAL1	W1	HPI4	well_1	F005	22	Count/cell	6.65	5	5.091531
ST6GAL1	W1	HPI4	well_1	F006	20	Count/cell	7.157895	7	4.045068
ST6GAL1	W1	HPI4	well_2	F001	8	Count/cell	8.5	8.5	4.037326
ST6GAL1	W1	HPI4	well_2	F002	35	Count/cell	6.393939	6	3.831933
ST6GAL1	W1	HPI4	well_2	F003	18	Count/cell	5.529412	4	4.556831
ST6GAL1	W1	HPI4	well_2	F004	41	Count/cell	5.794872	5	3.434956
ST6GAL1	W1	HPI4	well_2	F005	9	Count/cell	4.333333	3	3
ST6GAL1	W1	HPI4	well_2	F006	7	Count/cell	5.4	5	2.073644
ST6GAL1	W1	HPI4	well_3	F001	8	Count/cell	5.333333	4	3.50238
ST6GAL1	W1	HPI4	well_3	F002	4	Count/cell	7	7	6.928203
ST6GAL1	W1	HPI4	well_3	F003	19	Count/cell	13.17647	12	8.442714
ST6GAL1	W1	HPI4	well_3	F004	31	Count/cell	6.241379	6	3.747906
ST6GAL1	W1	HPI4	well_3	F005	9	Count/cell	8.125	6.5	6.642665
ST6GAL1	W1	HPI4	well_3	F006	18	Count/cell	5.75	5	3.454466
ST6GAL1	W1	HPI4	well_4	F001	65	Count/cell	7.015873	6	4.248309
ST6GAL1	W1	HPI4	well_4	F002	28	Count/cell	6.083333	6.5	2.412227
ST6GAL1	W1	HPI4	well_4	F003	3	Count/cell	12	12	NA
ST6GAL1	W1	HPI4	well_4	F004	29	Count/cell	6.96	6	3.69098
ST6GAL1	W1	HPI4	well_4	F005	16	Count/cell	8.928571	7	4.598734
ST6GAL1	W1	HPI4	well_4	F006	14	Count/cell	4	3	3.605551
ST6GAL1	W1	HPI4	well_5	F001	7	Count/cell	2.714286	1	2.13809
ST6GAL1	W1	HPI4	well_5	F002	18	Count/cell	11.52941	12	6.236161
ST6GAL1	W1	HPI4	well_5	F003	31	Count/cell	7.111111	7	4.758259
ST6GAL1	W1	HPI4	well_5	F004	10	Count/cell	4.222222	4	1.787301
ST6GAL1	W1	HPI4	well_5	F005	26	Count/cell	6.28	6	3.657413
ST6GAL1	W1	HPI4	well_5	F006	25	Count/cell	7.333333	6	4.246567
ST6GAL1	W2	HPI4	well_1	F001	3	Count/cell	19	19	NA
ST6GAL1	W2	HPI4	well_1	F002	7	Count/cell	6.666667	5	7.089899
ST6GAL1	W2	HPI4	well_1	F003	5	Count/cell	5.25	4.5	2.872281
ST6GAL1	W2	HPI4	well_1	F004	25	Count/cell	9.181818	8	4.338492
ST6GAL1	W2	HPI4	well_1	F005	30	Count/cell	8.692308	7.5	4.688447
ST6GAL1	W2	HPI4	well_1	F006	40	Count/cell	6.447368	5	4.098104
ST6GAL1	W2	HPI4	well_2	F001	8	Count/cell	2.714286	2	2.360387
ST6GAL1	W2	HPI4	well_2	F002	8	Count/cell	13.5	17	8.479387
ST6GAL1	W2	HPI4	well_2	F003	20	Count/cell	13.05263	16	7.066931
ST6GAL1	W2	HPI4	well_2	F004	10	Count/cell	14.125	16.5	6.379375
ST6GAL1	W2	HPI4	well_2	F005	12	Count/cell	11.90909	11	6.833075
ST6GAL1	W2	HPI4	well_2	F006	13	Count/cell	6.454545	5	4.546727
ST6GAL1	W2	HPI4	well_3	F001	20	Count/cell	12.22222	11	6.804458
ST6GAL1	W2	HPI4	well_3	F002	5	Count/cell	1.4	1	0.547723
ST6GAL1	W2	HPI4	well_3	F003	15	Count/cell	5.214286	5	3.378178
ST6GAL1	W2	HPI4	well_3	F005	49	Count/cell	14.29545	13	6.006119
ST6GAL1	W2	HPI4	well_3	F006	12	Count/cell	16.8	15.5	7.699928
ST6GAL1	W2	HPI4	well_4	F001	5	Count/cell	6.666667	5	3.785939
ST6GAL1	W2	HPI4	well_4	F002	9	Count/cell	18.57143	19	6.679749
ST6GAL1	W2	HPI4	well_4	F003	28	Count/cell	8.875	8	4.194328
ST6GAL1	W2	HPI4	well_4	F004	10	Count/cell	13.625	14	4.240536
ST6GAL1	W2	HPI4	well_4	F005	13	Count/cell	20.81818	20	8.942239
ST6GAL1	W2	HPI4	well_4	F006	17	Count/cell	8.666667	8	2.870208

ST6GAL1	W2	HPI4	well_5	F001	7	Count/cell	11.8	10	4.147288
ST6GAL1	W2	HPI4	well_5	F002	10	Count/cell	16.625	17	5.878229
ST6GAL1	W2	HPI4	well_5	F003	13	Count/cell	16.90909	19	8.16645
ST6GAL1	W2	HPI4	well_5	F004	15	Count/cell	10.92857	9	6.462487
ST6GAL1	W2	HPI4	well_5	F005	10	Count/cell	12.125	11	4.12094
ST6GAL1	W2	HPI4	well_5	F006	43	Count/cell	12.05405	11	5.296466
ST6GAL1	W3	HPI4	well_1	F001	1	Count/cell	6	6	NA
ST6GAL1	W3	HPI4	well_1	F002	14	Count/cell	3.692308	1	3.705505
ST6GAL1	W3	HPI4	well_1	F003	23	Count/cell	6.047619	4	5.305433
ST6GAL1	W3	HPI4	well_1	F004	94	Count/cell	4.11236	4	2.428234
ST6GAL1	W3	HPI4	well_1	F005	76	Count/cell	8.536232	7	4.142959
ST6GAL1	W3	HPI4	well_1	F006	32	Count/cell	5.466667	5	3.401149
ST6GAL1	W3	HPI4	well_2	F001	1	Count/cell	3	3	NA
ST6GAL1	W3	HPI4	well_2	F003	6	Count/cell	13	11	4.690416
ST6GAL1	W3	HPI4	well_2	F004	5	Count/cell	9.333333	9	1.527525
ST6GAL1	W3	HPI4	well_2	F005	11	Count/cell	9.222222	7	5.911383
ST6GAL1	W3	HPI4	well_2	F006	15	Count/cell	6.285714	5.5	4.479502
ST6GAL1	W3	HPI4	well_3	F001	7	Count/cell	2.333333	2	1.505545
ST6GAL1	W3	HPI4	well_3	F003	3	Count/cell	18	18	NA
ST6GAL1	W3	HPI4	well_3	F005	11	Count/cell	11.88889	11	5.206833
ST6GAL1	W3	HPI4	well_3	F006	5	Count/cell	5.333333	4	4.163332
ST6GAL1	W3	HPI4	well_4	F001	6	Count/cell	3	3	0.816497
ST6GAL1	W3	HPI4	well_4	F002	5	Count/cell	2.25	2	0.5
ST6GAL1	W3	HPI4	well_4	F003	6	Count/cell	1.4	1	0.547723
ST6GAL1	W3	HPI4	well_4	F004	5	Count/cell	14.66667	13	3.785939
ST6GAL1	W3	HPI4	well_4	F005	29	Count/cell	7.37037	7	5.130213
ST6GAL1	W3	HPI4	well_4	F006	5	Count/cell	13.66667	13	2.081666
ST6GAL1	W3	HPI4	well_5	F001	8	Count/cell	10.5	9.5	4.037326
ST6GAL1	W3	HPI4	well_5	F002	21	Count/cell	9.210526	10	5.137865
ST6GAL1	W3	HPI4	well_5	F003	5	Count/cell	13	14	2.645751
ST6GAL1	W3	HPI4	well_5	F004	7	Count/cell	13	12	4.41588
ST6GAL1	W3	HPI4	well_5	F005	27	Count/cell	18.6087	17	8.305196
ST6GAL1	W3	HPI4	well_5	F006	83	Count/cell	14.19178	14	4.843832
ST6GAL1	W4	HPI4	well_1	F001	3	Count/cell	2	2	0
ST6GAL1	W4	HPI4	well_1	F002	4	Count/cell	6.5	6.5	4.949747
ST6GAL1	W4	HPI4	well_1	F003	3	Count/cell	6	6	NA
ST6GAL1	W4	HPI4	well_1	F004	9	Count/cell	12.85714	11	8.53285
ST6GAL1	W4	HPI4	well_1	F005	11	Count/cell	4.777778	3	4.176655
ST6GAL1	W4	HPI4	well_1	F006	19	Count/cell	5.222222	2.5	5.866711
ST6GAL1	W4	HPI4	well_2	F004	18	Count/cell	12.5625	11	7.311806
ST6GAL1	W4	HPI4	well_2	F006	13	Count/cell	12	10	7.141428
ST6GAL1	W4	HPI4	well_3	F001	2	Count/cell	1	1	0
ST6GAL1	W4	HPI4	well_3	F002	9	Count/cell	2.875	1.5	2.695896
ST6GAL1	W4	HPI4	well_3	F004	6	Count/cell	1.666667	2	0.516398
ST6GAL1	W4	HPI4	well_3	F005	1	Count/cell	10	10	NA
ST6GAL1	W4	HPI4	well_4	F001	39	Count/cell	3.162162	2	2.03461
ST6GAL1	W4	HPI4	well_4	F004	3	Count/cell	13	13	NA
ST6GAL1	W4	HPI4	well_4	F006	23	Count/cell	5.809524	3	5.455447
ST6GAL1	W4	HPI4	well_5	F001	6	Count/cell	3.8	4	2.774887

ST6GAL1	W4	HPI4	well_5	F003	9	Count/cell	2.375	2.5	1.06066
ST6GAL1	W4	HPI4	well_5	F004	16	Count/cell	9.642857	8	6.979168
ST6GAL1	W4	HPI4	well_5	F005	13	Count/cell	10.09091	8	5.430553
ST6GAL1	W4	HPI4	well_5	F006	5	Count/cell	6.333333	7	3.05505
ST6GAL1	W1	PGE2	well_1	F001	8	Count/cell	6.833333	6.5	2.639444
ST6GAL1	W1	PGE2	well_1	F002	8	Count/cell	5.666667	5.5	3.829708
ST6GAL1	W1	PGE2	well_1	F003	9	Count/cell	6	4	4.281744
ST6GAL1	W1	PGE2	well_1	F004	12	Count/cell	4.818182	3	5.510321
ST6GAL1	W1	PGE2	well_1	F005	23	Count/cell	6.947368	6	3.153018
ST6GAL1	W1	PGE2	well_1	F006	33	Count/cell	7.034483	6	4.075035
ST6GAL1	W1	PGE2	well_2	F001	8	Count/cell	6.5	6.5	2.880972
ST6GAL1	W1	PGE2	well_2	F002	11	Count/cell	7.666667	8	2.598076
ST6GAL1	W1	PGE2	well_2	F003	20	Count/cell	7	6	3.574602
ST6GAL1	W1	PGE2	well_2	F004	4	Count/cell	7	7	1.414214
ST6GAL1	W1	PGE2	well_2	F005	19	Count/cell	6.368421	6	4.412237
ST6GAL1	W1	PGE2	well_2	F006	14	Count/cell	6.230769	5	4.6575
ST6GAL1	W1	PGE2	well_3	F001	13	Count/cell	6.666667	6	4.96045
ST6GAL1	W1	PGE2	well_3	F002	5	Count/cell	8.666667	8	3.05505
ST6GAL1	W1	PGE2	well_3	F003	1	Count/cell	9	9	NA
ST6GAL1	W1	PGE2	well_3	F004	19	Count/cell	8.277778	8	4.848354
ST6GAL1	W1	PGE2	well_3	F005	18	Count/cell	11.41176	10	9.447611
ST6GAL1	W1	PGE2	well_3	F006	8	Count/cell	5.714286	4	4.231402
ST6GAL1	W1	PGE2	well_4	F001	11	Count/cell	6	5	2.179449
ST6GAL1	W1	PGE2	well_4	F002	6	Count/cell	9.75	10.5	4.349329
ST6GAL1	W1	PGE2	well_4	F003	7	Count/cell	4.6	4	2.50998
ST6GAL1	W1	PGE2	well_4	F004	1	Count/cell	30	30	NA
ST6GAL1	W1	PGE2	well_4	F005	88	Count/cell	4.238095	4	2.664443
ST6GAL1	W1	PGE2	well_4	F006	7	Count/cell	4.4	5	0.894427
ST6GAL1	W1	PGE2	well_5	F001	7	Count/cell	6.8	7	2.167948
ST6GAL1	W1	PGE2	well_5	F002	6	Count/cell	4.4	4	1.67332
ST6GAL1	W1	PGE2	well_5	F004	6	Count/cell	6.25	6.5	1.707825
ST6GAL1	W1	PGE2	well_5	F005	8	Count/cell	7.666667	7	4.501851
ST6GAL1	W1	PGE2	well_5	F006	1	Count/cell	10	10	NA
ST6GAL1	W2	PGE2	well_1	F001	111	Count/cell	4.226415	4	2.59407
ST6GAL1	W2	PGE2	well_1	F002	152	Count/cell	5.657534	5	3.299819
ST6GAL1	W2	PGE2	well_1	F003	160	Count/cell	9.578947	9	4.366529
ST6GAL1	W2	PGE2	well_1	F004	234	Count/cell	5.022124	5	2.83697
ST6GAL1	W2	PGE2	well_1	F005	148	Count/cell	6.179104	6	3.251028
ST6GAL1	W2	PGE2	well_1	F006	184	Count/cell	8.077844	8	3.829175
ST6GAL1	W2	PGE2	well_2	F001	64	Count/cell	6.719298	6	3.200192
ST6GAL1	W2	PGE2	well_2	F002	175	Count/cell	8.242236	8	3.589527
ST6GAL1	W2	PGE2	well_2	F003	338	Count/cell	7.605178	7	3.956678
ST6GAL1	W2	PGE2	well_2	F004	145	Count/cell	5.438849	5	2.719002
ST6GAL1	W2	PGE2	well_2	F005	88	Count/cell	6.525	6	3.28007
ST6GAL1	W2	PGE2	well_2	F006	268	Count/cell	5.003802	5	2.571865
ST6GAL1	W2	PGE2	well_3	F001	42	Count/cell	6.864865	7	2.830019
ST6GAL1	W2	PGE2	well_3	F002	220	Count/cell	3.775701	3	2.433369
ST6GAL1	W2	PGE2	well_3	F003	49	Count/cell	5.209302	4	2.988535
ST6GAL1	W2	PGE2	well_3	F004	77	Count/cell	4.726027	4	3.028844

ST6GAL1	W2	PGE2	well_3	F005	198	Count/cell	5.989418	6	3.321416
ST6GAL1	W2	PGE2	well_3	F006	276	Count/cell	6.745098	6	3.310941
ST6GAL1	W2	PGE2	well_4	F001	44	Count/cell	4.428571	5	2.28615
ST6GAL1	W2	PGE2	well_4	F002	291	Count/cell	8.397727	8	4.042191
ST6GAL1	W2	PGE2	well_4	F003	71	Count/cell	4.338235	4	2.702184
ST6GAL1	W2	PGE2	well_4	F004	72	Count/cell	4.666667	4	3.037349
ST6GAL1	W2	PGE2	well_4	F005	39	Count/cell	6.432432	6	2.764261
ST6GAL1	W2	PGE2	well_4	F006	53	Count/cell	3.4	3	2.020305
ST6GAL1	W2	PGE2	well_5	F001	239	Count/cell	5.214912	5	3.154236
ST6GAL1	W2	PGE2	well_5	F002	250	Count/cell	6.869198	6	3.652805
ST6GAL1	W2	PGE2	well_5	F003	158	Count/cell	4.225166	4	2.358452
ST6GAL1	W2	PGE2	well_5	F004	13	Count/cell	5.307692	4	2.657838
ST6GAL1	W2	PGE2	well_5	F005	11	Count/cell	3.4	2.5	2.366432
ST6GAL1	W2	PGE2	well_5	F006	117	Count/cell	4.535714	4	3.025205
ST6GAL1	W3	PGE2	well_1	F001	195	Count/cell	9.648352	9	4.257911
ST6GAL1	W3	PGE2	well_1	F002	350	Count/cell	10.10377	10	3.7834
ST6GAL1	W3	PGE2	well_1	F003	293	Count/cell	10.59559	10	5.238769
ST6GAL1	W3	PGE2	well_1	F004	136	Count/cell	9.341085	9	3.977468
ST6GAL1	W3	PGE2	well_1	F005	277	Count/cell	8.599206	8	3.868619
ST6GAL1	W3	PGE2	well_1	F006	339	Count/cell	9.086687	9	4.04308
ST6GAL1	W3	PGE2	well_2	F001	180	Count/cell	6.732558	7	3.724188
ST6GAL1	W3	PGE2	well_2	F002	60	Count/cell	7.490909	7	4.109036
ST6GAL1	W3	PGE2	well_2	F003	321	Count/cell	8.119048	8	3.597892
ST6GAL1	W3	PGE2	well_2	F004	295	Count/cell	7.659259	7	3.701026
ST6GAL1	W3	PGE2	well_2	F005	179	Count/cell	13.18293	13	5.537664
ST6GAL1	W3	PGE2	well_2	F006	324	Count/cell	9.686469	10	4.638314
ST6GAL1	W3	PGE2	well_3	F001	186	Count/cell	5.342697	5	3.267798
ST6GAL1	W3	PGE2	well_3	F002	183	Count/cell	3.844828	3	2.383482
ST6GAL1	W3	PGE2	well_3	F003	239	Count/cell	5.321586	5	3.221556
ST6GAL1	W3	PGE2	well_3	F004	152	Count/cell	4.719178	4	2.820475
ST6GAL1	W3	PGE2	well_3	F005	152	Count/cell	3.282759	3	1.730861
ST6GAL1	W3	PGE2	well_3	F006	233	Count/cell	4.172566	4	2.456981
ST6GAL1	W3	PGE2	well_4	F001	197	Count/cell	4.391534	4	3.086541
ST6GAL1	W3	PGE2	well_4	F002	218	Count/cell	3.600962	3	2.28826
ST6GAL1	W3	PGE2	well_4	F003	215	Count/cell	5.771845	5	3.404643
ST6GAL1	W3	PGE2	well_4	F004	205	Count/cell	4.193878	4	2.727479
ST6GAL1	W3	PGE2	well_4	F005	222	Count/cell	6.950739	7	3.186844
ST6GAL1	W3	PGE2	well_4	F006	214	Count/cell	6.34715	6	3.171932
ST6GAL1	W3	PGE2	well_5	F001	57	Count/cell	4.722222	4	2.967857
ST6GAL1	W3	PGE2	well_5	F002	70	Count/cell	3.606061	3	2.62461
ST6GAL1	W3	PGE2	well_5	F003	237	Count/cell	5.421053	5	3.150819
ST6GAL1	W3	PGE2	well_5	F004	265	Count/cell	4.527778	4	2.459882
ST6GAL1	W3	PGE2	well_5	F005	166	Count/cell	6.282895	6	2.836418
ST6GAL1	W3	PGE2	well_5	F006	38	Count/cell	4.972222	4	2.751479
ST6GAL1	W4	PGE2	well_1	F001	67	Count/cell	3.296875	3	2.237343
ST6GAL1	W4	PGE2	well_1	F002	46	Count/cell	11.68293	11	4.59042
ST6GAL1	W4	PGE2	well_1	F003	193	Count/cell	5.227027	4	3.605902
ST6GAL1	W4	PGE2	well_1	F004	130	Count/cell	8.349593	7	5.265078
ST6GAL1	W4	PGE2	well_1	F005	171	Count/cell	4.037037	4	2.364039

ST6GAL1	W4	PGE2	well_1	F006	173	Count/cell	4.45122	4	2.452756
ST6GAL1	W4	PGE2	well_2	F001	204	Count/cell	3.319588	3	2.157269
ST6GAL1	W4	PGE2	well_2	F002	61	Count/cell	4.068966	3	2.574245
ST6GAL1	W4	PGE2	well_2	F003	71	Count/cell	6.373134	6	4.202582
ST6GAL1	W4	PGE2	well_2	F004	97	Count/cell	4.537634	4	2.784113
ST6GAL1	W4	PGE2	well_2	F005	63	Count/cell	11.15	11	5.473588
ST6GAL1	W4	PGE2	well_2	F006	135	Count/cell	7.024	6	4.313265
ST6GAL1	W4	PGE2	well_3	F001	193	Count/cell	4.568306	4	2.724428
ST6GAL1	W4	PGE2	well_3	F002	13	Count/cell	1.5	1	0.797724
ST6GAL1	W4	PGE2	well_3	F003	13	Count/cell	5.833333	6	4.26046
ST6GAL1	W4	PGE2	well_3	F004	224	Count/cell	7.441176	7	3.91177
ST6GAL1	W4	PGE2	well_3	F005	182	Count/cell	4.626437	4	2.937419
ST6GAL1	W4	PGE2	well_3	F006	42	Count/cell	3.384615	3	1.941451
ST6GAL1	W4	PGE2	well_4	F001	166	Count/cell	7.1375	6	4.577065
ST6GAL1	W4	PGE2	well_4	F002	91	Count/cell	4.325581	4	2.842707
ST6GAL1	W4	PGE2	well_4	F003	232	Count/cell	5.486364	5	3.268054
ST6GAL1	W4	PGE2	well_4	F004	173	Count/cell	5.230303	4	3.623474
ST6GAL1	W4	PGE2	well_4	F005	263	Count/cell	3.972112	4	2.437051
ST6GAL1	W4	PGE2	well_4	F006	32	Count/cell	8.4	7.5	5.721104
ST6GAL1	W4	PGE2	well_5	F001	57	Count/cell	3.092593	2	2.349376
ST6GAL1	W4	PGE2	well_5	F002	96	Count/cell	3.473684	3	2.513123
ST6GAL1	W4	PGE2	well_5	F004	95	Count/cell	7.079545	7	3.346714
ST6GAL1	W4	PGE2	well_5	F006	4	Count/cell	8	8	1.414214
ST6GAL1	W1	HPI4	well_1	F001	30	X/Y distribution	5.700049	5.746571	1.612431
ST6GAL1	W1	HPI4	well_1	F002	3	X/Y distribution	8.090991	8.090991	NA
ST6GAL1	W1	HPI4	well_1	F003	7	X/Y distribution	6.124038	6.820102	1.256027
ST6GAL1	W1	HPI4	well_1	F004	25	X/Y distribution	5.341456	5.251309	1.189468
ST6GAL1	W1	HPI4	well_1	F005	22	X/Y distribution	6.020919	5.810905	1.954901
ST6GAL1	W1	HPI4	well_1	F006	20	X/Y distribution	5.781885	5.92622	1.740689
ST6GAL1	W1	HPI4	well_2	F001	8	X/Y distribution	7.303399	7.549484	1.536756
ST6GAL1	W1	HPI4	well_2	F002	35	X/Y distribution	6.476251	6.407008	1.824492
ST6GAL1	W1	HPI4	well_2	F003	18	X/Y distribution	6.519998	6.144876	1.597288
ST6GAL1	W1	HPI4	well_2	F004	41	X/Y distribution	5.697198	5.471722	1.931772
ST6GAL1	W1	HPI4	well_2	F005	9	X/Y distribution	4.764117	5.335214	1.76556
ST6GAL1	W1	HPI4	well_2	F006	7	X/Y distribution	6.056017	5.840517	1.674592
ST6GAL1	W1	HPI4	well_3	F001	8	X/Y distribution	7.469386	6.457606	4.182298
ST6GAL1	W1	HPI4	well_3	F002	4	X/Y distribution	6.335546	6.335546	0.523079
ST6GAL1	W1	HPI4	well_3	F003	19	X/Y distribution	6.541533	7.043429	1.980947
ST6GAL1	W1	HPI4	well_3	F004	31	X/Y distribution	6.212276	6.191771	1.543846
ST6GAL1	W1	HPI4	well_3	F005	9	X/Y distribution	6.906091	7.117054	0.833635
ST6GAL1	W1	HPI4	well_3	F006	18	X/Y distribution	6.442107	6.404484	1.469936
ST6GAL1	W1	HPI4	well_4	F001	65	X/Y distribution	7.264719	6.818509	1.478097
ST6GAL1	W1	HPI4	well_4	F002	28	X/Y distribution	6.560472	6.681462	1.850907
ST6GAL1	W1	HPI4	well_4	F003	3	X/Y distribution	7.265933	7.265933	NA
ST6GAL1	W1	HPI4	well_4	F004	29	X/Y distribution	7.36487	7.210227	2.146092
ST6GAL1	W1	HPI4	well_4	F005	16	X/Y distribution	6.813486	6.259097	1.798753
ST6GAL1	W1	HPI4	well_4	F006	14	X/Y distribution	5.564332	5.470273	2.198476
ST6GAL1	W1	HPI4	well_5	F001	7	X/Y distribution	9.528428	9.378572	2.729632
ST6GAL1	W1	HPI4	well_5	F002	18	X/Y distribution	6.604204	6.528774	1.399714

ST6GAL1	W1	HPI4	well_5	F003	31	X/Y distribution	6.822279	7.282109	1.673269
ST6GAL1	W1	HPI4	well_5	F004	10	X/Y distribution	5.063061	5.787096	2.414974
ST6GAL1	W1	HPI4	well_5	F005	26	X/Y distribution	5.766224	5.960473	1.431558
ST6GAL1	W1	HPI4	well_5	F006	25	X/Y distribution	6.382197	6.611951	1.530331
ST6GAL1	W2	HPI4	well_1	F001	3	X/Y distribution	8.078088	8.078088	NA
ST6GAL1	W2	HPI4	well_1	F002	7	X/Y distribution	7.016732	7.637671	1.525643
ST6GAL1	W2	HPI4	well_1	F003	5	X/Y distribution	4.013513	4.116236	0.268096
ST6GAL1	W2	HPI4	well_1	F004	25	X/Y distribution	6.79057	7.009744	1.655055
ST6GAL1	W2	HPI4	well_1	F005	30	X/Y distribution	5.713072	5.664748	1.917982
ST6GAL1	W2	HPI4	well_1	F006	40	X/Y distribution	5.57637	5.429045	1.831977
ST6GAL1	W2	HPI4	well_2	F001	8	X/Y distribution	6.675343	6.85911	1.723349
ST6GAL1	W2	HPI4	well_2	F002	8	X/Y distribution	7.33054	7.629466	1.913558
ST6GAL1	W2	HPI4	well_2	F003	20	X/Y distribution	6.947573	7.206873	1.648674
ST6GAL1	W2	HPI4	well_2	F004	10	X/Y distribution	6.27031	5.534523	2.226141
ST6GAL1	W2	HPI4	well_2	F005	12	X/Y distribution	7.854203	7.754908	2.110456
ST6GAL1	W2	HPI4	well_2	F006	13	X/Y distribution	6.655692	7.381902	2.468131
ST6GAL1	W2	HPI4	well_3	F001	20	X/Y distribution	7.680166	7.533964	1.034093
ST6GAL1	W2	HPI4	well_3	F002	5	X/Y distribution	6.734527	6.166407	2.672908
ST6GAL1	W2	HPI4	well_3	F003	15	X/Y distribution	4.989254	5.121591	1.807713
ST6GAL1	W2	HPI4	well_3	F005	49	X/Y distribution	7.354744	7.047549	1.20912
ST6GAL1	W2	HPI4	well_3	F006	12	X/Y distribution	7.155307	6.850636	2.673842
ST6GAL1	W2	HPI4	well_4	F001	5	X/Y distribution	7.596127	6.581027	1.864257
ST6GAL1	W2	HPI4	well_4	F002	9	X/Y distribution	7.947833	7.070367	2.138957
ST6GAL1	W2	HPI4	well_4	F003	28	X/Y distribution	6.082028	6.400705	1.327266
ST6GAL1	W2	HPI4	well_4	F004	10	X/Y distribution	6.814449	6.816433	1.218707
ST6GAL1	W2	HPI4	well_4	F005	13	X/Y distribution	8.838066	8.915079	1.136777
ST6GAL1	W2	HPI4	well_4	F006	17	X/Y distribution	6.246211	6.542303	1.344765
ST6GAL1	W2	HPI4	well_5	F001	7	X/Y distribution	8.500639	8.075841	1.716784
ST6GAL1	W2	HPI4	well_5	F002	10	X/Y distribution	6.69977	7.204835	1.052438
ST6GAL1	W2	HPI4	well_5	F003	13	X/Y distribution	8.283554	8.336331	2.200009
ST6GAL1	W2	HPI4	well_5	F004	15	X/Y distribution	8.246451	8.138271	1.622384
ST6GAL1	W2	HPI4	well_5	F005	10	X/Y distribution	6.508743	6.20455	2.098048
ST6GAL1	W2	HPI4	well_5	F006	43	X/Y distribution	6.310025	6.502809	2.019134
ST6GAL1	W3	HPI4	well_1	F001	1	X/Y distribution	3.075745	3.075745	NA
ST6GAL1	W3	HPI4	well_1	F002	14	X/Y distribution	6.988391	7.1113	1.748915
ST6GAL1	W3	HPI4	well_1	F003	23	X/Y distribution	6.706435	6.489399	1.651116
ST6GAL1	W3	HPI4	well_1	F004	94	X/Y distribution	5.570107	5.666916	1.154505
ST6GAL1	W3	HPI4	well_1	F005	76	X/Y distribution	6.859286	7.027368	1.365884
ST6GAL1	W3	HPI4	well_1	F006	32	X/Y distribution	7.215744	6.793487	2.019149
ST6GAL1	W3	HPI4	well_2	F001	1	X/Y distribution	5.157752	5.157752	NA
ST6GAL1	W3	HPI4	well_2	F003	6	X/Y distribution	8.558922	8.37	0.615202
ST6GAL1	W3	HPI4	well_2	F004	5	X/Y distribution	8.297865	8.44556	0.714522
ST6GAL1	W3	HPI4	well_2	F005	11	X/Y distribution	6.557528	6.596575	1.372983
ST6GAL1	W3	HPI4	well_2	F006	15	X/Y distribution	7.079747	7.234173	1.953632
ST6GAL1	W3	HPI4	well_3	F001	7	X/Y distribution	6.745914	6.639639	2.066783
ST6GAL1	W3	HPI4	well_3	F003	3	X/Y distribution	7.615505	7.615505	NA
ST6GAL1	W3	HPI4	well_3	F005	11	X/Y distribution	6.295644	6.008699	1.038252
ST6GAL1	W3	HPI4	well_3	F006	5	X/Y distribution	8.382976	8.32263	1.996579
ST6GAL1	W3	HPI4	well_4	F001	6	X/Y distribution	7.369656	7.386421	0.838412

ST6GAL1	W3	HPI4	well_4	F002	5	X/Y distribution	8.569467	8.110983	0.948828
ST6GAL1	W3	HPI4	well_4	F003	6	X/Y distribution	6.47232	6.640998	0.459357
ST6GAL1	W3	HPI4	well_4	F004	5	X/Y distribution	5.166573	4.363272	1.46424
ST6GAL1	W3	HPI4	well_4	F005	29	X/Y distribution	6.848704	7.086862	1.607929
ST6GAL1	W3	HPI4	well_4	F006	5	X/Y distribution	7.706141	7.263619	1.34813
ST6GAL1	W3	HPI4	well_5	F001	8	X/Y distribution	5.829271	6.431434	1.789148
ST6GAL1	W3	HPI4	well_5	F002	21	X/Y distribution	5.604457	5.85785	1.298801
ST6GAL1	W3	HPI4	well_5	F003	5	X/Y distribution	5.62584	5.660101	0.486529
ST6GAL1	W3	HPI4	well_5	F004	7	X/Y distribution	6.349005	6.140852	0.619877
ST6GAL1	W3	HPI4	well_5	F005	27	X/Y distribution	7.225996	7.16966	1.37288
ST6GAL1	W3	HPI4	well_5	F006	83	X/Y distribution	7.16935	7.282462	1.339917
ST6GAL1	W4	HPI4	well_1	F001	3	X/Y distribution	6.652998	6.652998	NA
ST6GAL1	W4	HPI4	well_1	F002	4	X/Y distribution	5.245686	5.245686	0.658463
ST6GAL1	W4	HPI4	well_1	F003	3	X/Y distribution	6.139926	6.139926	NA
ST6GAL1	W4	HPI4	well_1	F004	9	X/Y distribution	6.402715	6.150522	1.594021
ST6GAL1	W4	HPI4	well_1	F005	11	X/Y distribution	4.8111	4.879189	1.194534
ST6GAL1	W4	HPI4	well_1	F006	19	X/Y distribution	6.112371	6.679011	1.674701
ST6GAL1	W4	HPI4	well_2	F004	18	X/Y distribution	6.810348	6.876797	1.798202
ST6GAL1	W4	HPI4	well_2	F006	13	X/Y distribution	7.074962	6.995202	1.352001
ST6GAL1	W4	HPI4	well_3	F002	9	X/Y distribution	4.659907	4.233721	1.513588
ST6GAL1	W4	HPI4	well_3	F004	6	X/Y distribution	5.269634	4.902358	1.359572
ST6GAL1	W4	HPI4	well_3	F005	1	X/Y distribution	4.003586	4.003586	NA
ST6GAL1	W4	HPI4	well_4	F001	39	X/Y distribution	6.299632	6.42779	1.89858
ST6GAL1	W4	HPI4	well_4	F004	3	X/Y distribution	7.176772	7.176772	NA
ST6GAL1	W4	HPI4	well_4	F006	23	X/Y distribution	6.090955	6.173216	1.261717
ST6GAL1	W4	HPI4	well_5	F001	6	X/Y distribution	4.299464	4.720843	1.212114
ST6GAL1	W4	HPI4	well_5	F003	9	X/Y distribution	4.43173	4.144798	0.887508
ST6GAL1	W4	HPI4	well_5	F004	16	X/Y distribution	7.160842	7.598097	1.580806
ST6GAL1	W4	HPI4	well_5	F005	13	X/Y distribution	8.64749	8.398638	1.575597
ST6GAL1	W4	HPI4	well_5	F006	5	X/Y distribution	5.549945	5.454645	1.407213
ST6GAL1	W1	PGE2	well_1	F001	8	X/Y distribution	6.837966	6.219948	1.88976
ST6GAL1	W1	PGE2	well_1	F002	8	X/Y distribution	5.44188	5.879248	1.440729
ST6GAL1	W1	PGE2	well_1	F003	9	X/Y distribution	5.874194	6.86189	1.468954
ST6GAL1	W1	PGE2	well_1	F004	12	X/Y distribution	4.095408	4.290446	2.084862
ST6GAL1	W1	PGE2	well_1	F005	23	X/Y distribution	6.77204	6.022646	1.966419
ST6GAL1	W1	PGE2	well_1	F006	33	X/Y distribution	5.872275	6.117727	1.608853
ST6GAL1	W1	PGE2	well_2	F001	8	X/Y distribution	5.496599	4.742551	2.125387
ST6GAL1	W1	PGE2	well_2	F002	11	X/Y distribution	6.427128	6.477544	1.743042
ST6GAL1	W1	PGE2	well_2	F003	20	X/Y distribution	5.917772	5.410134	2.763839
ST6GAL1	W1	PGE2	well_2	F004	4	X/Y distribution	8.093586	8.093586	2.356486
ST6GAL1	W1	PGE2	well_2	F005	19	X/Y distribution	5.647399	5.930419	1.82092
ST6GAL1	W1	PGE2	well_2	F006	14	X/Y distribution	6.974073	5.999446	2.277903
ST6GAL1	W1	PGE2	well_3	F001	13	X/Y distribution	6.09343	6.5013	1.666961
ST6GAL1	W1	PGE2	well_3	F002	5	X/Y distribution	5.617874	5.531876	0.361634
ST6GAL1	W1	PGE2	well_3	F003	1	X/Y distribution	9.978987	9.978987	NA
ST6GAL1	W1	PGE2	well_3	F004	19	X/Y distribution	8.726051	8.981813	2.526398
ST6GAL1	W1	PGE2	well_3	F005	18	X/Y distribution	6.486369	7.230962	2.102522
ST6GAL1	W1	PGE2	well_3	F006	8	X/Y distribution	7.752446	8.416428	1.398803
ST6GAL1	W1	PGE2	well_4	F001	11	X/Y distribution	4.412058	4.088866	1.59128

ST6GAL1	W1	PGE2	well_4	F002	6	X/Y distribution	6.742873	7.178084	1.891528
ST6GAL1	W1	PGE2	well_4	F003	7	X/Y distribution	6.340814	5.911008	2.084109
ST6GAL1	W1	PGE2	well_4	F004	1	X/Y distribution	9.71013	9.71013	NA
ST6GAL1	W1	PGE2	well_4	F005	88	X/Y distribution	5.449625	5.27541	1.648844
ST6GAL1	W1	PGE2	well_4	F006	7	X/Y distribution	6.180542	5.639435	2.471884
ST6GAL1	W1	PGE2	well_5	F001	7	X/Y distribution	4.971958	4.770821	0.731404
ST6GAL1	W1	PGE2	well_5	F002	6	X/Y distribution	6.919054	7.411442	2.727095
ST6GAL1	W1	PGE2	well_5	F004	6	X/Y distribution	4.975357	4.893559	0.321
ST6GAL1	W1	PGE2	well_5	F005	8	X/Y distribution	5.600783	5.871635	0.645934
ST6GAL1	W1	PGE2	well_5	F006	1	X/Y distribution	9.827703	9.827703	NA
ST6GAL1	W2	PGE2	well_1	F001	111	X/Y distribution	6.161595	6.202651	1.85149
ST6GAL1	W2	PGE2	well_1	F002	152	X/Y distribution	5.377191	5.151992	1.60431
ST6GAL1	W2	PGE2	well_1	F003	160	X/Y distribution	6.176313	6.295436	1.072593
ST6GAL1	W2	PGE2	well_1	F004	234	X/Y distribution	5.488481	5.3705	1.702951
ST6GAL1	W2	PGE2	well_1	F005	148	X/Y distribution	5.837421	5.817002	1.346711
ST6GAL1	W2	PGE2	well_1	F006	184	X/Y distribution	5.367294	5.451799	1.298316
ST6GAL1	W2	PGE2	well_2	F001	64	X/Y distribution	5.0705	5.019228	1.199921
ST6GAL1	W2	PGE2	well_2	F002	175	X/Y distribution	6.287765	6.184333	1.368118
ST6GAL1	W2	PGE2	well_2	F003	338	X/Y distribution	5.91355	5.811497	1.233089
ST6GAL1	W2	PGE2	well_2	F004	145	X/Y distribution	5.040581	4.621615	1.506481
ST6GAL1	W2	PGE2	well_2	F005	88	X/Y distribution	5.716185	5.529254	1.371457
ST6GAL1	W2	PGE2	well_2	F006	268	X/Y distribution	4.557548	4.384103	1.22046
ST6GAL1	W2	PGE2	well_3	F001	42	X/Y distribution	4.488906	4.567036	0.957591
ST6GAL1	W2	PGE2	well_3	F002	220	X/Y distribution	5.480374	5.422122	1.513102
ST6GAL1	W2	PGE2	well_3	F003	49	X/Y distribution	5.015666	4.953097	1.448014
ST6GAL1	W2	PGE2	well_3	F004	77	X/Y distribution	4.921852	4.846196	1.399086
ST6GAL1	W2	PGE2	well_3	F005	198	X/Y distribution	4.521725	4.55419	1.278232
ST6GAL1	W2	PGE2	well_3	F006	276	X/Y distribution	5.677299	5.569691	1.308272
ST6GAL1	W2	PGE2	well_4	F001	44	X/Y distribution	4.70497	4.902821	1.311582
ST6GAL1	W2	PGE2	well_4	F002	291	X/Y distribution	5.266421	5.261944	1.067997
ST6GAL1	W2	PGE2	well_4	F003	71	X/Y distribution	4.867337	4.792134	1.609146
ST6GAL1	W2	PGE2	well_4	F004	72	X/Y distribution	4.87581	5.029371	1.482371
ST6GAL1	W2	PGE2	well_4	F005	39	X/Y distribution	5.067496	5.108105	1.466888
ST6GAL1	W2	PGE2	well_4	F006	53	X/Y distribution	4.0517	3.573622	2.078875
ST6GAL1	W2	PGE2	well_5	F001	239	X/Y distribution	5.833145	5.851461	1.515393
ST6GAL1	W2	PGE2	well_5	F002	250	X/Y distribution	5.673529	5.645863	1.351197
ST6GAL1	W2	PGE2	well_5	F003	158	X/Y distribution	5.052429	5.072598	1.552842
ST6GAL1	W2	PGE2	well_5	F004	13	X/Y distribution	4.001205	3.798825	1.503022
ST6GAL1	W2	PGE2	well_5	F005	11	X/Y distribution	4.070789	4.316131	1.300929
ST6GAL1	W2	PGE2	well_5	F006	117	X/Y distribution	5.427525	5.27438	1.753703
ST6GAL1	W3	PGE2	well_1	F001	195	X/Y distribution	7.034542	7.02396	1.36011
ST6GAL1	W3	PGE2	well_1	F002	350	X/Y distribution	5.358758	5.278045	0.990894
ST6GAL1	W3	PGE2	well_1	F003	293	X/Y distribution	5.894874	5.807671	0.96997
ST6GAL1	W3	PGE2	well_1	F004	136	X/Y distribution	6.669855	6.553837	1.178725
ST6GAL1	W3	PGE2	well_1	F005	277	X/Y distribution	6.502294	6.40919	1.345501
ST6GAL1	W3	PGE2	well_1	F006	339	X/Y distribution	5.48776	5.316045	1.01707
ST6GAL1	W3	PGE2	well_2	F001	180	X/Y distribution	6.959069	6.856002	1.607485
ST6GAL1	W3	PGE2	well_2	F002	60	X/Y distribution	6.522906	6.784395	1.197828
ST6GAL1	W3	PGE2	well_2	F003	321	X/Y distribution	5.412814	5.338973	1.013399

ST6GAL1	W3	PGE2	well_2	F004	295	X/Y distribution	6.101239	6.023275	1.422196
ST6GAL1	W3	PGE2	well_2	F005	179	X/Y distribution	7.691711	7.716636	1.564934
ST6GAL1	W3	PGE2	well_2	F006	324	X/Y distribution	6.031855	5.960623	1.124118
ST6GAL1	W3	PGE2	well_3	F001	186	X/Y distribution	7.028171	6.775481	1.663658
ST6GAL1	W3	PGE2	well_3	F002	183	X/Y distribution	6.235557	5.936188	1.55492
ST6GAL1	W3	PGE2	well_3	F003	239	X/Y distribution	6.006764	5.96236	1.355241
ST6GAL1	W3	PGE2	well_3	F004	152	X/Y distribution	6.56808	6.415369	1.608858
ST6GAL1	W3	PGE2	well_3	F005	152	X/Y distribution	5.246534	5.07435	1.47927
ST6GAL1	W3	PGE2	well_3	F006	233	X/Y distribution	5.896281	5.758157	1.489793
ST6GAL1	W3	PGE2	well_4	F001	197	X/Y distribution	6.05615	5.868667	1.678187
ST6GAL1	W3	PGE2	well_4	F002	218	X/Y distribution	5.568864	5.343827	1.580367
ST6GAL1	W3	PGE2	well_4	F003	215	X/Y distribution	5.955658	6.004174	1.536426
ST6GAL1	W3	PGE2	well_4	F004	205	X/Y distribution	6.709701	6.487599	1.962059
ST6GAL1	W3	PGE2	well_4	F005	222	X/Y distribution	6.096759	6.039778	1.262664
ST6GAL1	W3	PGE2	well_4	F006	214	X/Y distribution	5.236152	5.180775	1.418323
ST6GAL1	W3	PGE2	well_5	F001	57	X/Y distribution	5.389036	5.688642	1.138312
ST6GAL1	W3	PGE2	well_5	F002	70	X/Y distribution	6.589437	6.51822	1.165762
ST6GAL1	W3	PGE2	well_5	F003	237	X/Y distribution	6.215273	6.058914	1.658032
ST6GAL1	W3	PGE2	well_5	F004	265	X/Y distribution	5.938278	5.872068	1.587801
ST6GAL1	W3	PGE2	well_5	F005	166	X/Y distribution	6.778808	6.601756	1.598442
ST6GAL1	W3	PGE2	well_5	F006	38	X/Y distribution	4.862275	4.943202	0.971256
ST6GAL1	W4	PGE2	well_1	F001	67	X/Y distribution	6.14213	6.116443	1.523215
ST6GAL1	W4	PGE2	well_1	F002	46	X/Y distribution	5.825006	5.901595	0.726249
ST6GAL1	W4	PGE2	well_1	F003	193	X/Y distribution	5.846347	5.735298	1.195375
ST6GAL1	W4	PGE2	well_1	F004	130	X/Y distribution	5.409787	5.396974	0.979548
ST6GAL1	W4	PGE2	well_1	F005	171	X/Y distribution	6.336017	6.137208	1.712601
ST6GAL1	W4	PGE2	well_1	F006	173	X/Y distribution	5.663282	5.549472	1.458619
ST6GAL1	W4	PGE2	well_2	F001	204	X/Y distribution	5.739017	5.671185	1.48208
ST6GAL1	W4	PGE2	well_2	F002	61	X/Y distribution	5.567161	5.410081	1.0433
ST6GAL1	W4	PGE2	well_2	F003	71	X/Y distribution	5.5253	5.369375	1.097955
ST6GAL1	W4	PGE2	well_2	F004	97	X/Y distribution	7.096315	6.976491	1.510569
ST6GAL1	W4	PGE2	well_2	F005	63	X/Y distribution	6.322324	6.429767	1.022423
ST6GAL1	W4	PGE2	well_2	F006	135	X/Y distribution	6.200134	6.551292	1.393598
ST6GAL1	W4	PGE2	well_3	F001	193	X/Y distribution	4.994345	5.056494	0.99705
ST6GAL1	W4	PGE2	well_3	F002	13	X/Y distribution	6.094047	5.808104	2.709797
ST6GAL1	W4	PGE2	well_3	F003	13	X/Y distribution	4.907464	4.84246	1.708761
ST6GAL1	W4	PGE2	well_3	F004	224	X/Y distribution	5.912628	5.822508	1.276949
ST6GAL1	W4	PGE2	well_3	F005	182	X/Y distribution	6.722996	6.499048	1.632311
ST6GAL1	W4	PGE2	well_3	F006	42	X/Y distribution	4.939473	4.79945	1.141505
ST6GAL1	W4	PGE2	well_4	F001	166	X/Y distribution	5.983108	5.714807	1.225706
ST6GAL1	W4	PGE2	well_4	F002	91	X/Y distribution	5.815969	5.696951	1.592513
ST6GAL1	W4	PGE2	well_4	F003	232	X/Y distribution	6.386693	6.36369	1.329773
ST6GAL1	W4	PGE2	well_4	F004	173	X/Y distribution	6.900482	6.785957	1.488238
ST6GAL1	W4	PGE2	well_4	F005	263	X/Y distribution	5.426179	5.386429	1.086167
ST6GAL1	W4	PGE2	well_4	F006	32	X/Y distribution	5.389447	5.31593	1.233999
ST6GAL1	W4	PGE2	well_5	F001	57	X/Y distribution	5.513997	5.65933	1.4976
ST6GAL1	W4	PGE2	well_5	F002	96	X/Y distribution	6.162569	6.009812	1.748584
ST6GAL1	W4	PGE2	well_5	F004	95	X/Y distribution	6.230573	6.12735	1.515425
ST6GAL1	W4	PGE2	well_5	F006	4	X/Y distribution	5.892721	5.892721	0.066442

ST6GAL1	W1	HPI4	well_1	F001	30	Z distribution	-0.20391	-0.23547	0.495652
ST6GAL1	W1	HPI4	well_1	F002	3	Z distribution	-0.27896	-0.27896	NA
ST6GAL1	W1	HPI4	well_1	F003	7	Z distribution	-0.7516	-0.72347	0.172684
ST6GAL1	W1	HPI4	well_1	F004	25	Z distribution	-0.29435	-0.29243	0.275904
ST6GAL1	W1	HPI4	well_1	F005	22	Z distribution	-0.65869	-0.63731	0.248463
ST6GAL1	W1	HPI4	well_1	F006	20	Z distribution	-0.47092	-0.4682	0.421523
ST6GAL1	W1	HPI4	well_2	F001	8	Z distribution	-0.63619	-0.59438	0.311474
ST6GAL1	W1	HPI4	well_2	F002	35	Z distribution	0.387846	0.481184	0.479808
ST6GAL1	W1	HPI4	well_2	F003	18	Z distribution	-0.1625	-0.05628	0.390354
ST6GAL1	W1	HPI4	well_2	F004	41	Z distribution	-0.21609	-0.23798	0.455847
ST6GAL1	W1	HPI4	well_2	F005	9	Z distribution	-0.20517	-0.15622	0.273074
ST6GAL1	W1	HPI4	well_2	F006	7	Z distribution	-0.86755	-0.64448	0.624245
ST6GAL1	W1	HPI4	well_3	F001	8	Z distribution	-0.29469	-0.29441	0.459006
ST6GAL1	W1	HPI4	well_3	F002	4	Z distribution	-0.58696	-0.58696	0.081448
ST6GAL1	W1	HPI4	well_3	F003	19	Z distribution	-0.74034	-0.68813	0.417129
ST6GAL1	W1	HPI4	well_3	F004	31	Z distribution	-0.49003	-0.4301	0.480932
ST6GAL1	W1	HPI4	well_3	F005	9	Z distribution	-0.57884	-0.49571	0.384409
ST6GAL1	W1	HPI4	well_3	F006	18	Z distribution	0.009046	-0.10307	0.376726
ST6GAL1	W1	HPI4	well_4	F001	65	Z distribution	-0.03634	-0.00336	0.382971
ST6GAL1	W1	HPI4	well_4	F002	28	Z distribution	-0.41465	-0.52631	0.587569
ST6GAL1	W1	HPI4	well_4	F003	3	Z distribution	-0.50851	-0.50851	NA
ST6GAL1	W1	HPI4	well_4	F004	29	Z distribution	-0.39653	-0.38276	0.328308
ST6GAL1	W1	HPI4	well_4	F005	16	Z distribution	-0.48575	-0.52384	0.330393
ST6GAL1	W1	HPI4	well_4	F006	14	Z distribution	-0.87318	-0.94518	0.260436
ST6GAL1	W1	HPI4	well_5	F001	7	Z distribution	0.571507	0.597305	0.298782
ST6GAL1	W1	HPI4	well_5	F002	18	Z distribution	-0.33074	-0.3641	0.343991
ST6GAL1	W1	HPI4	well_5	F003	31	Z distribution	-0.17772	-0.0943	0.446198
ST6GAL1	W1	HPI4	well_5	F004	10	Z distribution	-0.2612	-0.3596	0.382099
ST6GAL1	W1	HPI4	well_5	F005	26	Z distribution	-0.51147	-0.4247	0.468153
ST6GAL1	W1	HPI4	well_5	F006	25	Z distribution	-0.26435	-0.3088	0.379018
ST6GAL1	W2	HPI4	well_1	F001	3	Z distribution	-0.34696	-0.34696	NA
ST6GAL1	W2	HPI4	well_1	F002	7	Z distribution	-0.36432	-0.38034	0.154136
ST6GAL1	W2	HPI4	well_1	F003	5	Z distribution	-0.89103	-0.86803	0.435756
ST6GAL1	W2	HPI4	well_1	F004	25	Z distribution	-0.37271	-0.41766	0.331751
ST6GAL1	W2	HPI4	well_1	F005	30	Z distribution	-0.50994	-0.58093	0.303929
ST6GAL1	W2	HPI4	well_1	F006	40	Z distribution	-0.49649	-0.4707	0.426199
ST6GAL1	W2	HPI4	well_2	F001	8	Z distribution	-0.28256	-0.42971	0.411664
ST6GAL1	W2	HPI4	well_2	F002	8	Z distribution	-0.43636	-0.47276	0.107759
ST6GAL1	W2	HPI4	well_2	F003	20	Z distribution	-0.50991	-0.41833	0.289193
ST6GAL1	W2	HPI4	well_2	F004	10	Z distribution	-0.66759	-0.59176	0.369315
ST6GAL1	W2	HPI4	well_2	F005	12	Z distribution	-0.37218	-0.38736	0.465758
ST6GAL1	W2	HPI4	well_2	F006	13	Z distribution	-0.39418	-0.41292	0.241533
ST6GAL1	W2	HPI4	well_3	F001	20	Z distribution	-0.43674	-0.34343	0.413012
ST6GAL1	W2	HPI4	well_3	F002	5	Z distribution	-0.59378	-0.52443	0.143586
ST6GAL1	W2	HPI4	well_3	F003	15	Z distribution	-0.41565	-0.3857	0.250816
ST6GAL1	W2	HPI4	well_3	F005	49	Z distribution	-0.16722	-0.17116	0.36332
ST6GAL1	W2	HPI4	well_3	F006	12	Z distribution	-0.76237	-0.69388	0.316019
ST6GAL1	W2	HPI4	well_4	F001	5	Z distribution	-0.42139	-0.34193	0.225387
ST6GAL1	W2	HPI4	well_4	F002	9	Z distribution	-0.47022	-0.44537	0.091599

ST6GAL1	W2	HPI4	well_4	F003	28	Z distribution	-0.78196	-0.90496	0.33133
ST6GAL1	W2	HPI4	well_4	F004	10	Z distribution	-0.4218	-0.35841	0.291571
ST6GAL1	W2	HPI4	well_4	F005	13	Z distribution	-0.3724	-0.36145	0.122221
ST6GAL1	W2	HPI4	well_4	F006	17	Z distribution	-0.31013	-0.12985	0.410239
ST6GAL1	W2	HPI4	well_5	F001	7	Z distribution	-0.61362	-0.53945	0.227769
ST6GAL1	W2	HPI4	well_5	F002	10	Z distribution	-0.70151	-0.63762	0.306618
ST6GAL1	W2	HPI4	well_5	F003	13	Z distribution	-0.52333	-0.49142	0.254028
ST6GAL1	W2	HPI4	well_5	F004	15	Z distribution	-0.34781	-0.32091	0.302412
ST6GAL1	W2	HPI4	well_5	F005	10	Z distribution	-0.1264	-0.09522	0.154903
ST6GAL1	W2	HPI4	well_5	F006	43	Z distribution	-0.69464	-0.69173	0.28998
ST6GAL1	W3	HPI4	well_1	F001	1	Z distribution	-1.93795	-1.93795	NA
ST6GAL1	W3	HPI4	well_1	F002	14	Z distribution	-0.57597	-0.47153	0.257498
ST6GAL1	W3	HPI4	well_1	F003	23	Z distribution	-0.52011	-0.51301	0.312002
ST6GAL1	W3	HPI4	well_1	F004	94	Z distribution	-0.39038	-0.31659	0.410218
ST6GAL1	W3	HPI4	well_1	F005	76	Z distribution	-0.52482	-0.36492	0.504525
ST6GAL1	W3	HPI4	well_1	F006	32	Z distribution	-0.02191	-0.0499	0.323448
ST6GAL1	W3	HPI4	well_2	F001	1	Z distribution	-0.66469	-0.66469	NA
ST6GAL1	W3	HPI4	well_2	F003	6	Z distribution	-0.84756	-0.75556	0.255709
ST6GAL1	W3	HPI4	well_2	F004	5	Z distribution	-0.23637	-0.2167	0.06066
ST6GAL1	W3	HPI4	well_2	F005	11	Z distribution	-0.2274	-0.15489	0.166373
ST6GAL1	W3	HPI4	well_2	F006	15	Z distribution	-0.45587	-0.37913	0.491724
ST6GAL1	W3	HPI4	well_3	F001	7	Z distribution	-0.33001	-0.28643	0.150895
ST6GAL1	W3	HPI4	well_3	F003	3	Z distribution	-0.4811	-0.4811	NA
ST6GAL1	W3	HPI4	well_3	F005	11	Z distribution	-0.66592	-0.48237	0.450028
ST6GAL1	W3	HPI4	well_3	F006	5	Z distribution	-0.53036	-0.47621	0.168097
ST6GAL1	W3	HPI4	well_4	F001	6	Z distribution	-0.21393	-0.38197	0.487291
ST6GAL1	W3	HPI4	well_4	F002	5	Z distribution	-0.04171	-0.05256	0.742719
ST6GAL1	W3	HPI4	well_4	F003	6	Z distribution	-0.34467	-0.31927	0.291978
ST6GAL1	W3	HPI4	well_4	F004	5	Z distribution	-0.14011	-0.05547	0.16281
ST6GAL1	W3	HPI4	well_4	F005	29	Z distribution	-0.86594	-0.90414	0.293407
ST6GAL1	W3	HPI4	well_4	F006	5	Z distribution	-0.25208	-0.21321	0.168332
ST6GAL1	W3	HPI4	well_5	F001	8	Z distribution	-0.39428	-0.27931	0.318612
ST6GAL1	W3	HPI4	well_5	F002	21	Z distribution	-0.70306	-0.7113	0.330396
ST6GAL1	W3	HPI4	well_5	F003	5	Z distribution	-0.63092	-0.74451	0.260574
ST6GAL1	W3	HPI4	well_5	F004	7	Z distribution	-0.29249	-0.27702	0.107708
ST6GAL1	W3	HPI4	well_5	F005	27	Z distribution	-0.50478	-0.46819	0.329399
ST6GAL1	W3	HPI4	well_5	F006	83	Z distribution	-0.2499	-0.20511	0.31902
ST6GAL1	W4	HPI4	well_1	F001	3	Z distribution	-1.06826	-1.06826	NA
ST6GAL1	W4	HPI4	well_1	F002	4	Z distribution	-0.32224	-0.32224	0.260612
ST6GAL1	W4	HPI4	well_1	F003	3	Z distribution	-0.36563	-0.36563	NA
ST6GAL1	W4	HPI4	well_1	F004	9	Z distribution	-0.47885	-0.50709	0.381013
ST6GAL1	W4	HPI4	well_1	F005	11	Z distribution	-0.88894	-0.74385	0.466147
ST6GAL1	W4	HPI4	well_1	F006	19	Z distribution	-0.74368	-0.72978	0.431736
ST6GAL1	W4	HPI4	well_2	F004	18	Z distribution	-0.97256	-0.95583	0.482797
ST6GAL1	W4	HPI4	well_2	F006	13	Z distribution	-0.822	-0.85967	0.393341
ST6GAL1	W4	HPI4	well_3	F002	9	Z distribution	-0.55787	-0.56699	0.461353
ST6GAL1	W4	HPI4	well_3	F004	6	Z distribution	-0.72638	-0.69751	0.205067
ST6GAL1	W4	HPI4	well_3	F005	1	Z distribution	-0.61936	-0.61936	NA
ST6GAL1	W4	HPI4	well_4	F001	39	Z distribution	-0.82902	-0.67733	0.607227

ST6GAL1	W4	HPI4	well_4	F004	3	Z distribution	-0.74369	-0.74369	NA
ST6GAL1	W4	HPI4	well_4	F006	23	Z distribution	-0.68017	-0.66149	0.32631
ST6GAL1	W4	HPI4	well_5	F001	6	Z distribution	-0.08051	-0.0648	0.558511
ST6GAL1	W4	HPI4	well_5	F003	9	Z distribution	-0.15547	-0.16938	0.541259
ST6GAL1	W4	HPI4	well_5	F004	16	Z distribution	-0.59105	-0.60136	0.241693
ST6GAL1	W4	HPI4	well_5	F005	13	Z distribution	-0.86969	-0.84795	0.568255
ST6GAL1	W4	HPI4	well_5	F006	5	Z distribution	-1.12517	-1.00809	0.450771
ST6GAL1	W1	PGE2	well_1	F001	8	Z distribution	-0.33085	-0.3308	0.130775
ST6GAL1	W1	PGE2	well_1	F002	8	Z distribution	-0.57229	-0.56911	0.285468
ST6GAL1	W1	PGE2	well_1	F003	9	Z distribution	-0.6494	-0.64257	0.429608
ST6GAL1	W1	PGE2	well_1	F004	12	Z distribution	-0.48079	-0.52544	0.251811
ST6GAL1	W1	PGE2	well_1	F005	23	Z distribution	-0.55156	-0.60827	0.308517
ST6GAL1	W1	PGE2	well_1	F006	33	Z distribution	-0.31903	-0.31787	0.464379
ST6GAL1	W1	PGE2	well_2	F001	8	Z distribution	-0.40995	-0.33432	0.214989
ST6GAL1	W1	PGE2	well_2	F002	11	Z distribution	-0.29006	-0.2224	0.374761
ST6GAL1	W1	PGE2	well_2	F003	20	Z distribution	-0.59344	-0.6382	0.443209
ST6GAL1	W1	PGE2	well_2	F004	4	Z distribution	-0.61798	-0.61798	0.262754
ST6GAL1	W1	PGE2	well_2	F005	19	Z distribution	-0.62965	-0.49995	0.324668
ST6GAL1	W1	PGE2	well_2	F006	14	Z distribution	-0.57587	-0.60175	0.35615
ST6GAL1	W1	PGE2	well_3	F001	13	Z distribution	-0.30212	-0.19051	0.298964
ST6GAL1	W1	PGE2	well_3	F002	5	Z distribution	-1.1244	-0.96444	0.310308
ST6GAL1	W1	PGE2	well_3	F003	1	Z distribution	-1.14824	-1.14824	NA
ST6GAL1	W1	PGE2	well_3	F004	19	Z distribution	-0.25901	-0.29929	0.479948
ST6GAL1	W1	PGE2	well_3	F005	18	Z distribution	-0.51211	-0.48775	0.265601
ST6GAL1	W1	PGE2	well_3	F006	8	Z distribution	-0.45994	-0.48966	0.207108
ST6GAL1	W1	PGE2	well_4	F001	11	Z distribution	-0.36228	-0.34462	0.165503
ST6GAL1	W1	PGE2	well_4	F002	6	Z distribution	-0.56258	-0.58371	0.138394
ST6GAL1	W1	PGE2	well_4	F003	7	Z distribution	-0.60433	-0.76973	0.604766
ST6GAL1	W1	PGE2	well_4	F004	1	Z distribution	-0.25864	-0.25864	NA
ST6GAL1	W1	PGE2	well_4	F005	88	Z distribution	-0.32093	-0.34729	0.52118
ST6GAL1	W1	PGE2	well_4	F006	7	Z distribution	-0.24683	-0.20692	0.171628
ST6GAL1	W1	PGE2	well_5	F001	7	Z distribution	-0.36153	-0.37669	0.060263
ST6GAL1	W1	PGE2	well_5	F002	6	Z distribution	-0.3283	-0.48109	0.619786
ST6GAL1	W1	PGE2	well_5	F004	6	Z distribution	-0.37679	-0.29302	0.179186
ST6GAL1	W1	PGE2	well_5	F005	8	Z distribution	-0.82878	-0.7262	0.225468
ST6GAL1	W1	PGE2	well_5	F006	1	Z distribution	-0.32247	-0.32247	NA
ST6GAL1	W2	PGE2	well_1	F001	111	Z distribution	0.202958	0.185178	0.471264
ST6GAL1	W2	PGE2	well_1	F002	152	Z distribution	-0.27526	-0.21512	0.739142
ST6GAL1	W2	PGE2	well_1	F003	160	Z distribution	-0.50672	-0.47834	0.417143
ST6GAL1	W2	PGE2	well_1	F004	234	Z distribution	0.312759	0.346058	0.610233
ST6GAL1	W2	PGE2	well_1	F005	148	Z distribution	-0.03185	-0.07554	0.386869
ST6GAL1	W2	PGE2	well_1	F006	184	Z distribution	-0.44524	-0.38703	0.560781
ST6GAL1	W2	PGE2	well_2	F001	64	Z distribution	-0.37872	-0.43771	0.389057
ST6GAL1	W2	PGE2	well_2	F002	175	Z distribution	-0.18515	-0.11674	0.50632
ST6GAL1	W2	PGE2	well_2	F003	338	Z distribution	-0.01727	0.0241	0.572331
ST6GAL1	W2	PGE2	well_2	F004	145	Z distribution	0.029645	0.061173	0.468819
ST6GAL1	W2	PGE2	well_2	F005	88	Z distribution	-0.18606	-0.16003	0.444106
ST6GAL1	W2	PGE2	well_2	F006	268	Z distribution	0.265531	0.327757	0.66921
ST6GAL1	W2	PGE2	well_3	F001	42	Z distribution	-0.60222	-0.54065	0.314905

ST6GAL1	W2	PGE2	well_3	F002	220	Z distribution	-0.05951	-0.07435	0.678705
ST6GAL1	W2	PGE2	well_3	F003	49	Z distribution	-0.19846	-0.25939	0.62169
ST6GAL1	W2	PGE2	well_3	F004	77	Z distribution	-0.2486	-0.24943	0.514125
ST6GAL1	W2	PGE2	well_3	F005	198	Z distribution	-0.58051	-0.63726	0.525967
ST6GAL1	W2	PGE2	well_3	F006	276	Z distribution	0.060733	0.024152	0.546322
ST6GAL1	W2	PGE2	well_4	F001	44	Z distribution	-0.18674	-0.28256	0.450641
ST6GAL1	W2	PGE2	well_4	F002	291	Z distribution	-0.32815	-0.32401	0.588606
ST6GAL1	W2	PGE2	well_4	F003	71	Z distribution	-0.35762	-0.25355	0.444138
ST6GAL1	W2	PGE2	well_4	F004	72	Z distribution	-0.80145	-0.63357	0.793472
ST6GAL1	W2	PGE2	well_4	F005	39	Z distribution	-0.22047	-0.23339	0.373217
ST6GAL1	W2	PGE2	well_4	F006	53	Z distribution	-0.25936	-0.3121	0.558982
ST6GAL1	W2	PGE2	well_5	F001	239	Z distribution	0.404602	0.426837	0.566761
ST6GAL1	W2	PGE2	well_5	F002	250	Z distribution	-0.06423	-0.05595	0.538614
ST6GAL1	W2	PGE2	well_5	F003	158	Z distribution	0.3287	0.33175	0.689563
ST6GAL1	W2	PGE2	well_5	F004	13	Z distribution	-0.31738	-0.26849	0.184998
ST6GAL1	W2	PGE2	well_5	F005	11	Z distribution	-0.06832	-0.03641	0.334967
ST6GAL1	W2	PGE2	well_5	F006	117	Z distribution	0.124911	0.113527	0.513477
ST6GAL1	W3	PGE2	well_1	F001	195	Z distribution	0.086711	0.088502	0.381916
ST6GAL1	W3	PGE2	well_1	F002	350	Z distribution	-0.47902	-0.40729	0.582585
ST6GAL1	W3	PGE2	well_1	F003	293	Z distribution	-0.68873	-0.68576	0.546032
ST6GAL1	W3	PGE2	well_1	F004	136	Z distribution	0.066373	0.025783	0.332339
ST6GAL1	W3	PGE2	well_1	F005	277	Z distribution	0.271195	0.287991	0.434119
ST6GAL1	W3	PGE2	well_1	F006	339	Z distribution	-0.67628	-0.656	0.668935
ST6GAL1	W3	PGE2	well_2	F001	180	Z distribution	0.050292	0.103527	0.58166
ST6GAL1	W3	PGE2	well_2	F002	60	Z distribution	0.011363	0.062118	0.356221
ST6GAL1	W3	PGE2	well_2	F003	321	Z distribution	-0.57798	-0.54444	0.640398
ST6GAL1	W3	PGE2	well_2	F004	295	Z distribution	0.204679	0.181078	0.475929
ST6GAL1	W3	PGE2	well_2	F005	179	Z distribution	-0.34102	-0.24623	0.444651
ST6GAL1	W3	PGE2	well_2	F006	324	Z distribution	-0.41865	-0.32107	0.571215
ST6GAL1	W3	PGE2	well_3	F001	186	Z distribution	-0.0651	-0.01668	0.454922
ST6GAL1	W3	PGE2	well_3	F002	183	Z distribution	0.079219	0.088684	0.57648
ST6GAL1	W3	PGE2	well_3	F003	239	Z distribution	-0.60428	-0.55829	0.715303
ST6GAL1	W3	PGE2	well_3	F004	152	Z distribution	0.154048	0.18787	0.574729
ST6GAL1	W3	PGE2	well_3	F005	152	Z distribution	0.113275	0.061234	0.598657
ST6GAL1	W3	PGE2	well_3	F006	233	Z distribution	-0.41723	-0.22938	1.040305
ST6GAL1	W3	PGE2	well_4	F001	197	Z distribution	-0.2816	-0.2189	0.646858
ST6GAL1	W3	PGE2	well_4	F002	218	Z distribution	-0.19746	-0.10597	0.879081
ST6GAL1	W3	PGE2	well_4	F003	215	Z distribution	0.483753	0.488808	0.536147
ST6GAL1	W3	PGE2	well_4	F004	205	Z distribution	0.168289	0.173247	0.643564
ST6GAL1	W3	PGE2	well_4	F005	222	Z distribution	-0.18059	-0.17194	0.520441
ST6GAL1	W3	PGE2	well_4	F006	214	Z distribution	-0.27344	-0.28966	0.734186
ST6GAL1	W3	PGE2	well_5	F001	57	Z distribution	-0.80083	-0.89758	0.558803
ST6GAL1	W3	PGE2	well_5	F002	70	Z distribution	-1.12669	-1.07418	0.681701
ST6GAL1	W3	PGE2	well_5	F003	237	Z distribution	-0.31742	-0.20246	0.683205
ST6GAL1	W3	PGE2	well_5	F004	265	Z distribution	0.352982	0.311698	0.595726
ST6GAL1	W3	PGE2	well_5	F005	166	Z distribution	0.002629	-0.02741	0.413934
ST6GAL1	W3	PGE2	well_5	F006	38	Z distribution	0.17874	0.223799	0.624009
ST6GAL1	W4	PGE2	well_1	F001	67	Z distribution	-0.28654	-0.2819	0.493784
ST6GAL1	W4	PGE2	well_1	F002	46	Z distribution	-0.10868	-0.09976	0.466685

ST6GAL1	W4	PGE2	well_1	F003	193	Z distribution	-0.17776	-0.11348	0.540855
ST6GAL1	W4	PGE2	well_1	F004	130	Z distribution	-0.67766	-0.58828	0.512716
ST6GAL1	W4	PGE2	well_1	F005	171	Z distribution	0.101665	0.026903	0.59022
ST6GAL1	W4	PGE2	well_1	F006	173	Z distribution	0.420922	0.347306	0.444296
ST6GAL1	W4	PGE2	well_2	F001	204	Z distribution	0.428682	0.417275	0.635301
ST6GAL1	W4	PGE2	well_2	F002	61	Z distribution	-0.34799	-0.26703	0.496379
ST6GAL1	W4	PGE2	well_2	F003	71	Z distribution	0.200602	0.340775	0.676176
ST6GAL1	W4	PGE2	well_2	F004	97	Z distribution	-0.04348	-0.04356	0.502808
ST6GAL1	W4	PGE2	well_2	F005	63	Z distribution	-0.76364	-0.70863	0.379646
ST6GAL1	W4	PGE2	well_2	F006	135	Z distribution	-0.31328	-0.2245	0.542124
ST6GAL1	W4	PGE2	well_3	F001	193	Z distribution	-0.62358	-0.6912	0.942089
ST6GAL1	W4	PGE2	well_3	F002	13	Z distribution	0.503292	0.361419	0.43767
ST6GAL1	W4	PGE2	well_3	F003	13	Z distribution	-0.98715	-0.94169	0.404204
ST6GAL1	W4	PGE2	well_3	F004	224	Z distribution	-0.17728	-0.12194	0.463982
ST6GAL1	W4	PGE2	well_3	F005	182	Z distribution	0.024717	0.014784	0.495242
ST6GAL1	W4	PGE2	well_3	F006	42	Z distribution	-0.68785	-0.73896	0.526285
ST6GAL1	W4	PGE2	well_4	F001	166	Z distribution	-0.15526	-0.13555	0.468334
ST6GAL1	W4	PGE2	well_4	F002	91	Z distribution	-0.41581	-0.45552	0.600775
ST6GAL1	W4	PGE2	well_4	F003	232	Z distribution	-0.0547	-0.02436	0.629934
ST6GAL1	W4	PGE2	well_4	F004	173	Z distribution	-0.118	-0.12876	0.52117
ST6GAL1	W4	PGE2	well_4	F005	263	Z distribution	0.154328	0.162968	0.73121
ST6GAL1	W4	PGE2	well_4	F006	32	Z distribution	-1.04683	-0.96271	0.488919
ST6GAL1	W4	PGE2	well_5	F001	57	Z distribution	-0.75488	-0.74426	0.440892
ST6GAL1	W4	PGE2	well_5	F002	96	Z distribution	-0.18178	-0.04529	0.765064
ST6GAL1	W4	PGE2	well_5	F004	95	Z distribution	-0.44382	-0.40584	0.470675
ST6GAL1	W4	PGE2	well_5	F006	4	Z distribution	0.122325	0.122325	0.091923
ST6GAL1	W1	HPI4	well_1	F001	30	Total volume	9.171067	7.722361	7.808691
ST6GAL1	W1	HPI4	well_1	F002	3	Total volume	14.34153	14.34153	NA
ST6GAL1	W1	HPI4	well_1	F003	7	Total volume	10.69747	9.846597	3.804305
ST6GAL1	W1	HPI4	well_1	F004	25	Total volume	8.662424	6.184931	7.324935
ST6GAL1	W1	HPI4	well_1	F005	22	Total volume	7.032539	4.917431	7.038606
ST6GAL1	W1	HPI4	well_1	F006	20	Total volume	10.5964	5.891528	12.36481
ST6GAL1	W1	HPI4	well_2	F001	8	Total volume	7.072963	5.621597	3.902003
ST6GAL1	W1	HPI4	well_2	F002	35	Total volume	8.835399	5.375139	9.049721
ST6GAL1	W1	HPI4	well_2	F003	18	Total volume	12.41128	6.267083	12.23804
ST6GAL1	W1	HPI4	well_2	F004	41	Total volume	9.66129	5.175625	9.136989
ST6GAL1	W1	HPI4	well_2	F005	9	Total volume	10.80058	3.450417	11.77568
ST6GAL1	W1	HPI4	well_2	F006	7	Total volume	4.459722	3.286111	4.241529
ST6GAL1	W1	HPI4	well_3	F001	8	Total volume	2.714954	2.910556	2.025786
ST6GAL1	W1	HPI4	well_3	F002	4	Total volume	14.83444	14.83444	NA
ST6GAL1	W1	HPI4	well_3	F003	19	Total volume	14.96285	13.52	11.26622
ST6GAL1	W1	HPI4	well_3	F004	31	Total volume	11.72528	8.802083	9.001837
ST6GAL1	W1	HPI4	well_3	F005	9	Total volume	10.40155	7.722361	9.29779
ST6GAL1	W1	HPI4	well_3	F006	18	Total volume	5.621597	1.948194	7.379696
ST6GAL1	W1	HPI4	well_4	F001	65	Total volume	12.15946	8.802083	10.90204
ST6GAL1	W1	HPI4	well_4	F002	28	Total volume	4.502755	3.567778	3.720378
ST6GAL1	W1	HPI4	well_4	F003	3	Total volume	13.28528	13.28528	NA
ST6GAL1	W1	HPI4	well_4	F004	29	Total volume	9.428322	6.76	9.047189
ST6GAL1	W1	HPI4	well_4	F005	16	Total volume	18.11553	11.39576	18.72519

ST6GAL1	W1	HPI4	well_4	F006	14	Total volume	1.798333	0.63375	2.252509
ST6GAL1	W1	HPI4	well_5	F001	7	Total volume	18.09708	12.45201	21.50427
ST6GAL1	W1	HPI4	well_5	F002	18	Total volume	16.77677	18.0384	9.052489
ST6GAL1	W1	HPI4	well_5	F003	31	Total volume	13.16792	10.02264	11.77696
ST6GAL1	W1	HPI4	well_5	F004	10	Total volume	5.753628	2.675833	7.261007
ST6GAL1	W1	HPI4	well_5	F005	26	Total volume	10.04078	7.734097	8.841394
ST6GAL1	W1	HPI4	well_5	F006	25	Total volume	12.59754	5.410347	14.06605
ST6GAL1	W2	HPI4	well_1	F001	3	Total volume	15.67944	15.67944	NA
ST6GAL1	W2	HPI4	well_1	F002	7	Total volume	5.501889	4.905694	4.793324
ST6GAL1	W2	HPI4	well_1	F003	5	Total volume	2.167269	1.854306	1.711596
ST6GAL1	W2	HPI4	well_1	F004	25	Total volume	14.20964	13.21486	9.330344
ST6GAL1	W2	HPI4	well_1	F005	30	Total volume	10.20951	8.438264	6.986228
ST6GAL1	W2	HPI4	well_1	F006	40	Total volume	5.883052	3.896389	4.968412
ST6GAL1	W2	HPI4	well_2	F001	8	Total volume	0.751111	0.434236	0.822131
ST6GAL1	W2	HPI4	well_2	F002	8	Total volume	13.8134	14.40021	9.959546
ST6GAL1	W2	HPI4	well_2	F003	20	Total volume	15.26738	11.85347	11.6452
ST6GAL1	W2	HPI4	well_2	F004	10	Total volume	15.23054	15.09264	12.46959
ST6GAL1	W2	HPI4	well_2	F005	12	Total volume	20.0711	15.62076	17.88833
ST6GAL1	W2	HPI4	well_2	F006	13	Total volume	7.526048	7.323333	4.450612
ST6GAL1	W2	HPI4	well_3	F001	20	Total volume	11.42315	10.24563	7.338607
ST6GAL1	W2	HPI4	well_3	F002	5	Total volume	0.070417	0.070417	0.033195
ST6GAL1	W2	HPI4	well_3	F003	15	Total volume	3.797083	2.605417	5.649424
ST6GAL1	W2	HPI4	well_3	F004	49	Total volume	NA	NA	NA
ST6GAL1	W2	HPI4	well_3	F005	12	Total volume	16.97096	15.70292	9.240597
ST6GAL1	W2	HPI4	well_3	F006	5	Total volume	15.26868	13.9425	8.301864
ST6GAL1	W2	HPI4	well_4	F001	9	Total volume	4.983935	6.360972	3.717316
ST6GAL1	W2	HPI4	well_4	F002	28	Total volume	17.34262	16.94694	8.553593
ST6GAL1	W2	HPI4	well_4	F003	10	Total volume	10.84808	9.025069	5.406032
ST6GAL1	W2	HPI4	well_4	F004	13	Total volume	10.48622	10.55076	3.381438
ST6GAL1	W2	HPI4	well_4	F005	17	Total volume	24.81227	26.54708	9.521639
ST6GAL1	W2	HPI4	well_4	F006	7	Total volume	11.78462	10.53903	7.910052
ST6GAL1	W2	HPI4	well_5	F001	10	Total volume	9.5485	6.314028	10.12897
ST6GAL1	W2	HPI4	well_5	F002	13	Total volume	12.59285	11.98257	8.749253
ST6GAL1	W2	HPI4	well_5	F003	15	Total volume	11.71477	11.07889	8.909318
ST6GAL1	W2	HPI4	well_5	F004	10	Total volume	13.06681	7.464167	14.75111
ST6GAL1	W2	HPI4	well_5	F005	43	Total volume	10.42167	12.00604	5.30843
ST6GAL1	W2	HPI4	well_5	F006	1	Total volume	11.06113	11.14931	6.736379
ST6GAL1	W3	HPI4	well_1	F001	14	Total volume	5.07	5.07	NA
ST6GAL1	W3	HPI4	well_1	F002	23	Total volume	2.300278	1.032778	3.406573
ST6GAL1	W3	HPI4	well_1	F003	94	Total volume	4.841455	3.990278	4.651613
ST6GAL1	W3	HPI4	well_1	F004	76	Total volume	2.492402	1.948194	1.936885
ST6GAL1	W3	HPI4	well_1	F005	32	Total volume	8.751687	7.640208	5.925172
ST6GAL1	W3	HPI4	well_1	F006	1	Total volume	8.517902	8.942917	6.103999
ST6GAL1	W3	HPI4	well_2	F001	6	Total volume	0.563333	0.563333	NA
ST6GAL1	W3	HPI4	well_2	F003	5	Total volume	8.678854	10.17521	3.216373
ST6GAL1	W3	HPI4	well_2	F004	11	Total volume	9.365417	11.12583	3.421554
ST6GAL1	W3	HPI4	well_2	F005	15	Total volume	11.38142	13.05056	6.127491
ST6GAL1	W3	HPI4	well_2	F006	7	Total volume	4.544583	2.347222	4.775204
ST6GAL1	W3	HPI4	well_3	F001	3	Total volume	1.314444	1.326181	0.510307

ST6GAL1	W3	HPI4	well_3	F003	11	Total volume	31.78139	31.78139	NA
ST6GAL1	W3	HPI4	well_3	F005	5	Total volume	8.48912	8.778611	5.16954
ST6GAL1	W3	HPI4	well_3	F006	6	Total volume	1.259676	0.845	1.419309
ST6GAL1	W3	HPI4	well_4	F001	5	Total volume	1.355521	1.255764	0.910564
ST6GAL1	W3	HPI4	well_4	F002	6	Total volume	2.355046	1.830833	0.928365
ST6GAL1	W3	HPI4	well_4	F003	5	Total volume	0.305139	0.258194	0.08131
ST6GAL1	W3	HPI4	well_4	F004	29	Total volume	12.97231	13.00361	7.698937
ST6GAL1	W3	HPI4	well_4	F005	5	Total volume	4.929167	3.192222	4.488061
ST6GAL1	W3	HPI4	well_4	F006	8	Total volume	10.69551	10.70333	0.246551
ST6GAL1	W3	HPI4	well_5	F001	21	Total volume	9.701852	9.834861	5.354336
ST6GAL1	W3	HPI4	well_5	F002	5	Total volume	8.513004	4.084167	8.121544
ST6GAL1	W3	HPI4	well_5	F003	7	Total volume	16.22713	17.81542	4.454428
ST6GAL1	W3	HPI4	well_5	F004	27	Total volume	7.121472	5.586389	2.435666
ST6GAL1	W3	HPI4	well_5	F005	83	Total volume	18.71042	20.09222	10.88578
ST6GAL1	W3	HPI4	well_5	F006	3	Total volume	15.54858	15.44472	7.282012
ST6GAL1	W4	HPI4	well_1	F001	4	Total volume	0.516389	0.516389	NA
ST6GAL1	W4	HPI4	well_1	F002	3	Total volume	6.865625	6.865625	9.443902
ST6GAL1	W4	HPI4	well_1	F003	9	Total volume	1.431806	1.431806	NA
ST6GAL1	W4	HPI4	well_1	F004	11	Total volume	16.06841	16.78264	9.273071
ST6GAL1	W4	HPI4	well_1	F005	19	Total volume	2.881867	0.774583	4.337597
ST6GAL1	W4	HPI4	well_1	F006	18	Total volume	2.600201	0.668958	4.203001
ST6GAL1	W4	HPI4	well_2	F004	13	Total volume	8.251953	4.893958	7.557025
ST6GAL1	W4	HPI4	well_2	F005	9	Total volume	NA	NA	NA
ST6GAL1	W4	HPI4	well_2	F006	6	Total volume	8.34971	2.347222	8.176397
ST6GAL1	W4	HPI4	well_3	F001	1	Total volume	0.258194	0.258194	NA
ST6GAL1	W4	HPI4	well_3	F002	39	Total volume	1.451925	0.891944	1.789256
ST6GAL1	W4	HPI4	well_3	F003	3	Total volume	NA	NA	NA
ST6GAL1	W4	HPI4	well_3	F004	23	Total volume	0.680694	0.469444	0.449663
ST6GAL1	W4	HPI4	well_3	F005	6	Total volume	8.543889	8.543889	NA
ST6GAL1	W4	HPI4	well_4	F001	9	Total volume	1.558682	1.244028	1.535468
ST6GAL1	W4	HPI4	well_4	F004	16	Total volume	16.07847	16.07847	NA
ST6GAL1	W4	HPI4	well_4	F005	13	Total volume	NA	NA	NA
ST6GAL1	W4	HPI4	well_4	F006	5	Total volume	2.974452	2.464583	2.658687
ST6GAL1	W4	HPI4	well_5	F001	8	Total volume	5.645069	5.292986	3.682098
ST6GAL1	W4	HPI4	well_5	F003	8	Total volume	0.848353	0.516389	0.681426
ST6GAL1	W4	HPI4	well_5	F004	9	Total volume	7.899306	3.309583	10.06492
ST6GAL1	W4	HPI4	well_5	F005	12	Total volume	10.44514	11.85347	6.470571
ST6GAL1	W4	HPI4	well_5	F006	23	Total volume	3.912037	2.042083	3.320497
ST6GAL1	W1	PGE2	well_1	F001	33	Total volume	4.258532	3.638194	3.910054
ST6GAL1	W1	PGE2	well_1	F002	8	Total volume	4.991759	3.192222	6.088437
ST6GAL1	W1	PGE2	well_1	F003	11	Total volume	9.968988	3.779028	11.20222
ST6GAL1	W1	PGE2	well_1	F004	20	Total volume	8.887437	1.431806	12.05252
ST6GAL1	W1	PGE2	well_1	F005	4	Total volume	5.795168	3.403472	4.80175
ST6GAL1	W1	PGE2	well_1	F006	19	Total volume	7.822725	5.163889	7.109205
ST6GAL1	W1	PGE2	well_2	F001	14	Total volume	4.41669	4.776597	3.1374
ST6GAL1	W1	PGE2	well_2	F002	13	Total volume	8.228318	7.464167	7.73846
ST6GAL1	W1	PGE2	well_2	F003	5	Total volume	11.09845	9.776181	8.995212
ST6GAL1	W1	PGE2	well_2	F004	1	Total volume	3.262639	3.262639	2.5228
ST6GAL1	W1	PGE2	well_2	F005	19	Total volume	6.220139	4.717917	5.618405

ST6GAL1	W1	PGE2	well_2	F006	18	Total volume	9.954178	2.992708	15.62641
ST6GAL1	W1	PGE2	well_3	F001	8	Total volume	6.719457	7.088611	3.597888
ST6GAL1	W1	PGE2	well_3	F002	11	Total volume	1.181435	1.032778	0.363883
ST6GAL1	W1	PGE2	well_3	F003	6	Total volume	10.63292	10.63292	NA
ST6GAL1	W1	PGE2	well_3	F004	7	Total volume	6.750335	3.16875	7.467151
ST6GAL1	W1	PGE2	well_3	F005	1	Total volume	11.05688	10.22215	9.6143
ST6GAL1	W1	PGE2	well_3	F006	88	Total volume	11.54833	12.01778	8.117182
ST6GAL1	W1	PGE2	well_4	F001	7	Total volume	6.068873	4.131111	5.589767
ST6GAL1	W1	PGE2	well_4	F002	7	Total volume	4.706181	4.682708	3.586184
ST6GAL1	W1	PGE2	well_4	F003	6	Total volume	1.441194	0.845	1.6379
ST6GAL1	W1	PGE2	well_4	F004	6	Total volume	59.31431	59.31431	NA
ST6GAL1	W1	PGE2	well_4	F005	8	Total volume	6.720159	4.342361	7.169389
ST6GAL1	W1	PGE2	well_4	F006	1	Total volume	3.750861	2.910556	2.74125
ST6GAL1	W1	PGE2	well_5	F001	111	Total volume	10.43575	6.642639	10.12269
ST6GAL1	W1	PGE2	well_5	F002	152	Total volume	1.954063	2.370694	1.085046
ST6GAL1	W1	PGE2	well_5	F003	160	Total volume	NA	NA	NA
ST6GAL1	W1	PGE2	well_5	F004	234	Total volume	26.10698	25.70208	8.290301
ST6GAL1	W1	PGE2	well_5	F005	148	Total volume	5.218657	5.07	2.528457
ST6GAL1	W1	PGE2	well_5	F006	184	Total volume	3.755556	3.755556	NA
ST6GAL1	W2	PGE2	well_1	F001	64	Total volume	8.041488	5.234306	8.156077
ST6GAL1	W2	PGE2	well_1	F002	175	Total volume	7.198966	4.213264	7.663435
ST6GAL1	W2	PGE2	well_1	F003	338	Total volume	7.118766	6.384444	4.432649
ST6GAL1	W2	PGE2	well_1	F004	145	Total volume	9.145252	6.243611	8.24392
ST6GAL1	W2	PGE2	well_1	F005	88	Total volume	9.868806	8.66125	7.557357
ST6GAL1	W2	PGE2	well_1	F006	268	Total volume	8.48168	6.431389	6.498515
ST6GAL1	W2	PGE2	well_2	F001	42	Total volume	6.301034	3.755556	6.750639
ST6GAL1	W2	PGE2	well_2	F002	220	Total volume	8.707297	7.088611	6.327334
ST6GAL1	W2	PGE2	well_2	F003	49	Total volume	7.201697	5.469028	6.210738
ST6GAL1	W2	PGE2	well_2	F004	77	Total volume	9.441335	7.452431	6.94305
ST6GAL1	W2	PGE2	well_2	F005	198	Total volume	7.240278	5.398611	6.638074
ST6GAL1	W2	PGE2	well_2	F006	276	Total volume	8.782573	6.642639	7.066933
ST6GAL1	W2	PGE2	well_3	F001	44	Total volume	9.782701	6.642639	9.676334
ST6GAL1	W2	PGE2	well_3	F002	291	Total volume	4.576481	3.333056	4.223714
ST6GAL1	W2	PGE2	well_3	F003	71	Total volume	8.144861	6.055833	6.358134
ST6GAL1	W2	PGE2	well_3	F004	72	Total volume	7.529481	3.614722	7.292742
ST6GAL1	W2	PGE2	well_3	F005	39	Total volume	6.649974	4.940903	5.764471
ST6GAL1	W2	PGE2	well_3	F006	53	Total volume	8.551841	6.689583	6.902582
ST6GAL1	W2	PGE2	well_4	F001	239	Total volume	4.650588	2.089028	5.077977
ST6GAL1	W2	PGE2	well_4	F002	250	Total volume	7.023035	5.609861	5.393214
ST6GAL1	W2	PGE2	well_4	F003	158	Total volume	8.62511	7.605	5.447058
ST6GAL1	W2	PGE2	well_4	F004	13	Total volume	4.668038	3.497361	4.5199
ST6GAL1	W2	PGE2	well_4	F005	11	Total volume	9.07302	6.501806	7.55928
ST6GAL1	W2	PGE2	well_4	F006	117	Total volume	5.277255	2.816667	5.483603
ST6GAL1	W2	PGE2	well_5	F001	195	Total volume	9.466695	7.123819	7.862967
ST6GAL1	W2	PGE2	well_5	F002	350	Total volume	8.101074	6.666111	5.973916
ST6GAL1	W2	PGE2	well_5	F003	293	Total volume	8.00769	6.478333	6.989268
ST6GAL1	W2	PGE2	well_5	F004	136	Total volume	4.688043	2.816667	3.927108
ST6GAL1	W2	PGE2	well_5	F005	277	Total volume	6.306204	5.234306	5.734024
ST6GAL1	W2	PGE2	well_5	F006	339	Total volume	7.638828	4.85875	7.587714

ST6GAL1	W3	PGE2	well_1	F001	180	Total volume	10.32468	8.356111	8.034867
ST6GAL1	W3	PGE2	well_1	F002	60	Total volume	11.1652	9.740972	6.880206
ST6GAL1	W3	PGE2	well_1	F003	321	Total volume	12.32355	9.154167	10.00066
ST6GAL1	W3	PGE2	well_1	F004	295	Total volume	9.954339	9.048542	6.476423
ST6GAL1	W3	PGE2	well_1	F005	179	Total volume	10.41676	8.614306	8.12722
ST6GAL1	W3	PGE2	well_1	F006	324	Total volume	8.602531	7.417222	6.324399
ST6GAL1	W3	PGE2	well_2	F001	186	Total volume	5.81814	4.882222	4.627067
ST6GAL1	W3	PGE2	well_2	F002	183	Total volume	5.389918	3.861181	5.354807
ST6GAL1	W3	PGE2	well_2	F003	239	Total volume	7.121068	5.879792	5.42733
ST6GAL1	W3	PGE2	well_2	F004	152	Total volume	9.545548	7.734097	7.195676
ST6GAL1	W3	PGE2	well_2	F005	152	Total volume	13.19814	10.24563	9.461848
ST6GAL1	W3	PGE2	well_2	F006	233	Total volume	9.180328	7.851458	6.484373
ST6GAL1	W3	PGE2	well_3	F001	197	Total volume	5.346657	2.793194	6.185723
ST6GAL1	W3	PGE2	well_3	F002	218	Total volume	3.183912	1.666528	3.47188
ST6GAL1	W3	PGE2	well_3	F003	215	Total volume	4.195854	2.863611	4.059091
ST6GAL1	W3	PGE2	well_3	F004	205	Total volume	4.4938	3.38	4.427289
ST6GAL1	W3	PGE2	well_3	F005	222	Total volume	6.165076	4.553611	5.611806
ST6GAL1	W3	PGE2	well_3	F006	214	Total volume	3.766621	1.807361	4.666131
ST6GAL1	W3	PGE2	well_4	F001	57	Total volume	3.92996	1.854306	4.424908
ST6GAL1	W3	PGE2	well_4	F002	70	Total volume	2.408126	1.290972	2.588384
ST6GAL1	W3	PGE2	well_4	F003	237	Total volume	9.437171	7.112083	8.394967
ST6GAL1	W3	PGE2	well_4	F004	265	Total volume	4.516895	1.830833	5.46463
ST6GAL1	W3	PGE2	well_4	F005	166	Total volume	6.839189	5.234306	5.770131
ST6GAL1	W3	PGE2	well_4	F006	38	Total volume	5.684023	4.318889	4.408052
ST6GAL1	W3	PGE2	well_5	F001	67	Total volume	2.916304	2.558472	2.639097
ST6GAL1	W3	PGE2	well_5	F002	46	Total volume	1.723315	0.997569	1.881903
ST6GAL1	W3	PGE2	well_5	F003	193	Total volume	3.758337	2.558472	3.577096
ST6GAL1	W3	PGE2	well_5	F004	130	Total volume	5.71191	3.966806	5.155941
ST6GAL1	W3	PGE2	well_5	F005	171	Total volume	6.315635	4.307153	5.147221
ST6GAL1	W3	PGE2	well_5	F006	173	Total volume	5.455911	3.215694	5.818932
ST6GAL1	W4	PGE2	well_1	F001	204	Total volume	1.302708	0.845	1.422281
ST6GAL1	W4	PGE2	well_1	F002	61	Total volume	8.969323	7.229444	6.292568
ST6GAL1	W4	PGE2	well_1	F003	71	Total volume	3.16875	2.182917	3.171494
ST6GAL1	W4	PGE2	well_1	F004	97	Total volume	4.143766	2.793194	3.458948
ST6GAL1	W4	PGE2	well_1	F005	63	Total volume	2.810564	1.666528	2.830077
ST6GAL1	W4	PGE2	well_1	F006	135	Total volume	6.341515	4.002014	5.786481
ST6GAL1	W4	PGE2	well_2	F001	193	Total volume	1.845358	1.126667	1.919657
ST6GAL1	W4	PGE2	well_2	F002	13	Total volume	1.596538	1.079722	1.487572
ST6GAL1	W4	PGE2	well_2	F003	13	Total volume	6.780065	5.175625	5.926095
ST6GAL1	W4	PGE2	well_2	F004	224	Total volume	3.063549	2.135972	3.05
ST6GAL1	W4	PGE2	well_2	F005	182	Total volume	5.913293	5.680278	4.015686
ST6GAL1	W4	PGE2	well_2	F006	42	Total volume	4.382459	2.699306	4.325274
ST6GAL1	W4	PGE2	well_3	F001	166	Total volume	3.440919	2.182917	3.409379
ST6GAL1	W4	PGE2	well_3	F002	91	Total volume	0.375556	0.105625	0.491339
ST6GAL1	W4	PGE2	well_3	F003	232	Total volume	2.52522	1.760417	2.610313
ST6GAL1	W4	PGE2	well_3	F004	173	Total volume	5.709883	3.943333	5.120751
ST6GAL1	W4	PGE2	well_3	F005	263	Total volume	3.93406	2.276806	4.161708
ST6GAL1	W4	PGE2	well_3	F006	32	Total volume	1.674775	0.962361	2.016185
ST6GAL1	W4	PGE2	well_4	F001	57	Total volume	7.478378	5.140417	6.862226

ST6GAL1	W4	PGE2	well_4	F002	96	Total volume	2.68262	1.666528	3.287134
ST6GAL1	W4	PGE2	well_4	F003	95	Total volume	4.159255	2.828403	3.573802
ST6GAL1	W4	PGE2	well_4	F004	4	Total volume	2.663567	1.502222	2.780711
ST6GAL1	W4	PGE2	well_4	F005		Total volume	2.757076	1.760417	2.749357
ST6GAL1	W4	PGE2	well_4	F006		Total volume	5.439688	3.685139	5.575898
ST6GAL1	W4	PGE2	well_5	F001		Total volume	1.037942	0.410764	1.23642
ST6GAL1	W4	PGE2	well_5	F002		Total volume	1.893426	0.856736	2.263225
ST6GAL1	W4	PGE2	well_5	F004		Total volume	4.280327	3.180486	3.981761
ST6GAL1	W4	PGE2	well_5	F006		Total volume	4.835278	4.835278	0.697089
TOM20	W1	HPI4	well_1	F001	210	Avg. Volume	3.604433	3.348789	1.404767
TOM20	W1	HPI4	well_1	F002	255	Avg. Volume	2.642671	2.466191	1.054336
TOM20	W1	HPI4	well_1	F003	300	Avg. Volume	3.910644	3.730372	1.450992
TOM20	W1	HPI4	well_1	F004	232	Avg. Volume	3.799002	3.623578	1.34292
TOM20	W1	HPI4	well_1	F005	317	Avg. Volume	4.079064	3.934798	1.534088
TOM20	W1	HPI4	well_1	F006	182	Avg. Volume	4.154165	3.902553	1.697521
TOM20	W1	HPI4	well_2	F001	267	Avg. Volume	3.759947	3.559465	1.424897
TOM20	W1	HPI4	well_2	F002	241	Avg. Volume	4.193716	4.036795	1.588465
TOM20	W1	HPI4	well_2	F003	231	Avg. Volume	4.062995	3.774195	1.667196
TOM20	W1	HPI4	well_2	F004	185	Avg. Volume	3.749535	3.408167	1.615316
TOM20	W1	HPI4	well_2	F005	466	Avg. Volume	3.767226	3.548103	1.390458
TOM20	W1	HPI4	well_2	F006	206	Avg. Volume	4.418944	4.101088	1.600384
TOM20	W1	HPI4	well_3	F001	312	Avg. Volume	4.64285	4.344715	1.784653
TOM20	W1	HPI4	well_3	F002	278	Avg. Volume	4.030648	3.715501	1.543828
TOM20	W1	HPI4	well_3	F003	234	Avg. Volume	4.873545	4.683379	1.834064
TOM20	W1	HPI4	well_3	F004	217	Avg. Volume	3.943035	3.834875	1.278982
TOM20	W1	HPI4	well_3	F005	341	Avg. Volume	3.245829	3.027917	1.216348
TOM20	W1	HPI4	well_3	F006	293	Avg. Volume	3.765713	3.564167	1.405257
TOM20	W1	HPI4	well_4	F001	244	Avg. Volume	3.895875	3.638586	1.579892
TOM20	W1	HPI4	well_4	F002	313	Avg. Volume	3.922978	3.716276	1.43349
TOM20	W1	HPI4	well_4	F003	176	Avg. Volume	3.54459	3.320259	1.628773
TOM20	W1	HPI4	well_4	F004	175	Avg. Volume	3.288912	3.073296	1.421901
TOM20	W1	HPI4	well_4	F005	351	Avg. Volume	4.050222	3.840541	1.579856
TOM20	W1	HPI4	well_4	F006	306	Avg. Volume	3.415519	3.247987	1.280657
TOM20	W1	HPI4	well_5	F001	243	Avg. Volume	3.694643	3.387545	1.502125
TOM20	W1	HPI4	well_5	F002	220	Avg. Volume	4.043993	3.842509	1.645075
TOM20	W1	HPI4	well_5	F003	315	Avg. Volume	4.323398	4.046785	1.753675
TOM20	W1	HPI4	well_5	F004	124	Avg. Volume	3.22953	3.079129	1.268967
TOM20	W1	HPI4	well_5	F005	165	Avg. Volume	4.166212	3.718816	1.638352
TOM20	W1	HPI4	well_5	F006	187	Avg. Volume	3.56292	3.147412	1.463721
TOM20	W1	PGE2	well_1	F001	286	Avg. Volume	3.555074	3.265747	1.3753
TOM20	W1	PGE2	well_1	F002	265	Avg. Volume	3.890893	3.676405	1.408966
TOM20	W1	PGE2	well_1	F003	184	Avg. Volume	3.880081	3.705416	1.390106
TOM20	W1	PGE2	well_1	F004	295	Avg. Volume	4.773425	4.454866	1.789138
TOM20	W1	PGE2	well_1	F005	435	Avg. Volume	3.267474	2.972069	1.372569
TOM20	W1	PGE2	well_1	F006	244	Avg. Volume	4.542136	4.229213	1.84248
TOM20	W1	PGE2	well_2	F001	326	Avg. Volume	2.884837	2.665858	1.099559
TOM20	W1	PGE2	well_2	F002	246	Avg. Volume	4.46961	4.280069	1.426243
TOM20	W1	PGE2	well_2	F003	350	Avg. Volume	3.663855	3.605282	1.423519
TOM20	W1	PGE2	well_2	F004	269	Avg. Volume	4.404834	4.252029	1.606053

TOM20	W1	PGE2	well_2	F005	334	Avg. Volume	3.674165	3.521959	1.384004
TOM20	W1	PGE2	well_2	F006	290	Avg. Volume	4.125368	3.979377	1.625184
TOM20	W1	PGE2	well_3	F001	312	Avg. Volume	3.710799	3.579069	1.365941
TOM20	W1	PGE2	well_3	F002	328	Avg. Volume	4.00226	3.828477	1.428579
TOM20	W1	PGE2	well_3	F003	249	Avg. Volume	3.979984	3.744471	1.465487
TOM20	W1	PGE2	well_3	F004	293	Avg. Volume	4.014783	3.837056	1.450139
TOM20	W1	PGE2	well_3	F005	316	Avg. Volume	3.410886	3.225225	1.248712
TOM20	W1	PGE2	well_3	F006	251	Avg. Volume	4.694099	4.461461	1.778986
TOM20	W1	PGE2	well_4	F001	265	Avg. Volume	4.193244	4.000337	1.414475
TOM20	W1	PGE2	well_4	F002	268	Avg. Volume	3.92639	3.77805	1.478712
TOM20	W1	PGE2	well_4	F003	284	Avg. Volume	3.607483	3.564189	1.333726
TOM20	W1	PGE2	well_4	F004	289	Avg. Volume	3.130138	2.94523	1.263664
TOM20	W1	PGE2	well_4	F005	201	Avg. Volume	4.436477	4.197336	1.68135
TOM20	W1	PGE2	well_4	F006	289	Avg. Volume	3.66136	3.444923	1.445741
TOM20	W1	PGE2	well_5	F001	130	Avg. Volume	3.727297	3.443642	1.523561
TOM20	W1	PGE2	well_5	F002	191	Avg. Volume	3.326546	3.30873	1.106634
TOM20	W1	PGE2	well_5	F003	277	Avg. Volume	3.827951	3.603769	1.426404
TOM20	W1	PGE2	well_5	F004	384	Avg. Volume	3.751077	3.571962	1.428399
TOM20	W1	PGE2	well_5	F005	253	Avg. Volume	3.654133	3.409731	1.477783
TOM20	W1	PGE2	well_5	F006	146	Avg. Volume	4.287141	3.925044	1.58711
TOM20	W2	HPI4	well_1	F001	253	Avg. Volume	2.723598	2.412944	1.466292
TOM20	W2	HPI4	well_1	F002	159	Avg. Volume	2.904149	2.572917	1.683275
TOM20	W2	HPI4	well_1	F003	177	Avg. Volume	3.143622	2.860929	1.409263
TOM20	W2	HPI4	well_1	F004	419	Avg. Volume	2.661792	2.479198	1.088865
TOM20	W2	HPI4	well_1	F005	229	Avg. Volume	3.090151	2.82099	1.381055
TOM20	W2	HPI4	well_1	F006	159	Avg. Volume	3.876297	3.394444	1.789733
TOM20	W2	HPI4	well_2	F001	220	Avg. Volume	2.604756	2.210916	1.306535
TOM20	W2	HPI4	well_2	F002	234	Avg. Volume	2.473426	2.158378	1.348167
TOM20	W2	HPI4	well_2	F003	234	Avg. Volume	2.19705	1.949033	1.030438
TOM20	W2	HPI4	well_2	F004	283	Avg. Volume	2.615648	2.371952	1.254916
TOM20	W2	HPI4	well_2	F005	274	Avg. Volume	3.022564	2.783145	1.414359
TOM20	W2	HPI4	well_2	F006	252	Avg. Volume	3.167478	2.892606	1.474619
TOM20	W2	HPI4	well_3	F001	294	Avg. Volume	3.151917	2.948772	1.358285
TOM20	W2	HPI4	well_3	F002	273	Avg. Volume	3.235583	3.007798	1.628889
TOM20	W2	HPI4	well_3	F003	219	Avg. Volume	3.774172	3.495939	1.796267
TOM20	W2	HPI4	well_3	F004	265	Avg. Volume	3.660622	3.325231	1.454073
TOM20	W2	HPI4	well_3	F005	194	Avg. Volume	2.788418	2.520761	1.332598
TOM20	W2	HPI4	well_3	F006	281	Avg. Volume	2.861041	2.541706	1.35228
TOM20	W2	HPI4	well_4	F001	200	Avg. Volume	1.834288	1.615447	0.947878
TOM20	W2	HPI4	well_4	F002	369	Avg. Volume	3.578235	3.178633	1.726314
TOM20	W2	HPI4	well_4	F003	410	Avg. Volume	2.12872	1.949261	0.930122
TOM20	W2	HPI4	well_4	F004	455	Avg. Volume	3.111075	2.778105	1.468872
TOM20	W2	HPI4	well_4	F005	292	Avg. Volume	2.329702	2.138506	1.119233
TOM20	W2	HPI4	well_4	F006	259	Avg. Volume	2.673741	2.303339	1.448787
TOM20	W2	HPI4	well_5	F001	250	Avg. Volume	1.992205	1.747915	1.083695
TOM20	W2	HPI4	well_5	F002	196	Avg. Volume	3.062854	2.767444	1.516234
TOM20	W2	HPI4	well_5	F003	321	Avg. Volume	2.50189	2.388951	0.990047
TOM20	W2	HPI4	well_5	F004	448	Avg. Volume	2.566333	2.405484	1.037594
TOM20	W2	HPI4	well_5	F005	319	Avg. Volume	2.618607	2.404226	1.187749

TOM20	W2	HPI4	well_5	F006	269	Avg. Volume	2.061214	1.801184	1.001738
TOM20	W2	PGE2	well_1	F001	186	Avg. Volume	5.241455	5.173501	1.735919
TOM20	W2	PGE2	well_1	F002	233	Avg. Volume	3.910402	3.752622	1.785715
TOM20	W2	PGE2	well_1	F003	443	Avg. Volume	4.208211	3.921667	1.623581
TOM20	W2	PGE2	well_1	F004	70	Avg. Volume	3.933953	3.460168	1.637866
TOM20	W2	PGE2	well_1	F005	190	Avg. Volume	3.907973	3.778245	1.657862
TOM20	W2	PGE2	well_1	F006	211	Avg. Volume	4.313959	3.823701	1.876377
TOM20	W2	PGE2	well_2	F001	424	Avg. Volume	3.986895	3.602932	1.785001
TOM20	W2	PGE2	well_2	F002	268	Avg. Volume	3.678766	3.471723	1.46257
TOM20	W2	PGE2	well_2	F003	595	Avg. Volume	3.534723	3.300781	1.559742
TOM20	W2	PGE2	well_2	F004	512	Avg. Volume	3.417745	3.143881	1.521443
TOM20	W2	PGE2	well_2	F005	224	Avg. Volume	3.483264	3.335664	1.584614
TOM20	W2	PGE2	well_2	F006	91	Avg. Volume	3.761951	3.365713	1.713515
TOM20	W2	PGE2	well_3	F001	399	Avg. Volume	4.832596	4.466764	2.270608
TOM20	W2	PGE2	well_3	F002	454	Avg. Volume	4.58583	4.35653	1.739127
TOM20	W2	PGE2	well_3	F003	289	Avg. Volume	4.337186	3.994792	1.81277
TOM20	W2	PGE2	well_3	F004	475	Avg. Volume	3.953201	3.759698	1.589867
TOM20	W2	PGE2	well_3	F005	308	Avg. Volume	3.794309	3.562562	1.636328
TOM20	W2	PGE2	well_3	F006	312	Avg. Volume	3.614774	3.215929	1.416365
TOM20	W2	PGE2	well_4	F001	463	Avg. Volume	4.864946	4.560946	2.053435
TOM20	W2	PGE2	well_4	F002	355	Avg. Volume	5.76336	5.227599	2.270926
TOM20	W2	PGE2	well_4	F003	336	Avg. Volume	5.39733	4.971057	2.039821
TOM20	W2	PGE2	well_4	F004	479	Avg. Volume	4.195883	3.935865	1.686718
TOM20	W2	PGE2	well_4	F005	511	Avg. Volume	5.538385	5.309752	1.89103
TOM20	W2	PGE2	well_4	F006	473	Avg. Volume	4.373186	4.093556	1.821375
TOM20	W2	PGE2	well_5	F001	401	Avg. Volume	5.017556	4.658546	2.019108
TOM20	W2	PGE2	well_5	F002	370	Avg. Volume	4.103802	3.629637	1.841985
TOM20	W2	PGE2	well_5	F003	452	Avg. Volume	4.021265	3.663405	1.715918
TOM20	W2	PGE2	well_5	F004	396	Avg. Volume	4.640298	4.177788	1.803146
TOM20	W2	PGE2	well_5	F005	344	Avg. Volume	5.438195	5.128192	1.883707
TOM20	W2	PGE2	well_5	F006	360	Avg. Volume	4.584893	4.107105	1.942344
TOM20	W3	HPI4	well_1	F001	92	Avg. Volume	2.980665	2.51834	1.509111
TOM20	W3	HPI4	well_1	F002	87	Avg. Volume	1.829004	1.699883	0.972172
TOM20	W3	HPI4	well_1	F003	120	Avg. Volume	3.449988	3.450628	1.587191
TOM20	W3	HPI4	well_1	F004	66	Avg. Volume	1.992035	1.775967	0.890018
TOM20	W3	HPI4	well_1	F005	165	Avg. Volume	3.789056	3.490319	1.761637
TOM20	W3	HPI4	well_1	F006	32	Avg. Volume	1.484166	1.515716	0.664594
TOM20	W3	HPI4	well_2	F001	64	Avg. Volume	2.197051	2.124236	0.967516
TOM20	W3	HPI4	well_2	F002	138	Avg. Volume	2.88099	2.482902	1.385349
TOM20	W3	HPI4	well_2	F003	154	Avg. Volume	2.155213	1.950391	0.909768
TOM20	W3	HPI4	well_2	F004	219	Avg. Volume	2.217611	1.910639	1.066781
TOM20	W3	HPI4	well_2	F005	180	Avg. Volume	2.679199	2.27671	1.494664
TOM20	W3	HPI4	well_2	F006	181	Avg. Volume	2.710907	2.353929	1.403767
TOM20	W3	HPI4	well_3	F001	110	Avg. Volume	3.182331	3.021355	1.629605
TOM20	W3	HPI4	well_3	F002	130	Avg. Volume	3.269797	3.053705	1.326413
TOM20	W3	HPI4	well_3	F003	159	Avg. Volume	3.400048	3.130151	1.522626
TOM20	W3	HPI4	well_3	F004	207	Avg. Volume	2.175096	1.961179	1.147151
TOM20	W3	HPI4	well_3	F005	50	Avg. Volume	1.870316	1.772961	0.660322
TOM20	W3	HPI4	well_3	F006	77	Avg. Volume	2.187296	2.013277	0.98972

TOM20	W3	HPI4	well_4	F001	128	Avg. Volume	1.658226	1.504738	0.81609
TOM20	W3	HPI4	well_4	F002	141	Avg. Volume	2.183375	2.012488	1.047876
TOM20	W3	HPI4	well_4	F003	232	Avg. Volume	2.71881	2.567808	1.284167
TOM20	W3	HPI4	well_4	F004	148	Avg. Volume	2.971665	2.652288	1.511183
TOM20	W3	HPI4	well_4	F005	163	Avg. Volume	3.131833	2.974759	1.260682
TOM20	W3	HPI4	well_4	F006	251	Avg. Volume	1.955055	1.826001	0.894352
TOM20	W3	HPI4	well_5	F001	127	Avg. Volume	2.446594	2.082942	1.292417
TOM20	W3	HPI4	well_5	F002	152	Avg. Volume	2.600717	2.500118	1.094819
TOM20	W3	HPI4	well_5	F003	318	Avg. Volume	2.53968	2.257408	1.266284
TOM20	W3	HPI4	well_5	F004	110	Avg. Volume	2.086566	1.932428	0.837332
TOM20	W3	HPI4	well_5	F005	239	Avg. Volume	2.199182	2.11563	0.92865
TOM20	W3	HPI4	well_5	F006	59	Avg. Volume	2.416466	2.272459	1.181917
TOM20	W3	PGE2	well_1	F001	287	Avg. Volume	4.814828	4.670972	1.889865
TOM20	W3	PGE2	well_1	F002	378	Avg. Volume	4.984749	4.65421	2.041834
TOM20	W3	PGE2	well_1	F003	324	Avg. Volume	4.428746	4.101573	1.880738
TOM20	W3	PGE2	well_1	F004	454	Avg. Volume	4.369126	3.850165	2.052554
TOM20	W3	PGE2	well_1	F005	421	Avg. Volume	5.14931	4.60212	2.265952
TOM20	W3	PGE2	well_1	F006	241	Avg. Volume	4.724618	4.44128	2.181478
TOM20	W3	PGE2	well_2	F001	299	Avg. Volume	4.386856	4.126568	1.837292
TOM20	W3	PGE2	well_2	F002	369	Avg. Volume	5.073171	4.66706	2.29151
TOM20	W3	PGE2	well_2	F003	402	Avg. Volume	5.570197	5.169306	2.524045
TOM20	W3	PGE2	well_2	F004	460	Avg. Volume	5.448503	4.729111	2.525924
TOM20	W3	PGE2	well_2	F005	438	Avg. Volume	5.062782	4.597449	2.368351
TOM20	W3	PGE2	well_2	F006	353	Avg. Volume	4.88796	4.651412	2.207718
TOM20	W3	PGE2	well_3	F001	277	Avg. Volume	3.677844	3.382934	1.765963
TOM20	W3	PGE2	well_3	F002	273	Avg. Volume	4.621181	4.041134	2.348296
TOM20	W3	PGE2	well_3	F003	424	Avg. Volume	5.934872	5.316565	2.80432
TOM20	W3	PGE2	well_3	F004	481	Avg. Volume	5.160853	4.607262	2.501662
TOM20	W3	PGE2	well_3	F005	432	Avg. Volume	5.204906	4.768593	2.177703
TOM20	W3	PGE2	well_3	F006	292	Avg. Volume	5.121241	4.892232	1.994942
TOM20	W3	PGE2	well_4	F001	402	Avg. Volume	5.073208	4.651675	2.055321
TOM20	W3	PGE2	well_4	F002	358	Avg. Volume	5.986787	5.480177	2.577745
TOM20	W3	PGE2	well_4	F003	257	Avg. Volume	5.76867	5.4925	2.601084
TOM20	W3	PGE2	well_4	F004	374	Avg. Volume	3.841956	3.372884	1.781062
TOM20	W3	PGE2	well_4	F005	384	Avg. Volume	4.473202	4.159765	1.736288
TOM20	W3	PGE2	well_4	F006	253	Avg. Volume	4.756845	4.421806	2.236794
TOM20	W3	PGE2	well_5	F001	116	Avg. Volume	4.678649	4.357894	2.062871
TOM20	W3	PGE2	well_5	F002	157	Avg. Volume	4.859225	4.758155	1.96433
TOM20	W3	PGE2	well_5	F003	160	Avg. Volume	4.548248	4.068868	1.976753
TOM20	W3	PGE2	well_5	F004	364	Avg. Volume	3.853132	3.633001	1.671233
TOM20	W3	PGE2	well_5	F005	367	Avg. Volume	5.079661	4.767097	2.131792
TOM20	W3	PGE2	well_5	F006	253	Avg. Volume	5.940882	5.728899	2.546902
TOM20	W4	HPI4	well_1	F001	60	Avg. Volume	2.508304	2.511254	1.304976
TOM20	W4	HPI4	well_1	F002	159	Avg. Volume	3.420367	3.200672	1.451527
TOM20	W4	HPI4	well_1	F003	130	Avg. Volume	2.23148	1.998781	1.028665
TOM20	W4	HPI4	well_1	F004	179	Avg. Volume	3.652293	3.515804	1.391273
TOM20	W4	HPI4	well_1	F005	141	Avg. Volume	2.797106	2.515861	1.594171
TOM20	W4	HPI4	well_1	F006	130	Avg. Volume	2.604981	2.295858	1.102138
TOM20	W4	HPI4	well_2	F001	158	Avg. Volume	2.696629	2.482126	1.156157

TOM20	W4	HPI4	well_2	F002	191	Avg. Volume	3.085917	2.79543	1.426463
TOM20	W4	HPI4	well_2	F003	211	Avg. Volume	2.861296	2.616035	1.237003
TOM20	W4	HPI4	well_2	F004	192	Avg. Volume	3.41181	3.125943	1.562746
TOM20	W4	HPI4	well_2	F005	152	Avg. Volume	2.961506	2.87067	1.238163
TOM20	W4	HPI4	well_2	F006	115	Avg. Volume	3.417136	3.243249	1.55491
TOM20	W4	HPI4	well_3	F001	109	Avg. Volume	3.485273	3.271022	1.615581
TOM20	W4	HPI4	well_3	F002	140	Avg. Volume	3.068533	2.986654	1.122207
TOM20	W4	HPI4	well_3	F003	180	Avg. Volume	1.996225	1.755099	0.998699
TOM20	W4	HPI4	well_3	F004	241	Avg. Volume	3.864444	3.598192	1.673092
TOM20	W4	HPI4	well_3	F005	208	Avg. Volume	3.306786	3.120281	1.30515
TOM20	W4	HPI4	well_3	F006	159	Avg. Volume	4.187661	3.812932	1.743624
TOM20	W4	HPI4	well_4	F001	151	Avg. Volume	3.096237	2.917534	1.611768
TOM20	W4	HPI4	well_4	F002	185	Avg. Volume	3.029376	2.903514	1.160655
TOM20	W4	HPI4	well_4	F003	207	Avg. Volume	3.069821	2.820163	1.575018
TOM20	W4	HPI4	well_4	F004	170	Avg. Volume	3.879931	3.809472	1.63292
TOM20	W4	HPI4	well_4	F005	172	Avg. Volume	4.560434	4.31122	1.71347
TOM20	W4	HPI4	well_4	F006	168	Avg. Volume	3.976177	3.924939	1.409539
TOM20	W4	HPI4	well_5	F001	177	Avg. Volume	3.428839	3.094722	1.778677
TOM20	W4	HPI4	well_5	F002	101	Avg. Volume	2.060468	1.844036	1.225087
TOM20	W4	HPI4	well_5	F003	244	Avg. Volume	2.080631	1.940615	0.819716
TOM20	W4	HPI4	well_5	F004	197	Avg. Volume	3.857881	3.476571	1.667708
TOM20	W4	HPI4	well_5	F005	220	Avg. Volume	2.447522	2.221613	1.193879
TOM20	W4	HPI4	well_5	F006	177	Avg. Volume	4.226088	3.827649	1.686137
TOM20	W4	PGE2	well_1	F001	136	Avg. Volume	4.401145	4.096909	1.847898
TOM20	W4	PGE2	well_1	F002	197	Avg. Volume	4.730961	4.178056	2.444427
TOM20	W4	PGE2	well_1	F003	180	Avg. Volume	4.380176	3.827008	2.184775
TOM20	W4	PGE2	well_1	F004	228	Avg. Volume	3.241498	2.708633	1.851186
TOM20	W4	PGE2	well_1	F005	276	Avg. Volume	3.187779	2.830305	1.825579
TOM20	W4	PGE2	well_1	F006	121	Avg. Volume	3.414759	3.421728	1.263469
TOM20	W4	PGE2	well_2	F001	298	Avg. Volume	3.181824	2.832394	1.504044
TOM20	W4	PGE2	well_2	F002	357	Avg. Volume	4.093791	3.608854	2.203721
TOM20	W4	PGE2	well_2	F003	63	Avg. Volume	3.416089	3.229778	1.369692
TOM20	W4	PGE2	well_2	F004	244	Avg. Volume	4.583211	4.117806	2.240782
TOM20	W4	PGE2	well_2	F005	173	Avg. Volume	4.066225	3.96485	1.628678
TOM20	W4	PGE2	well_2	F006	77	Avg. Volume	3.09305	2.953232	1.132685
TOM20	W4	PGE2	well_3	F001	137	Avg. Volume	3.523612	3.184398	1.82932
TOM20	W4	PGE2	well_3	F002	335	Avg. Volume	4.092474	3.453025	2.443815
TOM20	W4	PGE2	well_3	F003	248	Avg. Volume	3.193847	2.742993	1.53786
TOM20	W4	PGE2	well_3	F004	327	Avg. Volume	3.567242	3.201611	1.706482
TOM20	W4	PGE2	well_3	F005	77	Avg. Volume	3.341427	2.968726	1.488493
TOM20	W4	PGE2	well_3	F006	90	Avg. Volume	3.401785	2.924906	1.782272
TOM20	W4	PGE2	well_4	F001	179	Avg. Volume	5.062167	4.815718	2.342322
TOM20	W4	PGE2	well_4	F002	409	Avg. Volume	3.679589	3.415208	1.752797
TOM20	W4	PGE2	well_4	F003	296	Avg. Volume	4.680884	4.349878	2.080089
TOM20	W4	PGE2	well_4	F004	386	Avg. Volume	3.954181	3.761936	1.817537
TOM20	W4	PGE2	well_4	F005	417	Avg. Volume	4.863866	4.295417	2.346933
TOM20	W4	PGE2	well_4	F006	192	Avg. Volume	4.740013	4.20231	2.330748
TOM20	W4	PGE2	well_5	F001	220	Avg. Volume	2.460281	2.265893	1.130737
TOM20	W4	PGE2	well_5	F002	228	Avg. Volume	3.846667	3.765911	1.732226

TOM20	W4	PGE2	well_5	F003	341	Avg. Volume	4.456765	4.046887	1.932266
TOM20	W4	PGE2	well_5	F004	398	Avg. Volume	4.62159	4.371666	2.107194
TOM20	W4	PGE2	well_5	F005	277	Avg. Volume	4.516026	4.04309	2.110384
TOM20	W4	PGE2	well_5	F006	268	Avg. Volume	4.202548	3.890321	2.003979
TOM20	W1	HPI4	well_1	F001	210	Count/cell	36.2766	36.5	10.22151
TOM20	W1	HPI4	well_1	F002	255	Count/cell	32.93013	32	12.81154
TOM20	W1	HPI4	well_1	F003	300	Count/cell	31.15556	31	9.987253
TOM20	W1	HPI4	well_1	F004	232	Count/cell	33.74163	33	9.321204
TOM20	W1	HPI4	well_1	F005	317	Count/cell	31.35889	31	10.13649
TOM20	W1	HPI4	well_1	F006	182	Count/cell	32.68072	34	11.11049
TOM20	W1	HPI4	well_2	F001	267	Count/cell	35.25833	35.5	10.44251
TOM20	W1	HPI4	well_2	F002	241	Count/cell	35.94037	36	10.33499
TOM20	W1	HPI4	well_2	F003	231	Count/cell	30.25943	31	8.893453
TOM20	W1	HPI4	well_2	F004	185	Count/cell	29.95833	30	10.25212
TOM20	W1	HPI4	well_2	F005	466	Count/cell	28.27553	28	9.260777
TOM20	W1	HPI4	well_2	F006	206	Count/cell	35.77297	36	10.01859
TOM20	W1	HPI4	well_3	F001	312	Count/cell	32.29078	31.5	10.37142
TOM20	W1	HPI4	well_3	F002	278	Count/cell	33.88492	33	10.11875
TOM20	W1	HPI4	well_3	F003	234	Count/cell	31.05687	31	9.705992
TOM20	W1	HPI4	well_3	F004	217	Count/cell	32.15816	31	9.984628
TOM20	W1	HPI4	well_3	F005	341	Count/cell	28.30421	28	9.495283
TOM20	W1	HPI4	well_3	F006	293	Count/cell	30.5	30	9.849145
TOM20	W1	HPI4	well_4	F001	244	Count/cell	29.0137	29	9.317282
TOM20	W1	HPI4	well_4	F002	313	Count/cell	30.64808	32	10.26391
TOM20	W1	HPI4	well_4	F003	176	Count/cell	28.63924	28	9.774019
TOM20	W1	HPI4	well_4	F004	175	Count/cell	25.61146	25	9.882417
TOM20	W1	HPI4	well_4	F005	351	Count/cell	26.55063	27	9.446624
TOM20	W1	HPI4	well_4	F006	306	Count/cell	30.25547	29.5	9.436915
TOM20	W1	HPI4	well_5	F001	243	Count/cell	29.22374	30	12.34408
TOM20	W1	HPI4	well_5	F002	220	Count/cell	26.43	26	8.20743
TOM20	W1	HPI4	well_5	F003	315	Count/cell	27.37324	27	9.348463
TOM20	W1	HPI4	well_5	F004	124	Count/cell	22.68468	22	7.718905
TOM20	W1	HPI4	well_5	F005	165	Count/cell	28.32886	30	9.631342
TOM20	W1	HPI4	well_5	F006	187	Count/cell	25.97006	25	8.879668
TOM20	W1	PGE2	well_1	F001	286	Count/cell	31.98833	31	11.83826
TOM20	W1	PGE2	well_1	F002	265	Count/cell	34.96624	34	10.45607
TOM20	W1	PGE2	well_1	F003	184	Count/cell	33.14286	33	8.871866
TOM20	W1	PGE2	well_1	F004	295	Count/cell	32.27881	31	10.45604
TOM20	W1	PGE2	well_1	F005	435	Count/cell	28.89873	29	9.854777
TOM20	W1	PGE2	well_1	F006	244	Count/cell	31.68919	31	9.82967
TOM20	W1	PGE2	well_2	F001	326	Count/cell	32.06734	32	11.27672
TOM20	W1	PGE2	well_2	F002	246	Count/cell	35.20814	34	10.83606
TOM20	W1	PGE2	well_2	F003	350	Count/cell	29.97799	30	10.07149
TOM20	W1	PGE2	well_2	F004	269	Count/cell	32.71545	32	10.06835
TOM20	W1	PGE2	well_2	F005	334	Count/cell	30.90645	30	11.46096
TOM20	W1	PGE2	well_2	F006	290	Count/cell	32.40769	33	9.987402
TOM20	W1	PGE2	well_3	F001	312	Count/cell	35.02143	35	10.26597
TOM20	W1	PGE2	well_3	F002	328	Count/cell	32.79798	32	9.942721
TOM20	W1	PGE2	well_3	F003	249	Count/cell	32.87611	34	9.906795

TOM20	W1	PGE2	well_3	F004	293	Count/cell	31.82707	31	10.48018
TOM20	W1	PGE2	well_3	F005	316	Count/cell	32.38112	31	10.46355
TOM20	W1	PGE2	well_3	F006	251	Count/cell	33.23348	34	10.78483
TOM20	W1	PGE2	well_4	F001	265	Count/cell	34.10549	35	10.67199
TOM20	W1	PGE2	well_4	F002	268	Count/cell	32.76033	32	11.57872
TOM20	W1	PGE2	well_4	F003	284	Count/cell	32.17121	32	9.362589
TOM20	W1	PGE2	well_4	F004	289	Count/cell	34.28077	34	10.5242
TOM20	W1	PGE2	well_4	F005	201	Count/cell	34.67403	33	10.52504
TOM20	W1	PGE2	well_4	F006	289	Count/cell	35.50192	35	11.8538
TOM20	W1	PGE2	well_5	F001	130	Count/cell	34.62069	33.5	11.46083
TOM20	W1	PGE2	well_5	F002	191	Count/cell	38.05848	38	11.02762
TOM20	W1	PGE2	well_5	F003	277	Count/cell	33.30279	32	10.08186
TOM20	W1	PGE2	well_5	F004	384	Count/cell	27.96839	28	10.37658
TOM20	W1	PGE2	well_5	F005	253	Count/cell	31.25	31	9.550872
TOM20	W1	PGE2	well_5	F006	146	Count/cell	29.26515	29	9.023176
TOM20	W2	HPI4	well_1	F001	253	Count/cell	22.47826	22	9.249428
TOM20	W2	HPI4	well_1	F002	159	Count/cell	29.37241	29	9.72964
TOM20	W2	HPI4	well_1	F003	177	Count/cell	31.93125	30	12.01316
TOM20	W2	HPI4	well_1	F004	419	Count/cell	27.70619	27	11.54191
TOM20	W2	HPI4	well_1	F005	229	Count/cell	29.37073	28	10.04203
TOM20	W2	HPI4	well_1	F006	159	Count/cell	28.57639	26.5	9.754741
TOM20	W2	HPI4	well_2	F001	220	Count/cell	23.55276	23	8.371186
TOM20	W2	HPI4	well_2	F002	234	Count/cell	25.0093	25	9.248385
TOM20	W2	HPI4	well_2	F003	234	Count/cell	32.87204	31	13.79985
TOM20	W2	HPI4	well_2	F004	283	Count/cell	29.29008	28	10.99076
TOM20	W2	HPI4	well_2	F005	274	Count/cell	29.57371	29	10.29551
TOM20	W2	HPI4	well_2	F006	252	Count/cell	27.16157	27	9.842403
TOM20	W2	HPI4	well_3	F001	294	Count/cell	27.70412	27	7.954198
TOM20	W2	HPI4	well_3	F002	273	Count/cell	22.5813	21	9.562229
TOM20	W2	HPI4	well_3	F003	219	Count/cell	30.37	31	12.22663
TOM20	W2	HPI4	well_3	F004	265	Count/cell	29.58577	30	9.70099
TOM20	W2	HPI4	well_3	F005	194	Count/cell	27.57955	26.5	9.608593
TOM20	W2	HPI4	well_3	F006	281	Count/cell	26.79528	26.5	9.676457
TOM20	W2	HPI4	well_4	F001	200	Count/cell	23.04396	22	10.88758
TOM20	W2	HPI4	well_4	F002	369	Count/cell	20.7515	20	7.598873
TOM20	W2	HPI4	well_4	F003	410	Count/cell	20.00267	19	8.572823
TOM20	W2	HPI4	well_4	F004	455	Count/cell	21.84819	21	8.796881
TOM20	W2	HPI4	well_4	F005	292	Count/cell	24.9283	24	10.78081
TOM20	W2	HPI4	well_4	F006	259	Count/cell	24.40254	24.5	9.393243
TOM20	W2	HPI4	well_5	F001	250	Count/cell	22.41518	22	9.387614
TOM20	W2	HPI4	well_5	F002	196	Count/cell	29.65169	28	10.92202
TOM20	W2	HPI4	well_5	F003	321	Count/cell	32.43793	32	11.69796
TOM20	W2	HPI4	well_5	F004	448	Count/cell	29.23587	29	11.44653
TOM20	W2	HPI4	well_5	F005	319	Count/cell	26.7457	26	9.40931
TOM20	W2	HPI4	well_5	F006	269	Count/cell	24.00823	23	8.313084
TOM20	W2	PGE2	well_1	F001	186	Count/cell	21.625	21	6.61036
TOM20	W2	PGE2	well_1	F002	233	Count/cell	20.64286	20	6.946898
TOM20	W2	PGE2	well_1	F003	443	Count/cell	21.36543	21	6.787057
TOM20	W2	PGE2	well_1	F004	70	Count/cell	21.27419	21	7.263584

TOM20	W2	PGE2	well_1	F005	190	Count/cell	22.4186	21.5	8.925245
TOM20	W2	PGE2	well_1	F006	211	Count/cell	22.33158	22	7.045623
TOM20	W2	PGE2	well_2	F001	424	Count/cell	17.9375	17	6.398943
TOM20	W2	PGE2	well_2	F002	268	Count/cell	18.51867	18	6.422021
TOM20	W2	PGE2	well_2	F003	595	Count/cell	20.85688	21	7.778197
TOM20	W2	PGE2	well_2	F004	512	Count/cell	20.88793	21	7.40121
TOM20	W2	PGE2	well_2	F005	224	Count/cell	19.25	19	6.668893
TOM20	W2	PGE2	well_2	F006	91	Count/cell	19.81707	20	6.724064
TOM20	W2	PGE2	well_3	F001	399	Count/cell	17.08056	16	6.031178
TOM20	W2	PGE2	well_3	F002	454	Count/cell	18.73284	18	6.60762
TOM20	W2	PGE2	well_3	F003	289	Count/cell	20.96538	21	6.095346
TOM20	W2	PGE2	well_3	F004	475	Count/cell	22.86916	22	7.518389
TOM20	W2	PGE2	well_3	F005	308	Count/cell	21.90391	21	7.744677
TOM20	W2	PGE2	well_3	F006	312	Count/cell	21.18662	21	6.474497
TOM20	W2	PGE2	well_4	F001	463	Count/cell	19.07876	19	6.684339
TOM20	W2	PGE2	well_4	F002	355	Count/cell	19.4375	19	6.245939
TOM20	W2	PGE2	well_4	F003	336	Count/cell	21.31148	21	6.55177
TOM20	W2	PGE2	well_4	F004	479	Count/cell	21.34174	21	6.309285
TOM20	W2	PGE2	well_4	F005	511	Count/cell	20.36774	20	5.932108
TOM20	W2	PGE2	well_4	F006	473	Count/cell	20.11765	20	6.750981
TOM20	W2	PGE2	well_5	F001	401	Count/cell	17.10959	17	5.761662
TOM20	W2	PGE2	well_5	F002	370	Count/cell	19.32456	19	7.22836
TOM20	W2	PGE2	well_5	F003	452	Count/cell	22.33654	22	7.15624
TOM20	W2	PGE2	well_5	F004	396	Count/cell	23.55679	23	7.474974
TOM20	W2	PGE2	well_5	F005	344	Count/cell	22.83013	22	6.73141
TOM20	W2	PGE2	well_5	F006	360	Count/cell	21.47734	21	6.968888
TOM20	W3	HPI4	well_1	F001	92	Count/cell	34.54217	34	14.47395
TOM20	W3	HPI4	well_1	F002	87	Count/cell	24.87013	24	9.212907
TOM20	W3	HPI4	well_1	F003	120	Count/cell	29.69725	30	10.73201
TOM20	W3	HPI4	well_1	F004	66	Count/cell	25	23.5	10.48642
TOM20	W3	HPI4	well_1	F005	165	Count/cell	25.16327	24	7.73007
TOM20	W3	HPI4	well_1	F006	32	Count/cell	29	24.5	15.3019
TOM20	W3	HPI4	well_2	F001	64	Count/cell	24.7193	24	8.465802
TOM20	W3	HPI4	well_2	F002	138	Count/cell	26.97581	25	10.91167
TOM20	W3	HPI4	well_2	F003	154	Count/cell	30.78261	30	10.53097
TOM20	W3	HPI4	well_2	F004	219	Count/cell	28.70202	28	10.3378
TOM20	W3	HPI4	well_2	F005	180	Count/cell	29.58025	28.5	10.07471
TOM20	W3	HPI4	well_2	F006	181	Count/cell	27	26	10.39939
TOM20	W3	HPI4	well_3	F001	110	Count/cell	32.18182	31	12.11708
TOM20	W3	HPI4	well_3	F002	130	Count/cell	33.36752	33	9.375866
TOM20	W3	HPI4	well_3	F003	159	Count/cell	30.04196	29	9.663984
TOM20	W3	HPI4	well_3	F004	207	Count/cell	29.96257	29	10.32971
TOM20	W3	HPI4	well_3	F005	50	Count/cell	29.84091	30	7.733777
TOM20	W3	HPI4	well_3	F006	77	Count/cell	24.08571	22	9.564037
TOM20	W3	HPI4	well_4	F001	128	Count/cell	24.57018	24	11.16091
TOM20	W3	HPI4	well_4	F002	141	Count/cell	29.18605	28	11.00977
TOM20	W3	HPI4	well_4	F003	232	Count/cell	26.11005	25	9.685554
TOM20	W3	HPI4	well_4	F004	148	Count/cell	27.06061	27	8.813381
TOM20	W3	HPI4	well_4	F005	163	Count/cell	29.75342	29	9.141464

TOM20	W3	HPI4	well_4	F006	251	Count/cell	25.52193	24	11.09012
TOM20	W3	HPI4	well_5	F001	127	Count/cell	25.72566	26	9.541496
TOM20	W3	HPI4	well_5	F002	152	Count/cell	28.62319	27	11.59172
TOM20	W3	HPI4	well_5	F003	318	Count/cell	25.27178	25	9.453382
TOM20	W3	HPI4	well_5	F004	110	Count/cell	26.48485	26	9.855449
TOM20	W3	HPI4	well_5	F005	239	Count/cell	27.71628	27	9.088787
TOM20	W3	HPI4	well_5	F006	59	Count/cell	32.75472	31	12.27397
TOM20	W3	PGE2	well_1	F001	287	Count/cell	18.11154	17	6.217973
TOM20	W3	PGE2	well_1	F002	378	Count/cell	20.97118	20	7.239046
TOM20	W3	PGE2	well_1	F003	324	Count/cell	19.70748	18	7.767273
TOM20	W3	PGE2	well_1	F004	454	Count/cell	17.30602	17	5.269577
TOM20	W3	PGE2	well_1	F005	421	Count/cell	15.2739	15	4.834828
TOM20	W3	PGE2	well_1	F006	241	Count/cell	16.21005	15	5.925508
TOM20	W3	PGE2	well_2	F001	299	Count/cell	18.5	18	5.734598
TOM20	W3	PGE2	well_2	F002	369	Count/cell	19.73964	18	7.700069
TOM20	W3	PGE2	well_2	F003	402	Count/cell	16.3168	16	5.105129
TOM20	W3	PGE2	well_2	F004	460	Count/cell	14.95238	15	4.451555
TOM20	W3	PGE2	well_2	F005	438	Count/cell	15.89356	15	5.239766
TOM20	W3	PGE2	well_2	F006	353	Count/cell	19.18438	19	5.864756
TOM20	W3	PGE2	well_3	F001	277	Count/cell	17.10277	16	5.183662
TOM20	W3	PGE2	well_3	F002	273	Count/cell	17.46032	17	6.513426
TOM20	W3	PGE2	well_3	F003	424	Count/cell	15.39642	15	5.38363
TOM20	W3	PGE2	well_3	F004	481	Count/cell	16.31121	16	5.238284
TOM20	W3	PGE2	well_3	F005	432	Count/cell	18.00501	17	6.015472
TOM20	W3	PGE2	well_3	F006	292	Count/cell	20	19	6.133378
TOM20	W3	PGE2	well_4	F001	402	Count/cell	17.79501	17	5.346035
TOM20	W3	PGE2	well_4	F002	358	Count/cell	17.05573	17	5.600299
TOM20	W3	PGE2	well_4	F003	257	Count/cell	17.6309	17	5.456828
TOM20	W3	PGE2	well_4	F004	374	Count/cell	21.06785	21	7.277964
TOM20	W3	PGE2	well_4	F005	384	Count/cell	21.23164	21	6.126847
TOM20	W3	PGE2	well_4	F006	253	Count/cell	19.90789	19.5	6.166938
TOM20	W3	PGE2	well_5	F001	116	Count/cell	16.25472	16	5.112191
TOM20	W3	PGE2	well_5	F002	157	Count/cell	18.61644	17	6.25687
TOM20	W3	PGE2	well_5	F003	160	Count/cell	18.11724	17	6.178484
TOM20	W3	PGE2	well_5	F004	364	Count/cell	17.83891	17	6.64713
TOM20	W3	PGE2	well_5	F005	367	Count/cell	15.9256	16	4.942397
TOM20	W3	PGE2	well_5	F006	253	Count/cell	18.21277	18	6.086752
TOM20	W4	HPI4	well_1	F001	60	Count/cell	37.65455	38	15.2026
TOM20	W4	HPI4	well_1	F002	159	Count/cell	36.62759	36	10.71988
TOM20	W4	HPI4	well_1	F003	130	Count/cell	30.62931	30	9.288169
TOM20	W4	HPI4	well_1	F004	179	Count/cell	36.28395	36	10.52797
TOM20	W4	HPI4	well_1	F005	141	Count/cell	38.09449	36	13.89612
TOM20	W4	HPI4	well_1	F006	130	Count/cell	33.25	31	10.27185
TOM20	W4	HPI4	well_2	F001	158	Count/cell	38.28873	38	10.15873
TOM20	W4	HPI4	well_2	F002	191	Count/cell	33.41714	33	10.11388
TOM20	W4	HPI4	well_2	F003	211	Count/cell	33.42408	33	10.94644
TOM20	W4	HPI4	well_2	F004	192	Count/cell	35.3908	34.5	12.08291
TOM20	W4	HPI4	well_2	F005	152	Count/cell	35.16912	34	10.29387
TOM20	W4	HPI4	well_2	F006	115	Count/cell	34.17476	33	12.95121

TOM20	W4	HPI4	well_3	F001	109	Count/cell	31.43878	31	9.189939
TOM20	W4	HPI4	well_3	F002	140	Count/cell	42.00787	41	11.45626
TOM20	W4	HPI4	well_3	F003	180	Count/cell	40.47561	40	13.31333
TOM20	W4	HPI4	well_3	F004	241	Count/cell	35.46119	35	11.49121
TOM20	W4	HPI4	well_3	F005	208	Count/cell	35.81183	36.5	10.1194
TOM20	W4	HPI4	well_3	F006	159	Count/cell	36.36806	35	11.02936
TOM20	W4	HPI4	well_4	F001	151	Count/cell	32.13869	31	11.34109
TOM20	W4	HPI4	well_4	F002	185	Count/cell	36.93373	36	11.3111
TOM20	W4	HPI4	well_4	F003	207	Count/cell	36.22222	36	12.57968
TOM20	W4	HPI4	well_4	F004	170	Count/cell	43.63158	43.5	11.44274
TOM20	W4	HPI4	well_4	F005	172	Count/cell	37.14013	36	11.36992
TOM20	W4	HPI4	well_4	F006	168	Count/cell	33.32237	33	9.099598
TOM20	W4	HPI4	well_5	F001	177	Count/cell	34.18012	33	11.93361
TOM20	W4	HPI4	well_5	F002	101	Count/cell	40.68478	37	18.53297
TOM20	W4	HPI4	well_5	F003	244	Count/cell	35.26484	36	11.65573
TOM20	W4	HPI4	well_5	F004	197	Count/cell	40.11111	37.5	14.55519
TOM20	W4	HPI4	well_5	F005	220	Count/cell	38.64	36.5	13.54317
TOM20	W4	HPI4	well_5	F006	177	Count/cell	34.66667	36	9.719266
TOM20	W4	PGE2	well_1	F001	136	Count/cell	16.79508	16	4.638927
TOM20	W4	PGE2	well_1	F002	197	Count/cell	13.77901	14	4.878046
TOM20	W4	PGE2	well_1	F003	180	Count/cell	16.87647	16	5.494702
TOM20	W4	PGE2	well_1	F004	228	Count/cell	14.41546	14	4.884228
TOM20	W4	PGE2	well_1	F005	276	Count/cell	14.33735	14	5.211519
TOM20	W4	PGE2	well_1	F006	121	Count/cell	17.00909	17	4.503812
TOM20	W4	PGE2	well_2	F001	298	Count/cell	16.56827	16	6.284162
TOM20	W4	PGE2	well_2	F002	357	Count/cell	11.94479	11	4.466286
TOM20	W4	PGE2	well_2	F003	63	Count/cell	15.01818	15	3.659037
TOM20	W4	PGE2	well_2	F004	244	Count/cell	16.06364	15	5.459318
TOM20	W4	PGE2	well_2	F005	173	Count/cell	16.93671	17	4.94129
TOM20	W4	PGE2	well_2	F006	77	Count/cell	18.80282	19	6.762543
TOM20	W4	PGE2	well_3	F001	137	Count/cell	17.53659	18	5.141112
TOM20	W4	PGE2	well_3	F002	335	Count/cell	12.12871	12	4.265983
TOM20	W4	PGE2	well_3	F003	248	Count/cell	17	16	4.995534
TOM20	W4	PGE2	well_3	F004	327	Count/cell	16.93939	16	5.264269
TOM20	W4	PGE2	well_3	F005	77	Count/cell	18.05634	18	5.666663
TOM20	W4	PGE2	well_3	F006	90	Count/cell	21.26829	21	8.393191
TOM20	W4	PGE2	well_4	F001	179	Count/cell	19.41358	18	7.053166
TOM20	W4	PGE2	well_4	F002	409	Count/cell	17.20955	16	6.940179
TOM20	W4	PGE2	well_4	F003	296	Count/cell	17.14925	17	5.342933
TOM20	W4	PGE2	well_4	F004	386	Count/cell	18.33048	17	6.424877
TOM20	W4	PGE2	well_4	F005	417	Count/cell	16.84555	17	5.28949
TOM20	W4	PGE2	well_4	F006	192	Count/cell	16.61236	16	5.894675
TOM20	W4	PGE2	well_5	F001	220	Count/cell	19.99005	20	7.765301
TOM20	W4	PGE2	well_5	F002	228	Count/cell	14.93301	15	4.755089
TOM20	W4	PGE2	well_5	F003	341	Count/cell	16.76656	16	5.056242
TOM20	W4	PGE2	well_5	F004	398	Count/cell	17.53973	17	5.318089
TOM20	W4	PGE2	well_5	F005	277	Count/cell	18.64844	18.5	5.81491
TOM20	W4	PGE2	well_5	F006	268	Count/cell	17.62295	17	5.628159
TOM20	W1	HPI4	well_1	F001	210	X/Y distribution	6.965462	6.856016	1.15071

TOM20	W1	HPI4	well_1	F002	255	X/Y distribution	6.961209	6.894203	1.178805
TOM20	W1	HPI4	well_1	F003	300	X/Y distribution	6.589156	6.510964	1.060111
TOM20	W1	HPI4	well_1	F004	232	X/Y distribution	6.894518	6.787006	0.991466
TOM20	W1	HPI4	well_1	F005	317	X/Y distribution	6.65063	6.588239	1.224669
TOM20	W1	HPI4	well_1	F006	182	X/Y distribution	7.302957	7.196476	1.299447
TOM20	W1	HPI4	well_2	F001	267	X/Y distribution	6.79962	6.847759	1.014594
TOM20	W1	HPI4	well_2	F002	241	X/Y distribution	7.397989	7.367684	1.078945
TOM20	W1	HPI4	well_2	F003	231	X/Y distribution	7.37791	7.182454	1.323557
TOM20	W1	HPI4	well_2	F004	185	X/Y distribution	6.893054	6.771942	1.299061
TOM20	W1	HPI4	well_2	F005	466	X/Y distribution	5.78604	5.76449	0.80865
TOM20	W1	HPI4	well_2	F006	206	X/Y distribution	7.517858	7.358926	1.322954
TOM20	W1	HPI4	well_3	F001	312	X/Y distribution	6.775376	6.691072	1.154972
TOM20	W1	HPI4	well_3	F002	278	X/Y distribution	6.832292	6.718765	1.231089
TOM20	W1	HPI4	well_3	F003	234	X/Y distribution	7.244709	7.153016	1.130602
TOM20	W1	HPI4	well_3	F004	217	X/Y distribution	6.856273	6.72782	1.208011
TOM20	W1	HPI4	well_3	F005	341	X/Y distribution	6.115772	5.981775	0.944904
TOM20	W1	HPI4	well_3	F006	293	X/Y distribution	6.36187	6.386638	0.968367
TOM20	W1	HPI4	well_4	F001	244	X/Y distribution	6.434414	6.259261	1.129469
TOM20	W1	HPI4	well_4	F002	313	X/Y distribution	6.482357	6.358219	0.99848
TOM20	W1	HPI4	well_4	F003	176	X/Y distribution	7.449519	7.316676	1.206992
TOM20	W1	HPI4	well_4	F004	175	X/Y distribution	6.335498	6.400601	1.096487
TOM20	W1	HPI4	well_4	F005	351	X/Y distribution	6.105129	6.026884	1.063909
TOM20	W1	HPI4	well_4	F006	306	X/Y distribution	6.490368	6.442713	1.014947
TOM20	W1	HPI4	well_5	F001	243	X/Y distribution	6.196212	6.18613	1.175681
TOM20	W1	HPI4	well_5	F002	220	X/Y distribution	6.337052	6.242619	1.057416
TOM20	W1	HPI4	well_5	F003	315	X/Y distribution	6.032613	5.965004	1.013672
TOM20	W1	HPI4	well_5	F004	124	X/Y distribution	5.881273	5.653157	1.018097
TOM20	W1	HPI4	well_5	F005	165	X/Y distribution	6.732701	6.751009	1.165427
TOM20	W1	HPI4	well_5	F006	187	X/Y distribution	6.371614	6.231181	1.338108
TOM20	W1	PGE2	well_1	F001	286	X/Y distribution	7.185185	6.948523	1.41362
TOM20	W1	PGE2	well_1	F002	265	X/Y distribution	6.888824	6.842805	1.14974
TOM20	W1	PGE2	well_1	F003	184	X/Y distribution	7.938371	7.917074	1.127901
TOM20	W1	PGE2	well_1	F004	295	X/Y distribution	6.72673	6.585356	1.250359
TOM20	W1	PGE2	well_1	F005	435	X/Y distribution	6.005554	5.975114	0.942146
TOM20	W1	PGE2	well_1	F006	244	X/Y distribution	7.110081	7.036984	1.225118
TOM20	W1	PGE2	well_2	F001	326	X/Y distribution	6.465744	6.381888	1.172851
TOM20	W1	PGE2	well_2	F002	246	X/Y distribution	7.118488	7.000738	1.114987
TOM20	W1	PGE2	well_2	F003	350	X/Y distribution	6.389118	6.330822	1.054879
TOM20	W1	PGE2	well_2	F004	269	X/Y distribution	7.068591	6.971825	1.086478
TOM20	W1	PGE2	well_2	F005	334	X/Y distribution	6.56596	6.584764	1.149863
TOM20	W1	PGE2	well_2	F006	290	X/Y distribution	6.9279	6.868628	1.262884
TOM20	W1	PGE2	well_3	F001	312	X/Y distribution	6.575199	6.506714	1.070254
TOM20	W1	PGE2	well_3	F002	328	X/Y distribution	6.649467	6.602116	1.010575
TOM20	W1	PGE2	well_3	F003	249	X/Y distribution	7.005862	6.916338	1.20779
TOM20	W1	PGE2	well_3	F004	293	X/Y distribution	6.768355	6.63166	1.1214
TOM20	W1	PGE2	well_3	F005	316	X/Y distribution	6.665606	6.563597	1.169626
TOM20	W1	PGE2	well_3	F006	251	X/Y distribution	7.135233	7.074779	1.428103
TOM20	W1	PGE2	well_4	F001	265	X/Y distribution	6.980686	6.904939	1.232421
TOM20	W1	PGE2	well_4	F002	268	X/Y distribution	6.90426	6.962323	1.249919

TOM20	W1	PGE2	well_4	F003	284	X/Y distribution	6.640913	6.525347	0.857992
TOM20	W1	PGE2	well_4	F004	289	X/Y distribution	6.84422	6.75488	1.060797
TOM20	W1	PGE2	well_4	F005	201	X/Y distribution	7.62402	7.47053	1.30147
TOM20	W1	PGE2	well_4	F006	289	X/Y distribution	6.852131	6.800644	1.04442
TOM20	W1	PGE2	well_5	F001	130	X/Y distribution	7.773423	7.7844	1.484252
TOM20	W1	PGE2	well_5	F002	191	X/Y distribution	7.521866	7.328022	1.217477
TOM20	W1	PGE2	well_5	F003	277	X/Y distribution	7.028104	6.927008	1.109654
TOM20	W1	PGE2	well_5	F004	384	X/Y distribution	6.183661	6.116958	1.127374
TOM20	W1	PGE2	well_5	F005	253	X/Y distribution	6.81264	6.690718	1.166909
TOM20	W1	PGE2	well_5	F006	146	X/Y distribution	7.407761	7.288006	1.241626
TOM20	W2	HPI4	well_1	F001	253	X/Y distribution	6.867635	6.667645	1.358543
TOM20	W2	HPI4	well_1	F002	159	X/Y distribution	8.029405	7.987344	1.114869
TOM20	W2	HPI4	well_1	F003	177	X/Y distribution	8.072935	8.073332	1.420679
TOM20	W2	HPI4	well_1	F004	419	X/Y distribution	6.177016	6.215451	1.325249
TOM20	W2	HPI4	well_1	F005	229	X/Y distribution	7.518986	7.490772	1.117813
TOM20	W2	HPI4	well_1	F006	159	X/Y distribution	7.873203	7.834247	1.384826
TOM20	W2	HPI4	well_2	F001	220	X/Y distribution	7.113556	7.092699	1.118253
TOM20	W2	HPI4	well_2	F002	234	X/Y distribution	7.16798	7.129721	1.281835
TOM20	W2	HPI4	well_2	F003	234	X/Y distribution	7.288016	7.11843	1.575301
TOM20	W2	HPI4	well_2	F004	283	X/Y distribution	7.085427	7.154463	1.235104
TOM20	W2	HPI4	well_2	F005	274	X/Y distribution	7.028778	6.954043	1.074381
TOM20	W2	HPI4	well_2	F006	252	X/Y distribution	7.009829	7.074674	1.169246
TOM20	W2	HPI4	well_3	F001	294	X/Y distribution	6.826523	6.744804	0.956991
TOM20	W2	HPI4	well_3	F002	273	X/Y distribution	6.516252	6.501153	1.285663
TOM20	W2	HPI4	well_3	F003	219	X/Y distribution	7.667001	7.57206	1.622028
TOM20	W2	HPI4	well_3	F004	265	X/Y distribution	7.245477	7.213981	1.314385
TOM20	W2	HPI4	well_3	F005	194	X/Y distribution	7.738495	7.599533	1.201741
TOM20	W2	HPI4	well_3	F006	281	X/Y distribution	7.016702	6.824781	1.487511
TOM20	W2	HPI4	well_4	F001	200	X/Y distribution	6.908854	6.83127	1.156459
TOM20	W2	HPI4	well_4	F002	369	X/Y distribution	5.983767	5.961217	1.051323
TOM20	W2	HPI4	well_4	F003	410	X/Y distribution	5.705389	5.684347	1.173589
TOM20	W2	HPI4	well_4	F004	455	X/Y distribution	6.026896	5.979537	1.087325
TOM20	W2	HPI4	well_4	F005	292	X/Y distribution	6.719296	6.549985	1.51449
TOM20	W2	HPI4	well_4	F006	259	X/Y distribution	6.888568	6.818796	1.36322
TOM20	W2	HPI4	well_5	F001	250	X/Y distribution	7.063139	6.906403	1.302558
TOM20	W2	HPI4	well_5	F002	196	X/Y distribution	8.085492	7.987047	1.553472
TOM20	W2	HPI4	well_5	F003	321	X/Y distribution	6.676187	6.581684	1.275552
TOM20	W2	HPI4	well_5	F004	448	X/Y distribution	6.180352	6.187605	1.25152
TOM20	W2	HPI4	well_5	F005	319	X/Y distribution	6.682446	6.650118	1.049076
TOM20	W2	HPI4	well_5	F006	269	X/Y distribution	6.785513	6.769352	1.099944
TOM20	W2	PGE2	well_1	F001	186	X/Y distribution	4.861088	4.784479	0.731861
TOM20	W2	PGE2	well_1	F002	233	X/Y distribution	4.852144	4.722191	0.759805
TOM20	W2	PGE2	well_1	F003	443	X/Y distribution	4.881772	4.894202	0.65242
TOM20	W2	PGE2	well_1	F004	70	X/Y distribution	4.731259	4.749911	0.547292
TOM20	W2	PGE2	well_1	F005	190	X/Y distribution	4.978854	4.973422	0.838757
TOM20	W2	PGE2	well_1	F006	211	X/Y distribution	4.556169	4.491755	0.628003
TOM20	W2	PGE2	well_2	F001	424	X/Y distribution	4.526823	4.51621	0.592728
TOM20	W2	PGE2	well_2	F002	268	X/Y distribution	4.513961	4.420819	0.634417
TOM20	W2	PGE2	well_2	F003	595	X/Y distribution	4.783161	4.710151	0.820203

TOM20	W2	PGE2	well_2	F004	512	X/Y distribution	4.69493	4.672796	0.746873
TOM20	W2	PGE2	well_2	F005	224	X/Y distribution	4.750574	4.706702	0.775657
TOM20	W2	PGE2	well_2	F006	91	X/Y distribution	4.676325	4.66597	0.741725
TOM20	W2	PGE2	well_3	F001	399	X/Y distribution	4.594151	4.533829	0.712153
TOM20	W2	PGE2	well_3	F002	454	X/Y distribution	4.751581	4.726414	0.733852
TOM20	W2	PGE2	well_3	F003	289	X/Y distribution	4.813059	4.778128	0.701592
TOM20	W2	PGE2	well_3	F004	475	X/Y distribution	4.772493	4.728593	0.652821
TOM20	W2	PGE2	well_3	F005	308	X/Y distribution	5.013241	4.963111	0.747536
TOM20	W2	PGE2	well_3	F006	312	X/Y distribution	4.692072	4.692885	0.635118
TOM20	W2	PGE2	well_4	F001	463	X/Y distribution	4.789447	4.753678	0.739032
TOM20	W2	PGE2	well_4	F002	355	X/Y distribution	4.680259	4.630135	0.695543
TOM20	W2	PGE2	well_4	F003	336	X/Y distribution	4.705925	4.606607	0.682749
TOM20	W2	PGE2	well_4	F004	479	X/Y distribution	4.576153	4.606482	0.551325
TOM20	W2	PGE2	well_4	F005	511	X/Y distribution	4.724384	4.693704	0.577456
TOM20	W2	PGE2	well_4	F006	473	X/Y distribution	4.809112	4.795512	0.764645
TOM20	W2	PGE2	well_5	F001	401	X/Y distribution	4.670268	4.615149	0.752627
TOM20	W2	PGE2	well_5	F002	370	X/Y distribution	4.847745	4.789878	0.74961
TOM20	W2	PGE2	well_5	F003	452	X/Y distribution	5.02956	4.988436	0.677944
TOM20	W2	PGE2	well_5	F004	396	X/Y distribution	4.980619	4.958693	0.647826
TOM20	W2	PGE2	well_5	F005	344	X/Y distribution	4.928269	4.891806	0.594402
TOM20	W2	PGE2	well_5	F006	360	X/Y distribution	4.780946	4.726706	0.63137
TOM20	W3	HPI4	well_1	F001	92	X/Y distribution	7.974272	7.782933	1.836492
TOM20	W3	HPI4	well_1	F002	87	X/Y distribution	7.607718	7.414684	1.208998
TOM20	W3	HPI4	well_1	F003	120	X/Y distribution	7.774607	7.514337	1.343271
TOM20	W3	HPI4	well_1	F004	66	X/Y distribution	7.033523	7.001604	1.353646
TOM20	W3	HPI4	well_1	F005	165	X/Y distribution	7.340201	7.407631	1.167566
TOM20	W3	HPI4	well_1	F006	32	X/Y distribution	8.328663	8.341899	1.748551
TOM20	W3	HPI4	well_2	F001	64	X/Y distribution	7.908986	7.837276	1.207126
TOM20	W3	HPI4	well_2	F002	138	X/Y distribution	7.638504	7.572099	1.614299
TOM20	W3	HPI4	well_2	F003	154	X/Y distribution	7.369423	7.165544	1.515014
TOM20	W3	HPI4	well_2	F004	219	X/Y distribution	7.039651	6.909781	1.339184
TOM20	W3	HPI4	well_2	F005	180	X/Y distribution	7.711685	7.744151	1.173956
TOM20	W3	HPI4	well_2	F006	181	X/Y distribution	7.431928	7.283119	1.448355
TOM20	W3	HPI4	well_3	F001	110	X/Y distribution	8.314091	8.245335	1.246369
TOM20	W3	HPI4	well_3	F002	130	X/Y distribution	8.321259	8.252995	1.209451
TOM20	W3	HPI4	well_3	F003	159	X/Y distribution	7.873575	7.876947	1.442122
TOM20	W3	HPI4	well_3	F004	207	X/Y distribution	7.105302	6.943143	1.38367
TOM20	W3	HPI4	well_3	F005	50	X/Y distribution	7.750463	7.829613	1.077373
TOM20	W3	HPI4	well_3	F006	77	X/Y distribution	6.47579	6.527601	1.148663
TOM20	W3	HPI4	well_4	F001	128	X/Y distribution	7.334539	7.470667	1.587419
TOM20	W3	HPI4	well_4	F002	141	X/Y distribution	7.488332	7.362693	1.308239
TOM20	W3	HPI4	well_4	F003	232	X/Y distribution	7.19859	7.081482	1.499869
TOM20	W3	HPI4	well_4	F004	148	X/Y distribution	7.213257	7.154124	1.458796
TOM20	W3	HPI4	well_4	F005	163	X/Y distribution	7.51092	7.44823	1.203036
TOM20	W3	HPI4	well_4	F006	251	X/Y distribution	6.509885	6.419836	1.427781
TOM20	W3	HPI4	well_5	F001	127	X/Y distribution	7.624971	7.535595	1.342831
TOM20	W3	HPI4	well_5	F002	152	X/Y distribution	7.269378	7.409568	1.488459
TOM20	W3	HPI4	well_5	F003	318	X/Y distribution	6.34238	6.287209	1.139723
TOM20	W3	HPI4	well_5	F004	110	X/Y distribution	6.597294	6.339131	1.450443

TOM20	W3	HPI4	well_5	F005	239	X/Y distribution	6.533152	6.501823	1.249564
TOM20	W3	HPI4	well_5	F006	59	X/Y distribution	8.225956	8.374287	1.424135
TOM20	W3	PGE2	well_1	F001	287	X/Y distribution	4.742797	4.712585	0.695454
TOM20	W3	PGE2	well_1	F002	378	X/Y distribution	5.060221	5.056317	0.78745
TOM20	W3	PGE2	well_1	F003	324	X/Y distribution	4.891477	4.760177	0.892311
TOM20	W3	PGE2	well_1	F004	454	X/Y distribution	4.406506	4.350088	0.56366
TOM20	W3	PGE2	well_1	F005	421	X/Y distribution	4.46883	4.438913	0.692655
TOM20	W3	PGE2	well_1	F006	241	X/Y distribution	4.540714	4.527679	0.729087
TOM20	W3	PGE2	well_2	F001	299	X/Y distribution	4.62061	4.553962	0.805774
TOM20	W3	PGE2	well_2	F002	369	X/Y distribution	4.887173	4.784446	0.897823
TOM20	W3	PGE2	well_2	F003	402	X/Y distribution	4.736141	4.708074	0.705538
TOM20	W3	PGE2	well_2	F004	460	X/Y distribution	4.219142	4.225846	0.634854
TOM20	W3	PGE2	well_2	F005	438	X/Y distribution	4.241728	4.193803	0.589232
TOM20	W3	PGE2	well_2	F006	353	X/Y distribution	4.764268	4.754015	0.723655
TOM20	W3	PGE2	well_3	F001	277	X/Y distribution	4.542604	4.490336	0.676092
TOM20	W3	PGE2	well_3	F002	273	X/Y distribution	4.778759	4.607043	1.003729
TOM20	W3	PGE2	well_3	F003	424	X/Y distribution	4.355527	4.354344	0.631822
TOM20	W3	PGE2	well_3	F004	481	X/Y distribution	4.598422	4.531303	0.711502
TOM20	W3	PGE2	well_3	F005	432	X/Y distribution	4.531253	4.502839	0.671673
TOM20	W3	PGE2	well_3	F006	292	X/Y distribution	4.734836	4.677423	0.722478
TOM20	W3	PGE2	well_4	F001	402	X/Y distribution	4.485918	4.46923	0.559332
TOM20	W3	PGE2	well_4	F002	358	X/Y distribution	4.422173	4.345875	0.701245
TOM20	W3	PGE2	well_4	F003	257	X/Y distribution	4.748607	4.731131	0.695559
TOM20	W3	PGE2	well_4	F004	374	X/Y distribution	4.643111	4.595109	0.709142
TOM20	W3	PGE2	well_4	F005	384	X/Y distribution	4.782723	4.766041	0.566263
TOM20	W3	PGE2	well_4	F006	253	X/Y distribution	4.797681	4.740486	0.699238
TOM20	W3	PGE2	well_5	F001	116	X/Y distribution	4.541536	4.48364	0.608383
TOM20	W3	PGE2	well_5	F002	157	X/Y distribution	4.521597	4.311215	0.695844
TOM20	W3	PGE2	well_5	F003	160	X/Y distribution	4.537419	4.514393	0.712618
TOM20	W3	PGE2	well_5	F004	364	X/Y distribution	4.652423	4.596714	0.678298
TOM20	W3	PGE2	well_5	F005	367	X/Y distribution	4.469455	4.436514	0.629067
TOM20	W3	PGE2	well_5	F006	253	X/Y distribution	4.996293	4.941041	0.781743
TOM20	W4	HPI4	well_1	F001	60	X/Y distribution	8.47124	8.126886	1.679036
TOM20	W4	HPI4	well_1	F002	159	X/Y distribution	8.298621	8.268597	1.159084
TOM20	W4	HPI4	well_1	F003	130	X/Y distribution	7.648833	7.686754	1.061717
TOM20	W4	HPI4	well_1	F004	179	X/Y distribution	7.950317	7.945589	1.19841
TOM20	W4	HPI4	well_1	F005	141	X/Y distribution	7.987341	7.726207	1.527419
TOM20	W4	HPI4	well_1	F006	130	X/Y distribution	8.439978	8.413586	1.373865
TOM20	W4	HPI4	well_2	F001	158	X/Y distribution	8.407221	8.227065	1.132302
TOM20	W4	HPI4	well_2	F002	191	X/Y distribution	7.98853	8.058587	1.15517
TOM20	W4	HPI4	well_2	F003	211	X/Y distribution	7.629516	7.649566	1.391112
TOM20	W4	HPI4	well_2	F004	192	X/Y distribution	7.995064	8.078128	1.358
TOM20	W4	HPI4	well_2	F005	152	X/Y distribution	8.266349	8.298558	1.185269
TOM20	W4	HPI4	well_2	F006	115	X/Y distribution	8.903014	8.831392	1.540611
TOM20	W4	HPI4	well_3	F001	109	X/Y distribution	8.668121	8.591374	1.445733
TOM20	W4	HPI4	well_3	F002	140	X/Y distribution	8.473994	8.556756	1.210863
TOM20	W4	HPI4	well_3	F003	180	X/Y distribution	7.878642	7.946087	1.345613
TOM20	W4	HPI4	well_3	F004	241	X/Y distribution	7.560825	7.61745	1.294266
TOM20	W4	HPI4	well_3	F005	208	X/Y distribution	7.835109	7.759493	1.296792

TOM20	W4	HPI4	well_3	F006	159	X/Y distribution	8.585526	8.596981	1.123287
TOM20	W4	HPI4	well_4	F001	151	X/Y distribution	8.235536	8.197931	1.186248
TOM20	W4	HPI4	well_4	F002	185	X/Y distribution	8.009828	8.182938	1.160966
TOM20	W4	HPI4	well_4	F003	207	X/Y distribution	7.785801	7.885545	1.373992
TOM20	W4	HPI4	well_4	F004	170	X/Y distribution	8.390855	8.229082	1.084168
TOM20	W4	HPI4	well_4	F005	172	X/Y distribution	8.611878	8.661159	1.145147
TOM20	W4	HPI4	well_4	F006	168	X/Y distribution	8.15356	8.045489	0.991752
TOM20	W4	HPI4	well_5	F001	177	X/Y distribution	7.935894	7.835091	1.294457
TOM20	W4	HPI4	well_5	F002	101	X/Y distribution	8.399592	8.105803	1.973166
TOM20	W4	HPI4	well_5	F003	244	X/Y distribution	7.357646	7.335592	1.214828
TOM20	W4	HPI4	well_5	F004	197	X/Y distribution	8.22742	8.072782	1.530369
TOM20	W4	HPI4	well_5	F005	220	X/Y distribution	7.476155	7.314984	1.245977
TOM20	W4	HPI4	well_5	F006	177	X/Y distribution	8.2369	8.212778	1.077017
TOM20	W4	PGE2	well_1	F001	136	X/Y distribution	4.672285	4.607754	0.680747
TOM20	W4	PGE2	well_1	F002	197	X/Y distribution	4.647196	4.569651	0.647226
TOM20	W4	PGE2	well_1	F003	180	X/Y distribution	4.45119	4.359896	0.660112
TOM20	W4	PGE2	well_1	F004	228	X/Y distribution	4.449211	4.371317	0.67622
TOM20	W4	PGE2	well_1	F005	276	X/Y distribution	4.50802	4.394862	0.744816
TOM20	W4	PGE2	well_1	F006	121	X/Y distribution	4.381612	4.378927	0.563183
TOM20	W4	PGE2	well_2	F001	298	X/Y distribution	4.575077	4.510129	0.651156
TOM20	W4	PGE2	well_2	F002	357	X/Y distribution	4.469054	4.448934	0.761069
TOM20	W4	PGE2	well_2	F003	63	X/Y distribution	4.654055	4.592071	0.671981
TOM20	W4	PGE2	well_2	F004	244	X/Y distribution	4.625011	4.583921	0.720062
TOM20	W4	PGE2	well_2	F005	173	X/Y distribution	4.678423	4.642185	0.603379
TOM20	W4	PGE2	well_2	F006	77	X/Y distribution	4.728446	4.762163	0.54458
TOM20	W4	PGE2	well_3	F001	137	X/Y distribution	4.665388	4.604771	0.625512
TOM20	W4	PGE2	well_3	F002	335	X/Y distribution	4.385523	4.28005	0.67675
TOM20	W4	PGE2	well_3	F003	248	X/Y distribution	4.435863	4.348815	0.591819
TOM20	W4	PGE2	well_3	F004	327	X/Y distribution	4.520983	4.458445	0.555816
TOM20	W4	PGE2	well_3	F005	77	X/Y distribution	4.848184	4.816436	0.563625
TOM20	W4	PGE2	well_3	F006	90	X/Y distribution	5.160859	5.141737	0.791898
TOM20	W4	PGE2	well_4	F001	179	X/Y distribution	5.331632	5.110725	1.102937
TOM20	W4	PGE2	well_4	F002	409	X/Y distribution	4.694825	4.574816	0.827661
TOM20	W4	PGE2	well_4	F003	296	X/Y distribution	4.761255	4.681759	0.609996
TOM20	W4	PGE2	well_4	F004	386	X/Y distribution	4.986662	4.895852	0.751048
TOM20	W4	PGE2	well_4	F005	417	X/Y distribution	4.528888	4.470772	0.620399
TOM20	W4	PGE2	well_4	F006	192	X/Y distribution	4.834537	4.762619	0.702435
TOM20	W4	PGE2	well_5	F001	220	X/Y distribution	5.019987	4.980843	0.729376
TOM20	W4	PGE2	well_5	F002	228	X/Y distribution	4.708996	4.669576	0.688898
TOM20	W4	PGE2	well_5	F003	341	X/Y distribution	4.642537	4.601574	0.611385
TOM20	W4	PGE2	well_5	F004	398	X/Y distribution	4.660532	4.575861	0.6362
TOM20	W4	PGE2	well_5	F005	277	X/Y distribution	4.554009	4.555998	0.642394
TOM20	W4	PGE2	well_5	F006	268	X/Y distribution	4.571722	4.510072	0.592958
TOM20	W1	HPI4	well_1	F001	210	Z distribution	-0.47613	-0.45177	0.327976
TOM20	W1	HPI4	well_1	F002	255	Z distribution	-0.36228	-0.30361	0.349936
TOM20	W1	HPI4	well_1	F003	300	Z distribution	-0.56495	-0.53996	0.39213
TOM20	W1	HPI4	well_1	F004	232	Z distribution	-0.55737	-0.52591	0.358316
TOM20	W1	HPI4	well_1	F005	317	Z distribution	-0.486	-0.49709	0.380446
TOM20	W1	HPI4	well_1	F006	182	Z distribution	-0.47412	-0.44882	0.33296

TOM20	W1	HPI4	well_2	F001	267	Z distribution	-0.42464	-0.39177	0.38953
TOM20	W1	HPI4	well_2	F002	241	Z distribution	-0.41443	-0.34114	0.358734
TOM20	W1	HPI4	well_2	F003	231	Z distribution	-0.52328	-0.49274	0.333069
TOM20	W1	HPI4	well_2	F004	185	Z distribution	-0.54217	-0.54035	0.331428
TOM20	W1	HPI4	well_2	F005	466	Z distribution	-0.54449	-0.49557	0.448668
TOM20	W1	HPI4	well_2	F006	206	Z distribution	-0.48112	-0.50198	0.341761
TOM20	W1	HPI4	well_3	F001	312	Z distribution	-0.49628	-0.4341	0.416221
TOM20	W1	HPI4	well_3	F002	278	Z distribution	-0.50319	-0.47068	0.405402
TOM20	W1	HPI4	well_3	F003	234	Z distribution	-0.49475	-0.4514	0.329135
TOM20	W1	HPI4	well_3	F004	217	Z distribution	-0.57198	-0.47351	0.412983
TOM20	W1	HPI4	well_3	F005	341	Z distribution	-0.48952	-0.49409	0.376549
TOM20	W1	HPI4	well_3	F006	293	Z distribution	-0.47247	-0.44616	0.354113
TOM20	W1	HPI4	well_4	F001	244	Z distribution	-0.46898	-0.40585	0.383004
TOM20	W1	HPI4	well_4	F002	313	Z distribution	-0.45025	-0.44613	0.358471
TOM20	W1	HPI4	well_4	F003	176	Z distribution	-0.5448	-0.57275	0.311184
TOM20	W1	HPI4	well_4	F004	175	Z distribution	-0.54456	-0.50788	0.376795
TOM20	W1	HPI4	well_4	F005	351	Z distribution	-0.51604	-0.48682	0.399333
TOM20	W1	HPI4	well_4	F006	306	Z distribution	-0.48152	-0.44029	0.367457
TOM20	W1	HPI4	well_5	F001	243	Z distribution	-0.4507	-0.41628	0.35477
TOM20	W1	HPI4	well_5	F002	220	Z distribution	-0.39324	-0.332	0.356805
TOM20	W1	HPI4	well_5	F003	315	Z distribution	-0.65912	-0.64085	0.439346
TOM20	W1	HPI4	well_5	F004	124	Z distribution	-0.47903	-0.35954	0.363515
TOM20	W1	HPI4	well_5	F005	165	Z distribution	-0.46048	-0.44374	0.348241
TOM20	W1	HPI4	well_5	F006	187	Z distribution	-0.44145	-0.39942	0.33518
TOM20	W1	PGE2	well_1	F001	286	Z distribution	-0.35028	-0.34133	0.297116
TOM20	W1	PGE2	well_1	F002	265	Z distribution	-0.42366	-0.36826	0.317281
TOM20	W1	PGE2	well_1	F003	184	Z distribution	-0.59982	-0.54712	0.36869
TOM20	W1	PGE2	well_1	F004	295	Z distribution	-0.43173	-0.39583	0.345937
TOM20	W1	PGE2	well_1	F005	435	Z distribution	-0.50662	-0.47918	0.441229
TOM20	W1	PGE2	well_1	F006	244	Z distribution	-0.46423	-0.44202	0.340134
TOM20	W1	PGE2	well_2	F001	326	Z distribution	-0.42408	-0.38824	0.348005
TOM20	W1	PGE2	well_2	F002	246	Z distribution	-0.44714	-0.42913	0.349862
TOM20	W1	PGE2	well_2	F003	350	Z distribution	-0.48606	-0.46444	0.362495
TOM20	W1	PGE2	well_2	F004	269	Z distribution	-0.50478	-0.45551	0.37818
TOM20	W1	PGE2	well_2	F005	334	Z distribution	-0.40505	-0.39774	0.35025
TOM20	W1	PGE2	well_2	F006	290	Z distribution	-0.40324	-0.36923	0.350483
TOM20	W1	PGE2	well_3	F001	312	Z distribution	-0.44001	-0.42965	0.390196
TOM20	W1	PGE2	well_3	F002	328	Z distribution	-0.45336	-0.4348	0.376524
TOM20	W1	PGE2	well_3	F003	249	Z distribution	-0.43357	-0.38293	0.357407
TOM20	W1	PGE2	well_3	F004	293	Z distribution	-0.54025	-0.48175	0.420692
TOM20	W1	PGE2	well_3	F005	316	Z distribution	-0.35117	-0.34185	0.338218
TOM20	W1	PGE2	well_3	F006	251	Z distribution	-0.51908	-0.50513	0.3754
TOM20	W1	PGE2	well_4	F001	265	Z distribution	-0.41817	-0.41661	0.314896
TOM20	W1	PGE2	well_4	F002	268	Z distribution	-0.4947	-0.47476	0.345908
TOM20	W1	PGE2	well_4	F003	284	Z distribution	-0.50486	-0.47377	0.372388
TOM20	W1	PGE2	well_4	F004	289	Z distribution	-0.33766	-0.28358	0.341096
TOM20	W1	PGE2	well_4	F005	201	Z distribution	-0.46377	-0.4793	0.346839
TOM20	W1	PGE2	well_4	F006	289	Z distribution	-0.50994	-0.47169	0.384259
TOM20	W1	PGE2	well_5	F001	130	Z distribution	-0.49797	-0.48206	0.338918

TOM20	W1	PGE2	well_5	F002	191	Z distribution	-0.41626	-0.37423	0.338753
TOM20	W1	PGE2	well_5	F003	277	Z distribution	-0.46813	-0.4714	0.353248
TOM20	W1	PGE2	well_5	F004	384	Z distribution	-0.56969	-0.5266	0.426311
TOM20	W1	PGE2	well_5	F005	253	Z distribution	-0.44985	-0.40637	0.383285
TOM20	W1	PGE2	well_5	F006	146	Z distribution	-0.43313	-0.38818	0.307237
TOM20	W2	HPI4	well_1	F001	253	Z distribution	-0.85181	-0.92575	0.501506
TOM20	W2	HPI4	well_1	F002	159	Z distribution	-0.61515	-0.61559	0.461632
TOM20	W2	HPI4	well_1	F003	177	Z distribution	-0.65444	-0.65315	0.487273
TOM20	W2	HPI4	well_1	F004	419	Z distribution	-0.63671	-0.59041	0.529964
TOM20	W2	HPI4	well_1	F005	229	Z distribution	-0.67155	-0.68695	0.440503
TOM20	W2	HPI4	well_1	F006	159	Z distribution	-0.69636	-0.72609	0.437711
TOM20	W2	HPI4	well_2	F001	220	Z distribution	-0.67274	-0.69624	0.439133
TOM20	W2	HPI4	well_2	F002	234	Z distribution	-0.80864	-0.86186	0.553504
TOM20	W2	HPI4	well_2	F003	234	Z distribution	-0.74151	-0.79152	0.50799
TOM20	W2	HPI4	well_2	F004	283	Z distribution	-0.52021	-0.52651	0.480219
TOM20	W2	HPI4	well_2	F005	274	Z distribution	-0.6288	-0.61558	0.471073
TOM20	W2	HPI4	well_2	F006	252	Z distribution	-0.8662	-0.88929	0.52396
TOM20	W2	HPI4	well_3	F001	294	Z distribution	-0.78198	-0.81369	0.437178
TOM20	W2	HPI4	well_3	F002	273	Z distribution	-0.67451	-0.68895	0.440789
TOM20	W2	HPI4	well_3	F003	219	Z distribution	-0.64882	-0.6668	0.440147
TOM20	W2	HPI4	well_3	F004	265	Z distribution	-0.55306	-0.4864	0.429459
TOM20	W2	HPI4	well_3	F005	194	Z distribution	-0.68853	-0.70493	0.41276
TOM20	W2	HPI4	well_3	F006	281	Z distribution	-0.40572	-0.31336	0.408203
TOM20	W2	HPI4	well_4	F001	200	Z distribution	-0.57205	-0.49893	0.437919
TOM20	W2	HPI4	well_4	F002	369	Z distribution	-0.6813	-0.6883	0.486591
TOM20	W2	HPI4	well_4	F003	410	Z distribution	-0.61163	-0.56328	0.434271
TOM20	W2	HPI4	well_4	F004	455	Z distribution	-0.65359	-0.64846	0.54605
TOM20	W2	HPI4	well_4	F005	292	Z distribution	-0.64868	-0.60845	0.473679
TOM20	W2	HPI4	well_4	F006	259	Z distribution	-0.47851	-0.4323	0.410737
TOM20	W2	HPI4	well_5	F001	250	Z distribution	-0.51608	-0.49587	0.397582
TOM20	W2	HPI4	well_5	F002	196	Z distribution	-0.59005	-0.57077	0.406252
TOM20	W2	HPI4	well_5	F003	321	Z distribution	-0.64976	-0.66172	0.541882
TOM20	W2	HPI4	well_5	F004	448	Z distribution	-0.64743	-0.60295	0.511103
TOM20	W2	HPI4	well_5	F005	319	Z distribution	-0.49297	-0.41415	0.432401
TOM20	W2	HPI4	well_5	F006	269	Z distribution	-0.50639	-0.41481	0.452393
TOM20	W2	PGE2	well_1	F001	186	Z distribution	-0.90203	-0.87707	0.544445
TOM20	W2	PGE2	well_1	F002	233	Z distribution	-0.61311	-0.54627	0.542768
TOM20	W2	PGE2	well_1	F003	443	Z distribution	-0.69728	-0.67657	0.542521
TOM20	W2	PGE2	well_1	F004	70	Z distribution	-0.60221	-0.57256	0.404112
TOM20	W2	PGE2	well_1	F005	190	Z distribution	-0.62131	-0.54527	0.547647
TOM20	W2	PGE2	well_1	F006	211	Z distribution	-0.57452	-0.58612	0.552423
TOM20	W2	PGE2	well_2	F001	424	Z distribution	-0.77276	-0.78517	0.532337
TOM20	W2	PGE2	well_2	F002	268	Z distribution	-0.66419	-0.61031	0.480355
TOM20	W2	PGE2	well_2	F003	595	Z distribution	-0.46631	-0.40982	0.507051
TOM20	W2	PGE2	well_2	F004	512	Z distribution	-0.55973	-0.53459	0.421594
TOM20	W2	PGE2	well_2	F005	224	Z distribution	-0.58895	-0.6019	0.421813
TOM20	W2	PGE2	well_2	F006	91	Z distribution	-0.55806	-0.49082	0.556049
TOM20	W2	PGE2	well_3	F001	399	Z distribution	-0.62842	-0.62823	0.522994
TOM20	W2	PGE2	well_3	F002	454	Z distribution	-0.63909	-0.58379	0.537498

TOM20	W2	PGE2	well_3	F003	289	Z distribution	-0.60168	-0.5662	0.484675
TOM20	W2	PGE2	well_3	F004	475	Z distribution	-0.51191	-0.49077	0.503677
TOM20	W2	PGE2	well_3	F005	308	Z distribution	-0.41508	-0.41202	0.418379
TOM20	W2	PGE2	well_3	F006	312	Z distribution	-0.69173	-0.64912	0.494857
TOM20	W2	PGE2	well_4	F001	463	Z distribution	-0.60802	-0.63155	0.55128
TOM20	W2	PGE2	well_4	F002	355	Z distribution	-0.74816	-0.75177	0.541328
TOM20	W2	PGE2	well_4	F003	336	Z distribution	-0.66212	-0.65445	0.500904
TOM20	W2	PGE2	well_4	F004	479	Z distribution	-0.64893	-0.64488	0.481326
TOM20	W2	PGE2	well_4	F005	511	Z distribution	-0.67395	-0.62044	0.548914
TOM20	W2	PGE2	well_4	F006	473	Z distribution	-0.66498	-0.70295	0.549939
TOM20	W2	PGE2	well_5	F001	401	Z distribution	-0.55318	-0.54932	0.537844
TOM20	W2	PGE2	well_5	F002	370	Z distribution	-0.64116	-0.64409	0.498413
TOM20	W2	PGE2	well_5	F003	452	Z distribution	-0.52223	-0.51153	0.507625
TOM20	W2	PGE2	well_5	F004	396	Z distribution	-0.3505	-0.33901	0.486181
TOM20	W2	PGE2	well_5	F005	344	Z distribution	-0.81893	-0.83013	0.509296
TOM20	W2	PGE2	well_5	F006	360	Z distribution	-0.67801	-0.63997	0.512328
TOM20	W3	HPI4	well_1	F001	92	Z distribution	-0.43719	-0.38626	0.43264
TOM20	W3	HPI4	well_1	F002	87	Z distribution	-0.70764	-0.63966	0.408846
TOM20	W3	HPI4	well_1	F003	120	Z distribution	-0.93971	-0.95748	0.474446
TOM20	W3	HPI4	well_1	F004	66	Z distribution	-0.98878	-0.96932	0.442338
TOM20	W3	HPI4	well_1	F005	165	Z distribution	-1.00908	-1.09381	0.567719
TOM20	W3	HPI4	well_1	F006	32	Z distribution	-1.0746	-1.08773	0.406215
TOM20	W3	HPI4	well_2	F001	64	Z distribution	-0.71414	-0.58938	0.462434
TOM20	W3	HPI4	well_2	F002	138	Z distribution	-0.96592	-1.00158	0.490068
TOM20	W3	HPI4	well_2	F003	154	Z distribution	-0.89755	-0.87213	0.577743
TOM20	W3	HPI4	well_2	F004	219	Z distribution	-0.95765	-1.0188	0.547575
TOM20	W3	HPI4	well_2	F005	180	Z distribution	-0.80351	-0.83494	0.524702
TOM20	W3	HPI4	well_2	F006	181	Z distribution	-0.81683	-0.90071	0.497392
TOM20	W3	HPI4	well_3	F001	110	Z distribution	-0.91263	-0.87701	0.52865
TOM20	W3	HPI4	well_3	F002	130	Z distribution	-0.95569	-0.96863	0.365323
TOM20	W3	HPI4	well_3	F003	159	Z distribution	-1.12888	-1.24655	0.484313
TOM20	W3	HPI4	well_3	F004	207	Z distribution	-0.89952	-0.93679	0.512146
TOM20	W3	HPI4	well_3	F005	50	Z distribution	-0.67474	-0.59416	0.436461
TOM20	W3	HPI4	well_3	F006	77	Z distribution	-0.95577	-0.90265	0.458926
TOM20	W3	HPI4	well_4	F001	128	Z distribution	-0.7033	-0.68248	0.396038
TOM20	W3	HPI4	well_4	F002	141	Z distribution	-0.82035	-0.91473	0.502318
TOM20	W3	HPI4	well_4	F003	232	Z distribution	-0.9515	-1.02599	0.523705
TOM20	W3	HPI4	well_4	F004	148	Z distribution	-0.82416	-0.74462	0.513999
TOM20	W3	HPI4	well_4	F005	163	Z distribution	-0.93259	-1.01757	0.447691
TOM20	W3	HPI4	well_4	F006	251	Z distribution	-0.59175	-0.56994	0.499233
TOM20	W3	HPI4	well_5	F001	127	Z distribution	-0.64783	-0.57937	0.394169
TOM20	W3	HPI4	well_5	F002	152	Z distribution	-0.85784	-0.94235	0.52446
TOM20	W3	HPI4	well_5	F003	318	Z distribution	-0.68102	-0.5908	0.504995
TOM20	W3	HPI4	well_5	F004	110	Z distribution	-0.99967	-1.01731	0.615982
TOM20	W3	HPI4	well_5	F005	239	Z distribution	-0.64057	-0.51694	0.500268
TOM20	W3	HPI4	well_5	F006	59	Z distribution	-0.86103	-0.90435	0.471238
TOM20	W3	PGE2	well_1	F001	287	Z distribution	-0.68124	-0.65813	0.501883
TOM20	W3	PGE2	well_1	F002	378	Z distribution	-0.63152	-0.59802	0.513558
TOM20	W3	PGE2	well_1	F003	324	Z distribution	-0.54724	-0.54415	0.498722

TOM20	W3	PGE2	well_1	F004	454	Z distribution	-0.60533	-0.59287	0.482429
TOM20	W3	PGE2	well_1	F005	421	Z distribution	-0.51281	-0.52831	0.445466
TOM20	W3	PGE2	well_1	F006	241	Z distribution	-0.6219	-0.55934	0.596674
TOM20	W3	PGE2	well_2	F001	299	Z distribution	-0.71945	-0.70935	0.441601
TOM20	W3	PGE2	well_2	F002	369	Z distribution	-0.50188	-0.49826	0.466266
TOM20	W3	PGE2	well_2	F003	402	Z distribution	-0.45082	-0.43841	0.518387
TOM20	W3	PGE2	well_2	F004	460	Z distribution	-0.59711	-0.5718	0.495843
TOM20	W3	PGE2	well_2	F005	438	Z distribution	-0.56929	-0.58555	0.51107
TOM20	W3	PGE2	well_2	F006	353	Z distribution	-0.62894	-0.6121	0.506374
TOM20	W3	PGE2	well_3	F001	277	Z distribution	-0.60613	-0.6047	0.502119
TOM20	W3	PGE2	well_3	F002	273	Z distribution	-0.32661	-0.28253	0.490752
TOM20	W3	PGE2	well_3	F003	424	Z distribution	-0.59907	-0.57028	0.554139
TOM20	W3	PGE2	well_3	F004	481	Z distribution	-0.5957	-0.59479	0.456399
TOM20	W3	PGE2	well_3	F005	432	Z distribution	-0.61524	-0.61422	0.52679
TOM20	W3	PGE2	well_3	F006	292	Z distribution	-0.71202	-0.69669	0.581012
TOM20	W3	PGE2	well_4	F001	402	Z distribution	-0.73302	-0.74336	0.574452
TOM20	W3	PGE2	well_4	F002	358	Z distribution	-0.67099	-0.68549	0.556915
TOM20	W3	PGE2	well_4	F003	257	Z distribution	-0.6792	-0.66782	0.498256
TOM20	W3	PGE2	well_4	F004	374	Z distribution	-0.70242	-0.63797	0.558307
TOM20	W3	PGE2	well_4	F005	384	Z distribution	-0.79728	-0.79482	0.519828
TOM20	W3	PGE2	well_4	F006	253	Z distribution	-0.71689	-0.74581	0.507321
TOM20	W3	PGE2	well_5	F001	116	Z distribution	-0.73031	-0.75177	0.566858
TOM20	W3	PGE2	well_5	F002	157	Z distribution	-0.80289	-0.80923	0.503991
TOM20	W3	PGE2	well_5	F003	160	Z distribution	-0.91417	-0.88189	0.552026
TOM20	W3	PGE2	well_5	F004	364	Z distribution	-0.54566	-0.46615	0.519713
TOM20	W3	PGE2	well_5	F005	367	Z distribution	-0.68	-0.65151	0.49673
TOM20	W3	PGE2	well_5	F006	253	Z distribution	-0.70101	-0.69047	0.468907
TOM20	W4	HPI4	well_1	F001	60	Z distribution	-0.69545	-0.67903	0.467917
TOM20	W4	HPI4	well_1	F002	159	Z distribution	-0.82569	-0.85522	0.41995
TOM20	W4	HPI4	well_1	F003	130	Z distribution	-1.07627	-1.16033	0.372879
TOM20	W4	HPI4	well_1	F004	179	Z distribution	-0.87493	-0.95889	0.441706
TOM20	W4	HPI4	well_1	F005	141	Z distribution	-0.79958	-0.90683	0.585143
TOM20	W4	HPI4	well_1	F006	130	Z distribution	-0.81217	-0.86606	0.409586
TOM20	W4	HPI4	well_2	F001	158	Z distribution	-0.69204	-0.69248	0.343825
TOM20	W4	HPI4	well_2	F002	191	Z distribution	-0.98519	-1.09951	0.477737
TOM20	W4	HPI4	well_2	F003	211	Z distribution	-0.70341	-0.7439	0.42231
TOM20	W4	HPI4	well_2	F004	192	Z distribution	-0.91858	-0.97367	0.484154
TOM20	W4	HPI4	well_2	F005	152	Z distribution	-0.96239	-1.04631	0.485657
TOM20	W4	HPI4	well_2	F006	115	Z distribution	-0.84152	-0.9168	0.427119
TOM20	W4	HPI4	well_3	F001	109	Z distribution	-1.01089	-1.06141	0.522269
TOM20	W4	HPI4	well_3	F002	140	Z distribution	-0.57548	-0.56019	0.395083
TOM20	W4	HPI4	well_3	F003	180	Z distribution	-0.77807	-0.76868	0.406337
TOM20	W4	HPI4	well_3	F004	241	Z distribution	-0.74059	-0.78102	0.43658
TOM20	W4	HPI4	well_3	F005	208	Z distribution	-0.74235	-0.78283	0.475193
TOM20	W4	HPI4	well_3	F006	159	Z distribution	-1.02198	-1.01114	0.38728
TOM20	W4	HPI4	well_4	F001	151	Z distribution	-0.87383	-0.90962	0.486241
TOM20	W4	HPI4	well_4	F002	185	Z distribution	-0.47732	-0.46555	0.404598
TOM20	W4	HPI4	well_4	F003	207	Z distribution	-0.68407	-0.69641	0.47685
TOM20	W4	HPI4	well_4	F004	170	Z distribution	-0.7412	-0.83269	0.46167

TOM20	W4	HPI4	well_4	F005	172	Z distribution	-0.8345	-0.89111	0.38761
TOM20	W4	HPI4	well_4	F006	168	Z distribution	-1.08166	-1.1063	0.40469
TOM20	W4	HPI4	well_5	F001	177	Z distribution	-0.8407	-0.92509	0.507372
TOM20	W4	HPI4	well_5	F002	101	Z distribution	-0.37663	-0.36162	0.430552
TOM20	W4	HPI4	well_5	F003	244	Z distribution	-0.66752	-0.73851	0.493847
TOM20	W4	HPI4	well_5	F004	197	Z distribution	-0.82092	-0.89989	0.492483
TOM20	W4	HPI4	well_5	F005	220	Z distribution	-0.40376	-0.3317	0.373445
TOM20	W4	HPI4	well_5	F006	177	Z distribution	-1.36492	-1.4308	0.402891
TOM20	W4	PGE2	well_1	F001	136	Z distribution	-0.63482	-0.59029	0.547231
TOM20	W4	PGE2	well_1	F002	197	Z distribution	-0.63544	-0.61408	0.474289
TOM20	W4	PGE2	well_1	F003	180	Z distribution	-0.5323	-0.4415	0.469055
TOM20	W4	PGE2	well_1	F004	228	Z distribution	-0.57381	-0.57483	0.462857
TOM20	W4	PGE2	well_1	F005	276	Z distribution	-0.5076	-0.46551	0.498627
TOM20	W4	PGE2	well_1	F006	121	Z distribution	-0.4453	-0.42175	0.465564
TOM20	W4	PGE2	well_2	F001	298	Z distribution	-0.67989	-0.6566	0.544304
TOM20	W4	PGE2	well_2	F002	357	Z distribution	-0.7122	-0.71786	0.493884
TOM20	W4	PGE2	well_2	F003	63	Z distribution	-0.40263	-0.40281	0.390423
TOM20	W4	PGE2	well_2	F004	244	Z distribution	-0.44892	-0.40814	0.461837
TOM20	W4	PGE2	well_2	F005	173	Z distribution	-0.34491	-0.34372	0.367557
TOM20	W4	PGE2	well_2	F006	77	Z distribution	-0.73093	-0.74308	0.460584
TOM20	W4	PGE2	well_3	F001	137	Z distribution	-0.71442	-0.73518	0.476918
TOM20	W4	PGE2	well_3	F002	335	Z distribution	-0.73634	-0.70859	0.496914
TOM20	W4	PGE2	well_3	F003	248	Z distribution	-0.65081	-0.59608	0.440406
TOM20	W4	PGE2	well_3	F004	327	Z distribution	-0.66062	-0.66651	0.484016
TOM20	W4	PGE2	well_3	F005	77	Z distribution	-0.54503	-0.57313	0.343319
TOM20	W4	PGE2	well_3	F006	90	Z distribution	-0.53671	-0.47257	0.471307
TOM20	W4	PGE2	well_4	F001	179	Z distribution	-0.59067	-0.51887	0.498613
TOM20	W4	PGE2	well_4	F002	409	Z distribution	-0.60798	-0.55043	0.500391
TOM20	W4	PGE2	well_4	F003	296	Z distribution	-0.36175	-0.35205	0.434617
TOM20	W4	PGE2	well_4	F004	386	Z distribution	-0.41786	-0.4042	0.401057
TOM20	W4	PGE2	well_4	F005	417	Z distribution	-0.34694	-0.30242	0.457651
TOM20	W4	PGE2	well_4	F006	192	Z distribution	-0.43257	-0.3676	0.443071
TOM20	W4	PGE2	well_5	F001	220	Z distribution	-0.53956	-0.49455	0.431966
TOM20	W4	PGE2	well_5	F002	228	Z distribution	-0.90732	-0.89333	0.506805
TOM20	W4	PGE2	well_5	F003	341	Z distribution	-0.63323	-0.62173	0.448496
TOM20	W4	PGE2	well_5	F004	398	Z distribution	-0.57085	-0.54638	0.452493
TOM20	W4	PGE2	well_5	F005	277	Z distribution	-0.61638	-0.65929	0.486069
TOM20	W4	PGE2	well_5	F006	268	Z distribution	-0.69195	-0.72519	0.513786
TOM20	W1	HPI4	well_1	F001	210	Total volume	117.2717	111.0119	50.57456
TOM20	W1	HPI4	well_1	F002	255	Total volume	78.32271	74.45389	41.87031
TOM20	W1	HPI4	well_1	F003	300	Total volume	109.2317	103.7824	50.78189
TOM20	W1	HPI4	well_1	F004	232	Total volume	117.755	114.2393	46.57213
TOM20	W1	HPI4	well_1	F005	317	Total volume	113.4328	107.3854	54.28311
TOM20	W1	HPI4	well_1	F006	182	Total volume	127.7257	125.4238	62.96756
TOM20	W1	HPI4	well_2	F001	267	Total volume	118.9529	115.9763	48.74505
TOM20	W1	HPI4	well_2	F002	241	Total volume	138.3367	127.9001	68.7697
TOM20	W1	HPI4	well_2	F003	231	Total volume	110.5429	104.9443	47.6931
TOM20	W1	HPI4	well_2	F004	185	Total volume	103.0068	96.44736	56.18286
TOM20	W1	HPI4	well_2	F005	466	Total volume	91.51228	87.71569	39.81354

TOM20	W1	HPI4	well_2	F006	206	Total volume	145.1638	144.8119	56.4832
TOM20	W1	HPI4	well_3	F001	312	Total volume	136.4434	123.546	67.75355
TOM20	W1	HPI4	well_3	F002	278	Total volume	124.1958	117.0912	56.6049
TOM20	W1	HPI4	well_3	F003	234	Total volume	138.8724	136.7961	63.54564
TOM20	W1	HPI4	well_3	F004	217	Total volume	113.9859	110.2725	49.256
TOM20	W1	HPI4	well_3	F005	341	Total volume	82.23963	81.63639	34.61596
TOM20	W1	HPI4	well_3	F006	293	Total volume	101.9732	100.7897	43.63876
TOM20	W1	HPI4	well_4	F001	244	Total volume	100.4194	101.5174	46.36137
TOM20	W1	HPI4	well_4	F002	313	Total volume	108.6799	107.8079	48.86485
TOM20	W1	HPI4	well_4	F003	176	Total volume	93.136	89.62868	46.54981
TOM20	W1	HPI4	well_4	F004	175	Total volume	77.56892	74.66514	42.08693
TOM20	W1	HPI4	well_4	F005	351	Total volume	94.27999	89.93382	43.90235
TOM20	W1	HPI4	well_4	F006	306	Total volume	91.24509	84.55868	42.8953
TOM20	W1	HPI4	well_5	F001	243	Total volume	97.89734	94.19403	52.52947
TOM20	W1	HPI4	well_5	F002	220	Total volume	97.09624	88.60764	48.85716
TOM20	W1	HPI4	well_5	F003	315	Total volume	103.4347	102.0807	43.40807
TOM20	W1	HPI4	well_5	F004	124	Total volume	63.18744	61.21556	30.07571
TOM20	W1	HPI4	well_5	F005	165	Total volume	106.2664	103.9819	51.96875
TOM20	W1	HPI4	well_5	F006	187	Total volume	83.78557	73.35069	43.63042
TOM20	W1	PGE2	well_1	F001	286	Total volume	55.67206	47.06181	33.67057
TOM20	W1	PGE2	well_1	F002	265	Total volume	83.09216	72.29444	56.91341
TOM20	W1	PGE2	well_1	F003	184	Total volume	96.71353	89.24139	53.89646
TOM20	W1	PGE2	well_1	F004	295	Total volume	68.72948	60.04194	42.37639
TOM20	W1	PGE2	well_1	F005	435	Total volume	85.29451	80.15764	45.23795
TOM20	W1	PGE2	well_1	F006	244	Total volume	102.5336	91.35389	53.14283
TOM20	W1	PGE2	well_2	F001	326	Total volume	57.13981	53.86875	32.27424
TOM20	W1	PGE2	well_2	F002	246	Total volume	59.58519	51.26333	39.07862
TOM20	W1	PGE2	well_2	F003	350	Total volume	68.1383	59.58424	44.88431
TOM20	W1	PGE2	well_2	F004	269	Total volume	74.82898	67.76431	43.78547
TOM20	W1	PGE2	well_2	F005	334	Total volume	82.73853	75.97958	46.1927
TOM20	W1	PGE2	well_2	F006	290	Total volume	79.79496	74.72382	41.48514
TOM20	W1	PGE2	well_3	F001	312	Total volume	79.90282	75.34583	39.17997
TOM20	W1	PGE2	well_3	F002	328	Total volume	69.93096	58.83313	45.66679
TOM20	W1	PGE2	well_3	F003	249	Total volume	110.1137	101.8694	63.79015
TOM20	W1	PGE2	well_3	F004	293	Total volume	98.49321	94.26444	44.71087
TOM20	W1	PGE2	well_3	F005	316	Total volume	73.16224	67.74083	40.36297
TOM20	W1	PGE2	well_3	F006	251	Total volume	72.57183	63.04639	44.15205
TOM20	W1	PGE2	well_4	F001	265	Total volume	42.80577	32.20389	34.8444
TOM20	W1	PGE2	well_4	F002	268	Total volume	67.1641	64.19653	34.31748
TOM20	W1	PGE2	well_4	F003	284	Total volume	40.86584	35.77167	26.61136
TOM20	W1	PGE2	well_4	F004	289	Total volume	60.34594	54.47903	34.65923
TOM20	W1	PGE2	well_4	F005	201	Total volume	56.48796	45.51264	40.30117
TOM20	W1	PGE2	well_4	F006	289	Total volume	62.20552	49.17431	42.39295
TOM20	W1	PGE2	well_5	F001	130	Total volume	41.58356	36.85139	27.41594
TOM20	W1	PGE2	well_5	F002	191	Total volume	83.29945	74.53604	46.33885
TOM20	W1	PGE2	well_5	F003	277	Total volume	76.61041	68.21028	45.41577
TOM20	W1	PGE2	well_5	F004	384	Total volume	70.15124	62.78819	41.86302
TOM20	W1	PGE2	well_5	F005	253	Total volume	62.11641	56.35681	33.86711
TOM20	W1	PGE2	well_5	F006	146	Total volume	46.50626	39.57417	29.27841

TOM20	W2	HPI4	well_1	F001	253	Total volume	95.86972	88.1734	55.58388
TOM20	W2	HPI4	well_1	F002	159	Total volume	46.28052	38.40056	33.19041
TOM20	W2	HPI4	well_1	F003	177	Total volume	96.35804	95.41458	47.27859
TOM20	W2	HPI4	well_1	F004	419	Total volume	45.94485	42.93069	25.59319
TOM20	W2	HPI4	well_1	F005	229	Total volume	85.3054	80.67403	41.98406
TOM20	W2	HPI4	well_1	F006	159	Total volume	42.77645	47.94201	26.00932
TOM20	W2	HPI4	well_2	F001	220	Total volume	53.03716	49.78458	31.69402
TOM20	W2	HPI4	well_2	F002	234	Total volume	74.51389	63.33979	47.70527
TOM20	W2	HPI4	well_2	F003	234	Total volume	62.6909	53.81007	36.36713
TOM20	W2	HPI4	well_2	F004	283	Total volume	59.45657	49.855	35.01366
TOM20	W2	HPI4	well_2	F005	274	Total volume	74.09481	68.84403	43.56304
TOM20	W2	HPI4	well_2	F006	252	Total volume	67.58998	62.37743	39.94783
TOM20	W2	HPI4	well_3	F001	294	Total volume	102.4592	85.25111	64.72287
TOM20	W2	HPI4	well_3	F002	273	Total volume	99.70879	91.49472	36.51602
TOM20	W2	HPI4	well_3	F003	219	Total volume	100.0782	91.23653	54.17362
TOM20	W2	HPI4	well_3	F004	265	Total volume	61.68386	52.83597	38.20563
TOM20	W2	HPI4	well_3	F005	194	Total volume	53.98504	45.94688	27.38058
TOM20	W2	HPI4	well_3	F006	281	Total volume	48.69942	44.31556	28.33433
TOM20	W2	HPI4	well_4	F001	200	Total volume	40.4873	30.63125	28.62587
TOM20	W2	HPI4	well_4	F002	369	Total volume	63.07992	56.60326	41.65849
TOM20	W2	HPI4	well_4	F003	410	Total volume	69.43975	60.80479	41.56238
TOM20	W2	HPI4	well_4	F004	455	Total volume	76.99725	66.10951	48.20733
TOM20	W2	HPI4	well_4	F005	292	Total volume	89.801	84.5	44.24314
TOM20	W2	HPI4	well_4	F006	259	Total volume	48.14823	41.28764	33.21532
TOM20	W2	HPI4	well_5	F001	250	Total volume	57.65775	52.13181	37.29487
TOM20	W2	HPI4	well_5	F002	196	Total volume	72.45098	58.56319	44.78602
TOM20	W2	HPI4	well_5	F003	321	Total volume	59.94395	54.34993	36.20597
TOM20	W2	HPI4	well_5	F004	448	Total volume	51.68679	44.29208	29.50239
TOM20	W2	HPI4	well_5	F005	319	Total volume	55.72349	48.14153	32.09452
TOM20	W2	HPI4	well_5	F006	269	Total volume	78.05001	71.00347	47.72196
TOM20	W2	PGE2	well_1	F001	186	Total volume	95.8862	90.52063	63.14286
TOM20	W2	PGE2	well_1	F002	233	Total volume	117.1138	115.2017	54.36387
TOM20	W2	PGE2	well_1	F003	443	Total volume	65.02372	59.84243	36.30397
TOM20	W2	PGE2	well_1	F004	70	Total volume	125.4118	124.0976	54.16497
TOM20	W2	PGE2	well_1	F005	190	Total volume	106.2427	86.16653	74.52474
TOM20	W2	PGE2	well_1	F006	211	Total volume	81.28835	77.1884	37.32755
TOM20	W2	PGE2	well_2	F001	424	Total volume	95.84105	91.04875	45.10875
TOM20	W2	PGE2	well_2	F002	268	Total volume	97.19175	95.55542	47.02246
TOM20	W2	PGE2	well_2	F003	595	Total volume	92.77613	86.68292	51.01168
TOM20	W2	PGE2	well_2	F004	512	Total volume	116.3091	105.3199	65.67325
TOM20	W2	PGE2	well_2	F005	224	Total volume	99.39606	96.43563	46.81514
TOM20	W2	PGE2	well_2	F006	91	Total volume	112.807	103.935	58.4121
TOM20	W2	PGE2	well_3	F001	399	Total volume	105.1954	105.6015	51.38792
TOM20	W2	PGE2	well_3	F002	454	Total volume	119.819	124.7314	50.46085
TOM20	W2	PGE2	well_3	F003	289	Total volume	81.43724	68.63278	51.59497
TOM20	W2	PGE2	well_3	F004	475	Total volume	127.9036	128.7451	63.61862
TOM20	W2	PGE2	well_3	F005	308	Total volume	110.7366	105.8949	52.69307
TOM20	W2	PGE2	well_3	F006	312	Total volume	138.8622	126.8674	55.53656
TOM20	W2	PGE2	well_4	F001	463	Total volume	93.64716	90.26243	47.95235

TOM20	W2	PGE2	well_4	F002	355	Total volume	103.697	98.65375	49.64143
TOM20	W2	PGE2	well_4	F003	336	Total volume	112.349	97.12806	76.63713
TOM20	W2	PGE2	well_4	F004	479	Total volume	158.0244	151.9357	70.14828
TOM20	W2	PGE2	well_4	F005	511	Total volume	154.5115	149.1073	58.3796
TOM20	W2	PGE2	well_4	F006	473	Total volume	122.9811	122.7128	46.97053
TOM20	W2	PGE2	well_5	F001	401	Total volume	110.4341	107.7844	57.6806
TOM20	W2	PGE2	well_5	F002	370	Total volume	78.83133	70.08806	48.99328
TOM20	W2	PGE2	well_5	F003	452	Total volume	68.64344	60.91042	39.20237
TOM20	W2	PGE2	well_5	F004	396	Total volume	146.2561	124.1681	83.50072
TOM20	W2	PGE2	well_5	F005	344	Total volume	87.39135	77.11799	50.55836
TOM20	W2	PGE2	well_5	F006	360	Total volume	135.7281	138.5096	51.38482
TOM20	W3	HPI4	well_1	F001	92	Total volume	106.2403	96.55299	58.15096
TOM20	W3	HPI4	well_1	F002	87	Total volume	119.2775	109.85	47.65948
TOM20	W3	HPI4	well_1	F003	120	Total volume	114.9068	110.3547	42.06438
TOM20	W3	HPI4	well_1	F004	66	Total volume	136.8538	130.1769	62.28014
TOM20	W3	HPI4	well_1	F005	165	Total volume	84.79469	78.93708	42.48161
TOM20	W3	HPI4	well_1	F006	32	Total volume	130.674	124.3793	56.25882
TOM20	W3	HPI4	well_2	F001	64	Total volume	83.87459	75.81528	43.67632
TOM20	W3	HPI4	well_2	F002	138	Total volume	142.947	140.7042	52.63999
TOM20	W3	HPI4	well_2	F003	154	Total volume	99.08283	93.48986	44.39598
TOM20	W3	HPI4	well_2	F004	219	Total volume	132.3308	129.6371	57.87065
TOM20	W3	HPI4	well_2	F005	180	Total volume	102.3871	99.48701	48.4551
TOM20	W3	HPI4	well_2	F006	181	Total volume	119.8127	117.0794	58.75143
TOM20	W3	HPI4	well_3	F001	110	Total volume	116.8452	112.5493	53.51429
TOM20	W3	HPI4	well_3	F002	130	Total volume	116.9804	115.1313	50.47873
TOM20	W3	HPI4	well_3	F003	159	Total volume	121.1162	116.5631	51.89009
TOM20	W3	HPI4	well_3	F004	207	Total volume	116.8806	115.6711	53.47975
TOM20	W3	HPI4	well_3	F005	50	Total volume	101.3974	99.20535	48.78871
TOM20	W3	HPI4	well_3	F006	77	Total volume	144.784	140.1878	67.32918
TOM20	W3	HPI4	well_4	F001	128	Total volume	130.5269	124.3558	60.27783
TOM20	W3	HPI4	well_4	F002	141	Total volume	118.6716	109.0167	58.82573
TOM20	W3	HPI4	well_4	F003	232	Total volume	102.8263	97.98479	39.48062
TOM20	W3	HPI4	well_4	F004	148	Total volume	94.77811	88.74847	48.04087
TOM20	W3	HPI4	well_4	F005	163	Total volume	146.2131	144.6828	66.84802
TOM20	W3	HPI4	well_4	F006	251	Total volume	117.7492	109.8265	56.6236
TOM20	W3	HPI4	well_5	F001	127	Total volume	122.6685	111.9625	63.51694
TOM20	W3	HPI4	well_5	F002	152	Total volume	113.7314	109.5918	46.91176
TOM20	W3	HPI4	well_5	F003	318	Total volume	115.2329	110.5307	52.63615
TOM20	W3	HPI4	well_5	F004	110	Total volume	91.43939	91.15438	43.16264
TOM20	W3	HPI4	well_5	F005	239	Total volume	104.4761	94.56958	52.01592
TOM20	W3	HPI4	well_5	F006	59	Total volume	115.2761	104.2988	51.48288
TOM20	W3	PGE2	well_1	F001	287	Total volume	105.733	104.5805	33.27558
TOM20	W3	PGE2	well_1	F002	378	Total volume	72.70645	70.28757	33.94221
TOM20	W3	PGE2	well_1	F003	324	Total volume	81.5749	78.84319	30.15623
TOM20	W3	PGE2	well_1	F004	454	Total volume	76.56222	65.31146	39.3848
TOM20	W3	PGE2	well_1	F005	421	Total volume	75.02542	72.92819	38.04269
TOM20	W3	PGE2	well_1	F006	241	Total volume	87.01699	80.29847	34.08361
TOM20	W3	PGE2	well_2	F001	299	Total volume	63.33287	60.01847	26.36849
TOM20	W3	PGE2	well_2	F002	369	Total volume	61.24128	58.26979	22.83423

TOM20	W3	PGE2	well_2	F003	402	Total volume	65.63752	63.10507	31.30965
TOM20	W3	PGE2	well_2	F004	460	Total volume	63.47442	61.69674	31.42529
TOM20	W3	PGE2	well_2	F005	438	Total volume	59.68716	58.76271	30.35079
TOM20	W3	PGE2	well_2	F006	353	Total volume	70.14804	59.71333	38.7905
TOM20	W3	PGE2	well_3	F001	277	Total volume	73.62452	72.29444	30.57629
TOM20	W3	PGE2	well_3	F002	273	Total volume	76.69359	74.50083	29.16234
TOM20	W3	PGE2	well_3	F003	424	Total volume	84.3124	81.40167	33.72987
TOM20	W3	PGE2	well_3	F004	481	Total volume	79.6125	79.24222	30.79642
TOM20	W3	PGE2	well_3	F005	432	Total volume	74.06963	72.81083	31.4605
TOM20	W3	PGE2	well_3	F006	292	Total volume	70.83514	68.85576	27.00681
TOM20	W3	PGE2	well_4	F001	402	Total volume	83.5205	82.50486	32.98981
TOM20	W3	PGE2	well_4	F002	358	Total volume	100.6074	97.26889	28.62656
TOM20	W3	PGE2	well_4	F003	257	Total volume	106.8054	99.19361	37.34219
TOM20	W3	PGE2	well_4	F004	374	Total volume	80.68656	80.81486	31.66834
TOM20	W3	PGE2	well_4	F005	384	Total volume	102.3268	101.5643	31.18108
TOM20	W3	PGE2	well_4	F006	253	Total volume	79.2998	77.54049	30.39936
TOM20	W3	PGE2	well_5	F001	116	Total volume	76.91984	76.63681	26.67714
TOM20	W3	PGE2	well_5	F002	157	Total volume	71.74139	69.66556	30.16928
TOM20	W3	PGE2	well_5	F003	160	Total volume	82.6587	79.86424	35.12289
TOM20	W3	PGE2	well_5	F004	364	Total volume	97.51796	91.96417	34.15345
TOM20	W3	PGE2	well_5	F005	367	Total volume	114.6072	115.7767	35.96094
TOM20	W3	PGE2	well_5	F006	253	Total volume	89.17496	89.39396	32.12767
TOM20	W4	HPI4	well_1	F001	60	Total volume	80.608	80.01681	28.39526
TOM20	W4	HPI4	well_1	F002	159	Total volume	94.65643	92.04632	32.87295
TOM20	W4	HPI4	well_1	F003	130	Total volume	78.66553	72.14188	35.60118
TOM20	W4	HPI4	well_1	F004	179	Total volume	69.34845	69.82986	26.1214
TOM20	W4	HPI4	well_1	F005	141	Total volume	71.86978	68.89097	25.19426
TOM20	W4	HPI4	well_1	F006	130	Total volume	73.44588	67.38875	37.38704
TOM20	W4	HPI4	well_2	F001	158	Total volume	74.91208	72.50569	27.13985
TOM20	W4	HPI4	well_2	F002	191	Total volume	90.13547	89.83993	30.98524
TOM20	W4	HPI4	well_2	F003	211	Total volume	82.16843	79.7234	29.28894
TOM20	W4	HPI4	well_2	F004	192	Total volume	74.27592	74.03139	23.2516
TOM20	W4	HPI4	well_2	F005	152	Total volume	72.796	70.22889	26.17519
TOM20	W4	HPI4	well_2	F006	115	Total volume	83.24598	80.6975	28.4872
TOM20	W4	HPI4	well_3	F001	109	Total volume	57.21321	56.30986	23.66198
TOM20	W4	HPI4	well_3	F002	140	Total volume	78.11421	66.50854	41.83092
TOM20	W4	HPI4	well_3	F003	180	Total volume	83.13608	85.03986	32.24829
TOM20	W4	HPI4	well_3	F004	241	Total volume	76.90134	74.92333	30.12584
TOM20	W4	HPI4	well_3	F005	208	Total volume	85.22818	82.78653	27.14816
TOM20	W4	HPI4	well_3	F006	159	Total volume	95.24218	92.73875	34.89362
TOM20	W4	HPI4	well_4	F001	151	Total volume	83.15126	81.44861	26.6029
TOM20	W4	HPI4	well_4	F002	185	Total volume	91.84659	89.57	29.62613
TOM20	W4	HPI4	well_4	F003	207	Total volume	93.42209	92.50403	30.85687
TOM20	W4	HPI4	well_4	F004	170	Total volume	69.4667	65.88653	26.30767
TOM20	W4	HPI4	well_4	F005	172	Total volume	88.13553	82.85694	29.80724
TOM20	W4	HPI4	well_4	F006	168	Total volume	85.85032	82.41097	33.98508
TOM20	W4	HPI4	well_5	F001	177	Total volume	71.78505	71.89542	26.5916
TOM20	W4	HPI4	well_5	F002	101	Total volume	86.47217	80.95569	35.43346
TOM20	W4	HPI4	well_5	F003	244	Total volume	76.6637	70.69833	28.88847

TOM20	W4	HPI4	well_5	F004	197	Total volume	63.36694	62.31875	26.431
TOM20	W4	HPI4	well_5	F005	220	Total volume	75.51335	72.38833	28.08447
TOM20	W4	HPI4	well_5	F006	177	Total volume	99.98722	100.8132	32.13014
TOM20	W4	PGE2	well_1	F001	136	Total volume	70.78453	62.9525	32.80209
TOM20	W4	PGE2	well_1	F002	197	Total volume	61.60994	62.99944	26.55208
TOM20	W4	PGE2	well_1	F003	180	Total volume	68.62394	64.39604	29.76861
TOM20	W4	PGE2	well_1	F004	228	Total volume	47.89956	38.54139	32.03039
TOM20	W4	PGE2	well_1	F005	276	Total volume	46.34619	38.71743	33.34946
TOM20	W4	PGE2	well_1	F006	121	Total volume	54.13061	56.38028	18.24181
TOM20	W4	PGE2	well_2	F001	298	Total volume	49.7285	48.68139	24.27465
TOM20	W4	PGE2	well_2	F002	357	Total volume	46.63155	42.53167	25.25713
TOM20	W4	PGE2	well_2	F003	63	Total volume	51.17158	51.87361	23.20293
TOM20	W4	PGE2	well_2	F004	244	Total volume	69.25027	64.60729	34.70218
TOM20	W4	PGE2	well_2	F005	173	Total volume	64.44321	62.81167	21.84729
TOM20	W4	PGE2	well_2	F006	77	Total volume	56.10575	59.64292	19.92938
TOM20	W4	PGE2	well_3	F001	137	Total volume	57.99146	56.66194	24.98809
TOM20	W4	PGE2	well_3	F002	335	Total volume	47.75153	45.67694	27.32446
TOM20	W4	PGE2	well_3	F003	248	Total volume	51.49552	47.81292	22.86728
TOM20	W4	PGE2	well_3	F004	327	Total volume	56.56157	54.73722	25.84099
TOM20	W4	PGE2	well_3	F005	77	Total volume	56.27108	54.925	22.04388
TOM20	W4	PGE2	well_3	F006	90	Total volume	70.8553	63.76229	41.78752
TOM20	W4	PGE2	well_4	F001	179	Total volume	90.91083	85.86139	43.31417
TOM20	W4	PGE2	well_4	F002	409	Total volume	58.28754	57.46	26.82833
TOM20	W4	PGE2	well_4	F003	296	Total volume	73.5525	74.0666	24.92807
TOM20	W4	PGE2	well_4	F004	386	Total volume	67.26128	65.12368	29.93783
TOM20	W4	PGE2	well_4	F005	417	Total volume	74.65919	70.34625	28.9128
TOM20	W4	PGE2	well_4	F006	192	Total volume	71.21349	67.44743	29.85515
TOM20	W4	PGE2	well_5	F001	220	Total volume	45.01842	41.83924	21.93716
TOM20	W4	PGE2	well_5	F002	228	Total volume	54.06286	53.28194	21.3672
TOM20	W4	PGE2	well_5	F003	341	Total volume	70.58388	68.28069	29.79809
TOM20	W4	PGE2	well_5	F004	398	Total volume	76.83875	74.62993	31.39243
TOM20	W4	PGE2	well_5	F005	277	Total volume	79.93753	78.27986	33.30363
TOM20	W4	PGE2	well_5	F006	268	Total volume	69.64492	64.22	30.80111
LAMP1	W1	HPI4	well_1	F001	272	Avg. Volume	0.445673	0.436715	0.136151
LAMP1	W1	HPI4	well_1	F002	347	Avg. Volume	0.441485	0.435019	0.138954
LAMP1	W1	HPI4	well_1	F003	207	Avg. Volume	0.444601	0.435214	0.160635
LAMP1	W1	HPI4	well_1	F004	346	Avg. Volume	0.432757	0.425956	0.151114
LAMP1	W1	HPI4	well_1	F005	399	Avg. Volume	0.440205	0.445355	0.153977
LAMP1	W1	HPI4	well_1	F006	250	Avg. Volume	0.456304	0.450844	0.150098
LAMP1	W1	HPI4	well_2	F001	103	Avg. Volume	0.43196	0.414216	0.131548
LAMP1	W1	HPI4	well_2	F002	241	Avg. Volume	0.433069	0.439745	0.147282
LAMP1	W1	HPI4	well_2	F003	207	Avg. Volume	0.432927	0.426768	0.148163
LAMP1	W1	HPI4	well_2	F004	323	Avg. Volume	0.513467	0.512281	0.149793
LAMP1	W1	HPI4	well_2	F005	297	Avg. Volume	0.433387	0.431889	0.143809
LAMP1	W1	HPI4	well_2	F006	185	Avg. Volume	0.496154	0.481181	0.132283
LAMP1	W1	HPI4	well_3	F001	68	Avg. Volume	0.460156	0.443553	0.138253
LAMP1	W1	HPI4	well_3	F002	257	Avg. Volume	0.439781	0.443527	0.139987
LAMP1	W1	HPI4	well_3	F003	70	Avg. Volume	0.415049	0.411032	0.137273
LAMP1	W1	HPI4	well_3	F004	279	Avg. Volume	0.426059	0.405734	0.147733

LAMP1	W1	HPI4	well_3	F005	329	Avg. Volume	0.433971	0.414989	0.144272
LAMP1	W1	HPI4	well_3	F006	214	Avg. Volume	0.444928	0.440446	0.133357
LAMP1	W1	HPI4	well_4	F001	282	Avg. Volume	0.442678	0.415239	0.146184
LAMP1	W1	HPI4	well_4	F002	295	Avg. Volume	0.416983	0.40311	0.147842
LAMP1	W1	HPI4	well_4	F003	265	Avg. Volume	0.447401	0.437437	0.13931
LAMP1	W1	HPI4	well_4	F004	81	Avg. Volume	0.466637	0.458547	0.160225
LAMP1	W1	HPI4	well_4	F005	205	Avg. Volume	0.413151	0.398518	0.168535
LAMP1	W1	HPI4	well_4	F006	167	Avg. Volume	0.437742	0.434854	0.146445
LAMP1	W1	HPI4	well_5	F001	329	Avg. Volume	0.46609	0.465949	0.143327
LAMP1	W1	HPI4	well_5	F002	209	Avg. Volume	0.388359	0.378617	0.132949
LAMP1	W1	HPI4	well_5	F003	84	Avg. Volume	0.450141	0.447182	0.147397
LAMP1	W1	HPI4	well_5	F004	161	Avg. Volume	0.423816	0.399028	0.155873
LAMP1	W1	HPI4	well_5	F005	302	Avg. Volume	0.445042	0.434258	0.137702
LAMP1	W1	HPI4	well_5	F006	233	Avg. Volume	0.444949	0.435214	0.150789
LAMP1	W1	PGE2	well_1	F001	314	Avg. Volume	0.491587	0.473356	0.164516
LAMP1	W1	PGE2	well_1	F002	389	Avg. Volume	0.415506	0.417558	0.129025
LAMP1	W1	PGE2	well_1	F003	304	Avg. Volume	0.442848	0.43142	0.143506
LAMP1	W1	PGE2	well_1	F004	312	Avg. Volume	0.422726	0.415442	0.158053
LAMP1	W1	PGE2	well_1	F005	438	Avg. Volume	0.448695	0.442217	0.158726
LAMP1	W1	PGE2	well_1	F006	411	Avg. Volume	0.488945	0.477921	0.131985
LAMP1	W1	PGE2	well_2	F001	334	Avg. Volume	0.453857	0.441571	0.127484
LAMP1	W1	PGE2	well_2	F002	387	Avg. Volume	0.429768	0.408911	0.137273
LAMP1	W1	PGE2	well_2	F003	391	Avg. Volume	0.468825	0.469444	0.140891
LAMP1	W1	PGE2	well_2	F004	290	Avg. Volume	0.472838	0.467077	0.151167
LAMP1	W1	PGE2	well_2	F005	315	Avg. Volume	0.480225	0.481691	0.15323
LAMP1	W1	PGE2	well_2	F006	370	Avg. Volume	0.451663	0.45342	0.128485
LAMP1	W1	PGE2	well_3	F001	336	Avg. Volume	0.460324	0.454306	0.132456
LAMP1	W1	PGE2	well_3	F002	290	Avg. Volume	0.435877	0.445972	0.129651
LAMP1	W1	PGE2	well_3	F003	289	Avg. Volume	0.443583	0.43875	0.139125
LAMP1	W1	PGE2	well_3	F004	236	Avg. Volume	0.484358	0.4703	0.13397
LAMP1	W1	PGE2	well_3	F005	444	Avg. Volume	0.452272	0.438393	0.143326
LAMP1	W1	PGE2	well_3	F006	420	Avg. Volume	0.413	0.397256	0.136931
LAMP1	W1	PGE2	well_4	F001	320	Avg. Volume	0.464628	0.45692	0.15038
LAMP1	W1	PGE2	well_4	F002	371	Avg. Volume	0.465054	0.460797	0.151512
LAMP1	W1	PGE2	well_4	F003	179	Avg. Volume	0.500704	0.503186	0.158081
LAMP1	W1	PGE2	well_4	F004	328	Avg. Volume	0.434998	0.4225	0.165656
LAMP1	W1	PGE2	well_4	F005	375	Avg. Volume	0.455621	0.45914	0.158376
LAMP1	W1	PGE2	well_4	F006	348	Avg. Volume	0.44897	0.430554	0.150119
LAMP1	W1	PGE2	well_5	F001	299	Avg. Volume	0.44967	0.447901	0.14431
LAMP1	W1	PGE2	well_5	F002	351	Avg. Volume	0.413616	0.410087	0.130685
LAMP1	W1	PGE2	well_5	F003	131	Avg. Volume	0.466376	0.453796	0.123297
LAMP1	W1	PGE2	well_5	F004	302	Avg. Volume	0.425231	0.41775	0.135889
LAMP1	W1	PGE2	well_5	F005	339	Avg. Volume	0.459225	0.460417	0.156468
LAMP1	W1	PGE2	well_5	F006	256	Avg. Volume	0.478615	0.47253	0.147591
LAMP1	W2	HPI4	well_1	F001	299	Avg. Volume	0.263967	0.26385	0.088149
LAMP1	W2	HPI4	well_1	F002	376	Avg. Volume	0.286804	0.28374	0.086684
LAMP1	W2	HPI4	well_1	F003	372	Avg. Volume	0.193926	0.18517	0.078728
LAMP1	W2	HPI4	well_1	F004	229	Avg. Volume	0.285707	0.283285	0.082353
LAMP1	W2	HPI4	well_1	F005	216	Avg. Volume	0.305165	0.29275	0.096138

LAMP1	W2	HPI4	well_1	F006	415	Avg. Volume	0.182833	0.164306	0.094616
LAMP1	W2	HPI4	well_2	F001	357	Avg. Volume	0.228162	0.216403	0.080928
LAMP1	W2	HPI4	well_2	F002	291	Avg. Volume	0.242992	0.23733	0.079214
LAMP1	W2	HPI4	well_2	F003	344	Avg. Volume	0.24755	0.243062	0.083996
LAMP1	W2	HPI4	well_2	F004	332	Avg. Volume	0.198887	0.190255	0.073515
LAMP1	W2	HPI4	well_2	F005	325	Avg. Volume	0.194724	0.178976	0.07112
LAMP1	W2	HPI4	well_2	F006	409	Avg. Volume	0.221499	0.205727	0.08892
LAMP1	W2	HPI4	well_3	F001	346	Avg. Volume	0.263171	0.249939	0.099206
LAMP1	W2	HPI4	well_3	F002	406	Avg. Volume	0.238539	0.232446	0.083429
LAMP1	W2	HPI4	well_3	F003	153	Avg. Volume	0.30626	0.298171	0.099974
LAMP1	W2	HPI4	well_3	F004	273	Avg. Volume	0.284718	0.285682	0.092293
LAMP1	W2	HPI4	well_3	F005	210	Avg. Volume	0.234397	0.224895	0.08646
LAMP1	W2	HPI4	well_3	F006	400	Avg. Volume	0.205004	0.190148	0.079238
LAMP1	W2	HPI4	well_4	F001	475	Avg. Volume	0.217563	0.203426	0.076023
LAMP1	W2	HPI4	well_4	F002	445	Avg. Volume	0.182615	0.172688	0.07184
LAMP1	W2	HPI4	well_4	F003	274	Avg. Volume	0.265186	0.256861	0.093051
LAMP1	W2	HPI4	well_4	F004	388	Avg. Volume	0.190029	0.176042	0.070884
LAMP1	W2	HPI4	well_4	F005	275	Avg. Volume	0.181117	0.172912	0.061359
LAMP1	W2	HPI4	well_4	F006	414	Avg. Volume	0.180101	0.171063	0.066869
LAMP1	W2	HPI4	well_5	F001	352	Avg. Volume	0.192145	0.177427	0.076871
LAMP1	W2	HPI4	well_5	F002	361	Avg. Volume	0.198128	0.183264	0.070441
LAMP1	W2	HPI4	well_5	F003	268	Avg. Volume	0.241751	0.236764	0.074814
LAMP1	W2	HPI4	well_5	F004	267	Avg. Volume	0.214179	0.209869	0.061714
LAMP1	W2	HPI4	well_5	F005	229	Avg. Volume	0.201487	0.184844	0.09982
LAMP1	W2	HPI4	well_5	F006	293	Avg. Volume	0.164344	0.143181	0.08938
LAMP1	W2	PGE2	well_1	F001	249	Avg. Volume	0.387009	0.381815	0.124941
LAMP1	W2	PGE2	well_1	F002	283	Avg. Volume	0.395916	0.382699	0.112953
LAMP1	W2	PGE2	well_1	F003	198	Avg. Volume	0.416773	0.401713	0.129099
LAMP1	W2	PGE2	well_1	F004	174	Avg. Volume	0.433934	0.440958	0.127098
LAMP1	W2	PGE2	well_1	F005	235	Avg. Volume	0.415616	0.410764	0.105659
LAMP1	W2	PGE2	well_1	F006	381	Avg. Volume	0.400497	0.388822	0.116845
LAMP1	W2	PGE2	well_2	F001	162	Avg. Volume	0.421906	0.4225	0.108917
LAMP1	W2	PGE2	well_2	F002	82	Avg. Volume	0.459585	0.443475	0.118399
LAMP1	W2	PGE2	well_2	F003	370	Avg. Volume	0.467274	0.46143	0.140547
LAMP1	W2	PGE2	well_2	F004	242	Avg. Volume	0.436392	0.445972	0.125838
LAMP1	W2	PGE2	well_2	F005	233	Avg. Volume	0.409965	0.399028	0.117166
LAMP1	W2	PGE2	well_2	F006	128	Avg. Volume	0.387563	0.389033	0.111067
LAMP1	W2	PGE2	well_3	F001	300	Avg. Volume	0.440534	0.43802	0.123273
LAMP1	W2	PGE2	well_3	F002	274	Avg. Volume	0.437113	0.44911	0.117126
LAMP1	W2	PGE2	well_3	F003	179	Avg. Volume	0.453111	0.450373	0.136995
LAMP1	W2	PGE2	well_3	F004	280	Avg. Volume	0.447222	0.446461	0.144341
LAMP1	W2	PGE2	well_3	F005	261	Avg. Volume	0.447827	0.440851	0.13317
LAMP1	W2	PGE2	well_3	F006	213	Avg. Volume	0.468807	0.464028	0.129564
LAMP1	W2	PGE2	well_4	F001	202	Avg. Volume	0.446083	0.444194	0.106734
LAMP1	W2	PGE2	well_4	F002	226	Avg. Volume	0.448438	0.452141	0.108793
LAMP1	W2	PGE2	well_4	F003	300	Avg. Volume	0.449331	0.453456	0.130351
LAMP1	W2	PGE2	well_4	F004	291	Avg. Volume	0.445956	0.441031	0.142278
LAMP1	W2	PGE2	well_4	F005	202	Avg. Volume	0.480716	0.472018	0.128078
LAMP1	W2	PGE2	well_4	F006	252	Avg. Volume	0.433526	0.435545	0.11182

LAMP1	W2	PGE2	well_5	F001	319	Avg. Volume	0.414234	0.404635	0.11173
LAMP1	W2	PGE2	well_5	F002	218	Avg. Volume	0.443753	0.434003	0.120681
LAMP1	W2	PGE2	well_5	F003	381	Avg. Volume	0.418365	0.406852	0.151367
LAMP1	W2	PGE2	well_5	F004	272	Avg. Volume	0.475323	0.456588	0.144564
LAMP1	W2	PGE2	well_5	F005	376	Avg. Volume	0.444231	0.43856	0.137466
LAMP1	W2	PGE2	well_5	F006	97	Avg. Volume	0.371035	0.328611	0.129039
LAMP1	W3	HPI4	well_1	F001	82	Avg. Volume	0.229966	0.222784	0.080323
LAMP1	W3	HPI4	well_1	F002	197	Avg. Volume	0.239511	0.222986	0.094503
LAMP1	W3	HPI4	well_1	F003	139	Avg. Volume	0.207802	0.208903	0.070859
LAMP1	W3	HPI4	well_1	F004	81	Avg. Volume	0.20369	0.186633	0.0904
LAMP1	W3	HPI4	well_1	F005	105	Avg. Volume	0.224257	0.21125	0.070991
LAMP1	W3	HPI4	well_1	F006	104	Avg. Volume	0.211203	0.205845	0.073599
LAMP1	W3	HPI4	well_2	F001	94	Avg. Volume	0.188295	0.188906	0.063671
LAMP1	W3	HPI4	well_2	F002	76	Avg. Volume	0.216759	0.215976	0.073851
LAMP1	W3	HPI4	well_2	F003	28	Avg. Volume	0.258117	0.253101	0.059313
LAMP1	W3	HPI4	well_2	F004	48	Avg. Volume	0.250378	0.23452	0.080604
LAMP1	W3	HPI4	well_2	F005	91	Avg. Volume	0.199355	0.190907	0.069774
LAMP1	W3	HPI4	well_2	F006	92	Avg. Volume	0.171706	0.163523	0.06319
LAMP1	W3	HPI4	well_3	F001	139	Avg. Volume	0.209355	0.21125	0.064617
LAMP1	W3	HPI4	well_3	F002	162	Avg. Volume	0.203117	0.193841	0.074575
LAMP1	W3	HPI4	well_3	F003	44	Avg. Volume	0.175151	0.15689	0.070459
LAMP1	W3	HPI4	well_3	F004	113	Avg. Volume	0.195025	0.166401	0.115786
LAMP1	W3	HPI4	well_3	F005	162	Avg. Volume	0.141639	0.129097	0.06629
LAMP1	W3	HPI4	well_3	F006	146	Avg. Volume	0.221993	0.212279	0.085323
LAMP1	W3	HPI4	well_4	F001	88	Avg. Volume	0.19216	0.174394	0.08447
LAMP1	W3	HPI4	well_4	F002	64	Avg. Volume	0.211128	0.196449	0.078533
LAMP1	W3	HPI4	well_4	F003	49	Avg. Volume	0.289198	0.277502	0.09168
LAMP1	W3	HPI4	well_4	F004	43	Avg. Volume	0.216384	0.176944	0.141531
LAMP1	W3	HPI4	well_4	F005	109	Avg. Volume	0.206097	0.179709	0.081119
LAMP1	W3	HPI4	well_4	F006	274	Avg. Volume	0.175588	0.160394	0.077576
LAMP1	W3	HPI4	well_5	F001	87	Avg. Volume	0.163414	0.150222	0.067522
LAMP1	W3	HPI4	well_5	F002	21	Avg. Volume	0.325159	0.263084	0.165096
LAMP1	W3	HPI4	well_5	F003	36	Avg. Volume	0.282208	0.260408	0.086701
LAMP1	W3	HPI4	well_5	F004	43	Avg. Volume	0.216715	0.178593	0.128578
LAMP1	W3	HPI4	well_5	F005	91	Avg. Volume	0.267893	0.260974	0.099436
LAMP1	W3	HPI4	well_5	F006	48	Avg. Volume	0.240348	0.224649	0.0809
LAMP1	W3	PGE2	well_1	F001	76	Avg. Volume	0.445062	0.45024	0.11894
LAMP1	W3	PGE2	well_1	F002	229	Avg. Volume	0.538651	0.529506	0.124509
LAMP1	W3	PGE2	well_1	F003	276	Avg. Volume	0.487263	0.488468	0.142929
LAMP1	W3	PGE2	well_1	F004	432	Avg. Volume	0.487881	0.489716	0.157676
LAMP1	W3	PGE2	well_1	F005	374	Avg. Volume	0.469524	0.459678	0.125942
LAMP1	W3	PGE2	well_1	F006	142	Avg. Volume	0.45784	0.443276	0.124627
LAMP1	W3	PGE2	well_2	F001	111	Avg. Volume	0.442796	0.406312	0.154871
LAMP1	W3	PGE2	well_2	F002	212	Avg. Volume	0.498766	0.489572	0.152709
LAMP1	W3	PGE2	well_2	F003	153	Avg. Volume	0.459729	0.466383	0.128039
LAMP1	W3	PGE2	well_2	F004	72	Avg. Volume	0.417	0.399028	0.153015
LAMP1	W3	PGE2	well_2	F005	215	Avg. Volume	0.486313	0.467639	0.158003
LAMP1	W3	PGE2	well_2	F006	327	Avg. Volume	0.554108	0.545729	0.128534
LAMP1	W3	PGE2	well_3	F001	55	Avg. Volume	0.393338	0.406852	0.180446

LAMP1	W3	PGE2	well_3	F002	257	Avg. Volume	0.49138	0.486573	0.154706
LAMP1	W3	PGE2	well_3	F003	360	Avg. Volume	0.532392	0.518969	0.154974
LAMP1	W3	PGE2	well_3	F004	181	Avg. Volume	0.515522	0.51945	0.188156
LAMP1	W3	PGE2	well_3	F005	120	Avg. Volume	0.521965	0.486521	0.169941
LAMP1	W3	PGE2	well_3	F006	159	Avg. Volume	0.488932	0.476857	0.148831
LAMP1	W3	PGE2	well_4	F001	76	Avg. Volume	0.438656	0.434103	0.133653
LAMP1	W3	PGE2	well_4	F002	215	Avg. Volume	0.466035	0.452679	0.162944
LAMP1	W3	PGE2	well_4	F003	103	Avg. Volume	0.4887	0.488222	0.183274
LAMP1	W3	PGE2	well_4	F004	64	Avg. Volume	0.423211	0.393439	0.135619
LAMP1	W3	PGE2	well_4	F005	150	Avg. Volume	0.50412	0.489492	0.144953
LAMP1	W3	PGE2	well_4	F006	213	Avg. Volume	0.500213	0.504035	0.128489
LAMP1	W3	PGE2	well_5	F001	76	Avg. Volume	0.49203	0.482233	0.156881
LAMP1	W3	PGE2	well_5	F002	59	Avg. Volume	0.465601	0.472378	0.142954
LAMP1	W3	PGE2	well_5	F003	176	Avg. Volume	0.5405	0.533241	0.129372
LAMP1	W3	PGE2	well_5	F004	143	Avg. Volume	0.541085	0.526658	0.144987
LAMP1	W3	PGE2	well_5	F005	362	Avg. Volume	0.542362	0.522559	0.171817
LAMP1	W3	PGE2	well_5	F006	80	Avg. Volume	0.52509	0.485288	0.160242
LAMP1	W4	HPI4	well_1	F001	109	Avg. Volume	0.180553	0.161372	0.072898
LAMP1	W4	HPI4	well_1	F002	115	Avg. Volume	0.194117	0.187778	0.068022
LAMP1	W4	HPI4	well_1	F003	105	Avg. Volume	0.243294	0.242335	0.076858
LAMP1	W4	HPI4	well_1	F004	93	Avg. Volume	0.223673	0.226187	0.081885
LAMP1	W4	HPI4	well_1	F005	162	Avg. Volume	0.192773	0.181788	0.084924
LAMP1	W4	HPI4	well_1	F006	120	Avg. Volume	0.172114	0.152569	0.099185
LAMP1	W4	HPI4	well_2	F001	90	Avg. Volume	0.185255	0.174086	0.070602
LAMP1	W4	HPI4	well_2	F002	201	Avg. Volume	0.208158	0.21125	0.073228
LAMP1	W4	HPI4	well_2	F003	76	Avg. Volume	0.29786	0.293096	0.08592
LAMP1	W4	HPI4	well_2	F004	176	Avg. Volume	0.170925	0.164306	0.074751
LAMP1	W4	HPI4	well_2	F005	141	Avg. Volume	0.249404	0.233913	0.097389
LAMP1	W4	HPI4	well_2	F006	164	Avg. Volume	0.211118	0.203836	0.072243
LAMP1	W4	HPI4	well_3	F001	259	Avg. Volume	0.226659	0.202391	0.09886
LAMP1	W4	HPI4	well_3	F002	261	Avg. Volume	0.188215	0.18191	0.067814
LAMP1	W4	HPI4	well_3	F003	174	Avg. Volume	0.196055	0.17271	0.095415
LAMP1	W4	HPI4	well_3	F004	221	Avg. Volume	0.230188	0.227151	0.076383
LAMP1	W4	HPI4	well_3	F005	206	Avg. Volume	0.182566	0.164306	0.080476
LAMP1	W4	HPI4	well_3	F006	253	Avg. Volume	0.177661	0.151667	0.105193
LAMP1	W4	HPI4	well_4	F001	218	Avg. Volume	0.233558	0.217171	0.094959
LAMP1	W4	HPI4	well_4	F002	244	Avg. Volume	0.177534	0.159611	0.075141
LAMP1	W4	HPI4	well_4	F003	75	Avg. Volume	0.213736	0.188616	0.096027
LAMP1	W4	HPI4	well_4	F004	215	Avg. Volume	0.266414	0.253092	0.093523
LAMP1	W4	HPI4	well_4	F005	222	Avg. Volume	0.239741	0.232881	0.085559
LAMP1	W4	HPI4	well_4	F006	145	Avg. Volume	0.196858	0.178976	0.094469
LAMP1	W4	HPI4	well_5	F001	169	Avg. Volume	0.277369	0.265471	0.094044
LAMP1	W4	HPI4	well_5	F002	162	Avg. Volume	0.304946	0.28854	0.102955
LAMP1	W4	HPI4	well_5	F003	83	Avg. Volume	0.296654	0.289892	0.077238
LAMP1	W4	HPI4	well_5	F004	116	Avg. Volume	0.343886	0.339768	0.084924
LAMP1	W4	HPI4	well_5	F005	146	Avg. Volume	0.261156	0.260346	0.102906
LAMP1	W4	HPI4	well_5	F006	265	Avg. Volume	0.276703	0.265823	0.099323
LAMP1	W4	PGE2	well_1	F001	60	Avg. Volume	0.359523	0.346215	0.114123
LAMP1	W4	PGE2	well_1	F002	75	Avg. Volume	0.407865	0.393812	0.128099

LAMP1	W4	PGE2	well_1	F003	207	Avg. Volume	0.439195	0.434236	0.142785
LAMP1	W4	PGE2	well_1	F004	314	Avg. Volume	0.450709	0.444433	0.132277
LAMP1	W4	PGE2	well_1	F005	128	Avg. Volume	0.430044	0.392508	0.162009
LAMP1	W4	PGE2	well_1	F006	224	Avg. Volume	0.383263	0.369913	0.12921
LAMP1	W4	PGE2	well_2	F001	44	Avg. Volume	0.570745	0.593258	0.174989
LAMP1	W4	PGE2	well_2	F002	172	Avg. Volume	0.269057	0.242546	0.124532
LAMP1	W4	PGE2	well_2	F003	294	Avg. Volume	0.391426	0.375556	0.170371
LAMP1	W4	PGE2	well_2	F004	203	Avg. Volume	0.537985	0.516389	0.196349
LAMP1	W4	PGE2	well_2	F005	290	Avg. Volume	0.505108	0.495286	0.163899
LAMP1	W4	PGE2	well_2	F006	142	Avg. Volume	0.475976	0.461991	0.147246
LAMP1	W4	PGE2	well_3	F001	39	Avg. Volume	0.381579	0.37034	0.13287
LAMP1	W4	PGE2	well_3	F002	130	Avg. Volume	0.431161	0.421196	0.13622
LAMP1	W4	PGE2	well_3	F003	268	Avg. Volume	0.545746	0.522775	0.145331
LAMP1	W4	PGE2	well_3	F004	57	Avg. Volume	0.430104	0.356351	0.199671
LAMP1	W4	PGE2	well_3	F005	258	Avg. Volume	0.491925	0.463185	0.178382
LAMP1	W4	PGE2	well_3	F006	174	Avg. Volume	0.40202	0.393822	0.154361
LAMP1	W4	PGE2	well_4	F001	93	Avg. Volume	0.418438	0.40783	0.135232
LAMP1	W4	PGE2	well_4	F002	315	Avg. Volume	0.315229	0.289491	0.131752
LAMP1	W4	PGE2	well_4	F003	166	Avg. Volume	0.506945	0.456535	0.205569
LAMP1	W4	PGE2	well_4	F004	255	Avg. Volume	0.495994	0.478247	0.190544
LAMP1	W4	PGE2	well_4	F005	286	Avg. Volume	0.506455	0.495711	0.174837
LAMP1	W4	PGE2	well_4	F006	131	Avg. Volume	0.518695	0.488222	0.176549
LAMP1	W4	PGE2	well_5	F001	123	Avg. Volume	0.375672	0.359552	0.160451
LAMP1	W4	PGE2	well_5	F002	235	Avg. Volume	0.461928	0.444905	0.14679
LAMP1	W4	PGE2	well_5	F003	382	Avg. Volume	0.517737	0.495688	0.156002
LAMP1	W4	PGE2	well_5	F004	239	Avg. Volume	0.462647	0.457391	0.134317
LAMP1	W4	PGE2	well_5	F005	174	Avg. Volume	0.453196	0.440966	0.137777
LAMP1	W4	PGE2	well_5	F006	142	Avg. Volume	0.439391	0.437992	0.138873
LAMP1	W1	HPI4	well_1	F001	272	Count/cell	35.99184	34	14.87902
LAMP1	W1	HPI4	well_1	F002	347	Count/cell	28.73482	27	12.21456
LAMP1	W1	HPI4	well_1	F003	207	Count/cell	21.28877	20	10.73443
LAMP1	W1	HPI4	well_1	F004	346	Count/cell	30.40256	29	12.61248
LAMP1	W1	HPI4	well_1	F005	399	Count/cell	21.38889	20.5	9.166299
LAMP1	W1	HPI4	well_1	F006	250	Count/cell	27.76106	26	11.4401
LAMP1	W1	HPI4	well_2	F001	103	Count/cell	25.3617	23.5	12.81536
LAMP1	W1	HPI4	well_2	F002	241	Count/cell	24.12844	23	10.43576
LAMP1	W1	HPI4	well_2	F003	207	Count/cell	27.82162	27	11.67008
LAMP1	W1	HPI4	well_2	F004	323	Count/cell	29.23368	29	9.9589
LAMP1	W1	HPI4	well_2	F005	297	Count/cell	33.03346	30	15.43314
LAMP1	W1	HPI4	well_2	F006	185	Count/cell	24.18182	22	11.31062
LAMP1	W1	HPI4	well_3	F001	68	Count/cell	32.05	29	13.12189
LAMP1	W1	HPI4	well_3	F002	257	Count/cell	25.9188	23	13.75373
LAMP1	W1	HPI4	well_3	F003	70	Count/cell	34.45161	33	14.73334
LAMP1	W1	HPI4	well_3	F004	279	Count/cell	27.90551	25	11.58535
LAMP1	W1	HPI4	well_3	F005	329	Count/cell	27.3661	24	14.00249
LAMP1	W1	HPI4	well_3	F006	214	Count/cell	25.81771	23.5	11.62877
LAMP1	W1	HPI4	well_4	F001	282	Count/cell	30.13439	29	11.58465
LAMP1	W1	HPI4	well_4	F002	295	Count/cell	23.39474	22	10.28308
LAMP1	W1	HPI4	well_4	F003	265	Count/cell	31.12605	29.5	14.97906

LAMP1	W1	HPI4	well_4	F004	81	Count/cell	29.01351	28	10.97131
LAMP1	W1	HPI4	well_4	F005	205	Count/cell	20.22162	19	9.356442
LAMP1	W1	HPI4	well_4	F006	167	Count/cell	21.49007	21	9.61656
LAMP1	W1	HPI4	well_5	F001	329	Count/cell	27.78983	27	10.87232
LAMP1	W1	HPI4	well_5	F002	209	Count/cell	28.3369	26	13.48551
LAMP1	W1	HPI4	well_5	F003	84	Count/cell	30.28378	29	13.8589
LAMP1	W1	HPI4	well_5	F004	161	Count/cell	26.27397	24	13.98251
LAMP1	W1	HPI4	well_5	F005	302	Count/cell	26.4652	24	11.14311
LAMP1	W1	HPI4	well_5	F006	233	Count/cell	26.25592	25	11.32216
LAMP1	W1	PGE2	well_1	F001	314	Count/cell	35.47887	34	14.33088
LAMP1	W1	PGE2	well_1	F002	389	Count/cell	34.16997	31	15.68129
LAMP1	W1	PGE2	well_1	F003	304	Count/cell	33.53285	32	14.11598
LAMP1	W1	PGE2	well_1	F004	312	Count/cell	32.57092	30	15.11464
LAMP1	W1	PGE2	well_1	F005	438	Count/cell	24.84304	22	12.0963
LAMP1	W1	PGE2	well_1	F006	411	Count/cell	30.49864	29	11.95715
LAMP1	W1	PGE2	well_2	F001	334	Count/cell	30.20598	28	12.09893
LAMP1	W1	PGE2	well_2	F002	387	Count/cell	30.98286	29	15.01269
LAMP1	W1	PGE2	well_2	F003	391	Count/cell	28.90341	26	13.00194
LAMP1	W1	PGE2	well_2	F004	290	Count/cell	34.37405	32	14.44229
LAMP1	W1	PGE2	well_2	F005	315	Count/cell	29.46479	27	11.09016
LAMP1	W1	PGE2	well_2	F006	370	Count/cell	33.03012	31	13.90143
LAMP1	W1	PGE2	well_3	F001	336	Count/cell	32.17822	29	14.62991
LAMP1	W1	PGE2	well_3	F002	290	Count/cell	32.83969	31	13.54138
LAMP1	W1	PGE2	well_3	F003	289	Count/cell	28.92278	27	13.00841
LAMP1	W1	PGE2	well_3	F004	236	Count/cell	42.86385	41	16.76292
LAMP1	W1	PGE2	well_3	F005	444	Count/cell	22.22388	20	10.87538
LAMP1	W1	PGE2	well_3	F006	420	Count/cell	26.19737	25	10.7146
LAMP1	W1	PGE2	well_4	F001	320	Count/cell	32.28028	31	13.8188
LAMP1	W1	PGE2	well_4	F002	371	Count/cell	30.2819	28	13.92008
LAMP1	W1	PGE2	well_4	F003	179	Count/cell	30.17284	30	10.4754
LAMP1	W1	PGE2	well_4	F004	328	Count/cell	27.40203	25	12.90292
LAMP1	W1	PGE2	well_4	F005	375	Count/cell	24.87941	24	11.84836
LAMP1	W1	PGE2	well_4	F006	348	Count/cell	28.47284	27	11.33692
LAMP1	W1	PGE2	well_5	F001	299	Count/cell	34.91144	31	16.24852
LAMP1	W1	PGE2	well_5	F002	351	Count/cell	25.91429	25	12.58433
LAMP1	W1	PGE2	well_5	F003	131	Count/cell	39.32203	36	16.09793
LAMP1	W1	PGE2	well_5	F004	302	Count/cell	30.50549	27	13.40712
LAMP1	W1	PGE2	well_5	F005	339	Count/cell	27.57655	27	11.94007
LAMP1	W1	PGE2	well_5	F006	256	Count/cell	29.90948	28.5	12.79054
LAMP1	W2	HPI4	well_1	F001	299	Count/cell	30.17037	24	18.47596
LAMP1	W2	HPI4	well_1	F002	376	Count/cell	40.53097	37	19.30129
LAMP1	W2	HPI4	well_1	F003	372	Count/cell	19.84211	16	15.44506
LAMP1	W2	HPI4	well_1	F004	229	Count/cell	64.68447	61	30.4274
LAMP1	W2	HPI4	well_1	F005	216	Count/cell	54.51282	51	24.44463
LAMP1	W2	HPI4	well_1	F006	415	Count/cell	11.14213	9	8.240661
LAMP1	W2	HPI4	well_2	F001	357	Count/cell	29.94753	26	18.39068
LAMP1	W2	HPI4	well_2	F002	291	Count/cell	38.6553	36.5	18.39032
LAMP1	W2	HPI4	well_2	F003	344	Count/cell	39.8539	37	21.48194
LAMP1	W2	HPI4	well_2	F004	332	Count/cell	23.73	19	17.21875

LAMP1	W2	HPI4	well_2	F005	325	Count/cell	26.19113	23	16.13293
LAMP1	W2	HPI4	well_2	F006	409	Count/cell	33.872	31	19.2902
LAMP1	W2	HPI4	well_3	F001	346	Count/cell	32.59425	30	18.01971
LAMP1	W2	HPI4	well_3	F002	406	Count/cell	25.57418	24	15.9328
LAMP1	W2	HPI4	well_3	F003	153	Count/cell	52.81159	50.5	23.78141
LAMP1	W2	HPI4	well_3	F004	273	Count/cell	65.75102	61	28.65723
LAMP1	W2	HPI4	well_3	F005	210	Count/cell	31.34392	25	21.47916
LAMP1	W2	HPI4	well_3	F006	400	Count/cell	21.82597	18	14.65939
LAMP1	W2	HPI4	well_4	F001	475	Count/cell	31.8548	29	17.85564
LAMP1	W2	HPI4	well_4	F002	445	Count/cell	15.16209	14	9.945158
LAMP1	W2	HPI4	well_4	F003	274	Count/cell	40.69919	39	22.49986
LAMP1	W2	HPI4	well_4	F004	388	Count/cell	25.67797	22	15.85695
LAMP1	W2	HPI4	well_4	F005	275	Count/cell	20.66935	18.5	11.63773
LAMP1	W2	HPI4	well_4	F006	414	Count/cell	16.93506	13	12.51784
LAMP1	W2	HPI4	well_5	F001	352	Count/cell	22.88013	19	14.98918
LAMP1	W2	HPI4	well_5	F002	361	Count/cell	25.19018	22	15.11232
LAMP1	W2	HPI4	well_5	F003	268	Count/cell	45.01646	40	22.97735
LAMP1	W2	HPI4	well_5	F004	267	Count/cell	36.52917	32.5	19.49078
LAMP1	W2	HPI4	well_5	F005	229	Count/cell	15.10599	11	13.01789
LAMP1	W2	HPI4	well_5	F006	293	Count/cell	8.76259	6	7.989237
LAMP1	W2	PGE2	well_1	F001	249	Count/cell	19.3125	18	8.646554
LAMP1	W2	PGE2	well_1	F002	283	Count/cell	22.40541	21	10.29985
LAMP1	W2	PGE2	well_1	F003	198	Count/cell	19.07735	17	9.13872
LAMP1	W2	PGE2	well_1	F004	174	Count/cell	26.35669	23	12.63441
LAMP1	W2	PGE2	well_1	F005	235	Count/cell	30.65566	29	11.99839
LAMP1	W2	PGE2	well_1	F006	381	Count/cell	27.59942	25	13.10687
LAMP1	W2	PGE2	well_2	F001	162	Count/cell	22.4898	20	8.492839
LAMP1	W2	PGE2	well_2	F002	82	Count/cell	20.63514	21	7.062489
LAMP1	W2	PGE2	well_2	F003	370	Count/cell	21.11976	20	9.202952
LAMP1	W2	PGE2	well_2	F004	242	Count/cell	22.06912	19	10.51937
LAMP1	W2	PGE2	well_2	F005	233	Count/cell	21.27619	19.5	10.29702
LAMP1	W2	PGE2	well_2	F006	128	Count/cell	19.14783	18	8.771208
LAMP1	W2	PGE2	well_3	F001	300	Count/cell	22.44853	21	11.02384
LAMP1	W2	PGE2	well_3	F002	274	Count/cell	24.32927	23	8.623728
LAMP1	W2	PGE2	well_3	F003	179	Count/cell	17.01227	15	7.824453
LAMP1	W2	PGE2	well_3	F004	280	Count/cell	16.48638	15	8.171072
LAMP1	W2	PGE2	well_3	F005	261	Count/cell	21.73729	19	10.82387
LAMP1	W2	PGE2	well_3	F006	213	Count/cell	20.58247	19.5	9.126395
LAMP1	W2	PGE2	well_4	F001	202	Count/cell	24.73889	23	10.11408
LAMP1	W2	PGE2	well_4	F002	226	Count/cell	23.61084	24	9.407774
LAMP1	W2	PGE2	well_4	F003	300	Count/cell	20.02952	19	8.881058
LAMP1	W2	PGE2	well_4	F004	291	Count/cell	18.47191	17	9.035964
LAMP1	W2	PGE2	well_4	F005	202	Count/cell	22.03846	19	11.96513
LAMP1	W2	PGE2	well_4	F006	252	Count/cell	24.52174	22	12.76208
LAMP1	W2	PGE2	well_5	F001	319	Count/cell	25.97561	24	11.32465
LAMP1	W2	PGE2	well_5	F002	218	Count/cell	23.7602	22	9.594264
LAMP1	W2	PGE2	well_5	F003	381	Count/cell	17.20461	14	11.75404
LAMP1	W2	PGE2	well_5	F004	272	Count/cell	15.61943	14	7.427215
LAMP1	W2	PGE2	well_5	F005	376	Count/cell	19.50585	17	10.72989

LAMP1	W2	PGE2	well_5	F006	97	Count/cell	16.32955	16	6.540276
LAMP1	W3	HPI4	well_1	F001	82	Count/cell	32.06757	33	17.76817
LAMP1	W3	HPI4	well_1	F002	197	Count/cell	31.24157	29	16.49767
LAMP1	W3	HPI4	well_1	F003	139	Count/cell	28.46094	22.5	19.82876
LAMP1	W3	HPI4	well_1	F004	81	Count/cell	30.28767	28	18.05276
LAMP1	W3	HPI4	well_1	F005	105	Count/cell	37.51579	34	18.62786
LAMP1	W3	HPI4	well_1	F006	104	Count/cell	34.60638	30.5	18.13107
LAMP1	W3	HPI4	well_2	F001	94	Count/cell	32.45238	28.5	19.2888
LAMP1	W3	HPI4	well_2	F002	76	Count/cell	48.82609	47	20.01981
LAMP1	W3	HPI4	well_2	F003	28	Count/cell	69.54167	72.5	19.93899
LAMP1	W3	HPI4	well_2	F004	48	Count/cell	35.45238	33	20.66717
LAMP1	W3	HPI4	well_2	F005	91	Count/cell	36.92683	35	15.19323
LAMP1	W3	HPI4	well_2	F006	92	Count/cell	20.79762	16	13.36943
LAMP1	W3	HPI4	well_3	F001	139	Count/cell	29.49606	28	14.27723
LAMP1	W3	HPI4	well_3	F002	162	Count/cell	27.66897	26	15.407
LAMP1	W3	HPI4	well_3	F003	44	Count/cell	24.71795	24	12.62818
LAMP1	W3	HPI4	well_3	F004	113	Count/cell	17.39216	12.5	12.49725
LAMP1	W3	HPI4	well_3	F005	162	Count/cell	7.27451	5	6.549644
LAMP1	W3	HPI4	well_3	F006	146	Count/cell	30.0229	27	16.8776
LAMP1	W3	HPI4	well_4	F001	88	Count/cell	21.5	19	15.13725
LAMP1	W3	HPI4	well_4	F002	64	Count/cell	31.86207	26	21.25654
LAMP1	W3	HPI4	well_4	F003	49	Count/cell	71.81395	69	29.56209
LAMP1	W3	HPI4	well_4	F004	43	Count/cell	19.76316	19	11.9852
LAMP1	W3	HPI4	well_4	F005	109	Count/cell	30.45455	29	16.12785
LAMP1	W3	HPI4	well_4	F006	274	Count/cell	11.13462	10	7.939999
LAMP1	W3	HPI4	well_5	F001	87	Count/cell	16.7125	16	13.2409
LAMP1	W3	HPI4	well_5	F002	21	Count/cell	44.10526	40	20.50987
LAMP1	W3	HPI4	well_5	F003	36	Count/cell	83.46875	80	33.95821
LAMP1	W3	HPI4	well_5	F004	43	Count/cell	26.91892	23	15.70806
LAMP1	W3	HPI4	well_5	F005	91	Count/cell	55.45679	55	24.80476
LAMP1	W3	HPI4	well_5	F006	48	Count/cell	56.97619	45.5	33.20703
LAMP1	W3	PGE2	well_1	F001	76	Count/cell	18.68116	16	8.92698
LAMP1	W3	PGE2	well_1	F002	229	Count/cell	25.10096	22.5	11.37649
LAMP1	W3	PGE2	well_1	F003	276	Count/cell	19.95181	18	8.941436
LAMP1	W3	PGE2	well_1	F004	432	Count/cell	17.81218	16	8.467585
LAMP1	W3	PGE2	well_1	F005	374	Count/cell	21.47478	21	7.37879
LAMP1	W3	PGE2	well_1	F006	142	Count/cell	20.5	17	12.20817
LAMP1	W3	PGE2	well_2	F001	111	Count/cell	13	12	6.451059
LAMP1	W3	PGE2	well_2	F002	212	Count/cell	17.86911	15	9.402051
LAMP1	W3	PGE2	well_2	F003	153	Count/cell	23.99275	23	10.48774
LAMP1	W3	PGE2	well_2	F004	72	Count/cell	13.74242	14	6.535843
LAMP1	W3	PGE2	well_2	F005	215	Count/cell	19.52062	18	9.518027
LAMP1	W3	PGE2	well_2	F006	327	Count/cell	23.44218	22	9.029061
LAMP1	W3	PGE2	well_3	F001	55	Count/cell	13.86538	10	13.51837
LAMP1	W3	PGE2	well_3	F002	257	Count/cell	21.24034	19	10.74239
LAMP1	W3	PGE2	well_3	F003	360	Count/cell	15.78834	15	6.045569
LAMP1	W3	PGE2	well_3	F004	181	Count/cell	14.57576	14	5.640826
LAMP1	W3	PGE2	well_3	F005	120	Count/cell	19.91667	17.5	8.441248
LAMP1	W3	PGE2	well_3	F006	159	Count/cell	20.86577	19	10.03821

LAMP1	W3	PGE2	well_4	F001	76	Count/cell	13	11	6.830572
LAMP1	W3	PGE2	well_4	F002	215	Count/cell	14.7513	13	8.43667
LAMP1	W3	PGE2	well_4	F003	103	Count/cell	11.52174	11	4.117714
LAMP1	W3	PGE2	well_4	F004	64	Count/cell	18.40351	16	8.766535
LAMP1	W3	PGE2	well_4	F005	150	Count/cell	15.27612	14	6.630293
LAMP1	W3	PGE2	well_4	F006	213	Count/cell	22.07732	19	11.61289
LAMP1	W3	PGE2	well_5	F001	76	Count/cell	16.94203	15	7.09574
LAMP1	W3	PGE2	well_5	F002	59	Count/cell	18.41509	17	6.890445
LAMP1	W3	PGE2	well_5	F003	176	Count/cell	20.33544	18	8.785201
LAMP1	W3	PGE2	well_5	F004	143	Count/cell	15.78295	15	5.985609
LAMP1	W3	PGE2	well_5	F005	362	Count/cell	16.91692	15	7.579365
LAMP1	W3	PGE2	well_5	F006	80	Count/cell	20.0411	19	6.644627
LAMP1	W4	HPI4	well_1	F001	109	Count/cell	15.87129	13	12.52091
LAMP1	W4	HPI4	well_1	F002	115	Count/cell	23.97115	21	13.87283
LAMP1	W4	HPI4	well_1	F003	105	Count/cell	56.88172	59	27.06428
LAMP1	W4	HPI4	well_1	F004	93	Count/cell	35.66265	33	20.68479
LAMP1	W4	HPI4	well_1	F005	162	Count/cell	17.42568	14.5	14.05194
LAMP1	W4	HPI4	well_1	F006	120	Count/cell	9.245614	7.5	7.370788
LAMP1	W4	HPI4	well_2	F001	90	Count/cell	22.7125	18.5	13.643
LAMP1	W4	HPI4	well_2	F002	201	Count/cell	25.89617	23	13.1356
LAMP1	W4	HPI4	well_2	F003	76	Count/cell	64.39706	57.5	30.11626
LAMP1	W4	HPI4	well_2	F004	176	Count/cell	16.2515	13	13.02302
LAMP1	W4	HPI4	well_2	F005	141	Count/cell	38.63281	37.5	20.50384
LAMP1	W4	HPI4	well_2	F006	164	Count/cell	28.18792	25	19.0952
LAMP1	W4	HPI4	well_3	F001	259	Count/cell	29.76793	24	21.42807
LAMP1	W4	HPI4	well_3	F002	261	Count/cell	20.99167	18	13.87813
LAMP1	W4	HPI4	well_3	F003	174	Count/cell	26.37342	23	17.40726
LAMP1	W4	HPI4	well_3	F004	221	Count/cell	36.22886	33	19.08134
LAMP1	W4	HPI4	well_3	F005	206	Count/cell	17.0107	15	11.00195
LAMP1	W4	HPI4	well_3	F006	253	Count/cell	9.916667	8	7.763861
LAMP1	W4	HPI4	well_4	F001	218	Count/cell	34.99492	31	21.0227
LAMP1	W4	HPI4	well_4	F002	244	Count/cell	25.6347	21	18.20737
LAMP1	W4	HPI4	well_4	F003	75	Count/cell	37.77941	37	24.74638
LAMP1	W4	HPI4	well_4	F004	215	Count/cell	33.5	29	20.51661
LAMP1	W4	HPI4	well_4	F005	222	Count/cell	34.48241	32	19.09674
LAMP1	W4	HPI4	well_4	F006	145	Count/cell	15.10949	13	12.02918
LAMP1	W4	HPI4	well_5	F001	169	Count/cell	54.96689	48	29.3254
LAMP1	W4	HPI4	well_5	F002	162	Count/cell	86.95862	84	38.94741
LAMP1	W4	HPI4	well_5	F003	83	Count/cell	87.58108	77	45.03302
LAMP1	W4	HPI4	well_5	F004	116	Count/cell	81.38095	76	34.79428
LAMP1	W4	HPI4	well_5	F005	146	Count/cell	32.95385	28	22.49853
LAMP1	W4	HPI4	well_5	F006	265	Count/cell	27.04508	24.5	13.93496
LAMP1	W4	PGE2	well_1	F001	60	Count/cell	21.24074	19	10.14412
LAMP1	W4	PGE2	well_1	F002	75	Count/cell	27.1194	23	12.73806
LAMP1	W4	PGE2	well_1	F003	207	Count/cell	20.46277	18	9.078605
LAMP1	W4	PGE2	well_1	F004	314	Count/cell	22.72125	22	8.959446
LAMP1	W4	PGE2	well_1	F005	128	Count/cell	18.26087	15	8.76605
LAMP1	W4	PGE2	well_1	F006	224	Count/cell	16.295	15	6.715913
LAMP1	W4	PGE2	well_2	F001	44	Count/cell	20.5	20.5	5.726892

LAMP1	W4	PGE2	well_2	F002	172	Count/cell	18.27273	19	11.55811
LAMP1	W4	PGE2	well_2	F003	294	Count/cell	9.072464	8	5.908561
LAMP1	W4	PGE2	well_2	F004	203	Count/cell	16.77174	16	7.690891
LAMP1	W4	PGE2	well_2	F005	290	Count/cell	16.79699	16	5.734713
LAMP1	W4	PGE2	well_2	F006	142	Count/cell	20.39683	19	7.130867
LAMP1	W4	PGE2	well_3	F001	39	Count/cell	17.54286	14	9.353649
LAMP1	W4	PGE2	well_3	F002	130	Count/cell	19.11864	18	7.36261
LAMP1	W4	PGE2	well_3	F003	268	Count/cell	22.5	21	8.867221
LAMP1	W4	PGE2	well_3	F004	57	Count/cell	13.7037	11.5	6.916291
LAMP1	W4	PGE2	well_3	F005	258	Count/cell	18.7437	18	7.263389
LAMP1	W4	PGE2	well_3	F006	174	Count/cell	19.39241	18	9.266635
LAMP1	W4	PGE2	well_4	F001	93	Count/cell	16.66265	15	8.823855
LAMP1	W4	PGE2	well_4	F002	315	Count/cell	8.679577	7	5.057913
LAMP1	W4	PGE2	well_4	F003	166	Count/cell	12.46795	12	5.897424
LAMP1	W4	PGE2	well_4	F004	255	Count/cell	19.40517	18.5	9.520066
LAMP1	W4	PGE2	well_4	F005	286	Count/cell	16.91538	16	6.931585
LAMP1	W4	PGE2	well_4	F006	131	Count/cell	20.52542	20	6.657316
LAMP1	W4	PGE2	well_5	F001	123	Count/cell	14.18182	12	7.288525
LAMP1	W4	PGE2	well_5	F002	235	Count/cell	19.95283	18	9.364025
LAMP1	W4	PGE2	well_5	F003	382	Count/cell	20.05476	19	7.177751
LAMP1	W4	PGE2	well_5	F004	239	Count/cell	21.09174	19	9.231824
LAMP1	W4	PGE2	well_5	F005	174	Count/cell	19.49682	18	7.999699
LAMP1	W4	PGE2	well_5	F006	142	Count/cell	19.2619	18.5	7.394245
LAMP1	W1	HPI4	well_1	F001	272	X/Y distribution	5.724348	5.647613	1.022346
LAMP1	W1	HPI4	well_1	F002	347	X/Y distribution	5.016819	4.967981	0.901813
LAMP1	W1	HPI4	well_1	F003	207	X/Y distribution	4.838027	4.878292	1.023743
LAMP1	W1	HPI4	well_1	F004	346	X/Y distribution	5.465481	5.408039	0.952029
LAMP1	W1	HPI4	well_1	F005	399	X/Y distribution	4.764973	4.760224	0.91592
LAMP1	W1	HPI4	well_1	F006	250	X/Y distribution	4.93714	4.890645	0.957848
LAMP1	W1	HPI4	well_2	F001	103	X/Y distribution	4.640617	4.622886	0.974305
LAMP1	W1	HPI4	well_2	F002	241	X/Y distribution	5.040926	4.946134	0.98438
LAMP1	W1	HPI4	well_2	F003	207	X/Y distribution	5.476627	5.364673	1.082162
LAMP1	W1	HPI4	well_2	F004	323	X/Y distribution	5.378933	5.296906	0.891505
LAMP1	W1	HPI4	well_2	F005	297	X/Y distribution	5.589899	5.544192	1.034848
LAMP1	W1	HPI4	well_2	F006	185	X/Y distribution	4.384182	4.261229	0.929923
LAMP1	W1	HPI4	well_3	F001	68	X/Y distribution	5.519702	5.458663	0.920127
LAMP1	W1	HPI4	well_3	F002	257	X/Y distribution	4.946806	4.915919	1.005059
LAMP1	W1	HPI4	well_3	F003	70	X/Y distribution	5.722109	5.630402	1.115279
LAMP1	W1	HPI4	well_3	F004	279	X/Y distribution	5.515549	5.468653	0.89661
LAMP1	W1	HPI4	well_3	F005	329	X/Y distribution	5.15004	5.08516	0.971113
LAMP1	W1	HPI4	well_3	F006	214	X/Y distribution	5.104153	5.06274	0.918104
LAMP1	W1	HPI4	well_4	F001	282	X/Y distribution	5.471144	5.423988	0.994677
LAMP1	W1	HPI4	well_4	F002	295	X/Y distribution	4.858476	4.760214	0.974175
LAMP1	W1	HPI4	well_4	F003	265	X/Y distribution	5.700709	5.635953	1.044004
LAMP1	W1	HPI4	well_4	F004	81	X/Y distribution	5.772693	5.724351	1.008032
LAMP1	W1	HPI4	well_4	F005	205	X/Y distribution	4.666983	4.756038	1.098251
LAMP1	W1	HPI4	well_4	F006	167	X/Y distribution	4.481812	4.518532	1.100159
LAMP1	W1	HPI4	well_5	F001	329	X/Y distribution	4.949674	4.848508	0.773504
LAMP1	W1	HPI4	well_5	F002	209	X/Y distribution	5.107196	5.181472	1.019485

LAMP1	W1	HPI4	well_5	F003	84	X/Y distribution	5.563498	5.483439	1.318069
LAMP1	W1	HPI4	well_5	F004	161	X/Y distribution	5.000515	5.036331	1.114227
LAMP1	W1	HPI4	well_5	F005	302	X/Y distribution	4.970809	4.92107	0.894759
LAMP1	W1	HPI4	well_5	F006	233	X/Y distribution	4.893327	4.793188	0.9152
LAMP1	W1	PGE2	well_1	F001	314	X/Y distribution	5.462635	5.432656	0.95655
LAMP1	W1	PGE2	well_1	F002	389	X/Y distribution	5.408974	5.370774	0.97909
LAMP1	W1	PGE2	well_1	F003	304	X/Y distribution	5.661724	5.634291	1.073135
LAMP1	W1	PGE2	well_1	F004	312	X/Y distribution	5.838933	5.661037	1.241556
LAMP1	W1	PGE2	well_1	F005	438	X/Y distribution	4.770036	4.74925	0.931382
LAMP1	W1	PGE2	well_1	F006	411	X/Y distribution	4.312264	4.250508	0.657443
LAMP1	W1	PGE2	well_2	F001	334	X/Y distribution	5.14279	5.148192	1.034053
LAMP1	W1	PGE2	well_2	F002	387	X/Y distribution	5.116178	5.148028	1.0703
LAMP1	W1	PGE2	well_2	F003	391	X/Y distribution	5.20035	5.190166	0.904785
LAMP1	W1	PGE2	well_2	F004	290	X/Y distribution	5.838445	5.709808	1.047108
LAMP1	W1	PGE2	well_2	F005	315	X/Y distribution	5.370295	5.293995	0.95254
LAMP1	W1	PGE2	well_2	F006	370	X/Y distribution	4.985256	4.955015	0.771358
LAMP1	W1	PGE2	well_3	F001	336	X/Y distribution	5.096963	5.004871	0.899847
LAMP1	W1	PGE2	well_3	F002	290	X/Y distribution	5.778468	5.772011	0.942758
LAMP1	W1	PGE2	well_3	F003	289	X/Y distribution	5.820033	5.819389	1.034478
LAMP1	W1	PGE2	well_3	F004	236	X/Y distribution	6.154849	6.059718	0.91333
LAMP1	W1	PGE2	well_3	F005	444	X/Y distribution	4.702472	4.650117	0.978441
LAMP1	W1	PGE2	well_3	F006	420	X/Y distribution	5.078945	5.016803	0.817014
LAMP1	W1	PGE2	well_4	F001	320	X/Y distribution	5.618792	5.631244	1.057993
LAMP1	W1	PGE2	well_4	F002	371	X/Y distribution	5.325559	5.277979	1.051061
LAMP1	W1	PGE2	well_4	F003	179	X/Y distribution	5.863862	5.700429	0.979148
LAMP1	W1	PGE2	well_4	F004	328	X/Y distribution	5.134799	5.032373	1.097889
LAMP1	W1	PGE2	well_4	F005	375	X/Y distribution	4.983808	5.081069	1.046223
LAMP1	W1	PGE2	well_4	F006	348	X/Y distribution	5.159885	5.147384	0.953963
LAMP1	W1	PGE2	well_5	F001	299	X/Y distribution	5.476073	5.371979	1.111892
LAMP1	W1	PGE2	well_5	F002	351	X/Y distribution	4.922054	4.839895	1.057792
LAMP1	W1	PGE2	well_5	F003	131	X/Y distribution	6.285004	6.041778	1.32256
LAMP1	W1	PGE2	well_5	F004	302	X/Y distribution	5.663595	5.546896	1.029642
LAMP1	W1	PGE2	well_5	F005	339	X/Y distribution	5.510273	5.487565	1.002603
LAMP1	W1	PGE2	well_5	F006	256	X/Y distribution	4.822017	4.733953	0.913657
LAMP1	W2	HPI4	well_1	F001	299	X/Y distribution	5.538917	5.560347	1.161789
LAMP1	W2	HPI4	well_1	F002	376	X/Y distribution	5.437996	5.497177	0.99483
LAMP1	W2	HPI4	well_1	F003	372	X/Y distribution	5.775084	5.741638	1.386794
LAMP1	W2	HPI4	well_1	F004	229	X/Y distribution	6.626747	6.501597	1.221799
LAMP1	W2	HPI4	well_1	F005	216	X/Y distribution	6.663543	6.686963	1.047198
LAMP1	W2	HPI4	well_1	F006	415	X/Y distribution	5.495058	5.530675	1.20185
LAMP1	W2	HPI4	well_2	F001	357	X/Y distribution	5.486516	5.48276	1.070109
LAMP1	W2	HPI4	well_2	F002	291	X/Y distribution	5.813513	5.888346	1.01718
LAMP1	W2	HPI4	well_2	F003	344	X/Y distribution	5.84742	5.893069	1.015758
LAMP1	W2	HPI4	well_2	F004	332	X/Y distribution	5.729408	5.790044	1.174834
LAMP1	W2	HPI4	well_2	F005	325	X/Y distribution	6.098712	6.133519	1.283916
LAMP1	W2	HPI4	well_2	F006	409	X/Y distribution	5.703947	5.737261	1.090943
LAMP1	W2	HPI4	well_3	F001	346	X/Y distribution	5.658651	5.645518	1.204668
LAMP1	W2	HPI4	well_3	F002	406	X/Y distribution	5.373072	5.407618	1.04583
LAMP1	W2	HPI4	well_3	F003	153	X/Y distribution	7.384776	7.50691	1.40294

LAMP1	W2	HPI4	well_3	F004	273	X/Y distribution	6.696125	6.640394	1.138755
LAMP1	W2	HPI4	well_3	F005	210	X/Y distribution	6.883933	6.934334	1.412589
LAMP1	W2	HPI4	well_3	F006	400	X/Y distribution	5.44071	5.417414	1.219661
LAMP1	W2	HPI4	well_4	F001	475	X/Y distribution	5.48162	5.559876	1.105039
LAMP1	W2	HPI4	well_4	F002	445	X/Y distribution	5.347885	5.394011	1.192056
LAMP1	W2	HPI4	well_4	F003	274	X/Y distribution	6.381742	6.397282	1.045822
LAMP1	W2	HPI4	well_4	F004	388	X/Y distribution	5.71312	5.711163	1.137131
LAMP1	W2	HPI4	well_4	F005	275	X/Y distribution	6.35045	6.173901	1.092143
LAMP1	W2	HPI4	well_4	F006	414	X/Y distribution	5.505328	5.612459	1.278828
LAMP1	W2	HPI4	well_5	F001	352	X/Y distribution	5.828718	5.860498	1.111418
LAMP1	W2	HPI4	well_5	F002	361	X/Y distribution	5.648984	5.609426	1.086083
LAMP1	W2	HPI4	well_5	F003	268	X/Y distribution	6.234348	6.256354	1.038531
LAMP1	W2	HPI4	well_5	F004	267	X/Y distribution	6.29285	6.209007	1.162154
LAMP1	W2	HPI4	well_5	F005	229	X/Y distribution	6.798473	6.809435	1.390578
LAMP1	W2	HPI4	well_5	F006	293	X/Y distribution	5.879968	5.948965	1.464024
LAMP1	W2	PGE2	well_1	F001	249	X/Y distribution	3.839775	3.817076	0.641056
LAMP1	W2	PGE2	well_1	F002	283	X/Y distribution	4.087327	3.993939	0.688046
LAMP1	W2	PGE2	well_1	F003	198	X/Y distribution	4.190312	4.128512	0.810146
LAMP1	W2	PGE2	well_1	F004	174	X/Y distribution	3.993105	3.974927	0.576134
LAMP1	W2	PGE2	well_1	F005	235	X/Y distribution	4.177125	4.15816	0.569365
LAMP1	W2	PGE2	well_1	F006	381	X/Y distribution	4.138373	4.067139	0.663673
LAMP1	W2	PGE2	well_2	F001	162	X/Y distribution	4.014778	4.001781	0.659492
LAMP1	W2	PGE2	well_2	F002	82	X/Y distribution	3.895292	3.864231	0.511919
LAMP1	W2	PGE2	well_2	F003	370	X/Y distribution	3.880225	3.891129	0.656787
LAMP1	W2	PGE2	well_2	F004	242	X/Y distribution	4.203249	4.164846	0.896915
LAMP1	W2	PGE2	well_2	F005	233	X/Y distribution	3.841309	3.760571	0.69654
LAMP1	W2	PGE2	well_2	F006	128	X/Y distribution	3.870023	3.823743	0.669444
LAMP1	W2	PGE2	well_3	F001	300	X/Y distribution	3.994766	4.031568	0.689562
LAMP1	W2	PGE2	well_3	F002	274	X/Y distribution	3.944055	3.970626	0.557474
LAMP1	W2	PGE2	well_3	F003	179	X/Y distribution	3.691872	3.654154	0.702427
LAMP1	W2	PGE2	well_3	F004	280	X/Y distribution	3.690133	3.62882	0.717586
LAMP1	W2	PGE2	well_3	F005	261	X/Y distribution	4.069106	4.04552	0.704937
LAMP1	W2	PGE2	well_3	F006	213	X/Y distribution	3.939896	3.92666	0.623399
LAMP1	W2	PGE2	well_4	F001	202	X/Y distribution	4.157288	4.164184	0.590165
LAMP1	W2	PGE2	well_4	F002	226	X/Y distribution	3.923536	3.863066	0.740308
LAMP1	W2	PGE2	well_4	F003	300	X/Y distribution	3.812757	3.769141	0.606334
LAMP1	W2	PGE2	well_4	F004	291	X/Y distribution	3.801242	3.788431	0.661205
LAMP1	W2	PGE2	well_4	F005	202	X/Y distribution	3.772786	3.768302	0.665118
LAMP1	W2	PGE2	well_4	F006	252	X/Y distribution	4.076121	4.038003	0.683506
LAMP1	W2	PGE2	well_5	F001	319	X/Y distribution	4.02565	3.968564	0.649556
LAMP1	W2	PGE2	well_5	F002	218	X/Y distribution	4.094228	4.025604	0.684437
LAMP1	W2	PGE2	well_5	F003	381	X/Y distribution	4.14973	4.009414	0.981162
LAMP1	W2	PGE2	well_5	F004	272	X/Y distribution	3.675934	3.591924	0.690008
LAMP1	W2	PGE2	well_5	F005	376	X/Y distribution	3.948167	3.923358	0.735475
LAMP1	W2	PGE2	well_5	F006	97	X/Y distribution	3.593485	3.547572	0.714752
LAMP1	W3	HPI4	well_1	F001	82	X/Y distribution	6.211729	6.057323	1.167044
LAMP1	W3	HPI4	well_1	F002	197	X/Y distribution	5.940987	5.819009	1.22826
LAMP1	W3	HPI4	well_1	F003	139	X/Y distribution	5.715188	5.753087	1.308287
LAMP1	W3	HPI4	well_1	F004	81	X/Y distribution	5.949694	5.780387	1.16437

LAMP1	W3	HPI4	well_1	F005	105	X/Y distribution	7.145179	6.741906	1.417966
LAMP1	W3	HPI4	well_1	F006	104	X/Y distribution	5.85608	5.843301	1.133125
LAMP1	W3	HPI4	well_2	F001	94	X/Y distribution	6.480442	6.431148	1.266328
LAMP1	W3	HPI4	well_2	F002	76	X/Y distribution	7.067358	6.959264	1.552661
LAMP1	W3	HPI4	well_2	F003	28	X/Y distribution	9.253605	9.221712	1.286378
LAMP1	W3	HPI4	well_2	F004	48	X/Y distribution	7.070355	6.918501	1.576473
LAMP1	W3	HPI4	well_2	F005	91	X/Y distribution	5.873336	5.872356	1.333138
LAMP1	W3	HPI4	well_2	F006	92	X/Y distribution	5.883284	5.712976	1.386427
LAMP1	W3	HPI4	well_3	F001	139	X/Y distribution	6.733108	6.644573	1.344087
LAMP1	W3	HPI4	well_3	F002	162	X/Y distribution	6.278002	6.116721	1.3427
LAMP1	W3	HPI4	well_3	F003	44	X/Y distribution	5.42171	4.954823	1.36541
LAMP1	W3	HPI4	well_3	F004	113	X/Y distribution	5.044838	4.855355	1.45226
LAMP1	W3	HPI4	well_3	F005	162	X/Y distribution	5.569594	5.376156	1.595981
LAMP1	W3	HPI4	well_3	F006	146	X/Y distribution	6.393091	6.315086	1.346782
LAMP1	W3	HPI4	well_4	F001	88	X/Y distribution	6.42336	6.256773	1.405865
LAMP1	W3	HPI4	well_4	F002	64	X/Y distribution	5.994005	6.081727	1.500287
LAMP1	W3	HPI4	well_4	F003	49	X/Y distribution	8.445178	8.339236	1.43091
LAMP1	W3	HPI4	well_4	F004	43	X/Y distribution	5.491611	5.132856	1.39071
LAMP1	W3	HPI4	well_4	F005	109	X/Y distribution	5.923138	5.698051	1.325781
LAMP1	W3	HPI4	well_4	F006	274	X/Y distribution	5.718969	5.857451	1.43451
LAMP1	W3	HPI4	well_5	F001	87	X/Y distribution	5.640556	5.532985	1.68347
LAMP1	W3	HPI4	well_5	F002	21	X/Y distribution	6.869762	6.692956	1.994882
LAMP1	W3	HPI4	well_5	F003	36	X/Y distribution	7.701124	7.628348	1.226125
LAMP1	W3	HPI4	well_5	F004	43	X/Y distribution	5.384606	5.547046	1.207519
LAMP1	W3	HPI4	well_5	F005	91	X/Y distribution	7.220845	7.157799	1.533078
LAMP1	W3	HPI4	well_5	F006	48	X/Y distribution	7.332776	7.555717	1.497512
LAMP1	W3	PGE2	well_1	F001	76	X/Y distribution	3.844847	3.829727	0.669245
LAMP1	W3	PGE2	well_1	F002	229	X/Y distribution	4.051571	4.045993	0.630548
LAMP1	W3	PGE2	well_1	F003	276	X/Y distribution	3.807884	3.799123	0.634622
LAMP1	W3	PGE2	well_1	F004	432	X/Y distribution	3.771632	3.780575	0.650621
LAMP1	W3	PGE2	well_1	F005	374	X/Y distribution	3.859952	3.803656	0.587375
LAMP1	W3	PGE2	well_1	F006	142	X/Y distribution	3.724655	3.653719	0.648413
LAMP1	W3	PGE2	well_2	F001	111	X/Y distribution	3.557217	3.423347	0.850476
LAMP1	W3	PGE2	well_2	F002	212	X/Y distribution	4.008203	4.001429	0.670803
LAMP1	W3	PGE2	well_2	F003	153	X/Y distribution	4.004013	4.086786	0.680644
LAMP1	W3	PGE2	well_2	F004	72	X/Y distribution	3.543699	3.543383	0.764111
LAMP1	W3	PGE2	well_2	F005	215	X/Y distribution	3.963442	3.971423	0.813923
LAMP1	W3	PGE2	well_2	F006	327	X/Y distribution	3.985945	4.005881	0.515949
LAMP1	W3	PGE2	well_3	F001	55	X/Y distribution	3.855613	3.676658	0.958818
LAMP1	W3	PGE2	well_3	F002	257	X/Y distribution	3.902304	3.876774	0.726564
LAMP1	W3	PGE2	well_3	F003	360	X/Y distribution	3.654751	3.633493	0.671123
LAMP1	W3	PGE2	well_3	F004	181	X/Y distribution	3.687637	3.597183	0.663769
LAMP1	W3	PGE2	well_3	F005	120	X/Y distribution	4.020391	4.022517	0.649603
LAMP1	W3	PGE2	well_3	F006	159	X/Y distribution	3.931802	3.92087	0.563659
LAMP1	W3	PGE2	well_4	F001	76	X/Y distribution	3.606099	3.66192	0.824331
LAMP1	W3	PGE2	well_4	F002	215	X/Y distribution	3.914659	3.893403	0.784992
LAMP1	W3	PGE2	well_4	F003	103	X/Y distribution	3.412259	3.380338	0.601061
LAMP1	W3	PGE2	well_4	F004	64	X/Y distribution	3.8379	3.768885	0.733954
LAMP1	W3	PGE2	well_4	F005	150	X/Y distribution	3.770822	3.829883	0.778635

LAMP1	W3	PGE2	well_4	F006	213	X/Y distribution	4.038803	3.985034	0.698496
LAMP1	W3	PGE2	well_5	F001	76	X/Y distribution	3.852791	3.813027	0.778181
LAMP1	W3	PGE2	well_5	F002	59	X/Y distribution	3.811268	3.655258	0.767251
LAMP1	W3	PGE2	well_5	F003	176	X/Y distribution	3.96174	3.908351	0.594698
LAMP1	W3	PGE2	well_5	F004	143	X/Y distribution	3.801528	3.803244	0.594742
LAMP1	W3	PGE2	well_5	F005	362	X/Y distribution	3.854042	3.784947	0.64884
LAMP1	W3	PGE2	well_5	F006	80	X/Y distribution	3.963302	3.903998	0.568029
LAMP1	W4	HPI4	well_1	F001	109	X/Y distribution	5.669261	5.481364	1.512124
LAMP1	W4	HPI4	well_1	F002	115	X/Y distribution	6.293952	6.326151	1.425064
LAMP1	W4	HPI4	well_1	F003	105	X/Y distribution	7.032115	6.844368	1.424514
LAMP1	W4	HPI4	well_1	F004	93	X/Y distribution	6.591155	6.534225	1.429631
LAMP1	W4	HPI4	well_1	F005	162	X/Y distribution	5.683704	5.608597	1.426198
LAMP1	W4	HPI4	well_1	F006	120	X/Y distribution	5.698006	5.778364	1.603867
LAMP1	W4	HPI4	well_2	F001	90	X/Y distribution	5.918076	5.673902	1.506484
LAMP1	W4	HPI4	well_2	F002	201	X/Y distribution	6.211023	6.289718	1.334464
LAMP1	W4	HPI4	well_2	F003	76	X/Y distribution	8.764248	8.390614	1.679466
LAMP1	W4	HPI4	well_2	F004	176	X/Y distribution	6.695964	6.613792	1.747814
LAMP1	W4	HPI4	well_2	F005	141	X/Y distribution	6.543075	6.514169	1.360294
LAMP1	W4	HPI4	well_2	F006	164	X/Y distribution	6.724972	6.727452	1.259712
LAMP1	W4	HPI4	well_3	F001	259	X/Y distribution	6.067577	6.031826	1.274968
LAMP1	W4	HPI4	well_3	F002	261	X/Y distribution	6.21119	6.126272	1.394989
LAMP1	W4	HPI4	well_3	F003	174	X/Y distribution	7.101781	6.941344	1.848538
LAMP1	W4	HPI4	well_3	F004	221	X/Y distribution	6.539564	6.382772	1.377033
LAMP1	W4	HPI4	well_3	F005	206	X/Y distribution	6.854422	6.700029	1.61308
LAMP1	W4	HPI4	well_3	F006	253	X/Y distribution	5.75185	5.82826	1.343565
LAMP1	W4	HPI4	well_4	F001	218	X/Y distribution	6.603455	6.518892	1.22306
LAMP1	W4	HPI4	well_4	F002	244	X/Y distribution	6.723004	6.855304	1.287485
LAMP1	W4	HPI4	well_4	F003	75	X/Y distribution	8.451685	8.437844	2.100446
LAMP1	W4	HPI4	well_4	F004	215	X/Y distribution	6.586471	6.667065	1.323462
LAMP1	W4	HPI4	well_4	F005	222	X/Y distribution	6.687017	6.572263	1.249213
LAMP1	W4	HPI4	well_4	F006	145	X/Y distribution	7.034583	6.816962	1.610922
LAMP1	W4	HPI4	well_5	F001	169	X/Y distribution	7.492595	7.502028	1.450558
LAMP1	W4	HPI4	well_5	F002	162	X/Y distribution	7.301541	7.279362	1.14189
LAMP1	W4	HPI4	well_5	F003	83	X/Y distribution	7.630306	7.714411	1.493694
LAMP1	W4	HPI4	well_5	F004	116	X/Y distribution	8.286698	8.413573	1.560682
LAMP1	W4	HPI4	well_5	F005	146	X/Y distribution	6.65672	6.460074	1.39442
LAMP1	W4	HPI4	well_5	F006	265	X/Y distribution	6.389323	6.325309	1.240434
LAMP1	W4	PGE2	well_1	F001	60	X/Y distribution	4.414931	4.293316	0.901378
LAMP1	W4	PGE2	well_1	F002	75	X/Y distribution	4.448439	4.432336	0.785169
LAMP1	W4	PGE2	well_1	F003	207	X/Y distribution	3.949377	3.969033	0.673129
LAMP1	W4	PGE2	well_1	F004	314	X/Y distribution	3.985909	3.932264	0.581853
LAMP1	W4	PGE2	well_1	F005	128	X/Y distribution	3.824419	3.773773	0.640277
LAMP1	W4	PGE2	well_1	F006	224	X/Y distribution	3.973239	3.900864	0.767088
LAMP1	W4	PGE2	well_2	F001	44	X/Y distribution	3.978723	3.881499	0.547644
LAMP1	W4	PGE2	well_2	F002	172	X/Y distribution	4.52615	4.475726	0.821075
LAMP1	W4	PGE2	well_2	F003	294	X/Y distribution	3.599179	3.591017	0.979876
LAMP1	W4	PGE2	well_2	F004	203	X/Y distribution	3.807012	3.777518	0.710488
LAMP1	W4	PGE2	well_2	F005	290	X/Y distribution	3.573194	3.519954	0.550446
LAMP1	W4	PGE2	well_2	F006	142	X/Y distribution	3.741875	3.750298	0.502384

LAMP1	W4	PGE2	well_3	F001	39	X/Y distribution	3.784413	3.670825	0.829707
LAMP1	W4	PGE2	well_3	F002	130	X/Y distribution	3.942556	3.921013	0.637777
LAMP1	W4	PGE2	well_3	F003	268	X/Y distribution	4.297748	4.281127	0.67111
LAMP1	W4	PGE2	well_3	F004	57	X/Y distribution	3.578587	3.501836	0.68273
LAMP1	W4	PGE2	well_3	F005	258	X/Y distribution	3.700287	3.650062	0.591237
LAMP1	W4	PGE2	well_3	F006	174	X/Y distribution	4.075379	3.960112	0.673258
LAMP1	W4	PGE2	well_4	F001	93	X/Y distribution	3.707607	3.707959	0.761278
LAMP1	W4	PGE2	well_4	F002	315	X/Y distribution	3.488146	3.413776	0.917258
LAMP1	W4	PGE2	well_4	F003	166	X/Y distribution	3.769862	3.721113	0.772398
LAMP1	W4	PGE2	well_4	F004	255	X/Y distribution	3.924268	3.88222	0.714585
LAMP1	W4	PGE2	well_4	F005	286	X/Y distribution	3.73034	3.685654	0.63109
LAMP1	W4	PGE2	well_4	F006	131	X/Y distribution	3.922316	3.824128	0.644496
LAMP1	W4	PGE2	well_5	F001	123	X/Y distribution	3.646442	3.638639	0.815805
LAMP1	W4	PGE2	well_5	F002	235	X/Y distribution	3.755716	3.738974	0.653427
LAMP1	W4	PGE2	well_5	F003	382	X/Y distribution	3.813959	3.735269	0.546627
LAMP1	W4	PGE2	well_5	F004	239	X/Y distribution	3.704453	3.739142	0.579901
LAMP1	W4	PGE2	well_5	F005	174	X/Y distribution	3.883764	3.846294	0.68171
LAMP1	W4	PGE2	well_5	F006	142	X/Y distribution	3.836207	3.822969	0.517458
LAMP1	W1	HPI4	well_1	F001	272	Z distribution	0.212248	0.159189	0.377416
LAMP1	W1	HPI4	well_1	F002	347	Z distribution	0.295217	0.268881	0.403058
LAMP1	W1	HPI4	well_1	F003	207	Z distribution	0.190015	0.16623	0.47422
LAMP1	W1	HPI4	well_1	F004	346	Z distribution	0.114818	0.104224	0.385695
LAMP1	W1	HPI4	well_1	F005	399	Z distribution	0.386863	0.383753	0.434176
LAMP1	W1	HPI4	well_1	F006	250	Z distribution	0.3921	0.310572	0.487355
LAMP1	W1	HPI4	well_2	F001	103	Z distribution	0.153274	0.119433	0.371433
LAMP1	W1	HPI4	well_2	F002	241	Z distribution	0.186769	0.1296	0.402374
LAMP1	W1	HPI4	well_2	F003	207	Z distribution	-0.01615	-0.02492	0.361768
LAMP1	W1	HPI4	well_2	F004	323	Z distribution	0.11862	0.086598	0.321822
LAMP1	W1	HPI4	well_2	F005	297	Z distribution	0.1215	0.123031	0.383359
LAMP1	W1	HPI4	well_2	F006	185	Z distribution	0.456293	0.437246	0.501004
LAMP1	W1	HPI4	well_3	F001	68	Z distribution	0.084375	0.053884	0.36599
LAMP1	W1	HPI4	well_3	F002	257	Z distribution	0.396523	0.368685	0.492019
LAMP1	W1	HPI4	well_3	F003	70	Z distribution	0.005245	0.037814	0.267404
LAMP1	W1	HPI4	well_3	F004	279	Z distribution	0.15371	0.152384	0.33513
LAMP1	W1	HPI4	well_3	F005	329	Z distribution	0.305677	0.256808	0.508124
LAMP1	W1	HPI4	well_3	F006	214	Z distribution	0.191477	0.166056	0.400531
LAMP1	W1	HPI4	well_4	F001	282	Z distribution	0.273806	0.242589	0.393379
LAMP1	W1	HPI4	well_4	F002	295	Z distribution	0.259231	0.217156	0.458897
LAMP1	W1	HPI4	well_4	F003	265	Z distribution	0.025595	0.054189	0.336988
LAMP1	W1	HPI4	well_4	F004	81	Z distribution	0.02881	0.016522	0.341311
LAMP1	W1	HPI4	well_4	F005	205	Z distribution	0.117456	0.014792	0.376097
LAMP1	W1	HPI4	well_4	F006	167	Z distribution	0.282852	0.18661	0.494634
LAMP1	W1	HPI4	well_5	F001	329	Z distribution	0.378164	0.333816	0.499022
LAMP1	W1	HPI4	well_5	F002	209	Z distribution	0.321979	0.273416	0.406329
LAMP1	W1	HPI4	well_5	F003	84	Z distribution	0.040344	0.01221	0.313254
LAMP1	W1	HPI4	well_5	F004	161	Z distribution	0.15977	0.105333	0.448632
LAMP1	W1	HPI4	well_5	F005	302	Z distribution	0.243957	0.203874	0.395454
LAMP1	W1	HPI4	well_5	F006	233	Z distribution	0.276154	0.22315	0.449095
LAMP1	W1	PGE2	well_1	F001	314	Z distribution	0.234336	0.190732	0.385231

LAMP1	W1	PGE2	well_1	F002	389	Z distribution	0.195349	0.136346	0.445272
LAMP1	W1	PGE2	well_1	F003	304	Z distribution	0.126685	0.102	0.372536
LAMP1	W1	PGE2	well_1	F004	312	Z distribution	0.140674	0.102593	0.352956
LAMP1	W1	PGE2	well_1	F005	438	Z distribution	0.37146	0.385432	0.494857
LAMP1	W1	PGE2	well_1	F006	411	Z distribution	0.446185	0.424458	0.460824
LAMP1	W1	PGE2	well_2	F001	334	Z distribution	0.490334	0.430675	0.452171
LAMP1	W1	PGE2	well_2	F002	387	Z distribution	0.305725	0.281995	0.443879
LAMP1	W1	PGE2	well_2	F003	391	Z distribution	0.205932	0.210606	0.422298
LAMP1	W1	PGE2	well_2	F004	290	Z distribution	0.044326	0.066264	0.356799
LAMP1	W1	PGE2	well_2	F005	315	Z distribution	0.326029	0.280953	0.396342
LAMP1	W1	PGE2	well_2	F006	370	Z distribution	0.313387	0.267547	0.453654
LAMP1	W1	PGE2	well_3	F001	336	Z distribution	0.226229	0.199231	0.370743
LAMP1	W1	PGE2	well_3	F002	290	Z distribution	0.088997	0.053086	0.404632
LAMP1	W1	PGE2	well_3	F003	289	Z distribution	0.171844	0.173474	0.371154
LAMP1	W1	PGE2	well_3	F004	236	Z distribution	0.068819	0.085355	0.345519
LAMP1	W1	PGE2	well_3	F005	444	Z distribution	0.453037	0.43387	0.502367
LAMP1	W1	PGE2	well_3	F006	420	Z distribution	0.302163	0.289269	0.465812
LAMP1	W1	PGE2	well_4	F001	320	Z distribution	0.182751	0.164903	0.398461
LAMP1	W1	PGE2	well_4	F002	371	Z distribution	0.336247	0.284063	0.467493
LAMP1	W1	PGE2	well_4	F003	179	Z distribution	0.085779	0.062662	0.340743
LAMP1	W1	PGE2	well_4	F004	328	Z distribution	0.224629	0.146244	0.448589
LAMP1	W1	PGE2	well_4	F005	375	Z distribution	0.171245	0.15005	0.414645
LAMP1	W1	PGE2	well_4	F006	348	Z distribution	0.276136	0.26043	0.415118
LAMP1	W1	PGE2	well_5	F001	299	Z distribution	0.240965	0.185753	0.399288
LAMP1	W1	PGE2	well_5	F002	351	Z distribution	0.388683	0.374095	0.483412
LAMP1	W1	PGE2	well_5	F003	131	Z distribution	-0.05341	-0.07356	0.308292
LAMP1	W1	PGE2	well_5	F004	302	Z distribution	0.19377	0.164884	0.384068
LAMP1	W1	PGE2	well_5	F005	339	Z distribution	0.215829	0.21178	0.37171
LAMP1	W1	PGE2	well_5	F006	256	Z distribution	0.24578	0.205102	0.475231
LAMP1	W2	HPI4	well_1	F001	299	Z distribution	-0.16754	-0.13455	0.391962
LAMP1	W2	HPI4	well_1	F002	376	Z distribution	-0.33091	-0.34463	0.399522
LAMP1	W2	HPI4	well_1	F003	372	Z distribution	-0.16122	-0.10749	0.539689
LAMP1	W2	HPI4	well_1	F004	229	Z distribution	-0.28622	-0.18666	0.420787
LAMP1	W2	HPI4	well_1	F005	216	Z distribution	-0.23787	-0.24436	0.325571
LAMP1	W2	HPI4	well_1	F006	415	Z distribution	-0.44716	-0.49952	0.553539
LAMP1	W2	HPI4	well_2	F001	357	Z distribution	-0.1662	-0.1864	0.391466
LAMP1	W2	HPI4	well_2	F002	291	Z distribution	-0.39747	-0.33736	0.465378
LAMP1	W2	HPI4	well_2	F003	344	Z distribution	-0.39601	-0.3708	0.474951
LAMP1	W2	HPI4	well_2	F004	332	Z distribution	-0.24129	-0.24041	0.511437
LAMP1	W2	HPI4	well_2	F005	325	Z distribution	-0.31332	-0.27049	0.494575
LAMP1	W2	HPI4	well_2	F006	409	Z distribution	-0.3842	-0.34732	0.493087
LAMP1	W2	HPI4	well_3	F001	346	Z distribution	-0.21486	-0.17243	0.43456
LAMP1	W2	HPI4	well_3	F002	406	Z distribution	-0.21409	-0.22829	0.485894
LAMP1	W2	HPI4	well_3	F003	153	Z distribution	-0.15306	-0.11755	0.268772
LAMP1	W2	HPI4	well_3	F004	273	Z distribution	-0.29184	-0.20905	0.438606
LAMP1	W2	HPI4	well_3	F005	210	Z distribution	-0.32487	-0.26416	0.417896
LAMP1	W2	HPI4	well_3	F006	400	Z distribution	-0.30942	-0.29267	0.558221
LAMP1	W2	HPI4	well_4	F001	475	Z distribution	-0.1367	-0.1058	0.37063
LAMP1	W2	HPI4	well_4	F002	445	Z distribution	-0.28403	-0.27468	0.536588

LAMP1	W2	HPI4	well_4	F003	274	Z distribution	-0.20417	-0.156	0.420949
LAMP1	W2	HPI4	well_4	F004	388	Z distribution	-0.29456	-0.24599	0.487388
LAMP1	W2	HPI4	well_4	F005	275	Z distribution	-0.35123	-0.30268	0.503138
LAMP1	W2	HPI4	well_4	F006	414	Z distribution	-0.27384	-0.25538	0.516612
LAMP1	W2	HPI4	well_5	F001	352	Z distribution	-0.18078	-0.12399	0.419773
LAMP1	W2	HPI4	well_5	F002	361	Z distribution	-0.41349	-0.34205	0.546936
LAMP1	W2	HPI4	well_5	F003	268	Z distribution	-0.36055	-0.30919	0.462316
LAMP1	W2	HPI4	well_5	F004	267	Z distribution	-0.38234	-0.30177	0.491678
LAMP1	W2	HPI4	well_5	F005	229	Z distribution	-0.20735	-0.21962	0.466374
LAMP1	W2	HPI4	well_5	F006	293	Z distribution	-0.46404	-0.48725	0.584612
LAMP1	W2	PGE2	well_1	F001	249	Z distribution	0.07767	0.020794	0.625805
LAMP1	W2	PGE2	well_1	F002	283	Z distribution	0.364474	0.364485	0.572854
LAMP1	W2	PGE2	well_1	F003	198	Z distribution	0.309809	0.311444	0.554092
LAMP1	W2	PGE2	well_1	F004	174	Z distribution	0.355375	0.374535	0.46365
LAMP1	W2	PGE2	well_1	F005	235	Z distribution	0.185347	0.21711	0.469905
LAMP1	W2	PGE2	well_1	F006	381	Z distribution	0.415914	0.378564	0.612674
LAMP1	W2	PGE2	well_2	F001	162	Z distribution	0.275821	0.268577	0.469275
LAMP1	W2	PGE2	well_2	F002	82	Z distribution	0.185873	0.213178	0.50079
LAMP1	W2	PGE2	well_2	F003	370	Z distribution	0.3115	0.320318	0.57408
LAMP1	W2	PGE2	well_2	F004	242	Z distribution	0.314827	0.282506	0.557484
LAMP1	W2	PGE2	well_2	F005	233	Z distribution	0.400932	0.389675	0.512044
LAMP1	W2	PGE2	well_2	F006	128	Z distribution	0.229869	0.279817	0.620036
LAMP1	W2	PGE2	well_3	F001	300	Z distribution	0.467699	0.471783	0.639737
LAMP1	W2	PGE2	well_3	F002	274	Z distribution	0.071271	0.029408	0.568226
LAMP1	W2	PGE2	well_3	F003	179	Z distribution	0.486062	0.549998	0.603736
LAMP1	W2	PGE2	well_3	F004	280	Z distribution	0.509928	0.518995	0.73598
LAMP1	W2	PGE2	well_3	F005	261	Z distribution	0.243691	0.208836	0.683402
LAMP1	W2	PGE2	well_3	F006	213	Z distribution	0.4604	0.485846	0.59025
LAMP1	W2	PGE2	well_4	F001	202	Z distribution	0.211245	0.210463	0.591302
LAMP1	W2	PGE2	well_4	F002	226	Z distribution	0.343681	0.333815	0.665212
LAMP1	W2	PGE2	well_4	F003	300	Z distribution	0.313226	0.288981	0.539024
LAMP1	W2	PGE2	well_4	F004	291	Z distribution	0.647299	0.65123	0.688348
LAMP1	W2	PGE2	well_4	F005	202	Z distribution	0.644088	0.627071	0.658557
LAMP1	W2	PGE2	well_4	F006	252	Z distribution	0.385516	0.338098	0.616371
LAMP1	W2	PGE2	well_5	F001	319	Z distribution	0.121761	0.132491	0.584753
LAMP1	W2	PGE2	well_5	F002	218	Z distribution	0.456543	0.463113	0.578005
LAMP1	W2	PGE2	well_5	F003	381	Z distribution	0.387353	0.359726	0.61387
LAMP1	W2	PGE2	well_5	F004	272	Z distribution	0.483666	0.496916	0.618692
LAMP1	W2	PGE2	well_5	F005	376	Z distribution	0.400932	0.408268	0.59472
LAMP1	W2	PGE2	well_5	F006	97	Z distribution	0.260998	0.202449	0.615358
LAMP1	W3	HPI4	well_1	F001	82	Z distribution	-0.86954	-0.87087	0.483201
LAMP1	W3	HPI4	well_1	F002	197	Z distribution	-0.32026	-0.199	0.597613
LAMP1	W3	HPI4	well_1	F003	139	Z distribution	-0.6198	-0.55648	0.53349
LAMP1	W3	HPI4	well_1	F004	81	Z distribution	-0.74406	-0.63352	0.512275
LAMP1	W3	HPI4	well_1	F005	105	Z distribution	-0.53614	-0.47888	0.409588
LAMP1	W3	HPI4	well_1	F006	104	Z distribution	-0.596	-0.53417	0.470867
LAMP1	W3	HPI4	well_2	F001	94	Z distribution	-0.28504	-0.28555	0.347925
LAMP1	W3	HPI4	well_2	F002	76	Z distribution	-0.64308	-0.56552	0.540138
LAMP1	W3	HPI4	well_2	F003	28	Z distribution	-0.41126	-0.39605	0.259144

LAMP1	W3	HPI4	well_2	F004	48	Z distribution	-0.35117	-0.41058	0.259555
LAMP1	W3	HPI4	well_2	F005	91	Z distribution	-0.89555	-0.88518	0.47525
LAMP1	W3	HPI4	well_2	F006	92	Z distribution	-0.66319	-0.64475	0.42201
LAMP1	W3	HPI4	well_3	F001	139	Z distribution	-0.66706	-0.67044	0.511613
LAMP1	W3	HPI4	well_3	F002	162	Z distribution	-0.72178	-0.75936	0.550442
LAMP1	W3	HPI4	well_3	F003	44	Z distribution	-0.74473	-0.77386	0.417102
LAMP1	W3	HPI4	well_3	F004	113	Z distribution	-0.51224	-0.43167	0.471523
LAMP1	W3	HPI4	well_3	F005	162	Z distribution	-0.43431	-0.37987	0.439207
LAMP1	W3	HPI4	well_3	F006	146	Z distribution	-0.48214	-0.56186	0.50852
LAMP1	W3	HPI4	well_4	F001	88	Z distribution	-0.53308	-0.51313	0.447906
LAMP1	W3	HPI4	well_4	F002	64	Z distribution	-0.51676	-0.49022	0.388848
LAMP1	W3	HPI4	well_4	F003	49	Z distribution	-0.43041	-0.39771	0.296323
LAMP1	W3	HPI4	well_4	F004	43	Z distribution	-0.41144	-0.25089	0.442778
LAMP1	W3	HPI4	well_4	F005	109	Z distribution	-0.52774	-0.5234	0.489117
LAMP1	W3	HPI4	well_4	F006	274	Z distribution	-0.503	-0.54806	0.635579
LAMP1	W3	HPI4	well_5	F001	87	Z distribution	-0.48215	-0.45969	0.420502
LAMP1	W3	HPI4	well_5	F002	21	Z distribution	-0.23703	-0.17577	0.234723
LAMP1	W3	HPI4	well_5	F003	36	Z distribution	-0.57674	-0.58561	0.3861
LAMP1	W3	HPI4	well_5	F004	43	Z distribution	-0.56694	-0.45236	0.431176
LAMP1	W3	HPI4	well_5	F005	91	Z distribution	-0.27259	-0.26544	0.26812
LAMP1	W3	HPI4	well_5	F006	48	Z distribution	-0.56196	-0.58499	0.451878
LAMP1	W3	PGE2	well_1	F001	76	Z distribution	-0.4272	-0.49765	0.548528
LAMP1	W3	PGE2	well_1	F002	229	Z distribution	0.006861	0.028748	0.664487
LAMP1	W3	PGE2	well_1	F003	276	Z distribution	0.193562	0.184103	0.66021
LAMP1	W3	PGE2	well_1	F004	432	Z distribution	0.203856	0.156264	0.716159
LAMP1	W3	PGE2	well_1	F005	374	Z distribution	0.061525	0.049381	0.580918
LAMP1	W3	PGE2	well_1	F006	142	Z distribution	0.037274	0.000463	0.546312
LAMP1	W3	PGE2	well_2	F001	111	Z distribution	0.120862	0.124524	0.623359
LAMP1	W3	PGE2	well_2	F002	212	Z distribution	-0.04929	-0.04762	0.634732
LAMP1	W3	PGE2	well_2	F003	153	Z distribution	-0.07624	-0.02595	0.577639
LAMP1	W3	PGE2	well_2	F004	72	Z distribution	0.095553	0.094655	0.549146
LAMP1	W3	PGE2	well_2	F005	215	Z distribution	0.01001	-0.01948	0.589691
LAMP1	W3	PGE2	well_2	F006	327	Z distribution	-0.02165	0.003653	0.534129
LAMP1	W3	PGE2	well_3	F001	55	Z distribution	0.076427	-0.0219	0.690818
LAMP1	W3	PGE2	well_3	F002	257	Z distribution	0.131843	0.079484	0.722898
LAMP1	W3	PGE2	well_3	F003	360	Z distribution	0.35476	0.366141	0.746145
LAMP1	W3	PGE2	well_3	F004	181	Z distribution	0.072154	0.057569	0.725899
LAMP1	W3	PGE2	well_3	F005	120	Z distribution	-0.13784	-0.11276	0.468919
LAMP1	W3	PGE2	well_3	F006	159	Z distribution	0.022772	0.089108	0.623255
LAMP1	W3	PGE2	well_4	F001	76	Z distribution	0.20288	0.128857	0.596681
LAMP1	W3	PGE2	well_4	F002	215	Z distribution	0.322182	0.318928	0.70542
LAMP1	W3	PGE2	well_4	F003	103	Z distribution	0.329706	0.306863	0.758243
LAMP1	W3	PGE2	well_4	F004	64	Z distribution	-0.00389	-0.01649	0.527577
LAMP1	W3	PGE2	well_4	F005	150	Z distribution	0.167523	0.156562	0.566434
LAMP1	W3	PGE2	well_4	F006	213	Z distribution	0.209909	0.203613	0.658526
LAMP1	W3	PGE2	well_5	F001	76	Z distribution	-0.02521	-0.03045	0.618614
LAMP1	W3	PGE2	well_5	F002	59	Z distribution	0.128132	0.217793	0.607402
LAMP1	W3	PGE2	well_5	F003	176	Z distribution	0.176096	0.149292	0.49452
LAMP1	W3	PGE2	well_5	F004	143	Z distribution	0.175562	0.113813	0.490486

LAMP1	W3	PGE2	well_5	F005	362	Z distribution	0.127867	0.133413	0.607667
LAMP1	W3	PGE2	well_5	F006	80	Z distribution	-0.1956	-0.17788	0.552289
LAMP1	W4	HPI4	well_1	F001	109	Z distribution	-1.15551	-1.17472	0.484232
LAMP1	W4	HPI4	well_1	F002	115	Z distribution	-0.8762	-0.92033	0.549039
LAMP1	W4	HPI4	well_1	F003	105	Z distribution	-0.53746	-0.50573	0.454858
LAMP1	W4	HPI4	well_1	F004	93	Z distribution	-0.81882	-0.7942	0.51096
LAMP1	W4	HPI4	well_1	F005	162	Z distribution	-0.50857	-0.46233	0.476439
LAMP1	W4	HPI4	well_1	F006	120	Z distribution	-0.76598	-0.76565	0.448245
LAMP1	W4	HPI4	well_2	F001	90	Z distribution	-1.06534	-1.07878	0.448541
LAMP1	W4	HPI4	well_2	F002	201	Z distribution	-0.88735	-0.9985	0.564429
LAMP1	W4	HPI4	well_2	F003	76	Z distribution	-0.12641	-0.04675	0.337897
LAMP1	W4	HPI4	well_2	F004	176	Z distribution	-0.52524	-0.4911	0.523838
LAMP1	W4	HPI4	well_2	F005	141	Z distribution	-0.3998	-0.35111	0.392409
LAMP1	W4	HPI4	well_2	F006	164	Z distribution	-0.76545	-0.82464	0.491197
LAMP1	W4	HPI4	well_3	F001	259	Z distribution	-0.19105	-0.17129	0.440937
LAMP1	W4	HPI4	well_3	F002	261	Z distribution	-0.41008	-0.40863	0.509015
LAMP1	W4	HPI4	well_3	F003	174	Z distribution	-0.46146	-0.49556	0.486069
LAMP1	W4	HPI4	well_3	F004	221	Z distribution	-0.5031	-0.52509	0.530887
LAMP1	W4	HPI4	well_3	F005	206	Z distribution	-0.36176	-0.32447	0.46721
LAMP1	W4	HPI4	well_3	F006	253	Z distribution	-0.50476	-0.57075	0.598437
LAMP1	W4	HPI4	well_4	F001	218	Z distribution	-0.39674	-0.41387	0.473032
LAMP1	W4	HPI4	well_4	F002	244	Z distribution	-0.51148	-0.53027	0.574027
LAMP1	W4	HPI4	well_4	F003	75	Z distribution	-0.21163	-0.17087	0.393785
LAMP1	W4	HPI4	well_4	F004	215	Z distribution	-0.44555	-0.44578	0.47063
LAMP1	W4	HPI4	well_4	F005	222	Z distribution	-0.48758	-0.50643	0.542566
LAMP1	W4	HPI4	well_4	F006	145	Z distribution	-0.8032	-0.84804	0.50306
LAMP1	W4	HPI4	well_5	F001	169	Z distribution	-0.18944	-0.18085	0.383468
LAMP1	W4	HPI4	well_5	F002	162	Z distribution	-0.38613	-0.37673	0.410577
LAMP1	W4	HPI4	well_5	F003	83	Z distribution	-0.12442	-0.05078	0.398821
LAMP1	W4	HPI4	well_5	F004	116	Z distribution	0.003186	0.012883	0.23748
LAMP1	W4	HPI4	well_5	F005	146	Z distribution	-0.72276	-0.78021	0.556528
LAMP1	W4	HPI4	well_5	F006	265	Z distribution	-0.14878	-0.08125	0.492848
LAMP1	W4	PGE2	well_1	F001	60	Z distribution	-0.15371	-0.25188	0.476457
LAMP1	W4	PGE2	well_1	F002	75	Z distribution	-0.43796	-0.46316	0.605685
LAMP1	W4	PGE2	well_1	F003	207	Z distribution	0.014453	0.002228	0.582589
LAMP1	W4	PGE2	well_1	F004	314	Z distribution	0.058289	0.062744	0.449977
LAMP1	W4	PGE2	well_1	F005	128	Z distribution	0.114473	0.100479	0.574031
LAMP1	W4	PGE2	well_1	F006	224	Z distribution	0.102129	0.10778	0.507172
LAMP1	W4	PGE2	well_2	F001	44	Z distribution	-0.20785	-0.22246	0.492684
LAMP1	W4	PGE2	well_2	F002	172	Z distribution	-0.61955	-0.52461	0.60349
LAMP1	W4	PGE2	well_2	F003	294	Z distribution	0.570201	0.485298	0.65868
LAMP1	W4	PGE2	well_2	F004	203	Z distribution	0.062765	0.050935	0.506336
LAMP1	W4	PGE2	well_2	F005	290	Z distribution	0.119566	0.120485	0.501308
LAMP1	W4	PGE2	well_2	F006	142	Z distribution	-0.045	-0.03776	0.454959
LAMP1	W4	PGE2	well_3	F001	39	Z distribution	-0.23346	-0.0741	0.43715
LAMP1	W4	PGE2	well_3	F002	130	Z distribution	0.022383	-0.00295	0.471403
LAMP1	W4	PGE2	well_3	F003	268	Z distribution	-0.19151	-0.16025	0.40864
LAMP1	W4	PGE2	well_3	F004	57	Z distribution	0.198087	0.290149	0.599721
LAMP1	W4	PGE2	well_3	F005	258	Z distribution	-0.04155	-0.04665	0.493131

LAMP1	W4	PGE2	well_3	F006	174	Z distribution	-0.16913	-0.15452	0.477382
LAMP1	W4	PGE2	well_4	F001	93	Z distribution	0.01284	0.035278	0.565218
LAMP1	W4	PGE2	well_4	F002	315	Z distribution	0.453507	0.383123	0.715427
LAMP1	W4	PGE2	well_4	F003	166	Z distribution	0.209206	0.131541	0.581117
LAMP1	W4	PGE2	well_4	F004	255	Z distribution	0.009597	-0.00728	0.497544
LAMP1	W4	PGE2	well_4	F005	286	Z distribution	0.104777	0.072495	0.446486
LAMP1	W4	PGE2	well_4	F006	131	Z distribution	-0.00533	0.023362	0.471711
LAMP1	W4	PGE2	well_5	F001	123	Z distribution	0.202472	0.159688	0.565282
LAMP1	W4	PGE2	well_5	F002	235	Z distribution	0.265654	0.280826	0.44615
LAMP1	W4	PGE2	well_5	F003	382	Z distribution	0.176408	0.171438	0.445546
LAMP1	W4	PGE2	well_5	F004	239	Z distribution	-0.01046	-0.0542	0.565473
LAMP1	W4	PGE2	well_5	F005	174	Z distribution	0.131463	0.114722	0.446869
LAMP1	W4	PGE2	well_5	F006	142	Z distribution	-0.08305	-0.07042	0.40773
LC3B	W1	HPI4	well_1	F001	154	Avg. Volume	0.512357	0.473356	0.237731
LC3B	W1	HPI4	well_1	F002	100	Avg. Volume	0.506703	0.480203	0.239506
LC3B	W1	HPI4	well_1	F003	64	Avg. Volume	0.510237	0.487049	0.196251
LC3B	W1	HPI4	well_1	F004	73	Avg. Volume	0.380642	0.356246	0.203976
LC3B	W1	HPI4	well_1	F005	67	Avg. Volume	0.474231	0.457708	0.21639
LC3B	W1	HPI4	well_1	F006	72	Avg. Volume	0.468992	0.445972	0.190605
LC3B	W1	HPI4	well_2	F001	120	Avg. Volume	0.452316	0.441278	0.228039
LC3B	W1	HPI4	well_2	F002	136	Avg. Volume	0.445741	0.400704	0.21778
LC3B	W1	HPI4	well_2	F003	97	Avg. Volume	0.461542	0.453796	0.205394
LC3B	W1	HPI4	well_2	F004	75	Avg. Volume	0.338259	0.305139	0.148919
LC3B	W1	HPI4	well_2	F005	91	Avg. Volume	0.493837	0.445972	0.255017
LC3B	W1	HPI4	well_2	F006	45	Avg. Volume	0.442411	0.413111	0.156013
LC3B	W1	HPI4	well_3	F001	79	Avg. Volume	0.513239	0.489563	0.246759
LC3B	W1	HPI4	well_3	F002	91	Avg. Volume	0.482628	0.457708	0.229329
LC3B	W1	HPI4	well_3	F003	97	Avg. Volume	0.486158	0.487328	0.212148
LC3B	W1	HPI4	well_3	F004	103	Avg. Volume	0.467934	0.434236	0.25029
LC3B	W1	HPI4	well_3	F005	84	Avg. Volume	0.397104	0.387292	0.207488
LC3B	W1	HPI4	well_3	F006	59	Avg. Volume	0.551033	0.524213	0.23024
LC3B	W1	HPI4	well_4	F001	118	Avg. Volume	0.519621	0.491111	0.237565
LC3B	W1	HPI4	well_4	F002	106	Avg. Volume	0.536735	0.498785	0.272914
LC3B	W1	HPI4	well_4	F003	80	Avg. Volume	0.531372	0.487049	0.280853
LC3B	W1	HPI4	well_4	F004	65	Avg. Volume	0.507287	0.467488	0.226915
LC3B	W1	HPI4	well_4	F005	96	Avg. Volume	0.43439	0.416632	0.229072
LC3B	W1	HPI4	well_4	F006	116	Avg. Volume	0.483214	0.440756	0.23166
LC3B	W1	HPI4	well_5	F001	108	Avg. Volume	0.509058	0.482159	0.231705
LC3B	W1	HPI4	well_5	F002	163	Avg. Volume	0.492588	0.490961	0.220822
LC3B	W1	HPI4	well_5	F003	70	Avg. Volume	0.558764	0.504653	0.27216
LC3B	W1	HPI4	well_5	F004	88	Avg. Volume	0.481173	0.445972	0.226478
LC3B	W1	HPI4	well_5	F005	104	Avg. Volume	0.524802	0.52656	0.219045
LC3B	W1	HPI4	well_5	F006	92	Avg. Volume	0.494493	0.436248	0.250015
LC3B	W1	PGE2	well_1	F001	154	Avg. Volume	0.540457	0.515085	0.238232
LC3B	W1	PGE2	well_1	F002	167	Avg. Volume	0.493671	0.477269	0.219715
LC3B	W1	PGE2	well_1	F003	124	Avg. Volume	0.528182	0.480447	0.222913
LC3B	W1	PGE2	well_1	F004	97	Avg. Volume	0.460469	0.426412	0.235851
LC3B	W1	PGE2	well_1	F005	120	Avg. Volume	0.475897	0.434236	0.18866
LC3B	W1	PGE2	well_1	F006	166	Avg. Volume	0.532103	0.508565	0.247943

LC3B	W1	PGE2	well_2	F001	95	Avg. Volume	0.500918	0.443364	0.229878
LC3B	W1	PGE2	well_2	F002	96	Avg. Volume	0.440646	0.415794	0.193196
LC3B	W1	PGE2	well_2	F003	90	Avg. Volume	0.456992	0.434236	0.190044
LC3B	W1	PGE2	well_2	F004	59	Avg. Volume	0.38935	0.366167	0.201242
LC3B	W1	PGE2	well_2	F005	94	Avg. Volume	0.524293	0.470911	0.292271
LC3B	W1	PGE2	well_2	F006	66	Avg. Volume	0.452115	0.43597	0.197188
LC3B	W1	PGE2	well_3	F001	101	Avg. Volume	0.501572	0.504653	0.226379
LC3B	W1	PGE2	well_3	F002	116	Avg. Volume	0.482327	0.475313	0.207707
LC3B	W1	PGE2	well_3	F003	72	Avg. Volume	0.38855	0.390421	0.167647
LC3B	W1	PGE2	well_3	F004	54	Avg. Volume	0.353632	0.34552	0.135788
LC3B	W1	PGE2	well_3	F005	50	Avg. Volume	0.477644	0.35376	0.282943
LC3B	W1	PGE2	well_3	F006	69	Avg. Volume	0.419679	0.399069	0.19153
LC3B	W1	PGE2	well_4	F001	73	Avg. Volume	0.505305	0.445972	0.228791
LC3B	W1	PGE2	well_4	F002	73	Avg. Volume	0.4559	0.408417	0.222942
LC3B	W1	PGE2	well_4	F003	92	Avg. Volume	0.408795	0.374252	0.200685
LC3B	W1	PGE2	well_4	F004	55	Avg. Volume	0.394561	0.389843	0.178784
LC3B	W1	PGE2	well_4	F005	23	Avg. Volume	0.513029	0.569201	0.25105
LC3B	W1	PGE2	well_4	F006	55	Avg. Volume	0.467128	0.441278	0.244562
LC3B	W1	PGE2	well_5	F001	82	Avg. Volume	0.482342	0.453796	0.197864
LC3B	W1	PGE2	well_5	F002	89	Avg. Volume	0.507735	0.492917	0.226121
LC3B	W1	PGE2	well_5	F003	30	Avg. Volume	0.432911	0.433584	0.174683
LC3B	W1	PGE2	well_5	F004	35	Avg. Volume	0.373367	0.3437	0.201954
LC3B	W1	PGE2	well_5	F005	60	Avg. Volume	0.453214	0.399028	0.227901
LC3B	W1	PGE2	well_5	F006	78	Avg. Volume	0.545491	0.539861	0.228115
LC3B	W2	HPI4	well_1	F001	29	Avg. Volume	0.36621	0.33487	0.177052
LC3B	W2	HPI4	well_1	F002	58	Avg. Volume	0.362058	0.354536	0.117038
LC3B	W2	HPI4	well_1	F003	46	Avg. Volume	0.312962	0.316169	0.134252
LC3B	W2	HPI4	well_1	F004	21	Avg. Volume	0.371004	0.347389	0.18094
LC3B	W2	HPI4	well_1	F005	79	Avg. Volume	0.334252	0.327144	0.12719
LC3B	W2	HPI4	well_1	F006	44	Avg. Volume	0.403035	0.421838	0.135511
LC3B	W2	HPI4	well_2	F001	25	Avg. Volume	0.342199	0.273843	0.151111
LC3B	W2	HPI4	well_2	F002	59	Avg. Volume	0.324668	0.312963	0.130036
LC3B	W2	HPI4	well_2	F003	39	Avg. Volume	0.378964	0.382076	0.157265
LC3B	W2	HPI4	well_2	F004	39	Avg. Volume	0.39621	0.336435	0.176456
LC3B	W2	HPI4	well_2	F005	48	Avg. Volume	0.400312	0.393998	0.164182
LC3B	W2	HPI4	well_2	F006	28	Avg. Volume	0.216326	0.187778	0.139945
LC3B	W2	HPI4	well_3	F001	102	Avg. Volume	0.347026	0.318036	0.161348
LC3B	W2	HPI4	well_3	F002	92	Avg. Volume	0.3269	0.29767	0.146629
LC3B	W2	HPI4	well_3	F003	66	Avg. Volume	0.360744	0.325873	0.188542
LC3B	W2	HPI4	well_3	F004	22	Avg. Volume	0.400805	0.425015	0.101143
LC3B	W2	HPI4	well_3	F005	9	Avg. Volume	0.462404	0.487701	0.185507
LC3B	W2	HPI4	well_3	F006	52	Avg. Volume	0.325116	0.293403	0.153942
LC3B	W2	HPI4	well_4	F001	77	Avg. Volume	0.316386	0.281667	0.144343
LC3B	W2	HPI4	well_4	F002	71	Avg. Volume	0.361149	0.356973	0.12533
LC3B	W2	HPI4	well_4	F003	18	Avg. Volume	0.589854	0.539077	0.271022
LC3B	W2	HPI4	well_4	F004	13	Avg. Volume	0.344304	0.281667	0.168211
LC3B	W2	HPI4	well_4	F005	5	Avg. Volume	0.321018	0.272064	0.138426
LC3B	W2	HPI4	well_4	F006	33	Avg. Volume	0.361385	0.381793	0.19466
LC3B	W2	HPI4	well_5	F001	22	Avg. Volume	0.356056	0.352083	0.130458

LC3B	W2	HPI4	well_5	F002	44	Avg. Volume	0.34487	0.330437	0.147559
LC3B	W2	HPI4	well_5	F003	45	Avg. Volume	0.305874	0.310169	0.119351
LC3B	W2	HPI4	well_5	F004	27	Avg. Volume	0.323661	0.315198	0.148446
LC3B	W2	HPI4	well_5	F005	27	Avg. Volume	0.349209	0.30875	0.164788
LC3B	W2	HPI4	well_5	F006	6	Avg. Volume	0.50113	0.496253	0.064822
LC3B	W2	PGE2	well_1	F001	40	Avg. Volume	0.301637	0.250521	0.167891
LC3B	W2	PGE2	well_1	F002	39	Avg. Volume	0.334736	0.312963	0.141352
LC3B	W2	PGE2	well_1	F003	46	Avg. Volume	0.32125	0.320076	0.123991
LC3B	W2	PGE2	well_1	F004	106	Avg. Volume	0.335972	0.306628	0.112351
LC3B	W2	PGE2	well_1	F005	200	Avg. Volume	0.328595	0.31918	0.111991
LC3B	W2	PGE2	well_1	F006	134	Avg. Volume	0.350206	0.319966	0.133163
LC3B	W2	PGE2	well_2	F001	40	Avg. Volume	0.320439	0.304161	0.138567
LC3B	W2	PGE2	well_2	F002	65	Avg. Volume	0.421016	0.363819	0.19152
LC3B	W2	PGE2	well_2	F003	158	Avg. Volume	0.35281	0.317106	0.132505
LC3B	W2	PGE2	well_2	F004	149	Avg. Volume	0.380347	0.356387	0.131986
LC3B	W2	PGE2	well_2	F005	121	Avg. Volume	0.369346	0.339211	0.142852
LC3B	W2	PGE2	well_2	F006	165	Avg. Volume	0.302304	0.295079	0.113126
LC3B	W2	PGE2	well_3	F001	71	Avg. Volume	0.296607	0.272278	0.129957
LC3B	W2	PGE2	well_3	F002	147	Avg. Volume	0.314326	0.288373	0.130693
LC3B	W2	PGE2	well_3	F003	236	Avg. Volume	0.337969	0.332523	0.134709
LC3B	W2	PGE2	well_3	F004	54	Avg. Volume	0.389366	0.37639	0.120884
LC3B	W2	PGE2	well_3	F005	58	Avg. Volume	0.298192	0.285481	0.114191
LC3B	W2	PGE2	well_3	F006	35	Avg. Volume	0.281457	0.275799	0.130566
LC3B	W2	PGE2	well_4	F001	114	Avg. Volume	0.273602	0.247762	0.104556
LC3B	W2	PGE2	well_4	F002	59	Avg. Volume	0.304368	0.285934	0.124017
LC3B	W2	PGE2	well_4	F003	76	Avg. Volume	0.380653	0.371644	0.161737
LC3B	W2	PGE2	well_4	F004	235	Avg. Volume	0.335359	0.315085	0.131716
LC3B	W2	PGE2	well_4	F005	47	Avg. Volume	0.311887	0.321569	0.097289
LC3B	W2	PGE2	well_4	F006	60	Avg. Volume	0.273852	0.249392	0.118189
LC3B	W2	PGE2	well_5	F001	122	Avg. Volume	0.30654	0.283458	0.119801
LC3B	W2	PGE2	well_5	F002	139	Avg. Volume	0.320183	0.296337	0.124103
LC3B	W2	PGE2	well_5	F003	104	Avg. Volume	0.382117	0.354154	0.158636
LC3B	W2	PGE2	well_5	F004	155	Avg. Volume	0.424887	0.413965	0.129466
LC3B	W2	PGE2	well_5	F005	85	Avg. Volume	0.363418	0.340703	0.120217
LC3B	W2	PGE2	well_5	F006	67	Avg. Volume	0.333256	0.330288	0.098514
LC3B	W3	HPI4	well_1	F001	20	Avg. Volume	0.427572	0.416632	0.179513
LC3B	W3	HPI4	well_1	F002	5	Avg. Volume	0.411353	0.415165	0.0224
LC3B	W3	HPI4	well_1	F003	8	Avg. Volume	0.36694	0.335066	0.066309
LC3B	W3	HPI4	well_1	F004	14	Avg. Volume	0.356598	0.359907	0.156266
LC3B	W3	HPI4	well_1	F005	14	Avg. Volume	0.3693	0.392626	0.238947
LC3B	W3	HPI4	well_1	F006	28	Avg. Volume	0.450254	0.454448	0.115788
LC3B	W3	HPI4	well_2	F001	1	Avg. Volume	0.406852	0.406852	NA
LC3B	W3	HPI4	well_2	F002	6	Avg. Volume	0.337255	0.373004	0.078595
LC3B	W3	HPI4	well_2	F003	23	Avg. Volume	0.346089	0.370861	0.133618
LC3B	W3	HPI4	well_2	F004	7	Avg. Volume	0.426986	0.338	0.312615
LC3B	W3	HPI4	well_2	F005	9	Avg. Volume	0.189619	0.178389	0.08283
LC3B	W3	HPI4	well_2	F006	10	Avg. Volume	0.486654	0.452707	0.202287
LC3B	W3	HPI4	well_3	F001	7	Avg. Volume	0.587161	0.539861	0.247876
LC3B	W3	HPI4	well_3	F003	1	Avg. Volume	0.62447	0.62447	NA

LC3B	W3	HPI4	well_3	F004	1	Avg. Volume	0.226898	0.226898	NA
LC3B	W3	HPI4	well_3	F005	4	Avg. Volume	0.452679	0.452679	0.018968
LC3B	W3	HPI4	well_3	F006	19	Avg. Volume	0.396481	0.377791	0.14712
LC3B	W3	HPI4	well_4	F002	5	Avg. Volume	0.442619	0.457708	0.031813
LC3B	W3	HPI4	well_4	F005	4	Avg. Volume	0.240199	0.240199	0.002213
LC3B	W3	HPI4	well_4	F006	10	Avg. Volume	0.30861	0.332523	0.123174
LC3B	W3	HPI4	well_5	F001	4	Avg. Volume	0.262302	0.262302	0.052282
LC3B	W3	HPI4	well_5	F002	1	Avg. Volume	1.046367	1.046367	NA
LC3B	W3	HPI4	well_5	F005	3	Avg. Volume	0.481181	0.481181	NA
LC3B	W3	PGE2	well_1	F001	159	Avg. Volume	0.431277	0.416662	0.154752
LC3B	W3	PGE2	well_1	F002	191	Avg. Volume	0.402334	0.39316	0.155854
LC3B	W3	PGE2	well_1	F003	213	Avg. Volume	0.398934	0.377512	0.166158
LC3B	W3	PGE2	well_1	F004	159	Avg. Volume	0.406312	0.390492	0.152715
LC3B	W3	PGE2	well_1	F005	181	Avg. Volume	0.302086	0.302498	0.117168
LC3B	W3	PGE2	well_1	F006	264	Avg. Volume	0.395894	0.381364	0.122218
LC3B	W3	PGE2	well_2	F001	52	Avg. Volume	0.426536	0.430175	0.143515
LC3B	W3	PGE2	well_2	F002	95	Avg. Volume	0.480904	0.457708	0.172528
LC3B	W3	PGE2	well_2	F003	143	Avg. Volume	0.448738	0.4225	0.170234
LC3B	W3	PGE2	well_2	F004	207	Avg. Volume	0.374201	0.378144	0.114504
LC3B	W3	PGE2	well_2	F005	166	Avg. Volume	0.476096	0.461397	0.149424
LC3B	W3	PGE2	well_2	F006	123	Avg. Volume	0.420551	0.411495	0.126361
LC3B	W3	PGE2	well_3	F001	114	Avg. Volume	0.406524	0.394899	0.132795
LC3B	W3	PGE2	well_3	F002	171	Avg. Volume	0.419986	0.399028	0.166489
LC3B	W3	PGE2	well_3	F003	194	Avg. Volume	0.464238	0.46788	0.141276
LC3B	W3	PGE2	well_3	F004	172	Avg. Volume	0.377394	0.362132	0.124443
LC3B	W3	PGE2	well_3	F005	274	Avg. Volume	0.474422	0.468378	0.144954
LC3B	W3	PGE2	well_3	F006	128	Avg. Volume	0.479382	0.450528	0.136064
LC3B	W3	PGE2	well_4	F001	61	Avg. Volume	0.454632	0.469444	0.151045
LC3B	W3	PGE2	well_4	F002	132	Avg. Volume	0.449315	0.427922	0.156807
LC3B	W3	PGE2	well_4	F003	117	Avg. Volume	0.454712	0.438148	0.146537
LC3B	W3	PGE2	well_4	F004	255	Avg. Volume	0.37644	0.368849	0.12448
LC3B	W3	PGE2	well_4	F005	198	Avg. Volume	0.47514	0.470147	0.129445
LC3B	W3	PGE2	well_4	F006	223	Avg. Volume	0.440072	0.4225	0.14499
LC3B	W3	PGE2	well_5	F001	223	Avg. Volume	0.431011	0.426768	0.133329
LC3B	W3	PGE2	well_5	F002	264	Avg. Volume	0.428888	0.427607	0.126707
LC3B	W3	PGE2	well_5	F003	90	Avg. Volume	0.434547	0.408578	0.130885
LC3B	W3	PGE2	well_5	F004	189	Avg. Volume	0.398087	0.370274	0.197527
LC3B	W3	PGE2	well_5	F005	202	Avg. Volume	0.489345	0.475313	0.173629
LC3B	W3	PGE2	well_5	F006	188	Avg. Volume	0.416813	0.401962	0.135321
LC3B	W4	HPI4	well_1	F001	1	Avg. Volume	0.524514	0.524514	NA
LC3B	W4	HPI4	well_1	F002	1	Avg. Volume	0.464624	0.464624	NA
LC3B	W4	HPI4	well_1	F003	1	Avg. Volume	0.246458	0.246458	NA
LC3B	W4	HPI4	well_1	F005	4	Avg. Volume	0.519393	0.519393	0.102449
LC3B	W4	HPI4	well_2	F005	1	Avg. Volume	0.575196	0.575196	NA
LC3B	W4	HPI4	well_3	F001	1	Avg. Volume	0.34656	0.34656	NA
LC3B	W4	HPI4	well_3	F005	1	Avg. Volume	0.564714	0.564714	NA
LC3B	W4	HPI4	well_3	F006	7	Avg. Volume	0.500658	0.586806	0.209689
LC3B	W4	HPI4	well_4	F001	3	Avg. Volume	0.348171	0.348171	NA
LC3B	W4	HPI4	well_4	F003	1	Avg. Volume	0.105625	0.105625	NA

LC3B	W4	HPI4	well_4	F004	3	Avg. Volume	0.400263	0.400263	NA
LC3B	W4	PGE2	well_1	F001	105	Avg. Volume	0.382903	0.352083	0.127655
LC3B	W4	PGE2	well_1	F002	100	Avg. Volume	0.365322	0.356356	0.129248
LC3B	W4	PGE2	well_1	F003	224	Avg. Volume	0.36425	0.363261	0.118964
LC3B	W4	PGE2	well_1	F004	187	Avg. Volume	0.393027	0.362352	0.175366
LC3B	W4	PGE2	well_1	F005	222	Avg. Volume	0.386201	0.371644	0.164198
LC3B	W4	PGE2	well_1	F006	218	Avg. Volume	0.416819	0.390226	0.162448
LC3B	W4	PGE2	well_2	F001	253	Avg. Volume	0.490523	0.486515	0.167321
LC3B	W4	PGE2	well_2	F002	142	Avg. Volume	0.464225	0.440104	0.161379
LC3B	W4	PGE2	well_2	F003	209	Avg. Volume	0.431807	0.417806	0.153106
LC3B	W4	PGE2	well_2	F004	216	Avg. Volume	0.438257	0.41897	0.165502
LC3B	W4	PGE2	well_2	F005	248	Avg. Volume	0.470235	0.46651	0.165311
LC3B	W4	PGE2	well_2	F006	230	Avg. Volume	0.481591	0.474823	0.160973
LC3B	W4	PGE2	well_3	F001	162	Avg. Volume	0.511124	0.483975	0.198137
LC3B	W4	PGE2	well_3	F002	150	Avg. Volume	0.462313	0.43228	0.168713
LC3B	W4	PGE2	well_3	F003	216	Avg. Volume	0.426493	0.386365	0.182194
LC3B	W4	PGE2	well_3	F004	175	Avg. Volume	0.395588	0.372202	0.14854
LC3B	W4	PGE2	well_3	F005	260	Avg. Volume	0.359045	0.354561	0.14929
LC3B	W4	PGE2	well_3	F006	237	Avg. Volume	0.450008	0.431302	0.167854
LC3B	W4	PGE2	well_4	F001	73	Avg. Volume	0.434603	0.438148	0.167992
LC3B	W4	PGE2	well_4	F002	74	Avg. Volume	0.4548	0.446875	0.156665
LC3B	W4	PGE2	well_4	F003	109	Avg. Volume	0.423461	0.414676	0.1564
LC3B	W4	PGE2	well_4	F004	164	Avg. Volume	0.446596	0.431186	0.156313
LC3B	W4	PGE2	well_4	F005	221	Avg. Volume	0.419763	0.410764	0.154
LC3B	W4	PGE2	well_4	F006	255	Avg. Volume	0.530514	0.513036	0.205259
LC3B	W4	PGE2	well_5	F001	128	Avg. Volume	0.466437	0.460838	0.17133
LC3B	W4	PGE2	well_5	F002	34	Avg. Volume	0.49632	0.481914	0.171519
LC3B	W4	PGE2	well_5	F003	25	Avg. Volume	0.470878	0.487049	0.144918
LC3B	W4	PGE2	well_5	F004	81	Avg. Volume	0.489345	0.469444	0.190722
LC3B	W4	PGE2	well_5	F005	53	Avg. Volume	0.437729	0.416632	0.195731
LC3B	W4	PGE2	well_5	F006	75	Avg. Volume	0.333284	0.326851	0.153477
LC3B	W1	HPI4	well_1	F001	154	Count/cell	4.777027	5	2.457312
LC3B	W1	HPI4	well_1	F002	100	Count/cell	4.705263	4	2.551069
LC3B	W1	HPI4	well_1	F003	64	Count/cell	5.75	5.5	2.937222
LC3B	W1	HPI4	well_1	F004	73	Count/cell	8.144928	5	8.31242
LC3B	W1	HPI4	well_1	F005	67	Count/cell	4.746032	4	3.287161
LC3B	W1	HPI4	well_1	F006	72	Count/cell	4.367647	4	2.596809
LC3B	W1	HPI4	well_2	F001	120	Count/cell	5.491228	5	3.286458
LC3B	W1	HPI4	well_2	F002	136	Count/cell	3.709924	3	2.210077
LC3B	W1	HPI4	well_2	F003	97	Count/cell	4.473118	4	2.729036
LC3B	W1	HPI4	well_2	F004	75	Count/cell	9.5	8	5.855218
LC3B	W1	HPI4	well_2	F005	91	Count/cell	5.767442	5	3.39437
LC3B	W1	HPI4	well_2	F006	45	Count/cell	7.119048	6	5.636131
LC3B	W1	HPI4	well_3	F001	79	Count/cell	4.986667	5	3.117056
LC3B	W1	HPI4	well_3	F002	91	Count/cell	5.337349	5	2.715255
LC3B	W1	HPI4	well_3	F003	97	Count/cell	6.795699	6	3.996083
LC3B	W1	HPI4	well_3	F004	103	Count/cell	4.683673	4.5	2.726718
LC3B	W1	HPI4	well_3	F005	84	Count/cell	4.5375	4	3.419763
LC3B	W1	HPI4	well_3	F006	59	Count/cell	5.62963	5.5	2.810333

LC3B	W1	HPI4	well_4	F001	118	Count/cell	5.877193	5	3.145837
LC3B	W1	HPI4	well_4	F002	106	Count/cell	4.45	4	2.371442
LC3B	W1	HPI4	well_4	F003	80	Count/cell	4	3.5	2.558409
LC3B	W1	HPI4	well_4	F004	65	Count/cell	5	5	3.032086
LC3B	W1	HPI4	well_4	F005	96	Count/cell	4.395604	4	2.502795
LC3B	W1	HPI4	well_4	F006	116	Count/cell	4.196429	3.5	2.84063
LC3B	W1	HPI4	well_5	F001	108	Count/cell	4.519608	4	2.627842
LC3B	W1	HPI4	well_5	F002	163	Count/cell	4.954839	5	2.903965
LC3B	W1	HPI4	well_5	F003	70	Count/cell	5.296875	5	2.646829
LC3B	W1	HPI4	well_5	F004	88	Count/cell	4.674699	5	2.566712
LC3B	W1	HPI4	well_5	F005	104	Count/cell	4.505051	4	2.387782
LC3B	W1	HPI4	well_5	F006	92	Count/cell	4.238636	4	2.067784
LC3B	W1	PGE2	well_1	F001	154	Count/cell	4.537415	4	2.37646
LC3B	W1	PGE2	well_1	F002	167	Count/cell	5.658228	5	3.415536
LC3B	W1	PGE2	well_1	F003	124	Count/cell	4.882353	4	2.604353
LC3B	W1	PGE2	well_1	F004	97	Count/cell	5.043011	5	2.661783
LC3B	W1	PGE2	well_1	F005	120	Count/cell	5.201754	5	3.040086
LC3B	W1	PGE2	well_1	F006	166	Count/cell	5.128834	5	3.079497
LC3B	W1	PGE2	well_2	F001	95	Count/cell	5.456522	5	2.910432
LC3B	W1	PGE2	well_2	F002	96	Count/cell	5.467391	5	3.629704
LC3B	W1	PGE2	well_2	F003	90	Count/cell	5.259259	4	2.978161
LC3B	W1	PGE2	well_2	F004	59	Count/cell	4.087719	4	2.115257
LC3B	W1	PGE2	well_2	F005	94	Count/cell	4.384615	4	2.48895
LC3B	W1	PGE2	well_2	F006	66	Count/cell	5.442623	5	2.759979
LC3B	W1	PGE2	well_3	F001	101	Count/cell	4.760417	4	2.882779
LC3B	W1	PGE2	well_3	F002	116	Count/cell	5.5	5	3.019053
LC3B	W1	PGE2	well_3	F003	72	Count/cell	5.676471	4	4.300091
LC3B	W1	PGE2	well_3	F004	54	Count/cell	7.442308	5	6.625599
LC3B	W1	PGE2	well_3	F005	50	Count/cell	3.638298	3	2.297688
LC3B	W1	PGE2	well_3	F006	69	Count/cell	5.181818	4	3.842712
LC3B	W1	PGE2	well_4	F001	73	Count/cell	4.507246	4	2.374021
LC3B	W1	PGE2	well_4	F002	73	Count/cell	5.434783	5	3.362764
LC3B	W1	PGE2	well_4	F003	92	Count/cell	5.16092	4	3.238122
LC3B	W1	PGE2	well_4	F004	55	Count/cell	6.788462	6.5	4.598962
LC3B	W1	PGE2	well_4	F005	23	Count/cell	6.947368	6	4.707838
LC3B	W1	PGE2	well_4	F006	55	Count/cell	4.211538	3.5	2.96606
LC3B	W1	PGE2	well_5	F001	82	Count/cell	4.766234	4	2.694517
LC3B	W1	PGE2	well_5	F002	89	Count/cell	5.188235	5	3.04527
LC3B	W1	PGE2	well_5	F003	30	Count/cell	8.107143	7.5	5.756409
LC3B	W1	PGE2	well_5	F004	35	Count/cell	6.411765	4.5	4.961168
LC3B	W1	PGE2	well_5	F005	60	Count/cell	6.490909	6	3.862798
LC3B	W1	PGE2	well_5	F006	78	Count/cell	4.256757	3	2.44983
LC3B	W2	HPI4	well_1	F001	29	Count/cell	14.07407	15	9.67521
LC3B	W2	HPI4	well_1	F002	58	Count/cell	26.23077	26.5	15.95658
LC3B	W2	HPI4	well_1	F003	46	Count/cell	13.09302	9	11.85189
LC3B	W2	HPI4	well_1	F004	21	Count/cell	14.36842	13	11.00611
LC3B	W2	HPI4	well_1	F005	79	Count/cell	19.61644	16	12.95358
LC3B	W2	HPI4	well_1	F006	44	Count/cell	21.07895	21	12.23504
LC3B	W2	HPI4	well_2	F001	25	Count/cell	9.782609	5	9.638731

LC3B	W2	HPI4	well_2	F002	59	Count/cell	10.64286	9	7.418475
LC3B	W2	HPI4	well_2	F003	39	Count/cell	11.67568	12	8.432918
LC3B	W2	HPI4	well_2	F004	39	Count/cell	11.83333	10	6.452021
LC3B	W2	HPI4	well_2	F005	48	Count/cell	10.60465	11	6.532481
LC3B	W2	HPI4	well_2	F006	28	Count/cell	2.038462	1	1.37057
LC3B	W2	HPI4	well_3	F001	102	Count/cell	8.73913	8	5.251624
LC3B	W2	HPI4	well_3	F002	92	Count/cell	12.18605	10	8.226945
LC3B	W2	HPI4	well_3	F003	66	Count/cell	7.080645	6	5.032023
LC3B	W2	HPI4	well_3	F004	22	Count/cell	10.33333	10	5.830952
LC3B	W2	HPI4	well_3	F005	9	Count/cell	5.75	6	3.058945
LC3B	W2	HPI4	well_3	F006	52	Count/cell	7.608696	5	6.244921
LC3B	W2	HPI4	well_4	F001	77	Count/cell	12.2027	11.5	8.034139
LC3B	W2	HPI4	well_4	F002	71	Count/cell	14.14706	13.5	9.940517
LC3B	W2	HPI4	well_4	F003	18	Count/cell	14.47059	13	10.94942
LC3B	W2	HPI4	well_4	F004	13	Count/cell	7.916667	5	8.349832
LC3B	W2	HPI4	well_4	F005	5	Count/cell	11.75	11	10.17759
LC3B	W2	HPI4	well_4	F006	33	Count/cell	7.03125	6	5.336934
LC3B	W2	HPI4	well_5	F001	22	Count/cell	17.11111	13	13.67217
LC3B	W2	HPI4	well_5	F002	44	Count/cell	11	10	8.709191
LC3B	W2	HPI4	well_5	F003	45	Count/cell	12.46341	10	8.832037
LC3B	W2	HPI4	well_5	F004	27	Count/cell	12.6087	13	8.133763
LC3B	W2	HPI4	well_5	F005	27	Count/cell	8.8	6	7.382412
LC3B	W2	HPI4	well_5	F006	6	Count/cell	27.75	30	10.65755
LC3B	W2	PGE2	well_1	F001	40	Count/cell	7	6	5.337273
LC3B	W2	PGE2	well_1	F002	39	Count/cell	4.891892	4	3.762267
LC3B	W2	PGE2	well_1	F003	46	Count/cell	4.930233	3	4.40992
LC3B	W2	PGE2	well_1	F004	106	Count/cell	14.31915	13	7.714269
LC3B	W2	PGE2	well_1	F005	200	Count/cell	17.10383	14	10.73175
LC3B	W2	PGE2	well_1	F006	134	Count/cell	16.12397	15	8.688661
LC3B	W2	PGE2	well_2	F001	40	Count/cell	14.2973	11	10.74157
LC3B	W2	PGE2	well_2	F002	65	Count/cell	6	5	4.489847
LC3B	W2	PGE2	well_2	F003	158	Count/cell	15.08451	12	10.40083
LC3B	W2	PGE2	well_2	F004	149	Count/cell	13.08148	12	7.741198
LC3B	W2	PGE2	well_2	F005	121	Count/cell	16.73394	15	10.03479
LC3B	W2	PGE2	well_2	F006	165	Count/cell	15.40397	14	9.448936
LC3B	W2	PGE2	well_3	F001	71	Count/cell	8.359375	7	5.769012
LC3B	W2	PGE2	well_3	F002	147	Count/cell	10.29104	8	7.199441
LC3B	W2	PGE2	well_3	F003	236	Count/cell	10.77169	9	7.08422
LC3B	W2	PGE2	well_3	F004	54	Count/cell	16.9375	13.5	10.50715
LC3B	W2	PGE2	well_3	F005	58	Count/cell	9.480769	8.5	5.899453
LC3B	W2	PGE2	well_3	F006	35	Count/cell	10.54545	8	9.421023
LC3B	W2	PGE2	well_4	F001	114	Count/cell	9.647619	9	5.540031
LC3B	W2	PGE2	well_4	F002	59	Count/cell	8.410714	8	6.239979
LC3B	W2	PGE2	well_4	F003	76	Count/cell	11.61765	11	6.852452
LC3B	W2	PGE2	well_4	F004	235	Count/cell	18.87558	15	12.67793
LC3B	W2	PGE2	well_4	F005	47	Count/cell	12.7907	9	10.40732
LC3B	W2	PGE2	well_4	F006	60	Count/cell	5.070175	4	3.299673
LC3B	W2	PGE2	well_5	F001	122	Count/cell	14.7193	13.5	9.370929
LC3B	W2	PGE2	well_5	F002	139	Count/cell	14.97619	12	10.12124

LC3B	W2	PGE2	well_5	F003	104	Count/cell	11.80412	11	6.839894
LC3B	W2	PGE2	well_5	F004	155	Count/cell	14.38028	12	7.341452
LC3B	W2	PGE2	well_5	F005	85	Count/cell	14.91026	12	9.876097
LC3B	W2	PGE2	well_5	F006	67	Count/cell	16.98333	17	8.524127
LC3B	W3	HPI4	well_1	F001	20	Count/cell	12.5	8.5	10.58439
LC3B	W3	HPI4	well_1	F002	5	Count/cell	13.33333	16	4.618802
LC3B	W3	HPI4	well_1	F003	8	Count/cell	8	7.5	4.604346
LC3B	W3	HPI4	well_1	F004	14	Count/cell	5.076923	4	4.212314
LC3B	W3	HPI4	well_1	F005	14	Count/cell	8.416667	8	5.517877
LC3B	W3	HPI4	well_1	F006	28	Count/cell	11.69231	9.5	7.98383
LC3B	W3	HPI4	well_2	F001	1	Count/cell	18	18	NA
LC3B	W3	HPI4	well_2	F002	6	Count/cell	10.25	10.5	3.304038
LC3B	W3	HPI4	well_2	F003	23	Count/cell	10.15789	6	8.661942
LC3B	W3	HPI4	well_2	F004	7	Count/cell	11.2	12	4.086563
LC3B	W3	HPI4	well_2	F005	9	Count/cell	9.428571	6	6.729466
LC3B	W3	HPI4	well_2	F006	10	Count/cell	21.5	21	7.982123
LC3B	W3	HPI4	well_3	F001	7	Count/cell	8.833333	5.5	7.440878
LC3B	W3	HPI4	well_3	F003	1	Count/cell	43	43	NA
LC3B	W3	HPI4	well_3	F004	1	Count/cell	9	9	NA
LC3B	W3	HPI4	well_3	F005	4	Count/cell	31.5	31.5	4.949747
LC3B	W3	HPI4	well_3	F006	19	Count/cell	11.72222	7.5	10.02236
LC3B	W3	HPI4	well_4	F002	5	Count/cell	8.333333	9	2.081666
LC3B	W3	HPI4	well_4	F005	4	Count/cell	8.666667	10	2.309401
LC3B	W3	HPI4	well_4	F006	10	Count/cell	10.875	8.5	9.448167
LC3B	W3	HPI4	well_5	F001	4	Count/cell	9	9	1.414214
LC3B	W3	HPI4	well_5	F002	1	Count/cell	19	19	NA
LC3B	W3	HPI4	well_5	F005	3	Count/cell	5	5	NA
LC3B	W3	PGE2	well_1	F001	159	Count/cell	14.15686	13	8.564091
LC3B	W3	PGE2	well_1	F002	191	Count/cell	9.010989	8	5.610752
LC3B	W3	PGE2	well_1	F003	213	Count/cell	8.735751	8	5.384934
LC3B	W3	PGE2	well_1	F004	159	Count/cell	11.98621	12	6.953203
LC3B	W3	PGE2	well_1	F005	181	Count/cell	7.804734	7	4.272389
LC3B	W3	PGE2	well_1	F006	264	Count/cell	14.7384	14	7.15855
LC3B	W3	PGE2	well_2	F001	52	Count/cell	13.58	13	8.882659
LC3B	W3	PGE2	well_2	F002	95	Count/cell	16.23529	15	7.734658
LC3B	W3	PGE2	well_2	F003	143	Count/cell	6.882353	6	4.432304
LC3B	W3	PGE2	well_2	F004	207	Count/cell	13.3369	12	6.173032
LC3B	W3	PGE2	well_2	F005	166	Count/cell	10.25503	10	4.313953
LC3B	W3	PGE2	well_2	F006	123	Count/cell	17.71171	17	7.836265
LC3B	W3	PGE2	well_3	F001	114	Count/cell	13.42718	12	6.728054
LC3B	W3	PGE2	well_3	F002	171	Count/cell	12.55484	12	7.05649
LC3B	W3	PGE2	well_3	F003	194	Count/cell	13.28814	13	6.142394
LC3B	W3	PGE2	well_3	F004	172	Count/cell	12.89375	11	7.641822
LC3B	W3	PGE2	well_3	F005	274	Count/cell	12.31855	11	5.763853
LC3B	W3	PGE2	well_3	F006	128	Count/cell	18.4569	18	9.034612
LC3B	W3	PGE2	well_4	F001	61	Count/cell	14.94545	14	6.32578
LC3B	W3	PGE2	well_4	F002	132	Count/cell	15.34167	14	6.66421
LC3B	W3	PGE2	well_4	F003	117	Count/cell	10.80556	11	5.215825
LC3B	W3	PGE2	well_4	F004	255	Count/cell	17.46725	17	7.82736

LC3B	W3	PGE2	well_4	F005	198	Count/cell	13.77473	14	5.377343
LC3B	W3	PGE2	well_4	F006	223	Count/cell	16.3399	16	8.378292
LC3B	W3	PGE2	well_5	F001	223	Count/cell	18.34951	17.5	8.377914
LC3B	W3	PGE2	well_5	F002	264	Count/cell	16.37975	16	7.711927
LC3B	W3	PGE2	well_5	F003	90	Count/cell	10.01235	9	5.606902
LC3B	W3	PGE2	well_5	F004	189	Count/cell	6.050279	5	4.696728
LC3B	W3	PGE2	well_5	F005	202	Count/cell	13.8913	14	6.527716
LC3B	W3	PGE2	well_5	F006	188	Count/cell	16.32759	15	8.341112
LC3B	W4	HPI4	well_1	F001	1	Count/cell	26	26	NA
LC3B	W4	HPI4	well_1	F002	1	Count/cell	112	112	NA
LC3B	W4	HPI4	well_1	F003	1	Count/cell	18	18	NA
LC3B	W4	HPI4	well_1	F005	4	Count/cell	19	19	7.071068
LC3B	W4	HPI4	well_2	F005	1	Count/cell	93	93	NA
LC3B	W4	HPI4	well_3	F001	1	Count/cell	17	17	NA
LC3B	W4	HPI4	well_3	F005	1	Count/cell	17	17	NA
LC3B	W4	HPI4	well_3	F006	7	Count/cell	10.4	11	5.319774
LC3B	W4	HPI4	well_4	F001	3	Count/cell	6	6	NA
LC3B	W4	HPI4	well_4	F003	1	Count/cell	4	4	NA
LC3B	W4	HPI4	well_4	F004	3	Count/cell	23	23	NA
LC3B	W4	PGE2	well_1	F001	105	Count/cell	15.19355	14	6.732882
LC3B	W4	PGE2	well_1	F002	100	Count/cell	13.4	12	6.042546
LC3B	W4	PGE2	well_1	F003	224	Count/cell	15.4505	13	9.45058
LC3B	W4	PGE2	well_1	F004	187	Count/cell	5.847458	5	3.918837
LC3B	W4	PGE2	well_1	F005	222	Count/cell	7.075377	6	4.236607
LC3B	W4	PGE2	well_1	F006	218	Count/cell	9.014925	8	5.919863
LC3B	W4	PGE2	well_2	F001	253	Count/cell	8.123932	7	4.209842
LC3B	W4	PGE2	well_2	F002	142	Count/cell	11.43307	10	6.577488
LC3B	W4	PGE2	well_2	F003	209	Count/cell	11.34921	11	5.747781
LC3B	W4	PGE2	well_2	F004	216	Count/cell	10.76142	10	5.644117
LC3B	W4	PGE2	well_2	F005	248	Count/cell	8.438596	8	4.328036
LC3B	W4	PGE2	well_2	F006	230	Count/cell	10.42453	9	5.521948
LC3B	W4	PGE2	well_3	F001	162	Count/cell	7.804054	7	4.285173
LC3B	W4	PGE2	well_3	F002	150	Count/cell	7.345324	7	3.592892
LC3B	W4	PGE2	well_3	F003	216	Count/cell	7.780612	7	4.890371
LC3B	W4	PGE2	well_3	F004	175	Count/cell	10.5375	9	6.507748
LC3B	W4	PGE2	well_3	F005	260	Count/cell	8.108434	6	5.711502
LC3B	W4	PGE2	well_3	F006	237	Count/cell	10.31982	9	5.392425
LC3B	W4	PGE2	well_4	F001	73	Count/cell	7.318841	5	5.323347
LC3B	W4	PGE2	well_4	F002	74	Count/cell	7.536232	7	4.053247
LC3B	W4	PGE2	well_4	F003	109	Count/cell	11.21212	9	7.869176
LC3B	W4	PGE2	well_4	F004	164	Count/cell	11.34228	10	6.371147
LC3B	W4	PGE2	well_4	F005	221	Count/cell	7.796209	7	5.118704
LC3B	W4	PGE2	well_4	F006	255	Count/cell	8.481013	8	4.241225
LC3B	W4	PGE2	well_5	F001	128	Count/cell	8.817391	8	4.606598
LC3B	W4	PGE2	well_5	F002	34	Count/cell	8.266667	8	4.653018
LC3B	W4	PGE2	well_5	F003	25	Count/cell	10.81818	10.5	5.482755
LC3B	W4	PGE2	well_5	F004	81	Count/cell	10.51351	9	6.129299
LC3B	W4	PGE2	well_5	F005	53	Count/cell	8.02	7	5.040611
LC3B	W4	PGE2	well_5	F006	75	Count/cell	3.837838	3	2.471908

LC3B	W1	HPI4	well_1	F001	154	X/Y distribution	4.796031	4.620207	1.307056
LC3B	W1	HPI4	well_1	F002	100	X/Y distribution	4.631675	4.605639	1.37817
LC3B	W1	HPI4	well_1	F003	64	X/Y distribution	5.090407	5.270309	1.338254
LC3B	W1	HPI4	well_1	F004	73	X/Y distribution	5.922436	5.77342	1.34048
LC3B	W1	HPI4	well_1	F005	67	X/Y distribution	5.931768	5.416193	1.496812
LC3B	W1	HPI4	well_1	F006	72	X/Y distribution	5.137868	5.14109	1.545605
LC3B	W1	HPI4	well_2	F001	120	X/Y distribution	4.89841	4.956468	1.246824
LC3B	W1	HPI4	well_2	F002	136	X/Y distribution	4.84349	4.838986	1.266243
LC3B	W1	HPI4	well_2	F003	97	X/Y distribution	5.18053	5.275118	1.377943
LC3B	W1	HPI4	well_2	F004	75	X/Y distribution	5.59756	5.462433	1.151715
LC3B	W1	HPI4	well_2	F005	91	X/Y distribution	5.822062	5.775868	1.365717
LC3B	W1	HPI4	well_2	F006	45	X/Y distribution	5.806904	5.765457	1.719497
LC3B	W1	HPI4	well_3	F001	79	X/Y distribution	5.286565	5.263905	1.34588
LC3B	W1	HPI4	well_3	F002	91	X/Y distribution	5.165726	4.937831	1.296405
LC3B	W1	HPI4	well_3	F003	97	X/Y distribution	4.966144	4.954765	1.266286
LC3B	W1	HPI4	well_3	F004	103	X/Y distribution	5.201149	4.878739	1.209514
LC3B	W1	HPI4	well_3	F005	84	X/Y distribution	5.230907	5.113182	1.604104
LC3B	W1	HPI4	well_3	F006	59	X/Y distribution	4.930205	4.525548	1.586002
LC3B	W1	HPI4	well_4	F001	118	X/Y distribution	4.648922	4.499353	1.152418
LC3B	W1	HPI4	well_4	F002	106	X/Y distribution	5.060357	5.221163	1.438341
LC3B	W1	HPI4	well_4	F003	80	X/Y distribution	4.599016	4.349788	1.47847
LC3B	W1	HPI4	well_4	F004	65	X/Y distribution	5.278862	5.286946	1.592935
LC3B	W1	HPI4	well_4	F005	96	X/Y distribution	5.091634	5.166937	1.79529
LC3B	W1	HPI4	well_4	F006	116	X/Y distribution	4.825016	4.710591	1.333187
LC3B	W1	HPI4	well_5	F001	108	X/Y distribution	4.068716	3.99812	1.080764
LC3B	W1	HPI4	well_5	F002	163	X/Y distribution	4.883544	5.001954	1.26986
LC3B	W1	HPI4	well_5	F003	70	X/Y distribution	5.6431	5.587598	1.320299
LC3B	W1	HPI4	well_5	F004	88	X/Y distribution	4.460402	4.38513	1.430659
LC3B	W1	HPI4	well_5	F005	104	X/Y distribution	4.615732	4.567802	1.195763
LC3B	W1	HPI4	well_5	F006	92	X/Y distribution	4.990481	5.195038	1.082749
LC3B	W1	PGE2	well_1	F001	154	X/Y distribution	4.855127	4.712137	1.333744
LC3B	W1	PGE2	well_1	F002	167	X/Y distribution	4.87663	4.807606	1.234173
LC3B	W1	PGE2	well_1	F003	124	X/Y distribution	4.641058	4.524436	1.293729
LC3B	W1	PGE2	well_1	F004	97	X/Y distribution	4.795082	4.741231	1.153347
LC3B	W1	PGE2	well_1	F005	120	X/Y distribution	4.935593	4.854792	1.367083
LC3B	W1	PGE2	well_1	F006	166	X/Y distribution	5.378808	5.230109	1.464756
LC3B	W1	PGE2	well_2	F001	95	X/Y distribution	5.178183	5.15004	1.159373
LC3B	W1	PGE2	well_2	F002	96	X/Y distribution	4.984462	5.164332	1.249406
LC3B	W1	PGE2	well_2	F003	90	X/Y distribution	5.612029	5.324713	1.455605
LC3B	W1	PGE2	well_2	F004	59	X/Y distribution	5.393787	5.043389	2.04814
LC3B	W1	PGE2	well_2	F005	94	X/Y distribution	5.744269	5.677844	1.418726
LC3B	W1	PGE2	well_2	F006	66	X/Y distribution	5.453935	5.356209	1.283865
LC3B	W1	PGE2	well_3	F001	101	X/Y distribution	4.967319	5.007744	1.40403
LC3B	W1	PGE2	well_3	F002	116	X/Y distribution	4.901105	4.832697	1.189997
LC3B	W1	PGE2	well_3	F003	72	X/Y distribution	5.275747	5.425897	1.443492
LC3B	W1	PGE2	well_3	F004	54	X/Y distribution	5.33506	5.288461	1.216868
LC3B	W1	PGE2	well_3	F005	50	X/Y distribution	4.615774	4.521118	1.273963
LC3B	W1	PGE2	well_3	F006	69	X/Y distribution	4.734095	4.689392	0.981931
LC3B	W1	PGE2	well_4	F001	73	X/Y distribution	4.792792	4.712579	1.545203

LC3B	W1	PGE2	well_4	F002	73	X/Y distribution	5.651872	5.716522	1.220684
LC3B	W1	PGE2	well_4	F003	92	X/Y distribution	4.992407	5.052406	1.258821
LC3B	W1	PGE2	well_4	F004	55	X/Y distribution	5.296651	5.387583	1.273119
LC3B	W1	PGE2	well_4	F005	23	X/Y distribution	6.272783	6.391413	1.233103
LC3B	W1	PGE2	well_4	F006	55	X/Y distribution	5.514984	5.598719	1.716495
LC3B	W1	PGE2	well_5	F001	82	X/Y distribution	4.850798	4.672295	1.045881
LC3B	W1	PGE2	well_5	F002	89	X/Y distribution	5.232584	5.153855	1.283044
LC3B	W1	PGE2	well_5	F003	30	X/Y distribution	5.351089	5.022301	1.232492
LC3B	W1	PGE2	well_5	F004	35	X/Y distribution	4.991518	4.832465	1.770086
LC3B	W1	PGE2	well_5	F005	60	X/Y distribution	4.863813	4.929851	1.156301
LC3B	W1	PGE2	well_5	F006	78	X/Y distribution	5.271112	5.489042	1.327253
LC3B	W2	HPI4	well_1	F001	29	X/Y distribution	5.072374	4.640346	1.373496
LC3B	W2	HPI4	well_1	F002	58	X/Y distribution	6.446462	6.505312	1.342435
LC3B	W2	HPI4	well_1	F003	46	X/Y distribution	6.198645	6.543475	1.819657
LC3B	W2	HPI4	well_1	F004	21	X/Y distribution	5.648498	6.096507	1.83881
LC3B	W2	HPI4	well_1	F005	79	X/Y distribution	6.591524	6.536467	1.506056
LC3B	W2	HPI4	well_1	F006	44	X/Y distribution	6.451767	6.63412	1.157277
LC3B	W2	HPI4	well_2	F001	25	X/Y distribution	5.070676	4.920566	1.319676
LC3B	W2	HPI4	well_2	F002	59	X/Y distribution	5.359703	5.105182	1.539016
LC3B	W2	HPI4	well_2	F003	39	X/Y distribution	5.258725	5.327463	1.284037
LC3B	W2	HPI4	well_2	F004	39	X/Y distribution	5.71796	5.580792	1.287542
LC3B	W2	HPI4	well_2	F005	48	X/Y distribution	6.832858	6.580405	1.603936
LC3B	W2	HPI4	well_2	F006	28	X/Y distribution	4.749808	4.710523	1.324776
LC3B	W2	HPI4	well_3	F001	102	X/Y distribution	5.848186	5.701354	1.146535
LC3B	W2	HPI4	well_3	F002	92	X/Y distribution	6.199496	6.190311	1.347599
LC3B	W2	HPI4	well_3	F003	66	X/Y distribution	5.371601	5.319796	1.282064
LC3B	W2	HPI4	well_3	F004	22	X/Y distribution	4.805748	4.545243	1.110812
LC3B	W2	HPI4	well_3	F005	9	X/Y distribution	4.568467	4.381015	0.98269
LC3B	W2	HPI4	well_3	F006	52	X/Y distribution	5.947815	5.938244	1.773236
LC3B	W2	HPI4	well_4	F001	77	X/Y distribution	6.025201	6.062085	1.152404
LC3B	W2	HPI4	well_4	F002	71	X/Y distribution	5.419827	5.384323	1.442212
LC3B	W2	HPI4	well_4	F003	18	X/Y distribution	5.140938	5.056084	1.167185
LC3B	W2	HPI4	well_4	F004	13	X/Y distribution	6.230247	6.245615	1.096235
LC3B	W2	HPI4	well_4	F005	5	X/Y distribution	4.144352	4.395652	0.499619
LC3B	W2	HPI4	well_4	F006	33	X/Y distribution	4.566721	4.308358	1.480336
LC3B	W2	HPI4	well_5	F001	22	X/Y distribution	5.101109	4.792993	1.165913
LC3B	W2	HPI4	well_5	F002	44	X/Y distribution	4.379996	4.033189	1.132592
LC3B	W2	HPI4	well_5	F003	45	X/Y distribution	5.17141	5.467098	1.398587
LC3B	W2	HPI4	well_5	F004	27	X/Y distribution	4.287065	4.227931	0.573373
LC3B	W2	HPI4	well_5	F005	27	X/Y distribution	4.337991	4.423977	0.515654
LC3B	W2	HPI4	well_5	F006	6	X/Y distribution	4.21774	4.105156	0.298608
LC3B	W2	PGE2	well_1	F001	40	X/Y distribution	5.09879	4.917366	1.261628
LC3B	W2	PGE2	well_1	F002	39	X/Y distribution	4.752355	5.039911	1.180993
LC3B	W2	PGE2	well_1	F003	46	X/Y distribution	5.612115	5.616469	1.424917
LC3B	W2	PGE2	well_1	F004	106	X/Y distribution	5.689356	5.637447	0.809417
LC3B	W2	PGE2	well_1	F005	200	X/Y distribution	5.478009	5.454751	0.996758
LC3B	W2	PGE2	well_1	F006	134	X/Y distribution	5.660054	5.601658	0.971345
LC3B	W2	PGE2	well_2	F001	40	X/Y distribution	5.47708	5.453913	1.137628
LC3B	W2	PGE2	well_2	F002	65	X/Y distribution	4.598109	4.571279	1.411874

LC3B	W2	PGE2	well_2	F003	158	X/Y distribution	6.078379	6.219403	0.901231
LC3B	W2	PGE2	well_2	F004	149	X/Y distribution	5.954361	5.910113	0.862308
LC3B	W2	PGE2	well_2	F005	121	X/Y distribution	5.699027	5.660532	1.009711
LC3B	W2	PGE2	well_2	F006	165	X/Y distribution	5.631233	5.615261	0.935912
LC3B	W2	PGE2	well_3	F001	71	X/Y distribution	5.068431	5.110882	1.22803
LC3B	W2	PGE2	well_3	F002	147	X/Y distribution	5.844892	5.947156	0.964629
LC3B	W2	PGE2	well_3	F003	236	X/Y distribution	5.812213	5.802337	1.0712
LC3B	W2	PGE2	well_3	F004	54	X/Y distribution	5.294558	5.180102	1.264964
LC3B	W2	PGE2	well_3	F005	58	X/Y distribution	5.922065	6.030756	1.439595
LC3B	W2	PGE2	well_3	F006	35	X/Y distribution	5.870517	6.100383	1.311619
LC3B	W2	PGE2	well_4	F001	114	X/Y distribution	5.552404	5.514792	1.122927
LC3B	W2	PGE2	well_4	F002	59	X/Y distribution	5.382591	5.404684	1.542597
LC3B	W2	PGE2	well_4	F003	76	X/Y distribution	5.566267	5.460027	1.32441
LC3B	W2	PGE2	well_4	F004	235	X/Y distribution	6.258621	6.08734	1.148588
LC3B	W2	PGE2	well_4	F005	47	X/Y distribution	6.232456	5.881455	1.078933
LC3B	W2	PGE2	well_4	F006	60	X/Y distribution	5.45024	5.578738	1.685756
LC3B	W2	PGE2	well_5	F001	122	X/Y distribution	5.726527	5.687592	1.01905
LC3B	W2	PGE2	well_5	F002	139	X/Y distribution	5.893796	5.934777	1.072758
LC3B	W2	PGE2	well_5	F003	104	X/Y distribution	5.845276	5.779758	1.289982
LC3B	W2	PGE2	well_5	F004	155	X/Y distribution	5.784557	5.837718	1.14033
LC3B	W2	PGE2	well_5	F005	85	X/Y distribution	5.603586	5.529471	1.293673
LC3B	W2	PGE2	well_5	F006	67	X/Y distribution	5.785347	5.765764	0.953699
LC3B	W3	HPI4	well_1	F001	20	X/Y distribution	8.802979	8.925983	1.979184
LC3B	W3	HPI4	well_1	F002	5	X/Y distribution	9.119599	8.748656	1.088674
LC3B	W3	HPI4	well_1	F003	8	X/Y distribution	7.294795	7.459604	1.739144
LC3B	W3	HPI4	well_1	F004	14	X/Y distribution	8.781589	9.130196	3.093806
LC3B	W3	HPI4	well_1	F005	14	X/Y distribution	8.590675	6.931905	3.439119
LC3B	W3	HPI4	well_1	F006	28	X/Y distribution	6.216118	5.94425	1.357327
LC3B	W3	HPI4	well_2	F001	1	X/Y distribution	3.899908	3.899908	NA
LC3B	W3	HPI4	well_2	F002	6	X/Y distribution	6.544151	6.351375	0.652782
LC3B	W3	HPI4	well_2	F003	23	X/Y distribution	8.439389	8.239373	2.683843
LC3B	W3	HPI4	well_2	F004	7	X/Y distribution	5.745107	6.266206	1.69866
LC3B	W3	HPI4	well_2	F005	9	X/Y distribution	7.768297	5.86673	3.299837
LC3B	W3	HPI4	well_2	F006	10	X/Y distribution	7.249204	6.99965	2.124715
LC3B	W3	HPI4	well_3	F001	7	X/Y distribution	7.705817	7.230374	2.281046
LC3B	W3	HPI4	well_3	F003	1	X/Y distribution	9.418958	9.418958	NA
LC3B	W3	HPI4	well_3	F004	1	X/Y distribution	7.353848	7.353848	NA
LC3B	W3	HPI4	well_3	F005	4	X/Y distribution	6.932448	6.932448	2.072457
LC3B	W3	HPI4	well_3	F006	19	X/Y distribution	5.661509	5.61184	1.254975
LC3B	W3	HPI4	well_4	F002	5	X/Y distribution	9.206351	9.730335	0.93363
LC3B	W3	HPI4	well_4	F005	4	X/Y distribution	3.509449	3.509449	0.743766
LC3B	W3	HPI4	well_4	F006	10	X/Y distribution	4.748551	4.749636	0.862492
LC3B	W3	HPI4	well_5	F001	4	X/Y distribution	8.390508	8.390508	0.371477
LC3B	W3	HPI4	well_5	F002	1	X/Y distribution	4.587101	4.587101	NA
LC3B	W3	HPI4	well_5	F005	3	X/Y distribution	4.653513	4.653513	NA
LC3B	W3	PGE2	well_1	F001	159	X/Y distribution	6.101173	6.352605	1.447076
LC3B	W3	PGE2	well_1	F002	191	X/Y distribution	5.58957	5.554623	1.193326
LC3B	W3	PGE2	well_1	F003	213	X/Y distribution	6.001436	6.014774	1.337309
LC3B	W3	PGE2	well_1	F004	159	X/Y distribution	6.424819	6.338322	1.530869

LC3B	W3	PGE2	well_1	F005	181	X/Y distribution	6.10902	6.104239	1.489593
LC3B	W3	PGE2	well_1	F006	264	X/Y distribution	6.411426	6.309956	1.239741
LC3B	W3	PGE2	well_2	F001	52	X/Y distribution	5.675281	5.580777	1.083928
LC3B	W3	PGE2	well_2	F002	95	X/Y distribution	5.690779	5.564516	0.992034
LC3B	W3	PGE2	well_2	F003	143	X/Y distribution	6.161779	6.107386	1.193043
LC3B	W3	PGE2	well_2	F004	207	X/Y distribution	5.53214	5.582668	0.912722
LC3B	W3	PGE2	well_2	F005	166	X/Y distribution	5.946148	5.922037	0.938909
LC3B	W3	PGE2	well_2	F006	123	X/Y distribution	5.992686	5.927113	0.948615
LC3B	W3	PGE2	well_3	F001	114	X/Y distribution	5.708608	5.748189	1.045732
LC3B	W3	PGE2	well_3	F002	171	X/Y distribution	5.380553	5.245115	1.08778
LC3B	W3	PGE2	well_3	F003	194	X/Y distribution	5.952412	5.96148	0.950641
LC3B	W3	PGE2	well_3	F004	172	X/Y distribution	6.285438	6.197389	0.995475
LC3B	W3	PGE2	well_3	F005	274	X/Y distribution	5.852026	5.816236	0.904854
LC3B	W3	PGE2	well_3	F006	128	X/Y distribution	5.926372	5.806848	1.012713
LC3B	W3	PGE2	well_4	F001	61	X/Y distribution	6.029312	5.990394	1.075172
LC3B	W3	PGE2	well_4	F002	132	X/Y distribution	5.493514	5.499651	0.918298
LC3B	W3	PGE2	well_4	F003	117	X/Y distribution	5.327832	5.471843	0.967852
LC3B	W3	PGE2	well_4	F004	255	X/Y distribution	5.596799	5.475761	0.988465
LC3B	W3	PGE2	well_4	F005	198	X/Y distribution	6.281868	6.200543	0.87123
LC3B	W3	PGE2	well_4	F006	223	X/Y distribution	5.793018	5.713943	1.016658
LC3B	W3	PGE2	well_5	F001	223	X/Y distribution	6.654414	6.586034	1.138889
LC3B	W3	PGE2	well_5	F002	264	X/Y distribution	6.084653	6.034173	1.066357
LC3B	W3	PGE2	well_5	F003	90	X/Y distribution	6.195444	6.188397	1.156385
LC3B	W3	PGE2	well_5	F004	189	X/Y distribution	6.249936	6.11551	1.542864
LC3B	W3	PGE2	well_5	F005	202	X/Y distribution	5.998514	5.950013	0.984224
LC3B	W3	PGE2	well_5	F006	188	X/Y distribution	6.110125	6.05868	1.101594
LC3B	W4	HPI4	well_1	F001	1	X/Y distribution	15.00674	15.00674	NA
LC3B	W4	HPI4	well_1	F002	1	X/Y distribution	9.425223	9.425223	NA
LC3B	W4	HPI4	well_1	F003	1	X/Y distribution	9.074056	9.074056	NA
LC3B	W4	HPI4	well_1	F005	4	X/Y distribution	3.662242	3.662242	0.115788
LC3B	W4	HPI4	well_2	F005	1	X/Y distribution	9.431712	9.431712	NA
LC3B	W4	HPI4	well_3	F001	1	X/Y distribution	6.596253	6.596253	NA
LC3B	W4	HPI4	well_3	F005	1	X/Y distribution	4.764613	4.764613	NA
LC3B	W4	HPI4	well_3	F006	7	X/Y distribution	3.673255	3.70425	0.199058
LC3B	W4	HPI4	well_4	F001	3	X/Y distribution	3.487548	3.487548	NA
LC3B	W4	HPI4	well_4	F003	1	X/Y distribution	7.370401	7.370401	NA
LC3B	W4	HPI4	well_4	F004	3	X/Y distribution	5.524685	5.524685	NA
LC3B	W4	PGE2	well_1	F001	105	X/Y distribution	5.792243	5.72885	0.770588
LC3B	W4	PGE2	well_1	F002	100	X/Y distribution	5.604169	5.565352	0.770668
LC3B	W4	PGE2	well_1	F003	224	X/Y distribution	5.680812	5.650503	0.872156
LC3B	W4	PGE2	well_1	F004	187	X/Y distribution	5.905939	5.78499	1.249853
LC3B	W4	PGE2	well_1	F005	222	X/Y distribution	5.506703	5.47358	1.106956
LC3B	W4	PGE2	well_1	F006	218	X/Y distribution	5.591043	5.594333	0.997423
LC3B	W4	PGE2	well_2	F001	253	X/Y distribution	5.343014	5.237592	0.982684
LC3B	W4	PGE2	well_2	F002	142	X/Y distribution	6.012921	5.985144	1.200479
LC3B	W4	PGE2	well_2	F003	209	X/Y distribution	5.828726	5.711771	1.02526
LC3B	W4	PGE2	well_2	F004	216	X/Y distribution	5.651454	5.558145	0.95502
LC3B	W4	PGE2	well_2	F005	248	X/Y distribution	5.362787	5.380972	0.962365
LC3B	W4	PGE2	well_2	F006	230	X/Y distribution	5.700353	5.66944	0.963875

LC3B	W4	PGE2	well_3	F001	162	X/Y distribution	6.192404	6.0388	1.309044
LC3B	W4	PGE2	well_3	F002	150	X/Y distribution	5.459203	5.390961	0.896782
LC3B	W4	PGE2	well_3	F003	216	X/Y distribution	5.564593	5.504992	1.187019
LC3B	W4	PGE2	well_3	F004	175	X/Y distribution	5.91379	5.827759	1.032206
LC3B	W4	PGE2	well_3	F005	260	X/Y distribution	5.71616	5.568006	1.438274
LC3B	W4	PGE2	well_3	F006	237	X/Y distribution	5.958071	5.842859	1.294099
LC3B	W4	PGE2	well_4	F001	73	X/Y distribution	5.961512	5.761438	1.464643
LC3B	W4	PGE2	well_4	F002	74	X/Y distribution	5.738566	5.694922	1.021241
LC3B	W4	PGE2	well_4	F003	109	X/Y distribution	5.961107	5.990652	1.127723
LC3B	W4	PGE2	well_4	F004	164	X/Y distribution	6.385072	6.082904	1.34982
LC3B	W4	PGE2	well_4	F005	221	X/Y distribution	5.700354	5.664923	1.070648
LC3B	W4	PGE2	well_4	F006	255	X/Y distribution	5.308911	5.230117	0.980908
LC3B	W4	PGE2	well_5	F001	128	X/Y distribution	5.609253	5.450364	0.955065
LC3B	W4	PGE2	well_5	F002	34	X/Y distribution	5.865111	5.551822	1.27649
LC3B	W4	PGE2	well_5	F003	25	X/Y distribution	5.554715	5.377891	1.050686
LC3B	W4	PGE2	well_5	F004	81	X/Y distribution	5.643009	5.394985	1.276286
LC3B	W4	PGE2	well_5	F005	53	X/Y distribution	5.856975	5.861792	1.28191
LC3B	W4	PGE2	well_5	F006	75	X/Y distribution	5.946138	5.925777	1.198723
LC3B	W1	HPI4	well_1	F001	154	Z distribution	-0.03966	-0.00588	0.483574
LC3B	W1	HPI4	well_1	F002	100	Z distribution	-0.14353	-0.20654	0.535513
LC3B	W1	HPI4	well_1	F003	64	Z distribution	-0.10202	-0.15637	0.464433
LC3B	W1	HPI4	well_1	F004	73	Z distribution	-0.35814	-0.38034	0.763814
LC3B	W1	HPI4	well_1	F005	67	Z distribution	-0.30888	-0.2447	0.452929
LC3B	W1	HPI4	well_1	F006	72	Z distribution	-0.17047	-0.21586	0.460617
LC3B	W1	HPI4	well_2	F001	120	Z distribution	-0.11111	-0.1051	0.494304
LC3B	W1	HPI4	well_2	F002	136	Z distribution	-0.13726	-0.12698	0.661703
LC3B	W1	HPI4	well_2	F003	97	Z distribution	-0.17212	-0.10805	0.444309
LC3B	W1	HPI4	well_2	F004	75	Z distribution	-0.30501	-0.2975	0.369105
LC3B	W1	HPI4	well_2	F005	91	Z distribution	-0.16756	-0.16283	0.431404
LC3B	W1	HPI4	well_2	F006	45	Z distribution	-0.01871	-0.03768	0.356187
LC3B	W1	HPI4	well_3	F001	79	Z distribution	0.043863	-0.00079	0.487527
LC3B	W1	HPI4	well_3	F002	91	Z distribution	-0.24834	-0.27647	0.463291
LC3B	W1	HPI4	well_3	F003	97	Z distribution	0.023163	0.057878	0.515358
LC3B	W1	HPI4	well_3	F004	103	Z distribution	-0.2811	-0.25115	0.601074
LC3B	W1	HPI4	well_3	F005	84	Z distribution	-0.30427	-0.16567	0.68132
LC3B	W1	HPI4	well_3	F006	59	Z distribution	-0.05944	-0.12335	0.407559
LC3B	W1	HPI4	well_4	F001	118	Z distribution	0.104641	0.072129	0.494191
LC3B	W1	HPI4	well_4	F002	106	Z distribution	-0.23633	-0.29174	0.506395
LC3B	W1	HPI4	well_4	F003	80	Z distribution	-0.13517	-0.11751	0.465152
LC3B	W1	HPI4	well_4	F004	65	Z distribution	-0.19963	-0.18551	0.463926
LC3B	W1	HPI4	well_4	F005	96	Z distribution	-0.08938	-0.07252	0.550758
LC3B	W1	HPI4	well_4	F006	116	Z distribution	-0.01233	-0.00752	0.572038
LC3B	W1	HPI4	well_5	F001	108	Z distribution	0.016989	-0.04394	0.515878
LC3B	W1	HPI4	well_5	F002	163	Z distribution	0.146311	0.100489	0.565305
LC3B	W1	HPI4	well_5	F003	70	Z distribution	-0.09264	-0.14252	0.341572
LC3B	W1	HPI4	well_5	F004	88	Z distribution	-0.22225	-0.19	0.477459
LC3B	W1	HPI4	well_5	F005	104	Z distribution	0.045673	-0.04154	0.471511
LC3B	W1	HPI4	well_5	F006	92	Z distribution	-0.12011	-0.12565	0.564282
LC3B	W1	PGE2	well_1	F001	154	Z distribution	0.042307	0.019393	0.593337

LC3B	W1	PGE2	well_1	F002	167	Z distribution	0.112211	0.083381	0.575169
LC3B	W1	PGE2	well_1	F003	124	Z distribution	-0.0609	-0.05649	0.459688
LC3B	W1	PGE2	well_1	F004	97	Z distribution	-0.20958	-0.2303	0.582602
LC3B	W1	PGE2	well_1	F005	120	Z distribution	-0.17823	-0.13696	0.511074
LC3B	W1	PGE2	well_1	F006	166	Z distribution	-0.11598	-0.15976	0.556832
LC3B	W1	PGE2	well_2	F001	95	Z distribution	-0.06028	-0.11102	0.470247
LC3B	W1	PGE2	well_2	F002	96	Z distribution	-0.0799	-0.10649	0.643463
LC3B	W1	PGE2	well_2	F003	90	Z distribution	-0.13488	-0.11418	0.434095
LC3B	W1	PGE2	well_2	F004	59	Z distribution	-0.31182	-0.37304	0.504688
LC3B	W1	PGE2	well_2	F005	94	Z distribution	-0.04395	-0.02379	0.538553
LC3B	W1	PGE2	well_2	F006	66	Z distribution	-0.12666	-0.13568	0.496758
LC3B	W1	PGE2	well_3	F001	101	Z distribution	-0.11523	-0.08056	0.529561
LC3B	W1	PGE2	well_3	F002	116	Z distribution	-0.19805	-0.17365	0.479007
LC3B	W1	PGE2	well_3	F003	72	Z distribution	-0.25569	-0.27837	0.40819
LC3B	W1	PGE2	well_3	F004	54	Z distribution	-0.39513	-0.34507	0.591056
LC3B	W1	PGE2	well_3	F005	50	Z distribution	-0.21733	-0.23391	0.429436
LC3B	W1	PGE2	well_3	F006	69	Z distribution	-0.20127	-0.20809	0.435542
LC3B	W1	PGE2	well_4	F001	73	Z distribution	-0.19843	-0.02467	0.532436
LC3B	W1	PGE2	well_4	F002	73	Z distribution	-0.10499	-0.08663	0.410666
LC3B	W1	PGE2	well_4	F003	92	Z distribution	-0.40696	-0.32319	0.530967
LC3B	W1	PGE2	well_4	F004	55	Z distribution	-0.36259	-0.36737	0.500042
LC3B	W1	PGE2	well_4	F005	23	Z distribution	-0.25357	-0.25225	0.258038
LC3B	W1	PGE2	well_4	F006	55	Z distribution	-0.43299	-0.29149	0.458898
LC3B	W1	PGE2	well_5	F001	82	Z distribution	-0.31461	-0.30602	0.497748
LC3B	W1	PGE2	well_5	F002	89	Z distribution	-0.30121	-0.27048	0.512709
LC3B	W1	PGE2	well_5	F003	30	Z distribution	-0.22474	-0.1609	0.327966
LC3B	W1	PGE2	well_5	F004	35	Z distribution	-0.25478	-0.08457	0.387987
LC3B	W1	PGE2	well_5	F005	60	Z distribution	-0.3285	-0.26011	0.327911
LC3B	W1	PGE2	well_5	F006	78	Z distribution	-0.0504	-0.11538	0.535627
LC3B	W2	HPI4	well_1	F001	29	Z distribution	-0.12846	-0.10509	0.331203
LC3B	W2	HPI4	well_1	F002	58	Z distribution	-0.2751	-0.20905	0.331507
LC3B	W2	HPI4	well_1	F003	46	Z distribution	-0.54484	-0.47991	0.373504
LC3B	W2	HPI4	well_1	F004	21	Z distribution	-0.27618	-0.15462	0.576627
LC3B	W2	HPI4	well_1	F005	79	Z distribution	-0.05896	-0.05669	0.360457
LC3B	W2	HPI4	well_1	F006	44	Z distribution	-0.12554	-0.06767	0.255754
LC3B	W2	HPI4	well_2	F001	25	Z distribution	-0.53561	-0.39446	0.544514
LC3B	W2	HPI4	well_2	F002	59	Z distribution	-0.00661	0.068328	0.501558
LC3B	W2	HPI4	well_2	F003	39	Z distribution	-0.19643	-0.10242	0.432701
LC3B	W2	HPI4	well_2	F004	39	Z distribution	-0.13655	-0.04619	0.340847
LC3B	W2	HPI4	well_2	F005	48	Z distribution	-0.30816	-0.34152	0.409579
LC3B	W2	HPI4	well_2	F006	28	Z distribution	-0.53695	-0.57546	0.544136
LC3B	W2	HPI4	well_3	F001	102	Z distribution	0.081038	0.060552	0.333142
LC3B	W2	HPI4	well_3	F002	92	Z distribution	0.070791	0.014066	0.35308
LC3B	W2	HPI4	well_3	F003	66	Z distribution	-0.06103	-0.03774	0.355764
LC3B	W2	HPI4	well_3	F004	22	Z distribution	-0.34501	-0.27706	0.291107
LC3B	W2	HPI4	well_3	F005	9	Z distribution	-0.38554	-0.43221	0.231725
LC3B	W2	HPI4	well_3	F006	52	Z distribution	-0.15971	-0.12911	0.429579
LC3B	W2	HPI4	well_4	F001	77	Z distribution	0.144158	0.114491	0.312844
LC3B	W2	HPI4	well_4	F002	71	Z distribution	-0.05832	-0.04587	0.34067

LC3B	W2	HPI4	well_4	F003	18	Z distribution	-0.22717	-0.23523	0.27253
LC3B	W2	HPI4	well_4	F004	13	Z distribution	-0.28199	-0.22777	0.290639
LC3B	W2	HPI4	well_4	F005	5	Z distribution	-0.21688	-0.17816	0.186965
LC3B	W2	HPI4	well_4	F006	33	Z distribution	-0.29195	-0.21088	0.508338
LC3B	W2	HPI4	well_5	F001	22	Z distribution	-0.23342	-0.21893	0.189706
LC3B	W2	HPI4	well_5	F002	44	Z distribution	-0.4191	-0.2537	0.536221
LC3B	W2	HPI4	well_5	F003	45	Z distribution	-0.45608	-0.37764	0.554519
LC3B	W2	HPI4	well_5	F004	27	Z distribution	-0.34735	-0.27824	0.539355
LC3B	W2	HPI4	well_5	F005	27	Z distribution	-0.228	-0.18766	0.587649
LC3B	W2	HPI4	well_5	F006	6	Z distribution	-0.04325	-0.06276	0.253216
LC3B	W2	PGE2	well_1	F001	40	Z distribution	-0.46394	-0.34848	0.521474
LC3B	W2	PGE2	well_1	F002	39	Z distribution	-0.16974	-0.2636	0.662491
LC3B	W2	PGE2	well_1	F003	46	Z distribution	-0.25809	-0.36107	0.506435
LC3B	W2	PGE2	well_1	F004	106	Z distribution	-0.2266	-0.21052	0.354518
LC3B	W2	PGE2	well_1	F005	200	Z distribution	-0.32537	-0.32631	0.518646
LC3B	W2	PGE2	well_1	F006	134	Z distribution	-0.18489	-0.15141	0.427298
LC3B	W2	PGE2	well_2	F001	40	Z distribution	-0.3404	-0.29864	0.385902
LC3B	W2	PGE2	well_2	F002	65	Z distribution	-0.24053	-0.23702	0.499522
LC3B	W2	PGE2	well_2	F003	158	Z distribution	-0.08897	-0.09599	0.439737
LC3B	W2	PGE2	well_2	F004	149	Z distribution	-0.28161	-0.22643	0.410093
LC3B	W2	PGE2	well_2	F005	121	Z distribution	-0.23535	-0.17403	0.397933
LC3B	W2	PGE2	well_2	F006	165	Z distribution	-0.1879	-0.23245	0.444147
LC3B	W2	PGE2	well_3	F001	71	Z distribution	-0.31239	-0.33649	0.433638
LC3B	W2	PGE2	well_3	F002	147	Z distribution	-0.13063	-0.12796	0.427369
LC3B	W2	PGE2	well_3	F003	236	Z distribution	-0.06865	-0.13349	0.502807
LC3B	W2	PGE2	well_3	F004	54	Z distribution	-0.37127	-0.29951	0.399669
LC3B	W2	PGE2	well_3	F005	58	Z distribution	-0.30737	-0.35534	0.419195
LC3B	W2	PGE2	well_3	F006	35	Z distribution	-0.45096	-0.48184	0.4862
LC3B	W2	PGE2	well_4	F001	114	Z distribution	-0.32593	-0.29457	0.420928
LC3B	W2	PGE2	well_4	F002	59	Z distribution	-0.38114	-0.32784	0.501532
LC3B	W2	PGE2	well_4	F003	76	Z distribution	-0.11727	-0.09854	0.399178
LC3B	W2	PGE2	well_4	F004	235	Z distribution	-0.32435	-0.32849	0.41898
LC3B	W2	PGE2	well_4	F005	47	Z distribution	-0.37007	-0.36902	0.326203
LC3B	W2	PGE2	well_4	F006	60	Z distribution	-0.41106	-0.45634	0.376105
LC3B	W2	PGE2	well_5	F001	122	Z distribution	-0.2829	-0.26707	0.424894
LC3B	W2	PGE2	well_5	F002	139	Z distribution	-0.23993	-0.21799	0.378148
LC3B	W2	PGE2	well_5	F003	104	Z distribution	-0.26266	-0.31496	0.444265
LC3B	W2	PGE2	well_5	F004	155	Z distribution	-0.09115	-0.08109	0.378542
LC3B	W2	PGE2	well_5	F005	85	Z distribution	-0.16138	-0.19359	0.336773
LC3B	W2	PGE2	well_5	F006	67	Z distribution	-0.24864	-0.26503	0.392681
LC3B	W3	HPI4	well_1	F001	20	Z distribution	-0.64552	-0.5075	0.401128
LC3B	W3	HPI4	well_1	F002	5	Z distribution	-0.30625	-0.3578	0.250789
LC3B	W3	HPI4	well_1	F003	8	Z distribution	-0.61394	-0.63857	0.354082
LC3B	W3	HPI4	well_1	F004	14	Z distribution	-0.2818	-0.30445	0.29963
LC3B	W3	HPI4	well_1	F005	14	Z distribution	-0.14382	-0.20162	0.222874
LC3B	W3	HPI4	well_1	F006	28	Z distribution	-0.32501	-0.35922	0.321762
LC3B	W3	HPI4	well_2	F001	1	Z distribution	-0.03895	-0.03895	NA
LC3B	W3	HPI4	well_2	F002	6	Z distribution	-0.26477	-0.16996	0.34652
LC3B	W3	HPI4	well_2	F003	23	Z distribution	-0.3802	-0.39937	0.28295

LC3B	W3	HPI4	well_2	F004	7	Z distribution	-0.26627	-0.29351	0.209822
LC3B	W3	HPI4	well_2	F005	9	Z distribution	-0.28727	-0.43809	0.328133
LC3B	W3	HPI4	well_2	F006	10	Z distribution	-0.42314	-0.21228	0.64741
LC3B	W3	HPI4	well_3	F001	7	Z distribution	-0.32169	-0.18367	0.237658
LC3B	W3	HPI4	well_3	F003	1	Z distribution	-0.10173	-0.10173	NA
LC3B	W3	HPI4	well_3	F004	1	Z distribution	0.11567	0.11567	NA
LC3B	W3	HPI4	well_3	F005	4	Z distribution	-0.10606	-0.10606	0.070565
LC3B	W3	HPI4	well_3	F006	19	Z distribution	-0.62868	-0.47423	0.53318
LC3B	W3	HPI4	well_4	F002	5	Z distribution	-0.23115	-0.21889	0.108846
LC3B	W3	HPI4	well_4	F005	4	Z distribution	-0.71043	-0.71043	0.270027
LC3B	W3	HPI4	well_4	F006	10	Z distribution	-0.27169	-0.31115	0.370403
LC3B	W3	HPI4	well_5	F001	4	Z distribution	-0.38519	-0.38519	0.154548
LC3B	W3	HPI4	well_5	F002	1	Z distribution	-0.4647	-0.4647	NA
LC3B	W3	HPI4	well_5	F005	3	Z distribution	-0.50161	-0.50161	NA
LC3B	W3	PGE2	well_1	F001	159	Z distribution	-0.49827	-0.53667	0.46366
LC3B	W3	PGE2	well_1	F002	191	Z distribution	-0.60189	-0.63955	0.469407
LC3B	W3	PGE2	well_1	F003	213	Z distribution	-0.66089	-0.73325	0.540706
LC3B	W3	PGE2	well_1	F004	159	Z distribution	-0.62965	-0.65768	0.45763
LC3B	W3	PGE2	well_1	F005	181	Z distribution	-0.39274	-0.40985	0.428826
LC3B	W3	PGE2	well_1	F006	264	Z distribution	-0.29616	-0.28879	0.443704
LC3B	W3	PGE2	well_2	F001	52	Z distribution	-0.36642	-0.41113	0.325321
LC3B	W3	PGE2	well_2	F002	95	Z distribution	-0.38475	-0.3668	0.32229
LC3B	W3	PGE2	well_2	F003	143	Z distribution	-0.44475	-0.42523	0.397039
LC3B	W3	PGE2	well_2	F004	207	Z distribution	-0.54475	-0.52568	0.382285
LC3B	W3	PGE2	well_2	F005	166	Z distribution	-0.30981	-0.28379	0.452464
LC3B	W3	PGE2	well_2	F006	123	Z distribution	-0.31325	-0.34993	0.352599
LC3B	W3	PGE2	well_3	F001	114	Z distribution	-0.34815	-0.32863	0.390779
LC3B	W3	PGE2	well_3	F002	171	Z distribution	-0.21968	-0.23616	0.426162
LC3B	W3	PGE2	well_3	F003	194	Z distribution	-0.4027	-0.37311	0.427407
LC3B	W3	PGE2	well_3	F004	172	Z distribution	-0.60712	-0.59761	0.436987
LC3B	W3	PGE2	well_3	F005	274	Z distribution	-0.54488	-0.54934	0.424715
LC3B	W3	PGE2	well_3	F006	128	Z distribution	-0.3316	-0.29276	0.353547
LC3B	W3	PGE2	well_4	F001	61	Z distribution	-0.38816	-0.33501	0.42791
LC3B	W3	PGE2	well_4	F002	132	Z distribution	-0.34899	-0.35766	0.371107
LC3B	W3	PGE2	well_4	F003	117	Z distribution	-0.43691	-0.43816	0.424769
LC3B	W3	PGE2	well_4	F004	255	Z distribution	-0.7554	-0.78283	0.409606
LC3B	W3	PGE2	well_4	F005	198	Z distribution	-0.49397	-0.54056	0.383885
LC3B	W3	PGE2	well_4	F006	223	Z distribution	-0.33645	-0.35018	0.386297
LC3B	W3	PGE2	well_5	F001	223	Z distribution	-0.34404	-0.33471	0.43574
LC3B	W3	PGE2	well_5	F002	264	Z distribution	-0.29717	-0.27137	0.399503
LC3B	W3	PGE2	well_5	F003	90	Z distribution	-0.52976	-0.60119	0.397163
LC3B	W3	PGE2	well_5	F004	189	Z distribution	-0.44844	-0.48486	0.546629
LC3B	W3	PGE2	well_5	F005	202	Z distribution	-0.45812	-0.44248	0.360624
LC3B	W3	PGE2	well_5	F006	188	Z distribution	-0.30524	-0.34033	0.316521
LC3B	W4	HPI4	well_1	F001	1	Z distribution	-0.29412	-0.29412	NA
LC3B	W4	HPI4	well_1	F002	1	Z distribution	-0.26229	-0.26229	NA
LC3B	W4	HPI4	well_1	F003	1	Z distribution	0.22708	0.22708	NA
LC3B	W4	HPI4	well_1	F005	4	Z distribution	-0.51036	-0.51036	0.068435
LC3B	W4	HPI4	well_2	F005	1	Z distribution	-0.34005	-0.34005	NA

LC3B	W4	HPI4	well_3	F001	1	Z distribution	-0.36165	-0.36165	NA
LC3B	W4	HPI4	well_3	F005	1	Z distribution	0.611224	0.611224	NA
LC3B	W4	HPI4	well_3	F006	7	Z distribution	-0.20287	-0.25953	0.129271
LC3B	W4	HPI4	well_4	F001	3	Z distribution	-0.26656	-0.26656	NA
LC3B	W4	HPI4	well_4	F003	1	Z distribution	-0.32348	-0.32348	NA
LC3B	W4	HPI4	well_4	F004	3	Z distribution	-0.32879	-0.32879	NA
LC3B	W4	PGE2	well_1	F001	105	Z distribution	-0.49168	-0.48566	0.371496
LC3B	W4	PGE2	well_1	F002	100	Z distribution	-0.53543	-0.56628	0.522543
LC3B	W4	PGE2	well_1	F003	224	Z distribution	-0.30564	-0.30321	0.504993
LC3B	W4	PGE2	well_1	F004	187	Z distribution	-0.05743	0.012535	0.680582
LC3B	W4	PGE2	well_1	F005	222	Z distribution	-0.23248	-0.22947	0.716759
LC3B	W4	PGE2	well_1	F006	218	Z distribution	-0.28799	-0.32747	0.621657
LC3B	W4	PGE2	well_2	F001	253	Z distribution	-0.05807	-0.02703	0.580825
LC3B	W4	PGE2	well_2	F002	142	Z distribution	-0.02895	-0.03748	0.528327
LC3B	W4	PGE2	well_2	F003	209	Z distribution	-0.1979	-0.194	0.438154
LC3B	W4	PGE2	well_2	F004	216	Z distribution	-0.33945	-0.31805	0.517714
LC3B	W4	PGE2	well_2	F005	248	Z distribution	-0.01323	-0.01867	0.588391
LC3B	W4	PGE2	well_2	F006	230	Z distribution	-0.20225	-0.22619	0.535959
LC3B	W4	PGE2	well_3	F001	162	Z distribution	-0.11907	-0.16776	0.532629
LC3B	W4	PGE2	well_3	F002	150	Z distribution	-0.21023	-0.26659	0.529359
LC3B	W4	PGE2	well_3	F003	216	Z distribution	-0.1467	-0.17145	0.59005
LC3B	W4	PGE2	well_3	F004	175	Z distribution	-0.21513	-0.16703	0.473798
LC3B	W4	PGE2	well_3	F005	260	Z distribution	-0.08348	-0.08174	0.690203
LC3B	W4	PGE2	well_3	F006	237	Z distribution	-0.05285	-0.06167	0.555959
LC3B	W4	PGE2	well_4	F001	73	Z distribution	-0.26325	-0.35144	0.52419
LC3B	W4	PGE2	well_4	F002	74	Z distribution	-0.24917	-0.23151	0.454105
LC3B	W4	PGE2	well_4	F003	109	Z distribution	-0.27819	-0.28847	0.45494
LC3B	W4	PGE2	well_4	F004	164	Z distribution	-0.43972	-0.46128	0.501618
LC3B	W4	PGE2	well_4	F005	221	Z distribution	-0.23141	-0.20066	0.584381
LC3B	W4	PGE2	well_4	F006	255	Z distribution	-0.09593	-0.12168	0.56495
LC3B	W4	PGE2	well_5	F001	128	Z distribution	-0.42338	-0.33671	0.574559
LC3B	W4	PGE2	well_5	F002	34	Z distribution	-0.15619	-0.21116	0.540101
LC3B	W4	PGE2	well_5	F003	25	Z distribution	-0.567	-0.55317	0.53713
LC3B	W4	PGE2	well_5	F004	81	Z distribution	-0.37312	-0.43535	0.440367
LC3B	W4	PGE2	well_5	F005	53	Z distribution	-0.33022	-0.45969	0.482193
LC3B	W4	PGE2	well_5	F006	75	Z distribution	-0.27307	-0.26036	0.708424
RAB5	W1	HPI4	well_1	F001	48	Avg. Volume	0.422017	0.428574	0.092972
RAB5	W1	HPI4	well_1	F002	67	Avg. Volume	0.663473	0.640225	0.186192
RAB5	W1	HPI4	well_1	F003	105	Avg. Volume	0.732987	0.752285	0.201647
RAB5	W1	HPI4	well_1	F004	15	Avg. Volume	0.426848	0.482031	0.132706
RAB5	W1	HPI4	well_1	F005	42	Avg. Volume	0.649479	0.622046	0.180845
RAB5	W1	HPI4	well_1	F006	34	Avg. Volume	0.519907	0.47683	0.15204
RAB5	W1	HPI4	well_2	F001	57	Avg. Volume	0.41748	0.426642	0.108201
RAB5	W1	HPI4	well_2	F002	22	Avg. Volume	0.213891	0.199514	0.10021
RAB5	W1	HPI4	well_2	F003	116	Avg. Volume	0.683591	0.658252	0.174284
RAB5	W1	HPI4	well_2	F004	67	Avg. Volume	0.482638	0.467768	0.141473
RAB5	W1	HPI4	well_2	F005	190	Avg. Volume	0.699281	0.678322	0.183367
RAB5	W1	HPI4	well_2	F006	79	Avg. Volume	0.207635	0.199514	0.087494
RAB5	W1	HPI4	well_3	F001	78	Avg. Volume	0.468796	0.437186	0.149522

RAB5	W1	HPI4	well_3	F002	56	Avg. Volume	0.58173	0.580874	0.156196
RAB5	W1	HPI4	well_3	F003	178	Avg. Volume	0.676703	0.652107	0.173673
RAB5	W1	HPI4	well_3	F004	146	Avg. Volume	0.567728	0.565606	0.156497
RAB5	W1	HPI4	well_3	F005	25	Avg. Volume	0.53528	0.469444	0.161713
RAB5	W1	HPI4	well_3	F006	123	Avg. Volume	0.561816	0.527566	0.163138
RAB5	W1	HPI4	well_4	F001	83	Avg. Volume	0.467997	0.474547	0.134792
RAB5	W1	HPI4	well_4	F002	98	Avg. Volume	0.633238	0.636708	0.181902
RAB5	W1	HPI4	well_4	F003	85	Avg. Volume	0.595811	0.579583	0.166084
RAB5	W1	HPI4	well_4	F004	89	Avg. Volume	0.716618	0.687475	0.192164
RAB5	W1	HPI4	well_4	F005	225	Avg. Volume	0.668165	0.649628	0.189759
RAB5	W1	HPI4	well_4	F006	140	Avg. Volume	0.682833	0.675886	0.186608
RAB5	W1	HPI4	well_5	F001	166	Avg. Volume	0.518381	0.47791	0.167695
RAB5	W1	HPI4	well_5	F002	135	Avg. Volume	0.578463	0.563333	0.144242
RAB5	W1	HPI4	well_5	F003	157	Avg. Volume	0.706327	0.684542	0.178589
RAB5	W1	HPI4	well_5	F004	74	Avg. Volume	0.522538	0.515411	0.163665
RAB5	W1	HPI4	well_5	F005	42	Avg. Volume	0.558587	0.528873	0.189186
RAB5	W1	HPI4	well_5	F006	19	Avg. Volume	0.370766	0.351632	0.100603
RAB5	W1	PGE2	well_1	F001	88	Avg. Volume	0.664755	0.613793	0.171708
RAB5	W1	PGE2	well_1	F002	111	Avg. Volume	0.706581	0.689439	0.185556
RAB5	W1	PGE2	well_1	F003	84	Avg. Volume	0.679975	0.6738	0.146998
RAB5	W1	PGE2	well_1	F004	20	Avg. Volume	0.543855	0.50668	0.209584
RAB5	W1	PGE2	well_1	F005	96	Avg. Volume	0.683605	0.659236	0.207679
RAB5	W1	PGE2	well_1	F006	30	Avg. Volume	0.648806	0.639258	0.165298
RAB5	W1	PGE2	well_2	F001	118	Avg. Volume	0.677235	0.659157	0.221188
RAB5	W1	PGE2	well_2	F002	184	Avg. Volume	0.613518	0.594574	0.18833
RAB5	W1	PGE2	well_2	F003	103	Avg. Volume	0.587078	0.565681	0.171229
RAB5	W1	PGE2	well_2	F004	113	Avg. Volume	0.607547	0.589343	0.191129
RAB5	W1	PGE2	well_2	F005	97	Avg. Volume	0.535351	0.53064	0.153433
RAB5	W1	PGE2	well_2	F006	70	Avg. Volume	0.531788	0.53941	0.158005
RAB5	W1	PGE2	well_3	F001	22	Avg. Volume	0.251713	0.226898	0.084372
RAB5	W1	PGE2	well_3	F002	71	Avg. Volume	0.594636	0.602114	0.179933
RAB5	W1	PGE2	well_3	F003	57	Avg. Volume	0.582315	0.573661	0.168725
RAB5	W1	PGE2	well_3	F004	89	Avg. Volume	0.59985	0.605299	0.136845
RAB5	W1	PGE2	well_3	F005	51	Avg. Volume	0.556294	0.537727	0.145792
RAB5	W1	PGE2	well_3	F006	48	Avg. Volume	0.480787	0.442944	0.172273
RAB5	W1	PGE2	well_4	F001	49	Avg. Volume	0.52184	0.49124	0.165417
RAB5	W1	PGE2	well_4	F002	67	Avg. Volume	0.559817	0.558392	0.184125
RAB5	W1	PGE2	well_4	F003	29	Avg. Volume	0.622773	0.602287	0.14568
RAB5	W1	PGE2	well_4	F004	64	Avg. Volume	0.671152	0.645184	0.161002
RAB5	W1	PGE2	well_4	F005	41	Avg. Volume	0.594043	0.543693	0.199732
RAB5	W1	PGE2	well_4	F006	71	Avg. Volume	0.539085	0.510521	0.179678
RAB5	W1	PGE2	well_5	F001	8	Avg. Volume	0.453458	0.428633	0.135222
RAB5	W1	PGE2	well_5	F002	91	Avg. Volume	0.561288	0.566534	0.167158
RAB5	W1	PGE2	well_5	F003	72	Avg. Volume	0.61313	0.604521	0.158122
RAB5	W1	PGE2	well_5	F004	95	Avg. Volume	0.629383	0.580546	0.190646
RAB5	W1	PGE2	well_5	F005	56	Avg. Volume	0.563169	0.572936	0.126778
RAB5	W1	PGE2	well_5	F006	167	Avg. Volume	0.318266	0.306944	0.108991
RAB5	W2	HPI4	well_1	F004	19	Avg. Volume	0.529718	0.393925	0.278996
RAB5	W2	HPI4	well_1	F005	26	Avg. Volume	0.835182	0.784999	0.261966

RAB5	W2	HPI4	well_1	F006	11	Avg. Volume	0.793224	0.544208	0.42811
RAB5	W2	HPI4	well_2	F001	1	Avg. Volume	0.733839	0.733839	NA
RAB5	W2	HPI4	well_2	F004	3	Avg. Volume	0.975164	0.975164	NA
RAB5	W2	HPI4	well_3	F001	1	Avg. Volume	0.105625	0.105625	NA
RAB5	W2	HPI4	well_3	F002	8	Avg. Volume	0.401199	0.378502	0.126989
RAB5	W2	HPI4	well_3	F003	4	Avg. Volume	3.908125	3.908125	0.492389
RAB5	W2	HPI4	well_3	F004	12	Avg. Volume	0.471762	0.462222	0.221383
RAB5	W2	HPI4	well_3	F005	72	Avg. Volume	0.478591	0.446728	0.2063
RAB5	W2	HPI4	well_3	F006	29	Avg. Volume	0.653098	0.662438	0.289471
RAB5	W2	HPI4	well_4	F001	3	Avg. Volume	0.457708	0.457708	NA
RAB5	W2	HPI4	well_4	F002	10	Avg. Volume	0.560803	0.413812	0.461393
RAB5	W2	HPI4	well_4	F004	7	Avg. Volume	1.63062	1.790066	0.386682
RAB5	W2	HPI4	well_4	F005	56	Avg. Volume	0.85553	0.837773	0.247349
RAB5	W2	HPI4	well_4	F006	7	Avg. Volume	0.539647	0.28108	0.516671
RAB5	W2	HPI4	well_5	F001	5	Avg. Volume	1.259459	1.056902	0.544754
RAB5	W2	HPI4	well_5	F002	3	Avg. Volume	0.329157	0.329157	NA
RAB5	W2	HPI4	well_5	F004	19	Avg. Volume	1.133994	1.02691	0.391123
RAB5	W2	HPI4	well_5	F005	52	Avg. Volume	0.400263	0.386434	0.109315
RAB5	W2	HPI4	well_5	F006	13	Avg. Volume	0.526831	0.509434	0.206501
RAB5	W2	PGE2	well_1	F001	181	Avg. Volume	0.542881	0.542678	0.160224
RAB5	W2	PGE2	well_1	F002	107	Avg. Volume	0.562836	0.563333	0.167786
RAB5	W2	PGE2	well_1	F003	82	Avg. Volume	0.586837	0.56423	0.161143
RAB5	W2	PGE2	well_1	F004	184	Avg. Volume	0.651165	0.642345	0.159421
RAB5	W2	PGE2	well_1	F005	177	Avg. Volume	0.548864	0.532883	0.168374
RAB5	W2	PGE2	well_1	F006	194	Avg. Volume	0.551805	0.54324	0.145326
RAB5	W2	PGE2	well_2	F001	77	Avg. Volume	0.621696	0.610278	0.162829
RAB5	W2	PGE2	well_2	F002	114	Avg. Volume	0.568279	0.558916	0.146192
RAB5	W2	PGE2	well_2	F003	147	Avg. Volume	0.544431	0.522961	0.16453
RAB5	W2	PGE2	well_2	F004	58	Avg. Volume	0.732213	0.719794	0.189372
RAB5	W2	PGE2	well_2	F005	155	Avg. Volume	0.521032	0.503186	0.156565
RAB5	W2	PGE2	well_2	F006	132	Avg. Volume	0.572835	0.54517	0.182476
RAB5	W2	PGE2	well_3	F001	43	Avg. Volume	0.631301	0.636922	0.124063
RAB5	W2	PGE2	well_3	F002	108	Avg. Volume	0.548467	0.52371	0.151324
RAB5	W2	PGE2	well_3	F003	19	Avg. Volume	0.641892	0.663843	0.199043
RAB5	W2	PGE2	well_3	F004	69	Avg. Volume	0.587563	0.574287	0.188351
RAB5	W2	PGE2	well_3	F005	32	Avg. Volume	0.527619	0.536683	0.158316
RAB5	W2	PGE2	well_3	F006	72	Avg. Volume	0.585573	0.58231	0.174448
RAB5	W2	PGE2	well_4	F001	20	Avg. Volume	0.282123	0.281667	0.127555
RAB5	W2	PGE2	well_4	F002	19	Avg. Volume	0.608429	0.607404	0.139707
RAB5	W2	PGE2	well_4	F003	37	Avg. Volume	0.580754	0.581471	0.149871
RAB5	W2	PGE2	well_4	F004	101	Avg. Volume	0.576253	0.546218	0.17409
RAB5	W2	PGE2	well_4	F005	61	Avg. Volume	0.628309	0.606231	0.153374
RAB5	W2	PGE2	well_4	F006	39	Avg. Volume	0.407391	0.411382	0.112388
RAB5	W2	PGE2	well_5	F001	21	Avg. Volume	0.516653	0.535029	0.141663
RAB5	W2	PGE2	well_5	F002	70	Avg. Volume	0.44551	0.424847	0.147987
RAB5	W2	PGE2	well_5	F003	30	Avg. Volume	0.586041	0.52015	0.185066
RAB5	W2	PGE2	well_5	F004	47	Avg. Volume	0.654414	0.657222	0.151999
RAB5	W2	PGE2	well_5	F005	33	Avg. Volume	0.483971	0.479504	0.093652
RAB5	W2	PGE2	well_5	F006	41	Avg. Volume	0.526779	0.488516	0.166828

RAB5	W3	HPI4	well_1	F001	18	Avg. Volume	0.632945	0.63375	0.456163
RAB5	W3	HPI4	well_1	F002	4	Avg. Volume	0.688163	0.688163	0.454164
RAB5	W3	HPI4	well_1	F004	4	Avg. Volume	0.707644	0.707644	0.336865
RAB5	W3	HPI4	well_1	F005	3	Avg. Volume	0.477269	0.477269	NA
RAB5	W3	HPI4	well_1	F006	1	Avg. Volume	0.32221	0.32221	NA
RAB5	W3	HPI4	well_2	F001	14	Avg. Volume	0.911206	0.74434	0.457724
RAB5	W3	HPI4	well_2	F003	3	Avg. Volume	1.211528	1.211528	NA
RAB5	W3	HPI4	well_2	F004	4	Avg. Volume	0.264649	0.264649	0.034025
RAB5	W3	HPI4	well_2	F005	12	Avg. Volume	0.536519	0.517041	0.332111
RAB5	W3	HPI4	well_3	F002	1	Avg. Volume	1.360771	1.360771	NA
RAB5	W3	HPI4	well_3	F004	8	Avg. Volume	0.599778	0.430194	0.48547
RAB5	W3	HPI4	well_3	F006	1	Avg. Volume	0.22131	0.22131	NA
RAB5	W3	HPI4	well_4	F001	4	Avg. Volume	0.529592	0.529592	0.417009
RAB5	W3	HPI4	well_4	F002	1	Avg. Volume	0.312963	0.312963	NA
RAB5	W3	HPI4	well_4	F004	4	Avg. Volume	0.741052	0.741052	0.417305
RAB5	W3	HPI4	well_4	F005	4	Avg. Volume	0.350319	0.350319	0.120521
RAB5	W3	HPI4	well_5	F003	1	Avg. Volume	2.462907	2.462907	NA
RAB5	W3	HPI4	well_5	F004	7	Avg. Volume	0.393884	0.396792	0.130371
RAB5	W3	HPI4	well_5	F006	5	Avg. Volume	0.97401	0.906028	0.358258
RAB5	W3	PGE2	well_1	F001	164	Avg. Volume	0.524477	0.521627	0.111613
RAB5	W3	PGE2	well_1	F002	178	Avg. Volume	0.48739	0.445623	0.172701
RAB5	W3	PGE2	well_1	F003	197	Avg. Volume	0.516822	0.494174	0.141044
RAB5	W3	PGE2	well_1	F004	73	Avg. Volume	0.474821	0.46823	0.114417
RAB5	W3	PGE2	well_1	F005	214	Avg. Volume	0.537118	0.524699	0.134165
RAB5	W3	PGE2	well_1	F006	233	Avg. Volume	0.610986	0.594994	0.169021
RAB5	W3	PGE2	well_2	F001	234	Avg. Volume	0.472562	0.4494	0.145783
RAB5	W3	PGE2	well_2	F002	277	Avg. Volume	0.509684	0.489005	0.174854
RAB5	W3	PGE2	well_2	F003	223	Avg. Volume	0.531876	0.500741	0.171164
RAB5	W3	PGE2	well_2	F004	280	Avg. Volume	0.440586	0.404049	0.190122
RAB5	W3	PGE2	well_2	F005	285	Avg. Volume	0.537966	0.521157	0.146152
RAB5	W3	PGE2	well_2	F006	302	Avg. Volume	0.611952	0.591641	0.157832
RAB5	W3	PGE2	well_3	F001	173	Avg. Volume	0.486041	0.482857	0.145206
RAB5	W3	PGE2	well_3	F002	226	Avg. Volume	0.398807	0.353993	0.18311
RAB5	W3	PGE2	well_3	F003	243	Avg. Volume	0.494575	0.475846	0.140366
RAB5	W3	PGE2	well_3	F004	194	Avg. Volume	0.553139	0.541087	0.143991
RAB5	W3	PGE2	well_3	F005	230	Avg. Volume	0.411414	0.35722	0.168867
RAB5	W3	PGE2	well_3	F006	259	Avg. Volume	0.511823	0.491515	0.149574
RAB5	W3	PGE2	well_4	F001	185	Avg. Volume	0.368554	0.360885	0.131205
RAB5	W3	PGE2	well_4	F002	158	Avg. Volume	0.484567	0.384292	0.288696
RAB5	W3	PGE2	well_4	F003	211	Avg. Volume	0.503683	0.475688	0.193109
RAB5	W3	PGE2	well_4	F004	161	Avg. Volume	0.458579	0.458714	0.129061
RAB5	W3	PGE2	well_4	F005	247	Avg. Volume	0.40786	0.375556	0.148631
RAB5	W3	PGE2	well_4	F006	217	Avg. Volume	0.447328	0.430113	0.127095
RAB5	W3	PGE2	well_5	F001	91	Avg. Volume	0.466833	0.399028	0.256596
RAB5	W3	PGE2	well_5	F002	123	Avg. Volume	0.479836	0.44858	0.188049
RAB5	W3	PGE2	well_5	F003	134	Avg. Volume	0.448125	0.444406	0.13885
RAB5	W3	PGE2	well_5	F004	167	Avg. Volume	0.417862	0.353201	0.234302
RAB5	W3	PGE2	well_5	F005	134	Avg. Volume	0.4267	0.403722	0.14855
RAB5	W3	PGE2	well_5	F006	154	Avg. Volume	0.395175	0.352665	0.163663

RAB5	W4	HPI4	well_1	F002	7	Avg. Volume	0.456167	0.466959	0.131946
RAB5	W4	HPI4	well_1	F004	6	Avg. Volume	1.447772	1.601576	0.347643
RAB5	W4	HPI4	well_1	F005	9	Avg. Volume	0.404253	0.321199	0.233778
RAB5	W4	HPI4	well_1	F006	21	Avg. Volume	0.544255	0.467358	0.278561
RAB5	W4	HPI4	well_2	F001	5	Avg. Volume	0.444521	0.445972	0.104358
RAB5	W4	HPI4	well_2	F004	5	Avg. Volume	0.691277	0.704878	0.119378
RAB5	W4	HPI4	well_2	F005	14	Avg. Volume	0.562788	0.569095	0.154151
RAB5	W4	HPI4	well_2	F006	7	Avg. Volume	0.528166	0.563333	0.213774
RAB5	W4	HPI4	well_3	F001	4	Avg. Volume	0.341014	0.341014	0.005092
RAB5	W4	HPI4	well_3	F002	1	Avg. Volume	0.664706	0.664706	NA
RAB5	W4	HPI4	well_3	F003	6	Avg. Volume	0.677968	0.695731	0.061199
RAB5	W4	HPI4	well_3	F004	3	Avg. Volume	0.77526	0.77526	NA
RAB5	W4	HPI4	well_3	F005	6	Avg. Volume	0.315099	0.308248	0.04945
RAB5	W4	HPI4	well_3	F006	4	Avg. Volume	0.417267	0.417267	0.033761
RAB5	W4	HPI4	well_4	F001	10	Avg. Volume	0.398967	0.337609	0.275973
RAB5	W4	HPI4	well_4	F002	1	Avg. Volume	0.440556	0.440556	NA
RAB5	W4	HPI4	well_4	F003	5	Avg. Volume	0.432326	0.411938	0.122096
RAB5	W4	HPI4	well_4	F004	26	Avg. Volume	0.465298	0.391815	0.203336
RAB5	W4	HPI4	well_4	F005	13	Avg. Volume	0.310779	0.322398	0.071613
RAB5	W4	HPI4	well_4	F006	15	Avg. Volume	0.912754	0.975875	0.279903
RAB5	W4	HPI4	well_5	F001	14	Avg. Volume	0.540887	0.489054	0.218901
RAB5	W4	HPI4	well_5	F003	7	Avg. Volume	0.537676	0.602706	0.237669
RAB5	W4	HPI4	well_5	F004	19	Avg. Volume	0.49436	0.495525	0.123634
RAB5	W4	HPI4	well_5	F005	25	Avg. Volume	0.303868	0.299923	0.070011
RAB5	W4	PGE2	well_1	F001	29	Avg. Volume	0.570117	0.460056	0.393003
RAB5	W4	PGE2	well_1	F002	125	Avg. Volume	0.376977	0.326806	0.179046
RAB5	W4	PGE2	well_1	F003	68	Avg. Volume	0.794984	0.715903	0.455958
RAB5	W4	PGE2	well_1	F004	90	Avg. Volume	0.404626	0.340934	0.235608
RAB5	W4	PGE2	well_1	F005	29	Avg. Volume	0.354335	0.283914	0.278877
RAB5	W4	PGE2	well_1	F006	11	Avg. Volume	0.616545	0.527343	0.339951
RAB5	W4	PGE2	well_2	F001	95	Avg. Volume	0.475558	0.479562	0.140411
RAB5	W4	PGE2	well_2	F002	48	Avg. Volume	0.439288	0.2873	0.36608
RAB5	W4	PGE2	well_2	F003	99	Avg. Volume	0.523509	0.454914	0.272072
RAB5	W4	PGE2	well_2	F004	68	Avg. Volume	0.507927	0.434677	0.267129
RAB5	W4	PGE2	well_2	F005	106	Avg. Volume	0.478273	0.416691	0.191825
RAB5	W4	PGE2	well_2	F006	53	Avg. Volume	0.322889	0.293403	0.202171
RAB5	W4	PGE2	well_3	F001	160	Avg. Volume	0.525618	0.515076	0.199704
RAB5	W4	PGE2	well_3	F002	225	Avg. Volume	0.719443	0.681183	0.21759
RAB5	W4	PGE2	well_3	F003	309	Avg. Volume	0.543086	0.53346	0.198089
RAB5	W4	PGE2	well_3	F004	243	Avg. Volume	0.643097	0.634985	0.211336
RAB5	W4	PGE2	well_3	F005	181	Avg. Volume	0.498786	0.475057	0.156088
RAB5	W4	PGE2	well_3	F006	207	Avg. Volume	0.394051	0.372948	0.144242
RAB5	W4	PGE2	well_4	F001	49	Avg. Volume	0.528946	0.513722	0.178881
RAB5	W4	PGE2	well_4	F002	144	Avg. Volume	0.633771	0.635364	0.201132
RAB5	W4	PGE2	well_4	F003	190	Avg. Volume	0.560704	0.557127	0.176654
RAB5	W4	PGE2	well_4	F004	172	Avg. Volume	0.567711	0.549776	0.165485
RAB5	W4	PGE2	well_4	F005	308	Avg. Volume	0.517382	0.494754	0.185419
RAB5	W4	PGE2	well_4	F006	238	Avg. Volume	0.590477	0.562839	0.202415
RAB5	W4	PGE2	well_5	F001	102	Avg. Volume	0.46202	0.450864	0.134222

RAB5	W4	PGE2	well_5	F002	192	Avg. Volume	0.508047	0.478848	0.15951
RAB5	W4	PGE2	well_5	F003	123	Avg. Volume	0.520298	0.460642	0.281976
RAB5	W4	PGE2	well_5	F004	171	Avg. Volume	0.512589	0.464588	0.224161
RAB5	W4	PGE2	well_5	F005	112	Avg. Volume	0.449282	0.342499	0.273324
RAB5	W4	PGE2	well_5	F006	134	Avg. Volume	0.44528	0.398028	0.221798
RAB5	W1	HPI4	well_1	F001	48	Count/cell	20.23256	19	9.458776
RAB5	W1	HPI4	well_1	F002	67	Count/cell	28.83333	29	12.42355
RAB5	W1	HPI4	well_1	F003	105	Count/cell	32.42105	30	13.87603
RAB5	W1	HPI4	well_1	F004	15	Count/cell	25.92308	25	11.96844
RAB5	W1	HPI4	well_1	F005	42	Count/cell	33.41667	32	11.84754
RAB5	W1	HPI4	well_1	F006	34	Count/cell	35.93548	31	14.50043
RAB5	W1	HPI4	well_2	F001	57	Count/cell	28.19608	25	16.15799
RAB5	W1	HPI4	well_2	F002	22	Count/cell	1.5	1	0.888523
RAB5	W1	HPI4	well_2	F003	116	Count/cell	43.14423	41.5	15.10508
RAB5	W1	HPI4	well_2	F004	67	Count/cell	40.72881	37	19.16429
RAB5	W1	HPI4	well_2	F005	190	Count/cell	48.67647	48.5	17.74327
RAB5	W1	HPI4	well_2	F006	79	Count/cell	5.146667	3	4.267075
RAB5	W1	HPI4	well_3	F001	78	Count/cell	26.14286	23	13.2044
RAB5	W1	HPI4	well_3	F002	56	Count/cell	38.05882	37	13.67393
RAB5	W1	HPI4	well_3	F003	178	Count/cell	41.06832	37	19.21071
RAB5	W1	HPI4	well_3	F004	146	Count/cell	32.2197	31	13.14761
RAB5	W1	HPI4	well_3	F005	25	Count/cell	38.57143	35	18.60799
RAB5	W1	HPI4	well_3	F006	123	Count/cell	32.16514	30	13.66837
RAB5	W1	HPI4	well_4	F001	83	Count/cell	19.08	20	7.045719
RAB5	W1	HPI4	well_4	F002	98	Count/cell	42.09091	40	18.66562
RAB5	W1	HPI4	well_4	F003	85	Count/cell	28.17333	27	9.834266
RAB5	W1	HPI4	well_4	F004	89	Count/cell	43.79747	45	15.49887
RAB5	W1	HPI4	well_4	F005	225	Count/cell	40.38119	39	15.83303
RAB5	W1	HPI4	well_4	F006	140	Count/cell	37.11905	35	13.82844
RAB5	W1	HPI4	well_5	F001	166	Count/cell	24.43243	22	11.12707
RAB5	W1	HPI4	well_5	F002	135	Count/cell	34.72131	32.5	13.92914
RAB5	W1	HPI4	well_5	F003	157	Count/cell	44.29078	42	16.13627
RAB5	W1	HPI4	well_5	F004	74	Count/cell	38.50746	32	22.64641
RAB5	W1	HPI4	well_5	F005	42	Count/cell	39.69444	35.5	21.2345
RAB5	W1	HPI4	well_5	F006	19	Count/cell	39.88235	30	24.15803
RAB5	W1	PGE2	well_1	F001	88	Count/cell	39.51282	38	13.76778
RAB5	W1	PGE2	well_1	F002	111	Count/cell	40.15152	39	15.12557
RAB5	W1	PGE2	well_1	F003	84	Count/cell	40.58108	37.5	17.87553
RAB5	W1	PGE2	well_1	F004	20	Count/cell	34.61111	30.5	18.56353
RAB5	W1	PGE2	well_1	F005	96	Count/cell	34.89535	30.5	14.3912
RAB5	W1	PGE2	well_1	F006	30	Count/cell	41.65385	40.5	13.27537
RAB5	W1	PGE2	well_2	F001	118	Count/cell	28.06542	27	10.19829
RAB5	W1	PGE2	well_2	F002	184	Count/cell	34.64286	34	14.72392
RAB5	W1	PGE2	well_2	F003	103	Count/cell	34.25275	34	12.45757
RAB5	W1	PGE2	well_2	F004	113	Count/cell	26.98095	27	11.30264
RAB5	W1	PGE2	well_2	F005	97	Count/cell	28.51724	26	11.76006
RAB5	W1	PGE2	well_2	F006	70	Count/cell	35.87097	34	15.815
RAB5	W1	PGE2	well_3	F001	22	Count/cell	12.5	12	5.501337
RAB5	W1	PGE2	well_3	F002	71	Count/cell	36.77778	36	16.08891

RAB5	W1	PGE2	well_3	F003	57	Count/cell	45.11765	38	23.34236
RAB5	W1	PGE2	well_3	F004	89	Count/cell	30.7375	29	12.0922
RAB5	W1	PGE2	well_3	F005	51	Count/cell	35.28261	35.5	11.45554
RAB5	W1	PGE2	well_3	F006	48	Count/cell	41.61905	34.5	26.02715
RAB5	W1	PGE2	well_4	F001	49	Count/cell	32.06977	29	14.29518
RAB5	W1	PGE2	well_4	F002	67	Count/cell	40.41667	36.5	19.39797
RAB5	W1	PGE2	well_4	F003	29	Count/cell	41.34615	40.5	16.53104
RAB5	W1	PGE2	well_4	F004	64	Count/cell	47	44	19.75384
RAB5	W1	PGE2	well_4	F005	41	Count/cell	35.54054	37	18.37056
RAB5	W1	PGE2	well_4	F006	71	Count/cell	29.20635	28	12.48503
RAB5	W1	PGE2	well_5	F001	8	Count/cell	42	44	14.77836
RAB5	W1	PGE2	well_5	F002	91	Count/cell	35.09756	31.5	16.09893
RAB5	W1	PGE2	well_5	F003	72	Count/cell	39.35938	37.5	17.35472
RAB5	W1	PGE2	well_5	F004	95	Count/cell	37.06977	36	15.13375
RAB5	W1	PGE2	well_5	F005	56	Count/cell	29.76	28.5	10.94058
RAB5	W1	PGE2	well_5	F006	167	Count/cell	8.882353	8	4.653721
RAB5	W2	HPI4	well_1	F004	19	Count/cell	15.11111	14	8.581025
RAB5	W2	HPI4	well_1	F005	26	Count/cell	23.72727	23	5.945641
RAB5	W2	HPI4	well_1	F006	11	Count/cell	15.22222	14	5.380004
RAB5	W2	HPI4	well_2	F001	1	Count/cell	53	53	NA
RAB5	W2	HPI4	well_2	F004	3	Count/cell	11	11	NA
RAB5	W2	HPI4	well_3	F001	1	Count/cell	6	6	NA
RAB5	W2	HPI4	well_3	F002	8	Count/cell	22.16667	15	16.63029
RAB5	W2	HPI4	well_3	F003	4	Count/cell	4.666667	3	2.886751
RAB5	W2	HPI4	well_3	F004	12	Count/cell	15.4	14.5	5.758086
RAB5	W2	HPI4	well_3	F005	72	Count/cell	14.76812	14	7.710087
RAB5	W2	HPI4	well_3	F006	29	Count/cell	15.92	15	5.251349
RAB5	W2	HPI4	well_4	F001	3	Count/cell	9	9	NA
RAB5	W2	HPI4	well_4	F002	10	Count/cell	11.625	9	7.28869
RAB5	W2	HPI4	well_4	F004	7	Count/cell	27	27	8.746428
RAB5	W2	HPI4	well_4	F005	56	Count/cell	25.68	25	6.783654
RAB5	W2	HPI4	well_4	F006	7	Count/cell	22.4	22	7.700649
RAB5	W2	HPI4	well_5	F001	5	Count/cell	19	18	5.567764
RAB5	W2	HPI4	well_5	F002	3	Count/cell	43	43	NA
RAB5	W2	HPI4	well_5	F004	19	Count/cell	22.94118	21	8.496106
RAB5	W2	HPI4	well_5	F005	52	Count/cell	19.12766	18	6.595839
RAB5	W2	HPI4	well_5	F006	13	Count/cell	25.36364	26	8.127395
RAB5	W2	PGE2	well_1	F001	181	Count/cell	26.41818	25	10.13511
RAB5	W2	PGE2	well_1	F002	107	Count/cell	28.11458	28	9.382929
RAB5	W2	PGE2	well_1	F003	82	Count/cell	31.23611	31	10.57168
RAB5	W2	PGE2	well_1	F004	184	Count/cell	31.19277	31	9.28532
RAB5	W2	PGE2	well_1	F005	177	Count/cell	21.74534	21	7.326561
RAB5	W2	PGE2	well_1	F006	194	Count/cell	28.79429	28	10.35258
RAB5	W2	PGE2	well_2	F001	77	Count/cell	31.08571	31	8.601892
RAB5	W2	PGE2	well_2	F002	114	Count/cell	28.82353	28	10.44402
RAB5	W2	PGE2	well_2	F003	147	Count/cell	24.31061	22.5	9.129378
RAB5	W2	PGE2	well_2	F004	58	Count/cell	32.90385	30	12.1013
RAB5	W2	PGE2	well_2	F005	155	Count/cell	30.51429	29	11.80567
RAB5	W2	PGE2	well_2	F006	132	Count/cell	25.82353	26	7.855309

RAB5	W2	PGE2	well_3	F001	43	Count/cell	33.43243	33	9.973689
RAB5	W2	PGE2	well_3	F002	108	Count/cell	25.14433	26	9.697497
RAB5	W2	PGE2	well_3	F003	19	Count/cell	26.88235	24	9.013062
RAB5	W2	PGE2	well_3	F004	69	Count/cell	28.51613	27.5	11.46876
RAB5	W2	PGE2	well_3	F005	32	Count/cell	26.48276	26	9.952102
RAB5	W2	PGE2	well_3	F006	72	Count/cell	22.28125	22.5	7.198917
RAB5	W2	PGE2	well_4	F001	20	Count/cell	3.222222	3	1.114374
RAB5	W2	PGE2	well_4	F002	19	Count/cell	26.41176	29	8.261801
RAB5	W2	PGE2	well_4	F003	37	Count/cell	23.45455	22	6.026457
RAB5	W2	PGE2	well_4	F004	101	Count/cell	29.33696	29	12.04869
RAB5	W2	PGE2	well_4	F005	61	Count/cell	29.96429	28.5	13.09818
RAB5	W2	PGE2	well_4	F006	39	Count/cell	19.41667	19	8.21975
RAB5	W2	PGE2	well_5	F001	21	Count/cell	23.84211	24	5.993174
RAB5	W2	PGE2	well_5	F002	70	Count/cell	14.90323	13.5	5.262642
RAB5	W2	PGE2	well_5	F003	30	Count/cell	24.23077	22	7.747555
RAB5	W2	PGE2	well_5	F004	47	Count/cell	29	27	8.163945
RAB5	W2	PGE2	well_5	F005	33	Count/cell	19.2069	19	4.270993
RAB5	W2	PGE2	well_5	F006	41	Count/cell	26.07895	26	8.575205
RAB5	W3	HPI4	well_1	F001	18	Count/cell	8.470588	10	4.913217
RAB5	W3	HPI4	well_1	F002	4	Count/cell	7	7	2.828427
RAB5	W3	HPI4	well_1	F004	4	Count/cell	9.5	9.5	7.778175
RAB5	W3	HPI4	well_1	F005	3	Count/cell	3	3	NA
RAB5	W3	HPI4	well_1	F006	1	Count/cell	11	11	NA
RAB5	W3	HPI4	well_2	F001	14	Count/cell	11.07692	12	3.839738
RAB5	W3	HPI4	well_2	F003	3	Count/cell	10	10	NA
RAB5	W3	HPI4	well_2	F004	4	Count/cell	5.333333	4	2.309401
RAB5	W3	HPI4	well_2	F005	12	Count/cell	14.8	8.5	12.42578
RAB5	W3	HPI4	well_3	F002	1	Count/cell	76	76	NA
RAB5	W3	HPI4	well_3	F004	8	Count/cell	7.833333	8.5	2.401388
RAB5	W3	HPI4	well_3	F006	1	Count/cell	7	7	NA
RAB5	W3	HPI4	well_4	F001	4	Count/cell	8	8	4.242641
RAB5	W3	HPI4	well_4	F002	1	Count/cell	3	3	NA
RAB5	W3	HPI4	well_4	F004	4	Count/cell	5	5	2.828427
RAB5	W3	HPI4	well_4	F005	4	Count/cell	17	17	0
RAB5	W3	HPI4	well_5	F003	1	Count/cell	14	14	NA
RAB5	W3	HPI4	well_5	F004	7	Count/cell	14.6	14	6.188699
RAB5	W3	HPI4	well_5	F006	5	Count/cell	8.666667	9	1.527525
RAB5	W3	PGE2	well_1	F001	164	Count/cell	48.64384	50	15.97444
RAB5	W3	PGE2	well_1	F002	178	Count/cell	33.54601	34	12.93106
RAB5	W3	PGE2	well_1	F003	197	Count/cell	37.96703	37	14.02126
RAB5	W3	PGE2	well_1	F004	73	Count/cell	42.30769	39	15.34288
RAB5	W3	PGE2	well_1	F005	214	Count/cell	47.16495	45	15.15801
RAB5	W3	PGE2	well_1	F006	233	Count/cell	54.4717	53	17.30118
RAB5	W3	PGE2	well_2	F001	234	Count/cell	34.24074	33	12.00494
RAB5	W3	PGE2	well_2	F002	277	Count/cell	37.00797	36	13.53927
RAB5	W3	PGE2	well_2	F003	223	Count/cell	32.87562	32	9.965915
RAB5	W3	PGE2	well_2	F004	280	Count/cell	26.76563	26	11.35176
RAB5	W3	PGE2	well_2	F005	285	Count/cell	47.27907	47	14.93513
RAB5	W3	PGE2	well_2	F006	302	Count/cell	44.22794	44	13.51914

RAB5	W3	PGE2	well_3	F001	173	Count/cell	45.45161	43	16.64066
RAB5	W3	PGE2	well_3	F002	226	Count/cell	23.1068	23	8.092047
RAB5	W3	PGE2	well_3	F003	243	Count/cell	39.10092	37	15.06327
RAB5	W3	PGE2	well_3	F004	194	Count/cell	57.01705	57	19.88466
RAB5	W3	PGE2	well_3	F005	230	Count/cell	25.53846	25	8.654867
RAB5	W3	PGE2	well_3	F006	259	Count/cell	47.94017	48	16.80935
RAB5	W3	PGE2	well_4	F001	185	Count/cell	21.73054	20	9.934385
RAB5	W3	PGE2	well_4	F002	158	Count/cell	19.11806	18	8.885044
RAB5	W3	PGE2	well_4	F003	211	Count/cell	28.49738	28	10.49505
RAB5	W3	PGE2	well_4	F004	161	Count/cell	36.19178	36.5	12.56614
RAB5	W3	PGE2	well_4	F005	247	Count/cell	23.25991	23	9.091391
RAB5	W3	PGE2	well_4	F006	217	Count/cell	36.14359	36	12.85285
RAB5	W3	PGE2	well_5	F001	91	Count/cell	16.91358	16	7.096121
RAB5	W3	PGE2	well_5	F002	123	Count/cell	26.3578	26	11.98033
RAB5	W3	PGE2	well_5	F003	134	Count/cell	34.29167	31.5	14.64906
RAB5	W3	PGE2	well_5	F004	167	Count/cell	15.22222	15	7.033961
RAB5	W3	PGE2	well_5	F005	134	Count/cell	26.56667	26	8.710791
RAB5	W3	PGE2	well_5	F006	154	Count/cell	21.54676	21	9.542599
RAB5	W4	HPI4	well_1	F002	7	Count/cell	32.8	33	9.782638
RAB5	W4	HPI4	well_1	F004	6	Count/cell	34	33.5	8.75595
RAB5	W4	HPI4	well_1	F005	9	Count/cell	19.42857	19	5.287001
RAB5	W4	HPI4	well_1	F006	21	Count/cell	26.05263	27	11.66416
RAB5	W4	HPI4	well_2	F001	5	Count/cell	26.66667	26	7.023769
RAB5	W4	HPI4	well_2	F004	5	Count/cell	27	33	14.93318
RAB5	W4	HPI4	well_2	F005	14	Count/cell	40.25	42	10.4805
RAB5	W4	HPI4	well_2	F006	7	Count/cell	17	18	7.035624
RAB5	W4	HPI4	well_3	F001	4	Count/cell	29	29	9.899495
RAB5	W4	HPI4	well_3	F002	1	Count/cell	69	69	NA
RAB5	W4	HPI4	well_3	F003	6	Count/cell	16	15.5	6.164414
RAB5	W4	HPI4	well_3	F004	3	Count/cell	48	48	NA
RAB5	W4	HPI4	well_3	F005	6	Count/cell	38	38.5	15.55635
RAB5	W4	HPI4	well_3	F006	4	Count/cell	29.5	29.5	6.363961
RAB5	W4	HPI4	well_4	F001	10	Count/cell	8	5	9.287088
RAB5	W4	HPI4	well_4	F002	1	Count/cell	13	13	NA
RAB5	W4	HPI4	well_4	F003	5	Count/cell	21	20	11.53256
RAB5	W4	HPI4	well_4	F004	26	Count/cell	26.95455	24.5	13.46416
RAB5	W4	HPI4	well_4	F005	13	Count/cell	17.27273	19	7.0582
RAB5	W4	HPI4	well_4	F006	15	Count/cell	50	48	16.75808
RAB5	W4	HPI4	well_5	F001	14	Count/cell	24.84615	24	17.88317
RAB5	W4	HPI4	well_5	F003	7	Count/cell	42.8	35	27.53543
RAB5	W4	HPI4	well_5	F004	19	Count/cell	40.70588	35	15.77484
RAB5	W4	HPI4	well_5	F005	25	Count/cell	18.04762	19	6.591481
RAB5	W4	PGE2	well_1	F001	29	Count/cell	15.11538	14.5	7.333904
RAB5	W4	PGE2	well_1	F002	125	Count/cell	9.451327	9	4.884361
RAB5	W4	PGE2	well_1	F003	68	Count/cell	20.62295	20	8.606517
RAB5	W4	PGE2	well_1	F004	90	Count/cell	14.47561	14	7.389057
RAB5	W4	PGE2	well_1	F005	29	Count/cell	17.07692	15.5	10.63926
RAB5	W4	PGE2	well_1	F006	11	Count/cell	16.22222	16	4.994441
RAB5	W4	PGE2	well_2	F001	95	Count/cell	25.4023	24	9.735406

RAB5	W4	PGE2	well_2	F002	48	Count/cell	10.84444	9	6.973833
RAB5	W4	PGE2	well_2	F003	99	Count/cell	15	15	6.204384
RAB5	W4	PGE2	well_2	F004	68	Count/cell	20.88889	21	8.389915
RAB5	W4	PGE2	well_2	F005	106	Count/cell	23.57292	21.5	9.596869
RAB5	W4	PGE2	well_2	F006	53	Count/cell	14.85714	15	7.955606
RAB5	W4	PGE2	well_3	F001	160	Count/cell	25.42069	25	8.756481
RAB5	W4	PGE2	well_3	F002	225	Count/cell	34.3122	33	10.12375
RAB5	W4	PGE2	well_3	F003	309	Count/cell	20.67376	20.5	7.38807
RAB5	W4	PGE2	well_3	F004	243	Count/cell	31.28054	32	10.06312
RAB5	W4	PGE2	well_3	F005	181	Count/cell	32.17683	30	11.36669
RAB5	W4	PGE2	well_3	F006	207	Count/cell	15.20321	15	6.51643
RAB5	W4	PGE2	well_4	F001	49	Count/cell	31.11364	30.5	9.97722
RAB5	W4	PGE2	well_4	F002	144	Count/cell	31.20155	33	9.438435
RAB5	W4	PGE2	well_4	F003	190	Count/cell	28.08824	28	7.609429
RAB5	W4	PGE2	well_4	F004	172	Count/cell	26.50641	27	6.614253
RAB5	W4	PGE2	well_4	F005	308	Count/cell	29.9278	29	10.60294
RAB5	W4	PGE2	well_4	F006	238	Count/cell	29.52093	29	9.761563
RAB5	W4	PGE2	well_5	F001	102	Count/cell	30.71429	31	12.58596
RAB5	W4	PGE2	well_5	F002	192	Count/cell	38.6	38	14.16569
RAB5	W4	PGE2	well_5	F003	123	Count/cell	16.30909	17	5.783154
RAB5	W4	PGE2	well_5	F004	171	Count/cell	23.95484	22	9.766988
RAB5	W4	PGE2	well_5	F005	112	Count/cell	12.72816	12	6.440062
RAB5	W4	PGE2	well_5	F006	134	Count/cell	17.95902	18	7.988005
RAB5	W1	HPI4	well_1	F001	48	X/Y distribution	5.521344	5.361886	0.964396
RAB5	W1	HPI4	well_1	F002	67	X/Y distribution	5.128153	5.100059	1.088488
RAB5	W1	HPI4	well_1	F003	105	X/Y distribution	5.458866	5.224599	1.205306
RAB5	W1	HPI4	well_1	F004	15	X/Y distribution	6.526012	6.545228	0.978799
RAB5	W1	HPI4	well_1	F005	42	X/Y distribution	5.809302	5.807885	0.904022
RAB5	W1	HPI4	well_1	F006	34	X/Y distribution	6.300408	6.005099	1.098782
RAB5	W1	HPI4	well_2	F001	57	X/Y distribution	5.915948	5.72821	1.472697
RAB5	W1	HPI4	well_2	F002	22	X/Y distribution	4.477537	4.289445	1.878729
RAB5	W1	HPI4	well_2	F003	116	X/Y distribution	6.291295	6.210534	1.209619
RAB5	W1	HPI4	well_2	F004	67	X/Y distribution	6.869509	6.830252	1.174599
RAB5	W1	HPI4	well_2	F005	190	X/Y distribution	6.207477	6.167942	1.1008
RAB5	W1	HPI4	well_2	F006	79	X/Y distribution	5.495906	5.229171	1.818029
RAB5	W1	HPI4	well_3	F001	78	X/Y distribution	5.715809	5.56887	1.463884
RAB5	W1	HPI4	well_3	F002	56	X/Y distribution	6.337961	6.340788	1.205531
RAB5	W1	HPI4	well_3	F003	178	X/Y distribution	6.103544	5.912838	1.155805
RAB5	W1	HPI4	well_3	F004	146	X/Y distribution	5.94626	5.866261	1.011412
RAB5	W1	HPI4	well_3	F005	25	X/Y distribution	6.685791	6.380264	1.174177
RAB5	W1	HPI4	well_3	F006	123	X/Y distribution	5.960277	5.8597	1.125834
RAB5	W1	HPI4	well_4	F001	83	X/Y distribution	5.302257	5.264691	1.175348
RAB5	W1	HPI4	well_4	F002	98	X/Y distribution	6.08941	6.028006	1.118652
RAB5	W1	HPI4	well_4	F003	85	X/Y distribution	5.287088	5.209522	1.146947
RAB5	W1	HPI4	well_4	F004	89	X/Y distribution	6.269685	6.247421	0.92313
RAB5	W1	HPI4	well_4	F005	225	X/Y distribution	6.178858	6.086314	1.135938
RAB5	W1	HPI4	well_4	F006	140	X/Y distribution	5.751169	5.579369	1.052465
RAB5	W1	HPI4	well_5	F001	166	X/Y distribution	4.976829	4.881305	1.097363
RAB5	W1	HPI4	well_5	F002	135	X/Y distribution	5.705909	5.602508	1.074854

RAB5	W1	HPI4	well_5	F003	157	X/Y distribution	6.149502	6.073837	1.050775
RAB5	W1	HPI4	well_5	F004	74	X/Y distribution	6.119031	6.209658	1.325432
RAB5	W1	HPI4	well_5	F005	42	X/Y distribution	6.214609	6.31104	1.33214
RAB5	W1	HPI4	well_5	F006	19	X/Y distribution	8.242397	7.87371	2.138704
RAB5	W1	PGE2	well_1	F001	88	X/Y distribution	6.434174	6.333014	1.20481
RAB5	W1	PGE2	well_1	F002	111	X/Y distribution	6.262128	6.243619	1.049213
RAB5	W1	PGE2	well_1	F003	84	X/Y distribution	6.063575	5.853429	1.354766
RAB5	W1	PGE2	well_1	F004	20	X/Y distribution	6.751645	6.385898	1.59566
RAB5	W1	PGE2	well_1	F005	96	X/Y distribution	5.866256	5.784721	1.07593
RAB5	W1	PGE2	well_1	F006	30	X/Y distribution	6.369502	6.351079	1.118266
RAB5	W1	PGE2	well_2	F001	118	X/Y distribution	5.129147	5.073925	0.96795
RAB5	W1	PGE2	well_2	F002	184	X/Y distribution	5.506896	5.521727	0.874275
RAB5	W1	PGE2	well_2	F003	103	X/Y distribution	5.878453	5.851201	0.847775
RAB5	W1	PGE2	well_2	F004	113	X/Y distribution	5.070907	5.056781	0.919246
RAB5	W1	PGE2	well_2	F005	97	X/Y distribution	5.570476	5.490303	1.157547
RAB5	W1	PGE2	well_2	F006	70	X/Y distribution	6.162797	6.214153	1.313254
RAB5	W1	PGE2	well_3	F001	22	X/Y distribution	6.582941	6.401388	1.298015
RAB5	W1	PGE2	well_3	F002	71	X/Y distribution	5.87809	5.926652	1.07761
RAB5	W1	PGE2	well_3	F003	57	X/Y distribution	6.282232	5.83249	1.648849
RAB5	W1	PGE2	well_3	F004	89	X/Y distribution	5.815482	5.796615	1.019432
RAB5	W1	PGE2	well_3	F005	51	X/Y distribution	6.205313	6.208307	1.227001
RAB5	W1	PGE2	well_3	F006	48	X/Y distribution	6.355547	5.845056	1.531678
RAB5	W1	PGE2	well_4	F001	49	X/Y distribution	5.913765	5.507773	1.231777
RAB5	W1	PGE2	well_4	F002	67	X/Y distribution	6.46621	6.118369	1.42461
RAB5	W1	PGE2	well_4	F003	29	X/Y distribution	6.367985	6.421115	1.096408
RAB5	W1	PGE2	well_4	F004	64	X/Y distribution	6.440198	6.157787	1.535136
RAB5	W1	PGE2	well_4	F005	41	X/Y distribution	5.472314	5.4496	1.540054
RAB5	W1	PGE2	well_4	F006	71	X/Y distribution	5.609405	5.749022	1.04848
RAB5	W1	PGE2	well_5	F001	8	X/Y distribution	6.961126	6.631941	1.106761
RAB5	W1	PGE2	well_5	F002	91	X/Y distribution	6.425811	6.370314	1.046523
RAB5	W1	PGE2	well_5	F003	72	X/Y distribution	6.142497	5.696433	1.338879
RAB5	W1	PGE2	well_5	F004	95	X/Y distribution	5.896426	5.763518	1.099479
RAB5	W1	PGE2	well_5	F005	56	X/Y distribution	5.772353	5.598426	0.958775
RAB5	W1	PGE2	well_5	F006	167	X/Y distribution	4.973444	4.976286	1.134131
RAB5	W2	HPI4	well_1	F004	19	X/Y distribution	4.800591	4.771866	1.007245
RAB5	W2	HPI4	well_1	F005	26	X/Y distribution	4.857977	4.689671	0.584696
RAB5	W2	HPI4	well_1	F006	11	X/Y distribution	4.027173	4.003842	0.332885
RAB5	W2	HPI4	well_2	F001	1	X/Y distribution	6.452877	6.452877	NA
RAB5	W2	HPI4	well_2	F004	3	X/Y distribution	4.030255	4.030255	NA
RAB5	W2	HPI4	well_3	F001	1	X/Y distribution	5.695724	5.695724	NA
RAB5	W2	HPI4	well_3	F002	8	X/Y distribution	5.314634	5.315487	1.45966
RAB5	W2	HPI4	well_3	F003	4	X/Y distribution	3.085491	3.085491	0.090402
RAB5	W2	HPI4	well_3	F004	12	X/Y distribution	5.217629	4.843258	1.802049
RAB5	W2	HPI4	well_3	F005	72	X/Y distribution	5.09841	5.024454	0.865069
RAB5	W2	HPI4	well_3	F006	29	X/Y distribution	4.362339	4.354619	0.556392
RAB5	W2	HPI4	well_4	F001	3	X/Y distribution	4.388772	4.388772	NA
RAB5	W2	HPI4	well_4	F002	10	X/Y distribution	4.767807	4.962547	1.00608
RAB5	W2	HPI4	well_4	F004	7	X/Y distribution	4.949047	5.137934	0.724843
RAB5	W2	HPI4	well_4	F005	56	X/Y distribution	4.619852	4.511116	0.622104

RAB5	W2	HPI4	well_4	F006	7	X/Y distribution	5.722382	5.520369	0.670771
RAB5	W2	HPI4	well_5	F001	5	X/Y distribution	3.83223	3.754721	0.705079
RAB5	W2	HPI4	well_5	F002	3	X/Y distribution	8.900343	8.900343	NA
RAB5	W2	HPI4	well_5	F004	19	X/Y distribution	4.399435	4.260479	0.63372
RAB5	W2	HPI4	well_5	F005	52	X/Y distribution	5.319746	5.084024	1.436934
RAB5	W2	HPI4	well_5	F006	13	X/Y distribution	6.57941	6.682588	1.491563
RAB5	W2	PGE2	well_1	F001	181	X/Y distribution	5.613865	5.493859	1.167787
RAB5	W2	PGE2	well_1	F002	107	X/Y distribution	5.132816	5.108646	1.107724
RAB5	W2	PGE2	well_1	F003	82	X/Y distribution	5.828829	5.817473	0.855224
RAB5	W2	PGE2	well_1	F004	184	X/Y distribution	5.777864	5.738961	1.043227
RAB5	W2	PGE2	well_1	F005	177	X/Y distribution	5.22253	5.273705	1.054184
RAB5	W2	PGE2	well_1	F006	194	X/Y distribution	5.257646	5.332224	0.911716
RAB5	W2	PGE2	well_2	F001	77	X/Y distribution	5.29196	5.192376	0.789659
RAB5	W2	PGE2	well_2	F002	114	X/Y distribution	4.939964	4.796847	0.895726
RAB5	W2	PGE2	well_2	F003	147	X/Y distribution	5.088847	5.034915	0.873657
RAB5	W2	PGE2	well_2	F004	58	X/Y distribution	4.866864	4.867375	0.973399
RAB5	W2	PGE2	well_2	F005	155	X/Y distribution	5.991949	5.926923	0.962748
RAB5	W2	PGE2	well_2	F006	132	X/Y distribution	5.069361	5.145937	0.748554
RAB5	W2	PGE2	well_3	F001	43	X/Y distribution	5.643075	5.458329	0.940727
RAB5	W2	PGE2	well_3	F002	108	X/Y distribution	5.278034	5.120155	1.013322
RAB5	W2	PGE2	well_3	F003	19	X/Y distribution	5.112759	5.498448	1.164161
RAB5	W2	PGE2	well_3	F004	69	X/Y distribution	5.721196	5.76032	1.150717
RAB5	W2	PGE2	well_3	F005	32	X/Y distribution	5.071497	5.105514	0.831746
RAB5	W2	PGE2	well_3	F006	72	X/Y distribution	5.07109	5.038997	1.0522
RAB5	W2	PGE2	well_4	F001	20	X/Y distribution	3.489672	3.001651	1.686726
RAB5	W2	PGE2	well_4	F002	19	X/Y distribution	4.802171	4.726932	1.170223
RAB5	W2	PGE2	well_4	F003	37	X/Y distribution	4.564123	4.272032	1.037567
RAB5	W2	PGE2	well_4	F004	101	X/Y distribution	5.302156	5.389917	1.121737
RAB5	W2	PGE2	well_4	F005	61	X/Y distribution	5.381123	5.226034	0.863907
RAB5	W2	PGE2	well_4	F006	39	X/Y distribution	5.426578	5.557571	1.123549
RAB5	W2	PGE2	well_5	F001	21	X/Y distribution	5.03181	5.134441	0.850164
RAB5	W2	PGE2	well_5	F002	70	X/Y distribution	5.417659	5.596076	1.255257
RAB5	W2	PGE2	well_5	F003	30	X/Y distribution	4.668905	4.23795	1.125146
RAB5	W2	PGE2	well_5	F004	47	X/Y distribution	5.235149	5.183371	0.780965
RAB5	W2	PGE2	well_5	F005	33	X/Y distribution	4.762858	4.856594	0.808596
RAB5	W2	PGE2	well_5	F006	41	X/Y distribution	5.389058	5.364097	0.966121
RAB5	W3	HPI4	well_1	F001	18	X/Y distribution	3.848793	3.66556	0.861326
RAB5	W3	HPI4	well_1	F002	4	X/Y distribution	4.116832	4.116832	0.435195
RAB5	W3	HPI4	well_1	F004	4	X/Y distribution	4.349784	4.349784	0.217351
RAB5	W3	HPI4	well_1	F005	3	X/Y distribution	2.600963	2.600963	NA
RAB5	W3	HPI4	well_1	F006	1	X/Y distribution	9.213297	9.213297	NA
RAB5	W3	HPI4	well_2	F001	14	X/Y distribution	3.628188	3.320148	0.48032
RAB5	W3	HPI4	well_2	F003	3	X/Y distribution	3.74365	3.74365	NA
RAB5	W3	HPI4	well_2	F004	4	X/Y distribution	3.952676	3.952676	0.317478
RAB5	W3	HPI4	well_2	F005	12	X/Y distribution	5.860145	5.609027	1.11671
RAB5	W3	HPI4	well_3	F002	1	X/Y distribution	10.61253	10.61253	NA
RAB5	W3	HPI4	well_3	F004	8	X/Y distribution	3.586207	3.603088	0.188868
RAB5	W3	HPI4	well_3	F006	1	X/Y distribution	4.052152	4.052152	NA
RAB5	W3	HPI4	well_4	F001	4	X/Y distribution	4.341467	4.341467	0.157785

RAB5	W3	HPI4	well_4	F002	1	X/Y distribution	5.158416	5.158416	NA
RAB5	W3	HPI4	well_4	F004	4	X/Y distribution	3.857397	3.857397	0.340043
RAB5	W3	HPI4	well_4	F005	4	X/Y distribution	4.551573	4.551573	1.184819
RAB5	W3	HPI4	well_5	F003	1	X/Y distribution	3.453031	3.453031	NA
RAB5	W3	HPI4	well_5	F004	7	X/Y distribution	4.785671	3.837025	2.487999
RAB5	W3	HPI4	well_5	F006	5	X/Y distribution	3.66826	3.685404	0.061406
RAB5	W3	PGE2	well_1	F001	164	X/Y distribution	6.889882	6.885608	1.075719
RAB5	W3	PGE2	well_1	F002	178	X/Y distribution	6.091373	6.150792	1.104708
RAB5	W3	PGE2	well_1	F003	197	X/Y distribution	5.965888	6.03957	1.236637
RAB5	W3	PGE2	well_1	F004	73	X/Y distribution	6.56133	6.209902	1.517862
RAB5	W3	PGE2	well_1	F005	214	X/Y distribution	6.876352	6.81243	1.130736
RAB5	W3	PGE2	well_1	F006	233	X/Y distribution	6.571873	6.486469	0.976471
RAB5	W3	PGE2	well_2	F001	234	X/Y distribution	6.787608	6.612621	1.105172
RAB5	W3	PGE2	well_2	F002	277	X/Y distribution	6.448318	6.480525	1.058693
RAB5	W3	PGE2	well_2	F003	223	X/Y distribution	6.252547	6.272704	0.964895
RAB5	W3	PGE2	well_2	F004	280	X/Y distribution	6.339875	6.447153	1.409403
RAB5	W3	PGE2	well_2	F005	285	X/Y distribution	6.457646	6.393483	0.912415
RAB5	W3	PGE2	well_2	F006	302	X/Y distribution	6.274355	6.228162	0.771281
RAB5	W3	PGE2	well_3	F001	173	X/Y distribution	7.109155	7.025431	1.363905
RAB5	W3	PGE2	well_3	F002	226	X/Y distribution	6.463535	6.450355	1.098181
RAB5	W3	PGE2	well_3	F003	243	X/Y distribution	6.503488	6.373545	0.981426
RAB5	W3	PGE2	well_3	F004	194	X/Y distribution	7.268752	7.296517	1.299061
RAB5	W3	PGE2	well_3	F005	230	X/Y distribution	6.467875	6.436307	1.31063
RAB5	W3	PGE2	well_3	F006	259	X/Y distribution	6.681589	6.719845	0.97595
RAB5	W3	PGE2	well_4	F001	185	X/Y distribution	6.205806	6.146925	1.242309
RAB5	W3	PGE2	well_4	F002	158	X/Y distribution	6.419794	6.339859	1.603062
RAB5	W3	PGE2	well_4	F003	211	X/Y distribution	6.363997	6.256467	1.063449
RAB5	W3	PGE2	well_4	F004	161	X/Y distribution	6.714984	6.963872	1.223349
RAB5	W3	PGE2	well_4	F005	247	X/Y distribution	6.188705	6.223341	0.986139
RAB5	W3	PGE2	well_4	F006	217	X/Y distribution	6.846511	6.811048	1.282977
RAB5	W3	PGE2	well_5	F001	91	X/Y distribution	5.492782	5.439213	1.181132
RAB5	W3	PGE2	well_5	F002	123	X/Y distribution	5.48532	5.45642	1.626902
RAB5	W3	PGE2	well_5	F003	134	X/Y distribution	6.083312	5.974134	1.221229
RAB5	W3	PGE2	well_5	F004	167	X/Y distribution	5.811622	5.924361	1.304892
RAB5	W3	PGE2	well_5	F005	134	X/Y distribution	5.990278	6.117498	1.156761
RAB5	W3	PGE2	well_5	F006	154	X/Y distribution	6.09772	6.094832	1.429557
RAB5	W4	HPI4	well_1	F002	7	X/Y distribution	10.8767	10.42969	0.828317
RAB5	W4	HPI4	well_1	F004	6	X/Y distribution	5.899361	6.102316	1.076266
RAB5	W4	HPI4	well_1	F005	9	X/Y distribution	7.696077	7.734906	1.906727
RAB5	W4	HPI4	well_1	F006	21	X/Y distribution	7.435588	7.322198	1.791875
RAB5	W4	HPI4	well_2	F001	5	X/Y distribution	7.725015	7.840884	0.54354
RAB5	W4	HPI4	well_2	F004	5	X/Y distribution	5.883374	6.484372	1.145555
RAB5	W4	HPI4	well_2	F005	14	X/Y distribution	8.067413	7.913465	1.459141
RAB5	W4	HPI4	well_2	F006	7	X/Y distribution	6.003025	4.503148	2.422087
RAB5	W4	HPI4	well_3	F001	4	X/Y distribution	8.436991	8.436991	0.303905
RAB5	W4	HPI4	well_3	F002	1	X/Y distribution	7.841959	7.841959	NA
RAB5	W4	HPI4	well_3	F003	6	X/Y distribution	9.195716	9.88914	1.890009
RAB5	W4	HPI4	well_3	F004	3	X/Y distribution	8.883272	8.883272	NA
RAB5	W4	HPI4	well_3	F005	6	X/Y distribution	8.791764	8.915291	0.970287

RAB5	W4	HPI4	well_3	F006	4	X/Y distribution	7.686683	7.686683	0.060396
RAB5	W4	HPI4	well_4	F001	10	X/Y distribution	8.955559	9.411535	2.79119
RAB5	W4	HPI4	well_4	F002	1	X/Y distribution	10.98064	10.98064	NA
RAB5	W4	HPI4	well_4	F003	5	X/Y distribution	9.503771	9.597852	0.753405
RAB5	W4	HPI4	well_4	F004	26	X/Y distribution	7.369968	7.566095	1.411544
RAB5	W4	HPI4	well_4	F005	13	X/Y distribution	6.921887	7.331708	1.446214
RAB5	W4	HPI4	well_4	F006	15	X/Y distribution	6.983224	6.518233	1.491467
RAB5	W4	HPI4	well_5	F001	14	X/Y distribution	8.98155	8.844373	1.429935
RAB5	W4	HPI4	well_5	F003	7	X/Y distribution	9.634833	8.965287	1.253396
RAB5	W4	HPI4	well_5	F004	19	X/Y distribution	8.474102	8.753022	1.753266
RAB5	W4	HPI4	well_5	F005	25	X/Y distribution	10.48934	10.6134	1.694276
RAB5	W4	PGE2	well_1	F001	29	X/Y distribution	5.097092	4.753533	0.827238
RAB5	W4	PGE2	well_1	F002	125	X/Y distribution	5.305463	5.159094	1.092172
RAB5	W4	PGE2	well_1	F003	68	X/Y distribution	5.518529	5.282545	1.156867
RAB5	W4	PGE2	well_1	F004	90	X/Y distribution	5.45268	5.279779	1.172902
RAB5	W4	PGE2	well_1	F005	29	X/Y distribution	5.683066	5.424105	2.061832
RAB5	W4	PGE2	well_1	F006	11	X/Y distribution	4.299776	4.366146	1.309512
RAB5	W4	PGE2	well_2	F001	95	X/Y distribution	6.096411	6.034383	0.859263
RAB5	W4	PGE2	well_2	F002	48	X/Y distribution	5.406988	5.700144	1.4024
RAB5	W4	PGE2	well_2	F003	99	X/Y distribution	5.318166	5.576737	1.129204
RAB5	W4	PGE2	well_2	F004	68	X/Y distribution	5.75346	5.642297	1.344825
RAB5	W4	PGE2	well_2	F005	106	X/Y distribution	6.422206	6.35888	1.592713
RAB5	W4	PGE2	well_2	F006	53	X/Y distribution	5.538887	5.492907	1.131097
RAB5	W4	PGE2	well_3	F001	160	X/Y distribution	6.313988	6.369163	0.850275
RAB5	W4	PGE2	well_3	F002	225	X/Y distribution	6.425134	6.410379	0.787143
RAB5	W4	PGE2	well_3	F003	309	X/Y distribution	5.979515	5.971404	0.768548
RAB5	W4	PGE2	well_3	F004	243	X/Y distribution	6.354703	6.332909	0.960313
RAB5	W4	PGE2	well_3	F005	181	X/Y distribution	6.84876	6.790686	1.015756
RAB5	W4	PGE2	well_3	F006	207	X/Y distribution	5.84978	5.746518	1.166507
RAB5	W4	PGE2	well_4	F001	49	X/Y distribution	6.436561	6.151609	0.901531
RAB5	W4	PGE2	well_4	F002	144	X/Y distribution	6.455031	6.391944	0.830336
RAB5	W4	PGE2	well_4	F003	190	X/Y distribution	6.333149	6.323746	0.801846
RAB5	W4	PGE2	well_4	F004	172	X/Y distribution	5.942977	6.043082	0.723709
RAB5	W4	PGE2	well_4	F005	308	X/Y distribution	6.112252	6.023044	0.906007
RAB5	W4	PGE2	well_4	F006	238	X/Y distribution	6.37532	6.264439	1.013963
RAB5	W4	PGE2	well_5	F001	102	X/Y distribution	6.518587	6.22015	1.049427
RAB5	W4	PGE2	well_5	F002	192	X/Y distribution	6.999357	6.894033	1.055284
RAB5	W4	PGE2	well_5	F003	123	X/Y distribution	5.974839	5.974702	1.185777
RAB5	W4	PGE2	well_5	F004	171	X/Y distribution	6.044347	6.02512	1.426496
RAB5	W4	PGE2	well_5	F005	112	X/Y distribution	5.583942	5.589972	1.24664
RAB5	W4	PGE2	well_5	F006	134	X/Y distribution	6.37647	6.54609	1.463092
RAB5	W1	HPI4	well_1	F001	48	Z distribution	-0.07237	-0.07933	0.192861
RAB5	W1	HPI4	well_1	F002	67	Z distribution	-0.14759	-0.11101	0.173583
RAB5	W1	HPI4	well_1	F003	105	Z distribution	-0.20539	-0.21515	0.233553
RAB5	W1	HPI4	well_1	F004	15	Z distribution	-0.28266	-0.2591	0.162819
RAB5	W1	HPI4	well_1	F005	42	Z distribution	-0.22058	-0.18415	0.221521
RAB5	W1	HPI4	well_1	F006	34	Z distribution	-0.13448	-0.07885	0.214701
RAB5	W1	HPI4	well_2	F001	57	Z distribution	0.014639	-0.0165	0.208926
RAB5	W1	HPI4	well_2	F002	22	Z distribution	0.181437	0.227125	0.999821

RAB5	W1	HPI4	well_2	F003	116	Z distribution	-0.25214	-0.18816	0.279783
RAB5	W1	HPI4	well_2	F004	67	Z distribution	-0.21094	-0.114	0.259927
RAB5	W1	HPI4	well_2	F005	190	Z distribution	-0.20732	-0.16077	0.233301
RAB5	W1	HPI4	well_2	F006	79	Z distribution	0.545692	0.488189	0.805786
RAB5	W1	HPI4	well_3	F001	78	Z distribution	-0.09695	-0.11543	0.179452
RAB5	W1	HPI4	well_3	F002	56	Z distribution	-0.14669	-0.10722	0.179164
RAB5	W1	HPI4	well_3	F003	178	Z distribution	-0.12469	-0.11364	0.226887
RAB5	W1	HPI4	well_3	F004	146	Z distribution	-0.09815	-0.09034	0.211125
RAB5	W1	HPI4	well_3	F005	25	Z distribution	-0.15692	-0.17252	0.139194
RAB5	W1	HPI4	well_3	F006	123	Z distribution	-0.17922	-0.09434	0.270861
RAB5	W1	HPI4	well_4	F001	83	Z distribution	-0.10993	-0.1088	0.263641
RAB5	W1	HPI4	well_4	F002	98	Z distribution	-0.29009	-0.27295	0.216176
RAB5	W1	HPI4	well_4	F003	85	Z distribution	-0.18148	-0.16	0.192967
RAB5	W1	HPI4	well_4	F004	89	Z distribution	-0.17526	-0.14806	0.235121
RAB5	W1	HPI4	well_4	F005	225	Z distribution	-0.24145	-0.18831	0.22735
RAB5	W1	HPI4	well_4	F006	140	Z distribution	-0.12334	-0.10813	0.188221
RAB5	W1	HPI4	well_5	F001	166	Z distribution	-0.12336	-0.07541	0.243905
RAB5	W1	HPI4	well_5	F002	135	Z distribution	-0.11342	-0.10718	0.223138
RAB5	W1	HPI4	well_5	F003	157	Z distribution	-0.14207	-0.11285	0.213665
RAB5	W1	HPI4	well_5	F004	74	Z distribution	-0.17713	-0.15298	0.194389
RAB5	W1	HPI4	well_5	F005	42	Z distribution	-0.16651	-0.14444	0.17512
RAB5	W1	HPI4	well_5	F006	19	Z distribution	-0.17381	-0.11427	0.196676
RAB5	W1	PGE2	well_1	F001	88	Z distribution	-0.13579	-0.1031	0.197199
RAB5	W1	PGE2	well_1	F002	111	Z distribution	-0.2271	-0.21113	0.212159
RAB5	W1	PGE2	well_1	F003	84	Z distribution	-0.26971	-0.26615	0.186376
RAB5	W1	PGE2	well_1	F004	20	Z distribution	-0.23852	-0.20937	0.220291
RAB5	W1	PGE2	well_1	F005	96	Z distribution	-0.09315	-0.08125	0.156625
RAB5	W1	PGE2	well_1	F006	30	Z distribution	-0.18034	-0.13423	0.15239
RAB5	W1	PGE2	well_2	F001	118	Z distribution	-0.10983	-0.08311	0.212573
RAB5	W1	PGE2	well_2	F002	184	Z distribution	-0.11074	-0.09718	0.214971
RAB5	W1	PGE2	well_2	F003	103	Z distribution	-0.12271	-0.08181	0.178833
RAB5	W1	PGE2	well_2	F004	113	Z distribution	-0.10604	-0.06945	0.238324
RAB5	W1	PGE2	well_2	F005	97	Z distribution	-0.12791	-0.14802	0.196374
RAB5	W1	PGE2	well_2	F006	70	Z distribution	-0.19235	-0.16678	0.186976
RAB5	W1	PGE2	well_3	F001	22	Z distribution	-0.02969	0.007747	0.212818
RAB5	W1	PGE2	well_3	F002	71	Z distribution	-0.13917	-0.10653	0.223421
RAB5	W1	PGE2	well_3	F003	57	Z distribution	-0.18054	-0.14996	0.198755
RAB5	W1	PGE2	well_3	F004	89	Z distribution	-0.08963	-0.06173	0.221303
RAB5	W1	PGE2	well_3	F005	51	Z distribution	-0.10572	-0.08257	0.166254
RAB5	W1	PGE2	well_3	F006	48	Z distribution	-0.11868	-0.11382	0.184301
RAB5	W1	PGE2	well_4	F001	49	Z distribution	-0.09921	-0.09747	0.1873
RAB5	W1	PGE2	well_4	F002	67	Z distribution	-0.1506	-0.10206	0.195975
RAB5	W1	PGE2	well_4	F003	29	Z distribution	-0.13838	-0.09437	0.179179
RAB5	W1	PGE2	well_4	F004	64	Z distribution	-0.01159	-0.01477	0.149433
RAB5	W1	PGE2	well_4	F005	41	Z distribution	-0.13361	-0.12478	0.182058
RAB5	W1	PGE2	well_4	F006	71	Z distribution	-0.12629	-0.07996	0.225731
RAB5	W1	PGE2	well_5	F001	8	Z distribution	-0.57736	-0.58923	0.144183
RAB5	W1	PGE2	well_5	F002	91	Z distribution	-0.13476	-0.0973	0.189354
RAB5	W1	PGE2	well_5	F003	72	Z distribution	-0.12297	-0.09485	0.189092

RAB5	W1	PGE2	well_5	F004	95	Z distribution	-0.18249	-0.1612	0.230008
RAB5	W1	PGE2	well_5	F005	56	Z distribution	-0.10649	-0.06867	0.222298
RAB5	W1	PGE2	well_5	F006	167	Z distribution	0.314971	0.261936	0.485355
RAB5	W2	HPI4	well_1	F004	19	Z distribution	-0.36992	-0.36304	0.290338
RAB5	W2	HPI4	well_1	F005	26	Z distribution	-0.32883	-0.31182	0.165653
RAB5	W2	HPI4	well_1	F006	11	Z distribution	-0.23525	-0.19687	0.218016
RAB5	W2	HPI4	well_2	F001	1	Z distribution	-0.89856	-0.89856	NA
RAB5	W2	HPI4	well_2	F004	3	Z distribution	0.052382	0.052382	NA
RAB5	W2	HPI4	well_3	F001	1	Z distribution	-0.72422	-0.72422	NA
RAB5	W2	HPI4	well_3	F002	8	Z distribution	-0.40405	-0.41215	0.066269
RAB5	W2	HPI4	well_3	F003	4	Z distribution	-0.20199	-0.20199	0.267533
RAB5	W2	HPI4	well_3	F004	12	Z distribution	-0.25266	-0.24423	0.156259
RAB5	W2	HPI4	well_3	F005	72	Z distribution	-0.35993	-0.32381	0.288588
RAB5	W2	HPI4	well_3	F006	29	Z distribution	-0.25053	-0.2166	0.243255
RAB5	W2	HPI4	well_4	F001	3	Z distribution	-0.8393	-0.8393	NA
RAB5	W2	HPI4	well_4	F002	10	Z distribution	-0.25293	-0.2536	0.167338
RAB5	W2	HPI4	well_4	F004	7	Z distribution	-0.23251	-0.19611	0.118177
RAB5	W2	HPI4	well_4	F005	56	Z distribution	-0.4416	-0.4104	0.32935
RAB5	W2	HPI4	well_4	F006	7	Z distribution	-0.29186	-0.25312	0.119171
RAB5	W2	HPI4	well_5	F001	5	Z distribution	-0.19042	-0.1302	0.155491
RAB5	W2	HPI4	well_5	F002	3	Z distribution	-0.34615	-0.34615	NA
RAB5	W2	HPI4	well_5	F004	19	Z distribution	-0.30081	-0.31762	0.220596
RAB5	W2	HPI4	well_5	F005	52	Z distribution	-0.43892	-0.4127	0.264728
RAB5	W2	HPI4	well_5	F006	13	Z distribution	-0.41321	-0.54446	0.243067
RAB5	W2	PGE2	well_1	F001	181	Z distribution	-0.26404	-0.2545	0.239202
RAB5	W2	PGE2	well_1	F002	107	Z distribution	-0.26604	-0.22617	0.256236
RAB5	W2	PGE2	well_1	F003	82	Z distribution	-0.11703	-0.05922	0.210171
RAB5	W2	PGE2	well_1	F004	184	Z distribution	-0.23001	-0.18536	0.256625
RAB5	W2	PGE2	well_1	F005	177	Z distribution	-0.19363	-0.18997	0.275482
RAB5	W2	PGE2	well_1	F006	194	Z distribution	-0.32701	-0.29881	0.280546
RAB5	W2	PGE2	well_2	F001	77	Z distribution	-0.22347	-0.21882	0.258059
RAB5	W2	PGE2	well_2	F002	114	Z distribution	-0.33361	-0.32382	0.225308
RAB5	W2	PGE2	well_2	F003	147	Z distribution	-0.215	-0.19487	0.297757
RAB5	W2	PGE2	well_2	F004	58	Z distribution	-0.21095	-0.2213	0.217273
RAB5	W2	PGE2	well_2	F005	155	Z distribution	-0.23648	-0.21108	0.235079
RAB5	W2	PGE2	well_2	F006	132	Z distribution	-0.22957	-0.22372	0.267097
RAB5	W2	PGE2	well_3	F001	43	Z distribution	-0.25447	-0.27131	0.165473
RAB5	W2	PGE2	well_3	F002	108	Z distribution	-0.47344	-0.45514	0.324623
RAB5	W2	PGE2	well_3	F003	19	Z distribution	-0.34113	-0.31282	0.214762
RAB5	W2	PGE2	well_3	F004	69	Z distribution	-0.18636	-0.13007	0.179782
RAB5	W2	PGE2	well_3	F005	32	Z distribution	-0.38038	-0.33718	0.274274
RAB5	W2	PGE2	well_3	F006	72	Z distribution	-0.23	-0.24987	0.21835
RAB5	W2	PGE2	well_4	F001	20	Z distribution	-0.08297	-0.01997	0.440271
RAB5	W2	PGE2	well_4	F002	19	Z distribution	-0.26287	-0.21097	0.182574
RAB5	W2	PGE2	well_4	F003	37	Z distribution	-0.2926	-0.23586	0.214144
RAB5	W2	PGE2	well_4	F004	101	Z distribution	-0.1758	-0.15922	0.190393
RAB5	W2	PGE2	well_4	F005	61	Z distribution	-0.24753	-0.21692	0.217187
RAB5	W2	PGE2	well_4	F006	39	Z distribution	-0.30829	-0.25143	0.308417
RAB5	W2	PGE2	well_5	F001	21	Z distribution	-0.39033	-0.39615	0.165761

RAB5	W2	PGE2	well_5	F002	70	Z distribution	-0.03455	-0.0207	0.236125
RAB5	W2	PGE2	well_5	F003	30	Z distribution	-0.35459	-0.37396	0.172702
RAB5	W2	PGE2	well_5	F004	47	Z distribution	-0.20545	-0.19899	0.179313
RAB5	W2	PGE2	well_5	F005	33	Z distribution	-0.26265	-0.2603	0.221505
RAB5	W2	PGE2	well_5	F006	41	Z distribution	-0.19455	-0.15418	0.230585
RAB5	W3	HPI4	well_1	F001	18	Z distribution	-0.47203	-0.32106	0.605237
RAB5	W3	HPI4	well_1	F002	4	Z distribution	0.002404	0.002404	0.092255
RAB5	W3	HPI4	well_1	F004	4	Z distribution	-0.25119	-0.25119	0.27306
RAB5	W3	HPI4	well_1	F005	3	Z distribution	-0.2026	-0.2026	NA
RAB5	W3	HPI4	well_1	F006	1	Z distribution	-0.16243	-0.16243	NA
RAB5	W3	HPI4	well_2	F001	14	Z distribution	-0.43895	-0.38974	0.418378
RAB5	W3	HPI4	well_2	F003	3	Z distribution	-0.01954	-0.01954	NA
RAB5	W3	HPI4	well_2	F004	4	Z distribution	-0.25589	-0.25589	0.293992
RAB5	W3	HPI4	well_2	F005	12	Z distribution	-0.32806	-0.33072	0.207867
RAB5	W3	HPI4	well_3	F002	1	Z distribution	-0.18504	-0.18504	NA
RAB5	W3	HPI4	well_3	F004	8	Z distribution	-0.38502	-0.43122	0.203889
RAB5	W3	HPI4	well_3	F006	1	Z distribution	-0.40231	-0.40231	NA
RAB5	W3	HPI4	well_4	F001	4	Z distribution	-0.64519	-0.64519	0.046535
RAB5	W3	HPI4	well_4	F002	1	Z distribution	-0.34789	-0.34789	NA
RAB5	W3	HPI4	well_4	F004	4	Z distribution	-0.7646	-0.7646	0.668359
RAB5	W3	HPI4	well_4	F005	4	Z distribution	-0.45886	-0.45886	0.127361
RAB5	W3	HPI4	well_5	F003	1	Z distribution	-0.0094	-0.0094	NA
RAB5	W3	HPI4	well_5	F004	7	Z distribution	-0.23825	-0.31107	0.242707
RAB5	W3	HPI4	well_5	F006	5	Z distribution	-0.00673	-0.1809	0.308438
RAB5	W3	PGE2	well_1	F001	164	Z distribution	-0.48839	-0.464	0.333774
RAB5	W3	PGE2	well_1	F002	178	Z distribution	-0.50656	-0.45764	0.376874
RAB5	W3	PGE2	well_1	F003	197	Z distribution	-0.52643	-0.51016	0.365639
RAB5	W3	PGE2	well_1	F004	73	Z distribution	-0.44198	-0.35556	0.360883
RAB5	W3	PGE2	well_1	F005	214	Z distribution	-0.279	-0.21386	0.255812
RAB5	W3	PGE2	well_1	F006	233	Z distribution	-0.5133	-0.50668	0.320592
RAB5	W3	PGE2	well_2	F001	234	Z distribution	-0.36858	-0.28969	0.327397
RAB5	W3	PGE2	well_2	F002	277	Z distribution	-0.38886	-0.32306	0.344037
RAB5	W3	PGE2	well_2	F003	223	Z distribution	-0.52985	-0.52842	0.351301
RAB5	W3	PGE2	well_2	F004	280	Z distribution	-0.32363	-0.24651	0.326346
RAB5	W3	PGE2	well_2	F005	285	Z distribution	-0.4055	-0.33239	0.371825
RAB5	W3	PGE2	well_2	F006	302	Z distribution	-0.57975	-0.59092	0.370388
RAB5	W3	PGE2	well_3	F001	173	Z distribution	-0.47281	-0.39921	0.386244
RAB5	W3	PGE2	well_3	F002	226	Z distribution	-0.48926	-0.42207	0.41522
RAB5	W3	PGE2	well_3	F003	243	Z distribution	-0.2914	-0.23369	0.297607
RAB5	W3	PGE2	well_3	F004	194	Z distribution	-0.3903	-0.33387	0.302518
RAB5	W3	PGE2	well_3	F005	230	Z distribution	-0.52369	-0.51055	0.400642
RAB5	W3	PGE2	well_3	F006	259	Z distribution	-0.56397	-0.51178	0.472427
RAB5	W3	PGE2	well_4	F001	185	Z distribution	-0.42951	-0.4138	0.306349
RAB5	W3	PGE2	well_4	F002	158	Z distribution	-0.41177	-0.34433	0.342373
RAB5	W3	PGE2	well_4	F003	211	Z distribution	-0.46843	-0.40238	0.351353
RAB5	W3	PGE2	well_4	F004	161	Z distribution	-0.5336	-0.47261	0.373638
RAB5	W3	PGE2	well_4	F005	247	Z distribution	-0.5559	-0.50464	0.382987
RAB5	W3	PGE2	well_4	F006	217	Z distribution	-0.54573	-0.51676	0.440912
RAB5	W3	PGE2	well_5	F001	91	Z distribution	-0.38663	-0.38297	0.265811

RAB5	W3	PGE2	well_5	F002	123	Z distribution	-0.58947	-0.55746	0.29102
RAB5	W3	PGE2	well_5	F003	134	Z distribution	-0.67109	-0.62385	0.419196
RAB5	W3	PGE2	well_5	F004	167	Z distribution	-0.38283	-0.29688	0.345054
RAB5	W3	PGE2	well_5	F005	134	Z distribution	-0.63323	-0.59544	0.421075
RAB5	W3	PGE2	well_5	F006	154	Z distribution	-0.48382	-0.47598	0.292333
RAB5	W4	HPI4	well_1	F002	7	Z distribution	-0.2403	-0.17936	0.177113
RAB5	W4	HPI4	well_1	F004	6	Z distribution	-0.20265	-0.236	0.182738
RAB5	W4	HPI4	well_1	F005	9	Z distribution	-0.39394	-0.30787	0.24644
RAB5	W4	HPI4	well_1	F006	21	Z distribution	-0.40083	-0.39527	0.312449
RAB5	W4	HPI4	well_2	F001	5	Z distribution	-0.32009	-0.39716	0.212519
RAB5	W4	HPI4	well_2	F004	5	Z distribution	0.025656	0.02941	0.11516
RAB5	W4	HPI4	well_2	F005	14	Z distribution	-0.19523	-0.18555	0.207541
RAB5	W4	HPI4	well_2	F006	7	Z distribution	-0.38081	-0.28127	0.27499
RAB5	W4	HPI4	well_3	F001	4	Z distribution	-0.23029	-0.23029	0.047036
RAB5	W4	HPI4	well_3	F002	1	Z distribution	-0.13646	-0.13646	NA
RAB5	W4	HPI4	well_3	F003	6	Z distribution	-0.60536	-0.48506	0.359216
RAB5	W4	HPI4	well_3	F004	3	Z distribution	-0.05877	-0.05877	NA
RAB5	W4	HPI4	well_3	F005	6	Z distribution	-0.3425	-0.32159	0.125336
RAB5	W4	HPI4	well_3	F006	4	Z distribution	-0.09656	-0.09656	0.07381
RAB5	W4	HPI4	well_4	F001	10	Z distribution	-0.24841	-0.21438	0.239989
RAB5	W4	HPI4	well_4	F002	1	Z distribution	-0.28642	-0.28642	NA
RAB5	W4	HPI4	well_4	F003	5	Z distribution	-0.31481	-0.29416	0.050569
RAB5	W4	HPI4	well_4	F004	26	Z distribution	-0.0954	-0.08806	0.142958
RAB5	W4	HPI4	well_4	F005	13	Z distribution	-0.3511	-0.43571	0.195261
RAB5	W4	HPI4	well_4	F006	15	Z distribution	-0.28322	-0.16397	0.30976
RAB5	W4	HPI4	well_5	F001	14	Z distribution	-0.22115	-0.19498	0.129839
RAB5	W4	HPI4	well_5	F003	7	Z distribution	-0.20311	-0.1185	0.198284
RAB5	W4	HPI4	well_5	F004	19	Z distribution	-0.12459	-0.07987	0.199908
RAB5	W4	HPI4	well_5	F005	25	Z distribution	-0.1109	-0.02494	0.316943
RAB5	W4	PGE2	well_1	F001	29	Z distribution	-0.34219	-0.35119	0.23793
RAB5	W4	PGE2	well_1	F002	125	Z distribution	-0.44801	-0.33533	0.361854
RAB5	W4	PGE2	well_1	F003	68	Z distribution	-0.40908	-0.37069	0.245113
RAB5	W4	PGE2	well_1	F004	90	Z distribution	-0.49165	-0.42041	0.257808
RAB5	W4	PGE2	well_1	F005	29	Z distribution	-0.60657	-0.55472	0.360654
RAB5	W4	PGE2	well_1	F006	11	Z distribution	-0.60724	-0.37014	0.354466
RAB5	W4	PGE2	well_2	F001	95	Z distribution	-0.24907	-0.20249	0.22258
RAB5	W4	PGE2	well_2	F002	48	Z distribution	-0.75769	-0.81383	0.402912
RAB5	W4	PGE2	well_2	F003	99	Z distribution	-0.40447	-0.35665	0.294779
RAB5	W4	PGE2	well_2	F004	68	Z distribution	-0.63112	-0.48275	0.42926
RAB5	W4	PGE2	well_2	F005	106	Z distribution	-0.31714	-0.2494	0.259478
RAB5	W4	PGE2	well_2	F006	53	Z distribution	-0.62125	-0.56626	0.330626
RAB5	W4	PGE2	well_3	F001	160	Z distribution	-0.76799	-0.7731	0.501818
RAB5	W4	PGE2	well_3	F002	225	Z distribution	-0.83296	-0.83575	0.384045
RAB5	W4	PGE2	well_3	F003	309	Z distribution	-0.64562	-0.66031	0.372579
RAB5	W4	PGE2	well_3	F004	243	Z distribution	-0.53768	-0.48566	0.37745
RAB5	W4	PGE2	well_3	F005	181	Z distribution	-0.22489	-0.18454	0.249615
RAB5	W4	PGE2	well_3	F006	207	Z distribution	-0.92304	-0.93096	0.562263
RAB5	W4	PGE2	well_4	F001	49	Z distribution	-0.80119	-0.86819	0.373822
RAB5	W4	PGE2	well_4	F002	144	Z distribution	-0.85914	-0.92172	0.502704

RAB5	W4	PGE2	well_4	F003	190	Z distribution	-0.73609	-0.69528	0.428749
RAB5	W4	PGE2	well_4	F004	172	Z distribution	-1.00077	-1.02558	0.448537
RAB5	W4	PGE2	well_4	F005	308	Z distribution	-0.36932	-0.3197	0.371472
RAB5	W4	PGE2	well_4	F006	238	Z distribution	-0.77522	-0.80938	0.446271
RAB5	W4	PGE2	well_5	F001	102	Z distribution	-0.26643	-0.2365	0.230943
RAB5	W4	PGE2	well_5	F002	192	Z distribution	-0.39595	-0.329	0.347152
RAB5	W4	PGE2	well_5	F003	123	Z distribution	-0.60484	-0.50267	0.448718
RAB5	W4	PGE2	well_5	F004	171	Z distribution	-0.60786	-0.57169	0.372084
RAB5	W4	PGE2	well_5	F005	112	Z distribution	-0.53932	-0.43051	0.358615
RAB5	W4	PGE2	well_5	F006	134	Z distribution	-0.58031	-0.54109	0.368336
SLC25A17	W1	HPI4	well_1	F001	82	Avg. Volume	0.45219	0.446284	0.078889
SLC25A17	W1	HPI4	well_1	F002	82	Avg. Volume	0.426453	0.426332	0.081543
SLC25A17	W1	HPI4	well_1	F003	58	Avg. Volume	0.394634	0.369283	0.091592
SLC25A17	W1	HPI4	well_1	F004	36	Avg. Volume	0.380251	0.40459	0.087924
SLC25A17	W1	HPI4	well_1	F005	72	Avg. Volume	0.204793	0.203883	0.046866
SLC25A17	W1	HPI4	well_1	F006	55	Avg. Volume	0.430649	0.427242	0.084074
SLC25A17	W1	HPI4	well_2	F001	81	Avg. Volume	0.433218	0.438608	0.083613
SLC25A17	W1	HPI4	well_2	F002	25	Avg. Volume	0.380742	0.372898	0.057198
SLC25A17	W1	HPI4	well_2	F003	68	Avg. Volume	0.451606	0.462941	0.073822
SLC25A17	W1	HPI4	well_2	F004	40	Avg. Volume	0.43321	0.428388	0.090517
SLC25A17	W1	HPI4	well_2	F005	46	Avg. Volume	0.448021	0.456879	0.095175
SLC25A17	W1	HPI4	well_2	F006	69	Avg. Volume	0.452656	0.455855	0.097259
SLC25A17	W1	HPI4	well_3	F001	72	Avg. Volume	0.315646	0.316931	0.074395
SLC25A17	W1	HPI4	well_3	F002	64	Avg. Volume	0.393533	0.378519	0.092229
SLC25A17	W1	HPI4	well_3	F003	69	Avg. Volume	0.432189	0.434841	0.070754
SLC25A17	W1	HPI4	well_3	F004	80	Avg. Volume	0.420935	0.433357	0.089807
SLC25A17	W1	HPI4	well_3	F005	53	Avg. Volume	0.406947	0.395116	0.089734
SLC25A17	W1	HPI4	well_3	F006	73	Avg. Volume	0.420155	0.415458	0.074324
SLC25A17	W1	HPI4	well_4	F001	47	Avg. Volume	0.432428	0.439968	0.087283
SLC25A17	W1	HPI4	well_4	F002	93	Avg. Volume	0.401011	0.391835	0.076931
SLC25A17	W1	HPI4	well_4	F003	119	Avg. Volume	0.422353	0.417986	0.074471
SLC25A17	W1	HPI4	well_4	F004	155	Avg. Volume	0.414719	0.415404	0.094089
SLC25A17	W1	HPI4	well_4	F005	142	Avg. Volume	0.436105	0.428771	0.085163
SLC25A17	W1	HPI4	well_4	F006	147	Avg. Volume	0.418915	0.426072	0.084171
SLC25A17	W1	HPI4	well_5	F001	69	Avg. Volume	0.391609	0.369432	0.08444
SLC25A17	W1	HPI4	well_5	F002	134	Avg. Volume	0.412865	0.41214	0.094532
SLC25A17	W1	HPI4	well_5	F003	136	Avg. Volume	0.384323	0.388549	0.073135
SLC25A17	W1	HPI4	well_5	F004	85	Avg. Volume	0.421986	0.426111	0.075966
SLC25A17	W1	HPI4	well_5	F005	55	Avg. Volume	0.411271	0.416688	0.098769
SLC25A17	W1	HPI4	well_5	F006	70	Avg. Volume	0.4081	0.412754	0.082989
SLC25A17	W1	PGE2	well_1	F001	58	Avg. Volume	0.446361	0.442535	0.085049
SLC25A17	W1	PGE2	well_1	F002	79	Avg. Volume	0.43118	0.443625	0.091765
SLC25A17	W1	PGE2	well_1	F003	145	Avg. Volume	0.430739	0.429034	0.077105
SLC25A17	W1	PGE2	well_1	F004	72	Avg. Volume	0.412096	0.402997	0.112387
SLC25A17	W1	PGE2	well_1	F005	124	Avg. Volume	0.418427	0.412535	0.099181
SLC25A17	W1	PGE2	well_1	F006	188	Avg. Volume	0.427657	0.424283	0.087113
SLC25A17	W1	PGE2	well_2	F001	131	Avg. Volume	0.37539	0.372826	0.059328
SLC25A17	W1	PGE2	well_2	F002	35	Avg. Volume	0.442836	0.439436	0.087793
SLC25A17	W1	PGE2	well_2	F003	75	Avg. Volume	0.434718	0.434854	0.091216

SLC25A17	W1	PGE2	well_2	F004	46	Avg. Volume	0.257666	0.245639	0.059197
SLC25A17	W1	PGE2	well_2	F005	88	Avg. Volume	0.384712	0.387646	0.094728
SLC25A17	W1	PGE2	well_2	F006	98	Avg. Volume	0.435798	0.424838	0.090465
SLC25A17	W1	PGE2	well_3	F001	58	Avg. Volume	0.38992	0.401012	0.086853
SLC25A17	W1	PGE2	well_3	F002	95	Avg. Volume	0.430644	0.433767	0.092639
SLC25A17	W1	PGE2	well_3	F003	88	Avg. Volume	0.410255	0.403817	0.086469
SLC25A17	W1	PGE2	well_3	F004	48	Avg. Volume	0.434586	0.429079	0.063905
SLC25A17	W1	PGE2	well_3	F005	101	Avg. Volume	0.429195	0.423324	0.0907
SLC25A17	W1	PGE2	well_3	F006	38	Avg. Volume	0.414191	0.415452	0.079118
SLC25A17	W1	PGE2	well_4	F001	27	Avg. Volume	0.466846	0.460056	0.094615
SLC25A17	W1	PGE2	well_4	F002	65	Avg. Volume	0.423434	0.427551	0.072311
SLC25A17	W1	PGE2	well_4	F003	58	Avg. Volume	0.445815	0.420854	0.096363
SLC25A17	W1	PGE2	well_4	F004	114	Avg. Volume	0.405712	0.403063	0.074749
SLC25A17	W1	PGE2	well_4	F005	81	Avg. Volume	0.392499	0.376824	0.109113
SLC25A17	W1	PGE2	well_4	F006	37	Avg. Volume	0.427889	0.445972	0.078754
SLC25A17	W1	PGE2	well_5	F001	81	Avg. Volume	0.366041	0.364849	0.068588
SLC25A17	W1	PGE2	well_5	F002	107	Avg. Volume	0.418156	0.414887	0.077675
SLC25A17	W1	PGE2	well_5	F003	63	Avg. Volume	0.418524	0.432882	0.086882
SLC25A17	W1	PGE2	well_5	F004	46	Avg. Volume	0.409532	0.415902	0.077814
SLC25A17	W1	PGE2	well_5	F005	35	Avg. Volume	0.418743	0.416156	0.105661
SLC25A17	W1	PGE2	well_5	F006	22	Avg. Volume	0.421427	0.424195	0.08406
SLC25A17	W2	HPI4	well_1	F001	25	Avg. Volume	0.356165	0.389145	0.091239
SLC25A17	W2	HPI4	well_1	F002	113	Avg. Volume	0.410334	0.402639	0.109358
SLC25A17	W2	HPI4	well_1	F003	63	Avg. Volume	0.38433	0.378909	0.101242
SLC25A17	W2	HPI4	well_1	F004	58	Avg. Volume	0.398734	0.397881	0.108322
SLC25A17	W2	HPI4	well_1	F005	61	Avg. Volume	0.384336	0.386422	0.113827
SLC25A17	W2	HPI4	well_1	F006	74	Avg. Volume	0.462725	0.449677	0.117346
SLC25A17	W2	HPI4	well_2	F001	31	Avg. Volume	0.399821	0.396094	0.083057
SLC25A17	W2	HPI4	well_2	F002	33	Avg. Volume	0.375446	0.364797	0.14131
SLC25A17	W2	HPI4	well_2	F003	44	Avg. Volume	0.376945	0.36725	0.103125
SLC25A17	W2	HPI4	well_2	F004	57	Avg. Volume	0.381659	0.385381	0.136198
SLC25A17	W2	HPI4	well_2	F005	1	Avg. Volume	0.070417	0.070417	NA
SLC25A17	W2	HPI4	well_2	F006	23	Avg. Volume	0.450569	0.446442	0.067579
SLC25A17	W2	HPI4	well_3	F001	18	Avg. Volume	0.413466	0.377122	0.141041
SLC25A17	W2	HPI4	well_3	F002	44	Avg. Volume	0.411889	0.407379	0.135528
SLC25A17	W2	HPI4	well_3	F003	45	Avg. Volume	0.647116	0.647461	0.252669
SLC25A17	W2	HPI4	well_3	F004	35	Avg. Volume	0.506549	0.505957	0.186593
SLC25A17	W2	HPI4	well_3	F005	111	Avg. Volume	0.397129	0.397513	0.100705
SLC25A17	W2	HPI4	well_3	F006	80	Avg. Volume	0.402155	0.415114	0.108998
SLC25A17	W2	HPI4	well_4	F001	26	Avg. Volume	0.4096	0.386352	0.153153
SLC25A17	W2	HPI4	well_4	F002	30	Avg. Volume	0.519726	0.455732	0.241302
SLC25A17	W2	HPI4	well_4	F003	50	Avg. Volume	0.477019	0.448011	0.157985
SLC25A17	W2	HPI4	well_4	F004	25	Avg. Volume	0.587971	0.596549	0.210343
SLC25A17	W2	HPI4	well_4	F005	24	Avg. Volume	0.55665	0.598305	0.205906
SLC25A17	W2	HPI4	well_4	F006	22	Avg. Volume	0.324726	0.311725	0.092999
SLC25A17	W2	HPI4	well_5	F001	59	Avg. Volume	0.609507	0.563333	0.212273
SLC25A17	W2	HPI4	well_5	F002	23	Avg. Volume	0.690262	0.672244	0.258336
SLC25A17	W2	HPI4	well_5	F003	10	Avg. Volume	0.660049	0.68901	0.177476
SLC25A17	W2	HPI4	well_5	F004	14	Avg. Volume	0.711435	0.50058	0.441072

SLC25A17	W2	HPI4	well_5	F005	28	Avg. Volume	0.578224	0.583757	0.178465
SLC25A17	W2	HPI4	well_5	F006	41	Avg. Volume	0.535061	0.488135	0.188445
SLC25A17	W2	PGE2	well_1	F001	29	Avg. Volume	0.38984	0.395898	0.105492
SLC25A17	W2	PGE2	well_1	F002	164	Avg. Volume	0.373539	0.376847	0.09417
SLC25A17	W2	PGE2	well_1	F003	79	Avg. Volume	0.449898	0.458882	0.127114
SLC25A17	W2	PGE2	well_1	F004	71	Avg. Volume	0.398564	0.385035	0.099114
SLC25A17	W2	PGE2	well_1	F005	188	Avg. Volume	0.389847	0.391845	0.084122
SLC25A17	W2	PGE2	well_1	F006	224	Avg. Volume	0.433317	0.441532	0.098277
SLC25A17	W2	PGE2	well_2	F001	85	Avg. Volume	0.409383	0.412512	0.106579
SLC25A17	W2	PGE2	well_2	F002	178	Avg. Volume	0.462834	0.467967	0.109412
SLC25A17	W2	PGE2	well_2	F003	62	Avg. Volume	0.461648	0.487946	0.11321
SLC25A17	W2	PGE2	well_2	F004	142	Avg. Volume	0.427301	0.436244	0.087229
SLC25A17	W2	PGE2	well_2	F005	176	Avg. Volume	0.389381	0.390449	0.078181
SLC25A17	W2	PGE2	well_2	F006	184	Avg. Volume	0.480535	0.485347	0.095252
SLC25A17	W2	PGE2	well_3	F001	142	Avg. Volume	0.420779	0.42898	0.087909
SLC25A17	W2	PGE2	well_3	F002	76	Avg. Volume	0.348719	0.338554	0.11602
SLC25A17	W2	PGE2	well_3	F003	188	Avg. Volume	0.48033	0.48663	0.08705
SLC25A17	W2	PGE2	well_3	F004	107	Avg. Volume	0.452684	0.439322	0.102244
SLC25A17	W2	PGE2	well_3	F005	63	Avg. Volume	0.465896	0.480398	0.103388
SLC25A17	W2	PGE2	well_3	F006	93	Avg. Volume	0.386579	0.374885	0.09984
SLC25A17	W2	PGE2	well_4	F001	287	Avg. Volume	0.510456	0.518572	0.105673
SLC25A17	W2	PGE2	well_4	F002	258	Avg. Volume	0.515813	0.516233	0.111809
SLC25A17	W2	PGE2	well_4	F003	344	Avg. Volume	0.478014	0.480611	0.10062
SLC25A17	W2	PGE2	well_4	F004	275	Avg. Volume	0.485596	0.485209	0.08656
SLC25A17	W2	PGE2	well_4	F005	216	Avg. Volume	0.468963	0.474503	0.092097
SLC25A17	W2	PGE2	well_4	F006	279	Avg. Volume	0.488128	0.491494	0.103168
SLC25A17	W2	PGE2	well_5	F001	190	Avg. Volume	0.530316	0.531937	0.129015
SLC25A17	W2	PGE2	well_5	F002	287	Avg. Volume	0.530028	0.521605	0.105028
SLC25A17	W2	PGE2	well_5	F003	153	Avg. Volume	0.460516	0.466009	0.101678
SLC25A17	W2	PGE2	well_5	F004	114	Avg. Volume	0.403064	0.417211	0.092191
SLC25A17	W2	PGE2	well_5	F005	126	Avg. Volume	0.459232	0.464004	0.114536
SLC25A17	W2	PGE2	well_5	F006	342	Avg. Volume	0.55668	0.557807	0.11038
SLC25A17	W3	HPI4	well_1	F002	10	Avg. Volume	0.370202	0.386743	0.066047
SLC25A17	W3	HPI4	well_1	F003	15	Avg. Volume	0.35039	0.341651	0.133042
SLC25A17	W3	HPI4	well_1	F004	7	Avg. Volume	0.341936	0.334425	0.114661
SLC25A17	W3	HPI4	well_1	F005	5	Avg. Volume	0.442639	0.431334	0.08361
SLC25A17	W3	HPI4	well_1	F006	6	Avg. Volume	0.372321	0.36583	0.077194
SLC25A17	W3	HPI4	well_2	F001	5	Avg. Volume	0.260284	0.251051	0.02217
SLC25A17	W3	HPI4	well_2	F002	5	Avg. Volume	0.39848	0.383007	0.053532
SLC25A17	W3	HPI4	well_2	F003	1	Avg. Volume	0.348511	0.348511	NA
SLC25A17	W3	HPI4	well_2	F004	15	Avg. Volume	0.420823	0.340683	0.182334
SLC25A17	W3	HPI4	well_2	F006	7	Avg. Volume	0.67396	0.497829	0.358904
SLC25A17	W3	HPI4	well_3	F001	7	Avg. Volume	0.431368	0.408612	0.141783
SLC25A17	W3	HPI4	well_3	F002	15	Avg. Volume	0.495356	0.445396	0.200407
SLC25A17	W3	HPI4	well_3	F003	5	Avg. Volume	0.272295	0.296841	0.080258
SLC25A17	W3	HPI4	well_3	F004	3	Avg. Volume	0.385421	0.385421	NA
SLC25A17	W3	HPI4	well_3	F005	9	Avg. Volume	0.406806	0.37849	0.087753
SLC25A17	W3	HPI4	well_3	F006	29	Avg. Volume	0.365872	0.37712	0.06723
SLC25A17	W3	HPI4	well_4	F001	13	Avg. Volume	0.485196	0.43717	0.20764

SLC25A17	W3	HPI4	well_4	F002	11	Avg. Volume	0.505158	0.459965	0.106718
SLC25A17	W3	HPI4	well_4	F003	8	Avg. Volume	0.472794	0.482944	0.050074
SLC25A17	W3	HPI4	well_4	F004	5	Avg. Volume	0.33462	0.284304	0.144614
SLC25A17	W3	HPI4	well_4	F005	9	Avg. Volume	0.306917	0.287535	0.064759
SLC25A17	W3	HPI4	well_4	F006	8	Avg. Volume	0.375751	0.388002	0.086524
SLC25A17	W3	HPI4	well_5	F001	5	Avg. Volume	0.274319	0.267184	0.019244
SLC25A17	W3	HPI4	well_5	F002	3	Avg. Volume	0.323917	0.323917	NA
SLC25A17	W3	HPI4	well_5	F004	5	Avg. Volume	0.581714	0.442266	0.334647
SLC25A17	W3	HPI4	well_5	F006	33	Avg. Volume	0.356568	0.333827	0.147799
SLC25A17	W3	PGE2	well_1	F001	104	Avg. Volume	0.332597	0.318656	0.090614
SLC25A17	W3	PGE2	well_1	F002	108	Avg. Volume	0.377717	0.388667	0.096308
SLC25A17	W3	PGE2	well_1	F003	172	Avg. Volume	0.406397	0.395537	0.10819
SLC25A17	W3	PGE2	well_1	F004	88	Avg. Volume	0.320866	0.328061	0.099128
SLC25A17	W3	PGE2	well_1	F005	133	Avg. Volume	0.357868	0.356395	0.101266
SLC25A17	W3	PGE2	well_1	F006	204	Avg. Volume	0.376795	0.375667	0.080323
SLC25A17	W3	PGE2	well_2	F001	149	Avg. Volume	0.427561	0.43717	0.098152
SLC25A17	W3	PGE2	well_2	F002	96	Avg. Volume	0.483887	0.482187	0.119323
SLC25A17	W3	PGE2	well_2	F003	70	Avg. Volume	0.397221	0.402109	0.120725
SLC25A17	W3	PGE2	well_2	F004	90	Avg. Volume	0.48206	0.479189	0.123612
SLC25A17	W3	PGE2	well_2	F005	209	Avg. Volume	0.441717	0.435083	0.098657
SLC25A17	W3	PGE2	well_2	F006	339	Avg. Volume	0.445952	0.447956	0.096119
SLC25A17	W3	PGE2	well_3	F001	238	Avg. Volume	0.392463	0.391632	0.078143
SLC25A17	W3	PGE2	well_3	F002	188	Avg. Volume	0.478165	0.48431	0.110665
SLC25A17	W3	PGE2	well_3	F003	204	Avg. Volume	0.431064	0.429323	0.084513
SLC25A17	W3	PGE2	well_3	F004	155	Avg. Volume	0.455468	0.457926	0.118257
SLC25A17	W3	PGE2	well_3	F005	372	Avg. Volume	0.458969	0.451435	0.095898
SLC25A17	W3	PGE2	well_3	F006	197	Avg. Volume	0.438408	0.433831	0.098139
SLC25A17	W3	PGE2	well_4	F001	239	Avg. Volume	0.438364	0.437688	0.091894
SLC25A17	W3	PGE2	well_4	F002	160	Avg. Volume	0.449285	0.454428	0.098932
SLC25A17	W3	PGE2	well_4	F003	93	Avg. Volume	0.461143	0.466371	0.106267
SLC25A17	W3	PGE2	well_4	F004	228	Avg. Volume	0.526495	0.524268	0.089686
SLC25A17	W3	PGE2	well_4	F005	201	Avg. Volume	0.471759	0.460358	0.113222
SLC25A17	W3	PGE2	well_4	F006	175	Avg. Volume	0.456739	0.461319	0.119077
SLC25A17	W3	PGE2	well_5	F001	139	Avg. Volume	0.404515	0.407018	0.102083
SLC25A17	W3	PGE2	well_5	F002	182	Avg. Volume	0.420424	0.415953	0.0919
SLC25A17	W3	PGE2	well_5	F003	241	Avg. Volume	0.442532	0.431849	0.100527
SLC25A17	W3	PGE2	well_5	F004	205	Avg. Volume	0.396406	0.399833	0.09708
SLC25A17	W3	PGE2	well_5	F005	314	Avg. Volume	0.370085	0.368682	0.086188
SLC25A17	W3	PGE2	well_5	F006	252	Avg. Volume	0.377016	0.36748	0.090985
SLC25A17	W4	HPI4	well_1	F001	7	Avg. Volume	0.750614	0.623188	0.219869
SLC25A17	W4	HPI4	well_1	F002	1	Avg. Volume	0.231961	0.231961	NA
SLC25A17	W4	HPI4	well_1	F003	1	Avg. Volume	0.548396	0.548396	NA
SLC25A17	W4	HPI4	well_1	F006	1	Avg. Volume	0.497611	0.497611	NA
SLC25A17	W4	HPI4	well_2	F003	1	Avg. Volume	0.228704	0.228704	NA
SLC25A17	W4	HPI4	well_3	F001	13	Avg. Volume	0.569416	0.540699	0.143185
SLC25A17	W4	HPI4	well_3	F003	1	Avg. Volume	0.292252	0.292252	NA
SLC25A17	W4	HPI4	well_3	F004	1	Avg. Volume	0.265686	0.265686	NA
SLC25A17	W4	HPI4	well_4	F001	3	Avg. Volume	0.690754	0.690754	NA
SLC25A17	W4	HPI4	well_4	F002	1	Avg. Volume	0.394213	0.394213	NA

SLC25A17	W4	HPI4	well_5	F001	1	Avg. Volume	0.054769	0.054769	NA
SLC25A17	W4	HPI4	well_5	F002	1	Avg. Volume	0.15909	0.15909	NA
SLC25A17	W4	HPI4	well_5	F004	1	Avg. Volume	0.331002	0.331002	NA
SLC25A17	W4	HPI4	well_5	F006	4	Avg. Volume	0.224656	0.224656	0.029935
SLC25A17	W4	PGE2	well_1	F001	222	Avg. Volume	0.453383	0.452465	0.077075
SLC25A17	W4	PGE2	well_1	F002	273	Avg. Volume	0.414444	0.415366	0.075625
SLC25A17	W4	PGE2	well_1	F003	246	Avg. Volume	0.440139	0.432768	0.077374
SLC25A17	W4	PGE2	well_1	F004	144	Avg. Volume	0.453673	0.457344	0.071633
SLC25A17	W4	PGE2	well_1	F005	267	Avg. Volume	0.460272	0.461696	0.077475
SLC25A17	W4	PGE2	well_1	F006	208	Avg. Volume	0.477864	0.482094	0.09187
SLC25A17	W4	PGE2	well_2	F001	216	Avg. Volume	0.427883	0.431384	0.082734
SLC25A17	W4	PGE2	well_2	F002	215	Avg. Volume	0.461	0.466807	0.086471
SLC25A17	W4	PGE2	well_2	F003	192	Avg. Volume	0.435781	0.42853	0.08814
SLC25A17	W4	PGE2	well_2	F004	202	Avg. Volume	0.430633	0.427523	0.077809
SLC25A17	W4	PGE2	well_2	F005	251	Avg. Volume	0.43723	0.434458	0.081853
SLC25A17	W4	PGE2	well_2	F006	200	Avg. Volume	0.424384	0.425162	0.076546
SLC25A17	W4	PGE2	well_3	F001	280	Avg. Volume	0.420922	0.416098	0.080157
SLC25A17	W4	PGE2	well_3	F002	258	Avg. Volume	0.469447	0.467257	0.07977
SLC25A17	W4	PGE2	well_3	F003	204	Avg. Volume	0.418455	0.426334	0.076353
SLC25A17	W4	PGE2	well_3	F004	203	Avg. Volume	0.377271	0.378083	0.088866
SLC25A17	W4	PGE2	well_3	F005	227	Avg. Volume	0.348258	0.350859	0.068157
SLC25A17	W4	PGE2	well_3	F006	291	Avg. Volume	0.413416	0.415143	0.074067
SLC25A17	W4	PGE2	well_4	F001	34	Avg. Volume	0.352492	0.336569	0.082721
SLC25A17	W4	PGE2	well_4	F002	254	Avg. Volume	0.459116	0.456427	0.090012
SLC25A17	W4	PGE2	well_4	F003	22	Avg. Volume	0.379268	0.345883	0.084767
SLC25A17	W4	PGE2	well_4	F004	31	Avg. Volume	0.431077	0.413991	0.121321
SLC25A17	W4	PGE2	well_4	F005	84	Avg. Volume	0.420129	0.411869	0.094215
SLC25A17	W4	PGE2	well_4	F006	43	Avg. Volume	0.401242	0.406412	0.077579
SLC25A17	W4	PGE2	well_5	F001	72	Avg. Volume	0.360964	0.358281	0.093337
SLC25A17	W4	PGE2	well_5	F002	46	Avg. Volume	0.411597	0.409853	0.082213
SLC25A17	W4	PGE2	well_5	F003	13	Avg. Volume	0.426154	0.476737	0.08684
SLC25A17	W4	PGE2	well_5	F004	39	Avg. Volume	0.390161	0.397238	0.096719
SLC25A17	W4	PGE2	well_5	F005	33	Avg. Volume	0.321005	0.296111	0.100606
SLC25A17	W4	PGE2	well_5	F006	108	Avg. Volume	0.40935	0.412151	0.094849
SLC25A17	W1	HPI4	well_1	F001	82	Count/cell	75.30556	76	22.92218
SLC25A17	W1	HPI4	well_1	F002	82	Count/cell	60.61111	61.5	23.11914
SLC25A17	W1	HPI4	well_1	F003	58	Count/cell	66.15385	59.5	35.10845
SLC25A17	W1	HPI4	well_1	F004	36	Count/cell	70.25	70.5	27.50367
SLC25A17	W1	HPI4	well_1	F005	72	Count/cell	55.90625	53.5	21.29663
SLC25A17	W1	HPI4	well_1	F006	55	Count/cell	62.5102	60	24.46436
SLC25A17	W1	HPI4	well_2	F001	81	Count/cell	76.30137	75	26.19409
SLC25A17	W1	HPI4	well_2	F002	25	Count/cell	74.52381	79	30.51167
SLC25A17	W1	HPI4	well_2	F003	68	Count/cell	93.61667	85.5	40.70859
SLC25A17	W1	HPI4	well_2	F004	40	Count/cell	77.86111	73.5	31.02456
SLC25A17	W1	HPI4	well_2	F005	46	Count/cell	73.55	66.5	32.37833
SLC25A17	W1	HPI4	well_2	F006	69	Count/cell	64.90164	63	25.36185
SLC25A17	W1	HPI4	well_3	F001	72	Count/cell	72.60938	70.5	32.5672
SLC25A17	W1	HPI4	well_3	F002	64	Count/cell	73.23214	70.5	26.35077
SLC25A17	W1	HPI4	well_3	F003	69	Count/cell	73.70492	70	33.55659

SLC25A17	W1	HPI4	well_3	F004	80	Count/cell	66.65278	63	27.54774
SLC25A17	W1	HPI4	well_3	F005	53	Count/cell	71.76596	75	23.76261
SLC25A17	W1	HPI4	well_3	F006	73	Count/cell	88.89231	78	43.61806
SLC25A17	W1	HPI4	well_4	F001	47	Count/cell	60.65854	63	24.77157
SLC25A17	W1	HPI4	well_4	F002	93	Count/cell	71.24096	71	17.89508
SLC25A17	W1	HPI4	well_4	F003	119	Count/cell	69.61682	63	25.41306
SLC25A17	W1	HPI4	well_4	F004	155	Count/cell	74.68345	72	27.81097
SLC25A17	W1	HPI4	well_4	F005	142	Count/cell	72.65873	68.5	27.95344
SLC25A17	W1	HPI4	well_4	F006	147	Count/cell	70.78788	72	26.70478
SLC25A17	W1	HPI4	well_5	F001	69	Count/cell	59.37097	56.5	25.6344
SLC25A17	W1	HPI4	well_5	F002	134	Count/cell	69.09091	70	24.08803
SLC25A17	W1	HPI4	well_5	F003	136	Count/cell	62.89344	59	27.31778
SLC25A17	W1	HPI4	well_5	F004	85	Count/cell	74.82667	73	22.21296
SLC25A17	W1	HPI4	well_5	F005	55	Count/cell	79.44898	79	32.01371
SLC25A17	W1	HPI4	well_5	F006	70	Count/cell	59.98387	61	21.31381
SLC25A17	W1	PGE2	well_1	F001	58	Count/cell	73.43396	74	22.23347
SLC25A17	W1	PGE2	well_1	F002	79	Count/cell	59.6338	61	21.56137
SLC25A17	W1	PGE2	well_1	F003	145	Count/cell	69.93798	68	23.13978
SLC25A17	W1	PGE2	well_1	F004	72	Count/cell	71.24615	72	26.59184
SLC25A17	W1	PGE2	well_1	F005	124	Count/cell	67.55455	66	27.57994
SLC25A17	W1	PGE2	well_1	F006	188	Count/cell	70.63314	69	23.50168
SLC25A17	W1	PGE2	well_2	F001	131	Count/cell	63.46154	66	20.75744
SLC25A17	W1	PGE2	well_2	F002	35	Count/cell	67	68	14.79189
SLC25A17	W1	PGE2	well_2	F003	75	Count/cell	69.76119	71	23.13275
SLC25A17	W1	PGE2	well_2	F004	46	Count/cell	51.6	52.5	16.19877
SLC25A17	W1	PGE2	well_2	F005	88	Count/cell	65.7875	62	28.58905
SLC25A17	W1	PGE2	well_2	F006	98	Count/cell	68.13483	68	27.80091
SLC25A17	W1	PGE2	well_3	F001	58	Count/cell	78.48077	66	37.4336
SLC25A17	W1	PGE2	well_3	F002	95	Count/cell	66.68605	64.5	28.69116
SLC25A17	W1	PGE2	well_3	F003	88	Count/cell	62.05128	60.5	24.87365
SLC25A17	W1	PGE2	well_3	F004	48	Count/cell	75.97619	74	24.03096
SLC25A17	W1	PGE2	well_3	F005	101	Count/cell	54.86813	54	22.02131
SLC25A17	W1	PGE2	well_3	F006	38	Count/cell	63.41176	63	24.04682
SLC25A17	W1	PGE2	well_4	F001	27	Count/cell	63.13043	61	23.75501
SLC25A17	W1	PGE2	well_4	F002	65	Count/cell	69.38596	73	20.90092
SLC25A17	W1	PGE2	well_4	F003	58	Count/cell	56.34615	59.5	24.98932
SLC25A17	W1	PGE2	well_4	F004	114	Count/cell	69.79612	68	30.92158
SLC25A17	W1	PGE2	well_4	F005	81	Count/cell	60.60274	59	28.34595
SLC25A17	W1	PGE2	well_4	F006	37	Count/cell	56.39394	56	21.29839
SLC25A17	W1	PGE2	well_5	F001	81	Count/cell	58.94595	57.5	21.94944
SLC25A17	W1	PGE2	well_5	F002	107	Count/cell	68.54737	69	23.05948
SLC25A17	W1	PGE2	well_5	F003	63	Count/cell	62.16364	57	25.68957
SLC25A17	W1	PGE2	well_5	F004	46	Count/cell	50.65	41.5	28.27842
SLC25A17	W1	PGE2	well_5	F005	35	Count/cell	70.41935	72	28.07107
SLC25A17	W1	PGE2	well_5	F006	22	Count/cell	81	71.5	26.99673
SLC25A17	W2	HPI4	well_1	F001	25	Count/cell	41.71429	38	19.40655
SLC25A17	W2	HPI4	well_1	F002	113	Count/cell	73.32353	71.5	33.21104
SLC25A17	W2	HPI4	well_1	F003	63	Count/cell	63.50909	59	27.33663
SLC25A17	W2	HPI4	well_1	F004	58	Count/cell	51.05769	51	23.07568

SLC25A17	W2	HPI4	well_1	F005	61	Count/cell	44.17857	41.5	24.73651
SLC25A17	W2	HPI4	well_1	F006	74	Count/cell	56.81818	54.5	23.10305
SLC25A17	W2	HPI4	well_2	F001	31	Count/cell	48.33333	44	19.19335
SLC25A17	W2	HPI4	well_2	F002	33	Count/cell	33.13793	26	22.57167
SLC25A17	W2	HPI4	well_2	F003	44	Count/cell	39.9	37	23.9752
SLC25A17	W2	HPI4	well_2	F004	57	Count/cell	47.73077	47.5	27.12507
SLC25A17	W2	HPI4	well_2	F005	1	Count/cell	1	1	NA
SLC25A17	W2	HPI4	well_2	F006	23	Count/cell	46.84211	49	10.88966
SLC25A17	W2	HPI4	well_3	F001	18	Count/cell	50	44.5	25.22697
SLC25A17	W2	HPI4	well_3	F002	44	Count/cell	42.92105	41.5	21.3141
SLC25A17	W2	HPI4	well_3	F003	45	Count/cell	42.58974	44	17.65267
SLC25A17	W2	HPI4	well_3	F004	35	Count/cell	43.48387	38	22.31124
SLC25A17	W2	HPI4	well_3	F005	111	Count/cell	52.51515	51	21.75061
SLC25A17	W2	HPI4	well_3	F006	80	Count/cell	62.58904	60	33.51983
SLC25A17	W2	HPI4	well_4	F001	26	Count/cell	42.18182	44	18.77089
SLC25A17	W2	HPI4	well_4	F002	30	Count/cell	39.88462	43	15.49536
SLC25A17	W2	HPI4	well_4	F003	50	Count/cell	54.61364	49	25.85669
SLC25A17	W2	HPI4	well_4	F004	25	Count/cell	43.26087	41	25.09838
SLC25A17	W2	HPI4	well_4	F005	24	Count/cell	36.3	36	16.18349
SLC25A17	W2	HPI4	well_4	F006	22	Count/cell	51.44444	57	21.44548
SLC25A17	W2	HPI4	well_5	F001	59	Count/cell	43.35849	39	19.80005
SLC25A17	W2	HPI4	well_5	F002	23	Count/cell	38.73684	36	14.05877
SLC25A17	W2	HPI4	well_5	F003	10	Count/cell	44.44444	46	18.1873
SLC25A17	W2	HPI4	well_5	F004	14	Count/cell	29.25	34.5	14.05913
SLC25A17	W2	HPI4	well_5	F005	28	Count/cell	41.75	43.5	17.09373
SLC25A17	W2	HPI4	well_5	F006	41	Count/cell	40.7027	37	18.23438
SLC25A17	W2	PGE2	well_1	F001	29	Count/cell	39.68	33	16.80208
SLC25A17	W2	PGE2	well_1	F002	164	Count/cell	60.40541	62.5	26.22271
SLC25A17	W2	PGE2	well_1	F003	79	Count/cell	77.51389	78	27.60281
SLC25A17	W2	PGE2	well_1	F004	71	Count/cell	66.20313	65.5	31.13931
SLC25A17	W2	PGE2	well_1	F005	188	Count/cell	65.25595	68	24.93512
SLC25A17	W2	PGE2	well_1	F006	224	Count/cell	68.37624	71	25.22845
SLC25A17	W2	PGE2	well_2	F001	85	Count/cell	63.08	63	24.1503
SLC25A17	W2	PGE2	well_2	F002	178	Count/cell	71.3625	72	21.49959
SLC25A17	W2	PGE2	well_2	F003	62	Count/cell	70.98148	71.5	24.5929
SLC25A17	W2	PGE2	well_2	F004	142	Count/cell	79.05556	74.5	25.79901
SLC25A17	W2	PGE2	well_2	F005	176	Count/cell	70.11321	70	27.89287
SLC25A17	W2	PGE2	well_2	F006	184	Count/cell	78.77108	80	28.56903
SLC25A17	W2	PGE2	well_3	F001	142	Count/cell	60.67717	63	26.12725
SLC25A17	W2	PGE2	well_3	F002	76	Count/cell	51.30882	49.5	29.55605
SLC25A17	W2	PGE2	well_3	F003	188	Count/cell	77.14286	74.5	30.0545
SLC25A17	W2	PGE2	well_3	F004	107	Count/cell	70.88421	71	24.71879
SLC25A17	W2	PGE2	well_3	F005	63	Count/cell	68.8	74	23.13039
SLC25A17	W2	PGE2	well_3	F006	93	Count/cell	62.44578	59	26.28885
SLC25A17	W2	PGE2	well_4	F001	287	Count/cell	68.52529	66	23.63625
SLC25A17	W2	PGE2	well_4	F002	258	Count/cell	61.67949	60	19.76047
SLC25A17	W2	PGE2	well_4	F003	344	Count/cell	66.37097	64	21.99002
SLC25A17	W2	PGE2	well_4	F004	275	Count/cell	78.58704	76	23.57481
SLC25A17	W2	PGE2	well_4	F005	216	Count/cell	81.06186	80.5	27.07802

SLC25A17	W2	PGE2	well_4	F006	279	Count/cell	71.3834	70	23.61562
SLC25A17	W2	PGE2	well_5	F001	190	Count/cell	61.25581	56	24.54531
SLC25A17	W2	PGE2	well_5	F002	287	Count/cell	59.32308	58	18.16302
SLC25A17	W2	PGE2	well_5	F003	153	Count/cell	68.80292	68	20.53936
SLC25A17	W2	PGE2	well_5	F004	114	Count/cell	58.875	61.5	28.56595
SLC25A17	W2	PGE2	well_5	F005	126	Count/cell	63.92035	65	25.44572
SLC25A17	W2	PGE2	well_5	F006	342	Count/cell	63.37785	64	20.18543
SLC25A17	W3	HPI4	well_1	F002	10	Count/cell	74.625	73.5	23.71219
SLC25A17	W3	HPI4	well_1	F003	15	Count/cell	60.69231	61	34.89361
SLC25A17	W3	HPI4	well_1	F004	7	Count/cell	34.8	28	26.56501
SLC25A17	W3	HPI4	well_1	F005	5	Count/cell	65	74	33.42155
SLC25A17	W3	HPI4	well_1	F006	6	Count/cell	75	76	5.09902
SLC25A17	W3	HPI4	well_2	F001	5	Count/cell	43.33333	42	2.309401
SLC25A17	W3	HPI4	well_2	F002	5	Count/cell	63.66667	63	6.027714
SLC25A17	W3	HPI4	well_2	F003	1	Count/cell	138	138	NA
SLC25A17	W3	HPI4	well_2	F004	15	Count/cell	40.15385	40	23.33755
SLC25A17	W3	HPI4	well_2	F006	7	Count/cell	43.4	43	17.4442
SLC25A17	W3	HPI4	well_3	F001	7	Count/cell	56.8	68	34.18625
SLC25A17	W3	HPI4	well_3	F002	15	Count/cell	43.23077	43	21.43655
SLC25A17	W3	HPI4	well_3	F003	5	Count/cell	85.66667	99	36.85557
SLC25A17	W3	HPI4	well_3	F004	3	Count/cell	67	67	NA
SLC25A17	W3	HPI4	well_3	F005	9	Count/cell	49	52	11.35782
SLC25A17	W3	HPI4	well_3	F006	29	Count/cell	56.44	59	25.0118
SLC25A17	W3	HPI4	well_4	F001	13	Count/cell	28.81818	30	17.63416
SLC25A17	W3	HPI4	well_4	F002	11	Count/cell	50.77778	52	17.73258
SLC25A17	W3	HPI4	well_4	F003	8	Count/cell	80.83333	77.5	25.16678
SLC25A17	W3	HPI4	well_4	F004	5	Count/cell	53.66667	64	31.78574
SLC25A17	W3	HPI4	well_4	F005	9	Count/cell	46.28571	45	17.41373
SLC25A17	W3	HPI4	well_4	F006	8	Count/cell	53.16667	54	20.39036
SLC25A17	W3	HPI4	well_5	F001	5	Count/cell	30.33333	28	5.859465
SLC25A17	W3	HPI4	well_5	F002	3	Count/cell	51	51	NA
SLC25A17	W3	HPI4	well_5	F004	5	Count/cell	26	24	8.185353
SLC25A17	W3	HPI4	well_5	F006	33	Count/cell	45.73333	39	38.68701
SLC25A17	W3	PGE2	well_1	F001	104	Count/cell	49.83871	46	25.37685
SLC25A17	W3	PGE2	well_1	F002	108	Count/cell	57.38144	56	17.60412
SLC25A17	W3	PGE2	well_1	F003	172	Count/cell	63.72078	60.5	25.58249
SLC25A17	W3	PGE2	well_1	F004	88	Count/cell	52.23077	51	23.3182
SLC25A17	W3	PGE2	well_1	F005	133	Count/cell	54.86555	49	23.35646
SLC25A17	W3	PGE2	well_1	F006	204	Count/cell	72.90217	71	26.08815
SLC25A17	W3	PGE2	well_2	F001	149	Count/cell	65.8797	64	25.05752
SLC25A17	W3	PGE2	well_2	F002	96	Count/cell	89.25581	86.5	28.67144
SLC25A17	W3	PGE2	well_2	F003	70	Count/cell	63.75806	60.5	32.66255
SLC25A17	W3	PGE2	well_2	F004	90	Count/cell	65.8875	64	22.4691
SLC25A17	W3	PGE2	well_2	F005	209	Count/cell	62.70899	63	21.00367
SLC25A17	W3	PGE2	well_2	F006	339	Count/cell	67.51623	67	21.76832
SLC25A17	W3	PGE2	well_3	F001	238	Count/cell	65.58605	65	20.93293
SLC25A17	W3	PGE2	well_3	F002	188	Count/cell	75.60588	75	26.72151
SLC25A17	W3	PGE2	well_3	F003	204	Count/cell	69.10929	67	21.06007
SLC25A17	W3	PGE2	well_3	F004	155	Count/cell	61.23741	63	21.55969

SLC25A17	W3	PGE2	well_3	F005	372	Count/cell	67.26946	67	21.44087
SLC25A17	W3	PGE2	well_3	F006	197	Count/cell	68.59887	65	23.20909
SLC25A17	W3	PGE2	well_4	F001	239	Count/cell	69.77209	68	22.52639
SLC25A17	W3	PGE2	well_4	F002	160	Count/cell	74.85417	75.5	22.34033
SLC25A17	W3	PGE2	well_4	F003	93	Count/cell	68.60241	69	23.06562
SLC25A17	W3	PGE2	well_4	F004	228	Count/cell	84.90686	84	21.6578
SLC25A17	W3	PGE2	well_4	F005	201	Count/cell	69.28177	70	18.35856
SLC25A17	W3	PGE2	well_4	F006	175	Count/cell	71.31847	68	23.34772
SLC25A17	W3	PGE2	well_5	F001	139	Count/cell	77.12	77	31.86833
SLC25A17	W3	PGE2	well_5	F002	182	Count/cell	76.74074	75	19.09166
SLC25A17	W3	PGE2	well_5	F003	241	Count/cell	78.96313	78	28.63028
SLC25A17	W3	PGE2	well_5	F004	205	Count/cell	64.09239	65	24.35904
SLC25A17	W3	PGE2	well_5	F005	314	Count/cell	57.40426	56	19.8024
SLC25A17	W3	PGE2	well_5	F006	252	Count/cell	63.29204	60	20.79431
SLC25A17	W4	HPI4	well_1	F001	7	Count/cell	23.6	21	6.655825
SLC25A17	W4	HPI4	well_1	F002	1	Count/cell	119	119	NA
SLC25A17	W4	HPI4	well_1	F003	1	Count/cell	33	33	NA
SLC25A17	W4	HPI4	well_1	F006	1	Count/cell	75	75	NA
SLC25A17	W4	HPI4	well_2	F003	1	Count/cell	39	39	NA
SLC25A17	W4	HPI4	well_3	F001	13	Count/cell	37.54545	32	18.37043
SLC25A17	W4	HPI4	well_3	F003	1	Count/cell	51	51	NA
SLC25A17	W4	HPI4	well_3	F004	1	Count/cell	94	94	NA
SLC25A17	W4	HPI4	well_4	F001	3	Count/cell	27	27	NA
SLC25A17	W4	HPI4	well_4	F002	1	Count/cell	39	39	NA
SLC25A17	W4	HPI4	well_5	F001	1	Count/cell	3	3	NA
SLC25A17	W4	HPI4	well_5	F002	1	Count/cell	18	18	NA
SLC25A17	W4	HPI4	well_5	F004	1	Count/cell	108	108	NA
SLC25A17	W4	HPI4	well_5	F006	4	Count/cell	75.5	75.5	2.12132
SLC25A17	W4	PGE2	well_1	F001	222	Count/cell	106.3731	103	28.24456
SLC25A17	W4	PGE2	well_1	F002	273	Count/cell	91.33469	91	21.38432
SLC25A17	W4	PGE2	well_1	F003	246	Count/cell	103.8227	104	25.10472
SLC25A17	W4	PGE2	well_1	F004	144	Count/cell	136.2734	135	30.16065
SLC25A17	W4	PGE2	well_1	F005	267	Count/cell	100.5208	100	27.13147
SLC25A17	W4	PGE2	well_1	F006	208	Count/cell	106.3387	107.5	28.46958
SLC25A17	W4	PGE2	well_2	F001	216	Count/cell	100.8196	102	25.70324
SLC25A17	W4	PGE2	well_2	F002	215	Count/cell	101.2021	100	24.41152
SLC25A17	W4	PGE2	well_2	F003	192	Count/cell	112.2965	111.5	35.55458
SLC25A17	W4	PGE2	well_2	F004	202	Count/cell	111.6222	113	32.23121
SLC25A17	W4	PGE2	well_2	F005	251	Count/cell	100.6444	101	22.38079
SLC25A17	W4	PGE2	well_2	F006	200	Count/cell	104.1989	104	31.22738
SLC25A17	W4	PGE2	well_3	F001	280	Count/cell	84.17391	84	20.59727
SLC25A17	W4	PGE2	well_3	F002	258	Count/cell	98.11588	96	26.18172
SLC25A17	W4	PGE2	well_3	F003	204	Count/cell	91.87912	93	27.55179
SLC25A17	W4	PGE2	well_3	F004	203	Count/cell	86.30055	85	33.59
SLC25A17	W4	PGE2	well_3	F005	227	Count/cell	96.30732	96	34.10621
SLC25A17	W4	PGE2	well_3	F006	291	Count/cell	95.60687	95	27.03046
SLC25A17	W4	PGE2	well_4	F001	34	Count/cell	71.53333	64	23.65956
SLC25A17	W4	PGE2	well_4	F002	254	Count/cell	74.71616	75	17.05134
SLC25A17	W4	PGE2	well_4	F003	22	Count/cell	77.16667	76	15.39003

SLC25A17	W4	PGE2	well_4	F004	31	Count/cell	77.92593	74	26.67025
SLC25A17	W4	PGE2	well_4	F005	84	Count/cell	86.35135	86	20.72087
SLC25A17	W4	PGE2	well_4	F006	43	Count/cell	80.45946	82	17.79918
SLC25A17	W4	PGE2	well_5	F001	72	Count/cell	65.95313	72	27.5159
SLC25A17	W4	PGE2	well_5	F002	46	Count/cell	71.825	69.5	22.11263
SLC25A17	W4	PGE2	well_5	F003	13	Count/cell	76.72727	81	21.82701
SLC25A17	W4	PGE2	well_5	F004	39	Count/cell	73.2	73	33.87069
SLC25A17	W4	PGE2	well_5	F005	33	Count/cell	71.86207	72	24.19522
SLC25A17	W4	PGE2	well_5	F006	108	Count/cell	67.3125	69.5	20.47042
SLC25A17	W1	HPI4	well_1	F001	82	X/Y distribution	5.328627	5.2557	0.89991
SLC25A17	W1	HPI4	well_1	F002	82	X/Y distribution	5.032474	5.037129	0.800066
SLC25A17	W1	HPI4	well_1	F003	58	X/Y distribution	5.389024	5.149559	1.618335
SLC25A17	W1	HPI4	well_1	F004	36	X/Y distribution	5.848189	5.950126	0.672765
SLC25A17	W1	HPI4	well_1	F005	72	X/Y distribution	5.700313	5.46128	1.142223
SLC25A17	W1	HPI4	well_1	F006	55	X/Y distribution	5.078919	5.036839	0.925003
SLC25A17	W1	HPI4	well_2	F001	81	X/Y distribution	5.746595	5.589895	1.035441
SLC25A17	W1	HPI4	well_2	F002	25	X/Y distribution	6.099353	6.341882	1.439203
SLC25A17	W1	HPI4	well_2	F003	68	X/Y distribution	6.356761	5.698235	1.647417
SLC25A17	W1	HPI4	well_2	F004	40	X/Y distribution	5.761026	5.619519	1.225775
SLC25A17	W1	HPI4	well_2	F005	46	X/Y distribution	5.644561	5.484061	0.815945
SLC25A17	W1	HPI4	well_2	F006	69	X/Y distribution	5.196876	5.108079	0.974179
SLC25A17	W1	HPI4	well_3	F001	72	X/Y distribution	5.733476	5.657571	1.014333
SLC25A17	W1	HPI4	well_3	F002	64	X/Y distribution	6.075518	5.818085	1.015888
SLC25A17	W1	HPI4	well_3	F003	69	X/Y distribution	5.593424	5.417204	1.156359
SLC25A17	W1	HPI4	well_3	F004	80	X/Y distribution	5.217009	5.270601	1.23
SLC25A17	W1	HPI4	well_3	F005	53	X/Y distribution	5.237666	5.22425	1.014618
SLC25A17	W1	HPI4	well_3	F006	73	X/Y distribution	6.066556	5.82646	1.173317
SLC25A17	W1	HPI4	well_4	F001	47	X/Y distribution	4.838929	4.729723	1.094965
SLC25A17	W1	HPI4	well_4	F002	93	X/Y distribution	5.650736	5.608616	0.830126
SLC25A17	W1	HPI4	well_4	F003	119	X/Y distribution	5.297604	5.250709	0.825411
SLC25A17	W1	HPI4	well_4	F004	155	X/Y distribution	5.262809	5.148931	0.908298
SLC25A17	W1	HPI4	well_4	F005	142	X/Y distribution	5.394349	5.284028	1.03674
SLC25A17	W1	HPI4	well_4	F006	147	X/Y distribution	4.958249	4.991596	0.923123
SLC25A17	W1	HPI4	well_5	F001	69	X/Y distribution	4.84633	4.703407	1.00015
SLC25A17	W1	HPI4	well_5	F002	134	X/Y distribution	4.970981	4.911031	0.811985
SLC25A17	W1	HPI4	well_5	F003	136	X/Y distribution	4.995409	4.86226	0.984138
SLC25A17	W1	HPI4	well_5	F004	85	X/Y distribution	5.555158	5.498825	0.788319
SLC25A17	W1	HPI4	well_5	F005	55	X/Y distribution	5.867578	5.804146	1.196901
SLC25A17	W1	HPI4	well_5	F006	70	X/Y distribution	5.262922	5.239953	0.76016
SLC25A17	W1	PGE2	well_1	F001	58	X/Y distribution	5.319468	5.225592	0.843054
SLC25A17	W1	PGE2	well_1	F002	79	X/Y distribution	5.432392	5.035103	1.211232
SLC25A17	W1	PGE2	well_1	F003	145	X/Y distribution	5.181028	5.054167	0.861895
SLC25A17	W1	PGE2	well_1	F004	72	X/Y distribution	5.218556	4.960156	1.008461
SLC25A17	W1	PGE2	well_1	F005	124	X/Y distribution	4.556714	4.505285	0.974827
SLC25A17	W1	PGE2	well_1	F006	188	X/Y distribution	5.194499	5.10446	0.804923
SLC25A17	W1	PGE2	well_2	F001	131	X/Y distribution	5.083548	5.090116	0.814395
SLC25A17	W1	PGE2	well_2	F002	35	X/Y distribution	5.728598	5.664553	0.891175
SLC25A17	W1	PGE2	well_2	F003	75	X/Y distribution	5.339085	5.154441	1.0482
SLC25A17	W1	PGE2	well_2	F004	46	X/Y distribution	5.060958	4.869656	1.124728

SLC25A17	W1	PGE2	well_2	F005	88	X/Y distribution	5.018317	4.922698	0.957078
SLC25A17	W1	PGE2	well_2	F006	98	X/Y distribution	5.193819	5.167569	0.925918
SLC25A17	W1	PGE2	well_3	F001	58	X/Y distribution	5.6751	5.654209	1.290693
SLC25A17	W1	PGE2	well_3	F002	95	X/Y distribution	4.964821	5.024318	1.111262
SLC25A17	W1	PGE2	well_3	F003	88	X/Y distribution	5.278791	5.036963	0.95396
SLC25A17	W1	PGE2	well_3	F004	48	X/Y distribution	5.746591	5.686829	0.77419
SLC25A17	W1	PGE2	well_3	F005	101	X/Y distribution	4.738448	4.659315	0.90126
SLC25A17	W1	PGE2	well_3	F006	38	X/Y distribution	5.581265	5.592843	0.968551
SLC25A17	W1	PGE2	well_4	F001	27	X/Y distribution	5.893219	6.007506	0.873818
SLC25A17	W1	PGE2	well_4	F002	65	X/Y distribution	5.408489	5.374864	0.68832
SLC25A17	W1	PGE2	well_4	F003	58	X/Y distribution	5.002606	5.002675	1.42039
SLC25A17	W1	PGE2	well_4	F004	114	X/Y distribution	4.896704	4.93333	1.03201
SLC25A17	W1	PGE2	well_4	F005	81	X/Y distribution	5.102203	5.086617	1.020974
SLC25A17	W1	PGE2	well_4	F006	37	X/Y distribution	4.969111	4.845176	0.983031
SLC25A17	W1	PGE2	well_5	F001	81	X/Y distribution	5.458046	5.368773	0.916201
SLC25A17	W1	PGE2	well_5	F002	107	X/Y distribution	5.484644	5.479041	0.944153
SLC25A17	W1	PGE2	well_5	F003	63	X/Y distribution	5.341595	5.171133	1.098592
SLC25A17	W1	PGE2	well_5	F004	46	X/Y distribution	4.646425	4.642904	1.062841
SLC25A17	W1	PGE2	well_5	F005	35	X/Y distribution	5.391512	5.161546	0.993613
SLC25A17	W1	PGE2	well_5	F006	22	X/Y distribution	6.563311	6.541144	1.330173
SLC25A17	W2	HPI4	well_1	F001	25	X/Y distribution	4.340417	3.911272	1.687515
SLC25A17	W2	HPI4	well_1	F002	113	X/Y distribution	5.906993	5.744501	1.381335
SLC25A17	W2	HPI4	well_1	F003	63	X/Y distribution	5.452727	5.620435	1.193784
SLC25A17	W2	HPI4	well_1	F004	58	X/Y distribution	5.126253	4.92278	0.893966
SLC25A17	W2	HPI4	well_1	F005	61	X/Y distribution	4.953225	4.889809	1.210569
SLC25A17	W2	HPI4	well_1	F006	74	X/Y distribution	4.988186	4.831754	1.275775
SLC25A17	W2	HPI4	well_2	F001	31	X/Y distribution	5.115258	5.158659	1.156179
SLC25A17	W2	HPI4	well_2	F002	33	X/Y distribution	4.044719	3.932269	1.068333
SLC25A17	W2	HPI4	well_2	F003	44	X/Y distribution	4.669317	4.708627	0.881871
SLC25A17	W2	HPI4	well_2	F004	57	X/Y distribution	4.913005	5.076383	1.256887
SLC25A17	W2	HPI4	well_2	F005	1	X/Y distribution	4.724799	4.724799	NA
SLC25A17	W2	HPI4	well_2	F006	23	X/Y distribution	4.437662	4.380663	0.809234
SLC25A17	W2	HPI4	well_3	F001	18	X/Y distribution	5.439754	5.730194	1.379849
SLC25A17	W2	HPI4	well_3	F002	44	X/Y distribution	4.637703	4.427819	1.012837
SLC25A17	W2	HPI4	well_3	F003	45	X/Y distribution	4.085665	3.909783	0.796309
SLC25A17	W2	HPI4	well_3	F004	35	X/Y distribution	4.10489	3.964815	0.98768
SLC25A17	W2	HPI4	well_3	F005	111	X/Y distribution	4.9579	4.989762	1.081498
SLC25A17	W2	HPI4	well_3	F006	80	X/Y distribution	5.265012	5.478132	1.316411
SLC25A17	W2	HPI4	well_4	F001	26	X/Y distribution	4.741191	4.929192	0.783148
SLC25A17	W2	HPI4	well_4	F002	30	X/Y distribution	4.354255	4.25461	0.83629
SLC25A17	W2	HPI4	well_4	F003	50	X/Y distribution	5.071339	5.098973	1.045296
SLC25A17	W2	HPI4	well_4	F004	25	X/Y distribution	4.233459	4.544337	0.53945
SLC25A17	W2	HPI4	well_4	F005	24	X/Y distribution	3.613693	3.439685	0.749883
SLC25A17	W2	HPI4	well_4	F006	22	X/Y distribution	5.237448	5.339509	0.937906
SLC25A17	W2	HPI4	well_5	F001	59	X/Y distribution	3.757501	3.734963	0.496183
SLC25A17	W2	HPI4	well_5	F002	23	X/Y distribution	3.632387	3.616512	0.386871
SLC25A17	W2	HPI4	well_5	F003	10	X/Y distribution	4.158134	4.116628	0.592408
SLC25A17	W2	HPI4	well_5	F004	14	X/Y distribution	3.937466	3.777406	0.519834
SLC25A17	W2	HPI4	well_5	F005	28	X/Y distribution	3.683737	3.636453	0.458961

SLC25A17	W2	HPI4	well_5	F006	41	X/Y distribution	3.889526	3.818208	0.728244
SLC25A17	W2	PGE2	well_1	F001	29	X/Y distribution	4.204622	3.897289	1.064872
SLC25A17	W2	PGE2	well_1	F002	164	X/Y distribution	4.821555	4.854388	0.804455
SLC25A17	W2	PGE2	well_1	F003	79	X/Y distribution	5.819705	5.668178	0.923225
SLC25A17	W2	PGE2	well_1	F004	71	X/Y distribution	4.891939	4.854117	0.947638
SLC25A17	W2	PGE2	well_1	F005	188	X/Y distribution	5.08966	5.141786	0.841038
SLC25A17	W2	PGE2	well_1	F006	224	X/Y distribution	5.054487	5.145137	0.788024
SLC25A17	W2	PGE2	well_2	F001	85	X/Y distribution	5.527533	5.549169	0.924981
SLC25A17	W2	PGE2	well_2	F002	178	X/Y distribution	4.99774	4.872694	0.772595
SLC25A17	W2	PGE2	well_2	F003	62	X/Y distribution	5.593527	5.605985	0.928002
SLC25A17	W2	PGE2	well_2	F004	142	X/Y distribution	5.647279	5.61045	0.777248
SLC25A17	W2	PGE2	well_2	F005	176	X/Y distribution	5.249259	5.195801	0.953667
SLC25A17	W2	PGE2	well_2	F006	184	X/Y distribution	5.457767	5.423092	0.876806
SLC25A17	W2	PGE2	well_3	F001	142	X/Y distribution	4.676329	4.739769	0.948639
SLC25A17	W2	PGE2	well_3	F002	76	X/Y distribution	4.768447	4.778262	1.25914
SLC25A17	W2	PGE2	well_3	F003	188	X/Y distribution	5.493115	5.445203	0.990287
SLC25A17	W2	PGE2	well_3	F004	107	X/Y distribution	4.984652	4.994201	0.744736
SLC25A17	W2	PGE2	well_3	F005	63	X/Y distribution	5.556776	5.446966	1.006935
SLC25A17	W2	PGE2	well_3	F006	93	X/Y distribution	4.969166	4.977563	0.771049
SLC25A17	W2	PGE2	well_4	F001	287	X/Y distribution	5.04798	4.904258	0.921209
SLC25A17	W2	PGE2	well_4	F002	258	X/Y distribution	5.184773	5.161411	0.924438
SLC25A17	W2	PGE2	well_4	F003	344	X/Y distribution	5.112992	5.017081	0.917109
SLC25A17	W2	PGE2	well_4	F004	275	X/Y distribution	5.534203	5.484556	0.746207
SLC25A17	W2	PGE2	well_4	F005	216	X/Y distribution	5.877053	5.876759	0.849473
SLC25A17	W2	PGE2	well_4	F006	279	X/Y distribution	5.111312	5.019762	0.838173
SLC25A17	W2	PGE2	well_5	F001	190	X/Y distribution	5.097845	4.95449	1.126256
SLC25A17	W2	PGE2	well_5	F002	287	X/Y distribution	4.733595	4.632113	0.89475
SLC25A17	W2	PGE2	well_5	F003	153	X/Y distribution	5.428591	5.405062	0.858349
SLC25A17	W2	PGE2	well_5	F004	114	X/Y distribution	4.379873	4.39269	0.962058
SLC25A17	W2	PGE2	well_5	F005	126	X/Y distribution	4.838902	4.747561	0.959596
SLC25A17	W2	PGE2	well_5	F006	342	X/Y distribution	4.870917	4.839245	0.896114
SLC25A17	W3	HPI4	well_1	F002	10	X/Y distribution	7.392976	7.525868	1.281709
SLC25A17	W3	HPI4	well_1	F003	15	X/Y distribution	6.17524	6.108648	1.179807
SLC25A17	W3	HPI4	well_1	F004	7	X/Y distribution	8.448616	7.592133	1.736645
SLC25A17	W3	HPI4	well_1	F005	5	X/Y distribution	5.237512	5.345514	2.157684
SLC25A17	W3	HPI4	well_1	F006	6	X/Y distribution	7.703149	7.799091	0.750223
SLC25A17	W3	HPI4	well_2	F001	5	X/Y distribution	7.353204	8.167029	2.102649
SLC25A17	W3	HPI4	well_2	F002	5	X/Y distribution	5.708894	5.698982	0.456687
SLC25A17	W3	HPI4	well_2	F003	1	X/Y distribution	8.476899	8.476899	NA
SLC25A17	W3	HPI4	well_2	F004	15	X/Y distribution	4.552428	4.53375	1.331182
SLC25A17	W3	HPI4	well_2	F006	7	X/Y distribution	4.589491	4.204132	1.346611
SLC25A17	W3	HPI4	well_3	F001	7	X/Y distribution	6.173209	5.535984	1.074023
SLC25A17	W3	HPI4	well_3	F002	15	X/Y distribution	4.955525	3.899082	2.091614
SLC25A17	W3	HPI4	well_3	F003	5	X/Y distribution	9.583365	9.420233	0.550095
SLC25A17	W3	HPI4	well_3	F004	3	X/Y distribution	5.818921	5.818921	NA
SLC25A17	W3	HPI4	well_3	F005	9	X/Y distribution	5.064254	4.59527	0.986848
SLC25A17	W3	HPI4	well_3	F006	29	X/Y distribution	5.114191	4.942827	1.432602
SLC25A17	W3	HPI4	well_4	F001	13	X/Y distribution	4.060063	4.196948	1.187045
SLC25A17	W3	HPI4	well_4	F002	11	X/Y distribution	4.8109	4.916047	1.07711

SLC25A17	W3	HPI4	well_4	F003	8	X/Y distribution	6.846682	6.842461	1.520512
SLC25A17	W3	HPI4	well_4	F004	5	X/Y distribution	6.206706	6.133311	0.719611
SLC25A17	W3	HPI4	well_4	F005	9	X/Y distribution	6.041023	5.525239	1.326526
SLC25A17	W3	HPI4	well_4	F006	8	X/Y distribution	5.211142	5.294033	0.296201
SLC25A17	W3	HPI4	well_5	F001	5	X/Y distribution	4.763785	4.568784	0.421035
SLC25A17	W3	HPI4	well_5	F002	3	X/Y distribution	7.280423	7.280423	NA
SLC25A17	W3	HPI4	well_5	F004	5	X/Y distribution	3.786233	3.957656	0.328843
SLC25A17	W3	HPI4	well_5	F006	33	X/Y distribution	5.054942	5.114041	1.24651
SLC25A17	W3	PGE2	well_1	F001	104	X/Y distribution	4.839041	4.847982	1.021839
SLC25A17	W3	PGE2	well_1	F002	108	X/Y distribution	5.134401	5.129053	0.873878
SLC25A17	W3	PGE2	well_1	F003	172	X/Y distribution	5.335443	5.286528	1.060675
SLC25A17	W3	PGE2	well_1	F004	88	X/Y distribution	5.427684	5.392282	1.229725
SLC25A17	W3	PGE2	well_1	F005	133	X/Y distribution	5.20627	5.094736	0.891854
SLC25A17	W3	PGE2	well_1	F006	204	X/Y distribution	5.677463	5.564381	0.948783
SLC25A17	W3	PGE2	well_2	F001	149	X/Y distribution	5.483178	5.445445	0.940914
SLC25A17	W3	PGE2	well_2	F002	96	X/Y distribution	6.103232	6.057186	0.978327
SLC25A17	W3	PGE2	well_2	F003	70	X/Y distribution	5.457614	5.393405	1.274706
SLC25A17	W3	PGE2	well_2	F004	90	X/Y distribution	5.522779	5.370564	1.25512
SLC25A17	W3	PGE2	well_2	F005	209	X/Y distribution	5.11408	5.144789	0.85636
SLC25A17	W3	PGE2	well_2	F006	339	X/Y distribution	5.274489	5.153434	0.825872
SLC25A17	W3	PGE2	well_3	F001	238	X/Y distribution	5.399107	5.349444	0.815269
SLC25A17	W3	PGE2	well_3	F002	188	X/Y distribution	5.715125	5.627883	0.968798
SLC25A17	W3	PGE2	well_3	F003	204	X/Y distribution	5.649839	5.667639	0.967269
SLC25A17	W3	PGE2	well_3	F004	155	X/Y distribution	5.216274	5.261047	0.915184
SLC25A17	W3	PGE2	well_3	F005	372	X/Y distribution	5.2173	5.220993	0.834925
SLC25A17	W3	PGE2	well_3	F006	197	X/Y distribution	5.455149	5.361835	0.85723
SLC25A17	W3	PGE2	well_4	F001	239	X/Y distribution	5.397509	5.36236	0.874177
SLC25A17	W3	PGE2	well_4	F002	160	X/Y distribution	5.915928	5.907235	0.862161
SLC25A17	W3	PGE2	well_4	F003	93	X/Y distribution	5.64992	5.79378	0.879464
SLC25A17	W3	PGE2	well_4	F004	228	X/Y distribution	5.843567	5.7496	0.830098
SLC25A17	W3	PGE2	well_4	F005	201	X/Y distribution	5.589481	5.549562	0.826578
SLC25A17	W3	PGE2	well_4	F006	175	X/Y distribution	5.504392	5.321191	1.026873
SLC25A17	W3	PGE2	well_5	F001	139	X/Y distribution	6.301166	6.300217	1.143083
SLC25A17	W3	PGE2	well_5	F002	182	X/Y distribution	6.512825	6.395631	0.978418
SLC25A17	W3	PGE2	well_5	F003	241	X/Y distribution	5.855195	5.828732	1.021131
SLC25A17	W3	PGE2	well_5	F004	205	X/Y distribution	5.741624	5.647697	1.02183
SLC25A17	W3	PGE2	well_5	F005	314	X/Y distribution	5.297145	5.213249	0.856291
SLC25A17	W3	PGE2	well_5	F006	252	X/Y distribution	5.571699	5.541494	0.914573
SLC25A17	W4	HPI4	well_1	F001	7	X/Y distribution	4.529373	4.445436	0.69495
SLC25A17	W4	HPI4	well_1	F002	1	X/Y distribution	9.652736	9.652736	NA
SLC25A17	W4	HPI4	well_1	F003	1	X/Y distribution	4.459461	4.459461	NA
SLC25A17	W4	HPI4	well_1	F006	1	X/Y distribution	6.49754	6.49754	NA
SLC25A17	W4	HPI4	well_2	F003	1	X/Y distribution	5.535218	5.535218	NA
SLC25A17	W4	HPI4	well_3	F001	13	X/Y distribution	4.663118	4.538685	0.679489
SLC25A17	W4	HPI4	well_3	F003	1	X/Y distribution	7.441887	7.441887	NA
SLC25A17	W4	HPI4	well_3	F004	1	X/Y distribution	6.539693	6.539693	NA
SLC25A17	W4	HPI4	well_4	F001	3	X/Y distribution	5.38181	5.38181	NA
SLC25A17	W4	HPI4	well_4	F002	1	X/Y distribution	6.645558	6.645558	NA
SLC25A17	W4	HPI4	well_5	F001	1	X/Y distribution	7.352029	7.352029	NA

SLC25A17	W4	HPI4	well_5	F002	1	X/Y distribution	5.201527	5.201527	NA
SLC25A17	W4	HPI4	well_5	F004	1	X/Y distribution	6.484046	6.484046	NA
SLC25A17	W4	HPI4	well_5	F006	4	X/Y distribution	6.72546	6.72546	0.412849
SLC25A17	W4	PGE2	well_1	F001	222	X/Y distribution	6.066038	5.996191	0.680659
SLC25A17	W4	PGE2	well_1	F002	273	X/Y distribution	5.576915	5.505751	0.546627
SLC25A17	W4	PGE2	well_1	F003	246	X/Y distribution	5.880355	5.863115	0.609471
SLC25A17	W4	PGE2	well_1	F004	144	X/Y distribution	6.820308	6.793796	0.656358
SLC25A17	W4	PGE2	well_1	F005	267	X/Y distribution	5.711097	5.675757	0.640963
SLC25A17	W4	PGE2	well_1	F006	208	X/Y distribution	5.918418	5.891433	0.657096
SLC25A17	W4	PGE2	well_2	F001	216	X/Y distribution	6.209843	6.173525	0.70914
SLC25A17	W4	PGE2	well_2	F002	215	X/Y distribution	6.13373	6.099378	0.716435
SLC25A17	W4	PGE2	well_2	F003	192	X/Y distribution	6.547029	6.402127	0.926658
SLC25A17	W4	PGE2	well_2	F004	202	X/Y distribution	6.363082	6.286694	0.787776
SLC25A17	W4	PGE2	well_2	F005	251	X/Y distribution	6.050763	5.972411	0.607767
SLC25A17	W4	PGE2	well_2	F006	200	X/Y distribution	5.901802	5.862274	0.692705
SLC25A17	W4	PGE2	well_3	F001	280	X/Y distribution	5.839174	5.860337	0.696169
SLC25A17	W4	PGE2	well_3	F002	258	X/Y distribution	6.164072	6.148088	0.755669
SLC25A17	W4	PGE2	well_3	F003	204	X/Y distribution	6.116475	6.186228	0.772043
SLC25A17	W4	PGE2	well_3	F004	203	X/Y distribution	5.964053	5.988795	0.868491
SLC25A17	W4	PGE2	well_3	F005	227	X/Y distribution	6.135208	5.94881	0.992453
SLC25A17	W4	PGE2	well_3	F006	291	X/Y distribution	5.821954	5.763	0.590172
SLC25A17	W4	PGE2	well_4	F001	34	X/Y distribution	6.229329	6.446612	0.787274
SLC25A17	W4	PGE2	well_4	F002	254	X/Y distribution	5.56425	5.531493	0.691072
SLC25A17	W4	PGE2	well_4	F003	22	X/Y distribution	6.062343	5.883283	0.599707
SLC25A17	W4	PGE2	well_4	F004	31	X/Y distribution	5.81269	5.923249	0.713563
SLC25A17	W4	PGE2	well_4	F005	84	X/Y distribution	5.8982	5.856733	0.683223
SLC25A17	W4	PGE2	well_4	F006	43	X/Y distribution	6.061482	6.090857	0.584759
SLC25A17	W4	PGE2	well_5	F001	72	X/Y distribution	5.785624	5.722739	1.037389
SLC25A17	W4	PGE2	well_5	F002	46	X/Y distribution	5.961012	5.824329	1.06301
SLC25A17	W4	PGE2	well_5	F003	13	X/Y distribution	6.258473	6.569246	0.939025
SLC25A17	W4	PGE2	well_5	F004	39	X/Y distribution	6.085847	6.144639	1.271892
SLC25A17	W4	PGE2	well_5	F005	33	X/Y distribution	6.06653	5.907818	0.951885
SLC25A17	W4	PGE2	well_5	F006	108	X/Y distribution	5.577577	5.616208	0.76515
SLC25A17	W1	HPI4	well_1	F001	82	Z distribution	-0.18768	-0.13756	0.24475
SLC25A17	W1	HPI4	well_1	F002	82	Z distribution	-0.35868	-0.33729	0.23404
SLC25A17	W1	HPI4	well_1	F003	58	Z distribution	-0.25704	-0.26301	0.190536
SLC25A17	W1	HPI4	well_1	F004	36	Z distribution	-0.25678	-0.23989	0.257304
SLC25A17	W1	HPI4	well_1	F005	72	Z distribution	-0.2524	-0.24453	0.24875
SLC25A17	W1	HPI4	well_1	F006	55	Z distribution	-0.28471	-0.25628	0.221664
SLC25A17	W1	HPI4	well_2	F001	81	Z distribution	-0.29276	-0.29423	0.245365
SLC25A17	W1	HPI4	well_2	F002	25	Z distribution	-0.2785	-0.19653	0.218009
SLC25A17	W1	HPI4	well_2	F003	68	Z distribution	-0.34057	-0.35517	0.241608
SLC25A17	W1	HPI4	well_2	F004	40	Z distribution	-0.30927	-0.31731	0.188788
SLC25A17	W1	HPI4	well_2	F005	46	Z distribution	-0.1627	-0.16395	0.21249
SLC25A17	W1	HPI4	well_2	F006	69	Z distribution	-0.22872	-0.22661	0.192592
SLC25A17	W1	HPI4	well_3	F001	72	Z distribution	-0.32635	-0.34851	0.262426
SLC25A17	W1	HPI4	well_3	F002	64	Z distribution	-0.17598	-0.17154	0.167441
SLC25A17	W1	HPI4	well_3	F003	69	Z distribution	-0.2867	-0.28507	0.242543
SLC25A17	W1	HPI4	well_3	F004	80	Z distribution	-0.35029	-0.32608	0.224805

SLC25A17	W1	HPI4	well_3	F005	53	Z distribution	-0.49375	-0.49159	0.241137
SLC25A17	W1	HPI4	well_3	F006	73	Z distribution	-0.30034	-0.26978	0.238691
SLC25A17	W1	HPI4	well_4	F001	47	Z distribution	-0.34533	-0.33454	0.243828
SLC25A17	W1	HPI4	well_4	F002	93	Z distribution	-0.12284	-0.1283	0.218948
SLC25A17	W1	HPI4	well_4	F003	119	Z distribution	-0.21416	-0.21263	0.225056
SLC25A17	W1	HPI4	well_4	F004	155	Z distribution	-0.24791	-0.24092	0.252071
SLC25A17	W1	HPI4	well_4	F005	142	Z distribution	-0.25197	-0.23562	0.252134
SLC25A17	W1	HPI4	well_4	F006	147	Z distribution	-0.40923	-0.37913	0.265127
SLC25A17	W1	HPI4	well_5	F001	69	Z distribution	-0.2587	-0.25825	0.210543
SLC25A17	W1	HPI4	well_5	F002	134	Z distribution	-0.41433	-0.36997	0.277432
SLC25A17	W1	HPI4	well_5	F003	136	Z distribution	-0.24001	-0.25659	0.250873
SLC25A17	W1	HPI4	well_5	F004	85	Z distribution	-0.26379	-0.23883	0.266516
SLC25A17	W1	HPI4	well_5	F005	55	Z distribution	-0.27623	-0.22264	0.219868
SLC25A17	W1	HPI4	well_5	F006	70	Z distribution	-0.20725	-0.20664	0.214326
SLC25A17	W1	PGE2	well_1	F001	58	Z distribution	-0.19236	-0.19361	0.19488
SLC25A17	W1	PGE2	well_1	F002	79	Z distribution	-0.16495	-0.17293	0.23663
SLC25A17	W1	PGE2	well_1	F003	145	Z distribution	-0.29408	-0.27251	0.288617
SLC25A17	W1	PGE2	well_1	F004	72	Z distribution	-0.35612	-0.29937	0.330527
SLC25A17	W1	PGE2	well_1	F005	124	Z distribution	-0.54362	-0.54459	0.347127
SLC25A17	W1	PGE2	well_1	F006	188	Z distribution	-0.18085	-0.21052	0.226351
SLC25A17	W1	PGE2	well_2	F001	131	Z distribution	-0.27927	-0.27154	0.208116
SLC25A17	W1	PGE2	well_2	F002	35	Z distribution	-0.15611	-0.11825	0.157115
SLC25A17	W1	PGE2	well_2	F003	75	Z distribution	-0.32703	-0.32704	0.229184
SLC25A17	W1	PGE2	well_2	F004	46	Z distribution	-0.26147	-0.23807	0.231705
SLC25A17	W1	PGE2	well_2	F005	88	Z distribution	-0.39746	-0.34936	0.262944
SLC25A17	W1	PGE2	well_2	F006	98	Z distribution	-0.24689	-0.21558	0.202863
SLC25A17	W1	PGE2	well_3	F001	58	Z distribution	-0.29061	-0.29344	0.192429
SLC25A17	W1	PGE2	well_3	F002	95	Z distribution	-0.36006	-0.34127	0.226169
SLC25A17	W1	PGE2	well_3	F003	88	Z distribution	-0.27693	-0.24449	0.216697
SLC25A17	W1	PGE2	well_3	F004	48	Z distribution	-0.25065	-0.23863	0.237001
SLC25A17	W1	PGE2	well_3	F005	101	Z distribution	-0.23482	-0.26554	0.207115
SLC25A17	W1	PGE2	well_3	F006	38	Z distribution	-0.14599	-0.13488	0.146391
SLC25A17	W1	PGE2	well_4	F001	27	Z distribution	-0.14841	-0.13494	0.155599
SLC25A17	W1	PGE2	well_4	F002	65	Z distribution	-0.20911	-0.20761	0.194678
SLC25A17	W1	PGE2	well_4	F003	58	Z distribution	-0.40977	-0.35365	0.263025
SLC25A17	W1	PGE2	well_4	F004	114	Z distribution	-0.39914	-0.38697	0.274455
SLC25A17	W1	PGE2	well_4	F005	81	Z distribution	-0.33018	-0.28511	0.276761
SLC25A17	W1	PGE2	well_4	F006	37	Z distribution	-0.25218	-0.24604	0.19088
SLC25A17	W1	PGE2	well_5	F001	81	Z distribution	-0.27395	-0.26484	0.246253
SLC25A17	W1	PGE2	well_5	F002	107	Z distribution	-0.12957	-0.12119	0.213156
SLC25A17	W1	PGE2	well_5	F003	63	Z distribution	-0.3379	-0.31063	0.240117
SLC25A17	W1	PGE2	well_5	F004	46	Z distribution	-0.44998	-0.46128	0.232702
SLC25A17	W1	PGE2	well_5	F005	35	Z distribution	-0.25362	-0.24142	0.241071
SLC25A17	W1	PGE2	well_5	F006	22	Z distribution	-0.14054	-0.11399	0.157381
SLC25A17	W2	HPI4	well_1	F001	25	Z distribution	-0.50137	-0.4676	0.285259
SLC25A17	W2	HPI4	well_1	F002	113	Z distribution	-0.32877	-0.30184	0.253977
SLC25A17	W2	HPI4	well_1	F003	63	Z distribution	-0.29582	-0.25561	0.320457
SLC25A17	W2	HPI4	well_1	F004	58	Z distribution	-0.21756	-0.15971	0.259185
SLC25A17	W2	HPI4	well_1	F005	61	Z distribution	-0.15076	-0.135	0.274629

SLC25A17	W2	HPI4	well_1	F006	74	Z distribution	-0.30094	-0.21889	0.32253
SLC25A17	W2	HPI4	well_2	F001	31	Z distribution	-0.34974	-0.31023	0.291293
SLC25A17	W2	HPI4	well_2	F002	33	Z distribution	-0.41379	-0.2053	0.512428
SLC25A17	W2	HPI4	well_2	F003	44	Z distribution	-0.26534	-0.15936	0.409435
SLC25A17	W2	HPI4	well_2	F004	57	Z distribution	-0.32941	-0.3112	0.318138
SLC25A17	W2	HPI4	well_2	F005	1	Z distribution	0.301501	0.301501	NA
SLC25A17	W2	HPI4	well_2	F006	23	Z distribution	-0.35988	-0.33481	0.198986
SLC25A17	W2	HPI4	well_3	F001	18	Z distribution	-0.37706	-0.34958	0.221472
SLC25A17	W2	HPI4	well_3	F002	44	Z distribution	-0.23371	-0.18487	0.206113
SLC25A17	W2	HPI4	well_3	F003	45	Z distribution	-0.35902	-0.30828	0.377357
SLC25A17	W2	HPI4	well_3	F004	35	Z distribution	-0.27806	-0.16266	0.37841
SLC25A17	W2	HPI4	well_3	F005	111	Z distribution	-0.15613	-0.09781	0.252396
SLC25A17	W2	HPI4	well_3	F006	80	Z distribution	-0.25954	-0.20092	0.272248
SLC25A17	W2	HPI4	well_4	F001	26	Z distribution	-0.15649	-0.11458	0.203268
SLC25A17	W2	HPI4	well_4	F002	30	Z distribution	-0.52458	-0.369	0.51566
SLC25A17	W2	HPI4	well_4	F003	50	Z distribution	-0.22534	-0.1577	0.265446
SLC25A17	W2	HPI4	well_4	F004	25	Z distribution	-0.19745	-0.08354	0.243316
SLC25A17	W2	HPI4	well_4	F005	24	Z distribution	-0.4198	-0.32318	0.373517
SLC25A17	W2	HPI4	well_4	F006	22	Z distribution	-0.19889	-0.15203	0.195902
SLC25A17	W2	HPI4	well_5	F001	59	Z distribution	-0.25084	-0.16536	0.357157
SLC25A17	W2	HPI4	well_5	F002	23	Z distribution	-0.45294	-0.39122	0.306013
SLC25A17	W2	HPI4	well_5	F003	10	Z distribution	-0.21406	-0.17522	0.236379
SLC25A17	W2	HPI4	well_5	F004	14	Z distribution	-0.73015	-0.75034	0.381228
SLC25A17	W2	HPI4	well_5	F005	28	Z distribution	-0.24751	-0.17917	0.322306
SLC25A17	W2	HPI4	well_5	F006	41	Z distribution	-0.22298	-0.16573	0.341047
SLC25A17	W2	PGE2	well_1	F001	29	Z distribution	-0.53757	-0.54078	0.308577
SLC25A17	W2	PGE2	well_1	F002	164	Z distribution	-0.51259	-0.4977	0.303271
SLC25A17	W2	PGE2	well_1	F003	79	Z distribution	-0.5105	-0.53328	0.239785
SLC25A17	W2	PGE2	well_1	F004	71	Z distribution	-0.45077	-0.43179	0.288173
SLC25A17	W2	PGE2	well_1	F005	188	Z distribution	-0.42978	-0.42503	0.328703
SLC25A17	W2	PGE2	well_1	F006	224	Z distribution	-0.42786	-0.44066	0.294685
SLC25A17	W2	PGE2	well_2	F001	85	Z distribution	-0.59034	-0.60261	0.259852
SLC25A17	W2	PGE2	well_2	F002	178	Z distribution	-0.43887	-0.45631	0.318965
SLC25A17	W2	PGE2	well_2	F003	62	Z distribution	-0.35632	-0.36507	0.246678
SLC25A17	W2	PGE2	well_2	F004	142	Z distribution	-0.35468	-0.31024	0.269296
SLC25A17	W2	PGE2	well_2	F005	176	Z distribution	-0.38206	-0.36798	0.27031
SLC25A17	W2	PGE2	well_2	F006	184	Z distribution	-0.32719	-0.32969	0.269884
SLC25A17	W2	PGE2	well_3	F001	142	Z distribution	-0.47389	-0.45265	0.267866
SLC25A17	W2	PGE2	well_3	F002	76	Z distribution	-0.40923	-0.42769	0.287587
SLC25A17	W2	PGE2	well_3	F003	188	Z distribution	-0.32979	-0.3327	0.297293
SLC25A17	W2	PGE2	well_3	F004	107	Z distribution	-0.33028	-0.28802	0.27027
SLC25A17	W2	PGE2	well_3	F005	63	Z distribution	-0.42457	-0.42003	0.248743
SLC25A17	W2	PGE2	well_3	F006	93	Z distribution	-0.34498	-0.35847	0.318011
SLC25A17	W2	PGE2	well_4	F001	287	Z distribution	-0.3907	-0.39917	0.305716
SLC25A17	W2	PGE2	well_4	F002	258	Z distribution	-0.53328	-0.50638	0.316003
SLC25A17	W2	PGE2	well_4	F003	344	Z distribution	-0.42784	-0.43852	0.338497
SLC25A17	W2	PGE2	well_4	F004	275	Z distribution	-0.24876	-0.2488	0.265453
SLC25A17	W2	PGE2	well_4	F005	216	Z distribution	-0.26283	-0.26216	0.294682
SLC25A17	W2	PGE2	well_4	F006	279	Z distribution	-0.27681	-0.29046	0.305483

SLC25A17	W2	PGE2	well_5	F001	190	Z distribution	-0.49565	-0.46701	0.32113
SLC25A17	W2	PGE2	well_5	F002	287	Z distribution	-0.72763	-0.73085	0.378022
SLC25A17	W2	PGE2	well_5	F003	153	Z distribution	-0.36467	-0.34914	0.280247
SLC25A17	W2	PGE2	well_5	F004	114	Z distribution	-0.41449	-0.41595	0.303405
SLC25A17	W2	PGE2	well_5	F005	126	Z distribution	-0.37681	-0.3555	0.270998
SLC25A17	W2	PGE2	well_5	F006	342	Z distribution	-0.29968	-0.27106	0.28576
SLC25A17	W3	HPI4	well_1	F002	10	Z distribution	-0.2828	-0.1787	0.300886
SLC25A17	W3	HPI4	well_1	F003	15	Z distribution	-0.41816	-0.44664	0.306137
SLC25A17	W3	HPI4	well_1	F004	7	Z distribution	-0.07646	-0.15071	0.182077
SLC25A17	W3	HPI4	well_1	F005	5	Z distribution	-0.05153	-0.0636	0.046387
SLC25A17	W3	HPI4	well_1	F006	6	Z distribution	-0.0269	0.001797	0.097435
SLC25A17	W3	HPI4	well_2	F001	5	Z distribution	-0.13746	-0.14527	0.065167
SLC25A17	W3	HPI4	well_2	F002	5	Z distribution	-0.17951	-0.19876	0.087342
SLC25A17	W3	HPI4	well_2	F003	1	Z distribution	-0.25866	-0.25866	NA
SLC25A17	W3	HPI4	well_2	F004	15	Z distribution	-0.27908	-0.21864	0.253131
SLC25A17	W3	HPI4	well_2	F006	7	Z distribution	-0.499	-0.63662	0.393284
SLC25A17	W3	HPI4	well_3	F001	7	Z distribution	-0.24219	-0.19114	0.214027
SLC25A17	W3	HPI4	well_3	F002	15	Z distribution	-0.20799	-0.13409	0.30635
SLC25A17	W3	HPI4	well_3	F003	5	Z distribution	-0.14481	-0.11748	0.097899
SLC25A17	W3	HPI4	well_3	F004	3	Z distribution	-0.23838	-0.23838	NA
SLC25A17	W3	HPI4	well_3	F005	9	Z distribution	-0.31595	-0.33229	0.134821
SLC25A17	W3	HPI4	well_3	F006	29	Z distribution	-0.26914	-0.2142	0.196768
SLC25A17	W3	HPI4	well_4	F001	13	Z distribution	-0.12546	-0.1165	0.19717
SLC25A17	W3	HPI4	well_4	F002	11	Z distribution	-0.29998	-0.31986	0.24151
SLC25A17	W3	HPI4	well_4	F003	8	Z distribution	-0.88095	-0.96835	0.469594
SLC25A17	W3	HPI4	well_4	F004	5	Z distribution	-0.34427	-0.1941	0.336626
SLC25A17	W3	HPI4	well_4	F005	9	Z distribution	-0.17867	-0.16679	0.146854
SLC25A17	W3	HPI4	well_4	F006	8	Z distribution	-0.19492	-0.17701	0.266751
SLC25A17	W3	HPI4	well_5	F001	5	Z distribution	-0.30517	-0.33234	0.179091
SLC25A17	W3	HPI4	well_5	F002	3	Z distribution	0.023115	0.023115	NA
SLC25A17	W3	HPI4	well_5	F004	5	Z distribution	-0.17762	-0.15521	0.146375
SLC25A17	W3	HPI4	well_5	F006	33	Z distribution	-0.20922	-0.15923	0.29054
SLC25A17	W3	PGE2	well_1	F001	104	Z distribution	-0.53294	-0.50487	0.342708
SLC25A17	W3	PGE2	well_1	F002	108	Z distribution	-0.76587	-0.80431	0.41775
SLC25A17	W3	PGE2	well_1	F003	172	Z distribution	-0.6646	-0.73222	0.405188
SLC25A17	W3	PGE2	well_1	F004	88	Z distribution	-0.5859	-0.60048	0.366551
SLC25A17	W3	PGE2	well_1	F005	133	Z distribution	-0.49452	-0.49942	0.417843
SLC25A17	W3	PGE2	well_1	F006	204	Z distribution	-0.74517	-0.76324	0.392998
SLC25A17	W3	PGE2	well_2	F001	149	Z distribution	-0.29517	-0.3005	0.284819
SLC25A17	W3	PGE2	well_2	F002	96	Z distribution	-0.41963	-0.43078	0.280742
SLC25A17	W3	PGE2	well_2	F003	70	Z distribution	-0.31094	-0.27935	0.247174
SLC25A17	W3	PGE2	well_2	F004	90	Z distribution	-0.49347	-0.46946	0.321609
SLC25A17	W3	PGE2	well_2	F005	209	Z distribution	-0.54268	-0.5716	0.315551
SLC25A17	W3	PGE2	well_2	F006	339	Z distribution	-0.43944	-0.47604	0.365115
SLC25A17	W3	PGE2	well_3	F001	238	Z distribution	-0.3098	-0.32705	0.299021
SLC25A17	W3	PGE2	well_3	F002	188	Z distribution	-0.52878	-0.57457	0.293934
SLC25A17	W3	PGE2	well_3	F003	204	Z distribution	-0.26473	-0.23706	0.259844
SLC25A17	W3	PGE2	well_3	F004	155	Z distribution	-0.46122	-0.44017	0.302411
SLC25A17	W3	PGE2	well_3	F005	372	Z distribution	-0.37153	-0.40628	0.328519

SLC25A17	W3	PGE2	well_3	F006	197	Z distribution	-0.39522	-0.41382	0.305482
SLC25A17	W3	PGE2	well_4	F001	239	Z distribution	-0.46118	-0.47099	0.279426
SLC25A17	W3	PGE2	well_4	F002	160	Z distribution	-0.58256	-0.56988	0.288155
SLC25A17	W3	PGE2	well_4	F003	93	Z distribution	-0.42395	-0.42765	0.282638
SLC25A17	W3	PGE2	well_4	F004	228	Z distribution	-0.4242	-0.43645	0.297659
SLC25A17	W3	PGE2	well_4	F005	201	Z distribution	-0.49969	-0.48634	0.292699
SLC25A17	W3	PGE2	well_4	F006	175	Z distribution	-0.41592	-0.37194	0.289184
SLC25A17	W3	PGE2	well_5	F001	139	Z distribution	-0.54368	-0.55453	0.322275
SLC25A17	W3	PGE2	well_5	F002	182	Z distribution	-0.43089	-0.44241	0.303272
SLC25A17	W3	PGE2	well_5	F003	241	Z distribution	-0.27199	-0.2269	0.301933
SLC25A17	W3	PGE2	well_5	F004	205	Z distribution	-0.26985	-0.27236	0.283145
SLC25A17	W3	PGE2	well_5	F005	314	Z distribution	-0.20437	-0.2026	0.283588
SLC25A17	W3	PGE2	well_5	F006	252	Z distribution	-0.34071	-0.34269	0.275691
SLC25A17	W4	HPI4	well_1	F001	7	Z distribution	-0.23433	-0.17395	0.175324
SLC25A17	W4	HPI4	well_1	F002	1	Z distribution	-0.47701	-0.47701	NA
SLC25A17	W4	HPI4	well_1	F003	1	Z distribution	-0.90073	-0.90073	NA
SLC25A17	W4	HPI4	well_1	F006	1	Z distribution	-0.40851	-0.40851	NA
SLC25A17	W4	HPI4	well_2	F003	1	Z distribution	-0.40008	-0.40008	NA
SLC25A17	W4	HPI4	well_3	F001	13	Z distribution	-0.15738	-0.07095	0.232798
SLC25A17	W4	HPI4	well_3	F003	1	Z distribution	-0.11268	-0.11268	NA
SLC25A17	W4	HPI4	well_3	F004	1	Z distribution	-0.08072	-0.08072	NA
SLC25A17	W4	HPI4	well_4	F001	3	Z distribution	0.230459	0.230459	NA
SLC25A17	W4	HPI4	well_4	F002	1	Z distribution	-0.52341	-0.52341	NA
SLC25A17	W4	HPI4	well_5	F001	1	Z distribution	-1.01628	-1.01628	NA
SLC25A17	W4	HPI4	well_5	F002	1	Z distribution	0.439899	0.439899	NA
SLC25A17	W4	HPI4	well_5	F004	1	Z distribution	-0.10365	-0.10365	NA
SLC25A17	W4	HPI4	well_5	F006	4	Z distribution	-1.15031	-1.15031	0.427046
SLC25A17	W4	PGE2	well_1	F001	222	Z distribution	-0.35328	-0.35254	0.227037
SLC25A17	W4	PGE2	well_1	F002	273	Z distribution	-0.41686	-0.38385	0.298672
SLC25A17	W4	PGE2	well_1	F003	246	Z distribution	-0.39692	-0.38741	0.256753
SLC25A17	W4	PGE2	well_1	F004	144	Z distribution	-0.35121	-0.36381	0.277862
SLC25A17	W4	PGE2	well_1	F005	267	Z distribution	-0.33234	-0.36089	0.257033
SLC25A17	W4	PGE2	well_1	F006	208	Z distribution	-0.30246	-0.29584	0.2439
SLC25A17	W4	PGE2	well_2	F001	216	Z distribution	-0.32903	-0.29463	0.293402
SLC25A17	W4	PGE2	well_2	F002	215	Z distribution	-0.40754	-0.42774	0.236817
SLC25A17	W4	PGE2	well_2	F003	192	Z distribution	-0.38196	-0.38779	0.279903
SLC25A17	W4	PGE2	well_2	F004	202	Z distribution	-0.40928	-0.40697	0.265336
SLC25A17	W4	PGE2	well_2	F005	251	Z distribution	-0.43815	-0.44268	0.263298
SLC25A17	W4	PGE2	well_2	F006	200	Z distribution	-0.35596	-0.35184	0.262963
SLC25A17	W4	PGE2	well_3	F001	280	Z distribution	-0.3856	-0.39538	0.265894
SLC25A17	W4	PGE2	well_3	F002	258	Z distribution	-0.34258	-0.31448	0.282336
SLC25A17	W4	PGE2	well_3	F003	204	Z distribution	-0.22355	-0.22146	0.245917
SLC25A17	W4	PGE2	well_3	F004	203	Z distribution	-0.35167	-0.33849	0.279801
SLC25A17	W4	PGE2	well_3	F005	227	Z distribution	-0.23718	-0.1874	0.296069
SLC25A17	W4	PGE2	well_3	F006	291	Z distribution	-0.20819	-0.19763	0.28016
SLC25A17	W4	PGE2	well_4	F001	34	Z distribution	-0.40713	-0.40608	0.263899
SLC25A17	W4	PGE2	well_4	F002	254	Z distribution	-0.33467	-0.31838	0.279817
SLC25A17	W4	PGE2	well_4	F003	22	Z distribution	-0.38905	-0.40216	0.25903
SLC25A17	W4	PGE2	well_4	F004	31	Z distribution	-0.37477	-0.42099	0.201006

SLC25A17	W4	PGE2	well_4	F005	84	Z distribution	-0.33398	-0.35422	0.24973
SLC25A17	W4	PGE2	well_4	F006	43	Z distribution	-0.3904	-0.40972	0.275307
SLC25A17	W4	PGE2	well_5	F001	72	Z distribution	-0.30558	-0.31284	0.205286
SLC25A17	W4	PGE2	well_5	F002	46	Z distribution	-0.49302	-0.47411	0.357809
SLC25A17	W4	PGE2	well_5	F003	13	Z distribution	-0.67135	-0.61531	0.226101
SLC25A17	W4	PGE2	well_5	F004	39	Z distribution	-0.31512	-0.35209	0.212351
SLC25A17	W4	PGE2	well_5	F005	33	Z distribution	-0.38668	-0.41739	0.171377
SLC25A17	W4	PGE2	well_5	F006	108	Z distribution	-0.24113	-0.21618	0.279169

Table S13

Description	endog	Treatment	pval_const	pval_Week	pval_Week_squared	coef_const	coef_Week	coef_Week_squared	diff_last-first_week	rate change	min/max_week
Avg cell	Cell Volume	PGE2	0	4.12E-16	4.43E-16	1044.897774	-149.4650343	29.39418737	-7.482292418	ACC	2.542425011
Avg cell	Cell Volume	HPI4	8.38E-225	4.70E-07	9.74E-12	998.7578213	-143.4138161	38.16692436	142.2624171	ACC	1.878770932
Avg cell	Cell AR	PGE2	0	4.39E-34	3.10E-27	1.780987216	-0.176326181	0.030829059	-0.066542652	ACC	2.859739874
Avg cell	Cell AR	HPI4	0	0.763530627	0.000961992	1.627045123	-0.004658164	0.010063708	0.136981124	ACC	0.231433766
Avg cell	Cell Area	PGE2	0	1.38E-69	1.61E-56	376.7037436	-127.356116	22.52597193	-44.17876912	ACC	2.82687283
Avg cell	Cell Area	HPI4	2.06E-144	0.265304145	4.66E-05	272.9458347	-10.83995487	7.800930817	84.49409764	ACC	0.694785989
Avg nuclei	Nucleus Volume	PGE2	0	1.85E-228	1.28E-115	506.5642342	-138.0736177	19.25287621	-125.42771	ACC	3.585791967
Avg nuclei	Nucleus Volume	HPI4	0	6.84E-73	9.77E-45	489.857369	-131.678589	20.14629286	-92.84137403	ACC	3.268060032
Avg nuclei	Nucleus Area	PGE2	0	6.31E-120	8.56E-75	140.7267506	-46.38435702	7.176207402	-31.50996002	ACC	3.231815527
Avg nuclei	Nucleus Area	HPI4	0	2.62E-09	3.54E-09	112.4134894	-13.53786069	2.643020473	-0.968274972	ACC	2.561058612
Avg nuclei	Nucleus AR	PGE2	0	5.89E-41	2.32E-29	1.749169106	-0.183157352	0.030268503	-0.095444514	ACC	3.025543637
Avg nuclei	Nucleus AR	HPI4	0	0.001189915	0.284252291	1.53681949	0.068895938	-0.004481063	0.139471865	DEC	7.687454184
Avg cell - ACTB	Avg. Volume	PGE2	2.69E-06	2.27E-09	3.06E-07	0.623513334	0.724451549	-0.122158579	0.340975963	DEC	2.965209465
Avg cell - ACTB	Avg. Volume	HPI4	0.043765445	0.152050866	0.080510353	0.883648212	0.586474717	-0.142203226	-0.373624236	DEC	2.062100608
Avg cell - MYH10	Avg. Volume	PGE2	0.003651279	1.53E-26	1.49E-15	0.342452789	1.145930395	-0.168808193	0.905668296	DEC	3.394178853
Avg cell - MYH10	Avg. Volume	HPI4	1.07E-54	0.000501587	0.000880491	1.590752001	-0.324238232	0.06101417	-0.057502141	ACC	2.657073185
Avg cell - ACTB	Count/cell	PGE2	0	1.77E-46	3.68E-29	5.193038462	-0.844638752	0.133687916	-36.30212234	ACC	3.158994382
Avg cell - ACTB	Count/cell	HPI4	0	7.25E-08	0.000231428	4.679244931	-0.312544901	0.043074496	-20.79886184	ACC	3.627957725
Avg cell - MYH10	Count/cell	PGE2	0	3.97E-34	4.18E-24	5.190167193	-0.609844614	0.101440823	-28.61238851	ACC	3.005913188
Avg cell - MYH10	Count/cell	HPI4	0	8.71E-14	3.70E-17	4.946431627	-0.338046398	0.074822921	12.35507694	ACC	2.258976214
Avg organelle - FBL	Count/cell	PGE2	4.11E-06	0.795821005	0.757271856	1.327428017	-0.070543041	-0.017036349	-1.289540631	DEC	-2.070368533
Avg organelle - FBL	Count/cell	HPI4	3.36E-08	0.072361903	0.213693307	1.659617274	-0.520661665	0.072806883	-1.259519217	ACC	3.575634911
Avg organelle - FBL	Z distribution	PGE2	1.84E-30	0.020863526	0.055810339	-0.576276474	0.106011269	-0.017308858	0.058400933	DEC	3.062341464
Avg organelle - FBL	Z distribution	HPI4	2.02E-71	1.14E-27	1.89E-23	-0.889652209	0.495684094	-0.089496073	0.144611193	DEC	2.769306404
Avg organelle - FBL	X/Y distribution	PGE2	2.31E-140	7.08E-09	4.59E-07	4.617532036	-0.967993483	0.16631681	-0.409228298	ACC	2.910089132
Avg organelle - FBL	X/Y distribution	HPI4	4.16E-43	2.94E-11	5.38E-18	5.55021384	-2.448800848	0.627984068	2.073358474	ACC	1.949731668
Avg organelle - DSP	Avg. Volume	PGE2	1.17E-10	6.48E-10	4.39E-08	0.233443514	0.204199349	-0.035619738	0.078301984	DEC	2.866379191
Avg organelle - DSP	Avg. Volume	HPI4	1.35E-40	0.125844195	0.026764373	0.431046932	-0.045121291	0.012854161	0.057448544	ACC	1.755123904
Avg organelle - GJA1	Avg. Volume	PGE2	0.001944052	0.163735489	0.248419522	1.324580002	-0.537873723	0.087390959	-0.302756785	ACC	3.077399137
Avg organelle - GJA1	Avg. Volume	HPI4	0.109951076	0.164917178	0.228311727	0.318883972	0.254193319	-0.043561451	0.109158199	DEC	2.917640665
Avg organelle - DSP	Count/cell	PGE2	2.39E-179	9.00E-17	4.06E-05	2.611725453	0.63062274	-0.058019897	42.92975263	DEC	5.434538595
Avg organelle - DSP	Count/cell	HPI4	8.30E-198	0.03034839	0.085596049	3.024386565	0.195704793	-0.030363881	3.416840052	DEC	3.222657804
Avg organelle - GJA1	Count/cell	PGE2	0	2.38E-23	1.26E-12	4.424237457	-1.005223166	0.146370742	-19.78414213	ACC	3.433825474
Avg organelle - GJA1	Count/cell	HPI4	1.07E-273	0.000880597	4.20E-05	3.50384365	-0.302598037	0.07300604	5.442827821	ACC	2.072417815
Avg organelle - SEC61B	Avg. Volume	PGE2	2.29E-10	0.001775014	0.020353526	1.492325213	0.671067389	-0.098057456	0.542340321	DEC	3.421807039
Avg organelle - SEC61B	Avg. Volume	HPI4	1.92E-40	0.001723857	0.060832764	2.445464595	-0.525167431	0.061845741	-0.647816176	ACC	4.245784926
Avg organelle - ST6GAL1	Avg. Volume	PGE2	0.018953642	0.2083979	0.048876001	1.213785671	0.595071349	-0.184143451	-0.976937715	DEC	1.615782007
Avg organelle - ST6GAL1	Avg. Volume	HPI4	1.55E-09	0.00491073	0.048504945	3.193882717	-1.375212416	0.192463978	-1.238677574	ACC	3.572648836
Avg organelle - TOM20	Avg. Volume	PGE2	1.06E-11	2.98E-11	2.05E-10	2.289302446	2.042147713	-0.384521241	0.358624531	DEC	2.655442012
Avg organelle - TOM20	Avg. Volume	HPI4	5.58E-89	4.91E-18	2.58E-15	5.97245629	-2.358077921	0.424234446	-0.710717078	ACC	2.779215532
Avg organelle - SEC61B	Count/cell	PGE2	0	2.36E-75	2.65E-42	5.499997585	-1.189889118	0.181038776	-51.24367943	ACC	3.286282492
Avg organelle - SEC61B	Count/cell	HPI4	0	0.000726724	0.000737763	4.63465451	-0.168191643	0.033099314	-0.724557115	ACC	2.540711946
Avg organelle - LAMP1	Avg. Volume	PGE2	1.47E-70	0.353355979	0.470185241	0.434684571	0.020724196	-0.003174807	0.014550477	DEC	3.263850916
Avg organelle - LAMP1	Avg. Volume	HPI4	5.78E-261	5.98E-71	3.10E-49	0.726333805	-0.341960425	0.055756337	-0.189536216	ACC	3.066561057
Avg organelle - LC3B	Avg. Volume	PGE2	2.64E-101	4.78E-13	3.95E-13	0.629203566	-0.194259767	0.038380231	-0.007075831	ACC	2.530726892
Avg organelle - LC3B	Avg. Volume	HPI4	3.11E-17	0.078884142	0.177466723	0.587830707	-0.114836913	0.01800478	-0.074439038	ACC	3.189067367
Avg organelle - RAB5	Avg. Volume	PGE2	8.91E-51	0.019485743	0.043388572	0.673949256	-0.095836067	0.016314257	-0.042794345	ACC	2.937187601
Avg organelle - RAB5	Avg. Volume	HPI4	0.641915297	0.005276647	0.004607825	0.103180836	0.582580967	-0.117164281	-0.009721311	DEC	2.486171396
Avg organelle - SLC25A17	Avg. Volume	PGE2	9.98E-63	0.01116827	0.007896878	0.377779437	0.052313664	-0.010782498	-0.004796485	DEC	2.425860029
Avg organelle - SLC25A17	Avg. Volume	HPI4	4.99E-06	0.115522421	0.090213391	0.33242948	0.107166831	-0.02349188	-0.030877713	DEC	2.280933438

Table S14

description	distribution	endog	treatment	week1	week2	week3	week4	rsquared	AIC	week1_lower	week2_lower	week3_lower	week4_lower	week1_upper	week2_upper	week3_upper	week4_upper	param_const	param_const_lower	param_const_upper	param_week	param_week_lower	param_week_upper	param_week_squared	param_week_squared_lower	param_week_squared_upper
Avg cell	gaussian	Cell Volume	PGE2	924.827	863.544	861.05	917.345	0.42626	1226.57	913.5638871	851.1971942	849.967795	903.7048431	934.165562	875.1386454	872.1131005	932.8007635	1044.897774	1008.386845	1081.034678	-149.465034	-186.8082501	-112.4049433	29.39418737	21.93090369	36.9829814
Avg cell	gaussian	Cell Volume	HPI4	893.511	864.598	912.019	1035.77	0.67782	1331.65	878.8671846	850.2905771	896.9516056	1008.938275	905.6414646	878.8798006	927.2296997	1063.809328	998.7578213	951.5418772	1045.813912	-143.413816	-192.2645589	-96.36492049	38.16692436	28.27100551	48.59262031
Avg cell	gaussian	Cell AR	PGE2	1.63549	1.55165	1.52947	1.56895	0.79894	-488.331	1.626794549	1.543176399	1.5200983	1.558773756	1.643764605	1.561022186	1.537820286	1.579997977	1.780987216	1.753343899	1.809883275	-0.17632618	-0.204322708	-0.149117482	0.030829059	0.025121514	0.036475217
Avg cell	gaussian	Cell AR	HPI4	1.63245	1.65798	1.70364	1.76943	0.91182	-472.355	1.624529526	1.649252629	1.694499829	1.756839753	1.638809069	1.667238996	1.712933841	1.784004028	1.627045123	1.600129456	1.65292644	-0.00465816	-0.031784174	0.023303589	0.010063708	0.004008555	0.015908601
Avg cell	gaussian	Cell Area	PGE2	271.874	212.095	197.369	227.695	0.9596	1002.49	266.2762677	207.4543437	192.4028211	224.2985106	276.0248204	217.1158008	202.4452014	232.3178269	376.7037436	360.6766405	389.9222558	-127.356116	-141.0926692	-112.3760917	22.52597193	19.55130724	25.17898387
Avg cell	gaussian	Cell Area	HPI4	269.907	282.47	310.634	354.401	0.90835	1074.04	265.1801483	276.8950266	304.9454938	345.2646478	274.2804555	288.1864172	316.5004307	363.8022209	272.9458347	255.6270906	289.7898427	-10.8399549	-28.63421398	7.148908103	7.800930817	3.903316442	11.56164979
Avg nuclei	gaussian	Nucleus Volume	PGE2	387.743	307.429	265.619	262.316	1	876.837	384.2840915	305.0673444	263.4045649	259.066446	391.0713264	309.7152598	267.7297707	265.7034473	506.5642342	496.992192	515.7882742	-138.073618	-146.6831173	-129.8607768	19.25287621	17.55535891	20.95944366
Avg nuclei	gaussian	Nucleus Volume	HPI4	378.325	307.085	276.138	285.484	0.99847	1004.79	374.5877428	302.9665254	272.0640598	279.0940472	382.2119301	311.0008275	280.0998353	293.3017083	489.857369	477.4576809	503.2142194	-131.678589	-145.8114579	-119.0074977	20.14629286	17.5314581	23.21263409
Avg nuclei	gaussian	Nucleus Area	PGE2	101.519	76.6629	66.1595	70.0086	0.99997	693.36	100.0766524	75.43699758	64.85945	69.16334762	102.6259942	78.01616219	67.47112506	71.11934757	140.7267506	136.3761571	144.545224	-46.384357	-50.15579032	-42.2553256	7.176207402	6.395385864	7.90338312
Avg nuclei	gaussian	Nucleus Area	HPI4	101.519	95.9098	95.5871	100.55	0.25639	725.104	100.2389638	94.63263913	94.29336686	98.59536835	102.72735	97.17072224	96.86693714	102.6992071	112.4134894	108.2842866	116.6405674	-13.5378607	-17.72297392	-9.508248536	2.643020473	1.817568823	3.519483515
Avg nuclei	gaussian	Nucleus AR	PGE2	1.59628	1.50393	1.47211	1.50084	0.91885	-502.313	1.58797813	1.496005474	1.463865087	1.490494448	1.60401752	1.5120277	1.480204826	1.511609466	1.749169106	1.723865072	1.773780645	-0.18315735	-0.207211392	-0.158902042	0.030268503	0.025271416	0.03516193
Avg nuclei	gaussian	Nucleus AR	HPI4	1.60123	1.65669	1.70318	1.74071	0.72625	-396.31	1.589370711	1.644980441	1.689960601	1.723453428	1.611555052	1.670491291	1.717198884	1.761371093	1.53681949	1.498873689	1.57696059	0.068895938	0.029835379	0.111266656	-0.004481063	-0.013235692	0.003815688
Avg cell - ACTB	gaussian	Avg. Volume	PGE2	1.22581	1.58378	1.69744	1.56678	0.36562	21.4766	1.166012671	1.50952627	1.624819286	1.468493892	1.288061866	1.660491319	1.771982385	1.666917302	0.623513334	0.408260578	0.826659553	0.724451549	0.513119129	0.963163653	-0.122158579	-0.172434858	-0.077529682
Avg cell - ACTB	gaussian	Avg. Volume	HPI4	1.32792	1.48778	1.36324	0.9543	0.0524	279.387	1.225054108	1.155535854	1.085097078	0.750705077	1.475872608	1.891100541	1.70415755	1.160568355	0.883648212	0.240188679	1.36407568	0.586474717	-0.107727235	1.477599856	-0.142203226	-0.333212882	0.012831954
Avg cell - MYH10	gaussian	Avg. Volume	PGE2	1.31957	1.95908	2.26097	2.22524	0.92943	-7.35549	1.26923091	1.894161313	2.199004811	2.137422903	1.381532565	2.025345611	2.31766423	2.310297458	0.342452789	0.162951948	0.51848217	1.145930395	0.959017689	1.344910647	-0.168808193	-0.209840477	-0.127343748
Avg cell - MYH10	gaussian	Avg. Volume	HPI4	1.32753	1.18633	1.16716	1.27003	0.09867	-41.6266	1.275167227	1.136693546	1.119427436	1.174208442	1.379504001	1.234438711	1.214654536	1.356773311	1.590752001	1.430064329	1.761981858	-0.32423823	-0.494248811	-0.165567049	0.06101417	0.025508022	0.095685173
Avg cell - ACTB	poisson	Count/cell	PGE2	88.4191	56.7419	47.5753	52.1169	0.9801	915.722	83.43782119	54.08556089	45.31712239	49.65837273	92.92635478	59.5853901	50.02410536	54.79862398	5.193038462	5.021188336	5.356364165	-0.84463875	-1.001973541	-0.693616645	0.133687916	0.103228861	0.164090712
Avg cell - ACTB	poisson	Count/cell	HPI4	82.2509	68.4739	62.1332	61.4521	0.65747	1717.43	76.97379993	57.94321507	53.24449334	54.72768551	87.42277738	81.27779507	72.1938401	67.74928809	4.679244931	4.370482155	5.001033796	-0.3125449	-0.725291155	0.098873265	0.043074496	-0.043265587	0.130324361
Avg cell - MYH10	poisson	Count/cell	PGE2	107.96	79.5385	71.7796	79.3479	0.88405	962.735	101.6137037	76.69128063	69.04969679	76.0101516	113.6681275	82.60427991	74.48250722	83.0381254	5.190167193	5.032057347	5.336188016	-0.60984461	-0.737140961	-0.470793662	0.101440823	0.076098566	0.126171307
Avg cell - MYH10	poisson	Count/cell	HPI4	108.116	96.5084	100.053	120.471	0.54466	1480.13	102.8190409	90.6492963	94.42949103	107.2476248	113.0931413	102.7282572	105.6926228	133.4266485	4.946431627	4.767028424	5.123222894	-0.3380464	-0.533242784	-0.143174774	0.074822921	0.0312902	0.116080029
Avg organelle - FBL	poisson	Count/cell	PGE2	3.45509	3.05933	2.61816	2.16555	0.07924	355.076	3.399818981	3.011446974	2.5757008	2.095874302	3.501664249	3.113743651	2.658769341	2.243395636	1.327428017	1.273566246	1.379248541	-0.07054304	-0.12683128	-0.013785398	-0.017036349	-0.029372394	-0.00436221
Avg organelle - FBL	poisson	Count/cell	HPI4	3.3594	2.48313	2.12314	2.09988	0.09394	344.376	3.282206056	2.38166608	2.028654324	2.019951044	3.43258469	2.581283837	2.212188764	2.186672733	1.659617274	1.55882521	1.754794744	-0.52066166	-0.631524068	-0.411732479	0.072806883	0.051126938	0.094788373
Avg organelle - FBL	gaussian	Z distribution	PGE2	-0.48757	-0.43349	-0.41402	-0.42917	0.07709	-210.419	-0.50589587	-0.457890682	-0.4350986	-0.477010604	-0.468420001	-0.409949833	-0.393651631	-0.374261555	-0.57627647	-0.640637182	-0.506018796	0.106011269	0.031026773	0.179218055	-0.017308858	-0.033893511	2.93E-05
Avg organelle - FBL	gaussian	Z distribution	HPI4	-0.48346	-0.25627	-0.20806	-0.33885	0.68338	-212.574	-0.500466146	-0.286208779	-0.238210131	-0.379329407	-0.465342834	-0.229224059	-0.179130834	-0.29686177	-0.88965221	-0.964796718	-0.809135839	0.495684094	0.407751669	0.580353567	-0.089496073	-0.107688057	-0.070902051
Avg organelle - FBL	gaussian	X/Y distribution	PGE2	3.81586	3.34681	3.2104	3.40663	0.32766	97.3514	3.713496099	3.260515676	3.132296227	3.256401874	3.931830266	3.43324594	3.295944453	3.574649425	4.617532036	4.312601462	4.934894997	-0.96799348	-1.285109704	-0.67681014	0.16631681	0.10686947	0.232295834
Avg organelle - FBL	gaussian	X/Y distribution	HPI4	3.7294	3.16455	3.85567	5.80276	0.78889	285.273	3.574103251	2.970026921	3.664888512	5.400976457	3.919017776	3.336007761	4.027658711	6.249333319	5.55021384	4.999346564	6.159421035	-2.44880085	-3.124884428	-1.875963495	0.627984068	0.497860392	0.783866297
Avg organelle - DSP	gaussian	Avg. Volume	PGE2	0.40202	0.49936	0.52546	0.48033	0.34141	-290.37	0.389939893	0.476505587	0.503075875	0.45602779	0.416466409	0.521295819	0.547326739	0.503890417	0.233443514	0.181759462	0.289184193	0.204199349	0.14124211	0.263401922	-0.035619738	-0.048339311	-0.022765405
Avg organelle - DSP	gaussian	Avg. Volume	HPI4	0.39878	0.39222	0.41137	0.45623	0.14326	-317.829	0.386682725	0.372599928	0.392533392	0.429269071	0.41151492	0.408888951	0.428911546	0.483745969	0.431046932	0.383278634	0.482107554	-0.04512129	-0.101863983	0.006090253	0.012854161	0.002410585	0.024796777
Avg organelle - GJA1	gaussian	Avg. Volume	PGE2	0.8741	0.5984	0.49748	0.57134	0.02909	291.612	0.504863609	0.522576543	0.391056518	0.500061595	1.530182199	0.710774713	0.576014512	0.647882282	1.324580002	0.42994301	2.881631772	-0.53787372	-1.61754829	0.09544609	0.087390959	-0.018765294	0.260151677
Avg organelle - GJA1	gaussian	Avg. Volume	HPI4	0.52952	0.65302	0.68941	0.63867	0.0238	115.276	0.417933465	0.543878059	0.577634024	0.571207493	0.652590661	0.791987183	0.82535299	0.704229969	0.318883972	-0.058915375	0.66653587	0.254193319	-0.080717034	0.632846184	-0.043561451	-0.118230793	0.018883227
Avg organelle - DSP	poisson	Count/cell	PGE2	24.1511	38.1258	53.5928	67.0808	0.99729	1196.98	22.33380337	34.64168616	48.8175133	60.04030959	26.10971718	42.80846083	59.06579742	73.84719855	2.611725453	2.356442901	2.877193117	0.63062274	0.342501069	0.905362837	-0.058019897	-0.118033715	0.001042389
Avg organelle - DSP	poisson	Count/cell	HPI4	24.2818	26.9596	28.1691	27.6986	0.08237	945.237	22.79153692	24.49656905	25.32889847	24.70191797	25.93825672	29.5515657	30.96042499	30.57580861	3.024386565	2.773947811	3.298491549	0.195704793	-0.085733512	0.469136491	-0.030363881	-0.087831769	0.025259349
Avg organelle - GJA1	poisson	Count/cell	PGE2	35.3531	20.071	15.2702	15.5689	0.93701	1299.17	27.18727981	17.87900124	13.38492465	12.75176094	45.21192307	22.36851364	17.32058588	18.83565606	4.424237457	3.754134358	5.113788198	-1.00522317	-1.571562044	-0.44198545	0.146370742	0.040550847	0.246378769
Avg organelle - GJA1	poisson	Count/cell	HPI4	26.4234	24.3047	25.8705	31.8663	0.24944	1450.64	21.0461546	21.0749183	22.1540413	28.03793915	33.81870682	27.8704532	29.64166808	36.13651006	3.50384365	2.902739745	4.129319482	-0.30259804	-0.815357095	0.197903212	0.07300604	-0.018261555	0.167361637
Avg organelle - SEC61B	gaussian	Avg. Volume	PGE2	2.06534	2.44223	2.62301	2.60768	0.20997	158.726	1.919763236	2.326234702	2.508220479	2.412213993	2.205183253	2.56063022	2.745033031	2.790285412	1.492325213	1.065144442	1.910448875	0.671067389	0.291836709	1.070797635	-0.098057456	-0.181230885	-0.015940234
Avg organelle - SEC61B	gaussian	Avg. Volume	HPI4	1.98214	1.64251	1.42657	1.33433	0.37868	99.2304	1.851133976	1.550446953	1.326153428	1.227634524	2.112151679	1.734452665	1.528674903	1.452583075	2.445464595	2.07212688	2.829259862	-0.52516743	-0.877497003	-0.181675405	0.061845741	-0.00180525	0.128298648
Avg organelle - ST6GAL1	gaussian	Avg. Volume	PGE2	1.62471	1.66735	1.34171	0.64778	0.14533	341.375	1.186000403	1.487697571	1.153543494	0.525991777	2.391616275	1.857002364	1.521512007	0.786190406	1.213785671	0.109043049	3.111526282	0.595071349	-0.735382205	1.488134523	-0.184143451	-0.340044684	0.037405736
Avg organelle - ST6GAL1	gaussian	Avg. Volume	HPI4	2.01113	1.21331	0.80042	0.77246	0.2183	319.336	1.509756965	1.05724073	0.636238651	0.645253801	2.706311075	1.39300112	0.962216562	0.90609333	3.193882717	1.95433692	4.844683393	-1.37521242	-2.55912841	-0.446952282	0.192463978	0.035695877	0.387191981
Avg organelle - TOM20	gaussian	Avg. Volume	PGE2	3.94693	4.83551	4.95505	4.30555	0.31465	244.71	3.777377266	4.674182735	4.778834009	4.049088169	4.111281131	5.027661048	5.14648058	4.58682331	2.289302446	1.728233834	2.843831253	2.042147713	1.503022939	2.603303447	-0.384521241	-0.504254683	-0.274954143
Avg organelle - TOM20	gaussian	Avg. Volume	HPI4	4.03861	2.95324	2.71633	3.3279	0.51562	215.878	3.870776408	2.79786802	2.565928525	3.089695065	4.194819694	3.104883543	2.869426897	3.579208857	5.97245629	5.458565188	6.460553312	-2.35807792	-2.866630012	-1.869173754	0.424234446	0.325213463	0.533856437
Avg organelle - SEC61B	poisson	Count/cell	PGE2	89.2237	46.7294	35.1519	37.9801	0.99972	824.922	84.59002082	44.97937452	33.77519645	36.43067571	93.4195337	48.76504853	36.6050961	39.80463895	5.499997585	5.355990288	5.640123692	-1.18988912	-1.327007068	-1.06106551	0.181038776	0.156302824	0.206986035
Avg organelle - SEC61B	poisson	Count/cell	HPI4	89.9777	83.9875	83.7615	89.2532	0.09137	1028.97	85.58381685	80.6218083	80.11902412	82.48088101	94.18763677	87.39665064	87.17445346	94.90634994	4.63465451	4.476834742	4.775649283	-0.16819164	-0.302576812	-0.02012664	0.033099314	0.003175284	0.060468157
Avg organelle - LAMP1	gaussian	Avg. Volume	PGE2	0.45223	0.46343	0.46828	0.46678	0.01719	-384.48	0.443163384	0.452169255	0.45606966	0.440774272	0.46139923	0.475106569	0.479249233	0.489676382	0.434684571	0.399676795	0.469386415	0.020724196	-0.016503601	0.05966441	-0.003174807	-0.011956277	0.005044793
Avg organelle - LAMP1	gaussian	Avg. Volume	HPI4	0.44013	0.26544	0.20226	0.25059	0.99111	-420.707	0.431208616	0.253358953	0.190414891	0.23777285	0.448718298	0.278170969	0.214526567	0.268062624	0.726333805	0.694121171	0.760462378	-0.34196043	-0.377910876	-0.305784243	0.055756337	0.048326227	0.063165591
Avg organelle - LC3B	gaussian	Avg. Volume	PGE2	0.47332	0.3942	0.39185	0.46625	0.35678	-340.121	0.450612358	0.378903051	0.376741221	0.449188057	0.493811745	0.410343049	0.407240217	0.483831044	0.629203566	0.566479453	0.689295171	-0.19425977	-0.247832095	-0.137867218	0.038380231	0.027590367	0.048010215
Avg organelle - LC3B	gaussian	Avg. Volume	HPI4	0.491	0.43018	0.40536	0.41656	0.06627	-122.074	0.468364631	0.396298002	0.363764163	0.346809323	0.511591888	0.470681394	0.451989258	0.496152542	0.587830707	0.46944946	0.695154727	-0.11483691	-0.232147631	0.014151331	0.01800478	-0.008600776	0.043747997
Avg organelle - RAB5	gaussian	Avg. Volume	PGE2	0.59443	0.54753	0.53327	0.55163	0.06463	-238.503	0.558483594	0.525309021	0.512988721	0.516098579	0.626689335	0.568046913	0.552401516	0.588852427	0.673949256	0.578537283	0.765035521	-0.09583607	-0.174630653	-0.01597648	0.016314257	0.000854217	0.031626932
Avg organelle - RAB5	gaussian	Avg. Volume	HPI4	0.5686	0.79969	0.79645	0.55888	0.07609	113.242	0.508702818	0.641174468	0.644604101	0.469428597	0.630061413	0.984430095	0.973400477	0.659709	0.103180836	-0.252687178	0.430538376	0.582580967	0.19194584	1.052411398	-0.117164281	-0.210443715	-0.03645812
Avg organelle - SLC25A17	gaussian	Avg. Volume	PGE2	0.41931	0.43928	0.43768	0.41451	0.05893	-403.631	0.405591901	0.42556411	0.424804532	0.398758069	0.431319569	0.452446679	0.450842763	0.427915855	0.377779437	0.332539223	0.416600816	0.052313664	0.015065626	0.094378275	-0.010782498	-0.018695914	-0.003359768
Avg organelle - SLC25A17	gaussian	Avg. Volume	HPI4	0.4161	0.4528	0.4425	0.38523	0.02946	-113.789	0.395104353	0.41542945	0.40715088	0.275167349	0.434995274	0.488748421	0.477364389	0.504783256	0.33242948	0.225555237	0.445233116	0.107166831	-0.033523166	0.236670453	-0.02349188	-0.054813645	0.009960313

Table S15

Week	Treatment	Organelles	ACTB	CETN2	CTNNB1	DSP	FBL	GJA1	LAMP1	LC3B	LMNB1	MYH10	RAB5	SLC25A17	ST6GAL1	SEC61B	TOM20	TJP1
W1	PGE2	ACTB	1	0.0523	0.69897	0.62762	-0.27808	0.13838	0.35453	0.29119	-0.22496	0.7622	0.38716	0.49835	0.2402	0.59889	0.60251	0.61637
W1	PGE2	CETN2	0.0523	1	-0.10364	0.11001	-0.02673	0.13822	0.47343	0.00749	-0.14654	0.10249	0.41326	0.21401	-0.0165	0.19578	0.17219	0.10556
W1	PGE2	CTNNB1	0.69897	-0.10364	1	0.54511	-0.2273	0.05869	-0.00476	0.19644	-0.19826	0.68519	0.12272	0.28414	0.25064	0.51896	0.54915	0.4624
W1	PGE2	DSP	0.62762	0.11001	0.54511	1	-0.24041	0.20816	0.30093	0.21375	-0.22725	0.67999	0.27968	0.39021	0.11676	0.46568	0.45467	0.70382
W1	PGE2	FBL	-0.27808	-0.02673	-0.2273	-0.24041	1	0.01312	-0.29318	-0.16092	-0.46072	-0.09772	-0.29087	-0.24776	-0.07272	-0.33199	-0.42049	-0.3097
W1	PGE2	GJA1	0.13838	0.13822	0.05869	0.20816	0.01312	1	0.12799	-0.00587	-0.00012	0.18032	-0.03433	0.09269	0.04787	0.09562	0.0474	0.07685
W1	PGE2	LAMP1	0.35453	0.47343	-0.00476	0.30093	-0.29318	0.12799	1	0.18819	-0.21383	0.40336	0.75505	0.60543	0.05182	0.50977	0.5824	0.43788
W1	PGE2	LC3B	0.29119	0.00749	0.19644	0.21375	-0.16092	-0.00587	0.18819	1	-0.10705	0.29329	0.24354	0.26184	0.09015	0.29463	0.31754	0.28889
W1	PGE2	LMNB1	-0.22496	-0.14654	-0.19826	-0.22725	-0.46072	-0.00012	-0.21383	-0.10705	1	-0.37701	-0.28646	-0.2243	-0.05093	-0.21353	-0.20413	-0.29474
W1	PGE2	MYH10	0.7622	0.10249	0.68519	0.67999	-0.09772	0.18032	0.40336	0.29329	-0.37701	1	0.39274	0.57123	0.18154	0.64714	0.65214	0.68783
W1	PGE2	RAB5	0.38716	0.41326	0.12272	0.27968	-0.29087	-0.03433	0.75505	0.24354	-0.28646	0.39274	1	0.54613	0.08929	0.53772	0.60439	0.4644
W1	PGE2	SLC25A17	0.49835	0.21401	0.28414	0.39021	-0.24776	0.09269	0.60543	0.26184	-0.2243	0.57123	0.54613	1	0.14951	0.64204	0.66033	0.46349
W1	PGE2	ST6GAL1	0.2402	-0.0165	0.25064	0.11676	-0.07272	0.04787	0.05182	0.09015	-0.05093	0.18154	0.08929	0.14951	1	0.21643	0.23829	0.08629
W1	PGE2	SEC61B	0.59889	0.19578	0.51896	0.46568	-0.33199	0.09562	0.50977	0.29463	-0.21353	0.64714	0.53772	0.64204	0.21643	1	0.83319	0.49309
W1	PGE2	TOM20	0.60251	0.17219	0.54915	0.45467	-0.42049	0.0474	0.5824	0.31754	-0.20413	0.65214	0.60439	0.66033	0.23829	0.83319	1	0.52435
W1	PGE2	TJP1	0.61637	0.10556	0.4624	0.70382	-0.3097	0.07685	0.43788	0.28889	-0.29474	0.68783	0.4644	0.46349	0.08629	0.49309	0.52435	1
W1	HPI4	ACTB	1	0.14753	0.7298	0.61635	-0.12523	0.16391	0.31836	0.27203	-0.16964	0.75129	0.37406	0.48783	0.08271	0.5718	0.51728	0.58741
W1	HPI4	CETN2	0.14753	1	-0.02445	0.18777	-0.20455	-0.00125	0.50447	0.26745	-0.16362	0.21959	0.49494	0.32478	-0.09878	0.2301	0.35364	0.19361
W1	HPI4	CTNNB1	0.7298	-0.02445	1	0.61589	-0.22426	0.21318	0.00747	0.20798	-0.14353	0.70267	0.15092	0.36561	0.00796	0.56227	0.44662	0.52047
W1	HPI4	DSP	0.61635	0.18777	0.61589	1	-0.28616	0.17026	0.29884	0.27886	-0.16013	0.69118	0.32089	0.43346	0.08663	0.48107	0.44031	0.76546
W1	HPI4	FBL	-0.12523	-0.20455	-0.22426	-0.28616	1	-0.07435	-0.24244	-0.19301	-0.30165	-0.2161	-0.31428	-0.15589	0.25023	-0.3751	-0.47529	-0.31049
W1	HPI4	GJA1	0.16391	-0.00125	0.21318	0.17026	-0.07435	1	0.01099	0.04924	-0.05267	0.17682	0.05123	0.08917	0.01307	0.14653	0.12277	0.1587
W1	HPI4	LAMP1	0.31836	0.50447	0.00747	0.29884	-0.24244	0.01099	1	0.31334	-0.09981	0.41245	0.74001	0.48123	0.08706	0.4324	0.63186	0.39135
W1	HPI4	LC3B	0.27203	0.26745	0.20798	0.27886	-0.19301	0.04924	0.31334	1	-0.12895	0.32964	0.33463	0.33194	0.01973	0.3308	0.37317	0.30111
W1	HPI4	LMNB1	-0.16964	-0.16362	-0.14353	-0.16013	-0.30165	-0.05267	-0.09981	-0.12895	1	-0.1513	-0.18618	-0.22801	-0.05563	-0.05783	-0.02761	-0.14249
W1	HPI4	MYH10	0.75129	0.21959	0.70267	0.69118	-0.2161	0.17682	0.41245	0.32964	-0.1513	1	0.46334	0.60043	0.06367	0.66832	0.65286	0.69022
W1	HPI4	RAB5	0.37406	0.49494	0.15092	0.32089	-0.31428	0.05123	0.74001	0.33463	-0.18618	0.46334	1	0.52576	-0.03456	0.5068	0.67573	0.36704
W1	HPI4	SLC25A17	0.48783	0.32478	0.36561	0.43346	-0.15589	0.08917	0.48123	0.33194	-0.22801	0.60043	0.52576	1	0.06111	0.61287	0.60734	0.46226
W1	HPI4	ST6GAL1	0.08271	-0.09878	0.00796	0.08663	0.25023	0.01307	0.08706	0.01973	-0.05563	0.06367	-0.03456	0.06111	1	0.06676	0.03248	0.20177
W1	HPI4	SEC61B	0.5718	0.2301	0.56227	0.48107	-0.3751	0.14653	0.4324	0.3308	-0.05783	0.66832	0.5068	0.61287	0.06676	1	0.81289	0.49303
W1	HPI4	TOM20	0.51728	0.35364	0.44662	0.44031	-0.47529	0.12277	0.63186	0.37317	-0.02761	0.65286	0.67573	0.60734	0.03248	0.81289	1	0.49109
W1	HPI4	TJP1	0.58741	0.19361	0.52047	0.76546	-0.31049	0.1587	0.39135	0.30111	-0.14249	0.69022	0.36704	0.46226	0.20177	0.49303	0.49109	1
W2	PGE2	ACTB	1	0.25429	0.72112	0.62717	-0.25773	0.50201	0.29245	0.37621	-0.15673	0.80746	0.39465	0.55636	0.42785	0.54556	0.39565	0.59828
W2	PGE2	CETN2	0.25429	1	-0.03787	0.20303	-0.15973	0.13461	0.7157	0.1837	0.02329	0.49063	0.69315	0.5345	0.38335	0.24777	0.58005	0.16704
W2	PGE2	CTNNB1	0.72112	-0.03787	1	0.41924	-0.12261	0.42283	0.09135	0.26275	-0.05961	0.54789	0.13309	0.32763	0.21283	0.40744	0.15818	0.40919
W2	PGE2	DSP	0.62717	0.20303	0.41924	1	-0.33961	0.51111	0.31967	0.23255	-0.03893	0.78713	0.36894	0.48718	0.39844	0.48346	0.39572	0.92087
W2	PGE2	FBL	-0.25773	-0.15973	-0.12261	-0.33961	1	-0.21062	-0.22103	-0.01629	-0.16695	-0.41685	-0.0977	-0.26031	-0.29713	-0.39048	-0.5262	-0.28996
W2	PGE2	GJA1	0.50201	0.13461	0.42283	0.51111	-0.21062	1	0.23059	0.22971	-0.03871	0.51655	0.29299	0.42919	0.4094	0.40209	0.29317	0.5073
W2	PGE2	LAMP1	0.29245	0.7157	0.09135	0.31967	-0.22103	0.23059	1	0.17619	0.14789	0.54173	0.63374	0.64083	0.47244	0.41525	0.70198	0.30384
W2	PGE2	LC3B	0.37621	0.1837	0.26275	0.23255	-0.01629	0.22971	0.17619	1	-0.12217	0.36504	0.27587	0.3923	0.314	0.31706	0.24282	0.21677
W2	PGE2	LMNB1	-0.15673	0.02329	-0.05961	-0.03893	-0.16695	-0.03871	0.14789	-0.12217	1	-0.06207	-0.03676	-0.10322	-0.14022	0.01478	0.18004	-0.04651
W2	PGE2	MYH10	0.80746	0.49063	0.54789	0.78713	-0.41685	0.51655	0.54173	0.36504	-0.06207	1	0.58275	0.6961	0.53827	0.63803	0.64398	0.74604
W2	PGE2	RAB5	0.39465	0.69315	0.13309	0.36894	-0.0977	0.29299	0.63374	0.27587	-0.03676	0.58275	1	0.65644	0.44052	0.3986	0.56545	0.31918
W2	PGE2	SLC25A17	0.55636	0.5345	0.32763	0.48718	-0.26031	0.42919	0.64083	0.3923	-0.10322	0.6961	0.65644	1	0.63269	0.65011	0.68962	0.44159
W2	PGE2	ST6GAL1	0.42785	0.38335	0.21283	0.39844	-0.29713	0.4094	0.47244	0.314	-0.14022	0.53827	0.44052	0.63269	1	0.48623	0.54151	0.39873
W2	PGE2	SEC61B	0.54556	0.24777	0.40744	0.48346	-0.39048	0.40209	0.41525	0.31706	0.01478	0.63803	0.3986	0.65011	0.48623	1	0.73293	0.38642
W2	PGE2	TOM20	0.39565	0.58005	0.15818	0.39572	-0.5262	0.29317	0.70198	0.24282	0.18004	0.64398	0.56545	0.68962	0.54151	0.73293	1	0.30144
W2	PGE2	TJP1	0.59828	0.16704	0.40919	0.92087	-0.28996	0.5073	0.30384	0.21677	-0.04651	0.74604	0.31918	0.44159	0.39873	0.38642	0.30144	1
W2	HPI4	ACTB	1	0.22396	0.36756	-0.04349	0.34525	0.34354	0.08707	-0.10905	-0.01112	0.06848	-0.0199	0.04655	-0.03985	0.17599	0.29399	0.05784
W2	HPI4	CETN2	0.22396	1	0.3935	-0.02154	0.2826	0.36897	0.29812	0.20552	-0.2286	0.33278	0.111	0.40455	0.15387	0.27763	0.32715	0.1471
W2	HPI4	CTNNB1	0.36756	0.3935	1	0.10398	0.30699	0.39036	0.27189	0.067	-0.09116	0.39781	-0.00249	0.26192	0.04219	0.35043	0.45203	0.24173
W2	HPI4	DSP	-0.04349	-0.02154	0.10398	1	-0.25648	-0.00516	0.35184	0.10839	-0.17498	0.6192	0.19802	0.2412	0.13802	0.42716	0.31673	0.75646
W2	HPI4	FBL	0.34525	0.2826	0.30699	-0.25648	1	0.41	-0.0689	-0.05222	-0.3288	-0.07266	-0.14656	0.00193	-0.10644	-0.02715	0.04033	-0.17273
W2	HPI4	GJA1	0.34354	0.36897	0.39036	-0.00516	0.41	1	0.2668	0.12997	-0.15631	0.2868	0.06428	0.20032	0.06478	0.22906	0.31155	0.13762
W2	HPI4	LAMP1	0.08707	0.29812	0.27189	0.35184	-0.0689	0.2668	1	0.25789	-0.14418	0.61928	0.26213	0.48118	0.23992	0.60358	0.65883	0.45913
W2	HPI4	LC3B	-0.10905	0.20552	0.067	0.10839	-0.05222	0.12997	0.25789	1	-0.124	0.35589	0.15186	0.31692	0.24623	0.22436	0.18723	0.21327
W2	HPI4	LMNB1	-0.01112	-0.2286	-0.09116	-0.17498	-0.3288	-0.15631	-0.14418	-0.124	1	-0.20264	-0.13717	-0.24385	-0.15737	-0.16004	-0.0761	-0.19194
W2	HPI4	MYH10	0.06848	0.33278	0.39781	0.6192	-0.07266	0.2868	0.61928	0.35589	-0.20264	1	0.29057	0.55136	0.28881	0.69139	0.6643	0.76337
W2	HPI4	RAB5	-0.0199	0.111	-0.00249	0.19802	-0.14656	0.06428	0.26213	0.15186	-0.13717	0.29057	1	0.20108	0.14958	0.2842	0.22206	0.2044
W2	HPI4	SLC25A17	0.04655	0.40455	0.26192	0.2412	0.00193	0.20032	0.48118	0.31692	-0.24385	0.55136	0.20108	1	0.26626	0.54212	0.50424	0.37663
W2	HPI4	ST6GAL1	-0.03985	0.15387	0.04219	0.13802	-0.10644	0.06478	0.23992	0.24623	-0.15737	0.28881	0.14958	0.26626	1	0.27708	0.19136	0.20935
W2	HPI4	SEC61B	0.17599	0.27763	0.35043	0.42716	-0.02715	0.22906	0.60358	0.22436	-0.16004	0.69139	0.2842	0.54212	0.27708	1	0.79641	0.48027
W2	HPI4	TOM20	0.29399	0.32715	0.45203	0.31673	0.04033	0.31155	0.65883	0.18723	-0.0761	0.6643	0.22206	0.50424	0.19136	0.79641	1	0.41458
W2	HPI4	TJP1	0.05784	0.1471	0.24173	0.75646	-0.17273	0.13762	0.45913	0.21327	-0.19194	0.76337	0.2044	0.37663	0.20935	0.48027	0.41458	1
W3	PGE2	ACTB	1	0.17067	0.83088	0.71606	-0.30942	0.63117	0.13496	0.36423	-0.22071	0.79614	0.50655	0.4417	0.45147	0.49672	0.23765	0.5924
W3	PGE2	CETN2	0.17067	1	0.18327	0.19851	-0.11475	0.16508	0.0792	0.1285	-0.07256	0.19838	0.15016	0.19159	0.17337	0.19124	0.1105	0.23562
W3	PGE2	CTNNB1	0.83088	0.18327	1	0.58727	-0.12131	0.58848	0.11155	0.36861	-0.16737	0.64421	0.55796	0.47324	0.38467	0.48001	0.16795	0.55811
W3	PGE2	DSP	0.71606	0.19851	0.58727	1	-0.44065	0.59403	0.27796	0.40371	-0.12339	0.78528	0.46753	0.51813	0.51088	0.60078	0.53172	0.77001
W3	PGE2	FBL	-0.30942	-0.11475	-0.12131	-0.44065	1	-0.27911	-0.23993	-0.2503	-0.15513	-0.43919	-0.08581	-0.25102	-0.32142	-0.26105	-0.52801	-0.2894
W3	PGE2	GJA1	0.63117	0.16508	0.58848	0.59403	-0.27911	1	0.4192	0.4327	-0.22507	0.70878	0.43292	0.55153	0.52268	0.4668	0.34917	0.56594
W3	PGE2	LAMP1	0.13496	0.0792	0.11155	0.27796	-0.23993	0.4192	1	0.47954	-0.16148	0.47826	0.31705	0.62256	0.50332	0.34004	0.55549	0.19111
W3	PGE2	LC3B	0.36423	0.1285	0.36861	0.40371	-0.2503	0.4327	0.47954	1	-0.15359	0.50489	0.47539	0.6188	0.47175	0.4855	0.44897	0.3041
W3	PGE2	LMNB1	-0.22071	-0.07256	-0.16737	-0.12339	-0.15513	-0.22507	-0.16148	-0.15359	1	-0.24142	-0.1627	-0.21833	-0.232	-0.2022	0.14641	-0.17608
W3	PGE2	MYH10	0.79614	0.19838	0.64421	0.78528	-0.43919	0.70878	0.47826	0.50489	-0.24142	1	0.51074	0.59286	0.60067	0.55587	0.50193	0.63717
W3	PGE2	RAB5	0.50655	0.15016	0.55796	0.46753	-0.08581	0.43292	0.31705	0.47539	-0.1627	0.51074	1	0.66608	0.52242	0.5976	0.42943	0.27187
W3	PGE2	SLC25A17	0.4417	0.19159	0.47324	0.51813	-0.25102	0.55153	0.62256	0.6188	-0.21833	0.59286	0.66608	1	0.65391	0.66727	0.60257	0.37111
W3	PGE2	ST6GAL1	0.45147	0.17337	0.38467	0.51088	-0.32142	0.52268	0.50332	0.47175	-0.232	0.60067	0.52242	0.65391	1	0.57198	0.55249	0.3509
W3	PGE2	SEC61B	0.49672	0.19124	0.48001	0.60078	-0.26105	0.4668	0.34004	0.4855	-0.2022	0.55587	0.5976	0.66727	0.57198	1	0.58316	0.36643
W3	PGE2	TOM20	0.23765	0.1105	0.16795	0.53172	-0.52801	0.34917	0.55549	0.44897	0.14641	0.50193	0.42943	0.60257	0.55249	0.58316	1	0.15639
W3	PGE2	TJP1	0.5924	0.23562	0.55811	0.77001	-0.2894	0.56594	0.19111	0.3041	-0.17608	0.63717	0.27187	0.37111	0.3509	0.36643	0.15639	1
W3	HPI4	ACTB	1	0.03826	0.14373	0.63511	-0.15156	0.11699	0.26047	0.06347	-0.21536	0.59303	0.10124	0.1238	0.01358	0.56934	0.5244	0.67589
W3	HPI4	CETN2	0.03826	1	-0.00189	0.02484	-0.02291	0.00917	0.02629	0.00827	-0.00779	0.00785	0.02805	0.00767	0.02201	0.04547	0.06119	0.0166
W3	HPI4	CTNNB1	0.14373	-0.00189	1	0.10141	0.2888	-0.01602	0.33885	0.02209	0.04998	0.23892	0.06109	0.01963	-0.05338	0.26092	0.23833	0.11974
W3	HPI4	DSP	0.63511	0.02484	0.10141	1	-0.22897	0.15396	0.21441	0.06017	-0.2475	0.64598	0.14195	0.18676	-0.01667	0.47021	0.35641	0.70412
W3	HPI4	FBL	-0.15156	-0.02291	0.2888	-0.22897	1	0.0903	0.11693	-0.00877	0.12526	-0.09185	-0.07136	-0.04353	0.15906	-0.00647	-0.01269	-0.14335
W3	HPI4	GJA1	0.11699	0.00917	-0.01602	0.15396	0.0903	1	-0.00284	-0.00705	0.05853	0.08507	0.06973	0.03543	0.08209	0.10753	0.10437	0.12219
W3	HPI4	LAMP1	0.26047	0.02629	0.33885	0.21441	0.11693	-0.00284	1	0.03285	-0.09934	0.34588	0.0761	0.1144	0.05426	0.43105	0.37135	0.20127

W3	HPI4	LC3B	0.06347	0.00827	0.02209	0.06017	-0.00877	-0.00705	0.03285	1	-0.03652	0.07766	0.02584	0.03184	0.00017	0.11302	0.07733	0.05627
W3	HPI4	LMNB1	-0.21536	-0.00779	0.04998	-0.2475	0.12526	0.05853	-0.09934	-0.03652	1	-0.30923	-0.05871	-0.1175	0.0588	-0.13939	-0.05675	-0.1345
W3	HPI4	MYH10	0.59303	0.00785	0.23892	0.64598	-0.09185	0.08507	0.34588	0.07766	-0.30923	1	0.15186	0.35646	-0.07179	0.6122	0.45	0.59914
W3	HPI4	RAB5	0.10124	0.02805	0.06109	0.14195	-0.07136	0.06973	0.0761	0.02584	-0.05871	0.15186	1	0.01479	-0.01028	0.16365	0.11297	0.06499
W3	HPI4	SLC25A17	0.1238	0.00767	0.01963	0.18676	-0.04353	0.03543	0.1144	0.03184	-0.1175	0.35646	0.01479	1	-0.04544	0.26045	0.17821	0.20508
W3	HPI4	ST6GAL1	0.01358	0.02201	-0.05338	-0.01667	0.15906	0.08209	0.05426	0.00017	0.0588	-0.07179	-0.01028	-0.04544	1	0.04667	0.03918	0.00757
W3	HPI4	SEC61B	0.56934	0.04547	0.26092	0.47021	-0.00647	0.10753	0.43105	0.11302	-0.13939	0.6122	0.16365	0.26045	0.04667	1	0.72484	0.50857
W3	HPI4	TOM20	0.5244	0.06119	0.23833	0.35641	-0.01269	0.10437	0.37135	0.07733	-0.05675	0.45	0.11297	0.17821	0.03918	0.72484	1	0.39669
W3	HPI4	TJP1	0.67589	0.0166	0.11974	0.70412	-0.14335	0.12219	0.20127	0.05627	-0.1345	0.59914	0.06499	0.20508	0.00757	0.50857	0.39669	1
W4	PGE2	ACTB	1	-0.0035	0.74265	0.73729	-0.34398	0.45068	-0.00265	0.23929	-0.09128	0.74615	0.47527	0.55669	0.40811	0.50603	0.31492	0.57118
W4	PGE2	CETN2	-0.0035	1	0.09124	-0.04481	-0.25149	-0.06057	0.4968	0.28273	-0.10078	0.3689	0.1609	0.23655	0.22189	0.02788	0.36493	0.03964
W4	PGE2	CTNNB1	0.74265	0.09124	1	0.5443	-0.21809	0.44328	0.25719	0.30473	-0.09383	0.67757	0.54702	0.57765	0.40878	0.43474	0.37072	0.6171
W4	PGE2	DSP	0.73729	-0.04481	0.5443	1	-0.39768	0.35331	0.03995	0.27134	0.1151	0.65388	0.48415	0.64945	0.37395	0.70221	0.57781	0.54376
W4	PGE2	FBL	-0.34398	-0.25149	-0.21809	-0.39768	1	0.08018	-0.23358	-0.29777	0.11008	-0.48605	-0.22541	-0.35462	-0.28252	-0.23141	-0.48725	-0.27774
W4	PGE2	GJA1	0.45068	-0.06057	0.44328	0.35331	0.08018	1	-0.04417	0.15883	-0.0757	0.34884	0.30243	0.36247	0.28968	0.3474	0.08383	0.36993
W4	PGE2	LAMP1	-0.00265	0.4968	0.25719	0.03995	-0.23358	-0.04417	1	0.43902	-0.01482	0.49813	0.28863	0.38178	0.17349	0.13858	0.60381	0.09329
W4	PGE2	LC3B	0.23929	0.28273	0.30473	0.27134	-0.29777	0.15883	0.43902	1	-0.14699	0.4515	0.35939	0.49026	0.37024	0.27959	0.47384	0.21861
W4	PGE2	LMNB1	-0.09128	-0.10078	-0.09383	0.1151	0.11008	-0.0757	-0.01482	-0.14699	1	-0.08204	-0.05563	-0.00819	-0.16285	0.1211	0.09585	-0.10432
W4	PGE2	MYH10	0.74615	0.3689	0.67757	0.65388	-0.48605	0.34884	0.49813	0.4515	-0.08204	1	0.49988	0.68911	0.42016	0.47315	0.633	0.54354
W4	PGE2	RAB5	0.47527	0.1609	0.54702	0.48415	-0.22541	0.30243	0.28863	0.35939	-0.05563	0.49988	1	0.65626	0.41799	0.58424	0.53405	0.19359
W4	PGE2	SLC25A17	0.55669	0.23655	0.57765	0.64945	-0.35462	0.36247	0.38178	0.49026	-0.00819	0.68911	0.65626	1	0.47856	0.66997	0.71367	0.35057
W4	PGE2	ST6GAL1	0.40811	0.22189	0.40878	0.37395	-0.28252	0.28968	0.17349	0.37024	-0.16285	0.42016	0.41799	0.47856	1	0.27336	0.33071	0.34709
W4	PGE2	SEC61B	0.50603	0.02788	0.43474	0.70221	-0.23141	0.3474	0.13858	0.27959	0.1211	0.47315	0.58424	0.66997	0.27336	1	0.67138	0.1918
W4	PGE2	TOM20	0.31492	0.36493	0.37072	0.57781	-0.48725	0.08383	0.60381	0.47384	0.09585	0.633	0.53405	0.71367	0.33071	0.67138	1	0.1827
W4	PGE2	TJP1	0.57118	0.03964	0.6171	0.54376	-0.27774	0.36993	0.09329	0.21861	-0.10432	0.54354	0.19359	0.35057	0.34709	0.1918	0.1827	1
W4	HPI4	ACTB	1	0.05915	0.2111	0.51423	0.12469	0.17868	0.29153	0.42213	-0.13421	0.64129	0.07143	-0.20084	0.03477	0.37138	0.30901	0.50435
W4	HPI4	CETN2	0.05915	1	0.08302	0.12844	-0.04069	0.01187	0.04871	0.04629	-0.02314	0.07904	0.01299	-0.00334	-0.00712	0.13272	0.1026	0.11298
W4	HPI4	CTNNB1	0.2111	0.08302	1	0.26284	-0.02395	0.04435	0.09057	0.13768	-0.07139	0.22271	0.07623	-0.09664	-0.01363	0.27018	0.24478	0.26242
W4	HPI4	DSP	0.51423	0.12844	0.26284	1	-0.0112	0.19385	0.24797	0.36829	-0.17115	0.67285	0.09844	-0.2344	-0.04797	0.44312	0.4133	0.68118
W4	HPI4	FBL	0.12469	-0.04069	-0.02395	-0.0112	1	0.08975	0.13953	0.05318	-0.13428	0.20442	-0.04835	-0.27691	0.09688	0.04551	-0.02885	0.01872
W4	HPI4	GJA1	0.17868	0.01187	0.04435	0.19385	0.08975	1	-0.01141	0.21179	-0.22356	0.29948	0.03226	-0.21713	-0.01273	0.12761	0.09974	0.11951
W4	HPI4	LAMP1	0.29153	0.04871	0.09057	0.24797	0.13953	-0.01141	1	0.28991	0.00898	0.4154	0.0673	0.01993	0.0043	0.36386	0.40148	0.19896
W4	HPI4	LC3B	0.42213	0.04629	0.13768	0.36829	0.05318	0.21179	0.28991	1	-0.21734	0.58689	0.05603	0.04105	-0.05831	0.26848	0.2672	0.34494
W4	HPI4	LMNB1	-0.13421	-0.02314	-0.07139	-0.17115	-0.13428	-0.22356	0.00898	-0.21734	1	-0.2332	-0.0366	0.08421	0.01396	-0.06643	0.03192	-0.13547
W4	HPI4	MYH10	0.64129	0.07904	0.22271	0.67285	0.20442	0.29948	0.4154	0.58689	-0.2332	1	0.07672	-0.30705	-0.06685	0.46499	0.4358	0.60777
W4	HPI4	RAB5	0.07143	0.01299	0.07623	0.09844	-0.04835	0.03226	0.0673	0.05603	-0.0366	0.07672	1	0.00021	-0.00603	0.12165	0.10076	0.09167
W4	HPI4	SLC25A17	-0.20084	-0.00334	-0.09664	-0.2344	-0.27691	-0.21713	0.01993	0.04105	0.08421	-0.30705	0.00021	1	0.06694	-0.07967	0.00536	-0.23655
W4	HPI4	ST6GAL1	0.03477	-0.00712	-0.01363	-0.04797	0.09688	-0.01273	0.0043	-0.05831	0.01396	-0.06685	-0.00603	0.06694	1	0.04238	0.02696	-0.00592
W4	HPI4	SEC61B	0.37138	0.13272	0.27018	0.44312	0.04551	0.12761	0.36386	0.26848	-0.06643	0.46499	0.12165	-0.07967	0.04238	1	0.73581	0.40189
W4	HPI4	TOM20	0.30901	0.1026	0.24478	0.4133	-0.02885	0.09974	0.40148	0.2672	0.03192	0.4358	0.10076	0.00536	0.02696	0.73581	1	0.35071
W4	HPI4	TJP1	0.50435	0.11298	0.26242	0.68118	0.01872	0.11951	0.19896	0.34494	-0.13547	0.60777	0.09167	-0.23655	-0.00592	0.40189	0.35071	1

Table S16

		Week 1		Week 2		Week 3		Week 4	
		Percentage Volume	Sd	Percentage Volume	Sd	Percentage Volume	Sd	Percentage Volume	Sd
PGE2	ACTB	8.4	4.2	9.0	4.9	6.6	3.5	5.4	3.5
PGE2	MYH10	8.6	4.3	11.8	4	12.9	3.7	12.4	3.9
PGE2	FBL	1.6	0.7	1.3	0.5	1.6	0.4	1.0	0.7
PGE2	LMNB1	4.6	3.7	4.4	2.6	4.4	2.8	4.1	2.9
PGE2	CTNNB1	7.4	2.7	6.5	3.2	6.7	2.9	7.1	2.8
PGE2	DSP	0.9	0.7	2.1	2.3	3.4	4.4	4.4	5.2
PGE2	GJA1	2.2	4	1.5	1.9	1.0	1.8	1.0	2.3
PGE2	TJP1	1.7	1.1	1.8	1.2	1.7	1.1	1.5	1
PGE2	CETN2	0.2	0.2	0.2	0.2	0.4	0.2	0.3	0.1
PGE2	SEC61B	11.6	6.1	10.0	6.5	9.6	5.9	11.8	6.6
PGE2	ST6GAL1	1.2	1.6	1.2	1.3	1.0	1.1	1.0	1.2
PGE2	TOM20	10.4	3.6	10.5	4.3	10.2	4.2	10.0	4.4
PGE2	LAMP1	1.3	0.8	1.0	0.9	1.1	0.8	1.1	1
PGE2	LC3B	0.3	0.5	0.5	0.7	0.7	0.6	0.4	0.4
PGE2	RAB5	2.7	1.6	2.7	1.9	2.5	1.8	2.1	1.8
PGE2	SLC25A17	4.1	1.6	3.9	1.4	3.9	1.4	4.5	1.1
PGE2	Nucleus	32.7	5.7	31.6	5	32.2	4.3	31.7	3.3
PGE2	Totals	100.0		100.0		100.0		100.0	
HPI4	ACTB	8.7	4.3	12.3	11.3	7.7	3.4	9.9	4.2
HPI4	MYH10	8.4	4.2	8.9	4.8	11.9	4.6	14.0	5.3
HPI4	FBL	1.6	0.7	1.6	0.8	1.7	0.6	1.0	0.6
HPI4	LMNB1	4.5	3.8	4.3	3.2	2.8	3.2	3.3	3
HPI4	CTNNB1	7.4	2.6	6.4	7.5	7.1	7.3	6.7	3.2
HPI4	DSP	0.9	0.8	1.5	1.2	1.8	1.1	1.8	1.6
HPI4	GJA1	1.9	3.9	2.6	3.6	3.2	4.1	2.6	3.2
HPI4	TJP1	1.9	1.2	1.9	1.2	2.6	1.4	2.3	1.2
HPI4	CETN2	0.2	0.2	0.3	0.2	0.4	0.5	0.6	0.6
HPI4	SEC61B	12.2	5.7	9.5	5.9	11.1	6.4	8.9	5.9
HPI4	ST6GAL1	1.2	1.4	2.5	2	1.9	1.9	1.7	1.7
HPI4	TOM20	10.1	3.6	7.5	3.9	8.1	3.8	10.0	4
HPI4	LAMP1	1.3	0.8	1.0	1.2	1.2	1.3	1.0	1.1
HPI4	LC3B	0.3	0.4	0.9	1.2	0.7	1.1	1.6	2.6
HPI4	RAB5	2.3	1.7	2.4	3.1	1.1	1.1	1.4	1.9
HPI4	SLC25A17	4.1	1.6	4.3	2.5	4.3	2.2	2.4	1.7
HPI4	Nucleus	33.0	5.4	32.0	3.7	32.3	4.8	30.8	2.8
HPI4	Totals	100.0		100.0		100.0		100.0	

Table S17

description	endog	treatment	week1	week2	week3	week4	week1_lower	week2_lower	week3_lower	week4_lower	week1_upper	week2_upper	week3_upper	week4_upper	param_const	param_week	param_week_squared
Mixed model - cell	Cell Volume	PGE2	769.1438119	752.9284579	754.3609778	773.4413716	692.2294307	677.6356121	678.92488	696.0972345	846.0581931	828.2213036	829.7970755	850.7855088	803.0070399	-42.68716496	8.823936974
Mixed model - cell	Cell Volume	HPI4	751.8356297	695.5062879	706.1413784	783.7409011	676.6520667	625.9556591	635.5272405	705.3668109	827.0191927	765.0569167	776.7555162	862.1149912	875.1294037	-156.7759901	33.48221611
Mixed model - cell	Cell AR	PGE2	1.628053466	1.550516492	1.52969886	1.565600569	1.465248119	1.395464843	1.376728974	1.409040512	1.790858813	1.705568141	1.682668746	1.722160626	1.762309781	-0.162615986	0.028359671
Mixed model - cell	Cell AR	HPI4	1.632768151	1.65073575	1.698152619	1.77501876	1.469491336	1.485662175	1.528337357	1.597516884	1.796044967	1.815809325	1.867967881	1.952520636	1.644249824	-0.026206308	0.014724636
Mixed model - cell	Cell Area	PGE2	250.7124025	200.1006417	186.6044545	210.223841	225.6411622	180.0905775	167.9440091	189.2014569	275.7836427	220.1107059	205.2649	231.2462251	338.4397369	-106.2851212	18.55778681
Mixed model - cell	Cell Area	HPI4	250.3382138	255.7046458	278.9978104	320.2177075	225.3043924	230.1341813	251.0980294	288.1959368	275.3720352	281.2751104	306.8975915	352.2394783	262.8985143	-21.5236668	8.963366275
Mixed model - nuclei	Nucleus Volume	PGE2	379.1927533	302.5466486	261.3904307	255.7240994	341.273478	272.2919838	235.2513876	230.1516894	417.1120286	332.8013135	287.5294737	281.2965093	491.3287447	-129.8809347	17.74494335
Mixed model - nuclei	Nucleus Volume	HPI4	363.7234758	289.1299353	253.9242852	258.1065253	327.3511282	260.2169418	228.5318567	232.2958728	400.0958233	318.0429288	279.3167137	283.9171779	477.7049065	-133.6753759	19.69394515
Mixed model - nuclei	Nucleus Area	PGE2	98.89719684	75.78780427	65.62774541	68.41702024	89.00747716	68.20902385	59.06497087	61.57531822	108.7869165	83.3665847	72.19051995	75.25872227	134.9559231	-42.53339312	6.474666851
Mixed model - nuclei	Nucleus Area	HPI4	101.323269	94.72473323	94.31880836	100.1054944	91.19094214	85.25225991	84.88692752	90.09494499	111.455596	104.1972066	103.7506892	110.1160439	114.1144158	-15.88745223	3.096305472
Mixed model - organelle ACTB	Avg. Volume	PGE2	1.185368084	1.475643008	1.61202858	1.594524803	1.066831276	1.328078707	1.450825722	1.435072323	1.303904893	1.623207308	1.773231438	1.753977284	0.741203811	0.521108948	-0.076944675
Mixed model - organelle ACTB	Avg. Volume	HPI4	1.231276613	1.235932435	1.217257975	1.175253231	1.108148951	1.112339192	1.095532177	1.057727907	1.354404274	1.359525679	1.338983772	1.292778554	1.203290506	0.039651248	-0.011665142
Mixed model - organelle MYH	Avg. Volume	PGE2	1.130093	1.894012916	2.216286856	2.096914821	1.0170837	1.704611624	1.99465817	1.887223338	1.2431023	2.083414207	2.437915542	2.306606303	-0.075472892	1.42638888	-0.220822988
Mixed model - organelle MYH	Avg. Volume	HPI4	1.18919095	1.090308059	1.071272869	1.132085382	1.070271855	0.981277253	0.964145582	1.018876844	1.308110045	1.199338864	1.178400156	1.24529392	1.367921543	-0.218654444	0.039923851
Mixed model - organelle ACTB	Count/cell	PGE2	83.93730582	59.93959602	48.63012725	50.00889951	75.54357523	53.94563642	43.76711453	45.00800956	92.3310364	65.93355563	53.49313998	55.00978946	120.6232566	-43.03007133	6.344120512
Mixed model - organelle ACTB	Count/cell	HPI4	77.93508692	61.06853054	53.69801284	55.82353383	70.14157823	54.96167749	48.32821156	50.24118044	85.72859561	67.17538359	59.06781413	61.40588721	104.297682	-31.1106144	4.74801934
Mixed model - organelle MYH	Count/cell	PGE2	99.21632396	77.68933276	69.6776094	75.1811539	89.29469157	69.92039948	62.70984846	67.66303851	109.1379564	85.45826603	76.64537034	82.69926929	134.258583	-41.79989298	6.757633926
Mixed model - organelle MYH	Count/cell	HPI4	92.9266514	85.63939012	87.13417124	97.41099475	83.63398626	77.07545111	78.42075411	87.66989527	102.2193165	94.20332913	95.84758836	107.1520942	108.9959551	-20.46032487	4.391021197
Mixed model - organelle FBL	Count/cell	PGE2	3.458630128	3.084722517	2.64807259	2.148680347	3.112767116	2.776250265	2.383265331	1.933812312	3.804493141	3.393194769	2.912879849	2.363548381	3.769795424	-0.279794138	-0.031371158
Mixed model - organelle FBL	Count/cell	HPI4	3.391073716	2.449037729	2.042146325	2.170399502	3.051966344	2.204133956	1.837931692	1.953359552	3.730181087	2.693941502	2.246360957	2.387439453	4.868254284	-1.74475286	0.267572291
Mixed model - organelle FBL	Z distribution	PGE2	-0.483373041	-0.412403002	-0.40156028	-0.450844876	-0.435035737	-0.371162702	-0.361404252	-0.405760388	-0.531710346	-0.453643302	-0.441716308	-0.495929363	-0.614470398	0.161161016	-0.030063659
Mixed model - organelle FBL	Z distribution	HPI4	-0.468757345	-0.173892911	-0.088597367	-0.212870713	-0.421881611	-0.15650362	-0.07973763	-0.191583642	-0.51563308	-0.191282202	-0.097457104	-0.234157785	-0.97319067	0.60921777	-0.104784445
Mixed model - organelle FBL	X/Y distribution	PGE2	3.761856328	3.279978515	3.154795803	3.386308192	3.385670695	2.951980663	2.839316223	3.047677373	4.13804196	3.607976366	3.470275383	3.724939011	4.600429241	-1.016920464	0.17834755
Mixed model - organelle FBL	X/Y distribution	HPI4	3.676634821	3.251371425	3.96330519	5.812436115	3.308971339	2.926234283	3.566974671	5.231192503	4.044298303	3.576508568	4.359635709	6.393679726	5.239095377	-2.131059137	0.56859858
Mixed model - organelle DSP	Avg. Volume	PGE2	0.40487765	0.500692966	0.524009524	0.474827326	0.364389885	0.450623669	0.471608572	0.427344593	0.445365415	0.550762262	0.576410477	0.522310058	0.236563578	0.204563451	-0.036249379
Mixed model - organelle DSP	Avg. Volume	HPI4	0.387352424	0.377800187	0.395902008	0.441657887	0.348617182	0.340020168	0.356311807	0.397492099	0.426087667	0.415580205	0.435492208	0.485823676	0.424558721	-0.051033326	0.013827029
Mixed model - organelle GJA1	Avg. Volume	PGE2	0.648169422	0.587160349	0.560774186	0.569010936	0.58335248	0.528444314	0.504696768	0.512109842	0.712986364	0.645876383	0.616851605	0.62591203	0.743801407	-0.112943441	0.017311456
Mixed model - organelle GJA1	Avg. Volume	HPI4	0.504481183	0.551413426	0.58923041	0.617932136	0.454033065	0.496272083	0.530307369	0.556138923	0.554929302	0.606554768	0.648153451	0.67972535	0.448433683	0.06060513	-0.004557629
Mixed model - organelle DSP	Count/cell	PGE2	23.24629608	36.54808095	51.17935534	67.14011924	20.92166647	32.89327285	46.0614198	60.42610732	25.57092569	40.20288904	56.29729087	73.85413117	11.27400073	11.30755059	0.66474476
Mixed model - organelle DSP	Count/cell	HPI4	21.5522349	25.95237446	26.67876384	23.73140305	19.39701141	23.35713701	24.01088746	21.35826274	23.70745839	28.5476119	29.34664023	26.10454335	13.47834516	9.910764826	-1.836875088
Mixed model - organelle GJA1	Count/cell	PGE2	29.89800409	22.567637	17.19207866	13.77132907	26.90820368	20.3108733	15.47287079	12.39419617	32.88780449	24.8244007	18.91128653	15.14846198	39.18317992	-10.26258021	0.977404375
Mixed model - organelle GJA1	Count/cell	HPI4	24.67517199	23.5149232	24.49234905	27.60744952	22.20765479	21.16343088	22.04311414	24.84670457	27.14268919	25.86641552	26.94158395	30.36819448	27.97309541	-4.366760734	1.068837316
Mixed model - organelle ST6GAL1	Avg. Volume	PGE2	1.971260842	1.652018672	1.247178201	0.756739429	1.774134758	1.486816805	1.122460381	0.681065486	2.168386927	1.817220539	1.371896021	0.832413372	2.204904713	-0.19084472	-0.04279915
Mixed model - organelle ST6GAL1	Avg. Volume	HPI4	1.873999095	1.263588425	0.87890264	0.71994174	1.686599185	1.137229582	0.791012376	0.647947566	2.061399004	1.389947267	0.966792904	0.791935914	2.71013465	-0.948997998	0.112862443
Mixed model - organelle TOM20	Avg. Volume	PGE2	3.826153151	4.807863753	4.986518406	4.36211711	3.443537836	4.327077378	4.487866566	3.925905399	4.208768466	5.288650128	5.485170247	4.798328821	2.0413866	2.186294525	-0.401527974
Mixed model - organelle TOM20	Avg. Volume	HPI4	3.979369664	2.877480699	2.613762782	3.188215913	3.581432698	2.589732629	2.352386504	2.869394322	4.37730663	3.165228769	2.87513906	3.507037504	5.919429677	-2.359145537	0.419085524
Mixed model - organelle LAMP1	Avg. Volume	PGE2	0.421810241	0.44616726	0.453489432	0.443776758	0.379629217	0.401550534	0.408140489	0.399399082	0.463991265	0.490783986	0.498838375	0.488154434	0.380418376	0.049909288	-0.008517423
Mixed model - organelle LAMP1	Avg. Volume	HPI4	0.435647077	0.2309652	0.158159484	0.217229928	0.392082369	0.20786868	0.142343535	0.195506935	0.479211784	0.25406172	0.173975432	0.238952921	0.772205114	-0.402496117	0.06593808
Mixed model - organelle LC3B	Avg. Volume	PGE2	0.476555624	0.40503558	0.400158359	0.46192396	0.428900061	0.364532022	0.360142523	0.415731564	0.524211186	0.445539138	0.440174194	0.508116356	0.61471849	-0.171484278	0.033321411
Mixed model - organelle LC3B	Avg. Volume	HPI4	0.500055932	0.362609598	0.352531925	0.469822913	0.450050339	0.326348638	0.317278733	0.422840622	0.550061525	0.398870558	0.387785118	0.516805204	0.764870927	-0.328499325	0.06368433
Mixed model - organelle RAB5	Avg. Volume	PGE2	0.60070254	0.540434799	0.523670797	0.550410534	0.540632286	0.486391319	0.471303717	0.49536948	0.660772794	0.594478279	0.576037877	0.605451587	0.70447402	-0.125523349	0.021751869
Mixed model - organelle RAB5	Avg. Volume	HPI4	0.601246416	0.682331144	0.646526582	0.49383273	0.541121774	0.61409803	0.581873924	0.444449457	0.661371057	0.750564259	0.711179241	0.543216003	0.403272396	0.256418665	-0.058444645
Mixed model - organelle SLC	Avg. Volume	PGE2	0.418489408	0.442369609	0.440728744	0.413566814	0.376640467	0.398132648	0.39665587	0.372210132	0.460338349	0.48660657	0.484801618	0.454923495	0.369088142	0.062161799	-0.012760533
Mixed model - organelle SLC	Avg. Volume	HPI4	0.408755508	0.44739456	0.448293197	0.411451418	0.367879957	0.402655104	0.403463877	0.370306276	0.449631058	0.492134016	0.493122516	0.452596559	0.33237604	0.095249676	-0.018870208

Table S18

Operator	Treatment	Week	Count	Mean	Median	Sd	Metric
Classical	PGE2	W4	73	125.0838	114.2158	105.1009	Cell Area
Mask R-CNN	PGE2	W4	275	141.7674	140.3639	26.89054	Cell Area
P1	PGE2	W4	297	129.3689	128.3461	28.4169	Cell Area
P2	PGE2	W4	27	135.819	133.3222	23.87578	Cell Area
P3	PGE2	W4	26	134.7847	132.4303	22.66664	Cell Area
P4	PGE2	W4	126	158.2631	146.631	66.09734	Cell Area
P5	PGE2	W4	39	135.6032	134.8714	24.03764	Cell Area
P6	PGE2	W4	40	127.149	125.2008	53.11069	Cell Area
Classical	PGE2	W4	73	1.415182	1.410714	0.188694	Cell AR
Mask R-CNN	PGE2	W4	275	1.391458	1.36	0.17278	Cell AR
P1	PGE2	W4	297	1.410225	1.372549	0.191124	Cell AR
P2	PGE2	W4	27	1.387916	1.297872	0.210074	Cell AR
P3	PGE2	W4	26	1.395255	1.329545	0.225109	Cell AR
P4	PGE2	W4	126	1.43	1.390306	0.218832	Cell AR
P5	PGE2	W4	39	1.356584	1.296875	0.161013	Cell AR
P6	PGE2	W4	40	1.386811	1.329932	0.226478	Cell AR
Classical	PGE2	W4	73	463.6909	515.5908	300.2878	Cell Volume
Mask R-CNN	PGE2	W4	275	548.357	534.2278	142.5527	Cell Volume
P1	PGE2	W4	297	478.4988	479.7018	138.5741	Cell Volume
P2	PGE2	W4	27	589.7231	599.3867	158.4143	Cell Volume
P3	PGE2	W4	26	629.5268	672.3853	149.648	Cell Volume
P4	PGE2	W4	126	546.81	501.0967	276.2771	Cell Volume
P5	PGE2	W4	39	579.9962	585.3268	126.7203	Cell Volume
P6	PGE2	W4	40	489.1318	506.2137	288.2527	Cell Volume

Table S19

Line	Treatment	Week	0-1um(%)	1-6um(%)
CTNNB1	HPI4	W1	100	0
CTNNB1	HPI4	W2	78.9	21.1
CTNNB1	HPI4	W3	85.7	14.3
CTNNB1	HPI4	W4	95.8	4.2
CTNNB1	PGE2	W1	100	0
CTNNB1	PGE2	W2	96.7	3.3
CTNNB1	PGE2	W3	100	0
CTNNB1	PGE2	W4	100	0
DSP	HPI4	W1	100	0
DSP	HPI4	W2	100	0
DSP	HPI4	W3	100	0
DSP	HPI4	W4	100	0
DSP	PGE2	W1	100	0
DSP	PGE2	W2	100	0
DSP	PGE2	W3	100	0
DSP	PGE2	W4	100	0
GJA1	HPI4	W1	73.3	26.7
GJA1	HPI4	W2	89.3	10.7
GJA1	HPI4	W3	76.7	23.3
GJA1	HPI4	W4	79.3	20.7
GJA1	PGE2	W1	67.9	32.1
GJA1	PGE2	W2	100	0
GJA1	PGE2	W3	93.3	6.7
GJA1	PGE2	W4	100	0
TJP1	HPI4	W1	100	0
TJP1	HPI4	W2	100	0
TJP1	HPI4	W3	100	0
TJP1	HPI4	W4	100	0
TJP1	PGE2	W1	100	0
TJP1	PGE2	W2	100	0
TJP1	PGE2	W3	100	0
TJP1	PGE2	W4	100	0
